# Comparative Transcriptomic Analyses of a Vero Cell Line in Suspension versus Adherent Culture Conditions

**DOI:** 10.1155/2023/9364689

**Published:** 2023-08-30

**Authors:** Marie-Angélique Sène, Yu Xia, Amine A. Kamen

**Affiliations:** Department of Bioengineering, McGill University, Montreal, QC, Canada

## Abstract

The Vero cell line is the most used continuous cell line for viral vaccine manufacturing. Its anchorage-dependent use renders scaling up challenging and operations very labor-intensive which affects cost effectiveness. Thus, efforts to adapt Vero cells to suspension cultures have been invested, but hurdles such as the long doubling time and low cell viability remain to be addressed. In this study, building on the recently published Vero cell line annotated genome, a functional genomics analysis of the Vero cells adapted to suspension is performed to better understand the genetic and phenotypic switches at play during the adaptation of Vero cells from anchorage-dependent to suspension cultures. Results show downregulation of the epithelial-to-mesenchymal transition (EMT) pathway, highlighting the dissociation between the adaptation to suspension process and EMT. Surprisingly, an upregulation of cell adhesion components is observed, notably the CDH18 gene, the cytoskeleton pathway, and the extracellular pathway. Moreover, a downregulation of the glycolytic pathway is balanced by an upregulation of the asparagine metabolism pathway, promoting cell adaptation to nutrient deprivation. A downregulation of the adherens junctions and the folate pathways alongside with the FYN gene are possible explanations behind the currently observed low-cell viability and long doubling time.

## 1. Introduction

Derived from a female *Chlorocebus sabaeus* (African green monkey) kidney, the Vero cell line is susceptible to infection by a wide range of viruses. Consequently, the Vero cell line served as a platform for the development and production of approved vaccines against dengue fever, influenza, Japanese encephalitis, polio, rabies, rotavirus, smallpox, and more recently, Ebola (using a recombinant vesicular stomatitis virus) and COVID-19 (Sinopharm, Sinovac, and CoronaVac) [1–3], thus representing the most widely used continuous cell line for the production of viral vaccines over more than 40 years of manufacturing experience [4].

Most cell-based vaccine production platforms, including Vero cells, are adherent cells cultured in an anchorage-dependent manner, thus hindering scale-up attempts due to the time and cost challenges posed by the development of large-scale anchorage-dependent cell culture platforms [5–7]. Therefore, in an effort to circumvent those challenges, stable cell lines adapted to suspension culture have been proposed over the years [8]. In the case of Vero cells in particular, significant efforts have been dedicated to developing microcarrier cultures [9], agitation [10], and medium engineering [11] to achieve scalable processes and industrialization. Despite several reports stating that Vero cells adapted to suspension showed a higher production rate for viruses such as measles virus, rabies virus, and vesicular stomatitis virus (VSV) [10, 11], some issues such as the low cell viability and long doubling time of these cells were reported underlining the needs for more effective adaptation of Vero cells to suspension by exploring new avenues such as genetic engineering [10, 12].

Recent advances in functional genomics and gene editing paved the way for a better understanding and high-throughput engineering of vaccine production cell lines, thus providing new possibilities for cell line development and vaccine bioprocessing intensification. With the recent publication of an annotated assembly of the Vero cell genome [13], we propose a transcriptomic analysis of the differences between adherent and suspension Vero cells developed by Shen et al. [11]. This process will help highlight key differentially expressed genes and their impact on the cell's phenotype and their metabolic pathways for a better understanding of the process of adaptation to suspension, thus providing insight for new strategies to successfully adapt Vero cells to suspension cultures and enabling streamlined scale-up and industrialization.

## 2. Material and Methods

### 2.1. Cell Lines and Culture Media

The adherent Vero WHO cell line studied in this work was at passage 138 (Neovacs). This cell line was itself derived from a vial of Vero ATCC CCL-81 which was send to WHO at passage 124 for analysis and establishment of the Vero WHO master cell bank approved for vaccine production. The cells were grown in static culture at 37°C and 5% CO_2_ in a humidified incubator (Infors HT, Switzerland). Cells were passaged twice weekly using TrypLE Express (Thermo Fisher Scientific) as dissociation reagent. A serum-free adapted sub-cell line grown in OptiPRO medium (Thermo Fisher Scientific) supplemented with 4 mM GlutaMAX (Thermo Fisher Scientific) was cryopreserved at a passage number of 151 in OptiPRO medium supplemented with 4 mM GlutaMAX and 10% DMSO (Sigma, USA).

The suspension Vero cell line was provided by the National Research Council (NRC) of Canada [11]. The cells were maintained at 37°C, 135 rpm, and 5% CO_2_ in a humidified Multitron orbital shaker (Infors HT, Switzerland) and were cultivated in 20 mL working volume of either IHM03 medium, provided by the NRC, or in MDXK medium (Xell AG, Germany), supplemented with 4 mM GlutaMAX (Thermo Fisher Scientific, USA) [4].

For transcriptome analysis, the media used for adherent and suspension cultures were serum-free. Vero WHO cells at passage 153 were washed in PBS (Wisent, Canada), harvested using TrypLE Express and centrifuged at 300 × g for 5 minutes. Suspension cells were also harvested and centrifuged in similar conditions. Cell pellets of around 6 million cells were quickly frozen in a mixture of dry ice/ethanol and stored at −80°C until further analysis. Adherent and suspension samples were generated in triplicates.

### 2.2. Differential Gene Expression Analysis

Total RNA sequencing (TrueSeq) was performed using Illumina NovaSeq6000 Sprime v1.5, PE100. Following standard quality control, the reads were first aligned to the recently published Vero cell genome [13] using STAR [14], and the resulting BAM files were sorted by name using SAMtools [15] before read count. Transcripts were quantified using featureCounts [16]. Differential expression analysis of the raw read counts was done using DESeq2 [17], and quality control graphs were produced using DESeq2 and R package. The resulting differentially expressed gene list was filtered with a *p* value cut-off of 0.0001.

### 2.3. Downstream Analysis of Differentially Expressed Genes

The differentially expressed (DE) genes were ranked based on their log2 fold change. Upregulated and downregulated DE genes between adherent and suspension cell lines were used for pathway enrichment analysis via the Metascape webtool and plotted using default parameters [18].

In order to identify the metabolic deregulations that distinguish adherent cells from suspension cells, metabolic genes from our list of differentially expressed genes were extracted, and their Reaction Activity Score (RAS) was computed by solving gene-protein-reaction (GPR) association rules based on the HMRcore metabolic network model via MaREA (Metabolic Reaction Enrichment Analysis) [19]. A statistical comparison of the RAS computed for adherent samples and suspension samples was done via the Kolmogorov-Smirnov test, and a metabolic map was generated.

WebGestalt (WEB-based GEne SeT AnaLysis Toolkit) [20] was used for gene set enrichment analysis with the hallmark gene set collection. To find differentially expressed pathways of genes between adherent and suspension cell lines, gene sets were filtered, and the top 20 gene sets with an adjusted *p* value lower than 0.05 were considered significantly changed.

The upregulated part of the gene list generated by DESeq2 was filtered to consider genes with a |log2 fold change| > 2 for network topology analysis (NTA) based on the network retrieval & prioritization construction method [20] by first using random walk analysis to calculate random walk probability for the input gene IDs (seeds), then identifying the relationships among the seeds in the selected network to return a retrieval sub-network where the top 20 genes with the top random walk probability are highlighted. Indeed, assuming a tight connection between mechanistically important genes and a random distribution of other genes on the network, the network topology-based analysis (NTA) uses random walk-based network propagation by identifying those genes which are potentially biologically significant. Our input gene IDs (upregulated genes previously filtered) were used as seeds, and based on its overall proximity (quantified by the random walk similarity) to the input seeds, each gene in the protein-protein interaction (PPI) network was attributed a score. Then the statistical significance of those scores was calculated via two *p* values: a global *p* value which significance is the result of a nonrandom association between the gene in the PPI network and the input seeds, and a local *p* value which significance ensures that the gene did not acquire a significant association with the input seeds simply because of network topology.

Finally, enrichment analysis of the retrieved subnetworks was done using the PPI BioGRID [21] database and Gene Ontology (GO) Biology Process terms [19]. The GO terms were first ranked based on their adjusted *p* value, and only the top 10 highly significant terms with an adjusted *p* value cut-off of 0.01 were considered.

## 3. Results

### 3.1. Differential Expression Analysis and Pathway Enrichment Analysis

**Figure 1:**
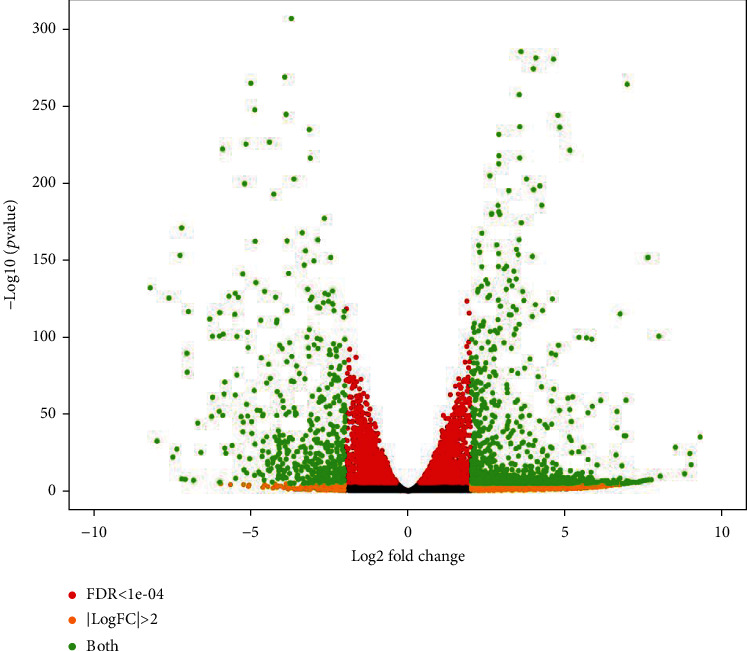
DESeq2 differential expression data quality control: volcano plot of DE genes with applied *p* value and log2 fold change cut-offs. FDR: false discovery rate; LogFC: log2 fold change.

Following the DESeq2 differential expression (DE) analysis (using adherent Vero cell samples as control and suspension Vero cell samples as case) and an applied *p* value cut-off of 10^−4^, among the 6627 DE genes that have been identified as highly significant (*p* value <10^−4^), 1753 genes were identified as highly differentially expressed, with a |log2 fold change| > 2 (Figure 1, Supplementary table S1).

**Figure 2:**
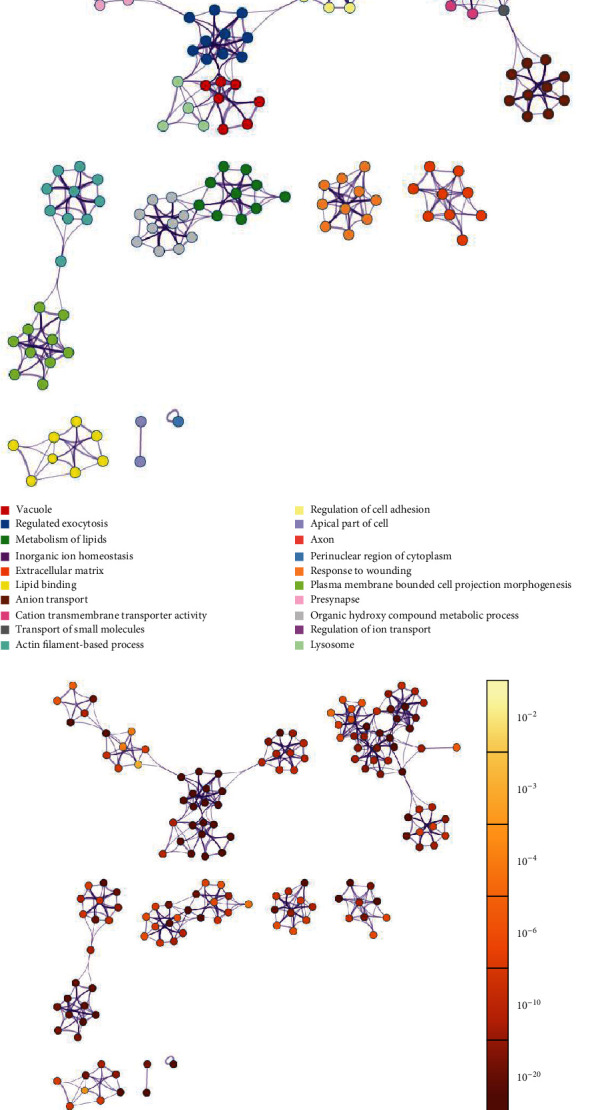
Network of terms enriched by upregulated DE genes: (a) colored by cluster ID, where nodes that share the same cluster ID are typically close to each other; (b) colored by *p* value, where terms containing more genes tend to have a more significant *p* value. Each node represents one enriched term, and edges link similar terms (the edge thickness is proportional to the similarity between terms).

**Figure 3:**
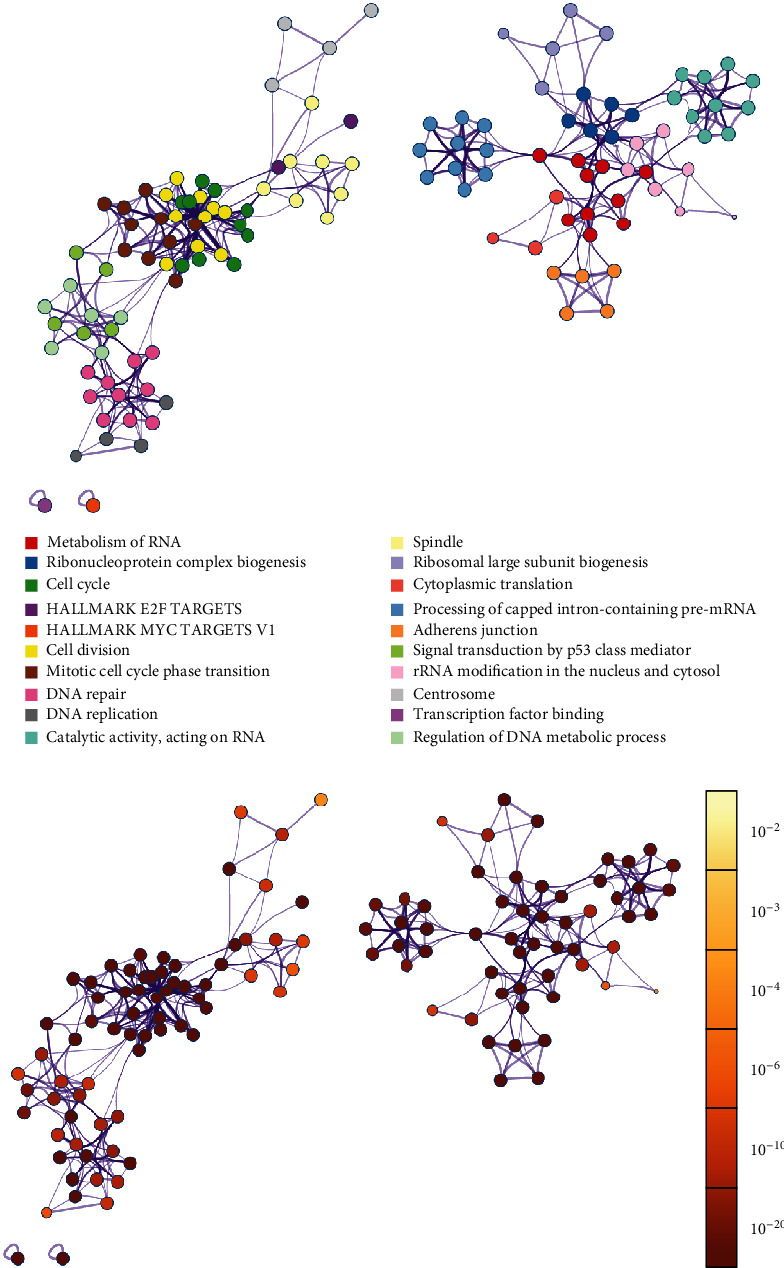
Network of terms enriched by downregulated DE genes: (a) colored by cluster ID, where nodes that share the same cluster ID are typically close to each other; (b) colored by *p* value, where terms containing more genes tend to have a more significant *p* value. Each node represents one enriched term, and edges link similar terms (the edge thickness is proportional to the similarity between terms).

The upregulated DE genes are highly enriched in key pathways such as vacuole, regulated exocytosis, plasma membrane-bounded cell projection morphogenesis, actin filament-based process, and regulation of cell adhesion with a *p* value <10^−10^ (Figure 2). On the other hand, downregulated DE genes are highly enriched in adherens junction, MYC targets, RNA processing, and cell cycle-related pathways with a *p* value <10^−10^ (Figure 3).

### 3.2. Metabolic Pathway Analysis

**Figure 4:**
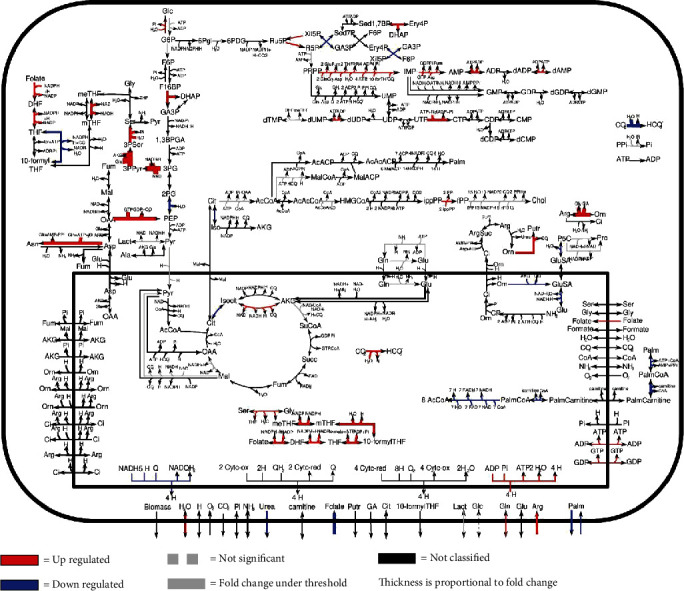
Adherent and suspension comparative metabolic map. Blue and red arrows refer, respectively, to downregulated and upregulated reactions. Dashed gray arrows refer to nonsignificant dysregulations according to the Kolmogorov-Smirnov test with *p* value 0.01. Solid gray arrows refer to reactions with a variation lower than 20%.

The extraction of metabolic genes and their scoring based on their relation with established metabolic pathways revealed an upregulation of the metabolic pathway of gluconeogenesis, galactose, pyrimidine, glycine, and threonine, but also the serine pathway which plays a key role in unrestrained cell cycle progression [22], the asparagine metabolism pathway that is linked to cells adaptation to nutrient deprivation and/or hypoxia [23] and the mitochondrial one-carbon metabolism pathway which is implicated in rapid cell proliferation [24]. On the other hand, the adaptation to suspension led to a downregulation of key metabolic pathways such as proline, folate, aspartate, lipids, and glycolytic pathways (Figure 4, Supplementary table S2). Notably, deficiencies in the folate metabolism pathway were linked to growth limitations of BHK-21 in suspension culture [25].

### 3.3. Gene Set Enrichment and Network Topology Analysis

**Figure 5:**
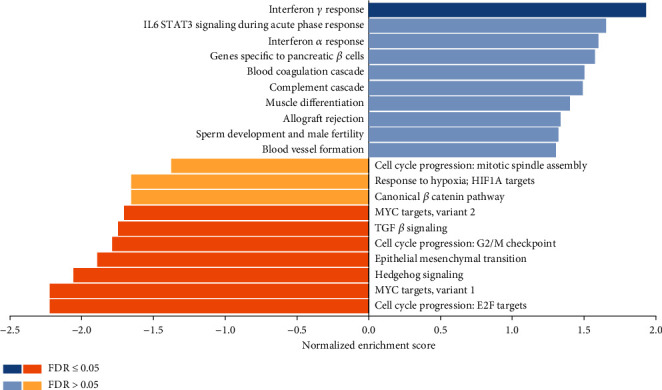
GSEA bar chart with significantly enriched hallmark pathways highlighted (FDR < 0.05) (IL6: interleukin-6; STAT3: signal transducer and activator of transcription 3; HIF1A: hypoxia-inducible factor-1*α*; MYC: cellular myelocytomatosis oncogene; TGF: transforming growth factor; G2/M: Second growth phase/mitosis; E2F: eukaryote cellular transcription factor; FDR: false discovery rate).

Gene set enrichment analysis (GSEA) with the hallmark gene set showed a significant upregulation of the interferon gamma response pathway and a downregulation of MYC targets (variants 1 and 2), E2F target pathways, but most importantly, the epithelial mesenchymal transition pathway (Figure 5). Only pathways with an FDR (false discovery rate) < 0.05 were considered.

**Figure 6:**
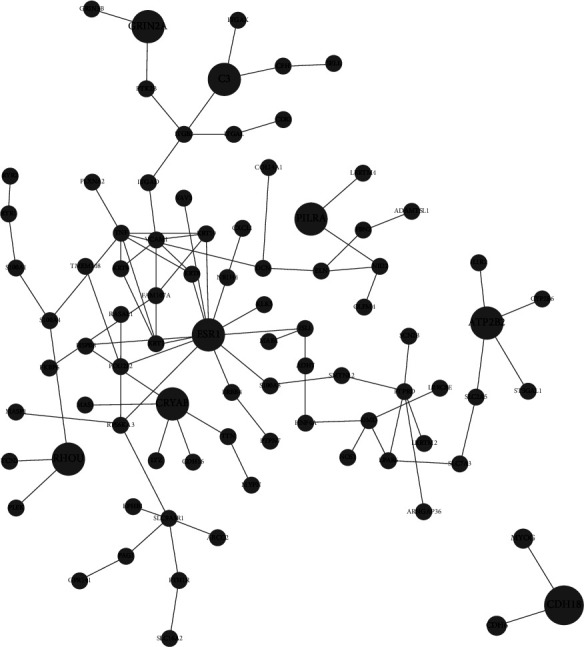
Layout of key upregulated subnetworks and their top associated genes (network details in Table 1).

**Figure 7:**
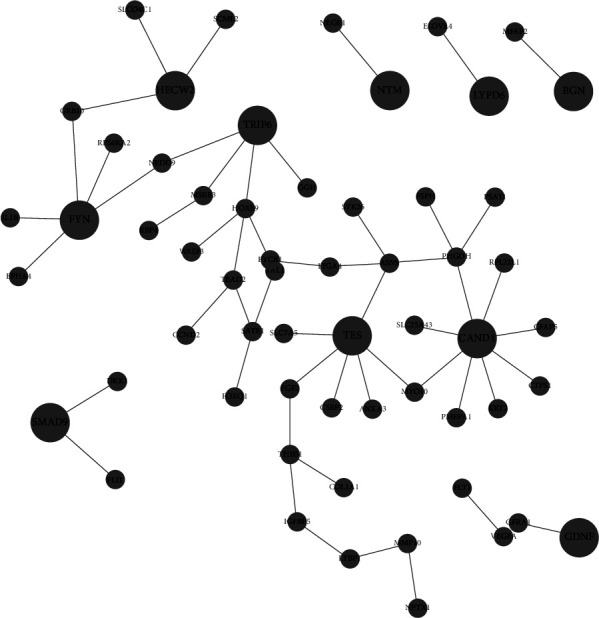
Layout of key downregulated subnetworks and their top-associated genes (network details in Table 2).

In order to identify key genes involved in protein-protein interaction (PPI) networks, network topology analysis (NTA) was done for both upregulated genes (Table 1, Figure 6) and downregulated genes (Table 2, Figure 7). The signaling response to chemical and anatomical structure pathway associated with CRYAB, RHOU, ESR1, and C3 are upregulated alongside the cell adhesion pathway which is associated with CDH18. On the other hand, pathways associated with FYN and SMAD9 (cell differentiation, system development, cellular response to growth factor) and the positive regulation of cell migration pathway associated with TRIP6 are downregulated.

## 4. Discussion

Over the years, significant efforts have been made to adapt Vero cells to suspension in order to engineer high-throughput and scalable vaccine production platforms; however, those efforts were limited by hurdles such as low cell viability and long doubling time. In order to design more efficient strategies for successful adaptation to suspension cultures maintaining acceptable cell viability and doubling time, it is necessary to better understand the genetic and phenotypic changes triggered by the adaptation to the suspension state. Thus, we propose in this paper a comparative functional genomics analysis of adherent and suspension Vero cells from the gene to the protein interaction network level.

Indeed, correlations between differential expression analysis (Figures 2 and 3), metabolic pathway enrichment analyses (Figure 4), gene set enrichment analyses (Figure 5), and network topology analyses (Tables 1 and 2) highlight key events such as the upregulation of immune response, exocytosis, and vacuole pathways which are involved in the storage of waste and other exocytosis molecules.

Furthermore, several events of checks and balances were observed across the different analysis methods used (Table 3). Lipid metabolism and lipid pathways are also upregulated via pathway enrichment, while the metabolic analysis revealed a downregulation of CPT1 and palmitate-associated metabolic reactions which regulate fatty acid oxidation in mitochondria and whose upregulation impairs glucose homeostasis [26]. In parallel, the gluconeogenesis metabolic pathway is upregulated while the glycolytic pathway is downregulated, thus hindering ATP generation. That resulting stress is met with not only a downregulation of the proline metabolic pathway which constitutes a checkpoint that is reported to promote proline accumulation during stress [27] but also an upregulation of asparagine metabolism which promotes the cell adaptation to nutrient deprivation and/or hypoxia [23], thus promoting cell survival.

Surprisingly, as observed in HEK293 cells adapted to suspension [28], cellular component organization pathways associated with cell adhesion such as actin filament-based process, regulation of cell adhesion, apical part of the cell, plasma membrane-bounded cell projection morphogenesis, and extracellular matrix are upregulated via pathway enrichment analysis, while NTA showed an upregulation of the cell adhesion PPI network with CDH18 as its central gene and which can be considered as possible target for engineering in order to improve the cell adaptation to suspension. This upregulation of cell adhesion-related genes could be due to the cells' attempt to restore the attachment to culture surfaces and surrounding cells which could explain the aggregates that are often observed in Vero cell suspension cultures and the cell rings that form on the suspension culture dishes.

On the other hand, the adherens junction pathway, which regulates cell-cell adhesion and is essential for viability (via the control of cell proliferation, polarity, shape, motility, and survival) [29], is downregulated alongside the aspartate metabolic pathway, the mitochondrial 1-carbon metabolic pathway, the MYC, and E2F target pathway, which could explain the low cell density observed during Vero cells adaptation to suspension.

Moreover, the pathways related to cell division and mitotic cell cycle phase transition are downregulated via pathway enrichment, which is confirmed by the downregulation of the folate metabolic pathway which leads to cell cycle arrest at G0/G1 [30], and the downregulation of FYN which is central to the networks associated with the control of cell growth, thus providing some insight in the origin of the long doubling time observed in Vero cells adapted to suspension culture. Nonetheless, Vero cells attempt to balance that effect on doubling time via the upregulation of the glycine, threonines and serine pathways which are associated with an unrestrained cell cycle progression [22], and the upregulation of RHOU and ESR1 which are central to the anatomical structure morphogenesis pathway and more precisely cell proliferation and antiapoptotic regulations.

In this study, we presented possible gene candidates for CRISPR/Cas gene editing in order to reduce the tendency of Vero cells adapted to suspension to upregulate their adhesion-related pathways. Inspired by Ley et al. AA catabolism engineering in CHO cells using CRISPR/Cas9 [31], several culture conditions of suspension Vero cells with a variation in amino acid intake (based on the results obtain from our metabolic pathways analysis and more precisely the regulation of the serine, threonine, and glycine pathway) can be sampled and analyzed via transcriptomics to highlight key gene targets for gene editing, thus combining medium development and gene engineering to improve the growth and fitness of Vero cells adapted to suspension. The results reported by Ley et al. show that this strategy might be also promising for Vero cells, but a prior, more in-depth genomic analysis for the specific case of Vero cells is necessary before drawing any conclusions.

Lastly, the epithelial-to-mesenchymal transition (EMT) pathway is downregulated in Vero cells adapted to suspension, thus highlighting the fact that the adaptation to suspension is not associated with EMT as previously shown with HEK293 cells adapted to suspension [28].

Based on our findings, we speculated that the following avenues could be taken to improve the adaptation of Vero cells to suspension via media formulation: (i) reducing cell aggregates by increasing calcium and magnesium intake; (ii) reducing cell doubling time by increasing the glycine, threonine, serine, and folate intake; and (iii) increasing cell density by increasing aspartate and glucose intake. Then the following media formulation was shown to improve the adaptation to suspension by improving the viable cell density and the doubling time while reducing cell aggregates [32]. Additional improvements can also be done by combining media formulation and gene editing targeting the genes highlighted in the network topology analysis (Table 4).

To conclude, we present in this paper key gene pathways at play during the adaptation of Vero cells to suspension and their complex checks and balances which could assist in the successful adaptation of Vero cells to suspension. Indeed, those key genes, notably associated with cell adhesion, hindering cell viability or doubling time, could be potentially targeted via gene editing as new strategies for the adaptation to suspension, but also the observed competitions between the regulation of competing pathways can be studied more in detail via targeted perturbations using gene editing tools such as CRISPR [33].

## Figures and Tables

**Table 1:** Key upregulated networks and their top-associated genes (NTA) (subnetwork layouts in Figure 6).

Pathway GO ID	Pathway GO name	Adjusted *p*-value	Top-ranking associated genes
GO:0042221	Response to chemical	1.4515*e*-9	CRYAB, RHOU, ESR1, GRIN2A
GO:0048731	System development	1.4515*e*-9	CRYAB, ESR1, C3, GRIN2A
GO:0023052	Signaling	5.052*e*-9	PILRA, CRYAB, RHOU, ESR1, C3, GRIN2A, ATP2B2
GO:0009605	Response to external stimulus	5.052*e*-9	ESR1, C3, GRIN2A
GO:0009653	Anatomical structure morphogenesis	5.052*e*-9	CRYAB, RHOU, ESR1, C3
GO:0007154	Cell communication	1.5582*e*-8	PILRA, CRYAB, RHOU, ESR1, C3, GRIN2A
GO:0007155	Cell adhesion	1.7218*e*-8	CDH18

**Table 2:** Key downregulated networks and their top-associated genes (NTA) (subnetwork layouts in Figure 7).

Pathway GO ID	Pathway GO name	Adjusted *p*-value	Top-ranking associated genes
GO:0009653	Anatomical structure morphogenesis	8.6426*e*-8	FYN, GDNF, HECW2
GO:0007167	Enzyme-linked receptor protein signaling pathway	0.0000018879	FYN, SMAD9
GO:0030154	Cell differentiation	0.0000030284	CAND1, FYN, GDNF, HECW2, NMT
GO:0048731	System development	0.0000031743	FYN, GDNF, HECW2, NMT
GO:0030335	Positive regulation of cell migration	0.0004	TRIP6
GO:0071363	Cellular response to growth factor stimulus	0.0002	FYN, SMAD9
GO:0045860	Positive regulation of protein kinase activity	0.0001	FYN

**Table 3:** Correlation between analysis methods for key findings. PEA: pathway enrichment analysis; MPA: metabolic pathway analysis; GSEA: gene set enrichment analysis; NTA: network topology-based analysis.

Key finding	Analysis methods confirming finding
Lipid metabolism	(i) PEA: lipid metabolism and lipid pathways upregulated (ii) MPA: downregulation of CPT1 and palmitate associated metabolic reactions
Response to stress	(i) PEA: response to wounding pathway upregulated (ii) MPA: downregulation of the proline metabolic pathway and upregulation of asparagine metabolism
Low cell density	(i) PEA: adherens junction, MYC, and E2F pathways downregulated (ii) MPA: aspartate metabolic pathway and the mitochondrial 1-carbon metabolic pathway downregulated (iii) GSEA: MYC and E2F pathways downregulated
Long doubling time	(i) PEA: cell division and mitotic cell cycle phase transition pathways downregulated (ii) MA: folate metabolic pathway downregulated (iii) GSEA: G2/M checkpoint downregulated (iv) NTA: FYN-related PPI networks downregulated
Attempt to balance long doubling time	(i) MPA: glycine, threonine, and serine pathways upregulated (ii) NTA: RHOU- and ESR1-related PPI networks upregulated
Upregulation of cell adhesion	(i) PEA: actin filament-based process, regulation of cell adhesion, apical part of cell, plasma membrane-bounded cell projection morphogenesis, and extracellular matrix pathways upregulated (ii) NTA: upregulation of the cell adhesion PPI network(CDH18)

**Table 4:** Proposed strategies for Vero cell adaptation to suspension improvement.

Phenotype observed	Proposed improvement
Long doubling time	(i) Gene editing: targeted knock-out of FYN gene (ii) Gene editing: enhance upregulation of RHOU and ESR1 (iii) Medium formulation: upregulation of glycine, threonine, and serine intake (iv) Medium formulation: reduce folate intake
Low cell density	(i) Gene editing: enhance upregulation of adherens junction-related genes (ii) Gene editing: enhance upregulation of MYC and E2F (iii) Medium formulation: upregulation aspartate intake (iv) Medium formulation: upregulate mitochondrial 1-carbon metabolism
Adhesion	(i) Gene editing: targeted knock-out of CDH18

## Data Availability

The Vero genome assembly and generated RNA sequencing reads are accessible under the Bioproject PRJNA644395. All other relevant data are available upon request.

## Supplementary Materials

Supplementary Materials Two supplementary materials are made available. Supplementary Table S1: compiling the differentially expressed gene list. Supplementary Table S2: containing the raw results from metabolic analysis.

## References

[1] Suder E., Furuyama W., Feldmann H., Marzi A., de Wit E. (2018). The vesicular stomatitis virus-based Ebola virus vaccine: from concept to clinical trials. *Human Vaccines & Immunotherapeutics*.

[2] COVID-19 vaccine tracker and landscape. https://www.who.int/teams/blueprint/covid-19/covid-19-vaccine-tracker-and-landscape.

[3] Kiesslich S., Kamen A. A. (2020). Vero cell upstream bioprocess development for the production of viral vectors and vaccines. *Biotechnology Advances*.

[4] Madeline B., Ribaud S., Xenopoulos A., Simler J., Schwamborn K., Léon A. (2015). Culturing a duck es-derived cell line in single-use bioreactors: a rapid, efficient, and cost-effective vaccine manufacturing system based on suspension culture. *Bioprocess International*.

[5] Peschel B., Frentzel S., Laske T., Genzel Y., Reichl U. (2013). Comparison of influenza virus yields and apoptosis-induction in an adherent and a suspension MDCK cell line. *Vaccine*.

[6] Yang J., Guertin P., Jia G., Lv Z., Yang H., Ju D. (2019). Large-scale microcarrier culture of HEK293T cells and Vero cells in single-use bioreactors. *AMB Express*.

[7] Merten O. W. (2002). Development of serum-free media for cell growth and production of viruses/viral vaccines: safety issues of animal products used in serum-free media. *Developmental Biology*.

[8] Chen A., Poh S. L., Dietzsch C., Roethl E., Yan M. L., Ng S. K. (2011). Serum-free microcarrier based production of replication deficient influenza vaccine candidate virus lacking NS1 using Vero cells. *BMC Biotechnology*.

[9] Rourou S., Ben Ayed Y., Trabelsi K., Majoul S., Kallel H. (2014). An animal component free medium that promotes the growth of various animal cell lines for the production of viral vaccines. *Vaccine*.

[10] Shen C. F., Guilbault C., Li X. (2019). Development of suspension adapted Vero cell culture process technology for production of viral vaccines. *Vaccine*.

[11] Logan M., Aucoin M. Media formulation to support the growth of Vero cells in suspension.

[12] Sène M. A., Kiesslich S., Djambazian H., Ragoussis J., Xia Y., Kamen A. A. (2021). Haplotype-resolved de novo assembly of the Vero cell line genome. *NPJ Vaccines*.

[13] Dobin A., Davis C. A., Schlesinger F. (2013). STAR: ultrafast universal RNA-seq aligner. *Bioinformatics*.

[14] Li H., Handsaker B., Wysoker A. (2009). The sequence alignment/map format and SAMtools. *Bioinformatics*.

[15] Liao Y., Smyth G. K., Shi W. (2014). featureCounts: an efficient general purpose program for assigning sequence reads to genomic features. *Bioinformatics*.

[16] Love M. I., Huber W., Anders S. (2014). Moderated estimation of fold change and dispersion for RNA-seq data with DESeq2. *Genome Biology*.

[17] Zhou Y., Zhou B., Pache L. (2019). Metascape provides a biologist-oriented resource for the analysis of systems-level datasets. *Nature Communications*.

[18] Damiani C., Rovida L., Maspero D. (2020). MaREA4Galaxy: metabolic reaction enrichment analysis and visualization of RNA- seq data within Galaxy. *Computational and Structural Biotechnology Journal*.

[19] Liao Y., Wang J., Jaehnig E. J., Shi Z., Zhang B. (2019). WebGestalt 2019: gene set analysis toolkit with revamped UIs and APIs. *Nucleic Acids Research*.

[20] Stark C., Breitkreutz B. J., Reguly T., Boucher L., Breitkreutz A., Tyers M. (2006). BioGRID: a general repository for interaction datasets. *Nucleic Acids Research*.

[21] Amelio I., Cutruzzolá F., Antonov A., Agostini M., Melino G. (2014). Serine and glycine metabolism in cancer. *Trends in Biochemical Sciences*.

[22] Balasubramanian M. N., Butterworth E. A., Kilberg M. S. (2013). Asparagine synthetase: regulation by cell stress and involvement in tumor biology. *American Journal of Physiology. Endocrinology and Metabolism*.

[23] Nilsson R., Jain M., Madhusudhan N. (2014). Metabolic enzyme expression highlights a key role for MTHFD2 and the mitochondrial folate pathway in cancer. *Nature Communications*.

[24] Neugut A., Weinstein I. (1979). Growth limitation of BHK-21 cells and its relation to folate metabolism. *In Vitro*.

[25] Assimacopoulos-Jeannet F., Thumelin S., Roche E., Esser V., McGarry J. D., Prentki M. (1997). Fatty acids rapidly induce the carnitine palmitoyltransferase I gene in the pancreatic *β*-cell line INS-1. *The Journal of Biological Chemistry*.

[26] Liang X., Zhang L., Natarajan S. K., Becker D. F. (2013). Proline mechanisms of stress survival. *Antioxidants & Redox Signaling*.

[27] Malm M., Saghaleyni R., Lundqvist M. (2020). Evolution from adherent to suspension: systems biology of HEK293 cell line development. *Scientific Reports*.

[28] Ivanov A. I., Naydenov N. G. (2013). Dynamics and regulation of epithelial adherens junctions: recent discoveries and controversies. *International Review of Cell and Molecular Biology*.

[29] Yang Y., Li X., Sun Q. (2016). Folate deprivation induces cell cycle arrest at G0/G1 phase and apoptosis in hippocampal neuron cells through down-regulation of IGF-1 signaling pathway. *The International Journal of Biochemistry & Cell Biology*.

[30] Ley D., Pereira S., Pedersen L. E. (2019). Reprogramming AA catabolism in CHO cells with CRISPR/Cas9 genome editing improves cell growth and reduces byproduct secretion. *Metabolic Engineering*.

[31] Logan M. (2022). Development of a serum-free chemically defined medium for adherent and suspension culture. http://hdl.handle.net/10012/17844.

[32] Adli M. (2018). The CRISPR tool kit for genome editing and beyond. *Nature Communications*.

[33] Sène M.-A., Xia Y., Kamen A. (2021). Comparative transcriptomics analyses of a Vero cell line in suspension versus adherent culture conditions.

