# Supplementary material for: Comparative Transcriptomic Analyses of a Vero Cell Line in Suspension versus Adherent Culture Conditions
Source: Int J Cell Biol. 2023 Aug 30;2023:9364689. doi: 10.1155/2023/9364689 (PMC10482560; doi:10.1155/2023/9364689)
Supplement: Supplementary Materials — Two supplementary materials are made available. Supplementary Table S1: compiling the differentially expressed gene list. Supplementary Table S2: containing the raw results from metabolic analysis. [file 9364689.f1.zip › Supplementary_table_S1_Differentially_expressed_genes.pdf]

	Gene	baseMean	log2FoldCh	lfcSE	stat	pvalue	padj	Adh_A	Adh_B	Adh_C	Susp_A	Susp_B	Susp_C
14	OLFM4	11425.84	9.605845	0.162528	59.10277	0	0	18.43094	35.58177	34.78657	22444.04	22024.28	23997.9
752	TINAG	245.6462	9.296898	0.746108	12.46053	1.23E-35	4.33E-34	0	2.189647	0	473.2109	474.8826	523.5943
1774	S100A8	101.3059	9.009863	1.057069	8.523438	1.55E-17	2.32E-16	0.542086	0.547412	0	203.1236	226.7457	176.8764
1157	PCSK2	147.3753	8.969103	0.871282	10.29415	7.49E-25	1.72E-23	0	0.547412	1.391463	218.7484	320.8666	342.698
2718	LOC11961	24.38332	8.80018	1.29128	6.815085	9.42E-12	9.21E-11	0	0	0	58.0353	47.06043	41.20416
962	TLR4	141.7974	8.505927	0.762736	11.15186	7.01E-29	1.94E-27	1.084173	0.547412	0.695731	245.534	265.2497	337.6731
3102	LINGO4	27.3629	8.019831	1.277926	6.275663	3.48E-10	2.98E-09	0	0.547412	0	49.10679	49.19955	65.32367
165	LOC11962	445.0497	7.976744	0.373542	21.35436	3.55E-101	5.72E-99	3.794605	3.284471	3.478657	935.2612	797.8883	926.5911
3983	LOC10322	11.85902	7.741729	1.437259	5.386454	7.19E-08	4.80E-07	0	0	0	29.01765	32.08666	10.04979
3993	LOC11962	11.08743	7.652298	1.422354	5.380025	7.45E-08	4.96E-07	0	0	0	26.78552	25.66933	14.06971
78	DTX4	689.7059	7.632218	0.290228	26.29733	2.06E-152	7.01E-150	3.252518	5.474118	13.2189	1415.169	1210.737	1490.385
4154	ZNF552	10.6534	7.598905	1.446306	5.254011	1.49E-07	9.52E-07	0	0	0	15.62489	34.22577	14.06971
4081	SLC51B	10.37827	7.583856	1.427614	5.312261	1.08E-07	7.05E-07	0	0	0	15.62489	23.53022	23.11453
4369	LOC11962	9.998912	7.499648	1.470415	5.10036	3.39E-07	2.06E-06	0	0	0	17.85702	32.08666	10.04979
4620	LOC10322	9.604941	7.434555	1.506173	4.936055	7.97E-07	4.59E-06	0	0	0	33.4819	17.11289	7.034856
4688	F11	9.087373	7.36273	1.505098	4.891862	9.99E-07	5.66E-06	0	0	0	13.39276	32.08666	9.044815
5057	GABRG1	8.962927	7.336224	1.575203	4.657319	3.20E-06	1.68E-05	0	0	0	13.39276	36.36488	4.019918
5091	LPL	8.417387	7.245737	1.563321	4.634837	3.57E-06	1.87E-05	0	0	0	13.39276	32.08666	5.024897
4980	LOC10323	8.25937	7.217483	1.536008	4.698858	2.62E-06	1.40E-05	0	0	0	17.85702	25.66933	6.029877
4963	CYP4B1	8.070348	7.185987	1.52695	4.706105	2.52E-06	1.35E-05	0	0	0	17.85702	23.53022	7.034856
4999	ITGBL1	8.054846	7.184161	1.532171	4.688876	2.75E-06	1.46E-05	0	0	0	15.62489	25.66933	7.034856
5030	LOC10323	7.943337	7.164587	1.533116	4.673218	2.97E-06	1.57E-05	0	0	0	26.78552	12.83466	8.039836
5106	PTPRZ1	7.698327	7.119087	1.539209	4.625159	3.74E-06	1.95E-05	0	0	0	15.62489	23.53022	7.034856
5507	DCN	7.242346	7.023323	1.59318	4.408369	1.04E-05	5.03E-05	0	0	0	22.32127	17.11289	4.019918
5347	TMEM273	7.22082	7.023075	1.564214	4.489841	7.13E-06	3.54E-05	0	0	0	22.32127	14.97377	6.029877
4545	LOC11961	14.14509	7.020664	1.409616	4.98055	6.34E-07	3.71E-06	0	0	0.695731	33.4819	40.6431	10.04979
5246	FAM81B	7.168289	7.019072	1.540988	4.554918	5.24E-06	2.66E-05	0	0	0	17.85702	17.11289	8.039836
5515	MAGEB10	7.21134	7.018101	1.593187	4.40507	1.06E-05	5.10E-05	0	0	0	17.85702	21.39111	4.019918
5605	LOC10323	7.180335	7.013856	1.604529	4.371287	1.24E-05	5.86E-05	0	0	0	13.39276	25.66933	4.019918
2	SYT6	1734.788	6.996363	0.162158	43.14529	0	0	31.44101	24.08612	25.04633	3401.762	3396.908	3529.488
5511	LOC10324	7.006815	6.979101	1.583596	4.407122	1.05E-05	5.05E-05	0	0	0	15.62489	21.39111	5.024897
28	RORC	1056.123	6.97492	0.200571	34.77526	5.75E-265	5.46E-262	16.80468	14.23271	19.48048	2053.557	1942.312	2290.348
4467	DIPK1C	13.51628	6.962877	1.383845	5.031544	4.87E-07	2.90E-06	0	0.547412	0	31.24978	34.22577	15.07469
5306	LOC11962	6.753218	6.950471	1.539683	4.514223	6.35E-06	3.18E-05	0	0	0	15.62489	12.83466	12.05975
359	KCND2	181.2872	6.938544	0.426398	16.27246	1.55E-59	1.15E-57	3.252518	2.189647	3.478657	337.0512	350.8141	390.937
734	FAXDC2	110.3051	6.931597	0.550601	12.58915	2.42E-36	8.78E-35	0.542086	0.547412	4.87012	229.9091	213.9111	212.0507
5626	GPR149	6.597766	6.900418	1.582296	4.361016	1.29E-05	6.12E-05	0	0	0	11.16063	21.39111	7.034856
6063	LOC11962	6.625314	6.898716	1.66627	4.140214	3.47E-05	0.000152	0	0	0	8.928508	27.80844	3.014938
732	CCL2	104.9757	6.877118	0.546133	12.5924	2.33E-36	8.44E-35	0.542086	2.189647	2.782925	209.8199	207.4937	207.0258
5865	LOC10324	6.529309	6.873851	1.623173	4.234825	2.29E-05	0.000104	0	0	0	22.32127	12.83466	4.019918
6054	LOC11962	6.315304	6.825164	1.647736	4.142146	3.44E-05	0.000151	0	0	0	15.62489	19.252	3.014938
6055	LOC10324	6.315304	6.825164	1.647736	4.142146	3.44E-05	0.000151	0	0	0	15.62489	19.252	3.014938
5743	MNDA	6.256751	6.823566	1.586113	4.302067	1.69E-05	7.83E-05	0	0	0	13.39276	17.11289	7.034856
6087	DUX4	6.299801	6.822609	1.65301	4.127385	3.67E-05	0.00016	0	0	0	13.39276	21.39111	3.014938
6088	NLRP8	6.299801	6.822609	1.65301	4.127385	3.67E-05	0.00016	0	0	0	13.39276	21.39111	3.014938
5789	MLANA	6.241248	6.821145	1.593528	4.280531	1.86E-05	8.56E-05	0	0	0	11.16063	19.252	7.034856
1844	LOC10321	44.77034	6.806983	0.81728	8.328831	8.16E-17	1.18E-15	0	0.547412	2.087194	84.82083	87.70354	93.46309
5881	NFILZ	6.092712	6.797297	1.608814	4.225037	2.39E-05	0.000108	0	0	0	20.08914	6.417332	10.04979
6344	HTR3A	6.132305	6.782529	1.691983	4.008628	6.11E-05	0.000256	0	0	0	13.39276	21.39111	2.009959
6046	IL22RA2	6.110779	6.780377	1.635507	4.145734	3.39E-05	0.000149	0	0	0	13.39276	19.252	4.019918
6096	RCVRN	6.095277	6.777986	1.643935	4.123027	3.74E-05	0.000163	0	0	0	11.16063	21.39111	4.019918
5979	PRR9	6.058249	6.776595	1.624215	4.172228	3.02E-05	0.000134	0	0	0	8.928508	21.39111	6.029877
6277	LOC10323	6.064271	6.774172	1.677426	4.038431	5.38E-05	0.000228	0	0	0	6.696381	25.66933	4.019918
3581	LST1	21.68797	6.753314	1.169637	5.773854	7.75E-09	5.75E-08	0	0.547412	0.695731	62.49956	34.22577	32.15934
136	LOC10321	388.014	6.752322	0.295487	22.85149	1.41E-115	2.76E-113	3.252518	6.02153	13.2189	776.7802	752.967	775.8442
6841	LOC10324	5.933803	6.737636	1.772262	3.801717	0.000144	0.000558	0	0	0	8.928508	25.66933	1.004979
6159	LOC11962	5.890752	6.733136	1.64486	4.09344	4.25E-05	0.000183	0	0	0	8.928508	21.39111	5.024897
6267	LOC11961	5.875249	6.731227	1.664746	4.043396	5.27E-05	0.000223	0	0	0	6.696381	23.53022	5.024897
6051	LOC10323	5.646634	6.71065	1.619592	4.143419	3.42E-05	0.00015	0	0	0	13.39276	6.417332	14.06971
3913	IRF6	21.20265	6.705584	1.233649	5.435571	5.46E-08	3.71E-07	0	0.547412	0.695731	40.17829	72.72976	13.06473
6255	ELF5	5.800769	6.703805	1.656311	4.047432	5.18E-05	0.00022	0	0	0	20.08914	10.69555	4.019918
5941	LOC11961	5.674182	6.699599	1.599195	4.189356	2.80E-05	0.000125	0	0	0	11.16063	12.83466	10.04979
6537	LOC11962	5.775786	6.695326	1.705828	3.924971	8.67E-05	0.000353	0	0	0	13.39276	19.252	2.009959
6075	LOC11961	5.732736	6.695119	1.619586	4.133846	3.57E-05	0.000156	0	0	0	13.39276	14.97377	6.029877
6352	LOC11961	5.723255	6.688641	1.669628	4.006067	6.17E-05	0.000258	0	0	0	8.928508	21.39111	4.019918
6281	LOC11961	5.686227	6.687723	1.65653	4.037189	5.41E-05	0.000229	0	0	0	6.696381	21.39111	6.029877
6317	IGFL4	5.611747	6.659907	1.657489	4.018071	5.87E-05	0.000247	0	0	0	20.08914	8.556443	5.024897
6143	NHLH2	5.559216	6.65513	1.623155	4.10012	4.13E-05	0.000179	0	0	0	15.62489	10.69555	7.034856
607	LOC11962	118.4742	6.65307	0.491654	13.53203	1.01E-41	4.43E-40	3.252518	3.284471	0	238.8376	252.4151	213.0556
440	LOC10324	149.606	6.650074	0.437291	15.20742	3.16E-52	1.91E-50	1.626259	2.737059	4.87012	303.5693	265.2497	319.5835
5006	MS4A7	10.64622	6.645385	1.418241	4.685653	2.79E-06	1.48E-05	0	0	0.695731	13.39276	25.66933	24.11951
5510	NOX5	10.91427	6.64303	1.507234	4.407432	1.05E-05	5.05E-05	0	0.547412	0	24.5534	36.36488	4.019918
5219	GADL1	10.76893	6.62232	1.449041	4.570141	4.87E-06	2.48E-05	0	0	0.695731	33.4819	21.39111	9.044815

1230	MIOX	287.6528	6.618252	0.657146	10.0712	7.41E-24	1.60E-22	4.336691	1.642235	11.82743	551.3354	483.439	673.3362
5349	MYH1	10.67272	6.612944	1.472922	4.489676	7.13E-06	3.55E-05	0	0.547412	0	20.08914	36.36488	7.034856
6224	NT5C1B	5.333167	6.612241	1.62672	4.064768	4.81E-05	0.000205	0	0	0	13.39276	8.556443	10.04979
6465	PTN	5.428748	6.608733	1.671778	3.953116	7.71E-05	0.000317	0	0	0	17.85702	10.69555	4.019918
6488	LOC119618	5.38224	6.598537	1.672428	3.945482	7.96E-05	0.000326	0	0	0	11.16063	17.11289	4.019918
6375	LOC119620	5.345212	6.597783	1.65252	3.992559	6.54E-05	0.000273	0	0	0	8.928508	17.11289	6.029877
6	LOC103218	21930.51	6.585508	0.088827	74.13822	0	0	454.8105	466.9423	433.4406	43144.78	41789.67	45293.42
5289	LOC119625	10.40639	6.577078	1.453859	4.523877	6.07E-06	3.05E-05	0	0	0.695731	35.71403	14.97377	11.05477
5221	P2RX7	10.33516	6.570129	1.437807	4.569548	4.89E-06	2.49E-05	0	0.547412	0	29.01765	21.39111	11.05477
6697	LOC119627	5.230246	6.553086	1.698951	3.857136	0.000115	0.000455	0	0	0	13.39276	14.97377	3.014938
6540	LOC103230	5.140687	6.548494	1.668767	3.924151	8.70E-05	0.000354	0	0	0	6.696381	17.11289	7.034856
6574	AGR2	5.177715	6.54812	1.674572	3.910323	9.22E-05	0.000373	0	0	0	8.928508	17.11289	5.024897
6677	LOC119621	5.162212	6.545632	1.692905	3.86651	0.00011	0.00044	0	0	0	6.696381	19.252	5.024897
4172	LOC119620	19.39827	6.54072	1.247114	5.244685	1.57E-07	9.98E-07	0	0	1.391463	33.4819	68.45154	13.06473
6716	CYP4F57	5.056727	6.507212	1.690454	3.849388	0.000118	0.000469	0	0	0	15.62489	10.69555	4.019918
6567	LOC103239	5.019699	6.506324	1.662814	3.912838	9.12E-05	0.000369	0	0	0	13.39276	10.69555	6.029877
6732	LOC103238	5.025721	6.499981	1.691031	3.843798	0.000121	0.000478	0	0	0	11.16063	14.97377	4.019918
6785	LOC103230	4.936163	6.497604	1.699527	3.823183	0.000132	0.000516	0	0	0	4.464254	17.11289	8.039836
6796	FGF20	5.010219	6.497007	1.701105	3.819286	0.000134	0.000524	0	0	0	8.928508	17.11289	4.019918
5390	MTRNR2L5	9.835862	6.493099	1.452976	4.468829	7.86E-06	3.88E-05	0	0	0.695731	29.01765	19.252	10.04979
7050	ECRG4	4.979213	6.492363	1.748775	3.71252	0.000205	0.000774	0	0	0	4.464254	21.39111	4.019918
7032	CHST9	4.89871	6.467386	1.737591	3.722043	0.000198	0.000747	0	0	0	20.08914	4.278221	5.024897
6690	S100A9	4.830677	6.457453	1.673014	3.859772	0.000113	0.000451	0	0	0	13.39276	8.556443	7.034856
5756	GRIN2A	9.581372	6.454649	1.502216	4.296753	1.73E-05	8.00E-05	0	0	0.695731	13.39276	36.36488	7.034856
6769	TTLL8	4.852202	6.453164	1.685767	3.82803	0.000129	0.000507	0	0	0	13.39276	10.69555	5.024897
6798	DYTN	4.784169	6.446949	1.688667	3.817774	0.000135	0.000527	0	0	0	6.696381	14.97377	7.034856
7042	CLDN14	4.842722	6.444499	1.734105	3.716327	0.000202	0.000763	0	0	0	8.928508	17.11289	3.014938
5661	LOC103242	9.32735	6.414509	1.477504	4.341451	1.42E-05	6.65E-05	0	0	0.695731	31.24978	14.97377	9.044815
7459	LOC119627	4.752739	6.412021	1.792019	3.578098	0.000346	0.001234	0	0	0	20.08914	6.417332	2.009959
5429	LOC103235	9.145038	6.40843	1.441259	4.446412	8.73E-06	4.28E-05	0	0.547412	0	17.85702	21.39111	15.07469
7117	LOC103222	4.715711	6.40764	1.736406	3.690173	0.000224	0.000837	0	0	0	17.85702	6.417332	4.019918
6836	RBP3	4.61065	6.399479	1.682558	3.803423	0.000143	0.000555	0	0	0	8.928508	10.69555	8.039836
6873	TBX22	4.647678	6.398097	1.689053	3.787979	0.000152	0.000587	0	0	0	11.16063	10.69555	6.029877
6906	LOC119625	4.632175	6.394494	1.694021	3.774743	0.00016	0.000617	0	0	0	8.928508	12.83466	6.029877
7005	LOC119622	4.669203	6.39444	1.7138	3.731148	0.000191	0.000723	0	0	0	11.16063	12.83466	4.019918
7347	LOC103248	4.690728	6.394131	1.767952	3.616688	0.000298	0.00108	0	0	0	11.16063	14.97377	2.009959
7051	OPRK1	4.6537	6.391037	1.721707	3.712036	0.000206	0.000775	0	0	0	8.928508	14.97377	4.019918
7413	INSL4	4.675225	6.390808	1.777643	3.595102	0.000324	0.001163	0	0	0	8.928508	17.11289	2.009959
7169	NLRP11	4.638197	6.388098	1.738522	3.674443	0.000238	0.000884	0	0	0	6.696381	17.11289	4.019918
7363	SOHLH1	4.622695	6.385648	1.767591	3.612628	0.000303	0.001094	0	0	0	4.464254	19.252	4.019918
5547	LOC103226	9.014569	6.378145	1.451955	4.392799	1.12E-05	5.36E-05	0	0.547412	0	20.08914	21.39111	12.05975
5802	FKBP6	8.973196	6.370329	1.491059	4.272351	1.93E-05	8.86E-05	0.542086	0	0	11.16063	32.08666	10.04979
4513	MYH13	16.83744	6.367245	1.272036	5.005552	5.57E-07	3.28E-06	0	0.547412	0.695731	24.5534	64.17332	11.05477
5608	MGAT4C	8.834764	6.349104	1.452903	4.369944	1.24E-05	5.89E-05	0	0	0.695731	17.85702	21.39111	13.06473
7805	LOC119618	4.554237	6.348653	1.832901	3.463718	0.000533	0.001815	0	0	0	15.62489	10.69555	1.004979
7316	LOC119626	4.517209	6.341797	1.747698	3.628658	0.000285	0.001035	0	0	0	13.39276	10.69555	3.014938
7088	LOC119621	4.480181	6.340505	1.712982	3.701444	0.000214	0.000804	0	0	0	11.16063	10.69555	5.024897
7834	LOC119625	4.523232	6.340239	1.834078	3.456907	0.000546	0.001854	0	0	0	11.16063	14.97377	1.004979
4342	RNASE11	16.81309	6.337267	1.23642	5.125497	2.97E-07	1.82E-06	0	0	1.391463	26.78552	55.61688	17.08465
7712	LOC119625	4.41817	6.328266	1.813917	3.488729	0.000485	0.001673	0	0	0	2.232127	19.252	5.024897
7994	CUNH7orf3	4.439695	6.326904	1.852925	3.41455	0.000639	0.002125	0	0	0	2.232127	21.39111	3.014938
4381	UPK3BL2	16.71362	6.32339	1.241708	5.092495	3.53E-07	2.14E-06	0	0	1.391463	33.4819	51.33866	14.06971
5946	LOC103236	8.731844	6.314554	1.507936	4.187547	2.82E-05	0.000126	0	0	0.695731	17.85702	27.80844	6.029877
6189	SLC30A8	8.608085	6.308879	1.546434	4.07963	4.51E-05	0.000194	0	0.547412	0	6.696381	36.36488	8.039836
8889	CUNH21or	4.38674	6.299335	2.001616	3.147125	0.001649	0.004931	0	0	0	15.62489	10.69555	0
7696	SLCO6A1	4.365215	6.288652	1.799578	3.494514	0.000475	0.00164	0	0	0	15.62489	8.556443	2.009959
7697	LOC103217	4.365215	6.288652	1.799578	3.494514	0.000475	0.00164	0	0	0	15.62489	8.556443	2.009959
6064	INSYN2B	8.595353	6.288472	1.519004	4.139864	3.48E-05	0.000152	0	0	0.695731	22.32127	23.53022	5.024897
7258	LOC103222	4.291159	6.285423	1.722071	3.649922	0.000262	0.000961	0	0	0	11.16063	8.556443	6.029877
7401	LOC119622	4.328187	6.285268	1.745944	3.599925	0.000318	0.001143	0	0	0	13.39276	8.556443	4.019918
7396	DMRT3	4.2016	6.284115	1.745045	3.60112	0.000317	0.001139	0	0	0	4.464254	10.69555	10.04979
7417	TMCO5A	4.297182	6.276924	1.746963	3.593049	0.000327	0.001171	0	0	0	8.928508	12.83466	4.019918
7726	LOC103234	4.318707	6.275783	1.801415	3.483807	0.000494	0.001701	0	0	0	8.928508	14.97377	2.009959
7497	LOC103241	4.244651	6.27418	1.761618	3.5616	0.000369	0.001307	0	0	0	4.464254	14.97377	6.029877
7498	LOC119625	4.244651	6.27418	1.761618	3.5616	0.000369	0.001307	0	0	0	4.464254	14.97377	6.029877
7500	LOC119625	4.281679	6.273514	1.761827	3.560801	0.00037	0.001311	0	0	0	6.696381	14.97377	4.019918
7671	LOC119623	4.266176	6.270628	1.790016	3.503114	0.00046	0.001594	0	0	0	4.464254	17.11289	4.019918
8057	LOC103227	4.287701	6.269824	1.84562	3.397137	0.000681	0.002247	0	0	0	4.464254	19.252	2.009959
4477	XIRP2	15.702	6.263699	1.246445	5.025249	5.03E-07	2.99E-06	0.542086	0	0.695731	42.41041	38.50399	12.05975
8297	LOC119625	4.213221	6.235845	1.881771	3.313818	0.00092	0.002949	0	0	0	17.85702	6.417332	1.004979
7461	LOC103219	4.065109	6.230311	1.741621	3.577306	0.000347	0.001237	0	0	0	8.928508	6.417332	9.044815
8234	LOC119626	4.197718	6.230242	1.867639	3.335892	0.00085	0.002745	0	0	0	15.62489	8.556443	1.004979
7780	LOC103241	4.176193	6.229064	1.794903	3.470417	0.00052	0.001776	0	0	0	15.62489	6.417332	3.014938
7468	LOC119627	4.049606	6.226095	1.74226	3.573574	0.000352	0.001254	0	0	0	6.696381	8.556443	9.044815

7439	LOC10321	4.086634	6.224301	1.736042	3.58534	0.000337	0.001203	0	0	0	8.928508	8.556443	7.034856
8222	LOC11962	4.166713	6.220665	1.862239	3.340423	0.000837	0.002705	0	0	0	11.16063	12.83466	1.004979
7488	LOC10323	4.071132	6.22016	1.743751	3.567115	0.000361	0.001281	0	0	0	6.696381	10.69555	7.034856
8264	LOC10323	4.15121	6.216583	1.869849	3.324645	0.000885	0.002848	0	0	0	8.928508	14.97377	1.004979
4530	LOC11961	15.11084	6.207831	1.242904	4.994616	5.90E-07	3.46E-06	0	0.547412	0.695731	35.71403	40.6431	13.06473
6191	DBH	8.065315	6.196574	1.519069	4.079191	4.52E-05	0.000194	0	0	0.695731	24.5534	17.11289	6.029877
6952	RGSL1	7.940869	6.171408	1.643453	3.755146	0.000173	0.000662	0	0	0.695731	24.5534	21.39111	1.004979
9326	LOC11962	4.014719	6.170697	2.04041	3.024243	0.002493	0.007105	0	0	0	13.39276	10.69555	0
8039	TMEM176	3.987171	6.168855	1.813412	3.401795	0.000669	0.002214	0	0	0	15.62489	4.278221	4.019918
7843	LOC10323	3.845082	6.161758	1.783464	3.454939	0.00055	0.001866	0	0	0	4.464254	8.556443	10.04979
9406	LOC11962	3.983714	6.16119	2.04968	3.005927	0.002648	0.007483	0	0	0	8.928508	14.97377	0
7673	CAPN13	3.919138	6.158747	1.758937	3.501403	0.000463	0.001604	0	0	0	8.928508	8.556443	6.029877
4427	LOC11962	14.48522	6.158198	1.217277	5.058995	4.21E-07	2.53E-06	0	0.547412	0.695731	33.4819	32.08666	20.09959
6090	LOC10323	7.731009	6.157857	1.492126	4.126901	3.68E-05	0.000161	0	0.547412	0	13.39276	21.39111	11.05477
7837	FRG2	3.866607	6.156118	1.781056	3.456442	0.000547	0.001857	0	0	0	4.464254	10.69555	8.039836
6134	CLEC10A	7.740226	6.156001	1.500533	4.102544	4.09E-05	0.000177	0	0	0.695731	11.16063	23.53022	11.05477
6373	LOC11962	7.8102	6.154942	1.541387	3.99312	6.52E-05	0.000272	0.542086	0	0	15.62489	25.66933	5.024897
7728	CYP8B1	3.903635	6.154466	1.766731	3.483533	0.000495	0.001702	0	0	0	6.696381	10.69555	6.029877
6411	LOC11962	7.820305	6.153213	1.548116	3.974647	7.05E-05	0.000292	0	0	0.695731	13.39276	27.80844	5.024897
8161	LOC10323	3.962188	6.151037	1.830142	3.360962	0.000777	0.00253	0	0	0	8.928508	12.83466	2.009959
7913	LOC10324	3.92516	6.149326	1.790497	3.434425	0.000594	0.001995	0	0	0	6.696381	12.83466	4.019918
8244	TASL	3.946686	6.147264	1.844645	3.332493	0.000861	0.002776	0	0	0	6.696381	14.97377	2.009959
8096	LOC11962	3.909658	6.145906	1.817133	3.382199	0.000719	0.002361	0	0	0	4.464254	14.97377	4.019918
362	KCND3	169.0222	6.131888	0.377424	16.24668	2.36E-59	1.73E-57	1.084173	5.474118	8.348776	319.1942	327.2839	352.7478
6212	LOC10324	7.514176	6.125635	1.505742	4.068183	4.74E-05	0.000203	0	0	0.695731	8.928508	21.39111	14.06971
6360	LY6K	7.622066	6.121102	1.530431	3.999593	6.35E-05	0.000265	0	0.547412	0	15.62489	23.53022	6.029877
6361	DCAF4L2	7.622066	6.121102	1.530431	3.999593	6.35E-05	0.000265	0	0.547412	0	15.62489	23.53022	6.029877
6336	KCNJ3	7.47353	6.100077	1.520893	4.010853	6.05E-05	0.000254	0	0.547412	0	24.5534	10.69555	9.044815
8679	LOC10323	3.825697	6.095769	1.899838	3.208573	0.001334	0.004086	0	0	0	13.39276	8.556443	1.004979
8226	FCRL3	3.804172	6.095558	1.825646	3.33885	0.000841	0.002719	0	0	0	13.39276	6.417332	3.014938
8006	SLN	3.751641	6.090171	1.785823	3.410288	0.000649	0.002155	0	0	0	8.928508	8.556443	5.024897
8171	LOC10323	3.788669	6.090095	1.815241	3.354979	0.000794	0.002582	0	0	0	11.16063	8.556443	3.014938
8362	OLIG1	3.662083	6.087808	1.847929	3.294394	0.000986	0.003136	0	0	0	2.232127	10.69555	9.044815
8110	LOC10322	3.699111	6.087492	1.802819	3.376652	0.000734	0.002405	0	0	0	4.464254	10.69555	7.034856
8067	AKNAD1	3.736139	6.085727	1.793691	3.392852	0.000692	0.002279	0	0	0	6.696381	10.69555	5.024897
8068	KCNS2	3.736139	6.085727	1.793691	3.392852	0.000692	0.002279	0	0	0	6.696381	10.69555	5.024897
8069	LOC11962	3.736139	6.085727	1.793691	3.392852	0.000692	0.002279	0	0	0	6.696381	10.69555	5.024897
8187	LOC11962	3.773167	6.085321	1.816038	3.350879	0.000806	0.002616	0	0	0	8.928508	10.69555	3.014938
8188	CLEC4D	3.773167	6.085321	1.816038	3.350879	0.000806	0.002616	0	0	0	8.928508	10.69555	3.014938
6550	LOC11962	7.411256	6.082138	1.551763	3.919501	8.87E-05	0.00036	0	0	0.695731	8.928508	27.80844	7.034856
8954	LOC11961	3.763686	6.078264	1.942073	3.129781	0.001749	0.005193	0	0	0	4.464254	17.11289	1.004979
8955	LOC11962	3.763686	6.078264	1.942073	3.129781	0.001749	0.005193	0	0	0	4.464254	17.11289	1.004979
8416	LOC11961	3.742161	6.077659	1.854419	3.277393	0.001048	0.00331	0	0	0	4.464254	14.97377	3.014938
8755	PSKH2	3.726658	6.075044	1.906716	3.18613	0.001442	0.004377	0	0	0	2.232127	17.11289	3.014938
8756	SERTM2	3.726658	6.075044	1.906716	3.18613	0.001442	0.004377	0	0	0	2.232127	17.11289	3.014938
7927	PNLIPRP1	7.330123	6.064898	1.767694	3.430966	0.000601	0.002017	0	0.547412	0	15.62489	27.80844	0
6452	GRHL2	7.330753	6.064027	1.531527	3.959465	7.51E-05	0.00031	0	0	0.695731	24.5534	10.69555	8.039836
5078	RAB17	13.12655	6.038063	1.300195	4.643968	3.42E-06	1.79E-05	0	1.094824	0	31.24978	36.36488	10.04979
8788	AQP10	3.652178	6.032355	1.898464	3.177492	0.001486	0.004494	0	0	0	15.62489	4.278221	2.009959
9870	LOC11961	3.642698	6.029703	2.087569	2.888385	0.003872	0.010428	0	0	0	11.16063	10.69555	0
9871	CCDC185	3.642698	6.029703	2.087569	2.888385	0.003872	0.010428	0	0	0	11.16063	10.69555	0
9872	KRT34	3.642698	6.029703	2.087569	2.888385	0.003872	0.010428	0	0	0	11.16063	10.69555	0
8460	TACR3	3.61515	6.029678	1.846666	3.26517	0.001094	0.003438	0	0	0	13.39276	4.278221	4.019918
8658	LOC10324	3.636675	6.025661	1.874733	3.214144	0.001308	0.004017	0	0	0	13.39276	6.417332	2.009959
8659	OPRPN	3.636675	6.025661	1.874733	3.214144	0.001308	0.004017	0	0	0	13.39276	6.417332	2.009959
8240	SPATA21	3.562619	6.024933	1.806644	3.334876	0.000853	0.002753	0	0	0	8.928508	6.417332	6.029877
9933	CGAS	3.627195	6.024512	2.093097	2.878276	0.003999	0.010701	0	0	0	8.928508	12.83466	0
6436	KLRD1	6.968636	6.023344	1.518761	3.96596	7.31E-05	0.000302	0	0	0.695731	8.928508	17.11289	15.07469
6511	LOC10322	7.132251	6.020886	1.53033	3.93437	8.34E-05	0.000341	0	0	0.695731	20.08914	14.97377	7.034856
8249	SOX17	3.547117	6.020061	1.807573	3.330465	0.000867	0.002794	0	0	0	6.696381	8.556443	6.029877
1797	LOC11962	43.93032	6.017214	0.711175	8.460948	2.65E-17	3.92E-16	3.252518	0.547412	0	60.26743	91.98176	107.5328
10191	HPGDS	3.59619	6.015456	2.135218	2.817256	0.004844	0.012635	0	0	0	4.464254	17.11289	0
4913	SCARA5	12.87897	6.014946	1.269629	4.737561	2.16E-06	1.17E-05	0	1.094824	0	29.01765	32.08666	15.07469
6565	LOC10322	7.060135	6.013752	1.536187	3.914726	9.05E-05	0.000367	0.542086	0	0	13.39276	21.39111	7.034856
6592	LOC11962	7.033212	6.012497	1.540005	3.904206	9.45E-05	0.000381	0	0	0.695731	8.928508	23.53022	9.044815
8703	LOC10323	3.590167	6.010393	1.877626	3.20106	0.001369	0.004182	0	0	0	6.696381	12.83466	2.009959
8704	LOC10324	3.590167	6.010393	1.877626	3.20106	0.001369	0.004182	0	0	0	6.696381	12.83466	2.009959
8525	DDX53	3.553139	6.009674	1.850486	3.247619	0.001164	0.003629	0	0	0	4.464254	12.83466	4.019918
6853	KLHL40	7.076263	6.009363	1.583827	3.794204	0.000148	0.000575	0	0	0.695731	8.928508	27.80844	5.024897
8850	LOC11961	3.537637	6.006298	1.90261	3.156873	0.001595	0.00479	0	0	0	2.232127	14.97377	4.019918
9855	TDRD15	3.522134	6.005058	2.076615	2.891753	0.003831	0.010335	0	0	0	0	17.11289	4.019918
9136	DSG4	3.559162	6.004038	1.955195	3.070813	0.002135	0.006212	0	0	0	2.232127	17.11289	2.009959
7138	LOC11962	6.992302	5.982579	1.623976	3.683908	0.00023	0.000855	0	0	0.695731	17.85702	21.39111	2.009959
5275	CSMD1	13.37103	5.97751	1.318027	4.535196	5.76E-06	2.90E-05	0	0	1.391463	29.01765	42.78221	7.034856

6894	ACP3	6.939771	5.97537	1.581723	3.77776	0.000158	0.00061	0	0	0.695731	13.39276	23.53022	4.019918
6766	LOC119618	6.902743	5.973818	1.559503	3.830592	0.000128	0.000502	0	0	0.695731	11.16063	23.53022	6.029877
8533	LOC103214	3.358095	5.951596	1.833755	3.245579	0.001172	0.003652	0	0	0	6.696381	6.417332	7.034856
6613	TMEM275	6.717179	5.951337	1.526663	3.898265	9.69E-05	0.000389	0	0	0.695731	17.85702	10.69555	11.05477
8548	CFHR2	3.395123	5.949431	1.835691	3.240977	0.001191	0.003705	0	0	0	8.928508	6.417332	5.024897
9151	LOC119626	3.453676	5.947845	1.939654	3.066447	0.002166	0.006292	0	0	0	11.16063	8.556443	1.004979
9152	LOC119627	3.453676	5.947845	1.939654	3.066447	0.002166	0.006292	0	0	0	11.16063	8.556443	1.004979
9153	LOC103243	3.453676	5.947845	1.939654	3.066447	0.002166	0.006292	0	0	0	11.16063	8.556443	1.004979
8634	LOC103241	3.342592	5.9464	1.847283	3.218998	0.001286	0.003961	0	0	0	4.464254	8.556443	7.034856
6694	LOC119620	6.76023	5.943644	1.540326	3.858692	0.000114	0.000453	0	0	0.695731	17.85702	14.97377	7.034856
8715	CELA3B	3.416648	5.943239	1.858246	3.198306	0.001382	0.004216	0	0	0	8.928508	8.556443	3.014938
8717	SELE	3.416648	5.943239	1.858246	3.198306	0.001382	0.004216	0	0	0	8.928508	8.556443	3.014938
9173	APOD	3.438173	5.942415	1.940808	3.061825	0.0022	0.006374	0	0	0	8.928508	10.69555	1.004979
9174	IQCF6	3.438173	5.942415	1.940808	3.061825	0.0022	0.006374	0	0	0	8.928508	10.69555	1.004979
9175	LOC119620	3.438173	5.942415	1.940808	3.061825	0.0022	0.006374	0	0	0	8.928508	10.69555	1.004979
9176	KLRB1	3.438173	5.942415	1.940808	3.061825	0.0022	0.006374	0	0	0	8.928508	10.69555	1.004979
6834	SULT2A1	6.781755	5.941784	1.561897	3.804211	0.000142	0.000553	0	0	0.695731	17.85702	17.11289	5.024897
9259	TYRP1	3.422671	5.937645	1.952613	3.040871	0.002359	0.006771	0	0	0	6.696381	12.83466	1.004979
9260	LOC103221	3.422671	5.937645	1.952613	3.040871	0.002359	0.006771	0	0	0	6.696381	12.83466	1.004979
9261	PLA2G12B	3.422671	5.937645	1.952613	3.040871	0.002359	0.006771	0	0	0	6.696381	12.83466	1.004979
9262	LOC103235	3.422671	5.937645	1.952613	3.040871	0.002359	0.006771	0	0	0	6.696381	12.83466	1.004979
6881	LOC103244	6.75075	5.936971	1.568545	3.785018	0.000154	0.000594	0	0	0.695731	13.39276	21.39111	5.024897
9019	ADAMDEC1	3.348615	5.935199	1.908122	3.110492	0.001868	0.005506	0	0	0	2.232127	12.83466	5.024897
9983	CRH	3.311587	5.934099	2.070711	2.865731	0.00416	0.011079	0	0	0	0	12.83466	7.034856
4889	ADAM18	12.43928	5.912476	1.245191	4.748249	2.05E-06	1.12E-05	0	0	1.391463	22.32127	27.80844	23.11453
6733	LOC119625	6.503174	5.902808	1.535979	3.843026	0.000122	0.00048	0	0	0.695731	11.16063	17.11289	10.04979
6773	LOC103224	6.514595	5.901298	1.541991	3.827064	0.00013	0.000509	0.542086	0	0	13.39276	17.11289	8.039836
6767	BFSP2	6.540202	5.901233	1.541006	3.829467	0.000128	0.000505	0	0	0.695731	13.39276	17.11289	8.039836
7033	LOC103235	6.598756	5.899899	1.585102	3.722093	0.000198	0.000747	0	0	0.695731	15.62489	19.252	4.019918
6815	LOC103238	6.5247	5.898954	1.547856	3.811048	0.000138	0.00054	0	0	0.695731	11.16063	19.252	8.039836
7617	ISX	6.459494	5.891242	1.673342	3.520644	0.000431	0.001502	0	0.547412	0	2.232127	29.94755	6.029877
9587	LOC103231	3.29566	5.8862	1.985774	2.964184	0.003035	0.008416	0	0	0	15.62489	2.139111	2.009959
10549	LOC103221	3.301682	5.886158	2.152776	2.734218	0.006253	0.015757	0	0	0	13.39276	6.417332	0
10550	NTSR2	3.301682	5.886158	2.152776	2.734218	0.006253	0.015757	0	0	0	13.39276	6.417332	0
9277	PRSS48	3.258632	5.883403	1.9365	3.038163	0.00238	0.00682	0	0	0	13.39276	2.139111	4.019918
10511	SUN5	3.28618	5.879684	2.144181	2.742158	0.006104	0.015438	0	0	0	11.16063	8.556443	0
8949	LOC103219	3.169073	5.879326	1.877076	3.132173	0.001735	0.005155	0	0	0	6.696381	4.278221	8.039836
9295	LOC119625	3.280157	5.877705	1.937495	3.033662	0.002416	0.00691	0	0	0	13.39276	4.278221	2.009959
9296	LOC103248	3.280157	5.877705	1.937495	3.033662	0.002416	0.00691	0	0	0	13.39276	4.278221	2.009959
6950	SMOC2	6.376163	5.876979	1.565004	3.755248	0.000173	0.000662	0	0	0.695731	20.08914	6.417332	11.05477
3933	LOC119625	18.24643	5.872193	1.083856	5.417873	6.03E-08	4.08E-07	0	0.547412	1.391463	35.71403	57.75599	14.06971
8858	LRRTM3	3.190598	5.871995	1.861252	3.154864	0.001606	0.004819	0	0	0	6.696381	6.417332	6.029877
8859	LOC119622	3.190598	5.871995	1.861252	3.154864	0.001606	0.004819	0	0	0	6.696381	6.417332	6.029877
9159	LOC119624	3.264654	5.870746	1.916365	3.063479	0.002188	0.00635	0	0	0	11.16063	6.417332	2.009959
8925	LOC119620	3.227626	5.869896	1.870453	3.138222	0.0017	0.005063	0	0	0	8.928508	6.417332	4.019918
8959	LOC119622	3.175096	5.866629	1.874998	3.128872	0.001755	0.005207	0	0	0	4.464254	8.556443	6.029877
6911	MOG	6.403711	5.865219	1.554744	3.772466	0.000162	0.000622	0	0	0.695731	17.85702	12.83466	7.034856
9138	LOC103235	3.249152	5.86474	1.910899	3.0691	0.002147	0.006246	0	0	0	8.928508	8.556443	2.009959
9139	CLEC12A	3.249152	5.86474	1.910899	3.0691	0.002147	0.006246	0	0	0	8.928508	8.556443	2.009959
8944	LOC119627	3.212124	5.864437	1.871669	3.133266	0.001729	0.005138	0	0	0	6.696381	8.556443	4.019918
398	GPIHBP1	139.8037	5.863908	0.373137	15.71518	1.19E-55	7.95E-54	2.710432	5.474118	6.261582	272.3195	271.6671	280.3893
10733	ANKRD53	3.239671	5.863328	2.184359	2.684233	0.00727	0.018003	0	0	0	4.464254	14.97377	0
7819	FREM3	6.437282	5.862284	1.693785	3.461056	0.000538	0.001829	0	0	0.695731	13.39276	23.53022	1.004979
6867	LOC119618	6.335678	5.860922	1.546649	3.789433	0.000151	0.000584	0	0	0.695731	11.16063	17.11289	9.044815
9049	LOC119628	3.196621	5.859578	1.892243	3.09663	0.001957	0.00575	0	0	0	4.464254	10.69555	4.019918
9205	GPR20	3.233649	5.859536	1.918853	3.053666	0.002261	0.006526	0	0	0	6.696381	10.69555	2.009959
9207	LOC103225	3.233649	5.859536	1.918853	3.053666	0.002261	0.006526	0	0	0	6.696381	10.69555	2.009959
9208	LOC103230	3.233649	5.859536	1.918853	3.053666	0.002261	0.006526	0	0	0	6.696381	10.69555	2.009959
11044	IL26	3.224169	5.859365	2.238005	2.618119	0.008842	0.021283	0	0	0	2.232127	17.11289	0
9371	LOC103214	3.218146	5.855117	1.942616	3.014037	0.002578	0.007313	0	0	0	4.464254	12.83466	2.009959
9372	LOC103244	3.218146	5.855117	1.942616	3.014037	0.002578	0.007313	0	0	0	4.464254	12.83466	2.009959
172	SUCNR1	257.4266	5.84657	0.276496	21.14523	3.05E-99	4.72E-97	9.757555	8.211177	8.348776	499.9964	502.691	515.5545
5125	TMEM244	11.66664	5.832776	1.263517	4.616301	3.91E-06	2.03E-05	0	0.547412	0.695731	29.01765	25.66933	14.06971
5526	ABCB5	11.37496	5.829877	1.324664	4.401022	1.08E-05	5.18E-05	0	1.094824	0	35.71403	21.39111	10.04979
5222	SLAMF7	11.65716	5.827897	1.275407	4.569439	4.89E-06	2.49E-05	0	0.547412	0.695731	24.5534	32.08666	12.05975
3385	COL6A6	23.79716	5.827198	0.978339	5.956217	2.58E-09	2.03E-08	0	0.547412	2.087194	51.33892	72.72976	16.07967
7276	LOC119622	6.273243	5.824391	1.599837	3.640616	0.000272	0.000994	0	0	0.695731	20.08914	12.83466	4.019918
7049	LOC119623	6.094125	5.821559	1.568081	3.712537	0.000205	0.000774	0	0	0.695731	6.696381	17.11289	12.05975
7265	LOC119620	6.25774	5.821044	1.596183	3.646851	0.000265	0.000971	0	0	0.695731	17.85702	14.97377	4.019918
2503	LOC103225	29.20455	5.820255	0.816748	7.126134	1.03E-12	1.10E-11	0	0.547412	2.782925	62.49956	64.17332	45.22408
5407	CDH9	11.27592	5.820229	1.304917	4.460228	8.19E-06	4.03E-05	0	1.094824	0	24.5534	29.94755	12.05975
6999	LOC103224	6.168181	5.817861	1.55878	3.732317	0.00019	0.000721	0	0	0.695731	11.16063	17.11289	8.039836
7616	LOC119622	6.24826	5.81725	1.6522	3.520911	0.00043	0.001501	0	0	0.695731	13.39276	21.39111	2.009959
7431	SLC2A7	6.195729	5.811246	1.620252	3.58663	0.000335	0.001198	0	0	0.695731	8.928508	23.53022	4.019918

9939	FOXI2	3.11266	5.797316	2.015065	2.876987	0.004015	0.010738	0	0	0	13.39276	4.278221	1.004979
9940	LOC10322!	3.11266	5.797316	2.015065	2.876987	0.004015	0.010738	0	0	0	13.39276	4.278221	1.004979
5336	TAFA5	11.74389	5.796749	1.288666	4.498255	6.85E-06	3.41E-05	0	0	1.391463	33.4819	23.53022	12.05975
9435	LOC11961{	3.075632	5.792777	1.931237	2.999516	0.002704	0.007618	0	0	0	11.16063	4.278221	3.014938
9436	LOC10323:	3.075632	5.792777	1.931237	2.999516	0.002704	0.007618	0	0	0	11.16063	4.278221	3.014938
7493	AARD	6.115226	5.791584	1.625362	3.563258	0.000366	0.0013	0	0	0.695731	24.5534	6.417332	5.024897
9813	LOC10322:	3.097158	5.789913	1.994998	2.902215	0.003705	0.010038	0	0	0	11.16063	6.417332	1.004979
9191	LOC11961{	3.023102	5.787715	1.893441	3.056718	0.002238	0.006472	0	0	0	6.696381	6.417332	5.024897
9192	LOC10324:	3.023102	5.787715	1.893441	3.056718	0.002238	0.006472	0	0	0	6.696381	6.417332	5.024897
7562	LOC10323!	6.096529	5.786133	1.635698	3.537409	0.000404	0.001421	0	0.547412	0	22.32127	10.69555	3.014938
9329	FABP2	3.06013	5.786047	1.913507	3.023792	0.002496	0.007113	0	0	0	8.928508	6.417332	3.014938
9330	LOC10322(	3.06013	5.786047	1.913507	3.023792	0.002496	0.007113	0	0	0	8.928508	6.417332	3.014938
9789	MFSD6L	3.081655	5.783487	1.990141	2.906069	0.00366	0.00994	0	0	0	8.928508	8.556443	1.004979
9350	LOC10324:	3.044627	5.780226	1.914915	3.018529	0.00254	0.007221	0	0	0	6.696381	8.556443	3.014938
9351	IDO2	3.044627	5.780226	1.914915	3.018529	0.00254	0.007221	0	0	0	6.696381	8.556443	3.014938
9853	REG3G	3.066152	5.777848	1.998005	2.891809	0.00383	0.010334	0	0	0	6.696381	10.69555	1.004979
9854	KLHL6	3.066152	5.777848	1.998005	2.891809	0.00383	0.010334	0	0	0	6.696381	10.69555	1.004979
7292	LOC11961{	6.028495	5.776168	1.588136	3.637074	0.000276	0.001005	0	0.547412	0	15.62489	14.97377	5.024897
9500	FOXR1	3.029124	5.775185	1.935536	2.983765	0.002847	0.007967	0	0	0	4.464254	10.69555	3.014938
9501	PGLYRP2	3.029124	5.775185	1.935536	2.983765	0.002847	0.007967	0	0	0	4.464254	10.69555	3.014938
7313	LOC10322(	6.012105	5.773351	1.590445	3.630022	0.000283	0.00103	0.542086	0	0	13.39276	17.11289	5.024897
7536	IL10	6.059238	5.772854	1.627385	3.547318	0.000389	0.001373	0	0	0.695731	13.39276	19.252	3.014938
10702	ZNF804A	2.976594	5.771929	2.144523	2.691474	0.007114	0.017671	0	0	0	0	12.83466	5.024897
9793	PMP2	3.013622	5.771044	1.986288	2.905442	0.003667	0.009954	0	0	0	2.232127	12.83466	3.014938
9795	ERP27	3.013622	5.771044	1.986288	2.905442	0.003667	0.009954	0	0	0	2.232127	12.83466	3.014938
7352	CCKBR	6.02221	5.770793	1.595981	3.615829	0.000299	0.001083	0	0	0.695731	11.16063	19.252	5.024897
10316	LOC11962(	3.035147	5.769398	2.071401	2.785264	0.005348	0.013784	0	0	0	2.232127	14.97377	1.004979
5502	ADGB	11.03411	5.752749	1.304626	4.4095	1.04E-05	5.01E-05	0	0.547412	0.695731	13.39276	38.50399	13.06473
3378	CSMD3	22.90175	5.750838	0.964422	5.96299	2.48E-09	1.95E-08	0	0	2.782925	58.0353	53.47777	23.11453
3988	RD3	16.97901	5.746076	1.067542	5.382529	7.34E-08	4.90E-07	0	0	2.087194	40.17829	38.50399	21.10457
7506	LOC10324:	5.901222	5.734862	1.611626	3.558432	0.000373	0.001321	0	0	0.695731	17.85702	12.83466	4.019918
7330	ICAM4	5.79616	5.729533	1.581533	3.622773	0.000291	0.001057	0	0	0.695731	8.928508	17.11289	8.039836
1451	SULT1C2	54.8577	5.728027	0.611893	9.361162	7.89E-21	1.44E-19	0	1.094824	5.565851	100.4457	115.512	106.5278
5717	GLP2R	10.59991	5.727509	1.326833	4.316676	1.58E-05	7.37E-05	0	1.094824	0	26.78552	25.66933	10.04979
5876	CA8	10.57493	5.724815	1.354029	4.227984	2.36E-05	0.000107	0	1.094824	0	20.08914	34.22577	8.039836
450	LOC11962:	131.6532	5.724753	0.379313	15.09242	1.82E-51	1.07E-49	7.589209	2.737059	4.174388	276.7837	252.4151	246.22
7958	LOC10323:	5.749652	5.723149	1.671994	3.422949	0.000619	0.002069	0	0	0.695731	2.232127	23.53022	8.039836
5653	LOC10324:	11.2414	5.722947	1.316982	4.345501	1.39E-05	6.53E-05	0	0	1.391463	33.4819	23.53022	9.044815
5437	TACSTD2	11.13634	5.722769	1.287614	4.444475	8.81E-06	4.31E-05	0	0	1.391463	24.5534	27.80844	13.06473
11301	LOC10323!	2.945164	5.720283	2.235414	2.558936	0.010499	0.024699	0	0	0	13.39276	4.278221	0
5421	LTB	10.80161	5.717903	1.28465	4.450942	8.55E-06	4.19E-05	0	0.547412	0.695731	31.24978	19.252	13.06473
5898	CAMK4	11.15441	5.706361	1.353915	4.214711	2.50E-05	0.000113	0	0	1.391463	17.85702	40.6431	7.034856
9850	LOC10324:	2.892633	5.697721	1.969906	2.892382	0.003823	0.010319	0	0	0	8.928508	6.417332	2.009959
3946	LOC10322!	15.58914	5.694448	1.052447	5.410674	6.28E-08	4.23E-07	1.084173	0	0.695731	37.94616	25.66933	28.13943
9715	LOC11962!	2.840102	5.692436	1.946011	2.925181	0.003443	0.009419	0	0	0	4.464254	8.556443	4.019918
9716	LOC11962:	2.840102	5.692436	1.946011	2.925181	0.003443	0.009419	0	0	0	4.464254	8.556443	4.019918
9717	LOC10322:	2.840102	5.692436	1.946011	2.925181	0.003443	0.009419	0	0	0	4.464254	8.556443	4.019918
9881	LOC10322·	2.87713	5.691473	1.971542	2.886812	0.003892	0.010466	0	0	0	6.696381	8.556443	2.009959
9882	HTR2B	2.87713	5.691473	1.971542	2.886812	0.003892	0.010466	0	0	0	6.696381	8.556443	2.009959
9883	LOC10321!	2.87713	5.691473	1.971542	2.886812	0.003892	0.010466	0	0	0	6.696381	8.556443	2.009959
9884	FMO2	2.87713	5.691473	1.971542	2.886812	0.003892	0.010466	0	0	0	6.696381	8.556443	2.009959
9885	LOC10323!	2.87713	5.691473	1.971542	2.886812	0.003892	0.010466	0	0	0	6.696381	8.556443	2.009959
7380	SPEM2	5.597033	5.690004	1.577136	3.607809	0.000309	0.001112	0.542086	0	0	11.16063	12.83466	9.044815
10047	LOC10323!	2.8246	5.687191	1.994503	2.851433	0.004352	0.011515	0	0	0	2.232127	10.69555	4.019918
10048	LOC10323·	2.8246	5.687191	1.994503	2.851433	0.004352	0.011515	0	0	0	2.232127	10.69555	4.019918
8307	LOC10323(	5.724245	5.687038	1.717426	3.311374	0.000928	0.002971	0	0	0.695731	13.39276	19.252	1.004979
10030	ANHX	2.861628	5.686143	1.991814	2.854756	0.004307	0.011411	0	0	0	4.464254	10.69555	2.009959
10031	LOC11962·	2.861628	5.686143	1.991814	2.854756	0.004307	0.011411	0	0	0	4.464254	10.69555	2.009959
10032	LOC11962(	2.861628	5.686143	1.991814	2.854756	0.004307	0.011411	0	0	0	4.464254	10.69555	2.009959
10033	HMGCLL1	2.861628	5.686143	1.991814	2.854756	0.004307	0.011411	0	0	0	4.464254	10.69555	2.009959
10034	LOC11962:	2.861628	5.686143	1.991814	2.854756	0.004307	0.011411	0	0	0	4.464254	10.69555	2.009959
7452	LOC10323:	5.619446	5.685029	1.587659	3.580762	0.000343	0.001222	0	0.547412	0	11.16063	14.97377	7.034856
8367	LOC10321!	5.684022	5.684327	1.725935	3.293477	0.00099	0.003144	0	0.547412	0	11.16063	21.39111	1.004979
5523	SLC9C2	10.5566	5.683998	1.290814	4.403423	1.07E-05	5.13E-05	0	0.547412	0.695731	20.08914	29.94755	12.05975
10326	MCHR2	2.846125	5.681945	2.042148	2.782338	0.005397	0.013893	0	0	0	2.232127	12.83466	2.009959
10327	LOC10323:	2.846125	5.681945	2.042148	2.782338	0.005397	0.013893	0	0	0	2.232127	12.83466	2.009959
11400	LOC10321·	2.830622	5.681724	2.239811	2.536697	0.01119	0.026096	0	0	0	0	14.97377	2.009959
7587	APOA2	5.640972	5.681477	1.609251	3.53051	0.000415	0.001453	0	0.547412	0	11.16063	17.11289	5.024897
6353	ADCY8	10.26146	5.681448	1.418491	4.005276	6.19E-05	0.000259	0	1.094824	0	20.08914	36.36488	4.019918
7867	LOC11962(	5.687217	5.680354	1.646338	3.450297	0.00056	0.001892	0	0	0.695731	11.16063	19.252	3.014938
7620	CPB1	5.588441	5.68007	1.61389	3.51949	0.000432	0.001508	0	0.547412	0	6.696381	19.252	7.034856
7621	CASP14	5.588441	5.68007	1.61389	3.51949	0.000432	0.001508	0	0.547412	0	6.696381	19.252	7.034856
170	LIPH	288.0051	5.679678	0.267332	21.24581	3.60E-100	5.64E-98	5.96295	14.23271	13.2189	575.8888	569.0034	549.7238
7946	KRT26	5.671714	5.678058	1.657848	3.424957	0.000615	0.002057	0	0	0.695731	8.928508	21.39111	3.014938

8540	UPK1A	5.599598	5.674261	1.749364	3.243613	0.00118	0.003674	0.542086	0	0	2.232127	27.80844	3.014938
4025	LOC11962(	15.40445	5.666977	1.058388	5.354345	8.59E-08	5.67E-07	0	1.094824	0.695731	29.01765	38.50399	23.11453
4998	NRXN3	16.45367	5.662214	1.207529	4.689093	2.74E-06	1.46E-05	0	0	2.087194	42.41041	49.19955	5.024897
4364	RGS6	16.06401	5.652994	1.107434	5.104588	3.32E-07	2.02E-06	0	0	2.087194	29.01765	49.19955	16.07967
5602	LOC11961‹	10.5753	5.649805	1.291846	4.373437	1.22E-05	5.80E-05	0	0	1.391463	22.32127	25.66933	14.06971
7896	LOC11962‹	5.560206	5.647737	1.640936	3.441777	0.000578	0.001946	0	0	0.695731	20.08914	8.556443	4.019918
7518	BHMT2	5.470647	5.644358	1.588557	3.553134	0.000381	0.001346	0	0	0.695731	13.39276	10.69555	8.039836
8138	LOC10324(	5.550726	5.640154	1.674714	3.367831	0.000758	0.002475	0	0	0.695731	15.62489	14.97377	2.009959
8167	LOC11962(	5.510503	5.637041	1.678205	3.358971	0.000782	0.002547	0	0.547412	0	13.39276	17.11289	2.009959
7794	RFTN2	5.513698	5.636358	1.626008	3.466378	0.000528	0.001799	0	0	0.695731	13.39276	14.97377	4.019918
8276	LOC10324‹	5.479498	5.631685	1.69581	3.320942	0.000897	0.002882	0	0.547412	0	8.928508	21.39111	2.009959
8268	LOC11961‹	5.504217	5.631571	1.694482	3.323477	0.000889	0.002859	0	0	0.695731	8.928508	21.39111	2.009959
7933	LOC10322(	5.482692	5.63099	1.641655	3.430069	0.000603	0.002022	0	0	0.695731	8.928508	19.252	4.019918
8070	LOC10324‹	5.467189	5.62894	1.659156	3.392653	0.000692	0.00228	0	0	0.695731	6.696381	21.39111	4.019918
11310	DLK1	2.660561	5.623752	2.200274	2.555932	0.01059	0.024893	0	0	0	8.928508	0	7.034856
10860	LOC10322‹	2.756142	5.622739	2.114965	2.65855	0.007848	0.019211	0	0	0	13.39276	2.139111	1.004979
6172	ACSM1	9.830886	5.62069	1.374531	4.08917	4.33E-05	0.000186	0	1.094824	0	15.62489	34.22577	8.039836
10416	LOC11962(	2.719114	5.618473	2.034111	2.762127	0.005743	0.014657	0	0	0	11.16063	2.139111	3.014938
10629	LOC10323(	2.740639	5.6132	2.070047	2.71163	0.006695	0.016746	0	0	0	11.16063	4.278221	1.004979
10182	KLF18	2.703611	5.61012	1.989934	2.819249	0.004814	0.012568	0	0	0	8.928508	4.278221	3.014938
10093	LOC10323‹	2.651081	5.605731	1.973774	2.840108	0.00451	0.011879	0	0	0	4.464254	6.417332	5.024897
10569	LOC11962‹	2.725137	5.605353	2.054224	2.728696	0.006359	0.015992	0	0	0	8.928508	6.417332	1.004979
10571	ANKRD40C	2.725137	5.605353	2.054224	2.728696	0.006359	0.015992	0	0	0	8.928508	6.417332	1.004979
6177	ACSBG2	10.08077	5.603706	1.370774	4.087988	4.35E-05	0.000187	0.542086	0	0.695731	17.85702	36.36488	5.024897
4003	BHLHE22	21.02829	5.603486	1.04298	5.372571	7.76E-08	5.16E-07	0	0	2.782925	53.57105	57.75599	12.05975
10134	LOC10322‹	2.688109	5.603086	1.979366	2.830748	0.004644	0.01218	0	0	0	6.696381	6.417332	3.014938
10135	LOC11961‹	2.688109	5.603086	1.979366	2.830748	0.004644	0.01218	0	0	0	6.696381	6.417332	3.014938
10594	LOC10324‹	2.709634	5.598579	2.056182	2.722803	0.006473	0.01624	0	0	0	6.696381	8.556443	1.004979
10596	LOC11962‹	2.709634	5.598579	2.056182	2.722803	0.006473	0.01624	0	0	0	6.696381	8.556443	1.004979
10597	LOC10324‹	2.709634	5.598579	2.056182	2.722803	0.006473	0.01624	0	0	0	6.696381	8.556443	1.004979
10232	LOC11962‹	2.672606	5.596954	1.993588	2.807478	0.004993	0.012972	0	0	0	4.464254	8.556443	3.014938
10233	LOC11961‹	2.672606	5.596954	1.993588	2.807478	0.004993	0.012972	0	0	0	4.464254	8.556443	3.014938
10529	LOC11962‹	2.657103	5.591728	2.042122	2.738195	0.006178	0.015591	0	0	0	2.232127	10.69555	3.014938
10531	LOC10322‹	2.657103	5.591728	2.042122	2.738195	0.006178	0.015591	0	0	0	2.232127	10.69555	3.014938
10532	LOC10323‹	2.657103	5.591728	2.042122	2.738195	0.006178	0.015591	0	0	0	2.232127	10.69555	3.014938
10533	CHRNB3	2.657103	5.591728	2.042122	2.738195	0.006178	0.015591	0	0	0	2.232127	10.69555	3.014938
10534	LOC10321‹	2.657103	5.591728	2.042122	2.738195	0.006178	0.015591	0	0	0	2.232127	10.69555	3.014938
7862	AMER3	5.324676	5.589453	1.619649	3.451028	0.000558	0.001888	0	0	0.695731	13.39276	12.83466	5.024897
7863	SOX14	5.324676	5.589453	1.619649	3.451028	0.000558	0.001888	0	0	0.695731	13.39276	12.83466	5.024897
11621	LOC11961‹	2.6416	5.589165	2.2456	2.48894	0.012812	0.029311	0	0	0	0	12.83466	3.014938
8109	PABPC1L2I	5.346201	5.587476	1.654719	3.376693	0.000734	0.002405	0	0	0.695731	13.39276	14.97377	3.014938
8151	GCSAML	5.330698	5.584445	1.659791	3.364547	0.000767	0.002501	0	0	0.695731	11.16063	17.11289	3.014938
8845	LOC10321‹	5.296498	5.582801	1.767805	3.15804	0.001588	0.004774	0	0.547412	0	6.696381	23.53022	1.004979
970	CPAMD8	67.47464	5.582098	0.501236	11.13667	8.32E-29	2.28E-27	1.084173	3.831883	3.478657	133.9276	136.9031	125.6224
8331	LOC10324‹	5.274973	5.579804	1.688829	3.303948	0.000953	0.003042	0	0.547412	0	6.696381	21.39111	3.014938
8325	LOC10324‹	5.299693	5.579696	1.687431	3.306622	0.000944	0.003015	0	0	0.695731	6.696381	21.39111	3.014938
5978	LOC11961‹	10.15634	5.571141	1.335174	4.172595	3.01E-05	0.000134	0	0	1.391463	31.24978	19.252	9.044815
5963	LOC11962‹	10.09433	5.564133	1.330801	4.18104	2.90E-05	0.000129	0	0	1.391463	22.32127	27.80844	9.044815
5909	COL3A1	9.759601	5.559211	1.321393	4.207085	2.59E-05	0.000116	0	0.547412	0.695731	29.01765	19.252	9.044815
6290	ARSF	10.15288	5.558794	1.378058	4.033788	5.49E-05	0.000232	0	0	1.391463	24.5534	29.94755	5.024897
3416	RALYL	25.36779	5.55765	0.9371	5.930693	3.02E-09	2.35E-08	0	0	3.478657	40.17829	83.42532	25.12449
5894	LOC10324‹	9.727708	5.55698	1.317599	4.217503	2.47E-05	0.000111	0.542086	0	0.695731	24.5534	23.53022	9.044815
5860	LOC11961‹	9.660562	5.552221	1.310414	4.236996	2.27E-05	0.000103	0	0.547412	0.695731	17.85702	27.80844	11.05477
6161	SLC16A11	9.955275	5.550633	1.356121	4.093023	4.26E-05	0.000184	0	0	1.391463	35.71403	8.556443	14.06971
8103	LOC11961‹	5.166659	5.550407	1.642237	3.379785	0.000725	0.00238	0	0	0.695731	17.85702	6.417332	6.029877
2530	LOC10324‹	34.03594	5.548566	0.783398	7.082688	1.41E-12	1.49E-11	0	1.642235	2.782925	66.96381	102.6773	30.14938
4366	KCNH5	14.61322	5.548473	1.087132	5.10377	3.33E-07	2.03E-06	0	0.547412	1.391463	29.01765	40.6431	16.07967
7887	APELA	5.098626	5.544269	1.609258	3.445233	0.000571	0.001923	0	0	0.695731	11.16063	10.69555	8.039836
7888	INMT	5.098626	5.544269	1.609258	3.445233	0.000571	0.001923	0	0	0.695731	11.16063	10.69555	8.039836
7979	GABRR2	5.110934	5.542162	1.621773	3.417347	0.000632	0.002106	0	0.547412	0	13.39276	10.69555	6.029877
7980	LOC10322‹	5.110934	5.542162	1.621773	3.417347	0.000632	0.002106	0	0.547412	0	13.39276	10.69555	6.029877
8154	PRDM13	5.147962	5.541997	1.647481	3.363921	0.000768	0.002505	0	0.547412	0	15.62489	10.69555	4.019918
7917	LOC10321‹	5.083123	5.540991	1.613619	3.433891	0.000595	0.001998	0	0	0.695731	8.928508	12.83466	8.039836
7977	LOC11961‹	5.120151	5.538619	1.620499	3.417847	0.000631	0.002104	0	0	0.695731	11.16063	12.83466	6.029877
5999	LOC10323‹	9.76322	5.535951	1.329286	4.164605	3.12E-05	0.000138	0	0	1.391463	11.16063	29.94755	16.07967
8497	LOC11962(	5.138482	5.534729	1.699287	3.257089	0.001126	0.003522	0	0.547412	0	11.16063	17.11289	2.009959
11983	LOC11962(	2.56712	5.533955	2.297034	2.409174	0.015989	0.035471	0	0	0	13.39276	0	2.009959
11984	KIF2B	2.56712	5.533955	2.297034	2.409174	0.015989	0.035471	0	0	0	13.39276	0	2.009959
8218	LOC10324‹	5.126174	5.531964	1.655569	3.341429	0.000833	0.002696	0	0	0.695731	8.928508	17.11289	4.019918
6011	CUNH10or	9.576602	5.52905	1.329421	4.158992	3.20E-05	0.000141	0	0.547412	0.695731	26.78552	21.39111	8.039836
8629	MLN	5.05814	5.528807	1.716807	3.2204	0.00128	0.003944	0	0	0.695731	2.232127	21.39111	6.029877
6221	GPR45	9.880326	5.528316	1.359642	4.066009	4.78E-05	0.000204	0	0	1.391463	15.62489	34.22577	8.039836
8512	PRG3	5.095168	5.527753	1.699148	3.25325	0.001141	0.003563	0	0	0.695731	4.464254	21.39111	4.019918
8823	LOC11961‹	5.079666	5.526663	1.745614	3.166028	0.001545	0.004656	0	0	0.695731	2.232127	23.53022	4.019918

12087	IRGM	2.573143	5.524021	2.311572	2.389725	0.016861	0.037082	0	0	0	11.16063	4.278221	0
6374	LOC11962	9.849321	5.522028	1.382878	3.993141	6.52E-05	0.000272	0	0	1.391463	11.16063	38.50399	8.039836
10974	LOC11962	2.551617	5.518736	2.094391	2.635007	0.008414	0.020379	0	0	0	11.16063	2.139111	2.009959
10976	UMOD	2.551617	5.518736	2.094391	2.635007	0.008414	0.020379	0	0	0	11.16063	2.139111	2.009959
10719	LOC11962	2.514589	5.518428	2.05381	2.686923	0.007211	0.017885	0	0	0	8.928508	2.139111	4.019918
12026	CLEC12B	2.55764	5.515603	2.296598	2.401641	0.016322	0.036081	0	0	0	8.928508	6.417332	0
6089	SFTPC	9.768818	5.514232	1.336169	4.126897	3.68E-05	0.000161	0	0	1.391463	26.78552	21.39111	9.044815
10587	LOC11962	2.462059	5.513605	2.022804	2.725723	0.006416	0.016111	0	0	0	4.464254	4.278221	6.029877
10544	LOC10322	2.499087	5.510725	2.015278	2.734474	0.006248	0.015749	0	0	0	6.696381	4.278221	4.019918
10545	LOC11962	2.499087	5.510725	2.015278	2.734474	0.006248	0.015749	0	0	0	6.696381	4.278221	4.019918
10546	LOC11962	2.499087	5.510725	2.015278	2.734474	0.006248	0.015749	0	0	0	6.696381	4.278221	4.019918
10547	SEBOX	2.499087	5.510725	2.015278	2.734474	0.006248	0.015749	0	0	0	6.696381	4.278221	4.019918
10722	GCM2	2.536115	5.509443	2.051027	2.686188	0.007227	0.017916	0	0	0	8.928508	4.278221	2.009959
10723	LOC10324	2.536115	5.509443	2.051027	2.686188	0.007227	0.017916	0	0	0	8.928508	4.278221	2.009959
10724	SLC10A1	2.536115	5.509443	2.051027	2.686188	0.007227	0.017916	0	0	0	8.928508	4.278221	2.009959
12057	ANKRD66	2.542137	5.508023	2.299591	2.395219	0.01661	0.036622	0	0	0	6.696381	8.556443	0
12058	BECN2	2.542137	5.508023	2.299591	2.395219	0.01661	0.036622	0	0	0	6.696381	8.556443	0
10746	LOC11962	2.446556	5.50611	2.053988	2.680693	0.007347	0.018176	0	0	0	2.232127	6.417332	6.029877
2863	LOC10321	23.18401	5.504422	0.835022	6.591951	4.34E-11	4.03E-10	0	1.094824	2.087194	44.64254	47.06043	44.2191
10575	CMA1	2.483584	5.503769	2.017538	2.727963	0.006373	0.016019	0	0	0	4.464254	6.417332	4.019918
10576	LOC10322	2.483584	5.503769	2.017538	2.727963	0.006373	0.016019	0	0	0	4.464254	6.417332	4.019918
10684	LOC10324	2.520612	5.501768	2.04105	2.695558	0.007027	0.017484	0	0	0	6.696381	6.417332	2.009959
10685	LOC11962	2.520612	5.501768	2.04105	2.695558	0.007027	0.017484	0	0	0	6.696381	6.417332	2.009959
12176	LOC11962	2.526635	5.501202	2.320478	2.370719	0.017754	0.038753	0	0	0	4.464254	10.69555	0
12177	MTNR1B	2.526635	5.501202	2.320478	2.370719	0.017754	0.038753	0	0	0	4.464254	10.69555	0
12178	LOC11961	2.526635	5.501202	2.320478	2.370719	0.017754	0.038753	0	0	0	4.464254	10.69555	0
12179	LOC11961	2.526635	5.501202	2.320478	2.370719	0.017754	0.038753	0	0	0	4.464254	10.69555	0
13627	LOC11962	2.495629	5.495562	2.637762	2.083419	0.037213	0.072599	0	0	0	0	14.97377	0
12423	LOC11962	2.511132	5.495502	2.37252	2.316315	0.020541	0.043954	0	0	0	2.232127	12.83466	0
10776	KNG1	2.505109	5.495223	2.055481	2.673449	0.007508	0.018511	0	0	0	4.464254	8.556443	2.009959
10777	CCR9	2.505109	5.495223	2.055481	2.673449	0.007508	0.018511	0	0	0	4.464254	8.556443	2.009959
10781	PYCARD	2.505109	5.495223	2.055481	2.673449	0.007508	0.018511	0	0	0	4.464254	8.556443	2.009959
10782	LOC10323	2.505109	5.495223	2.055481	2.673449	0.007508	0.018511	0	0	0	4.464254	8.556443	2.009959
10783	LOC11962	2.505109	5.495223	2.055481	2.673449	0.007508	0.018511	0	0	0	4.464254	8.556443	2.009959
8131	LOC10321	4.921025	5.493942	1.630034	3.370446	0.00075	0.002454	0.542086	0	0	13.39276	8.556443	7.034856
8114	LOC11962	4.946632	5.493873	1.628452	3.373679	0.000742	0.00243	0	0	0.695731	13.39276	8.556443	7.034856
11079	LOC11962	2.489607	5.489887	2.103643	2.609704	0.009062	0.021741	0	0	0	2.232127	10.69555	2.009959
11080	LOC11962	2.489607	5.489887	2.103643	2.609704	0.009062	0.021741	0	0	0	2.232127	10.69555	2.009959
11081	DNAJB8	2.489607	5.489887	2.103643	2.609704	0.009062	0.021741	0	0	0	2.232127	10.69555	2.009959
8419	LOC11962	5.005185	5.489357	1.675435	3.276378	0.001051	0.00332	0	0	0.695731	15.62489	10.69555	3.014938
12181	LOC11961	2.474104	5.488565	2.315555	2.370303	0.017774	0.038791	0	0	0	0	12.83466	2.009959
8211	FOXI3	4.952655	5.484763	1.640693	3.342954	0.000829	0.002684	0	0	0.695731	11.16063	12.83466	5.024897
8238	TSPYL6	4.874516	5.483765	1.644101	3.335419	0.000852	0.002749	0.542086	0	0	6.696381	14.97377	7.034856
8399	CD1B	4.859901	5.480938	1.669675	3.282638	0.001028	0.003254	0	0.547412	0	4.464254	17.11289	7.034856
8365	LOC10323	4.921649	5.478937	1.66343	3.293759	0.000989	0.003141	0	0	0.695731	6.696381	17.11289	5.024897
3742	OC90	18.09638	5.478175	0.978063	5.601047	2.13E-08	1.51E-07	0	1.642235	0.695731	37.94616	49.19955	19.09461
8551	LOC11962	4.906146	5.476713	1.690061	3.240541	0.001193	0.003709	0	0	0.695731	4.464254	19.252	5.024897
3080	LOC10321	27.98853	5.469197	0.86769	6.303171	2.92E-10	2.52E-09	0	1.094824	2.782925	93.74933	49.19955	21.10457
6450	MARCO	9.148592	5.460374	1.378736	3.96042	7.48E-05	0.000308	0	0.547412	0.695731	13.39276	34.22577	6.029877
6663	LOC11962	8.818997	5.455089	1.408645	3.872578	0.000108	0.00043	0.542086	0.547412	0	17.85702	29.94755	4.019918
6581	SGCZ	8.713935	5.448533	1.393976	3.908628	9.28E-05	0.000375	0.542086	0.547412	0	8.928508	34.22577	8.039836
9863	LOC11962	4.859214	5.446441	1.884289	2.89045	0.003847	0.010369	0	0	0.695731	15.62489	12.83466	0
6094	LOC11962	8.994033	5.446128	1.320198	4.125236	3.70E-05	0.000162	0	0.547412	0.695731	24.5534	17.11289	11.05477
8339	LOC10323	4.754416	5.438044	1.647781	3.300222	0.000966	0.00308	0	0.547412	0	13.39276	8.556443	6.029877
8340	LOC11962	4.754416	5.438044	1.647781	3.300222	0.000966	0.00308	0	0.547412	0	13.39276	8.556443	6.029877
169	DNAJC12	284.5749	5.437884	0.255451	21.28738	1.49E-100	2.34E-98	16.80468	10.40082	11.1317	531.2462	519.8039	618.0624
12	CFH	3958.017	5.436907	0.103474	52.54385	0	0	164.7943	186.6674	185.0645	7723.159	7568.174	7920.243
8316	LOC11962	4.763633	5.434033	1.642146	3.309105	0.000936	0.002992	0	0	0.695731	11.16063	10.69555	6.029877
1095	LOC10324	60.27703	5.430855	0.5155	10.53513	5.95E-26	1.44E-24	3.794605	2.189647	2.087194	140.624	98.39909	114.5677
5181	HTR4	14.0265	5.430751	1.183498	4.588728	4.46E-06	2.29E-05	0	0	2.087194	31.24978	42.78221	8.039836
8359	KLHL1	4.72341	5.430637	1.648286	3.294717	0.000985	0.003133	0	0.547412	0	8.928508	12.83466	6.029877
8527	NKG7	4.670879	5.430048	1.672208	3.247233	0.001165	0.003633	0	0.547412	0	4.464254	14.97377	8.039836
6555	XIRP1	9.297334	5.427508	1.385727	3.916721	8.98E-05	0.000364	0	0	1.391463	31.24978	17.11289	6.029877
6590	LAIR1	8.640343	5.427191	1.389818	3.904964	9.42E-05	0.00038	0	1.094824	0	22.32127	21.39111	7.034856
8661	TACR1	4.754153	5.42315	1.687608	3.213513	0.001311	0.004025	0	0	0.695731	6.696381	17.11289	4.019918
9120	LOC10324	4.723147	5.419528	1.7609	3.077704	0.002086	0.006081	0	0	0.695731	2.232127	21.39111	4.019918
9487	GATA4	4.719953	5.419135	1.813774	2.987767	0.00281	0.007875	0	0.547412	0	2.232127	23.53022	2.009959
2671	LOC10323	26.25398	5.41332	0.787507	6.873992	6.24E-12	6.21E-11	0	1.642235	2.087194	53.57105	62.03421	38.18922
11659	EVI2B	2.384121	5.412999	2.18345	2.479104	0.013171	0.030033	0	0	0	11.16063	2.139111	1.004979
11170	LOC10323	2.310065	5.412152	2.09224	2.586774	0.009688	0.023058	0	0	0	6.696381	2.139111	5.024897
11047	ODF4	2.294562	5.404842	2.065919	2.616192	0.008892	0.021398	0	0	0	4.464254	4.278221	5.024897
11468	FGA	2.368618	5.402795	2.141774	2.52258	0.01165	0.026988	0	0	0	8.928508	4.278221	1.004979
11469	CTXND2	2.368618	5.402795	2.141774	2.52258	0.01165	0.026988	0	0	0	8.928508	4.278221	1.004979
11470	LOC11962	2.368618	5.402795	2.141774	2.52258	0.01165	0.026988	0	0	0	8.928508	4.278221	1.004979

11472	LOC10322(	2.368618	5.402795	2.141774	2.52258	0.01165	0.026988	0	0	0	8.928508	4.278221	1.004979
11473	LOC10324;	2.368618	5.402795	2.141774	2.52258	0.01165	0.026988	0	0	0	8.928508	4.278221	1.004979
11474	LOC11962(	2.368618	5.402795	2.141774	2.52258	0.01165	0.026988	0	0	0	8.928508	4.278221	1.004979
11476	LOC11962:	2.368618	5.402795	2.141774	2.52258	0.01165	0.026988	0	0	0	8.928508	4.278221	1.004979
11072	GPR182	2.33159	5.401628	2.068741	2.61107	0.009026	0.021668	0	0	0	6.696381	4.278221	3.014938
11073	MYL1	2.33159	5.401628	2.068741	2.61107	0.009026	0.021668	0	0	0	6.696381	4.278221	3.014938
11075	UCMA	2.33159	5.401628	2.068741	2.61107	0.009026	0.021668	0	0	0	6.696381	4.278221	3.014938
6582	FAM71F2	8.792074	5.401196	1.381907	3.908509	9.29E-05	0.000375	0	0.547412	0.695731	13.39276	32.08666	6.029877
11434	TRIM42	2.353115	5.394405	2.132804	2.529255	0.011431	0.026572	0	0	0	6.696381	6.417332	1.004979
11435	MASP2	2.353115	5.394405	2.132804	2.529255	0.011431	0.026572	0	0	0	6.696381	6.417332	1.004979
11436	LOC10322:	2.353115	5.394405	2.132804	2.529255	0.011431	0.026572	0	0	0	6.696381	6.417332	1.004979
11102	LOC11962‹	2.316087	5.394106	2.071369	2.604127	0.009211	0.022052	0	0	0	4.464254	6.417332	3.014938
11104	LOC11962(	2.316087	5.394106	2.071369	2.604127	0.009211	0.022052	0	0	0	4.464254	6.417332	3.014938
11346	HJV	2.300585	5.387471	2.114987	2.547283	0.010857	0.025427	0	0	0	2.232127	8.556443	3.014938
11347	LOC10323‹	2.300585	5.387471	2.114987	2.547283	0.010857	0.025427	0	0	0	2.232127	8.556443	3.014938
11348	LOC11961ł	2.300585	5.387471	2.114987	2.547283	0.010857	0.025427	0	0	0	2.232127	8.556443	3.014938
11349	WFDC13	2.300585	5.387471	2.114987	2.547283	0.010857	0.025427	0	0	0	2.232127	8.556443	3.014938
11350	LOC10323;	2.300585	5.387471	2.114987	2.547283	0.010857	0.025427	0	0	0	2.232127	8.556443	3.014938
11351	LOC10322;	2.300585	5.387471	2.114987	2.547283	0.010857	0.025427	0	0	0	2.232127	8.556443	3.014938
12359	LOC10322!	2.263557	5.387429	2.311861	2.330343	0.019788	0.042565	0	0	0	0	8.556443	5.024897
11520	LOC10324!	2.337613	5.387254	2.147074	2.509114	0.012103	0.027924	0	0	0	4.464254	8.556443	1.004979
11521	SPATA48	2.337613	5.387254	2.147074	2.509114	0.012103	0.027924	0	0	0	4.464254	8.556443	1.004979
11522	GDF3	2.337613	5.387254	2.147074	2.509114	0.012103	0.027924	0	0	0	4.464254	8.556443	1.004979
11523	LOC11961ł	2.337613	5.387254	2.147074	2.509114	0.012103	0.027924	0	0	0	4.464254	8.556443	1.004979
8565	LOC10322:	4.565394	5.386455	1.663414	3.238193	0.001203	0.003734	0	0.547412	0	13.39276	6.417332	7.034856
8546	DGAT2L6	4.590114	5.386397	1.661674	3.241548	0.001189	0.003698	0	0	0.695731	13.39276	6.417332	7.034856
12467	SLC27A6	2.285082	5.38304	2.333818	2.306538	0.021081	0.044946	0	0	0	0	10.69555	3.014938
12468	LOC11962(	2.285082	5.38304	2.333818	2.306538	0.021081	0.044946	0	0	0	0	10.69555	3.014938
12469	LOC10322:	2.285082	5.38304	2.333818	2.306538	0.021081	0.044946	0	0	0	0	10.69555	3.014938
11782	LOC11962(	2.32211	5.38154	2.194344	2.45246	0.014188	0.031996	0	0	0	2.232127	10.69555	1.004979
11784	FAM170B	2.32211	5.38154	2.194344	2.45246	0.014188	0.031996	0	0	0	2.232127	10.69555	1.004979
11785	LOC11962ł	2.32211	5.38154	2.194344	2.45246	0.014188	0.031996	0	0	0	2.232127	10.69555	1.004979
11786	LOC11961ł	2.32211	5.38154	2.194344	2.45246	0.014188	0.031996	0	0	0	2.232127	10.69555	1.004979
11787	ZP4	2.32211	5.38154	2.194344	2.45246	0.014188	0.031996	0	0	0	2.232127	10.69555	1.004979
11788	LOC11962(	2.32211	5.38154	2.194344	2.45246	0.014188	0.031996	0	0	0	2.232127	10.69555	1.004979
11789	LOC11962:	2.32211	5.38154	2.194344	2.45246	0.014188	0.031996	0	0	0	2.232127	10.69555	1.004979
12859	LOC11962!	2.306607	5.381222	2.416624	2.226752	0.025964	0.053674	0	0	0	0	12.83466	1.004979
12860	KRT31	2.306607	5.381222	2.416624	2.226752	0.025964	0.053674	0	0	0	0	12.83466	1.004979
8835	TMEM235	4.648667	5.380396	1.701752	3.16168	0.001569	0.00472	0	0	0.695731	15.62489	8.556443	3.014938
8586	OVCH2	4.543605	5.374925	1.662518	3.233002	0.001225	0.003792	0	0	0.695731	6.696381	12.83466	7.034856
8587	GPAT2	4.543605	5.374925	1.662518	3.233002	0.001225	0.003792	0	0	0.695731	6.696381	12.83466	7.034856
8763	LOC10322;	4.528103	5.371625	1.686618	3.184849	0.001448	0.004394	0	0	0.695731	4.464254	14.97377	7.034856
8728	LOC11961ł	4.565131	5.36908	1.681125	3.193742	0.001404	0.004278	0	0	0.695731	6.696381	14.97377	5.024897
8873	LOC11962(	4.577439	5.368586	1.70448	3.149692	0.001634	0.004897	0	0.547412	0	8.928508	14.97377	3.014938
8977	SNX20	4.586656	5.365586	1.717904	3.123332	0.001788	0.005296	0	0	0.695731	6.696381	17.11289	3.014938
8978	EDDM3A	4.586656	5.365586	1.717904	3.123332	0.001788	0.005296	0	0	0.695731	6.696381	17.11289	3.014938
2784	MS4A8	24.39368	5.359251	0.797014	6.724161	1.77E-11	1.69E-10	2.168345	1.094824	0	53.57105	51.33866	38.18922
7	PLA1A	1629.574	5.354161	0.13208	40.5374	0	0	82.39713	73.90059	77.22618	3011.139	3069.624	3463.159
6871	LOC11962!	8.471696	5.342722	1.410365	3.788185	0.000152	0.000587	0.542086	0	0.695731	15.62489	29.94755	4.019918
5185	GALNT14	13.16493	5.34199	1.164432	4.587635	4.48E-06	2.30E-05	0	0	2.087194	40.17829	25.66933	11.05477
10362	LOC10322‹	4.493478	5.337413	1.924182	2.773861	0.00554	0.014212	0	0.547412	0	17.85702	8.556443	0
4672	CSF2	12.08382	5.337368	1.088551	4.903186	9.43E-07	5.37E-06	0.542086	0.547412	0.695731	22.32127	19.252	29.1444
4852	LOC10324:	12.70016	5.334563	1.116047	4.779871	1.75E-06	9.61E-06	0	0.547412	1.391463	31.24978	29.94755	13.06473
4308	KCNJ2	17.43252	5.332478	1.035253	5.150892	2.59E-07	1.60E-06	0	0	2.782925	31.24978	53.47777	17.08465
9213	PCDH20	4.45645	5.322273	1.743754	3.052192	0.002272	0.006555	0	0.547412	0	15.62489	8.556443	2.009959
9182	CLNK	4.440948	5.317474	1.737472	3.060466	0.00221	0.006398	0	0.547412	0	13.39276	10.69555	2.009959
6536	NCR3	8.41466	5.3158	1.354313	3.92509	8.67E-05	0.000353	0	0	1.391463	22.32127	10.69555	16.07967
8883	LOC11962:	4.42864	5.315474	1.688374	3.148281	0.001642	0.004915	0	0	0.695731	11.16063	10.69555	4.019918
8846	GIPC2	4.351389	5.31407	1.683032	3.157439	0.001592	0.004783	0	0.547412	0	6.696381	12.83466	6.029877
6504	APOBEC1	8.240511	5.313407	1.348864	3.939173	8.18E-05	0.000334	0	0.547412	0.695731	15.62489	23.53022	9.044815
6564	LOC11962:	8.473213	5.311904	1.3569	3.914734	9.05E-05	0.000367	0	0	1.391463	24.5534	12.83466	12.05975
9235	AGXT	4.434662	5.309488	1.743062	3.04607	0.002319	0.006674	0	0	0.695731	8.928508	14.97377	2.009959
5104	ZNF37A	11.91092	5.309421	1.147657	4.626315	3.72E-06	1.94E-05	1.084173	0.547412	0	24.5534	34.22577	11.05477
9184	CFAP97D1	4.356524	5.305989	1.733981	3.060005	0.002213	0.006407	0.542086	0	0	4.464254	17.11289	4.019918
3124	VWA5B1	24.97744	5.305142	0.849081	6.248099	4.15E-10	3.54E-09	0.542086	0.547412	2.782925	46.87467	77.00798	22.10955
6521	LOC10324:	8.411202	5.304406	1.349262	3.931339	8.45E-05	0.000344	0	0	1.391463	15.62489	21.39111	12.05975
9544	WNT16	4.403657	5.304058	1.783584	2.97382	0.002941	0.008192	0	0	0.695731	4.464254	19.252	2.009959
5483	PAX3	12.16949	5.297871	1.199353	4.417273	1.00E-05	4.85E-05	0	1.094824	0.695731	24.5534	40.6431	6.029877
11746	SGCG	2.10554	5.297594	2.154197	2.459196	0.013925	0.031516	0	0	0	4.464254	2.139111	6.029877
13038	LOC11961ł	2.201122	5.2973	2.410629	2.197477	0.027986	0.057052	0	0	0	8.928508	4.278221	0
13039	LOC10321(	2.201122	5.2973	2.410629	2.197477	0.027986	0.057052	0	0	0	8.928508	4.278221	0
13041	LOC10322;	2.201122	5.2973	2.410629	2.197477	0.027986	0.057052	0	0	0	8.928508	4.278221	0
7173	LOC11962ł	7.902323	5.297034	1.441884	3.67369	0.000239	0.000886	0	1.094824	0	15.62489	25.66933	5.024897
12033	LOC11962!	2.068512	5.296819	2.206445	2.400612	0.016368	0.036162	0	0	0	2.232127	2.139111	8.039836

11707	GDF10	2.142568	5.295262	2.143518	2.47036	0.013498	0.030651	0	0	0	6.696381	2.139111	4.019918
11862	LOC11962;	2.179596	5.293834	2.175018	2.433926	0.014936	0.033472	0	0	0	8.928508	2.139111	2.009959
11863	ELANE	2.179596	5.293834	2.175018	2.433926	0.014936	0.033472	0	0	0	8.928508	2.139111	2.009959
6862	DEFB128	8.491281	5.293668	1.395855	3.792421	0.000149	0.000578	0	0	1.391463	17.85702	25.66933	6.029877
6770	LOC11962‹	8.364694	5.293045	1.382952	3.827353	0.00013	0.000509	0	0	1.391463	8.928508	27.80844	12.05975
13018	MYF6	2.185619	5.288013	2.403049	2.200543	0.027768	0.056695	0	0	0	6.696381	6.417332	0
13019	LOC11962‹	2.185619	5.288013	2.403049	2.200543	0.027768	0.056695	0	0	0	6.696381	6.417332	0
13020	CLEC4C	2.185619	5.288013	2.403049	2.200543	0.027768	0.056695	0	0	0	6.696381	6.417332	0
13021	LOC11962;	2.185619	5.288013	2.403049	2.200543	0.027768	0.056695	0	0	0	6.696381	6.417332	0
5083	LOC11962!	12.31777	5.287043	1.138836	4.642496	3.44E-06	1.80E-05	0.542086	0	1.391463	24.5534	36.36488	11.05477
11588	CD3G	2.127066	5.287009	2.117351	2.496993	0.012525	0.028735	0	0	0	4.464254	4.278221	4.019918
5007	ODAD2	12.28163	5.286162	1.128345	4.684881	2.80E-06	1.49E-05	0	0.547412	1.391463	22.32127	36.36488	13.06473
3606	TMEM108	20.4442	5.284338	0.92169	5.733315	9.85E-09	7.26E-08	0	1.094824	2.087194	44.64254	57.75599	17.08465
11695	PKD2L2	2.164094	5.283987	2.137713	2.471794	0.013444	0.030544	0	0	0	6.696381	4.278221	2.009959
11696	CD2	2.164094	5.283987	2.137713	2.471794	0.013444	0.030544	0	0	0	6.696381	4.278221	2.009959
11697	LOC11962!	2.164094	5.283987	2.137713	2.471794	0.013444	0.030544	0	0	0	6.696381	4.278221	2.009959
11699	MAGEB4	2.164094	5.283987	2.137713	2.471794	0.013444	0.030544	0	0	0	6.696381	4.278221	2.009959
11701	LOC10323:	2.164094	5.283987	2.137713	2.471794	0.013444	0.030544	0	0	0	6.696381	4.278221	2.009959
11702	LOC11962(	2.164094	5.283987	2.137713	2.471794	0.013444	0.030544	0	0	0	6.696381	4.278221	2.009959
3604	DEFB1	19.01276	5.28097	0.920875	5.73473	9.77E-09	7.20E-08	2.710432	0	0	37.94616	34.22577	39.1942
13123	FHL5	2.170116	5.279694	2.418649	2.18291	0.029042	0.058799	0	0	0	4.464254	8.556443	0
13124	LOC11962;	2.170116	5.279694	2.418649	2.18291	0.029042	0.058799	0	0	0	4.464254	8.556443	0
13126	LOC10323:	2.170116	5.279694	2.418649	2.18291	0.029042	0.058799	0	0	0	4.464254	8.556443	0
13127	EXOC1L	2.170116	5.279694	2.418649	2.18291	0.029042	0.058799	0	0	0	4.464254	8.556443	0
13128	PDE6H	2.170116	5.279694	2.418649	2.18291	0.029042	0.058799	0	0	0	4.464254	8.556443	0
13129	LOC10322;	2.170116	5.279694	2.418649	2.18291	0.029042	0.058799	0	0	0	4.464254	8.556443	0
13130	LOC11962;	2.170116	5.279694	2.418649	2.18291	0.029042	0.058799	0	0	0	4.464254	8.556443	0
13131	LOC11962:	2.170116	5.279694	2.418649	2.18291	0.029042	0.058799	0	0	0	4.464254	8.556443	0
11761	LOC11962(	2.111563	5.27895	2.149992	2.455335	0.014075	0.031803	0	0	0	2.232127	6.417332	4.019918
11764	ENTHD1	2.111563	5.27895	2.149992	2.455335	0.014075	0.031803	0	0	0	2.232127	6.417332	4.019918
11765	MAGEL2	2.111563	5.27895	2.149992	2.455335	0.014075	0.031803	0	0	0	2.232127	6.417332	4.019918
11766	LOC11962:	2.111563	5.27895	2.149992	2.455335	0.014075	0.031803	0	0	0	2.232127	6.417332	4.019918
11723	MS4A1	2.148591	5.27578	2.14079	2.464408	0.013724	0.031112	0	0	0	4.464254	6.417332	2.009959
11724	LOC10322;	2.148591	5.27578	2.14079	2.464408	0.013724	0.031112	0	0	0	4.464254	6.417332	2.009959
11725	LOC10324;	2.148591	5.27578	2.14079	2.464408	0.013724	0.031112	0	0	0	4.464254	6.417332	2.009959
11726	WFDC10B	2.148591	5.27578	2.14079	2.464408	0.013724	0.031112	0	0	0	4.464254	6.417332	2.009959
11727	LOC10323;	2.148591	5.27578	2.14079	2.464408	0.013724	0.031112	0	0	0	4.464254	6.417332	2.009959
12872	LHX8	2.074535	5.275593	2.371884	2.224221	0.026134	0.053975	0	0	0	0	6.417332	6.029877
13352	LOC11962(	2.154613	5.272637	2.468506	2.135963	0.032682	0.065074	0	0	0	2.232127	10.69555	0
12897	LOC11962!	2.09606	5.27042	2.374947	2.219174	0.026475	0.054561	0	0	0	0	8.556443	4.019918
8981	LOC11962;	4.156344	5.2692	1.687585	3.122332	0.001794	0.005311	0	0.547412	0	8.928508	6.417332	9.044815
11960	LOC11962{	2.133088	5.268909	2.184471	2.411984	0.015866	0.035244	0	0	0	2.232127	8.556443	2.009959
11962	TRHR	2.133088	5.268909	2.184471	2.411984	0.015866	0.035244	0	0	0	2.232127	8.556443	2.009959
11963	IMPG1	2.133088	5.268909	2.184471	2.411984	0.015866	0.035244	0	0	0	2.232127	8.556443	2.009959
11964	P2RY13	2.133088	5.268909	2.184471	2.411984	0.015866	0.035244	0	0	0	2.232127	8.556443	2.009959
11965	LOC10324:	2.133088	5.268909	2.184471	2.411984	0.015866	0.035244	0	0	0	2.232127	8.556443	2.009959
11966	CUNH6orf:	2.133088	5.268909	2.184471	2.411984	0.015866	0.035244	0	0	0	2.232127	8.556443	2.009959
11967	TBPL2	2.133088	5.268909	2.184471	2.411984	0.015866	0.035244	0	0	0	2.232127	8.556443	2.009959
11968	LOC10321!	2.133088	5.268909	2.184471	2.411984	0.015866	0.035244	0	0	0	2.232127	8.556443	2.009959
9201	TMEM233	4.270623	5.268573	1.724931	3.054368	0.002255	0.006516	0	0	0.695731	15.62489	4.278221	5.024897
9202	LOC11962:	4.270623	5.268573	1.724931	3.054368	0.002255	0.006516	0	0	0.695731	15.62489	4.278221	5.024897
8983	BTNL10	4.218092	5.265753	1.686824	3.121696	0.001798	0.005322	0	0	0.695731	11.16063	6.417332	7.034856
8979	LOC11962:	4.165562	5.265156	1.686011	3.122848	0.001791	0.005303	0	0	0.695731	6.696381	8.556443	9.044815
8953	NLRP9	4.20259	5.261519	1.680855	3.130263	0.001747	0.005186	0	0	0.695731	8.928508	8.556443	7.034856
9085	LOC11962:	4.150059	5.260753	1.703589	3.088041	0.002015	0.005896	0	0	0.695731	4.464254	10.69555	9.044815
9014	AGTR1	4.162367	5.257558	1.689695	3.111544	0.001861	0.005489	0	0.547412	0	6.696381	10.69555	7.034856
5339	LOC10322(	11.83795	5.256748	1.169204	4.496008	6.92E-06	3.45E-05	0	1.094824	0.695731	31.24978	29.94755	8.039836
9216	NLRP13	4.236423	5.253175	1.721985	3.050651	0.002283	0.006587	0	0.547412	0	11.16063	10.69555	3.014938
9204	LOC11962:	4.261143	5.253015	1.720018	3.054046	0.002258	0.006521	0	0	0.695731	11.16063	10.69555	3.014938
3431	OLFM3	19.32175	5.251934	0.887731	5.916135	3.30E-09	2.55E-08	0.542086	1.642235	0.695731	53.57105	36.36488	23.11453
9477	LOC10323:	4.156082	5.249923	1.755064	2.991299	0.002778	0.007793	0	0	0.695731	2.232127	14.97377	7.034856
9772	NPTX2	4.267166	5.249811	1.80378	2.91045	0.003609	0.009819	0	0	0.695731	8.928508	14.97377	1.004979
5272	SLC34A3	12.06029	5.249353	1.15716	4.536409	5.72E-06	2.89E-05	0.542086	0	1.391463	35.71403	25.66933	9.044815
9269	LOC11962:	4.220033	5.249214	1.726536	3.040315	0.002363	0.006778	0.542086	0	0	8.928508	12.83466	3.014938
9268	LOC10321!	4.22092	5.249208	1.726465	3.040438	0.002362	0.006776	0	0.547412	0	8.928508	12.83466	3.014938
9243	LOC11961{	4.24564	5.249054	1.724485	3.043839	0.002336	0.006718	0	0	0.695731	8.928508	12.83466	3.014938
6916	NLRP7	7.958048	5.247539	1.39237	3.768782	0.000164	0.000631	0	0.547412	0.695731	20.08914	21.39111	5.024897
7234	LOC10322‹	7.964071	5.244813	1.434342	3.656598	0.000256	0.000939	0	0.547412	0.695731	17.85702	25.66933	3.014938
6848	SPN	7.890015	5.243196	1.380122	3.799081	0.000145	0.000564	0	0.547412	0.695731	13.39276	25.66933	7.034856
7025	LOC11962:	7.91154	5.241184	1.406995	3.72509	0.000195	0.000739	0	0.547412	0.695731	13.39276	27.80844	5.024897
5993	ART5	11.698	5.236983	1.256789	4.166954	3.09E-05	0.000137	0	1.094824	0.695731	29.01765	36.36488	3.014938
340	CCL7	189.8797	5.23594	0.316012	16.56879	1.17E-61	9.16E-60	5.420864	13.6853	10.43597	308.0335	444.935	356.7677
3778	COL19A1	20.0969	5.232436	0.939991	5.566477	2.60E-08	1.83E-07	0	0.547412	2.782925	42.41041	57.75599	17.08465
4137	LOC10321;	15.60049	5.231613	0.993424	5.266243	1.39E-07	8.95E-07	0	1.094824	1.391463	31.24978	42.78221	17.08465

7015	WIPF3	8.134762	5.228946	1.402614	3.728	0.000193	0.000731	0	0	1.391463	17.85702	23.53022	6.029877
4993	LOC11962:	11.6405	5.226383	1.113985	4.691611	2.71E-06	1.44E-05	0	0.547412	1.391463	17.85702	29.94755	20.09959
5013	KCNA1	11.71456	5.224444	1.116125	4.680878	2.86E-06	1.51E-05	0	0.547412	1.391463	22.32127	29.94755	16.07967
7298	WFDC8	8.156287	5.224049	1.43687	3.635714	0.000277	0.00101	0	0	1.391463	17.85702	25.66933	4.019918
4699	KLHL34	16.30053	5.22103	1.068945	4.884283	1.04E-06	5.87E-06	0	0	2.782925	40.17829	42.78221	12.05975
3822	BLK	19.9449	5.219431	0.945219	5.521925	3.35E-08	2.33E-07	0	0.547412	2.782925	44.64254	55.61688	16.07967
6781	LOC10324	7.731111	5.21719	1.364011	3.824887	0.000131	0.000513	0.542086	0	0.695731	17.85702	19.252	8.039836
1124	MROH2A	71.70198	5.21381	0.500199	10.42347	1.94E-25	4.58E-24	4.336691	1.094824	6.261582	91.51721	156.1551	170.8465
6954	LOC11962	7.927673	5.213744	1.388777	3.754198	0.000174	0.000665	0	0	1.391463	24.5534	8.556443	13.06473
6807	LOC11962(	7.716496	5.213022	1.366666	3.814409	0.000137	0.000533	0	0.547412	0.695731	15.62489	21.39111	8.039836
5472	LOC11962!	12.06437	5.21102	1.176971	4.427482	9.53E-06	4.63E-05	0	0	2.087194	35.71403	23.53022	11.05477
4578	PDE6A	15.48206	5.210751	1.051888	4.953711	7.28E-07	4.23E-06	0	1.094824	1.391463	29.01765	51.33866	10.04979
9552	LOC10322!	4.103127	5.203488	1.750947	2.971813	0.00296	0.00824	0	0	0.695731	15.62489	4.278221	4.019918
10794	CHRNB4	4.105955	5.203358	1.947663	2.671591	0.007549	0.018592	0	0.547412	0	13.39276	10.69555	0
6888	CLEC14A	7.924215	5.202684	1.376131	3.78066	0.000156	0.000604	0	0	1.391463	17.85702	19.252	9.044815
3992	KANK4	19.09323	5.202416	0.966973	5.380108	7.44E-08	4.96E-07	1.084173	0.547412	1.391463	46.87467	55.61688	9.044815
5772	UTS2R	11.76321	5.201722	1.213296	4.287267	1.81E-05	8.33E-05	0	0.547412	1.391463	37.94616	25.66933	5.024897
4439	LOC11962(	15.66826	5.20144	1.029825	5.050801	4.40E-07	2.63E-06	0	0.547412	2.087194	31.24978	47.06043	13.06473
9197	MINDY4B	3.998065	5.199188	1.70175	3.055201	0.002249	0.006501	0	0	0.695731	6.696381	8.556443	8.039836
10796	NELL2	4.115172	5.198346	1.94589	2.67145	0.007552	0.018596	0	0	0.695731	11.16063	12.83466	0
540	CXCL8	110.6205	5.196219	0.366186	14.19009	1.06E-45	5.19E-44	6.505036	5.474118	5.565851	247.7661	190.3808	208.0308
9340	LOC10323!	3.982562	5.194778	1.719539	3.021029	0.002519	0.00717	0	0	0.695731	4.464254	10.69555	8.039836
10857	LOC10322{	4.099669	5.193924	1.953301	2.65905	0.007836	0.019188	0	0	0.695731	8.928508	14.97377	0
9335	SHCBP1L	4.072121	5.192449	1.718003	3.022375	0.002508	0.007142	0	0	0.695731	11.16063	8.556443	4.019918
808	LOC10324(	76.5844	5.192397	0.431494	12.03354	2.37E-33	7.79E-32	4.336691	4.379295	3.478657	136.1597	143.3204	167.8316
10984	LOC11962!	4.059446	5.190101	1.971107	2.633089	0.008461	0.020477	0	0.547412	0	6.696381	17.11289	0
9665	LOC10323:	4.068926	5.188826	1.764	2.941511	0.003266	0.008984	0	0.547412	0	11.16063	10.69555	2.009959
9432	GPR148	4.004088	5.187349	1.729071	3.000078	0.002699	0.007606	0	0	0.695731	4.464254	12.83466	6.029877
9433	BICDL2	4.004088	5.187349	1.729071	3.000078	0.002699	0.007606	0	0	0.695731	4.464254	12.83466	6.029877
13685	LOC10323!	2.027602	5.187239	2.503394	2.072082	0.038258	0.074321	0	0	0	11.16063	0	1.004979
11113	CD5L	4.068664	5.186505	1.994662	2.600193	0.009317	0.022286	0	0	0.695731	4.464254	19.252	0
9685	MUC7	4.078144	5.184438	1.766341	2.935129	0.003334	0.009152	0	0	0.695731	8.928508	12.83466	2.009959
9450	GGT6	4.041116	5.184287	1.729442	2.997664	0.002721	0.007654	0	0	0.695731	6.696381	12.83466	4.019918
13378	TRNAM-CA	1.990574	5.180727	2.431143	2.130984	0.03309	0.065758	0	0	0	8.928508	0	3.014938
4355	LOC11962	15.33672	5.173055	1.012177	5.110822	3.21E-07	1.96E-06	0	0.547412	2.087194	37.94616	36.36488	15.07469
7065	SFTPD	7.517994	5.172531	1.395725	3.705981	0.000211	0.000792	0	0.547412	0.695731	11.16063	25.66933	7.034856
12315	LOC11962	1.938044	5.170973	2.207504	2.342453	0.019157	0.041349	0	0	0	4.464254	2.139111	5.024897
7302	CTXND1	7.539519	5.170171	1.422491	3.634589	0.000278	0.001014	0	0.547412	0.695731	11.16063	27.80844	5.024897
12639	LOC10322	2.0121	5.168332	2.274372	2.272422	0.023061	0.048503	0	0	0	8.928508	2.139111	1.004979
12640	LOC11962(	2.0121	5.168332	2.274372	2.272422	0.023061	0.048503	0	0	0	8.928508	2.139111	1.004979
4077	LOC10322	19.4749	5.168247	0.972367	5.31512	1.07E-07	6.95E-07	0	0	3.478657	22.32127	59.8951	31.15436
12309	LOC11962	1.975072	5.167925	2.206085	2.342577	0.019151	0.041346	0	0	0	6.696381	2.139111	3.014938
12310	LOC11962	1.975072	5.167925	2.206085	2.342577	0.019151	0.041346	0	0	0	6.696381	2.139111	3.014938
12311	LORICRIN	1.975072	5.167925	2.206085	2.342577	0.019151	0.041346	0	0	0	6.696381	2.139111	3.014938
12312	CPA2	1.975072	5.167925	2.206085	2.342577	0.019151	0.041346	0	0	0	6.696381	2.139111	3.014938
12314	LOC11962:	1.975072	5.167925	2.206085	2.342577	0.019151	0.041346	0	0	0	6.696381	2.139111	3.014938
2296	LOC11962!	29.54494	5.166834	0.696098	7.422563	1.15E-13	1.33E-12	1.084173	1.094824	2.782925	51.33892	72.72976	48.23901
12349	LOC11962	1.922541	5.162133	2.211451	2.334274	0.019581	0.042155	0	0	0	2.232127	4.278221	5.024897
5578	SLC8A3	11.09391	5.160914	1.177773	4.381925	1.18E-05	5.61E-05	0	1.094824	0.695731	26.78552	29.94755	8.039836
3734	LOC11962:	23.22605	5.159989	0.919834	5.609699	2.03E-08	1.44E-07	0	0.547412	3.478657	62.49956	57.75599	15.07469
12203	IL21	1.959569	5.158573	2.180556	2.365715	0.017995	0.039194	0	0	0	4.464254	4.278221	3.014938
12204	LOC11962	1.959569	5.158573	2.180556	2.365715	0.017995	0.039194	0	0	0	4.464254	4.278221	3.014938
12205	IL17A	1.959569	5.158573	2.180556	2.365715	0.017995	0.039194	0	0	0	4.464254	4.278221	3.014938
12482	LOC11962(	1.996597	5.157301	2.239069	2.303324	0.021261	0.045253	0	0	0	6.696381	4.278221	1.004979
12483	CUNH11or	1.996597	5.157301	2.239069	2.303324	0.021261	0.045253	0	0	0	6.696381	4.278221	1.004979
12484	LOC10324	1.996597	5.157301	2.239069	2.303324	0.021261	0.045253	0	0	0	6.696381	4.278221	1.004979
12486	BEND2	1.996597	5.157301	2.239069	2.303324	0.021261	0.045253	0	0	0	6.696381	4.278221	1.004979
12487	LOC11962(	1.996597	5.157301	2.239069	2.303324	0.021261	0.045253	0	0	0	6.696381	4.278221	1.004979
12488	LOC11962:	1.996597	5.157301	2.239069	2.303324	0.021261	0.045253	0	0	0	6.696381	4.278221	1.004979
12489	PRSS37	1.996597	5.157301	2.239069	2.303324	0.021261	0.045253	0	0	0	6.696381	4.278221	1.004979
12490	LOC10323	1.996597	5.157301	2.239069	2.303324	0.021261	0.045253	0	0	0	6.696381	4.278221	1.004979
40	SHISAL1	872.7307	5.150748	0.161919	31.81056	4.62E-222	3.07E-219	45.53526	42.69812	56.35424	1649.542	1567.968	1874.287
10695	LOC10322:	3.892579	5.150322	1.912452	2.693047	0.00708	0.0176	0	0	0.695731	15.62489	0	7.034856
12374	GZMB	1.944066	5.150104	2.213665	2.326505	0.019992	0.042927	0	0	0	2.232127	6.417332	3.014938
12375	NPY2R	1.944066	5.150104	2.213665	2.326505	0.019992	0.042927	0	0	0	2.232127	6.417332	3.014938
12376	LOC11962!	1.944066	5.150104	2.213665	2.326505	0.019992	0.042927	0	0	0	2.232127	6.417332	3.014938
12377	LOC11962!	1.944066	5.150104	2.213665	2.326505	0.019992	0.042927	0	0	0	2.232127	6.417332	3.014938
12378	LOC11962	1.944066	5.150104	2.213665	2.326505	0.019992	0.042927	0	0	0	2.232127	6.417332	3.014938
12379	LOC10323:	1.944066	5.150104	2.213665	2.326505	0.019992	0.042927	0	0	0	2.232127	6.417332	3.014938
12380	LOC10323:	1.944066	5.150104	2.213665	2.326505	0.019992	0.042927	0	0	0	2.232127	6.417332	3.014938
12381	LOC11962:	1.944066	5.150104	2.213665	2.326505	0.019992	0.042927	0	0	0	2.232127	6.417332	3.014938
13476	LOC11962(	1.907038	5.148878	2.438474	2.111516	0.034728	0.0685	0	0	0	0	6.417332	5.024897
13477	LOC11962	1.907038	5.148878	2.438474	2.111516	0.034728	0.0685	0	0	0	0	6.417332	5.024897
13478	LOC10323:	1.907038	5.148878	2.438474	2.111516	0.034728	0.0685	0	0	0	0	6.417332	5.024897

4584	MHC-DOA	15.14424	5.148586	1.039849	4.951283	7.37E-07	4.28E-06	0	0.547412	2.087194	31.24978	44.92132	12.05975
12527	LOC103219	1.981094	5.148236	2.242746	2.295506	0.021704	0.046017	0	0	0	4.464254	6.417332	1.004979
12528	MS4A14	1.981094	5.148236	2.242746	2.295506	0.021704	0.046017	0	0	0	4.464254	6.417332	1.004979
12529	LOC119624	1.981094	5.148236	2.242746	2.295506	0.021704	0.046017	0	0	0	4.464254	6.417332	1.004979
12530	LOC103218	1.981094	5.148236	2.242746	2.295506	0.021704	0.046017	0	0	0	4.464254	6.417332	1.004979
12532	DUOXA2	1.981094	5.148236	2.242746	2.295506	0.021704	0.046017	0	0	0	4.464254	6.417332	1.004979
12533	LOC119628	1.981094	5.148236	2.242746	2.295506	0.021704	0.046017	0	0	0	4.464254	6.417332	1.004979
12534	CALML5	1.981094	5.148236	2.242746	2.295506	0.021704	0.046017	0	0	0	4.464254	6.417332	1.004979
12535	LOC103215	1.981094	5.148236	2.242746	2.295506	0.021704	0.046017	0	0	0	4.464254	6.417332	1.004979
12537	LOC103242	1.981094	5.148236	2.242746	2.295506	0.021704	0.046017	0	0	0	4.464254	6.417332	1.004979
12538	LOC103226	1.981094	5.148236	2.242746	2.295506	0.021704	0.046017	0	0	0	4.464254	6.417332	1.004979
12539	LOC103232	1.981094	5.148236	2.242746	2.295506	0.021704	0.046017	0	0	0	4.464254	6.417332	1.004979
422	CXCL1	140.5768	5.147389	0.334431	15.39147	1.87E-53	1.18E-51	5.420864	7.663765	10.43597	319.1942	237.4413	263.3046
7119	LOC103243	7.428011	5.147357	1.395035	3.689768	0.000224	0.000838	0	0.547412	0.695731	22.32127	14.97377	6.029877
13550	LOC119626	1.928564	5.14297	2.45093	2.098375	0.035872	0.070355	0	0	0	0	8.556443	3.014938
13551	LOC119618	1.928564	5.14297	2.45093	2.098375	0.035872	0.070355	0	0	0	0	8.556443	3.014938
13552	LOC119619	1.928564	5.14297	2.45093	2.098375	0.035872	0.070355	0	0	0	0	8.556443	3.014938
13553	LOC119619	1.928564	5.14297	2.45093	2.098375	0.035872	0.070355	0	0	0	0	8.556443	3.014938
13554	LOC119620	1.928564	5.14297	2.45093	2.098375	0.035872	0.070355	0	0	0	0	8.556443	3.014938
12748	ANKRD7	1.965592	5.140816	2.285736	2.249086	0.024507	0.051064	0	0	0	2.232127	8.556443	1.004979
12749	LOC119627	1.965592	5.140816	2.285736	2.249086	0.024507	0.051064	0	0	0	2.232127	8.556443	1.004979
12750	LOC103216	1.965592	5.140816	2.285736	2.249086	0.024507	0.051064	0	0	0	2.232127	8.556443	1.004979
12751	LOC119619	1.965592	5.140816	2.285736	2.249086	0.024507	0.051064	0	0	0	2.232127	8.556443	1.004979
12752	LOC119619	1.965592	5.140816	2.285736	2.249086	0.024507	0.051064	0	0	0	2.232127	8.556443	1.004979
12753	LOC103214	1.965592	5.140816	2.285736	2.249086	0.024507	0.051064	0	0	0	2.232127	8.556443	1.004979
12754	LOC119620	1.965592	5.140816	2.285736	2.249086	0.024507	0.051064	0	0	0	2.232127	8.556443	1.004979
12755	LOC119620	1.965592	5.140816	2.285736	2.249086	0.024507	0.051064	0	0	0	2.232127	8.556443	1.004979
12756	PRR35	1.965592	5.140816	2.285736	2.249086	0.024507	0.051064	0	0	0	2.232127	8.556443	1.004979
12757	CD300LD	1.965592	5.140816	2.285736	2.249086	0.024507	0.051064	0	0	0	2.232127	8.556443	1.004979
12758	SPPL2C	1.965592	5.140816	2.285736	2.249086	0.024507	0.051064	0	0	0	2.232127	8.556443	1.004979
12759	LOC119623	1.965592	5.140816	2.285736	2.249086	0.024507	0.051064	0	0	0	2.232127	8.556443	1.004979
7359	LRTM1	7.418531	5.140294	1.422414	3.613781	0.000302	0.00109	0	0.547412	0.695731	17.85702	21.39111	4.019918
13880	LOC119624	1.950089	5.139189	2.527484	2.033322	0.04202	0.080471	0	0	0	0	10.69555	1.004979
13881	LOC119627	1.950089	5.139189	2.527484	2.033322	0.04202	0.080471	0	0	0	0	10.69555	1.004979
13882	LOC103234	1.950089	5.139189	2.527484	2.033322	0.04202	0.080471	0	0	0	0	10.69555	1.004979
9575	LOC119623	24.50977	5.139156	1.732238	2.966772	0.003009	0.008356	0	0	4.174388	44.64254	79.14709	19.09461
9919	AWAT1	3.93563	5.135714	1.7832	2.880054	0.003976	0.010657	0	0	0.695731	15.62489	4.278221	3.014938
10289	CD69	3.957155	5.134242	1.839572	2.790997	0.005255	0.013577	0	0	0.695731	15.62489	6.417332	1.004979
9618	HS3ST4	3.793541	5.13355	1.736812	2.955731	0.003119	0.008622	0	0	0.695731	4.464254	8.556443	9.044815
756	PILRA	102.0255	5.132079	0.412486	12.44184	1.55E-35	5.45E-34	3.252518	3.284471	11.1317	207.5878	216.0502	170.8465
4456	DYDC1	14.86566	5.131392	1.018633	5.037528	4.72E-07	2.81E-06	0	0.547412	2.087194	24.5534	44.92132	17.08465
9761	STPG3	3.920127	5.129326	1.760586	2.913419	0.003575	0.009737	0	0	0.695731	13.39276	6.417332	3.014938
5311	KLK1	15.37993	5.129163	1.136515	4.513064	6.39E-06	3.20E-05	0	0	2.782925	13.39276	62.03421	14.06971
8242	LOC103244	7.043315	5.127822	1.538407	3.333203	0.000859	0.002769	0	1.094824	0	15.62489	23.53022	2.009959
9540	KBTBD13	3.867597	5.125894	1.723425	2.974249	0.002937	0.008185	0	0	0.695731	8.928508	8.556443	5.024897
9617	LOC103231	3.827374	5.121722	1.732708	2.955907	0.003118	0.008618	0	0.547412	0	6.696381	10.69555	5.024897
9955	LOC119622	3.799563	5.121004	1.781996	2.873746	0.004056	0.010833	0	0	0.695731	2.232127	12.83466	7.034856
10264	FPR3	3.910647	5.118655	1.829417	2.79797	0.005142	0.01332	0	0	0.695731	8.928508	12.83466	1.004979
6150	LOC119624	11.11517	5.116494	1.248935	4.096686	4.19E-05	0.000181	0	0.547412	1.391463	20.08914	40.6431	4.019918
7626	ZC3H12D	6.932231	5.116454	1.454493	3.517689	0.000435	0.001518	0	1.094824	0	8.928508	23.53022	8.039836
9830	LOC103236	3.848899	5.11548	1.764633	2.898892	0.003745	0.010127	0	0.547412	0	6.696381	12.83466	3.014938
9811	LOC103243	3.873619	5.115322	1.762333	2.902586	0.003701	0.010029	0	0	0.695731	6.696381	12.83466	3.014938
10009	CD14	3.833396	5.112263	1.788611	2.858232	0.00426	0.011313	0	0.547412	0	4.464254	14.97377	3.014938
10011	LOC103230	3.833396	5.112263	1.788611	2.858232	0.00426	0.011313	0	0.547412	0	4.464254	14.97377	3.014938
10526	CCL19	3.879642	5.111633	1.866596	2.738478	0.006172	0.015587	0	0	0.695731	4.464254	17.11289	1.004979
10304	LOC103214	3.842614	5.109903	1.833141	2.787512	0.005311	0.013704	0	0	0.695731	2.232127	17.11289	3.014938
7259	LMLN2	7.351127	5.107567	1.399393	3.649844	0.000262	0.000961	0	0	1.391463	20.08914	8.556443	14.06971
4445	LOC119619	13.68314	5.102275	1.011033	5.046597	4.50E-07	2.69E-06	1.084173	1.094824	0	22.32127	38.50399	19.09461
5058	LY6L	15.10999	5.102081	1.095749	4.656249	3.22E-06	1.69E-05	0	0	2.782925	46.87467	29.94755	11.05477
5337	LOC103225	13.71807	5.101615	1.13464	4.496239	6.92E-06	3.45E-05	0	2.189647	0	35.71403	36.36488	8.039836
4480	KLK5	14.52465	5.097199	1.014558	5.02406	5.06E-07	3.00E-06	0	0.547412	2.087194	26.78552	40.6431	17.08465
5408	LOC119623	10.57352	5.096347	1.143031	4.458625	8.25E-06	4.05E-05	0.542086	0	1.391463	24.5534	12.83466	24.11951
3345	ITGA9	21.06396	5.089373	0.848219	6.00007	1.97E-09	1.57E-08	1.084173	1.094824	1.391463	49.10679	55.61688	18.08963
8112	LOC103227	6.869796	5.088356	1.507309	3.375788	0.000736	0.002412	0	1.094824	0	17.85702	19.252	3.014938
5822	LOC103236	11.12183	5.087021	1.193675	4.261645	2.03E-05	9.27E-05	0	0	2.087194	26.78552	27.80844	10.04979
348	PIK3IP1	169.632	5.086733	0.309387	16.44132	9.68E-61	7.39E-59	9.757555	10.94824	8.348776	334.819	260.9715	392.947
16	SLC2A2	2269.865	5.08617	0.11719	43.40113	0	0	130.6428	115.5039	144.7121	4357.112	4348.812	4522.408
7507	LOC103215	7.39072	5.084195	1.429791	3.555901	0.000377	0.001334	0	0	1.391463	13.39276	23.53022	6.029877
5931	TF	10.50788	5.081464	1.211791	4.193349	2.75E-05	0.000123	0	1.094824	0.695731	17.85702	36.36488	7.034856
8074	LOC103237	6.786259	5.078475	1.497646	3.390971	0.000696	0.002293	0	1.094824	0	8.928508	25.66933	5.024897
5593	LOC103242	10.74059	5.078409	1.1604	4.37643	1.21E-05	5.73E-05	0	0.547412	1.391463	26.78552	25.66933	10.04979
6388	CDH12	10.44456	5.066568	1.271974	3.983232	6.80E-05	0.000283	0.542086	0.547412	0.695731	26.78552	32.08666	2.009959
7385	ERVV-2	7.226681	5.062957	1.404107	3.60582	0.000311	0.00112	0	0	1.391463	20.08914	12.83466	9.044815
15398	LOC119620	1.860106	5.062307	2.876798	1.759702	0.078458	0.135451	0	0	0	11.16063	0	0

9782	DUSP21	3.678575	5.061873	1.740027	2.909077	0.003625	0.009852	0	0	0.695731	8.928508	6.417332	6.029877
9914	LOC119622	3.601324	5.061291	1.756739	2.881072	0.003963	0.010628	0	0.547412	0	4.464254	8.556443	8.039836
10243	LOC119623	3.752631	5.058593	1.803799	2.80441	0.005041	0.013083	0	0	0.695731	13.39276	6.417332	2.009959
9950	LOC103227	3.690883	5.058497	1.759339	2.875226	0.004037	0.010787	0	0.547412	0	11.16063	6.417332	4.019918
9925	ATP1B2	3.715603	5.058355	1.756888	2.879156	0.003987	0.010681	0	0	0.695731	11.16063	6.417332	4.019918
3870	FBN2	17.50852	5.057384	0.923253	5.477786	4.31E-08	2.96E-07	0	1.094824	2.087194	35.71403	47.06043	19.09461
7465	LOC119623	7.232703	5.05592	1.414268	3.574938	0.00035	0.001248	0	0	1.391463	17.85702	17.11289	7.034856
7397	CCRL2	7.180173	5.055387	1.403901	3.600956	0.000317	0.001139	0	0	1.391463	13.39276	19.252	9.044815
9896	PEAK3	3.7001	5.053203	1.751172	2.885611	0.003907	0.010495	0	0	0.695731	8.928508	8.556443	4.019918
4870	LOC119628	13.95051	5.051994	1.061509	4.759257	1.94E-06	1.06E-05	0	1.094824	1.391463	37.94616	34.22577	9.044815
9977	LOC103247	3.659877	5.048892	1.760674	2.86759	0.004136	0.011021	0	0.547412	0	6.696381	10.69555	4.019918
9966	RBMXL2	3.684597	5.048762	1.758191	2.871566	0.004084	0.010894	0	0	0.695731	6.696381	10.69555	4.019918
9967	GJD2	3.684597	5.048762	1.758191	2.871566	0.004084	0.010894	0	0	0.695731	6.696381	10.69555	4.019918
5753	LOC103222	10.54208	5.048549	1.17469	4.29777	1.73E-05	7.97E-05	0	0.547412	1.391463	22.32127	29.94755	9.044815
4966	LOC103246	14.49641	5.048067	1.072753	4.705714	2.53E-06	1.35E-05	0	0	2.782925	40.17829	29.94755	14.06971
10216	LOC103217	3.721625	5.047889	1.79512	2.812006	0.004923	0.012811	0	0	0.695731	8.928508	10.69555	2.009959
10217	LOC103221	3.721625	5.047889	1.79512	2.812006	0.004923	0.012811	0	0	0.695731	8.928508	10.69555	2.009959
8023	AVPR1A	7.275754	5.045044	1.481319	3.405779	0.00066	0.002186	0	0	1.391463	17.85702	21.39111	3.014938
14070	DHRS7C	1.823078	5.044765	2.522819	1.999654	0.045538	0.086036	0	0	0	8.928508	0	2.009959
14071	LOC103219	1.823078	5.044765	2.522819	1.999654	0.045538	0.086036	0	0	0	8.928508	0	2.009959
7345	ART1	6.823917	5.044419	1.394238	3.618047	0.000297	0.001074	0	0.547412	0.695731	20.08914	8.556443	11.05477
11140	FBXW12	3.616564	5.044383	1.944801	2.593778	0.009493	0.022654	0	0	0.695731	0	14.97377	6.029877
3925	PHACTR3	17.34102	5.041474	0.929313	5.424946	5.80E-08	3.93E-07	0	1.094824	2.087194	35.71403	47.06043	18.08963
14341	LOC103223	1.844603	5.041057	2.579562	1.95423	0.050674	0.093932	0	0	0	8.928508	2.139111	0
14342	LOC119625	1.844603	5.041057	2.579562	1.95423	0.050674	0.093932	0	0	0	8.928508	2.139111	0
13975	LOC103229	1.78605	5.040639	2.499483	2.016673	0.04373	0.083188	0	0	0	6.696381	0	4.019918
10450	LOC119619	3.69062	5.040357	1.829522	2.755013	0.005869	0.014929	0	0	0.695731	4.464254	14.97377	2.009959
10451	LOC103241	3.69062	5.040357	1.829522	2.755013	0.005869	0.014929	0	0	0.695731	4.464254	14.97377	2.009959
10726	LOC103227	3.675117	5.038148	1.875941	2.685665	0.007239	0.017941	0	0	0.695731	2.232127	17.11289	2.009959
5929	TMEM30B	10.7498	5.03579	1.200547	4.194579	2.73E-05	0.000123	0	0	2.087194	24.5534	27.80844	10.04979
7850	LOC119623	6.909131	5.032403	1.45743	3.452929	0.000555	0.001878	0.542086	0	0.695731	20.08914	17.11289	3.014938
12937	LOC119628	1.770547	5.031791	2.271345	2.215335	0.026737	0.054941	0	0	0	4.464254	2.139111	4.019918
14203	LOC103223	1.8291	5.028896	2.545126	1.975893	0.048167	0.090108	0	0	0	6.696381	4.278221	0
14204	ASPN	1.8291	5.028896	2.545126	1.975893	0.048167	0.090108	0	0	0	6.696381	4.278221	0
14205	WNT6	1.8291	5.028896	2.545126	1.975893	0.048167	0.090108	0	0	0	6.696381	4.278221	0
14206	LOC119619	1.8291	5.028896	2.545126	1.975893	0.048167	0.090108	0	0	0	6.696381	4.278221	0
14207	LOC119620	1.8291	5.028896	2.545126	1.975893	0.048167	0.090108	0	0	0	6.696381	4.278221	0
14208	TMPRSS11	1.8291	5.028896	2.545126	1.975893	0.048167	0.090108	0	0	0	6.696381	4.278221	0
14209	LOC119621	1.8291	5.028896	2.545126	1.975893	0.048167	0.090108	0	0	0	6.696381	4.278221	0
14210	RAB19	1.8291	5.028896	2.545126	1.975893	0.048167	0.090108	0	0	0	6.696381	4.278221	0
14211	LOC119622	1.8291	5.028896	2.545126	1.975893	0.048167	0.090108	0	0	0	6.696381	4.278221	0
13026	LOC103219	1.807575	5.028343	2.286463	2.19918	0.027865	0.056857	0	0	0	6.696381	2.139111	2.009959
13027	LOC103235	1.807575	5.028343	2.286463	2.19918	0.027865	0.056857	0	0	0	6.696381	2.139111	2.009959
13028	TAS2R40	1.807575	5.028343	2.286463	2.19918	0.027865	0.056857	0	0	0	6.696381	2.139111	2.009959
13029	PPP1R2C	1.807575	5.028343	2.286463	2.19918	0.027865	0.056857	0	0	0	6.696381	2.139111	2.009959
12984	ANGPTL5	1.755044	5.022085	2.276011	2.206529	0.027347	0.055989	0	0	0	2.232127	4.278221	4.019918
12985	LOC119621	1.755044	5.022085	2.276011	2.206529	0.027347	0.055989	0	0	0	2.232127	4.278221	4.019918
8947	TEX55	6.534802	5.018661	1.602011	3.132725	0.001732	0.005146	0	1.094824	0	17.85702	19.252	1.004979
14253	LOC103242	1.813598	5.018419	2.550713	1.967457	0.049131	0.091575	0	0	0	4.464254	6.417332	0
14255	LOC119625	1.813598	5.018419	2.550713	1.967457	0.049131	0.091575	0	0	0	4.464254	6.417332	0
14257	NXPE1	1.813598	5.018419	2.550713	1.967457	0.049131	0.091575	0	0	0	4.464254	6.417332	0
14258	LOC119619	1.813598	5.018419	2.550713	1.967457	0.049131	0.091575	0	0	0	4.464254	6.417332	0
14259	LOC119618	1.813598	5.018419	2.550713	1.967457	0.049131	0.091575	0	0	0	4.464254	6.417332	0
14260	LOC119619	1.813598	5.018419	2.550713	1.967457	0.049131	0.091575	0	0	0	4.464254	6.417332	0
14261	LOC103227	1.813598	5.018419	2.550713	1.967457	0.049131	0.091575	0	0	0	4.464254	6.417332	0
14262	LOC103239	1.813598	5.018419	2.550713	1.967457	0.049131	0.091575	0	0	0	4.464254	6.417332	0
14263	LOC103241	1.813598	5.018419	2.550713	1.967457	0.049131	0.091575	0	0	0	4.464254	6.417332	0
12902	LCE6A	1.792072	5.017677	2.261639	2.218602	0.026514	0.054578	0	0	0	4.464254	4.278221	2.009959
12903	CSRP3	1.792072	5.017677	2.261639	2.218602	0.026514	0.054578	0	0	0	4.464254	4.278221	2.009959
12904	SLC7A13	1.792072	5.017677	2.261639	2.218602	0.026514	0.054578	0	0	0	4.464254	4.278221	2.009959
12905	TAAR8	1.792072	5.017677	2.261639	2.218602	0.026514	0.054578	0	0	0	4.464254	4.278221	2.009959
12906	LOC119627	1.792072	5.017677	2.261639	2.218602	0.026514	0.054578	0	0	0	4.464254	4.278221	2.009959
12908	CETN1	1.792072	5.017677	2.261639	2.218602	0.026514	0.054578	0	0	0	4.464254	4.278221	2.009959
12909	F13B	1.792072	5.017677	2.261639	2.218602	0.026514	0.054578	0	0	0	4.464254	4.278221	2.009959
12910	FMO3	1.792072	5.017677	2.261639	2.218602	0.026514	0.054578	0	0	0	4.464254	4.278221	2.009959
12911	GIMAP1	1.792072	5.017677	2.261639	2.218602	0.026514	0.054578	0	0	0	4.464254	4.278221	2.009959
12912	LOC103231	1.792072	5.017677	2.261639	2.218602	0.026514	0.054578	0	0	0	4.464254	4.278221	2.009959
12913	LOC119622	1.792072	5.017677	2.261639	2.218602	0.026514	0.054578	0	0	0	4.464254	4.278221	2.009959
12914	LOC103229	1.792072	5.017677	2.261639	2.218602	0.026514	0.054578	0	0	0	4.464254	4.278221	2.009959
14162	R3HDML	1.718016	5.016034	2.529992	1.982628	0.047409	0.08899	0	0	0	0	4.278221	6.029877
14163	LOC119622	1.718016	5.016034	2.529992	1.982628	0.047409	0.08899	0	0	0	0	4.278221	6.029877
7948	LOC119623	7.086732	5.009957	1.462912	3.424647	0.000616	0.002059	0	0	1.391463	17.85702	19.252	4.019918
14121	LOC119628	1.739542	5.009711	2.517475	1.989975	0.046594	0.08772	0	0	0	0	6.417332	4.019918
14474	LOC119620	1.798095	5.009415	2.596405	1.929366	0.053685	0.098579	0	0	0	2.232127	8.556443	0

14475	TCHHL1	1.798095	5.009415	2.596405	1.929366	0.053685	0.098579	0	0	0	2.232127	8.556443	0
14476	LOC10323!	1.798095	5.009415	2.596405	1.929366	0.053685	0.098579	0	0	0	2.232127	8.556443	0
14477	LOC11962!	1.798095	5.009415	2.596405	1.929366	0.053685	0.098579	0	0	0	2.232127	8.556443	0
14478	LOC10323:	1.798095	5.009415	2.596405	1.929366	0.053685	0.098579	0	0	0	2.232127	8.556443	0
13134	LOC10323!	1.77657	5.008656	2.295441	2.182001	0.029109	0.058881	0	0	0	2.232127	6.417332	2.009959
13135	TAAR4	1.77657	5.008656	2.295441	2.182001	0.029109	0.058881	0	0	0	2.232127	6.417332	2.009959
13136	LOC10321.	1.77657	5.008656	2.295441	2.182001	0.029109	0.058881	0	0	0	2.232127	6.417332	2.009959
13137	NXPH2	1.77657	5.008656	2.295441	2.182001	0.029109	0.058881	0	0	0	2.232127	6.417332	2.009959
13140	LRRC52	1.77657	5.008656	2.295441	2.182001	0.029109	0.058881	0	0	0	2.232127	6.417332	2.009959
13141	TMPRSS11	1.77657	5.008656	2.295441	2.182001	0.029109	0.058881	0	0	0	2.232127	6.417332	2.009959
13142	LOC11962:	1.77657	5.008656	2.295441	2.182001	0.029109	0.058881	0	0	0	2.232127	6.417332	2.009959
13143	MAGEE2	1.77657	5.008656	2.295441	2.182001	0.029109	0.058881	0	0	0	2.232127	6.417332	2.009959
15685	GUCA2B	1.782592	5.007607	2.912289	1.719475	0.085528	0.144927	0	0	0	0	10.69555	0
15686	LOC11962	1.782592	5.007607	2.912289	1.719475	0.085528	0.144927	0	0	0	0	10.69555	0
15687	LHCGR	1.782592	5.007607	2.912289	1.719475	0.085528	0.144927	0	0	0	0	10.69555	0
15688	LYZL1	1.782592	5.007607	2.912289	1.719475	0.085528	0.144927	0	0	0	0	10.69555	0
15689	LOC11961!	1.782592	5.007607	2.912289	1.719475	0.085528	0.144927	0	0	0	0	10.69555	0
6132	ATP13A5	10.01488	5.006453	1.219846	4.104168	4.06E-05	0.000176	0	1.094824	0.695731	22.32127	29.94755	6.029877
8053	LOC10324:	7.055727	5.003773	1.472438	3.398291	0.000678	0.002239	0	0	1.391463	13.39276	23.53022	4.019918
14277	TEX53	1.761067	5.003162	2.545991	1.965114	0.049401	0.091938	0	0	0	0	8.556443	2.009959
14278	LOC11962(	1.761067	5.003162	2.545991	1.965114	0.049401	0.091938	0	0	0	0	8.556443	2.009959
14279	LOC11962	1.761067	5.003162	2.545991	1.965114	0.049401	0.091938	0	0	0	0	8.556443	2.009959
14281	LOC11962(	1.761067	5.003162	2.545991	1.965114	0.049401	0.091938	0	0	0	0	8.556443	2.009959
14282	LOC11962(	1.761067	5.003162	2.545991	1.965114	0.049401	0.091938	0	0	0	0	8.556443	2.009959
14284	LOC10324!	1.761067	5.003162	2.545991	1.965114	0.049401	0.091938	0	0	0	0	8.556443	2.009959
14285	ZPBP2	1.761067	5.003162	2.545991	1.965114	0.049401	0.091938	0	0	0	0	8.556443	2.009959
7955	LOC11962!	6.966168	5.001913	1.460937	3.423771	0.000618	0.002064	0	0	1.391463	6.696381	25.66933	8.039836
5737	TNF	10.17954	5.001773	1.161504	4.306289	1.66E-05	7.69E-05	0	0.547412	1.391463	24.5534	23.53022	11.05477
8139	LOC11962(	6.987693	4.996885	1.483895	3.367411	0.000759	0.002478	0	0	1.391463	6.696381	27.80844	6.029877
6014	GJA10	10.20709	4.994586	1.201056	4.158496	3.20E-05	0.000142	0	0.547412	1.391463	22.32127	29.94755	7.034856
4825	LOC11962.	13.38344	4.994583	1.041427	4.795902	1.62E-06	8.92E-06	0	1.094824	1.391463	37.94616	27.80844	12.05975
7559	CLEC4E	6.759078	4.991357	1.410524	3.538654	0.000402	0.001414	0	0	1.391463	13.39276	10.69555	15.07469
10143	LOC11962(	3.44933	4.990156	1.764759	2.82767	0.004689	0.012289	0	0.547412	0	6.696381	6.417332	7.034856
8206	INSM1	6.727019	4.987756	1.491406	3.344331	0.000825	0.002672	0	0.547412	0.695731	17.85702	19.252	2.009959
7619	GABRR1	6.652963	4.98612	1.416564	3.519869	0.000432	0.001507	0	0.547412	0.695731	13.39276	19.252	6.029877
7836	EGFL6	6.689991	4.985713	1.442348	3.456663	0.000547	0.001855	0	0.547412	0.695731	15.62489	19.252	4.019918
10152	LOC10324	3.511078	4.985473	1.764661	2.825173	0.004726	0.012375	0	0	0.695731	8.928508	6.417332	5.024897
7622	CLEC6A	6.600432	4.985297	1.416513	3.519414	0.000433	0.001509	0	0.547412	0.695731	8.928508	21.39111	8.039836
10331	LOC11961!	3.548106	4.982581	1.791461	2.781295	0.005414	0.013933	0	0	0.695731	11.16063	6.417332	3.014938
10195	TTC29	3.470855	4.98059	1.768118	2.816887	0.004849	0.012644	0	0.547412	0	6.696381	8.556443	5.024897
10196	LOC11962:	3.470855	4.98059	1.768118	2.816887	0.004849	0.012644	0	0.547412	0	6.696381	8.556443	5.024897
4019	GALNTL6	20.84901	4.980587	0.929685	5.357287	8.45E-08	5.59E-07	0	0	4.174388	40.17829	55.61688	25.12449
6412	LOC11962!	10.46734	4.980584	1.253101	3.974608	7.05E-05	0.000292	0	0	2.087194	29.01765	25.66933	6.029877
5097	LOC11962.	13.6032	4.980387	1.075423	4.631095	3.64E-06	1.90E-05	0	0.547412	2.087194	35.71403	34.22577	9.044815
10780	CUNH13or	3.544911	4.980243	1.862868	2.673428	0.007508	0.018511	0	0.547412	0	11.16063	8.556443	1.004979
7943	SYCE1	6.913213	4.976458	1.452832	3.425349	0.000614	0.002055	0	0	1.391463	20.08914	14.97377	5.024897
10384	LOC11962:	3.492381	4.972557	1.79564	2.769239	0.005619	0.014384	0	0.547412	0	6.696381	10.69555	3.014938
10385	LOC11961!	3.492381	4.972557	1.79564	2.769239	0.005619	0.014384	0	0.547412	0	6.696381	10.69555	3.014938
10893	CYLC2	3.513906	4.970394	1.874401	2.651724	0.008008	0.019544	0	0.547412	0	6.696381	12.83466	1.004979
10870	FOXB2	3.538626	4.970179	1.871663	2.655488	0.007919	0.019365	0	0	0.695731	6.696381	12.83466	1.004979
10871	SLC17A6	3.538626	4.970179	1.871663	2.655488	0.007919	0.019365	0	0	0.695731	6.696381	12.83466	1.004979
10872	LOC10321.	3.538626	4.970179	1.871663	2.655488	0.007919	0.019365	0	0	0.695731	6.696381	12.83466	1.004979
5250	CYP4F8	13.22799	4.969921	1.091575	4.552981	5.29E-06	2.68E-05	0	1.094824	1.391463	33.4819	36.36488	7.034856
11035	LOC10322(	3.523123	4.96647	1.894605	2.621375	0.008758	0.021098	0	0	0.695731	4.464254	14.97377	1.004979
11317	LOC10322!	3.482901	4.964184	1.94343	2.554341	0.010639	0.024992	0	0.547412	0	2.232127	17.11289	1.004979
6127	LOC11962	9.971562	4.963301	1.208896	4.105647	4.03E-05	0.000175	0	0.547412	1.391463	15.62489	34.22577	8.039836
8406	LOC10323(	6.681565	4.959008	1.511378	3.281116	0.001034	0.00327	0	0	1.391463	2.232127	21.39111	15.07469
8208	TMEM213	6.584505	4.956609	1.482192	3.344106	0.000825	0.002674	0	0.547412	0.695731	24.5534	10.69555	3.014938
8965	SLC6A14	6.909755	4.954291	1.584092	3.127528	0.001763	0.005228	0	0	1.391463	13.39276	25.66933	1.004979
8162	POU4F2	6.569003	4.95323	1.473789	3.36088	0.000777	0.002531	0	0.547412	0.695731	22.32127	12.83466	3.014938
3428	IL1RL2	19.26435	4.949665	0.836386	5.917921	3.26E-09	2.53E-08	0.542086	1.094824	2.087194	31.24978	53.47777	27.13445
7828	MEP1B	6.516472	4.949317	1.430924	3.458826	0.000543	0.001843	0	0.547412	0.695731	17.85702	14.97377	5.024897
6668	TEX15	10.25334	4.945856	1.278075	3.869771	0.000109	0.000434	0	0	2.087194	22.32127	32.08666	5.024897
7718	LOC11962.	6.448438	4.94529	1.417909	3.487736	0.000487	0.001678	0	0.547412	0.695731	11.16063	19.252	7.034856
6705	LOC10322(	10.23783	4.94341	1.283048	3.852863	0.000117	0.000463	0	0	2.087194	20.08914	34.22577	5.024897
7826	PRSS57	6.687163	4.942005	1.428704	3.459083	0.000542	0.001841	0	0	1.391463	17.85702	12.83466	8.039836
7784	PGLYRP4	6.61913	4.938454	1.4232	3.469964	0.000521	0.001778	0	0	1.391463	11.16063	17.11289	10.04979
8337	LOC10324:	6.475986	4.936687	1.495176	3.301744	0.000961	0.003064	0	0.547412	0.695731	8.928508	25.66933	3.014938
4819	LOC10321{	12.73886	4.929014	1.026678	4.800937	1.58E-06	8.71E-06	0	1.094824	1.391463	26.78552	32.08666	15.07469
3494	FAM163A	23.06731	4.928172	0.840632	5.86246	4.56E-09	3.47E-08	0	0.547412	4.174388	44.64254	59.8951	29.1444
8229	LOC11962!	6.714711	4.928	1.476617	3.337357	0.000846	0.002732	0	0	1.391463	15.62489	19.252	4.019918
8230	NPAP1	6.714711	4.928	1.476617	3.337357	0.000846	0.002732	0	0	1.391463	15.62489	19.252	4.019918
6263	LOC10324.	10.00576	4.927395	1.218247	4.044659	5.24E-05	0.000222	0	0	2.087194	20.08914	27.80844	10.04979
8099	LOC11962	6.66218	4.92709	1.457557	3.380376	0.000724	0.002376	0	0	1.391463	11.16063	21.39111	6.029877

8100	PLCXD3	6.66218	4.92709	1.457557	3.380376	0.000724	0.002376	0	0	1.391463	11.16063	21.39111	6.029877
8269	LOC10323(	6.699208	4.92472	1.482024	3.32297	0.000891	0.002863	0	0	1.391463	13.39276	21.39111	4.019918
6584	DNPH1	9.442577	4.923242	1.25981	3.907923	9.31E-05	0.000376	1.084173	0	0.695731	15.62489	34.22577	5.024897
7851	CPS1	6.007759	4.921758	1.425438	3.452804	0.000555	0.001879	0.542086	0.547412	0	8.928508	14.97377	11.05477
4532	TM4SF18	15.80281	4.919794	0.985259	4.993402	5.93E-07	3.48E-06	0	1.642235	1.391463	37.94616	42.78221	11.05477
8387	CLCA4	6.104228	4.918147	1.496348	3.286768	0.001013	0.003212	0	1.094824	0	13.39276	17.11289	5.024897
11947	LOC10324;	3.417638	4.917574	2.036505	2.414713	0.015748	0.035042	0	0	0.695731	13.39276	6.417332	0
8259	RLN2	6.0672	4.917172	1.478125	3.326627	0.000879	0.00283	0	1.094824	0	11.16063	17.11289	7.034856
8457	TEX33	6.668203	4.917038	1.505508	3.266033	0.001091	0.003429	0	0	1.391463	8.928508	25.66933	4.019918
10560	LOC10324;	3.269526	4.914384	1.799601	2.730818	0.006318	0.015905	0	0	0.695731	4.464254	6.417332	8.039836
6252	LOC10322;	9.615043	4.908685	1.211984	4.050122	5.12E-05	0.000218	0	0.547412	1.391463	15.62489	32.08666	8.039836
7891	LOC11961!	6.311947	4.906941	1.424615	3.444398	0.000572	0.001928	0	0.547412	0.695731	15.62489	14.97377	6.029877
10566	CATSPERB	3.318862	4.90503	1.797105	2.729407	0.006345	0.015964	0	0.547412	0	8.928508	6.417332	4.019918
4636	DPPA4	15.65081	4.904109	0.995588	4.925844	8.40E-07	4.82E-06	0	1.642235	1.391463	40.17829	40.6431	10.04979
10825	LOC10321!	3.35589	4.903643	1.839813	2.665295	0.007692	0.018887	0	0.547412	0	11.16063	6.417332	2.009959
10826	LOC10321{	3.35589	4.903643	1.839813	2.665295	0.007692	0.018887	0	0.547412	0	11.16063	6.417332	2.009959
10827	LOC10324:	3.35589	4.903643	1.839813	2.665295	0.007692	0.018887	0	0.547412	0	11.16063	6.417332	2.009959
7892	LOC10323/	6.44561	4.903504	1.423635	3.444355	0.000572	0.001928	0	0	1.391463	13.39276	12.83466	11.05477
10807	LOC10321!	3.38061	4.903419	1.83694	2.669341	0.0076	0.018694	0	0	0.695731	11.16063	6.417332	2.009959
10808	SPATA12	3.38061	4.903419	1.83694	2.669341	0.0076	0.018694	0	0	0.695731	11.16063	6.417332	2.009959
12050	MEPE	3.346409	4.90026	2.044105	2.397264	0.016518	0.036442	0	0.547412	0	6.696381	12.83466	0
10589	CXCR5	3.303359	4.899752	1.797996	2.725118	0.006428	0.016138	0	0.547412	0	6.696381	8.556443	4.019918
6400	FOLR2	9.838265	4.898168	1.230728	3.979895	6.89E-05	0.000286	0	0	2.087194	20.08914	27.80844	9.044815
10805	LOC10324:	3.339499	4.897738	1.834698	2.669506	0.007596	0.01869	0.542086	0	0	8.928508	8.556443	2.009959
10773	CBLIF	3.365107	4.897508	1.831704	2.673744	0.007501	0.018509	0	0	0.695731	8.928508	8.556443	2.009959
10774	LOC10321{	3.365107	4.897508	1.831704	2.673744	0.007501	0.018509	0	0	0.695731	8.928508	8.556443	2.009959
5409	CCDC166	13.06128	4.896582	1.09831	4.458286	8.26E-06	4.06E-05	0	0	2.782925	17.85702	40.6431	17.08465
6104	SFTPB	9.243285	4.896106	1.188576	4.119304	3.80E-05	0.000166	0	1.094824	0.695731	20.08914	23.53022	10.04979
8020	LOC10323;	6.414605	4.895836	1.437194	3.406525	0.000658	0.002181	0	0	1.391463	8.928508	17.11289	11.05477
12174	PROKR2	3.355627	4.895506	2.064653	2.371103	0.017735	0.038729	0	0	0.695731	4.464254	14.97377	0
10689	LOC11962:	3.287856	4.895161	1.816409	2.694967	0.00704	0.017508	0	0.547412	0	4.464254	10.69555	4.019918
10669	FAM83F	3.312576	4.895045	1.813444	2.699309	0.006948	0.017312	0	0	0.695731	4.464254	10.69555	4.019918
10670	LOC11961{	3.312576	4.895045	1.813444	2.699309	0.006948	0.017312	0	0	0.695731	4.464254	10.69555	4.019918
10849	LOC10321!	3.349604	4.892527	1.838743	2.660799	0.007796	0.019101	0	0	0.695731	6.696381	10.69555	2.009959
10850	LOC11962:	3.349604	4.892527	1.838743	2.660799	0.007796	0.019101	0	0	0.695731	6.696381	10.69555	2.009959
10990	LOC10324;	3.297073	4.891203	1.858728	2.631479	0.008501	0.020565	0	0	0.695731	2.232127	12.83466	4.019918
6117	PGLYRP3	9.488032	4.890259	1.189522	4.111112	3.94E-05	0.000171	0	0.547412	1.391463	24.5534	21.39111	9.044815
11861	LOC10323;	3.281571	4.889268	2.008707	2.434037	0.014931	0.033467	0	0	0.695731	0	14.97377	4.019918
6599	TAFA2	9.754729	4.889007	1.25248	3.90346	9.48E-05	0.000382	0	0	2.087194	11.16063	34.22577	11.05477
3528	SGPP2	18.56744	4.886409	0.83843	5.828044	5.61E-09	4.23E-08	0.542086	0.547412	2.782925	42.41041	29.94755	35.17428
4843	LOC10322;	16.32693	4.882504	1.019981	4.78686	1.69E-06	9.30E-06	0	0	3.478657	33.4819	44.92132	16.07967
13813	TCF24	1.566022	4.88084	2.386368	2.045301	0.040825	0.078572	0	0	0	2.232127	2.139111	5.024897
6586	GJA8	9.484575	4.878992	1.249046	3.906174	9.38E-05	0.000379	0	0.547412	1.391463	17.85702	32.08666	5.024897
8669	CKM	5.952234	4.877767	1.518839	3.21151	0.00132	0.004049	0	1.094824	0	15.62489	14.97377	4.019918
13663	FGG	1.603051	4.877359	2.349783	2.075664	0.037925	0.073782	0	0	0	4.464254	2.139111	3.014938
13665	LOC10321{	1.603051	4.877359	2.349783	2.075664	0.037925	0.073782	0	0	0	4.464254	2.139111	3.014938
13900	LOC11962/	1.640079	4.874752	2.401963	2.029486	0.042409	0.08107	0	0	0	6.696381	2.139111	1.004979
13901	LOC10323!	1.640079	4.874752	2.401963	2.029486	0.042409	0.08107	0	0	0	6.696381	2.139111	1.004979
13902	CXCR6	1.640079	4.874752	2.401963	2.029486	0.042409	0.08107	0	0	0	6.696381	2.139111	1.004979
13903	LOC11961{	1.640079	4.874752	2.401963	2.029486	0.042409	0.08107	0	0	0	6.696381	2.139111	1.004979
13904	PRSS38	1.640079	4.874752	2.401963	2.029486	0.042409	0.08107	0	0	0	6.696381	2.139111	1.004979
13905	LOC11962(	1.640079	4.874752	2.401963	2.029486	0.042409	0.08107	0	0	0	6.696381	2.139111	1.004979
13906	LOC11962:	1.640079	4.874752	2.401963	2.029486	0.042409	0.08107	0	0	0	6.696381	2.139111	1.004979
13907	LOC11962:	1.640079	4.874752	2.401963	2.029486	0.042409	0.08107	0	0	0	6.696381	2.139111	1.004979
8619	EVX1	6.463678	4.87396	1.511717	3.224122	0.001264	0.003898	0	0	1.391463	6.696381	25.66933	5.024897
15052	LOC11962:	1.528994	4.868089	2.668948	1.823973	0.068156	0.120378	0	0	0	0	2.139111	7.034856
7008	LOC11962;	9.77583	4.867919	1.305091	3.729946	0.000192	0.000727	0	0	2.087194	29.01765	23.53022	4.019918
13715	LOC11962(	1.587548	4.866583	2.355373	2.066163	0.038813	0.075197	0	0	0	2.232127	4.278221	3.014938
13717	LOC11961!	1.587548	4.866583	2.355373	2.066163	0.038813	0.075197	0	0	0	2.232127	4.278221	3.014938
13718	LOC10322!	1.587548	4.866583	2.355373	2.066163	0.038813	0.075197	0	0	0	2.232127	4.278221	3.014938
13719	LOC10322!	1.587548	4.866583	2.355373	2.066163	0.038813	0.075197	0	0	0	2.232127	4.278221	3.014938
13720	HS3ST2	1.587548	4.866583	2.355373	2.066163	0.038813	0.075197	0	0	0	2.232127	4.278221	3.014938
13722	LOC10324:	1.587548	4.866583	2.355373	2.066163	0.038813	0.075197	0	0	0	2.232127	4.278221	3.014938
13723	LOC10323:	1.587548	4.866583	2.355373	2.066163	0.038813	0.075197	0	0	0	2.232127	4.278221	3.014938
6625	LOC10324(	9.655266	4.865664	1.250402	3.89128	9.97E-05	0.0004	0	0	2.087194	17.85702	29.94755	8.039836
8142	LOC10322(	6.159953	4.865161	1.445246	3.366321	0.000762	0.002487	0	0.547412	0.695731	17.85702	12.83466	5.024897
8143	AICDA	6.159953	4.865161	1.445246	3.366321	0.000762	0.002487	0	0.547412	0.695731	17.85702	12.83466	5.024897
6628	LOC10321{	9.118839	4.864973	1.250894	3.889198	0.000101	0.000403	0	1.094824	0.695731	20.08914	27.80844	5.024897
14879	LOC11961{	1.55052	4.863133	2.617493	1.857936	0.063178	0.112845	0	0	0	0	4.278221	5.024897
13820	LOC11962(	1.624576	4.862547	2.378649	2.044248	0.040929	0.078671	0	0	0	4.464254	4.278221	1.004979
13822	LHFPL4	1.624576	4.862547	2.378649	2.044248	0.040929	0.078671	0	0	0	4.464254	4.278221	1.004979
13823	LOC11962;	1.624576	4.862547	2.378649	2.044248	0.040929	0.078671	0	0	0	4.464254	4.278221	1.004979
13824	LOC11962{	1.624576	4.862547	2.378649	2.044248	0.040929	0.078671	0	0	0	4.464254	4.278221	1.004979
13826	LOC11961!	1.624576	4.862547	2.378649	2.044248	0.040929	0.078671	0	0	0	4.464254	4.278221	1.004979

13827	LOC10323	1.624576	4.862547	2.378649	2.044248	0.040929	0.078671	0	0	0	4.464254	4.278221	1.004979
13828	LOC11962	1.624576	4.862547	2.378649	2.044248	0.040929	0.078671	0	0	0	4.464254	4.278221	1.004979
13830	LOC10323	1.624576	4.862547	2.378649	2.044248	0.040929	0.078671	0	0	0	4.464254	4.278221	1.004979
13831	LOC11962	1.624576	4.862547	2.378649	2.044248	0.040929	0.078671	0	0	0	4.464254	4.278221	1.004979
8588	RHCG	5.985971	4.85918	1.50299	3.23301	0.001225	0.003792	0.542086	0	0.695731	2.232127	21.39111	11.05477
8082	LBX2	6.278114	4.858985	1.434384	3.387505	0.000705	0.00232	0	0	1.391463	13.39276	12.83466	10.04979
8320	RFX8	6.023887	4.856969	1.468232	3.308039	0.00094	0.003002	0	0.547412	0.695731	4.464254	21.39111	9.044815
14878	GZMK	1.572045	4.855422	2.613323	1.85795	0.063176	0.112845	0	0	0	0	6.417332	3.014938
14880	LOC10321	1.572045	4.855422	2.613323	1.85795	0.063176	0.112845	0	0	0	0	6.417332	3.014938
14881	LOC11961	1.572045	4.855422	2.613323	1.85795	0.063176	0.112845	0	0	0	0	6.417332	3.014938
14884	LOC11961	1.572045	4.855422	2.613323	1.85795	0.063176	0.112845	0	0	0	0	6.417332	3.014938
20	C3	22604.7	4.853395	0.126933	38.23588	0	0	1350.879	1811.933	1371.287	42519.79	40688.02	47886.27
8273	CDH19	6.35217	4.853366	1.461166	3.32157	0.000895	0.002876	0	0	1.391463	17.85702	12.83466	6.029877
13992	LOC10321	1.609073	4.852649	2.412805	2.011207	0.044304	0.084069	0	0	0	2.232127	6.417332	1.004979
13993	LOC10321	1.609073	4.852649	2.412805	2.011207	0.044304	0.084069	0	0	0	2.232127	6.417332	1.004979
13995	LOC11962	1.609073	4.852649	2.412805	2.011207	0.044304	0.084069	0	0	0	2.232127	6.417332	1.004979
13996	LOC10322	1.609073	4.852649	2.412805	2.011207	0.044304	0.084069	0	0	0	2.232127	6.417332	1.004979
13997	LOC11962	1.609073	4.852649	2.412805	2.011207	0.044304	0.084069	0	0	0	2.232127	6.417332	1.004979
13998	LOC10323	1.609073	4.852649	2.412805	2.011207	0.044304	0.084069	0	0	0	2.232127	6.417332	1.004979
13999	LOC11962	1.609073	4.852649	2.412805	2.011207	0.044304	0.084069	0	0	0	2.232127	6.417332	1.004979
14001	LOC10321	1.609073	4.852649	2.412805	2.011207	0.044304	0.084069	0	0	0	2.232127	6.417332	1.004979
14003	LOC11961	1.609073	4.852649	2.412805	2.011207	0.044304	0.084069	0	0	0	2.232127	6.417332	1.004979
14004	LOC10323	1.609073	4.852649	2.412805	2.011207	0.044304	0.084069	0	0	0	2.232127	6.417332	1.004979
14005	LOC10323	1.609073	4.852649	2.412805	2.011207	0.044304	0.084069	0	0	0	2.232127	6.417332	1.004979
14006	TEKT1	1.609073	4.852649	2.412805	2.011207	0.044304	0.084069	0	0	0	2.232127	6.417332	1.004979
14007	EDDM3B	1.609073	4.852649	2.412805	2.011207	0.044304	0.084069	0	0	0	2.232127	6.417332	1.004979
14008	LOC10323	1.609073	4.852649	2.412805	2.011207	0.044304	0.084069	0	0	0	2.232127	6.417332	1.004979
14009	LOC11962	1.609073	4.852649	2.412805	2.011207	0.044304	0.084069	0	0	0	2.232127	6.417332	1.004979
14010	CACNG1	1.609073	4.852649	2.412805	2.011207	0.044304	0.084069	0	0	0	2.232127	6.417332	1.004979
8590	SCRG1	6.404701	4.852604	1.501272	3.232329	0.001228	0.0038	0	0	1.391463	22.32127	10.69555	4.019918
2595	LOC10323	27.00619	4.85123	0.694234	6.987884	2.79E-12	2.86E-11	1.084173	1.642235	2.782925	64.73168	55.61688	36.17926
15124	LOC11962	1.59357	4.848859	2.679551	1.809579	0.070361	0.123656	0	0	0	0	8.556443	1.004979
15125	LOC11961	1.59357	4.848859	2.679551	1.809579	0.070361	0.123656	0	0	0	0	8.556443	1.004979
15126	LOC10321	1.59357	4.848859	2.679551	1.809579	0.070361	0.123656	0	0	0	0	8.556443	1.004979
15127	LOC10323	1.59357	4.848859	2.679551	1.809579	0.070361	0.123656	0	0	0	0	8.556443	1.004979
2210	LOC10323	32.66754	4.845532	0.638981	7.583215	3.37E-14	4.06E-13	1.626259	1.642235	3.478657	87.05295	44.92132	57.28383
11974	LOC11962	3.185565	4.845444	2.009556	2.411201	0.0159	0.035302	0	0	0.695731	13.39276	0	5.024897
6715	LOC11962	9.518775	4.840228	1.257268	3.849798	0.000118	0.000468	0	0	2.087194	22.32127	25.66933	7.034856
5099	THEG	11.97932	4.839238	1.045368	4.629218	3.67E-06	1.91E-05	0	1.094824	1.391463	20.08914	34.22577	15.07469
11763	NLRP14	3.218511	4.838596	1.970641	2.455341	0.014075	0.031803	0.542086	0	0	15.62489	2.139111	1.004979
4284	KCNQ3	18.43822	4.836869	0.936468	5.165015	2.40E-07	1.49E-06	0.542086	0.547412	2.782925	37.94616	57.75599	11.05477
11120	MUC15	3.170062	4.836709	1.861871	2.597769	0.009383	0.022433	0	0	0.695731	11.16063	2.139111	5.024897
8024	SBK3	5.85896	4.834685	1.420143	3.404364	0.000663	0.002197	0.542086	0	0.695731	11.16063	10.69555	12.05975
5679	FAM180A	12.62683	4.83452	1.114841	4.33651	1.45E-05	6.78E-05	0	0	2.782925	24.5534	36.36488	12.05975
7418	TMEM211	8.856551	4.831407	1.344782	3.592705	0.000327	0.001173	1.084173	0	0.695731	6.696381	40.6431	4.019918
10923	LOC10322	3.128952	4.83028	1.825134	2.646535	0.008132	0.019792	0.542086	0	0	8.928508	4.278221	5.024897
10895	LOC10323	3.102029	4.829848	1.82169	2.651301	0.008018	0.019565	0	0	0.695731	4.464254	6.417332	7.034856
5113	TH	15.83994	4.829673	1.044965	4.62185	3.80E-06	1.98E-05	0	0	3.478657	35.71403	42.78221	13.06473
11542	LOC10322	3.203896	4.829333	1.927675	2.505263	0.012236	0.028181	0	0.547412	0	13.39276	4.278221	1.004979
3110	PVRIG	23.87981	4.828727	0.770242	6.269102	3.63E-10	3.10E-09	0.542086	1.642235	2.782925	46.87467	66.31243	25.12449
11062	LOC11961	3.191588	4.826523	1.847273	2.612783	0.008981	0.021581	0	0	0.695731	11.16063	4.278221	3.014938
34	PLEK	890.2486	4.825235	0.146748	32.88116	4.09E-237	3.20E-234	52.04029	64.59459	66.09448	1736.595	1651.393	1770.774
10836	LOC10323	3.139057	4.824457	1.811641	2.663031	0.007744	0.018997	0	0	0.695731	6.696381	6.417332	5.024897
10837	LOC10323	3.139057	4.824457	1.811641	2.663031	0.007744	0.018997	0	0	0.695731	6.696381	6.417332	5.024897
6381	CDH15	9.175618	4.823828	1.209987	3.986676	6.70E-05	0.000279	0	0	2.087194	15.62489	19.252	18.08963
11455	LOC10323	3.187505	4.821868	1.909103	2.525724	0.011546	0.026796	0.542086	0	0	11.16063	6.417332	1.004979
4100	NKX6-3	21.75488	4.821071	0.910323	5.296003	1.18E-07	7.67E-07	0	0.547412	4.174388	42.41041	66.31243	17.08465
12036	CLIC3	3.049498	4.820207	2.008583	2.399805	0.016404	0.03623	0	0	0.695731	0	8.556443	9.044815
12037	LOC11962	3.049498	4.820207	2.008583	2.399805	0.016404	0.03623	0	0	0.695731	0	8.556443	9.044815
5613	OPTC	12.48431	4.819984	1.103949	4.366128	1.26E-05	5.99E-05	0	0	2.782925	31.24978	27.80844	13.06473
430	ZNF227	130.4179	4.816124	0.314423	15.31733	5.86E-53	3.62E-51	10.84173	8.758589	6.957314	238.8376	258.8324	258.2797
11438	RFPL4A	3.172003	4.815508	1.904142	2.528964	0.01144	0.02659	0.542086	0	0	8.928508	8.556443	1.004979
11437	IL9	3.17289	4.815498	1.904027	2.529113	0.011435	0.026581	0	0.547412	0	8.928508	8.556443	1.004979
11030	NCR1	3.135862	4.814416	1.835245	2.623309	0.008708	0.020988	0	0.547412	0	6.696381	8.556443	3.014938
11228	LOC10322	3.083332	4.81414	1.869779	2.574711	0.010032	0.023752	0	0.547412	0	2.232127	10.69555	5.024897
11229	LOC10322	3.083332	4.81414	1.869779	2.574711	0.010032	0.023752	0	0.547412	0	2.232127	10.69555	5.024897
7220	KLK9	9.419312	4.810617	1.313958	3.661164	0.000251	0.000924	0	0	2.087194	29.01765	21.39111	4.019918
5350	SLC7A14	12.1091	4.810415	1.071543	4.48924	7.15E-06	3.55E-05	0	0.547412	2.087194	29.01765	29.94755	11.05477
11479	ANO9	3.182108	4.809835	1.907868	2.521052	0.0117	0.027095	0	0	0.695731	6.696381	10.69555	1.004979
11480	LOC10324	3.182108	4.809835	1.907868	2.521052	0.0117	0.027095	0	0	0.695731	6.696381	10.69555	1.004979
11135	PPP1R17	3.12036	4.809671	1.853611	2.594757	0.009466	0.022596	0	0.547412	0	4.464254	10.69555	3.014938
11137	MC2R	3.12036	4.809671	1.853611	2.594757	0.009466	0.022596	0	0.547412	0	4.464254	10.69555	3.014938
11118	LOC11962	3.14508	4.809506	1.850393	2.599182	0.009345	0.022346	0	0	0.695731	4.464254	10.69555	3.014938
8353	CACNG3	6.132143	4.807133	1.45831	3.296374	0.000979	0.003117	0	0	1.391463	13.39276	14.97377	7.034856

11424	MMP26	3.104857	4.805965	1.899047	2.530725	0.011383	0.026489	0	0.547412	0	2.232127	12.83466	3.014938
11403	STX19	3.129577	4.805824	1.895733	2.535075	0.011242	0.026206	0	0	0.695731	2.232127	12.83466	3.014938
11404	LOC11961!	3.129577	4.805824	1.895733	2.535075	0.011242	0.026206	0	0	0.695731	2.232127	12.83466	3.014938
11405	LOC11962(	3.129577	4.805824	1.895733	2.535075	0.011242	0.026206	0	0	0.695731	2.232127	12.83466	3.014938
11632	STEAP4	3.141885	4.805642	1.932295	2.487013	0.012882	0.029443	0	0.547412	0	4.464254	12.83466	1.004979
11612	LY9	3.166605	4.80541	1.929039	2.49109	0.012735	0.029151	0	0	0.695731	4.464254	12.83466	1.004979
11613	SLC5A4	3.166605	4.80541	1.929039	2.49109	0.012735	0.029151	0	0	0.695731	4.464254	12.83466	1.004979
8374	TNFAIP8L2	6.079612	4.805222	1.460125	3.290967	0.000998	0.003169	0	0	1.391463	8.928508	17.11289	9.044815
8375	LOC10322;	6.079612	4.805222	1.460125	3.290967	0.000998	0.003169	0	0	1.391463	8.928508	17.11289	9.044815
12304	SPAM1	3.114074	4.805055	2.050561	2.343288	0.019115	0.041297	0	0	0.695731	0	14.97377	3.014938
12305	LOC10321(	3.114074	4.805055	2.050561	2.343288	0.019115	0.041297	0	0	0.695731	0	14.97377	3.014938
4727	LOC11962‹	18.71246	4.804814	0.987536	4.865458	1.14E-06	6.42E-06	0	0	4.174388	31.24978	57.75599	19.09461
4201	GRM1	21.50296	4.804651	0.921036	5.216575	1.82E-07	1.15E-06	0.542086	0	4.174388	33.4819	72.72976	18.08963
4984	LOC10322;	11.42367	4.802588	1.022502	4.696899	2.64E-06	1.41E-05	0.542086	1.094824	0.695731	22.32127	27.80844	16.07967
6337	LOC10322;	8.843453	4.797906	1.196386	4.010332	6.06E-05	0.000254	0	0.547412	1.391463	13.39276	25.66933	12.05975
2797	TP63	39.462	4.796422	0.715855	6.700268	2.08E-11	1.98E-10	0.542086	1.094824	6.957314	87.05295	106.9555	34.1693
8482	LOC10323{	5.738859	4.791718	1.470131	3.259382	0.001117	0.003499	0	0.547412	0.695731	17.85702	4.278221	11.05477
9025	ZNF560	5.631856	4.788746	1.540413	3.108742	0.001879	0.005535	0.542086	0.547412	0	17.85702	12.83466	2.009959
184	TSPAN8	524.9707	4.784266	0.230926	20.71773	2.40E-95	3.46E-93	32.52518	22.9913	56.35424	1058.028	983.9909	995.9347
8829	BCL2L15	5.543185	4.781763	1.511782	3.162997	0.001562	0.004702	0	1.094824	0	11.16063	14.97377	6.029877
8293	LOC11962‹	5.766407	4.778123	1.441333	3.315073	0.000916	0.002937	0	0.547412	0.695731	15.62489	10.69555	7.034856
8430	LOC10324!	5.787932	4.772652	1.458027	3.273364	0.001063	0.003352	0	0.547412	0.695731	15.62489	12.83466	5.024897
8414	LOC10324;	5.921595	4.770742	1.455358	3.278053	0.001045	0.003303	0	0	1.391463	13.39276	10.69555	10.04979
9257	LOC10322;	5.830983	4.769064	1.568058	3.041381	0.002355	0.006763	0	0.547412	0.695731	15.62489	17.11289	1.004979
32	PDZK1IP1	1861.137	4.768997	0.142706	33.41841	7.41E-245	6.15E-242	145.8212	105.6505	144.7121	3332.566	3482.472	3955.599
9299	CBLL2	5.81548	4.765863	1.571609	3.032473	0.002426	0.006935	0	0.547412	0.695731	13.39276	19.252	1.004979
8466	CUNH5orf(	5.943121	4.765403	1.46033	3.263237	0.001101	0.003459	0	0	1.391463	13.39276	12.83466	8.039836
8507	LOC11962!	5.89059	4.763491	1.463407	3.255068	0.001134	0.003543	0	0	1.391463	8.928508	14.97377	10.04979
12629	LOC11962(	3.055097	4.761406	2.092512	2.27545	0.022879	0.048162	0	0	0.695731	15.62489	0	2.009959
9607	SLC16A8	5.768084	4.760058	1.608188	2.959889	0.003077	0.008516	0.542086	0	0.695731	6.696381	25.66933	1.004979
6660	LOC10324(	8.914681	4.759909	1.228934	3.873202	0.000107	0.000429	0	0	2.087194	20.08914	19.252	12.05975
9254	LOC10321!	5.694028	4.757957	1.564059	3.042056	0.00235	0.00675	0.542086	0	0.695731	2.232127	25.66933	5.024897
8657	LOC10321!	5.875087	4.75788	1.480264	3.214211	0.001308	0.004017	0	0	1.391463	6.696381	17.11289	10.04979
5630	FOXL2	15.21646	4.757773	1.091683	4.3582	1.31E-05	6.19E-05	0	0	3.478657	42.41041	36.36488	9.044815
7307	MAPK4	8.77756	4.755997	1.309132	3.63294	0.00028	0.001019	0	0.547412	1.391463	15.62489	32.08666	3.014938
5521	LOC11961{	15.10192	4.751448	1.078955	4.403749	1.06E-05	5.12E-05	0	0	3.478657	29.01765	47.06043	11.05477
2524	LOC10322;	37.61039	4.750731	0.669945	7.091229	1.33E-12	1.40E-11	0	2.189647	6.261582	78.12444	89.84265	49.24399
11565	LOC11962;	2.977846	4.747597	1.89748	2.502054	0.012348	0.028382	0	0.547412	0	11.16063	2.139111	4.019918
6490	ATP10B	8.325724	4.746111	1.20316	3.944703	7.99E-05	0.000327	0	1.094824	0.695731	17.85702	19.252	11.05477
11248	LOC11961{	2.950035	4.745625	1.845995	2.570767	0.010147	0.023984	0	0	0.695731	6.696381	4.278221	6.029877
6638	TEX11	8.331746	4.741117	1.220937	3.883178	0.000103	0.000413	0	1.094824	0.695731	15.62489	23.53022	9.044815
11324	LIX1	2.961456	4.740194	1.857445	2.551997	0.010711	0.025145	0.542086	0	0	8.928508	4.278221	4.019918
11304	TTLL2	2.987063	4.740063	1.853829	2.556904	0.010561	0.024833	0	0	0.695731	8.928508	4.278221	4.019918
11305	LOC11962(	2.987063	4.740063	1.853829	2.556904	0.010561	0.024833	0	0	0.695731	8.928508	4.278221	4.019918
11306	LOC10323(	2.987063	4.740063	1.853829	2.556904	0.010561	0.024833	0	0	0.695731	8.928508	4.278221	4.019918
11269	LOC10321!	2.934532	4.739816	1.847242	2.565889	0.010291	0.024278	0	0	0.695731	4.464254	6.417332	6.029877
16745	LOC10324{	1.488085	4.738446	3.08885	1.534048	0.125018	0.198459	0	0	0	8.928508	0	0
16746	DAOA	1.488085	4.738446	3.08885	1.534048	0.125018	0.198459	0	0	0	8.928508	0	0
16747	LOC11962(	1.488085	4.738446	3.08885	1.534048	0.125018	0.198459	0	0	0	8.928508	0	0
8582	WFDC9	5.592888	4.737851	1.465065	3.233884	0.001221	0.003783	0	0.547412	0.695731	17.85702	6.417332	8.039836
11600	MARCOL	2.999371	4.737721	1.898977	2.49488	0.0126	0.028877	0	0.547412	0	11.16063	4.278221	2.009959
11578	LOC10321‹	3.024091	4.737467	1.895382	2.499479	0.012438	0.028559	0	0	0.695731	11.16063	4.278221	2.009959
11261	ETDB	2.97156	4.73379	1.843805	2.567403	0.010246	0.024187	0	0	0.695731	6.696381	6.417332	4.019918
11262	LOC11962:	2.97156	4.73379	1.843805	2.567403	0.010246	0.024187	0	0	0.695731	6.696381	6.417332	4.019918
9625	LOC11962;	5.961188	4.732852	1.602288	2.953809	0.003139	0.00867	0	0	1.391463	6.696381	25.66933	2.009959
6806	PPEF2	8.598019	4.732796	1.240693	3.814638	0.000136	0.000533	0	0.547412	1.391463	20.08914	23.53022	6.029877
7585	ITPRID1	8.901743	4.73035	1.33976	3.530743	0.000414	0.001452	0	0	2.087194	8.928508	36.36488	6.029877
11495	LOC11962(	3.008588	4.730212	1.879625	2.516572	0.01185	0.0274	0	0	0.695731	8.928508	6.417332	2.009959
11496	LOC10322!	3.008588	4.730212	1.879625	2.516572	0.01185	0.0274	0	0	0.695731	8.928508	6.417332	2.009959
11497	LOC11962(	3.008588	4.730212	1.879625	2.516572	0.01185	0.0274	0	0	0.695731	8.928508	6.417332	2.009959
11498	LOC10321{	3.008588	4.730212	1.879625	2.516572	0.01185	0.0274	0	0	0.695731	8.928508	6.417332	2.009959
6818	KCNV2	8.762687	4.729185	1.241216	3.810122	0.000139	0.000542	0	0	2.087194	22.32127	17.11289	11.05477
6744	TRARG1	8.710157	4.728815	1.231447	3.840046	0.000123	0.000485	0	0	2.087194	17.85702	19.252	13.06473
8537	SLC6A5	5.59891	4.728605	1.457485	3.244361	0.001177	0.003666	0	0.547412	0.695731	15.62489	10.69555	6.029877
5303	LOC10322!	11.24193	4.728305	1.047105	4.515599	6.31E-06	3.17E-05	0	0.547412	2.087194	22.32127	21.39111	21.10457
11352	LOC10321{	2.956058	4.728258	1.85623	2.547237	0.010858	0.025428	0	0	0.695731	4.464254	8.556443	4.019918
6049	ASB4	11.51359	4.72651	1.140471	4.144349	3.41E-05	0.00015	0	0.547412	2.087194	15.62489	42.78221	8.039836
12441	LOC11962:	2.878807	4.725799	2.043427	2.312683	0.02074	0.044319	0	0.547412	0	0	10.69555	6.029877
12833	KCNA7	2.973501	4.725176	2.11582	2.233259	0.025532	0.052892	0.542086	0	0	4.464254	12.83466	0
12830	ARG1	2.974388	4.725167	2.115665	2.233419	0.025521	0.052883	0	0.547412	0	4.464254	12.83466	0
12804	RIPK3	2.999108	4.724916	2.11136	2.237855	0.025231	0.052386	0	0	0.695731	4.464254	12.83466	0
10235	TAC1	5.663486	4.724866	1.683264	2.806967	0.005001	0.01299	0	0.547412	0.695731	15.62489	17.11289	0
5271	LOC10321!	14.73336	4.724318	1.041276	4.537046	5.70E-06	2.88E-05	0	0	3.478657	33.4819	36.36488	15.07469
11513	LOC11961!	2.993086	4.724165	1.880812	2.511768	0.012013	0.027737	0	0	0.695731	6.696381	8.556443	2.009959

11514	LOC10324	2.993086	4.724165	1.880812	2.511768	0.012013	0.027737	0	0	0.695731	6.696381	8.556443	2.009959
8743	LOC11962	5.478346	4.723952	1.480822	3.190089	0.001422	0.004325	0	0.547412	0.695731	4.464254	17.11289	10.04979
11626	LOC11962	2.940555	4.72339	1.899071	2.487211	0.012875	0.029433	0	0	0.695731	2.232127	10.69555	4.019918
11627	LOC10324	2.940555	4.72339	1.899071	2.487211	0.012875	0.029433	0	0	0.695731	2.232127	10.69555	4.019918
11628	LOC11961	2.940555	4.72339	1.899071	2.487211	0.012875	0.029433	0	0	0.695731	2.232127	10.69555	4.019918
11629	IL20	2.940555	4.72339	1.899071	2.487211	0.012875	0.029433	0	0	0.695731	2.232127	10.69555	4.019918
13149	LOC10324	2.958886	4.721073	2.164472	2.181166	0.029171	0.058974	0	0.547412	0	2.232127	14.97377	0
13150	LOC10324	2.958886	4.721073	2.164472	2.181166	0.029171	0.058974	0	0.547412	0	2.232127	14.97377	0
13113	LOC10322	2.983606	4.720851	2.160114	2.185464	0.028855	0.0585	0	0	0.695731	2.232127	14.97377	0
9113	TCEAL6	5.85916	4.719543	1.532439	3.07976	0.002072	0.006044	0	0	1.391463	22.32127	6.417332	5.024897
11653	LOC11962	2.952863	4.719423	1.902539	2.480592	0.013116	0.029924	0	0.547412	0	4.464254	10.69555	2.009959
11639	TCERG1L	2.977583	4.719202	1.89902	2.485072	0.012953	0.02958	0	0	0.695731	4.464254	10.69555	2.009959
11640	LOC11961	2.977583	4.719202	1.89902	2.485072	0.012953	0.02958	0	0	0.695731	4.464254	10.69555	2.009959
11641	LOC11961	2.977583	4.719202	1.89902	2.485072	0.012953	0.02958	0	0	0.695731	4.464254	10.69555	2.009959
15502	C1QTNF4	1.414029	4.717321	2.705305	1.74373	0.081206	0.139264	0	0	0	4.464254	0	4.019918
1762	FGF1	50.3743	4.716428	0.550567	8.566499	1.07E-17	1.61E-16	1.626259	1.642235	8.348776	95.98146	111.2338	83.4133
7127	SPATA22	8.774732	4.716076	1.278797	3.687901	0.000226	0.000843	0	0	2.087194	17.85702	25.66933	7.034856
12794	LOC11962	2.946577	4.715936	2.104635	2.240738	0.025043	0.052034	0	0	0.695731	0	14.97377	2.009959
6937	PIWIL3	8.247786	4.71502	1.253349	3.761936	0.000169	0.000646	0	1.094824	0.695731	24.5534	17.11289	6.029877
8725	LOC10321	5.723093	4.714607	1.475877	3.194444	0.001401	0.004268	0	0	1.391463	8.928508	14.97377	9.044815
6743	LOC10322	8.433092	4.714214	1.227565	3.840296	0.000123	0.000484	0.542086	0	1.391463	26.78552	12.83466	9.044815
15749	LOC10323	1.472582	4.714011	2.760103	1.707912	0.087653	0.147877	0	0	0	6.696381	2.139111	0
15750	LOC11962	1.472582	4.714011	2.760103	1.707912	0.087653	0.147877	0	0	0	6.696381	2.139111	0
15751	LOC11962	1.472582	4.714011	2.760103	1.707912	0.087653	0.147877	0	0	0	6.696381	2.139111	0
15752	LOC11962	1.472582	4.714011	2.760103	1.707912	0.087653	0.147877	0	0	0	6.696381	2.139111	0
15754	LOC10324	1.472582	4.714011	2.760103	1.707912	0.087653	0.147877	0	0	0	6.696381	2.139111	0
15755	LOC10324	1.472582	4.714011	2.760103	1.707912	0.087653	0.147877	0	0	0	6.696381	2.139111	0
15756	BPIFA3	1.472582	4.714011	2.760103	1.707912	0.087653	0.147877	0	0	0	6.696381	2.139111	0
15757	LOC11962	1.472582	4.714011	2.760103	1.707912	0.087653	0.147877	0	0	0	6.696381	2.139111	0
15758	LOC10323	1.472582	4.714011	2.760103	1.707912	0.087653	0.147877	0	0	0	6.696381	2.139111	0
2944	SLC22A2	24.90296	4.712674	0.725721	6.493782	8.37E-11	7.56E-10	1.626259	0.547412	3.478657	60.26743	51.33866	32.15934
6630	LAX1	8.105696	4.712115	1.211941	3.888071	0.000101	0.000405	0	1.094824	0.695731	13.39276	21.39111	12.05975
15773	LOC10322	1.377001	4.711509	2.761484	1.706151	0.08798	0.148288	0	0	0	2.232127	0	6.029877
4179	FAM83A	19.83816	4.710502	0.898515	5.242539	1.58E-07	1.01E-06	0.542086	0.547412	3.478657	26.78552	70.59065	17.08465
8895	TRAF3IP3	5.797149	4.709597	1.497059	3.1459	0.001656	0.004949	0	0	1.391463	13.39276	14.97377	5.024897
14609	LOC10324	1.398526	4.709321	2.472431	1.904733	0.056815	0.103356	0	0	0	2.232127	2.139111	4.019918
14610	LOC10323	1.398526	4.709321	2.472431	1.904733	0.056815	0.103356	0	0	0	2.232127	2.139111	4.019918
14613	LOC11961	1.398526	4.709321	2.472431	1.904733	0.056815	0.103356	0	0	0	2.232127	2.139111	4.019918
14614	RNASE9	1.398526	4.709321	2.472431	1.904733	0.056815	0.103356	0	0	0	2.232127	2.139111	4.019918
6986	LOC10324	8.185775	4.706261	1.258397	3.739884	0.000184	0.000701	0	1.094824	0.695731	15.62489	25.66933	6.029877
8951	SLAMF6	5.781647	4.705868	1.502633	3.131748	0.001738	0.005161	0	0	1.391463	11.16063	17.11289	5.024897
4946	LOC11962	14.20012	4.704808	0.997493	4.716634	2.40E-06	1.29E-05	0	0.547412	2.782925	35.71403	32.08666	14.06971
5186	IGF1	20.38993	4.704571	1.025508	4.587551	4.48E-06	2.30E-05	0	0.547412	4.174388	35.71403	74.86887	7.034856
14516	SPRR4	1.435554	4.704104	2.450404	1.919726	0.054893	0.100461	0	0	0	4.464254	2.139111	2.009959
14517	LOC11962	1.435554	4.704104	2.450404	1.919726	0.054893	0.100461	0	0	0	4.464254	2.139111	2.009959
14519	AADAC	1.435554	4.704104	2.450404	1.919726	0.054893	0.100461	0	0	0	4.464254	2.139111	2.009959
14520	LOC10322	1.435554	4.704104	2.450404	1.919726	0.054893	0.100461	0	0	0	4.464254	2.139111	2.009959
14521	HBD	1.435554	4.704104	2.450404	1.919726	0.054893	0.100461	0	0	0	4.464254	2.139111	2.009959
14525	LOC11962	1.435554	4.704104	2.450404	1.919726	0.054893	0.100461	0	0	0	4.464254	2.139111	2.009959
14526	LOC11962	1.435554	4.704104	2.450404	1.919726	0.054893	0.100461	0	0	0	4.464254	2.139111	2.009959
14527	LOC10323	1.435554	4.704104	2.450404	1.919726	0.054893	0.100461	0	0	0	4.464254	2.139111	2.009959
14528	LOC11962	1.435554	4.704104	2.450404	1.919726	0.054893	0.100461	0	0	0	4.464254	2.139111	2.009959
6915	NDST3	8.153882	4.702584	1.24768	3.769062	0.000164	0.00063	0.542086	0.547412	0.695731	11.16063	29.94755	6.029877
6695	CUNH16or	8.356466	4.701836	1.218571	3.858483	0.000114	0.000453	0	0.547412	1.391463	15.62489	23.53022	9.044815
9783	LCN6	5.871205	4.701715	1.616948	2.907771	0.00364	0.009892	0	0	1.391463	17.85702	14.97377	1.004979
9033	SAMD7	5.766144	4.701551	1.514344	3.104678	0.001905	0.005606	0	0	1.391463	8.928508	19.252	5.024897
6824	LGALS12	8.393494	4.700404	1.233989	3.809113	0.000139	0.000543	0	0.547412	1.391463	17.85702	23.53022	7.034856
15701	LOC10323	1.457079	4.699888	2.739581	1.71555	0.086244	0.145899	0	0	0	4.464254	4.278221	0
15702	LOC11962	1.457079	4.699888	2.739581	1.71555	0.086244	0.145899	0	0	0	4.464254	4.278221	0
15703	LOC10323	1.457079	4.699888	2.739581	1.71555	0.086244	0.145899	0	0	0	4.464254	4.278221	0
15704	LOC10323	1.457079	4.699888	2.739581	1.71555	0.086244	0.145899	0	0	0	4.464254	4.278221	0
15705	LOC10324	1.457079	4.699888	2.739581	1.71555	0.086244	0.145899	0	0	0	4.464254	4.278221	0
15706	LOC11962	1.457079	4.699888	2.739581	1.71555	0.086244	0.145899	0	0	0	4.464254	4.278221	0
15707	PATE4	1.457079	4.699888	2.739581	1.71555	0.086244	0.145899	0	0	0	4.464254	4.278221	0
15708	LOC11962	1.457079	4.699888	2.739581	1.71555	0.086244	0.145899	0	0	0	4.464254	4.278221	0
15709	LOC11962	1.457079	4.699888	2.739581	1.71555	0.086244	0.145899	0	0	0	4.464254	4.278221	0
15710	LOC11961	1.457079	4.699888	2.739581	1.71555	0.086244	0.145899	0	0	0	4.464254	4.278221	0
15711	LOC11961	1.457079	4.699888	2.739581	1.71555	0.086244	0.145899	0	0	0	4.464254	4.278221	0
15712	TECRL	1.457079	4.699888	2.739581	1.71555	0.086244	0.145899	0	0	0	4.464254	4.278221	0
15713	TAS2R50	1.457079	4.699888	2.739581	1.71555	0.086244	0.145899	0	0	0	4.464254	4.278221	0
15714	OMG	1.457079	4.699888	2.739581	1.71555	0.086244	0.145899	0	0	0	4.464254	4.278221	0
15715	GPR142	1.457079	4.699888	2.739581	1.71555	0.086244	0.145899	0	0	0	4.464254	4.278221	0
206	LRCOL1	257.113	4.699883	0.234938	20.00477	5.00E-89	6.46E-87	22.22554	13.6853	21.56767	506.6928	477.0217	501.4848
7106	SMYD1	7.70027	4.697717	1.271569	3.694425	0.00022	0.000825	1.626259	0	0	13.39276	17.11289	14.06971

5639	GPR12	11.17603	4.693031	1.077544	4.355305	1.33E-05	6.26E-05	0	0.547412	2.087194	24.5534	27.80844	12.05975
14580	LOC10321(	1.420051	4.691961	2.457211	1.909466	0.056202	0.102429	0	0	0	2.232127	4.278221	2.009959
14582	LOC10323	1.420051	4.691961	2.457211	1.909466	0.056202	0.102429	0	0	0	2.232127	4.278221	2.009959
14583	LOC10323(	1.420051	4.691961	2.457211	1.909466	0.056202	0.102429	0	0	0	2.232127	4.278221	2.009959
14584	LOC10323	1.420051	4.691961	2.457211	1.909466	0.056202	0.102429	0	0	0	2.232127	4.278221	2.009959
14585	LOC11962	1.420051	4.691961	2.457211	1.909466	0.056202	0.102429	0	0	0	2.232127	4.278221	2.009959
14586	TAS2R39	1.420051	4.691961	2.457211	1.909466	0.056202	0.102429	0	0	0	2.232127	4.278221	2.009959
14587	LOC11962	1.420051	4.691961	2.457211	1.909466	0.056202	0.102429	0	0	0	2.232127	4.278221	2.009959
15662	LOC10324	1.383023	4.691705	2.723399	1.722739	0.084936	0.144153	0	0	0	0	4.278221	4.019918
15663	LOC10323	1.383023	4.691705	2.723399	1.722739	0.084936	0.144153	0	0	0	0	4.278221	4.019918
15664	LOC10323	1.383023	4.691705	2.723399	1.722739	0.084936	0.144153	0	0	0	0	4.278221	4.019918
3548	IKZF1	31.4114	4.688798	0.807595	5.805878	6.40E-09	4.80E-08	0	1.094824	6.261582	71.42806	85.56443	24.11951
9165	CYP1A2	5.202169	4.688402	1.53086	3.062594	0.002194	0.006366	0	1.094824	0	13.39276	10.69555	6.029877
15863	TRIM60	1.441576	4.687919	2.776628	1.68835	0.091344	0.152972	0	0	0	2.232127	6.417332	0
15864	SCGB1D2	1.441576	4.687919	2.776628	1.68835	0.091344	0.152972	0	0	0	2.232127	6.417332	0
15865	CRYGC	1.441576	4.687919	2.776628	1.68835	0.091344	0.152972	0	0	0	2.232127	6.417332	0
15866	LOC11962	1.441576	4.687919	2.776628	1.68835	0.091344	0.152972	0	0	0	2.232127	6.417332	0
15868	LOC11962	1.441576	4.687919	2.776628	1.68835	0.091344	0.152972	0	0	0	2.232127	6.417332	0
15869	LOC10321	1.441576	4.687919	2.776628	1.68835	0.091344	0.152972	0	0	0	2.232127	6.417332	0
15870	LOC11961	1.441576	4.687919	2.776628	1.68835	0.091344	0.152972	0	0	0	2.232127	6.417332	0
15871	LOC11962(	1.441576	4.687919	2.776628	1.68835	0.091344	0.152972	0	0	0	2.232127	6.417332	0
15872	LOC11962(	1.441576	4.687919	2.776628	1.68835	0.091344	0.152972	0	0	0	2.232127	6.417332	0
15873	LOC11962	1.441576	4.687919	2.776628	1.68835	0.091344	0.152972	0	0	0	2.232127	6.417332	0
15874	DEFB132	1.441576	4.687919	2.776628	1.68835	0.091344	0.152972	0	0	0	2.232127	6.417332	0
15875	LOC10324	1.441576	4.687919	2.776628	1.68835	0.091344	0.152972	0	0	0	2.232127	6.417332	0
9373	LOC11962(	5.259835	4.686959	1.555126	3.013878	0.002579	0.007316	0.542086	0.547412	0	15.62489	12.83466	2.009959
10810	TULP1	21.19703	4.685611	1.755465	2.669157	0.007604	0.018701	0	0	4.87012	40.17829	62.03421	20.09959
16969	LOC11962	1.426074	4.684159	3.130176	1.496452	0.134536	0.21071	0	0	0	0	8.556443	0
16970	LOC11962	1.426074	4.684159	3.130176	1.496452	0.134536	0.21071	0	0	0	0	8.556443	0
16971	LOC10322(	1.426074	4.684159	3.130176	1.496452	0.134536	0.21071	0	0	0	0	8.556443	0
16972	LOC10322	1.426074	4.684159	3.130176	1.496452	0.134536	0.21071	0	0	0	0	8.556443	0
16973	LOC11961	1.426074	4.684159	3.130176	1.496452	0.134536	0.21071	0	0	0	0	8.556443	0
16974	LOC11961	1.426074	4.684159	3.130176	1.496452	0.134536	0.21071	0	0	0	0	8.556443	0
16975	LOC11962	1.426074	4.684159	3.130176	1.496452	0.134536	0.21071	0	0	0	0	8.556443	0
15729	LOC11961	1.404548	4.682429	2.73429	1.712484	0.086807	0.146665	0	0	0	0	6.417332	2.009959
15730	CXCL9	1.404548	4.682429	2.73429	1.712484	0.086807	0.146665	0	0	0	0	6.417332	2.009959
15731	ZFP42	1.404548	4.682429	2.73429	1.712484	0.086807	0.146665	0	0	0	0	6.417332	2.009959
15732	LOC10323	1.404548	4.682429	2.73429	1.712484	0.086807	0.146665	0	0	0	0	6.417332	2.009959
15733	LOC10321	1.404548	4.682429	2.73429	1.712484	0.086807	0.146665	0	0	0	0	6.417332	2.009959
15734	GLT6D1	1.404548	4.682429	2.73429	1.712484	0.086807	0.146665	0	0	0	0	6.417332	2.009959
15735	LOC11962	1.404548	4.682429	2.73429	1.712484	0.086807	0.146665	0	0	0	0	6.417332	2.009959
10173	TRNAP-UG	5.793692	4.681755	1.659587	2.821036	0.004787	0.012509	0	0	1.391463	6.696381	25.66933	1.004979
11042	CYP11B1	5.112186	4.677684	1.786368	2.618544	0.008831	0.021261	0	1.094824	0	24.5534	0	5.024897
5699	LOC10322	10.86796	4.676022	1.08056	4.327405	1.51E-05	7.04E-05	0.542086	0.547412	1.391463	29.01765	25.66933	8.039836
5415	KCNJ10	10.7784	4.675311	1.049623	4.454277	8.42E-06	4.13E-05	0.542086	0.547412	1.391463	22.32127	27.80844	12.05975
9048	LOC10323	5.452939	4.673051	1.50877	3.097258	0.001953	0.005739	0	0.547412	0.695731	15.62489	12.83466	3.014938
8214	CRYBA2	8.650286	4.671029	1.397739	3.341846	0.000832	0.002694	0	0	2.087194	17.85702	29.94755	2.009959
5716	PLCL1	10.74829	4.670977	1.082019	4.316909	1.58E-05	7.36E-05	0	1.094824	1.391463	17.85702	32.08666	12.05975
13280	LOC10324(	2.8876	4.670903	2.170955	2.151542	0.031433	0.062926	0	0	0.695731	15.62489	0	1.004979
8872	CLEC7A	5.5711	4.6682	1.482095	3.149731	0.001634	0.004897	0	0	1.391463	11.16063	12.83466	8.039836
8982	LOC10322	5.608128	4.665168	1.494305	3.121965	0.001796	0.005317	0	0	1.391463	13.39276	12.83466	6.029877
9000	APOL1	5.518569	4.664433	1.49694	3.115979	0.001833	0.005416	0	0	1.391463	6.696381	14.97377	10.04979
12681	NPVF	2.776516	4.664108	2.060487	2.263594	0.023599	0.049474	0	0	0.695731	8.928508	0	7.034856
7141	LOC11962	8.427694	4.661789	1.26558	3.68352	0.00023	0.000856	0	0	2.087194	22.32127	17.11289	9.044815
9164	LOC10322	5.645156	4.661594	1.522138	3.062531	0.002195	0.006366	0	0	1.391463	15.62489	12.83466	4.019918
9166	LOC11961	5.645156	4.661594	1.522138	3.062531	0.002195	0.006366	0	0	1.391463	15.62489	12.83466	4.019918
9660	LOC11962(	5.697686	4.660892	1.583225	2.943923	0.003241	0.008919	0	0	1.391463	20.08914	10.69555	2.009959
5316	RARRES1	13.58293	4.659163	1.032904	4.51074	6.46E-06	3.23E-05	0	1.094824	2.087194	46.87467	21.39111	10.04979
9082	LOC11962	5.577122	4.657188	1.507741	3.088852	0.002009	0.005882	0	0	1.391463	8.928508	17.11289	6.029877
11873	LOC10322	2.773321	4.656357	1.914265	2.432452	0.014997	0.03358	0	0.547412	0	8.928508	2.139111	5.024897
11847	LOC10322	2.798041	4.656318	1.910316	2.437459	0.014791	0.033191	0	0	0.695731	8.928508	2.139111	5.024897
4997	CUNH10or	13.03327	4.652768	0.992164	4.689514	2.74E-06	1.46E-05	1.084173	0.547412	1.391463	11.16063	44.92132	19.09461
5694	QRFPR	10.78249	4.645609	1.073087	4.329203	1.50E-05	6.99E-05	0	0.547412	2.087194	22.32127	25.66933	14.06971
4130	LOC10322(	21.94516	4.645484	0.881287	5.271247	1.35E-07	8.72E-07	0	1.094824	4.174388	53.57105	57.75599	15.07469
12251	DRD3	2.831875	4.644687	1.971639	2.355749	0.018485	0.040114	0	0.547412	0	11.16063	4.278221	1.004979
11808	LOC11962	2.794847	4.644248	1.897442	2.447636	0.01438	0.032375	0	0.547412	0	8.928508	4.278221	3.014938
11783	LOC10323	2.819567	4.644056	1.893622	2.452473	0.014188	0.031996	0	0	0.695731	8.928508	4.278221	3.014938
7919	LOC11962	7.88781	4.643346	1.352536	3.433066	0.000597	0.002004	0	1.094824	0.695731	17.85702	25.66933	2.009959
7749	LOC11962	8.408734	4.641417	1.334433	3.478195	0.000505	0.001732	0	0	2.087194	13.39276	29.94755	5.024897
8814	LOC10323	5.168336	4.638148	1.462564	3.171244	0.001518	0.004578	0	0.547412	0.695731	11.16063	8.556443	10.04979
11938	LOC10324	2.726813	4.637388	1.918848	2.416757	0.015659	0.034872	0	0.547412	0	2.232127	8.556443	5.024897
11912	LOC11962	2.751533	4.637384	1.914797	2.421868	0.015441	0.034458	0	0	0.695731	2.232127	8.556443	5.024897
11913	SIRPD	2.751533	4.637384	1.914797	2.421868	0.015441	0.034458	0	0	0.695731	2.232127	8.556443	5.024897
11735	LOC10322	2.804064	4.637193	1.883819	2.461591	0.013832	0.031333	0	0	0.695731	6.696381	6.417332	3.014938

11736	LOC11962:	2.804064	4.637193	1.883819	2.461591	0.013832	0.031333	0	0	0.695731	6.696381	6.417332	3.014938
6984	DSC1	8.052478	4.637142	1.239804	3.740222	0.000184	0.0007	0	0.547412	1.391463	20.08914	19.252	7.034856
6985	ACSM4	8.052478	4.637142	1.239804	3.740222	0.000184	0.0007	0	0.547412	1.391463	20.08914	19.252	7.034856
5934	AQP6	10.81003	4.637125	1.106026	4.1926	2.76E-05	0.000124	0	0.547412	2.087194	20.08914	32.08666	10.04979
5935	LOC10323(	10.81003	4.637125	1.106026	4.1926	2.76E-05	0.000124	0	0.547412	2.087194	20.08914	32.08666	10.04979
12183	LOC11962[illegible]	2.816372	4.63682	1.95651	2.369944	0.017791	0.038822	0	0.547412	0	8.928508	6.417332	1.004979
7229	HCK	8.089506	4.636113	1.267326	3.658185	0.000254	0.000934	0	0.547412	1.391463	22.32127	19.252	5.024897
310	HR	181.9427	4.634363	0.268556	17.25661	9.98E-67	8.56E-65	11.38381	17.51718	13.2189	381.6937	325.1448	342.698
11829	LOC10321[illegible]	2.788561	4.63139	1.896388	2.442216	0.014597	0.032804	0	0	0.695731	4.464254	8.556443	3.014938
11830	LOC10323[illegible]	2.788561	4.63139	1.896388	2.442216	0.014597	0.032804	0	0	0.695731	4.464254	8.556443	3.014938
12214	MBOAT4	2.799982	4.63025	1.958114	2.364648	0.018047	0.039282	0.542086	0	0	6.696381	8.556443	1.004979
12213	LOC10323[illegible]	2.800869	4.630239	1.957974	2.364812	0.018039	0.039267	0	0.547412	0	6.696381	8.556443	1.004979
12186	LOC11962[illegible]	2.825589	4.629946	1.954107	2.369342	0.01782	0.038872	0	0	0.695731	6.696381	8.556443	1.004979
12187	LOC11962(	2.825589	4.629946	1.954107	2.369342	0.01782	0.038872	0	0	0.695731	6.696381	8.556443	1.004979
10121	ACY3	4.935634	4.629766	1.634551	2.83244	0.004619	0.012134	0	1.094824	0	2.232127	19.252	7.034856
24	ITGAX	3318.726	4.627588	0.129093	35.84701	2.05E-281	2.27E-278	244.481	240.8612	290.8157	6158.438	5683.617	7294.141
3744	LOC11961[illegible]	18.11739	4.62673	0.826287	5.599422	2.15E-08	1.53E-07	1.626259	0	2.782925	40.17829	29.94755	34.1693
13339	LOC11961[illegible]	305.901	4.626041	2.162105	2.139601	0.032387	0.064548	0	0.547412	70.9646	716.5128	802.1665	245.215
6068	CPA3	10.18405	4.625218	1.117841	4.137636	3.51E-05	0.000154	0	1.642235	0.695731	15.62489	32.08666	11.05477
11242	LOC11961[illegible]	5.626195	4.62491	1.797865	2.572446	0.010098	0.02388	0	0	1.391463	6.696381	25.66933	0
12326	LOC11962[illegible]	2.785366	4.624824	1.976097	2.340383	0.019264	0.041549	0	0.547412	0	4.464254	10.69555	1.004979
12298	LOC11962[illegible]	2.810086	4.62455	1.972188	2.344883	0.019033	0.041144	0	0	0.695731	4.464254	10.69555	1.004979
4903	CD93	19.51348	4.622406	0.975008	4.74089	2.13E-06	1.15E-05	0	0	4.87012	51.33892	42.78221	18.08963
12559	LOC11962[illegible]	2.794584	4.620659	2.016621	2.291288	0.021947	0.046453	0	0	0.695731	2.232127	12.83466	1.004979
12560	FOXL3	2.794584	4.620659	2.016621	2.291288	0.021947	0.046453	0	0	0.695731	2.232127	12.83466	1.004979
5878	CD180	10.66752	4.619277	1.093024	4.226145	2.38E-05	0.000108	0	0.547412	2.087194	26.78552	23.53022	11.05477
9117	LOC10322[illegible]	5.232912	4.61781	1.499831	3.078887	0.002078	0.006059	0	0.547412	0.695731	11.16063	14.97377	4.019918
2098	LOC10324(	37.74026	4.617399	0.592077	7.798647	6.26E-15	7.93E-14	4.878777	0.547412	3.478657	62.49956	87.70354	67.33362
9273	PGK2	5.456134	4.615461	1.518698	3.039091	0.002373	0.006803	0	0	1.391463	15.62489	10.69555	5.024897
3807	KCNK9	27.33085	4.613571	0.834032	5.531645	3.17E-08	2.22E-07	0	0.547412	6.261582	46.87467	79.14709	31.15436
9199	LOC10321[illegible]	5.351072	4.612403	1.509884	3.054806	0.002252	0.006508	0	0	1.391463	6.696381	14.97377	9.044815
9178	INHBC	5.3881	4.611345	1.506201	3.061574	0.002202	0.006378	0	0	1.391463	8.928508	14.97377	7.034856
9293	PDCD1LG2	5.425128	4.608564	1.519041	3.033865	0.002414	0.006906	0	0	1.391463	11.16063	14.97377	5.024897
9730	LOC10322(	5.223432	4.608371	1.577748	2.920854	0.003491	0.009538	0	0.547412	0.695731	6.696381	21.39111	2.009959
9588	LOC10322[illegible]	5.462156	4.604972	1.553604	2.964058	0.003036	0.008418	0	0	1.391463	13.39276	14.97377	3.014938
1660	LOC11962[illegible]	44.66446	4.604086	0.522151	8.817538	1.17E-18	1.87E-17	1.626259	5.474118	3.478657	95.98146	77.00798	84.41828
7046	ADARB2	7.832451	4.600531	1.238594	3.714318	0.000204	0.000769	0	0.547412	1.391463	15.62489	21.39111	8.039836
10212	KRT24	5.499184	4.600465	1.635041	2.813669	0.004898	0.012751	0	0	1.391463	15.62489	14.97377	1.004979
9594	LOC11961[illegible]	5.357095	4.598987	1.552185	2.962911	0.003047	0.008444	0	0	1.391463	4.464254	19.252	7.034856
5919	LOC10323[illegible]	13.66034	4.598278	1.094272	4.202135	2.64E-05	0.000119	0	0	3.478657	26.78552	40.6431	11.05477
8184	NOTO	7.604884	4.597062	1.371704	3.351351	0.000804	0.002612	0.542086	0.547412	0.695731	4.464254	36.36488	3.014938
113	LOC11962[illegible]	445.9497	4.587803	0.192629	23.8168	2.24E-125	5.26E-123	29.81475	39.96106	37.56949	848.2083	759.3843	960.7604
8265	CUNH10or	8.18226	4.587575	1.380115	3.324052	0.000887	0.002854	0	0	2.087194	29.01765	14.97377	3.014938
7652	TBX10	7.252974	4.587217	1.307053	3.509587	0.000449	0.001559	0	1.642235	0	17.85702	14.97377	9.044815
4628	GIMAP8	18.38155	4.585994	0.930077	4.930767	8.19E-07	4.71E-06	0	1.094824	3.478657	40.17829	53.47777	12.05975
6208	CD244	10.49054	4.585377	1.126693	4.069768	4.71E-05	0.000202	0	0.547412	2.087194	22.32127	29.94755	8.039836
10192	LOC11961[illegible]	5.363117	4.584145	1.627182	2.817229	0.004844	0.012635	0	0	1.391463	2.232127	23.53022	5.024897
9679	LOC11962[illegible]	4.81287	4.583458	1.560534	2.937109	0.003313	0.009099	1.084173	0	0	8.928508	12.83466	6.029877
4862	FFAR2	15.77358	4.583252	0.961558	4.766483	1.87E-06	1.03E-05	0	0.547412	3.478657	22.32127	49.19955	19.09461
9148	ADRA1D	5.05337	4.582631	1.494094	3.067164	0.002161	0.00628	0	0.547412	0.695731	15.62489	6.417332	7.034856
9481	KCNK12	4.881791	4.581924	1.532666	2.989512	0.002794	0.007835	0.542086	0.547412	0	15.62489	8.556443	4.019918
3062	LOC10323[illegible]	36.34829	4.580192	0.724844	6.318864	2.63E-10	2.29E-09	0	2.737059	6.261582	84.82083	94.12087	30.14938
989	LGI3	69.83164	4.578159	0.414363	11.04867	2.22E-28	5.98E-27	8.131296	4.379295	4.174388	127.2312	128.3466	146.727
9218	LOC11962(	5.074895	4.575837	1.500158	3.050237	0.002287	0.006595	0	0.547412	0.695731	15.62489	8.556443	5.024897
7388	PIWIL1	9.882626	4.573002	1.269277	3.602839	0.000315	0.001132	0	1.642235	0.695731	11.16063	42.78221	3.014938
8948	LOC10322[illegible]	8.141774	4.57092	1.459152	3.132587	0.001733	0.005148	0	0	2.087194	20.08914	25.66933	1.004979
6554	CXCL13	10.63023	4.56852	1.166313	3.917063	8.96E-05	0.000364	0	0	2.782925	17.85702	32.08666	11.05477
5906	WDR72	10.20505	4.568223	1.085225	4.20947	2.56E-05	0.000115	0.542086	0	2.087194	17.85702	25.66933	15.07469
366	GASK1B	159.9574	4.566475	0.281905	16.19862	5.16E-59	3.75E-57	15.17842	14.78012	8.348776	299.105	303.7537	318.5785
569	FAM107A	178.5108	4.566013	0.3283	13.90805	5.66E-44	2.64E-42	8.673382	25.18094	9.044508	337.0512	344.3968	346.7179
5188	MAS1	12.85712	4.564791	0.995258	4.58654	4.51E-06	2.31E-05	0.542086	0	2.782925	26.78552	29.94755	17.08465
7814	LOC11962[illegible]	7.754513	4.564627	1.318504	3.461976	0.000536	0.001824	0	0.547412	1.391463	22.32127	19.252	3.014938
7694	UROC1	7.42623	4.563891	1.305565	3.495721	0.000473	0.001634	0	1.094824	0.695731	8.928508	27.80844	6.029877
10088	LOC11962[illegible]	5.08694	4.56171	1.605891	2.84061	0.004503	0.011864	0	0.547412	0.695731	11.16063	17.11289	1.004979
7779	NAA11	7.934684	4.561396	1.314299	3.470592	0.000519	0.001775	0	0	2.087194	26.78552	10.69555	8.039836
9402	CBLN1	5.236106	4.561069	1.51701	3.006617	0.002642	0.007469	0	0	1.391463	11.16063	12.83466	6.029877
9403	LYPD4	5.236106	4.561069	1.51701	3.006617	0.002642	0.007469	0	0	1.391463	11.16063	12.83466	6.029877
9630	LOC11962[illegible]	5.288637	4.560747	1.544418	2.953051	0.003146	0.008686	0	0	1.391463	15.62489	10.69555	4.019918
6359	SCN10A	10.01027	4.560582	1.139865	4.000984	6.31E-05	0.000264	0	1.094824	1.391463	11.16063	36.36488	10.04979
12274	IGFL3	2.593517	4.560019	1.940447	2.349984	0.018774	0.040664	0	0	0.695731	6.696381	2.139111	6.029877
9580	CCDC105	5.049912	4.559875	1.537648	2.965487	0.003022	0.008386	0	0.547412	0.695731	8.928508	17.11289	3.014938
9690	HTR3E	5.033522	4.559494	1.554057	2.933929	0.003347	0.009183	0.542086	0	0.695731	6.696381	19.252	3.014938
203	DOK5	250.4927	4.557519	0.226442	20.12667	4.31E-90	5.66E-88	21.14137	22.44388	17.39328	466.5145	459.9088	515.5545
9692	P2RY14	5.03441	4.556779	1.553707	2.932843	0.003359	0.009213	0	0.547412	0.695731	6.696381	19.252	3.014938

9484	FAM217A	5.220604	4.556722	1.5249	2.98821	0.002806	0.007866	0	0	1.391463	8.928508	14.97377	6.029877
7335	CDA	7.139062	4.556571	1.25872	3.620004	0.000295	0.001068	0	1.094824	0.695731	13.39276	8.556443	19.09461
7703	KBTBD12	7.54439	4.55534	1.30429	3.492583	0.000478	0.001651	0	0.547412	1.391463	4.464254	27.80844	11.05477
13510	LOC119624	2.689098	4.553963	2.163072	2.105323	0.035263	0.069371	0	0	0.695731	11.16063	4.278221	0
4920	DSCAM	15.53763	4.553753	0.96204	4.733433	2.21E-06	1.19E-05	0	0.547412	3.478657	33.4819	40.6431	15.07469
6749	HNF1A	10.31268	4.552323	1.18613	3.837965	0.000124	0.000489	0.542086	0	2.087194	17.85702	36.36488	5.024897
9633	LOC10323	5.205101	4.551358	1.541637	2.952289	0.003154	0.008704	0	0	1.391463	6.696381	17.11289	6.029877
9634	CD300LF	5.205101	4.551358	1.541637	2.952289	0.003154	0.008704	0	0	1.391463	6.696381	17.11289	6.029877
12553	NEU2	2.667573	4.551174	1.985261	2.292482	0.021878	0.04633	0	0	0.695731	11.16063	2.139111	2.009959
12554	LOC10322(	2.667573	4.551174	1.985261	2.292482	0.021878	0.04633	0	0	0.695731	11.16063	2.139111	2.009959
5625	CCDC190	15.94039	4.549544	1.043093	4.36159	1.29E-05	6.10E-05	0	0	4.174388	33.4819	44.92132	13.06473
12139	LOC11961	2.615042	4.5469	1.911553	2.378643	0.017377	0.038052	0	0	0.695731	6.696381	4.278221	4.019918
12140	HES3	2.615042	4.5469	1.911553	2.378643	0.017377	0.038052	0	0	0.695731	6.696381	4.278221	4.019918
11979	LOC11962	25.03461	4.546617	1.886707	2.409817	0.015961	0.035421	0	0	6.261582	49.10679	72.72976	22.10955
13481	LOC11961	2.648875	4.545837	2.153493	2.110913	0.03478	0.068587	0	0.547412	0	8.928508	6.417332	0
13457	LRIT2	2.673595	4.545504	2.14835	2.115811	0.034361	0.067872	0	0	0.695731	8.928508	6.417332	0
13458	LOC11961	2.673595	4.545504	2.14835	2.115811	0.034361	0.067872	0	0	0.695731	8.928508	6.417332	0
13459	LOC10323	2.673595	4.545504	2.14835	2.115811	0.034361	0.067872	0	0	0.695731	8.928508	6.417332	0
12364	LINC02843	2.626462	4.542029	1.950113	2.32911	0.019853	0.042688	0.542086	0	0	8.928508	4.278221	2.009959
12362	LOC11962	2.62735	4.542019	1.949961	2.329287	0.019844	0.042673	0	0.547412	0	8.928508	4.278221	2.009959
12191	PTH2R	2.574819	4.540329	1.917527	2.367805	0.017894	0.039012	0	0.547412	0	4.464254	6.417332	4.019918
12192	LOC11962	2.574819	4.540329	1.917527	2.367805	0.017894	0.039012	0	0.547412	0	4.464254	6.417332	4.019918
12194	LOC10323	2.574819	4.540329	1.917527	2.367805	0.017894	0.039012	0	0.547412	0	4.464254	6.417332	4.019918
12164	ADAM29	2.599539	4.540248	1.913188	2.373132	0.017638	0.038547	0	0	0.695731	4.464254	6.417332	4.019918
12165	LOC10321	2.599539	4.540248	1.913188	2.373132	0.017638	0.038547	0	0	0.695731	4.464254	6.417332	4.019918
13511	LOC11962	2.633373	4.538454	2.155722	2.105306	0.035265	0.069371	0	0.547412	0	6.696381	8.556443	0
13512	LOC11962	2.633373	4.538454	2.155722	2.105306	0.035265	0.069371	0	0.547412	0	6.696381	8.556443	0
13514	LOC10323	2.633373	4.538454	2.155722	2.105306	0.035265	0.069371	0	0.547412	0	6.696381	8.556443	0
6637	TMEM125	9.947831	4.537022	1.168115	3.884053	0.000103	0.000411	0	1.094824	1.391463	20.08914	32.08666	5.024897
4482	GPR141	17.89473	4.535666	0.903215	5.021693	5.12E-07	3.04E-06	0.542086	0	4.174388	33.4819	47.06043	22.10955
12335	LOC11962(	2.611847	4.534494	1.940614	2.336629	0.019458	0.041928	0	0.547412	0	6.696381	6.417332	2.009959
12336	LOC11962	2.611847	4.534494	1.940614	2.336629	0.019458	0.041928	0	0.547412	0	6.696381	6.417332	2.009959
12321	CALHM6	2.636567	4.53423	1.936375	2.341608	0.019201	0.041423	0	0	0.695731	6.696381	6.417332	2.009959
12322	LIPJ	2.636567	4.53423	1.936375	2.341608	0.019201	0.041423	0	0	0.695731	6.696381	6.417332	2.009959
12323	LOC11962(	2.636567	4.53423	1.936375	2.341608	0.019201	0.041423	0	0	0.695731	6.696381	6.417332	2.009959
8198	LOC10324	7.293986	4.532586	1.354048	3.347432	0.000816	0.002645	1.084173	0	0.695731	11.16063	27.80844	3.014938
950	SMIM5	71.93788	4.532175	0.40459	11.20189	3.99E-29	1.12E-27	4.878777	4.926706	8.348776	131.6955	156.1551	125.6224
13625	LOC11962	2.61787	4.532049	2.174887	2.083808	0.037178	0.072541	0	0.547412	0	4.464254	10.69555	0
13602	LOC11962	2.64259	4.531741	2.16966	2.088687	0.036736	0.0718	0	0	0.695731	4.464254	10.69555	0
16453	LOC11962	1.28356	4.530288	2.870048	1.578471	0.114457	0.184796	0	0	0	6.696381	0	1.004979
16454	LOC10324	1.28356	4.530288	2.870048	1.578471	0.114457	0.184796	0	0	0	6.696381	0	1.004979
16455	LOC11962	1.28356	4.530288	2.870048	1.578471	0.114457	0.184796	0	0	0	6.696381	0	1.004979
16456	LOC11962	1.28356	4.530288	2.870048	1.578471	0.114457	0.184796	0	0	0	6.696381	0	1.004979
16457	LOC10321	1.28356	4.530288	2.870048	1.578471	0.114457	0.184796	0	0	0	6.696381	0	1.004979
16458	ACOD1	1.28356	4.530288	2.870048	1.578471	0.114457	0.184796	0	0	0	6.696381	0	1.004979
16459	LOC11962	1.28356	4.530288	2.870048	1.578471	0.114457	0.184796	0	0	0	6.696381	0	1.004979
16460	WFDC5	1.28356	4.530288	2.870048	1.578471	0.114457	0.184796	0	0	0	6.696381	0	1.004979
16462	NDN	1.28356	4.530288	2.870048	1.578471	0.114457	0.184796	0	0	0	6.696381	0	1.004979
16463	LOC11962	1.28356	4.530288	2.870048	1.578471	0.114457	0.184796	0	0	0	6.696381	0	1.004979
16464	SLCO1B1	1.28356	4.530288	2.870048	1.578471	0.114457	0.184796	0	0	0	6.696381	0	1.004979
16466	LOC10323	1.28356	4.530288	2.870048	1.578471	0.114457	0.184796	0	0	0	6.696381	0	1.004979
9971	THSD7B	4.660876	4.529919	1.578512	2.86974	0.004108	0.010953	1.084173	0	0	11.16063	10.69555	5.024897
12411	LOC10321	2.595457	4.528301	1.953432	2.318125	0.020443	0.043789	0.542086	0	0	4.464254	8.556443	2.009959
12410	TXLNB	2.596345	4.528292	1.953277	2.318305	0.020433	0.043772	0	0.547412	0	4.464254	8.556443	2.009959
13841	LOC11962(	2.627087	4.52676	2.217374	2.041496	0.041202	0.079138	0	0	0.695731	2.232127	12.83466	0
7640	LOC11962	7.257846	4.52612	1.288646	3.512305	0.000444	0.001546	0.542086	0.547412	0.695731	8.928508	27.80844	5.024897
16348	LOC11961	1.246532	4.525825	2.835755	1.595986	0.110492	0.17967	0	0	0	4.464254	0	3.014938
16349	LOC11962	1.246532	4.525825	2.835755	1.595986	0.110492	0.17967	0	0	0	4.464254	0	3.014938
12674	LOC10323(	2.580842	4.523515	1.996422	2.265811	0.023463	0.049216	0	0.547412	0	2.232127	10.69555	2.009959
12650	LOC10324	2.605562	4.523301	1.992029	2.2707	0.023165	0.04868	0	0	0.695731	2.232127	10.69555	2.009959
12651	LOC11962	2.605562	4.523301	1.992029	2.2707	0.023165	0.04868	0	0	0.695731	2.232127	10.69555	2.009959
7928	TMEM212	7.751685	4.523063	1.318366	3.43081	0.000602	0.002018	0	0	2.087194	24.5534	12.83466	7.034856
13590	LOC10322	2.590059	4.522681	2.162348	2.09156	0.036478	0.071359	0	0	0.695731	0	12.83466	2.009959
6312	GRM6	13.06185	4.522424	1.124681	4.021073	5.79E-05	0.000244	0	0	3.478657	40.17829	25.66933	9.044815
9611	DUSP26	5.062587	4.514739	1.52602	2.958505	0.003091	0.008551	0	0	1.391463	13.39276	8.556443	7.034856
15470	OOSP2	1.231029	4.513898	2.580142	1.749477	0.080209	0.137784	0	0	0	2.232127	2.139111	3.014938
15471	LOC10323	1.231029	4.513898	2.580142	1.749477	0.080209	0.137784	0	0	0	2.232127	2.139111	3.014938
15472	LOC11962	1.231029	4.513898	2.580142	1.749477	0.080209	0.137784	0	0	0	2.232127	2.139111	3.014938
15473	LOC11962	1.231029	4.513898	2.580142	1.749477	0.080209	0.137784	0	0	0	2.232127	2.139111	3.014938
15474	PPP1R3A	1.231029	4.513898	2.580142	1.749477	0.080209	0.137784	0	0	0	2.232127	2.139111	3.014938
15475	LOC10323(	1.231029	4.513898	2.580142	1.749477	0.080209	0.137784	0	0	0	2.232127	2.139111	3.014938
15476	LOC11962	1.231029	4.513898	2.580142	1.749477	0.080209	0.137784	0	0	0	2.232127	2.139111	3.014938
7275	ZNF610	7.118789	4.512067	1.239289	3.640853	0.000272	0.000993	0.542086	0.547412	0.695731	22.32127	8.556443	10.04979
9748	LOC11962	5.099615	4.510984	1.546092	2.91767	0.003527	0.009618	0	0	1.391463	15.62489	8.556443	5.024897

9903	LOC10322	4.913421	4.510736	1.563806	2.884461	0.003921	0.010526	0	0.547412	0.695731	13.39276	12.83466	2.009959
15524	LOC10323	1.268057	4.507539	2.593554	1.737977	0.082215	0.140667	0	0	0	4.464254	2.139111	1.004979
15525	LOC11962	1.268057	4.507539	2.593554	1.737977	0.082215	0.140667	0	0	0	4.464254	2.139111	1.004979
15526	LOC10324	1.268057	4.507539	2.593554	1.737977	0.082215	0.140667	0	0	0	4.464254	2.139111	1.004979
15527	LOC10324	1.268057	4.507539	2.593554	1.737977	0.082215	0.140667	0	0	0	4.464254	2.139111	1.004979
15528	LOC10324	1.268057	4.507539	2.593554	1.737977	0.082215	0.140667	0	0	0	4.464254	2.139111	1.004979
15529	LOC11962	1.268057	4.507539	2.593554	1.737977	0.082215	0.140667	0	0	0	4.464254	2.139111	1.004979
15530	LOC11962	1.268057	4.507539	2.593554	1.737977	0.082215	0.140667	0	0	0	4.464254	2.139111	1.004979
15531	LOC11961	1.268057	4.507539	2.593554	1.737977	0.082215	0.140667	0	0	0	4.464254	2.139111	1.004979
15532	LOC11961	1.268057	4.507539	2.593554	1.737977	0.082215	0.140667	0	0	0	4.464254	2.139111	1.004979
15533	LOC10323	1.268057	4.507539	2.593554	1.737977	0.082215	0.140667	0	0	0	4.464254	2.139111	1.004979
15534	LOC11962	1.268057	4.507539	2.593554	1.737977	0.082215	0.140667	0	0	0	4.464254	2.139111	1.004979
15535	LOC10323	1.268057	4.507539	2.593554	1.737977	0.082215	0.140667	0	0	0	4.464254	2.139111	1.004979
15536	LOC11962	1.268057	4.507539	2.593554	1.737977	0.082215	0.140667	0	0	0	4.464254	2.139111	1.004979
15537	KRTAP27-1	1.268057	4.507539	2.593554	1.737977	0.082215	0.140667	0	0	0	4.464254	2.139111	1.004979
15538	LOC11962	1.268057	4.507539	2.593554	1.737977	0.082215	0.140667	0	0	0	4.464254	2.139111	1.004979
16611	LOC11962	1.194001	4.506043	2.895913	1.556001	0.119708	0.191551	0	0	0	0	2.139111	5.024897
16612	FIGLA	1.194001	4.506043	2.895913	1.556001	0.119708	0.191551	0	0	0	0	2.139111	5.024897
16613	DUXB	1.194001	4.506043	2.895913	1.556001	0.119708	0.191551	0	0	0	0	2.139111	5.024897
16614	LOC11962	1.194001	4.506043	2.895913	1.556001	0.119708	0.191551	0	0	0	0	2.139111	5.024897
7179	OR52A1	10.30126	4.505869	1.227226	3.671587	0.000241	0.000893	0	0	2.782925	15.62489	36.36488	7.034856
10018	NEUROD4	5.121141	4.503927	1.576359	2.85717	0.004274	0.011343	0	0	1.391463	15.62489	10.69555	3.014938
5341	LOC10324	12.32797	4.502163	1.001729	4.49439	6.98E-06	3.47E-05	0	0.547412	2.782925	29.01765	23.53022	18.08963
7615	TOX2	7.370447	4.501395	1.2784	3.521117	0.00043	0.0015	0	0.547412	1.391463	24.5534	10.69555	7.034856
10005	CD248	5.105638	4.500352	1.573626	2.859862	0.004238	0.011262	0	0	1.391463	13.39276	12.83466	3.014938
6463	CCL24	9.396531	4.500277	1.138003	3.954539	7.67E-05	0.000315	0	1.642235	0.695731	29.01765	14.97377	10.04979
9780	PIRT	5.053107	4.499892	1.54685	2.909067	0.003625	0.009852	0	0	1.391463	8.928508	14.97377	5.024897
7534	ESR2	7.354944	4.499069	1.268191	3.547627	0.000389	0.001372	0	0.547412	1.391463	22.32127	12.83466	7.034856
8386	LOC11961	7.56911	4.497955	1.36842	3.28697	0.001013	0.00321	0	0	2.087194	4.464254	27.80844	11.05477
16474	GIMD1	1.215527	4.496686	2.852771	1.576252	0.114968	0.185488	0	0	0	0	4.278221	3.014938
16476	LOC10322	1.215527	4.496686	2.852771	1.576252	0.114968	0.185488	0	0	0	0	4.278221	3.014938
16484	LOC11962	1.215527	4.496686	2.852771	1.576252	0.114968	0.185488	0	0	0	0	4.278221	3.014938
16485	LOC11962	1.215527	4.496686	2.852771	1.576252	0.114968	0.185488	0	0	0	0	4.278221	3.014938
16494	LOC11962	1.215527	4.496686	2.852771	1.576252	0.114968	0.185488	0	0	0	0	4.278221	3.014938
9951	LOC11962	5.037604	4.494596	1.563567	2.874578	0.004046	0.010808	0	0	1.391463	6.696381	17.11289	5.024897
15616	LOC11962	1.252555	4.493556	2.602035	1.726939	0.084179	0.143097	0	0	0	2.232127	4.278221	1.004979
15617	INS	1.252555	4.493556	2.602035	1.726939	0.084179	0.143097	0	0	0	2.232127	4.278221	1.004979
15618	TAAR6	1.252555	4.493556	2.602035	1.726939	0.084179	0.143097	0	0	0	2.232127	4.278221	1.004979
15619	LOC11962	1.252555	4.493556	2.602035	1.726939	0.084179	0.143097	0	0	0	2.232127	4.278221	1.004979
15620	CLDN20	1.252555	4.493556	2.602035	1.726939	0.084179	0.143097	0	0	0	2.232127	4.278221	1.004979
15622	LOC11962	1.252555	4.493556	2.602035	1.726939	0.084179	0.143097	0	0	0	2.232127	4.278221	1.004979
15623	LOC11962	1.252555	4.493556	2.602035	1.726939	0.084179	0.143097	0	0	0	2.232127	4.278221	1.004979
15624	LOC10322	1.252555	4.493556	2.602035	1.726939	0.084179	0.143097	0	0	0	2.232127	4.278221	1.004979
15626	LOC11961	1.252555	4.493556	2.602035	1.726939	0.084179	0.143097	0	0	0	2.232127	4.278221	1.004979
15627	PTCHD3	1.252555	4.493556	2.602035	1.726939	0.084179	0.143097	0	0	0	2.232127	4.278221	1.004979
15628	LOC11961	1.252555	4.493556	2.602035	1.726939	0.084179	0.143097	0	0	0	2.232127	4.278221	1.004979
15629	LOC10323	1.252555	4.493556	2.602035	1.726939	0.084179	0.143097	0	0	0	2.232127	4.278221	1.004979
15630	LOC11962	1.252555	4.493556	2.602035	1.726939	0.084179	0.143097	0	0	0	2.232127	4.278221	1.004979
15631	VGLL1	1.252555	4.493556	2.602035	1.726939	0.084179	0.143097	0	0	0	2.232127	4.278221	1.004979
15633	ZG16	1.252555	4.493556	2.602035	1.726939	0.084179	0.143097	0	0	0	2.232127	4.278221	1.004979
15634	KRTAP11-1	1.252555	4.493556	2.602035	1.726939	0.084179	0.143097	0	0	0	2.232127	4.278221	1.004979
15635	LOC10323	1.252555	4.493556	2.602035	1.726939	0.084179	0.143097	0	0	0	2.232127	4.278221	1.004979
15636	LOC11962	1.252555	4.493556	2.602035	1.726939	0.084179	0.143097	0	0	0	2.232127	4.278221	1.004979
15638	NKX2-4	1.252555	4.493556	2.602035	1.726939	0.084179	0.143097	0	0	0	2.232127	4.278221	1.004979
3921	LOC11962	21.795	4.492993	0.827529	5.429407	5.65E-08	3.83E-07	1.626259	0.547412	3.478657	46.87467	64.17332	14.06971
7576	LOC10323	7.181849	4.492846	1.271417	3.533731	0.00041	0.001438	0	0.547412	1.391463	6.696381	21.39111	13.06473
7705	TGM7	7.344576	4.491933	1.28648	3.491646	0.00048	0.001656	0.542086	0	1.391463	17.85702	19.252	5.024897
12105	FAM135B	26.84104	4.491302	1.882811	2.385423	0.017059	0.037466	0	0	6.957314	51.33892	70.59065	32.15934
7087	PLD5	9.674849	4.489334	1.212857	3.701452	0.000214	0.000804	0	1.094824	1.391463	29.01765	23.53022	3.014938
8144	LOC11962	7.054209	4.488324	1.333325	3.366263	0.000762	0.002487	0	1.094824	0.695731	6.696381	27.80844	6.029877
16659	LOC11962	1.237052	4.485991	2.901658	1.546009	0.122102	0.194691	0	0	0	0	6.417332	1.004979
16660	LOC10323	1.237052	4.485991	2.901658	1.546009	0.122102	0.194691	0	0	0	0	6.417332	1.004979
16661	LOC11962	1.237052	4.485991	2.901658	1.546009	0.122102	0.194691	0	0	0	0	6.417332	1.004979
16662	CLRN1	1.237052	4.485991	2.901658	1.546009	0.122102	0.194691	0	0	0	0	6.417332	1.004979
16663	TAS2R16	1.237052	4.485991	2.901658	1.546009	0.122102	0.194691	0	0	0	0	6.417332	1.004979
16664	LOC10322	1.237052	4.485991	2.901658	1.546009	0.122102	0.194691	0	0	0	0	6.417332	1.004979
16665	LOC11962	1.237052	4.485991	2.901658	1.546009	0.122102	0.194691	0	0	0	0	6.417332	1.004979
16666	LOC11962	1.237052	4.485991	2.901658	1.546009	0.122102	0.194691	0	0	0	0	6.417332	1.004979
16667	LOC11961	1.237052	4.485991	2.901658	1.546009	0.122102	0.194691	0	0	0	0	6.417332	1.004979
16668	CPB2	1.237052	4.485991	2.901658	1.546009	0.122102	0.194691	0	0	0	0	6.417332	1.004979
16669	LOC10323	1.237052	4.485991	2.901658	1.546009	0.122102	0.194691	0	0	0	0	6.417332	1.004979
16670	LOC11961	1.237052	4.485991	2.901658	1.546009	0.122102	0.194691	0	0	0	0	6.417332	1.004979
16672	LOC11962	1.237052	4.485991	2.901658	1.546009	0.122102	0.194691	0	0	0	0	6.417332	1.004979
16673	LOC10323	1.237052	4.485991	2.901658	1.546009	0.122102	0.194691	0	0	0	0	6.417332	1.004979

11909	LOC10324{	4.538206	4.485271	1.851233	2.422856	0.015399	0.034376	0	1.094824	0	11.16063	14.97377	0
4173	LOC11962(	16.37225	4.483355	0.854878	5.244437	1.57E-07	9.99E-07	1.626259	0	2.782925	24.5534	32.08666	37.18424
7823	LRIT3	7.463624	4.481942	1.295443	3.459775	0.000541	0.001837	0	0	2.087194	13.39276	19.252	10.04979
5115	LOC11962(	14.80909	4.480823	0.969612	4.621254	3.81E-06	1.98E-05	0	0.547412	3.478657	31.24978	38.50399	15.07469
7922	LOC11962:	7.500652	4.480387	1.305391	3.432219	0.000599	0.002009	0	0	2.087194	15.62489	19.252	8.039836
7329	GALNT5	10.14926	4.48001	1.236244	3.623887	0.00029	0.001053	0	0	2.782925	17.85702	34.22577	6.029877
5467	CHL1	12.01131	4.479988	1.011271	4.430057	9.42E-06	4.58E-05	0	1.094824	2.087194	29.01765	27.80844	12.05975
10585	RXFP4	5.006599	4.479303	1.643039	2.726231	0.006406	0.01609	0	0	1.391463	2.232127	21.39111	5.024897
10167	SPRR3	4.471855	4.475787	1.586009	2.822044	0.004772	0.012478	1.084173	0	0	11.16063	8.556443	6.029877
8646	GLRA3	6.772009	4.474907	1.391415	3.216084	0.0013	0.003996	0	1.642235	0	17.85702	17.11289	4.019918
6575	CAPSL	9.69397	4.472087	1.143706	3.91017	9.22E-05	0.000373	0	0.547412	2.087194	13.39276	32.08666	10.04979
10346	LOC10322:	4.510658	4.470318	1.609458	2.77753	0.005477	0.014075	0	1.094824	0	13.39276	8.556443	4.019918
8445	LOC10322:	7.543703	4.46971	1.366732	3.270363	0.001074	0.003381	0	0	2.087194	15.62489	23.53022	4.019918
9742	PRKACG	4.717489	4.469303	1.531329	2.918577	0.003516	0.009596	0.542086	0	0.695731	15.62489	6.417332	5.024897
4183	FFAR4	22.00078	4.467648	0.853077	5.237097	1.63E-07	1.04E-06	1.084173	0	4.87012	51.33892	55.61688	19.09461
5820	LOC11962(	14.81165	4.466311	1.047677	4.263062	2.02E-05	9.21E-05	0	0.547412	3.478657	22.32127	53.47777	9.044815
7512	BSPH1	7.128894	4.466283	1.256496	3.554555	0.000379	0.00134	0	0.547412	1.391463	20.08914	10.69555	10.04979
9842	LOC10324(	4.836537	4.464453	1.541772	2.895664	0.003784	0.01022	0	0	1.391463	11.16063	6.417332	10.04979
6311	GP1BA	15.21143	4.463931	1.110038	4.021423	5.78E-05	0.000244	0	0	4.174388	49.10679	29.94755	8.039836
7884	HAPLN1	7.20295	4.462172	1.295044	3.445575	0.00057	0.001922	0	0.547412	1.391463	24.5534	10.69555	6.029877
9787	LOC11962(	4.858063	4.459842	1.533985	2.907357	0.003645	0.009901	0	0	1.391463	11.16063	8.556443	8.039836
7924	LOC11962:	6.964226	4.459233	1.299368	3.431849	0.000599	0.002011	0	1.094824	0.695731	17.85702	17.11289	5.024897
12817	GABRA6	2.364272	4.456541	1.994126	2.234834	0.025428	0.052743	0	0.547412	0	4.464254	2.139111	7.034856
4793	LMO3	19.68522	4.455488	0.924802	4.817777	1.45E-06	8.05E-06	0	0.547412	4.87012	35.71403	59.8951	17.08465
9922	DDI1	4.708009	4.454876	1.547067	2.879563	0.003982	0.01067	0.542086	0	0.695731	11.16063	12.83466	3.014938
6218	TDRD9	9.327347	4.454805	1.095357	4.066991	4.76E-05	0.000204	0.542086	0.547412	1.391463	15.62489	27.80844	10.04979
10208	LOC10322:	4.602948	4.452628	1.581891	2.814749	0.004882	0.012713	0.542086	0	0.695731	2.232127	17.11289	7.034856
10127	HIGD2B	4.932119	4.452355	1.572415	2.831539	0.004632	0.012161	0	0	1.391463	15.62489	8.556443	4.019918
7242	CYP11B2	9.907712	4.452306	1.218206	3.654807	0.000257	0.000945	0	0	2.782925	13.39276	34.22577	9.044815
4572	LOC10324(	16.46917	4.451995	0.897727	4.959187	7.08E-07	4.12E-06	0	1.642235	2.782925	31.24978	47.06043	16.07967
4096	SLFN12L	18.89519	4.451992	0.840035	5.299772	1.16E-07	7.53E-07	0	1.642235	3.478657	37.94616	49.19955	21.10457
7809	TAF7L	7.125437	4.449241	1.284768	3.463069	0.000534	0.001818	0	0.547412	1.391463	13.39276	21.39111	6.029877
8193	CR1L	7.379664	4.448055	1.328398	3.348435	0.000813	0.002637	0	0	2.087194	22.32127	12.83466	7.034856
3994	LOC10322(	26.50347	4.446834	0.826799	5.378375	7.52E-08	5.00E-07	0	1.642235	5.565851	53.57105	79.14709	19.09461
12619	PYDC1	2.410517	4.443987	1.951476	2.277243	0.022772	0.047974	0	0	0.695731	4.464254	4.278221	5.024897
12646	CCDC153	2.385797	4.443941	1.956373	2.271521	0.023115	0.048594	0	0.547412	0	4.464254	4.278221	5.024897
10146	LOC11961!	4.811555	4.443454	1.57197	2.826679	0.004703	0.012324	0	0	1.391463	4.464254	14.97377	8.039836
7006	PNOC	9.827209	4.442948	1.190943	3.730615	0.000191	0.000725	0	0	2.782925	29.01765	17.11289	10.04979
5031	LY6H	19.79133	4.442039	0.950584	4.672958	2.97E-06	1.57E-05	0	0	5.565851	35.71403	51.33866	26.12947
12788	CTSG	2.395015	4.436568	1.979158	2.241644	0.024984	0.05194	0	0	0.695731	2.232127	6.417332	5.024897
11187	LOC10323:	4.668411	4.436557	1.716459	2.584714	0.009746	0.023161	0	0.547412	0.695731	2.232127	23.53022	1.004979
8402	LILRB4	7.354681	4.435507	1.351319	3.282355	0.001029	0.003257	0	0	2.087194	15.62489	21.39111	5.024897
13753	LOC10323(	2.333267	4.43525	2.155592	2.057555	0.039633	0.076612	0	0.547412	0	0	6.417332	7.034856
4939	LOC10323(	16.64546	4.433986	0.939444	4.719797	2.36E-06	1.27E-05	0	1.094824	3.478657	46.87467	36.36488	12.05975
13110	CUNH1orf:	2.459853	4.433711	2.028475	2.185736	0.028835	0.058468	0	0.547412	0	8.928508	4.278221	1.004979
13111	LOC10324(	2.459853	4.433711	2.028475	2.185736	0.028835	0.058468	0	0.547412	0	8.928508	4.278221	1.004979
4812	VIT	16.3232	4.433674	0.922562	4.805827	1.54E-06	8.51E-06	0	1.642235	2.782925	31.24978	49.19955	13.06473
13081	LOC11962:	2.484573	4.433364	2.023761	2.190655	0.028477	0.057872	0	0	0.695731	8.928508	4.278221	1.004979
13082	LOC11962:	2.484573	4.433364	2.023761	2.190655	0.028477	0.057872	0	0	0.695731	8.928508	4.278221	1.004979
6931	LOC10324:	9.296805	4.431787	1.177508	3.7637	0.000167	0.000642	0	1.094824	1.391463	29.01765	19.252	5.024897
7976	SULT4A1	6.784684	4.431511	1.296582	3.41784	0.000631	0.002104	0	1.094824	0.695731	22.32127	8.556443	8.039836
5784	VAT1L	11.86251	4.429747	1.034199	4.283266	1.84E-05	8.47E-05	0	0.547412	2.782925	31.24978	23.53022	13.06473
12707	LOC11962(	2.407323	4.429063	1.962557	2.256781	0.024022	0.050257	0	0.547412	0	4.464254	6.417332	3.014938
12687	LOC11962(	2.432043	4.428898	1.957746	2.262244	0.023682	0.049614	0	0	0.695731	4.464254	6.417332	3.014938
12688	LOC11962(	2.432043	4.428898	1.957746	2.262244	0.023682	0.049614	0	0	0.695731	4.464254	6.417332	3.014938
12689	LOC11962(	2.432043	4.428898	1.957746	2.262244	0.023682	0.049614	0	0	0.695731	4.464254	6.417332	3.014938
12690	IL36B	2.432043	4.428898	1.957746	2.262244	0.023682	0.049614	0	0	0.695731	4.464254	6.417332	3.014938
13742	LOC11962!	2.379512	4.427185	2.147714	2.061348	0.03927	0.075971	0	0	0.695731	0	8.556443	5.024897
10705	GASK1A	4.891633	4.427176	1.645664	2.690207	0.007141	0.017734	0	0	1.391463	6.696381	19.252	2.009959
1262	STC1	81.94153	4.426048	0.444037	9.967744	2.11E-23	4.44E-22	2.710432	7.116354	12.52316	124.9991	209.6328	134.6672
13077	LOC11962:	2.444351	4.425441	2.01975	2.191083	0.028446	0.05782	0	0.547412	0	6.696381	6.417332	1.004979
13050	RHEX	2.469071	4.425095	2.014996	2.196081	0.028086	0.057212	0	0	0.695731	6.696381	6.417332	1.004979
13051	MUC3A	2.469071	4.425095	2.014996	2.196081	0.028086	0.057212	0	0	0.695731	6.696381	6.417332	1.004979
5873	CD1C	11.56178	4.423827	1.046132	4.228746	2.35E-05	0.000106	0	1.094824	2.087194	15.62489	38.50399	12.05975
608	ACP5	116.7415	4.423687	0.32692	13.53141	1.02E-41	4.46E-40	11.38381	13.6853	5.565851	212.0521	224.6066	233.1552
12966	PPP4R3C	2.39182	4.422897	2.001078	2.210256	0.027087	0.055535	0	0.547412	0	2.232127	8.556443	3.014938
12967	LOC11962:	2.39182	4.422897	2.001078	2.210256	0.027087	0.055535	0	0.547412	0	2.232127	8.556443	3.014938
12934	LLCFC1	2.41654	4.422758	1.99615	2.215644	0.026716	0.054913	0	0	0.695731	2.232127	8.556443	3.014938
8155	PLA2G2D	7.035454	4.42198	1.314569	3.363825	0.000769	0.002506	0	0.547412	1.391463	24.5534	10.69555	5.024897
3832	FBN3	31.32336	4.4199	0.80188	5.511922	3.55E-08	2.46E-07	0.542086	0.547412	7.653045	55.80317	96.25998	27.13445
13179	S100G	2.428848	4.41867	2.032439	2.174073	0.0297	0.059913	0	0.547412	0	4.464254	8.556443	1.004979
13158	LOC11962:	2.453568	4.418343	2.027634	2.179063	0.029327	0.059249	0	0	0.695731	4.464254	8.556443	1.004979
13159	CUNH10or	2.453568	4.418343	2.027634	2.179063	0.029327	0.059249	0	0	0.695731	4.464254	8.556443	1.004979
10563	BOD1L2	4.373279	4.417987	1.61823	2.730136	0.006331	0.015933	0.542086	0.547412	0	17.85702	4.278221	3.014938

10829	HEMGN	4.358664	4.415912	1.656942	2.665097	0.007697	0.018895	0	1.094824	0	15.62489	6.417332	3.014938
7731	LMX1A	6.899387	4.415392	1.267719	3.482942	0.000496	0.001705	0	0.547412	1.391463	11.16063	19.252	9.044815
8075	SPINT1	7.101083	4.414077	1.301865	3.390581	0.000697	0.002296	0	0	2.087194	15.62489	12.83466	12.05975
14295	LOC11962!	2.422562	4.413913	2.249253	1.962391	0.049717	0.092461	0	0	0.695731	0	12.83466	1.004979
11279	LOC11961{	4.860628	4.413878	1.722659	2.562247	0.0104	0.024513	0	0	1.391463	2.232127	23.53022	2.009959
13404	LOC11962(	2.412458	4.413634	2.075432	2.126609	0.033453	0.066349	0.542086	0	0	2.232127	10.69555	1.004979
13375	LOC11962:	2.438065	4.413334	2.070352	2.131683	0.033033	0.065662	0	0	0.695731	2.232127	10.69555	1.004979
5506	CUNH12or	16.91924	4.412478	1.00082	4.408864	1.04E-05	5.02E-05	0	0	4.87012	22.32127	49.19955	25.12449
5118	SGCD	16.38495	4.409143	0.954325	4.62017	3.83E-06	1.99E-05	0	1.094824	3.478657	33.4819	49.19955	11.05477
7357	KCNA6	9.665735	4.407987	1.219701	3.613992	0.000302	0.00109	0	0	2.782925	26.78552	21.39111	7.034856
6109	LOC10321:	11.25459	4.407075	1.070822	4.115599	3.86E-05	0.000168	0	1.642235	1.391463	20.08914	36.36488	8.039836
7490	LOC11961!	9.402028	4.405676	1.23531	3.566453	0.000362	0.001284	0	0.547412	2.087194	13.39276	36.36488	4.019918
8181	LOC10321	6.942437	4.40567	1.314298	3.352109	0.000802	0.002606	0	0.547412	1.391463	11.16063	23.53022	5.024897
2003	GIMAP7	38.70656	4.4006	0.551968	7.972557	1.55E-15	2.06E-14	2.168345	6.02153	2.087194	87.05295	70.59065	64.31869
4948	GCGR	13.64076	4.396798	0.932698	4.714065	2.43E-06	1.30E-05	0	1.094824	2.782925	26.78552	32.08666	19.09461
7683	LOC11962(	9.104326	4.395811	1.256784	3.497667	0.000469	0.001624	0	1.094824	1.391463	22.32127	27.80844	2.009959
10120	UGT3A1	4.675063	4.395574	1.551682	2.832781	0.004614	0.012122	0	0	1.391463	8.928508	10.69555	7.034856
6896	GRIFIN	12.04179	4.394988	1.163487	3.777429	0.000158	0.000611	0	0	3.478657	20.08914	40.6431	8.039836
7436	LOC11961{	9.477138	4.394115	1.225362	3.585973	0.000336	0.001201	0	0	2.782925	8.928508	32.08666	13.06473
2349	CHST4	34.36709	4.393	0.598342	7.341957	2.10E-13	2.38E-12	4.336691	1.642235	3.478657	37.94616	83.42532	75.37346
10226	PRSS54	4.712091	4.392026	1.563735	2.808677	0.004975	0.012934	0	0	1.391463	11.16063	10.69555	5.024897
8807	LOC10324(	7.171682	4.391279	1.384338	3.172116	0.001513	0.004568	0	0	2.087194	13.39276	23.53022	4.019918
6530	LOC10321(	11.69669	4.391049	1.118224	3.926805	8.61E-05	0.00035	0.542086	0	2.782925	22.32127	38.50399	6.029877
7477	LOC10322!	12.10034	4.389951	1.229465	3.570619	0.000356	0.001266	0	0	3.478657	22.32127	42.78221	4.019918
11611	LOC11962(	4.547423	4.389562	1.761669	2.491707	0.012713	0.029108	0	0.547412	0.695731	8.928508	17.11289	0
10457	MINAR1	4.525897	4.387369	1.592786	2.754524	0.005878	0.014943	0	0.547412	0.695731	8.928508	14.97377	2.009959
9310	LOC11962(	7.193207	4.384833	1.446649	3.031027	0.002437	0.006959	0	0	2.087194	13.39276	25.66933	2.009959
10578	LOC10322(	4.510395	4.383819	1.607213	2.727591	0.00638	0.016034	0	0.547412	0.695731	6.696381	17.11289	2.009959
4677	LOC10322	21.18121	4.383303	0.894638	4.899529	9.61E-07	5.46E-06	0	0.547412	5.565851	62.49956	36.36488	22.10955
5067	LOC10324:	12.95339	4.382546	0.942224	4.651276	3.30E-06	1.73E-05	1.084173	1.642235	0.695731	29.01765	34.22577	11.05477
10391	HTR3B	4.681086	4.382255	1.583238	2.767907	0.005642	0.014434	0	0	1.391463	6.696381	14.97377	5.024897
7098	PLA2G5	9.080397	4.380451	1.185103	3.696262	0.000219	0.000819	0	0.547412	2.087194	6.696381	32.08666	13.06473
8235	RHOH	6.592204	4.377089	1.31221	3.335662	0.000851	0.002747	0	1.094824	0.695731	15.62489	17.11289	5.024897
7247	LOC11962:	9.393177	4.375879	1.197567	3.653975	0.000258	0.000947	0	0	2.782925	17.85702	25.66933	10.04979
6617	LOC10323{	8.844705	4.374986	1.123129	3.895356	9.81E-05	0.000394	0	1.094824	1.391463	24.5534	14.97377	11.05477
6478	SYNDIG1	8.792175	4.374692	1.108056	3.94808	7.88E-05	0.000323	0	1.094824	1.391463	20.08914	17.11289	13.06473
3	VCAM1	18494.96	4.372594	0.088988	49.13691	0	0	1702.693	1748.433	1659.319	34089.04	33768	38002.29
6903	NPSR1	9.094588	4.367833	1.156742	3.775977	0.000159	0.000614	0.542086	0	2.087194	26.78552	17.11289	8.039836
7244	LOC10322:	8.633529	4.367165	1.195128	3.65414	0.000258	0.000947	0	1.642235	0.695731	15.62489	27.80844	6.029877
4237	LOC10324:	23.03519	4.366787	0.840459	5.195715	2.04E-07	1.28E-06	0.542086	0.547412	5.565851	53.57105	59.8951	18.08963
6709	LOC10323(	11.54559	4.366614	1.133559	3.852127	0.000117	0.000464	0	0.547412	2.782925	24.5534	36.36488	5.024897
3162	LOC11962	21.37026	4.366586	0.703962	6.202868	5.54E-10	4.66E-09	2.168345	1.094824	2.782925	46.87467	32.08666	43.21412
6442	SYNDIG1L	14.21635	4.363793	1.10081	3.964166	7.37E-05	0.000304	0	0	4.174388	35.71403	36.36488	9.044815
8411	RESP18	6.924107	4.362185	1.33051	3.278581	0.001043	0.003298	0	0	2.087194	11.16063	19.252	9.044815
9054	LOC11961!	7.066196	4.361104	1.40904	3.09509	0.001968	0.005777	0	0	2.087194	22.32127	14.97377	3.014938
3817	IL25	27.36091	4.360616	0.788841	5.527874	3.24E-08	2.26E-07	0	1.642235	6.261582	66.96381	64.17332	25.12449
8274	FABP12	6.123977	4.360079	1.312763	3.321299	0.000896	0.002879	0.542086	1.094824	0	15.62489	6.417332	13.06473
2415	NR0B1	33.22953	4.359982	0.601288	7.25107	4.13E-13	4.55E-12	1.626259	6.02153	1.391463	64.73168	66.31243	59.29379
575	RET	135.2603	4.357173	0.314727	13.84429	1.38E-43	6.37E-42	7.589209	15.32753	15.30609	216.5163	267.3888	289.4341
9445	TRIM40	6.252241	4.353894	1.452107	2.998329	0.002715	0.007641	1.084173	0.547412	0	15.62489	19.252	1.004979
9333	LOC10324(	6.247994	4.353799	1.440293	3.022856	0.002504	0.007133	0	1.642235	0	17.85702	14.97377	3.014938
7753	TMEM132(	8.562408	4.353728	1.252043	3.477299	0.000506	0.001737	1.626259	0	0.695731	26.78552	19.252	3.014938
6830	GLOD5	11.66375	4.351992	1.143236	3.806731	0.000141	0.000548	0	0	3.478657	20.08914	36.36488	10.04979
5371	DIPK2B	13.08856	4.351552	0.971346	4.479921	7.47E-06	3.70E-05	0	1.642235	2.087194	20.08914	40.6431	14.06971
7245	LOC11962:	9.219658	4.350832	1.190683	3.654064	0.000258	0.000947	0	0	2.782925	20.08914	21.39111	11.05477
10125	SCRT2	4.309328	4.349198	1.535777	2.83192	0.004627	0.012149	0	0.547412	0.695731	11.16063	6.417332	7.034856
9135	LOC10324:	7.004185	4.349133	1.416099	3.071206	0.002132	0.006205	0	0	2.087194	13.39276	23.53022	3.014938
4828	SLC6A2	17.80625	4.347358	0.906559	4.795452	1.62E-06	8.94E-06	0	1.642235	3.478657	46.87467	42.78221	12.05975
5589	LOC10323:	13.09972	4.346881	0.99313	4.376949	1.20E-05	5.72E-05	0.542086	1.094824	2.087194	15.62489	49.19955	10.04979
13589	ANKUB1	2.159748	4.343012	2.076262	2.091745	0.036461	0.071331	0	0.547412	0	2.232127	2.139111	8.039836
14641	LOC10323(	2.306972	4.337732	2.285005	1.898347	0.05765	0.104674	0.542086	0	0	11.16063	2.139111	0
14642	LOC11962!	2.306972	4.337732	2.285005	1.898347	0.05765	0.104674	0.542086	0	0	11.16063	2.139111	0
14621	LOC11962(	2.332579	4.337344	2.278576	1.903533	0.056971	0.103582	0	0	0.695731	11.16063	2.139111	0
8534	HGF	6.760068	4.335513	1.336076	3.24496	0.001175	0.003659	0	0	2.087194	17.85702	8.556443	12.05975
8685	LOC10322:	6.16357	4.335115	1.351406	3.207856	0.001337	0.004094	0.542086	1.094824	0	8.928508	21.39111	5.024897
8676	LOC11962	6.445346	4.333594	1.349908	3.210287	0.001326	0.004063	0.542086	0.547412	0.695731	15.62489	19.252	2.009959
10963	LOC10322!	4.389406	4.333184	1.643606	2.636389	0.008379	0.02032	0	0.547412	0.695731	13.39276	10.69555	1.004979
1508	LOC10323	62.42067	4.331254	0.469202	9.23111	2.68E-20	4.72E-19	5.420864	10.40082	1.391463	127.2312	115.512	114.5677
8328	LOC11961{	6.616924	4.330935	1.310092	3.305825	0.000947	0.003023	0	0.547412	1.391463	15.62489	17.11289	5.024897
6001	LOC10323:	69.09739	4.329492	1.039915	4.163313	3.14E-05	0.000139	0.542086	6.02153	13.2189	176.338	156.1551	62.30873
10472	LOC11961!	4.336876	4.328262	1.573444	2.75082	0.005945	0.015091	0	0.547412	0.695731	8.928508	12.83466	3.014938
10489	CFHR5	4.284345	4.327936	1.575305	2.747363	0.006008	0.015227	0	0.547412	0.695731	4.464254	14.97377	5.024897
1708	CRB2	47.65576	4.327818	0.497346	8.701834	3.27E-18	5.08E-17	2.168345	6.568942	4.87012	73.66019	111.2338	87.43321
6973	FGF4	8.82894	4.326959	1.155147	3.745807	0.00018	0.000686	0	0.547412	2.087194	15.62489	25.66933	9.044815

10556	LOC10322	4.492064	4.326925	1.583553	2.732417	0.006287	0.015834	0	0	1.391463	6.696381	12.83466	6.029877
13471	LOC11961	2.295551	4.3262	2.047756	2.112654	0.03463	0.068333	0	0	0.695731	8.928508	2.139111	2.009959
13472	POF1B	2.295551	4.3262	2.047756	2.112654	0.03463	0.068333	0	0	0.695731	8.928508	2.139111	2.009959
13473	INSL3	2.295551	4.3262	2.047756	2.112654	0.03463	0.068333	0	0	0.695731	8.928508	2.139111	2.009959
14460	LOC11962	2.317077	4.326177	2.23899	1.9322	0.053335	0.098057	0	0	0.695731	8.928508	4.278221	0
13224	LOC11961	2.217413	4.324656	1.998479	2.163974	0.030466	0.061248	0.542086	0	0	4.464254	4.278221	4.019918
13194	LOC11962	2.243021	4.324625	1.992812	2.170112	0.029998	0.060445	0	0	0.695731	4.464254	4.278221	4.019918
3689	LOC11962	28.48315	4.32453	0.765428	5.649818	1.61E-08	1.16E-07	0.542086	2.189647	5.565851	55.80317	87.70354	19.09461
10979	LOC10323	4.581623	4.323613	1.641569	2.63383	0.008443	0.020442	0	0	1.391463	13.39276	10.69555	2.009959
10980	VSIG4	4.581623	4.323613	1.641569	2.63383	0.008443	0.020442	0	0	1.391463	13.39276	10.69555	2.009959
8744	LOC11962	6.809141	4.32131	1.354672	3.189932	0.001423	0.004327	0	0	2.087194	15.62489	17.11289	6.029877
18373	LOC11962	1.116063	4.321142	3.409414	1.267415	0.205007	0.296443	0	0	0	6.696381	0	0
18374	TRNAH-GU	1.116063	4.321142	3.409414	1.267415	0.205007	0.296443	0	0	0	6.696381	0	0
18375	LOC10324	1.116063	4.321142	3.409414	1.267415	0.205007	0.296443	0	0	0	6.696381	0	0
18376	LOC10321	1.116063	4.321142	3.409414	1.267415	0.205007	0.296443	0	0	0	6.696381	0	0
18378	LOC11962	1.116063	4.321142	3.409414	1.267415	0.205007	0.296443	0	0	0	6.696381	0	0
18379	WFDC10A	1.116063	4.321142	3.409414	1.267415	0.205007	0.296443	0	0	0	6.696381	0	0
18380	LOC11962	1.116063	4.321142	3.409414	1.267415	0.205007	0.296443	0	0	0	6.696381	0	0
18381	LOC11962	1.116063	4.321142	3.409414	1.267415	0.205007	0.296443	0	0	0	6.696381	0	0
18382	LOC11962	1.116063	4.321142	3.409414	1.267415	0.205007	0.296443	0	0	0	6.696381	0	0
18383	LOC10322	1.116063	4.321142	3.409414	1.267415	0.205007	0.296443	0	0	0	6.696381	0	0
10742	LOC11962	4.30587	4.320886	1.611278	2.681652	0.007326	0.018131	0	0.547412	0.695731	4.464254	17.11289	3.014938
14473	PTGDR	2.276854	4.317396	2.23739	1.929657	0.053649	0.098547	0	0.547412	0	6.696381	6.417332	0
4947	SLC36A2	15.11113	4.317314	0.915594	4.715317	2.41E-06	1.30E-05	0	1.642235	2.782925	37.94616	34.22577	14.06971
13386	ARL14EPL	2.202798	4.316991	2.026744	2.130013	0.033171	0.065878	0	0.547412	0	2.232127	6.417332	4.019918
14445	LRRC2	2.301574	4.31699	2.230921	1.93507	0.052982	0.097502	0	0	0.695731	6.696381	6.417332	0
14446	PRR4	2.301574	4.31699	2.230921	1.93507	0.052982	0.097502	0	0	0.695731	6.696381	6.417332	0
13353	LOC10323	2.227518	4.316982	2.021151	2.135903	0.032687	0.065077	0	0	0.695731	2.232127	6.417332	4.019918
13316	LOC10323	2.280049	4.316373	2.013688	2.143516	0.032072	0.063992	0	0	0.695731	6.696381	4.278221	2.009959
13317	HTR1E	2.280049	4.316373	2.013688	2.143516	0.032072	0.063992	0	0	0.695731	6.696381	4.278221	2.009959
13319	LOC11962	2.280049	4.316373	2.013688	2.143516	0.032072	0.063992	0	0	0.695731	6.696381	4.278221	2.009959
13321	RERGL	2.280049	4.316373	2.013688	2.143516	0.032072	0.063992	0	0	0.695731	6.696381	4.278221	2.009959
13324	LOC11962	2.280049	4.316373	2.013688	2.143516	0.032072	0.063992	0	0	0.695731	6.696381	4.278221	2.009959
4879	LOC11962	17.85869	4.315402	0.907913	4.7531	2.00E-06	1.09E-05	0.542086	0	4.87012	35.71403	44.92132	21.10457
10987	LOC10322	4.498087	4.311791	1.637868	2.632564	0.008474	0.020505	0	0	1.391463	4.464254	17.11289	4.019918
11131	LOC10322	4.461059	4.310051	1.660208	2.59609	0.009429	0.02252	0	0	1.391463	2.232127	17.11289	6.029877
13373	PRDM6	2.239826	4.308792	2.021285	2.131709	0.033031	0.065662	0	0.547412	0	4.464254	6.417332	2.009959
6192	LOC10324	10.96559	4.308538	1.056686	4.077405	4.55E-05	0.000196	0.542086	0	2.782925	29.01765	21.39111	12.05975
13348	LOC10324	2.264546	4.308522	2.01591	2.137259	0.032577	0.064873	0	0	0.695731	4.464254	6.417332	2.009959
13349	CPHXL	2.264546	4.308522	2.01591	2.137259	0.032577	0.064873	0	0	0.695731	4.464254	6.417332	2.009959
13350	LOC11962	2.264546	4.308522	2.01591	2.137259	0.032577	0.064873	0	0	0.695731	4.464254	6.417332	2.009959
6187	CUNH5orf	10.78028	4.308348	1.055784	4.08071	4.49E-05	0.000193	0	1.094824	2.087194	26.78552	25.66933	9.044815
2287	LOC11962	59.85873	4.307931	0.57895	7.440936	1.00E-13	1.16E-12	0.542086	6.568942	10.43597	113.8385	160.4333	67.33362
4108	CUNH8orf	19.20043	4.307704	0.814225	5.29056	1.22E-07	7.89E-07	0	2.189647	3.478657	44.64254	42.78221	22.10955
5428	TMEM176	12.93977	4.307574	0.968613	4.447158	8.70E-06	4.26E-05	0	1.094824	2.782925	22.32127	36.36488	15.07469
17363	LOC11962	1.079035	4.304465	2.996847	1.436331	0.150908	0.23098	0	0	0	4.464254	0	2.009959
17364	LOC10323	1.079035	4.304465	2.996847	1.436331	0.150908	0.23098	0	0	0	4.464254	0	2.009959
17365	LOC10323	1.079035	4.304465	2.996847	1.436331	0.150908	0.23098	0	0	0	4.464254	0	2.009959
17366	DLEU7	1.079035	4.304465	2.996847	1.436331	0.150908	0.23098	0	0	0	4.464254	0	2.009959
17367	LOC11962	1.079035	4.304465	2.996847	1.436331	0.150908	0.23098	0	0	0	4.464254	0	2.009959
17369	LOC11962	1.079035	4.304465	2.996847	1.436331	0.150908	0.23098	0	0	0	4.464254	0	2.009959
15759	TFF1	2.255066	4.303518	2.519936	1.707789	0.087676	0.147906	0	0	0.695731	0	12.83466	0
14797	LOC10324	2.245849	4.303188	2.296916	1.873464	0.061004	0.109603	0	0.547412	0	2.232127	10.69555	0
11338	LOC10322	4.482584	4.303138	1.688313	2.54878	0.01081	0.025347	0	0	1.391463	2.232127	19.252	4.019918
6505	IL7R	11.02503	4.303102	1.092394	3.93915	8.18E-05	0.000334	0	0.547412	2.782925	31.24978	23.53022	8.039836
10387	SLITRK4	27.92745	4.302881	1.554139	2.76866	0.005629	0.014406	0	0.547412	7.653045	53.57105	87.70354	18.08963
14765	LOC11961	2.270569	4.302842	2.290316	1.878711	0.060284	0.108536	0	0	0.695731	2.232127	10.69555	0
14766	LOC10323	2.270569	4.302842	2.290316	1.878711	0.060284	0.108536	0	0	0.695731	2.232127	10.69555	0
8403	LOC10322	5.999531	4.302641	1.310881	3.282251	0.00103	0.003258	0.542086	1.094824	0	15.62489	10.69555	8.039836
13607	RBBP8NL	2.223436	4.302513	2.060304	2.088291	0.036772	0.071843	0.542086	0	0	2.232127	8.556443	2.009959
13603	LOC10322	2.224323	4.302504	2.060104	2.088489	0.036754	0.071814	0	0.547412	0	2.232127	8.556443	2.009959
13605	LOC11962	2.224323	4.302504	2.060104	2.088489	0.036754	0.071814	0	0.547412	0	2.232127	8.556443	2.009959
13606	LOC11962	2.224323	4.302504	2.060104	2.088489	0.036754	0.071814	0	0.547412	0	2.232127	8.556443	2.009959
13571	FABP6	2.249043	4.302263	2.054479	2.09409	0.036252	0.071011	0	0	0.695731	2.232127	8.556443	2.009959
13572	LOC10323	2.249043	4.302263	2.054479	2.09409	0.036252	0.071011	0	0	0.695731	2.232127	8.556443	2.009959
6313	ATOH1	10.95611	4.302169	1.070017	4.020655	5.80E-05	0.000244	0.542086	0	2.782925	24.5534	27.80844	10.04979
6258	LOC11962	13.47619	4.300873	1.06299	4.046014	5.21E-05	0.000221	0	0	4.174388	20.08914	38.50399	18.08963
14547	LOC10323	2.208821	4.300286	2.243343	1.916909	0.055249	0.10097	0	0.547412	0	0	10.69555	2.009959
17474	LOC11962	1.042007	4.300194	3.03882	1.415087	0.157043	0.23883	0	0	0	2.232127	0	4.019918
17475	LOC10321	1.042007	4.300194	3.03882	1.415087	0.157043	0.23883	0	0	0	2.232127	0	4.019918
17477	APOC3	1.042007	4.300194	3.03882	1.415087	0.157043	0.23883	0	0	0	2.232127	0	4.019918
17478	LOC11962	1.042007	4.300194	3.03882	1.415087	0.157043	0.23883	0	0	0	2.232127	0	4.019918
17480	LOC10323	1.042007	4.300194	3.03882	1.415087	0.157043	0.23883	0	0	0	2.232127	0	4.019918
17481	LOC11962	1.042007	4.300194	3.03882	1.415087	0.157043	0.23883	0	0	0	2.232127	0	4.019918

8216	ATP6V0D2	6.40549	4.299161	1.286573	3.341561	0.000833	0.002696	0.542086	0	1.391463	15.62489	12.83466	8.039836
6269	LOC10323	10.85193	4.298031	1.063137	4.042784	5.28E-05	0.000224	0	0.547412	2.782925	15.62489	32.08666	14.06971
4606	RIOX1	19.47875	4.298016	0.869954	4.940509	7.79E-07	4.50E-06	0	1.642235	4.174388	44.64254	51.33866	15.07469
12890	LOC11962	26.0235	4.296839	1.935044	2.220538	0.026382	0.054412	0	0	7.653045	64.73168	55.61688	28.13943
12478	DYNLT5	4.55664	4.295812	1.863765	2.304911	0.021172	0.045107	0	0	1.391463	6.696381	19.252	0
3154	NCKAP1L	30.04069	4.294815	0.69134	6.212306	5.22E-10	4.40E-09	1.084173	1.642235	6.261582	73.66019	68.45154	29.1444
8687	IFIT1B	6.480433	4.292662	1.338314	3.207516	0.001339	0.004097	0	0.547412	1.391463	20.08914	12.83466	4.019918
17487	LOC11961	1.100561	4.291847	3.037936	1.412751	0.157729	0.23947	0	0	0	4.464254	2.139111	0
17488	LOC11962	1.100561	4.291847	3.037936	1.412751	0.157729	0.23947	0	0	0	4.464254	2.139111	0
17490	LOC10323	1.100561	4.291847	3.037936	1.412751	0.157729	0.23947	0	0	0	4.464254	2.139111	0
17491	LOC11962	1.100561	4.291847	3.037936	1.412751	0.157729	0.23947	0	0	0	4.464254	2.139111	0
17492	CCR3	1.100561	4.291847	3.037936	1.412751	0.157729	0.23947	0	0	0	4.464254	2.139111	0
17493	LOC10322	1.100561	4.291847	3.037936	1.412751	0.157729	0.23947	0	0	0	4.464254	2.139111	0
17494	LOC10324	1.100561	4.291847	3.037936	1.412751	0.157729	0.23947	0	0	0	4.464254	2.139111	0
17495	LOC10322	1.100561	4.291847	3.037936	1.412751	0.157729	0.23947	0	0	0	4.464254	2.139111	0
17496	SYT5	1.100561	4.291847	3.037936	1.412751	0.157729	0.23947	0	0	0	4.464254	2.139111	0
17497	LOC11962	1.100561	4.291847	3.037936	1.412751	0.157729	0.23947	0	0	0	4.464254	2.139111	0
17498	LOC11962	1.100561	4.291847	3.037936	1.412751	0.157729	0.23947	0	0	0	4.464254	2.139111	0
17499	LOC10324	1.100561	4.291847	3.037936	1.412751	0.157729	0.23947	0	0	0	4.464254	2.139111	0
17500	LOC11962	1.100561	4.291847	3.037936	1.412751	0.157729	0.23947	0	0	0	4.464254	2.139111	0
17502	CUNH20or	1.100561	4.291847	3.037936	1.412751	0.157729	0.23947	0	0	0	4.464254	2.139111	0
17503	LOC11961	1.100561	4.291847	3.037936	1.412751	0.157729	0.23947	0	0	0	4.464254	2.139111	0
17504	LOC10323	1.100561	4.291847	3.037936	1.412751	0.157729	0.23947	0	0	0	4.464254	2.139111	0
17505	LOC10324	1.100561	4.291847	3.037936	1.412751	0.157729	0.23947	0	0	0	4.464254	2.139111	0
17506	LOC10323	1.100561	4.291847	3.037936	1.412751	0.157729	0.23947	0	0	0	4.464254	2.139111	0
17509	LOC10323	1.100561	4.291847	3.037936	1.412751	0.157729	0.23947	0	0	0	4.464254	2.139111	0
17510	LOC10321	1.100561	4.291847	3.037936	1.412751	0.157729	0.23947	0	0	0	4.464254	2.139111	0
17511	LOC11962	1.100561	4.291847	3.037936	1.412751	0.157729	0.23947	0	0	0	4.464254	2.139111	0
1029	TJP3	76.07073	4.289968	0.394734	10.86799	1.64E-27	4.23E-26	5.96295	6.02153	10.43597	174.1059	143.3204	116.5776
4560	RASL12	17.08045	4.288832	0.863573	4.96638	6.82E-07	3.98E-06	1.084173	0	4.174388	42.41041	25.66933	29.1444
8384	DMRTB1	6.359869	4.287955	1.304326	3.287488	0.001011	0.003205	0	0.547412	1.391463	8.928508	19.252	8.039836
5983	GPHB5	10.16891	4.287702	1.027951	4.171116	3.03E-05	0.000135	0.542086	1.642235	0.695731	24.5534	23.53022	10.04979
13167	DPP10	23.51007	4.287482	1.969045	2.177442	0.029448	0.059457	0	0	6.957314	60.26743	57.75599	16.07967
5386	KLK4	14.81962	4.287356	0.959031	4.470507	7.80E-06	3.85E-05	0	1.642235	2.782925	20.08914	51.33866	13.06473
16473	CUNH5orf	1.063533	4.286907	2.720691	1.575669	0.115102	0.185488	0	0	0	2.232127	2.139111	2.009959
16475	LOC11962	1.063533	4.286907	2.720691	1.575669	0.115102	0.185488	0	0	0	2.232127	2.139111	2.009959
16477	LOC11962	1.063533	4.286907	2.720691	1.575669	0.115102	0.185488	0	0	0	2.232127	2.139111	2.009959
16478	INSL6	1.063533	4.286907	2.720691	1.575669	0.115102	0.185488	0	0	0	2.232127	2.139111	2.009959
16482	LOC11962	1.063533	4.286907	2.720691	1.575669	0.115102	0.185488	0	0	0	2.232127	2.139111	2.009959
16483	LOC10324	1.063533	4.286907	2.720691	1.575669	0.115102	0.185488	0	0	0	2.232127	2.139111	2.009959
16486	LRIT1	1.063533	4.286907	2.720691	1.575669	0.115102	0.185488	0	0	0	2.232127	2.139111	2.009959
16487	LOC10322	1.063533	4.286907	2.720691	1.575669	0.115102	0.185488	0	0	0	2.232127	2.139111	2.009959
16489	LOC10322	1.063533	4.286907	2.720691	1.575669	0.115102	0.185488	0	0	0	2.232127	2.139111	2.009959
16490	LOC10324	1.063533	4.286907	2.720691	1.575669	0.115102	0.185488	0	0	0	2.232127	2.139111	2.009959
16491	LOC10323	1.063533	4.286907	2.720691	1.575669	0.115102	0.185488	0	0	0	2.232127	2.139111	2.009959
16492	LOC11962	1.063533	4.286907	2.720691	1.575669	0.115102	0.185488	0	0	0	2.232127	2.139111	2.009959
16493	PRM2	1.063533	4.286907	2.720691	1.575669	0.115102	0.185488	0	0	0	2.232127	2.139111	2.009959
16496	SYCN	1.063533	4.286907	2.720691	1.575669	0.115102	0.185488	0	0	0	2.232127	2.139111	2.009959
16497	CSN3	1.063533	4.286907	2.720691	1.575669	0.115102	0.185488	0	0	0	2.232127	2.139111	2.009959
6205	LOC11962	13.3552	4.286621	1.053144	4.070308	4.70E-05	0.000201	0	0	4.174388	26.78552	32.08666	17.08465
4640	LRRC18	17.23547	4.285946	0.870443	4.923867	8.49E-07	4.86E-06	0.542086	0.547412	4.174388	49.10679	29.94755	19.09461
6331	LOC10324	15.79887	4.284115	1.067789	4.012136	6.02E-05	0.000253	0	0	4.87012	44.64254	34.22577	11.05477
8806	LOC10322	6.583091	4.283412	1.350178	3.172479	0.001511	0.004563	0	0	2.087194	13.39276	14.97377	9.044815
17588	GPR6	1.026505	4.283168	3.053752	1.402592	0.160739	0.242949	0	0	0	0	2.139111	4.019918
17589	LOC11962	1.026505	4.283168	3.053752	1.402592	0.160739	0.242949	0	0	0	0	2.139111	4.019918
9395	BEST4	6.694175	4.277147	1.421801	3.00826	0.002627	0.007435	0	0	2.087194	20.08914	14.97377	3.014938
8923	FGF6	6.589114	4.275884	1.362348	3.138613	0.001697	0.005057	0	0	2.087194	11.16063	19.252	7.034856
8924	LOC11962	6.589114	4.275884	1.362348	3.138613	0.001697	0.005057	0	0	2.087194	11.16063	19.252	7.034856
130	SMPD3	386.9101	4.275179	0.185303	23.07126	9.00E-118	1.84E-115	39.5723	38.31883	36.17803	743.2983	663.1243	800.9686
17592	ASIC5	1.085058	4.274666	3.051365	1.400903	0.161243	0.24309	0	0	0	2.232127	4.278221	0
17593	LOC11962	1.085058	4.274666	3.051365	1.400903	0.161243	0.24309	0	0	0	2.232127	4.278221	0
17594	LOC11961	1.085058	4.274666	3.051365	1.400903	0.161243	0.24309	0	0	0	2.232127	4.278221	0
17595	LOC11962	1.085058	4.274666	3.051365	1.400903	0.161243	0.24309	0	0	0	2.232127	4.278221	0
17596	LOC10323	1.085058	4.274666	3.051365	1.400903	0.161243	0.24309	0	0	0	2.232127	4.278221	0
17597	LOC11962	1.085058	4.274666	3.051365	1.400903	0.161243	0.24309	0	0	0	2.232127	4.278221	0
17599	LOC11962	1.085058	4.274666	3.051365	1.400903	0.161243	0.24309	0	0	0	2.232127	4.278221	0
17600	LOC11962	1.085058	4.274666	3.051365	1.400903	0.161243	0.24309	0	0	0	2.232127	4.278221	0
17601	LOC11962	1.085058	4.274666	3.051365	1.400903	0.161243	0.24309	0	0	0	2.232127	4.278221	0
17602	LOC10322	1.085058	4.274666	3.051365	1.400903	0.161243	0.24309	0	0	0	2.232127	4.278221	0
17603	LOC11962	1.085058	4.274666	3.051365	1.400903	0.161243	0.24309	0	0	0	2.232127	4.278221	0
17604	LOC10324	1.085058	4.274666	3.051365	1.400903	0.161243	0.24309	0	0	0	2.232127	4.278221	0
17606	GKN2	1.085058	4.274666	3.051365	1.400903	0.161243	0.24309	0	0	0	2.232127	4.278221	0
17607	LOC11962	1.085058	4.274666	3.051365	1.400903	0.161243	0.24309	0	0	0	2.232127	4.278221	0
17608	LOC11962	1.085058	4.274666	3.051365	1.400903	0.161243	0.24309	0	0	0	2.232127	4.278221	0

17609	LOC119628	1.085058	4.274666	3.051365	1.400903	0.161243	0.24309	0	0	0	2.232127	4.278221	0
17610	LOC119628	1.085058	4.274666	3.051365	1.400903	0.161243	0.24309	0	0	0	2.232127	4.278221	0
17611	LOC103216	1.085058	4.274666	3.051365	1.400903	0.161243	0.24309	0	0	0	2.232127	4.278221	0
17612	LOC119618	1.085058	4.274666	3.051365	1.400903	0.161243	0.24309	0	0	0	2.232127	4.278221	0
17614	LOC119618	1.085058	4.274666	3.051365	1.400903	0.161243	0.24309	0	0	0	2.232127	4.278221	0
17615	LOC119618	1.085058	4.274666	3.051365	1.400903	0.161243	0.24309	0	0	0	2.232127	4.278221	0
17617	LOC119619	1.085058	4.274666	3.051365	1.400903	0.161243	0.24309	0	0	0	2.232127	4.278221	0
17618	LOC119619	1.085058	4.274666	3.051365	1.400903	0.161243	0.24309	0	0	0	2.232127	4.278221	0
17620	UBE2QL1	1.085058	4.274666	3.051365	1.400903	0.161243	0.24309	0	0	0	2.232127	4.278221	0
17623	LOC119621	1.085058	4.274666	3.051365	1.400903	0.161243	0.24309	0	0	0	2.232127	4.278221	0
17625	LOC103234	1.085058	4.274666	3.051365	1.400903	0.161243	0.24309	0	0	0	2.232127	4.278221	0
17626	KRTAP1-3	1.085058	4.274666	3.051365	1.400903	0.161243	0.24309	0	0	0	2.232127	4.278221	0
17627	KRT40	1.085058	4.274666	3.051365	1.400903	0.161243	0.24309	0	0	0	2.232127	4.278221	0
17628	LOC103241	1.085058	4.274666	3.051365	1.400903	0.161243	0.24309	0	0	0	2.232127	4.278221	0
17630	LOC103235	1.085058	4.274666	3.051365	1.400903	0.161243	0.24309	0	0	0	2.232127	4.278221	0
17631	LOC103235	1.085058	4.274666	3.051365	1.400903	0.161243	0.24309	0	0	0	2.232127	4.278221	0
17632	LOC103247	1.085058	4.274666	3.051365	1.400903	0.161243	0.24309	0	0	0	2.232127	4.278221	0
17633	LOC103229	1.085058	4.274666	3.051365	1.400903	0.161243	0.24309	0	0	0	2.232127	4.278221	0
17634	LOC119623	1.085058	4.274666	3.051365	1.400903	0.161243	0.24309	0	0	0	2.232127	4.278221	0
10485	SHE	4.110826	4.273438	1.554913	2.748345	0.00599	0.015187	0	0.547412	0.695731	6.696381	10.69555	6.029877
9553	PLD4	6.408055	4.273287	1.437989	2.971711	0.002961	0.008241	0.542086	0	1.391463	6.696381	27.80844	2.009959
7165	HS6ST3	8.534432	4.272987	1.162476	3.675763	0.000237	0.00088	0	0.547412	2.087194	24.5534	14.97377	9.044815
8851	EYA1	8.664008	4.27237	1.353618	3.156259	0.001598	0.0048	0	0.547412	2.087194	6.696381	40.6431	2.009959
1090	KCNT1	66.42083	4.271937	0.405008	10.54779	5.20E-26	1.27E-24	6.505036	4.379295	9.044508	116.0706	136.9031	125.6224
17514	LOC119626	1.04803	4.270506	3.025108	1.411687	0.158042	0.239678	0	0	0	0	4.278221	2.009959
17515	LOC119623	1.04803	4.270506	3.025108	1.411687	0.158042	0.239678	0	0	0	0	4.278221	2.009959
17516	MS4A5	1.04803	4.270506	3.025108	1.411687	0.158042	0.239678	0	0	0	0	4.278221	2.009959
17517	AADACL2	1.04803	4.270506	3.025108	1.411687	0.158042	0.239678	0	0	0	0	4.278221	2.009959
17518	NRSN1	1.04803	4.270506	3.025108	1.411687	0.158042	0.239678	0	0	0	0	4.278221	2.009959
17519	LOC103219	1.04803	4.270506	3.025108	1.411687	0.158042	0.239678	0	0	0	0	4.278221	2.009959
17520	LOC119627	1.04803	4.270506	3.025108	1.411687	0.158042	0.239678	0	0	0	0	4.278221	2.009959
17523	LOC103218	1.04803	4.270506	3.025108	1.411687	0.158042	0.239678	0	0	0	0	4.278221	2.009959
17524	LOC119619	1.04803	4.270506	3.025108	1.411687	0.158042	0.239678	0	0	0	0	4.278221	2.009959
17525	LOC103227	1.04803	4.270506	3.025108	1.411687	0.158042	0.239678	0	0	0	0	4.278221	2.009959
17528	SPACA3	1.04803	4.270506	3.025108	1.411687	0.158042	0.239678	0	0	0	0	4.278221	2.009959
17529	LOC119622	1.04803	4.270506	3.025108	1.411687	0.158042	0.239678	0	0	0	0	4.278221	2.009959
17530	LOC119622	1.04803	4.270506	3.025108	1.411687	0.158042	0.239678	0	0	0	0	4.278221	2.009959
9137	KRT71	6.610639	4.270094	1.391125	3.069527	0.002144	0.006238	0	0	2.087194	11.16063	21.39111	5.024897
10736	LOC103235	4.303042	4.269109	1.590964	2.683348	0.007289	0.018047	0	0	1.391463	6.696381	10.69555	7.034856
10737	OLIG3	4.303042	4.269109	1.590964	2.683348	0.007289	0.018047	0	0	1.391463	6.696381	10.69555	7.034856
5	SOD2	65902.44	4.268737	0.080038	53.33394	0	0	6571.713	6666.928	6262.278	124550.5	121323.9	130039.3
6535	CHP2	10.70596	4.268182	1.087388	3.925169	8.67E-05	0.000353	0	0.547412	2.782925	15.62489	34.22577	11.05477
6884	CCNP	8.279317	4.268045	1.128206	3.783035	0.000155	0.000598	0.542086	0.547412	1.391463	15.62489	23.53022	8.039836
4293	LOC119625	15.84583	4.267913	0.827145	5.159813	2.47E-07	1.53E-06	2.168345	0.547412	2.087194	17.85702	34.22577	38.18922
18605	CUNH1orf1	1.069555	4.26734	3.459098	1.233657	0.217331	0.309867	0	0	0	0	6.417332	0
18606	ANXA10	1.069555	4.26734	3.459098	1.233657	0.217331	0.309867	0	0	0	0	6.417332	0
18608	LOC119622	1.069555	4.26734	3.459098	1.233657	0.217331	0.309867	0	0	0	0	6.417332	0
18609	LOC119624	1.069555	4.26734	3.459098	1.233657	0.217331	0.309867	0	0	0	0	6.417332	0
18612	LOC119624	1.069555	4.26734	3.459098	1.233657	0.217331	0.309867	0	0	0	0	6.417332	0
18613	LOC119625	1.069555	4.26734	3.459098	1.233657	0.217331	0.309867	0	0	0	0	6.417332	0
18614	LOC119624	1.069555	4.26734	3.459098	1.233657	0.217331	0.309867	0	0	0	0	6.417332	0
18616	LOC119625	1.069555	4.26734	3.459098	1.233657	0.217331	0.309867	0	0	0	0	6.417332	0
18617	LOC103241	1.069555	4.26734	3.459098	1.233657	0.217331	0.309867	0	0	0	0	6.417332	0
18618	LOC103248	1.069555	4.26734	3.459098	1.233657	0.217331	0.309867	0	0	0	0	6.417332	0
18619	LOC103248	1.069555	4.26734	3.459098	1.233657	0.217331	0.309867	0	0	0	0	6.417332	0
18620	LOC119626	1.069555	4.26734	3.459098	1.233657	0.217331	0.309867	0	0	0	0	6.417332	0
18623	LOC119627	1.069555	4.26734	3.459098	1.233657	0.217331	0.309867	0	0	0	0	6.417332	0
18626	LOC103247	1.069555	4.26734	3.459098	1.233657	0.217331	0.309867	0	0	0	0	6.417332	0
18628	ACTBL2	1.069555	4.26734	3.459098	1.233657	0.217331	0.309867	0	0	0	0	6.417332	0
18630	LOC119618	1.069555	4.26734	3.459098	1.233657	0.217331	0.309867	0	0	0	0	6.417332	0
18632	LOC119619	1.069555	4.26734	3.459098	1.233657	0.217331	0.309867	0	0	0	0	6.417332	0
18633	DEFA1	1.069555	4.26734	3.459098	1.233657	0.217331	0.309867	0	0	0	0	6.417332	0
18635	LOC103230	1.069555	4.26734	3.459098	1.233657	0.217331	0.309867	0	0	0	0	6.417332	0
18637	LOC119620	1.069555	4.26734	3.459098	1.233657	0.217331	0.309867	0	0	0	0	6.417332	0
18638	CCL26	1.069555	4.26734	3.459098	1.233657	0.217331	0.309867	0	0	0	0	6.417332	0
18639	LOC103227	1.069555	4.26734	3.459098	1.233657	0.217331	0.309867	0	0	0	0	6.417332	0
18640	CNFN	1.069555	4.26734	3.459098	1.233657	0.217331	0.309867	0	0	0	0	6.417332	0
18641	LOC103234	1.069555	4.26734	3.459098	1.233657	0.217331	0.309867	0	0	0	0	6.417332	0
18642	LOC119622	1.069555	4.26734	3.459098	1.233657	0.217331	0.309867	0	0	0	0	6.417332	0
18644	LOC103229	1.069555	4.26734	3.459098	1.233657	0.217331	0.309867	0	0	0	0	6.417332	0
18645	LOC119623	1.069555	4.26734	3.459098	1.233657	0.217331	0.309867	0	0	0	0	6.417332	0
6707	ABHD12B	10.86461	4.265395	1.107226	3.852325	0.000117	0.000464	0	0	3.478657	20.08914	23.53022	18.08963
4240	LOC103240	15.85362	4.264083	0.821123	5.192987	2.07E-07	1.30E-06	1.084173	1.642235	2.087194	15.62489	38.50399	36.17926
5721	C1QB	12.82197	4.263626	0.988539	4.313058	1.61E-05	7.48E-05	0	0.547412	3.478657	29.01765	27.80844	16.07967

5903	LOC10323!	12.69433	4.263499	1.012748	4.209831	2.56E-05	0.000115	0	1.094824	2.782925	29.01765	34.22577	9.044815
5680	CD28	12.76944	4.262754	0.983045	4.336273	1.45E-05	6.78E-05	0	0.547412	3.478657	24.5534	29.94755	18.08963
6456	CUNH1orf:	10.24705	4.26241	1.076972	3.957771	7.57E-05	0.000312	0	1.642235	1.391463	29.01765	21.39111	8.039836
8311	PLIN5	6.180327	4.260589	1.286966	3.31057	0.000931	0.002978	0	0.547412	1.391463	13.39276	10.69555	11.05477
6151	CHRNA2	10.15749	4.260478	1.040067	4.096349	4.20E-05	0.000181	0	1.642235	1.391463	22.32127	23.53022	12.05975
1775	ADAM28	42.83312	4.258421	0.49974	8.521278	1.58E-17	2.36E-16	4.336691	5.474118	2.782925	64.73168	96.25998	83.4133
11045	LOC10323ℇ	4.153876	4.25733	1.626513	2.617458	0.008859	0.021323	0	0.547412	0.695731	6.696381	14.97377	2.009959
9563	LIM2	5.712358	4.257315	1.434083	2.968668	0.002991	0.008314	0	1.642235	0	4.464254	17.11289	11.05477
8423	CUNH1orf:	5.972608	4.25624	1.299382	3.275589	0.001054	0.003328	0	1.094824	0.695731	11.16063	12.83466	10.04979
4454	DNAH9	25.9716	4.255381	0.844609	5.038287	4.70E-07	2.80E-06	0	1.094824	6.957314	62.49956	64.17332	21.10457
5328	CRHR2	14.20018	4.2551	0.944472	4.50527	6.63E-06	3.31E-05	1.626259	0.547412	2.087194	13.39276	53.47777	14.06971
53	CRYAB	1028.178	4.254996	0.146126	29.11875	2.08E-186	1.04E-183	81.31296	113.8617	112.7085	1908.469	1982.956	1969.76
8638	LOC11962€	5.763114	4.254807	1.322249	3.217855	0.001292	0.003975	1.084173	0.547412	0	8.928508	14.97377	9.044815
12365	PON1	4.159899	4.254463	1.826855	2.328846	0.019867	0.042711	0	0.547412	0.695731	4.464254	19.252	0
12350	LOC11962!	4.106481	4.251121	1.821294	2.334121	0.019589	0.042169	0.542086	0	0.695731	0	21.39111	2.009959
8778	LOC10322:	5.784639	4.250214	1.336633	3.179791	0.001474	0.004464	1.084173	0.547412	0	8.928508	17.11289	7.034856
8696	LOC11962(	6.275909	4.248961	1.325915	3.204551	0.001353	0.004136	0	0.547412	1.391463	17.85702	12.83466	5.024897
7866	ANKRD34C	8.649135	4.247865	1.230999	3.450747	0.000559	0.001889	0	0	2.782925	13.39276	25.66933	10.04979
9147	HSD17B13	5.754521	4.247312	1.384764	3.067175	0.002161	0.00628	0.542086	1.094824	0	4.464254	21.39111	7.034856
11673	MS4A2	4.383121	4.24693	1.71465	2.476849	0.013255	0.030187	0	0	1.391463	8.928508	14.97377	1.004979
301	LOC10321:	234.6052	4.246165	0.243174	17.46144	2.82E-68	2.49E-66	20.59928	18.612	32.00364	435.2648	494.1346	407.0167
9572	LOC11961ℇ	6.041431	4.24449	1.430427	2.967289	0.003004	0.008344	1.084173	0	0.695731	8.928508	23.53022	2.009959
8626	KRT75	5.983766	4.240753	1.31674	3.220645	0.001279	0.003942	0.542086	0.547412	0.695731	6.696381	21.39111	6.029877
3467	KCNK10	33.68207	4.236841	0.719165	5.891335	3.83E-09	2.94E-08	1.626259	0.547412	8.348776	78.12444	81.28621	32.15934
4830	HHLA1	21.2285	4.236174	0.883545	4.794518	1.63E-06	8.97E-06	0	1.094824	5.565851	46.87467	57.75599	16.07967
6607	TRPV6	12.9737	4.235886	1.086231	3.899618	9.63E-05	0.000388	0	0	4.174388	20.08914	38.50399	15.07469
8347	LOC10324:	8.639655	4.235521	1.284539	3.297307	0.000976	0.003109	0	0	2.782925	8.928508	32.08666	8.039836
2867	LOC10322:	32.2868	4.234388	0.642677	6.588669	4.44E-11	4.12E-10	0.542086	3.831883	5.565851	84.82083	57.75599	41.20416
9314	EPHA5	6.182005	4.23152	1.396572	3.029933	0.002446	0.006982	0.542086	0	1.391463	4.464254	25.66933	5.024897
11749	LOC11962!	3.702039	4.231455	1.720871	2.458904	0.013936	0.031534	0	1.094824	0	8.928508	2.139111	10.04979
6528	LOC11962:	10.44246	4.228862	1.076823	3.927167	8.60E-05	0.00035	0	0.547412	2.782925	29.01765	19.252	11.05477
7911	TPPP2	8.543649	4.224792	1.230013	3.434754	0.000593	0.001993	0	0	2.782925	22.32127	17.11289	9.044815
7817	TESPA1	8.475616	4.221188	1.219498	3.461416	0.000537	0.001827	0	0	2.782925	15.62489	21.39111	11.05477
5925	KCNAB1	11.82156	4.219872	1.005075	4.198563	2.69E-05	0.000121	1.084173	1.094824	1.391463	13.39276	44.92132	9.044815
4027	SCN2B	24.88723	4.219847	0.788378	5.35257	8.67E-08	5.72E-07	0	1.642235	6.261582	49.10679	64.17332	28.13943
8106	HPR	8.332473	4.219272	1.249049	3.377988	0.00073	0.002395	0	0.547412	2.087194	13.39276	29.94755	4.019918
5594	CMTM5	16.93725	4.218812	0.964066	4.37606	1.21E-05	5.74E-05	0.542086	0	4.87012	44.64254	38.50399	13.06473
6424	LOC10321!	14.87465	4.218565	1.062465	3.970546	7.17E-05	0.000297	0	0.547412	4.174388	35.71403	42.78221	6.029877
15070	LOC10322:	2.143558	4.217842	2.315374	1.821668	0.068505	0.12085	0	0	0.695731	11.16063	0	1.004979
5855	LOC11962€	12.12167	4.217578	0.994061	4.242777	2.21E-05	0.0001	0	1.094824	2.782925	13.39276	36.36488	19.09461
14767	FGF17	2.10653	4.215827	2.244229	1.878519	0.06031	0.108576	0	0	0.695731	8.928508	0	3.014938
15035	FAM166C	1.995446	4.215703	2.30744	1.827005	0.067699	0.11971	0	0	0.695731	2.232127	0	9.044815
11134	CCL11	4.145026	4.214446	1.623663	2.595641	0.009441	0.022544	0	0	1.391463	11.16063	4.278221	8.039836
5957	LOC11962:	12.40344	4.212768	1.007087	4.183124	2.88E-05	0.000128	0	0.547412	3.478657	20.08914	34.22577	16.07967
13891	PLA2G3	2.029279	4.211962	2.072615	2.032198	0.042134	0.080639	0	0.547412	0	4.464254	2.139111	5.024897
13892	LOC10324:	2.028391	4.211957	2.072844	2.03197	0.042157	0.080675	0.542086	0	0	4.464254	2.139111	5.024897
4765	LOC10323ℇ	23.11369	4.211939	0.871272	4.834243	1.34E-06	7.46E-06	0.542086	0.547412	6.261582	69.19594	47.06043	15.07469
4265	OVCH1	20.20295	4.211889	0.813108	5.179988	2.22E-07	1.38E-06	0	2.189647	4.174388	42.41041	51.33866	21.10457
6156	LOC10322⁄	12.462	4.209607	1.028217	4.094083	4.24E-05	0.000183	0	0.547412	3.478657	22.32127	36.36488	12.05975
7112	SEC14L3	7.880636	4.208828	1.139755	3.692748	0.000222	0.000829	0	1.094824	1.391463	15.62489	17.11289	12.05975
11183	OPALIN	4.076992	4.20732	1.627546	2.585071	0.009736	0.023145	0	0	1.391463	4.464254	8.556443	10.04979
6283	LOC11962€	9.748441	4.206163	1.042112	4.036192	5.43E-05	0.00023	0	1.642235	1.391463	17.85702	23.53022	14.06971
8369	LOC11962€	8.450633	4.205306	1.276985	3.293151	0.000991	0.003147	0	0	2.782925	8.928508	29.94755	9.044815
4314	LOC10323€	19.88974	4.204678	0.81708	5.145983	2.66E-07	1.64E-06	0	2.737059	3.478657	49.10679	44.92132	19.09461
11823	LOC10324:	3.788141	4.203645	1.720428	2.443371	0.014551	0.032719	0	1.094824	0	8.928508	10.69555	2.009959
13941	KRTAP4-3	2.012889	4.203573	2.075784	2.025054	0.042862	0.081736	0.542086	0	0	2.232127	4.278221	5.024897
13918	CUNH22or	2.066307	4.203336	2.073504	2.027166	0.042645	0.081434	0	0.547412	0	6.696381	2.139111	3.014938
13919	LIPN	2.066307	4.203336	2.073504	2.027166	0.042645	0.081434	0	0.547412	0	6.696381	2.139111	3.014938
13920	TLR8	2.066307	4.203336	2.073504	2.027166	0.042645	0.081434	0	0.547412	0	6.696381	2.139111	3.014938
13921	IGSF6	2.066307	4.203336	2.073504	2.027166	0.042645	0.081434	0	0.547412	0	6.696381	2.139111	3.014938
13922	LOC11962:	2.066307	4.203336	2.073504	2.027166	0.042645	0.081434	0	0.547412	0	6.696381	2.139111	3.014938
10828	LOC10324€	3.943329	4.202352	1.576739	2.665218	0.007694	0.01889	0	0.547412	0.695731	6.696381	10.69555	5.024897
11337	TRNAC-GC.	3.802756	4.201928	1.648453	2.549013	0.010803	0.025332	0.542086	0.547412	0	11.16063	8.556443	2.009959
6008	ADAMTS2(	12.1798	4.201513	1.009836	4.160589	3.17E-05	0.00014	0	1.094824	2.782925	33.4819	25.66933	10.04979
7530	LOC11962!	9.558789	4.201327	1.183824	3.548946	0.000387	0.001366	0	2.189647	0.695731	8.928508	38.50399	7.034856
11189	SLC39A12	4.098518	4.201118	1.625442	2.584601	0.009749	0.023164	0	0	1.391463	4.464254	10.69555	8.039836
8952	LOC11962(	5.863665	4.200902	1.341904	3.130553	0.001745	0.005182	0	1.094824	0.695731	13.39276	14.97377	5.024897
3622	CXCR4	32.925	4.200761	0.73474	5.717343	1.08E-08	7.94E-08	0	1.642235	9.044508	69.19594	51.33866	66.32864
14874	CRYBB2	2.001468	4.200727	2.259962	1.85876	0.063061	0.112712	0	0	0.695731	0	4.278221	7.034856
8876	LOC10322(	5.811134	4.199974	1.333679	3.149164	0.001637	0.004904	0	1.094824	0.695731	8.928508	17.11289	7.034856
11554	FGF16	4.039964	4.199869	1.677446	2.503729	0.012289	0.028277	0	0	1.391463	2.232127	8.556443	12.05975
14242	CELA2B	2.128055	4.198431	2.132566	1.968723	0.048985	0.091426	0	0	0.695731	8.928508	2.139111	1.004979
14244	RLN3	2.128055	4.198431	2.132566	1.968723	0.048985	0.091426	0	0	0.695731	8.928508	2.139111	1.004979
8158	LOC11962:	7.72199	4.198407	1.248643	3.362376	0.000773	0.002518	0	1.642235	0.695731	11.16063	27.80844	5.024897

5995	CR1	12.14879	4.198375	1.0077	4.166295	3.10E-05	0.000137	0	1.094824	2.782925	29.01765	29.94755	10.04979
5069	CACNB2	18.04	4.198181	0.903035	4.648971	3.34E-06	1.75E-05	0	2.189647	3.478657	40.17829	51.33866	11.05477
5882	TTLL10	12.09626	4.197403	0.993504	4.22485	2.39E-05	0.000108	0	1.094824	2.782925	24.5534	32.08666	12.05975
11531	FAM71C	4.225104	4.196087	1.673574	2.507261	0.012167	0.028051	0	0	1.391463	13.39276	8.556443	2.009959
5484	LOC10323(	14.20881	4.195144	0.949916	4.416332	1.00E-05	4.87E-05	0	1.094824	3.478657	31.24978	36.36488	13.06473
13808	LOC11962(	2.050804	4.194299	2.050115	2.045885	0.040768	0.07848	0	0.547412	0	4.464254	4.278221	3.014938
13809	LOC10324!	2.050804	4.194299	2.050115	2.045885	0.040768	0.07848	0	0.547412	0	4.464254	4.278221	3.014938
13810	NPPA	2.050804	4.194299	2.050115	2.045885	0.040768	0.07848	0	0.547412	0	4.464254	4.278221	3.014938
12894	LOC10321{	4.277635	4.1923	1.888456	2.219961	0.026421	0.054472	0	0	1.391463	17.85702	6.417332	0
5576	LOC10323;	14.17781	4.191969	0.956446	4.382862	1.17E-05	5.58E-05	0	1.094824	3.478657	26.78552	40.6431	13.06473
14859	LOC10322(	1.998274	4.190613	2.252259	1.860627	0.062797	0.112353	0	0.547412	0	0	6.417332	5.024897
49	LOC10323;	989.772	4.190044	0.139242	30.09178	6.21E-199	3.37E-196	95.4072	119.3358	93.228	1866.058	1796.853	1967.75
14125	GALNTL5	2.087832	4.187719	2.105526	1.988919	0.04671	0.087914	0	0.547412	0	6.696381	4.278221	1.004979
14100	CNR2	2.112552	4.18731	2.099494	1.994438	0.046104	0.086928	0	0	0.695731	6.696381	4.278221	1.004979
13983	LOC10324(	2.035302	4.186335	2.079185	2.01345	0.044067	0.083782	0	0.547412	0	2.232127	6.417332	3.014938
13958	LOC11962!	2.060021	4.186228	2.072889	2.019513	0.043434	0.082714	0	0	0.695731	2.232127	6.417332	3.014938
13959	LOC10323:	2.060021	4.186228	2.072889	2.019513	0.043434	0.082714	0	0	0.695731	2.232127	6.417332	3.014938
13960	LOC11962:	2.060021	4.186228	2.072889	2.019513	0.043434	0.082714	0	0	0.695731	2.232127	6.417332	3.014938
4950	GTSF1	13.62845	4.186089	0.888051	4.713793	2.43E-06	1.31E-05	0.542086	1.642235	2.087194	33.4819	29.94755	14.06971
12136	LOC11962(	628.3651	4.185661	1.75956	2.378811	0.017369	0.03805	0.542086	6.02153	189.9347	1354.901	1627.863	590.9279
8160	LOC10324{	8.345147	4.185356	1.245177	3.361253	0.000776	0.002528	0	0	2.782925	17.85702	21.39111	8.039836
7035	LOC10324{	10.20347	4.185076	1.12469	3.721091	0.000198	0.00075	0	0.547412	2.782925	15.62489	34.22577	8.039836
7202	LOC10324:	9.775564	4.184585	1.14141	3.666152	0.000246	0.000909	0	1.642235	1.391463	33.4819	17.11289	5.024897
11677	CCL20	4.178596	4.181314	1.688776	2.475943	0.013288	0.030253	0	0	1.391463	6.696381	14.97377	2.009959
14955	C1QTNF7	2.019799	4.17979	2.266172	1.844428	0.065121	0.115763	0	0.547412	0	0	8.556443	3.014938
14917	LOC10321!	2.044519	4.17972	2.258367	1.850771	0.064203	0.114406	0	0	0.695731	0	8.556443	3.014938
14918	LOC11962:	2.044519	4.17972	2.258367	1.850771	0.064203	0.114406	0	0	0.695731	0	8.556443	3.014938
14919	LOC10322(	2.044519	4.17972	2.258367	1.850771	0.064203	0.114406	0	0	0.695731	0	8.556443	3.014938
14170	LOC11961!	2.071442	4.178996	2.10848	1.981995	0.04748	0.089068	0.542086	0	0	4.464254	6.417332	1.004979
14164	LOC11962;	2.07233	4.178982	2.108259	1.982196	0.047457	0.089062	0	0.547412	0	4.464254	6.417332	1.004979
14166	SECTM1	2.07233	4.178982	2.108259	1.982196	0.047457	0.089062	0	0.547412	0	4.464254	6.417332	1.004979
532	CERS4	119.8754	4.178622	0.293191	14.25222	4.34E-46	2.17E-44	11.38381	13.13788	13.2189	229.9091	222.4675	229.1353
14132	LOC11962(	2.097049	4.178589	2.102159	1.987761	0.046838	0.088074	0	0	0.695731	4.464254	6.417332	1.004979
14133	USP29	2.097049	4.178589	2.102159	1.987761	0.046838	0.088074	0	0	0.695731	4.464254	6.417332	1.004979
14134	LOC10321!	2.097049	4.178589	2.102159	1.987761	0.046838	0.088074	0	0	0.695731	4.464254	6.417332	1.004979
14135	LOC10321⁄	2.097049	4.178589	2.102159	1.987761	0.046838	0.088074	0	0	0.695731	4.464254	6.417332	1.004979
14136	LOC10323(	2.097049	4.178589	2.102159	1.987761	0.046838	0.088074	0	0	0.695731	4.464254	6.417332	1.004979
14137	TAS2R8	2.097049	4.178589	2.102159	1.987761	0.046838	0.088074	0	0	0.695731	4.464254	6.417332	1.004979
14138	LOC11962:	2.097049	4.178589	2.102159	1.987761	0.046838	0.088074	0	0	0.695731	4.464254	6.417332	1.004979
9915	LOC11962{	6.275646	4.177607	1.450124	2.880862	0.003966	0.010633	0	0	2.087194	11.16063	21.39111	3.014938
9916	ERICH6B	6.275646	4.177607	1.450124	2.880862	0.003966	0.010633	0	0	2.087194	11.16063	21.39111	3.014938
4629	COL6A5	19.59178	4.176966	0.847328	4.929572	8.24E-07	4.73E-06	0	2.737059	3.478657	51.33892	44.92132	15.07469
5892	HIF3A	11.69952	4.17694	0.989894	4.219585	2.45E-05	0.00011	0	1.642235	2.087194	22.32127	32.08666	12.05975
6158	LOC10324(	11.97952	4.17685	1.020334	4.093609	4.25E-05	0.000183	1.084173	0	2.782925	29.01765	29.94755	9.044815
10831	ZIC4	32.29737	4.175618	1.567211	2.664363	0.007713	0.018933	0	0.547412	9.740239	62.49956	89.84265	31.15436
6510	UBXN10	9.98088	4.17487	1.06099	3.934882	8.32E-05	0.00034	0	0.547412	2.782925	20.08914	21.39111	15.07469
7509	TLX2	7.973389	4.173504	1.173878	3.555313	0.000378	0.001337	0	0.547412	2.087194	22.32127	12.83466	10.04979
7062	UPB1	10.14361	4.172349	1.125673	3.706538	0.00021	0.000791	0.542086	0	2.782925	31.24978	19.252	7.034856
14400	CD5	2.055939	4.172299	2.1472	1.943135	0.052	0.096001	0.542086	0	0	2.232127	8.556443	1.004979
14397	LOC10321(	2.056827	4.172287	2.146975	1.943333	0.051976	0.09597	0	0.547412	0	2.232127	8.556443	1.004979
14398	LOC11961{	2.056827	4.172287	2.146975	1.943333	0.051976	0.09597	0	0.547412	0	2.232127	8.556443	1.004979
14369	LOC11962!	2.081547	4.171936	2.140755	1.948815	0.051318	0.09493	0	0	0.695731	2.232127	8.556443	1.004979
14372	PAGE1	2.081547	4.171936	2.140755	1.948815	0.051318	0.09493	0	0	0.695731	2.232127	8.556443	1.004979
5617	LOC10322!	13.92075	4.170208	0.955511	4.364375	1.27E-05	6.03E-05	0	1.094824	3.478657	20.08914	42.78221	16.07967
7714	KPNA7	7.452889	4.16901	1.195024	3.488641	0.000485	0.001673	0	1.642235	0.695731	8.928508	21.39111	12.05975
5471	LOC10322(	16.09186	4.168706	0.941494	4.427754	9.52E-06	4.63E-05	0	1.094824	4.174388	29.01765	49.19955	13.06473
8173	BTBD18	8.053468	4.164196	1.241232	3.354888	0.000794	0.002583	0	0.547412	2.087194	24.5534	17.11289	4.019918
12654	LOC11962(	4.089037	4.162833	1.834045	2.269756	0.023222	0.048788	0	0	1.391463	0	17.11289	6.029877
6085	KCNA3	14.23955	4.162775	1.008326	4.128401	3.65E-05	0.00016	0	0.547412	4.174388	29.01765	40.6431	11.05477
4961	SORCS1	15.72523	4.161631	0.883802	4.708784	2.49E-06	1.34E-05	0.542086	1.094824	3.478657	31.24978	44.92132	13.06473
8683	TMIGD2	8.058603	4.160452	1.296938	3.207903	0.001337	0.004094	0.542086	0	2.087194	22.32127	21.39111	2.009959
5829	LOC10324:	11.27523	4.160365	0.977001	4.258301	2.06E-05	9.39E-05	0	2.189647	1.391463	22.32127	25.66933	16.07967
6142	OLFML1	11.806	4.156459	1.013556	4.10087	4.12E-05	0.000178	1.084173	0	2.782925	31.24978	25.66933	10.04979
5342	MAPK15	13.78337	4.15619	0.924796	4.49417	6.98E-06	3.48E-05	0.542086	0.547412	3.478657	24.5534	38.50399	15.07469
8699	EMCN	5.673755	4.156031	1.29734	3.203501	0.001358	0.004149	0.542086	0.547412	0.695731	13.39276	12.83466	6.029877
9712	LOC10322⁄	5.733196	4.154941	1.420067	2.925877	0.003435	0.0094	0	1.094824	0.695731	15.62489	14.97377	2.009959
9713	P2RY12	5.733196	4.154941	1.420067	2.925877	0.003435	0.0094	0	1.094824	0.695731	15.62489	14.97377	2.009959
9285	SOX1	6.022048	4.153132	1.368238	3.035386	0.002402	0.006878	0	0	2.087194	11.16063	12.83466	10.04979
9276	LOC10324(	5.696168	4.152404	1.366695	3.038281	0.002379	0.006819	0	1.094824	0.695731	13.39276	14.97377	4.019918
5830	LOC11961!	11.67082	4.15123	0.974948	4.257899	2.06E-05	9.41E-05	0.542086	0.547412	2.782925	17.85702	34.22577	14.06971
8243	LOC11962;	8.140623	4.150972	1.245546	3.332653	0.00086	0.002774	0	0	2.782925	15.62489	21.39111	9.044815
9502	EPB42	6.096104	4.14911	1.39061	2.983663	0.002848	0.007968	0	0	2.087194	15.62489	12.83466	6.029877
9503	LOC10324(	6.096104	4.14911	1.39061	2.983663	0.002848	0.007968	0	0	2.087194	15.62489	12.83466	6.029877
5145	HTR1B	13.33242	4.14895	0.900751	4.606101	4.10E-06	2.12E-05	0	1.642235	2.782925	26.78552	25.66933	23.11453

7937	LOC10322!	10.31173	4.148485	1.210325	3.42758	0.000609	0.00204	0	0	3.478657	24.5534	27.80844	6.029877
11614	CLEC1A	3.837843	4.147549	1.664946	2.491101	0.012735	0.029151	0	0.547412	0.695731	15.62489	2.139111	4.019918
9762	CDX2	6.133132	4.146149	1.423212	2.913234	0.003577	0.009741	0	0	2.087194	17.85702	12.83466	4.019918
5544	LOC10324:	12.84895	4.145474	0.943583	4.393333	1.12E-05	5.35E-05	0	2.737059	1.391463	15.62489	34.22577	23.11453
9080	LOC10322	7.20426	4.144434	1.341522	3.089351	0.002006	0.005873	0	2.189647	0	15.62489	21.39111	4.019918
9834	CCN6	8.288735	4.141243	1.428879	2.898245	0.003753	0.010145	0	0	2.782925	24.5534	21.39111	1.004979
6957	LOC10321{	9.440995	4.140962	1.103308	3.753225	0.000175	0.000667	0	1.642235	1.391463	15.62489	29.94755	8.039836
4537	WDFY4	18.97863	4.139989	0.829457	4.991201	6.00E-07	3.52E-06	0	2.737059	3.478657	26.78552	57.75599	23.11453
10880	LOC11962!	89.2995	4.138923	1.559683	2.653695	0.007962	0.019454	0.542086	0.547412	27.82925	165.1774	233.1631	108.5378
2417	XKR7	41.45257	4.138645	0.570963	7.248539	4.21E-13	4.63E-12	1.084173	5.474118	6.957314	78.12444	104.8164	52.25893
5997	MROH9	11.03944	4.138239	0.993493	4.165344	3.11E-05	0.000138	1.626259	1.094824	0.695731	31.24978	23.53022	8.039836
9676	NLRP5	6.012568	4.137863	1.408787	2.937181	0.003312	0.009099	0	0	2.087194	6.696381	19.252	8.039836
253	SSTR3	432.723	4.136307	0.225937	18.30733	7.23E-75	7.60E-73	38.48813	33.93953	68.87741	857.1368	748.6887	849.2077
5234	LOC11962:	24.44925	4.13623	0.906632	4.562192	5.06E-06	2.57E-05	0	0.547412	7.653045	69.19594	49.19955	20.09959
11825	LOC10322	4.051585	4.135656	1.692867	2.442989	0.014566	0.032748	0	0	1.391463	15.62489	4.278221	3.014938
12662	LOC10324(	4.104116	4.134256	1.822945	2.2679	0.023335	0.048995	0	0	1.391463	20.08914	2.139111	1.004979
4796	OTOL1	28.22808	4.133707	0.858239	4.816497	1.46E-06	8.10E-06	0	1.094824	8.348776	49.10679	87.70354	23.11453
7632	LOC10323!	9.952015	4.133075	1.175955	3.514653	0.00044	0.001534	0	0.547412	2.782925	24.5534	27.80844	4.019918
10318	SAMD3	6.123652	4.132777	1.484206	2.784504	0.005361	0.013813	0	0	2.087194	13.39276	19.252	2.009959
11676	LOC10322	4.036083	4.132461	1.668826	2.476268	0.013276	0.030229	0	0	1.391463	13.39276	6.417332	3.014938
7935	FMO1	7.285393	4.131392	1.204869	3.428914	0.000606	0.00203	0	1.642235	0.695731	8.928508	21.39111	11.05477
5167	ABCB11	15.39459	4.130145	0.898834	4.595003	4.33E-06	2.23E-05	0	1.642235	3.478657	37.94616	34.22577	15.07469
12285	LOC11962	3.664749	4.130052	1.759224	2.347655	0.018892	0.040883	0	0.547412	0.695731	0	10.69555	10.04979
2111	ADH7	36.62113	4.129945	0.530717	7.781823	7.15E-15	9.00E-14	4.336691	3.284471	4.174388	66.96381	87.70354	53.26391
5381	EIF4E1B	17.77341	4.128875	0.92277	4.474435	7.66E-06	3.79E-05	0	1.094824	4.87012	49.10679	38.50399	13.06473
7484	FOLH1B	7.374064	4.128284	1.157059	3.567912	0.00036	0.001278	0.542086	1.094824	0.695731	15.62489	19.252	7.034856
4547	FOXL2NB	19.15492	4.12777	0.829148	4.978329	6.41E-07	3.75E-06	0	2.189647	4.174388	42.41041	47.06043	19.09461
12022	LOC10322	3.583616	4.127676	1.717999	2.402607	0.016279	0.035998	0	1.094824	0	6.696381	10.69555	3.014938
8055	LOC11962	12.30931	4.125374	1.214246	3.397478	0.00068	0.002245	0	0	4.174388	35.71403	29.94755	4.019918
6171	DCC	13.89854	4.125289	1.008801	4.0893	4.33E-05	0.000186	0	0.547412	4.174388	31.24978	36.36488	11.05477
1587	CASP1	50.84169	4.123956	0.457928	9.005694	2.14E-19	3.59E-18	3.252518	7.116354	6.261582	82.5887	113.3729	92.45811
5539	CCBE1	15.66599	4.123556	0.937929	4.396448	1.10E-05	5.28E-05	0.542086	0.547412	4.174388	40.17829	38.50399	10.04979
6676	SMIM28	9.258791	4.122808	1.066262	3.866601	0.00011	0.000439	1.626259	0	1.391463	20.08914	21.39111	11.05477
7581	LOC10324:	10.03315	4.122346	1.166934	3.53263	0.000411	0.001443	0	0	3.478657	17.85702	27.80844	11.05477
7525	LOC10322	9.99612	4.122231	1.161032	3.550489	0.000385	0.001358	0	0	3.478657	15.62489	27.80844	13.06473
5423	LOC10323:	12.98476	4.121215	0.926274	4.449238	8.62E-06	4.22E-05	0	2.189647	2.087194	22.32127	34.22577	17.08465
11591	LOC11961!	3.797358	4.119047	1.650091	2.496255	0.012551	0.028783	0	0.547412	0.695731	6.696381	12.83466	2.009959
11592	LOC10321!	3.797358	4.119047	1.650091	2.496255	0.012551	0.028783	0	0.547412	0.695731	6.696381	12.83466	2.009959
9066	LOC10324:	5.446818	4.118857	1.332508	3.091055	0.001994	0.005848	1.084173	0	0.695731	11.16063	10.69555	9.044815
11722	IL27	3.989574	4.118074	1.670736	2.464827	0.013708	0.031089	0	0	1.391463	6.696381	12.83466	3.014938
12195	LOC11962(	4.042105	4.118068	1.739324	2.367626	0.017903	0.039028	0	0	1.391463	11.16063	10.69555	1.004979
6306	FNDC1	13.09238	4.116472	1.023138	4.023381	5.74E-05	0.000242	0	2.189647	2.087194	22.32127	44.92132	7.034856
11904	LOC10323:	3.974072	4.111429	1.696216	2.423882	0.015356	0.034296	0	0	1.391463	4.464254	14.97377	3.014938
12014	LOC10322!	3.937044	4.111235	1.710132	2.404046	0.016215	0.035881	0	0	1.391463	2.232127	14.97377	5.024897
13187	NEUROD1	20.89131	4.11042	1.892662	2.171766	0.029873	0.060225	0	0	6.957314	58.0353	34.22577	26.12947
3900	GRIA1	32.87046	4.108667	0.754779	5.443536	5.22E-08	3.56E-07	0	2.737059	8.348776	71.42806	85.56443	29.1444
7268	CLDN18	9.725965	4.107583	1.126889	3.645065	0.000267	0.000978	0	0.547412	2.782925	22.32127	25.66933	7.034856
3401	LOC10323:	40.24405	4.106717	0.690859	5.944362	2.78E-09	2.17E-08	0	7.663765	5.565851	91.51721	100.5382	36.17926
4145	LOC10322!	27.00574	4.104512	0.780214	5.260754	1.43E-07	9.20E-07	0.542086	1.642235	6.957314	82.5887	49.19955	21.10457
9263	DRD2	5.66747	4.1039	1.349623	3.040775	0.00236	0.006772	0.542086	0	1.391463	8.928508	17.11289	6.029877
9405	LOC11962	5.720888	4.102534	1.364755	3.00606	0.002647	0.007481	0	0.547412	1.391463	13.39276	14.97377	4.019918
9827	LOC10321(	5.495891	4.101396	1.414546	2.899444	0.003738	0.010113	1.084173	0	0.695731	8.928508	19.252	3.014938
12493	SPRR1A	3.995597	4.101142	1.7811	2.302589	0.021302	0.045327	0	0	1.391463	4.464254	17.11289	1.004979
12494	LOC11962	3.995597	4.101142	1.7811	2.302589	0.021302	0.045327	0	0	1.391463	4.464254	17.11289	1.004979
8940	SOWAHB	6.92568	4.100029	1.308072	3.134405	0.001722	0.005121	0	2.189647	0	8.928508	21.39111	9.044815
9825	LOC11962:	5.928607	4.099741	1.413729	2.899949	0.003732	0.010099	0	0	2.087194	15.62489	12.83466	5.024897
8199	TTC24	7.674536	4.098809	1.224884	3.346284	0.000819	0.002656	0.542086	0	2.087194	24.5534	12.83466	6.029877
4271	RHAG	18.78908	4.098387	0.791702	5.176682	2.26E-07	1.41E-06	0.542086	1.094824	4.87012	29.01765	47.06043	30.14938
8570	SALL3	7.504733	4.098073	1.266011	3.236997	0.001208	0.003747	0	1.094824	1.391463	24.5534	14.97377	3.014938
7678	GML	9.896656	4.098012	1.171301	3.498684	0.000468	0.001619	0	0	3.478657	22.32127	23.53022	10.04979
1561	LOC10323:	67.31045	4.095727	0.452019	9.060958	1.29E-19	2.20E-18	10.84173	2.737059	9.044508	111.6063	104.8164	164.8166
7710	BIN2	9.881154	4.095676	1.17393	3.488858	0.000485	0.001673	0	0	3.478657	20.08914	25.66933	10.04979
5498	CSF2RB	13.03614	4.093867	0.928129	4.410883	1.03E-05	4.98E-05	0.542086	1.094824	2.782925	20.08914	40.6431	13.06473
8871	LOC11961!	7.494365	4.093534	1.299223	3.150754	0.001628	0.00488	0.542086	0.547412	1.391463	20.08914	21.39111	1.004979
6031	LOC11961{	10.98046	4.085645	0.984336	4.150662	3.32E-05	0.000146	0	1.642235	2.087194	24.5534	23.53022	14.06971
4143	SCML4	20.2296	4.084844	0.776326	5.261764	1.43E-07	9.16E-07	0	2.737059	4.174388	42.41041	44.92132	27.13445
7272	SMTNL1	9.567949	4.084507	1.120917	3.643898	0.000269	0.000982	0	0.547412	2.782925	26.78552	19.252	8.039836
12342	KLF1	3.31274	4.084036	1.748278	2.336033	0.01949	0.041981	1.084173	0	0	4.464254	4.278221	10.04979
6047	MYBPC1	11.12015	4.083966	0.985172	4.145434	3.39E-05	0.000149	0	1.094824	2.782925	20.08914	25.66933	17.08465
5769	SLC7A3	10.94857	4.083755	0.9523	4.288307	1.80E-05	8.30E-05	0.542086	1.094824	2.087194	20.08914	27.80844	14.06971
5070	MYPN	21.17136	4.083424	0.878572	4.647795	3.36E-06	1.76E-05	0	1.094824	6.261582	31.24978	66.31243	22.10955
8506	WNT8B	7.374688	4.082139	1.254049	3.255166	0.001133	0.003542	0	1.094824	1.391463	8.928508	27.80844	5.024897
7104	PRAMEF1C	9.483525	4.081071	1.104551	3.694777	0.00022	0.000824	0.542086	0	2.782925	17.85702	25.66933	10.04979
15296	LOC10324:	1.902005	4.079544	2.292374	1.779615	0.075139	0.130586	0	0	0.695731	6.696381	0	4.019918

15297	LOC11962:	1.902005	4.079544	2.292374	1.779615	0.075139	0.130586	0	0	0.695731	6.696381	0	4.019918
4776	CUNH14or	16.31409	4.078908	0.844983	4.827209	1.38E-06	7.71E-06	0	2.737059	2.782925	40.17829	32.08666	20.09959
6030	LOC11962:	10.99162	4.07801	0.982132	4.152201	3.29E-05	0.000145	0.542086	1.094824	2.087194	20.08914	32.08666	10.04979
15410	LOC10323:	1.939033	4.07752	2.318982	1.758323	0.078693	0.135732	0	0	0.695731	8.928508	0	2.009959
15411	SLC26A3	1.939033	4.07752	2.318982	1.758323	0.078693	0.135732	0	0	0.695731	8.928508	0	2.009959
15412	LOC11961{	1.939033	4.07752	2.318982	1.758323	0.078693	0.135732	0	0	0.695731	8.928508	0	2.009959
8747	LOC10323!	7.80563	4.077112	1.278275	3.189541	0.001425	0.004331	0	0	2.782925	15.62489	21.39111	7.034856
10502	ABHD16B	5.851094	4.07658	1.485719	2.743843	0.006072	0.015372	0	0	2.087194	4.464254	23.53022	5.024897
9706	LOC10324	5.555962	4.073412	1.391543	2.927263	0.00342	0.009366	0.542086	0	1.391463	20.08914	4.278221	7.034856
5223	SLC2A5	14.99387	4.071546	0.891121	4.569016	4.90E-06	2.49E-05	0.542086	0.547412	4.174388	31.24978	36.36488	17.08465
14544	LOC11962!	1.861782	4.071244	2.123594	1.917148	0.055219	0.100935	0	0.547412	0	4.464254	2.139111	4.019918
5821	LOC10323!	19.44564	4.071226	0.955085	4.262685	2.02E-05	9.22E-05	0	0.547412	6.261582	40.17829	55.61688	14.06971
13229	HMGB4	18.30984	4.071135	1.88174	2.163494	0.030503	0.061299	0	0	6.261582	46.87467	40.6431	16.07967
11537	LOC10322!	3.617816	4.06981	1.624094	2.505896	0.012214	0.028145	0	0.547412	0.695731	11.16063	4.278221	5.024897
11822	KIAA1210	3.810033	4.069368	1.665434	2.443429	0.014548	0.032717	0	0	1.391463	11.16063	4.278221	6.029877
15766	LOC10322(	1.934951	4.069358	2.38324	1.70749	0.087731	0.147932	0.542086	0	0	8.928508	2.139111	0
15767	LOC10322	1.934951	4.069358	2.38324	1.70749	0.087731	0.147932	0.542086	0	0	8.928508	2.139111	0
15761	LOC10324!	1.935838	4.06934	2.382943	1.707695	0.087693	0.147908	0	0.547412	0	8.928508	2.139111	0
15762	LOC10323:	1.935838	4.06934	2.382943	1.707695	0.087693	0.147908	0	0.547412	0	8.928508	2.139111	0
6059	CHRM1	15.17387	4.069049	0.982571	4.141226	3.45E-05	0.000152	0	1.094824	4.174388	44.64254	32.08666	9.044815
11499	LOC10321{	3.549783	4.066571	1.616151	2.516207	0.011863	0.027426	0	0.547412	0.695731	4.464254	8.556443	7.034856
14062	KLK11	26.50944	4.066024	2.031543	2.001447	0.045344	0.085726	0	0	9.044508	46.87467	77.00798	26.12947
5713	LOC10322!	19.25102	4.064785	0.941117	4.319106	1.57E-05	7.29E-05	0	0.547412	6.261582	24.5534	62.03421	22.10955
23	LOC10322	1357.156	4.063381	0.113166	35.9063	2.44E-282	2.82E-279	151.7842	159.2968	148.1908	2526.768	2569.072	2587.822
6482	TEX47	15.59921	4.063163	1.029437	3.946977	7.91E-05	0.000325	0	0	5.565851	35.71403	34.22577	18.08963
11820	TLE7	3.741999	4.062923	1.662698	2.443573	0.014543	0.032709	0	0	1.391463	4.464254	8.556443	8.039836
14551	LOC11961{	1.871	4.062157	2.119748	1.916339	0.055322	0.101074	0	0	0.695731	2.232127	4.278221	4.019918
14648	LOC11962	1.897923	4.061309	2.140428	1.897428	0.057771	0.104851	0.542086	0	0	6.696381	2.139111	2.009959
14646	LOC11962{	1.89881	4.061299	2.140175	1.897648	0.057743	0.104813	0	0.547412	0	6.696381	2.139111	2.009959
11461	LOC10322	3.586811	4.060232	1.608484	2.52426	0.011594	0.026892	0	0.547412	0.695731	6.696381	8.556443	5.024897
12226	LOC11962(	3.410097	4.059915	1.719324	2.361344	0.018209	0.039594	0	1.094824	0	8.928508	6.417332	4.019918
11597	SLC5A8	3.57042	4.05806	1.626347	2.495199	0.012589	0.028858	0.542086	0	0.695731	4.464254	10.69555	5.024897
5967	LOC10324:	10.42675	4.056972	0.970913	4.17851	2.93E-05	0.000131	1.084173	1.642235	0.695731	24.5534	23.53022	11.05477
15576	PRR20G	1.945056	4.056475	2.341382	1.732513	0.083182	0.141929	0	0	0.695731	6.696381	4.278221	0
15579	LOC10323:	1.945056	4.056475	2.341382	1.732513	0.083182	0.141929	0	0	0.695731	6.696381	4.278221	0
15580	LOC10323	1.945056	4.056475	2.341382	1.732513	0.083182	0.141929	0	0	0.695731	6.696381	4.278221	0
15581	LOC11962:	1.945056	4.056475	2.341382	1.732513	0.083182	0.141929	0	0	0.695731	6.696381	4.278221	0
8163	SMC1B	7.439894	4.055687	1.207018	3.360087	0.000779	0.002538	0	0.547412	2.087194	17.85702	17.11289	7.034856
11876	KNCN	3.763524	4.054665	1.667581	2.431466	0.015038	0.03366	0	0	1.391463	4.464254	10.69555	6.029877
11622	LOC11962(	3.623839	4.053896	1.629053	2.488499	0.012828	0.029145	0	0.547412	0.695731	8.928508	8.556443	3.014938
7840	LOC10323:	7.231287	4.053709	1.173035	3.455743	0.000549	0.001861	0.542086	0.547412	1.391463	15.62489	19.252	6.029877
8031	LOC10323!	7.054573	4.053181	1.190823	3.40368	0.000665	0.002201	0.542086	1.094824	0.695731	17.85702	17.11289	5.024897
7187	LOC11962:	11.37651	4.052137	1.104187	3.669791	0.000243	0.000898	0	0.547412	3.478657	37.94616	19.252	7.034856
12146	TNFSF10	3.853083	4.051921	1.704969	2.376536	0.017476	0.038251	0	0	1.391463	11.16063	8.556443	2.009959
7764	LOC10323	9.42109	4.051857	1.166171	3.474497	0.000512	0.001753	0.542086	0	2.782925	26.78552	21.39111	5.024897
14561	LOC11962:	1.883308	4.050913	2.117421	1.913135	0.055731	0.101751	0	0.547412	0	4.464254	4.278221	2.009959
8561	SEZ6L	7.573557	4.050811	1.250861	3.238419	0.001202	0.003733	0	0	2.782925	15.62489	14.97377	12.05975
9810	NANOGNB	5.553392	4.050795	1.39557	2.902611	0.003701	0.010029	0	0.547412	1.391463	13.39276	14.97377	3.014938
14529	ADH4	1.908028	4.050631	2.110416	1.919352	0.05494	0.100494	0	0	0.695731	4.464254	4.278221	2.009959
14530	LOC11962(	1.908028	4.050631	2.110416	1.919352	0.05494	0.100494	0	0	0.695731	4.464254	4.278221	2.009959
14531	LOC10321!	1.908028	4.050631	2.110416	1.919352	0.05494	0.100494	0	0	0.695731	4.464254	4.278221	2.009959
14532	LOC11962(	1.908028	4.050631	2.110416	1.919352	0.05494	0.100494	0	0	0.695731	4.464254	4.278221	2.009959
14533	VSTM2B	1.908028	4.050631	2.110416	1.919352	0.05494	0.100494	0	0	0.695731	4.464254	4.278221	2.009959
14534	LINC02876	1.908028	4.050631	2.110416	1.919352	0.05494	0.100494	0	0	0.695731	4.464254	4.278221	2.009959
7674	RPL3L	8.938506	4.050215	1.157144	3.500181	0.000465	0.001611	0	1.642235	1.391463	15.62489	29.94755	5.024897
15422	LOC10323:	1.855497	4.050214	2.305192	1.756996	0.078918	0.136043	0	0	0.695731	0	6.417332	4.019918
122	LOC10321{	494.7688	4.048483	0.17257	23.45991	1.05E-121	2.28E-119	47.16151	62.40495	59.8329	874.9938	1026.773	897.4467
8446	SLC9C1	7.445916	4.048447	1.238099	3.269889	0.001076	0.003387	0	0.547412	2.087194	15.62489	21.39111	5.024897
5996	LOC11962(	12.94589	4.047767	0.971687	4.165709	3.10E-05	0.000138	0.542086	0.547412	3.478657	24.5534	38.50399	10.04979
6573	DCAF8L1	10.89064	4.047478	1.034922	3.910903	9.20E-05	0.000372	0	1.094824	2.782925	11.16063	34.22577	16.07967
15691	SOSTDC1	1.904833	4.046861	2.353789	1.719296	0.08556	0.144964	0	0.547412	0	4.464254	6.417332	0
15648	SMPX	1.929553	4.046363	2.345371	1.725255	0.084482	0.143501	0	0	0.695731	4.464254	6.417332	0
15649	LOC10324:	1.929553	4.046363	2.345371	1.725255	0.084482	0.143501	0	0	0.695731	4.464254	6.417332	0
15650	LOC11962(	1.929553	4.046363	2.345371	1.725255	0.084482	0.143501	0	0	0.695731	4.464254	6.417332	0
15651	TAS2R41	1.929553	4.046363	2.345371	1.725255	0.084482	0.143501	0	0	0.695731	4.464254	6.417332	0
8633	SPDYE4	9.599116	4.046048	1.256716	3.219539	0.001284	0.003954	0	0	3.478657	6.696381	36.36488	11.05477
11833	ZIM3	3.592833	4.044526	1.656869	2.441066	0.014644	0.0329	0	0.547412	0.695731	4.464254	12.83466	3.014938
7986	SEL1L2	9.555641	4.042618	1.183218	3.41663	0.000634	0.002111	0	0	3.478657	24.5534	19.252	10.04979
7166	RSPO4	15.56433	4.042602	1.099805	3.675744	0.000237	0.00088	0	0	5.565851	42.41041	36.36488	9.044815
14733	LOC11962(	1.867805	4.042509	2.146822	1.88302	0.059698	0.107681	0	0.547412	0	2.232127	6.417332	2.009959
14734	TMPRSS12	1.867805	4.042509	2.146822	1.88302	0.059698	0.107681	0	0.547412	0	2.232127	6.417332	2.009959
14736	ASB18	1.867805	4.042509	2.146822	1.88302	0.059698	0.107681	0	0.547412	0	2.232127	6.417332	2.009959
14737	LOC11962!	1.867805	4.042509	2.146822	1.88302	0.059698	0.107681	0	0.547412	0	2.232127	6.417332	2.009959
14739	LOC11962(	1.867805	4.042509	2.146822	1.88302	0.059698	0.107681	0	0.547412	0	2.232127	6.417332	2.009959

18465	TXNDC8	0.911539	4.042449	3.225694	1.253203	0.210132	0.301974	0	0	0	4.464254	0	1.004979
18466	SPRR1B	0.911539	4.042449	3.225694	1.253203	0.210132	0.301974	0	0	0	4.464254	0	1.004979
18469	PTH	0.911539	4.042449	3.225694	1.253203	0.210132	0.301974	0	0	0	4.464254	0	1.004979
18470	LOC10324	0.911539	4.042449	3.225694	1.253203	0.210132	0.301974	0	0	0	4.464254	0	1.004979
18471	LOC10324	0.911539	4.042449	3.225694	1.253203	0.210132	0.301974	0	0	0	4.464254	0	1.004979
18473	LOC10323	0.911539	4.042449	3.225694	1.253203	0.210132	0.301974	0	0	0	4.464254	0	1.004979
18475	LOC11962	0.911539	4.042449	3.225694	1.253203	0.210132	0.301974	0	0	0	4.464254	0	1.004979
18476	LOC11962	0.911539	4.042449	3.225694	1.253203	0.210132	0.301974	0	0	0	4.464254	0	1.004979
18477	LOC11962	0.911539	4.042449	3.225694	1.253203	0.210132	0.301974	0	0	0	4.464254	0	1.004979
18478	LOC11962	0.911539	4.042449	3.225694	1.253203	0.210132	0.301974	0	0	0	4.464254	0	1.004979
18479	LOC10322	0.911539	4.042449	3.225694	1.253203	0.210132	0.301974	0	0	0	4.464254	0	1.004979
18480	LOC11962	0.911539	4.042449	3.225694	1.253203	0.210132	0.301974	0	0	0	4.464254	0	1.004979
18481	TRNAT-AG	0.911539	4.042449	3.225694	1.253203	0.210132	0.301974	0	0	0	4.464254	0	1.004979
18482	LOC11962	0.911539	4.042449	3.225694	1.253203	0.210132	0.301974	0	0	0	4.464254	0	1.004979
18483	LOC11962	0.911539	4.042449	3.225694	1.253203	0.210132	0.301974	0	0	0	4.464254	0	1.004979
18484	LOC10322	0.911539	4.042449	3.225694	1.253203	0.210132	0.301974	0	0	0	4.464254	0	1.004979
18485	LOC10322	0.911539	4.042449	3.225694	1.253203	0.210132	0.301974	0	0	0	4.464254	0	1.004979
18486	LOC11961	0.911539	4.042449	3.225694	1.253203	0.210132	0.301974	0	0	0	4.464254	0	1.004979
18487	PRAP1	0.911539	4.042449	3.225694	1.253203	0.210132	0.301974	0	0	0	4.464254	0	1.004979
18489	LOC10321	0.911539	4.042449	3.225694	1.253203	0.210132	0.301974	0	0	0	4.464254	0	1.004979
18490	LOC10323	0.911539	4.042449	3.225694	1.253203	0.210132	0.301974	0	0	0	4.464254	0	1.004979
18491	LOC11962	0.911539	4.042449	3.225694	1.253203	0.210132	0.301974	0	0	0	4.464254	0	1.004979
18494	TNP2	0.911539	4.042449	3.225694	1.253203	0.210132	0.301974	0	0	0	4.464254	0	1.004979
18496	IRGC	0.911539	4.042449	3.225694	1.253203	0.210132	0.301974	0	0	0	4.464254	0	1.004979
18498	TAS2R9	0.911539	4.042449	3.225694	1.253203	0.210132	0.301974	0	0	0	4.464254	0	1.004979
14708	LOC10321	1.892525	4.042264	2.139681	1.88919	0.058866	0.106388	0	0	0.695731	2.232127	6.417332	2.009959
14709	LOC10324	1.892525	4.042264	2.139681	1.88919	0.058866	0.106388	0	0	0.695731	2.232127	6.417332	2.009959
14710	LOC11961	1.892525	4.042264	2.139681	1.88919	0.058866	0.106388	0	0	0.695731	2.232127	6.417332	2.009959
10266	DSG3	5.619021	4.041466	1.445023	2.796818	0.005161	0.013363	0	0	2.087194	4.464254	17.11289	10.04979
18527	LOC11962	0.874511	4.038968	3.240356	1.246458	0.212596	0.304922	0	0	0	2.232127	0	3.014938
18529	LOC10323	0.874511	4.038968	3.240356	1.246458	0.212596	0.304922	0	0	0	2.232127	0	3.014938
18530	FSHB	0.874511	4.038968	3.240356	1.246458	0.212596	0.304922	0	0	0	2.232127	0	3.014938
18531	LOC11962	0.874511	4.038968	3.240356	1.246458	0.212596	0.304922	0	0	0	2.232127	0	3.014938
18533	LOC11962	0.874511	4.038968	3.240356	1.246458	0.212596	0.304922	0	0	0	2.232127	0	3.014938
18534	LOC10322	0.874511	4.038968	3.240356	1.246458	0.212596	0.304922	0	0	0	2.232127	0	3.014938
18535	TNMD	0.874511	4.038968	3.240356	1.246458	0.212596	0.304922	0	0	0	2.232127	0	3.014938
18536	LOC10324	0.874511	4.038968	3.240356	1.246458	0.212596	0.304922	0	0	0	2.232127	0	3.014938
16798	LOC10322	1.873828	4.038466	2.646355	1.526049	0.126998	0.20099	0	0.547412	0	0	10.69555	0
15843	LOC10323	1.91405	4.038252	2.386915	1.691829	0.090679	0.152132	0	0	0.695731	2.232127	8.556443	0
15844	LOC11962	1.91405	4.038252	2.386915	1.691829	0.090679	0.152132	0	0	0.695731	2.232127	8.556443	0
15845	LOC11962	1.91405	4.038252	2.386915	1.691829	0.090679	0.152132	0	0	0.695731	2.232127	8.556443	0
15846	LOC10323	1.91405	4.038252	2.386915	1.691829	0.090679	0.152132	0	0	0.695731	2.232127	8.556443	0
16754	LOC11962	1.898547	4.038116	2.635922	1.531956	0.125533	0.199147	0	0	0.695731	0	10.69555	0
16755	LOC10324	1.898547	4.038116	2.635922	1.531956	0.125533	0.199147	0	0	0.695731	0	10.69555	0
16756	LOC10323	1.898547	4.038116	2.635922	1.531956	0.125533	0.199147	0	0	0.695731	0	10.69555	0
16758	LOC10322	1.898547	4.038116	2.635922	1.531956	0.125533	0.199147	0	0	0.695731	0	10.69555	0
10281	IL24	5.677575	4.038008	1.445819	2.792886	0.005224	0.013508	0	0	2.087194	6.696381	19.252	6.029877
6529	LOC10321	15.2526	4.037934	1.028289	3.926846	8.61E-05	0.00035	0	0	5.565851	22.32127	38.50399	25.12449
15673	LOC10322	1.851415	4.03789	2.34467	1.722158	0.085041	0.144249	0.542086	0	0	0	8.556443	2.009959
15603	LOC10324	1.877022	4.037701	2.335503	1.728835	0.083839	0.142829	0	0	0.695731	0	8.556443	2.009959
15604	MHC-DPB1	1.877022	4.037701	2.335503	1.728835	0.083839	0.142829	0	0	0.695731	0	8.556443	2.009959
15605	LOC10323	1.877022	4.037701	2.335503	1.728835	0.083839	0.142829	0	0	0.695731	0	8.556443	2.009959
810	LOC11962	94.92672	4.0371	0.335703	12.0258	2.60E-33	8.54E-32	9.757555	8.758589	14.61036	158.481	201.0764	176.8764
8093	GP2	9.328961	4.036229	1.193147	3.382843	0.000717	0.002357	0	0.547412	2.782925	13.39276	34.22577	5.024897
5208	KHDRBS2	16.39107	4.035204	0.882125	4.574413	4.78E-06	2.44E-05	0	1.642235	4.174388	37.94616	38.50399	16.07967
13497	LOC10323	3.859106	4.032767	1.913574	2.107453	0.035078	0.069094	0	0	1.391463	8.928508	12.83466	0
11207	LOC10323	5.417325	4.031757	1.561848	2.581401	0.00984	0.02334	0	0.547412	1.391463	0	23.53022	7.034856
9109	TCEAL5	7.5701	4.031558	1.308678	3.080635	0.002066	0.006029	0	0	2.782925	8.928508	25.66933	8.039836
8071	TRIML2	7.191431	4.029401	1.18784	3.39221	0.000693	0.002284	0.542086	0	2.087194	15.62489	12.83466	12.05975
5871	LOC11961	12.35693	4.029258	0.952581	4.229833	2.34E-05	0.000106	0.542086	1.642235	2.087194	22.32127	38.50399	9.044815
6689	GRIA4	17.26825	4.027511	1.043436	3.859852	0.000113	0.000451	0	0	6.261582	26.78552	53.47777	17.08465
6580	ARHGAP2	10.69276	4.026733	1.030182	3.908759	9.28E-05	0.000375	1.084173	0.547412	2.087194	22.32127	32.08666	6.029877
4958	LOC11962	13.58388	4.021715	0.853899	4.709827	2.48E-06	1.33E-05	0.542086	2.737059	1.391463	26.78552	29.94755	20.09959
3830	LOC11962	24.45326	4.021554	0.729173	5.515224	3.48E-08	2.42E-07	3.252518	1.094824	4.174388	37.94616	79.14709	21.10457
6454	AZGP1	12.78442	4.02127	1.015775	3.958822	7.53E-05	0.00031	0.542086	0.547412	3.478657	22.32127	42.78221	7.034856
18650	LOC11961	0.859008	4.018662	3.260153	1.232661	0.217702	0.310012	0	0	0	0	2.139111	3.014938
18651	LOC10323	0.859008	4.018662	3.260153	1.232661	0.217702	0.310012	0	0	0	0	2.139111	3.014938
18652	APCS	0.859008	4.018662	3.260153	1.232661	0.217702	0.310012	0	0	0	0	2.139111	3.014938
18653	LOC10322	0.859008	4.018662	3.260153	1.232661	0.217702	0.310012	0	0	0	0	2.139111	3.014938
18657	LOC11962	0.859008	4.018662	3.260153	1.232661	0.217702	0.310012	0	0	0	0	2.139111	3.014938
18660	LOC11962	0.859008	4.018662	3.260153	1.232661	0.217702	0.310012	0	0	0	0	2.139111	3.014938
18662	LOC11961	0.859008	4.018662	3.260153	1.232661	0.217702	0.310012	0	0	0	0	2.139111	3.014938
18667	LOC11962	0.859008	4.018662	3.260153	1.232661	0.217702	0.310012	0	0	0	0	2.139111	3.014938
18668	LOC11961	0.859008	4.018662	3.260153	1.232661	0.217702	0.310012	0	0	0	0	2.139111	3.014938

10001	LOC119622	5.179858	4.017231	1.404029	2.861215	0.00422	0.011218	1.084173	0	0.695731	17.85702	6.417332	5.024897
17747	LOC103218	0.896036	4.016615	2.921116	1.375028	0.169123	0.252436	0	0	0	2.232127	2.139111	1.004979
17748	LOC103235	0.896036	4.016615	2.921116	1.375028	0.169123	0.252436	0	0	0	2.232127	2.139111	1.004979
17749	LOC119621	0.896036	4.016615	2.921116	1.375028	0.169123	0.252436	0	0	0	2.232127	2.139111	1.004979
17750	LOC119624	0.896036	4.016615	2.921116	1.375028	0.169123	0.252436	0	0	0	2.232127	2.139111	1.004979
17751	KERA	0.896036	4.016615	2.921116	1.375028	0.169123	0.252436	0	0	0	2.232127	2.139111	1.004979
17753	CCIN	0.896036	4.016615	2.921116	1.375028	0.169123	0.252436	0	0	0	2.232127	2.139111	1.004979
17754	LOC103246	0.896036	4.016615	2.921116	1.375028	0.169123	0.252436	0	0	0	2.232127	2.139111	1.004979
17755	LOC119624	0.896036	4.016615	2.921116	1.375028	0.169123	0.252436	0	0	0	2.232127	2.139111	1.004979
17757	LOC119624	0.896036	4.016615	2.921116	1.375028	0.169123	0.252436	0	0	0	2.232127	2.139111	1.004979
17759	LOC119625	0.896036	4.016615	2.921116	1.375028	0.169123	0.252436	0	0	0	2.232127	2.139111	1.004979
17761	LOC103241	0.896036	4.016615	2.921116	1.375028	0.169123	0.252436	0	0	0	2.232127	2.139111	1.004979
17762	LOC119625	0.896036	4.016615	2.921116	1.375028	0.169123	0.252436	0	0	0	2.232127	2.139111	1.004979
17763	LOC119625	0.896036	4.016615	2.921116	1.375028	0.169123	0.252436	0	0	0	2.232127	2.139111	1.004979
17765	SLC5A7	0.896036	4.016615	2.921116	1.375028	0.169123	0.252436	0	0	0	2.232127	2.139111	1.004979
17767	LOC103248	0.896036	4.016615	2.921116	1.375028	0.169123	0.252436	0	0	0	2.232127	2.139111	1.004979
17768	LOC119626	0.896036	4.016615	2.921116	1.375028	0.169123	0.252436	0	0	0	2.232127	2.139111	1.004979
17770	LOC103220	0.896036	4.016615	2.921116	1.375028	0.169123	0.252436	0	0	0	2.232127	2.139111	1.004979
17771	LOC119627	0.896036	4.016615	2.921116	1.375028	0.169123	0.252436	0	0	0	2.232127	2.139111	1.004979
17772	LOC103214	0.896036	4.016615	2.921116	1.375028	0.169123	0.252436	0	0	0	2.232127	2.139111	1.004979
17773	CCL22	0.896036	4.016615	2.921116	1.375028	0.169123	0.252436	0	0	0	2.232127	2.139111	1.004979
17774	LOC119628	0.896036	4.016615	2.921116	1.375028	0.169123	0.252436	0	0	0	2.232127	2.139111	1.004979
17775	PNLIPRP3	0.896036	4.016615	2.921116	1.375028	0.169123	0.252436	0	0	0	2.232127	2.139111	1.004979
17778	LOC119628	0.896036	4.016615	2.921116	1.375028	0.169123	0.252436	0	0	0	2.232127	2.139111	1.004979
17779	LOC119618	0.896036	4.016615	2.921116	1.375028	0.169123	0.252436	0	0	0	2.232127	2.139111	1.004979
17780	GTSF1L	0.896036	4.016615	2.921116	1.375028	0.169123	0.252436	0	0	0	2.232127	2.139111	1.004979
17781	DEFB115	0.896036	4.016615	2.921116	1.375028	0.169123	0.252436	0	0	0	2.232127	2.139111	1.004979
17782	SMIM18	0.896036	4.016615	2.921116	1.375028	0.169123	0.252436	0	0	0	2.232127	2.139111	1.004979
17783	LOC119618	0.896036	4.016615	2.921116	1.375028	0.169123	0.252436	0	0	0	2.232127	2.139111	1.004979
17784	LOC119619	0.896036	4.016615	2.921116	1.375028	0.169123	0.252436	0	0	0	2.232127	2.139111	1.004979
17786	LOC103246	0.896036	4.016615	2.921116	1.375028	0.169123	0.252436	0	0	0	2.232127	2.139111	1.004979
17787	LOC119619	0.896036	4.016615	2.921116	1.375028	0.169123	0.252436	0	0	0	2.232127	2.139111	1.004979
17788	LOC119619	0.896036	4.016615	2.921116	1.375028	0.169123	0.252436	0	0	0	2.232127	2.139111	1.004979
17789	LOC103230	0.896036	4.016615	2.921116	1.375028	0.169123	0.252436	0	0	0	2.232127	2.139111	1.004979
17790	LOC103230	0.896036	4.016615	2.921116	1.375028	0.169123	0.252436	0	0	0	2.232127	2.139111	1.004979
17791	LOC103247	0.896036	4.016615	2.921116	1.375028	0.169123	0.252436	0	0	0	2.232127	2.139111	1.004979
17792	LOC119620	0.896036	4.016615	2.921116	1.375028	0.169123	0.252436	0	0	0	2.232127	2.139111	1.004979
17793	LOC119620	0.896036	4.016615	2.921116	1.375028	0.169123	0.252436	0	0	0	2.232127	2.139111	1.004979
17794	LOC103242	0.896036	4.016615	2.921116	1.375028	0.169123	0.252436	0	0	0	2.232127	2.139111	1.004979
17795	LOC103231	0.896036	4.016615	2.921116	1.375028	0.169123	0.252436	0	0	0	2.232127	2.139111	1.004979
17796	LOC119620	0.896036	4.016615	2.921116	1.375028	0.169123	0.252436	0	0	0	2.232127	2.139111	1.004979
17797	LOC119620	0.896036	4.016615	2.921116	1.375028	0.169123	0.252436	0	0	0	2.232127	2.139111	1.004979
17799	CAPZA3	0.896036	4.016615	2.921116	1.375028	0.169123	0.252436	0	0	0	2.232127	2.139111	1.004979
17800	LOC119621	0.896036	4.016615	2.921116	1.375028	0.169123	0.252436	0	0	0	2.232127	2.139111	1.004979
17804	KRTAP29-1	0.896036	4.016615	2.921116	1.375028	0.169123	0.252436	0	0	0	2.232127	2.139111	1.004979
17805	LOC103214	0.896036	4.016615	2.921116	1.375028	0.169123	0.252436	0	0	0	2.232127	2.139111	1.004979
17806	LOC119622	0.896036	4.016615	2.921116	1.375028	0.169123	0.252436	0	0	0	2.232127	2.139111	1.004979
17807	LOC103247	0.896036	4.016615	2.921116	1.375028	0.169123	0.252436	0	0	0	2.232127	2.139111	1.004979
17808	LOC103247	0.896036	4.016615	2.921116	1.375028	0.169123	0.252436	0	0	0	2.232127	2.139111	1.004979
17811	LOC119623	0.896036	4.016615	2.921116	1.375028	0.169123	0.252436	0	0	0	2.232127	2.139111	1.004979
11144	MAGEA8	4.995439	4.016173	1.548798	2.59309	0.009512	0.022691	0	1.642235	0	15.62489	10.69555	2.009959
5066	CNTNAP5	24.14391	4.014179	0.86296	4.651642	3.29E-06	1.73E-05	0	1.094824	7.653045	42.41041	70.59065	23.11453
19681	LOC119623	0.837483	4.012891	3.739449	1.073124	0.283216	0.382566	0	0	0	0	0	5.024897
14060	LOC119625	3.812598	4.012671	2.004486	2.001845	0.045301	0.085657	0	0	1.391463	2.232127	19.252	0
3707	LOC119626	36.81682	4.012263	0.712117	5.634278	1.76E-08	1.26E-07	1.084173	1.094824	11.1317	91.51721	74.86887	41.20416
8186	FAM163B	9.388144	4.011612	1.19709	3.351135	0.000805	0.002614	0	0	3.478657	24.5534	19.252	9.044815
5151	CDC20B	16.01859	4.011473	0.871656	4.60213	4.18E-06	2.16E-05	0.542086	1.094824	4.174388	17.85702	51.33866	21.10457
8961	LOC119620	7.070701	4.008615	1.281329	3.128483	0.001757	0.005213	0	1.094824	1.391463	13.39276	23.53022	3.014938
11606	LOC103234	5.437538	4.005964	1.607385	2.492225	0.012695	0.029078	0.542086	0	1.391463	17.85702	12.83466	0
9815	RXRG	5.150628	4.004515	1.38019	2.901422	0.003715	0.010062	0	1.094824	0.695731	13.39276	10.69555	5.024897
5105	VIL1	24.03408	4.004408	0.865685	4.62571	3.73E-06	1.94E-05	0.542086	0.547412	7.653045	44.64254	72.72976	18.08963
10322	LOC119627	5.572089	4.002138	1.438019	2.78309	0.005384	0.013866	0	0	2.087194	15.62489	10.69555	5.024897
3618	RGS8	24.751	4.001972	0.699602	5.720355	1.06E-08	7.81E-08	1.626259	1.094824	6.261582	58.0353	51.33866	30.14938
18703	C1QA	0.880533	4.001685	3.262894	1.226422	0.22004	0.312237	0	0	0	0	4.278221	1.004979
18704	LOC119623	0.880533	4.001685	3.262894	1.226422	0.22004	0.312237	0	0	0	0	4.278221	1.004979
18705	LOC119624	0.880533	4.001685	3.262894	1.226422	0.22004	0.312237	0	0	0	0	4.278221	1.004979
18706	LOC119624	0.880533	4.001685	3.262894	1.226422	0.22004	0.312237	0	0	0	0	4.278221	1.004979
18707	LOC119624	0.880533	4.001685	3.262894	1.226422	0.22004	0.312237	0	0	0	0	4.278221	1.004979
18708	GJE1	0.880533	4.001685	3.262894	1.226422	0.22004	0.312237	0	0	0	0	4.278221	1.004979
18709	LOC103240	0.880533	4.001685	3.262894	1.226422	0.22004	0.312237	0	0	0	0	4.278221	1.004979
18710	LOC119626	0.880533	4.001685	3.262894	1.226422	0.22004	0.312237	0	0	0	0	4.278221	1.004979
18711	CCK	0.880533	4.001685	3.262894	1.226422	0.22004	0.312237	0	0	0	0	4.278221	1.004979
18713	LOC119626	0.880533	4.001685	3.262894	1.226422	0.22004	0.312237	0	0	0	0	4.278221	1.004979
18714	LOC103219	0.880533	4.001685	3.262894	1.226422	0.22004	0.312237	0	0	0	0	4.278221	1.004979

18715	LOC11962‹	0.880533	4.001685	3.262894	1.226422	0.22004	0.312237	0	0	0	0	4.278221	1.004979
18716	LOC10324!	0.880533	4.001685	3.262894	1.226422	0.22004	0.312237	0	0	0	0	4.278221	1.004979
18717	LOC11962‹	0.880533	4.001685	3.262894	1.226422	0.22004	0.312237	0	0	0	0	4.278221	1.004979
18718	LOC11961‹	0.880533	4.001685	3.262894	1.226422	0.22004	0.312237	0	0	0	0	4.278221	1.004979
18719	CUNH1orf1	0.880533	4.001685	3.262894	1.226422	0.22004	0.312237	0	0	0	0	4.278221	1.004979
18720	LOC10323(	0.880533	4.001685	3.262894	1.226422	0.22004	0.312237	0	0	0	0	4.278221	1.004979
18721	LOC10323(	0.880533	4.001685	3.262894	1.226422	0.22004	0.312237	0	0	0	0	4.278221	1.004979
18722	LOC11962(	0.880533	4.001685	3.262894	1.226422	0.22004	0.312237	0	0	0	0	4.278221	1.004979
18723	LOC10324‹	0.880533	4.001685	3.262894	1.226422	0.22004	0.312237	0	0	0	0	4.278221	1.004979
18724	LOC10323‹	0.880533	4.001685	3.262894	1.226422	0.22004	0.312237	0	0	0	0	4.278221	1.004979
18725	LOC11962(	0.880533	4.001685	3.262894	1.226422	0.22004	0.312237	0	0	0	0	4.278221	1.004979
18728	LOC11962‹	0.880533	4.001685	3.262894	1.226422	0.22004	0.312237	0	0	0	0	4.278221	1.004979
18729	LOC10321‹	0.880533	4.001685	3.262894	1.226422	0.22004	0.312237	0	0	0	0	4.278221	1.004979
18730	LOC11962‹	0.880533	4.001685	3.262894	1.226422	0.22004	0.312237	0	0	0	0	4.278221	1.004979
18731	LOC10323‹	0.880533	4.001685	3.262894	1.226422	0.22004	0.312237	0	0	0	0	4.278221	1.004979
18732	LOC10321‹	0.880533	4.001685	3.262894	1.226422	0.22004	0.312237	0	0	0	0	4.278221	1.004979
18733	LOC11962‹	0.880533	4.001685	3.262894	1.226422	0.22004	0.312237	0	0	0	0	4.278221	1.004979
18734	LOC11962‹	0.880533	4.001685	3.262894	1.226422	0.22004	0.312237	0	0	0	0	4.278221	1.004979
18735	LOC11962‹	0.880533	4.001685	3.262894	1.226422	0.22004	0.312237	0	0	0	0	4.278221	1.004979
6858	LOC10323‹	10.89943	3.999777	1.054518	3.792989	0.000149	0.000577	0	0.547412	3.478657	26.78552	23.53022	11.05477
6839	LOC10322‹	10.71324	3.999712	1.052015	3.801953	0.000144	0.000558	0	1.094824	2.782925	24.5534	27.80844	8.039836
9889	LOC10323(	5.348867	3.999325	1.385612	2.886324	0.003898	0.010477	0	0.547412	1.391463	11.16063	14.97377	4.019918
4261	GAS7	24.81866	3.999111	0.771738	5.181957	2.20E-07	1.37E-06	0	2.737059	6.261582	40.17829	70.59065	29.1444
6750	CYP2G2	10.8469	3.99904	1.042297	3.836758	0.000125	0.000491	0	0.547412	3.478657	22.32127	25.66933	13.06473
7022	LOC11962‹	10.93646	3.998603	1.073151	3.726041	0.000195	0.000736	0	0.547412	3.478657	29.01765	23.53022	9.044815
3512	AGMO	21.45422	3.997919	0.683895	5.845805	5.04E-09	3.82E-08	4.336691	1.094824	2.087194	44.64254	36.36488	40.19918
10108	GDF2	5.067092	3.997861	1.409222	2.836928	0.004555	0.011979	0	1.094824	0.695731	4.464254	17.11289	7.034856
12793	IDO1	3.19955	3.997408	1.783739	2.241027	0.025024	0.052003	0	1.094824	0	8.928508	2.139111	7.034856
6457	LOC11962‹	12.70842	3.996172	1.009815	3.957332	7.58E-05	0.000312	0	0.547412	4.174388	20.08914	36.36488	15.07469
6211	TMEM45B	10.02525	3.994919	0.981882	4.068636	4.73E-05	0.000202	0.542086	1.642235	1.391463	26.78552	10.69555	19.09461
6044	CALN1	22.40783	3.994603	0.963409	4.146322	3.38E-05	0.000149	0.542086	0	7.653045	46.87467	66.31243	13.06473
7212	IGSF22	8.694125	3.993107	1.090054	3.663221	0.000249	0.000918	0	1.094824	2.087194	13.39276	23.53022	12.05975
6987	LOC10324‹	10.83653	3.992595	1.067748	3.739267	0.000185	0.000702	0.542086	0	3.478657	17.85702	32.08666	11.05477
10156	TACR2	5.317862	3.991926	1.413396	2.824352	0.004738	0.012402	0	0.547412	1.391463	6.696381	19.252	4.019918
12094	PLA2G2E	3.590005	3.991369	1.67116	2.388382	0.016923	0.0372	0	0	1.391463	6.696381	6.417332	7.034856
2673	CCN5	31.94785	3.990082	0.580533	6.873134	6.28E-12	6.25E-11	3.252518	2.189647	6.261582	40.17829	68.45154	71.35354
50	CLDN10	927.7291	3.98984	0.133369	29.91569	1.23E-196	6.54E-194	124.1378	101.2712	103.664	1763.38	1726.262	1747.659
7610	PLEKHB1	8.805209	3.989541	1.132528	3.522687	0.000427	0.001492	0	1.094824	2.087194	20.08914	23.53022	6.029877
9322	VWA2	6.766976	3.988255	1.318252	3.025412	0.002483	0.007081	0	1.642235	0.695731	24.5534	10.69555	3.014938
7464	SIDT1	8.752678	3.988082	1.115285	3.57584	0.000349	0.001243	0	1.094824	2.087194	15.62489	25.66933	8.039836
5488	LOC11962‹	13.45723	3.987786	0.903335	4.414516	1.01E-05	4.90E-05	1.626259	1.642235	1.391463	44.64254	21.39111	10.04979
17	IGFBP7	5153.577	3.987536	0.089754	44.42726	0	0	627.736	623.5021	581.6314	9841.448	9384.278	9862.869
12072	LOC10323‹	57.4747	3.987086	1.665825	2.393461	0.01669	0.036755	0	1.094824	19.48048	93.74933	156.1551	74.36848
8059	SIGLEC14	9.034454	3.98558	1.173419	3.396554	0.000682	0.002251	0	0.547412	2.782925	22.32127	23.53022	5.024897
25	SMIM10L2	2251.156	3.984997	0.112429	35.44466	3.51E-275	3.73E-272	279.7166	272.0637	250.4633	4066.935	4010.833	4626.925
11865	LOC10324‹	3.382286	3.984535	1.637284	2.433625	0.014948	0.033494	0	0.547412	0.695731	4.464254	8.556443	6.029877
3422	EPHB1	38.49717	3.982482	0.672419	5.922623	3.17E-09	2.46E-08	0.542086	4.379295	9.044508	75.89232	106.9555	34.1693
6157	ELAVL4	12.32718	3.982475	0.97282	4.093743	4.24E-05	0.000183	0	1.094824	3.478657	20.08914	34.22577	15.07469
12142	CLLU1-AS1	3.611531	3.982356	1.674527	2.378197	0.017398	0.038089	0	0	1.391463	6.696381	8.556443	5.024897
12143	LOC11962‹	3.611531	3.982356	1.674527	2.378197	0.017398	0.038089	0	0	1.391463	6.696381	8.556443	5.024897
3549	PIGR	20.02719	3.982092	0.686038	5.804475	6.46E-09	4.84E-08	1.626259	2.737059	2.782925	37.94616	44.92132	30.14938
5462	LRRC74A	15.81789	3.981787	0.89798	4.434158	9.24E-06	4.50E-05	1.626259	0	4.174388	31.24978	42.78221	15.07469
5519	DRAXIN	19.67018	3.981543	0.904031	4.404209	1.06E-05	5.11E-05	0	1.642235	5.565851	35.71403	62.03421	13.06473
4144	PREX2	16.89567	3.980779	0.756591	5.261465	1.43E-07	9.17E-07	1.626259	1.642235	2.782925	35.71403	38.50399	21.10457
12170	MEP1A	3.470957	3.980344	1.67816	2.37185	0.017699	0.038664	0.542086	0	0.695731	11.16063	6.417332	2.009959
8029	LOC11962(	8.763836	3.97979	1.169193	3.403877	0.000664	0.0022	0.542086	0.547412	2.087194	11.16063	34.22577	4.019918
8351	ZSWIM2	7.030845	3.979405	1.207126	3.296596	0.000979	0.003115	0	0.547412	2.087194	13.39276	17.11289	9.044815
13765	LOC11961‹	165.3533	3.979188	1.937393	2.053888	0.039987	0.077228	0	2.189647	57.04997	357.1403	464.187	111.5527
913	TMEM151/	81.99758	3.978788	0.348596	11.41376	3.57E-30	1.04E-28	8.673382	9.853413	11.1317	149.5525	136.9031	175.8714
6910	SIGLEC16	9.946678	3.978321	1.054256	3.773582	0.000161	0.000619	0.542086	2.189647	0.695731	24.5534	25.66933	6.029877
6571	LOC11962‹	10.13712	3.977701	1.016905	3.911576	9.17E-05	0.000371	1.626259	0.547412	1.391463	24.5534	25.66933	7.034856
10280	FAT3	38.80927	3.977406	1.423782	2.79355	0.005213	0.013482	0	2.189647	11.82743	89.28508	98.39909	31.15436
12296	P2RX2	3.648559	3.977194	1.696013	2.345026	0.019026	0.041134	0	0	1.391463	8.928508	8.556443	3.014938
13198	FFAR1	3.264126	3.975752	1.832662	2.169386	0.030053	0.060537	0	1.094824	0	8.928508	8.556443	1.004979
12289	RNASE2	3.596028	3.975231	1.694037	2.346602	0.018945	0.040985	0	0	1.391463	4.464254	10.69555	5.024897
11119	EN1	5.457547	3.975047	1.529799	2.598411	0.009366	0.022393	0	0	2.087194	2.232127	21.39111	7.034856
7872	LOC11962‹	13.00224	3.974403	1.152658	3.448032	0.000565	0.001907	0	0	4.87012	22.32127	42.78221	8.039836
2732	LOC11961‹	35.87802	3.974305	0.584175	6.803273	1.02E-11	9.95E-11	1.626259	2.737059	9.044508	58.0353	68.45154	75.37346
8448	LOC10324‹	6.896294	3.972314	1.214937	3.269563	0.001077	0.00339	0.542086	0.547412	1.391463	15.62489	19.252	4.019918
2707	GSDMC	49.22808	3.971709	0.581787	6.826742	8.69E-12	8.53E-11	2.168345	6.568942	9.044508	80.35657	154.016	43.21412
12156	LOC11962‹	3.456342	3.971654	1.672532	2.374636	0.017566	0.038414	0	0.547412	0.695731	8.928508	8.556443	2.009959
12157	DKK4	3.456342	3.971654	1.672532	2.374636	0.017566	0.038414	0	0.547412	0.695731	8.928508	8.556443	2.009959
13775	ZNF677	19.10782	3.971524	1.935339	2.052107	0.040159	0.077507	0	0	6.957314	42.41041	49.19955	16.07967
7301	CADPS	8.586864	3.97126	1.092436	3.635233	0.000278	0.001011	1.084173	0	2.087194	22.32127	14.97377	11.05477

12806	DBX2	3.685587	3.971174	1.774733	2.237617	0.025246	0.052408	0	0	1.391463	11.16063	8.556443	1.004979
12215	CD36	3.241713	3.971009	1.679385	2.364562	0.018051	0.039288	0.542086	0.547412	0	8.928508	6.417332	3.014938
7262	SMIM22	10.73796	3.967798	1.087284	3.649276	0.000263	0.000963	0	0.547412	3.478657	24.5534	27.80844	8.039836
12333	CEACAM5	3.388309	3.966815	1.696869	2.337726	0.019401	0.041822	0	0.547412	0.695731	2.232127	12.83466	4.019918
12223	GPR143	3.440839	3.966568	1.679278	2.362067	0.018173	0.039527	0	0.547412	0.695731	6.696381	10.69555	2.009959
5793	LOC10323ɛ	11.9812	3.965794	0.927106	4.277605	1.89E-05	8.67E-05	1.084173	0.547412	2.782925	22.32127	32.08666	13.06473
7281	LOC11962ɛ	8.328389	3.964001	1.089285	3.639085	0.000274	0.000999	0	1.642235	1.391463	15.62489	19.252	12.05975
8108	LOC10323ɛ	8.89194	3.962243	1.173286	3.377049	0.000733	0.002402	0	0.547412	2.782925	29.01765	14.97377	6.029877
6768	LOC11962ɛ	16.49666	3.962198	1.034865	3.828709	0.000129	0.000506	0	0	6.261582	24.5534	47.06043	21.10457
7752	LOC11962ɛ	8.839409	3.961813	1.139252	3.477557	0.000506	0.001735	0	0.547412	2.782925	24.5534	17.11289	8.039836
7338	SLC15A1	9.595557	3.961688	1.094559	3.619439	0.000295	0.00107	1.084173	2.189647	0	29.01765	19.252	6.029877
5061	LOC11962ɛ	16.72135	3.960606	0.850886	4.654687	3.24E-06	1.70E-05	0	3.284471	2.782925	31.24978	44.92132	18.08963
9773	LOC10322ɛ	5.137432	3.960068	1.360731	2.910251	0.003611	0.009824	0.542086	0	1.391463	11.16063	10.69555	7.034856
12929	LOC10324ɛ	3.654581	3.959814	1.786789	2.216162	0.02668	0.054857	0	0	1.391463	6.696381	12.83466	1.004979
10445	LOC10324ɛ	5.324514	3.959467	1.436522	2.756287	0.005846	0.01488	0	0	2.087194	13.39276	6.417332	10.04979
9677	ABCG8	7.30314	3.958748	1.34782	2.937149	0.003312	0.009099	0	0	2.782925	15.62489	21.39111	4.019918
9678	OTC	7.30314	3.958748	1.34782	2.937149	0.003312	0.009099	0	0	2.782925	15.62489	21.39111	4.019918
9862	LOC11962ɛ	5.085789	3.957545	1.369125	2.890565	0.003846	0.010366	0	0.547412	1.391463	6.696381	12.83466	9.044815
76	SLC43A2	2853.447	3.955873	0.150102	26.35453	4.55E-153	1.59E-150	392.4705	268.2318	377.0864	5167.374	4834.39	6081.131
7690	MAT1A	8.585182	3.95475	1.131109	3.496347	0.000472	0.001631	0	1.094824	2.087194	15.62489	25.66933	7.034856
7002	MARVELD3	12.27679	3.953536	1.059494	3.731533	0.00019	0.000723	0	1.094824	3.478657	24.5534	38.50399	6.029877
6868	NME8	14.34603	3.952177	1.042949	3.789425	0.000151	0.000584	0	0.547412	4.87012	24.5534	47.06043	9.044815
15093	LOC11961ɛ	32.26808	3.95101	2.174149	1.817267	0.069176	0.121848	0	0	11.82743	60.26743	98.39909	23.11453
140	FLRT2	412.2327	3.950466	0.174093	22.69169	5.41E-114	1.03E-111	46.61943	58.02565	45.22254	787.9408	748.6887	786.8989
4471	SELL	22.74822	3.950133	0.785588	5.028252	4.95E-07	2.94E-06	0.542086	1.094824	6.957314	53.57105	49.19955	25.12449
5670	SLC16A4	11.80254	3.949726	0.910196	4.339423	1.43E-05	6.70E-05	0.542086	1.094824	2.782925	26.78552	23.53022	16.07967
10413	LOC11962ɛ	5.315034	3.94904	1.428998	2.763503	0.005718	0.0146	0	0	2.087194	8.928508	12.83466	8.039836
5575	TFAP2B	19.64645	3.948932	0.900969	4.382985	1.17E-05	5.58E-05	0	0.547412	6.957314	46.87467	36.36488	27.13445
10696	SPATC1	5.404592	3.947655	1.465967	2.692867	0.007084	0.017606	0	0	2.087194	15.62489	10.69555	4.019918
10284	CDHR3	5.196873	3.947249	1.413736	2.792069	0.005237	0.013538	0	0.547412	1.391463	13.39276	12.83466	3.014938
10488	LOC11962ɛ	5.352062	3.947194	1.436689	2.747423	0.006007	0.015225	0	0	2.087194	11.16063	12.83466	6.029877
10068	LOC10324ɛ	5.144342	3.946559	1.386388	2.846648	0.004418	0.011667	0	0.547412	1.391463	8.928508	14.97377	5.024897
10000	LOC11961ɛ	7.182576	3.944845	1.37848	2.861736	0.004213	0.011201	0	0	2.782925	4.464254	27.80844	8.039836
10321	LOC10324ɛ	5.18137	3.943584	1.416966	2.783119	0.005384	0.013866	0	0.547412	1.391463	11.16063	14.97377	3.014938
8592	LOC11962ɛ	6.789292	3.940771	1.219471	3.231541	0.001231	0.00381	0	0.547412	2.087194	8.928508	17.11289	12.05975
4754	PTPRD	22.37636	3.938801	0.813688	4.840677	1.29E-06	7.23E-06	2.168345	0	6.261582	49.10679	55.61688	21.10457
9116	SEMA3E	6.96841	3.93834	1.279139	3.078899	0.002078	0.006059	0	0.547412	2.087194	22.32127	12.83466	4.019918
8282	ZAN	68.97099	3.937969	1.186373	3.319335	0.000902	0.002896	1.626259	1.642235	22.2634	156.2489	164.7115	67.33362
4468	MYH3	22.36866	3.937347	0.782588	5.031185	4.87E-07	2.90E-06	1.084173	1.094824	6.261582	44.64254	62.03421	19.09461
11158	SMIM9	5.410615	3.936968	1.520063	2.590004	0.009597	0.022867	0	0	2.087194	13.39276	14.97377	2.009959
10813	ADAD1	5.284028	3.936136	1.475462	2.66773	0.007637	0.018775	0	0	2.087194	4.464254	17.11289	8.039836
7144	ARRDC5	8.38925	3.93254	1.067879	3.68257	0.000231	0.000859	0.542086	0.547412	2.087194	17.85702	19.252	10.04979
5586	OPCML	13.3474	3.932479	0.898053	4.378896	1.19E-05	5.68E-05	0.542086	1.642235	2.782925	24.5534	38.50399	12.05975
14390	NXPH1	3.608073	3.931744	2.021158	1.945293	0.05174	0.095585	0	0	1.391463	0	19.252	1.004979
7870	LOC10324ɛ	10.70412	3.930879	1.139824	3.44867	0.000563	0.001903	0	0	4.174388	22.32127	25.66933	12.05975
14084	RARRES2	18.60533	3.929414	1.967551	1.997109	0.045813	0.086477	0	0	6.957314	42.41041	49.19955	13.06473
1890	CRTAC1	42.88463	3.928386	0.478067	8.217229	2.08E-16	2.93E-15	3.794605	6.02153	6.261582	82.5887	64.17332	94.46807
7769	LOC10322ɛ	8.619382	3.927789	1.131321	3.471862	0.000517	0.001769	0	0.547412	2.782925	20.08914	19.252	9.044815
16032	LOC10324ɛ	1.734508	3.926438	2.364575	1.660526	0.096809	0.160533	0	0	0.695731	6.696381	0	3.014938
9657	IRS4	6.943427	3.925967	1.333132	2.94492	0.00323	0.008893	0	0.547412	2.087194	15.62489	21.39111	2.009959
15349	CTXN3	1.657258	3.925524	2.216917	1.770713	0.076608	0.132688	0	0.547412	0	2.232127	2.139111	5.024897
16248	LOC11962ɛ	1.771536	3.925249	2.426443	1.617697	0.105728	0.172992	0	0	0.695731	8.928508	0	1.004979
7249	SLC26A8	8.16514	3.924903	1.074283	3.653509	0.000259	0.000949	1.084173	0.547412	1.391463	13.39276	23.53022	9.044815
8056	LYVE1	8.624517	3.922858	1.154765	3.397106	0.000681	0.002247	0.542086	0	2.782925	17.85702	23.53022	7.034856
9266	TRDN	6.838365	3.921316	1.289647	3.040611	0.002361	0.006774	0	0.547412	2.087194	6.696381	25.66933	6.029877
7639	SMIM23	12.49719	3.919761	1.115944	3.512507	0.000444	0.001545	0	0	4.87012	31.24978	27.80844	11.05477
16297	LOC11962ɛ	1.62023	3.918765	2.438256	1.6072	0.10801	0.176196	0	0.547412	0	0	2.139111	7.034856
8094	ITLN1	8.402183	3.918399	1.158533	3.382208	0.000719	0.002361	0	1.094824	2.087194	13.39276	27.80844	6.029877
5346	FUT7	15.18414	3.91695	0.872357	4.490076	7.12E-06	3.54E-05	0.542086	1.094824	4.174388	42.41041	27.80844	15.07469
5198	HNF4A	28.20596	3.916758	0.855057	4.580698	4.63E-06	2.37E-05	0	1.094824	9.740239	66.96381	66.31243	25.12449
7271	LOC11962ɛ	10.34441	3.916154	1.074519	3.644566	0.000268	0.000979	0	0.547412	3.478657	22.32127	25.66933	10.04979
12477	LOC10323ɛ	3.26041	3.91604	1.698839	2.305128	0.021159	0.045085	0.542086	0	0.695731	11.16063	2.139111	5.024897
8702	SHISA7	8.848626	3.915696	1.222975	3.201779	0.001366	0.004173	0	0	3.478657	22.32127	19.252	8.039836
4981	PDZRN4	14.30666	3.915248	0.833318	4.698387	2.62E-06	1.40E-05	2.710432	1.094824	1.391463	33.4819	32.08666	15.07469
15229	LOC11961ɛ	1.694286	3.914625	2.186508	1.790355	0.073397	0.128128	0	0.547412	0	4.464254	2.139111	3.014938
15192	LOC10323ɛ	1.719006	3.914569	2.178227	1.797135	0.072314	0.126537	0	0	0.695731	4.464254	2.139111	3.014938
15193	LOC11962ɛ	1.719006	3.914569	2.178227	1.797135	0.072314	0.126537	0	0	0.695731	4.464254	2.139111	3.014938
6238	LOC10324ɛ	13.42554	3.910169	0.963759	4.057206	4.97E-05	0.000212	0	1.642235	3.478657	29.01765	36.36488	10.04979
2211	EBI3	36.05627	3.9097	0.515647	7.58212	3.40E-14	4.09E-13	4.336691	4.379295	4.87012	49.10679	81.28621	72.35852
4743	CACNA1S	18.53862	3.908879	0.805374	4.853494	1.21E-06	6.80E-06	0.542086	1.094824	5.565851	42.41041	38.50399	23.11453
4649	LOC10321ɛ	18.44906	3.908755	0.79493	4.917103	8.78E-07	5.02E-06	0.542086	1.094824	5.565851	35.71403	40.6431	27.13445
12475	COL10A1	3.422509	3.908542	1.695374	2.305416	0.021143	0.045058	0	0	1.391463	6.696381	6.417332	6.029877
4842	LOC11962ɛ	22.17256	3.908214	0.816405	4.787102	1.69E-06	9.29E-06	0	1.642235	6.957314	49.10679	49.19955	26.12947
12260	LYPD6B	3.245795	3.908119	1.660358	2.353782	0.018584	0.040297	0	0.547412	0.695731	8.928508	4.278221	5.024897

12202	LOC10321(	3.229405	3.905983	1.650779	2.366146	0.017974	0.039161	0.542086	0	0.695731	6.696381	6.417332	5.024897
3590	LOC10324(	25.59005	3.905126	0.678401	5.75637	8.59E-09	6.36E-08	0.542086	4.926706	4.174388	42.41041	66.31243	35.17428
15498	LOC11961(	1.730426	3.904666	2.23797	1.744736	0.081031	0.138999	0.542086	0	0	6.696381	2.139111	1.004979
15497	LOC10324(	1.731314	3.904649	2.237678	1.744955	0.080993	0.138942	0	0.547412	0	6.696381	2.139111	1.004979
15238	LOC10322(	1.703503	3.904417	2.182218	1.789197	0.073583	0.128327	0	0	0.695731	2.232127	4.278221	3.014938
15240	LOC11961(	1.703503	3.904417	2.182218	1.789197	0.073583	0.128327	0	0	0.695731	2.232127	4.278221	3.014938
15241	TYR	1.703503	3.904417	2.182218	1.789197	0.073583	0.128327	0	0	0.695731	2.232127	4.278221	3.014938
15242	B3GALT2	1.703503	3.904417	2.182218	1.789197	0.073583	0.128327	0	0	0.695731	2.232127	4.278221	3.014938
15243	LOC11961(	1.703503	3.904417	2.182218	1.789197	0.073583	0.128327	0	0	0.695731	2.232127	4.278221	3.014938
15449	ALDOB	1.756034	3.904173	2.229658	1.751019	0.079943	0.13746	0	0	0.695731	6.696381	2.139111	1.004979
15450	CCR6	1.756034	3.904173	2.229658	1.751019	0.079943	0.13746	0	0	0.695731	6.696381	2.139111	1.004979
15453	LOC10321(	1.756034	3.904173	2.229658	1.751019	0.079943	0.13746	0	0	0.695731	6.696381	2.139111	1.004979
15455	LOC11962(	1.756034	3.904173	2.229658	1.751019	0.079943	0.13746	0	0	0.695731	6.696381	2.139111	1.004979
15457	RANBP3L	1.756034	3.904173	2.229658	1.751019	0.079943	0.13746	0	0	0.695731	6.696381	2.139111	1.004979
15458	LOC10322(	1.756034	3.904173	2.229658	1.751019	0.079943	0.13746	0	0	0.695731	6.696381	2.139111	1.004979
15459	TBC1D3B	1.756034	3.904173	2.229658	1.751019	0.079943	0.13746	0	0	0.695731	6.696381	2.139111	1.004979
15461	LOC11962(	1.756034	3.904173	2.229658	1.751019	0.079943	0.13746	0	0	0.695731	6.696381	2.139111	1.004979
12571	SBSPON	3.459537	3.90364	1.705161	2.289309	0.022061	0.046648	0	0	1.391463	8.928508	6.417332	4.019918
12572	TCL1A	3.459537	3.90364	1.705161	2.289309	0.022061	0.046648	0	0	1.391463	8.928508	6.417332	4.019918
12573	KIT	3.459537	3.90364	1.705161	2.289309	0.022061	0.046648	0	0	1.391463	8.928508	6.417332	4.019918
2638	LOC11962(	47.31377	3.903507	0.564179	6.91892	4.55E-12	4.59E-11	4.878777	3.284471	9.740239	82.5887	141.1813	42.20914
9710	LOC11962(	6.831031	3.902973	1.333793	2.926221	0.003431	0.009394	0.542086	0	2.087194	26.78552	8.556443	3.014938
12480	LOC11962(	3.177762	3.902804	1.69353	2.304538	0.021192	0.045144	0	0.547412	0.695731	2.232127	8.556443	7.034856
8673	LOC11962(	6.495837	3.902052	1.215324	3.21071	0.001324	0.004059	1.084173	0	1.391463	15.62489	12.83466	8.039836
12598	LOC10324(	3.407006	3.90169	1.708233	2.28405	0.022369	0.047203	0	0	1.391463	4.464254	8.556443	6.029877
7735	ZNF366	12.33917	3.9006	1.120376	3.481511	0.000499	0.001714	0	0	4.87012	35.71403	21.39111	12.05975
1310	LRRTM4	59.64114	3.900571	0.397142	9.821609	9.09E-23	1.84E-21	7.047123	7.663765	7.653045	116.0706	121.9293	97.48301
8734	KCNF1	6.674326	3.899503	1.221361	3.192752	0.001409	0.004289	0	0.547412	2.087194	13.39276	14.97377	9.044815
6706	CTSE	13.59277	3.899295	1.012094	3.852702	0.000117	0.000463	0	1.094824	4.174388	22.32127	44.92132	9.044815
12595	SIGLECL1	3.444034	3.898193	1.706092	2.284867	0.022321	0.047113	0	0	1.391463	6.696381	8.556443	4.019918
12351	LOC11962(	3.266433	3.898173	1.67033	2.333774	0.019608	0.042204	0.542086	0	0.695731	8.928508	6.417332	3.014938
13365	GPR152	3.090606	3.897422	1.82658	2.133726	0.032865	0.065374	0	1.094824	0	11.16063	4.278221	2.009959
13031	LOC11962(	3.036301	3.897397	1.772628	2.198655	0.027902	0.056925	1.084173	0	0	6.696381	6.417332	4.019918
10308	DYRK4	6.103699	3.895406	1.397713	2.786987	0.00532	0.013721	0	2.189647	0	11.16063	19.252	4.019918
11533	TRIM50	26.8781	3.894508	1.553395	2.507093	0.012173	0.02806	0	0.547412	9.740239	49.10679	72.72976	29.1444
15424	LOC11962(	1.715811	3.892653	2.215896	1.756695	0.07897	0.136087	0	0.547412	0	4.464254	4.278221	1.004979
15425	LOC11962(	1.715811	3.892653	2.215896	1.756695	0.07897	0.136087	0	0.547412	0	4.464254	4.278221	1.004979
15426	SMCO3	1.715811	3.892653	2.215896	1.756695	0.07897	0.136087	0	0.547412	0	4.464254	4.278221	1.004979
15427	LOC10322(	1.715811	3.892653	2.215896	1.756695	0.07897	0.136087	0	0.547412	0	4.464254	4.278221	1.004979
6532	SCN7A	13.52777	3.892386	0.991417	3.926084	8.63E-05	0.000351	0	1.094824	4.174388	40.17829	25.66933	10.04979
15383	LOC10324(	1.740531	3.89218	2.207808	1.762916	0.077915	0.134626	0	0	0.695731	4.464254	4.278221	1.004979
15384	C1QTNF8	1.740531	3.89218	2.207808	1.762916	0.077915	0.134626	0	0	0.695731	4.464254	4.278221	1.004979
15385	KLRK1	1.740531	3.89218	2.207808	1.762916	0.077915	0.134626	0	0	0.695731	4.464254	4.278221	1.004979
15386	LOC10323(	1.740531	3.89218	2.207808	1.762916	0.077915	0.134626	0	0	0.695731	4.464254	4.278221	1.004979
12717	TOX3	2.984657	3.891989	1.726481	2.25429	0.024178	0.050544	0.542086	0.547412	0	2.232127	8.556443	6.029877
10358	PATE1	4.778607	3.891746	1.402764	2.774341	0.005531	0.014197	0	1.094824	0.695731	11.16063	10.69555	5.024897
10834	LOC11962(	5.184565	3.890692	1.460767	2.663459	0.007734	0.018977	0	0	2.087194	11.16063	12.83466	5.024897
10835	ADAD2	5.184565	3.890692	1.460767	2.663459	0.007734	0.018977	0	0	2.087194	11.16063	12.83466	5.024897
4277	SLC34A1	33.98228	3.889945	0.752095	5.172147	2.31E-07	1.44E-06	2.168345	0	11.1317	82.5887	57.75599	50.24897
10074	LOC11962(	6.99313	3.888031	1.366675	2.844884	0.004443	0.011724	0	0	2.782925	22.32127	12.83466	4.019918
8620	LOC10324(	8.50547	3.887809	1.206088	3.223486	0.001266	0.003906	0	0	3.478657	15.62489	12.83466	19.09461
11818	LOC10324(	5.274124	3.88706	1.590424	2.444041	0.014524	0.032672	0	0	2.087194	17.85702	10.69555	1.004979
9181	LOC11962(	6.52516	3.887041	1.270078	3.060474	0.00221	0.006398	0	1.094824	1.391463	13.39276	19.252	4.019918
8077	LOC11961(	8.213161	3.886702	1.146529	3.389974	0.000699	0.002301	0	1.094824	2.087194	13.39276	25.66933	7.034856
12919	FMOD	3.465559	3.886332	1.752027	2.218192	0.026542	0.05462	0	0	1.391463	6.696381	10.69555	2.009959
10929	TBXT	5.169062	3.886192	1.469255	2.645009	0.008169	0.019871	0	0	2.087194	8.928508	14.97377	5.024897
13169	LOC10323(	3.022573	3.885809	1.785116	2.176783	0.029497	0.059547	0	1.094824	0	4.464254	8.556443	4.019918
10437	SYT2	42.72222	3.885375	1.408625	2.758274	0.005811	0.014801	0.542086	0.547412	15.30609	73.66019	124.0684	42.20914
7824	GRK1	7.842455	3.884857	1.122938	3.459548	0.000541	0.001839	1.084173	1.094824	0.695731	15.62489	23.53022	5.024897
2372	LOC10324(	34.72315	3.884422	0.53143	7.309375	2.68E-13	3.01E-12	4.336691	2.737059	6.261582	64.73168	77.00798	53.26391
12503	LOC11962(	3.236315	3.884393	1.688424	2.300603	0.021414	0.045533	0	0.547412	0.695731	4.464254	10.69555	3.014938
14654	TRIM77	3.013093	3.884102	2.048208	1.896342	0.057915	0.105068	0	1.094824	0	0	14.97377	2.009959
15601	LOC11962(	1.700308	3.883504	2.245698	1.729308	0.083754	0.142728	0	0.547412	0	2.232127	6.417332	1.004979
15548	UTS2B	1.725028	3.883077	2.237459	1.735485	0.082655	0.141302	0	0	0.695731	2.232127	6.417332	1.004979
15550	LOC10323(	1.725028	3.883077	2.237459	1.735485	0.082655	0.141302	0	0	0.695731	2.232127	6.417332	1.004979
15551	LOC11962(	1.725028	3.883077	2.237459	1.735485	0.082655	0.141302	0	0	0.695731	2.232127	6.417332	1.004979
13095	LOC11962(	3.413029	3.881292	1.773665	2.188289	0.028649	0.058157	0	0	1.391463	2.232127	12.83466	4.019918
13096	LOC11961(	3.413029	3.881292	1.773665	2.188289	0.028649	0.058157	0	0	1.391463	2.232127	12.83466	4.019918
16449	LOC11961(	1.684806	3.880972	2.456233	1.58005	0.114095	0.184408	0	0.547412	0	0	8.556443	1.004979
11082	ALX3	5.15356	3.880805	1.487145	2.609568	0.009066	0.021746	0	0	2.087194	6.696381	17.11289	5.024897
16414	LOC11962(	1.709526	3.880639	2.446079	1.586473	0.112632	0.18238	0	0	0.695731	0	8.556443	1.004979
16415	LOC10322(	1.709526	3.880639	2.446079	1.586473	0.112632	0.18238	0	0	0.695731	0	8.556443	1.004979
16416	LOC11962(	1.709526	3.880639	2.446079	1.586473	0.112632	0.18238	0	0	0.695731	0	8.556443	1.004979
16417	LOC10323(	1.709526	3.880639	2.446079	1.586473	0.112632	0.18238	0	0	0.695731	0	8.556443	1.004979

16418	SCGB3A1	1.709526	3.880639	2.446079	1.586473	0.112632	0.18238	0	0	0.695731	0	8.556443	1.004979
8489	PRG2	10.25699	3.880254	1.190926	3.258182	0.001121	0.003511	0	0.547412	3.478657	24.5534	29.94755	3.014938
10051	TMPRSS3	6.946621	3.879674	1.361009	2.850587	0.004364	0.011542	0	0	2.782925	15.62489	19.252	4.019918
13109	LOC119618	3.450057	3.879441	1.774802	2.185844	0.028827	0.058461	0	0	1.391463	4.464254	12.83466	2.009959
11815	OTUD6A	5.243118	3.879377	1.58634	2.445489	0.014466	0.032549	0	0	2.087194	13.39276	14.97377	1.004979
5385	CUNH1orf5	18.32718	3.879016	0.867297	4.472536	7.73E-06	3.82E-05	0.542086	1.094824	5.565851	26.78552	59.8951	16.07967
215	GJB5	303.0472	3.876193	0.196779	19.69822	2.23E-86	2.76E-84	36.86187	38.31883	41.04815	622.7634	498.4128	580.8781
2977	LOC103232	26.44357	3.875413	0.600408	6.454631	1.08E-10	9.69E-10	2.710432	3.831883	3.478657	53.57105	59.8951	35.17428
11855	TREML1	5.227616	3.87516	1.591727	2.434563	0.01491	0.033435	0	0	2.087194	11.16063	17.11289	1.004979
7871	LACRT	12.12516	3.872554	1.123043	3.448267	0.000564	0.001906	0	0	4.87012	29.01765	27.80844	11.05477
5460	LOC103248	15.72026	3.872426	0.872889	4.43633	9.15E-06	4.46E-05	0	3.284471	2.782925	20.08914	47.06043	21.10457
1869	CYP17A1	45.11557	3.871877	0.46896	8.256301	1.50E-16	2.14E-15	6.505036	7.663765	2.782925	80.35657	91.98176	81.40334
5157	EXOC3L2	16.38558	3.871841	0.841875	4.599072	4.24E-06	2.19E-05	0.542086	1.094824	4.87012	26.78552	44.92132	20.09959
13337	LOC103231	3.242337	3.871177	1.808505	2.14054	0.032311	0.064407	0	0.547412	0.695731	2.232127	14.97377	1.004979
4123	ADAMTSL2	19.13879	3.870155	0.733577	5.27573	1.32E-07	8.53E-07	2.168345	1.094824	4.174388	44.64254	40.6431	22.10955
8447	LOC119627	10.32262	3.867399	1.182822	3.269637	0.001077	0.003389	0	0	4.174388	15.62489	32.08666	10.04979
697	TTN	166.7919	3.86704	0.301139	12.84137	9.62E-38	3.67E-36	15.7205	20.80165	27.82925	310.2657	400.0137	226.1204
7602	LOC103215	13.83469	3.86487	1.096506	3.524714	0.000424	0.001483	0	0	5.565851	29.01765	36.36488	12.05975
7932	FABP7	7.928557	3.86119	1.12557	3.43043	0.000603	0.00202	0	1.094824	2.087194	8.928508	21.39111	14.06971
4556	EXD1	15.6285	3.860962	0.776917	4.96959	6.71E-07	3.92E-06	1.084173	2.189647	2.782925	31.24978	36.36488	20.09959
15430	CLDN7	35.78805	3.8587	2.1983	1.755311	0.079206	0.136459	0	0	13.91463	91.51721	79.14709	30.14938
481	ENPP2	165.3467	3.858339	0.260444	14.81444	1.18E-49	6.53E-48	17.34676	23.53871	23.65487	299.105	271.6671	356.7677
6725	LOC119623	13.40092	3.857493	1.002805	3.846703	0.00012	0.000473	0	0.547412	4.87012	24.5534	36.36488	14.06971
9986	TMPRSS2	5.88713	3.856811	1.34654	2.864239	0.00418	0.011128	0	2.189647	0	13.39276	10.69555	9.044815
5317	GALNT15	14.16115	3.853855	0.854422	4.51048	6.47E-06	3.23E-05	0.542086	2.189647	2.782925	29.01765	36.36488	14.06971
10463	LOC103232	4.796417	3.853336	1.39934	2.75368	0.005893	0.014973	0.542086	0	1.391463	13.39276	6.417332	7.034856
12561	GYPA	4.716172	3.853265	1.682147	2.290684	0.021982	0.046524	0	1.094824	0.695731	20.08914	6.417332	0
9874	LOC119626	6.76708	3.850766	1.333332	2.888077	0.003876	0.010436	0	0	2.782925	20.08914	10.69555	7.034856
8346	ITK	7.408686	3.850678	1.167511	3.298195	0.000973	0.0031	1.084173	1.642235	0	11.16063	23.53022	7.034856
9460	LOC103230	6.580886	3.84996	1.285569	2.994752	0.002747	0.007719	0	0.547412	2.087194	17.85702	14.97377	4.019918
8790	PLAAT5	8.402549	3.84953	1.21207	3.175997	0.001493	0.004516	0	0	3.478657	15.62489	19.252	12.05975
10368	LOC103220	4.781801	3.848039	1.388202	2.771959	0.005572	0.014287	0	0.547412	1.391463	11.16063	8.556443	7.034856
8538	SLITRK1	13.82479	3.847603	1.185991	3.244211	0.001178	0.003667	0	0	5.565851	42.41041	29.94755	5.024897
11078	LOC119621	4.657618	3.845163	1.473319	2.609865	0.009058	0.021737	0	1.094824	0.695731	17.85702	4.278221	4.019918
6469	HEATR9	12.64127	3.84446	0.972788	3.952002	7.75E-05	0.000319	0	2.189647	2.782925	22.32127	38.50399	10.04979
10170	UNCX	8.587689	3.843702	1.362075	2.821947	0.004773	0.012479	0	0	3.478657	26.78552	19.252	2.009959
6591	SLC9A4	13.22138	3.843189	0.984266	3.904623	9.44E-05	0.000381	0	0.547412	4.87012	29.01765	27.80844	17.08465
4851	LOC103224	17.2351	3.842175	0.803669	4.780793	1.75E-06	9.57E-06	1.084173	2.189647	3.478657	31.24978	51.33866	14.06971
8061	GRID2	11.87413	3.841905	1.131344	3.395876	0.000684	0.002256	0	0	4.87012	20.08914	34.22577	12.05975
6718	CD84	11.27915	3.840433	0.997898	3.848523	0.000119	0.00047	0	1.094824	3.478657	20.08914	29.94755	13.06473
898	SERPINA3	87.90061	3.840045	0.334495	11.48014	1.66E-30	4.91E-29	9.757555	10.94824	13.91463	151.7846	188.2417	152.7569
9929	FZD10	6.705069	3.838984	1.333659	2.878535	0.003995	0.010697	0	0	2.782925	11.16063	19.252	7.034856
3727	TNFSF14	30.61167	3.838856	0.683605	5.615606	1.96E-08	1.40E-07	1.084173	2.189647	9.044508	58.0353	79.14709	34.1693
5396	LOC103224	23.22085	3.838306	0.859748	4.464456	8.03E-06	3.95E-05	0.542086	0.547412	8.348776	29.01765	72.72976	28.13943
4418	SLITRK3	24.32895	3.838214	0.757601	5.066276	4.06E-07	2.44E-06	0.542086	1.642235	7.653045	49.10679	59.8951	27.13445
4424	CD72	18.57722	3.837997	0.758267	5.06154	4.16E-07	2.50E-06	0.542086	3.284471	3.478657	42.41041	40.6431	21.10457
7384	LOC103215	9.724814	3.836895	1.063944	3.606293	0.000311	0.001118	0	0.547412	3.478657	17.85702	21.39111	15.07469
7129	MAFB	9.627291	3.836055	1.040364	3.687225	0.000227	0.000845	0.542086	0.547412	2.782925	22.32127	23.53022	8.039836
6349	LOC119618	14.01765	3.836012	0.957425	4.006591	6.16E-05	0.000258	2.710432	0	2.782925	26.78552	42.78221	9.044815
4368	ZNF660	20.24962	3.835571	0.751904	5.101142	3.38E-07	2.05E-06	2.168345	1.642235	4.174388	31.24978	64.17332	18.08963
10707	SULT6B1	4.735293	3.83449	1.425622	2.689696	0.007152	0.017757	0	0.547412	1.391463	4.464254	14.97377	7.034856
12355	BMP10	32.93817	3.833325	1.644402	2.331137	0.019746	0.042489	0	0.547412	12.52316	66.96381	83.42532	34.1693
6260	SLC39A4	12.85387	3.832686	0.947481	4.045132	5.23E-05	0.000222	1.084173	0	4.174388	31.24978	23.53022	17.08465
7326	LOC119624	11.52992	3.832362	1.057232	3.624901	0.000289	0.001049	0	0.547412	4.174388	22.32127	32.08666	10.04979
7515	LOC119618	9.729949	3.832355	1.078399	3.553743	0.00038	0.001344	0.542086	0	3.478657	15.62489	25.66933	13.06473
6989	MUC20	9.063741	3.831935	1.025106	3.738086	0.000185	0.000705	0.542086	1.642235	1.391463	8.928508	27.80844	14.06971
11505	SLC6A4	4.381866	3.831622	1.52389	2.514369	0.011925	0.027555	0	1.642235	0	8.928508	10.69555	5.024897
10678	RBM46	4.772321	3.831478	1.420334	2.69759	0.006984	0.017389	0	0.547412	1.391463	6.696381	14.97377	5.024897
7856	LOC103238	13.55568	3.83098	1.109747	3.45212	0.000556	0.001882	0	0	5.565851	40.17829	23.53022	12.05975
10803	LAMB4	4.396481	3.828727	1.433899	2.670152	0.007582	0.018658	0.542086	1.094824	0	11.16063	8.556443	5.024897
12825	LOC103233	3.056773	3.828597	1.713895	2.233858	0.025492	0.052843	0	0.547412	0.695731	8.928508	2.139111	6.029877
12826	FAAH2	3.056773	3.828597	1.713895	2.233858	0.025492	0.052843	0	0.547412	0.695731	8.928508	2.139111	6.029877
10727	LOC103216	4.647251	3.828143	1.425557	2.685367	0.007245	0.017954	0.542086	0.547412	0.695731	13.39276	10.69555	2.009959
12627	LOC103224	3.040383	3.827593	1.681868	2.275799	0.022858	0.048126	0.542086	0	0.695731	6.696381	4.278221	6.029877
11488	CACNG5	4.846377	3.826922	1.519139	2.519139	0.011764	0.027224	0	0.547412	1.391463	11.16063	14.97377	1.004979
6050	DGKK	14.16691	3.825885	0.923287	4.143766	3.42E-05	0.00015	0.542086	1.642235	3.478657	20.08914	49.19955	10.04979
6330	LOC119622	12.67547	3.825571	0.953451	4.012341	6.01E-05	0.000252	0	1.642235	3.478657	26.78552	32.08666	12.05975
6508	LOC119621	14.35735	3.824342	0.971351	3.937137	8.25E-05	0.000337	1.626259	0	4.174388	20.08914	49.19955	11.05477
12880	TRIM48	2.98874	3.823561	1.720466	2.222399	0.026256	0.054194	0	0.547412	0.695731	2.232127	6.417332	8.039836
11477	LOC119620	4.986063	3.822018	1.515432	2.522064	0.011667	0.027025	0	0	2.087194	6.696381	17.11289	4.019918
9064	LOC119628	6.295395	3.822012	1.236175	3.091804	0.001989	0.005835	0.542086	0	2.087194	13.39276	10.69555	11.05477
12926	LYZL6	3.255012	3.820497	1.723676	2.216482	0.026658	0.054829	0	0	1.391463	6.696381	6.417332	5.024897
9070	LOC103237	6.296283	3.820307	1.23624	3.090263	0.002	0.00586	0	0.547412	2.087194	13.39276	10.69555	11.05477
7918	PROKR1	7.373965	3.820281	1.112712	3.433305	0.000596	0.002002	1.626259	0.547412	0.695731	8.928508	21.39111	11.05477

8330	SHISA9	7.627398	3.816561	1.154745	3.305112	0.000949	0.00303	0	1.642235	1.391463	11.16063	23.53022	8.039836
9255	SLC35F3	8.324611	3.815474	1.254271	3.041985	0.00235	0.006751	0	0	3.478657	22.32127	17.11289	7.034856
8605	LOC11962:	11.76519	3.815156	1.182313	3.226857	0.001252	0.003867	0	0	4.87012	22.32127	36.36488	7.034856
9672	LOC10324:	6.422869	3.814623	1.298213	2.938366	0.003299	0.009069	0	0.547412	2.087194	22.32127	8.556443	5.024897
13103	LOC10324(	3.29204	3.814588	1.743979	2.18729	0.028721	0.058273	0	0	1.391463	8.928508	6.417332	3.014938
13053	LOC11962(	3.239509	3.813483	1.736788	2.19571	0.028113	0.057257	0	0	1.391463	4.464254	8.556443	5.024897
8815	LOC11962:	10.00313	3.812599	1.202564	3.170392	0.001522	0.004591	0	0	4.174388	17.85702	29.94755	8.039836
8496	LOC10323:	7.403288	3.809986	1.169712	3.257199	0.001125	0.003521	0.542086	1.642235	0.695731	6.696381	27.80844	7.034856
13023	LOC11962(	3.115326	3.809146	1.731075	2.200451	0.027775	0.056699	0	0.547412	0.695731	11.16063	4.278221	2.009959
5171	CYP2B6	17.35965	3.808529	0.829075	4.593709	4.35E-06	2.24E-05	1.084173	0.547412	5.565851	26.78552	47.06043	23.11453
13244	ATP4B	5.044616	3.807692	1.764059	2.158484	0.03089	0.062007	0	0	2.087194	8.928508	19.252	0
10214	LOC10323:	6.599583	3.806358	1.353405	2.812431	0.004917	0.012798	0	0	2.782925	20.08914	10.69555	6.029877
12922	A1CF	3.098936	3.805768	1.715961	2.217864	0.026564	0.054653	0.542086	0	0.695731	8.928508	6.417332	2.009959
6717	LTA	12.36829	3.805357	0.988657	3.849018	0.000119	0.000469	0	2.189647	2.782925	31.24978	29.94755	8.039836
11	TXNDC16	2849.389	3.804496	0.096488	39.42984	0	0	383.2551	396.8736	361.0846	5209.784	5311.412	5433.924
9524	LOC11962:	6.360858	3.803211	1.277055	2.978111	0.0029	0.008096	0	0.547412	2.087194	13.39276	17.11289	5.024897
12927	LHFPL3	3.099824	3.802571	1.715619	2.216442	0.026661	0.05483	0	0.547412	0.695731	8.928508	6.417332	2.009959
7732	LOC10322:	9.615871	3.802538	1.091844	3.482676	0.000496	0.001707	0	0.547412	3.478657	20.08914	23.53022	10.04979
13585	LOC11962!	3.313565	3.802185	1.81754	2.09194	0.036444	0.071318	0	0	1.391463	8.928508	8.556443	1.004979
6280	ZNF831	12.18917	3.802179	0.941721	4.037477	5.40E-05	0.000229	0	2.189647	2.782925	17.85702	34.22577	16.07967
6229	PTPN5	16.11191	3.80203	0.935895	4.062452	4.86E-05	0.000207	0.542086	0.547412	5.565851	46.87467	32.08666	11.05477
13264	TUBAL3	3.261035	3.801606	1.764433	2.154576	0.031195	0.062519	0	0	1.391463	4.464254	10.69555	3.014938
13265	ADGRE3	3.261035	3.801606	1.764433	2.154576	0.031195	0.062519	0	0	1.391463	4.464254	10.69555	3.014938
13064	MYL2	3.030903	3.801387	1.732652	2.19397	0.028238	0.057463	0.542086	0	0.695731	2.232127	10.69555	4.019918
9423	DMP1	6.079083	3.800342	1.265854	3.002196	0.00268	0.007562	0	1.094824	1.391463	6.696381	19.252	8.039836
13537	SMCO1	2.922222	3.799177	1.808525	2.100705	0.035667	0.07005	0.542086	0.547412	0	11.16063	4.278221	1.004979
9074	ANGPTL4	5.953125	3.797191	1.228855	3.090023	0.002001	0.005864	1.084173	0.547412	0.695731	13.39276	14.97377	5.024897
8164	LOC11962:	9.451202	3.796512	1.129991	3.359772	0.00078	0.00254	0	1.094824	2.782925	17.85702	29.94755	5.024897
13580	CLEC9A	2.870579	3.796105	1.813968	2.092708	0.036375	0.071212	0	1.094824	0	6.696381	6.417332	3.014938
13647	LOC10321!	3.298063	3.795893	1.824878	2.08008	0.037518	0.073087	0	0	1.391463	6.696381	10.69555	1.004979
5566	LOC10324:	13.82935	3.795355	0.864992	4.387735	1.15E-05	5.47E-05	0.542086	1.642235	3.478657	29.01765	34.22577	14.06971
14416	LOC11962:	2.859324	3.793101	1.954565	1.940637	0.052302	0.096452	1.084173	0	0	2.232127	12.83466	1.004979
5389	ADAMTSL1	21.45376	3.792396	0.848459	4.469744	7.83E-06	3.86E-05	0	3.831883	4.87012	51.33892	55.61688	13.06473
14027	ABCG5	2.892104	3.79239	1.888289	2.008374	0.044604	0.084536	0	1.094824	0	6.696381	8.556443	1.004979
13581	LOC10324:	3.245532	3.79223	1.812391	2.092391	0.036404	0.071261	0	0	1.391463	2.232127	12.83466	3.014938
14247	FAM205C	3.089456	3.792124	1.926644	1.968253	0.049039	0.091495	0.542086	0	0.695731	4.464254	12.83466	0
14249	ANKS4B	3.089456	3.792124	1.926644	1.968253	0.049039	0.091495	0.542086	0	0.695731	4.464254	12.83466	0
10787	LOC10323(	4.62892	3.791141	1.418281	2.673053	0.007516	0.018525	0.542086	0	1.391463	13.39276	6.417332	6.029877
8395	ITIH1	7.890842	3.7909	1.154681	3.283071	0.001027	0.003252	0	0.547412	2.782925	17.85702	17.11289	9.044815
6918	LOC11962:	10.76744	3.790194	1.005914	3.767911	0.000165	0.000633	0	1.642235	2.782925	22.32127	27.80844	10.04979
13669	HRH2	54.40638	3.789318	1.826324	2.074834	0.038002	0.07391	0	0.547412	21.56767	100.4457	147.5986	56.27885
15746	LOC11961{	35.91648	3.788414	2.217105	1.708721	0.087503	0.147736	0	0	14.61036	69.19594	100.5382	31.15436
7312	SLC5A5	11.16738	3.787956	1.043225	3.631007	0.000282	0.001026	0	0.547412	4.174388	24.5534	25.66933	12.05975
9	EDIL3	4725.541	3.787756	0.100004	37.87598	0	0	591.9583	695.7604	626.854	8720.92	8505.104	9212.647
10920	CPA1	6.372904	3.787345	1.430848	2.646924	0.008123	0.019775	0	0.547412	2.087194	8.928508	25.66933	1.004979
12108	RAI2	4.52715	3.787176	1.588404	2.384265	0.017113	0.037566	0	1.094824	0.695731	20.08914	4.278221	1.004979
6760	F13A1	12.58868	3.786504	0.988212	3.831672	0.000127	0.000501	0	1.094824	4.174388	37.94616	19.252	13.06473
13381	LOC11962!	3.053316	3.786323	1.777029	2.130704	0.033114	0.065789	0	0.547412	0.695731	2.232127	12.83466	2.009959
1099	LOC10322⁄	78.89075	3.785868	0.359657	10.52632	6.53E-26	1.58E-24	6.505036	13.13788	12.52316	154.0168	145.4595	141.7021
9129	CTLA4	8.077036	3.785799	1.232045	3.072776	0.002121	0.006176	0	0	3.478657	20.08914	12.83466	12.05975
5973	LOC10324:	12.28015	3.785426	0.906464	4.176037	2.97E-05	0.000132	1.084173	0.547412	3.478657	24.5534	29.94755	14.06971
3592	LOC10324(	30.76172	3.784813	0.6578	5.753744	8.73E-09	6.46E-08	1.084173	3.284471	8.348776	66.96381	72.72976	32.15934
11826	VSTM1	4.89608	3.781494	1.547976	2.442864	0.014571	0.032756	0	0	2.087194	17.85702	6.417332	3.014938
6237	CALHM3	10.20966	3.779741	0.931415	4.058064	4.95E-05	0.000211	0.542086	1.642235	2.087194	15.62489	19.252	22.10955
11718	LOC10322:	4.880577	3.778396	1.531804	2.466632	0.013639	0.030943	0	0	2.087194	15.62489	8.556443	3.014938
7706	ADGRF1	9.287163	3.777259	1.08182	3.491579	0.00048	0.001657	0	1.094824	2.782925	24.5534	19.252	8.039836
3466	RYR2	55.12309	3.775246	0.640764	5.89179	3.82E-09	2.93E-08	1.084173	3.831883	18.08902	98.21359	141.1813	68.3386
7278	PRCD	9.077669	3.774797	1.036887	3.64051	0.000272	0.000994	1.084173	0.547412	2.087194	22.32127	21.39111	7.034856
12090	GFRA2	46.30028	3.774368	1.579778	2.389176	0.016886	0.037131	0	1.642235	17.39328	75.89232	132.6249	50.24897
9521	STAR	7.487449	3.773968	1.266948	2.978786	0.002894	0.008081	0	1.642235	1.391463	8.928508	29.94755	3.014938
10962	STRA8	4.422088	3.773919	1.431293	2.636721	0.008371	0.020302	0	1.094824	0.695731	11.16063	8.556443	5.024897
11399	LOC11961{	4.317027	3.773563	1.487501	2.536847	0.011186	0.026087	0	1.094824	0.695731	2.232127	12.83466	9.044815
6213	LOC10321:	15.74633	3.772952	0.927565	4.067589	4.75E-05	0.000203	0.542086	0.547412	5.565851	24.5534	49.19955	14.06971
9368	LOC11961!	7.977997	3.772559	1.251593	3.014206	0.002577	0.007312	0	0	3.478657	8.928508	21.39111	14.06971
11907	LOC10323:	4.140313	3.77169	1.556335	2.423443	0.015374	0.034326	0	1.642235	0	4.464254	10.69555	8.039836
6193	ELFN1	15.37698	3.770479	0.924858	4.076821	4.57E-05	0.000196	0.542086	1.642235	4.174388	44.64254	34.22577	7.034856
9223	CCDC73	8.868267	3.7684	1.235856	3.049222	0.002294	0.006613	0	2.189647	1.391463	13.39276	34.22577	2.009959
5431	MYO1H	16.31215	3.768376	0.847562	4.446138	8.74E-06	4.28E-05	2.168345	1.642235	2.782925	46.87467	36.36488	8.039836
9258	CDH5	5.936569	3.76794	1.238926	3.041296	0.002356	0.006764	0	1.094824	1.391463	13.39276	10.69555	9.044815
11083	LOC10322:	4.391083	3.766152	1.443202	2.60958	0.009065	0.021746	0	1.094824	0.695731	6.696381	12.83466	5.024897
46	VEPH1	1203.356	3.765886	0.123743	30.43316	2.00E-203	1.16E-200	165.3363	181.7407	146.1036	2191.949	2305.961	2229.044
17899	LOC11962(	1.60404	3.765697	2.766013	1.361417	0.173382	0.25752	0	0	0.695731	8.928508	0	0
16260	LOC10324:	47.17917	3.762925	2.329398	1.615407	0.106223	0.173673	0	0	19.48048	91.51721	136.9031	35.17428
9361	LOC11961!	5.905564	3.761646	1.24731	3.015808	0.002563	0.007279	0	1.094824	1.391463	8.928508	14.97377	9.044815

11235	MLNR	4.212594	3.761561	1.461526	2.573721	0.010061	0.023808	1.084173	0.547412	0	8.928508	10.69555	4.019918
7180	FGF14	10.14146	3.761532	1.024562	3.671357	0.000241	0.000893	0	2.737059	1.391463	17.85702	27.80844	11.05477
16735	LOC10323	1.529984	3.759956	2.446927	1.536603	0.12439	0.197605	0	0	0.695731	4.464254	0	4.019918
16780	LOC10323	1.505264	3.759708	2.459033	1.528937	0.12628	0.200069	0	0.547412	0	4.464254	0	4.019918
12044	SPEGNB	4.343687	3.758907	1.567555	2.397942	0.016487	0.036393	0.542086	0.547412	0.695731	0	19.252	5.024897
7457	PLA2G4D	10.89482	3.757334	1.04974	3.579302	0.000345	0.001228	0	0.547412	4.174388	15.62489	29.94755	15.07469
5374	TCEAL7	16.708	3.757114	0.839048	4.477829	7.54E-06	3.73E-05	1.084173	1.094824	4.87012	26.78552	51.33866	15.07469
6113	LOC10324	11.94196	3.756574	0.913253	4.1134	3.90E-05	0.00017	1.084173	1.094824	2.782925	24.5534	32.08666	10.04979
9907	LOC11961	6.193362	3.755841	1.302712	2.883095	0.003938	0.010567	0	0.547412	2.087194	13.39276	17.11289	4.019918
5842	IYD	11.69119	3.755794	0.883161	4.252672	2.11E-05	9.61E-05	1.084173	1.642235	2.087194	22.32127	29.94755	13.06473
4664	DPEP1	17.90776	3.755357	0.765028	4.908781	9.16E-07	5.22E-06	1.084173	1.094824	5.565851	35.71403	27.80844	36.17926
16821	BMP15	1.542292	3.754901	2.467843	1.521532	0.128126	0.202499	0	0.547412	0	6.696381	0	2.009959
16781	LOC11962	1.567012	3.754647	2.45622	1.528628	0.126357	0.200166	0	0	0.695731	6.696381	0	2.009959
16782	LOC10322	1.567012	3.754647	2.45622	1.528628	0.126357	0.200166	0	0	0.695731	6.696381	0	2.009959
10286	ST6GALNA	6.037286	3.754354	1.344846	2.791662	0.005244	0.013552	0.542086	0.547412	1.391463	15.62489	17.11289	1.004979
7816	LOC10323	7.278584	3.75434	1.084593	3.461519	0.000537	0.001827	1.084173	0.547412	1.391463	15.62489	14.97377	10.04979
7657	PCARE	10.76718	3.753821	1.070117	3.507862	0.000452	0.001568	0	1.094824	3.478657	15.62489	36.36488	8.039836
10332	NYX	6.34855	3.753145	1.349467	2.781206	0.005416	0.013935	0	0	2.782925	11.16063	17.11289	7.034856
16112	CUNH6orf	1.489761	3.752667	2.283812	1.643159	0.10035	0.165568	0	0.547412	0	2.232127	2.139111	4.019918
7535	LOC10324	14.41956	3.752535	1.057809	3.547458	0.000389	0.001372	0	0	6.261582	33.4819	25.66933	21.10457
8946	PRDM9	7.140674	3.750622	1.197158	3.132939	0.001731	0.005143	0	2.189647	0.695731	20.08914	12.83466	7.034856
7745	GRIK1	10.86382	3.750549	1.077778	3.479892	0.000502	0.001722	0	0.547412	4.174388	11.16063	34.22577	15.07469
8808	ENPP3	7.568157	3.749829	1.182168	3.171994	0.001514	0.00457	0	1.094824	2.087194	20.08914	17.11289	5.024897
8060	LOC10323	14.54269	3.749803	1.104125	3.396176	0.000683	0.002254	0	0	6.261582	35.71403	34.22577	11.05477
10859	GSG1L	6.422606	3.749646	1.410207	2.658933	0.007839	0.019191	0	0	2.782925	15.62489	17.11289	3.014938
10122	LOC11961	6.162356	3.748949	1.323603	2.832382	0.00462	0.012135	0	0.547412	2.087194	8.928508	21.39111	4.019918
8051	AQP2	9.135169	3.747464	1.102506	3.399041	0.000676	0.002233	0	1.094824	2.782925	26.78552	17.11289	7.034856
12543	GPR119	4.787561	3.746773	1.632985	2.294432	0.021766	0.046133	0	0	2.087194	2.232127	21.39111	3.014938
8912	IRX2	7.109668	3.744879	1.192234	3.141061	0.001683	0.005022	0	2.189647	0.695731	15.62489	17.11289	7.034856
9009	LOC10324	7.712978	3.744418	1.202967	3.112654	0.001854	0.005471	0.542086	0	2.782925	13.39276	23.53022	6.029877
2221	CCDC197	48.6896	3.744368	0.495063	7.563424	3.93E-14	4.70E-13	8.673382	2.189647	9.740239	100.4457	70.59065	100.4979
9017	LOC10323	7.713866	3.742905	1.203072	3.111124	0.001864	0.005495	0	0.547412	2.782925	13.39276	23.53022	6.029877
5847	ISL1	23.5728	3.740809	0.880201	4.249948	2.14E-05	9.72E-05	0	1.094824	9.044508	53.57105	55.61688	22.10955
17085	MARCHF1	1.56293	3.740593	2.520994	1.483777	0.137868	0.214516	0.542086	0	0	6.696381	2.139111	0
17086	LOC11962	1.56293	3.740593	2.520994	1.483777	0.137868	0.214516	0.542086	0	0	6.696381	2.139111	0
17079	LOC11962	1.563817	3.740569	2.520583	1.484009	0.137806	0.214458	0	0.547412	0	6.696381	2.139111	0
17080	PHOX2A	1.563817	3.740569	2.520583	1.484009	0.137806	0.214458	0	0.547412	0	6.696381	2.139111	0
17081	LOC11962	1.563817	3.740569	2.520583	1.484009	0.137806	0.214458	0	0.547412	0	6.696381	2.139111	0
17082	LOC10322	1.563817	3.740569	2.520583	1.484009	0.137806	0.214458	0	0.547412	0	6.696381	2.139111	0
17083	LOC11962	1.563817	3.740569	2.520583	1.484009	0.137806	0.214458	0	0.547412	0	6.696381	2.139111	0
17014	LOC10324	1.588537	3.739903	2.509277	1.49043	0.136111	0.212654	0	0	0.695731	6.696381	2.139111	0
17015	LOC10324	1.588537	3.739903	2.509277	1.49043	0.136111	0.212654	0	0	0.695731	6.696381	2.139111	0
17016	GSX2	1.588537	3.739903	2.509277	1.49043	0.136111	0.212654	0	0	0.695731	6.696381	2.139111	0
11654	LRRC14B	6.428629	3.739495	1.507861	2.48	0.013138	0.029971	0	0	2.782925	13.39276	21.39111	1.004979
16090	LOC11962	1.525902	3.738245	2.268295	1.648042	0.099344	0.164143	0.542086	0	0	4.464254	2.139111	2.009959
16055	PYDC5	1.551509	3.737981	2.258301	1.655218	0.09788	0.162027	0	0	0.695731	4.464254	2.139111	2.009959
16056	LOC10324	1.551509	3.737981	2.258301	1.655218	0.09788	0.162027	0	0	0.695731	4.464254	2.139111	2.009959
16057	PROK2	1.551509	3.737981	2.258301	1.655218	0.09788	0.162027	0	0	0.695731	4.464254	2.139111	2.009959
16058	ZFP57	1.551509	3.737981	2.258301	1.655218	0.09788	0.162027	0	0	0.695731	4.464254	2.139111	2.009959
16059	PCP4	1.551509	3.737981	2.258301	1.655218	0.09788	0.162027	0	0	0.695731	4.464254	2.139111	2.009959
16060	LOC11962	1.551509	3.737981	2.258301	1.655218	0.09788	0.162027	0	0	0.695731	4.464254	2.139111	2.009959
9912	TMEM239	7.958613	3.73715	1.296961	2.881466	0.003958	0.010617	0	0	3.478657	17.85702	21.39111	5.024897
16834	RBP2	1.498978	3.735935	2.459562	1.518943	0.128777	0.20337	0	0	0.695731	0	4.278221	4.019918
16870	LOC11962	1.474259	3.735596	2.472051	1.511132	0.130755	0.206041	0	0.547412	0	0	4.278221	4.019918
16871	LOC10323	1.474259	3.735596	2.472051	1.511132	0.130755	0.206041	0	0.547412	0	0	4.278221	4.019918
16872	LOC11962	1.473371	3.735583	2.472505	1.51085	0.130827	0.206142	0.542086	0	0	0	4.278221	4.019918
6461	BTK	13.56647	3.733969	0.944054	3.955251	7.65E-05	0.000315	0	1.642235	4.174388	35.71403	27.80844	12.05975
20516	LOC11962	0.744042	3.733414	3.964294	0.94176	0.346315	0.447515	0	0	0	4.464254	0	0
20518	LOC11961	0.744042	3.733414	3.964294	0.94176	0.346315	0.447515	0	0	0	4.464254	0	0
20520	LOC10324	0.744042	3.733414	3.964294	0.94176	0.346315	0.447515	0	0	0	4.464254	0	0
20521	LOC10322	0.744042	3.733414	3.964294	0.94176	0.346315	0.447515	0	0	0	4.464254	0	0
20522	LOC10322	0.744042	3.733414	3.964294	0.94176	0.346315	0.447515	0	0	0	4.464254	0	0
20523	LOC11962	0.744042	3.733414	3.964294	0.94176	0.346315	0.447515	0	0	0	4.464254	0	0
20524	LOC10324	0.744042	3.733414	3.964294	0.94176	0.346315	0.447515	0	0	0	4.464254	0	0
20525	LOC10323	0.744042	3.733414	3.964294	0.94176	0.346315	0.447515	0	0	0	4.464254	0	0
20526	CAVIN3	0.744042	3.733414	3.964294	0.94176	0.346315	0.447515	0	0	0	4.464254	0	0
20527	LOC11962	0.744042	3.733414	3.964294	0.94176	0.346315	0.447515	0	0	0	4.464254	0	0
20528	LOC11962	0.744042	3.733414	3.964294	0.94176	0.346315	0.447515	0	0	0	4.464254	0	0
20529	LOC11962	0.744042	3.733414	3.964294	0.94176	0.346315	0.447515	0	0	0	4.464254	0	0
20530	LOC10324	0.744042	3.733414	3.964294	0.94176	0.346315	0.447515	0	0	0	4.464254	0	0
20531	LOC10324	0.744042	3.733414	3.964294	0.94176	0.346315	0.447515	0	0	0	4.464254	0	0
20532	LOC10322	0.744042	3.733414	3.964294	0.94176	0.346315	0.447515	0	0	0	4.464254	0	0
20533	LOC10322	0.744042	3.733414	3.964294	0.94176	0.346315	0.447515	0	0	0	4.464254	0	0
20534	LOC11962	0.744042	3.733414	3.964294	0.94176	0.346315	0.447515	0	0	0	4.464254	0	0

20536	LOC10324(	0.744042	3.733414	3.964294	0.94176	0.346315	0.447515	0	0	0	4.464254	0	0
20538	LOC11962ε	0.744042	3.733414	3.964294	0.94176	0.346315	0.447515	0	0	0	4.464254	0	0
20539	LOC11961ε	0.744042	3.733414	3.964294	0.94176	0.346315	0.447515	0	0	0	4.464254	0	0
20541	LOC11961ε	0.744042	3.733414	3.964294	0.94176	0.346315	0.447515	0	0	0	4.464254	0	0
20542	LOC10324;	0.744042	3.733414	3.964294	0.94176	0.346315	0.447515	0	0	0	4.464254	0	0
20543	EBLN1	0.744042	3.733414	3.964294	0.94176	0.346315	0.447515	0	0	0	4.464254	0	0
20544	LOC11961‹	0.744042	3.733414	3.964294	0.94176	0.346315	0.447515	0	0	0	4.464254	0	0
20545	LOC11961‹	0.744042	3.733414	3.964294	0.94176	0.346315	0.447515	0	0	0	4.464254	0	0
20546	LOC10321‹	0.744042	3.733414	3.964294	0.94176	0.346315	0.447515	0	0	0	4.464254	0	0
20547	LOC11961‹	0.744042	3.733414	3.964294	0.94176	0.346315	0.447515	0	0	0	4.464254	0	0
20551	LOC11961‹	0.744042	3.733414	3.964294	0.94176	0.346315	0.447515	0	0	0	4.464254	0	0
20552	LOC11961‹	0.744042	3.733414	3.964294	0.94176	0.346315	0.447515	0	0	0	4.464254	0	0
20553	LOC11962(	0.744042	3.733414	3.964294	0.94176	0.346315	0.447515	0	0	0	4.464254	0	0
20554	LOC11962(	0.744042	3.733414	3.964294	0.94176	0.346315	0.447515	0	0	0	4.464254	0	0
20555	RNASE12	0.744042	3.733414	3.964294	0.94176	0.346315	0.447515	0	0	0	4.464254	0	0
20556	LOC10323:	0.744042	3.733414	3.964294	0.94176	0.346315	0.447515	0	0	0	4.464254	0	0
20557	LOC10323:	0.744042	3.733414	3.964294	0.94176	0.346315	0.447515	0	0	0	4.464254	0	0
20559	TEX13C	0.744042	3.733414	3.964294	0.94176	0.346315	0.447515	0	0	0	4.464254	0	0
20560	ZCCHC13	0.744042	3.733414	3.964294	0.94176	0.346315	0.447515	0	0	0	4.464254	0	0
20561	LOC11962:	0.744042	3.733414	3.964294	0.94176	0.346315	0.447515	0	0	0	4.464254	0	0
20563	LOC11962:	0.744042	3.733414	3.964294	0.94176	0.346315	0.447515	0	0	0	4.464254	0	0
20564	LOC11962:	0.744042	3.733414	3.964294	0.94176	0.346315	0.447515	0	0	0	4.464254	0	0
20566	LOC11962:	0.744042	3.733414	3.964294	0.94176	0.346315	0.447515	0	0	0	4.464254	0	0
20570	LOC11962:	0.744042	3.733414	3.964294	0.94176	0.346315	0.447515	0	0	0	4.464254	0	0
20571	LOC11962:	0.744042	3.733414	3.964294	0.94176	0.346315	0.447515	0	0	0	4.464254	0	0
20572	LOC11962:	0.744042	3.733414	3.964294	0.94176	0.346315	0.447515	0	0	0	4.464254	0	0
20573	LOC11962:	0.744042	3.733414	3.964294	0.94176	0.346315	0.447515	0	0	0	4.464254	0	0
20574	LOC11962:	0.744042	3.733414	3.964294	0.94176	0.346315	0.447515	0	0	0	4.464254	0	0
8782	LOC11961ε	9.377513	3.73162	1.173893	3.178843	0.001479	0.004476	0	0	4.174388	15.62489	21.39111	15.07469
12498	AJAP1	29.14206	3.729351	1.619927	2.302173	0.021325	0.045362	0	0.547412	11.82743	71.42806	59.8951	31.15436
14421	WT1	2.711675	3.728936	1.922415	1.939714	0.052414	0.096619	0.542086	0.547412	0	11.16063	0	4.019918
14959	LOC11961ε	3.171052	3.727908	2.021978	1.843694	0.065228	0.115922	0	0	1.391463	15.62489	0	2.009959
16139	LOC11961‹	1.510399	3.726773	2.273271	1.639388	0.101133	0.166591	0.542086	0	0	2.232127	4.278221	2.009959
16130	LOC10322:	1.511287	3.726765	2.272915	1.639641	0.10108	0.166522	0	0.547412	0	2.232127	4.278221	2.009959
16131	LOC11962‹	1.511287	3.726765	2.272915	1.639641	0.10108	0.166522	0	0.547412	0	2.232127	4.278221	2.009959
16135	SERPINB2	1.511287	3.726765	2.272915	1.639641	0.10108	0.166522	0	0.547412	0	2.232127	4.278221	2.009959
16096	LOC10321ε	1.536006	3.726551	2.263123	1.64664	0.099632	0.164486	0	0	0.695731	2.232127	4.278221	2.009959
16097	LOC11962!	1.536006	3.726551	2.263123	1.64664	0.099632	0.164486	0	0	0.695731	2.232127	4.278221	2.009959
16098	NMS	1.536006	3.726551	2.263123	1.64664	0.099632	0.164486	0	0	0.695731	2.232127	4.278221	2.009959
16100	LOC11962(	1.536006	3.726551	2.263123	1.64664	0.099632	0.164486	0	0	0.695731	2.232127	4.278221	2.009959
16101	PGLYRP1	1.536006	3.726551	2.263123	1.64664	0.099632	0.164486	0	0	0.695731	2.232127	4.278221	2.009959
16102	SPACA4	1.536006	3.726551	2.263123	1.64664	0.099632	0.164486	0	0	0.695731	2.232127	4.278221	2.009959
16103	LOC10322:	1.536006	3.726551	2.263123	1.64664	0.099632	0.164486	0	0	0.695731	2.232127	4.278221	2.009959
17010	LOC10323‹	1.547427	3.726541	2.49978	1.490747	0.136028	0.212598	0.542086	0	0	4.464254	4.278221	0
17008	OXGR1	1.548315	3.726517	2.499363	1.490987	0.135965	0.212525	0	0.547412	0	4.464254	4.278221	0
14271	LOC11962!	2.605726	3.726049	1.895123	1.966126	0.049284	0.09181	1.084173	0	0	2.232127	4.278221	8.039836
16961	LOC11962;	1.573034	3.725845	2.487892	1.497591	0.13424	0.21036	0	0	0.695731	4.464254	4.278221	0
16962	PSAPL1	1.573034	3.725845	2.487892	1.497591	0.13424	0.21036	0	0	0.695731	4.464254	4.278221	0
16963	LOC11961‹	1.573034	3.725845	2.487892	1.497591	0.13424	0.21036	0	0	0.695731	4.464254	4.278221	0
16964	ZNF232	1.573034	3.725845	2.487892	1.497591	0.13424	0.21036	0	0	0.695731	4.464254	4.278221	0
16965	LOC11961ε	1.573034	3.725845	2.487892	1.497591	0.13424	0.21036	0	0	0.695731	4.464254	4.278221	0
11710	FGF23	4.654527	3.725005	1.508408	2.469494	0.01353	0.030718	0	0	2.087194	13.39276	6.417332	6.029877
1847	LOC10321ε	51.37857	3.724532	0.447793	8.317527	8.98E-17	1.29E-15	4.336691	10.94824	6.261582	87.05295	111.2338	88.43819
15363	LOC10322:	2.755613	3.724354	2.108034	1.766743	0.077271	0.133706	0	1.094824	0	11.16063	4.278221	0
11211	GRIN3A	7.986161	3.724287	1.443348	2.580311	0.009871	0.023408	0	0	3.478657	15.62489	27.80844	1.004979
4954	SLC6A7	30.50869	3.722621	0.790056	4.711844	2.45E-06	1.32E-05	0	2.737059	10.43597	64.73168	77.00798	28.13943
11910	CXCR1	4.691555	3.721855	1.536174	2.422807	0.015401	0.034378	0	0	2.087194	15.62489	6.417332	4.019918
13716	UBD	3.034985	3.720034	1.800479	2.066136	0.038816	0.075197	0	0	1.391463	2.232127	8.556443	6.029877
6195	LHX5	24.97488	3.719992	0.912684	4.075884	4.58E-05	0.000197	0	1.094824	9.740239	51.33892	70.59065	17.08465
13682	LOC11961‹	3.124544	3.719728	1.795031	2.072237	0.038243	0.074304	0	0	1.391463	8.928508	6.417332	2.009959
13683	LOC10322(	3.124544	3.719728	1.795031	2.072237	0.038243	0.074304	0	0	1.391463	8.928508	6.417332	2.009959
13539	LOC10322:	3.072013	3.719385	1.770709	2.100506	0.035684	0.070059	0	0	1.391463	4.464254	8.556443	4.019918
13540	HIGD1B	3.072013	3.719385	1.770709	2.100506	0.035684	0.070059	0	0	1.391463	4.464254	8.556443	4.019918
13541	LOC11962:	3.072013	3.719385	1.770709	2.100506	0.035684	0.070059	0	0	1.391463	4.464254	8.556443	4.019918
26813	LOC11962:	0.707014	3.718531	3.517506	1.05715	0.290443	NA	0	0	0	2.232127	0	2.009959
27164	LOC10323(	0.707014	3.718531	3.517506	1.05715	0.290443	NA	0	0	0	2.232127	0	2.009959
27423	GP9	0.707014	3.718531	3.517506	1.05715	0.290443	NA	0	0	0	2.232127	0	2.009959
27501	LOC10322(	0.707014	3.718531	3.517506	1.05715	0.290443	NA	0	0	0	2.232127	0	2.009959
27689	MYMX	0.707014	3.718531	3.517506	1.05715	0.290443	NA	0	0	0	2.232127	0	2.009959
27811	LOC11962;	0.707014	3.718531	3.517506	1.05715	0.290443	NA	0	0	0	2.232127	0	2.009959
27873	LOC11962;	0.707014	3.718531	3.517506	1.05715	0.290443	NA	0	0	0	2.232127	0	2.009959
27968	LOC11962ε	0.707014	3.718531	3.517506	1.05715	0.290443	NA	0	0	0	2.232127	0	2.009959
28004	LOC10322:	0.707014	3.718531	3.517506	1.05715	0.290443	NA	0	0	0	2.232127	0	2.009959
28012	LOC10322‹	0.707014	3.718531	3.517506	1.05715	0.290443	NA	0	0	0	2.232127	0	2.009959

28135	PTF1A	0.707014	3.718531	3.517506	1.05715	0.290443	NA	0	0	0	2.232127	0	2.009959
28286	LOC10322(	0.707014	3.718531	3.517506	1.05715	0.290443	NA	0	0	0	2.232127	0	2.009959
28679	LOC10321!	0.707014	3.718531	3.517506	1.05715	0.290443	NA	0	0	0	2.232127	0	2.009959
28838	LOC10321{	0.707014	3.718531	3.517506	1.05715	0.290443	NA	0	0	0	2.232127	0	2.009959
29058	LOC10324:	0.707014	3.718531	3.517506	1.05715	0.290443	NA	0	0	0	2.232127	0	2.009959
29128	LOC11962;	0.707014	3.718531	3.517506	1.05715	0.290443	NA	0	0	0	2.232127	0	2.009959
29138	LOC10323:	0.707014	3.718531	3.517506	1.05715	0.290443	NA	0	0	0	2.232127	0	2.009959
29158	PRR27	0.707014	3.718531	3.517506	1.05715	0.290443	NA	0	0	0	2.232127	0	2.009959
29208	LOC10322(	0.707014	3.718531	3.517506	1.05715	0.290443	NA	0	0	0	2.232127	0	2.009959
29357	LOC10322:	0.707014	3.718531	3.517506	1.05715	0.290443	NA	0	0	0	2.232127	0	2.009959
16926	LOC11962(	1.520504	3.718063	2.474358	1.502637	0.132933	0.208792	0	0	0.695731	0	6.417332	2.009959
13387	LOC11961{	2.841881	3.71666	1.745217	2.129626	0.033202	0.065926	0.542086	0	0.695731	2.232127	8.556443	5.024897
8956	CCDC60	7.571352	3.716554	1.187573	3.129537	0.001751	0.005197	0	0.547412	2.782925	20.08914	14.97377	7.034856
15333	LOC10324;	2.722833	3.716324	2.095871	1.773164	0.076201	0.132127	1.084173	0	0	6.696381	8.556443	0
11247	LOC10323(	4.233066	3.715952	1.445417	2.570851	0.010145	0.02398	0	1.094824	0.695731	11.16063	6.417332	6.029877
14749	KLHL33	16.20571	3.715551	1.974985	1.881306	0.05993	0.108024	0	0	6.957314	29.01765	49.19955	12.05975
17208	LOC10322:	1.531924	3.71549	2.533032	1.466815	0.142426	0.219999	0.542086	0	0	2.232127	6.417332	0
17209	LOC10322:	1.531924	3.71549	2.533032	1.466815	0.142426	0.219999	0.542086	0	0	2.232127	6.417332	0
17210	LOC11962(	1.531924	3.71549	2.533032	1.466815	0.142426	0.219999	0.542086	0	0	2.232127	6.417332	0
17205	LOC10323(	1.532812	3.715468	2.532609	1.467052	0.142362	0.219951	0	0.547412	0	2.232127	6.417332	0
17206	SLAMF1	1.532812	3.715468	2.532609	1.467052	0.142362	0.219951	0	0.547412	0	2.232127	6.417332	0
17207	LOC11962;	1.532812	3.715468	2.532609	1.467052	0.142362	0.219951	0	0.547412	0	2.232127	6.417332	0
5130	HCN4	29.09209	3.715444	0.8053	4.613738	3.95E-06	2.05E-05	0.542086	1.642235	10.43597	66.96381	74.86887	20.09959
7084	AGBL1	10.34006	3.71544	1.003707	3.701717	0.000214	0.000804	0	1.094824	3.478657	17.85702	23.53022	16.07967
5177	CLVS1	16.00895	3.715002	0.809199	4.590964	4.41E-06	2.27E-05	2.168345	0.547412	4.174388	35.71403	36.36488	17.08465
17144	NEUROG1	1.557532	3.714851	2.52096	1.473585	0.140593	0.217915	0	0	0.695731	2.232127	6.417332	0
17145	LOC11962⁄	1.557532	3.714851	2.52096	1.473585	0.140593	0.217915	0	0	0.695731	2.232127	6.417332	0
17146	LOC10322:	1.557532	3.714851	2.52096	1.473585	0.140593	0.217915	0	0	0.695731	2.232127	6.417332	0
17147	SLC22A1	1.557532	3.714851	2.52096	1.473585	0.140593	0.217915	0	0	0.695731	2.232127	6.417332	0
17148	LOC11962!	1.557532	3.714851	2.52096	1.473585	0.140593	0.217915	0	0	0.695731	2.232127	6.417332	0
17149	RNF113B	1.557532	3.714851	2.52096	1.473585	0.140593	0.217915	0	0	0.695731	2.232127	6.417332	0
17150	CCDC172	1.557532	3.714851	2.52096	1.473585	0.140593	0.217915	0	0	0.695731	2.232127	6.417332	0
17151	TEX36	1.557532	3.714851	2.52096	1.473585	0.140593	0.217915	0	0	0.695731	2.232127	6.417332	0
7190	HSD17B6	8.449743	3.714675	1.012391	3.66921	0.000243	0.0009	0.542086	1.642235	1.391463	20.08914	14.97377	12.05975
11374	BTN1A1	4.467446	3.713661	1.460343	2.543006	0.01099	0.025688	0.542086	0	1.391463	11.16063	10.69555	3.014938
13705	LOC10322;	3.109041	3.713486	1.796272	2.06733	0.038703	0.075071	0	0	1.391463	6.696381	8.556443	2.009959
13706	LOC11962;	3.109041	3.713486	1.796272	2.06733	0.038703	0.075071	0	0	1.391463	6.696381	8.556443	2.009959
13397	SLCO1B3	2.842768	3.713356	1.744881	2.128143	0.033325	0.066131	0	0.547412	0.695731	2.232127	8.556443	5.024897
12220	ASCL2	4.697578	3.711084	1.570866	2.362445	0.018155	0.039496	0	0	2.087194	13.39276	10.69555	2.009959
11024	LRRC25	4.269207	3.710831	1.413923	2.624494	0.008678	0.02093	0.542086	0.547412	0.695731	13.39276	6.417332	4.019918
6674	LOC10323;	11.56569	3.710577	0.959393	3.867632	0.00011	0.000438	0	2.189647	2.782925	24.5534	27.80844	12.05975
6566	TREML2	15.01418	3.709604	0.947771	3.91403	9.08E-05	0.000368	0.542086	1.094824	4.87012	40.17829	36.36488	7.034856
8064	LOC10322(	8.884136	3.709419	1.09248	3.395411	0.000685	0.002259	0	1.094824	2.782925	17.85702	23.53022	8.039836
13836	AMBN	3.05651	3.709201	1.815939	2.042581	0.041094	0.078959	0	0	1.391463	2.232127	10.69555	4.019918
13626	LOC11961{	2.628139	3.708665	1.7798	2.083754	0.037183	0.072545	0.542086	0.547412	0	2.232127	6.417332	6.029877
7239	CYP4F3	10.1909	3.708512	1.014505	3.655487	0.000257	0.000943	0	1.642235	2.782925	17.85702	27.80844	11.05477
13314	LOC11962;	2.878909	3.708218	1.72929	2.144358	0.032004	0.063905	0.542086	0	0.695731	4.464254	8.556443	3.014938
5518	LOC10323;	24.37105	3.707604	0.841837	4.404184	1.06E-05	5.11E-05	0	1.642235	9.044508	55.80317	55.61688	24.11951
11381	LOC11961(	4.254592	3.707088	1.458389	2.541907	0.011025	0.025754	0	1.094824	0.695731	11.16063	8.556443	4.019918
11450	LOC11962⁄	4.4003	3.706392	1.467012	2.52649	0.011521	0.02675	0	0.547412	1.391463	4.464254	14.97377	5.024897
13839	RTP2	3.093538	3.706231	1.815269	2.041698	0.041181	0.079105	0	0	1.391463	4.464254	10.69555	2.009959
10948	AGBL4	4.253704	3.705963	1.403997	2.63958	0.008301	0.020157	0.542086	0.547412	0.695731	11.16063	8.556443	4.019918
396	PPP1R32	193.5744	3.705235	0.235442	15.73735	8.39E-56	5.63E-54	29.27266	21.34906	32.69937	350.4439	350.8141	376.8673
13327	ASB9	2.879796	3.704982	1.728951	2.142907	0.032121	0.064072	0	0.547412	0.695731	4.464254	8.556443	3.014938
9644	SIGLEC11	7.695535	3.704266	1.256097	2.949028	0.003188	0.008787	0	0	3.478657	13.39276	19.252	10.04979
231	TMEM140	355.6085	3.703983	0.19498	18.99675	1.81E-80	2.09E-78	52.04029	42.69812	58.44144	640.6204	586.1163	753.7346
7023	TTC22	11.73293	3.70375	0.994074	3.725828	0.000195	0.000737	0	1.642235	3.478657	17.85702	36.36488	11.05477
11481	ATP2C2	4.186558	3.703559	1.469097	2.520976	0.011703	0.027099	0	1.094824	0.695731	4.464254	12.83466	6.029877
9700	GJC3	7.583397	3.702783	1.264041	2.929321	0.003397	0.00931	0	0.547412	2.782925	15.62489	23.53022	3.014938
13586	LOC10323(	2.863406	3.702326	1.769858	2.091877	0.03645	0.071322	0.542086	0	0.695731	2.232127	10.69555	3.014938
11791	LOC11962;	4.258839	3.702134	1.510137	2.451523	0.014225	0.032074	1.084173	0	0.695731	8.928508	12.83466	2.009959
12301	LOC10321{	4.666573	3.701999	1.579253	2.344146	0.019071	0.041216	0	0	2.087194	8.928508	14.97377	2.009959
5372	TMIE	12.63259	3.701669	0.826317	4.479721	7.47E-06	3.70E-05	1.084173	1.642235	2.782925	26.78552	21.39111	22.10955
11756	GCSAM	4.420938	3.700679	1.50577	2.457666	0.013984	0.031624	0.542086	0	1.391463	4.464254	17.11289	3.014938
14046	MMP20	2.666055	3.699932	1.844746	2.005659	0.044893	0.084969	0	1.094824	0	4.464254	6.417332	4.019918
9778	CPNE6	7.567894	3.699848	1.271774	2.909202	0.003624	0.009852	0	0.547412	2.782925	13.39276	25.66933	3.014938
11840	IL23R	4.171056	3.699613	1.51684	2.439026	0.014727	0.033067	0	1.094824	0.695731	2.232127	14.97377	6.029877
13593	LOC10323(	2.864294	3.698961	1.769516	2.09038	0.036584	0.071534	0	0.547412	0.695731	2.232127	10.69555	3.014938
13595	REM1	2.864294	3.698961	1.769516	2.09038	0.036584	0.071534	0	0.547412	0.695731	2.232127	10.69555	3.014938
13596	LOC10322{	2.864294	3.698961	1.769516	2.09038	0.036584	0.071534	0	0.547412	0.695731	2.232127	10.69555	3.014938
10340	A4GNT	5.728587	3.698954	1.330902	2.779283	0.005448	0.014007	0	1.094824	1.391463	4.464254	21.39111	6.029877
6679	LOC10323{	16.83301	3.698458	0.956705	3.865829	0.000111	0.000441	0.542086	0	6.957314	40.17829	34.22577	19.09461
9556	FMR1NB	7.478336	3.697446	1.244426	2.971207	0.002966	0.008252	0	0.547412	2.782925	6.696381	27.80844	7.034856
14154	LOC11961(	3.078035	3.697235	1.862713	1.984865	0.047159	0.088572	0	0	1.391463	2.232127	12.83466	2.009959

14155	GNG13	3.078035	3.697235	1.862713	1.984865	0.047159	0.088572	0	0	1.391463	2.232127	12.83466	2.009959
14199	LOC103246	2.703083	3.695499	1.869156	1.977095	0.048031	0.089929	0	1.094824	0	6.696381	6.417332	2.009959
19835	NR0B2	0.72854	3.695342	3.535464	1.045221	0.295921	0.395012	0	0	0	2.232127	2.139111	0
19836	LOC119625	0.72854	3.695342	3.535464	1.045221	0.295921	0.395012	0	0	0	2.232127	2.139111	0
19837	LOC119626	0.72854	3.695342	3.535464	1.045221	0.295921	0.395012	0	0	0	2.232127	2.139111	0
19838	HMHB1	0.72854	3.695342	3.535464	1.045221	0.295921	0.395012	0	0	0	2.232127	2.139111	0
19839	LOC103219	0.72854	3.695342	3.535464	1.045221	0.295921	0.395012	0	0	0	2.232127	2.139111	0
19842	LOC119623	0.72854	3.695342	3.535464	1.045221	0.295921	0.395012	0	0	0	2.232127	2.139111	0
19844	LOC103239	0.72854	3.695342	3.535464	1.045221	0.295921	0.395012	0	0	0	2.232127	2.139111	0
19845	LOC119623	0.72854	3.695342	3.535464	1.045221	0.295921	0.395012	0	0	0	2.232127	2.139111	0
19846	LOC103238	0.72854	3.695342	3.535464	1.045221	0.295921	0.395012	0	0	0	2.232127	2.139111	0
19847	LOC119623	0.72854	3.695342	3.535464	1.045221	0.295921	0.395012	0	0	0	2.232127	2.139111	0
19848	LOC119624	0.72854	3.695342	3.535464	1.045221	0.295921	0.395012	0	0	0	2.232127	2.139111	0
19849	LOC119624	0.72854	3.695342	3.535464	1.045221	0.295921	0.395012	0	0	0	2.232127	2.139111	0
19851	IFITM5	0.72854	3.695342	3.535464	1.045221	0.295921	0.395012	0	0	0	2.232127	2.139111	0
19852	MS4A12	0.72854	3.695342	3.535464	1.045221	0.295921	0.395012	0	0	0	2.232127	2.139111	0
19853	LOC103242	0.72854	3.695342	3.535464	1.045221	0.295921	0.395012	0	0	0	2.232127	2.139111	0
19854	LOC103235	0.72854	3.695342	3.535464	1.045221	0.295921	0.395012	0	0	0	2.232127	2.139111	0
19855	LOC103237	0.72854	3.695342	3.535464	1.045221	0.295921	0.395012	0	0	0	2.232127	2.139111	0
19856	LOC119624	0.72854	3.695342	3.535464	1.045221	0.295921	0.395012	0	0	0	2.232127	2.139111	0
19857	LOC119624	0.72854	3.695342	3.535464	1.045221	0.295921	0.395012	0	0	0	2.232127	2.139111	0
19858	LOC119624	0.72854	3.695342	3.535464	1.045221	0.295921	0.395012	0	0	0	2.232127	2.139111	0
19860	LOC119626	0.72854	3.695342	3.535464	1.045221	0.295921	0.395012	0	0	0	2.232127	2.139111	0
19863	LOC119626	0.72854	3.695342	3.535464	1.045221	0.295921	0.395012	0	0	0	2.232127	2.139111	0
19864	IQCF2	0.72854	3.695342	3.535464	1.045221	0.295921	0.395012	0	0	0	2.232127	2.139111	0
19865	LOC119626	0.72854	3.695342	3.535464	1.045221	0.295921	0.395012	0	0	0	2.232127	2.139111	0
19866	LOC119626	0.72854	3.695342	3.535464	1.045221	0.295921	0.395012	0	0	0	2.232127	2.139111	0
19867	LOC103248	0.72854	3.695342	3.535464	1.045221	0.295921	0.395012	0	0	0	2.232127	2.139111	0
19868	LOC103247	0.72854	3.695342	3.535464	1.045221	0.295921	0.395012	0	0	0	2.232127	2.139111	0
19869	LOC103247	0.72854	3.695342	3.535464	1.045221	0.295921	0.395012	0	0	0	2.232127	2.139111	0
19870	LOC119627	0.72854	3.695342	3.535464	1.045221	0.295921	0.395012	0	0	0	2.232127	2.139111	0
19871	LOC103233	0.72854	3.695342	3.535464	1.045221	0.295921	0.395012	0	0	0	2.232127	2.139111	0
19873	LOC103216	0.72854	3.695342	3.535464	1.045221	0.295921	0.395012	0	0	0	2.232127	2.139111	0
19874	LOC119628	0.72854	3.695342	3.535464	1.045221	0.295921	0.395012	0	0	0	2.232127	2.139111	0
19876	LOC119618	0.72854	3.695342	3.535464	1.045221	0.295921	0.395012	0	0	0	2.232127	2.139111	0
19877	LOC119618	0.72854	3.695342	3.535464	1.045221	0.295921	0.395012	0	0	0	2.232127	2.139111	0
19878	BHLHE23	0.72854	3.695342	3.535464	1.045221	0.295921	0.395012	0	0	0	2.232127	2.139111	0
19879	LOC119618	0.72854	3.695342	3.535464	1.045221	0.295921	0.395012	0	0	0	2.232127	2.139111	0
19880	TMEM271	0.72854	3.695342	3.535464	1.045221	0.295921	0.395012	0	0	0	2.232127	2.139111	0
19881	LOC103237	0.72854	3.695342	3.535464	1.045221	0.295921	0.395012	0	0	0	2.232127	2.139111	0
19883	LOC119618	0.72854	3.695342	3.535464	1.045221	0.295921	0.395012	0	0	0	2.232127	2.139111	0
19884	LOC119619	0.72854	3.695342	3.535464	1.045221	0.295921	0.395012	0	0	0	2.232127	2.139111	0
19885	LOC119619	0.72854	3.695342	3.535464	1.045221	0.295921	0.395012	0	0	0	2.232127	2.139111	0
19887	LOC119619	0.72854	3.695342	3.535464	1.045221	0.295921	0.395012	0	0	0	2.232127	2.139111	0
19888	LOC103230	0.72854	3.695342	3.535464	1.045221	0.295921	0.395012	0	0	0	2.232127	2.139111	0
19889	LOC119619	0.72854	3.695342	3.535464	1.045221	0.295921	0.395012	0	0	0	2.232127	2.139111	0
19890	LOC103231	0.72854	3.695342	3.535464	1.045221	0.295921	0.395012	0	0	0	2.232127	2.139111	0
19891	LOC119620	0.72854	3.695342	3.535464	1.045221	0.295921	0.395012	0	0	0	2.232127	2.139111	0
19892	LOC119620	0.72854	3.695342	3.535464	1.045221	0.295921	0.395012	0	0	0	2.232127	2.139111	0
19894	LOC103231	0.72854	3.695342	3.535464	1.045221	0.295921	0.395012	0	0	0	2.232127	2.139111	0
19895	LOC103244	0.72854	3.695342	3.535464	1.045221	0.295921	0.395012	0	0	0	2.232127	2.139111	0
19896	LOC119620	0.72854	3.695342	3.535464	1.045221	0.295921	0.395012	0	0	0	2.232127	2.139111	0
19897	LOC119620	0.72854	3.695342	3.535464	1.045221	0.295921	0.395012	0	0	0	2.232127	2.139111	0
19898	LOC119620	0.72854	3.695342	3.535464	1.045221	0.295921	0.395012	0	0	0	2.232127	2.139111	0
19899	LOC103247	0.72854	3.695342	3.535464	1.045221	0.295921	0.395012	0	0	0	2.232127	2.139111	0
19900	LOC119620	0.72854	3.695342	3.535464	1.045221	0.295921	0.395012	0	0	0	2.232127	2.139111	0
19901	LOC103235	0.72854	3.695342	3.535464	1.045221	0.295921	0.395012	0	0	0	2.232127	2.139111	0
19902	CPXCR1	0.72854	3.695342	3.535464	1.045221	0.295921	0.395012	0	0	0	2.232127	2.139111	0
19903	LOC119621	0.72854	3.695342	3.535464	1.045221	0.295921	0.395012	0	0	0	2.232127	2.139111	0
19904	KRTAP13-1	0.72854	3.695342	3.535464	1.045221	0.295921	0.395012	0	0	0	2.232127	2.139111	0
19905	LOC119621	0.72854	3.695342	3.535464	1.045221	0.295921	0.395012	0	0	0	2.232127	2.139111	0
19906	LOC103234	0.72854	3.695342	3.535464	1.045221	0.295921	0.395012	0	0	0	2.232127	2.139111	0
19908	LOC119622	0.72854	3.695342	3.535464	1.045221	0.295921	0.395012	0	0	0	2.232127	2.139111	0
19909	LOC103241	0.72854	3.695342	3.535464	1.045221	0.295921	0.395012	0	0	0	2.232127	2.139111	0
19911	LOC103234	0.72854	3.695342	3.535464	1.045221	0.295921	0.395012	0	0	0	2.232127	2.139111	0
19912	TAS2R13	0.72854	3.695342	3.535464	1.045221	0.295921	0.395012	0	0	0	2.232127	2.139111	0
19913	LOC103247	0.72854	3.695342	3.535464	1.045221	0.295921	0.395012	0	0	0	2.232127	2.139111	0
19914	LOC119623	0.72854	3.695342	3.535464	1.045221	0.295921	0.395012	0	0	0	2.232127	2.139111	0
19915	LOC119623	0.72854	3.695342	3.535464	1.045221	0.295921	0.395012	0	0	0	2.232127	2.139111	0
19916	LOC119623	0.72854	3.695342	3.535464	1.045221	0.295921	0.395012	0	0	0	2.232127	2.139111	0
26979	LOC103219	0.669986	3.695061	4.01694	0.91987	0.357641	NA	0	0	0	0	0	4.019918
27036	OOSP4B	0.669986	3.695061	4.01694	0.91987	0.357641	NA	0	0	0	0	0	4.019918
27120	SLURP1	0.669986	3.695061	4.01694	0.91987	0.357641	NA	0	0	0	0	0	4.019918
27489	LOC103226	0.669986	3.695061	4.01694	0.91987	0.357641	NA	0	0	0	0	0	4.019918

27922	LOC119627	0.669986	3.695061	4.01694	0.91987	0.357641	NA	0	0	0	0	0	4.019918
28304	ACTRT2	0.669986	3.695061	4.01694	0.91987	0.357641	NA	0	0	0	0	0	4.019918
28898	LOC11962:	0.669986	3.695061	4.01694	0.91987	0.357641	NA	0	0	0	0	0	4.019918
26809	LOC119622	0.691512	3.693318	3.545337	1.041739	0.297533	NA	0	0	0	0	2.139111	2.009959
26838	LOC119624	0.691512	3.693318	3.545337	1.041739	0.297533	NA	0	0	0	0	2.139111	2.009959
27047	GLYAT	0.691512	3.693318	3.545337	1.041739	0.297533	NA	0	0	0	0	2.139111	2.009959
27212	LOC119625	0.691512	3.693318	3.545337	1.041739	0.297533	NA	0	0	0	0	2.139111	2.009959
27270	LOC119625	0.691512	3.693318	3.545337	1.041739	0.297533	NA	0	0	0	0	2.139111	2.009959
27474	PAX4	0.691512	3.693318	3.545337	1.041739	0.297533	NA	0	0	0	0	2.139111	2.009959
27589	LOC103247	0.691512	3.693318	3.545337	1.041739	0.297533	NA	0	0	0	0	2.139111	2.009959
27700	ZIM2	0.691512	3.693318	3.545337	1.041739	0.297533	NA	0	0	0	0	2.139111	2.009959
27799	CUNH22or	0.691512	3.693318	3.545337	1.041739	0.297533	NA	0	0	0	0	2.139111	2.009959
27853	LOC119627	0.691512	3.693318	3.545337	1.041739	0.297533	NA	0	0	0	0	2.139111	2.009959
28203	LOC119619	0.691512	3.693318	3.545337	1.041739	0.297533	NA	0	0	0	0	2.139111	2.009959
28218	TRNAA-AG	0.691512	3.693318	3.545337	1.041739	0.297533	NA	0	0	0	0	2.139111	2.009959
28269	LOC119619	0.691512	3.693318	3.545337	1.041739	0.297533	NA	0	0	0	0	2.139111	2.009959
28278	S100B	0.691512	3.693318	3.545337	1.041739	0.297533	NA	0	0	0	0	2.139111	2.009959
28303	LOC119619	0.691512	3.693318	3.545337	1.041739	0.297533	NA	0	0	0	0	2.139111	2.009959
28415	LOC103230	0.691512	3.693318	3.545337	1.041739	0.297533	NA	0	0	0	0	2.139111	2.009959
28462	LOC10323:	0.691512	3.693318	3.545337	1.041739	0.297533	NA	0	0	0	0	2.139111	2.009959
28548	LOC119620	0.691512	3.693318	3.545337	1.041739	0.297533	NA	0	0	0	0	2.139111	2.009959
28600	LOC10323:	0.691512	3.693318	3.545337	1.041739	0.297533	NA	0	0	0	0	2.139111	2.009959
28643	LOC103218	0.691512	3.693318	3.545337	1.041739	0.297533	NA	0	0	0	0	2.139111	2.009959
28665	LOC119620	0.691512	3.693318	3.545337	1.041739	0.297533	NA	0	0	0	0	2.139111	2.009959
28831	ART4	0.691512	3.693318	3.545337	1.041739	0.297533	NA	0	0	0	0	2.139111	2.009959
28843	LOC11962:	0.691512	3.693318	3.545337	1.041739	0.297533	NA	0	0	0	0	2.139111	2.009959
28858	KRTAP7-1	0.691512	3.693318	3.545337	1.041739	0.297533	NA	0	0	0	0	2.139111	2.009959
29126	LOC10324:	0.691512	3.693318	3.545337	1.041739	0.297533	NA	0	0	0	0	2.139111	2.009959
29199	LOC103247	0.691512	3.693318	3.545337	1.041739	0.297533	NA	0	0	0	0	2.139111	2.009959
13785	LOC119620	2.901322	3.692932	1.802249	2.049069	0.040455	0.07802	0	0.547412	0.695731	4.464254	10.69555	1.004979
9909	LOC103214	9.348648	3.692304	1.28082	2.882766	0.003942	0.010575	0	0	4.174388	20.08914	27.80844	4.019918
9910	TRIM72	9.348648	3.692304	1.28082	2.882766	0.003942	0.010575	0	0	4.174388	20.08914	27.80844	4.019918
5805	ASTN1	12.8799	3.690088	0.864015	4.270862	1.95E-05	8.92E-05	1.084173	1.094824	3.478657	22.32127	34.22577	15.07469
14073	ENAM	2.885819	3.687973	1.844866	1.999047	0.045603	0.086148	0	0.547412	0.695731	2.232127	12.83466	1.004979
6746	KCNA2	9.853505	3.687233	0.960399	3.839271	0.000123	0.000486	1.626259	0.547412	2.087194	24.5534	19.252	11.05477
8882	LOC119620	14.06173	3.687016	1.171078	3.148396	0.001642	0.004915	0	0	6.261582	35.71403	36.36488	6.029877
3458	FER1L4	45.09969	3.68633	0.625201	5.896236	3.72E-09	2.86E-08	4.336691	2.189647	13.2189	93.74933	121.9293	35.17428
8843	LOC119626	9.290676	3.686088	1.167149	3.158198	0.001587	0.004772	0.542086	3.284471	0	20.08914	27.80844	4.019918
7075	PROP1	15.1769	3.685563	0.995096	3.703726	0.000212	0.000798	0	0.547412	6.261582	44.64254	23.53022	16.07967
7149	RNF222	13.41244	3.685298	1.001178	3.680962	0.000232	0.000864	1.084173	0	4.87012	31.24978	34.22577	9.044815
8195	FAM151A	8.864918	3.684348	1.100491	3.347913	0.000814	0.002641	0.542086	0	3.478657	17.85702	19.252	12.05975
8779	LOC103234	6.936681	3.682804	1.158244	3.179644	0.001475	0.004465	1.626259	0	1.391463	17.85702	10.69555	10.04979
7874	ARHGEF16	10.09479	3.682623	1.068322	3.447109	0.000567	0.001913	1.626259	0	2.782925	22.32127	27.80844	6.029877
6451	LOC119622	12.32566	3.681697	0.929826	3.959553	7.51E-05	0.000309	0.542086	3.284471	1.391463	22.32127	36.36488	10.04979
116	RASGEF1B	692.9708	3.68165	0.155202	23.72167	2.15E-124	4.94E-122	81.31296	112.7668	107.1426	1330.348	1210.737	1315.518
26598	LOC119626	0.713037	3.680016	4.018067	0.915867	0.359737	NA	0	0	0	0	4.278221	0
26625	LOC119624	0.713037	3.680016	4.018067	0.915867	0.359737	NA	0	0	0	0	4.278221	0
26648	LOC119626	0.713037	3.680016	4.018067	0.915867	0.359737	NA	0	0	0	0	4.278221	0
26672	LOC119618	0.713037	3.680016	4.018067	0.915867	0.359737	NA	0	0	0	0	4.278221	0
26685	CUNH5orf4	0.713037	3.680016	4.018067	0.915867	0.359737	NA	0	0	0	0	4.278221	0
26756	LOC119624	0.713037	3.680016	4.018067	0.915867	0.359737	NA	0	0	0	0	4.278221	0
26775	LOC119624	0.713037	3.680016	4.018067	0.915867	0.359737	NA	0	0	0	0	4.278221	0
26841	LOC119624	0.713037	3.680016	4.018067	0.915867	0.359737	NA	0	0	0	0	4.278221	0
26844	LOC119624	0.713037	3.680016	4.018067	0.915867	0.359737	NA	0	0	0	0	4.278221	0
26868	LOC119623	0.713037	3.680016	4.018067	0.915867	0.359737	NA	0	0	0	0	4.278221	0
26881	LOC103238	0.713037	3.680016	4.018067	0.915867	0.359737	NA	0	0	0	0	4.278221	0
26911	FCER1A	0.713037	3.680016	4.018067	0.915867	0.359737	NA	0	0	0	0	4.278221	0
26920	LOC103223	0.713037	3.680016	4.018067	0.915867	0.359737	NA	0	0	0	0	4.278221	0
26921	LOC103223	0.713037	3.680016	4.018067	0.915867	0.359737	NA	0	0	0	0	4.278221	0
26964	LOC119624	0.713037	3.680016	4.018067	0.915867	0.359737	NA	0	0	0	0	4.278221	0
27074	LOC103235	0.713037	3.680016	4.018067	0.915867	0.359737	NA	0	0	0	0	4.278221	0
27093	LOC103242	0.713037	3.680016	4.018067	0.915867	0.359737	NA	0	0	0	0	4.278221	0
27111	LOC10324:	0.713037	3.680016	4.018067	0.915867	0.359737	NA	0	0	0	0	4.278221	0
27150	FABP9	0.713037	3.680016	4.018067	0.915867	0.359737	NA	0	0	0	0	4.278221	0
27178	PPDPFL	0.713037	3.680016	4.018067	0.915867	0.359737	NA	0	0	0	0	4.278221	0
27191	TAAR5	0.713037	3.680016	4.018067	0.915867	0.359737	NA	0	0	0	0	4.278221	0
27199	LOC119625	0.713037	3.680016	4.018067	0.915867	0.359737	NA	0	0	0	0	4.278221	0
27267	LOC119625	0.713037	3.680016	4.018067	0.915867	0.359737	NA	0	0	0	0	4.278221	0
27278	LOC119625	0.713037	3.680016	4.018067	0.915867	0.359737	NA	0	0	0	0	4.278221	0
27352	LOC119625	0.713037	3.680016	4.018067	0.915867	0.359737	NA	0	0	0	0	4.278221	0
27361	LOC119625	0.713037	3.680016	4.018067	0.915867	0.359737	NA	0	0	0	0	4.278221	0
27464	LYG1	0.713037	3.680016	4.018067	0.915867	0.359737	NA	0	0	0	0	4.278221	0
27516	LOC119626	0.713037	3.680016	4.018067	0.915867	0.359737	NA	0	0	0	0	4.278221	0
27543	LOC103248	0.713037	3.680016	4.018067	0.915867	0.359737	NA	0	0	0	0	4.278221	0

27582	LOC103247	0.713037	3.680016	4.018067	0.915867	0.359737	NA	0	0	0	0	4.278221	0
27583	LOC103247	0.713037	3.680016	4.018067	0.915867	0.359737	NA	0	0	0	0	4.278221	0
27616	LOC119626	0.713037	3.680016	4.018067	0.915867	0.359737	NA	0	0	0	0	4.278221	0
27635	TRNAV-CA	0.713037	3.680016	4.018067	0.915867	0.359737	NA	0	0	0	0	4.278221	0
27688	LOC119626	0.713037	3.680016	4.018067	0.915867	0.359737	NA	0	0	0	0	4.278221	0
27705	LOC119627	0.713037	3.680016	4.018067	0.915867	0.359737	NA	0	0	0	0	4.278221	0
27768	LOC119627	0.713037	3.680016	4.018067	0.915867	0.359737	NA	0	0	0	0	4.278221	0
27920	LOC119628	0.713037	3.680016	4.018067	0.915867	0.359737	NA	0	0	0	0	4.278221	0
27934	LIPF	0.713037	3.680016	4.018067	0.915867	0.359737	NA	0	0	0	0	4.278221	0
27936	CH25H	0.713037	3.680016	4.018067	0.915867	0.359737	NA	0	0	0	0	4.278221	0
28043	LOC119618	0.713037	3.680016	4.018067	0.915867	0.359737	NA	0	0	0	0	4.278221	0
28066	MTRNR2L3	0.713037	3.680016	4.018067	0.915867	0.359737	NA	0	0	0	0	4.278221	0
28086	LOC103245	0.713037	3.680016	4.018067	0.915867	0.359737	NA	0	0	0	0	4.278221	0
28155	LOC119618	0.713037	3.680016	4.018067	0.915867	0.359737	NA	0	0	0	0	4.278221	0
28156	LOC119618	0.713037	3.680016	4.018067	0.915867	0.359737	NA	0	0	0	0	4.278221	0
28284	LOC119618	0.713037	3.680016	4.018067	0.915867	0.359737	NA	0	0	0	0	4.278221	0
28285	LOC103229	0.713037	3.680016	4.018067	0.915867	0.359737	NA	0	0	0	0	4.278221	0
28374	LOC119619	0.713037	3.680016	4.018067	0.915867	0.359737	NA	0	0	0	0	4.278221	0
28388	LOC103230	0.713037	3.680016	4.018067	0.915867	0.359737	NA	0	0	0	0	4.278221	0
28390	LOC103230	0.713037	3.680016	4.018067	0.915867	0.359737	NA	0	0	0	0	4.278221	0
28409	LOC119619	0.713037	3.680016	4.018067	0.915867	0.359737	NA	0	0	0	0	4.278221	0
28457	LOC119620	0.713037	3.680016	4.018067	0.915867	0.359737	NA	0	0	0	0	4.278221	0
28493	STOML3	0.713037	3.680016	4.018067	0.915867	0.359737	NA	0	0	0	0	4.278221	0
28513	LOC103243	0.713037	3.680016	4.018067	0.915867	0.359737	NA	0	0	0	0	4.278221	0
28552	LOC103231	0.713037	3.680016	4.018067	0.915867	0.359737	NA	0	0	0	0	4.278221	0
28574	LOC103231	0.713037	3.680016	4.018067	0.915867	0.359737	NA	0	0	0	0	4.278221	0
28587	LOC103231	0.713037	3.680016	4.018067	0.915867	0.359737	NA	0	0	0	0	4.278221	0
28614	SMIM21	0.713037	3.680016	4.018067	0.915867	0.359737	NA	0	0	0	0	4.278221	0
28642	DPPA3	0.713037	3.680016	4.018067	0.915867	0.359737	NA	0	0	0	0	4.278221	0
28727	LOC119620	0.713037	3.680016	4.018067	0.915867	0.359737	NA	0	0	0	0	4.278221	0
28748	LOC103235	0.713037	3.680016	4.018067	0.915867	0.359737	NA	0	0	0	0	4.278221	0
28809	LOC119621	0.713037	3.680016	4.018067	0.915867	0.359737	NA	0	0	0	0	4.278221	0
28817	LOC103225	0.713037	3.680016	4.018067	0.915867	0.359737	NA	0	0	0	0	4.278221	0
28917	LOC103227	0.713037	3.680016	4.018067	0.915867	0.359737	NA	0	0	0	0	4.278221	0
28996	MPC1L	0.713037	3.680016	4.018067	0.915867	0.359737	NA	0	0	0	0	4.278221	0
29009	LOC103231	0.713037	3.680016	4.018067	0.915867	0.359737	NA	0	0	0	0	4.278221	0
29030	LOC119622	0.713037	3.680016	4.018067	0.915867	0.359737	NA	0	0	0	0	4.278221	0
29094	FABP1	0.713037	3.680016	4.018067	0.915867	0.359737	NA	0	0	0	0	4.278221	0
29122	LOC119622	0.713037	3.680016	4.018067	0.915867	0.359737	NA	0	0	0	0	4.278221	0
29195	LOC119622	0.713037	3.680016	4.018067	0.915867	0.359737	NA	0	0	0	0	4.278221	0
29310	LOC119623	0.713037	3.680016	4.018067	0.915867	0.359737	NA	0	0	0	0	4.278221	0
29387	LOC119623	0.713037	3.680016	4.018067	0.915867	0.359737	NA	0	0	0	0	4.278221	0
29454	LOC103234	0.713037	3.680016	4.018067	0.915867	0.359737	NA	0	0	0	0	4.278221	0
6390	LOC119624	24.55698	3.679858	0.923857	3.983147	6.80E-05	0.000283	0	0.547412	10.43597	51.33892	59.8951	25.12449
2877	ITGAD	64.22726	3.679797	0.55932	6.579051	4.73E-11	4.38E-10	3.252518	4.926706	20.17621	127.2312	160.4333	69.34358
15267	DIO3	20.64623	3.679221	2.060968	1.785191	0.07423	0.129259	0	0	9.044508	35.71403	62.03421	17.08465
9050	KRT13	8.384737	3.677375	1.187568	3.09656	0.001958	0.005751	0	2.189647	1.391463	22.32127	21.39111	3.014938
7412	ESM1	13.82165	3.676387	1.022412	3.5958	0.000323	0.00116	0	3.831883	2.087194	33.4819	38.50399	5.024897
9251	GUCA1A	8.319176	3.674427	1.207644	3.04264	0.002345	0.006739	2.168345	0	1.391463	13.39276	29.94755	3.014938
6021	NWD2	20.98678	3.67439	0.884393	4.154705	3.26E-05	0.000144	0	4.926706	4.174388	46.87467	59.8951	10.04979
10166	ACP4	5.846324	3.674344	1.301964	2.822155	0.00477	0.012475	0	0.547412	2.087194	17.85702	8.556443	6.029877
2300	GUCY1A1	47.97851	3.673317	0.495634	7.411357	1.25E-13	1.44E-12	2.168345	9.306001	9.740239	73.66019	100.5382	92.45811
9612	LOC119623	10.71156	3.673139	1.241964	2.957524	0.003101	0.008577	0	0	4.87012	6.696381	40.6431	12.05975
8156	FAM3B	8.148583	3.671601	1.091591	3.363532	0.00077	0.002508	0.542086	2.189647	0.695731	22.32127	17.11289	6.029877
9814	CDHR5	7.559044	3.671486	1.26521	2.901879	0.003709	0.010048	0	0	3.478657	17.85702	14.97377	9.044815
8705	LOC119620	10.63106	3.670706	1.146696	3.201116	0.001369	0.004182	0	0	4.87012	22.32127	23.53022	13.06473
7364	C9	9.643216	3.670032	1.01611	3.611845	0.000304	0.001098	1.084173	1.642235	1.391463	15.62489	32.08666	6.029877
10097	GBX1	5.798928	3.66687	1.291537	2.839153	0.004523	0.01191	0.542086	0	2.087194	11.16063	14.97377	6.029877
4491	ATP2B2	19.13276	3.665182	0.730735	5.015745	5.28E-07	3.13E-06	1.626259	3.831883	2.782925	42.41041	47.06043	17.08465
3478	CACNG6	27.68092	3.665106	0.623337	5.879811	4.11E-09	3.14E-08	4.336691	2.189647	5.565851	66.96381	59.8951	27.13445
8574	CLEC20A	6.949614	3.664602	1.132687	3.235318	0.001215	0.003768	1.084173	0.547412	1.391463	13.39276	19.252	6.029877
841	CYP19A1	99.70743	3.663755	0.31	11.81858	3.13E-32	9.89E-31	13.01007	15.32753	15.30609	216.5163	173.268	164.8166
11800	MLXIPL	29.13851	3.663581	1.49593	2.449032	0.014324	0.032272	1.084173	0	11.82743	58.0353	72.72976	31.15436
9831	LOC119626	9.159626	3.663303	1.263682	2.898913	0.003745	0.010127	0	0	4.174388	20.08914	25.66933	5.024897
8624	RPRML	10.3088	3.662675	1.136631	3.222396	0.001271	0.003919	0	0.547412	4.174388	6.696381	36.36488	14.06971
10343	HOXB1	5.836843	3.662195	1.318023	2.778552	0.00546	0.014035	0	0.547412	2.087194	13.39276	14.97377	4.019918
7554	NT5DC4	9.915253	3.661643	1.034139	3.540764	0.000399	0.001404	1.626259	0	2.782925	26.78552	19.252	9.044815
15494	MUC5B	23.52922	3.658105	2.095859	1.745397	0.080916	0.138837	0	0	10.43597	40.17829	68.45154	22.10955
10312	WNT8A	6.963434	3.657918	1.313235	2.785425	0.005346	0.013779	0	1.642235	1.391463	8.928508	27.80844	2.009959
10898	SCT	5.976529	3.657505	1.379733	2.650878	0.008028	0.019581	0	0	2.782925	8.928508	17.11289	7.034856
10900	CHRNA4	5.976529	3.657505	1.379733	2.650878	0.008028	0.019581	0	0	2.782925	8.928508	17.11289	7.034856
10537	LOC103228	5.80495	3.656897	1.335786	2.737638	0.006188	0.015613	0.542086	0	2.087194	8.928508	19.252	4.019918
9327	CCDC188	10.6121	3.656	1.208891	3.024259	0.002492	0.007105	0	0	4.87012	13.39276	36.36488	9.044815
7993	LOC103225	10.28685	3.655957	1.070613	3.414826	0.000638	0.002123	0	0.547412	4.174388	24.5534	21.39111	11.05477

11996	LOC119624	4.418997	3.654113	1.517962	2.40725	0.016073	0.035621	0	0	2.087194	6.696381	10.69555	7.034856
4512	TTBK1	25.77445	3.654009	0.729878	5.006331	5.55E-07	3.27E-06	0.542086	2.737059	8.348776	60.26743	55.61688	27.13445
11527	LOC103237	4.278424	3.65395	1.456823	2.508163	0.012136	0.02799	0.542086	0	1.391463	11.16063	8.556443	4.019918
10363	SIGLEC5	5.378354	3.650936	1.316256	2.773728	0.005542	0.014217	0	1.642235	0.695731	8.928508	14.97377	6.029877
4318	EXOC3L4	21.03718	3.650208	0.709805	5.142549	2.71E-07	1.67E-06	2.168345	1.094824	6.261582	44.64254	44.92132	27.13445
11051	LOC119625	5.827363	3.649796	1.39555	2.615311	0.008915	0.021446	0	0.547412	2.087194	8.928508	21.39111	2.009959
10633	CCDC175	5.613621	3.649461	1.346434	2.710465	0.006719	0.016799	0	1.094824	1.391463	8.928508	19.252	3.014938
6822	SLFN14	12.85344	3.649185	0.957899	3.809572	0.000139	0.000542	0	1.642235	4.174388	35.71403	23.53022	12.05975
12083	LOC103235	4.118101	3.64882	1.526453	2.390391	0.01683	0.03703	0	1.094824	0.695731	15.62489	4.278221	3.014938
11896	KCNMB2	3.761382	3.648788	1.504018	2.426027	0.015265	0.034114	0.542086	1.094824	0	4.464254	6.417332	10.04979
6602	ERG	23.87857	3.64876	0.935156	3.901765	9.55E-05	0.000385	1.084173	0	9.740239	44.64254	72.72976	15.07469
5559	LOC119622	23.36127	3.648525	0.831213	4.389397	1.14E-05	5.44E-05	1.626259	0	9.044508	46.87467	53.47777	29.1444
11886	BARHL1	4.17425	3.648341	1.502232	2.428612	0.015157	0.033901	0	0.547412	1.391463	2.232127	12.83466	8.039836
11558	TFAP2D	4.263809	3.647332	1.45716	2.503041	0.012313	0.028319	0	0.547412	1.391463	8.928508	10.69555	4.019918
11559	LOC119627	4.263809	3.647332	1.45716	2.503041	0.012313	0.028319	0	0.547412	1.391463	8.928508	10.69555	4.019918
12479	LINC02694	3.81816	3.643451	1.580826	2.304777	0.021179	0.045119	1.626259	0	0	6.696381	8.556443	6.029877
8680	LOC103215	8.354264	3.643206	1.13548	3.208515	0.001334	0.004086	1.626259	0	2.087194	17.85702	23.53022	5.024897
6875	LOC119620	16.22635	3.641971	0.961545	3.787624	0.000152	0.000588	0	0.547412	6.957314	31.24978	38.50399	20.09959
13963	SLC46A2	2.861466	3.641519	1.803584	2.019046	0.043482	0.082789	0	0	1.391463	4.464254	4.278221	7.034856
100	PTGER3	521.9578	3.641159	0.149899	24.29071	2.46E-130	6.47E-128	80.77087	75.54283	75.83472	1015.618	926.2349	957.7454
8459	MPIG6B	8.496612	3.640294	1.114788	3.265458	0.001093	0.003435	0	1.094824	2.782925	13.39276	25.66933	8.039836
5749	OXTR	11.79836	3.640269	0.846506	4.300347	1.71E-05	7.89E-05	1.626259	1.642235	2.087194	22.32127	14.97377	28.13943
11849	CD37	4.231916	3.640183	1.493944	2.436626	0.014825	0.033262	0.542086	0	1.391463	4.464254	14.97377	4.019918
12625	LOC103221	3.873353	3.639429	1.598785	2.276371	0.022824	0.048061	0	1.642235	0	11.16063	6.417332	4.019918
9668	LOC103246	7.075148	3.63868	1.237563	2.940197	0.00328	0.009019	0	1.094824	2.087194	24.5534	10.69555	4.019918
13408	LOC103225	49.02697	3.638515	1.711948	2.125366	0.033556	0.066534	0	1.094824	20.87194	87.05295	136.9031	48.23901
12444	LOC103234	4.462048	3.638421	1.574031	2.311531	0.020804	0.04444	0	0	2.087194	6.696381	14.97377	3.014938
12445	ANGPTL1	4.462048	3.638421	1.574031	2.311531	0.020804	0.04444	0	0	2.087194	6.696381	14.97377	3.014938
8915	ZNF662	12.01161	3.637524	1.158423	3.140065	0.001689	0.005037	0	0	5.565851	20.08914	36.36488	10.04979
11993	ZIC1	36.14111	3.636871	1.510418	2.407858	0.016046	0.03557	0	1.642235	14.61036	71.42806	91.98176	37.18424
13751	LOC103241	2.684752	3.636732	1.767287	2.057805	0.039609	0.076576	0	0.547412	0.695731	6.696381	2.139111	6.029877
9332	LOC119618	8.875023	3.636405	1.202769	3.02336	0.0025	0.007122	0	0	4.174388	15.62489	21.39111	12.05975
7954	TNK1	13.27144	3.634396	1.061489	3.423865	0.000617	0.002063	0	0.547412	5.565851	31.24978	34.22577	8.039836
9417	LOC103219	7.057241	3.63404	1.210003	3.00333	0.00267	0.007539	0	0.547412	2.782925	6.696381	19.252	13.06473
12602	RDH8	3.857851	3.633906	1.592285	2.282195	0.022478	0.047419	0	1.642235	0	8.928508	8.556443	4.019918
11408	LOC119623	4.018174	3.633856	1.433804	2.534416	0.011263	0.02625	0.542086	0.547412	0.695731	4.464254	12.83466	5.024897
5419	PAX5	26.0159	3.633852	0.816365	4.451261	8.54E-06	4.19E-05	0.542086	1.642235	9.740239	33.4819	85.56443	25.12449
7392	LOC119621	11.43213	3.633686	1.008755	3.60215	0.000316	0.001135	0	1.094824	4.174388	22.32127	29.94755	11.05477
12373	CLUL1	5.743827	3.633389	1.561414	2.326986	0.019966	0.0429	0	0.547412	2.087194	0	27.80844	4.019918
11856	ZP2	4.019062	3.633193	1.492396	2.43447	0.014914	0.033441	0	1.094824	0.695731	4.464254	12.83466	5.024897
6579	HEPH	10.77788	3.632439	0.929201	3.909209	9.26E-05	0.000374	2.168345	0.547412	2.087194	17.85702	29.94755	12.05975
5293	FRMPD4	30.13446	3.632184	0.803359	4.521247	6.15E-06	3.09E-05	0	3.284471	10.43597	66.96381	77.00798	23.11453
9864	ZP3	6.420892	3.631749	1.256506	2.890356	0.003848	0.010371	2.168345	0.547412	0	20.08914	10.69555	5.024897
14037	GCG	2.935522	3.6317	1.80976	2.00673	0.044778	0.084789	0	0	1.391463	8.928508	4.278221	3.014938
14039	LOC103231	2.935522	3.6317	1.80976	2.00673	0.044778	0.084789	0	0	1.391463	8.928508	4.278221	3.014938
9585	LOC103238	8.881045	3.630626	1.224798	2.964265	0.003034	0.008414	0	0	4.174388	13.39276	25.66933	10.04979
6759	LOC103237	13.82803	3.630501	0.947471	3.831781	0.000127	0.0005	0	2.737059	3.478657	33.4819	34.22577	9.044815
12473	CIBAR2	4.093118	3.63028	1.57423	2.306067	0.021107	0.044987	0	1.094824	0.695731	8.928508	12.83466	1.004979
3427	SYT16	34.10635	3.629717	0.613317	5.918173	3.26E-09	2.53E-08	3.252518	4.379295	7.653045	66.96381	96.25998	26.12947
9577	LOC103237	10.4446	3.628257	1.223012	2.966657	0.003011	0.008357	0	0	4.87012	13.39276	36.36488	8.039836
13069	LOC103214	3.863873	3.627944	1.654005	2.19343	0.028276	0.05752	0	1.642235	0	6.696381	12.83466	2.009959
13815	LOC103221	2.72178	3.627637	1.773737	2.045195	0.040836	0.078582	0	0.547412	0.695731	8.928508	2.139111	4.019918
9228	POTEA	6.923578	3.627167	1.190069	3.047864	0.002305	0.00664	0	1.094824	2.087194	8.928508	21.39111	8.039836
4735	LOC103224	20.74566	3.626863	0.746503	4.858473	1.18E-06	6.64E-06	2.168345	1.094824	6.261582	26.78552	62.03421	26.12947
13982	LOC103245	2.920019	3.626	1.799332	2.015192	0.043885	0.083441	0	0	1.391463	6.696381	6.417332	3.014938
657	SEMA5B	129.8758	3.625878	0.27714	13.08319	4.11E-39	1.66E-37	21.68345	14.78012	22.2634	218.7484	254.5542	247.2249
867	MYL3	126.6695	3.625348	0.309956	11.69632	1.33E-31	4.08E-30	15.7205	24.63353	16.69755	254.4625	171.1289	277.3743
10335	FBP2	7.39757	3.625307	1.303676	2.780833	0.005422	0.013947	0	0	3.478657	15.62489	19.252	6.029877
7531	MYO3B	9.592827	3.625253	1.021614	3.548554	0.000387	0.001367	0	1.642235	2.782925	13.39276	25.66933	14.06971
489	NID2	156.1515	3.624113	0.245712	14.74943	3.10E-49	1.69E-47	23.30971	20.80165	26.43779	283.4801	299.4755	283.4042
15175	REC114	2.795836	3.624094	2.012031	1.801212	0.071669	0.125557	0	0.547412	0.695731	13.39276	2.139111	0
8844	LOC119625	6.693446	3.624056	1.147538	3.158115	0.001588	0.004773	0.542086	1.094824	1.391463	6.696381	21.39111	9.044815
7716	MYH4	15.99579	3.622601	1.038421	3.488569	0.000486	0.001673	0	1.094824	6.261582	31.24978	51.33866	6.029877
13646	LOC119627	2.706277	3.62161	1.740966	2.08023	0.037504	0.073066	0	0.547412	0.695731	6.696381	4.278221	4.019918
14202	LOC103235	2.867488	3.621102	1.832362	1.976194	0.048133	0.090101	0	0	1.391463	2.232127	8.556443	5.024897
3748	CUNH1orf1	31.58087	3.620531	0.646968	5.596156	2.19E-08	1.55E-07	2.710432	3.284471	8.348776	58.0353	91.98176	25.12449
14089	LOC103220	2.758808	3.61988	1.812837	1.996804	0.045846	0.086509	0	0.547412	0.695731	11.16063	2.139111	2.009959
8470	BARX2	8.472259	3.619702	1.109496	3.262474	0.001104	0.003467	0	0.547412	3.478657	15.62489	17.11289	14.06971
14987	LOC119623	2.779446	3.618722	1.970486	1.836462	0.066289	0.117589	0.542086	0	0.695731	11.16063	4.278221	0
5831	SGIP1	27.79686	3.616678	0.849414	4.257853	2.06E-05	9.41E-05	0.542086	0.547412	11.82743	66.96381	57.75599	29.1444
10970	SIGLEC6	5.80301	3.616589	1.372364	2.635299	0.008406	0.020372	0	0	2.782925	11.16063	12.83466	8.039836
10627	TRIM17	7.21243	3.615701	1.333364	2.711713	0.006694	0.016745	0	0	3.478657	4.464254	19.252	16.07967
13868	ANKRD33	2.742418	3.615226	1.775384	2.036307	0.04172	0.079971	0.542086	0	0.695731	8.928508	4.278221	2.009959
13869	LOC119620	2.742418	3.615226	1.775384	2.036307	0.04172	0.079971	0.542086	0	0.695731	8.928508	4.278221	2.009959

13668	LOC10323	2.690775	3.615157	1.742301	2.074932	0.037993	0.073898	0	0.547412	0.695731	4.464254	6.417332	4.019918
7194	LOC10322(	8.976487	3.613409	0.984965	3.668567	0.000244	0.000901	1.626259	1.642235	0.695731	11.16063	25.66933	13.06473
11322	SFRP5	5.877066	3.61313	1.415617	2.552335	0.0107	0.025125	0	0	2.782925	15.62489	12.83466	4.019918
5880	PRKCB	29.26137	3.612773	0.855093	4.225007	2.39E-05	0.000108	0.542086	0.547412	12.52316	55.80317	77.00798	29.1444
7693	CYP2W1	8.108464	3.612558	1.033386	3.495847	0.000473	0.001633	0.542086	1.094824	2.087194	15.62489	19.252	10.04979
11052	LOC11962(	5.787507	3.612246	1.381227	2.615245	0.008916	0.021446	0	0	2.782925	8.928508	14.97377	8.039836
14947	LOC10324!	2.763943	3.610673	1.955799	1.846137	0.064872	0.115383	0.542086	0	0.695731	8.928508	6.417332	0
15158	LOC10322	2.566591	3.610174	2.000566	1.804576	0.071141	0.124771	0	1.094824	0	11.16063	2.139111	1.004979
13279	LOXHD1	49.35588	3.609859	1.677706	2.151663	0.031424	0.062912	0	1.642235	20.87194	102.6778	134.764	36.17926
9055	CCDC177	8.573863	3.607641	1.165896	3.094308	0.001973	0.005792	0	0.547412	3.478657	17.85702	23.53022	6.029877
6251	LOC10322	16.72024	3.60647	0.890433	4.050242	5.12E-05	0.000218	0	2.189647	5.565851	35.71403	42.78221	14.06971
5240	DNAH8	17.76367	3.6056	0.790921	4.558733	5.15E-06	2.61E-05	1.626259	1.642235	4.87012	46.87467	38.50399	13.06473
5904	LHFPL1	24.2674	3.604238	0.856196	4.209594	2.56E-05	0.000115	0	1.642235	9.740239	33.4819	70.59065	30.14938
14602	THEMIS2	2.926042	3.604142	1.891602	1.905338	0.056736	0.103289	0	0	1.391463	4.464254	10.69555	1.004979
418	MYOM2	198.6756	3.604052	0.23299	15.46868	5.65E-54	3.59E-52	34.15144	31.20247	25.04633	372.7652	320.8666	408.0217
6447	PLEKHS1	15.59276	3.602339	0.909424	3.96112	7.46E-05	0.000308	0.542086	0.547412	6.261582	40.17829	29.94755	16.07967
60	TMEM86A	846.4103	3.601035	0.127683	28.2029	5.39E-175	2.39E-172	124.1378	129.7366	133.5804	1497.757	1555.133	1638.117
10683	LOC11962!	5.027234	3.599423	1.335268	2.695656	0.007025	0.017482	0.542086	1.642235	0	13.39276	8.556443	6.029877
14894	SLC34A2	2.910539	3.595442	1.937843	1.855383	0.063541	0.113418	0	0	1.391463	2.232127	12.83466	1.004979
13079	LOC10323	4.35054	3.595284	1.640866	2.191089	0.028445	0.05782	0	0	2.087194	17.85702	2.139111	4.019918
11859	RTP5	5.719474	3.59397	1.476464	2.434174	0.014926	0.03346	0	0	2.782925	2.232127	19.252	10.04979
13172	KCNC2	23.71604	3.593765	1.651497	2.176065	0.02955	0.059642	0	0.547412	10.43597	44.64254	70.59065	16.07967
14867	LOC10323(	2.48128	3.593393	1.932206	1.859736	0.062923	0.112518	1.084173	0	0	2.232127	8.556443	3.014938
8942	GLDN	9.594968	3.591721	1.146055	3.133987	0.001724	0.005127	0	1.642235	2.782925	22.32127	27.80844	3.014938
3731	LOC11961!	39.85164	3.589968	0.639854	5.610602	2.02E-08	1.44E-07	1.084173	4.379295	13.2189	73.66019	100.5382	46.22906
22	ABCC2	8111.442	3.589561	0.099261	36.16302	2.32E-286	2.81E-283	1206.142	1323.642	1203.615	14258.83	14272.15	16404.28
10520	KRT27	5.231496	3.589198	1.310111	2.739614	0.006151	0.015544	0.542086	1.094824	0.695731	8.928508	17.11289	3.014938
497	DGAT2	155.116	3.588657	0.244705	14.66525	1.08E-48	5.76E-47	21.14137	24.08612	26.43779	285.7123	290.9191	282.3992
4858	PRSS42P	15.4796	3.58797	0.751548	4.774107	1.81E-06	9.88E-06	2.710432	2.189647	2.087194	35.71403	32.08666	18.08963
15265	CARD17	2.502805	3.587662	2.00954	1.785315	0.07421	0.129242	1.084173	0	0	2.232127	10.69555	1.004979
15049	LOC11962	2.681294	3.587409	1.966667	1.824106	0.068136	0.120351	0	0.547412	0.695731	0	12.83466	2.009959
16166	LOC11962	2.489078	3.586494	2.195929	1.633247	0.102417	0.168425	0	1.094824	0	0	12.83466	1.004979
6209	OPN5	16.59685	3.586356	0.881333	4.069243	4.72E-05	0.000202	0	1.642235	6.261582	26.78552	42.78221	22.10955
14890	BPIFB6	2.483055	3.58593	1.931442	1.856608	0.063367	0.11314	0	1.094824	0	2.232127	8.556443	3.014938
10376	LOC10321	7.156017	3.585197	1.294219	2.770164	0.005603	0.014356	0	0	3.478657	11.16063	19.252	9.044815
8450	LOC10324	7.84088	3.584547	1.096585	3.268826	0.00108	0.003398	1.084173	1.094824	1.391463	29.01765	6.417332	8.039836
15051	LOC11962	2.520083	3.583285	1.964373	1.824137	0.068131	0.120351	0	1.094824	0	4.464254	8.556443	1.004979
12056	ESX1	3.876548	3.582832	1.495758	2.395328	0.016606	0.036617	0	1.094824	0.695731	11.16063	4.278221	6.029877
9492	LOC11962(	6.59487	3.582639	1.199732	2.986199	0.002825	0.007911	0	1.642235	1.391463	13.39276	17.11289	6.029877
7261	SDK1	12.15271	3.582293	0.981627	3.649342	0.000263	0.000963	0	2.189647	3.478657	37.94616	19.252	10.04979
4251	BTNL9	47.72277	3.581865	0.690499	5.187355	2.13E-07	1.33E-06	1.084173	3.284471	18.08902	113.8385	104.8164	45.22408
11837	LOC11962(	4.059284	3.580923	1.468041	2.439253	0.014718	0.033052	0	0.547412	1.391463	6.696381	10.69555	5.024897
11838	PRTN3	4.059284	3.580923	1.468041	2.439253	0.014718	0.033052	0	0.547412	1.391463	6.696381	10.69555	5.024897
7472	PATL2	20.15144	3.578173	1.001848	3.571574	0.000355	0.001263	0	0.547412	9.044508	26.78552	68.45154	16.07967
12591	LOC10323	4.273026	3.577599	1.565282	2.285594	0.022278	0.047031	0	0	2.087194	6.696381	12.83466	4.019918
12592	CUNH2orf	4.273026	3.577599	1.565282	2.285594	0.022278	0.047031	0	0	2.087194	6.696381	12.83466	4.019918
12593	GSX1	4.273026	3.577599	1.565282	2.285594	0.022278	0.047031	0	0	2.087194	6.696381	12.83466	4.019918
10487	TNFSF8	5.436387	3.577562	1.30205	2.747638	0.006003	0.015217	0.542086	0	2.087194	13.39276	8.556443	8.039836
8920	LOC11962(	8.347813	3.577416	1.139593	3.139206	0.001694	0.005049	0	0.547412	3.478657	15.62489	21.39111	9.044815
8651	LOC11962	7.97622	3.577023	1.112537	3.215195	0.001304	0.004006	1.626259	0	2.087194	17.85702	19.252	7.034856
10542	LOC10324	6.975846	3.574834	1.306736	2.735696	0.006225	0.015698	0	0.547412	2.782925	6.696381	27.80844	4.019918
12141	PSMB11	4.079922	3.573197	1.502291	2.378499	0.017383	0.038064	0.542086	0	1.391463	6.696381	12.83466	3.014938
10461	OTX2	5.421772	3.572744	1.2973	2.753985	0.005887	0.014962	0	0.547412	2.087194	11.16063	10.69555	8.039836
14238	SUN3	4.362585	3.572651	1.814248	1.969219	0.048928	0.091358	0	0	2.087194	13.39276	10.69555	0
17716	PRR32	1.325459	3.571452	2.584426	1.381913	0.166998	0.250601	0	0	0.695731	2.232127	0	5.024897
6855	LOC11962!	21.43688	3.570894	0.941262	3.79373	0.000148	0.000576	0	0.547412	9.740239	53.57105	40.6431	24.11951
12785	NODAL	4.257523	3.570882	1.592624	2.242137	0.024953	0.051882	0	0	2.087194	4.464254	14.97377	4.019918
12786	LOC10322	4.257523	3.570882	1.592624	2.242137	0.024953	0.051882	0	0	2.087194	4.464254	14.97377	4.019918
8816	LOC11962	14.49115	3.570779	1.126352	3.170215	0.001523	0.004593	0	0	6.957314	35.71403	34.22577	10.04979
5885	LOC11961	28.16238	3.570777	0.845799	4.221778	2.42E-05	0.000109	0	1.642235	11.82743	40.17829	79.14709	36.17926
8308	SLC24A4	7.894459	3.568773	1.077782	3.31122	0.000929	0.002972	0.542086	1.094824	2.087194	8.928508	25.66933	9.044815
12070	LOC10324	3.882571	3.567578	1.490409	2.393691	0.01668	0.036735	0	1.094824	0.695731	8.928508	8.556443	4.019918
11462	FAM181A	5.688044	3.566265	1.412791	2.524268	0.011594	0.026892	0	0	2.782925	15.62489	10.69555	5.024897
17584	LOC10323	1.362487	3.566058	2.542093	1.402804	0.160675	0.242916	0	0	0.695731	4.464254	0	3.014938
17658	CHRNA6	1.337767	3.565938	2.556482	1.394862	0.163058	0.245491	0	0.547412	0	4.464254	0	3.014938
10151	LOC11962(	6.88932	3.563607	1.261288	2.825372	0.004723	0.012368	0	0.547412	2.782925	24.5534	6.417332	7.034856
10247	LOC10322	8.58308	3.563223	1.271074	2.803317	0.005058	0.013123	0	0	4.174388	15.62489	25.66933	6.029877
9982	ACKR2	8.450471	3.562415	1.243018	2.865941	0.004158	0.011073	0	0	4.174388	8.928508	23.53022	14.06971
5641	PEX5L	23.43914	3.561201	0.818029	4.353391	1.34E-05	6.32E-05	1.626259	0.547412	9.044508	53.57105	57.75599	18.08963
4516	ISLR	21.32754	3.560751	0.711772	5.002655	5.65E-07	3.33E-06	1.626259	1.642235	6.957314	42.41041	49.19955	26.12947
8754	IL17REL	9.737848	3.560496	1.117224	3.186912	0.001438	0.004367	0	0.547412	4.174388	17.85702	27.80844	8.039836
17844	LOC11962	1.374795	3.560158	2.59969	1.369455	0.170857	0.254538	0	0.547412	0	6.696381	0	1.004979
17845	LOC11961!	1.374795	3.560158	2.59969	1.369455	0.170857	0.254538	0	0.547412	0	6.696381	0	1.004979
8034	GLP1R	18.5375	3.560131	1.046048	3.403411	0.000666	0.002202	0	0.547412	8.348776	31.24978	62.03421	9.044815

593	HLF	154.3509	3.55987	0.260275	13.67735	1.39E-42	6.22E-41	24.93597	24.63353	22.95914	225.4448	316.5884	311.5436
17756	LOC11962	1.399515	3.559643	2.586001	1.376505	0.168665	0.252436	0	0	0.695731	6.696381	0	1.004979
17766	GNAT3	1.399515	3.559643	2.586001	1.376505	0.168665	0.252436	0	0	0.695731	6.696381	0	1.004979
17801	PRR19	1.399515	3.559643	2.586001	1.376505	0.168665	0.252436	0	0	0.695731	6.696381	0	1.004979
17809	KLRF1	1.399515	3.559643	2.586001	1.376505	0.168665	0.252436	0	0	0.695731	6.696381	0	1.004979
11836	LOC10322	3.850678	3.559104	1.459053	2.439325	0.014715	0.033051	0.542086	0.547412	0.695731	4.464254	12.83466	4.019918
10058	GAPDHS	10.05063	3.558559	1.248865	2.849436	0.00438	0.011575	0	0	4.87012	29.01765	21.39111	5.024897
10059	LOC11962	10.05063	3.558559	1.248865	2.849436	0.00438	0.011575	0	0	4.87012	29.01765	21.39111	5.024897
6778	ANKFN1	18.08566	3.556265	0.92972	3.825094	0.000131	0.000513	0	1.094824	7.653045	33.4819	49.19955	17.08465
12525	TMIGD1	3.888593	3.556111	1.548372	2.296677	0.021637	0.045926	0	1.094824	0.695731	6.696381	12.83466	2.009959
6345	ATP1A4	22.39315	3.556061	0.887263	4.007899	6.13E-05	0.000257	0	1.094824	9.740239	51.33892	47.06043	25.12449
5875	KCNK3	11.36045	3.555612	0.840866	4.22851	2.35E-05	0.000106	1.626259	1.642235	2.087194	22.32127	21.39111	19.09461
16880	LOC11962	1.346985	3.555223	2.355438	1.509368	0.131205	0.206612	0	0	0.695731	2.232127	2.139111	3.014938
16883	LOC11962	1.346985	3.555223	2.355438	1.509368	0.131205	0.206612	0	0	0.695731	2.232127	2.139111	3.014938
16934	LOC11961	1.322265	3.555028	2.367436	1.501636	0.133191	0.209087	0	0.547412	0	2.232127	2.139111	3.014938
16935	LOC10323	1.322265	3.555028	2.367436	1.501636	0.133191	0.209087	0	0.547412	0	2.232127	2.139111	3.014938
8516	RNF17	8.7353	3.554192	1.092685	3.252715	0.001143	0.003568	2.710432	0.547412	0.695731	15.62489	27.80844	5.024897
33	VAV3	1832.028	3.55405	0.10802	32.90192	2.06E-237	1.66E-234	274.8378	309.8351	277.5968	3430.779	3227.918	3471.199
42	CSF1	10326.51	3.553675	0.113002	31.44777	4.50E-217	2.85E-214	1470.68	1828.903	1563.308	18542.28	17641.25	20912.62
8122	MFAP4	9.761638	3.55244	1.053396	3.372369	0.000745	0.00244	1.626259	2.737059	0	26.78552	21.39111	6.029877
11114	FCRL5	5.396789	3.552111	1.366073	2.600236	0.009316	0.022286	0	0.547412	2.087194	4.464254	19.252	6.029877
15394	LOC10323	19.00961	3.551231	2.016911	1.760728	0.078284	0.135195	0	0	9.044508	35.71403	49.19955	20.09959
11363	PABPN1L	8.595125	3.549985	1.394712	2.545318	0.010918	0.025542	0	0	4.174388	11.16063	34.22577	2.009959
4373	JPH2	27.59015	3.549693	0.696222	5.098509	3.42E-07	2.08E-06	3.252518	2.189647	7.653045	44.64254	87.70354	20.09959
8792	SIX6	7.888012	3.54952	1.117641	3.175905	0.001494	0.004517	0.542086	1.094824	2.087194	29.01765	8.556443	6.029877
16113	GPR174	13.14071	3.549145	2.159927	1.643178	0.100346	0.165568	0	0	6.261582	26.78552	42.78221	3.014938
11753	LOC10324	4.808094	3.54691	1.442981	2.458043	0.01397	0.031599	0	2.189647	0	8.928508	10.69555	7.034856
9103	SLC1A6	8.211322	3.546449	1.150454	3.082651	0.002052	0.005992	0	0.547412	3.478657	20.08914	17.11289	8.039836
9771	DNAI2	8.300881	3.546164	1.218347	2.910636	0.003607	0.009814	0	0.547412	3.478657	26.78552	14.97377	4.019918
5804	SCN11A	18.3092	3.544199	0.829631	4.27202	1.94E-05	8.87E-05	0.542086	3.284471	4.87012	33.4819	55.61688	12.05975
9707	TCIM	8.269875	3.541476	1.209864	2.92717	0.003421	0.009368	0	0.547412	3.478657	22.32127	19.252	4.019918
11142	LOC10321	5.043361	3.540783	1.365361	2.593295	0.009506	0.022682	0	1.642235	0.695731	8.928508	14.97377	4.019918
14607	FCN3	2.709472	3.540693	1.858835	1.904792	0.056807	0.103356	0	0	1.391463	6.696381	2.139111	6.029877
13091	NTRK3	49.8184	3.540648	1.617507	2.188954	0.0286	0.058081	0	2.189647	21.56767	82.5887	143.3204	49.24399
3480	LOC10322	29.30027	3.539984	0.602249	5.877943	4.15E-09	3.17E-08	5.420864	1.094824	7.653045	58.0353	51.33866	52.25893
6958	CNTN4	20.79592	3.539225	0.942997	3.753166	0.000175	0.000667	1.084173	0	9.044508	31.24978	66.31243	17.08465
8628	LOC11962	7.73733	3.539	1.098901	3.22049	0.00128	0.003943	0	1.642235	2.087194	13.39276	19.252	10.04979
17702	LOC10323	1.331482	3.538773	2.557529	1.383669	0.16646	0.249977	0	0	0.695731	0	4.278221	3.014938
17703	LOC10324	1.331482	3.538773	2.557529	1.383669	0.16646	0.249977	0	0	0.695731	0	4.278221	3.014938
17764	GHRL	1.306762	3.538539	2.57243	1.375563	0.168957	0.252436	0	0.547412	0	0	4.278221	3.014938
17776	LOC11962	1.305874	3.538531	2.572973	1.375269	0.169048	0.252436	0.542086	0	0	0	4.278221	3.014938
14344	LOC10323	2.47934	3.53811	1.810991	1.953687	0.050738	0.094041	0.542086	0	0.695731	4.464254	2.139111	7.034856
9338	GALNT8	7.884555	3.537646	1.170632	3.021996	0.002511	0.007149	0.542086	1.094824	2.087194	22.32127	19.252	2.009959
8572	LOC11961	7.427748	3.537497	1.093312	3.23558	0.001214	0.003765	1.626259	1.094824	0.695731	24.5534	8.556443	8.039836
9655	DRD4	6.462627	3.537481	1.200944	2.945584	0.003223	0.008876	1.084173	0.547412	1.391463	15.62489	17.11289	3.014938
17093	LOC11962	1.359293	3.537243	2.385559	1.482773	0.138135	0.21483	0	0.547412	0	4.464254	2.139111	1.004979
17094	CUNHXorf	1.359293	3.537243	2.385559	1.482773	0.138135	0.21483	0	0.547412	0	4.464254	2.139111	1.004979
8869	POM121L1	8.008738	3.537114	1.122524	3.151036	0.001627	0.004877	0.542086	0.547412	2.782925	15.62489	23.53022	5.024897
17020	CUNH11or	1.384013	3.536684	2.374109	1.489689	0.136306	0.212795	0	0	0.695731	4.464254	2.139111	1.004979
17022	LOC11962	1.384013	3.536684	2.374109	1.489689	0.136306	0.212795	0	0	0.695731	4.464254	2.139111	1.004979
17023	WFDC6	1.384013	3.536684	2.374109	1.489689	0.136306	0.212795	0	0	0.695731	4.464254	2.139111	1.004979
17024	LOC10322	1.384013	3.536684	2.374109	1.489689	0.136306	0.212795	0	0	0.695731	4.464254	2.139111	1.004979
17025	LOC10324	1.384013	3.536684	2.374109	1.489689	0.136306	0.212795	0	0	0.695731	4.464254	2.139111	1.004979
17026	LOC10323	1.384013	3.536684	2.374109	1.489689	0.136306	0.212795	0	0	0.695731	4.464254	2.139111	1.004979
17027	ASGR2	1.384013	3.536684	2.374109	1.489689	0.136306	0.212795	0	0	0.695731	4.464254	2.139111	1.004979
17028	LOC10323	1.384013	3.536684	2.374109	1.489689	0.136306	0.212795	0	0	0.695731	4.464254	2.139111	1.004979
29	LOC11962	6243.791	3.536013	0.103009	34.32732	3.07E-258	2.81E-255	913.4155	1098.656	961.5008	11008.85	11335.15	12145.18
149	LOC10324	457.5171	3.535833	0.159486	22.17022	6.66E-109	1.19E-106	80.22878	66.78424	70.9646	803.5657	849.2269	874.3321
14655	TMEM247	2.7465	3.535723	1.864695	1.89614	0.057942	0.105109	0	0	1.391463	8.928508	2.139111	4.019918
15437	DKK2	15.90617	3.534576	2.014688	1.754404	0.079361	0.136673	0	0	7.653045	44.64254	32.08666	11.05477
14463	LOC11962	2.730997	3.53213	1.828315	1.931905	0.053371	0.098097	0	0	1.391463	6.696381	4.278221	4.019918
14464	LOC11962	2.730997	3.53213	1.828315	1.931905	0.053371	0.098097	0	0	1.391463	6.696381	4.278221	4.019918
11490	VPREB3	4.797726	3.531552	1.402176	2.518622	0.011782	0.027259	0.542086	1.642235	0	4.464254	17.11289	5.024897
64	PDGFB	835.5829	3.530363	0.129412	27.27995	7.34E-164	3.05E-161	142.0266	126.4521	130.7975	1593.739	1456.734	1563.748
956	ABCC6	88.25031	3.530001	0.315704	11.18137	5.03E-29	1.40E-27	14.09425	15.32753	12.52316	156.2489	177.5462	153.7619
8288	LOC10321	9.074835	3.529086	1.064061	3.316621	0.000911	0.002922	0	1.642235	2.782925	11.16063	27.80844	11.05477
6897	TRPM2	12.91995	3.528663	0.934285	3.776858	0.000159	0.000612	0	2.737059	3.478657	35.71403	23.53022	12.05975
14669	LOC11962	2.678466	3.526853	1.862314	1.893801	0.058251	0.10557	0	0	1.391463	2.232127	6.417332	6.029877
8431	ADGRD2	10.91734	3.525488	1.077104	3.273117	0.001064	0.003354	0	0.547412	4.87012	20.08914	29.94755	10.04979
5984	LINGO1	25.80078	3.525417	0.845218	4.171014	3.03E-05	0.000135	0	2.189647	10.43597	58.0353	62.03421	22.10955
14492	LOC10324	2.715494	3.525225	1.829995	1.926358	0.05406	0.09917	0	0	1.391463	4.464254	6.417332	4.019918
14695	LOC11962	2.768025	3.524377	1.863789	1.890974	0.058628	0.10605	0	0	1.391463	8.928508	4.278221	2.009959
14696	LCN8	2.768025	3.524377	1.863789	1.890974	0.058628	0.10605	0	0	1.391463	8.928508	4.278221	2.009959
14697	BCL2L14	2.768025	3.524377	1.863789	1.890974	0.058628	0.10605	0	0	1.391463	8.928508	4.278221	2.009959

17158	LOC10323(	1.342902	3.524031	2.392143	1.473169	0.140705	0.218012	0.542086	0	0	2.232127	4.278221	1.004979
17153	LECT2	1.34379	3.524012	2.391718	1.473423	0.140637	0.217932	0	0.547412	0	2.232127	4.278221	1.004979
17154	LOC11962:	1.34379	3.524012	2.391718	1.473423	0.140637	0.217932	0	0.547412	0	2.232127	4.278221	1.004979
17155	LOC10323!	1.34379	3.524012	2.391718	1.473423	0.140637	0.217932	0	0.547412	0	2.232127	4.278221	1.004979
17156	LOC10324:	1.34379	3.524012	2.391718	1.473423	0.140637	0.217932	0	0.547412	0	2.232127	4.278221	1.004979
17103	LOC10323(	1.36851	3.523498	2.380071	1.480417	0.138762	0.215617	0	0	0.695731	2.232127	4.278221	1.004979
17104	LOC10323{	1.36851	3.523498	2.380071	1.480417	0.138762	0.215617	0	0	0.695731	2.232127	4.278221	1.004979
17105	LOC10322(	1.36851	3.523498	2.380071	1.480417	0.138762	0.215617	0	0	0.695731	2.232127	4.278221	1.004979
17107	APOBEC4	1.36851	3.523498	2.380071	1.480417	0.138762	0.215617	0	0	0.695731	2.232127	4.278221	1.004979
17108	LOC10322:	1.36851	3.523498	2.380071	1.480417	0.138762	0.215617	0	0	0.695731	2.232127	4.278221	1.004979
17109	LOC10322:	1.36851	3.523498	2.380071	1.480417	0.138762	0.215617	0	0	0.695731	2.232127	4.278221	1.004979
10239	PPP1R14D	6.683908	3.523123	1.255779	2.805527	0.005023	0.013043	0.542086	0	2.782925	22.32127	6.417332	8.039836
14171	IL1F10	2.501753	3.519938	1.775849	1.982116	0.047466	0.089068	0	0.547412	0.695731	4.464254	4.278221	5.024897
7036	ADIPOQ	23.65591	3.519596	0.946336	3.719184	0.0002	0.000755	0	0.547412	11.1317	55.80317	51.33866	23.11453
12705	LOC10322:	4.099507	3.519426	1.559097	2.257349	0.023986	0.050191	0	0	2.087194	8.928508	8.556443	5.024897
12669	TBX5	37.58246	3.518584	1.552255	2.266757	0.023405	0.049114	0	2.189647	16.00182	87.05295	94.12087	26.12947
18010	CUNH1orf:	1.3274	3.518005	2.623204	1.34111	0.179885	0.265488	0.542086	0	0	0	6.417332	1.004979
18008	LOC11962:	1.328287	3.517991	2.622678	1.341373	0.179799	0.265421	0	0.547412	0	0	6.417332	1.004979
18009	TARM1	1.328287	3.517991	2.622678	1.341373	0.179799	0.265421	0	0.547412	0	0	6.417332	1.004979
14647	LOC11962:	2.752522	3.517897	1.853998	1.897465	0.057767	0.104849	0	0	1.391463	6.696381	6.417332	2.009959
17964	FAM240A	1.353007	3.517596	2.608251	1.348642	0.177452	0.262554	0	0	0.695731	0	6.417332	1.004979
17965	LOC11962(	1.353007	3.517596	2.608251	1.348642	0.177452	0.262554	0	0	0.695731	0	6.417332	1.004979
17967	RNASE7	1.353007	3.517596	2.608251	1.348642	0.177452	0.262554	0	0	0.695731	0	6.417332	1.004979
17968	CCL13	1.353007	3.517596	2.608251	1.348642	0.177452	0.262554	0	0	0.695731	0	6.417332	1.004979
6619	BNIP5	11.13334	3.516743	0.903236	3.893492	9.88E-05	0.000397	1.626259	2.189647	1.391463	29.01765	23.53022	9.044815
15347	LOC10322:	18.57302	3.516293	1.985539	1.770951	0.076569	0.132637	0	0	9.044508	33.4819	42.78221	26.12947
12960	LOC10323(	4.136535	3.515526	1.589714	2.21142	0.027007	0.055399	0	0	2.087194	11.16063	8.556443	3.014938
14354	LOC10323:	2.485362	3.515263	1.801736	1.951043	0.051052	0.094553	0.542086	0	0.695731	2.232127	6.417332	5.024897
14762	LOC10324:	2.699992	3.51468	1.869941	1.879568	0.060167	0.108355	0	0	1.391463	2.232127	8.556443	4.019918
5455	KASH5	16.54448	3.513904	0.7919	4.43731	9.11E-06	4.44E-05	2.168345	1.642235	4.174388	29.01765	49.19955	13.06473
8393	GHRHR	7.630957	3.511778	1.06936	3.284001	0.001023	0.003242	0.542086	1.094824	2.087194	22.32127	10.69555	9.044815
12287	LOC11962!	3.85476	3.510942	1.495965	2.346941	0.018928	0.040954	0	0.547412	1.391463	4.464254	10.69555	6.029877
11635	EFS	5.468017	3.510812	1.41203	2.486359	0.012906	0.029489	0	0	2.782925	11.16063	12.83466	6.029877
12979	ZNF750	4.121032	3.510691	1.590386	2.207447	0.027283	0.055884	0	0	2.087194	8.928508	10.69555	3.014938
12243	TRNAK-UU	3.907291	3.510343	1.48884	2.357771	0.018385	0.039922	0	0.547412	1.391463	8.928508	8.556443	4.019918
8682	LOC10324:	13.85003	3.510304	1.09412	3.208336	0.001335	0.004088	0	0	6.957314	31.24978	27.80844	17.08465
14755	LOC11962(	2.73702	3.510206	1.867109	1.880022	0.060105	0.108273	0	0	1.391463	4.464254	8.556443	2.009959
14756	ABRA	2.73702	3.510206	1.867109	1.880022	0.060105	0.108273	0	0	1.391463	4.464254	8.556443	2.009959
14757	RTP4	2.73702	3.510206	1.867109	1.880022	0.060105	0.108273	0	0	1.391463	4.464254	8.556443	2.009959
1271	NAP1L2	93.06323	3.510044	0.353505	9.929257	3.11E-23	6.50E-22	8.131296	15.87494	21.56767	191.9629	154.016	166.8266
2295	NAT8	43.20805	3.509867	0.472774	7.423981	1.14E-13	1.32E-12	8.131296	7.116354	5.565851	102.6778	51.33866	84.41828
11882	F5	5.335408	3.509663	1.444918	2.42897	0.015142	0.033878	0	0	2.782925	4.464254	10.69555	14.06971
8066	LOC11962:	8.947824	3.509532	1.03421	3.393441	0.00069	0.002275	0	1.642235	2.782925	20.08914	17.11289	12.05975
15164	LOC11962:	2.449222	3.509266	1.946348	1.803	0.071388	0.125155	0	0.547412	0.695731	0	6.417332	7.034856
15882	CUNH10or	2.78955	3.508587	2.07883	1.68777	0.091455	0.153088	0	0	1.391463	8.928508	6.417332	0
6466	HEATR4	12.911	3.507988	0.887478	3.952763	7.73E-05	0.000318	1.626259	0.547412	4.174388	31.24978	27.80844	12.05975
14235	CCDC179	2.538781	3.50798	1.781264	1.969377	0.04891	0.091337	0	0.547412	0.695731	6.696381	4.278221	3.014938
14236	LOC11962(	2.538781	3.50798	1.781264	1.969377	0.04891	0.091337	0	0.547412	0.695731	6.696381	4.278221	3.014938
12552	LOC10323:	3.760695	3.507848	1.530045	2.292643	0.021869	0.046317	0.542086	0.547412	0.695731	15.62489	2.139111	3.014938
10054	VRTN	8.155963	3.507717	1.230597	2.850418	0.004366	0.011545	0	0	4.174388	17.85702	12.83466	14.06971
13872	LOC11962(	3.981347	3.506733	1.72265	2.035662	0.041784	0.080078	0	0.547412	1.391463	13.39276	8.556443	0
8045	LOC10324:	10.5671	3.506446	1.030999	3.401018	0.000671	0.002219	0	1.094824	4.174388	24.5534	23.53022	10.04979
12306	LOC10323!	3.891788	3.505468	1.49607	2.343117	0.019123	0.041313	0	0.547412	1.391463	6.696381	10.69555	4.019918
8275	GZMM	10.72229	3.505226	1.05545	3.321072	0.000897	0.002881	0	0.547412	4.87012	22.32127	23.53022	13.06473
13104	LOC11962!	4.10553	3.505106	1.602581	2.187163	0.028731	0.058288	0	0	2.087194	6.696381	12.83466	3.014938
8802	ZNF648	9.359805	3.504503	1.104426	3.173143	0.001508	0.004555	0	0.547412	4.174388	17.85702	23.53022	10.04979
10194	BPIFB3	6.628808	3.503915	1.243849	2.816994	0.004848	0.012642	0	0.547412	2.782925	11.16063	19.252	6.029877
74	ZNF292	906.4474	3.503785	0.132439	26.45589	3.12E-154	1.12E-151	133.3532	166.9606	139.842	1667.399	1674.924	1656.206
3963	TMEM179	28.25839	3.503784	0.648642	5.401723	6.60E-08	4.43E-07	1.626259	3.284471	9.044508	60.26743	64.17332	31.15436
14660	KCNK15	2.574921	3.502933	1.847673	1.895862	0.057978	0.10514	0.542086	0	0.695731	8.928508	4.278221	1.004979
8125	TDRD5	9.027015	3.502607	1.03872	3.372043	0.000746	0.002441	0.542086	1.094824	2.782925	22.32127	21.39111	6.029877
11326	LOC11962(	5.297326	3.502552	1.372607	2.551751	0.010718	0.025159	0	0.547412	2.087194	11.16063	14.97377	3.014938
4317	MRO	22.83254	3.502258	0.681022	5.142649	2.71E-07	1.67E-06	2.168345	2.737059	6.261582	49.10679	55.61688	21.10457
9337	LOC11962:	8.892464	3.502111	1.158761	3.02229	0.002509	0.007143	1.084173	1.094824	2.087194	24.5534	23.53022	1.004979
12039	LOC11962:	35.42409	3.501638	1.459569	2.39909	0.016436	0.036294	0	2.737059	14.61036	62.49956	100.5382	32.15934
9230	LOC10322(	7.820604	3.501479	1.149046	3.047292	0.002309	0.006651	0	1.094824	2.782925	15.62489	21.39111	6.029877
6274	LOC10322:	15.72056	3.501065	0.866433	4.040777	5.33E-05	0.000226	1.084173	0.547412	6.261582	24.5534	42.78221	19.09461
14289	FAM181B	2.523278	3.501	1.782707	1.963867	0.049545	0.09218	0	0.547412	0.695731	4.464254	6.417332	3.014938
15911	BPIFA2	2.774048	3.500991	2.081018	1.682346	0.092502	0.154547	0	0	1.391463	6.696381	8.556443	0
15913	SULT1E1	2.774048	3.500991	2.081018	1.682346	0.092502	0.154547	0	0	1.391463	6.696381	8.556443	0
15326	TMEM35A	16.97457	3.500961	1.972631	1.774768	0.075936	0.131722	0	0	8.348776	40.17829	34.22577	19.09461
15012	LOC10322:	2.721517	3.500648	1.911979	1.830903	0.067115	0.118831	0	0	1.391463	2.232127	10.69555	2.009959
15013	TAAR1	2.721517	3.500648	1.911979	1.830903	0.067115	0.118831	0	0	1.391463	2.232127	10.69555	2.009959
15014	LOC11962!	2.721517	3.500648	1.911979	1.830903	0.067115	0.118831	0	0	1.391463	2.232127	10.69555	2.009959

10004	DUOXA1	6.452094	3.500597	1.224006	2.859951	0.004237	0.01126	0	1.094824	2.087194	13.39276	17.11289	5.024897
11060	LOC10324E	5.135227	3.499634	1.33924	2.613149	0.008971	0.021564	0.542086	0.547412	1.391463	15.62489	10.69555	2.009959
14668	LOC10323Z	2.575809	3.499386	1.847319	1.894305	0.058185	0.105456	0	0.547412	0.695731	8.928508	4.278221	1.004979
5915	AMER2	21.17637	3.499276	0.832433	4.203674	2.63E-05	0.000118	0.542086	1.642235	8.348776	49.10679	51.33866	16.07967
9324	LOC11962	7.805101	3.498807	1.156645	3.024963	0.002487	0.007091	0	1.094824	2.782925	13.39276	23.53022	6.029877
15190	CD79B	2.470747	3.497668	1.945569	1.797761	0.072215	0.126388	0	0.547412	0.695731	0	8.556443	5.024897
10615	CUNH5orf5	5.082696	3.497635	1.287339	2.716949	0.006589	0.016501	0.542086	0.547412	1.391463	11.16063	12.83466	4.019918
10218	KRT35	9.476911	3.497029	1.243879	2.811389	0.004933	0.012834	0	0.547412	4.174388	22.32127	27.80844	2.009959
11620	LOC10324E	4.957626	3.497015	1.404871	2.489208	0.012803	0.029291	1.084173	0.547412	0.695731	17.85702	8.556443	1.004979
7941	CSF3R	14.93288	3.496917	1.020701	3.425997	0.000613	0.002051	0	0.547412	6.957314	37.94616	32.08666	12.05975
9279	PAK5	6.184934	3.496314	1.151037	3.037535	0.002385	0.006834	0.542086	1.094824	1.391463	8.928508	17.11289	8.039836
8934	PTPRT	10.77137	3.495617	1.114582	3.136259	0.001711	0.005092	0	0.547412	4.87012	20.08914	32.08666	7.034856
12696	LOC11962€	3.913313	3.495506	1.546585	2.260144	0.023812	0.049862	0	0.547412	1.391463	6.696381	12.83466	2.009959
7704	KY	8.562503	3.495403	1.001074	3.491655	0.00048	0.001656	0.542086	2.189647	1.391463	20.08914	17.11289	10.04979
9097	TSPAN32	12.35105	3.49432	1.132979	3.084189	0.002041	0.005965	0	0	6.261582	31.24978	23.53022	13.06473
14514	LOC10324:	2.507775	3.49387	1.818706	1.921075	0.054722	0.100234	0	0.547412	0.695731	2.232127	8.556443	3.014938
8054	LOC11962	13.46533	3.493563	1.028064	3.398195	0.000678	0.002239	0	0.547412	6.261582	24.5534	36.36488	13.06473
9467	LOC10322:	8.969251	3.493259	1.166796	2.99389	0.002754	0.007734	1.626259	0	2.782925	11.16063	34.22577	4.019918
11535	IKZF3	5.105109	3.492894	1.393398	2.506745	0.012185	0.028083	0	1.094824	1.391463	11.16063	14.97377	2.009959
12882	SYNPR	3.89781	3.490643	1.570926	2.222029	0.026281	0.054233	0	0.547412	1.391463	4.464254	14.97377	2.009959
12883	LOC11962	3.89781	3.490643	1.570926	2.222029	0.026281	0.054233	0	0.547412	1.391463	4.464254	14.97377	2.009959
4755	LOC10322(	29.70803	3.490587	0.721087	4.840728	1.29E-06	7.23E-06	1.084173	3.284471	10.43597	40.17829	94.12087	29.1444
7148	LOC10323(	15.73438	3.4898	0.948056	3.681007	0.000232	0.000864	0	1.642235	6.261582	20.08914	51.33866	15.07469
15343	LOC10321!	2.491385	3.489146	1.96906	1.771986	0.076397	0.132374	0.542086	0	0.695731	0	10.69555	3.014938
13721	SLCO1B7	3.53836	3.489002	1.688664	2.066132	0.038816	0.075197	0	1.642235	0	11.16063	6.417332	2.009959
12112	TEKT3	3.714186	3.488444	1.463482	2.383661	0.017141	0.037624	0.542086	0.547412	0.695731	8.928508	8.556443	3.014938
14725	GSC2	2.543916	3.48827	1.850965	1.884568	0.059488	0.107402	0.542086	0	0.695731	4.464254	8.556443	1.004979
15288	LOC10323!	2.292258	3.488227	1.957721	1.781779	0.074785	0.130048	1.084173	0	0	2.232127	6.417332	4.019918
7777	LOC10321	10.71587	3.487975	1.004904	3.470952	0.000519	0.001773	2.710432	1.642235	0.695731	17.85702	36.36488	5.024897
10832	LOC11962(	6.067661	3.48792	1.30932	2.663918	0.007724	0.018956	0	2.189647	0.695731	13.39276	17.11289	3.014938
15129	LOC10323	2.309536	3.487155	1.927243	1.809401	0.070389	0.123689	0	1.094824	0	4.464254	4.278221	4.019918
246	PTK2B	304.8116	3.486956	0.187782	18.56918	5.71E-77	6.17E-75	53.66655	43.79295	52.87558	551.3354	526.2212	600.9777
12770	ZNF781	3.515947	3.486806	1.551747	2.247019	0.024639	0.051294	0.542086	1.094824	0	11.16063	4.278221	4.019918
5884	GPD1	20.12981	3.485872	0.825635	4.222048	2.42E-05	0.000109	0	4.379295	5.565851	42.41041	51.33866	17.08465
14738	CALY	2.544803	3.484714	1.850612	1.883006	0.0597	0.107681	0	0.547412	0.695731	4.464254	8.556443	1.004979
7414	LTF	11.54835	3.484442	0.969216	3.595114	0.000324	0.001163	0	1.642235	4.174388	29.01765	21.39111	13.06473
11106	LOC10321!	4.654325	3.483897	1.337997	2.603815	0.009219	0.022068	1.084173	1.094824	0	11.16063	8.556443	6.029877
4337	TMEM196	38.95633	3.482834	0.679193	5.127903	2.93E-07	1.80E-06	1.084173	3.831883	14.61036	69.19594	104.8164	40.19918
14239	SHISAL2A	4.096049	3.482345	1.768438	1.969165	0.048934	0.091363	0	0	2.087194	2.232127	19.252	1.004979
3095	YPEL4	29.6682	3.481789	0.553839	6.286642	3.24E-10	2.79E-09	3.794605	7.116354	3.478657	69.19594	49.19955	45.22408
16391	CUNH1orf	2.352587	3.481601	2.190595	1.58934	0.111984	0.181629	0	1.094824	0	4.464254	8.556443	0
7296	SLC22A7	12.90738	3.479796	0.957067	3.635895	0.000277	0.001009	0	1.642235	4.87012	26.78552	32.08666	12.05975
14631	LOC10324	189.2441	3.479711	1.83024	1.901233	0.057272	0.104064	0	9.306001	84.1835	314.7299	543.3341	183.9112
4460	NTRK2	26.60077	3.479419	0.690972	5.035547	4.76E-07	2.84E-06	0.542086	3.831883	9.044508	49.10679	59.8951	37.18424
14743	MMP12	2.293146	3.479414	1.848704	1.882083	0.059825	0.10787	0.542086	0.547412	0	2.232127	6.417332	4.019918
14744	POMC	2.293146	3.479414	1.848704	1.882083	0.059825	0.10787	0.542086	0.547412	0	2.232127	6.417332	4.019918
15744	LOC11962(	2.367202	3.47856	2.035066	1.709311	0.087393	0.147571	0.542086	0.547412	0	6.696381	6.417332	0
8493	LOC10321!	10.41936	3.477627	1.067542	3.257603	0.001124	0.003517	1.084173	0	4.174388	24.5534	25.66933	7.034856
357	LOC11962	366.859	3.477264	0.213636	16.2766	1.45E-59	1.08E-57	72.09749	47.07742	62.61582	743.2983	528.3603	747.7047
7965	MAGEA9	11.75261	3.477045	1.016479	3.420677	0.000625	0.002085	0	1.094824	4.87012	24.5534	29.94755	10.04979
7802	PLG	14.69133	3.476637	1.00344	3.464717	0.000531	0.001809	0	0.547412	6.957314	33.4819	32.08666	15.07469
8922	LOC11961!	7.677202	3.476546	1.107558	3.138929	0.001696	0.005053	0.542086	0.547412	2.782925	22.32127	12.83466	7.034856
10022	KLK13	6.396735	3.474323	1.21646	2.856094	0.004289	0.011377	0	0.547412	2.782925	11.16063	12.83466	11.05477
6541	LOC10323:	12.18147	3.473614	0.885266	3.923807	8.72E-05	0.000354	2.168345	1.642235	2.087194	33.4819	25.66933	8.039836
3295	LOC11962!	30.90362	3.473011	0.574502	6.045258	1.49E-09	1.20E-08	4.336691	2.189647	9.044508	64.73168	59.8951	45.22408
15299	RXFP2	2.331061	3.472492	1.951382	1.779504	0.075157	0.1306	0	1.094824	0	4.464254	6.417332	2.009959
3368	LOC10323:	39.62291	3.471407	0.581374	5.971037	2.36E-09	1.86E-08	2.710432	6.568942	10.43597	75.89232	106.9555	35.17428
10189	MPZL2	6.469903	3.471403	1.231903	2.817919	0.004834	0.012612	0.542086	0	2.782925	15.62489	12.83466	7.034856
5762	LOC10323	17.91922	3.471346	0.808404	4.294072	1.75E-05	8.09E-05	0.542086	1.642235	6.957314	33.4819	42.78221	22.10955
10252	TP53TG5	7.951439	3.470896	1.239241	2.800823	0.005097	0.013218	0	0	4.174388	15.62489	12.83466	15.07469
7291	LOC11962E	14.03193	3.470529	0.954183	3.637172	0.000276	0.001005	1.084173	1.094824	4.87012	15.62489	53.47777	8.039836
10436	LOC11962:	6.506932	3.469	1.257569	2.758496	0.005807	0.014792	0.542086	0	2.782925	17.85702	12.83466	5.024897
9270	SHISA6	12.16805	3.468986	1.141018	3.040255	0.002364	0.006779	0	0	6.261582	29.01765	25.66933	12.05975
6960	LOC10322	11.05274	3.467992	0.924043	3.753062	0.000175	0.000667	1.626259	1.094824	2.782925	13.39276	36.36488	11.05477
9439	LOC10323(	6.240659	3.467698	1.156209	2.999195	0.002707	0.007624	0.542086	0.547412	2.087194	13.39276	12.83466	8.039836
11224	KCNJ6	8.142601	3.466063	1.345893	2.575289	0.010016	0.023723	0	0	4.174388	24.5534	17.11289	3.014938
6144	LOC10321	19.34193	3.464989	0.845155	4.099829	4.13E-05	0.000179	1.084173	1.094824	7.653045	31.24978	59.8951	15.07469
6501	LOC10322E	15.38646	3.462612	0.878834	3.940007	8.15E-05	0.000333	0.542086	1.094824	6.261582	24.5534	42.78221	17.08465
12051	LOC10322	5.331526	3.461817	1.444167	2.397103	0.016525	0.036455	0	0	2.782925	15.62489	8.556443	5.024897
10490	LOC11962(	8.000512	3.461716	1.260244	2.746861	0.006017	0.015249	0	0	4.174388	13.39276	21.39111	9.044815
9237	LOC10321	12.04749	3.460676	1.136255	3.045685	0.002322	0.006682	0	0	6.261582	17.85702	32.08666	16.07967
12600	LOC10322	3.658828	3.46063	1.515439	2.283582	0.022396	0.047254	0.542086	0	1.391463	6.696381	4.278221	9.044815
7331	KRT15	14.33675	3.460553	0.955244	3.622692	0.000292	0.001057	0.542086	0.547412	6.261582	31.24978	36.36488	11.05477
11905	LOC10321!	5.204939	3.459982	1.4276	2.423636	0.015366	0.034314	0	0	2.782925	6.696381	10.69555	11.05477

7350	PLAAT4	12.93121	3.459324	0.956626	3.616173	0.000299	0.001081	0.542086	0.547412	5.565851	26.78552	32.08666	12.05975
11580	LOC10322!	6.55087	3.458635	1.384046	2.49893	0.012457	0.028598	0	0.547412	2.782925	17.85702	17.11289	1.004979
9036	DMRTA2	7.292769	3.458396	1.114339	3.103539	0.001912	0.005626	0.542086	1.642235	1.391463	22.32127	12.83466	5.024897
10165	SLC5A9	7.798815	3.456861	1.224769	2.822461	0.004766	0.012464	0	0.547412	3.478657	8.928508	27.80844	6.029877
13547	LOC10323:	3.941491	3.455379	1.646292	2.098886	0.035827	0.070308	0	0	2.087194	13.39276	2.139111	6.029877
12574	AQP4	3.696743	3.453196	1.508495	2.289166	0.02207	0.046662	0	0.547412	1.391463	8.928508	4.278221	7.034856
3320	LOC10323!	30.182	3.452476	0.573211	6.023044	1.71E-09	1.37E-08	2.710432	7.116354	5.565851	35.71403	55.61688	74.36848
8089	ARR3	10.12103	3.451572	1.020127	3.383474	0.000716	0.002352	0	1.094824	4.174388	17.85702	23.53022	14.06971
10238	ARSH	7.591096	3.451522	1.229986	2.806147	0.005014	0.013019	0	1.094824	2.782925	6.696381	29.94755	5.024897
11394	LOC11962:	5.108304	3.451232	1.359527	2.538553	0.011131	0.025972	0	0.547412	2.087194	11.16063	12.83466	4.019918
12976	ZBP1	3.749274	3.450119	1.56279	2.207667	0.027268	0.055865	0	0.547412	1.391463	13.39276	2.139111	5.024897
8714	MMP25	7.192843	3.449488	1.078485	3.198458	0.001382	0.004215	1.084173	1.094824	1.391463	11.16063	21.39111	7.034856
11215	LOC10324(	4.910065	3.448652	1.337767	2.577917	0.00994	0.023562	0	1.094824	1.391463	13.39276	8.556443	5.024897
7243	LOC11962(	9.802852	3.448551	0.943617	3.654608	0.000258	0.000945	1.084173	1.094824	2.782925	24.5534	19.252	10.04979
13239	LOC11961!	3.947513	3.44844	1.597174	2.159088	0.030843	0.061936	0	0	2.087194	11.16063	6.417332	4.019918
9684	CUNH19or	6.06908	3.44819	1.174594	2.935643	0.003329	0.009138	1.084173	0.547412	1.391463	13.39276	14.97377	5.024897
10751	LOC10324:	4.65752	3.44744	1.286855	2.678965	0.007385	0.018262	1.084173	0.547412	0.695731	11.16063	6.417332	8.039836
70	GPRIN3	1839.238	3.447124	0.128891	26.74445	1.43E-157	5.44E-155	264.5381	361.8392	300.556	3283.459	3332.734	3492.304
5790	CUX2	19.38001	3.446363	0.805195	4.28016	1.87E-05	8.58E-05	0	4.926706	4.87012	46.87467	38.50399	21.10457
556	CUNH11or	156.5895	3.446255	0.244291	14.10719	3.43E-45	1.64E-43	27.6464	28.46541	22.2634	287.9444	305.8928	267.3245
13246	LOC10324:	3.786302	3.44596	1.59659	2.158326	0.030903	0.062022	0	0.547412	1.391463	15.62489	2.139111	3.014938
8326	CHI3L2	8.757651	3.443717	1.041602	3.306173	0.000946	0.00302	0.542086	0.547412	3.478657	13.39276	23.53022	11.05477
9282	LOC11962:	7.450265	3.443617	1.133849	3.037103	0.002389	0.006842	1.084173	0	2.782925	20.08914	10.69555	10.04979
9063	LOC10323:	9.003286	3.442915	1.113494	3.091992	0.001988	0.005832	0	0.547412	4.174388	17.85702	21.39111	10.04979
10506	LOC10323:	6.03985	3.442903	1.255008	2.743331	0.006082	0.01539	0	1.642235	1.391463	8.928508	19.252	5.024897
8255	LANCL3	10.76372	3.44215	1.034421	3.327612	0.000876	0.002821	0	3.284471	2.087194	20.08914	32.08666	7.034856
11065	LOC11962:	4.930702	3.441539	1.317297	2.612576	0.008986	0.021591	0.542086	0.547412	1.391463	13.39276	10.69555	3.014938
128	LRRC4C	535.6355	3.441365	0.148825	23.1236	2.68E-118	5.57E-116	83.4813	100.1764	86.96642	984.368	983.9909	974.8301
9838	NEFH	7.701922	3.440752	1.187349	2.897844	0.003757	0.010153	0.542086	0	3.478657	22.32127	12.83466	7.034856
7489	KCNQ2	15.33093	3.440538	0.9646	3.566804	0.000361	0.001283	0	1.642235	6.261582	31.24978	42.78221	10.04979
8600	FAM9C	10.09604	3.440256	1.065398	3.22908	0.001242	0.003839	0	1.094824	4.174388	11.16063	32.08666	12.05975
88	TSPAN18	1177.492	3.439073	0.13728	25.05146	1.68E-138	5.08E-136	183.2252	236.4819	176.02	2140.61	2083.494	2245.124
12765	CKMT2	3.754409	3.438462	1.529798	2.247658	0.024598	0.051229	0.542086	0	1.391463	11.16063	6.417332	3.014938
13288	LOC10323!	3.916508	3.438415	1.598639	2.150839	0.031489	0.063	0	0	2.087194	6.696381	10.69555	4.019918
12332	FAM178B	3.451109	3.438195	1.470549	2.338034	0.019385	0.041791	0.542086	0.547412	0.695731	4.464254	6.417332	8.039836
11519	LOC11962!	4.916087	3.438109	1.370185	2.509229	0.0121	0.027924	0	1.094824	1.391463	11.16063	12.83466	3.014938
9993	LOC11962:	10.62257	3.437241	1.200558	2.863036	0.004196	0.011163	0	0	5.565851	22.32127	27.80844	8.039836
8874	LOC10324:	8.743036	3.437008	1.09132	3.149405	0.001636	0.004901	0	1.094824	3.478657	11.16063	25.66933	11.05477
8712	ACP7	8.618853	3.436548	1.074265	3.198977	0.001379	0.004209	0	1.642235	2.782925	17.85702	21.39111	8.039836
9363	LOC10324:	8.890261	3.436511	1.139593	3.015561	0.002565	0.007283	0.542086	0.547412	3.478657	20.08914	25.66933	3.014938
6340	GUCY2F	19.02244	3.435746	0.856909	4.009465	6.09E-05	0.000255	1.084173	1.094824	7.653045	33.4819	57.75599	13.06473
15091	ZBTB16	52.97001	3.434954	1.889502	1.817915	0.069077	0.121689	0	0.547412	26.43779	102.6778	136.9031	51.25395
9663	LOC11961!	7.618386	3.434654	1.167285	2.94243	0.003256	0.008959	0.542086	0	3.478657	13.39276	19.252	9.044815
13569	NLRP4	3.953536	3.434542	1.639505	2.094866	0.036183	0.070889	0	0	2.087194	8.928508	10.69555	2.009959
5365	FOXI1	32.93869	3.433765	0.766144	4.481878	7.40E-06	3.67E-05	3.252518	0.547412	13.2189	44.64254	104.8164	31.15436
10650	FFAR3	7.879524	3.432751	1.269093	2.704885	0.006833	0.017056	0	0	4.174388	20.08914	14.97377	8.039836
13464	CRYBB1	3.901005	3.431529	1.6232	2.114052	0.034511	0.068137	0	0	2.087194	4.464254	12.83466	4.019918
13465	CCDC196	3.901005	3.431529	1.6232	2.114052	0.034511	0.068137	0	0	2.087194	4.464254	12.83466	4.019918
1001	CREG2	107.1096	3.431528	0.312383	10.98499	4.51E-28	1.20E-26	21.14137	13.6853	20.17621	194.195	158.2942	235.1652
11291	GPR35	4.884194	3.431062	1.340255	2.560006	0.010467	0.024641	0.542086	0.547412	1.391463	6.696381	17.11289	3.014938
12730	LOC10324:	3.739794	3.431051	1.523707	2.25178	0.024336	0.050823	0	0.547412	1.391463	8.928508	8.556443	3.014938
5707	SV2B	15.18384	3.430908	0.793618	4.323122	1.54E-05	7.17E-05	4.336691	0.547412	2.782925	31.24978	32.08666	20.09959
154	PDZRN3	438.9367	3.430838	0.156923	21.86323	5.82E-106	1.00E-103	70.47123	78.8273	74.44326	772.3159	806.4447	831.118
6606	MAGEC1	21.5606	3.430811	0.87969	3.900022	9.62E-05	0.000387	0	2.189647	9.044508	24.5534	68.45154	25.12449
8601	ASCL4	14.16669	3.429228	1.062105	3.228709	0.001244	0.003844	0	1.094824	6.261582	24.5534	47.06043	6.029877
13657	ZNF544	3.938033	3.428849	1.651289	2.076468	0.037851	0.073681	0	0	2.087194	6.696381	12.83466	2.009959
7741	LOC10322:	13.74737	3.428237	0.984857	3.480948	0.0005	0.001716	0.542086	1.642235	4.87012	46.87467	23.53022	5.024897
10946	LOC11961{	4.648927	3.428206	1.298695	2.639732	0.008297	0.020152	0.542086	1.094824	0.695731	6.696381	12.83466	6.029877
6268	ABCA9	13.77557	3.425862	0.847319	4.043177	5.27E-05	0.000224	3.794605	1.642235	1.391463	44.64254	17.11289	14.06971
8698	KCNMB3	9.955046	3.424678	1.068854	3.204065	0.001355	0.004142	0.542086	1.094824	3.478657	33.4819	17.11289	4.019918
15218	LOC10323:	2.489445	3.424579	1.91074	1.792279	0.073088	0.127665	0	0	1.391463	2.232127	4.278221	7.034856
15219	DACH2	2.489445	3.424579	1.91074	1.792279	0.073088	0.127665	0	0	1.391463	2.232127	4.278221	7.034856
15009	LOC11962(	2.526473	3.424366	1.869478	1.831723	0.066993	0.118646	0	0	1.391463	4.464254	4.278221	5.024897
15010	TMEM238l	2.526473	3.424366	1.869478	1.831723	0.066993	0.118646	0	0	1.391463	4.464254	4.278221	5.024897
15011	HPD	2.526473	3.424366	1.869478	1.831723	0.066993	0.118646	0	0	1.391463	4.464254	4.278221	5.024897
11198	LOC10323!	6.39993	3.424143	1.325819	2.582662	0.009804	0.023276	0	0	3.478657	11.16063	10.69555	13.06473
5183	MYT1L	30.11916	3.42387	0.746281	4.587909	4.48E-06	2.30E-05	0.542086	3.284471	11.82743	75.89232	62.03421	27.13445
10527	GRM3	9.17052	3.423742	1.250235	2.738479	0.006172	0.015587	0	0	4.87012	11.16063	29.94755	9.044815
12818	LOC11961{	3.524277	3.423693	1.531985	2.234808	0.02543	0.052743	1.084173	0	0.695731	8.928508	6.417332	4.019918
7463	TMPRSS6	11.82239	3.423633	0.957388	3.576013	0.000349	0.001243	1.626259	2.189647	2.087194	26.78552	34.22577	4.019918
10185	REN	7.608906	3.423392	1.214569	2.818607	0.004823	0.01259	0.542086	0	3.478657	8.928508	25.66933	7.034856
13849	LOC11962:	3.92253	3.422393	1.677151	2.040599	0.041291	0.079263	0	0	2.087194	4.464254	14.97377	2.009959
10123	LOC11961!	5.999994	3.422379	1.208428	2.832091	0.004624	0.012145	0	1.094824	2.087194	8.928508	12.83466	11.05477
6100	MGAM2	20.47208	3.422268	0.830365	4.121403	3.77E-05	0.000164	0	4.926706	5.565851	51.33892	44.92132	16.07967

8391	STRA6	12.69961	3.42218	1.041949	3.284403	0.001022	0.003238	0	1.094824	5.565851	44.64254	12.83466	12.05975
11173	LOC10322!	6.421455	3.421893	1.323011	2.586443	0.009697	0.023074	0	0	3.478657	11.16063	12.83466	11.05477
9464	SCNN1G	7.234841	3.421068	1.142594	2.994123	0.002752	0.007731	0	1.642235	2.087194	13.39276	19.252	7.034856
9465	LILRA4	7.234841	3.421068	1.142594	2.994123	0.002752	0.007731	0	1.642235	2.087194	13.39276	19.252	7.034856
9565	LOC11961{	6.965018	3.42092	1.152385	2.968557	0.002992	0.008316	2.168345	0	1.391463	8.928508	19.252	10.04979
3770	DLGAP2	53.43514	3.420037	0.61359	5.573816	2.49E-08	1.76E-07	5.96295	1.642235	20.17621	111.6063	121.9293	59.29379
13434	LOC10323{	3.744929	3.419555	1.612661	2.120443	0.033969	0.067217	0.542086	0	1.391463	6.696381	12.83466	1.004979
15900	GUCY2D	16.14311	3.419547	2.030379	1.684192	0.092145	0.154064	0	0	8.348776	37.94616	38.50399	12.05975
11123	LOC10323:	77.69962	3.419497	1.316466	2.597482	0.009391	0.022445	1.626259	2.189647	36.17803	145.0883	211.772	69.34358
7916	LOC11962{	10.56868	3.418314	0.995453	3.433927	0.000595	0.001998	0	3.284471	2.087194	22.32127	25.66933	10.04979
8417	KCNS1	10.03272	3.41797	1.043032	3.276955	0.001049	0.003314	0.542086	0.547412	4.174388	20.08914	27.80844	7.034856
15055	LOC10322:	2.563501	3.417402	1.873985	1.823602	0.068212	0.120453	0	0	1.391463	6.696381	4.278221	3.014938
6222	FAM186A	11.89556	3.417009	0.840501	4.065445	4.79E-05	0.000205	1.084173	1.642235	3.478657	24.5534	23.53022	17.08465
13678	CD19	23.86699	3.416134	1.647999	2.072898	0.038182	0.074211	0.542086	0	11.82743	42.41041	66.31243	22.10955
7333	SLC13A4	12.50064	3.415856	0.943205	3.621542	0.000293	0.001062	0.542086	0.547412	5.565851	22.32127	29.94755	16.07967
11608	FGR	4.891734	3.414912	1.370337	2.492024	0.012702	0.029087	0	0.547412	2.087194	13.39276	4.278221	9.044815
3804	COL9A2	28.47601	3.414631	0.617002	5.534231	3.13E-08	2.18E-07	2.710432	4.926706	6.957314	66.96381	64.17332	25.12449
13022	LOC11961!	3.493272	3.414049	1.551483	2.200507	0.027771	0.056696	1.084173	0	0.695731	4.464254	10.69555	4.019918
13846	LOC10322;	3.31231	3.412984	1.672151	2.041074	0.041243	0.079189	0	1.642235	0	8.928508	4.278221	5.024897
10883	SPON1	6.18273	3.412745	1.286219	2.653316	0.007971	0.01947	0	0.547412	2.782925	4.464254	19.252	10.04979
11362	MMP24	4.859841	3.412167	1.340437	2.545562	0.01091	0.025528	0.542086	0	2.087194	8.928508	8.556443	9.044815
16204	LOC10322;	21.45354	3.411529	2.097303	1.626626	0.103816	0.170326	0	0	11.1317	35.71403	57.75599	24.11951
377	CHI3L1	264.684	3.410907	0.212009	16.08851	3.07E-58	2.17E-56	42.28274	55.83601	38.26523	459.8182	449.2132	542.6889
11508	AOAH	6.449003	3.410345	1.357185	2.512807	0.011977	0.02767	0	0	3.478657	8.928508	19.252	7.034856
13199	HAO2	3.547577	3.409179	1.571569	2.169284	0.030061	0.060548	0	1.094824	0.695731	8.928508	8.556443	2.009959
14929	CLEC1B	2.297228	3.408726	1.843513	1.849039	0.064452	0.114774	0	0.547412	0.695731	2.232127	4.278221	6.029877
14511	LOC11961{	3.5536	3.40852	1.773824	1.921566	0.05466	0.100141	0	1.094824	0.695731	6.696381	12.83466	0
12268	RAMP3	5.015917	3.406416	1.448735	2.351304	0.018708	0.04054	0	0	2.782925	6.696381	8.556443	12.05975
12683	CUNH16or	3.54669	3.406377	1.50507	2.263268	0.023619	0.049508	0.542086	0.547412	0.695731	8.928508	8.556443	2.009959
6027	LOC10324	14.07709	3.40576	0.820131	4.1527	3.29E-05	0.000145	3.252518	0.547412	3.478657	29.01765	32.08666	16.07967
12843	MEOX2	3.478656	3.40566	1.526979	2.230325	0.025726	0.053253	0.542086	0.547412	0.695731	2.232127	12.83466	4.019918
9845	LOC11962(	6.665896	3.405244	1.176123	2.895313	0.003788	0.010228	1.084173	2.189647	0	17.85702	12.83466	6.029877
8785	CUNH6orf:	8.61168	3.404947	1.071311	3.178299	0.001481	0.004483	0.542086	0.547412	3.478657	13.39276	25.66933	8.039836
8190	CAPN6	8.282348	3.404927	1.016712	3.348958	0.000811	0.002633	1.084173	1.094824	2.087194	24.5534	12.83466	8.039836
14893	LOC10322;	2.349759	3.40486	1.834874	1.855637	0.063505	0.113361	0	0.547412	0.695731	6.696381	2.139111	4.019918
13258	LOC11962(	3.532075	3.404286	1.578746	2.156323	0.031058	0.062279	0	1.094824	0.695731	6.696381	10.69555	2.009959
12450	ICAM2	5.164029	3.404068	1.473064	2.310876	0.02084	0.0445	0	0	2.782925	15.62489	8.556443	4.019918
1769	ZMAT4	59.46231	3.403262	0.398656	8.536847	1.38E-17	2.07E-16	9.215468	14.23271	6.957314	102.6778	126.2075	97.48301
10279	ZNF878	9.028006	3.402909	1.218013	2.793819	0.005209	0.013472	0	0	4.87012	17.85702	21.39111	10.04979
12403	LOC10321!	5.148526	3.400625	1.465402	2.320609	0.020308	0.043529	0	0	2.782925	13.39276	10.69555	4.019918
13415	LOC11962	3.516572	3.400247	1.600995	2.123834	0.033684	0.066753	0	1.094824	0.695731	4.464254	12.83466	2.009959
15327	LOC10321!	2.532495	3.399559	1.915892	1.7744	0.075997	0.131818	0	0	1.391463	2.232127	8.556443	3.014938
8007	HCAR1	11.97909	3.399107	0.996764	3.410143	0.000649	0.002156	0.542086	2.189647	3.478657	17.85702	42.78221	5.024897
6675	WFIKKN2	25.39591	3.398901	0.878831	3.867524	0.00011	0.000438	0	1.642235	11.82743	58.0353	57.75599	23.11453
6426	COL25A1	16.62094	3.397999	0.855873	3.970214	7.18E-05	0.000297	0	3.831883	4.87012	29.01765	44.92132	17.08465
15080	LOC11962:	2.385899	3.396849	1.866808	1.819602	0.06882	0.121324	0.542086	0	0.695731	8.928508	2.139111	2.009959
14056	CPA6	3.484679	3.396527	1.69597	2.002705	0.045209	0.085507	0.542086	0.547412	0.695731	0	17.11289	2.009959
10174	LOC11962;	5.886081	3.39594	1.204116	2.820276	0.004798	0.012538	1.084173	0.547412	1.391463	11.16063	17.11289	4.019918
6738	RIMBP2	23.83173	3.395226	0.883767	3.841767	0.000122	0.000482	0	2.189647	10.43597	49.10679	64.17332	17.08465
7960	LOC11962!	12.41757	3.39326	0.991634	3.421887	0.000622	0.002077	0	1.094824	5.565851	31.24978	23.53022	13.06473
7424	SEC14L5	10.79049	3.392831	0.944924	3.590584	0.00033	0.001181	1.084173	0.547412	4.174388	29.01765	12.83466	17.08465
4608	NLRC3	21.43915	3.392449	0.686847	4.939164	7.85E-07	4.53E-06	1.626259	2.189647	7.653045	37.94616	47.06043	32.15934
10379	INA	7.347344	3.391847	1.224677	2.769585	0.005613	0.014375	1.084173	0	2.782925	20.08914	17.11289	3.014938
9286	LOC10322;	8.084899	3.390381	1.117019	3.035205	0.002404	0.006882	2.168345	0.547412	1.391463	17.85702	23.53022	3.014938
12867	FAM71B	5.154549	3.390036	1.523407	2.225299	0.026061	0.053846	0	0	2.782925	11.16063	14.97377	2.009959
7550	ACOXL	16.30909	3.389413	0.957073	3.541435	0.000398	0.001401	0	1.094824	7.653045	31.24978	42.78221	15.07469
10510	STUM	7.509443	3.38939	1.235906	2.742433	0.006099	0.015426	0.542086	0	3.478657	15.62489	21.39111	4.019918
8296	RXFP1	9.593817	3.388569	1.022459	3.314137	0.000919	0.002946	0	1.642235	3.478657	17.85702	23.53022	11.05477
6288	RPRM	19.86155	3.388519	0.83996	4.034141	5.48E-05	0.000232	1.084173	0.547412	9.044508	46.87467	38.50399	23.11453
14939	DNALI1	2.370396	3.388195	1.834398	1.847033	0.064742	0.115205	0.542086	0	0.695731	6.696381	4.278221	2.009959
11176	TEX13B	9.009046	3.385571	1.309186	2.586011	0.009709	0.023094	0	0	4.87012	8.928508	34.22577	6.029877
14950	PRM3	2.371284	3.384694	1.834047	1.845478	0.064968	0.11553	0	0.547412	0.695731	6.696381	4.278221	2.009959
16005	LOC10324(	2.408312	3.38442	2.033886	1.664017	0.096109	0.159646	0	0.547412	0.695731	8.928508	4.278221	0
10455	C6	5.930649	3.383489	1.228244	2.754737	0.005874	0.014935	0.542086	0.547412	2.087194	20.08914	4.278221	8.039836
16039	LOC11962	2.172157	3.383433	2.040328	1.658279	0.097261	0.161213	0.542086	0.547412	0	8.928508	0	3.014938
11169	LOC11962(	4.726178	3.382998	1.307728	2.586928	0.009684	0.023049	0.542086	0.547412	1.391463	11.16063	10.69555	4.019918
10590	S1PR4	6.077245	3.382292	1.241218	2.724977	0.006431	0.016143	0	0.547412	2.782925	13.39276	10.69555	9.044815
10681	SBSN	7.479325	3.381796	1.25414	2.696507	0.007007	0.017441	0	0.547412	3.478657	11.16063	25.66933	4.019918
7034	BTBD17	10.51987	3.381592	0.908748	3.721156	0.000198	0.00075	1.626259	0.547412	3.478657	17.85702	23.53022	16.07967
10730	SLC26A4	5.987686	3.380598	1.259213	2.684692	0.00726	0.017987	0	0.547412	2.782925	6.696381	12.83466	13.06473
13268	PAX7	25.70566	3.380094	1.568941	2.15438	0.03121	0.062536	0	1.094824	12.52316	44.64254	74.86887	21.10457
6429	CELF3	24.99242	3.379886	0.851714	3.968336	7.24E-05	0.000299	0.542086	1.094824	11.82743	33.4819	74.86887	28.13943
7058	NYAP2	15.65095	3.379159	0.911259	3.708233	0.000209	0.000786	2.168345	0.547412	5.565851	37.94616	40.6431	7.034856
6037	RSPH1	18.18717	3.378755	0.814524	4.148134	3.35E-05	0.000148	0.542086	1.642235	7.653045	31.24978	44.92132	23.11453

8635	LOC11962	11.09211	3.378733	1.049673	3.218845	0.001287	0.003963	0	1.094824	4.87012	29.01765	23.53022	8.039836
7790	DMRTC2	16.06994	3.378081	0.973995	3.468273	0.000524	0.001788	0	1.642235	6.957314	42.41041	36.36488	9.044815
14972	LIPK	2.355781	3.377063	1.835845	1.839515	0.06584	0.116908	0	0.547412	0.695731	4.464254	6.417332	2.009959
13513	KHDC3L	3.705961	3.376295	1.603697	2.105319	0.035264	0.069371	0	0	2.087194	6.696381	6.417332	7.034856
10512	LOC10323	6.927658	3.375985	1.231318	2.741766	0.006111	0.015455	0	2.189647	1.391463	17.85702	17.11289	3.014938
11623	MMP8	6.723299	3.37575	1.35689	2.487858	0.012851	0.029395	1.626259	1.094824	0.695731	13.39276	23.53022	0
15994	CYP2C8	2.392809	3.375574	2.025336	1.666674	0.095579	0.158871	0	0.547412	0.695731	6.696381	6.417332	0
5854	NPHS1	16.51421	3.374886	0.795371	4.243162	2.20E-05	0.0001	2.710432	0.547412	5.565851	37.94616	34.22577	18.08963
8394	CUNHXorf	13.47544	3.37406	1.02769	3.28315	0.001027	0.003251	0	1.642235	5.565851	31.24978	36.36488	6.029877
13343	C4BPB	5.071013	3.373244	1.577464	2.138397	0.032485	0.064723	0	0	2.782925	2.232127	21.39111	4.019918
11703	LOC11962	4.7322	3.372375	1.364384	2.47172	0.013446	0.030546	0.542086	0.547412	1.391463	8.928508	14.97377	2.009959
16066	LOC11962	2.376419	3.371672	2.038757	1.653788	0.098171	0.162452	0.542086	0	0.695731	4.464254	8.556443	0
2840	ADCYAP1R	54.40412	3.371265	0.508424	6.63082	3.34E-11	3.12E-10	4.336691	9.853413	14.61036	111.6063	134.764	51.25395
9846	CRYGN	7.056976	3.371264	1.164509	2.89501	0.003791	0.010237	0.542086	1.094824	2.087194	8.928508	25.66933	4.019918
163	TRIM22	527.7094	3.370636	0.156882	21.48515	2.14E-102	3.50E-100	102.4543	94.70224	81.40057	1006.689	877.0354	1003.974
7590	LOC11962	11.73529	3.370322	0.954958	3.529287	0.000417	0.001459	1.626259	4.379295	0	33.4819	12.83466	18.08963
7269	ALX4	21.2841	3.369608	0.924455	3.644968	0.000267	0.000978	0	1.094824	10.43597	46.87467	49.19955	20.09959
10600	MAB21L4	5.922056	3.369505	1.237702	2.722389	0.006481	0.016255	0	1.094824	2.087194	15.62489	10.69555	6.029877
10668	SYCP1	6.698414	3.369375	1.248086	2.699634	0.006942	0.017299	0	2.737059	0.695731	15.62489	17.11289	4.019918
13746	RFPL1	3.780017	3.369277	1.635675	2.05987	0.039411	0.076221	0	0	2.087194	11.16063	6.417332	3.014938
8958	PTPRC	8.209804	3.368731	1.076646	3.128912	0.001755	0.005207	0	1.642235	2.782925	13.39276	21.39111	10.04979
16685	NEUROG3	2.538518	3.368709	2.1808	1.544713	0.122416	0.195051	0	0	1.391463	0	12.83466	1.004979
13455	LOC11962	3.581777	3.368418	1.591669	2.11628	0.034321	0.067813	0	0.547412	1.391463	13.39276	2.139111	4.019918
5276	CCDC180	24.05963	3.366223	0.742358	4.534499	5.77E-06	2.91E-05	2.168345	1.094824	9.740239	58.0353	49.19955	24.11951
6810	VWC2L	12.60655	3.365613	0.882623	3.813196	0.000137	0.000536	2.710432	1.094824	2.782925	26.78552	34.22577	8.039836
15295	KRTAP4-1	2.156655	3.364731	1.89057	1.779745	0.075118	0.130566	0.542086	0.547412	0	6.696381	2.139111	3.014938
85	CSPG4	6364.862	3.364474	0.131911	25.50558	1.71E-143	5.35E-141	1212.647	955.781	1213.356	11348.13	10137.25	13322.01
3254	TMEM114	47.08412	3.363678	0.552047	6.093104	1.11E-09	9.05E-09	4.336691	4.926706	16.00182	111.6063	98.39909	47.23404
8890	LOC10323	8.251079	3.363609	1.068846	3.146954	0.00165	0.004934	1.084173	0.547412	2.782925	13.39276	25.66933	6.029877
7953	LOC10323	9.440935	3.361918	0.981881	3.423955	0.000617	0.002063	0.542086	1.094824	3.478657	20.08914	21.39111	10.04979
16267	LOC10323	2.361804	3.360879	2.081828	1.614389	0.106443	0.173959	0	0.547412	0.695731	2.232127	10.69555	0
12980	LOC11962	4.525369	3.360584	1.522417	2.2074	0.027286	0.055885	0	1.642235	0.695731	6.696381	17.11289	1.004979
12320	CRISPLD1	4.503843	3.360284	1.434911	2.341807	0.019191	0.041411	0	1.642235	0.695731	6.696381	14.97377	3.014938
14292	MSGN1	3.801542	3.359768	1.711797	1.962714	0.049679	0.09241	0	0	2.087194	11.16063	8.556443	1.004979
12144	LOC10321	6.303032	3.358877	1.412565	2.377857	0.017414	0.038121	0	0	3.478657	8.928508	21.39111	4.019918
5451	KCNH7	18.72402	3.35868	0.756569	4.439359	9.02E-06	4.40E-05	2.710432	1.094824	6.261582	42.41041	42.78221	17.08465
13493	LOC10321	4.311627	3.358532	1.592955	2.108366	0.034999	0.068953	0	2.189647	0	6.696381	14.97377	2.009959
3042	PKHD1	41.66733	3.358058	0.52886	6.349616	2.16E-10	1.89E-09	4.336691	9.306001	8.348776	89.28508	100.5382	38.18922
17137	LOC10322	2.346301	3.357596	2.277037	1.474546	0.140335	0.217704	0	0.547412	0.695731	0	12.83466	0
9222	AADACL3	11.14181	3.357502	1.101068	3.049314	0.002294	0.006612	0	0.547412	5.565851	35.71403	14.97377	10.04979
5102	CD3D	25.12033	3.357142	0.725488	4.627429	3.70E-06	1.93E-05	1.084173	2.189647	10.43597	49.10679	57.75599	30.14938
8491	DPRX	8.037338	3.357067	1.030392	3.258048	0.001122	0.003512	1.084173	1.094824	2.087194	13.39276	23.53022	7.034856
11582	VSX2	5.568629	3.356121	1.343287	2.498439	0.012474	0.028633	0	2.189647	0.695731	20.08914	6.417332	4.019918
6250	NSG2	24.46479	3.356051	0.828415	4.051172	5.10E-05	0.000217	0.542086	1.642235	11.1317	51.33892	62.03421	20.09959
10378	RBFOX1	63.90222	3.355637	1.211487	2.769848	0.005608	0.014366	1.626259	2.737059	29.91645	124.9991	166.8506	57.28383
11915	LOC10321	4.72335	3.355471	1.385676	2.421541	0.015455	0.034483	0.542086	0	2.087194	13.39276	4.278221	8.039836
15204	LOC10324	2.141152	3.354985	1.868416	1.795631	0.072553	0.126863	0.542086	0.547412	0	4.464254	4.278221	3.014938
10049	LOC11962	9.981444	3.354669	1.176597	2.851161	0.004356	0.011524	0	0	5.565851	17.85702	21.39111	15.07469
7915	LOC10323	10.08034	3.354443	0.976772	3.434212	0.000594	0.001996	1.084173	2.189647	2.087194	6.696381	36.36488	12.05975
13227	GABRA3	30.23097	3.3543	1.550302	2.163643	0.030492	0.061285	0	1.642235	14.61036	71.42806	70.59065	23.11453
14091	GRIA3	44.51944	3.353142	1.679584	1.996412	0.045889	0.086577	0	1.642235	22.2634	73.66019	128.3466	41.20416
13396	CCR4	3.5878	3.353016	1.575491	2.128236	0.033318	0.06612	0	0.547412	1.391463	11.16063	6.417332	2.009959
14057	SMIM35	41.8935	3.351914	1.67387	2.002494	0.045232	0.085543	0	1.642235	20.87194	75.89232	119.7902	33.16432
13971	LOC11962	3.733508	3.351844	1.661487	2.017376	0.043656	0.083072	0	0	2.087194	4.464254	12.83466	3.014938
6232	SLC17A8	15.28611	3.351536	0.825453	4.060237	4.90E-05	0.000209	0.542086	1.642235	6.261582	33.4819	25.66933	24.11951
9493	DDO	8.361277	3.351089	1.122386	2.985683	0.002829	0.007924	0.542086	0	4.174388	13.39276	14.97377	17.08465
14620	CLPS	3.608438	3.351034	1.760302	1.90367	0.056953	0.103564	0.542086	0	1.391463	11.16063	8.556443	0
13232	LOC11962	3.518879	3.35092	1.549749	2.162234	0.0306	0.06148	0.542086	0	1.391463	4.464254	10.69555	4.019918
18549	LOC11961	1.157963	3.350369	2.695147	1.243112	0.213827	0.306446	0	0	0.695731	2.232127	0	4.019918
18611	LOC11962	1.133243	3.349596	2.713511	1.234414	0.217049	0.309867	0	0.547412	0	2.232127	0	4.019918
11570	LOC11961	7.550553	3.349171	1.338903	2.50143	0.012369	0.028422	0	0	4.174388	17.85702	19.252	4.019918
8854	HOXC12	9.255629	3.348966	1.061202	3.155823	0.0016	0.004805	0	2.189647	2.782925	17.85702	25.66933	7.034856
15796	LYPD8	3.200704	3.347932	1.965991	1.702923	0.088582	0.149086	1.626259	0	0	11.16063	6.417332	0
7019	DCX	24.61139	3.347412	0.898063	3.727371	0.000193	0.000733	0	1.642235	11.82743	44.64254	68.45154	21.10457
10791	NLRC4	5.210135	3.347342	1.252725	2.672047	0.007539	0.018572	1.626259	1.094824	0	8.928508	8.556443	11.05477
19452	LOC10324	1.207299	3.346703	3.01588	1.109694	0.267131	0.365066	0	0.547412	0	6.696381	0	0
19453	LOC11962	1.207299	3.346703	3.01588	1.109694	0.267131	0.365066	0	0.547412	0	6.696381	0	0
19405	LOC11962	1.232019	3.345889	2.995844	1.116843	0.264061	0.361654	0	0	0.695731	6.696381	0	0
19406	BBOX1	1.232019	3.345889	2.995844	1.116843	0.264061	0.361654	0	0	0.695731	6.696381	0	0
19410	LOC10323	1.232019	3.345889	2.995844	1.116843	0.264061	0.361654	0	0	0.695731	6.696381	0	0
19411	LOC10323	1.232019	3.345889	2.995844	1.116843	0.264061	0.361654	0	0	0.695731	6.696381	0	0
16133	LOC11962	2.163565	3.345767	2.040375	1.63978	0.101051	0.166522	0	1.094824	0	4.464254	6.417332	1.004979
16137	LOC10323	2.163565	3.345767	2.040375	1.63978	0.101051	0.166522	0	1.094824	0	4.464254	6.417332	1.004979
9058	LOC10323	8.228501	3.345763	1.08177	3.092859	0.001982	0.005818	0	1.094824	3.478657	15.62489	17.11289	12.05975

6604	ABCC12	12.44942	3.344252	0.857305	3.900888	9.58E-05	0.000386	2.168345	1.642235	2.782925	31.24978	27.80844	9.044815
641	OSR1	148.691	3.34141	0.253054	13.20434	8.28E-40	3.44E-38	29.27266	22.9913	27.82925	308.0335	241.7195	262.2996
8132	TRPC5	14.63765	3.341126	0.991478	3.369843	0.000752	0.002459	0	0.547412	7.653045	24.5534	29.94755	25.12449
18467	LOC119624	1.194991	3.34075	2.665052	1.25354	0.210009	0.301974	0	0	0.695731	4.464254	0	2.009959
18468	LOC103241	1.194991	3.34075	2.665052	1.25354	0.210009	0.301974	0	0	0.695731	4.464254	0	2.009959
18472	LOC119618	1.194991	3.34075	2.665052	1.25354	0.210009	0.301974	0	0	0.695731	4.464254	0	2.009959
18474	TRNAY-GU	1.194991	3.34075	2.665052	1.25354	0.210009	0.301974	0	0	0.695731	4.464254	0	2.009959
18492	LOC119621	1.194991	3.34075	2.665052	1.25354	0.210009	0.301974	0	0	0.695731	4.464254	0	2.009959
18499	LOC119618	1.194991	3.34075	2.665052	1.25354	0.210009	0.301974	0	0	0.695731	4.464254	0	2.009959
18500	LOC119618	1.194991	3.34075	2.665052	1.25354	0.210009	0.301974	0	0	0.695731	4.464254	0	2.009959
5647	HTRA4	14.3088	3.340251	0.768006	4.349249	1.37E-05	6.43E-05	2.710432	2.737059	2.087194	29.01765	34.22577	15.07469
13241	DSG1	3.321528	3.339698	1.547171	2.158584	0.030882	0.062005	0	1.094824	0.695731	6.696381	6.417332	5.024897
124	MARCHF4	890.8347	3.33857	0.143119	23.32715	2.35E-120	5.04E-118	136.6058	188.8571	155.1481	1654.006	1589.359	1621.032
10626	NNAT	8.671488	3.338226	1.230746	2.71236	0.006681	0.016714	0	0	4.87012	17.85702	19.252	10.04979
16344	LOC119624	15.34308	3.338103	2.090503	1.596794	0.110312	0.179432	0	0	8.348776	22.32127	51.33866	10.04979
14684	MPPED1	27.91196	3.337564	1.763965	1.892081	0.05848	0.105877	0	0.547412	14.61036	62.49956	72.72976	17.08465
15570	GPR183	2.162677	3.336993	1.92526	1.733268	0.083048	0.141791	0.542086	0.547412	0	4.464254	6.417332	1.004979
6805	LOC103216	13.62634	3.334833	0.874175	3.814835	0.000136	0.000532	1.626259	2.189647	3.478657	26.78552	40.6431	7.034856
19567	LOC103233	1.095327	3.332959	3.059991	1.089205	0.276063	0.375078	0.542086	0	0	0	0	6.029877
12780	FBXO47	4.912996	3.332838	1.485857	2.243041	0.024894	0.051785	0	0	2.782925	6.696381	14.97377	5.024897
13410	KCNU1	3.358556	3.332432	1.568258	2.124925	0.033593	0.066599	0	1.094824	0.695731	8.928508	6.417332	3.014938
3267	LRP2	35.6652	3.331842	0.548344	6.076186	1.23E-09	1.00E-08	5.96295	4.926706	8.348776	58.0353	100.5382	36.17926
3686	IL1RL1	24.22829	3.33177	0.589318	5.653605	1.57E-08	1.13E-07	3.794605	4.379295	4.87012	42.41041	57.75599	32.15934
15814	FOXR2	3.187864	3.331467	1.961083	1.698789	0.089359	0.150225	0	1.642235	0	8.928508	8.556443	0
4722	KRT1	27.8948	3.331227	0.684307	4.868031	1.13E-06	6.35E-06	1.626259	3.284471	10.43597	46.87467	77.00798	28.13943
11093	RBPJL	8.699036	3.330674	1.277447	2.607289	0.009126	0.021872	0	0	4.87012	15.62489	25.66933	6.029877
13757	TBC1D28	4.809451	3.330401	1.620045	2.055746	0.039807	0.076921	0.542086	0	2.087194	13.39276	12.83466	0
11901	LOC119624	4.553546	3.330269	1.373628	2.424433	0.015332	0.03425	0	1.094824	1.391463	13.39276	6.417332	5.024897
12414	LOC119619	4.646724	3.329355	1.436373	2.31789	0.020455	0.043807	0	0.547412	2.087194	2.232127	14.97377	8.039836
10902	UCP1	5.894245	3.329062	1.256071	2.650376	0.00804	0.019606	0	0.547412	2.782925	11.16063	12.83466	8.039836
8599	LOC119624	9.237298	3.328811	1.030633	3.229869	0.001238	0.003829	0	1.642235	3.478657	17.85702	21.39111	11.05477
6524	LOC103225	26.78045	3.328781	0.847192	3.929191	8.52E-05	0.000347	0.542086	1.094824	13.2189	49.10679	70.59065	26.12947
9225	SYPL2	7.881096	3.328522	1.091679	3.048993	0.002296	0.006617	0	2.189647	2.087194	17.85702	17.11289	8.039836
6366	COL2A1	20.71824	3.327916	0.832679	3.996636	6.42E-05	0.000268	0	4.379295	6.957314	42.41041	53.47777	17.08465
10006	LOC103217	7.756913	3.327885	1.163736	2.859656	0.004241	0.011268	0	2.737059	1.391463	24.5534	12.83466	5.024897
12347	VTN	5.249728	3.327505	1.425283	2.334628	0.019563	0.042122	1.626259	1.094824	0	2.232127	23.53022	3.014938
13163	PLA2G2C	4.934522	3.326333	1.527224	2.178026	0.029404	0.059387	0	0	2.782925	6.696381	17.11289	3.014938
1704	LOC103247	58.84654	3.324837	0.381483	8.715553	2.89E-18	4.51E-17	11.9259	12.04306	7.653045	111.6063	113.3729	96.47803
18002	LOC103226	1.154768	3.324304	2.476783	1.342186	0.179536	0.26512	0	0.547412	0	2.232127	2.139111	2.009959
18003	ADAM7	1.154768	3.324304	2.476783	1.342186	0.179536	0.26512	0	0.547412	0	2.232127	2.139111	2.009959
17955	LELP1	1.179488	3.324245	2.462192	1.350116	0.176979	0.261984	0	0	0.695731	2.232127	2.139111	2.009959
17956	LOC103228	1.179488	3.324245	2.462192	1.350116	0.176979	0.261984	0	0	0.695731	2.232127	2.139111	2.009959
17957	LOC103231	1.179488	3.324245	2.462192	1.350116	0.176979	0.261984	0	0	0.695731	2.232127	2.139111	2.009959
17959	PRR15L	1.179488	3.324245	2.462192	1.350116	0.176979	0.261984	0	0	0.695731	2.232127	2.139111	2.009959
6664	CCN4	13.63839	3.323225	0.85827	3.872005	0.000108	0.000431	0.542086	2.189647	4.87012	15.62489	38.50399	20.09959
12402	CNTN6	4.705277	3.321704	1.431369	2.320648	0.020306	0.043528	0	0.547412	2.087194	4.464254	17.11289	4.019918
10964	ARGFX	5.752784	3.321071	1.259785	2.636222	0.008383	0.020326	1.084173	0	2.087194	15.62489	10.69555	5.024897
11164	LOC103235	5.86324	3.32046	1.282698	2.588654	0.009635	0.022944	0	0.547412	2.782925	6.696381	17.11289	8.039836
13369	LOC119627	4.919019	3.320444	1.557141	2.132397	0.032974	0.065571	0	0	2.782925	4.464254	19.252	3.014938
11936	GPR62	5.627714	3.320171	1.37347	2.417361	0.015634	0.03482	1.626259	0	1.391463	22.32127	6.417332	2.009959
10315	CUNH3orf8	7.109874	3.319757	1.191905	2.785253	0.005349	0.013784	0.542086	0	3.478657	15.62489	14.97377	8.039836
11952	LOC103226	4.507038	3.317948	1.37457	2.413809	0.015787	0.035115	0	1.094824	1.391463	6.696381	12.83466	5.024897
10741	LOC103234	5.702029	3.317857	1.236984	2.682216	0.007314	0.018102	0	1.094824	2.087194	11.16063	12.83466	7.034856
12803	SHH	4.688887	3.317787	1.48214	2.238511	0.025188	0.052302	0.542086	0	2.087194	2.232127	19.252	4.019918
9509	LOC103233	7.874976	3.317767	1.112332	2.982714	0.002857	0.007988	1.626259	0.547412	2.087194	11.16063	27.80844	4.019918
12303	LOC119618	4.595709	3.317366	1.415442	2.343695	0.019094	0.041261	0.542086	0.547412	1.391463	13.39276	10.69555	1.004979
18823	LOC119619	1.190908	3.316026	2.729615	1.214833	0.22443	0.316977	0.542086	0	0	4.464254	2.139111	0
18813	LOC119618	1.191796	3.315992	2.729001	1.215094	0.22433	0.316862	0	0.547412	0	4.464254	2.139111	0
18814	LOC103219	1.191796	3.315992	2.729001	1.215094	0.22433	0.316862	0	0.547412	0	4.464254	2.139111	0
18815	KRBOX1	1.191796	3.315992	2.729001	1.215094	0.22433	0.316862	0	0.547412	0	4.464254	2.139111	0
18816	LOC119618	1.191796	3.315992	2.729001	1.215094	0.22433	0.316862	0	0.547412	0	4.464254	2.139111	0
18817	LOC119619	1.191796	3.315992	2.729001	1.215094	0.22433	0.316862	0	0.547412	0	4.464254	2.139111	0
18818	LOC119620	1.191796	3.315992	2.729001	1.215094	0.22433	0.316862	0	0.547412	0	4.464254	2.139111	0
18821	PAGE3	1.191796	3.315992	2.729001	1.215094	0.22433	0.316862	0	0.547412	0	4.464254	2.139111	0
18822	LOC103222	1.191796	3.315992	2.729001	1.215094	0.22433	0.316862	0	0.547412	0	4.464254	2.139111	0
14025	CYP4F22	23.66822	3.315958	1.651032	2.008415	0.044599	0.084534	0	0.547412	12.52316	33.4819	66.31243	29.1444
18763	LOC103236	1.216516	3.315041	2.712157	1.222289	0.221598	0.313795	0	0	0.695731	4.464254	2.139111	0
18764	LOC103236	1.216516	3.315041	2.712157	1.222289	0.221598	0.313795	0	0	0.695731	4.464254	2.139111	0
18765	LALBA	1.216516	3.315041	2.712157	1.222289	0.221598	0.313795	0	0	0.695731	4.464254	2.139111	0
18766	LOC119624	1.216516	3.315041	2.712157	1.222289	0.221598	0.313795	0	0	0.695731	4.464254	2.139111	0
18767	LOC119626	1.216516	3.315041	2.712157	1.222289	0.221598	0.313795	0	0	0.695731	4.464254	2.139111	0
18768	IQCF3	1.216516	3.315041	2.712157	1.222289	0.221598	0.313795	0	0	0.695731	4.464254	2.139111	0
18769	LOC119626	1.216516	3.315041	2.712157	1.222289	0.221598	0.313795	0	0	0.695731	4.464254	2.139111	0
18770	RIBC2	1.216516	3.315041	2.712157	1.222289	0.221598	0.313795	0	0	0.695731	4.464254	2.139111	0

18771	GPR18	1.216516	3.315041	2.712157	1.222289	0.221598	0.313795	0	0	0.695731	4.464254	2.139111	0
18774	LOC10322!	1.216516	3.315041	2.712157	1.222289	0.221598	0.313795	0	0	0.695731	4.464254	2.139111	0
4051	SCUBE3	54.90004	3.314578	0.621637	5.332015	9.71E-08	6.37E-07	2.168345	6.02153	22.2634	98.21359	145.4595	55.27387
8227	GJA5	22.11283	3.313974	0.992794	3.338028	0.000844	0.002727	0	0.547412	11.82743	40.17829	62.03421	18.08963
26658	LOC11962(	0.50249	3.311691	4.047647	0.818177	0.413256	NA	0	0	0	0	0	3.014938
27013	OMD	0.50249	3.311691	4.047647	0.818177	0.413256	NA	0	0	0	0	0	3.014938
27077	LOC11962‹	0.50249	3.311691	4.047647	0.818177	0.413256	NA	0	0	0	0	0	3.014938
27245	LOC11962!	0.50249	3.311691	4.047647	0.818177	0.413256	NA	0	0	0	0	0	3.014938
27733	LOC11962.	0.50249	3.311691	4.047647	0.818177	0.413256	NA	0	0	0	0	0	3.014938
27763	LOC11962.	0.50249	3.311691	4.047647	0.818177	0.413256	NA	0	0	0	0	0	3.014938
27956	LOC11962.	0.50249	3.311691	4.047647	0.818177	0.413256	NA	0	0	0	0	0	3.014938
28033	TTR	0.50249	3.311691	4.047647	0.818177	0.413256	NA	0	0	0	0	0	3.014938
28035	LOC11961{	0.50249	3.311691	4.047647	0.818177	0.413256	NA	0	0	0	0	0	3.014938
28149	GOT1L1	0.50249	3.311691	4.047647	0.818177	0.413256	NA	0	0	0	0	0	3.014938
28243	LOC11961!	0.50249	3.311691	4.047647	0.818177	0.413256	NA	0	0	0	0	0	3.014938
28295	LOC11961!	0.50249	3.311691	4.047647	0.818177	0.413256	NA	0	0	0	0	0	3.014938
28312	LOC11961!	0.50249	3.311691	4.047647	0.818177	0.413256	NA	0	0	0	0	0	3.014938
28582	LOC10323:	0.50249	3.311691	4.047647	0.818177	0.413256	NA	0	0	0	0	0	3.014938
28783	LOC11962:	0.50249	3.311691	4.047647	0.818177	0.413256	NA	0	0	0	0	0	3.014938
28883	LOC11962:	0.50249	3.311691	4.047647	0.818177	0.413256	NA	0	0	0	0	0	3.014938
28966	LOC10323‹	0.50249	3.311691	4.047647	0.818177	0.413256	NA	0	0	0	0	0	3.014938
29005	LOC11962:	0.50249	3.311691	4.047647	0.818177	0.413256	NA	0	0	0	0	0	3.014938
29207	LOC10322!	0.50249	3.311691	4.047647	0.818177	0.413256	NA	0	0	0	0	0	3.014938
11008	LOC11962{	5.723554	3.311246	1.260127	2.627709	0.008596	0.02076	0	1.094824	2.087194	11.16063	14.97377	5.024897
14740	LOC11962{	3.098306	3.309596	1.757718	1.882893	0.059715	0.107701	0	1.642235	0	2.232127	10.69555	4.019918
18654	GADD45G	1.163985	3.30907	2.684343	1.23273	0.217677	0.310012	0	0	0.695731	0	4.278221	2.009959
18656	LOC11962‹	1.163985	3.30907	2.684343	1.23273	0.217677	0.310012	0	0	0.695731	0	4.278221	2.009959
18658	LOC11962!	1.163985	3.30907	2.684343	1.23273	0.217677	0.310012	0	0	0.695731	0	4.278221	2.009959
18659	LOC11962!	1.163985	3.30907	2.684343	1.23273	0.217677	0.310012	0	0	0.695731	0	4.278221	2.009959
18661	LOC10324!	1.163985	3.30907	2.684343	1.23273	0.217677	0.310012	0	0	0.695731	0	4.278221	2.009959
18664	LOC11962(	1.163985	3.30907	2.684343	1.23273	0.217677	0.310012	0	0	0.695731	0	4.278221	2.009959
18666	LOC11962:	1.163985	3.30907	2.684343	1.23273	0.217677	0.310012	0	0	0.695731	0	4.278221	2.009959
18669	LOC11962:	1.163985	3.30907	2.684343	1.23273	0.217677	0.310012	0	0	0.695731	0	4.278221	2.009959
18750	GK2	1.139265	3.309049	2.702524	1.224429	0.22079	0.313035	0	0.547412	0	0	4.278221	2.009959
18751	GNRHR	1.139265	3.309049	2.702524	1.224429	0.22079	0.313035	0	0.547412	0	0	4.278221	2.009959
4709	NOS1	38.32845	3.308841	0.67815	4.87922	1.07E-06	6.01E-06	3.794605	2.189647	15.30609	95.98146	85.56443	27.13445
6647	ENTPD1	20.03977	3.307436	0.852587	3.879293	0.000105	0.000419	0.542086	1.642235	9.044508	46.87467	47.06043	15.07469
93	SMTNL2	733.0564	3.307322	0.134239	24.63764	4.99E-134	1.43E-131	125.222	142.3271	136.3633	1381.687	1244.962	1367.777
2194	LOC10324‹	47.85344	3.307203	0.433843	7.623034	2.48E-14	3.00E-13	5.96295	10.94824	9.740239	73.66019	81.28621	105.5228
7971	IQSEC3	12.75078	3.305193	0.966757	3.418847	0.000629	0.002097	0	1.642235	5.565851	17.85702	36.36488	15.07469
6909	ARHGEF15	16.43993	3.305073	0.875649	3.774425	0.00016	0.000617	0.542086	1.094824	7.653045	40.17829	32.08666	17.08465
8566	NLRP3	9.261131	3.304969	1.020655	3.238086	0.001203	0.003735	0.542086	0.547412	4.174388	17.85702	21.39111	11.05477
10394	LOC11962!	5.456394	3.3047	1.194121	2.767476	0.005649	0.014449	0.542086	1.094824	1.391463	6.696381	14.97377	8.039836
7890	GJB1	12.73952	3.302945	0.95886	3.444657	0.000572	0.001927	1.084173	0.547412	5.565851	13.39276	42.78221	13.06473
15621	LOC11962!	2.358976	3.301679	1.911669	1.727118	0.084146	0.143097	0	0	1.391463	4.464254	4.278221	4.019918
10930	CYP4X1	8.488488	3.300913	1.248227	2.64448	0.008182	0.0199	0	0	4.87012	15.62489	21.39111	9.044815
7076	LOC11962(	14.87449	3.300327	0.891112	3.703606	0.000213	0.000799	0	2.189647	6.261582	22.32127	36.36488	22.10955
10613	MMD2	5.545065	3.299875	1.214057	2.718056	0.006567	0.016449	1.084173	0.547412	1.391463	13.39276	12.83466	4.019918
18901	CRYBA1	1.175406	3.29966	2.739253	1.204584	0.228364	0.321203	0.542086	0	0	2.232127	4.278221	0
18892	LOC11962(	1.176293	3.299628	2.738626	1.204848	0.228262	0.321094	0	0.547412	0	2.232127	4.278221	0
18893	LOC11962!	1.176293	3.299628	2.738626	1.204848	0.228262	0.321094	0	0.547412	0	2.232127	4.278221	0
18894	LOC11962.	1.176293	3.299628	2.738626	1.204848	0.228262	0.321094	0	0.547412	0	2.232127	4.278221	0
18896	LOC10324.	1.176293	3.299628	2.738626	1.204848	0.228262	0.321094	0	0.547412	0	2.232127	4.278221	0
18897	LUZP4	1.176293	3.299628	2.738626	1.204848	0.228262	0.321094	0	0.547412	0	2.232127	4.278221	0
18898	LOC11962:	1.176293	3.299628	2.738626	1.204848	0.228262	0.321094	0	0.547412	0	2.232127	4.278221	0
18899	LOC10324(	1.176293	3.299628	2.738626	1.204848	0.228262	0.321094	0	0.547412	0	2.232127	4.278221	0
11691	LYG2	4.329437	3.299042	1.333943	2.47315	0.013393	0.030455	0.542086	1.094824	0.695731	8.928508	10.69555	4.019918
18840	LOC11962(	1.201013	3.298722	2.721445	1.212122	0.225466	0.317985	0	0	0.695731	2.232127	4.278221	0
18841	LOC11962(	1.201013	3.298722	2.721445	1.212122	0.225466	0.317985	0	0	0.695731	2.232127	4.278221	0
18842	LOC10324:	1.201013	3.298722	2.721445	1.212122	0.225466	0.317985	0	0	0.695731	2.232127	4.278221	0
18845	LOC10322:	1.201013	3.298722	2.721445	1.212122	0.225466	0.317985	0	0	0.695731	2.232127	4.278221	0
18846	LOC11962.	1.201013	3.298722	2.721445	1.212122	0.225466	0.317985	0	0	0.695731	2.232127	4.278221	0
18848	PRND	1.201013	3.298722	2.721445	1.212122	0.225466	0.317985	0	0	0.695731	2.232127	4.278221	0
18850	DEFA5	1.201013	3.298722	2.721445	1.212122	0.225466	0.317985	0	0	0.695731	2.232127	4.278221	0
8662	LOC11962:	16.87474	3.298275	1.026458	3.213258	0.001312	0.004028	0	0.547412	9.044508	20.08914	53.47777	18.08963
4436	LOC10324:	32.84551	3.298195	0.652683	5.053285	4.34E-07	2.60E-06	1.084173	4.926706	12.52316	46.87467	83.42532	48.23901
9768	LOC10322!	6.641017	3.296918	1.132248	2.911833	0.003593	0.009779	1.084173	0.547412	2.087194	6.696381	21.39111	8.039836
9443	LOC10323:	11.99233	3.29577	1.099006	2.998864	0.00271	0.007629	0	0.547412	6.261582	35.71403	21.39111	8.039836
19636	LOC11961!	1.159903	3.295094	3.051946	1.07967	0.280289	0.379481	0.542086	0	0	0	6.417332	0
19632	LOC11962!	1.160791	3.295069	3.05117	1.079936	0.280171	0.379398	0	0.547412	0	0	6.417332	0
12856	LOC11962!	4.336347	3.295061	1.479153	2.227668	0.025903	0.053564	0	1.642235	0.695731	6.696381	14.97377	2.009959
19581	APOA4	1.185511	3.294375	3.029927	1.087278	0.276914	0.375811	0	0	0.695731	0	6.417332	0
19582	IL17C	1.185511	3.294375	3.029927	1.087278	0.276914	0.375811	0	0	0.695731	0	6.417332	0
19583	LOC10322‹	1.185511	3.294375	3.029927	1.087278	0.276914	0.375811	0	0	0.695731	0	6.417332	0

19584	HMX1	1.185511	3.294375	3.029927	1.087278	0.276914	0.375811	0	0	0.695731	0	6.417332	0
19585	ADAM2	1.185511	3.294375	3.029927	1.087278	0.276914	0.375811	0	0	0.695731	0	6.417332	0
19587	LOC10323:	1.185511	3.294375	3.029927	1.087278	0.276914	0.375811	0	0	0.695731	0	6.417332	0
19588	LOC11962(	1.185511	3.294375	3.029927	1.087278	0.276914	0.375811	0	0	0.695731	0	6.417332	0
19589	CUNH16or	1.185511	3.294375	3.029927	1.087278	0.276914	0.375811	0	0	0.695731	0	6.417332	0
11141	VIPR1	6.915087	3.293911	1.269995	2.59364	0.009497	0.022661	0	1.094824	2.782925	8.928508	25.66933	3.014938
15791	LOC11962‹	2.396004	3.292675	1.933209	1.703218	0.088527	0.149012	0	0	1.391463	6.696381	4.278221	2.009959
15793	NKX6-2	2.396004	3.292675	1.933209	1.703218	0.088527	0.149012	0	0	1.391463	6.696381	4.278221	2.009959
15794	LOC10322:	2.396004	3.292675	1.933209	1.703218	0.088527	0.149012	0	0	1.391463	6.696381	4.278221	2.009959
8804	LOC11962:	9.111965	3.291307	1.037341	3.17283	0.00151	0.004558	0.542086	1.094824	3.478657	17.85702	25.66933	6.029877
14672	GHRH	4.095847	3.291055	1.737938	1.893655	0.058271	0.105585	1.084173	1.094824	0	0	21.39111	1.004979
16178	LOC10321(	2.159849	3.290756	2.018383	1.630392	0.103019	0.169289	0.542086	0	0.695731	6.696381	0	5.024897
8498	CACNG2	19.33233	3.29039	1.010428	3.256433	0.001128	0.003529	0	0.547412	10.43597	53.57105	36.36488	15.07469
14032	LOC10324!	3.575492	3.290355	1.639247	2.007235	0.044725	0.084735	0	0	2.087194	8.928508	6.417332	4.019918
15315	CTXN2	3.465924	3.290239	1.851645	1.776928	0.07558	0.131198	0.542086	0	1.391463	17.85702	0	1.004979
3302	ZSCAN1	36.34999	3.290048	0.544935	6.037501	1.57E-09	1.26E-08	2.710432	10.40082	6.957314	73.66019	79.14709	45.22408
237	SERPINA5	432.298	3.289336	0.174974	18.79896	7.70E-79	8.64E-77	86.19173	70.06871	85.57496	716.5128	737.9932	897.4467
9357	LOC11962:	6.495309	3.288901	1.090271	3.01659	0.002556	0.007263	1.084173	1.094824	1.391463	13.39276	14.97377	7.034856
6425	LOC10322ℓ	34.73634	3.28889	0.828355	3.970388	7.18E-05	0.000297	0.542086	1.094824	18.08902	75.89232	70.59065	42.20914
7430	LOC11961!	9.735607	3.288604	0.916808	3.587014	0.000334	0.001197	1.626259	1.094824	2.782925	11.16063	25.66933	16.07967
8348	KLK10	19.2798	3.28848	0.997426	3.296968	0.000977	0.003112	0	0.547412	10.43597	49.10679	38.50399	17.08465
13278	NFAM1	39.21231	3.287858	1.527886	2.1519	0.031405	0.062879	0.542086	0.547412	20.87194	80.35657	104.8164	28.13943
10508	LOC10324:	7.722095	3.287724	1.198567	2.743045	0.006087	0.015401	2.168345	0	2.087194	13.39276	25.66933	3.014938
13890	LOC11962:	6.008948	3.287531	1.617734	2.032183	0.042135	0.080639	0	0	3.478657	0	23.53022	9.044815
7070	LOC10323‹	12.38416	3.287272	0.887346	3.704611	0.000212	0.000796	0.542086	1.642235	4.87012	20.08914	32.08666	15.07469
26600	LOC11962(	0.524015	3.287001	3.974917	0.826936	0.408274	NA	0	0	0	0	2.139111	1.004979
26630	ANGPTL3	0.524015	3.287001	3.974917	0.826936	0.408274	NA	0	0	0	0	2.139111	1.004979
26837	LOC10323!	0.524015	3.287001	3.974917	0.826936	0.408274	NA	0	0	0	0	2.139111	1.004979
26880	LOC10323ℓ	0.524015	3.287001	3.974917	0.826936	0.408274	NA	0	0	0	0	2.139111	1.004979
26887	LOC11962(	0.524015	3.287001	3.974917	0.826936	0.408274	NA	0	0	0	0	2.139111	1.004979
26912	LOC10322:	0.524015	3.287001	3.974917	0.826936	0.408274	NA	0	0	0	0	2.139111	1.004979
26938	LOC10322:	0.524015	3.287001	3.974917	0.826936	0.408274	NA	0	0	0	0	2.139111	1.004979
26967	LOC11962‹	0.524015	3.287001	3.974917	0.826936	0.408274	NA	0	0	0	0	2.139111	1.004979
26969	LOC11962‹	0.524015	3.287001	3.974917	0.826936	0.408274	NA	0	0	0	0	2.139111	1.004979
27012	LOC11962‹	0.524015	3.287001	3.974917	0.826936	0.408274	NA	0	0	0	0	2.139111	1.004979
27020	HSD17B3	0.524015	3.287001	3.974917	0.826936	0.408274	NA	0	0	0	0	2.139111	1.004979
27053	LOC11962‹	0.524015	3.287001	3.974917	0.826936	0.408274	NA	0	0	0	0	2.139111	1.004979
27095	LOC10323!	0.524015	3.287001	3.974917	0.826936	0.408274	NA	0	0	0	0	2.139111	1.004979
27233	UNC93A	0.524015	3.287001	3.974917	0.826936	0.408274	NA	0	0	0	0	2.139111	1.004979
27403	LOC11962!	0.524015	3.287001	3.974917	0.826936	0.408274	NA	0	0	0	0	2.139111	1.004979
27411	LOC11962!	0.524015	3.287001	3.974917	0.826936	0.408274	NA	0	0	0	0	2.139111	1.004979
27451	SPINK8	0.524015	3.287001	3.974917	0.826936	0.408274	NA	0	0	0	0	2.139111	1.004979
27499	LOC11962(	0.524015	3.287001	3.974917	0.826936	0.408274	NA	0	0	0	0	2.139111	1.004979
27526	LOC11962(	0.524015	3.287001	3.974917	0.826936	0.408274	NA	0	0	0	0	2.139111	1.004979
27549	CLDN25	0.524015	3.287001	3.974917	0.826936	0.408274	NA	0	0	0	0	2.139111	1.004979
27593	LOC10324:	0.524015	3.287001	3.974917	0.826936	0.408274	NA	0	0	0	0	2.139111	1.004979
27598	HBB	0.524015	3.287001	3.974917	0.826936	0.408274	NA	0	0	0	0	2.139111	1.004979
27622	LOC11962(	0.524015	3.287001	3.974917	0.826936	0.408274	NA	0	0	0	0	2.139111	1.004979
27649	POM121L2	0.524015	3.287001	3.974917	0.826936	0.408274	NA	0	0	0	0	2.139111	1.004979
27668	LOC10322:	0.524015	3.287001	3.974917	0.826936	0.408274	NA	0	0	0	0	2.139111	1.004979
27703	LOC11962:	0.524015	3.287001	3.974917	0.826936	0.408274	NA	0	0	0	0	2.139111	1.004979
27715	LOC11962:	0.524015	3.287001	3.974917	0.826936	0.408274	NA	0	0	0	0	2.139111	1.004979
27918	LOC11962ℓ	0.524015	3.287001	3.974917	0.826936	0.408274	NA	0	0	0	0	2.139111	1.004979
28060	LOC11961ℓ	0.524015	3.287001	3.974917	0.826936	0.408274	NA	0	0	0	0	2.139111	1.004979
28062	LOC11961ℓ	0.524015	3.287001	3.974917	0.826936	0.408274	NA	0	0	0	0	2.139111	1.004979
28075	LOC11961ℓ	0.524015	3.287001	3.974917	0.826936	0.408274	NA	0	0	0	0	2.139111	1.004979
28085	WFDC12	0.524015	3.287001	3.974917	0.826936	0.408274	NA	0	0	0	0	2.139111	1.004979
28179	LOC11961ℓ	0.524015	3.287001	3.974917	0.826936	0.408274	NA	0	0	0	0	2.139111	1.004979
28213	LOC11961ℓ	0.524015	3.287001	3.974917	0.826936	0.408274	NA	0	0	0	0	2.139111	1.004979
28244	RD3L	0.524015	3.287001	3.974917	0.826936	0.408274	NA	0	0	0	0	2.139111	1.004979
28255	LOC11961!	0.524015	3.287001	3.974917	0.826936	0.408274	NA	0	0	0	0	2.139111	1.004979
28272	LOC10321!	0.524015	3.287001	3.974917	0.826936	0.408274	NA	0	0	0	0	2.139111	1.004979
28313	LOC11961!	0.524015	3.287001	3.974917	0.826936	0.408274	NA	0	0	0	0	2.139111	1.004979
28321	LOC11961!	0.524015	3.287001	3.974917	0.826936	0.408274	NA	0	0	0	0	2.139111	1.004979
28370	LOC11961!	0.524015	3.287001	3.974917	0.826936	0.408274	NA	0	0	0	0	2.139111	1.004979
28398	LOC11961!	0.524015	3.287001	3.974917	0.826936	0.408274	NA	0	0	0	0	2.139111	1.004979
28422	LOC10323(	0.524015	3.287001	3.974917	0.826936	0.408274	NA	0	0	0	0	2.139111	1.004979
28438	LOC10324:	0.524015	3.287001	3.974917	0.826936	0.408274	NA	0	0	0	0	2.139111	1.004979
28549	LOC10324:	0.524015	3.287001	3.974917	0.826936	0.408274	NA	0	0	0	0	2.139111	1.004979
28551	LOC10323:	0.524015	3.287001	3.974917	0.826936	0.408274	NA	0	0	0	0	2.139111	1.004979
28558	LOC11962(	0.524015	3.287001	3.974917	0.826936	0.408274	NA	0	0	0	0	2.139111	1.004979
28575	LOC10323:	0.524015	3.287001	3.974917	0.826936	0.408274	NA	0	0	0	0	2.139111	1.004979
28603	LOC11962(	0.524015	3.287001	3.974917	0.826936	0.408274	NA	0	0	0	0	2.139111	1.004979
28613	LOC11962(	0.524015	3.287001	3.974917	0.826936	0.408274	NA	0	0	0	0	2.139111	1.004979

28723	LOC11962(	0.524015	3.287001	3.974917	0.826936	0.408274	NA	0	0	0	0	2.139111	1.004979
28771	LOC11962:	0.524015	3.287001	3.974917	0.826936	0.408274	NA	0	0	0	0	2.139111	1.004979
28791	LOC10323:	0.524015	3.287001	3.974917	0.826936	0.408274	NA	0	0	0	0	2.139111	1.004979
28800	LOC11962:	0.524015	3.287001	3.974917	0.826936	0.408274	NA	0	0	0	0	2.139111	1.004979
28803	LOC10322{	0.524015	3.287001	3.974917	0.826936	0.408274	NA	0	0	0	0	2.139111	1.004979
28819	LOC11962:	0.524015	3.287001	3.974917	0.826936	0.408274	NA	0	0	0	0	2.139111	1.004979
28829	LOC10321{	0.524015	3.287001	3.974917	0.826936	0.408274	NA	0	0	0	0	2.139111	1.004979
28912	LOC10322;	0.524015	3.287001	3.974917	0.826936	0.408274	NA	0	0	0	0	2.139111	1.004979
28937	LOC10323⁄	0.524015	3.287001	3.974917	0.826936	0.408274	NA	0	0	0	0	2.139111	1.004979
28971	LOC10324;	0.524015	3.287001	3.974917	0.826936	0.408274	NA	0	0	0	0	2.139111	1.004979
29077	SCP2D1	0.524015	3.287001	3.974917	0.826936	0.408274	NA	0	0	0	0	2.139111	1.004979
29098	LOC11962:	0.524015	3.287001	3.974917	0.826936	0.408274	NA	0	0	0	0	2.139111	1.004979
29175	LOC11962:	0.524015	3.287001	3.974917	0.826936	0.408274	NA	0	0	0	0	2.139111	1.004979
29237	LOC11962:	0.524015	3.287001	3.974917	0.826936	0.408274	NA	0	0	0	0	2.139111	1.004979
29415	LOC10324;	0.524015	3.287001	3.974917	0.826936	0.408274	NA	0	0	0	0	2.139111	1.004979
26594	LOC11962(	0.539518	3.286943	3.931957	0.835956	0.40318	NA	0	0	0	2.232127	0	1.004979
26606	LOC10322!	0.539518	3.286943	3.931957	0.835956	0.40318	NA	0	0	0	2.232127	0	1.004979
26662	LOC11961{	0.539518	3.286943	3.931957	0.835956	0.40318	NA	0	0	0	2.232127	0	1.004979
26680	IL17B	0.539518	3.286943	3.931957	0.835956	0.40318	NA	0	0	0	2.232127	0	1.004979
26780	IL2	0.539518	3.286943	3.931957	0.835956	0.40318	NA	0	0	0	2.232127	0	1.004979
26847	LOC11962:	0.539518	3.286943	3.931957	0.835956	0.40318	NA	0	0	0	2.232127	0	1.004979
26870	LOC11962:	0.539518	3.286943	3.931957	0.835956	0.40318	NA	0	0	0	2.232127	0	1.004979
26941	LOC10322:	0.539518	3.286943	3.931957	0.835956	0.40318	NA	0	0	0	2.232127	0	1.004979
26981	LOC10321!	0.539518	3.286943	3.931957	0.835956	0.40318	NA	0	0	0	2.232127	0	1.004979
26992	LOC11962⁄	0.539518	3.286943	3.931957	0.835956	0.40318	NA	0	0	0	2.232127	0	1.004979
27018	LOC11962⁄	0.539518	3.286943	3.931957	0.835956	0.40318	NA	0	0	0	2.232127	0	1.004979
27058	LOC10323!	0.539518	3.286943	3.931957	0.835956	0.40318	NA	0	0	0	2.232127	0	1.004979
27070	LOC10323!	0.539518	3.286943	3.931957	0.835956	0.40318	NA	0	0	0	2.232127	0	1.004979
27121	LOC11962⁄	0.539518	3.286943	3.931957	0.835956	0.40318	NA	0	0	0	2.232127	0	1.004979
27208	LOC11962!	0.539518	3.286943	3.931957	0.835956	0.40318	NA	0	0	0	2.232127	0	1.004979
27318	LOC11962!	0.539518	3.286943	3.931957	0.835956	0.40318	NA	0	0	0	2.232127	0	1.004979
27339	SPACA1	0.539518	3.286943	3.931957	0.835956	0.40318	NA	0	0	0	2.232127	0	1.004979
27357	LOC10324:	0.539518	3.286943	3.931957	0.835956	0.40318	NA	0	0	0	2.232127	0	1.004979
27416	LOC11962!	0.539518	3.286943	3.931957	0.835956	0.40318	NA	0	0	0	2.232127	0	1.004979
27420	LOC10322{	0.539518	3.286943	3.931957	0.835956	0.40318	NA	0	0	0	2.232127	0	1.004979
27529	LOC11962(	0.539518	3.286943	3.931957	0.835956	0.40318	NA	0	0	0	2.232127	0	1.004979
27538	LOC10324{	0.539518	3.286943	3.931957	0.835956	0.40318	NA	0	0	0	2.232127	0	1.004979
27599	LOC11962(	0.539518	3.286943	3.931957	0.835956	0.40318	NA	0	0	0	2.232127	0	1.004979
27636	TRNAA-CG	0.539518	3.286943	3.931957	0.835956	0.40318	NA	0	0	0	2.232127	0	1.004979
27670	LOC11962(	0.539518	3.286943	3.931957	0.835956	0.40318	NA	0	0	0	2.232127	0	1.004979
27743	LOC11962;	0.539518	3.286943	3.931957	0.835956	0.40318	NA	0	0	0	2.232127	0	1.004979
27867	LOC11962;	0.539518	3.286943	3.931957	0.835956	0.40318	NA	0	0	0	2.232127	0	1.004979
27929	LOC11962{	0.539518	3.286943	3.931957	0.835956	0.40318	NA	0	0	0	2.232127	0	1.004979
27999	LOC11961{	0.539518	3.286943	3.931957	0.835956	0.40318	NA	0	0	0	2.232127	0	1.004979
28061	LOC11961{	0.539518	3.286943	3.931957	0.835956	0.40318	NA	0	0	0	2.232127	0	1.004979
28080	LOC11961{	0.539518	3.286943	3.931957	0.835956	0.40318	NA	0	0	0	2.232127	0	1.004979
28109	CST9L	0.539518	3.286943	3.931957	0.835956	0.40318	NA	0	0	0	2.232127	0	1.004979
28141	LOC11961{	0.539518	3.286943	3.931957	0.835956	0.40318	NA	0	0	0	2.232127	0	1.004979
28198	LOC11961{	0.539518	3.286943	3.931957	0.835956	0.40318	NA	0	0	0	2.232127	0	1.004979
28207	LOC11961!	0.539518	3.286943	3.931957	0.835956	0.40318	NA	0	0	0	2.232127	0	1.004979
28214	LOC11961{	0.539518	3.286943	3.931957	0.835956	0.40318	NA	0	0	0	2.232127	0	1.004979
28282	LOC10322!	0.539518	3.286943	3.931957	0.835956	0.40318	NA	0	0	0	2.232127	0	1.004979
28302	LOC11961!	0.539518	3.286943	3.931957	0.835956	0.40318	NA	0	0	0	2.232127	0	1.004979
28308	SLC6A19	0.539518	3.286943	3.931957	0.835956	0.40318	NA	0	0	0	2.232127	0	1.004979
28446	LOC11962(	0.539518	3.286943	3.931957	0.835956	0.40318	NA	0	0	0	2.232127	0	1.004979
28487	LOC11962(	0.539518	3.286943	3.931957	0.835956	0.40318	NA	0	0	0	2.232127	0	1.004979
28567	LOC10323:	0.539518	3.286943	3.931957	0.835956	0.40318	NA	0	0	0	2.232127	0	1.004979
28638	LOC11962(	0.539518	3.286943	3.931957	0.835956	0.40318	NA	0	0	0	2.232127	0	1.004979
28651	LOC11962(	0.539518	3.286943	3.931957	0.835956	0.40318	NA	0	0	0	2.232127	0	1.004979
28737	LOC10324;	0.539518	3.286943	3.931957	0.835956	0.40318	NA	0	0	0	2.232127	0	1.004979
29101	LOC11962:	0.539518	3.286943	3.931957	0.835956	0.40318	NA	0	0	0	2.232127	0	1.004979
29105	LOC11962:	0.539518	3.286943	3.931957	0.835956	0.40318	NA	0	0	0	2.232127	0	1.004979
29198	LOC11962:	0.539518	3.286943	3.931957	0.835956	0.40318	NA	0	0	0	2.232127	0	1.004979
29209	LOC10322!	0.539518	3.286943	3.931957	0.835956	0.40318	NA	0	0	0	2.232127	0	1.004979
29320	LOC11962:	0.539518	3.286943	3.931957	0.835956	0.40318	NA	0	0	0	2.232127	0	1.004979
29585	LOC10322(	0.539518	3.286943	3.931957	0.835956	0.40318	NA	0	0	0	2.232127	0	1.004979
8418	SLC30A10	10.26045	3.286943	1.003054	3.276934	0.001049	0.003314	0.542086	1.094824	4.174388	15.62489	32.08666	8.039836
5955	LOC10322!	16.86658	3.286631	0.785511	4.184068	2.86E-05	0.000128	2.168345	1.094824	6.261582	44.64254	29.94755	17.08465
15467	KLK12	2.145234	3.286395	1.877765	1.750163	0.08009	0.137661	0	0.547412	0.695731	4.464254	2.139111	5.024897
14373	LOC10321;	3.61252	3.285627	1.686024	1.948742	0.051326	0.094935	0	0	2.087194	11.16063	6.417332	2.009959
4989	KCNC1	27.21748	3.285425	0.699695	4.695511	2.66E-06	1.42E-05	2.168345	2.737059	10.43597	66.96381	59.8951	21.10457
15817	ZNRF4	2.380501	3.284534	1.93544	1.697048	0.089688	0.150746	0	0	1.391463	4.464254	6.417332	2.009959
5678	SOX5	42.17455	3.283594	0.756996	4.337665	1.44E-05	6.74E-05	1.084173	3.284471	19.48048	78.12444	121.9293	29.1444
16860	GAST	2.433032	3.282807	2.169702	1.513022	0.130274	0.205417	0	0	1.391463	8.928508	4.278221	0

191	LRRC31	427.2524	3.281601	0.160071	20.50087	2.11E-93	2.94E-91	71.5554	82.65918	85.57496	772.3159	744.4105	806.9985
7700	NKX2-3	12.44873	3.280044	0.938883	3.493561	0.000477	0.001646	0.542086	1.642235	4.87012	20.08914	38.50399	9.044815
4528	TBC1D10C	27.78335	3.279883	0.65633	4.997307	5.81E-07	3.41E-06	4.336691	1.642235	9.740239	49.10679	72.72976	29.1444
12080	RASSF10	7.240543	3.278458	1.371303	2.390761	0.016813	0.037002	0	0	4.174388	24.5534	10.69555	4.019918
10197	ISLR2	52.18009	3.278343	1.163864	2.816774	0.004851	0.012647	1.626259	2.737059	25.04633	102.6778	134.764	46.22906
7561	LOC10322(	9.800813	3.278235	0.926714	3.537481	0.000404	0.00142	1.626259	0.547412	3.478657	20.08914	14.97377	18.08963
14218	LOC119628	3.544487	3.277793	1.660647	1.973805	0.048404	0.090506	0	0	2.087194	4.464254	10.69555	4.019918
9551	FXYD3	7.797927	3.277434	1.102737	2.972089	0.002958	0.008233	0	1.094824	3.478657	11.16063	14.97377	16.07967
5094	LOC11962:	40.47388	3.277315	0.707552	4.631908	3.62E-06	1.89E-05	1.626259	3.284471	18.08902	89.28508	98.39909	32.15934
14427	SPATA16	3.507459	3.276921	1.689953	1.939061	0.052494	0.096732	0	0	2.087194	2.232127	10.69555	6.029877
10885	NLRP2	9.304644	3.276572	1.235084	2.652915	0.00798	0.01949	0	1.094824	4.174388	35.71403	12.83466	2.009959
13001	CABP2	4.723975	3.27656	1.487124	2.203286	0.027575	0.056381	0	0	2.782925	6.696381	12.83466	6.029877
16697	RAX2	2.306445	3.275768	2.122884	1.543075	0.122813	0.195543	0	0	1.391463	0	6.417332	6.029877
7442	LOC119624	9.395907	3.275449	0.913863	3.584178	0.000338	0.001208	2.710432	1.094824	1.391463	17.85702	19.252	14.06971
14422	SLC14A2	3.581515	3.274298	1.688033	1.939712	0.052415	0.096619	0	0	2.087194	6.696381	10.69555	2.009959
16045	LOC11962:	2.364999	3.274276	1.975348	1.657569	0.097405	0.161398	0	0	1.391463	2.232127	8.556443	2.009959
139	HNMT	610.2359	3.272883	0.143644	22.78465	6.51E-115	1.25E-112	111.6698	118.241	112.7085	1216.509	1063.138	1039.149
18336	LOC11962:	2.042576	3.272799	2.562182	1.277348	0.201479	0.292128	0	1.094824	0	11.16063	0	0
8389	LOC10322!	9.806206	3.272711	0.995988	3.285895	0.001017	0.003222	1.626259	1.094824	2.782925	8.928508	36.36488	8.039836
6783	ZNF814	13.82837	3.271111	0.855283	3.824594	0.000131	0.000513	4.336691	0.547412	2.782925	29.01765	34.22577	12.05975
13609	TBC1D21	3.383275	3.271088	1.566574	2.088052	0.036793	0.071875	0	0.547412	1.391463	8.928508	6.417332	3.014938
10906	GRIN2B	48.74234	3.270145	1.234145	2.649726	0.008056	0.019637	1.084173	2.189647	24.3506	91.51721	124.0684	49.24399
694	PCSK6	157.3522	3.270076	0.254444	12.85185	8.40E-38	3.22E-36	28.18849	36.12918	24.3506	267.8552	260.9715	326.6183
13248	LOC11961!	4.708472	3.269869	1.51522	2.158016	0.030927	0.062061	0	0	2.782925	4.464254	14.97377	6.029877
10104	LOC10322:	7.262718	3.268524	1.151879	2.837557	0.004546	0.011961	2.710432	0.547412	0.695731	8.928508	25.66933	5.024897
9898	GNA15	10.22724	3.268214	1.132777	2.885136	0.003912	0.010508	1.626259	0	4.174388	29.01765	23.53022	3.014938
14143	DEFB116	3.420303	3.266013	1.643871	1.986782	0.046947	0.088247	0	0.547412	1.391463	11.16063	6.417332	1.004979
1715	ADAMTS2	62.39367	3.265811	0.375861	8.688872	3.66E-18	5.67E-17	8.673382	12.04306	14.61036	122.767	119.7902	96.47803
3002	B3GNT3	40.07785	3.265682	0.509034	6.415453	1.40E-10	1.24E-09	4.336691	6.02153	12.52316	69.19594	94.12087	54.26889
13850	LOC11962!	3.315242	3.265462	1.600824	2.039863	0.041364	0.079392	0	0.547412	1.391463	2.232127	10.69555	5.024897
10755	GJA9	6.788076	3.265451	1.219237	2.678275	0.0074	0.018291	0	1.094824	2.782925	17.85702	14.97377	4.019918
15423	GABRR3	2.166759	3.264544	1.858172	1.756857	0.078942	0.136074	0	0.547412	0.695731	4.464254	4.278221	3.014938
12115	BPIFB2	4.333519	3.26369	1.369751	2.382689	0.017187	0.037714	0	1.094824	1.391463	8.928508	8.556443	6.029877
6819	KHDC1L	10.58125	3.262819	0.85639	3.80997	0.000139	0.000542	1.084173	2.189647	2.782925	20.08914	19.252	18.08963
17665	LOC10324:	36.76157	3.262287	2.342424	1.392697	0.163711	0.246378	0	0	20.87194	64.73168	104.8164	30.14938
12588	TRIM15	5.872457	3.262122	1.427014	2.285978	0.022256	0.047002	0	0	3.478657	4.464254	19.252	8.039836
8526	LOC10322(	18.91423	3.26093	1.004107	3.247591	0.001164	0.003629	0	0.547412	10.43597	26.78552	55.61688	20.09959
11205	LOC10321:	6.918282	3.260591	1.262888	2.581853	0.009827	0.023316	0	0.547412	3.478657	8.928508	23.53022	5.024897
12147	HOXC11	7.147526	3.260494	1.372195	2.376115	0.017496	0.038292	0	0	4.174388	11.16063	23.53022	4.019918
3987	COL23A1	38.77312	3.260138	0.605644	5.382927	7.33E-08	4.89E-07	3.794605	7.663765	10.43597	66.96381	117.6511	26.12947
12982	LOC10322:	4.027556	3.259563	1.476898	2.207034	0.027312	0.05593	1.626259	0	0.695731	2.232127	8.556443	11.05477
12507	LOC10322:	5.447377	3.259321	1.41702	2.300124	0.021441	0.045576	0	1.642235	1.391463	20.08914	8.556443	1.004979
7897	SEZ6	13.45559	3.259031	0.947002	3.441418	0.000579	0.001948	0	2.189647	5.565851	24.5534	36.36488	12.05975
11821	LYZL4	4.369659	3.258882	1.333739	2.443418	0.014549	0.032717	0.542086	0.547412	1.391463	11.16063	8.556443	4.019918
10434	LOC10323!	6.302039	3.258146	1.18085	2.759154	0.005795	0.014766	0	2.189647	1.391463	15.62489	8.556443	10.04979
14644	LOC11962(	2.84382	3.257831	1.716535	1.897911	0.057708	0.104764	0.542086	1.094824	0	2.232127	2.139111	11.05477
7162	NR1I2	12.02444	3.257606	0.886141	3.676171	0.000237	0.000879	0.542086	2.189647	4.174388	20.08914	32.08666	13.06473
15615	LRRC10	2.151257	3.256082	1.88471	1.72763	0.084055	0.143097	0	0.547412	0.695731	2.232127	6.417332	3.014938
15637	LOC10324:	2.151257	3.256082	1.88471	1.72763	0.084055	0.143097	0	0.547412	0.695731	2.232127	6.417332	3.014938
865	LOC10324:	109.248	3.255538	0.278181	11.70296	1.23E-31	3.78E-30	18.97302	20.25424	22.95914	200.8914	207.4937	184.9162
15029	LOC11961!	2.933379	3.255226	1.780936	1.827818	0.067577	0.119542	0.542086	1.094824	0	8.928508	0	7.034856
4898	KRT9	44.75581	3.255095	0.686216	4.743545	2.10E-06	1.14E-05	0.542086	7.116354	18.08902	75.89232	117.6511	49.24399
5853	LOC10322:	25.88599	3.255068	0.766996	4.243917	2.20E-05	9.98E-05	0.542086	3.284471	11.1317	44.64254	70.59065	25.12449
884	TM4SF19	120.9089	3.255024	0.281209	11.57512	5.51E-31	1.66E-29	21.68345	19.15941	28.52499	238.8376	186.1026	231.1453
8382	LOC10324:	20.07177	3.254372	0.989534	3.288791	0.001006	0.003191	0	0.547412	11.1317	46.87467	42.78221	19.09461
13325	WFDC1	3.168646	3.253907	1.51833	2.143083	0.032106	0.064056	0.542086	0.547412	0.695731	8.928508	4.278221	4.019918
17605	TRNAV-AA	2.025299	3.253744	2.322091	1.401213	0.16115	0.24309	1.084173	0	0	8.928508	2.139111	0
6499	KCNH8	15.54275	3.25282	0.825563	3.940122	8.14E-05	0.000333	2.168345	4.379295	2.087194	20.08914	53.47777	11.05477
7744	RAX	11.9968	3.252371	0.934573	3.480061	0.000501	0.001721	2.168345	0.547412	4.174388	13.39276	40.6431	11.05477
15806	LOC10322!	2.203787	3.250949	1.912617	1.699739	0.08918	0.149997	0	0.547412	0.695731	6.696381	4.278221	1.004979
11717	SYCP2L	5.394846	3.250634	1.317466	2.467338	0.013612	0.030885	0	1.642235	1.391463	15.62489	10.69555	3.014938
8452	IQANK1	9.847316	3.250593	0.994613	3.2682	0.001082	0.003404	1.084173	1.094824	3.478657	11.16063	34.22577	8.039836
2959	OTOGL	32.1456	3.249167	0.501399	6.480208	9.16E-11	8.23E-10	6.505036	5.474118	6.261582	55.80317	70.59065	48.23901
14370	GSTA3	3.373795	3.248893	1.667103	1.948825	0.051316	0.09493	0	0.547412	1.391463	4.464254	12.83466	1.004979
11380	ARMC3	5.289785	3.247867	1.277736	2.541892	0.011025	0.025754	0	1.642235	1.391463	6.696381	14.97377	7.034856
7211	TNR	15.85301	3.246779	0.886292	3.663329	0.000249	0.000918	1.084173	0.547412	7.653045	31.24978	38.50399	16.07967
12642	ENPP6	4.324039	3.245839	1.428539	2.27214	0.023078	0.048531	0	1.094824	1.391463	4.464254	14.97377	4.019918
6389	LOC11962:	20.23472	3.245799	0.81486	3.98326	6.80E-05	0.000283	1.626259	1.094824	9.044508	37.94616	55.61688	16.07967
9381	NMBR	7.734604	3.245632	1.077477	3.012252	0.002593	0.007349	0.542086	0.547412	3.478657	20.08914	10.69555	11.05477
10743	LOC119618	6.400981	3.245628	1.210445	2.681351	0.007333	0.018145	1.626259	0.547412	1.391463	17.85702	14.97377	2.009959
8553	LOC119624	9.338607	3.245578	1.001718	3.240013	0.001195	0.003715	3.252518	0.547412	1.391463	24.5534	19.252	7.034856
13643	MRLN	3.205674	3.245278	1.559696	2.080712	0.03746	0.072996	0.542086	0.547412	0.695731	11.16063	4.278221	2.009959
15831	LOC11962:	2.188285	3.242467	1.914761	1.693405	0.090378	0.151763	0	0.547412	0.695731	4.464254	6.417332	1.004979
15832	STAP1	2.188285	3.242467	1.914761	1.693405	0.090378	0.151763	0	0.547412	0.695731	4.464254	6.417332	1.004979

15008	LOC11962	2.955792	3.242393	1.769728	1.832141	0.06693	0.118563	0	1.642235	0	8.928508	2.139111	5.024897
14432	SLC24A3	23.8212	3.242241	1.67269	1.93834	0.052582	0.096865	0	0.547412	13.2189	35.71403	66.31243	27.13445
8821	ADGRG4	16.00892	3.24219	1.023661	3.16725	0.001539	0.004638	0	1.642235	7.653045	37.94616	42.78221	6.029877
5122	LOC11962	17.60482	3.241315	0.701922	4.617771	3.88E-06	2.01E-05	3.794605	3.284471	2.782925	40.17829	38.50399	17.08465
14048	PTPN20	3.191059	3.241108	1.616391	2.005151	0.044947	0.085053	0	1.094824	0.695731	8.928508	6.417332	2.009959
14049	LOC10323	3.191059	3.241108	1.616391	2.005151	0.044947	0.085053	0	1.094824	0.695731	8.928508	6.417332	2.009959
7538	PRKG2	15.64707	3.240876	0.913807	3.546566	0.00039	0.001376	0	2.189647	6.957314	29.01765	40.6431	15.07469
10	ASAH1	11454.58	3.240573	0.082545	39.25833	0	0	2175.393	2242.746	2158.159	20591.37	19955.76	21604.04
14078	IQCM	2.902374	3.240334	1.621758	1.998038	0.045713	0.086316	0.542086	1.094824	0	4.464254	4.278221	7.034856
4973	MYOG	27.40446	3.239685	0.689072	4.70152	2.58E-06	1.38E-05	3.252518	1.642235	11.1317	78.12444	32.08666	38.18922
15950	TEX37	1.95213	3.238286	1.931943	1.67618	0.093703	0.156181	0.542086	0.547412	0	4.464254	2.139111	4.019918
7533	CUNH4orf	25.57247	3.238258	0.912653	3.548183	0.000388	0.001369	0	1.094824	13.91463	55.80317	53.47777	29.1444
9056	CDR1	10.09101	3.237261	1.046551	3.093265	0.00198	0.005812	0	1.094824	4.87012	17.85702	25.66933	11.05477
11389	LOC10321	5.33106	3.236664	1.274468	2.539619	0.011097	0.025904	1.084173	0.547412	1.391463	6.696381	19.252	3.014938
9369	SLC28A3	10.1857	3.236149	1.0737	3.014016	0.002578	0.007313	0.542086	0.547412	4.87012	22.32127	27.80844	5.024897
9198	LOC10323	7.115069	3.236075	1.059221	3.055146	0.00225	0.006502	2.168345	1.094824	0.695731	17.85702	12.83466	8.039836
10752	SLC18A1	11.80304	3.235926	1.208036	2.678666	0.007392	0.018276	0	0	6.957314	29.01765	27.80844	7.034856
6108	CMKLR1	35.51688	3.234788	0.785893	4.116067	3.85E-05	0.000168	0.542086	2.189647	18.08902	80.35657	72.72976	39.1942
13747	MYCT1	3.893005	3.234577	1.57086	2.059112	0.039483	0.076356	2.168345	0	0	4.464254	10.69555	6.029877
11812	TRIML1	6.983487	3.23432	1.322119	2.446316	0.014432	0.032483	0	0	4.174388	17.85702	12.83466	7.034856
14732	LOC11962	3.196194	3.2332	1.71672	1.883359	0.059652	0.107646	0.542086	0.547412	0.695731	6.696381	10.69555	0
6552	PNMA6E	15.03764	3.232506	0.824929	3.918529	8.91E-05	0.000361	1.084173	2.737059	4.87012	24.5534	44.92132	12.05975
9407	TMC2	9.628902	3.232107	1.075245	3.005926	0.002648	0.007483	0	2.737059	2.782925	31.24978	14.97377	6.029877
14044	LOC11961	3.986114	3.231327	1.610724	2.006134	0.044842	0.084889	0	2.189647	0	11.16063	8.556443	2.009959
8776	LOC11962	8.239457	3.231326	1.016153	3.179959	0.001473	0.004462	1.626259	1.642235	1.391463	8.928508	27.80844	8.039836
17489	HELT	2.011571	3.231325	2.287169	1.412806	0.157713	0.23947	0	1.094824	0	6.696381	4.278221	0
12852	LOC11962	3.978316	3.231178	1.450096	2.228251	0.025864	0.0535	1.084173	1.094824	0	13.39276	4.278221	4.019918
4663	BHLHE41	22.89701	3.230948	0.657818	4.911612	9.03E-07	5.15E-06	1.084173	6.02153	6.261582	33.4819	51.33866	39.1942
16791	TRIM61	2.157279	3.23048	2.11536	1.527154	0.126723	0.200624	0	0.547412	0.695731	0	10.69555	1.004979
16792	LOC10323	2.157279	3.23048	2.11536	1.527154	0.126723	0.200624	0	0.547412	0.695731	0	10.69555	1.004979
9301	SLC13A2	15.04077	3.229736	1.065231	3.031957	0.00243	0.006945	0	0.547412	8.348776	20.08914	49.19955	12.05975
12605	LOC10322	4.315188	3.229721	1.415395	2.281852	0.022498	0.04745	0	0.547412	2.087194	8.928508	4.278221	10.04979
11209	LOC11962	9.354509	3.229646	1.251169	2.581302	0.009843	0.023345	0	0	5.565851	17.85702	25.66933	7.034856
11682	PRDM14	5.168962	3.228909	1.304387	2.475422	0.013308	0.030283	1.626259	0.547412	0.695731	11.16063	14.97377	2.009959
4616	LOC11962	18.17383	3.228878	0.654026	4.936925	7.94E-07	4.57E-06	2.710432	4.379295	3.478657	33.4819	27.80844	37.18424
16591	LOC10323	1.990046	3.228803	2.07151	1.558671	0.119074	0.190802	0	1.094824	0	6.696381	2.139111	2.009959
4611	NTNG1	28.11488	3.228558	0.653781	4.938287	7.88E-07	4.54E-06	2.168345	7.663765	6.261582	51.33892	79.14709	22.10955
13899	HAND1	4.633992	3.228077	1.590409	2.029715	0.042386	0.08107	0	0	2.782925	17.85702	2.139111	5.024897
10539	LOC11962	9.131287	3.227054	1.179135	2.736798	0.006204	0.01565	0	0.547412	4.87012	13.39276	29.94755	6.029877
12659	LOC11962	4.976745	3.225431	1.422084	2.268102	0.023323	0.04898	1.626259	1.094824	0	11.16063	14.97377	1.004979
9141	NPAS4	15.0128	3.224859	1.050939	3.06855	0.002151	0.006256	0	0.547412	8.348776	40.17829	29.94755	11.05477
4970	LOC10322	17.1435	3.224291	0.685504	4.703533	2.56E-06	1.37E-05	3.794605	3.831883	2.087194	37.94616	32.08666	23.11453
8567	CALCR	10.8283	3.223282	0.995539	3.237724	0.001205	0.003739	1.084173	1.094824	4.174388	8.928508	40.6431	9.044815
7470	CATSPER1	10.53086	3.222626	0.90205	3.572556	0.000354	0.001258	1.084173	2.189647	2.782925	24.5534	23.53022	9.044815
7598	MYO7A	22.86688	3.221538	0.913423	3.526887	0.00042	0.001471	0	1.094824	12.52316	37.94616	53.47777	32.15934
12499	LOC10323	5.698938	3.22128	1.399333	2.302011	0.021335	0.045378	0	0	3.478657	6.696381	14.97377	9.044815
90	BMF	988.3651	3.221131	0.129091	24.95233	2.01E-137	5.95E-135	183.7673	190.4993	201.7621	1662.935	1713.428	1977.8
9075	DDX43	11.20204	3.220314	1.042204	3.089907	0.002002	0.005865	0	1.094824	5.565851	31.24978	19.252	10.04979
13564	LOC10324	4.624511	3.218545	1.53528	2.09639	0.036048	0.070652	0	0	2.782925	13.39276	8.556443	3.014938
16521	LOC11961	1.974543	3.21753	2.047633	1.571341	0.116103	0.18683	0	1.094824	0	4.464254	4.278221	2.009959
8248	LOC10323	8.017752	3.217332	0.965985	3.330624	0.000867	0.002793	1.084173	2.189647	1.391463	13.39276	14.97377	15.07469
9695	LOC10322	6.09574	3.217309	1.097222	2.932232	0.003365	0.009228	1.084173	1.094824	1.391463	13.39276	8.556443	11.05477
9852	LOC10324	6.315504	3.216504	1.112219	2.89197	0.003828	0.010331	1.084173	0.547412	2.087194	11.16063	14.97377	8.039836
14184	LOC11961	4.661539	3.215983	1.624539	1.979628	0.047745	0.089489	0	0	2.782925	15.62489	8.556443	1.004979
138	CDH16	4408.992	3.215673	0.141071	22.79476	5.17E-115	9.96E-113	925.8835	699.5923	947.5861	7549.053	7106.126	9225.712
11581	LOC10324	5.398041	3.215631	1.286822	2.498895	0.012458	0.028599	0	1.094824	2.087194	15.62489	8.556443	5.024897
14996	FAIM2	28.21621	3.215494	1.752324	1.834988	0.066508	0.117895	0	0.547412	16.00182	40.17829	83.42532	29.1444
9817	LOC10324	15.07843	3.213968	1.107854	2.901075	0.003719	0.010071	0	0	9.044508	31.24978	32.08666	18.08963
4256	LRRTM2	21.57643	3.213829	0.619836	5.184966	2.16E-07	1.35E-06	2.168345	4.379295	6.261582	37.94616	38.50399	40.19918
9875	PLA2G2F	8.923309	3.213701	1.112992	2.887442	0.003884	0.010456	0.542086	0	4.87012	20.08914	14.97377	13.06473
14252	LOC11962	2.944537	3.212724	1.632605	1.967851	0.049085	0.091561	1.084173	0.547412	0	4.464254	8.556443	3.014938
11385	FBXO40	5.330008	3.212248	1.264633	2.540063	0.011083	0.02588	0	1.094824	2.087194	8.928508	12.83466	7.034856
7125	RP1L1	25.87148	3.212203	0.870888	3.688424	0.000226	0.000842	0	2.189647	13.2189	37.94616	72.72976	29.1444
11512	LOC11961	5.382538	3.212092	1.278768	2.511864	0.01201	0.027734	0	1.094824	2.087194	13.39276	10.69555	5.024897
746	GALM	127.3752	3.211379	0.256919	12.49958	7.50E-36	2.67E-34	24.39389	24.63353	25.74206	216.5163	226.7457	246.22
16000	FNDC9	1.973655	3.211342	1.928274	1.665397	0.095834	0.159234	0.542086	0.547412	0	4.464254	4.278221	2.009959
13420	NANOG	4.556478	3.211317	1.512743	2.122843	0.033767	0.066892	0	0	2.782925	6.696381	12.83466	5.024897
12881	CCDC63	5.704961	3.210872	1.44498	2.222087	0.026277	0.054233	0	0	3.478657	4.464254	19.252	7.034856
16683	TPRX1	1.95904	3.210166	2.077874	1.544928	0.122364	0.194991	0	1.094824	0	2.232127	6.417332	2.009959
14269	LOC11961	2.945424	3.210153	1.632481	1.966425	0.049249	0.09176	0.542086	1.094824	0	4.464254	8.556443	3.014938
15251	SRRM4	31.69181	3.209243	1.79444	1.788437	0.073705	0.128481	0	0.547412	18.08902	73.66019	72.72976	25.12449
12453	TP53AIP1	4.142722	3.20692	1.388273	2.310007	0.020888	0.044591	1.084173	0	1.391463	8.928508	6.417332	7.034856
14324	CUNH9orf	31.35148	3.20639	1.638206	1.957257	0.050317	0.093388	0	1.094824	17.39328	64.73168	72.72976	32.15934
11853	RASGRP4	5.404064	3.206034	1.316644	2.435005	0.014892	0.033397	0	1.094824	2.087194	13.39276	12.83466	3.014938

12670	SLC22A8	4.379764	3.205541	1.414298	2.266524	0.023419	0.04914	0	0.547412	2.087194	8.928508	10.69555	4.019918
8456	TPBGL	12.41888	3.205481	0.981241	3.266761	0.001088	0.00342	0.542086	3.831883	2.782925	31.24978	32.08666	4.019918
16890	EN2	15.30704	3.204968	2.124653	1.508467	0.131435	0.206892	0	0	9.044508	24.5534	49.19955	9.044815
13728	LCN9	4.578003	3.204253	1.551571	2.065167	0.038907	0.075347	0	0	2.782925	6.696381	14.97377	3.014938
5950	LOC10321	19.29695	3.203369	0.765369	4.185389	2.85E-05	0.000127	2.710432	1.094824	7.653045	40.17829	47.06043	17.08465
12000	LOC11962	4.128107	3.202959	1.330931	2.406554	0.016104	0.035679	0.542086	0.547412	1.391463	6.696381	8.556443	7.034856
12649	TNP1	5.571297	3.202875	1.410304	2.271053	0.023144	0.048642	0	0.547412	2.782925	6.696381	21.39111	2.009959
8350	ZNF470	8.886863	3.202636	0.971483	3.296646	0.000978	0.003115	1.084173	3.284471	0.695731	20.08914	17.11289	11.05477
15191	LOC11962	3.460526	3.202556	1.781502	1.797671	0.072229	0.126405	0	0	2.087194	13.39276	4.278221	1.004979
14243	LOC11962	4.615031	3.20208	1.626475	1.968723	0.048985	0.091426	0	0	2.782925	8.928508	14.97377	1.004979
8101	ROS1	9.10574	3.201575	0.947156	3.380199	0.000724	0.002377	1.626259	2.189647	1.391463	17.85702	23.53022	8.039836
51	ELF3	2152.494	3.201027	0.10721	29.85756	7.00E-196	3.65E-193	430.9587	410.0114	427.1791	3770.062	3640.766	4235.988
8503	TDRD1	19.20051	3.200755	0.983095	3.255793	0.001131	0.003535	0	1.094824	10.43597	24.5534	62.03421	17.08465
12053	LOC10322	5.318752	3.200385	1.335199	2.396935	0.016533	0.036466	1.084173	0	2.087194	4.464254	19.252	5.024897
8831	CUNH9orf	8.544899	3.200041	1.011838	3.162603	0.001564	0.004707	0.542086	1.094824	3.478657	17.85702	19.252	9.044815
12885	PDE11A	5.555795	3.198576	1.439861	2.221448	0.026321	0.054306	0	0.547412	2.782925	4.464254	23.53022	2.009959
10082	ABCA6	7.58326	3.196754	1.124997	2.841566	0.004489	0.011838	3.252518	1.094824	0	6.696381	21.39111	13.06473
14628	LOC11962	3.392493	3.196197	1.680863	1.901522	0.057234	0.104014	0	0	2.087194	6.696381	8.556443	3.014938
538	MAF	244.21	3.195569	0.224885	14.20981	7.96E-46	3.94E-44	42.28274	41.05589	61.92009	484.3716	374.3444	461.2856
11560	LOC10322	7.964473	3.194569	1.276473	2.502653	0.012327	0.028348	0	0	4.87012	15.62489	19.252	8.039836
9346	WNT1	6.895042	3.194016	1.0577	3.019776	0.00253	0.007195	2.168345	1.094824	0.695731	13.39276	14.97377	9.044815
5244	RFX4	29.17369	3.193323	0.700888	4.55611	5.21E-06	2.64E-05	3.252518	1.094824	13.2189	60.26743	62.03421	35.17428
11157	EYA2	6.602249	3.192922	1.232707	2.590171	0.009593	0.022858	0	0.547412	3.478657	17.85702	10.69555	7.034856
10200	RHBDL2	6.335089	3.19273	1.133817	2.815912	0.004864	0.012676	0.542086	0.547412	2.782925	13.39276	10.69555	10.04979
8748	KCNK2	12.00108	3.190226	1.000227	3.189504	0.001425	0.004331	1.084173	0.547412	5.565851	24.5534	34.22577	6.029877
1373	SPOCK3	75.34146	3.189862	0.33068	9.646358	5.09E-22	9.86E-21	14.09425	15.87494	14.61036	154.0168	119.7902	133.6623
7029	PLCB2	22.65339	3.189587	0.856835	3.722522	0.000197	0.000746	0	3.831883	9.740239	37.94616	66.31243	18.08963
8884	TVP23A	8.247621	3.189074	1.012999	3.14815	0.001643	0.004917	0.542086	1.642235	2.782925	8.928508	23.53022	12.05975
12228	LOC11962	3.913477	3.188483	1.350479	2.361001	0.018226	0.039625	1.084173	0.547412	0.695731	6.696381	6.417332	8.039836
547	FOXJ1	240.8162	3.188239	0.22546	14.14106	2.12E-45	1.03E-43	43.909	41.05589	58.44144	515.6213	365.7879	420.0814
10188	LOC11962	6.135962	3.188192	1.131371	2.817991	0.004833	0.01261	1.084173	0.547412	2.087194	15.62489	6.417332	11.05477
8177	LOC11962	10.66834	3.187444	0.950495	3.353457	0.000798	0.002595	0.542086	1.094824	4.87012	15.62489	27.80844	14.06971
10270	LOC11962	6.356614	3.185903	1.139499	2.795881	0.005176	0.013398	0.542086	0.547412	2.782925	13.39276	12.83466	8.039836
759	MMP13	146.6927	3.185232	0.256295	12.42799	1.84E-35	6.45E-34	26.56223	35.58177	25.04633	258.9267	239.5804	294.459
9906	LRRC30	11.03109	3.184012	1.104103	2.883799	0.003929	0.010545	0	1.094824	5.565851	24.5534	29.94755	5.024897
17225	LOC11962	19.78904	3.183855	2.17557	1.463458	0.143342	0.221234	0	0	11.82743	44.64254	49.19955	13.06473
7947	GABRA4	20.03464	3.182912	0.929388	3.424741	0.000615	0.002059	1.084173	0	11.1317	37.94616	44.92132	25.12449
8304	MAGEA3	13.60172	3.181691	0.960662	3.311979	0.000926	0.002966	0.542086	2.737059	4.87012	8.928508	53.47777	11.05477
12762	LOC11962	5.614978	3.179933	1.414636	2.247881	0.024584	0.051211	0	0	3.478657	15.62489	8.556443	6.029877
12954	LOC10323	4.172045	3.178827	1.436811	2.212419	0.026938	0.055283	0	1.094824	1.391463	6.696381	12.83466	3.014938
11382	AQP9	4.891269	3.17626	1.249653	2.541713	0.011031	0.025765	1.084173	1.094824	0.695731	4.464254	14.97377	7.034856
4888	CD209	26.10582	3.17616	0.668876	4.748505	2.05E-06	1.11E-05	4.336691	1.642235	9.740239	58.0353	57.75599	25.12449
7650	LOC10323	12.21416	3.173359	0.904104	3.509951	0.000448	0.001557	3.252518	0	4.174388	20.08914	25.66933	20.09959
12936	LOC10322	3.936778	3.173245	1.432339	2.215429	0.026731	0.054935	0	1.642235	0.695731	6.696381	8.556443	6.029877
490	LOC10324	196.874	3.17264	0.215268	14.73806	3.67E-49	1.99E-47	44.45108	34.48694	38.96096	348.2118	359.3706	355.7627
12947	GLRA1	5.621	3.172106	1.432785	2.213945	0.026833	0.055097	0	0	3.478657	13.39276	12.83466	4.019918
9799	CD207	8.568731	3.172035	1.091924	2.904996	0.003673	0.009964	1.084173	0	4.174388	17.85702	19.252	9.044815
5742	HVCN1	15.29203	3.16966	0.736236	4.305226	1.67E-05	7.72E-05	2.168345	2.189647	4.87012	33.4819	29.94755	19.09461
10404	LOC11962	7.158579	3.169439	1.146008	2.765633	0.005681	0.014517	0	2.189647	2.087194	13.39276	19.252	6.029877
14536	CR2	4.125537	3.167272	1.650272	1.919243	0.054954	0.100505	0	1.094824	1.391463	0	19.252	3.014938
14159	PDE6C	3.775567	3.16631	1.596605	1.983152	0.04735	0.088905	0	2.189647	0	11.16063	4.278221	5.024897
12148	LOC11962	5.302197	3.166171	1.332635	2.375873	0.017507	0.038314	0	0.547412	2.782925	4.464254	14.97377	9.044815
11184	LOC10323	7.33006	3.166045	1.22476	2.585032	0.009737	0.023145	1.084173	0.547412	2.782925	4.464254	32.08666	3.014938
4120	ITGAL	27.61648	3.165889	0.600023	5.276277	1.32E-07	8.51E-07	3.794605	3.831883	9.044508	53.57105	66.31243	29.1444
6671	LOC10324	15.91709	3.165288	0.81827	3.868267	0.00011	0.000437	5.420864	0.547412	3.478657	33.4819	38.50399	14.06971
5137	LOC10321	20.32973	3.164654	0.686783	4.60794	4.07E-06	2.10E-05	1.084173	4.379295	6.957314	33.4819	44.92132	31.15436
6908	SCGN	12.16235	3.164031	0.838287	3.774399	0.00016	0.000617	1.084173	1.642235	4.87012	17.85702	21.39111	26.12947
14650	CT45A10	3.01754	3.163997	1.667833	1.89707	0.057819	0.104922	0	1.094824	0.695731	11.16063	2.139111	3.014938
5323	KRT76	19.61298	3.162999	0.701704	4.507594	6.56E-06	3.27E-05	2.168345	2.189647	7.653045	33.4819	47.06043	25.12449
14124	LOC10323	3.016652	3.16288	1.58956	1.989784	0.046615	0.087741	0.542086	0.547412	0.695731	11.16063	2.139111	3.014938
6866	CLEC17A	12.17183	3.161113	0.834147	3.789637	0.000151	0.000584	2.168345	1.642235	3.478657	29.01765	25.66933	11.05477
9736	SLC32A1	13.30327	3.160916	1.082558	2.919857	0.003502	0.009562	0	0.547412	7.653045	40.17829	21.39111	10.04979
10309	LOC11962	7.384996	3.159988	1.133879	2.786883	0.005322	0.013724	0	1.094824	3.478657	17.85702	12.83466	9.044815
13976	LOC11962	4.450992	3.159984	1.567289	2.01621	0.043778	0.083274	0	0	2.782925	15.62489	4.278221	4.019918
320	RASD2	309.234	3.159653	0.186773	16.91708	3.37E-64	2.80E-62	56.91907	64.04718	66.79021	535.7105	504.8301	627.1072
4133	LOC10322	26.46256	3.159652	0.599568	5.269884	1.37E-07	8.78E-07	4.336691	7.116354	4.174388	60.26743	57.75599	25.12449
14750	LOC10322	3.169271	3.159397	1.67943	1.881232	0.05994	0.108035	0	0.547412	1.391463	2.232127	12.83466	2.009959
9274	CD40LG	13.17669	3.157737	1.039092	3.03894	0.002374	0.006806	0	0.547412	7.653045	31.24978	23.53022	16.07967
16433	FAM3D	2.175977	3.156417	1.993218	1.583578	0.11329	0.183278	0	0	1.391463	2.232127	6.417332	3.014938
16574	LOC11962	2.228507	3.156331	2.020789	1.56193	0.118305	0.189751	0	0	1.391463	6.696381	4.278221	1.004979
16576	TRIM69	2.228507	3.156331	2.020789	1.56193	0.118305	0.189751	0	0	1.391463	6.696381	4.278221	1.004979
15647	LOC10324	3.15288	3.154915	1.828658	1.725263	0.08448	0.143501	0.542086	0	1.391463	0	14.97377	2.009959
12437	P2RY4	5.25207	3.15325	1.363157	2.313196	0.020712	0.044273	0	1.094824	2.087194	15.62489	10.69555	2.009959
12616	HRH3	5.382275	3.152393	1.383272	2.27894	0.022671	0.047773	0	0.547412	2.782925	6.696381	19.252	3.014938

4410	PCDH15	26.22194	3.152307	0.621429	5.072672	3.92E-07	2.36E-06	4.336691	3.831883	7.653045	69.19594	49.19955	23.11453
5577	CPNE5	16.96452	3.150257	0.718768	4.382855	1.17E-05	5.58E-05	2.710432	3.284471	4.174388	40.17829	36.36488	15.07469
11167	GRM5	7.573755	3.150099	1.217354	2.587662	0.009663	0.023004	0	0.547412	4.174388	11.16063	23.53022	6.029877
9781	LOC10321(	12.05486	3.149935	1.082801	2.909062	0.003625	0.009852	0.542086	0	6.957314	31.24978	23.53022	10.04979
14385	ZBBX	2.986534	3.149461	1.616997	1.947723	0.051448	0.095082	0	1.094824	0.695731	6.696381	6.417332	3.014938
584	LOC10323!	229.9178	3.149163	0.228593	13.77631	3.54E-43	1.61E-41	40.11439	38.86624	61.92009	435.2648	372.2053	431.1362
14426	LOC11962(	3.038178	3.14896	1.623857	1.939186	0.052479	0.096711	0.542086	0.547412	0.695731	11.16063	4.278221	1.004979
8597	KRT5	7.949031	3.148872	0.974803	3.230266	0.001237	0.003824	1.084173	1.642235	2.087194	17.85702	14.97377	10.04979
9835	PGM5	8.11804	3.148741	1.086501	2.898056	0.003755	0.01015	0	2.189647	2.782925	11.16063	23.53022	9.044815
13236	LOC11961{	3.743674	3.147901	1.457089	2.160405	0.030741	0.061745	0.542086	1.642235	0	6.696381	8.556443	5.024897
12569	ASCL5	7.824525	3.147813	1.374752	2.289732	0.022037	0.046611	0	0	4.87012	13.39276	25.66933	3.014938
618	SH3RF3	159.1603	3.147633	0.234751	13.40841	5.40E-41	2.32E-39	29.81475	31.74989	35.4823	310.2657	265.2497	282.3992
16617	LOC11961{	2.213005	3.147322	2.023424	1.555443	0.11984	0.191706	0	0	1.391463	4.464254	6.417332	1.004979
16618	CNMD	2.213005	3.147322	2.023424	1.555443	0.11984	0.191706	0	0	1.391463	4.464254	6.417332	1.004979
16619	LOC11962(	2.213005	3.147322	2.023424	1.555443	0.11984	0.191706	0	0	1.391463	4.464254	6.417332	1.004979
9118	LOC11962;	12.91298	3.146724	1.022082	3.078739	0.002079	0.006061	0	1.094824	6.957314	17.85702	38.50399	13.06473
12084	LOC11962;	7.691915	3.145818	1.316194	2.390088	0.016844	0.037057	0	0	4.87012	6.696381	23.53022	11.05477
4245	LOC10322(	47.83243	3.145496	0.605985	5.190719	2.09E-07	1.31E-06	2.710432	6.568942	20.17621	73.66019	132.6249	51.25395
10311	BHLHA9	5.719636	3.145447	1.129012	2.786016	0.005336	0.013758	1.626259	1.094824	0.695731	11.16063	10.69555	9.044815
14843	LOC11962(	3.023562	3.145434	1.688345	1.863028	0.062458	0.11186	0	1.094824	0.695731	8.928508	6.417332	1.004979
14844	LOC10324:	3.023562	3.145434	1.688345	1.863028	0.062458	0.11186	0	1.094824	0.695731	8.928508	6.417332	1.004979
10374	PRSS55	5.940288	3.145395	1.135333	2.77046	0.005598	0.014345	1.084173	1.094824	1.391463	8.928508	17.11289	6.029877
8730	PIK3R6	14.84661	3.144327	0.984546	3.193683	0.001405	0.004278	0	2.189647	6.957314	31.24978	40.6431	8.039836
308	ESR1	293.2712	3.144199	0.181704	17.30399	4.39E-67	3.79E-65	65.05036	57.47824	56.35424	497.7643	526.2212	556.7586
12230	CA10	5.168534	3.143364	1.331714	2.36039	0.018256	0.039683	0	1.094824	2.087194	6.696381	17.11289	4.019918
11168	LOC10322{	5.636988	3.141676	1.21436	2.587104	0.009679	0.02304	1.084173	1.642235	0.695731	2.232127	17.11289	11.05477
2155	APOL2	61.31537	3.140225	0.409196	7.67414	1.67E-14	2.05E-13	12.46799	6.568942	18.78475	116.0706	115.512	98.48799
14882	MSR1	3.00806	3.139014	1.689502	1.857952	0.063176	0.112845	0	1.094824	0.695731	6.696381	8.556443	1.004979
14780	SERPINB13	2.955529	3.138927	1.672619	1.876654	0.060566	0.10894	0	1.094824	0.695731	2.232127	10.69555	3.014938
13011	DEFB125	4.17524	3.138649	1.425521	2.201756	0.027683	0.056563	0	0.547412	2.087194	6.696381	10.69555	5.024897
11768	ASB14	5.836743	3.138381	1.278327	2.45507	0.014086	0.031821	1.626259	1.094824	0.695731	15.62489	14.97377	1.004979
290	S100A4	4358.28	3.138374	0.178053	17.62603	1.56E-69	1.43E-67	1045.685	582.9936	1039.423	7595.928	7655.877	8229.777
9105	LOC10324(	10.63063	3.137121	1.017911	3.081921	0.002057	0.006005	0.542086	0.547412	5.565851	24.5534	23.53022	9.044815
16824	LOC10323;	2.197502	3.137056	2.062959	1.520658	0.128346	0.20281	0	0	1.391463	2.232127	8.556443	1.004979
14012	LOC11962!	3.734194	3.136882	1.560003	2.010818	0.044345	0.084135	0.542086	1.642235	0	2.232127	14.97377	3.014938
11375	TRIM10	4.968519	3.135361	1.232963	2.542949	0.010992	0.02569	1.084173	0.547412	1.391463	8.928508	12.83466	5.024897
5413	ARHGAP3(	42.38651	3.134653	0.703566	4.455377	8.37E-06	4.11E-05	2.168345	3.284471	20.87194	64.73168	124.0684	39.1942
14995	LOC11962!	2.992557	3.133733	1.707448	1.835331	0.066457	0.117823	0	1.094824	0.695731	4.464254	10.69555	1.004979
9267	NEU4	10.27207	3.131889	1.030036	3.040561	0.002361	0.006774	0	2.189647	4.174388	31.24978	14.97377	9.044815
13144	ADAMTS4	72.61871	3.131854	1.435506	2.181706	0.029131	0.058921	0.542086	3.831883	40.35242	154.0168	179.6853	57.28383
81	ALOX5	909.523	3.130715	0.121353	25.79833	9.26E-147	3.04E-144	175.636	192.689	191.3261	1676.327	1567.968	1653.191
8678	CASQ1	10.36949	3.129816	0.975416	3.208699	0.001333	0.004085	1.084173	0.547412	4.87012	17.85702	27.80844	10.04979
9510	LOC10324:	15.11608	3.128818	1.04901	2.98264	0.002858	0.007989	0	1.094824	8.348776	35.71403	38.50399	7.034856
13333	C8A	4.196765	3.128684	1.460646	2.141986	0.032195	0.064194	0	0.547412	2.087194	6.696381	12.83466	3.014938
13125	LOC10323{	5.425956	3.128495	1.433098	2.18303	0.029034	0.058799	0	0	3.478657	15.62489	6.417332	7.034856
8936	LOC10322(	20.81219	3.127997	0.997699	3.135212	0.001717	0.005109	0	0.547412	12.52316	44.64254	47.06043	20.09959
13261	LOC10324:	4.029531	3.127801	1.451105	2.155461	0.031126	0.062399	0	1.094824	1.391463	13.39276	4.278221	4.019918
11943	LOC11962:	8.799488	3.127036	1.294317	2.415975	0.015693	0.034933	0	0	5.565851	13.39276	27.80844	6.029877
11574	DAPP1	4.718637	3.126133	1.2501	2.500706	0.012395	0.02847	0.542086	1.642235	0.695731	6.696381	10.69555	8.039836
13853	SLCO1A2	4.233793	3.125634	1.533078	2.038796	0.04147	0.079585	0	0.547412	2.087194	8.928508	12.83466	1.004979
7942	LOC10321!	9.576701	3.125159	0.912295	3.425601	0.000613	0.002053	2.710432	1.642235	1.391463	24.5534	17.11289	10.04979
8202	LOC10323;	16.96723	3.124841	0.933961	3.345794	0.00082	0.002659	0	1.642235	9.044508	31.24978	42.78221	17.08465
8615	PODNL1	8.664632	3.123702	0.968595	3.224982	0.00126	0.003888	0.542086	2.737059	2.087194	11.16063	21.39111	14.06971
8916	LOC11962!	10.1205	3.123105	0.994663	3.139864	0.00169	0.00504	0	2.189647	4.174388	15.62489	25.66933	13.06473
7406	TMEM119	10.80332	3.121554	0.867444	3.598564	0.00032	0.001149	2.168345	1.642235	2.782925	26.78552	21.39111	10.04979
97	BCHE	847.4551	3.121246	0.127801	24.42277	9.80E-132	2.69E-129	182.6831	181.1933	160.0182	1446.418	1514.49	1599.927
17732	NTSR1	2.181999	3.1211	2.263029	1.37917	0.167842	0.251641	0	0	1.391463	0	10.69555	1.004979
14779	LOC10323:	4.072582	3.120586	1.662327	1.87724	0.060485	0.108803	0	1.094824	1.391463	13.39276	8.556443	0
9247	LOC10324!	9.142706	3.119801	1.025171	3.043202	0.002341	0.00673	0	1.642235	4.174388	17.85702	17.11289	14.06971
2888	SERPINA4	34.07027	3.119288	0.475227	6.563789	5.25E-11	4.83E-10	5.96295	8.211177	6.957314	55.80317	64.17332	63.31371
15021	LOC11962(	3.218974	3.118736	1.705041	1.829127	0.067381	0.119254	0	0	2.087194	8.928508	4.278221	4.019918
15545	LOC11962:	3.271504	3.118244	1.795587	1.736615	0.082455	0.141014	0	0	2.087194	13.39276	2.139111	2.009959
9779	CCDC88B	11.76769	3.117432	1.071609	2.909114	0.003625	0.009852	0	0.547412	6.957314	20.08914	29.94755	13.06473
16212	HMGCS2	1.999263	3.116934	1.917597	1.625437	0.104069	0.170625	0	0.547412	0.695731	4.464254	4.278221	2.009959
16213	DPEP3	1.999263	3.116934	1.917597	1.625437	0.104069	0.170625	0	0.547412	0.695731	4.464254	4.278221	2.009959
16214	LOC11961!	1.999263	3.116934	1.917597	1.625437	0.104069	0.170625	0	0.547412	0.695731	4.464254	4.278221	2.009959
5217	LOC10323(	32.99889	3.114982	0.681437	4.571193	4.85E-06	2.47E-05	1.626259	3.831883	15.30609	69.19594	74.86887	33.16432
16740	ZNF683	3.308532	3.114804	2.029035	1.535116	0.124755	0.198126	0	0	2.087194	15.62489	2.139111	0
9087	LOC10324:	13.77298	3.114508	1.009011	3.086694	0.002024	0.005921	0	1.094824	7.653045	22.32127	38.50399	13.06473
16140	FEZF2	4.401027	3.114322	1.899752	1.639331	0.101144	0.1666	0	0	2.782925	2.232127	21.39111	0
12246	LOC11962(	5.186343	3.11399	1.320872	2.357527	0.018397	0.039939	0.542086	0	2.782925	8.928508	12.83466	6.029877
13447	XCR1	4.035554	3.113944	1.470293	2.117908	0.034183	0.06758	0	1.094824	1.391463	11.16063	8.556443	2.009959
15298	LOC11962:	3.256002	3.113689	1.74969	1.779566	0.075147	0.130591	0	0	2.087194	11.16063	4.278221	2.009959
14981	LOC11962(	3.203471	3.113497	1.694014	1.837941	0.066071	0.117249	0	0	2.087194	6.696381	6.417332	4.019918

9600	AKR1D1	11.5815	3.113052	1.051343	2.961025	0.003066	0.008491	0	1.094824	6.261582	17.85702	34.22577	10.04979
13461	LOC103236	5.438001	3.112489	1.471405	2.115318	0.034403	0.067944	0	0	3.478657	11.16063	14.97377	3.014938
3559	LOC103237	28.06284	3.112417	0.536712	5.799049	6.67E-09	4.98E-08	4.336691	7.663765	5.565851	40.17829	51.33866	59.29379
2313	LOC103218	47.02561	3.112053	0.42084	7.394857	1.42E-13	1.63E-12	10.84173	9.306001	9.044508	64.73168	104.8164	83.4133
16328	LOC119624	1.982873	3.111945	1.944944	1.600017	0.109595	0.17844	0.542086	0	0.695731	2.232127	6.417332	2.009959
9856	LOC119625	6.760063	3.111617	1.076045	2.891717	0.003831	0.010335	1.084173	1.642235	1.391463	11.16063	19.252	6.029877
9758	LYPD5	9.352103	3.111518	1.067698	2.914229	0.003566	0.009714	0.542086	0.547412	4.87012	11.16063	29.94755	9.044815
10934	WNT10A	6.002036	3.110771	1.176789	2.64344	0.008207	0.019954	1.084173	0.547412	2.087194	11.16063	17.11289	4.019918
14938	PPP1R1C	2.77704	3.110087	1.683674	1.847203	0.064718	0.115177	1.084173	0.547412	0	4.464254	8.556443	2.009959
5256	SLC22A3	16.85298	3.109813	0.683488	4.549918	5.37E-06	2.71E-05	4.336691	3.284471	2.782925	40.17829	21.39111	29.1444
11407	CLEC4F	7.332202	3.109197	1.226804	2.534388	0.011264	0.02625	0	0.547412	4.174388	6.696381	23.53022	9.044815
4234	NLGN4X	35.00272	3.107536	0.597899	5.197424	2.02E-07	1.27E-06	2.168345	6.02153	13.91463	60.26743	83.42532	44.2191
8289	MOGAT3	9.735177	3.107144	0.93684	3.316623	0.000911	0.002922	0.542086	2.737059	2.782925	15.62489	25.66933	11.05477
2890	LRG1	41.194	3.106933	0.473492	6.561741	5.32E-11	4.89E-10	10.84173	7.663765	6.957314	53.57105	104.8164	63.31371
7448	LOC119618	16.00395	3.106557	0.867066	3.58284	0.00034	0.001213	1.084173	3.284471	5.565851	49.10679	29.94755	7.034856
5465	LOC119621	17.76377	3.106417	0.70091	4.431978	9.34E-06	4.54E-05	2.168345	2.737059	6.261582	37.94616	36.36488	21.10457
15084	OTOP3	3.187968	3.106379	1.707636	1.819112	0.068894	0.121424	0	0	2.087194	4.464254	8.556443	4.019918
8469	LOC119625	16.69226	3.10601	0.951995	3.262632	0.001104	0.003465	0	5.474118	4.87012	35.71403	47.06043	7.034856
12275	NXNL1	5.041523	3.105703	1.321637	2.34989	0.018779	0.040671	0	1.094824	2.087194	15.62489	6.417332	5.024897
8593	LOC103218	11.54311	3.104222	0.960671	3.231305	0.001232	0.003812	0	4.379295	2.782925	20.08914	29.94755	12.05975
14343	LRAT	4.025186	3.103728	1.588666	1.95367	0.05074	0.094041	0.542086	0.547412	1.391463	6.696381	14.97377	0
17179	LOC103221	1.96737	3.103366	2.109768	1.470951	0.141304	0.218673	0.542086	0	0.695731	0	8.556443	2.009959
12604	GFRA3	4.563449	3.102702	1.359699	2.281904	0.022495	0.047448	0.542086	2.189647	0	8.928508	10.69555	5.024897
17238	DPPA5	2.020788	3.101529	2.12215	1.461504	0.143877	0.221892	0	0.547412	0.695731	4.464254	6.417332	0
9654	PNMA6F	12.61501	3.100768	1.052656	2.945661	0.003223	0.008874	0	1.094824	6.957314	20.08914	38.50399	9.044815
7325	MORN3	10.59277	3.100506	0.855136	3.625748	0.000288	0.001046	2.168345	1.642235	2.782925	26.78552	17.11289	13.06473
8253	EPN3	11.05535	3.099782	0.931136	3.329031	0.000871	0.002807	1.626259	0.547412	4.87012	31.24978	14.97377	13.06473
17189	CPO	1.968257	3.099239	2.109424	1.469235	0.141769	0.219264	0	0.547412	0.695731	0	8.556443	2.009959
2563	LOC119625	81.84411	3.09906	0.440801	7.030521	2.06E-12	2.13E-11	14.09425	7.663765	29.91645	151.7846	186.1026	101.5029
7730	ATP1B4	22.15242	3.099006	0.889709	3.48317	0.000496	0.001704	1.084173	0.547412	12.52316	33.4819	64.17332	21.10457
17305	LOC119619	1.800136	3.098698	2.140072	1.447941	0.147634	0.226808	0.542086	0.547412	0	6.696381	0	3.014938
10272	LOC103244	8.271654	3.098672	1.108562	2.795218	0.005186	0.013423	0.542086	0.547412	4.174388	20.08914	19.252	5.024897
10003	BRDT	8.015749	3.098274	1.083172	2.86037	0.004231	0.011246	0	1.642235	3.478657	20.08914	12.83466	10.04979
6979	CHRNE	20.74201	3.09816	0.827914	3.742126	0.000182	0.000695	0.542086	1.642235	11.1317	37.94616	47.06043	26.12947
9974	LOC119625	7.440947	3.097692	1.079593	2.869316	0.004114	0.010965	2.710432	1.094824	0.695731	24.5534	8.556443	7.034856
12209	LOC103239	9.66939	3.096037	1.308908	2.365359	0.018013	0.039223	0	0	6.261582	4.464254	34.22577	13.06473
10126	GHSR	10.26232	3.094761	1.09288	2.831749	0.004629	0.012154	0	1.642235	4.87012	20.08914	29.94755	5.024897
7241	GPR61	15.03669	3.094252	0.846517	3.655275	0.000257	0.000943	0.542086	2.737059	6.261582	31.24978	36.36488	13.06473
5290	SDK2	25.5112	3.093414	0.68398	4.522664	6.11E-06	3.07E-05	4.336691	3.284471	8.348776	53.57105	68.45154	15.07469
11841	CTSV	4.978624	3.092545	1.268024	2.43887	0.014733	0.033079	0.542086	0.547412	2.087194	6.696381	14.97377	5.024897
8237	LOC119627	11.04764	3.092208	0.927057	3.33551	0.000851	0.002748	0.542086	1.642235	4.87012	26.78552	21.39111	11.05477
6095	NXPH3	38.40201	3.09098	0.749473	4.124204	3.72E-05	0.000162	0.542086	3.831883	20.17621	80.35657	81.28621	44.2191
13214	MPPED2	5.051793	3.090803	1.427355	2.165406	0.030357	0.061074	1.084173	0	2.087194	11.16063	14.97377	1.004979
15835	LOC119622	30.53668	3.090338	1.825562	1.692815	0.090491	0.151923	0	0.547412	18.78475	44.64254	94.12087	25.12449
11401	LOC119625	7.272337	3.090265	1.218586	2.535944	0.011214	0.02615	0.542086	0	4.174388	22.32127	8.556443	8.039836
11980	IL31RA	8.456332	3.089979	1.282448	2.409437	0.015977	0.035455	0	0	5.565851	6.696381	21.39111	17.08465
6316	LOC103218	19.84709	3.088923	0.768685	4.01845	5.86E-05	0.000247	3.252518	1.642235	7.653045	35.71403	57.75599	13.06473
11191	FAM186B	5.642586	3.088572	1.195521	2.583452	0.009782	0.023235	1.084173	1.642235	0.695731	17.85702	8.556443	4.019918
7174	LOC103235	19.96456	3.088155	0.840671	3.673439	0.000239	0.000887	0	4.379295	8.348776	33.4819	53.47777	20.09959
8796	NR5A1	13.36587	3.088116	0.97244	3.175637	0.001495	0.004519	0.542086	1.094824	6.957314	15.62489	44.92132	11.05477
12283	CD48	8.579461	3.087784	1.314697	2.348666	0.018841	0.040779	0	0	5.565851	8.928508	29.94755	7.034856
19573	LOC103226	0.990466	3.087602	2.837659	1.088081	0.27656	0.375564	0	0	0.695731	2.232127	0	3.014938
19574	LOC103231	0.990466	3.087602	2.837659	1.088081	0.27656	0.375564	0	0	0.695731	2.232127	0	3.014938
19575	GFRAL	0.990466	3.087602	2.837659	1.088081	0.27656	0.375564	0	0	0.695731	2.232127	0	3.014938
19577	LOC103233	0.990466	3.087602	2.837659	1.088081	0.27656	0.375564	0	0	0.695731	2.232127	0	3.014938
8545	ZKSCAN7	11.16451	3.087321	0.952389	3.241659	0.001188	0.003697	3.794605	1.642235	1.391463	15.62489	38.50399	6.029877
19641	SERPINA11	0.965746	3.086865	2.860848	1.079004	0.280586	0.379786	0	0.547412	0	2.232127	0	3.014938
12252	LOC103232	6.257776	3.086463	1.310209	2.355702	0.018488	0.040116	0	0.547412	3.478657	13.39276	17.11289	3.014938
11738	LOC103240	4.726967	3.08589	1.254011	2.460815	0.013862	0.031396	1.084173	0.547412	1.391463	4.464254	12.83466	8.039836
10749	RSPO1	9.445744	3.08561	1.151412	2.679849	0.007366	0.018217	0	0.547412	5.565851	17.85702	25.66933	7.034856
3598	PEG3	30.11305	3.085489	0.536925	5.746588	9.11E-09	6.73E-08	4.336691	7.663765	6.957314	44.64254	74.86887	42.20914
14275	LOC119625	26.64824	3.084763	1.569359	1.965619	0.049343	0.091887	0	1.642235	15.30609	49.10679	72.72976	21.10457
9089	SYNPO2	10.10599	3.084657	0.999529	3.086112	0.002028	0.005932	1.084173	0.547412	4.87012	31.24978	12.83466	10.04979
11721	LOC119618	6.057761	3.084414	1.251345	2.46488	0.013706	0.031087	1.084173	0	2.782925	15.62489	12.83466	4.019918
10592	LOC119621	11.61224	3.084393	1.132353	2.72388	0.006452	0.016194	0	0.547412	6.957314	15.62489	38.50399	8.039836
11107	TSSK2	5.57544	3.084183	1.184499	2.603788	0.00922	0.022068	0.542086	2.189647	0.695731	11.16063	12.83466	6.029877
8241	RASAL3	17.43296	3.083119	0.924797	3.333832	0.000857	0.002763	2.168345	0	9.044508	31.24978	47.06043	15.07469
13275	NELL1	4.910591	3.082976	1.43225	2.15254	0.031355	0.062792	0.542086	0.547412	2.087194	0	19.252	7.034856
7438	LOC103218	12.81243	3.082397	0.859677	3.585528	0.000336	0.001202	1.626259	1.642235	4.87012	22.32127	36.36488	10.04979
5814	DMD	19.81938	3.082076	0.722286	4.267114	1.98E-05	9.05E-05	1.626259	2.737059	8.348776	40.17829	44.92132	21.10457
17283	CDRT4	1.768243	3.081503	2.120975	1.452871	0.14626	0.224979	1.084173	0	0	2.232127	4.278221	3.014938
15138	CUNH1orf1	3.744298	3.081065	1.704105	1.808025	0.070603	0.123991	0	1.642235	0.695731	0	17.11289	3.014938
14687	TMEM225	2.995752	3.079369	1.627803	1.891734	0.058526	0.105932	0	0.547412	1.391463	4.464254	8.556443	3.014938
14688	LOC103221	2.995752	3.079369	1.627803	1.891734	0.058526	0.105932	0	0.547412	1.391463	4.464254	8.556443	3.014938

15041	LOC119625	3.048282	3.078262	1.686164	1.825601	0.06791	0.120032	0	0.547412	1.391463	8.928508	6.417332	1.004979
6398	NLRP1	22.50261	3.077242	0.773073	3.980532	6.88E-05	0.000286	1.084173	4.926706	8.348776	24.5534	77.00798	19.09461
14818	LOC103222	2.758709	3.076391	1.645964	1.869051	0.061616	0.110547	1.084173	0	0.695731	4.464254	4.278221	6.029877
14593	LOC103224	3.577065	3.075868	1.612051	1.908046	0.056385	0.102721	0	2.189647	0	6.696381	8.556443	4.019918
10759	ACSM5	6.997472	3.074961	1.148383	2.677645	0.007414	0.01832	0	1.094824	3.478657	13.39276	14.97377	9.044815
17280	LOC103244	19.55582	3.074528	2.115493	1.453338	0.14613	0.224818	0	0	12.52316	40.17829	38.50399	26.12947
11687	CUNH16or	5.861298	3.074252	1.242656	2.473936	0.013363	0.030398	0	1.642235	2.087194	17.85702	8.556443	5.024897
13331	PM20D1	3.986218	3.073994	1.434909	2.142292	0.03217	0.064154	0	0.547412	2.087194	6.696381	8.556443	6.029877
11419	LOC103248	8.343769	3.073806	1.213988	2.53199	0.011342	0.026405	0	0.547412	4.87012	26.78552	12.83466	5.024897
10285	LOC103227	8.001666	3.073347	1.10075	2.792049	0.005238	0.013538	1.084173	0	4.174388	17.85702	12.83466	12.05975
10203	CALHM5	8.440888	3.072934	1.091394	2.815605	0.004869	0.012682	2.168345	1.642235	1.391463	15.62489	27.80844	2.009959
19679	LOC119619	1.001887	3.072814	2.861572	1.07382	0.282903	0.382183	0.542086	0	0	4.464254	0	1.004979
19672	AQP12B	1.002774	3.072793	2.860775	1.074112	0.282773	0.382045	0	0.547412	0	4.464254	0	1.004979
19673	SLC10A2	1.002774	3.072793	2.860775	1.074112	0.282773	0.382045	0	0.547412	0	4.464254	0	1.004979
19674	LOC119628	1.002774	3.072793	2.860775	1.074112	0.282773	0.382045	0	0.547412	0	4.464254	0	1.004979
19675	LOC103215	1.002774	3.072793	2.860775	1.074112	0.282773	0.382045	0	0.547412	0	4.464254	0	1.004979
19676	LOC119619	1.002774	3.072793	2.860775	1.074112	0.282773	0.382045	0	0.547412	0	4.464254	0	1.004979
19677	LOC119621	1.002774	3.072793	2.860775	1.074112	0.282773	0.382045	0	0.547412	0	4.464254	0	1.004979
19623	LOC119627	1.027494	3.072205	2.838992	1.082146	0.279188	0.37824	0	0	0.695731	4.464254	0	1.004979
7196	CIITA	14.67095	3.07201	0.837376	3.668613	0.000244	0.000901	0.542086	3.284471	5.565851	33.4819	32.08666	13.06473
6852	ALOX15B	12.39956	3.071876	0.80938	3.795345	0.000147	0.000572	2.168345	2.189647	3.478657	24.5534	29.94755	12.05975
8342	SCRT1	17.5654	3.071131	0.930759	3.299597	0.000968	0.003085	0.542086	1.094824	9.740239	40.17829	42.78221	11.05477
12261	LOC103226	4.838051	3.070032	1.304344	2.353698	0.018588	0.040303	1.084173	0.547412	1.391463	11.16063	12.83466	2.009959
5695	LOC103243	17.3506	3.069628	0.709102	4.328894	1.50E-05	7.00E-05	5.96295	2.189647	2.782925	44.64254	21.39111	27.13445
8422	GPR50	16.35366	3.069465	0.937057	3.275644	0.001054	0.003328	0	1.642235	9.044508	24.5534	42.78221	20.09959
14992	LY96	2.743207	3.069217	1.672255	1.835377	0.06645	0.11782	1.084173	0	0.695731	2.232127	6.417332	6.029877
19661	MAGEH1	0.974963	3.069001	2.850857	1.076519	0.281695	0.38088	0	0	0.695731	0	2.139111	3.014938
19662	LOC119623	0.974963	3.069001	2.850857	1.076519	0.281695	0.38088	0	0	0.695731	0	2.139111	3.014938
9581	LOC103243	13.42248	3.068789	1.034845	2.965458	0.003022	0.008386	0	1.094824	7.653045	20.08914	40.6431	11.05477
13867	LOC119622	3.558899	3.068276	1.506652	2.036487	0.041701	0.079948	1.626259	0.547412	0	4.464254	10.69555	4.019918
19710	LOC103223	0.950243	3.068139	2.874605	1.067325	0.285825	0.385445	0	0.547412	0	0	2.139111	3.014938
19711	LOC103246	0.950243	3.068139	2.874605	1.067325	0.285825	0.385445	0	0.547412	0	0	2.139111	3.014938
19713	LOC103233	0.950243	3.068139	2.874605	1.067325	0.285825	0.385445	0	0.547412	0	0	2.139111	3.014938
13047	AIRE	7.452503	3.067721	1.396726	2.196365	0.028066	0.057188	0	0	4.87012	11.16063	25.66933	3.014938
6184	ECE2	15.1867	3.066631	0.750976	4.083527	4.44E-05	0.000191	2.710432	2.737059	4.174388	26.78552	40.6431	14.06971
15270	LOC103221	2.848268	3.066314	1.718103	1.78471	0.074308	0.129371	1.084173	0	0.695731	11.16063	2.139111	2.009959
8817	EBF2	9.375464	3.065903	0.967177	3.169949	0.001525	0.004597	0.542086	3.284471	2.087194	15.62489	25.66933	9.044815
16912	MC4R	1.821662	3.065779	2.037833	1.504431	0.13247	0.208232	0.542086	0.547412	0	6.696381	2.139111	1.004979
8453	LOC103245	9.322046	3.065045	0.937851	3.268158	0.001082	0.003404	1.084173	2.737059	2.087194	11.16063	27.80844	11.05477
7675	PLA2G4F	21.38429	3.064633	0.875704	3.499622	0.000466	0.001614	2.168345	0.547412	11.1317	44.64254	57.75599	12.05975
11149	PROZ	6.599477	3.064424	1.182076	2.592409	0.009531	0.022726	0.542086	2.189647	1.391463	8.928508	23.53022	3.014938
17557	LOC103246	1.789769	3.062572	2.178025	1.406124	0.159687	0.2418	1.084173	0	0	2.232127	6.417332	1.004979
9988	DPP6	9.931213	3.061417	1.069013	2.86378	0.004186	0.011142	0	1.642235	4.87012	8.928508	32.08666	12.05975
11712	PERCC1	9.495447	3.061366	1.239798	2.469247	0.01354	0.030734	0	0	6.261582	24.5534	17.11289	9.044815
11762	LOC119624	8.303284	3.061094	1.24671	2.455338	0.014075	0.031803	0	0.547412	4.87012	17.85702	23.53022	3.014938
11852	RAB39B	9.516972	3.060125	1.256482	2.43547	0.014872	0.03336	0	0	6.261582	24.5534	19.252	7.034856
84	DKK3	1646.393	3.059663	0.11932	25.64247	5.13E-145	1.62E-142	323.0835	359.1022	377.7821	2901.765	2701.697	3214.929
14851	CD33	2.780235	3.059464	1.642996	1.862125	0.062586	0.112035	1.084173	0	0.695731	4.464254	6.417332	4.019918
2456	CIDEC	45.16175	3.058986	0.425471	7.18965	6.50E-13	7.03E-12	6.505036	10.94824	11.82743	78.12444	79.14709	84.41828
13961	C2CD4A	4.761425	3.058798	1.51466	2.019462	0.043439	0.082718	0.542086	1.094824	1.391463	0	23.53022	2.009959
13412	HAS1	59.26352	3.058641	1.439484	2.124817	0.033602	0.066605	1.084173	1.642235	35.4823	156.2489	121.9293	39.1942
6092	LOC103243	19.32449	3.058129	0.741212	4.125848	3.69E-05	0.000161	1.084173	3.831883	7.653045	26.78552	53.47777	23.11453
6285	LOC103230	19.72825	3.056036	0.75738	4.035012	5.46E-05	0.000231	2.168345	1.642235	9.044508	44.64254	42.78221	18.08963
8826	CYP2A28	16.40761	3.055615	0.965334	3.165346	0.001549	0.004666	1.084173	0	9.740239	29.01765	38.50399	20.09959
17585	CEACAM16	1.791544	3.054067	2.177142	1.402787	0.16068	0.242916	0	1.094824	0	2.232127	6.417332	1.004979
11658	OLIG2	10.54447	3.053517	1.23161	2.47929	0.013164	0.03002	0	0	6.957314	29.01765	19.252	8.039836
12862	LOC103246	3.808616	3.053261	1.371584	2.226084	0.026009	0.053758	0.542086	0.547412	1.391463	8.928508	6.417332	5.024897
13923	SCGB1C1	4.029268	3.053142	1.506149	2.027119	0.04265	0.081438	0	0.547412	2.087194	6.696381	12.83466	2.009959
5783	LOC103219	31.98161	3.051583	0.712447	4.28324	1.84E-05	8.47E-05	1.626259	3.284471	16.00182	66.96381	74.86887	29.1444
11790	GYS2	5.689719	3.051069	1.244327	2.451984	0.014207	0.032035	0.542086	1.642235	1.391463	17.85702	10.69555	2.009959
9621	NPM2	15.39996	3.050777	1.032305	2.955305	0.003124	0.008631	0	1.094824	9.044508	17.85702	51.33866	13.06473
12923	GAPT	5.055875	3.050413	1.375462	2.217737	0.026573	0.054665	0.542086	0	2.782925	11.16063	12.83466	3.014938
4704	LOC119620	21.67263	3.050395	0.624863	4.8817	1.05E-06	5.94E-06	3.252518	3.284471	7.653045	33.4819	49.19955	33.16432
180	ADAMTS14	775.5171	3.048871	0.145857	20.90319	5.01E-97	7.40E-95	181.5989	143.9693	177.4115	1352.669	1274.91	1522.544
6298	CTNND2	15.0511	3.048817	0.75718	4.026544	5.66E-05	0.000239	2.168345	3.284471	4.174388	31.24978	36.36488	13.06473
10470	TMEM178A	6.662907	3.048808	1.108056	2.751493	0.005932	0.015063	1.626259	0.547412	2.087194	17.85702	12.83466	5.024897
8852	LOC103228	11.50257	3.048588	0.965893	3.156239	0.001598	0.0048	0	3.284471	4.174388	31.24978	19.252	11.05477
8120	GAS2L2	14.76387	3.048569	0.903951	3.372493	0.000745	0.002439	0.542086	2.189647	6.957314	15.62489	49.19955	14.06971
19174	LOC103235	0.987271	3.047564	2.634381	1.156843	0.247337	0.342721	0	0.547412	0	2.232127	2.139111	1.004979
19175	LOC103235	0.987271	3.047564	2.634381	1.156843	0.247337	0.342721	0	0.547412	0	2.232127	2.139111	1.004979
19176	LOC119625	0.987271	3.047564	2.634381	1.156843	0.247337	0.342721	0	0.547412	0	2.232127	2.139111	1.004979
19177	GJB7	0.987271	3.047564	2.634381	1.156843	0.247337	0.342721	0	0.547412	0	2.232127	2.139111	1.004979
19178	LOC119625	0.987271	3.047564	2.634381	1.156843	0.247337	0.342721	0	0.547412	0	2.232127	2.139111	1.004979
19181	PDC	0.987271	3.047564	2.634381	1.156843	0.247337	0.342721	0	0.547412	0	2.232127	2.139111	1.004979

19182	LOC11962(	0.987271	3.047564	2.634381	1.156843	0.247337	0.342721	0	0.547412	0	2.232127	2.139111	1.004979
19183	LOC10322;	0.987271	3.047564	2.634381	1.156843	0.247337	0.342721	0	0.547412	0	2.232127	2.139111	1.004979
19184	CCDC182	0.987271	3.047564	2.634381	1.156843	0.247337	0.342721	0	0.547412	0	2.232127	2.139111	1.004979
19185	LOC10322!	0.987271	3.047564	2.634381	1.156843	0.247337	0.342721	0	0.547412	0	2.232127	2.139111	1.004979
19186	LOC10323	0.987271	3.047564	2.634381	1.156843	0.247337	0.342721	0	0.547412	0	2.232127	2.139111	1.004979
19122	LOC11962!	1.011991	3.046997	2.616102	1.164709	0.244137	0.339313	0	0	0.695731	2.232127	2.139111	1.004979
19123	LOC11962;	1.011991	3.046997	2.616102	1.164709	0.244137	0.339313	0	0	0.695731	2.232127	2.139111	1.004979
19124	VENTX	1.011991	3.046997	2.616102	1.164709	0.244137	0.339313	0	0	0.695731	2.232127	2.139111	1.004979
19125	LOC10323(	1.011991	3.046997	2.616102	1.164709	0.244137	0.339313	0	0	0.695731	2.232127	2.139111	1.004979
19126	LOC10323:	1.011991	3.046997	2.616102	1.164709	0.244137	0.339313	0	0	0.695731	2.232127	2.139111	1.004979
19127	HRG	1.011991	3.046997	2.616102	1.164709	0.244137	0.339313	0	0	0.695731	2.232127	2.139111	1.004979
19128	LOC11962:	1.011991	3.046997	2.616102	1.164709	0.244137	0.339313	0	0	0.695731	2.232127	2.139111	1.004979
12207	LOC10323!	4.608806	3.046693	1.287903	2.365623	0.018	0.039201	1.084173	1.094824	0.695731	8.928508	12.83466	3.014938
10482	KRT84	6.664682	3.046521	1.108282	2.748869	0.00598	0.015167	0.542086	1.642235	2.087194	17.85702	12.83466	5.024897
4109	HECW1	54.80439	3.045445	0.575854	5.288571	1.23E-07	7.98E-07	3.794605	10.94824	20.87194	95.98146	154.016	43.21412
9305	CUNH4orf:	10.53876	3.045329	1.004634	3.031283	0.002435	0.006957	0.542086	2.737059	3.478657	26.78552	25.66933	4.019918
9750	MROH2B	10.00758	3.043021	1.043146	2.917156	0.003532	0.009632	0.542086	0.547412	5.565851	13.39276	29.94755	10.04979
10244	NUTM2F	10.06099	3.042549	1.085068	2.804018	0.005047	0.013098	0	1.094824	5.565851	17.85702	27.80844	8.039836
14558	CFAP299	2.81815	3.04187	1.58932	1.913944	0.055627	0.101583	0.542086	0.547412	0.695731	6.696381	6.417332	2.009959
11651	MAGEA4	10.42992	3.041127	1.225791	2.48095	0.013103	0.029899	0	0	6.957314	15.62489	29.94755	10.04979
7073	CPXM2	14.87093	3.040919	0.820999	3.703926	0.000212	0.000798	2.168345	3.831883	3.478657	26.78552	44.92132	8.039836
8577	TAC3	10.72726	3.038929	0.939556	3.23443	0.001219	0.003778	1.084173	1.094824	4.87012	29.01765	19.252	9.044815
12297	LOC10322	8.331461	3.038547	1.295752	2.345007	0.019027	0.041134	0	0	5.565851	24.5534	12.83466	7.034856
15137	HTR2C	2.803535	3.038477	1.680491	1.808089	0.070593	0.123981	0	1.094824	0.695731	4.464254	8.556443	2.009959
16249	MS4A15	2.770755	3.038186	1.878443	1.617396	0.105793	0.173088	1.084173	0	0.695731	0	12.83466	2.009959
8026	GAD1	14.79629	3.038129	0.89248	3.404141	0.000664	0.002198	1.626259	0.547412	7.653045	20.08914	42.78221	16.07967
9957	IL12B	7.630165	3.036398	1.056791	2.873224	0.004063	0.010848	0.542086	1.642235	2.782925	13.39276	21.39111	6.029877
12514	LOC10322:	4.79049	3.035891	1.320632	2.298817	0.021515	0.045708	0	1.094824	2.087194	6.696381	12.83466	6.029877
19817	LOC10322{	0.970881	3.034603	2.887543	1.050929	0.293291	0.393378	0.542086	0	0	0	4.278221	1.004979
19818	LOC11962(	0.970881	3.034603	2.887543	1.050929	0.293291	0.393378	0.542086	0	0	0	4.278221	1.004979
19819	LOC11961!	0.970881	3.034603	2.887543	1.050929	0.293291	0.393378	0.542086	0	0	0	4.278221	1.004979
19820	LOC11962(	0.970881	3.034603	2.887543	1.050929	0.293291	0.393378	0.542086	0	0	0	4.278221	1.004979
19811	LOC11962(	0.971769	3.034588	2.886707	1.051228	0.293154	0.393293	0	0.547412	0	0	4.278221	1.004979
19812	LOC10323(	0.971769	3.034588	2.886707	1.051228	0.293154	0.393293	0	0.547412	0	0	4.278221	1.004979
19813	PHGR1	0.971769	3.034588	2.886707	1.051228	0.293154	0.393293	0	0.547412	0	0	4.278221	1.004979
19814	LOC10322!	0.971769	3.034588	2.886707	1.051228	0.293154	0.393293	0	0.547412	0	0	4.278221	1.004979
19815	LOC10322;	0.971769	3.034588	2.886707	1.051228	0.293154	0.393293	0	0.547412	0	0	4.278221	1.004979
19753	LOC11962:	0.996489	3.03418	2.863886	1.059462	0.289389	0.389303	0	0	0.695731	0	4.278221	1.004979
19754	LOC11962!	0.996489	3.03418	2.863886	1.059462	0.289389	0.389303	0	0	0.695731	0	4.278221	1.004979
19755	LOC10324{	0.996489	3.03418	2.863886	1.059462	0.289389	0.389303	0	0	0.695731	0	4.278221	1.004979
19756	LOC10322!	0.996489	3.03418	2.863886	1.059462	0.289389	0.389303	0	0	0.695731	0	4.278221	1.004979
19757	SRARP	0.996489	3.03418	2.863886	1.059462	0.289389	0.389303	0	0	0.695731	0	4.278221	1.004979
19758	LOC10324;	0.996489	3.03418	2.863886	1.059462	0.289389	0.389303	0	0	0.695731	0	4.278221	1.004979
19759	CUNH18or	0.996489	3.03418	2.863886	1.059462	0.289389	0.389303	0	0	0.695731	0	4.278221	1.004979
19760	LOC11962:	0.996489	3.03418	2.863886	1.059462	0.289389	0.389303	0	0	0.695731	0	4.278221	1.004979
19761	LOC11962;	0.996489	3.03418	2.863886	1.059462	0.289389	0.389303	0	0	0.695731	0	4.278221	1.004979
19762	LOC10323:	0.996489	3.03418	2.863886	1.059462	0.289389	0.389303	0	0	0.695731	0	4.278221	1.004979
12920	SLC6A1	7.278984	3.032678	1.367322	2.21797	0.026557	0.054645	0	0	4.87012	13.39276	21.39111	4.019918
8300	ANKRD45	22.34165	3.031914	0.915202	3.312836	0.000924	0.002958	0	1.642235	13.2189	46.87467	49.19955	23.11453
12645	AMPD1	4.827518	3.031718	1.334652	2.271543	0.023114	0.048594	0	1.094824	2.087194	8.928508	12.83466	4.019918
16785	LOC10323!	2.793167	3.031152	1.983509	1.528177	0.126469	0.200304	0.542086	0.547412	0.695731	0	14.97377	0
14104	KLK3	33.40478	3.031012	1.52047	1.99347	0.04621	0.087102	0	3.831883	18.08902	60.26743	94.12087	24.11951
7170	MAP6	13.87497	3.030664	0.824838	3.674253	0.000239	0.000884	3.794605	1.642235	3.478657	26.78552	38.50399	9.044815
17897	EPS8L3	24.37034	3.030627	2.225285	1.361905	0.173228	0.25732	0	0	16.00182	58.0353	47.06043	25.12449
8604	LOC11962:	11.14036	3.030599	0.939015	3.227423	0.001249	0.00386	3.252518	1.094824	2.782925	29.01765	25.66933	5.024897
17411	SIT1	14.80298	3.030358	2.121943	1.428105	0.153262	0.234016	0	0	9.740239	37.94616	32.08666	9.044815
15379	MUC5AC	21.96768	3.029769	1.717817	1.763732	0.077777	0.13445	0	0.547412	13.91463	44.64254	55.61688	17.08465
17077	LOC11961!	1.986955	3.027989	2.039792	1.48446	0.137687	0.214347	0	0	1.391463	2.232127	4.278221	4.019918
6962	INSRR	19.72693	3.027423	0.806916	3.751842	0.000176	0.00067	1.084173	1.642235	10.43597	40.17829	44.92132	20.09959
4356	FEZF1	22.63199	3.026603	0.592419	5.108892	3.24E-07	1.98E-06	2.710432	6.02153	6.261582	37.94616	40.6431	42.20914
10868	LOC10322(	9.0677	3.0252	1.139033	2.655937	0.007909	0.019345	0	0.547412	5.565851	17.85702	21.39111	9.044815
10869	LOC10324:	9.0677	3.0252	1.139033	2.655937	0.007909	0.019345	0	0.547412	5.565851	17.85702	21.39111	9.044815
7691	SYNE4	11.29449	3.024661	0.865109	3.496277	0.000472	0.001631	2.168345	1.094824	4.174388	29.01765	19.252	12.05975
654	SPOCK1	177.4838	3.024096	0.23104	13.08904	3.80E-39	1.55E-37	32.52518	47.07742	36.87376	303.5693	327.2839	317.5735
11884	LOC11962(	5.156286	3.024015	1.245092	2.428748	0.015151	0.033894	1.084173	2.189647	0	8.928508	10.69555	8.039836
9398	ANKLE1	11.82986	3.02357	1.005351	3.007477	0.002634	0.007452	0.542086	1.094824	6.261582	13.39276	40.6431	9.044815
5014	SIGLEC9	28.23423	3.023546	0.64601	4.680341	2.86E-06	1.52E-05	1.084173	6.02153	11.82743	46.87467	51.33866	52.25893
8196	LOC11962;	11.48414	3.022296	0.902746	3.34789	0.000814	0.002641	1.626259	1.094824	4.87012	22.32127	29.94755	9.044815
2724	LOC10323(	42.77277	3.020986	0.443475	6.812081	9.62E-12	9.39E-11	9.215468	6.02153	13.2189	73.66019	79.14709	75.37346
8787	LOC10322!	13.83143	3.020733	0.950629	3.177615	0.001485	0.004493	1.084173	0.547412	7.653045	17.85702	42.78221	13.06473
14553	GIMAP4	4.041943	3.020086	1.576163	1.916101	0.055352	0.101116	0	0	2.782925	11.16063	4.278221	6.029877
16374	LOC10322!	2.608656	3.019502	1.895105	1.593316	0.111089	0.180366	1.626259	0	0	4.464254	8.556443	1.004979
17048	NPHS2	2.023983	3.018701	2.03028	1.48684	0.137057	0.213704	0	0	1.391463	4.464254	4.278221	2.009959
17049	LOC10323:	2.023983	3.018701	2.03028	1.48684	0.137057	0.213704	0	0	1.391463	4.464254	4.278221	2.009959

17050	SAGE1	2.023983	3.018701	2.03028	1.48684	0.137057	0.213704	0	0	1.391463	4.464254	4.278221	2.009959
15500	KCNK16	2.998946	3.018402	1.730298	1.74444	0.081082	0.139069	0	0	2.087194	4.464254	6.417332	5.024897
6185	LOC11962:	20.74349	3.017666	0.739055	4.083141	4.44E-05	0.000191	2.710432	3.284471	7.653045	35.71403	62.03421	13.06473
6474	LOC10323!	16.89166	3.017255	0.763725	3.950709	7.79E-05	0.00032	1.084173	3.284471	6.957314	44.64254	19.252	26.12947
7137	RASGRF2	12.04199	3.017107	0.818894	3.684366	0.000229	0.000854	1.084173	2.737059	4.174388	26.78552	21.39111	16.07967
11058	ALAS2	7.832487	3.016897	1.154373	2.61345	0.008963	0.021549	0	1.094824	4.174388	11.16063	23.53022	7.034856
18168	LY6D	2.076514	3.016744	2.304052	1.309321	0.190426	0.278647	0	0	1.391463	8.928508	2.139111	0
3745	CACNA1E	62.11969	3.015718	0.538763	5.59749	2.17E-08	1.54E-07	7.589209	6.568942	27.13352	113.8385	158.2942	59.29379
2178	CFAP73	58.81647	3.015231	0.393984	7.65318	1.96E-14	2.39E-13	14.63633	10.40082	13.91463	73.66019	134.764	105.5228
9488	ESRRB	8.529761	3.015141	1.009404	2.987051	0.002817	0.007893	1.084173	1.094824	3.478657	26.78552	10.69555	8.039836
15554	FAM216B	3.035974	3.013834	1.736957	1.735123	0.082719	0.141384	0	0	2.087194	6.696381	6.417332	3.014938
16298	LOC11962!	4.153027	3.013489	1.875107	1.607103	0.108032	0.17622	0	0	2.782925	17.85702	4.278221	0
9414	TMEM174	9.382385	3.013102	1.003007	3.00407	0.002664	0.007523	2.168345	0	4.174388	15.62489	19.252	15.07469
12953	LOC10324{	4.650804	3.012927	1.361695	2.21263	0.026923	0.055258	0	1.642235	1.391463	11.16063	10.69555	3.014938
15269	LOC11962!	2.588018	3.012767	1.687868	1.784954	0.074269	0.12931	1.084173	0.547412	0	4.464254	6.417332	3.014938
8159	LOC10323{	15.82483	3.011676	0.895928	3.361516	0.000775	0.002526	0	4.926706	5.565851	22.32127	47.06043	15.07469
14967	RPH3A	42.88552	3.011541	1.636519	1.840212	0.065737	0.116765	0	2.737059	25.74206	66.96381	117.6511	44.2191
11900	PASD1	5.541344	3.011005	1.241713	2.424879	0.015313	0.034211	0.542086	1.094824	2.087194	2.232127	19.252	8.039836
12679	HSFX3	5.517087	3.010143	1.329139	2.264731	0.023529	0.049335	0	2.189647	1.391463	20.08914	6.417332	3.014938
13778	AQP8	3.812699	3.009869	1.466998	2.05172	0.040197	0.077561	0	0.547412	2.087194	8.928508	4.278221	7.034856
8490	LOC11962:	11.51834	3.009647	0.923727	3.258156	0.001121	0.003511	1.626259	0.547412	5.565851	26.78552	23.53022	11.05477
7100	CTSW	12.95919	3.009248	0.814213	3.6959	0.000219	0.00082	2.168345	1.642235	4.87012	15.62489	36.36488	17.08465
17439	MPO	15.6479	3.009192	2.118989	1.420107	0.155576	0.237169	0	0	10.43597	22.32127	47.06043	14.06971
7759	JPH4	24.80522	3.008327	0.865514	3.475768	0.000509	0.001745	0	2.189647	14.61036	44.64254	49.19955	38.18922
17243	FAM71F1	2.00848	3.007593	2.060317	1.459772	0.144353	0.222561	0	0	1.391463	2.232127	6.417332	2.009959
10306	LOC10322:	12.56699	3.006723	1.078833	2.787015	0.00532	0.013721	0	2.189647	6.261582	6.696381	49.19955	11.05477
18087	PDLIM3	2.061011	3.006679	2.270098	1.324471	0.185347	0.272415	0	0	1.391463	6.696381	4.278221	0
18088	LOC11962:	2.061011	3.006679	2.270098	1.324471	0.185347	0.272415	0	0	1.391463	6.696381	4.278221	0
15696	LOC11962!	3.020472	3.00649	1.750655	1.717351	0.085915	0.145518	0	0	2.087194	4.464254	8.556443	3.014938
5598	ELN	35.9882	3.006434	0.687242	4.374634	1.22E-05	5.78E-05	1.626259	3.831883	18.78475	69.19594	81.28621	41.20416
8608	LOC10323(	14.21	3.005557	0.931596	3.226247	0.001254	0.003874	0	3.284471	6.261582	17.85702	42.78221	15.07469
1827	WNT7B	71.56989	3.005147	0.358849	8.374395	5.55E-17	8.08E-16	12.46799	12.59047	22.95914	122.767	139.0422	119.5926
13878	RGS18	3.849727	3.004043	1.47718	2.033634	0.041989	0.080434	0	0.547412	2.087194	11.16063	4.278221	5.024897
8665	LOC11961!	12.17476	3.004032	0.935052	3.212688	0.001315	0.004035	1.084173	2.189647	4.87012	8.928508	44.92132	11.05477
11906	SLC13A5	5.412026	3.003736	1.239427	2.423488	0.015372	0.034325	0	2.189647	1.391463	11.16063	10.69555	7.034856
17395	FEV	17.66932	3.003201	2.099967	1.430118	0.152683	0.233348	0	0	11.82743	35.71403	36.36488	22.10955
13758	AFM	5.007426	3.001515	1.460065	2.05574	0.039808	0.076921	0	0	3.478657	6.696381	12.83466	7.034856
16053	LOC11962⁄	3.0575	3.001131	1.812805	1.655518	0.09782	0.161987	0	0	2.087194	6.696381	8.556443	1.004979
16054	IZUMO2	3.0575	3.001131	1.812805	1.655518	0.09782	0.161987	0	0	2.087194	6.696381	8.556443	1.004979
16953	LOC11962:	1.773213	2.999979	2.001293	1.499021	0.133868	0.209927	0	0.547412	0.695731	2.232127	2.139111	5.024897
15786	LOC10324{	2.610431	2.999104	1.75978	1.704249	0.088335	0.148763	0.542086	1.094824	0	4.464254	8.556443	1.004979
7966	CCDC141	10.89969	2.998075	0.876569	3.42024	0.000626	0.002088	2.710432	1.642235	2.782925	24.5534	25.66933	8.039836
18120	LOC10323:	2.045508	2.996267	2.274154	1.31753	0.187661	0.275329	0	0	1.391463	4.464254	6.417332	0
14977	CUNH11or	4.053988	2.995833	1.629373	1.838642	0.065968	0.117097	0	0	2.782925	6.696381	12.83466	2.009959
11178	SLC6A20	7.448054	2.994607	1.158028	2.585953	0.009711	0.023095	0	2.189647	2.782925	11.16063	23.53022	5.024897
13545	ACTL10	4.920271	2.994447	1.426098	2.099748	0.035751	0.070169	0	0.547412	2.782925	15.62489	8.556443	2.009959
11464	ASPG	5.415386	2.994092	1.186422	2.523632	0.011615	0.026935	1.626259	0.547412	1.391463	8.928508	14.97377	5.024897
10785	RPGRIP1	10.63576	2.993343	1.119734	2.673263	0.007512	0.018516	0	1.642235	5.565851	8.928508	40.6431	7.034856
10132	GRIA2	13.40786	2.992936	1.057275	2.830803	0.004643	0.01218	0	2.737059	6.261582	26.78552	40.6431	4.019918
15119	LOC10321{	3.409568	2.99266	1.65348	1.809916	0.070309	0.123629	0	2.189647	0	6.696381	8.556443	3.014938
8559	TSSK1B	8.709267	2.991743	0.923683	3.238929	0.0012	0.003727	2.710432	1.094824	2.087194	11.16063	17.11289	18.08963
12409	PLBD1	5.864229	2.991405	1.290016	2.318891	0.020401	0.043707	0	0.547412	3.478657	11.16063	14.97377	5.024897
8011	LOC11962:	12.69009	2.989981	0.877128	3.408831	0.000652	0.002165	3.252518	1.642235	3.478657	20.08914	40.6431	7.034856
9515	MYBPC3	16.84137	2.989472	1.003084	2.980282	0.00288	0.008046	0	1.094824	10.43597	20.08914	51.33866	18.08963
13978	LOC11962:	3.855749	2.988769	1.48253	2.015992	0.043801	0.083306	0	0.547412	2.087194	8.928508	8.556443	3.014938
8260	GABRE	11.30868	2.988459	0.898464	3.326188	0.00088	0.002834	1.084173	1.094824	5.565851	26.78552	19.252	14.06971
10041	LOC11962:	9.719091	2.988445	1.047428	2.853127	0.004329	0.011462	0.542086	0.547412	5.565851	20.08914	23.53022	8.039836
9478	IL12RB1	10.41998	2.988268	0.999042	2.991134	0.002779	0.007796	1.626259	0	5.565851	17.85702	21.39111	16.07967
10591	CCDC27	8.686462	2.988239	1.097052	2.72388	0.006452	0.016194	0	1.094824	4.87012	17.85702	19.252	9.044815
12716	PRPH2	4.632473	2.987875	1.325422	2.254282	0.024178	0.050544	0	1.094824	2.087194	11.16063	6.417332	7.034856
18108	LOC10323⁄	1.992977	2.987348	2.263611	1.319727	0.186926	0.274444	0	0	1.391463	0	8.556443	2.009959
13255	CPN1	4.799707	2.986691	1.384753	2.15684	0.031018	0.06221	0	0.547412	2.782925	4.464254	14.97377	6.029877
8723	LOC11962;	8.952332	2.986157	0.934397	3.195811	0.001394	0.00425	1.626259	1.642235	2.782925	8.928508	25.66933	13.06473
10582	KCNJ16	15.06773	2.985836	1.095053	2.726658	0.006398	0.016073	0	0.547412	9.740239	35.71403	36.36488	8.039836
4762	KEL	39.56747	2.985768	0.617318	4.836679	1.32E-06	7.37E-06	4.336691	4.379295	18.08902	75.89232	100.5382	34.1693
1904	RRAD	62.08757	2.985184	0.364396	8.192151	2.57E-16	3.58E-15	11.38381	13.13788	17.39328	104.91	126.2075	99.49297
10410	NR2E3	8.691597	2.984999	1.080029	2.763815	0.005713	0.01459	0.542086	0.547412	4.87012	15.62489	23.53022	7.034856
18221	GPR173	27.82882	2.984345	2.298707	1.298271	0.194194	0.283335	0	0	18.78475	51.33892	72.72976	24.11951
13759	TMPRSS9	5.283498	2.984129	1.45163	2.05571	0.03981	0.076921	0.542086	2.189647	0.695731	11.16063	17.11289	0
16105	LRRC55	46.95857	2.983833	1.8128	1.64598	0.099768	0.16469	0	1.094824	30.61218	78.12444	117.6511	54.26889
16876	LOC10324:	1.810241	2.982849	1.975077	1.510245	0.130981	0.206336	0	0.547412	0.695731	4.464254	2.139111	3.014938
10581	CD300E	13.98771	2.982336	1.093644	2.726972	0.006392	0.01606	0	0.547412	9.044508	26.78552	38.50399	9.044815
15239	PLPPR1	2.84287	2.981121	1.666016	1.789371	0.073555	0.128327	0.542086	0	1.391463	6.696381	6.417332	2.009959
13238	LOC11962!	4.374164	2.980889	1.380347	2.159522	0.03081	0.061873	0.542086	1.642235	0.695731	2.232127	17.11289	4.019918

16908	SERPINA12	2.989466	2.980249	1.980752	1.504605	0.132426	0.208217	0	0	2.087194	0	12.83466	3.014938
12727	SLC52A3	4.705642	2.979886	1.323178	2.252067	0.024318	0.050797	0.542086	0.547412	2.087194	15.62489	6.417332	3.014938
3126	LOC119621	661.7492	2.979793	0.477045	6.246352	4.20E-10	3.57E-09	149.0738	114.4091	182.9774	1285.705	1668.506	569.8234
12769	LRRC38	4.601468	2.979792	1.326044	2.247129	0.024632	0.051286	0	1.094824	2.087194	6.696381	10.69555	7.034856
3394	CIDEB	50.37414	2.976997	0.500425	5.94894	2.70E-09	2.11E-08	8.673382	20.25424	4.87012	104.91	62.03421	101.5029
16909	TRNAM-CA	1.793851	2.976957	1.978648	1.504541	0.132442	0.208231	0.542086	0	0.695731	2.232127	4.278221	3.014938
11296	LOC119628	7.650508	2.976486	1.162782	2.559798	0.010473	0.024649	4.336691	0	0.695731	17.85702	14.97377	8.039836
9328	LOC119619	7.952127	2.975726	0.984006	3.024092	0.002494	0.007107	2.168345	0.547412	2.782925	11.16063	14.97377	16.07967
13597	LOC119621	4.225793	2.975045	1.423354	2.090165	0.036603	0.071567	2.168345	0.547412	0	8.928508	10.69555	3.014938
12824	IL16	4.638496	2.974896	1.331747	2.23383	0.025494	0.052843	0	1.094824	2.087194	8.928508	10.69555	5.024897
11977	CWH43	6.578943	2.974635	1.234208	2.410157	0.015946	0.035394	0	1.094824	3.478657	4.464254	21.39111	9.044815
8918	KLK6	12.33335	2.973762	0.94726	3.139332	0.001693	0.005048	1.084173	0.547412	6.957314	33.4819	12.83466	19.09461
16922	LOC103247	1.794738	2.973125	1.978267	1.502894	0.132866	0.208737	0	0.547412	0.695731	2.232127	4.278221	3.014938
3706	LOC103247	27.56647	2.972286	0.527496	5.634706	1.75E-08	1.26E-07	5.420864	8.211177	4.87012	51.33892	51.33866	44.2191
8701	CSF3	11.18015	2.972092	0.928171	3.202097	0.001364	0.004169	1.626259	1.094824	4.87012	26.78552	25.66933	7.034856
15350	LOC103235	2.828255	2.971798	1.678441	1.77057	0.076632	0.132721	0	0.547412	1.391463	4.464254	8.556443	2.009959
13916	ADAMTS19	3.642007	2.968233	1.463828	2.02772	0.042589	0.081361	0	1.094824	1.391463	8.928508	6.417332	4.019918
6506	DSCAML1	15.71239	2.967218	0.753429	3.938283	8.21E-05	0.000335	3.252518	4.379295	2.782925	29.01765	42.78221	12.05975
15546	IGF2	29.10237	2.967005	1.708945	1.736162	0.082535	0.141142	0	1.094824	18.78475	51.33892	81.28621	22.10955
2996	DCHS2	43.59887	2.966699	0.461935	6.422332	1.34E-10	1.19E-09	8.131296	6.02153	16.00182	82.5887	68.45154	80.39836
7637	LOC103222	13.33545	2.965367	0.844159	3.512807	0.000443	0.001543	3.794605	1.642235	3.478657	24.5534	38.50399	8.039836
11517	CFD	5.403965	2.965081	1.181433	2.509734	0.012082	0.02789	1.084173	0.547412	2.087194	6.696381	14.97377	7.034856
15668	MAGEC3	2.812752	2.964922	1.72124	1.722549	0.08497	0.144177	0	0.547412	1.391463	2.232127	10.69555	2.009959
17232	AGR3	1.847269	2.964075	2.026488	1.462666	0.143559	0.221478	0	0.547412	0.695731	6.696381	2.139111	1.004979
14288	SPZ1	3.573974	2.963766	1.509027	1.964025	0.049527	0.092153	0	1.094824	1.391463	2.232127	10.69555	6.029877
9110	PRR29	15.23645	2.962583	0.961735	3.080456	0.002067	0.006031	2.168345	0	8.348776	33.4819	36.36488	11.05477
16524	LY86	2.84978	2.962229	1.88606	1.570591	0.116278	0.187076	0	0.547412	1.391463	4.464254	10.69555	0
10641	PLPPR3	7.324403	2.961928	1.09371	2.708148	0.006766	0.016904	1.626259	0.547412	2.782925	17.85702	17.11289	4.019918
13770	LOC119624	3.678148	2.961458	1.442672	2.05276	0.040096	0.077411	0.542086	0.547412	1.391463	11.16063	6.417332	2.009959
8825	LOC103232	12.22812	2.961431	0.935455	3.165765	0.001547	0.00466	0.542086	1.642235	6.261582	33.4819	21.39111	10.04979
11834	LOC103243	6.643261	2.96102	1.213093	2.440885	0.014651	0.032914	0.542086	0	4.174388	13.39276	10.69555	11.05477
4315	MAFA	27.91606	2.960123	0.575499	5.143578	2.70E-07	1.66E-06	4.336691	3.831883	11.1317	49.10679	59.8951	39.1942
7584	MATN4	12.42259	2.959812	0.838176	3.531253	0.000414	0.00145	0.542086	3.831883	4.174388	20.08914	27.80844	18.08963
9601	LOC119620	9.347333	2.958914	0.999396	2.960701	0.003069	0.008499	0.542086	1.094824	4.87012	24.5534	14.97377	10.04979
12869	ITIH2	6.894918	2.958469	1.329829	2.224699	0.026101	0.053921	0	0	4.87012	15.62489	12.83466	8.039836
17720	MCCD1	1.779236	2.958379	2.141217	1.381634	0.167084	0.250673	0	0.547412	0.695731	0	6.417332	3.014938
15214	VSTM2A	27.68416	2.956361	1.648272	1.793612	0.072875	0.127342	0	1.642235	17.39328	42.41041	85.56443	19.09461
7651	FRMD7	14.37216	2.956006	0.842181	3.509942	0.000448	0.001557	3.794605	1.094824	4.87012	26.78552	40.6431	9.044815
26751	LOC119624	0.334993	2.955864	4.056412	0.728689	0.466192	NA	0	0	0	0	0	2.009959
26824	TRNAF-GA	0.334993	2.955864	4.056412	0.728689	0.466192	NA	0	0	0	0	0	2.009959
26867	IFNG	0.334993	2.955864	4.056412	0.728689	0.466192	NA	0	0	0	0	0	2.009959
26885	LOC103238	0.334993	2.955864	4.056412	0.728689	0.466192	NA	0	0	0	0	0	2.009959
26890	LOC119619	0.334993	2.955864	4.056412	0.728689	0.466192	NA	0	0	0	0	0	2.009959
26894	LOC119623	0.334993	2.955864	4.056412	0.728689	0.466192	NA	0	0	0	0	0	2.009959
26897	LOC103238	0.334993	2.955864	4.056412	0.728689	0.466192	NA	0	0	0	0	0	2.009959
26923	LOC103223	0.334993	2.955864	4.056412	0.728689	0.466192	NA	0	0	0	0	0	2.009959
26976	LOC119624	0.334993	2.955864	4.056412	0.728689	0.466192	NA	0	0	0	0	0	2.009959
27054	LOC103235	0.334993	2.955864	4.056412	0.728689	0.466192	NA	0	0	0	0	0	2.009959
27172	MOS	0.334993	2.955864	4.056412	0.728689	0.466192	NA	0	0	0	0	0	2.009959
27187	LOC119625	0.334993	2.955864	4.056412	0.728689	0.466192	NA	0	0	0	0	0	2.009959
27240	LOC103218	0.334993	2.955864	4.056412	0.728689	0.466192	NA	0	0	0	0	0	2.009959
27319	LOC119625	0.334993	2.955864	4.056412	0.728689	0.466192	NA	0	0	0	0	0	2.009959
27410	LOC119625	0.334993	2.955864	4.056412	0.728689	0.466192	NA	0	0	0	0	0	2.009959
27478	LOC119626	0.334993	2.955864	4.056412	0.728689	0.466192	NA	0	0	0	0	0	2.009959
27530	LOC103226	0.334993	2.955864	4.056412	0.728689	0.466192	NA	0	0	0	0	0	2.009959
27580	LOC119626	0.334993	2.955864	4.056412	0.728689	0.466192	NA	0	0	0	0	0	2.009959
27676	LOC119626	0.334993	2.955864	4.056412	0.728689	0.466192	NA	0	0	0	0	0	2.009959
27711	LOC119627	0.334993	2.955864	4.056412	0.728689	0.466192	NA	0	0	0	0	0	2.009959
27731	LOC119627	0.334993	2.955864	4.056412	0.728689	0.466192	NA	0	0	0	0	0	2.009959
27747	LOC119627	0.334993	2.955864	4.056412	0.728689	0.466192	NA	0	0	0	0	0	2.009959
27782	LOC119627	0.334993	2.955864	4.056412	0.728689	0.466192	NA	0	0	0	0	0	2.009959
27847	LOC119627	0.334993	2.955864	4.056412	0.728689	0.466192	NA	0	0	0	0	0	2.009959
27915	LOC119628	0.334993	2.955864	4.056412	0.728689	0.466192	NA	0	0	0	0	0	2.009959
27974	LOC119628	0.334993	2.955864	4.056412	0.728689	0.466192	NA	0	0	0	0	0	2.009959
27998	LOC119618	0.334993	2.955864	4.056412	0.728689	0.466192	NA	0	0	0	0	0	2.009959
28021	LOC119618	0.334993	2.955864	4.056412	0.728689	0.466192	NA	0	0	0	0	0	2.009959
28057	CIDEA	0.334993	2.955864	4.056412	0.728689	0.466192	NA	0	0	0	0	0	2.009959
28158	LOC119618	0.334993	2.955864	4.056412	0.728689	0.466192	NA	0	0	0	0	0	2.009959
28250	OTOR	0.334993	2.955864	4.056412	0.728689	0.466192	NA	0	0	0	0	0	2.009959
28343	LOC119619	0.334993	2.955864	4.056412	0.728689	0.466192	NA	0	0	0	0	0	2.009959
28368	LOC119619	0.334993	2.955864	4.056412	0.728689	0.466192	NA	0	0	0	0	0	2.009959
28394	LOC119619	0.334993	2.955864	4.056412	0.728689	0.466192	NA	0	0	0	0	0	2.009959
28428	LOC103230	0.334993	2.955864	4.056412	0.728689	0.466192	NA	0	0	0	0	0	2.009959
28449	LOC119620	0.334993	2.955864	4.056412	0.728689	0.466192	NA	0	0	0	0	0	2.009959

28503	LOC11962(	0.334993	2.955864	4.056412	0.728689	0.466192	NA	0	0	0	0	0	2.009959
28595	LOC11962(	0.334993	2.955864	4.056412	0.728689	0.466192	NA	0	0	0	0	0	2.009959
28761	LOC11962:	0.334993	2.955864	4.056412	0.728689	0.466192	NA	0	0	0	0	0	2.009959
28767	LOC11962:	0.334993	2.955864	4.056412	0.728689	0.466192	NA	0	0	0	0	0	2.009959
28786	RTL3	0.334993	2.955864	4.056412	0.728689	0.466192	NA	0	0	0	0	0	2.009959
28795	LOC10323(	0.334993	2.955864	4.056412	0.728689	0.466192	NA	0	0	0	0	0	2.009959
28804	LOC10322ε	0.334993	2.955864	4.056412	0.728689	0.466192	NA	0	0	0	0	0	2.009959
28853	LOC11962:	0.334993	2.955864	4.056412	0.728689	0.466192	NA	0	0	0	0	0	2.009959
28921	LOC11962:	0.334993	2.955864	4.056412	0.728689	0.466192	NA	0	0	0	0	0	2.009959
28946	LOC11962:	0.334993	2.955864	4.056412	0.728689	0.466192	NA	0	0	0	0	0	2.009959
28992	LOC11962.	0.334993	2.955864	4.056412	0.728689	0.466192	NA	0	0	0	0	0	2.009959
29102	LOC11961ε	0.334993	2.955864	4.056412	0.728689	0.466192	NA	0	0	0	0	0	2.009959
29107	LOC11961ε	0.334993	2.955864	4.056412	0.728689	0.466192	NA	0	0	0	0	0	2.009959
29168	LOC11961ε	0.334993	2.955864	4.056412	0.728689	0.466192	NA	0	0	0	0	0	2.009959
29427	LOC11961ε	0.334993	2.955864	4.056412	0.728689	0.466192	NA	0	0	0	0	0	2.009959
29512	LOC10324!	0.334993	2.955864	4.056412	0.728689	0.466192	NA	0	0	0	0	0	2.009959
29574	LOC11962:	0.334993	2.955864	4.056412	0.728689	0.466192	NA	0	0	0	0	0	2.009959
29592	DEFB109B	0.334993	2.955864	4.056412	0.728689	0.466192	NA	0	0	0	0	0	2.009959
11911	LOC10323(	10.9179	2.955815	1.220234	2.422334	0.015421	0.03442	0	0	7.653045	17.85702	29.94755	10.04979
16778	LDHAL6B	2.834278	2.955716	1.932978	1.5291	0.12624	0.200029	0	0.547412	1.391463	2.232127	12.83466	0
6777	GJD3	12.25783	2.955271	0.772561	3.82529	0.000131	0.000512	2.710432	2.189647	3.478657	24.5534	23.53022	17.08465
14545	LOC11962:	5.994011	2.9539	1.540814	1.917103	0.055225	0.100939	0	0	4.174388	20.08914	10.69555	1.004979
8893	EGR4	10.82086	2.953642	0.938763	3.146313	0.001653	0.004943	1.626259	1.642235	4.174388	8.928508	38.50399	10.04979
17163	LOC11962ε	1.831766	2.952956	2.005268	1.472599	0.140859	0.218163	0	0.547412	0.695731	4.464254	4.278221	1.004979
17165	LOC10322!	1.831766	2.952956	2.005268	1.472599	0.140859	0.218163	0	0.547412	0.695731	4.464254	4.278221	1.004979
3683	LOC10324(	33.69807	2.952468	0.521357	5.663041	1.49E-08	1.07E-07	6.505036	4.926706	11.82743	80.35657	51.33866	47.23404
9900	LOC11962ʌ	8.318951	2.952288	1.023397	2.884791	0.003917	0.010518	1.084173	0.547412	4.174388	20.08914	14.97377	9.044815
8001	CCR1	10.77902	2.951524	0.865158	3.411543	0.000646	0.002146	2.168345	4.379295	0.695731	20.08914	19.252	18.08963
10688	LOC10324:	10.51797	2.950739	1.094879	2.695036	0.007038	0.017506	0	1.094824	6.261582	15.62489	32.08666	8.039836
12838	SI	5.448791	2.949788	1.322108	2.231125	0.025673	0.053163	0	1.642235	2.087194	6.696381	19.252	3.014938
3261	PRRG2	37.48723	2.948411	0.484384	6.086929	1.15E-09	9.38E-09	9.757555	8.758589	6.957314	95.98146	49.19955	54.26889
11532	NHLRC4	6.436429	2.946153	1.175123	2.507102	0.012173	0.02806	0	1.094824	3.478657	11.16063	12.83466	10.04979
12492	NEUROD6	5.638179	2.945952	1.279373	2.302652	0.021298	0.045327	0	0.547412	3.478657	8.928508	12.83466	8.039836
17301	LOC10324ε	1.816264	2.943671	2.032704	1.448155	0.147574	0.226751	0	0.547412	0.695731	2.232127	6.417332	1.004979
17302	LOC11961!	1.816264	2.943671	2.032704	1.448155	0.147574	0.226751	0	0.547412	0.695731	2.232127	6.417332	1.004979
8885	CYBB	13.18129	2.941458	0.934365	3.148082	0.001643	0.004917	0.542086	1.094824	7.653045	26.78552	29.94755	13.06473
13452	CER1	6.795879	2.939923	1.388775	2.116918	0.034267	0.067721	0	0	4.87012	4.464254	21.39111	10.04979
14265	LOC10324!	4.876958	2.939104	1.494277	1.966907	0.049194	0.091674	0	0	3.478657	8.928508	12.83466	4.019918
14266	TGM6	4.876958	2.939104	1.494277	1.966907	0.049194	0.091674	0	0	3.478657	8.928508	12.83466	4.019918
123	ARHGAP42	1373.527	2.938941	0.125809	23.36042	1.08E-120	2.33E-118	313.3259	355.8177	281.0755	2247.752	2483.507	2559.683
7842	C2	12.76843	2.93789	0.850228	3.455414	0.000549	0.001863	1.084173	1.642235	6.261582	29.01765	23.53022	15.07469
7348	NAALAD2	15.34838	2.937468	0.812287	3.616295	0.000299	0.001081	3.252518	0.547412	6.957314	29.01765	34.22577	18.08963
11986	ZNF541	11.83043	2.937416	1.219374	2.408955	0.015998	0.035486	0	0	8.348776	26.78552	27.80844	8.039836
7791	LOC10324:	13.40217	2.935479	0.846402	3.468185	0.000524	0.001788	2.710432	1.642235	4.87012	26.78552	36.36488	8.039836
8032	TIGIT	12.97663	2.935046	0.862372	3.403457	0.000665	0.002202	3.252518	2.737059	2.782925	24.5534	38.50399	6.029877
13732	OLR1	4.319434	2.934174	1.421496	2.064145	0.039004	0.075511	1.626259	0.547412	0.695731	15.62489	6.417332	1.004979
11598	LOC10324;	7.351785	2.933255	1.175645	2.495017	0.012595	0.028871	0	1.642235	3.478657	17.85702	17.11289	4.019918
15745	MUC2	30.50634	2.933015	1.715989	1.709227	0.087409	0.147588	0	1.094824	20.17621	69.19594	68.45154	24.11951
12149	ATCAY	9.763396	2.932353	1.234241	2.375836	0.017509	0.038315	0	0	6.957314	22.32127	19.252	10.04979
11391	TC2N	9.592704	2.930295	1.1541	2.539031	0.011116	0.025943	0	0.547412	6.261582	22.32127	21.39111	7.034856
954	LOC11962:	144.6374	2.929797	0.261921	11.18579	4.79E-29	1.33E-27	30.35684	29.01283	42.43961	234.3733	233.1631	298.4789
6437	ATP12A	15.70187	2.929339	0.738661	3.965743	7.32E-05	0.000302	4.336691	1.094824	5.565851	29.01765	32.08666	22.10955
10620	LOC10321!	10.2331	2.928543	1.078827	2.714562	0.006636	0.016613	1.626259	0	5.565851	26.78552	21.39111	6.029877
13594	PHOX2B	4.700244	2.927744	1.400544	2.090434	0.036579	0.071534	0	0.547412	2.782925	11.16063	10.69555	3.014938
11689	LOC11962ʌ	7.32078	2.927659	1.183636	2.473444	0.013382	0.030435	0	1.642235	3.478657	13.39276	21.39111	4.019918
8996	LOC11962;	11.9288	2.927425	0.93896	3.11773	0.001822	0.005386	1.626259	0.547412	6.261582	17.85702	34.22577	11.05477
12854	LOC11962(	7.770418	2.926787	1.313787	2.227748	0.025897	0.053561	0	0	5.565851	22.32127	10.69555	8.039836
10971	LOC10324:	6.71843	2.926163	1.110392	2.635252	0.008407	0.020373	2.710432	1.094824	0.695731	20.08914	10.69555	5.024897
6402	GPM6B	13.62394	2.926097	0.735486	3.978454	6.94E-05	0.000288	3.252518	2.737059	3.478657	20.08914	32.08666	20.09959
7577	TBX4	13.41751	2.925479	0.827871	3.53374	0.00041	0.001438	1.084173	2.737059	5.565851	31.24978	27.80844	12.05975
7115	HTR1F	13.82122	2.925274	0.792661	3.690448	0.000224	0.000836	1.626259	4.379295	3.478657	35.71403	25.66933	12.05975
17739	LOC10323:	2.474827	2.92489	2.122511	1.378033	0.168193	0.252067	0	1.642235	0	8.928508	4.278221	0
4056	LOC11962.	31.45992	2.924823	0.549012	5.327427	9.96E-08	6.53E-07	3.252518	6.568942	12.52316	49.10679	62.03421	55.27387
8720	RHOJ	10.10443	2.92464	0.914607	3.197702	0.001385	0.004223	3.794605	1.642235	1.391463	20.08914	25.66933	8.039836
18502	PAX1	31.7977	2.924019	2.335578	1.251947	0.210589	0.30259	0	0	22.2634	62.49956	74.86887	31.15436
15212	LOC11962.	4.112602	2.923764	1.62956	1.794204	0.072781	0.127194	1.084173	1.642235	0	13.39276	8.556443	0
13185	LOC11962ε	4.463202	2.923628	1.345564	2.172789	0.029796	0.060078	1.084173	0	2.087194	11.16063	6.417332	6.029877
18807	LOC11962ʌ	1.563719	2.922198	2.402317	1.216408	0.223829	0.316383	1.084173	0	0	0	4.278221	4.019918
153	MGST2	682.4327	2.920929	0.133417	21.89315	3.02E-106	5.25E-104	145.8212	172.4347	159.3225	1232.134	1182.928	1201.955
8777	CUNH2orf;	12.88119	2.920246	0.918334	3.17994	0.001473	0.004462	0.542086	1.094824	7.653045	20.08914	27.80844	20.09959
16072	LOC10322:	2.846952	2.919674	1.766643	1.652668	0.098399	0.162762	0	0	2.087194	6.696381	4.278221	4.019918
16073	LOC11961!	2.846952	2.919674	1.766643	1.652668	0.098399	0.162762	0	0	2.087194	6.696381	4.278221	4.019918
10292	BSX	9.11269	2.919598	1.046394	2.790153	0.005268	0.013609	0	1.642235	4.87012	17.85702	19.252	11.05477
58	PIK3R3	3584.312	2.919048	0.101902	28.6457	1.81E-180	8.31E-178	831.0184	900.4924	779.9149	6249.956	5998.066	6746.427

7649	ACSM2B	36.26781	2.918786	0.831544	3.510081	0.000448	0.001557	1.084173	1.642235	22.95914	80.35657	83.42532	28.13943
18132	LOC11962:	1.564606	2.917495	2.2171	1.315906	0.188206	0.275946	0.542086	0.547412	0	0	4.278221	4.019918
2179	ZNF469	62.7763	2.916671	0.381159	7.652104	1.98E-14	2.41E-13	13.01007	10.40082	20.87194	113.8385	124.0684	94.46807
16287	LOC10324:	4.889003	2.916062	1.812121	1.609198	0.107573	0.17559	0	0	3.478657	4.464254	21.39111	0
7746	LOC10322:	11.13867	2.915578	0.838059	3.478967	0.000503	0.001728	1.626259	3.284471	2.782925	24.5534	23.53022	11.05477
16679	LOC11961:	2.382606	2.915525	1.88672	1.545288	0.122277	0.194888	1.626259	0	0	2.232127	6.417332	4.019918
10552	ANKK1	9.118713	2.914001	1.066064	2.733419	0.006268	0.01579	0	1.642235	4.87012	15.62489	23.53022	9.044815
8886	AQP7	8.645516	2.913896	0.925697	3.147787	0.001645	0.004922	1.626259	1.094824	3.478657	15.62489	14.97377	15.07469
9746	MRC1	7.736279	2.91369	0.9985	2.918067	0.003522	0.009608	1.084173	2.189647	2.087194	22.32127	10.69555	8.039836
6362	FGF9	15.24852	2.913525	0.728677	3.998375	6.38E-05	0.000267	4.878777	1.642235	4.174388	22.32127	36.36488	22.10955
16255	LOC11962:	2.88398	2.913438	1.80312	1.615776	0.106143	0.173596	0	0	2.087194	8.928508	4.278221	2.009959
11056	NINJ2	7.126788	2.912877	1.114473	2.613681	0.008957	0.021538	1.084173	1.094824	2.782925	13.39276	21.39111	3.014938
16094	LOC11962!	2.83145	2.912707	1.768341	1.647141	0.099529	0.164408	0	0	2.087194	4.464254	6.417332	4.019918
5991	CTNNA3	16.74519	2.912185	0.698622	4.16847	3.07E-05	0.000136	4.878777	3.831883	2.782925	31.24978	40.6431	17.08465
6215	PDZD2	31.75534	2.912096	0.715939	4.067521	4.75E-05	0.000203	1.084173	8.758589	12.52316	44.64254	98.39909	25.12449
11758	LOC10323!	5.251971	2.910299	1.184537	2.456908	0.014014	0.031685	1.084173	0.547412	2.087194	8.928508	12.83466	6.029877
11019	LOC10323!	6.035606	2.909629	1.1085	2.624835	0.008669	0.020916	0.542086	1.642235	2.087194	8.928508	14.97377	8.039836
8194	NUTM1	12.60695	2.909434	0.868943	3.348243	0.000813	0.002639	1.084173	1.642235	6.261582	26.78552	27.80844	12.05975
12676	LOC10323(	5.024667	2.909397	1.284319	2.265322	0.023493	0.049271	1.626259	1.094824	0.695731	4.464254	19.252	3.014938
6672	KCNJ9	30.21922	2.909188	0.75212	3.867983	0.00011	0.000437	1.084173	4.379295	16.00182	71.42806	66.31243	22.10955
370	KLHL3	319.7652	2.908459	0.180301	16.13113	1.54E-58	1.11E-56	82.93921	77.73248	64.70302	535.7105	545.4732	612.0325
19748	TRNAR-UC	1.606769	2.908038	2.742276	1.060447	0.288941	0.388957	1.084173	0	0	0	8.556443	0
10153	RHBG	10.20104	2.907916	1.029357	2.824983	0.004728	0.012381	0.542086	0.547412	6.261582	24.5534	19.252	10.04979
6846	ALK	18.91844	2.907153	0.765219	3.799112	0.000145	0.000564	5.96295	1.642235	5.565851	37.94616	51.33866	11.05477
15896	LOC11961{	3.724393	2.906965	1.725276	1.684928	0.092002	0.153868	0.542086	0	2.087194	11.16063	8.556443	0
13787	SH2D1B	5.442505	2.904466	1.417738	2.048662	0.040495	0.078085	0	1.094824	2.782925	2.232127	23.53022	3.014938
18952	LOC11962:	1.63955	2.903692	2.43154	1.194178	0.232408	0.326012	0	1.094824	0	4.464254	4.278221	0
5738	LOC10324!	41.19668	2.903354	0.674248	4.306061	1.66E-05	7.70E-05	1.084173	8.758589	19.48048	78.12444	100.5382	39.1942
9800	CA7	8.856688	2.903181	0.99942	2.904866	0.003674	0.009967	1.084173	1.094824	4.174388	8.928508	27.80844	10.04979
16312	VTCN1	2.815947	2.902623	1.809569	1.604041	0.108705	0.177165	0	0	2.087194	2.232127	8.556443	4.019918
11353	USHBP1	6.268045	2.902462	1.13959	2.546937	0.010867	0.025448	0.542086	0.547412	3.478657	11.16063	12.83466	9.044815
14355	BTLA	3.415957	2.901912	1.487514	1.950847	0.051075	0.09459	0	1.094824	1.391463	6.696381	4.278221	7.034856
6847	SLITRK2	22.51595	2.901173	0.763637	3.799154	0.000145	0.000564	0.542086	3.831883	11.82743	49.10679	40.6431	29.1444
15760	RGR	2.632323	2.901045	1.69881	1.707693	0.087693	0.147908	0.542086	0	1.391463	6.696381	2.139111	5.024897
17386	CLRN2	2.905506	2.900674	2.026638	1.431274	0.152352	0.232962	0	0	2.087194	8.928508	6.417332	0
12733	LOC11962:	5.46466	2.900296	1.288171	2.251483	0.024355	0.050847	0	0.547412	3.478657	11.16063	8.556443	9.044815
9559	LOC10324:	13.71347	2.898778	0.975915	2.970319	0.002975	0.008274	0	2.189647	7.653045	35.71403	25.66933	11.05477
18126	LOC10322:	1.602522	2.897732	2.201041	1.316528	0.187997	0.275731	0	1.094824	0	2.232127	4.278221	2.009959
19776	LOC11962:	1.608544	2.897709	2.741317	1.05705	0.290489	0.390506	0	1.094824	0	0	8.556443	0
5426	MASP1	26.92411	2.896551	0.651252	4.447665	8.68E-06	4.25E-05	3.252518	6.02153	9.740239	60.26743	64.17332	18.08963
4013	LOC10324(	46.50016	2.895882	0.540136	5.361391	8.26E-08	5.47E-07	5.96295	7.116354	20.17621	71.42806	124.0684	50.24897
12155	DCDC2C	7.329997	2.895429	1.219283	2.374698	0.017563	0.038414	0	1.094824	4.174388	11.16063	23.53022	4.019918
18935	LOC11962(	1.587019	2.895391	2.41688	1.197987	0.230922	0.324205	0	1.094824	0	0	6.417332	2.009959
13037	ICOS	5.538716	2.895059	1.317239	2.197823	0.027962	0.057019	0	0.547412	3.478657	15.62489	8.556443	5.024897
6493	CELF4	37.81272	2.894475	0.73402	3.943318	8.04E-05	0.000329	1.626259	6.568942	18.78475	78.12444	102.6773	19.09461
10053	LOC10322:	9.99846	2.894174	1.015335	2.850461	0.004366	0.011545	1.084173	0.547412	5.565851	20.08914	25.66933	7.034856
17927	C1QC	16.53733	2.893919	2.134783	1.355603	0.175225	0.259852	0	0	11.82743	44.64254	25.66933	17.08465
3276	IFIT1	53.74774	2.893443	0.477128	6.064291	1.33E-09	1.08E-08	6.505036	8.758589	23.65487	78.12444	106.9555	98.48799
41	LOC11962:	44137.89	2.892363	0.091658	31.55608	1.48E-218	9.59E-216	10035.64	10814.67	10585.55	78691.4	71367.15	83332.9
10769	AGXT2	9.084879	2.892056	1.081114	2.675071	0.007471	0.018444	0	1.094824	5.565851	13.39276	21.39111	13.06473
18232	LOC11962!	1.586132	2.892004	2.233635	1.294752	0.195406	0.284931	0.542086	0.547412	0	0	6.417332	2.009959
11927	LOC11962‹	6.086196	2.891974	1.195409	2.419234	0.015553	0.034668	0	1.642235	2.782925	15.62489	6.417332	10.04979
6216	LOC11961!	15.10096	2.891891	0.710987	4.067432	4.75E-05	0.000203	2.168345	4.379295	4.174388	24.5534	34.22577	21.10457
36	CTSH	3216.829	2.891189	0.088814	32.55348	1.87E-232	1.38E-229	748.6213	782.2515	761.8259	5551.3	5762.764	5694.214
10983	PPP3R2	13.20664	2.891027	1.0979	2.633233	0.008458	0.020472	0	0.547412	9.044508	20.08914	38.50399	11.05477
2847	LOC10324:	43.60867	2.890403	0.436807	6.61712	3.66E-11	3.42E-10	9.757555	8.211177	13.2189	64.73168	98.39909	67.33362
16582	CUNH16or	2.837472	2.890052	1.852259	1.560285	0.118693	0.190293	0	0	2.087194	2.232127	10.69555	2.009959
13092	IRAG2	6.494719	2.888725	1.319919	2.188563	0.028629	0.058134	0	0.547412	4.174388	6.696381	23.53022	4.019918
15906	NKAIN4	2.670239	2.887616	1.715644	1.683109	0.092354	0.154359	0	0.547412	1.391463	8.928508	2.139111	3.014938
44	LOC10322‹	23509.52	2.886989	0.092611	31.17342	2.44E-213	1.48E-210	5457.183	6010.034	5331.389	40388.11	39562.85	44307.53
6500	MX2	15.45999	2.886974	0.732718	3.940089	8.15E-05	0.000333	2.168345	3.284471	5.565851	35.71403	29.94755	16.07967
7462	LOC10322:	14.02559	2.886739	0.80698	3.577215	0.000347	0.001237	1.626259	2.189647	6.261582	26.78552	34.22577	13.06473
15599	WDR87	36.04972	2.886735	1.669258	1.729352	0.083746	0.142726	0	2.189647	23.65487	51.33892	106.9555	32.15934
6386	CASP5	18.19627	2.886119	0.724374	3.984294	6.77E-05	0.000282	1.084173	7.663765	4.174388	40.17829	29.94755	26.12947
55	BMP1	3347.71	2.885322	0.100236	28.78529	3.28E-182	1.58E-179	791.9882	795.3894	809.1356	5734.334	5512.488	6442.923
4419	PTGER1	54.36548	2.884955	0.56944	5.066305	4.06E-07	2.44E-06	4.878777	6.568942	27.82925	102.6778	121.9293	62.30873
17926	AQP5	1.797933	2.884455	2.127737	1.355645	0.175212	0.259847	0	0	1.391463	2.232127	2.139111	5.024897
14454	G6PC1	3.42198	2.883263	1.491483	1.933151	0.053218	0.097882	0	1.094824	1.391463	4.464254	8.556443	5.024897
13691	ADRB3	4.294286	2.882909	1.392225	2.07072	0.038385	0.074535	0	1.642235	1.391463	11.16063	8.556443	3.014938
17323	LOC10323!	2.800444	2.882766	1.995507	1.444628	0.148562	0.227993	0	0	2.087194	0	10.69555	4.019918
6035	PTGS1	15.97646	2.882665	0.694874	4.148473	3.35E-05	0.000147	2.168345	4.379295	4.87012	31.24978	32.08666	21.10457
73	PAG1	4061.803	2.882042	0.108693	26.51549	6.43E-155	2.34E-152	980.0922	1072.927	857.141	6928.522	7009.866	7522.271
9873	SLC28A2	9.345448	2.879965	0.997128	2.88826	0.003874	0.010431	0.542086	3.284471	2.782925	15.62489	27.80844	6.029877
4753	BSND	33.8555	2.879489	0.59485	4.840696	1.29E-06	7.23E-06	4.878777	4.926706	14.61036	53.57105	91.98176	33.16432

14379	LOC10322	5.862344	2.879407	1.477849	1.948377	0.05137	0.094977	0	2.737059	1.391463	2.232127	27.80844	1.004979
9430	ROBO4	11.87448	2.879403	0.959593	3.000649	0.002694	0.007595	0	4.379295	4.174388	13.39276	34.22577	15.07469
13801	CFAP100	4.5052	2.87908	1.406656	2.046755	0.040682	0.078366	0	0.547412	2.782925	13.39276	4.278221	6.029877
10119	LOC11962	12.33857	2.878814	1.016175	2.832989	0.004611	0.012115	0	3.284471	5.565851	33.4819	25.66933	6.029877
11099	LOC11962	8.167582	2.878517	1.10476	2.605558	0.009172	0.02197	0.542086	0.547412	4.87012	15.62489	21.39111	6.029877
13711	IL12RB2	4.278783	2.878356	1.392638	2.066837	0.038749	0.075128	0	1.642235	1.391463	8.928508	10.69555	3.014938
6024	LOC10323	34.93006	2.878214	0.692906	4.153829	3.27E-05	0.000144	3.252518	3.284471	18.78475	53.57105	100.5382	30.14938
17819	OBP2B	14.44185	2.878201	2.094558	1.374133	0.1694	0.252742	0	0	10.43597	26.78552	36.36488	13.06473
2196	CRB1	55.03474	2.877714	0.377807	7.616893	2.60E-14	3.15E-13	12.46799	13.13788	13.91463	82.5887	117.6511	90.44815
17834	LOC10322	1.834961	2.876711	2.097593	1.371434	0.17024	0.253775	0	0	1.391463	4.464254	2.139111	3.014938
11457	SYN2	6.044823	2.876697	1.139083	2.525449	0.011555	0.026813	0.542086	1.094824	2.782925	6.696381	17.11289	8.039836
12517	LOC10322	7.416728	2.876633	1.251588	2.298387	0.02154	0.045749	0	0.547412	4.87012	20.08914	14.97377	4.019918
82	CPM	2097.432	2.87645	0.111549	25.7864	1.26E-146	4.08E-144	469.9889	564.3816	473.7931	3645.063	3604.401	3826.962
18458	OGN	1.850464	2.874351	2.290839	1.254715	0.209582	0.301844	0	0	1.391463	6.696381	0	3.014938
16124	LOC10322	2.690876	2.873957	1.750909	1.641409	0.100713	0.166059	0.542086	0	1.391463	8.928508	4.278221	1.004979
115	LOC11962	911.8081	2.873286	0.12098	23.75001	1.10E-124	2.54E-122	230.3867	215.1328	210.8066	1542.4	1696.315	1575.808
15286	KLF17	4.746489	2.872752	1.611919	1.782194	0.074718	0.129938	0	0	3.478657	11.16063	12.83466	1.004979
13736	LOC10324	4.299421	2.870211	1.390993	2.063425	0.039072	0.075621	0.542086	1.094824	1.391463	8.928508	12.83466	1.004979
14715	UGT3A2	3.443505	2.870034	1.520854	1.88712	0.059144	0.106853	0	1.094824	1.391463	4.464254	10.69555	3.014938
11263	LOC10321	7.936562	2.868352	1.117242	2.567352	0.010248	0.024189	1.626259	0	4.174388	13.39276	21.39111	7.034856
12916	LCN10	5.315494	2.868106	1.292845	2.218445	0.026525	0.054595	0	1.094824	2.782925	11.16063	12.83466	4.019918
13556	PRR30	6.559925	2.867806	1.366906	2.098027	0.035903	0.07041	0	0	4.87012	15.62489	12.83466	6.029877
13690	CD8B	4.458691	2.867398	1.384624	2.070885	0.03837	0.074511	0	0.547412	2.782925	6.696381	10.69555	6.029877
4820	ANKRD63	21.84381	2.867242	0.597327	4.800125	1.59E-06	8.75E-06	6.505036	4.379295	4.87012	26.78552	51.33866	37.18424
7579	LHX9	18.19757	2.866766	0.811372	3.533234	0.000411	0.00144	2.168345	3.284471	7.653045	62.49956	23.53022	10.04979
9890	LY6G6C	12.1715	2.866375	0.993087	2.886328	0.003898	0.010477	0	3.284471	5.565851	15.62489	38.50399	10.04979
17881	LOC11962	1.819458	2.866201	2.101436	1.363925	0.172591	0.25659	0	0	1.391463	2.232127	4.278221	3.014938
17882	LOC10323	1.819458	2.866201	2.101436	1.363925	0.172591	0.25659	0	0	1.391463	2.232127	4.278221	3.014938
5718	LOC11961	18.65734	2.865904	0.663961	4.316373	1.59E-05	7.37E-05	3.252518	2.737059	7.653045	37.94616	34.22577	26.12947
11022	LOC11962	9.972425	2.865823	1.091964	2.624466	0.008679	0.02093	0	1.094824	6.261582	15.62489	27.80844	9.044815
8926	ITIH3	17.97366	2.86549	0.91316	3.137993	0.001701	0.005066	0	2.737059	10.43597	37.94616	40.6431	16.07967
10443	TEX13A	9.641576	2.865372	1.03946	2.756598	0.005841	0.014869	0	1.642235	5.565851	11.16063	21.39111	18.08963
10428	LOC10322	7.613511	2.864619	1.037569	2.760894	0.005764	0.014696	0.542086	1.642235	3.478657	6.696381	19.252	14.06971
13012	KCNA10	5.299992	2.864287	1.300993	2.201617	0.027692	0.056574	0	1.094824	2.782925	8.928508	14.97377	4.019918
6214	RASGEF1A	15.56014	2.864228	0.704156	4.067605	4.75E-05	0.000203	2.710432	4.926706	3.478657	26.78552	36.36488	19.09461
12206	MANSC4	5.723454	2.864034	1.210619	2.365761	0.017993	0.039194	1.626259	1.642235	0.695731	13.39276	14.97377	2.009959
5656	LOC11961	17.70731	2.863537	0.659152	4.344271	1.40E-05	6.57E-05	2.710432	6.568942	3.478657	31.24978	32.08666	30.14938
18063	LOC10323	1.871989	2.863266	2.150397	1.331506	0.183023	0.269356	0	0	1.391463	6.696381	2.139111	1.004979
18064	LOC10323	1.871989	2.863266	2.150397	1.331506	0.183023	0.269356	0	0	1.391463	6.696381	2.139111	1.004979
10888	PLEKHG4B	11.8825	2.862535	1.079146	2.652593	0.007988	0.019503	0	0.547412	8.348776	15.62489	25.66933	21.10457
12255	MHC-DOB	6.762828	2.862251	1.215283	2.355213	0.018512	0.040159	0	2.737059	2.087194	15.62489	17.11289	3.014938
6115	LOC10322	17.23458	2.861453	0.695871	4.112048	3.92E-05	0.00017	5.96295	2.189647	4.174388	33.4819	38.50399	19.09461
394	KLHL14	311.4988	2.86137	0.18105	15.80427	2.91E-56	1.96E-54	64.50828	85.94365	75.83472	553.5675	528.3603	560.7785
54	SLC17A1	2191.117	2.860486	0.098266	29.10954	2.72E-186	1.34E-183	545.881	537.5584	507.8839	3765.598	3764.835	4024.943
6428	LOC11962	27.25207	2.859974	0.720611	3.968818	7.22E-05	0.000299	1.626259	4.379295	13.91463	64.73168	57.75599	21.10457
143	PLXNA2	1772.596	2.859776	0.127249	22.47391	7.47E-112	1.39E-109	375.1238	480.6276	433.4406	2970.961	2943.416	3432.005
9010	CD22	16.31772	2.859346	0.918626	3.112633	0.001854	0.005471	0.542086	1.094824	10.43597	31.24978	38.50399	16.07967
8481	P2RY6	25.69145	2.858982	0.877077	3.259671	0.001115	0.003496	0.542086	1.094824	17.39328	40.17829	57.75599	37.18424
11397	LOC10321	6.899685	2.858924	1.126729	2.537366	0.011169	0.026053	0	1.642235	3.478657	13.39276	12.83466	10.04979
15600	ANGPT2	3.449528	2.856949	1.652097	1.729286	0.083758	0.142728	0	1.094824	1.391463	2.232127	14.97377	1.004979
5648	DGKB	24.8737	2.856627	0.656942	4.348371	1.37E-05	6.46E-05	7.047123	3.831883	6.957314	46.87467	68.45154	16.07967
11881	LOC11962	5.955893	2.856296	1.175464	2.429931	0.015102	0.033792	1.626259	0.547412	2.087194	15.62489	12.83466	3.014938
2896	LOC10321	43.29971	2.855368	0.435665	6.55404	5.60E-11	5.14E-10	11.38381	8.758589	11.1317	78.12444	94.12087	56.27885
5603	B3GNT6	32.20561	2.854533	0.652799	4.372758	1.23E-05	5.82E-05	3.252518	4.379295	16.00182	58.0353	83.42532	28.13943
18014	NXPE4	1.856486	2.853087	2.127901	1.340798	0.179986	0.265608	0	0	1.391463	4.464254	4.278221	1.004979
18015	LOC11962	1.856486	2.853087	2.127901	1.340798	0.179986	0.265608	0	0	1.391463	4.464254	4.278221	1.004979
13122	PADI6	9.335386	2.853076	1.306919	2.183055	0.029032	0.058799	0	0	6.957314	8.928508	32.08666	8.039836
18133	LOC11962	15.1718	2.852506	2.167837	1.315831	0.188231	0.275968	0	0	11.1317	15.62489	49.19955	15.07469
881	GMFG	132.1776	2.852375	0.245572	11.61522	3.45E-31	1.04E-29	34.15144	29.01283	33.39511	236.6055	226.7457	233.1552
8405	CCR5	19.30362	2.851379	0.868823	3.281888	0.001031	0.003262	0	3.831883	10.43597	31.24978	49.19955	21.10457
16316	LOC11962	3.285654	2.849224	1.777823	1.602648	0.109012	0.177623	1.626259	0	0.695731	6.696381	10.69555	0
13108	TECTB	4.28109	2.847733	1.302705	2.186015	0.028814	0.05844	0.542086	0.547412	2.087194	8.928508	8.556443	5.024897
161	LGR5	2052.62	2.847199	0.13189	21.58775	2.34E-103	3.87E-101	428.2482	594.4892	480.0546	3618.278	3428.994	3765.658
5771	LOC11962	20.80667	2.846918	0.663976	4.287685	1.81E-05	8.32E-05	5.96295	5.474118	3.478657	26.78552	62.03421	21.10457
16151	BPIFA1	2.440994	2.846292	1.738375	1.637329	0.101562	0.167174	0	1.094824	0.695731	6.696381	2.139111	4.019918
13536	LOC10321	4.033148	2.846087	1.354844	2.100676	0.035669	0.07005	0.542086	1.642235	0.695731	4.464254	12.83466	4.019918
10083	XG	7.561609	2.845677	1.001572	2.841209	0.004494	0.011849	1.626259	1.094824	2.782925	17.85702	14.97377	7.034856
13729	LOC11962	6.060789	2.845245	1.378031	2.064717	0.03895	0.075423	0	1.642235	2.782925	26.78552	2.139111	3.014938
9597	LOC11962	8.350379	2.844819	0.960445	2.961979	0.003057	0.008467	1.626259	1.642235	2.782925	15.62489	21.39111	7.034856
14605	SPATA46	4.371536	2.843994	1.492767	1.905182	0.056756	0.103312	0	1.094824	2.087194	15.62489	6.417332	1.004979
11152	LOC11962	6.51117	2.843759	1.09721	2.591809	0.009547	0.02276	0.542086	2.737059	1.391463	13.39276	14.97377	6.029877
13852	GPR171	4.31723	2.843228	1.394336	2.039127	0.041437	0.079527	1.084173	0	2.087194	11.16063	8.556443	3.014938
13345	LOC11961	4.936991	2.841931	1.329158	2.138144	0.032505	0.064754	2.168345	0	1.391463	15.62489	6.417332	4.019918
15776	LOC10322	3.69747	2.841695	1.666118	1.705579	0.088087	0.148439	0	0	2.782925	6.696381	10.69555	2.009959

18124	MYOT	1.840984	2.8415	2.15789	1.316796	0.187907	0.275621	0	0	1.391463	2.232127	6.417332	1.004979
18670	NCR2	1.803956	2.840817	2.305196	1.232354	0.217817	0.310159	0	0	1.391463	0	6.417332	3.014938
5951	SLC1A2	16.35671	2.840079	0.678574	4.185366	2.85E-05	0.000127	3.794605	2.737059	5.565851	24.5534	36.36488	25.12449
12394	LRRC53	6.22426	2.840073	1.223073	2.322079	0.020229	0.043389	0	3.831883	0.695731	8.928508	12.83466	11.05477
18199	LRRN1	16.95983	2.84003	2.180856	1.302255	0.192829	0.281684	0	0	12.52316	31.24978	44.92132	13.06473
11418	EPX	9.857459	2.837537	1.120546	2.532281	0.011332	0.026386	0	1.094824	6.261582	20.08914	25.66933	6.029877
10424	LOC11962(	7.062898	2.837075	1.027446	2.761288	0.005757	0.014683	1.626259	2.737059	0.695731	11.16063	17.11289	9.044815
9891	SPAG17	9.496187	2.836931	0.982946	2.886151	0.0039	0.010481	0.542086	4.926706	1.391463	13.39276	25.66933	11.05477
14333	LOC10322(	3.781491	2.835963	1.450408	1.955286	0.050549	0.093759	1.084173	1.642235	0	2.232127	10.69555	7.034856
13889	KRT23	4.303503	2.83525	1.394948	2.032512	0.042102	0.080587	0	1.094824	2.087194	8.928508	10.69555	3.014938
15938	TDRD10	3.681967	2.835143	1.689551	1.678046	0.093338	0.155691	0	0	2.782925	4.464254	12.83466	2.009959
13432	LOC10321(	5.355717	2.834008	1.336287	2.120808	0.033938	0.067171	0	0.547412	3.478657	13.39276	10.69555	4.019918
6559	KCNJ15	21.44327	2.833732	0.723587	3.916227	8.99E-05	0.000365	2.710432	1.642235	11.82743	31.24978	47.06043	34.1693
6497	LOC11962;	24.15462	2.833098	0.718839	3.941214	8.11E-05	0.000332	3.252518	2.189647	12.52316	49.10679	57.75599	20.09959
13274	LOC10322(	5.303186	2.832848	1.315822	2.152911	0.031326	0.062739	0	0.547412	3.478657	8.928508	12.83466	6.029877
12196	LOC10322:	5.924722	2.832217	1.196489	2.367107	0.017928	0.039079	0	1.642235	2.782925	13.39276	10.69555	7.034856
16216	MS4A10	2.409989	2.831412	1.742142	1.625248	0.10411	0.170681	0	1.094824	0.695731	2.232127	6.417332	4.019918
18276	LOC11962;	19.6802	2.831223	2.197236	1.288538	0.197559	0.287377	0	0	14.61036	29.01765	51.33866	23.11453
5675	ERBB4	19.24101	2.831168	0.652663	4.337873	1.44E-05	6.74E-05	4.336691	5.474118	4.174388	29.01765	51.33866	21.10457
17758	LOC11962!	1.604829	2.828561	2.056868	1.375179	0.169076	0.252436	0.542086	0	0.695731	2.232127	2.139111	4.019918
16796	GFY	26.02507	2.828558	1.853043	1.526439	0.126901	0.20086	0	0.547412	18.78475	46.87467	74.86887	15.07469
12955	LOC11962:	5.172093	2.828361	1.278496	2.212257	0.026949	0.055302	0.542086	0.547412	2.782925	17.85702	4.278221	5.024897
13411	ME3	5.287684	2.828204	1.331007	2.12486	0.033598	0.066603	0	0.547412	3.478657	6.696381	14.97377	6.029877
9561	SLC39A2	9.180258	2.827876	0.952499	2.968903	0.002989	0.00831	1.084173	2.189647	3.478657	15.62489	25.66933	7.034856
16218	SPATA3	3.666464	2.827527	1.740011	1.625005	0.104162	0.170744	0	0	2.782925	2.232127	14.97377	2.009959
9725	CNTN5	7.902257	2.827052	0.966884	2.923878	0.003457	0.00945	1.084173	3.284471	1.391463	15.62489	14.97377	11.05477
9512	LOC11962:	18.79354	2.826161	0.947932	2.981395	0.002869	0.00802	0	1.642235	12.52316	35.71403	42.78221	20.09959
16208	CD80	2.462519	2.825816	1.737762	1.626123	0.103923	0.170459	0	1.094824	0.695731	6.696381	4.278221	2.009959
15200	SEPTIN12	4.471366	2.825793	1.573152	1.796262	0.072453	0.126715	0	0	3.478657	11.16063	2.139111	10.04979
11946	CD86	6.891625	2.824661	1.169651	2.41496	0.015737	0.035021	1.084173	0	4.174388	8.928508	17.11289	10.04979
18968	HCRTR1	1.825481	2.824135	2.3719	1.190664	0.233786	0.327636	0	0	1.391463	0	8.556443	1.004979
18969	FOXD3	1.825481	2.824135	2.3719	1.190664	0.233786	0.327636	0	0	1.391463	0	8.556443	1.004979
16224	SLCO1C1	2.445242	2.823728	1.739733	1.623081	0.104572	0.171354	1.084173	0	0.695731	4.464254	6.417332	2.009959
5692	LOC11962(	54.1108	2.823298	0.651884	4.330981	1.48E-05	6.93E-05	10.29964	1.642235	28.52499	82.5887	143.3204	58.28881
68	NPL	2221.112	2.822949	0.104559	26.99863	1.53E-160	6.00E-158	527.45	587.9203	535.7132	3747.741	3771.252	4156.595
11504	LOC10323(	9.790055	2.822846	1.12256	2.51465	0.011915	0.027535	0	0.547412	6.957314	22.32127	12.83466	16.07967
13245	PDILT	6.188167	2.822648	1.307747	2.158406	0.030896	0.062014	0	0.547412	4.174388	20.08914	4.278221	8.039836
9879	SOST	9.397195	2.821643	0.977294	2.887199	0.003887	0.010461	1.084173	1.094824	4.87012	15.62489	25.66933	8.039836
12399	LOC11962:	7.019987	2.821584	1.215483	2.32137	0.020267	0.043455	0	1.094824	4.174388	17.85702	14.97377	4.019918
3075	ERN2	42.17905	2.820416	0.447317	6.305182	2.88E-10	2.49E-09	13.01007	6.568942	11.82743	89.28508	62.03421	70.34856
5524	LOC1032⁄	19.92341	2.820288	0.640568	4.402793	1.07E-05	5.14E-05	4.878777	5.474118	4.174388	35.71403	49.19955	20.09959
12962	GZMA	5.089444	2.81943	1.275049	2.211233	0.02702	0.055417	0	1.094824	2.782925	8.928508	10.69555	7.034856
10690	IL2RA	9.361942	2.819406	1.046355	2.694503	0.007049	0.017531	0	2.189647	4.87012	13.39276	25.66933	10.04979
15054	LOC10323(	3.467338	2.818916	1.545756	1.823649	0.068205	0.120449	0.542086	0	2.087194	4.464254	10.69555	3.014938
13300	SH2D2A	8.233412	2.818207	1.312458	2.147274	0.031772	0.063507	0	0	6.261582	17.85702	19.252	6.029877
10193	LOC10322!	9.022407	2.817454	1.000085	2.817213	0.004844	0.012635	2.710432	1.094824	2.782925	17.85702	25.66933	4.019918
10945	TRIM55	7.292767	2.816655	1.066834	2.640201	0.008286	0.020126	1.084173	2.189647	2.087194	6.696381	25.66933	6.029877
6514	SPATA19	21.95303	2.816302	0.715949	3.933662	8.37E-05	0.000341	3.794605	2.189647	10.43597	46.87467	51.33866	17.08465
14403	AMELX	4.142292	2.815523	1.449129	1.942907	0.052027	0.096032	0	1.642235	1.391463	13.39276	6.417332	2.009959
17568	LOC1032⁄	2.136806	2.815331	2.00399	1.404863	0.160062	0.24219	0.542086	1.094824	0	0	2.139111	9.044815
12128	LOC1032;	6.317013	2.813963	1.182643	2.379385	0.017342	0.038013	0	3.284471	1.391463	15.62489	8.556443	9.044815
11590	LOC10322(	7.297902	2.813938	1.127186	2.496426	0.012545	0.028776	1.626259	1.642235	2.087194	4.464254	29.94755	4.019918
16439	PRR15	2.431514	2.812125	1.777312	1.582235	0.113596	0.183706	0	1.094824	0.695731	2.232127	8.556443	2.009959
7555	TMEM150I	27.12915	2.811737	0.794195	3.540361	0.0004	0.001406	2.710432	1.094824	16.69755	60.26743	59.8951	22.10955
15469	LOC11961{	3.505253	2.811473	1.606763	1.749775	0.080157	0.137758	0	0.547412	2.087194	6.696381	10.69555	1.004979
10040	ST14	9.321198	2.811245	0.985312	2.853151	0.004329	0.011462	0.542086	1.094824	5.565851	13.39276	19.252	16.07967
10717	MALL	9.405622	2.811219	1.046085	2.687372	0.007202	0.017865	0	1.642235	5.565851	22.32127	12.83466	14.06971
17655	LOC10324{	2.673433	2.810282	2.014131	1.395283	0.162931	0.245342	0	0	2.087194	8.928508	0	5.024897
11563	LOC1032⁄:	8.598058	2.810218	1.123027	2.50236	0.012337	0.028362	1.626259	0	4.87012	13.39276	25.66933	6.029877
7328	CYP2A24	16.93508	2.810189	0.77541	3.624136	0.00029	0.001052	2.168345	1.642235	9.044508	29.01765	40.6431	19.09461
13444	HEPACAM:	4.883666	2.809385	1.326307	2.118201	0.034158	0.067546	0.542086	1.642235	1.391463	4.464254	19.252	2.009959
12709	LOC11961{	5.505201	2.808857	1.244789	2.256493	0.02404	0.050286	0.542086	2.737059	0.695731	8.928508	17.11289	3.014938
8270	LOC1032:	14.0511	2.80881	0.845376	3.322558	0.000892	0.002867	3.794605	1.094824	5.565851	42.41041	21.39111	10.04979
14014	CYP4F12	4.074258	2.808644	1.396954	2.010549	0.044373	0.084177	0	1.642235	1.391463	6.696381	10.69555	4.019918
14290	LOC11962:	4.337703	2.808574	1.430359	1.963544	0.049583	0.092244	0	0.547412	2.782925	13.39276	4.278221	5.024897
8263	LOC10321!	11.62587	2.80841	0.84461	3.325097	0.000884	0.002844	2.168345	1.094824	5.565851	22.32127	23.53022	15.07469
7116	LOC10321⁄	14.5394	2.80795	0.760929	3.690163	0.000224	0.000837	3.794605	2.737059	4.174388	31.24978	34.22577	11.05477
8299	LOC11962:	14.22277	2.807739	0.847476	3.313059	0.000923	0.002956	1.084173	3.284471	6.261582	35.71403	29.94755	9.044815
11918	AIF1	7.842956	2.807439	1.159853	2.420514	0.015499	0.034572	0	1.094824	4.87012	20.08914	14.97377	6.029877
14019	LOC1032⁄:	4.268782	2.807051	1.396583	2.009942	0.044437	0.084269	0.542086	0	2.782925	6.696381	8.556443	7.034856
15598	LOC11962;	3.088303	2.80674	1.622565	1.729817	0.083663	0.142594	1.084173	1.094824	0	8.928508	6.417332	1.004979
19002	LOC11961!	28.67671	2.8061	2.3696	1.184208	0.236331	0.330624	0	0	21.56767	49.10679	81.28621	20.09959
16652	LOC11962:	2.679456	2.805963	1.812876	1.547796	0.121671	0.194249	0	0	2.087194	6.696381	4.278221	3.014938
7437	PSTPIP2	11.98396	2.805812	0.782515	3.585634	0.000336	0.001202	2.168345	3.284471	3.478657	20.08914	27.80844	15.07469

755	KRT19	158.1751	2.80532	0.225329	12.4499	1.40E-35	4.93E-34	42.28274	38.31883	38.26523	270.0874	271.6671	288.4291
9354	FAT2	12.92203	2.804361	0.929211	3.018003	0.002544	0.007232	0.542086	1.642235	7.653045	24.5534	32.08666	11.05477
1919	APOH	63.23879	2.803016	0.343844	8.151989	3.58E-16	4.96E-15	16.26259	16.42235	14.61036	120.5349	109.0946	102.5079
16983	LOC10324	2.452152	2.802868	1.874855	1.494978	0.13492	0.211195	0.542086	0.547412	0.695731	2.232127	10.69555	0
6301	DHH	29.92274	2.802566	0.696201	4.025515	5.69E-05	0.00024	2.710432	2.737059	17.39328	44.64254	74.86887	37.18424
11287	LOC10324	7.151306	2.802339	1.094264	2.560935	0.010439	0.024588	2.168345	1.642235	1.391463	11.16063	23.53022	3.014938
5331	FOXA2	25.42052	2.802161	0.6224	4.502184	6.73E-06	3.35E-05	2.168345	6.02153	11.1317	42.41041	55.61688	35.17428
16807	LOC11962	2.626925	2.801667	1.837984	1.524315	0.12743	0.201566	0	0	2.087194	2.232127	6.417332	5.024897
11596	LOC10321	6.758849	2.80156	1.122653	2.495481	0.012579	0.028838	0.542086	1.094824	3.478657	11.16063	19.252	5.024897
17841	LOC11962	1.642744	2.801553	2.045002	1.369951	0.170702	0.254364	0	0.547412	0.695731	4.464254	2.139111	2.009959
6928	LOC11961	14.6985	2.800534	0.744006	3.764128	0.000167	0.000641	1.626259	3.284471	6.261582	31.24978	25.66933	20.09959
17070	LOC10321	2.716484	2.79902	1.885098	1.484814	0.137593	0.214288	0	0	2.087194	8.928508	4.278221	1.004979
4592	LOC10322	36.4874	2.797672	0.565531	4.946981	7.54E-07	4.36E-06	5.96295	3.831883	18.08902	69.19594	70.59065	51.25395
8798	ETNPPL	9.542964	2.79565	0.880473	3.17517	0.001497	0.004525	2.168345	2.189647	2.782925	13.39276	25.66933	11.05477
3322	LOC10324	35.42599	2.794385	0.464014	6.022203	1.72E-09	1.38E-08	8.673382	6.568942	11.82743	60.26743	59.8951	65.32367
16356	APOF	3.344042	2.792762	1.750629	1.595291	0.110647	0.17984	0	1.094824	1.391463	11.16063	6.417332	0
10241	LOC10323	10.93804	2.79231	0.995409	2.805188	0.005029	0.013054	5.420864	0.547412	2.087194	29.01765	23.53022	5.024897
10756	LOC10321	18.79647	2.792081	1.042485	2.678293	0.0074	0.018291	0	0.547412	13.91463	29.01765	49.19955	20.09959
14364	LOC11962	4.274805	2.791847	1.432231	1.949299	0.05126	0.094864	0.542086	0	2.782925	4.464254	12.83466	5.024897
11091	LOC11962	7.529288	2.791119	1.070171	2.608105	0.009105	0.021823	0.542086	1.094824	4.174388	8.928508	21.39111	9.044815
17891	ASZ1	1.627242	2.790618	2.048815	1.362064	0.173178	0.25727	0	0.547412	0.695731	2.232127	4.278221	2.009959
17892	LOC10324	1.627242	2.790618	2.048815	1.362064	0.173178	0.25727	0	0.547412	0.695731	2.232127	4.278221	2.009959
17893	LOC11962	1.627242	2.790618	2.048815	1.362064	0.173178	0.25727	0	0.547412	0.695731	2.232127	4.278221	2.009959
8165	ONECUT3	28.89397	2.790458	0.830552	3.359765	0.00078	0.00254	0.542086	2.189647	19.48048	62.49956	53.47777	35.17428
252	KIF12	716.0432	2.789956	0.151509	18.41447	1.01E-75	1.06E-73	200.0299	154.9175	189.2389	1189.724	1159.398	1402.951
18624	SPACA7	1.679772	2.789923	2.261295	1.233772	0.217288	0.309867	0	0.547412	0.695731	6.696381	2.139111	0
18634	WNT3A	1.679772	2.789923	2.261295	1.233772	0.217288	0.309867	0	0.547412	0.695731	6.696381	2.139111	0
13516	LOC10324	5.108142	2.789582	1.325073	2.105229	0.035271	0.069376	0	0.547412	3.478657	11.16063	6.417332	9.044815
7853	ERICH4	14.9964	2.788783	0.807743	3.452562	0.000555	0.00188	1.084173	2.737059	7.653045	33.4819	29.94755	15.07469
16928	LOC11962	2.64845	2.788493	1.856062	1.502371	0.133001	0.208875	0	0	2.087194	2.232127	8.556443	3.014938
11413	LOC10322	6.596751	2.787423	1.100247	2.533453	0.011294	0.026307	1.084173	1.094824	2.782925	15.62489	14.97377	4.019918
12132	LOC10321	8.247519	2.787262	1.17165	2.378919	0.017363	0.038043	2.710432	3.284471	0	20.08914	21.39111	2.009959
13584	MEOX1	5.14517	2.786886	1.332154	2.092015	0.036437	0.071311	0	0.547412	3.478657	13.39276	6.417332	7.034856
9317	GJD4	11.10635	2.786024	0.920009	3.028256	0.00246	0.007018	1.084173	4.379295	2.782925	24.5534	27.80844	6.029877
12293	GLTPD2	6.80944	2.784873	1.187082	2.345982	0.018977	0.04104	0	1.094824	4.174388	17.85702	10.69555	7.034856
6382	LOC10322	18.6806	2.78394	0.698316	3.986645	6.70E-05	0.000279	5.420864	4.926706	3.478657	42.41041	42.78221	13.06473
4613	LOC10321	26.97608	2.783171	0.563626	4.937978	7.89E-07	4.55E-06	4.878777	7.116354	8.348776	51.33892	62.03421	28.13943
18562	LOC10324	1.663382	2.780805	2.240404	1.241207	0.214529	0.307255	0.542086	0	0.695731	4.464254	4.278221	0
7569	LRRC4	15.46789	2.78023	0.786408	3.535352	0.000407	0.00143	1.626259	3.831883	6.261582	37.94616	32.08666	11.05477
2346	LOC11962	112.0763	2.780027	0.378493	7.344997	2.06E-13	2.33E-12	20.59928	22.9913	41.74388	218.7484	258.8324	109.5428
3929	LOC10324	34.29127	2.779278	0.512527	5.422696	5.87E-08	3.97E-07	7.589209	6.02153	12.52316	60.26743	79.14709	40.19918
10042	SPEM1	8.42575	2.778689	0.974226	2.8522	0.004342	0.011494	2.168345	2.737059	1.391463	26.78552	6.417332	11.05477
16330	LOC11961	2.465714	2.776901	1.736098	1.599507	0.109708	0.178592	0	0.547412	1.391463	6.696381	2.139111	4.019918
12827	TCF23	5.652164	2.776536	1.242987	2.233761	0.025499	0.052845	0	1.642235	2.782925	4.464254	14.97377	10.04979
6811	LOC11962	18.05496	2.776496	0.728205	3.812793	0.000137	0.000536	1.626259	3.284471	9.044508	40.17829	32.08666	22.10955
11200	ACR	6.84784	2.776427	1.075145	2.582375	0.009812	0.023291	0.542086	3.284471	1.391463	6.696381	17.11289	12.05975
17726	LOC11962	2.236635	2.77627	2.011976	1.379872	0.167626	0.25139	1.626259	0	0	2.232127	8.556443	1.004979
13366	LOC10323	4.923001	2.776242	1.301156	2.133673	0.03287	0.065378	1.084173	0.547412	2.087194	6.696381	17.11289	2.009959
15888	LOC11961	3.589806	2.77543	1.645782	1.686389	0.091721	0.153474	2.710432	0	0	2.232127	8.556443	8.039836
8822	LOC11961	11.36022	2.77453	0.876213	3.1665	0.001543	0.004649	1.626259	1.642235	5.565851	11.16063	32.08666	16.07967
13934	RNASE13	5.18822	2.774076	1.369605	2.025457	0.04282	0.081698	0	0.547412	3.478657	13.39276	10.69555	3.014938
15281	LOC10322	4.318743	2.77391	1.555527	1.78326	0.074544	0.129687	0	0.547412	2.782925	4.464254	17.11289	1.004979
12796	HEY2	6.125587	2.771992	1.237252	2.240442	0.025062	0.052069	1.084173	2.737059	0.695731	6.696381	23.53022	2.009959
9694	HRC	24.36724	2.77155	0.945139	2.932426	0.003363	0.009223	0	2.189647	16.69755	33.4819	72.72976	21.10457
6971	CAPN14	17.8428	2.770962	0.739398	3.747592	0.000179	0.000681	4.336691	1.094824	8.348776	37.94616	34.22577	21.10457
16243	BOLL	2.450211	2.770707	1.712542	1.617891	0.105686	0.172945	0	0.547412	1.391463	4.464254	4.278221	4.019918
16245	LOC11962	2.450211	2.770707	1.712542	1.617891	0.105686	0.172945	0	0.547412	1.391463	4.464254	4.278221	4.019918
16246	ABO	2.450211	2.770707	1.712542	1.617891	0.105686	0.172945	0	0.547412	1.391463	4.464254	4.278221	4.019918
390	PTH1R	335.3956	2.770405	0.174882	15.84158	1.61E-56	1.10E-54	86.19173	81.56436	89.74935	649.549	513.3866	591.9329
18786	LOC10322	1.647879	2.769986	2.270776	1.219841	0.222525	0.314906	0.542086	0	0.695731	2.232127	6.417332	0
156	ADGRF2	1002.22	2.76757	0.12722	21.75416	6.31E-105	1.08E-102	279.7166	233.7448	257.4206	1616.06	1771.184	1855.192
9602	DCST1	8.802215	2.766657	0.934474	2.960658	0.00307	0.008499	1.084173	2.189647	3.478657	15.62489	21.39111	9.044815
501	LOC11962	241.4761	2.766007	0.188714	14.65712	1.21E-48	6.44E-47	61.25576	59.66789	65.39875	415.1756	402.1528	445.2059
16388	OTOP1	3.560979	2.765644	1.73913	1.590245	0.11178	0.181331	0	0	2.782925	11.16063	6.417332	1.004979
18800	LOC10322	1.648767	2.765619	2.27038	1.218131	0.223174	0.31559	0	0.547412	0.695731	2.232127	6.417332	0
3412	TM4SF20	33.22279	2.76525	0.46593	5.934906	2.94E-09	2.29E-08	9.215468	9.306001	6.957314	55.80317	57.75599	60.29877
3266	LOC11962	74.59625	2.764784	0.454993	6.076539	1.23E-09	1.00E-08	9.215468	16.96977	31.30791	136.1597	177.5462	76.37844
9727	TRNAL-AA	8.650484	2.764553	0.945738	2.923171	0.003465	0.00947	1.084173	2.737059	2.782925	24.5534	10.69555	10.04979
20738	LOC11962	0.82297	2.763414	3.030136	0.911977	0.361781	0.463761	0	0	0.695731	2.232127	0	2.009959
20739	LOC11962	0.82297	2.763414	3.030136	0.911977	0.361781	0.463761	0	0	0.695731	2.232127	0	2.009959
7334	CNTN2	14.56086	2.763207	0.763257	3.620283	0.000294	0.001067	2.168345	2.189647	6.957314	29.01765	29.94755	17.08465
10795	LOC10323	7.834424	2.762892	1.034168	2.671607	0.007549	0.018592	0.542086	3.284471	2.087194	20.08914	14.97377	6.029877
20815	LOC10322	0.79825	2.762889	3.060667	0.902708	0.366681	0.468348	0	0.547412	0	2.232127	0	2.009959
9079	LOC10323	13.33546	2.76237	0.894134	3.089437	0.002005	0.005872	5.420864	0.547412	4.174388	22.32127	38.50399	9.044815

12400	LOC11962(	7.250804	2.762067	1.190043	2.32098	0.020288	0.043496	0	2.737059	2.782925	17.85702	17.11289	3.014938
15672	LOC11962‹	3.022576	2.76114	1.603187	1.722281	0.085019	0.14422	1.626259	0	0.695731	2.232127	8.556443	5.024897
3772	NT5C1A	36.84337	2.760075	0.495263	5.572951	2.50E-08	1.77E-07	9.757555	9.306001	9.044508	51.33892	98.39909	43.21412
12896	ITIH6	5.580512	2.759834	1.243496	2.219415	0.026459	0.054544	0	2.189647	2.087194	15.62489	8.556443	5.024897
10621	LOC11961ŧ	9.092319	2.759353	1.016557	2.714411	0.006639	0.016619	1.626259	0.547412	4.87012	20.08914	21.39111	6.029877
21497	P3R3URF	0.785942	2.759227	3.432865	0.803768	0.421531	0.521301	0	0	0.695731	0	0	4.019918
5665	RGS16	19.36016	2.75765	0.635336	4.340457	1.42E-05	6.67E-05	4.336691	4.926706	5.565851	29.01765	49.19955	23.11453
16222	MAGEB3	3.455917	2.756344	1.69789	1.623393	0.104505	0.171266	0	0	2.782925	2.232127	10.69555	5.024897
16122	LOC10323!	3.492945	2.756259	1.6791	1.64151	0.100692	0.166039	0	0	2.782925	4.464254	10.69555	3.014938
13231	VSTM5	9.717877	2.755847	1.27415	2.162891	0.03055	0.061383	0	0	7.653045	20.08914	23.53022	7.034856
10337	LOC10321ŧ	9.025174	2.75536	0.991109	2.780078	0.005435	0.013977	1.084173	1.094824	4.87012	13.39276	25.66933	8.039836
21474	LOC11962‹	0.83439	2.755235	3.412642	0.807361	0.419458	0.519222	0.542086	0	0	4.464254	0	0
21476	LOC11962(	0.83439	2.755235	3.412642	0.807361	0.419458	0.519222	0.542086	0	0	4.464254	0	0
21477	LOC10324:	0.83439	2.755235	3.412642	0.807361	0.419458	0.519222	0.542086	0	0	4.464254	0	0
21469	LOC11962;	0.835278	2.755187	3.411377	0.807647	0.419294	0.51918	0	0.547412	0	4.464254	0	0
21470	LOC11961(	0.835278	2.755187	3.411377	0.807647	0.419294	0.51918	0	0.547412	0	4.464254	0	0
21472	LOC11962:	0.835278	2.755187	3.411377	0.807647	0.419294	0.51918	0	0.547412	0	4.464254	0	0
13494	LOC11962:	4.64699	2.754856	1.306599	2.108417	0.034995	0.068953	1.626259	0	2.087194	6.696381	6.417332	11.05477
13100	LOC10322(	4.958088	2.754413	1.258925	2.187909	0.028676	0.058195	0.542086	0.547412	2.782925	11.16063	10.69555	4.019918
196	ADGRF4	636.3143	2.754243	0.134861	20.4229	1.05E-92	1.42E-90	153.9525	179.0037	160.0182	1080.349	1105.92	1138.642
1524	TMEM72	92.92079	2.754034	0.300423	9.167195	4.85E-20	8.47E-19	23.8518	20.25424	28.52499	162.9453	139.0422	182.9063
21406	LOC10324:	0.859998	2.753837	3.376935	0.815484	0.414795	0.514935	0	0	0.695731	4.464254	0	0
21407	LOC11962‹	0.859998	2.753837	3.376935	0.815484	0.414795	0.514935	0	0	0.695731	4.464254	0	0
21409	ZNF80	0.859998	2.753837	3.376935	0.815484	0.414795	0.514935	0	0	0.695731	4.464254	0	0
21410	LOC11962ŧ	0.859998	2.753837	3.376935	0.815484	0.414795	0.514935	0	0	0.695731	4.464254	0	0
21412	LOC10324:	0.859998	2.753837	3.376935	0.815484	0.414795	0.514935	0	0	0.695731	4.464254	0	0
21413	LOC10323:	0.859998	2.753837	3.376935	0.815484	0.414795	0.514935	0	0	0.695731	4.464254	0	0
21414	AGTR2	0.859998	2.753837	3.376935	0.815484	0.414795	0.514935	0	0	0.695731	4.464254	0	0
21415	LOC11962:	0.859998	2.753837	3.376935	0.815484	0.414795	0.514935	0	0	0.695731	4.464254	0	0
10729	LOC10321ŧ	9.063089	2.753215	1.025344	2.685162	0.007249	0.017963	0.542086	1.642235	4.87012	15.62489	25.66933	6.029877
17470	LOC10323(	2.524267	2.752791	1.943914	1.416108	0.156744	0.238525	0	0.547412	1.391463	8.928508	4.278221	0
16430	LOC11962;	2.470849	2.751933	1.737228	1.584095	0.113172	0.183121	0.542086	0	1.391463	4.464254	6.417332	2.009959
14855	LOC10324‹	4.105001	2.75166	1.478303	1.861364	0.062693	0.112197	0	1.094824	2.087194	4.464254	14.97377	2.009959
4124	RHOV	35.55946	2.750943	0.521536	5.274689	1.33E-07	8.57E-07	5.420864	7.663765	14.61036	84.82083	55.61688	45.22408
13501	NPPC	5.151418	2.750243	1.305613	2.106476	0.035163	0.069234	2.710432	1.094824	0	13.39276	10.69555	3.014938
5052	LOC10322:	30.1399	2.749973	0.59018	4.65955	3.17E-06	1.67E-05	7.047123	3.831883	12.52316	80.35657	44.92132	32.15934
16437	LOC10323(	2.471737	2.749193	1.737155	1.582583	0.113516	0.1836	0	0.547412	1.391463	4.464254	6.417332	2.009959
7736	FRMD3	13.78294	2.748687	0.789573	3.481231	0.000499	0.001715	3.794605	3.831883	2.782925	29.01765	34.22577	9.044815
14841	LOC11962:	3.076882	2.747404	1.474427	1.863371	0.06241	0.111797	0.542086	1.094824	0.695731	6.696381	6.417332	3.014938
241	CTSD	26961.34	2.74721	0.147035	18.68404	6.68E-78	7.37E-76	7952.407	5451.127	7568.862	45059.95	41402.49	54333.21
13708	LOC11962:	7.892396	2.746659	1.328744	2.067109	0.038724	0.0751	0	0	6.261582	20.08914	14.97377	6.029877
13621	LOC11962(	6.468057	2.745875	1.317012	2.084927	0.037076	0.072363	0	2.189647	2.782925	17.85702	14.97377	1.004979
7399	SRPK3	14.15826	2.744886	0.762411	3.600273	0.000318	0.001142	3.794605	1.642235	5.565851	26.78552	32.08666	15.07469
9355	PNMA8C	11.35634	2.744613	0.909582	3.017444	0.002549	0.007244	1.626259	1.642235	5.565851	22.32127	29.94755	7.034856
17452	SLC5A12	2.508765	2.744084	1.935003	1.418129	0.156153	0.237871	0	0.547412	1.391463	6.696381	6.417332	0
9053	SRMS	10.13946	2.74363	0.886414	3.095201	0.001967	0.005776	2.168345	2.189647	3.478657	31.24978	10.69555	11.05477
4878	CACNA1I	40.13578	2.743012	0.577076	4.753296	2.00E-06	1.09E-05	4.878777	7.116354	19.48048	58.0353	109.0946	42.20914
17172	LOC10324(	3.418889	2.742997	1.863761	1.471754	0.141087	0.21844	0	0	2.782925	0	10.69555	7.034856
17200	SLC7A4	3.050124	2.742825	1.868721	1.467755	0.142171	0.219732	1.626259	0	0.695731	0	14.97377	1.004979
12424	LOC10322!	6.394001	2.742409	1.183943	2.316336	0.02054	0.043954	0	2.189647	2.782925	13.39276	14.97377	5.024897
16689	NKX2-5	2.456234	2.741299	1.77486	1.544516	0.122463	0.195068	0	0.547412	1.391463	2.232127	8.556443	2.009959
4167	LOC11962‹	26.4305	2.740881	0.522379	5.246924	1.55E-07	9.87E-07	6.505036	7.116354	6.957314	40.17829	55.61688	42.20914
15201	LOC11962:	3.044989	2.740729	1.525753	1.796313	0.072445	0.126715	1.084173	0.547412	0.695731	2.232127	10.69555	3.014938
20837	LOC10322:	0.807467	2.740523	3.048003	0.899121	0.368588	0.47013	0	0	0.695731	0	2.139111	2.009959
20838	LOC11962‹	0.807467	2.740523	3.048003	0.899121	0.368588	0.47013	0	0	0.695731	0	2.139111	2.009959
20839	LOC10324ŧ	0.807467	2.740523	3.048003	0.899121	0.368588	0.47013	0	0	0.695731	0	2.139111	2.009959
20840	LOC10322:	0.807467	2.740523	3.048003	0.899121	0.368588	0.47013	0	0	0.695731	0	2.139111	2.009959
20841	LOC10323:	0.807467	2.740523	3.048003	0.899121	0.368588	0.47013	0	0	0.695731	0	2.139111	2.009959
20842	LOC11962(	0.807467	2.740523	3.048003	0.899121	0.368588	0.47013	0	0	0.695731	0	2.139111	2.009959
20843	CLDN8	0.807467	2.740523	3.048003	0.899121	0.368588	0.47013	0	0	0.695731	0	2.139111	2.009959
20904	LOC11962‹	0.782747	2.739815	3.07943	0.889715	0.373619	0.474955	0	0.547412	0	0	2.139111	2.009959
20907	PANX3	0.782747	2.739815	3.07943	0.889715	0.373619	0.474955	0	0.547412	0	0	2.139111	2.009959
20908	LOC11962ŧ	0.782747	2.739815	3.07943	0.889715	0.373619	0.474955	0	0.547412	0	0	2.139111	2.009959
20909	LOC11961ŧ	0.782747	2.739815	3.07943	0.889715	0.373619	0.474955	0	0.547412	0	0	2.139111	2.009959
20910	TCL1B	0.782747	2.739815	3.07943	0.889715	0.373619	0.474955	0	0.547412	0	0	2.139111	2.009959
20912	LOC10323:	0.782747	2.739815	3.07943	0.889715	0.373619	0.474955	0	0.547412	0	0	2.139111	2.009959
20914	LOC10321!	0.782747	2.739815	3.07943	0.889715	0.373619	0.474955	0	0.547412	0	0	2.139111	2.009959
1194	CNIH2	117.5943	2.739781	0.269556	10.16404	2.87E-24	6.39E-23	30.35684	29.01283	32.69937	236.6055	166.8506	210.0407
12558	LOC11962ŧ	5.598321	2.739262	1.195175	2.291934	0.021909	0.046383	0.542086	1.094824	2.782925	17.85702	4.278221	7.034856
214	LOC10323:	573.5552	2.737751	0.138891	19.71153	1.72E-86	2.13E-84	150.7	149.9908	148.8865	973.2074	941.2087	1077.338
16111	NR1I3	3.083792	2.736907	1.665629	1.643167	0.100348	0.165568	0	1.642235	0.695731	4.464254	10.69555	1.004979
361	VSIR	435.2412	2.736868	0.168432	16.24911	2.27E-59	1.67E-57	119.259	96.89189	125.9274	743.2983	690.9327	835.1379
7792	GRM4	23.07436	2.736506	0.789119	3.467797	0.000525	0.00179	0.542086	3.831883	13.91463	49.10679	44.92132	26.12947
187	EEPD1	986.9771	2.736147	0.132766	20.60876	2.29E-94	3.26E-92	272.1274	282.4645	217.0682	1703.113	1681.341	1765.749

17573	CUNH11or	2.493262	2.735983	1.948361	1.404249	0.160245	0.242396	0	0.547412	1.391463	4.464254	8.556443	0
17574	GC	2.493262	2.735983	1.948361	1.404249	0.160245	0.242396	0	0.547412	1.391463	4.464254	8.556443	0
17575	KLRF2	2.493262	2.735983	1.948361	1.404249	0.160245	0.242396	0	0.547412	1.391463	4.464254	8.556443	0
14472	LOC11961	4.117676	2.735619	1.41737	1.930067	0.053599	0.09846	0	0.547412	2.782925	8.928508	6.417332	6.029877
17222	LOC11961	3.052787	2.735425	1.868721	1.463795	0.14325	0.22113	0	1.642235	0.695731	0	14.97377	1.004979
8294	RASD1	13.52991	2.735229	0.825206	3.314602	0.000918	0.002942	2.168345	2.189647	6.261582	17.85702	40.6431	12.05975
19204	LOC11962	30.12902	2.735063	2.373816	1.15218	0.249247	0.345045	0	0	23.65487	42.41041	85.56443	29.1444
4710	IFITM10	646.7748	2.734988	0.560615	4.87855	1.07E-06	6.03E-06	219.545	63.49977	224.0255	1180.795	864.2007	1328.583
14151	LOC11962	3.852456	2.734965	1.377668	1.985214	0.047121	0.088524	1.084173	0.547412	1.391463	2.232127	12.83466	5.024897
17521	LOC11961	2.439844	2.734753	1.937161	1.411732	0.158029	0.239678	0.542086	0	1.391463	0	10.69555	2.009959
13877	ASCL1	7.830385	2.734576	1.344462	2.033955	0.041956	0.080378	0	0	6.261582	11.16063	23.53022	6.029877
7950	LOC11962	16.21345	2.734307	0.798447	3.424533	0.000616	0.002059	1.626259	2.189647	9.044508	24.5534	42.78221	17.08465
9947	LOC10322	10.11784	2.734172	0.950648	2.876114	0.004026	0.01076	3.794605	0.547412	3.478657	22.32127	23.53022	7.034856
14588	FERD3L	4.065145	2.73412	1.432127	1.909132	0.056245	0.1025	0	0.547412	2.782925	4.464254	8.556443	8.039836
4756	DMKN	25.92334	2.73145	0.564337	4.840103	1.30E-06	7.25E-06	5.96295	7.116354	6.957314	58.0353	51.33866	26.12947
15042	PCDHB1	5.21294	2.731268	1.496157	1.825522	0.067922	0.12004	0	0	4.174388	13.39276	10.69555	3.014938
3695	LOC10323	30.76057	2.729765	0.483633	5.644287	1.66E-08	1.19E-07	8.673382	6.568942	9.044508	55.80317	49.19955	55.27387
15117	LOC10321	3.94379	2.729729	1.50779	1.810417	0.070231	0.12351	0	1.642235	1.391463	8.928508	10.69555	1.004979
17847	NPBWR2	2.477759	2.72854	1.992734	1.369245	0.170923	0.254607	0	0.547412	1.391463	2.232127	10.69555	0
11934	TMPRSS7	7.822018	2.727711	1.127994	2.418197	0.015598	0.034746	2.168345	1.094824	2.782925	8.928508	29.94755	2.009959
19075	GPR26	18.51934	2.727677	2.324142	1.173627	0.240544	0.335249	0	0	14.61036	42.41041	47.06043	7.034856
3346	LOC10323	37.1696	2.727038	0.454634	5.998312	1.99E-09	1.58E-08	8.131296	12.59047	8.348776	75.89232	57.75599	60.29877
7879	HOXD12	17.86541	2.726843	0.79117	3.446595	0.000568	0.001915	1.626259	2.189647	10.43597	26.78552	47.06043	19.09461
1484	DDC	106.6233	2.726544	0.293427	9.292072	1.51E-20	2.71E-19	27.6464	21.34906	35.4823	160.7131	209.6328	184.9162
10654	LOC10321	7.592872	2.726303	1.008802	2.702516	0.006882	0.017172	0.542086	3.284471	2.087194	15.62489	14.97377	9.044815
5108	RARB	35.84464	2.726107	0.589519	4.62429	3.76E-06	1.96E-05	14.09425	9.306001	4.174388	73.66019	87.70354	26.12947
6551	LOC10322	15.00627	2.725077	0.695286	3.91936	8.88E-05	0.00036	3.252518	3.831883	4.87012	17.85702	32.08666	28.13943
13492	CDH4	7.659694	2.724413	1.292179	2.108387	0.034998	0.068953	0	0.547412	5.565851	11.16063	25.66933	3.014938
859	LOC11961	156.5352	2.723954	0.232133	11.73444	8.49E-32	2.63E-30	47.7036	37.224	38.26523	285.7123	260.9715	269.3345
16766	FAM184B	3.336869	2.722981	1.779171	1.530478	0.125899	0.199609	0.542086	0	2.087194	6.696381	10.69555	0
7444	LOC11962	12.89303	2.722491	0.759658	3.583837	0.000339	0.001209	1.626259	4.379295	4.174388	24.5534	23.53022	19.09461
10223	CUNH1orf	7.79253	2.72243	0.969217	2.808898	0.004971	0.012927	1.626259	1.094824	3.478657	13.39276	17.11289	10.04979
16400	LOC11961	3.782579	2.722237	1.713852	1.588374	0.112202	0.181883	0	2.189647	0.695731	13.39276	6.417332	0
7738	SEMA3G	23.89618	2.722099	0.781937	3.481227	0.000499	0.001715	1.084173	2.737059	15.30609	44.64254	53.47777	26.12947
14099	LOC10321	5.34781	2.721795	1.364593	1.994583	0.046088	0.086904	0	2.737059	1.391463	6.696381	19.252	2.009959
12652	AIPL1	9.373667	2.721297	1.198604	2.270388	0.023184	0.048715	0	0.547412	6.957314	22.32127	21.39111	5.024897
12013	PON3	7.39552	2.72024	1.131465	2.404176	0.016209	0.035871	0	4.379295	1.391463	17.85702	10.69555	10.04979
8440	PNMT	13.44144	2.720225	0.83144	3.271705	0.001069	0.003367	1.626259	2.189647	6.957314	37.94616	12.83466	19.09461
2459	COL22A1	66.90887	2.719977	0.378673	7.182915	6.82E-13	7.38E-12	13.01007	18.06459	21.56767	127.2312	141.1813	80.39836
7920	GPR55	38.38533	2.719775	0.792254	3.432959	0.000597	0.002004	0.542086	4.379295	25.74206	58.0353	98.39909	43.21412
19059	LOC11962	1.448753	2.719752	2.312918	1.175897	0.239636	0.334264	1.084173	0	0	4.464254	2.139111	1.004979
3853	LOC10321	29.8461	2.719091	0.494745	5.49595	3.89E-08	2.68E-07	9.215468	6.568942	7.653045	55.80317	55.61688	44.2191
12446	LOC11962	9.21068	2.718571	1.176154	2.311408	0.02081	0.044449	1.084173	0	6.261582	26.78552	17.11289	4.019918
19570	LOC10323	1.397997	2.71785	2.496759	1.088551	0.276352	0.375412	0	1.094824	0	0	4.278221	3.014938
11368	LOC11962	7.151244	2.717722	1.067984	2.544723	0.010936	0.025576	0.542086	1.094824	4.174388	8.928508	17.11289	11.05477
16561	HOPX	4.32796	2.717718	1.737998	1.563705	0.117887	0.189251	0	0	3.478657	2.232127	19.252	1.004979
13372	LOC10323	6.585955	2.717459	1.274752	2.131755	0.033027	0.065661	0	1.094824	4.174388	6.696381	23.53022	4.019918
9309	BCL6B	9.279199	2.717368	0.896481	3.03115	0.002436	0.006959	2.168345	1.642235	3.478657	20.08914	19.252	9.044815
16596	CLIC2	3.248198	2.71617	1.743304	1.558058	0.119219	0.190977	0	0.547412	2.087194	0	12.83466	4.019918
21013	LOC11962	0.818887	2.715922	3.093766	0.877869	0.380015	0.48076	0.542086	0	0	2.232127	2.139111	0
21014	LOC11962	0.818887	2.715922	3.093766	0.877869	0.380015	0.48076	0.542086	0	0	2.232127	2.139111	0
20997	LOC11961	0.819775	2.715868	3.0927	0.878154	0.37986	0.480632	0	0.547412	0	2.232127	2.139111	0
20999	LINC02868	0.819775	2.715868	3.0927	0.878154	0.37986	0.480632	0	0.547412	0	2.232127	2.139111	0
21000	LOC11962	0.819775	2.715868	3.0927	0.878154	0.37986	0.480632	0	0.547412	0	2.232127	2.139111	0
21001	PLSCR5	0.819775	2.715868	3.0927	0.878154	0.37986	0.480632	0	0.547412	0	2.232127	2.139111	0
21002	LOC11962	0.819775	2.715868	3.0927	0.878154	0.37986	0.480632	0	0.547412	0	2.232127	2.139111	0
21004	LOC10323	0.819775	2.715868	3.0927	0.878154	0.37986	0.480632	0	0.547412	0	2.232127	2.139111	0
21005	LOC10324	0.819775	2.715868	3.0927	0.878154	0.37986	0.480632	0	0.547412	0	2.232127	2.139111	0
21009	KRTAP19-5	0.819775	2.715868	3.0927	0.878154	0.37986	0.480632	0	0.547412	0	2.232127	2.139111	0
21010	LOC10323	0.819775	2.715868	3.0927	0.878154	0.37986	0.480632	0	0.547412	0	2.232127	2.139111	0
2907	LOC10324	43.32542	2.715684	0.415387	6.537723	6.25E-11	5.71E-10	11.9259	9.306001	13.2189	84.82083	66.31243	74.36848
18649	IRX1	16.61378	2.715496	2.201707	1.23336	0.217442	0.309973	0	0	13.2189	40.17829	34.22577	12.05975
244	IFIT3	490.7448	2.714377	0.145412	18.66674	9.23E-78	1.01E-75	130.1007	135.7581	123.1445	897.315	797.8883	860.2624
20928	LOC11962	0.844495	2.714371	3.063689	0.885981	0.375628	0.47673	0	0	0.695731	2.232127	2.139111	0
20931	LOC11962	0.844495	2.714371	3.063689	0.885981	0.375628	0.47673	0	0	0.695731	2.232127	2.139111	0
20932	RETNLB	0.844495	2.714371	3.063689	0.885981	0.375628	0.47673	0	0	0.695731	2.232127	2.139111	0
20933	CUNH3orf	0.844495	2.714371	3.063689	0.885981	0.375628	0.47673	0	0	0.695731	2.232127	2.139111	0
20935	LOC11962	0.844495	2.714371	3.063689	0.885981	0.375628	0.47673	0	0	0.695731	2.232127	2.139111	0
20936	LOC11962	0.844495	2.714371	3.063689	0.885981	0.375628	0.47673	0	0	0.695731	2.232127	2.139111	0
20938	LOC11961	0.844495	2.714371	3.063689	0.885981	0.375628	0.47673	0	0	0.695731	2.232127	2.139111	0
20940	MAGEB1	0.844495	2.714371	3.063689	0.885981	0.375628	0.47673	0	0	0.695731	2.232127	2.139111	0
20941	LOC11962	0.844495	2.714371	3.063689	0.885981	0.375628	0.47673	0	0	0.695731	2.232127	2.139111	0
20943	NAA10	0.844495	2.714371	3.063689	0.885981	0.375628	0.47673	0	0	0.695731	2.232127	2.139111	0
20945	AMTN	0.844495	2.714371	3.063689	0.885981	0.375628	0.47673	0	0	0.695731	2.232127	2.139111	0

20946	LOC11962	0.844495	2.714371	3.063689	0.885981	0.375628	0.47673	0	0	0.695731	2.232127	2.139111	0
20947	LOC10321	0.844495	2.714371	3.063689	0.885981	0.375628	0.47673	0	0	0.695731	2.232127	2.139111	0
7213	PDYN	12.87894	2.713585	0.740841	3.662844	0.000249	0.000919	2.710432	2.737059	4.87012	22.32127	23.53022	21.10457
9321	L1TD1	11.89036	2.712822	0.896644	3.025529	0.002482	0.007079	2.168345	1.094824	6.261582	13.39276	36.36488	12.05975
16716	NMU	2.25622	2.711845	1.761514	1.539497	0.123683	0.196704	1.084173	0	0.695731	4.464254	4.278221	3.014938
849	IL1R2	152.854	2.710883	0.230085	11.78211	4.83E-32	1.51E-30	41.19856	37.77142	42.43961	256.6946	288.7799	250.2399
19079	LOC11962	1.450528	2.710782	2.311911	1.172528	0.240985	0.335775	0	1.094824	0	4.464254	2.139111	1.004979
5897	AATK	25.77583	2.710267	0.64288	4.215823	2.49E-05	0.000112	2.168345	6.568942	11.82743	51.33892	55.61688	27.13445
14302	LOC11962	3.638714	2.708406	1.381301	1.960764	0.049907	0.092768	1.084173	1.094824	0.695731	2.232127	10.69555	6.029877
4164	LOC11961	29.29855	2.707977	0.515957	5.24845	1.53E-07	9.79E-07	5.420864	8.758589	9.044508	51.33892	62.03421	39.1942
18597	LOC10322	1.630436	2.707905	2.190989	1.235928	0.216485	0.309472	0	0	1.391463	2.232127	2.139111	4.019918
15250	LOC11962	4.128833	2.707633	1.513962	1.788441	0.073705	0.128481	0.542086	0	2.782925	4.464254	14.97377	2.009959
19740	LOC11961	1.417747	2.707492	2.549685	1.061893	0.288284	0.388249	1.084173	0	0	0	6.417332	1.004979
16232	CUNH3orf	2.257107	2.707274	1.669963	1.621158	0.104984	0.171944	0.542086	0.547412	0.695731	4.464254	4.278221	3.014938
8471	LOC11961	11.2727	2.707094	0.829811	3.262302	0.001105	0.003468	1.084173	4.379295	3.478657	20.08914	23.53022	15.07469
19110	LDLRAD1	1.43325	2.706708	2.318679	1.167349	0.243069	0.338148	1.084173	0	0	2.232127	4.278221	1.004979
6752	BPIFC	16.72096	2.705682	0.705457	3.835361	0.000125	0.000494	2.168345	5.474118	5.565851	37.94616	32.08666	17.08465
21653	LOC10323	0.803385	2.704818	3.455698	0.782713	0.433796	0.532477	0.542086	0	0	0	4.278221	0
21654	LOC10323	0.803385	2.704818	3.455698	0.782713	0.433796	0.532477	0.542086	0	0	0	4.278221	0
21655	FANCD2OS	0.803385	2.704818	3.455698	0.782713	0.433796	0.532477	0.542086	0	0	0	4.278221	0
21657	LOC11961	0.803385	2.704818	3.455698	0.782713	0.433796	0.532477	0.542086	0	0	0	4.278221	0
21658	LOC11962	0.803385	2.704818	3.455698	0.782713	0.433796	0.532477	0.542086	0	0	0	4.278221	0
21659	LOC11962	0.803385	2.704818	3.455698	0.782713	0.433796	0.532477	0.542086	0	0	0	4.278221	0
21641	LOC11962	0.804272	2.704777	3.454358	0.783004	0.433625	0.532418	0	0.547412	0	0	4.278221	0
21642	LOC10322	0.804272	2.704777	3.454358	0.783004	0.433625	0.532418	0	0.547412	0	0	4.278221	0
21643	LOC11962	0.804272	2.704777	3.454358	0.783004	0.433625	0.532418	0	0.547412	0	0	4.278221	0
21644	LOC10324	0.804272	2.704777	3.454358	0.783004	0.433625	0.532418	0	0.547412	0	0	4.278221	0
21645	LOC11961	0.804272	2.704777	3.454358	0.783004	0.433625	0.532418	0	0.547412	0	0	4.278221	0
21646	LOC10323	0.804272	2.704777	3.454358	0.783004	0.433625	0.532418	0	0.547412	0	0	4.278221	0
21648	LOC11962	0.804272	2.704777	3.454358	0.783004	0.433625	0.532418	0	0.547412	0	0	4.278221	0
21651	LOC10324	0.804272	2.704777	3.454358	0.783004	0.433625	0.532418	0	0.547412	0	0	4.278221	0
21652	LOC10324	0.804272	2.704777	3.454358	0.783004	0.433625	0.532418	0	0.547412	0	0	4.278221	0
7026	BEST3	16.1648	2.704473	0.726039	3.72497	0.000195	0.000739	3.794605	2.737059	6.261582	40.17829	29.94755	14.06971
21582	LOC11962	0.828992	2.703626	3.417933	0.791012	0.428937	0.528028	0	0	0.695731	0	4.278221	0
21583	LOC11962	0.828992	2.703626	3.417933	0.791012	0.428937	0.528028	0	0	0.695731	0	4.278221	0
21584	LOC11962	0.828992	2.703626	3.417933	0.791012	0.428937	0.528028	0	0	0.695731	0	4.278221	0
21585	LOC11962	0.828992	2.703626	3.417933	0.791012	0.428937	0.528028	0	0	0.695731	0	4.278221	0
21586	LOC11962	0.828992	2.703626	3.417933	0.791012	0.428937	0.528028	0	0	0.695731	0	4.278221	0
21587	LOC11961	0.828992	2.703626	3.417933	0.791012	0.428937	0.528028	0	0	0.695731	0	4.278221	0
21588	RPL10L	0.828992	2.703626	3.417933	0.791012	0.428937	0.528028	0	0	0.695731	0	4.278221	0
21589	LOC11961	0.828992	2.703626	3.417933	0.791012	0.428937	0.528028	0	0	0.695731	0	4.278221	0
21590	LOC11962	0.828992	2.703626	3.417933	0.791012	0.428937	0.528028	0	0	0.695731	0	4.278221	0
21591	TAS2R38	0.828992	2.703626	3.417933	0.791012	0.428937	0.528028	0	0	0.695731	0	4.278221	0
21592	CRX	0.828992	2.703626	3.417933	0.791012	0.428937	0.528028	0	0	0.695731	0	4.278221	0
21593	LOC11962	0.828992	2.703626	3.417933	0.791012	0.428937	0.528028	0	0	0.695731	0	4.278221	0
21594	LOC10323	0.828992	2.703626	3.417933	0.791012	0.428937	0.528028	0	0	0.695731	0	4.278221	0
21595	PVALEF	0.828992	2.703626	3.417933	0.791012	0.428937	0.528028	0	0	0.695731	0	4.278221	0
21596	LOC10324	0.828992	2.703626	3.417933	0.791012	0.428937	0.528028	0	0	0.695731	0	4.278221	0
9073	FAM9B	11.08489	2.701286	0.87415	3.090186	0.002	0.005861	3.794605	2.737059	2.087194	15.62489	34.22577	8.039836
7812	POU3F2	22.38762	2.700789	0.779963	3.462713	0.000535	0.00182	1.084173	4.379295	12.52316	44.64254	55.61688	16.07967
12630	TMC1	5.412128	2.700546	1.187068	2.274972	0.022908	0.048218	0.542086	1.642235	2.087194	15.62489	8.556443	4.019918
18810	LOC10323	17.32823	2.700248	2.221128	1.21571	0.224095	0.316724	0	0	13.91463	26.78552	49.19955	14.06971
16718	TMEM252	2.309638	2.699903	1.754209	1.5391	0.12378	0.196835	0.542086	0.547412	0.695731	8.928508	2.139111	1.004979
16836	TRIM67	20.57643	2.699241	1.777581	1.518491	0.128891	0.203526	0	0.547412	16.00182	26.78552	62.03421	18.08963
6278	LOC10321	24.54344	2.69894	0.668397	4.03793	5.39E-05	0.000228	2.168345	7.663765	9.740239	42.41041	64.17332	21.10457
16889	SERPINA9	2.242492	2.698537	1.788963	1.508437	0.131443	0.206892	0	1.094824	0.695731	2.232127	6.417332	3.014938
19132	LOC10321	1.435025	2.697717	2.317665	1.16398	0.244432	0.339617	0	1.094824	0	2.232127	4.278221	1.004979
19133	HIPK4	1.435025	2.697717	2.317665	1.16398	0.244432	0.339617	0	1.094824	0	2.232127	4.278221	1.004979
19134	LOC11962	1.435025	2.697717	2.317665	1.16398	0.244432	0.339617	0	1.094824	0	2.232127	4.278221	1.004979
19765	CUNH5orf	1.419523	2.697711	2.548654	1.058485	0.289835	0.389843	0	1.094824	0	0	6.417332	1.004979
15289	LOC11962	3.714545	2.697162	1.513939	1.781552	0.074822	0.130103	0	2.189647	0.695731	6.696381	10.69555	2.009959
4641	LOC11962	38.89728	2.696512	0.547673	4.923584	8.50E-07	4.87E-06	8.131296	8.211177	14.61036	75.89232	98.39909	28.13943
18576	LOC10322	1.667464	2.695108	2.176112	1.238497	0.215532	0.308427	0	0	1.391463	4.464254	2.139111	2.009959
18578	NANOS2	1.667464	2.695108	2.176112	1.238497	0.215532	0.308427	0	0	1.391463	4.464254	2.139111	2.009959
321	LOC11962	737.8293	2.694573	0.159379	16.90675	4.01E-64	3.32E-62	169.673	244.6931	177.4115	1316.955	1227.85	1290.394
5872	LOC11962	42.85329	2.69426	0.637104	4.228914	2.35E-05	0.000106	2.710432	8.211177	23.65487	84.82083	100.5382	37.18424
14872	HDGFL1	4.57879	2.693913	1.448961	1.859204	0.062998	0.112615	1.084173	1.094824	1.391463	8.928508	14.97377	0
20174	DUSP29	1.719995	2.693292	2.691937	1.000503	0.317067	0.417753	0	0	1.391463	8.928508	0	0
14722	ASB15	3.946984	2.691258	1.427711	1.885016	0.059428	0.107317	0	1.094824	2.087194	8.928508	8.556443	3.014938
5172	LOC11962	27.96089	2.690727	0.58576	4.593568	4.36E-06	2.24E-05	7.589209	4.926706	9.740239	62.49956	59.8951	23.11453
3444	LDHD	48.2036	2.690192	0.455375	5.907641	3.47E-09	2.68E-08	14.09425	6.568942	18.78475	73.66019	70.59065	105.5228
17279	LOC11962	2.474931	2.690039	1.850754	1.453483	0.14609	0.22477	0	0	2.087194	4.464254	4.278221	4.019918
7195	CTCFL	15.05057	2.689572	0.733129	3.66862	0.000244	0.000901	3.794605	3.284471	4.87012	26.78552	38.50399	13.06473
12341	LOC10322	7.018469	2.689125	1.151144	2.336046	0.019489	0.041981	0	2.189647	3.478657	11.16063	19.252	6.029877

15397	LOC103234	3.423197	2.687053	1.526794	1.759931	0.07842	0.135402	2.168345	0.547412	0	2.232127	8.556443	7.034856
14219	LOC119622	6.647703	2.686153	1.361095	1.973524	0.048436	0.09056	0	0.547412	4.87012	8.928508	23.53022	2.009959
7901	GRIN1	24.74759	2.685742	0.781057	3.4386	0.000585	0.001967	1.084173	4.379295	14.61036	53.57105	57.75599	17.08465
6892	LOC103245	24.79255	2.685176	0.710634	3.778565	0.000158	0.000608	4.878777	9.853413	4.87012	37.94616	79.14709	12.05975
14758	PNPLA5	4.648502	2.684838	1.428065	1.880053	0.060101	0.108273	0.542086	0.547412	2.782925	0	14.97377	9.044815
11206	LOC103234	9.348648	2.684756	1.039986	2.581532	0.009836	0.023336	1.626259	0.547412	5.565851	4.464254	27.80844	16.07967
7767	MLPH	16.10253	2.684663	0.773025	3.47293	0.000515	0.001762	3.794605	2.189647	6.957314	24.5534	47.06043	12.05975
13879	FCRLA	4.482156	2.684514	1.32009	2.033584	0.041994	0.080438	0	1.642235	2.087194	6.696381	6.417332	10.04979
474	ZBTB7C	284.0414	2.683409	0.180579	14.86	5.99E-50	3.36E-48	68.30288	83.2066	78.61764	470.9788	485.5781	517.5644
18688	LOC119625	1.651962	2.683299	2.180672	1.230492	0.218513	0.3108	0	0	1.391463	2.232127	4.278221	2.009959
18689	LOC103233	1.651962	2.683299	2.180672	1.230492	0.218513	0.3108	0	0	1.391463	2.232127	4.278221	2.009959
18690	LOC103237	1.651962	2.683299	2.180672	1.230492	0.218513	0.3108	0	0	1.391463	2.232127	4.278221	2.009959
18691	LOC119618	1.651962	2.683299	2.180672	1.230492	0.218513	0.3108	0	0	1.391463	2.232127	4.278221	2.009959
10084	LOC103232	14.26938	2.683209	0.94438	2.841238	0.004494	0.011849	0	3.284471	8.348776	24.5534	36.36488	13.06473
16164	KCNG2	3.106737	2.682859	1.641965	1.633932	0.102273	0.168209	1.084173	0	1.391463	4.464254	10.69555	1.004979
772	NRN1	172.9064	2.682743	0.217991	12.30666	8.34E-35	2.87E-33	41.19856	49.26706	49.39693	299.105	308.0319	290.4391
17383	POU3F4	2.511959	2.682405	1.872887	1.43223	0.152078	0.232583	0	0	2.087194	6.696381	4.278221	2.009959
6649	LOC103224	16.04854	2.682194	0.691454	3.879065	0.000105	0.000419	5.96295	3.284471	3.478657	31.24978	34.22577	18.08963
10524	LOC103223	10.58119	2.681719	0.979183	2.738732	0.006168	0.015579	1.626259	3.284471	3.478657	17.85702	34.22577	3.014938
14794	CYP3A7	3.709055	2.681636	1.430697	1.874357	0.060881	0.109404	1.626259	0	1.391463	8.928508	4.278221	6.029877
14980	LOC103229	3.915979	2.681432	1.458746	1.838177	0.066036	0.117195	0	1.094824	2.087194	4.464254	12.83466	3.014938
11025	GATA1	18.33226	2.681146	1.021709	2.624179	0.008686	0.020944	0	1.094824	13.91463	42.41041	38.50399	14.06971
546	KLF9	474.0977	2.681088	0.189564	14.14342	2.05E-45	9.98E-44	114.9223	156.0124	113.4042	805.7978	686.6545	967.7952
19521	LOC119627	38.61932	2.680721	2.442088	1.097717	0.272328	0.370875	0	0	31.30791	66.96381	96.25998	37.18424
19491	LOC103247	1.704492	2.680628	2.432665	1.101931	0.270492	0.368922	0	0	1.391463	6.696381	2.139111	0
18988	LOC119619	2.115646	2.680301	2.258242	1.186897	0.235268	0.329398	1.626259	0	0	8.928508	2.139111	0
4882	LOC119623	29.33446	2.679681	0.563987	4.75132	2.02E-06	1.10E-05	3.794605	8.758589	11.1317	60.26743	59.8951	32.15934
12564	NRK	10.84263	2.679524	1.169941	2.290306	0.022004	0.046555	0	0.547412	8.348776	22.32127	27.80844	6.029877
11197	TNNT3	9.467529	2.678084	1.03695	2.582655	0.009804	0.023276	0.542086	1.642235	5.565851	8.928508	32.08666	8.039836
18101	LOC103246	2.248515	2.677896	2.0279	1.320526	0.186659	0.274147	0	1.094824	0.695731	0	10.69555	1.004979
13638	KCNK17	4.338388	2.677553	1.286443	2.081361	0.037401	0.072907	0.542086	2.189647	0.695731	11.16063	6.417332	5.024897
1586	TREM1	100.1955	2.676638	0.297156	9.007526	2.11E-19	3.53E-18	23.30971	34.48694	23.65487	171.8738	151.8769	195.971
16555	CEACAM7	3.356454	2.676069	1.710368	1.564616	0.117673	0.188955	0	0	2.782925	8.928508	6.417332	2.009959
10354	TEX35	11.04182	2.67535	0.963883	2.775597	0.00551	0.014148	0.542086	2.189647	6.261582	24.5534	25.66933	7.034856
4037	LOC119622	903.9871	2.673665	0.500209	5.345091	9.04E-08	5.95E-07	160.4576	193.2364	381.2608	1732.131	2017.181	939.6558
8298	CCDC187	14.38414	2.673546	0.806918	3.313282	0.000922	0.002954	1.084173	4.379295	6.261582	17.85702	40.6431	16.07967
7110	GAB3	17.45727	2.672932	0.723747	3.693186	0.000221	0.000828	6.505036	3.831883	3.478657	44.64254	34.22577	12.05975
16602	LOC119624	3.266895	2.672862	1.716985	1.556719	0.119537	0.191416	0	0	2.782925	2.232127	8.556443	6.029877
7970	CFAP126	12.8275	2.671773	0.781456	3.418967	0.000629	0.002097	1.626259	3.284471	5.565851	29.01765	21.39111	16.07967
16314	LOC103232	30.11974	2.671624	1.666884	1.602765	0.108986	0.177602	0	1.642235	22.95914	75.89232	47.06043	33.16432
18074	LOC119619	2.095009	2.671365	2.010531	1.328686	0.183951	0.270574	1.084173	0.547412	0	8.928508	0	2.009959
19013	LOC103247	2.118309	2.669978	2.257774	1.182571	0.236979	0.331357	0	1.642235	0	8.928508	2.139111	0
10342	CAPN9	8.150725	2.669826	0.960754	2.778886	0.005455	0.014021	1.626259	1.642235	3.478657	6.696381	21.39111	14.06971
16585	LOC119622	3.340951	2.669732	1.711628	1.559762	0.118816	0.190457	0	0	2.782925	6.696381	8.556443	2.009959
10210	LOC103230	7.712249	2.669274	0.948499	2.814208	0.00489	0.012732	2.710432	2.189647	1.391463	8.928508	14.97377	16.07967
9953	PTPRO	11.74936	2.66871	0.928534	2.874111	0.004052	0.010822	1.084173	1.642235	6.957314	13.39276	36.36488	11.05477
10095	DACT2	10.87972	2.668002	0.93955	2.83966	0.004516	0.011893	1.084173	2.189647	5.565851	29.01765	21.39111	6.029877
19465	LOC119627	1.68899	2.667866	2.411135	1.106477	0.26852	0.366722	0	0	1.391463	4.464254	4.278221	0
19466	TAC4	1.68899	2.667866	2.411135	1.106477	0.26852	0.366722	0	0	1.391463	4.464254	4.278221	0
11571	ATP6V1B1	10.50744	2.667597	1.066559	2.501125	0.01238	0.028444	0.542086	0.547412	7.653045	11.16063	32.08666	11.05477
4768	LIMS2	25.14991	2.666826	0.552007	4.83115	1.36E-06	7.57E-06	5.420864	5.474118	9.740239	53.57105	38.50399	38.18922
10027	LOC103224	10.61765	2.666767	0.934003	2.855202	0.004301	0.011403	1.626259	5.474118	1.391463	8.928508	34.22577	12.05975
12046	UNC45B	10.54535	2.665804	1.111769	2.397804	0.016494	0.036401	0	1.094824	7.653045	13.39276	32.08666	9.044815
16076	SLC22A6	4.074362	2.665634	1.613736	1.65184	0.098567	0.162984	0	0	3.478657	2.232127	10.69555	8.039836
16078	LOC103243	4.074362	2.665634	1.613736	1.65184	0.098567	0.162984	0	0	3.478657	2.232127	10.69555	8.039836
15941	PNMA5	29.49569	2.665553	1.588968	1.677537	0.093438	0.155827	0	3.284471	20.87194	53.57105	79.14709	20.09959
56	HMOX1	5517.724	2.664793	0.092911	28.68125	6.54E-181	3.10E-178	1507.542	1462.137	1542.436	9263.327	9056.995	10273.91
14702	LOC103225	3.783998	2.663413	1.408902	1.890418	0.058702	0.106149	1.084173	0.547412	1.391463	13.39276	4.278221	2.009959
1580	PRODH2	132.7006	2.663261	0.294785	9.034582	1.65E-19	2.77E-18	42.82482	21.89647	44.52681	216.5163	218.1893	252.2498
11054	CD6	12.85641	2.662691	1.01833	2.614763	0.008929	0.021474	1.626259	0	9.044508	22.32127	32.08666	12.05975
6060	KMO	19.91193	2.662019	0.642812	4.141211	3.45E-05	0.000152	5.96295	3.284471	6.957314	51.33892	27.80844	24.11951
8221	PGC	12.82227	2.66188	0.796761	3.340876	0.000835	0.002701	2.710432	2.189647	5.565851	22.32127	32.08666	12.05975
375	SLC16A2	478.005	2.661652	0.165311	16.10088	2.51E-58	1.78E-56	110.5856	143.4219	138.4505	850.4404	733.715	891.4168
10950	TOPAZ1	8.860137	2.661643	1.00849	2.639237	0.008309	0.020174	0.542086	3.831883	2.782925	11.16063	27.80844	7.034856
1261	COL14A1	131.7609	2.661297	0.266905	9.970951	2.04E-23	4.31E-22	29.27266	35.58177	43.13534	212.0521	269.5279	200.9959
16981	PVALB	2.260302	2.660571	1.779041	1.495508	0.134782	0.211011	0.542086	0	1.391463	4.464254	2.139111	5.024897
19445	KDF1	1.636459	2.660238	2.396093	1.11024	0.266896	0.364803	0	0	1.391463	0	6.417332	2.009959
19446	LOC103236	1.636459	2.660238	2.396093	1.11024	0.266896	0.364803	0	0	1.391463	0	6.417332	2.009959
19448	LOC119618	1.636459	2.660238	2.396093	1.11024	0.266896	0.364803	0	0	1.391463	0	6.417332	2.009959
17294	KCNB2	4.206971	2.660155	1.834783	1.449847	0.147101	0.22613	0	0	3.478657	8.928508	12.83466	0
10793	LOC119622	13.64501	2.660152	0.995557	2.672023	0.00754	0.018572	0	1.642235	9.740239	22.32127	32.08666	16.07967
11536	CCDC162P	8.088811	2.658999	1.060861	2.506453	0.012195	0.028103	1.084173	3.284471	2.087194	13.39276	25.66933	3.014938
17394	TXNDC2	3.210746	2.657979	1.858207	1.4304	0.152602	0.233237	0	0.547412	2.087194	15.62489	0	1.004979
16049	LOC103239	3.053154	2.656385	1.603025	1.657107	0.097498	0.161504	0	0.547412	2.087194	2.232127	6.417332	7.034856

6228	LOC10322	20.18639	2.656381	0.653622	4.064097	4.82E-05	0.000206	7.589209	3.831883	4.87012	31.24978	53.47777	20.09959
12578	ADGRE1	10.68461	2.656274	1.160691	2.288528	0.022107	0.046725	0	0.547412	8.348776	26.78552	21.39111	7.034856
9755	LOC10324	9.964361	2.655768	0.910871	2.915635	0.00355	0.009674	1.084173	1.642235	5.565851	22.32127	17.11289	12.05975
19599	LOC11962	1.673487	2.654905	2.443642	1.086454	0.277278	0.376052	0	0	1.391463	2.232127	6.417332	0
19600	LOC11962	1.673487	2.654905	2.443642	1.086454	0.277278	0.376052	0	0	1.391463	2.232127	6.417332	0
19603	TSPAN16	1.673487	2.654905	2.443642	1.086454	0.277278	0.376052	0	0	1.391463	2.232127	6.417332	0
19604	LOC11962	1.673487	2.654905	2.443642	1.086454	0.277278	0.376052	0	0	1.391463	2.232127	6.417332	0
17002	DCD	3.309946	2.654706	1.77973	1.491634	0.135795	0.212322	0	0	2.782925	2.232127	12.83466	2.009959
17003	LOC10323	3.309946	2.654706	1.77973	1.491634	0.135795	0.212322	0	0	2.782925	2.232127	12.83466	2.009959
11249	CD4	13.65706	2.65445	1.032671	2.570471	0.010156	0.024002	0	1.642235	9.740239	17.85702	40.6431	12.05975
17639	LINC02881	3.251393	2.652799	1.895514	1.399514	0.161659	0.243648	0	0	2.782925	0	10.69555	6.029877
57	FXYD2	17919.14	2.652678	0.092567	28.65682	1.32E-180	6.15E-178	4539.973	5333.433	4878.468	31586.83	29778.56	31397.57
2967	PCDH12	60.32286	2.652627	0.410066	6.468777	9.88E-11	8.85E-10	13.01007	12.59047	24.3506	80.35657	141.1813	90.44815
12660	LOC10322	8.026057	2.652362	1.169433	2.268075	0.023325	0.04898	0.542086	0.547412	5.565851	26.78552	10.69555	4.019918
12994	KRT20	6.581505	2.65059	1.202523	2.20419	0.027511	0.056286	0	3.284471	2.087194	6.696381	21.39111	6.029877
13099	SPEM3	8.986143	2.650493	1.21137	2.188012	0.028669	0.058184	0	0.547412	6.957314	17.85702	23.53022	5.024897
11356	FSIP2	10.61314	2.649969	1.040707	2.546317	0.010887	0.02548	0.542086	6.02153	2.087194	4.464254	38.50399	12.05975
4448	LOC11962	35.83744	2.649834	0.525333	5.044107	4.56E-07	2.72E-06	9.757555	6.02153	13.91463	37.94616	94.12087	53.26391
18777	TAS1R2	2.502479	2.649175	2.167873	1.222016	0.221702	0.313891	0	0	2.087194	2.232127	10.69555	0
17380	LOC11961	2.079506	2.648916	1.848963	1.43265	0.151958	0.23244	1.084173	0.547412	0	6.696381	2.139111	2.009959
11161	TTC9B	10.36064	2.648616	1.022704	2.589816	0.009603	0.022873	3.794605	1.094824	3.478657	37.94616	12.83466	3.014938
7303	ANKRD35	18.49152	2.646484	0.728266	3.633952	0.000279	0.001016	5.96295	2.189647	6.957314	35.71403	47.06043	13.06473
17387	LOC11961	2.080393	2.646005	1.848803	1.431199	0.152373	0.232981	0.542086	1.094824	0	6.696381	2.139111	2.009959
4084	PDK4	33.91817	2.645268	0.498296	5.308624	1.10E-07	7.19E-07	10.84173	12.04306	4.87012	46.87467	70.59065	58.28881
15930	LOC10321	3.126322	2.644792	1.574645	1.679612	0.093033	0.155259	0.542086	0	2.087194	6.696381	6.417332	3.014938
14704	LOC11962	4.800696	2.644691	1.399486	1.889758	0.05879	0.106294	0	0.547412	3.478657	8.928508	12.83466	3.014938
18033	CCDC152	2.065778	2.643691	1.977136	1.337132	0.18118	0.267102	0	1.642235	0	4.464254	4.278221	2.009959
12641	UCP3	6.073623	2.643391	1.163328	2.272266	0.02307	0.048519	0.542086	1.642235	2.782925	15.62489	12.83466	3.014938
12949	LOC10323	4.973389	2.642615	1.193801	2.213615	0.026855	0.055135	1.626259	1.642235	0.695731	11.16063	10.69555	4.019918
14414	LOC11961	3.465195	2.642411	1.361381	1.940978	0.052261	0.096389	1.084173	1.094824	0.695731	4.464254	6.417332	7.034856
5121	KLK14	39.2224	2.641434	0.571942	4.618358	3.87E-06	2.01E-05	5.420864	7.663765	19.48048	69.19594	98.39909	35.17428
14589	PADI3	3.466083	2.640494	1.383232	1.90893	0.056271	0.102541	0.542086	1.642235	0.695731	4.464254	6.417332	7.034856
7679	GPR179	29.71825	2.640326	0.754683	3.49859	0.000468	0.001619	1.084173	4.379295	19.48048	40.17829	77.00798	36.17926
20339	LOC11962	1.657984	2.639678	2.718884	0.970868	0.331614	0.433451	0	0	1.391463	0	8.556443	0
13151	NPFFR1	5.227353	2.639474	1.210384	2.18069	0.029206	0.059041	1.626259	0.547412	2.087194	13.39276	10.69555	3.014938
12969	LOC11962	6.14847	2.639005	1.194471	2.209351	0.02715	0.055655	0.542086	0.547412	4.174388	4.464254	17.11289	10.04979
12175	LOC10324	6.430471	2.638594	1.112841	2.371044	0.017738	0.038732	3.252518	0.547412	1.391463	13.39276	14.97377	5.024897
11748	LOC11961	6.810229	2.637798	1.072719	2.458983	0.013933	0.03153	0.542086	1.094824	4.174388	11.16063	12.83466	11.05477
3259	NEURL3	40.4228	2.637371	0.433254	6.08735	1.15E-09	9.36E-09	13.01007	10.94824	9.740239	53.57105	74.86887	80.39836
16030	LOC11962	3.111707	2.636126	1.587393	1.660664	0.096781	0.160507	0	0.547412	2.087194	4.464254	8.556443	3.014938
18148	LOC10323	2.050276	2.636102	2.007949	1.312833	0.189239	0.277216	0	1.642235	0	2.232127	6.417332	2.009959
197	PTX3	982.6591	2.635463	0.12936	20.3731	2.90E-92	3.91E-90	269.959	295.6024	252.5505	1589.274	1619.307	1869.262
2396	ZNF630	64.95356	2.635415	0.362126	7.277618	3.40E-13	3.77E-12	16.26259	15.32753	22.2634	133.9276	115.512	86.42823
11877	LOC11962	15.48248	2.633629	1.083135	2.431487	0.015037	0.03366	0	0.547412	12.52316	37.94616	27.80844	14.06971
1311	SYCE3	127.0575	2.632806	0.268213	9.816113	9.60E-23	1.95E-21	40.11439	30.65506	35.4823	236.6055	173.268	246.22
13768	PLK5	4.382858	2.631536	1.281564	2.053379	0.040036	0.077306	1.626259	0.547412	1.391463	11.16063	8.556443	3.014938
17445	CD3E	2.0485	2.63123	1.854073	1.419162	0.155852	0.237508	1.084173	0.547412	0	2.232127	6.417332	2.009959
6961	HPN	16.77093	2.630133	0.70082	3.752939	0.000175	0.000667	3.252518	3.284471	7.653045	40.17829	17.11289	29.1444
13240	CAPNS2	5.198123	2.629973	1.218318	2.158693	0.030874	0.061993	0.542086	1.642235	2.087194	8.928508	14.97377	3.014938
13614	IRX6	4.315712	2.629056	1.259855	2.086793	0.036907	0.072071	1.084173	1.094824	1.391463	4.464254	12.83466	5.024897
1927	LOC10322	86.02828	2.628814	0.322866	8.142117	3.88E-16	5.36E-15	21.68345	19.70683	30.61218	131.6955	181.8244	130.6473
16280	IGSF21	3.096204	2.628644	1.631166	1.611512	0.107068	0.17483	0	0.547412	2.087194	2.232127	10.69555	3.014938
16281	LOC10324	3.096204	2.628644	1.631166	1.611512	0.107068	0.17483	0	0.547412	2.087194	2.232127	10.69555	3.014938
17458	ERVFRD-1	2.049388	2.62831	1.853911	1.417711	0.156275	0.237975	0.542086	1.094824	0	2.232127	6.417332	2.009959
10786	MYLK3	8.884502	2.628127	0.983161	2.673141	0.007514	0.018521	2.710432	0.547412	4.174388	11.16063	25.66933	9.044815
10555	LOC11962	8.140821	2.627529	0.961397	2.733033	0.006275	0.015806	1.626259	1.642235	3.478657	20.08914	14.97377	7.034856
12328	CX3CR1	6.038902	2.627468	1.12334	2.338978	0.019337	0.041699	1.084173	0.547412	3.478657	13.39276	10.69555	7.034856
18593	LOC11962	1.437332	2.627337	2.125681	1.235998	0.216459	0.309452	0.542086	0	0.695731	2.232127	2.139111	3.014938
18594	LOC11962	1.437332	2.627337	2.125681	1.235998	0.216459	0.309452	0.542086	0	0.695731	2.232127	2.139111	3.014938
7839	ADRA1A	18.63495	2.627331	0.760287	3.45571	0.000549	0.001861	3.252518	3.831883	8.348776	42.41041	44.92132	9.044815
17180	LOC10321	2.267212	2.627292	1.786306	1.470796	0.141346	0.218725	0	0.547412	1.391463	2.232127	6.417332	3.014938
4835	LOC11962	26.8699	2.626065	0.547962	4.792422	1.65E-06	9.06E-06	4.336691	7.116354	11.1317	49.10679	51.33866	38.18922
6949	LOC10321	20.58106	2.625918	0.698919	3.757114	0.000172	0.000658	2.710432	6.02153	8.348776	35.71403	55.61688	15.07469
5808	LOC10323	24.30406	2.623847	0.614511	4.26981	1.96E-05	8.96E-05	6.505036	6.02153	7.653045	26.78552	72.72976	26.12947
18825	PNMA8B	13.23679	2.623827	2.160393	1.214514	0.224552	0.317116	0	0	11.1317	35.71403	23.53022	9.044815
15017	LOC11962	3.756187	2.623262	1.43305	1.830545	0.067168	0.11891	1.084173	0	2.087194	8.928508	6.417332	4.019918
18607	LOC10323	1.43822	2.623231	2.125245	1.234319	0.217084	0.309867	0	0.547412	0.695731	2.232127	2.139111	3.014938
11887	LOC10322	13.6359	2.622834	1.080025	2.428493	0.015162	0.033909	0	0.547412	11.1317	20.08914	29.94755	20.09959
8738	ECEL1	14.38382	2.622155	0.821414	3.192244	0.001412	0.004295	1.084173	2.737059	8.348776	31.24978	27.80844	15.07469
17331	CNGA2	2.319743	2.621534	1.816064	1.443525	0.148873	0.228351	0	0.547412	1.391463	6.696381	4.278221	1.004979
12231	APCDD1L	12.02699	2.621372	1.110629	2.36026	0.018262	0.039694	0	0.547412	9.740239	17.85702	29.94755	14.06971
5123	XDH	24.51465	2.620199	0.567455	4.617456	3.88E-06	2.02E-05	4.878777	6.568942	9.044508	55.80317	40.6431	30.14938
14264	MAP10	4.623095	2.620146	1.332018	1.96705	0.049177	0.091656	0.542086	0.547412	2.782925	11.16063	10.69555	2.009959
596	ZNF528	239.2293	2.618369	0.191964	13.63986	2.32E-42	1.03E-40	61.79785	73.35318	65.39875	406.2471	440.6568	387.9221

14382	POU4F3	5.384941	2.61751	1.343696	1.947993	0.051416	0.095042	0	1.094824	3.478657	4.464254	19.252	4.019918
4289	NEURL1	53.538	2.617178	0.506919	5.162906	2.43E-07	1.51E-06	9.757555	7.663765	27.82925	93.74933	121.9293	60.29877
17287	LOC10323!	2.981501	2.616789	1.803227	1.45117	0.146732	0.225654	0	1.094824	1.391463	13.39276	0	2.009959
158	NCAM1	7270.473	2.615837	0.120577	21.69432	2.32E-104	3.91E-102	1904.892	2395.474	1817.946	12182.95	11869.93	13451.65
15168	FGF8	3.576279	2.615504	1.451395	1.802062	0.071536	0.125377	1.084173	1.094824	0.695731	11.16063	6.417332	1.004979
16443	LIPM	3.512493	2.615376	1.653787	1.581447	0.113776	0.183953	2.168345	0	0.695731	2.232127	14.97377	1.004979
13293	SLC45A2	6.0467	2.614286	1.216254	2.149457	0.031598	0.063194	0	1.642235	3.478657	11.16063	14.97377	5.024897
11780	LOC11961ł	6.602509	2.613816	1.065492	2.453155	0.014161	0.031958	0.542086	1.642235	3.478657	8.928508	14.97377	10.04979
18403	LOC11962:	112.5302	2.613798	2.067723	1.264095	0.206196	0.297871	0	1.642235	93.228	220.9806	258.8324	100.4979
17361	GZMH	2.30424	2.613018	1.818136	1.437196	0.150662	0.23071	0	0.547412	1.391463	4.464254	6.417332	1.004979
14803	PTCRA	5.369439	2.612784	1.395669	1.872066	0.061197	0.109906	0	1.094824	3.478657	2.232127	21.39111	4.019918
19363	LOC10323:	1.490751	2.611825	2.320634	1.125479	0.260386	0.357505	0	0.547412	0.695731	6.696381	0	1.004979
13575	LOC10323!	4.33441	2.61137	1.247603	2.093109	0.036339	0.071166	1.084173	0.547412	2.087194	6.696381	8.556443	7.034856
9035	LOC11962:	14.12446	2.609808	0.840793	3.103982	0.001909	0.005618	0.542086	3.831883	7.653045	24.5534	32.08666	16.07967
10890	LOC10322ł	7.801912	2.609297	0.983762	2.652366	0.007993	0.019511	2.710432	0.547412	3.478657	11.16063	12.83466	16.07967
1073	LOC10324(	138.3107	2.608988	0.24526	10.63764	1.99E-26	4.93E-25	41.74065	38.31883	36.17803	229.9091	271.6671	212.0507
45	SPP1	367641.3	2.608866	0.085298	30.58516	1.93E-205	1.14E-202	103929.3	97032.03	109711.3	640645	646009.3	608521.1
12168	LOC11962:	9.380478	2.607051	1.098999	2.372206	0.017682	0.038633	0.542086	0.547412	6.957314	13.39276	27.80844	7.034856
6669	TRIM29	16.31784	2.606875	0.673866	3.868537	0.000109	0.000436	3.252518	4.926706	5.565851	24.5534	38.50399	21.10457
6639	UBQLNL	25.72834	2.60678	0.671346	3.882917	0.000103	0.000413	5.96295	3.284471	12.52316	33.4819	77.00798	22.10955
8781	POU5F1	20.86953	2.605644	0.819597	3.179176	0.001477	0.004472	1.626259	1.642235	14.61036	40.17829	47.06043	20.09959
7273	NWD1	22.24501	2.605512	0.715371	3.642185	0.00027	0.000988	2.710432	4.926706	11.1317	53.57105	47.06043	14.06971
8018	LOC10323:	20.64114	2.605109	0.764665	3.406862	0.000657	0.002179	5.420864	2.189647	9.740239	55.80317	40.6431	10.04979
17577	LOC10323!	2.288737	2.605067	1.855301	1.404121	0.160283	0.242412	0	0.547412	1.391463	2.232127	8.556443	1.004979
17578	LOC11962ł	2.288737	2.605067	1.855301	1.404121	0.160283	0.242412	0	0.547412	1.391463	2.232127	8.556443	1.004979
13686	LOC11961!	5.389347	2.604006	1.256764	2.071994	0.038266	0.074332	0.542086	3.284471	0.695731	15.62489	2.139111	10.04979
12995	LOC11962:	6.500904	2.603807	1.181344	2.204106	0.027517	0.056294	1.626259	1.642235	2.087194	13.39276	19.252	1.004979
19282	APCDD1	1.422717	2.603469	2.285619	1.139065	0.254676	0.351134	0	0.547412	0.695731	0	4.278221	3.014938
4822	ZNF385D	51.20206	2.603332	0.542634	4.797579	1.61E-06	8.85E-06	8.131296	17.51718	17.39328	78.12444	151.8769	34.1693
9722	PTGIR	11.67033	2.603303	0.890208	2.924377	0.003451	0.009438	2.710432	2.189647	4.87012	17.85702	36.36488	6.029877
7939	AURKC	16.3211	2.600657	0.758788	3.427381	0.000609	0.002041	5.96295	5.474118	2.087194	51.33892	14.97377	18.08963
3665	KLHL30	34.35481	2.600175	0.457495	5.683509	1.32E-08	9.57E-08	9.757555	10.94824	8.348776	51.33892	68.45154	57.28383
15602	LOC10324:	5.638906	2.600058	1.503822	1.728967	0.083815	0.142816	0	0	4.87012	6.696381	19.252	3.014938
5781	GAP43	37.34009	2.599979	0.606894	4.284071	1.84E-05	8.44E-05	5.96295	4.379295	21.56767	73.66019	81.28621	37.18424
17900	LOC10323!	2.687051	2.599343	1.909666	1.361151	0.173466	0.257617	0	2.189647	0	2.232127	10.69555	1.004979
899	PSTPIP1	252.9777	2.599308	0.22644	11.479	1.68E-30	4.97E-29	80.77087	50.36189	84.1835	486.6037	397.8746	418.0715
9378	CABP4	12.49561	2.598764	0.862572	3.012806	0.002588	0.007338	3.252518	1.094824	6.261582	20.08914	34.22577	10.04979
16529	LOC11962:	2.663751	2.598513	1.654813	1.570276	0.116351	0.187137	1.084173	1.094824	0	2.232127	8.556443	3.014938
10768	CTNNA2	10.41468	2.598148	0.971044	2.675625	0.007459	0.018415	1.084173	2.189647	5.565851	13.39276	34.22577	6.029877
1446	MMP3	119.4473	2.597821	0.276966	9.379562	6.62E-21	1.22E-19	29.81475	29.56024	43.13534	171.8738	218.1893	224.1104
13929	LOC10322!	5.153922	2.597126	1.281987	2.02586	0.042779	0.081649	1.084173	0.547412	2.782925	2.232127	19.252	5.024897
106	PXYLP1	2171.774	2.597013	0.108033	24.03902	1.09E-127	2.73E-125	626.6518	656.8942	564.9339	3555.778	3638.627	3987.759
12781	LOC11961ł	5.666777	2.596469	1.157589	2.242997	0.024897	0.051787	1.084173	2.189647	1.391463	15.62489	10.69555	3.014938
8989	TDO2	9.713389	2.5963	0.831988	3.120598	0.001805	0.005338	2.168345	3.284471	2.782925	17.85702	17.11289	15.07469
17091	USP26	3.369447	2.595375	1.749457	1.483531	0.137933	0.214555	0.542086	2.189647	0	8.928508	8.556443	0
254	STING1	523.1641	2.595197	0.141776	18.30487	7.57E-75	7.92E-73	155.5788	144.5167	146.1036	910.7078	836.3923	945.6857
16290	LOC11962:	2.882462	2.594092	1.612572	1.608667	0.107689	0.175747	0	1.094824	1.391463	2.232127	8.556443	4.019918
18873	KLRC2	1.475248	2.593493	2.147034	1.207942	0.22707	0.319856	0	0.547412	0.695731	4.464254	2.139111	1.004979
8569	MYO7B	18.05516	2.592631	0.800925	3.237047	0.001208	0.003747	1.084173	2.737059	11.82743	26.78552	42.78221	23.11453
11577	LOC10323:	6.207084	2.592066	1.036843	2.49996	0.012421	0.028523	1.626259	2.189647	1.391463	11.16063	12.83466	8.039836
14792	RHO	4.108881	2.590434	1.381936	1.874497	0.060862	0.109385	0.542086	2.189647	0.695731	2.232127	14.97377	4.019918
16285	OPN4	3.980922	2.590429	1.608539	1.610423	0.107305	0.175174	0	0	3.478657	6.696381	10.69555	3.014938
11991	OVOL2	8.305851	2.590302	1.075578	2.408288	0.016028	0.035534	1.084173	0.547412	5.565851	8.928508	25.66933	8.039836
16240	LOC11962:	2.934993	2.590298	1.60031	1.618623	0.105528	0.172761	0	1.094824	1.391463	6.696381	6.417332	2.009959
17616	LOC10324!	3.219963	2.589942	1.848235	1.401305	0.161123	0.24309	0	0	2.782925	13.39276	2.139111	1.004979
16573	LOC11962:	2.866072	2.588862	1.65732	1.562077	0.11827	0.189718	0.542086	0.547412	1.391463	0	10.69555	4.019918
9796	LOC10324:	9.161827	2.588526	0.890964	2.905308	0.003669	0.009957	2.710432	2.189647	2.782925	17.85702	21.39111	8.039836
7349	MAB21L2	26.96972	2.585473	0.714966	3.616216	0.000299	0.001081	4.336691	1.642235	17.39328	62.49956	42.78221	33.16432
8219	EPHA8	15.9793	2.584522	0.773527	3.341217	0.000834	0.002698	7.047123	2.189647	4.174388	46.87467	23.53022	12.05975
11299	LOC11962(	8.175646	2.584508	1.00987	2.559248	0.01049	0.024681	1.084173	1.094824	4.87012	17.85702	17.11289	7.034856
13152	LOC11961ł	5.039219	2.582764	1.184606	2.180272	0.029237	0.059099	1.084173	1.094824	2.087194	13.39276	8.556443	4.019918
7277	GIMAP6	15.68038	2.582556	0.709383	3.640567	0.000272	0.000994	3.252518	3.831883	6.261582	35.71403	29.94755	15.07469
14984	CUNH8orf:	4.363733	2.582039	1.405507	1.837087	0.066197	0.117449	0	1.642235	2.087194	4.464254	14.97377	3.014938
9426	PDE6B	18.03788	2.581935	0.860247	3.001389	0.002688	0.00758	1.084173	1.642235	13.2189	17.85702	34.22577	40.19918
15783	LOC11962‹	3.347035	2.581679	1.514311	1.704854	0.088222	0.148601	1.084173	1.642235	0	8.928508	6.417332	2.009959
12280	LOC11962:	8.424475	2.581655	1.098926	2.349252	0.018811	0.040721	0.542086	0.547412	6.261582	22.32127	12.83466	8.039836
12655	LOC10324:	6.096628	2.579473	1.136544	2.269576	0.023233	0.048807	2.710432	1.642235	0.695731	20.08914	6.417332	5.024897
26586	LOC11962(	0.372021	2.579362	4.067274	0.634175	0.525967	NA	0	0	0	2.232127	0	0
26596	LOC11962(	0.356518	2.579362	4.067274	0.634175	0.525967	NA	0	0	0	0	2.139111	0
26597	LOC11962(	0.356518	2.579362	4.067274	0.634175	0.525967	NA	0	0	0	0	2.139111	0
26604	LOC11962(	0.356518	2.579362	4.067274	0.634175	0.525967	NA	0	0	0	0	2.139111	0
26607	LOC11962(	0.372021	2.579362	4.067274	0.634175	0.525967	NA	0	0	0	2.232127	0	0
26610	LOC11962(	0.356518	2.579362	4.067274	0.634175	0.525967	NA	0	0	0	0	2.139111	0
26617	LOC11962‹	0.372021	2.579362	4.067274	0.634175	0.525967	NA	0	0	0	2.232127	0	0

26627	LOC11962(	0.372021	2.579362	4.067274	0.634175	0.525967	NA	0	0	0	2.232127	0	0
26628	LOC11962!	0.356518	2.579362	4.067274	0.634175	0.525967	NA	0	0	0	0	2.139111	0
26633	UBE2U	0.356518	2.579362	4.067274	0.634175	0.525967	NA	0	0	0	0	2.139111	0
26667	LOC10324‹	0.356518	2.579362	4.067274	0.634175	0.525967	NA	0	0	0	0	2.139111	0
26671	LOC11961{	0.356518	2.579362	4.067274	0.634175	0.525967	NA	0	0	0	0	2.139111	0
26675	LOC11961{	0.356518	2.579362	4.067274	0.634175	0.525967	NA	0	0	0	0	2.139111	0
26689	LOC11961{	0.372021	2.579362	4.067274	0.634175	0.525967	NA	0	0	0	2.232127	0	0
26699	IL13	0.372021	2.579362	4.067274	0.634175	0.525967	NA	0	0	0	2.232127	0	0
26702	MINAR2	0.356518	2.579362	4.067274	0.634175	0.525967	NA	0	0	0	0	2.139111	0
26704	LOC11961{	0.356518	2.579362	4.067274	0.634175	0.525967	NA	0	0	0	0	2.139111	0
26708	FTMT	0.356518	2.579362	4.067274	0.634175	0.525967	NA	0	0	0	0	2.139111	0
26709	LOC11962{	0.356518	2.579362	4.067274	0.634175	0.525967	NA	0	0	0	0	2.139111	0
26713	LOC11961{	0.356518	2.579362	4.067274	0.634175	0.525967	NA	0	0	0	0	2.139111	0
26717	LOC11962{	0.372021	2.579362	4.067274	0.634175	0.525967	NA	0	0	0	2.232127	0	0
26718	LOC11961{	0.372021	2.579362	4.067274	0.634175	0.525967	NA	0	0	0	2.232127	0	0
26721	LOC10321{	0.372021	2.579362	4.067274	0.634175	0.525967	NA	0	0	0	2.232127	0	0
26722	LOC10321{	0.372021	2.579362	4.067274	0.634175	0.525967	NA	0	0	0	2.232127	0	0
26726	LOC11961{	0.372021	2.579362	4.067274	0.634175	0.525967	NA	0	0	0	2.232127	0	0
26739	LOC11961{	0.372021	2.579362	4.067274	0.634175	0.525967	NA	0	0	0	2.232127	0	0
26741	LOC11961{	0.356518	2.579362	4.067274	0.634175	0.525967	NA	0	0	0	0	2.139111	0
26776	LOC11962:	0.356518	2.579362	4.067274	0.634175	0.525967	NA	0	0	0	0	2.139111	0
26778	LOC11962:	0.372021	2.579362	4.067274	0.634175	0.525967	NA	0	0	0	2.232127	0	0
26783	LOC11962‹	0.356518	2.579362	4.067274	0.634175	0.525967	NA	0	0	0	0	2.139111	0
26792	LOC11962‹	0.356518	2.579362	4.067274	0.634175	0.525967	NA	0	0	0	0	2.139111	0
26801	LOC11962:	0.372021	2.579362	4.067274	0.634175	0.525967	NA	0	0	0	2.232127	0	0
26805	LOC11962‹	0.356518	2.579362	4.067274	0.634175	0.525967	NA	0	0	0	0	2.139111	0
26814	LOC11962‹	0.356518	2.579362	4.067274	0.634175	0.525967	NA	0	0	0	0	2.139111	0
26825	LOC11962:	0.356518	2.579362	4.067274	0.634175	0.525967	NA	0	0	0	0	2.139111	0
26828	LOC11962‹	0.356518	2.579362	4.067274	0.634175	0.525967	NA	0	0	0	0	2.139111	0
26840	LOC11962:	0.372021	2.579362	4.067274	0.634175	0.525967	NA	0	0	0	2.232127	0	0
26851	LOC10323{	0.356518	2.579362	4.067274	0.634175	0.525967	NA	0	0	0	0	2.139111	0
26854	LOC11962‹	0.356518	2.579362	4.067274	0.634175	0.525967	NA	0	0	0	0	2.139111	0
26855	LOC11962‹	0.372021	2.579362	4.067274	0.634175	0.525967	NA	0	0	0	2.232127	0	0
26859	LOC11962:	0.356518	2.579362	4.067274	0.634175	0.525967	NA	0	0	0	0	2.139111	0
26862	LOC11962‹	0.372021	2.579362	4.067274	0.634175	0.525967	NA	0	0	0	2.232127	0	0
26863	LOC11962‹	0.372021	2.579362	4.067274	0.634175	0.525967	NA	0	0	0	2.232127	0	0
26869	LOC11962(	0.372021	2.579362	4.067274	0.634175	0.525967	NA	0	0	0	2.232127	0	0
26878	LOC11962‹	0.372021	2.579362	4.067274	0.634175	0.525967	NA	0	0	0	2.232127	0	0
26883	LOC10323{	0.356518	2.579362	4.067274	0.634175	0.525967	NA	0	0	0	0	2.139111	0
26884	LOC10323{	0.372021	2.579362	4.067274	0.634175	0.525967	NA	0	0	0	2.232127	0	0
26889	LOC10323{	0.372021	2.579362	4.067274	0.634175	0.525967	NA	0	0	0	2.232127	0	0
26898	LOC10323{	0.356518	2.579362	4.067274	0.634175	0.525967	NA	0	0	0	0	2.139111	0
26902	LOC11962‹	0.356518	2.579362	4.067274	0.634175	0.525967	NA	0	0	0	0	2.139111	0
26908	LOC10323:	0.372021	2.579362	4.067274	0.634175	0.525967	NA	0	0	0	2.232127	0	0
26913	LOC10322:	0.372021	2.579362	4.067274	0.634175	0.525967	NA	0	0	0	2.232127	0	0
26915	LOC10322:	0.356518	2.579362	4.067274	0.634175	0.525967	NA	0	0	0	0	2.139111	0
26917	LOC10322:	0.356518	2.579362	4.067274	0.634175	0.525967	NA	0	0	0	0	2.139111	0
26918	LOC10322:	0.356518	2.579362	4.067274	0.634175	0.525967	NA	0	0	0	0	2.139111	0
26919	LOC10322:	0.372021	2.579362	4.067274	0.634175	0.525967	NA	0	0	0	2.232127	0	0
26922	LOC10322:	0.356518	2.579362	4.067274	0.634175	0.525967	NA	0	0	0	0	2.139111	0
26932	LOC11962‹	0.356518	2.579362	4.067274	0.634175	0.525967	NA	0	0	0	0	2.139111	0
26934	LOC11962‹	0.356518	2.579362	4.067274	0.634175	0.525967	NA	0	0	0	0	2.139111	0
26935	LOC10322:	0.356518	2.579362	4.067274	0.634175	0.525967	NA	0	0	0	0	2.139111	0
26942	CUNH1orf(	0.372021	2.579362	4.067274	0.634175	0.525967	NA	0	0	0	2.232127	0	0
26945	LOC10322‹	0.356518	2.579362	4.067274	0.634175	0.525967	NA	0	0	0	0	2.139111	0
26948	LOC11962‹	0.372021	2.579362	4.067274	0.634175	0.525967	NA	0	0	0	2.232127	0	0
26963	LOC11962‹	0.372021	2.579362	4.067274	0.634175	0.525967	NA	0	0	0	2.232127	0	0
26968	LOC11962‹	0.356518	2.579362	4.067274	0.634175	0.525967	NA	0	0	0	0	2.139111	0
26970	LOC10322!	0.356518	2.579362	4.067274	0.634175	0.525967	NA	0	0	0	0	2.139111	0
26975	LOC11962‹	0.356518	2.579362	4.067274	0.634175	0.525967	NA	0	0	0	0	2.139111	0
26977	LOC11962‹	0.356518	2.579362	4.067274	0.634175	0.525967	NA	0	0	0	0	2.139111	0
26982	LOC10321{	0.372021	2.579362	4.067274	0.634175	0.525967	NA	0	0	0	2.232127	0	0
26984	LOC10321{	0.356518	2.579362	4.067274	0.634175	0.525967	NA	0	0	0	0	2.139111	0
26995	LOC11962‹	0.356518	2.579362	4.067274	0.634175	0.525967	NA	0	0	0	0	2.139111	0
27005	LOC11962‹	0.372021	2.579362	4.067274	0.634175	0.525967	NA	0	0	0	2.232127	0	0
27008	LOC11962‹	0.372021	2.579362	4.067274	0.634175	0.525967	NA	0	0	0	2.232127	0	0
27010	TRNAK-CU	0.356518	2.579362	4.067274	0.634175	0.525967	NA	0	0	0	0	2.139111	0
27022	ODF3	0.356518	2.579362	4.067274	0.634175	0.525967	NA	0	0	0	0	2.139111	0
27027	LOC11962‹	0.356518	2.579362	4.067274	0.634175	0.525967	NA	0	0	0	0	2.139111	0
27029	LOC11962‹	0.356518	2.579362	4.067274	0.634175	0.525967	NA	0	0	0	0	2.139111	0
27052	LOC10323!	0.372021	2.579362	4.067274	0.634175	0.525967	NA	0	0	0	2.232127	0	0
27059	LOC10323!	0.356518	2.579362	4.067274	0.634175	0.525967	NA	0	0	0	0	2.139111	0
27064	LOC10323!	0.356518	2.579362	4.067274	0.634175	0.525967	NA	0	0	0	0	2.139111	0
27066	LOC10323!	0.356518	2.579362	4.067274	0.634175	0.525967	NA	0	0	0	0	2.139111	0

27068	LOC103235	0.356518	2.579362	4.067274	0.634175	0.525967	NA	0	0	0	0	2.139111	0
27073	LOC119624	0.356518	2.579362	4.067274	0.634175	0.525967	NA	0	0	0	0	2.139111	0
27076	LOC103235	0.372021	2.579362	4.067274	0.634175	0.525967	NA	0	0	0	2.232127	0	0
27079	LOC103235	0.356518	2.579362	4.067274	0.634175	0.525967	NA	0	0	0	0	2.139111	0
27084	LOC103235	0.372021	2.579362	4.067274	0.634175	0.525967	NA	0	0	0	2.232127	0	0
27096	LOC119624	0.372021	2.579362	4.067274	0.634175	0.525967	NA	0	0	0	2.232127	0	0
27104	ASCL3	0.372021	2.579362	4.067274	0.634175	0.525967	NA	0	0	0	2.232127	0	0
27110	LOC103241	0.356518	2.579362	4.067274	0.634175	0.525967	NA	0	0	0	0	2.139111	0
27116	LOC103241	0.356518	2.579362	4.067274	0.634175	0.525967	NA	0	0	0	0	2.139111	0
27117	LOC119624	0.372021	2.579362	4.067274	0.634175	0.525967	NA	0	0	0	2.232127	0	0
27130	LOC119624	0.372021	2.579362	4.067274	0.634175	0.525967	NA	0	0	0	2.232127	0	0
27133	LOC119624	0.372021	2.579362	4.067274	0.634175	0.525967	NA	0	0	0	2.232127	0	0
27160	LOC119624	0.372021	2.579362	4.067274	0.634175	0.525967	NA	0	0	0	2.232127	0	0
27161	LOC119624	0.372021	2.579362	4.067274	0.634175	0.525967	NA	0	0	0	2.232127	0	0
27170	LOC119624	0.372021	2.579362	4.067274	0.634175	0.525967	NA	0	0	0	2.232127	0	0
27174	LOC119624	0.356518	2.579362	4.067274	0.634175	0.525967	NA	0	0	0	0	2.139111	0
27176	LOC103237	0.356518	2.579362	4.067274	0.634175	0.525967	NA	0	0	0	0	2.139111	0
27182	LOC119625	0.372021	2.579362	4.067274	0.634175	0.525967	NA	0	0	0	2.232127	0	0
27190	TAAR3	0.372021	2.579362	4.067274	0.634175	0.525967	NA	0	0	0	2.232127	0	0
27197	LOC119625	0.372021	2.579362	4.067274	0.634175	0.525967	NA	0	0	0	2.232127	0	0
27202	LOC119625	0.356518	2.579362	4.067274	0.634175	0.525967	NA	0	0	0	0	2.139111	0
27209	LOC119625	0.356518	2.579362	4.067274	0.634175	0.525967	NA	0	0	0	0	2.139111	0
27221	LOC119625	0.356518	2.579362	4.067274	0.634175	0.525967	NA	0	0	0	0	2.139111	0
27222	LOC119625	0.356518	2.579362	4.067274	0.634175	0.525967	NA	0	0	0	0	2.139111	0
27232	LOC103240	0.372021	2.579362	4.067274	0.634175	0.525967	NA	0	0	0	2.232127	0	0
27250	TEX44	0.356518	2.579362	4.067274	0.634175	0.525967	NA	0	0	0	0	2.139111	0
27260	LOC119625	0.356518	2.579362	4.067274	0.634175	0.525967	NA	0	0	0	0	2.139111	0
27262	LOC119625	0.356518	2.579362	4.067274	0.634175	0.525967	NA	0	0	0	0	2.139111	0
27299	LOC119625	0.372021	2.579362	4.067274	0.634175	0.525967	NA	0	0	0	2.232127	0	0
27310	LOC119625	0.372021	2.579362	4.067274	0.634175	0.525967	NA	0	0	0	2.232127	0	0
27326	LOC119625	0.356518	2.579362	4.067274	0.634175	0.525967	NA	0	0	0	0	2.139111	0
27330	MEI4	0.356518	2.579362	4.067274	0.634175	0.525967	NA	0	0	0	0	2.139111	0
27334	LOC119625	0.372021	2.579362	4.067274	0.634175	0.525967	NA	0	0	0	2.232127	0	0
27337	CGA	0.372021	2.579362	4.067274	0.634175	0.525967	NA	0	0	0	2.232127	0	0
27343	LOC119625	0.356518	2.579362	4.067274	0.634175	0.525967	NA	0	0	0	0	2.139111	0
27350	LOC119626	0.356518	2.579362	4.067274	0.634175	0.525967	NA	0	0	0	0	2.139111	0
27355	LOC119625	0.372021	2.579362	4.067274	0.634175	0.525967	NA	0	0	0	2.232127	0	0
27362	TRNAC-GC	0.372021	2.579362	4.067274	0.634175	0.525967	NA	0	0	0	2.232127	0	0
27365	LOC119625	0.356518	2.579362	4.067274	0.634175	0.525967	NA	0	0	0	0	2.139111	0
27366	LOC119626	0.356518	2.579362	4.067274	0.634175	0.525967	NA	0	0	0	0	2.139111	0
27371	LOC119626	0.356518	2.579362	4.067274	0.634175	0.525967	NA	0	0	0	0	2.139111	0
27382	LOC119625	0.372021	2.579362	4.067274	0.634175	0.525967	NA	0	0	0	2.232127	0	0
27385	LOC119626	0.372021	2.579362	4.067274	0.634175	0.525967	NA	0	0	0	2.232127	0	0
27387	LOC119626	0.356518	2.579362	4.067274	0.634175	0.525967	NA	0	0	0	0	2.139111	0
27397	LOC119626	0.356518	2.579362	4.067274	0.634175	0.525967	NA	0	0	0	0	2.139111	0
27405	LOC119626	0.356518	2.579362	4.067274	0.634175	0.525967	NA	0	0	0	0	2.139111	0
27413	LOC119626	0.356518	2.579362	4.067274	0.634175	0.525967	NA	0	0	0	0	2.139111	0
27425	LOC119626	0.356518	2.579362	4.067274	0.634175	0.525967	NA	0	0	0	0	2.139111	0
27432	LOC119625	0.356518	2.579362	4.067274	0.634175	0.525967	NA	0	0	0	0	2.139111	0
27436	LOC119625	0.356518	2.579362	4.067274	0.634175	0.525967	NA	0	0	0	0	2.139111	0
27448	LOC119625	0.356518	2.579362	4.067274	0.634175	0.525967	NA	0	0	0	0	2.139111	0
27453	LOC119626	0.356518	2.579362	4.067274	0.634175	0.525967	NA	0	0	0	0	2.139111	0
27467	SULT1C3	0.356518	2.579362	4.067274	0.634175	0.525967	NA	0	0	0	0	2.139111	0
27468	LOC119626	0.372021	2.579362	4.067274	0.634175	0.525967	NA	0	0	0	2.232127	0	0
27473	LOC119626	0.356518	2.579362	4.067274	0.634175	0.525967	NA	0	0	0	0	2.139111	0
27483	LOC103226	0.356518	2.579362	4.067274	0.634175	0.525967	NA	0	0	0	0	2.139111	0
27490	EFCAB10	0.372021	2.579362	4.067274	0.634175	0.525967	NA	0	0	0	2.232127	0	0
27491	LOC119626	0.372021	2.579362	4.067274	0.634175	0.525967	NA	0	0	0	2.232127	0	0
27492	LOC119626	0.356518	2.579362	4.067274	0.634175	0.525967	NA	0	0	0	0	2.139111	0
27505	LOC119626	0.356518	2.579362	4.067274	0.634175	0.525967	NA	0	0	0	0	2.139111	0
27509	LOC119626	0.372021	2.579362	4.067274	0.634175	0.525967	NA	0	0	0	2.232127	0	0
27517	NPY	0.356518	2.579362	4.067274	0.634175	0.525967	NA	0	0	0	0	2.139111	0
27519	LOC119626	0.372021	2.579362	4.067274	0.634175	0.525967	NA	0	0	0	2.232127	0	0
27520	LINC02860	0.356518	2.579362	4.067274	0.634175	0.525967	NA	0	0	0	0	2.139111	0
27537	LOC103248	0.356518	2.579362	4.067274	0.634175	0.525967	NA	0	0	0	0	2.139111	0
27539	LOC103248	0.356518	2.579362	4.067274	0.634175	0.525967	NA	0	0	0	0	2.139111	0
27542	LOC103248	0.372021	2.579362	4.067274	0.634175	0.525967	NA	0	0	0	2.232127	0	0
27572	LOC103247	0.356518	2.579362	4.067274	0.634175	0.525967	NA	0	0	0	0	2.139111	0
27575	LOC103247	0.356518	2.579362	4.067274	0.634175	0.525967	NA	0	0	0	0	2.139111	0
27576	LOC103247	0.372021	2.579362	4.067274	0.634175	0.525967	NA	0	0	0	2.232127	0	0
27578	LOC103247	0.372021	2.579362	4.067274	0.634175	0.525967	NA	0	0	0	2.232127	0	0
27586	LOC103247	0.356518	2.579362	4.067274	0.634175	0.525967	NA	0	0	0	0	2.139111	0
27587	LOC103247	0.372021	2.579362	4.067274	0.634175	0.525967	NA	0	0	0	2.232127	0	0
27588	LOC103247	0.372021	2.579362	4.067274	0.634175	0.525967	NA	0	0	0	2.232127	0	0

27595	LOC10324	0.356518	2.579362	4.067274	0.634175	0.525967	NA	0	0	0	0	2.139111	0	
27604	LOC10324	0.372021	2.579362	4.067274	0.634175	0.525967	NA	0	0	0	2.232127	0	0	
27611	LOC11962	0.356518	2.579362	4.067274	0.634175	0.525967	NA	0	0	0	0	2.139111	0	
27621	LOC11962	0.372021	2.579362	4.067274	0.634175	0.525967	NA	0	0	0	2.232127	0	0	
27623	LOC11962	0.356518	2.579362	4.067274	0.634175	0.525967	NA	0	0	0	0	2.139111	0	
27627	LOC11962	0.372021	2.579362	4.067274	0.634175	0.525967	NA	0	0	0	2.232127	0	0	
27657	LOC11962	0.372021	2.579362	4.067274	0.634175	0.525967	NA	0	0	0	2.232127	0	0	
27665	LOC10322	0.356518	2.579362	4.067274	0.634175	0.525967	NA	0	0	0	0	2.139111	0	
27678	LOC11962	0.372021	2.579362	4.067274	0.634175	0.525967	NA	0	0	0	2.232127	0	0	
27685	LOC11962	0.356518	2.579362	4.067274	0.634175	0.525967	NA	0	0	0	0	2.139111	0	
27686	LOC10322	0.356518	2.579362	4.067274	0.634175	0.525967	NA	0	0	0	0	2.139111	0	
27687	LOC11962	0.372021	2.579362	4.067274	0.634175	0.525967	NA	0	0	0	2.232127	0	0	
27692	LOC11962	0.356518	2.579362	4.067274	0.634175	0.525967	NA	0	0	0	0	2.139111	0	
27719	LOC10322	0.356518	2.579362	4.067274	0.634175	0.525967	NA	0	0	0	0	2.139111	0	
27729	LOC11962	0.356518	2.579362	4.067274	0.634175	0.525967	NA	0	0	0	0	2.139111	0	
27730	LOC10322	0.372021	2.579362	4.067274	0.634175	0.525967	NA	0	0	0	2.232127	0	0	
27736	LOC11962	0.356518	2.579362	4.067274	0.634175	0.525967	NA	0	0	0	0	2.139111	0	
27773	LOC11962	0.372021	2.579362	4.067274	0.634175	0.525967	NA	0	0	0	2.232127	0	0	
27777	LOC11962	0.372021	2.579362	4.067274	0.634175	0.525967	NA	0	0	0	2.232127	0	0	
27778	LOC11962	0.372021	2.579362	4.067274	0.634175	0.525967	NA	0	0	0	2.232127	0	0	
27785	LOC11962	0.356518	2.579362	4.067274	0.634175	0.525967	NA	0	0	0	0	2.139111	0	
27827	LOC10321	0.356518	2.579362	4.067274	0.634175	0.525967	NA	0	0	0	0	2.139111	0	
27833	LOC11962	0.356518	2.579362	4.067274	0.634175	0.525967	NA	0	0	0	0	2.139111	0	
27835	LOC11962	0.372021	2.579362	4.067274	0.634175	0.525967	NA	0	0	0	2.232127	0	0	
27843	TRNAG-GC	0.356518	2.579362	4.067274	0.634175	0.525967	NA	0	0	0	0	2.139111	0	
27851	LOC11962	0.356518	2.579362	4.067274	0.634175	0.525967	NA	0	0	0	0	2.139111	0	
27855	LOC11962	0.372021	2.579362	4.067274	0.634175	0.525967	NA	0	0	0	2.232127	0	0	
27858	LOC11962	0.356518	2.579362	4.067274	0.634175	0.525967	NA	0	0	0	0	2.139111	0	
27859	CCL17	0.356518	2.579362	4.067274	0.634175	0.525967	NA	0	0	0	0	2.139111	0	
27861	LOC10323	0.372021	2.579362	4.067274	0.634175	0.525967	NA	0	0	0	2.232127	0	0	
27863	LOC10323	0.356518	2.579362	4.067274	0.634175	0.525967	NA	0	0	0	0	2.139111	0	
27898	LOC11962	0.356518	2.579362	4.067274	0.634175	0.525967	NA	0	0	0	0	2.139111	0	
27910	LOC11962	0.372021	2.579362	4.067274	0.634175	0.525967	NA	0	0	0	2.232127	0	0	
27912	LOC11962	0.356518	2.579362	4.067274	0.634175	0.525967	NA	0	0	0	0	2.139111	0	
27940	LOC11962	0.372021	2.579362	4.067274	0.634175	0.525967	NA	0	0	0	2.232127	0	0	
27941	LOC11962	0.372021	2.579362	4.067274	0.634175	0.525967	NA	0	0	0	2.232127	0	0	
27943	LOC11962	0.372021	2.579362	4.067274	0.634175	0.525967	NA	0	0	0	2.232127	0	0	
27950	LOC11962	0.372021	2.579362	4.067274	0.634175	0.525967	NA	0	0	0	2.232127	0	0	
27955	NPS	0.356518	2.579362	4.067274	0.634175	0.525967	NA	0	0	0	0	2.139111	0	
27966	LOC10324	0.356518	2.579362	4.067274	0.634175	0.525967	NA	0	0	0	0	2.139111	0	
27967	LOC11962	0.372021	2.579362	4.067274	0.634175	0.525967	NA	0	0	0	2.232127	0	0	
27979	LOC11962	0.356518	2.579362	4.067274	0.634175	0.525967	NA	0	0	0	0	2.139111	0	
27980	LOC11962	0.356518	2.579362	4.067274	0.634175	0.525967	NA	0	0	0	0	2.139111	0	
27983	LOC11962	0.372021	2.579362	4.067274	0.634175	0.525967	NA	0	0	0	2.232127	0	0	
28003	LOC11961	0.356518	2.579362	4.067274	0.634175	0.525967	NA	0	0	0	0	2.139111	0	
28006	LOC11961	0.356518	2.579362	4.067274	0.634175	0.525967	NA	0	0	0	0	2.139111	0	
28009	LOC11961	0.356518	2.579362	4.067274	0.634175	0.525967	NA	0	0	0	0	2.139111	0	
28016	LOC11961	0.372021	2.579362	4.067274	0.634175	0.525967	NA	0	0	0	2.232127	0	0	
28031	LOC11961	0.356518	2.579362	4.067274	0.634175	0.525967	NA	0	0	0	0	2.139111	0	
28048	LOC11961	0.356518	2.579362	4.067274	0.634175	0.525967	NA	0	0	0	0	2.139111	0	
28055	LOC11961	0.372021	2.579362	4.067274	0.634175	0.525967	NA	0	0	0	2.232127	0	0	
28058	LOC11961	0.372021	2.579362	4.067274	0.634175	0.525967	NA	0	0	0	2.232127	0	0	
28059	LOC10324	0.372021	2.579362	4.067274	0.634175	0.525967	NA	0	0	0	2.232127	0	0	
28100	LOC11961	0.372021	2.579362	4.067274	0.634175	0.525967	NA	0	0	0	2.232127	0	0	
28104	DEFB121	0.372021	2.579362	4.067274	0.634175	0.525967	NA	0	0	0	2.232127	0	0	
28105	LOC11961	0.356518	2.579362	4.067274	0.634175	0.525967	NA	0	0	0	0	2.139111	0	
28108	LOC11961	0.372021	2.579362	4.067274	0.634175	0.525967	NA	0	0	0	2.232127	0	0	
28122	LOC11961	0.356518	2.579362	4.067274	0.634175	0.525967	NA	0	0	0	0	2.139111	0	
28124	LOC11961	0.356518	2.579362	4.067274	0.634175	0.525967	NA	0	0	0	0	2.139111	0	
28128	LOC11961	0.372021	2.579362	4.067274	0.634175	0.525967	NA	0	0	0	2.232127	0	0	
28129	LOC10323	0.372021	2.579362	4.067274	0.634175	0.525967	NA	0	0	0	2.232127	0	0	
28130	LOC11961	0.372021	2.579362	4.067274	0.634175	0.525967	NA	0	0	0	2.232127	0	0	
28132	LOC11961	0.372021	2.579362	4.067274	0.634175	0.525967	NA	0	0	0	2.232127	0	0	
28139	LOC11961	0.372021	2.579362	4.067274	0.634175	0.525967	NA	0	0	0	2.232127	0	0	
28146	LOC11961	0.356518	2.579362	4.067274	0.634175	0.525967	NA	0	0	0	0	2.139111	0	
28147	LOC11961	0.356518	2.579362	4.067274	0.634175	0.525967	NA	0	0	0	0	2.139111	0	
28165	LOC11961	0.356518	2.579362	4.067274	0.634175	0.525967	NA	0	0	0	0	2.139111	0	
28166	LOC11961	0.356518	2.579362	4.067274	0.634175	0.525967	NA	0	0	0	0	2.139111	0	
28168	LOC10321	0.356518	2.579362	4.067274	0.634175	0.525967	NA	0	0	0	0	2.139111	0	
28169	LOC11961	0.356518	2.579362	4.067274	0.634175	0.525967	NA	0	0	0	0	2.139111	0	
28177	DEFB135	0.372021	2.579362	4.067274	0.634175	0.525967	NA	0	0	0	2.232127	0	0	
28178	DEFB134	0.372021	2.579362	4.067274	0.634175	0.525967	NA	0	0	0	2.232127	0	0	
28180	LOC11961	0.356518	2.579362	4.067274	0.634175	0.525967	NA	0	0	0	0	2.139111	0	
28184	LOC11961	0.372021	2.579362	4.067274	0.634175	0.525967	NA	0	0	0	2.232127	0	0	

28185	LOC10321!	0.372021	2.579362	4.067274	0.634175	0.525967	NA	0	0	0	2.232127	0	0
28187	LOC11961!	0.372021	2.579362	4.067274	0.634175	0.525967	NA	0	0	0	2.232127	0	0
28190	LOC11961{	0.356518	2.579362	4.067274	0.634175	0.525967	NA	0	0	0	0	2.139111	0
28194	LOC11961!	0.372021	2.579362	4.067274	0.634175	0.525967	NA	0	0	0	2.232127	0	0
28196	LOC11961!	0.356518	2.579362	4.067274	0.634175	0.525967	NA	0	0	0	0	2.139111	0
28221	LOC11961!	0.356518	2.579362	4.067274	0.634175	0.525967	NA	0	0	0	0	2.139111	0
28245	LOC11961!	0.372021	2.579362	4.067274	0.634175	0.525967	NA	0	0	0	2.232127	0	0
28260	TFF2	0.372021	2.579362	4.067274	0.634175	0.525967	NA	0	0	0	2.232127	0	0
28263	LOC11961!	0.356518	2.579362	4.067274	0.634175	0.525967	NA	0	0	0	0	2.139111	0
28273	LOC11961!	0.372021	2.579362	4.067274	0.634175	0.525967	NA	0	0	0	2.232127	0	0
28280	LOC10322!	0.372021	2.579362	4.067274	0.634175	0.525967	NA	0	0	0	2.232127	0	0
28291	LOC11961!	0.356518	2.579362	4.067274	0.634175	0.525967	NA	0	0	0	0	2.139111	0
28345	LOC11961!	0.372021	2.579362	4.067274	0.634175	0.525967	NA	0	0	0	2.232127	0	0
28351	LOC11961!	0.356518	2.579362	4.067274	0.634175	0.525967	NA	0	0	0	0	2.139111	0
28356	DEFA4	0.356518	2.579362	4.067274	0.634175	0.525967	NA	0	0	0	0	2.139111	0
28357	LOC10321!	0.356518	2.579362	4.067274	0.634175	0.525967	NA	0	0	0	0	2.139111	0
28364	LOC11961!	0.372021	2.579362	4.067274	0.634175	0.525967	NA	0	0	0	2.232127	0	0
28367	TRNAT-UG	0.356518	2.579362	4.067274	0.634175	0.525967	NA	0	0	0	0	2.139111	0
28369	LOC11961!	0.356518	2.579362	4.067274	0.634175	0.525967	NA	0	0	0	0	2.139111	0
28371	LOC11961!	0.356518	2.579362	4.067274	0.634175	0.525967	NA	0	0	0	0	2.139111	0
28375	LOC11961!	0.356518	2.579362	4.067274	0.634175	0.525967	NA	0	0	0	0	2.139111	0
28380	LOC11961!	0.372021	2.579362	4.067274	0.634175	0.525967	NA	0	0	0	2.232127	0	0
28386	LOC11961!	0.356518	2.579362	4.067274	0.634175	0.525967	NA	0	0	0	0	2.139111	0
28387	LOC11961!	0.356518	2.579362	4.067274	0.634175	0.525967	NA	0	0	0	0	2.139111	0
28389	ATP6V1G3	0.356518	2.579362	4.067274	0.634175	0.525967	NA	0	0	0	0	2.139111	0
28393	RGS21	0.372021	2.579362	4.067274	0.634175	0.525967	NA	0	0	0	2.232127	0	0
28395	LOC11961!	0.356518	2.579362	4.067274	0.634175	0.525967	NA	0	0	0	0	2.139111	0
28412	LOC11961!	0.356518	2.579362	4.067274	0.634175	0.525967	NA	0	0	0	0	2.139111	0
28413	LOC11961!	0.372021	2.579362	4.067274	0.634175	0.525967	NA	0	0	0	2.232127	0	0
28416	LOC11961!	0.372021	2.579362	4.067274	0.634175	0.525967	NA	0	0	0	2.232127	0	0
28431	LOC10323(	0.372021	2.579362	4.067274	0.634175	0.525967	NA	0	0	0	2.232127	0	0
28432	LOC10323(	0.356518	2.579362	4.067274	0.634175	0.525967	NA	0	0	0	0	2.139111	0
28435	LOC10323(	0.372021	2.579362	4.067274	0.634175	0.525967	NA	0	0	0	2.232127	0	0
28442	LOC11962(	0.372021	2.579362	4.067274	0.634175	0.525967	NA	0	0	0	2.232127	0	0
28458	LOC11961!	0.356518	2.579362	4.067274	0.634175	0.525967	NA	0	0	0	0	2.139111	0
28465	LOC11962(	0.356518	2.579362	4.067274	0.634175	0.525967	NA	0	0	0	0	2.139111	0
28469	LOC11962(	0.372021	2.579362	4.067274	0.634175	0.525967	NA	0	0	0	2.232127	0	0
28470	LOC11962(	0.356518	2.579362	4.067274	0.634175	0.525967	NA	0	0	0	0	2.139111	0
28479	SERPINE3	0.356518	2.579362	4.067274	0.634175	0.525967	NA	0	0	0	0	2.139111	0
28507	LOC10321⁄	0.372021	2.579362	4.067274	0.634175	0.525967	NA	0	0	0	2.232127	0	0
28511	TRNAE-UU	0.356518	2.579362	4.067274	0.634175	0.525967	NA	0	0	0	0	2.139111	0
28512	LOC11962(	0.356518	2.579362	4.067274	0.634175	0.525967	NA	0	0	0	0	2.139111	0
28515	LOC11962(	0.372021	2.579362	4.067274	0.634175	0.525967	NA	0	0	0	2.232127	0	0
28527	LOC10324;	0.356518	2.579362	4.067274	0.634175	0.525967	NA	0	0	0	0	2.139111	0
28531	LOC11962(	0.372021	2.579362	4.067274	0.634175	0.525967	NA	0	0	0	2.232127	0	0
28536	LOC11962(	0.372021	2.579362	4.067274	0.634175	0.525967	NA	0	0	0	2.232127	0	0
28547	LOC10324:	0.356518	2.579362	4.067274	0.634175	0.525967	NA	0	0	0	0	2.139111	0
28554	LOC1032З:	0.356518	2.579362	4.067274	0.634175	0.525967	NA	0	0	0	0	2.139111	0
28557	LOC10323:	0.356518	2.579362	4.067274	0.634175	0.525967	NA	0	0	0	0	2.139111	0
28565	RNASE8	0.356518	2.579362	4.067274	0.634175	0.525967	NA	0	0	0	0	2.139111	0
28566	RNASE6	0.356518	2.579362	4.067274	0.634175	0.525967	NA	0	0	0	0	2.139111	0
28570	LOC10323:	0.372021	2.579362	4.067274	0.634175	0.525967	NA	0	0	0	2.232127	0	0
28571	LOC10323:	0.356518	2.579362	4.067274	0.634175	0.525967	NA	0	0	0	0	2.139111	0
28572	LOC10323:	0.372021	2.579362	4.067274	0.634175	0.525967	NA	0	0	0	2.232127	0	0
28588	LOC11962(	0.356518	2.579362	4.067274	0.634175	0.525967	NA	0	0	0	0	2.139111	0
28590	LOC11962(	0.372021	2.579362	4.067274	0.634175	0.525967	NA	0	0	0	2.232127	0	0
28592	LOC11962(	0.372021	2.579362	4.067274	0.634175	0.525967	NA	0	0	0	2.232127	0	0
28597	LOC11962(	0.372021	2.579362	4.067274	0.634175	0.525967	NA	0	0	0	2.232127	0	0
28601	LOC11962(	0.372021	2.579362	4.067274	0.634175	0.525967	NA	0	0	0	2.232127	0	0
28602	LOC10324⁄	0.372021	2.579362	4.067274	0.634175	0.525967	NA	0	0	0	2.232127	0	0
28625	LOC11962(	0.372021	2.579362	4.067274	0.634175	0.525967	NA	0	0	0	2.232127	0	0
28626	LOC11962(	0.356518	2.579362	4.067274	0.634175	0.525967	NA	0	0	0	0	2.139111	0
28632	LOC11962(	0.356518	2.579362	4.067274	0.634175	0.525967	NA	0	0	0	0	2.139111	0
28645	LOC10321{	0.356518	2.579362	4.067274	0.634175	0.525967	NA	0	0	0	0	2.139111	0
28646	LOC11962(	0.372021	2.579362	4.067274	0.634175	0.525967	NA	0	0	0	2.232127	0	0
28647	CCR8	0.372021	2.579362	4.067274	0.634175	0.525967	NA	0	0	0	2.232127	0	0
28669	LOC11962(	0.372021	2.579362	4.067274	0.634175	0.525967	NA	0	0	0	2.232127	0	0
28675	LOC11962(	0.356518	2.579362	4.067274	0.634175	0.525967	NA	0	0	0	0	2.139111	0
28682	LOC11962:	0.356518	2.579362	4.067274	0.634175	0.525967	NA	0	0	0	0	2.139111	0
28683	LOC11962(	0.372021	2.579362	4.067274	0.634175	0.525967	NA	0	0	0	2.232127	0	0
28721	LOC11962:	0.372021	2.579362	4.067274	0.634175	0.525967	NA	0	0	0	2.232127	0	0
28735	LOC11962:	0.356518	2.579362	4.067274	0.634175	0.525967	NA	0	0	0	0	2.139111	0
28747	LOC11962:	0.372021	2.579362	4.067274	0.634175	0.525967	NA	0	0	0	2.232127	0	0
28749	LOC1032З!	0.356518	2.579362	4.067274	0.634175	0.525967	NA	0	0	0	0	2.139111	0

28752	LOC11962:	0.372021	2.579362	4.067274	0.634175	0.525967	NA	0	0	0	2.232127	0	0
28777	LOC11962:	0.356518	2.579362	4.067274	0.634175	0.525967	NA	0	0	0	0	2.139111	0
28782	LOC11962:	0.356518	2.579362	4.067274	0.634175	0.525967	NA	0	0	0	0	2.139111	0
28785	LPAR4	0.356518	2.579362	4.067274	0.634175	0.525967	NA	0	0	0	0	2.139111	0
28801	LOC11962:	0.372021	2.579362	4.067274	0.634175	0.525967	NA	0	0	0	2.232127	0	0
28806	LOC11962:	0.356518	2.579362	4.067274	0.634175	0.525967	NA	0	0	0	0	2.139111	0
28816	TRNAR-UC	0.356518	2.579362	4.067274	0.634175	0.525967	NA	0	0	0	0	2.139111	0
28822	LOC11962:	0.372021	2.579362	4.067274	0.634175	0.525967	NA	0	0	0	2.232127	0	0
28823	LOC11962:	0.356518	2.579362	4.067274	0.634175	0.525967	NA	0	0	0	0	2.139111	0
28836	LOC11962:	0.372021	2.579362	4.067274	0.634175	0.525967	NA	0	0	0	2.232127	0	0
28846	LOC11962:	0.372021	2.579362	4.067274	0.634175	0.525967	NA	0	0	0	2.232127	0	0
28849	LOC11962:	0.356518	2.579362	4.067274	0.634175	0.525967	NA	0	0	0	0	2.139111	0
28860	LOC10321	0.356518	2.579362	4.067274	0.634175	0.525967	NA	0	0	0	0	2.139111	0
28872	LOC11962:	0.356518	2.579362	4.067274	0.634175	0.525967	NA	0	0	0	0	2.139111	0
28878	KRTAP25-1	0.356518	2.579362	4.067274	0.634175	0.525967	NA	0	0	0	0	2.139111	0
28879	LOC11962:	0.356518	2.579362	4.067274	0.634175	0.525967	NA	0	0	0	0	2.139111	0
28887	LOC11962:	0.356518	2.579362	4.067274	0.634175	0.525967	NA	0	0	0	0	2.139111	0
28889	LOC11962:	0.356518	2.579362	4.067274	0.634175	0.525967	NA	0	0	0	0	2.139111	0
28894	LOC11962:	0.372021	2.579362	4.067274	0.634175	0.525967	NA	0	0	0	2.232127	0	0
28910	LOC11962:	0.356518	2.579362	4.067274	0.634175	0.525967	NA	0	0	0	0	2.139111	0
28915	LOC11962:	0.372021	2.579362	4.067274	0.634175	0.525967	NA	0	0	0	2.232127	0	0
28924	LOC10322	0.372021	2.579362	4.067274	0.634175	0.525967	NA	0	0	0	2.232127	0	0
28928	LOC11962:	0.372021	2.579362	4.067274	0.634175	0.525967	NA	0	0	0	2.232127	0	0
28932	LOC11962:	0.356518	2.579362	4.067274	0.634175	0.525967	NA	0	0	0	0	2.139111	0
28948	LOC11962:	0.372021	2.579362	4.067274	0.634175	0.525967	NA	0	0	0	2.232127	0	0
28950	PTH2	0.356518	2.579362	4.067274	0.634175	0.525967	NA	0	0	0	0	2.139111	0
28970	ACTL9	0.372021	2.579362	4.067274	0.634175	0.525967	NA	0	0	0	2.232127	0	0
28974	CCL18	0.356518	2.579362	4.067274	0.634175	0.525967	NA	0	0	0	0	2.139111	0
28997	GPR82	0.372021	2.579362	4.067274	0.634175	0.525967	NA	0	0	0	2.232127	0	0
29016	LOC11962	0.372021	2.579362	4.067274	0.634175	0.525967	NA	0	0	0	2.232127	0	0
29023	LOC10324	0.372021	2.579362	4.067274	0.634175	0.525967	NA	0	0	0	2.232127	0	0
29024	LOC11962	0.372021	2.579362	4.067274	0.634175	0.525967	NA	0	0	0	2.232127	0	0
29029	LOC11962	0.356518	2.579362	4.067274	0.634175	0.525967	NA	0	0	0	0	2.139111	0
29034	LOC11962	0.372021	2.579362	4.067274	0.634175	0.525967	NA	0	0	0	2.232127	0	0
29037	LOC11962	0.372021	2.579362	4.067274	0.634175	0.525967	NA	0	0	0	2.232127	0	0
29040	LOC11962	0.356518	2.579362	4.067274	0.634175	0.525967	NA	0	0	0	0	2.139111	0
29049	KRTAP4-2	0.372021	2.579362	4.067274	0.634175	0.525967	NA	0	0	0	2.232127	0	0
29056	LOC10324	0.356518	2.579362	4.067274	0.634175	0.525967	NA	0	0	0	0	2.139111	0
29059	KRTAP3-1	0.356518	2.579362	4.067274	0.634175	0.525967	NA	0	0	0	0	2.139111	0
29061	KRTAP3-3	0.356518	2.579362	4.067274	0.634175	0.525967	NA	0	0	0	0	2.139111	0
29062	LOC11962	0.356518	2.579362	4.067274	0.634175	0.525967	NA	0	0	0	0	2.139111	0
29063	LOC11962	0.356518	2.579362	4.067274	0.634175	0.525967	NA	0	0	0	0	2.139111	0
29083	DEFB129	0.372021	2.579362	4.067274	0.634175	0.525967	NA	0	0	0	2.232127	0	0
29089	LOC11962	0.356518	2.579362	4.067274	0.634175	0.525967	NA	0	0	0	0	2.139111	0
29092	LOC11962	0.372021	2.579362	4.067274	0.634175	0.525967	NA	0	0	0	2.232127	0	0
29096	LOC11962	0.356518	2.579362	4.067274	0.634175	0.525967	NA	0	0	0	0	2.139111	0
29097	LOC10324	0.356518	2.579362	4.067274	0.634175	0.525967	NA	0	0	0	0	2.139111	0
29115	LOC11962	0.356518	2.579362	4.067274	0.634175	0.525967	NA	0	0	0	0	2.139111	0
29119	LOC11962	0.372021	2.579362	4.067274	0.634175	0.525967	NA	0	0	0	2.232127	0	0
29125	LOC10324	0.372021	2.579362	4.067274	0.634175	0.525967	NA	0	0	0	2.232127	0	0
29129	LOC11962	0.356518	2.579362	4.067274	0.634175	0.525967	NA	0	0	0	0	2.139111	0
29133	LOC10324	0.356518	2.579362	4.067274	0.634175	0.525967	NA	0	0	0	0	2.139111	0
29139	CATSPERD	0.372021	2.579362	4.067274	0.634175	0.525967	NA	0	0	0	2.232127	0	0
29145	LOC11962	0.356518	2.579362	4.067274	0.634175	0.525967	NA	0	0	0	0	2.139111	0
29153	LOC11962	0.356518	2.579362	4.067274	0.634175	0.525967	NA	0	0	0	0	2.139111	0
29157	ODAM	0.356518	2.579362	4.067274	0.634175	0.525967	NA	0	0	0	0	2.139111	0
29161	CSN2	0.372021	2.579362	4.067274	0.634175	0.525967	NA	0	0	0	2.232127	0	0
29162	LOC11962	0.356518	2.579362	4.067274	0.634175	0.525967	NA	0	0	0	0	2.139111	0
29170	LOC11962	0.372021	2.579362	4.067274	0.634175	0.525967	NA	0	0	0	2.232127	0	0
29171	LOC11962	0.356518	2.579362	4.067274	0.634175	0.525967	NA	0	0	0	0	2.139111	0
29178	LOC11962	0.372021	2.579362	4.067274	0.634175	0.525967	NA	0	0	0	2.232127	0	0
29187	CLECL1	0.372021	2.579362	4.067274	0.634175	0.525967	NA	0	0	0	2.232127	0	0
29202	LOC11962	0.372021	2.579362	4.067274	0.634175	0.525967	NA	0	0	0	2.232127	0	0
29225	LOC10323	0.356518	2.579362	4.067274	0.634175	0.525967	NA	0	0	0	0	2.139111	0
29226	LOC10323	0.356518	2.579362	4.067274	0.634175	0.525967	NA	0	0	0	0	2.139111	0
29232	LOC10322	0.372021	2.579362	4.067274	0.634175	0.525967	NA	0	0	0	2.232127	0	0
29246	LOC10324	0.372021	2.579362	4.067274	0.634175	0.525967	NA	0	0	0	2.232127	0	0
29247	LOC11961	0.356518	2.579362	4.067274	0.634175	0.525967	NA	0	0	0	0	2.139111	0
29248	LOC11962	0.356518	2.579362	4.067274	0.634175	0.525967	NA	0	0	0	0	2.139111	0
29294	LOC11962:	0.372021	2.579362	4.067274	0.634175	0.525967	NA	0	0	0	2.232127	0	0
29306	LOC11962:	0.356518	2.579362	4.067274	0.634175	0.525967	NA	0	0	0	0	2.139111	0
29321	LOC10323:	0.372021	2.579362	4.067274	0.634175	0.525967	NA	0	0	0	2.232127	0	0
29329	LOC10323:	0.356518	2.579362	4.067274	0.634175	0.525967	NA	0	0	0	0	2.139111	0
29331	LOC10322	0.372021	2.579362	4.067274	0.634175	0.525967	NA	0	0	0	2.232127	0	0

29380	LOC10323:	0.372021	2.579362	4.067274	0.634175	0.525967	NA	0	0	0	2.232127	0	0
29392	LOC10323:	0.372021	2.579362	4.067274	0.634175	0.525967	NA	0	0	0	2.232127	0	0
29424	LOC11962:	0.356518	2.579362	4.067274	0.634175	0.525967	NA	0	0	0	0	2.139111	0
29470	LOC10322:	0.356518	2.579362	4.067274	0.634175	0.525967	NA	0	0	0	0	2.139111	0
29484	LOC10324:	0.356518	2.579362	4.067274	0.634175	0.525967	NA	0	0	0	0	2.139111	0
29496	LOC10323:	0.372021	2.579362	4.067274	0.634175	0.525967	NA	0	0	0	2.232127	0	0
29497	LOC11962:	0.372021	2.579362	4.067274	0.634175	0.525967	NA	0	0	0	2.232127	0	0
29499	LOC11962:	0.372021	2.579362	4.067274	0.634175	0.525967	NA	0	0	0	2.232127	0	0
29501	LOC10322	0.372021	2.579362	4.067274	0.634175	0.525967	NA	0	0	0	2.232127	0	0
29513	LOC11962:	0.372021	2.579362	4.067274	0.634175	0.525967	NA	0	0	0	2.232127	0	0
29519	LOC11962:	0.356518	2.579362	4.067274	0.634175	0.525967	NA	0	0	0	0	2.139111	0
29531	LOC11962:	0.372021	2.579362	4.067274	0.634175	0.525967	NA	0	0	0	2.232127	0	0
29534	LOC10323(	0.372021	2.579362	4.067274	0.634175	0.525967	NA	0	0	0	2.232127	0	0
29535	LOC10324(	0.356518	2.579362	4.067274	0.634175	0.525967	NA	0	0	0	0	2.139111	0
29547	LOC10324	0.372021	2.579362	4.067274	0.634175	0.525967	NA	0	0	0	2.232127	0	0
29550	LOC10324!	0.372021	2.579362	4.067274	0.634175	0.525967	NA	0	0	0	2.232127	0	0
29551	LOC11962:	0.372021	2.579362	4.067274	0.634175	0.525967	NA	0	0	0	2.232127	0	0
29561	LOC10322:	0.356518	2.579362	4.067274	0.634175	0.525967	NA	0	0	0	0	2.139111	0
29563	LOC10322:	0.356518	2.579362	4.067274	0.634175	0.525967	NA	0	0	0	0	2.139111	0
29575	LOC11962:	0.372021	2.579362	4.067274	0.634175	0.525967	NA	0	0	0	2.232127	0	0
29587	LOC11962:	0.372021	2.579362	4.067274	0.634175	0.525967	NA	0	0	0	2.232127	0	0
12521	LOC10321	9.223349	2.578648	1.122386	2.297471	0.021592	0.045845	0	1.094824	6.957314	17.85702	21.39111	8.039836
15260	LOC10323:	4.632312	2.578044	1.443518	1.785945	0.074108	0.129106	0.542086	0	3.478657	8.928508	12.83466	2.009959
17538	LOC11961	2.05347	2.577828	1.827905	1.410264	0.158462	0.240205	0	1.094824	0.695731	2.232127	4.278221	4.019918
5176	SLA	26.75829	2.576635	0.561177	4.591485	4.40E-06	2.26E-05	6.505036	6.568942	9.740239	60.26743	51.33866	26.12947
11238	CYP3A43	7.061684	2.576317	1.001337	2.572876	0.010086	0.023858	1.084173	2.189647	2.782925	11.16063	17.11289	8.039836
19493	GUCA2A	1.443355	2.575915	2.338107	1.10171	0.270588	0.369034	0.542086	0	0.695731	0	6.417332	1.004979
15170	TAS2R14	4.579782	2.57557	1.429231	1.802067	0.071535	0.125377	0.542086	0	3.478657	4.464254	14.97377	4.019918
12686	PTCHD4	6.721923	2.575488	1.138374	2.262427	0.023671	0.049605	1.084173	0.547412	4.174388	13.39276	17.11289	4.019918
9483	MYOCD	31.14158	2.575335	0.861719	2.988603	0.002803	0.007857	1.626259	1.094824	24.3506	75.89232	57.75599	26.12947
9578	TRPM8	13.54346	2.574835	0.868111	2.96602	0.003017	0.008374	1.084173	4.926706	5.565851	17.85702	42.78221	9.044815
16142	LOC11962(	3.316917	2.574113	1.570383	1.639163	0.101179	0.166637	0.542086	2.189647	0	4.464254	10.69555	2.009959
17441	TAS2R60	2.888485	2.573945	1.813242	1.419526	0.155746	0.2374	0	1.094824	1.391463	0	12.83466	2.009959
4057	TNIP3	31.0137	2.573331	0.48305	5.327251	9.97E-08	6.53E-07	11.38381	8.211177	6.957314	55.80317	53.47777	50.24897
6335	LOC11962	23.60657	2.573063	0.641522	4.010874	6.05E-05	0.000254	5.96295	2.189647	12.52316	35.71403	47.06043	38.18922
13763	SCNN1D	5.046129	2.571885	1.25188	2.054419	0.039935	0.07714	0.542086	1.642235	2.087194	11.16063	12.83466	2.009959
10398	LOC10322:	15.19396	2.571832	0.929536	2.766792	0.005661	0.014474	1.084173	1.094824	11.1317	17.85702	44.92132	15.07469
15375	LOC10322:	4.617697	2.571789	1.457479	1.764547	0.07764	0.134248	0	0.547412	3.478657	6.696381	14.97377	2.009959
16018	LOC10324:	2.698838	2.57124	1.546853	1.662239	0.096465	0.160105	0.542086	1.094824	0.695731	6.696381	2.139111	5.024897
14830	CCDC170	3.548468	2.570969	1.377287	1.866691	0.061945	0.111045	1.084173	0.547412	1.391463	6.696381	8.556443	3.014938
15354	LOC10323	5.901842	2.569268	1.452976	1.76828	0.077014	0.13333	2.168345	2.737059	0	13.39276	17.11289	0
1403	FCHO1	121.9959	2.569141	0.269831	9.521299	1.71E-21	3.24E-20	42.82482	27.37059	35.4823	220.9806	188.2417	217.0756
13448	LOC11961!	5.857678	2.566971	1.212093	2.1178	0.034192	0.067593	0	1.642235	3.478657	11.16063	12.83466	6.029877
623	RASAL1	434.0818	2.5662	0.19222	13.35033	1.18E-40	5.03E-39	138.7741	97.98671	141.2335	676.3345	673.8199	876.3421
8945	SCN4A	14.70341	2.563781	0.818275	3.133152	0.001729	0.00514	1.626259	2.189647	9.044508	33.4819	27.80844	14.06971
16071	ID4	3.571769	2.563563	1.551193	1.652639	0.098404	0.162762	0	1.642235	1.391463	6.696381	10.69555	1.004979
15083	CAMK1G	6.272854	2.56346	1.409133	1.819175	0.068885	0.121415	0	0	5.565851	8.928508	17.11289	6.029877
15824	ARHGEF38	3.804208	2.563104	1.512273	1.694868	0.0901	0.151373	0	0.547412	2.782925	8.928508	8.556443	2.009959
17460	LOC11962:	3.157952	2.563025	1.80859	1.417139	0.156442	0.238203	0	0	2.782925	4.464254	10.69555	1.004979
14681	CELA1	5.269088	2.561718	1.353592	1.892533	0.05842	0.10579	0.542086	0.547412	3.478657	8.928508	17.11289	1.004979
5580	LOC10324	24.48255	2.560885	0.584514	4.381223	1.18E-05	5.62E-05	7.047123	8.211177	5.565851	58.0353	44.92132	23.11453
10144	A2ML1	10.95776	2.560714	0.905805	2.827005	0.004699	0.012314	1.626259	1.642235	6.261582	26.78552	21.39111	8.039836
13956	TNFRSF4	4.973492	2.56014	1.267188	2.020331	0.043349	0.082576	1.626259	0	2.782925	6.696381	10.69555	8.039836
17564	ADH6	2.090498	2.559754	1.821809	1.405062	0.160003	0.242168	0	1.094824	0.695731	4.464254	4.278221	2.009959
12575	LOC11961	5.621523	2.559213	1.11811	2.288874	0.022087	0.046694	0.542086	1.642235	2.782925	11.16063	8.556443	9.044815
15400	LOC10322!	4.130938	2.558459	1.454084	1.759499	0.078493	0.135493	2.168345	0	1.391463	2.232127	14.97377	4.019918
17087	LOC11962!	2.089611	2.55827	1.724391	1.483579	0.137921	0.214548	0.542086	0.547412	0.695731	4.464254	4.278221	2.009959
17088	MAGEB17	2.089611	2.55827	1.724391	1.483579	0.137921	0.214548	0.542086	0.547412	0.695731	4.464254	4.278221	2.009959
17089	LOC11962(	2.089611	2.55827	1.724391	1.483579	0.137921	0.214548	0.542086	0.547412	0.695731	4.464254	4.278221	2.009959
292	ARMCX1	602.3857	2.558193	0.145241	17.61349	1.94E-69	1.77E-67	162.0838	200.9001	161.4097	991.0644	1039.608	1059.248
6057	PRKCG	26.89545	2.557956	0.617593	4.141814	3.45E-05	0.000151	3.794605	7.663765	11.82743	62.49956	53.47777	22.10955
7796	CYP2A23	17.78408	2.555948	0.737393	3.466193	0.000528	0.0018	4.336691	2.737059	8.348776	29.01765	49.19955	13.06473
10944	LOC11962(	8.177481	2.555811	0.968009	2.640275	0.008284	0.020123	1.084173	3.831883	2.087194	22.32127	10.69555	9.044815
9210	LOC10322:	11.23551	2.555187	0.836868	3.053275	0.002264	0.006533	1.626259	3.284471	4.87012	15.62489	29.94755	12.05975
1701	SPEF1	88.05976	2.555006	0.292702	8.729048	2.57E-18	4.01E-17	26.02015	24.08612	26.43779	167.4095	149.7377	134.6672
14080	NEUROD2	9.350727	2.554748	1.278611	1.998065	0.04571	0.086316	0	0	8.348776	11.16063	23.53022	13.06473
2739	FLRT1	50.89351	2.554483	0.375995	6.79393	1.09E-11	1.06E-10	14.09425	14.23271	16.00182	98.21359	83.42532	79.39338
11586	COPZ2	9.116314	2.553814	1.022329	2.498035	0.012488	0.028656	3.794605	0.547412	3.478657	29.01765	12.83466	5.024897
16061	LOC10322!	3.773202	2.551969	1.542003	1.65497	0.09793	0.1621	0	0.547412	2.782925	4.464254	12.83466	2.009959
14225	LOC10323:	5.065714	2.551893	1.294248	1.971718	0.048642	0.090906	0	1.642235	2.782925	13.39276	8.556443	4.019918
18007	LOC11962	2.126639	2.550991	1.901737	1.3414	0.179791	0.265421	0.542086	0.547412	0.695731	6.696381	4.278221	0
13875	LOC11962	5.175508	2.550231	1.25314	2.035072	0.041844	0.080174	2.168345	2.189647	0	6.696381	14.97377	5.024897
177	NR1H4	1122.935	2.549439	0.121778	20.93509	2.57E-97	3.85E-95	295.9792	364.5763	322.1236	1886.147	1974.399	1894.386
7024	LOC11962	14.92514	2.549238	0.68423	3.725703	0.000195	0.000737	4.878777	3.284471	4.87012	22.32127	32.08666	22.10955

13529	HSPB9	5.048436	2.548983	1.212693	2.101918	0.03556	0.069878	1.084173	0.547412	2.782925	11.16063	10.69555	4.019918
11048	LOC10323:	7.550976	2.548577	0.974217	2.616026	0.008896	0.021407	2.168345	2.189647	2.087194	17.85702	14.97377	6.029877
18212	GKN1	2.344463	2.547531	1.960847	1.299199	0.193876	0.28301	0	0	2.087194	6.696381	4.278221	1.004979
17972	LOC11962(	2.058605	2.546605	1.888933	1.348172	0.177603	0.262719	0.542086	0.547412	0.695731	0	8.556443	2.009959
14824	LOC11962:	4.162666	2.546049	1.363186	1.86772	0.061801	0.110833	0	1.642235	2.087194	8.928508	4.278221	8.039836
1004	LRRC6	217.0524	2.545637	0.232011	10.97207	5.21E-28	1.38E-26	59.6295	80.46954	49.39693	339.2833	427.8221	345.7129
11567	CFAP47	10.03239	2.544956	1.017262	2.501771	0.012357	0.028402	0	3.284471	5.565851	15.62489	25.66933	10.04979
6377	LOC11962!	19.08613	2.544888	0.637722	3.990593	6.59E-05	0.000275	5.420864	5.474118	5.565851	46.87467	32.08666	19.09461
111	FOLR1	2682.028	2.544842	0.106386	23.92084	1.86E-126	4.45E-124	736.6954	878.0485	738.8667	4602.646	4504.967	4630.945
12508	LOC10322:	7.362781	2.543326	1.105792	2.300003	0.021448	0.045587	0.542086	1.094824	4.87012	13.39276	19.252	5.024897
15188	LOC10321:	3.351117	2.541912	1.413813	1.797913	0.072191	0.126367	0.542086	1.642235	0.695731	8.928508	4.278221	4.019918
19140	CALML3	13.35103	2.541039	2.185527	1.162666	0.244965	0.34025	0	0	11.82743	8.928508	34.22577	25.12449
9670	LOC11962:	12.94204	2.540049	0.864263	2.938978	0.003293	0.009053	2.168345	1.642235	7.653045	15.62489	38.50399	12.05975
14745	FCN2	5.177589	2.539733	1.349649	1.881773	0.059867	0.107939	0	0.547412	4.174388	4.464254	12.83466	9.044815
11709	TMEM132I	16.82265	2.538552	1.027901	2.469648	0.013525	0.030707	0	1.094824	13.91463	33.4819	36.36488	16.07967
18251	LMOD2	2.32896	2.538491	1.96341	1.292899	0.196046	0.285536	0	0	2.087194	4.464254	6.417332	1.004979
18252	SOWAHD	2.32896	2.538491	1.96341	1.292899	0.196046	0.285536	0	0	2.087194	4.464254	6.417332	1.004979
18253	LOC11962:	2.32896	2.538491	1.96341	1.292899	0.196046	0.285536	0	0	2.087194	4.464254	6.417332	1.004979
14664	ALOX12	5.251645	2.537455	1.338857	1.89524	0.058061	0.105261	0	0.547412	4.174388	8.928508	12.83466	5.024897
6322	LOC11962!	22.10686	2.537156	0.631809	4.015705	5.93E-05	0.000249	5.96295	4.379295	9.044508	31.24978	59.8951	22.10955
10528	CACNA1F	24.06214	2.536422	0.926222	2.738461	0.006173	0.015587	0	3.284471	18.08902	53.57105	51.33866	18.08963
17011	SPO11	2.742777	2.53634	1.701467	1.490679	0.136046	0.212602	0	1.642235	0.695731	6.696381	6.417332	1.004979
17012	LOC10321!	2.742777	2.53634	1.701467	1.490679	0.136046	0.212602	0	1.642235	0.695731	6.696381	6.417332	1.004979
14991	LOC11962(	3.334726	2.536257	1.381647	1.835677	0.066406	0.117763	1.084173	1.094824	0.695731	6.696381	6.417332	4.019918
15060	LOC11962(	4.985538	2.536132	1.391304	1.822845	0.068327	0.120616	1.626259	0	2.782925	2.232127	19.252	4.019918
16251	CUNH14or	2.689358	2.535714	1.568234	1.616924	0.105895	0.173233	0.542086	1.094824	0.695731	2.232127	8.556443	3.014938
15226	LOC11962(	3.318336	2.534484	1.415159	1.790954	0.073301	0.127985	1.626259	0.547412	0.695731	4.464254	8.556443	4.019918
6936	ADGRE2	18.43006	2.533553	0.673386	3.762405	0.000168	0.000645	2.168345	7.116354	6.957314	42.41041	27.80844	24.11951
21110	CA3	1.298534	2.532755	2.932304	0.863742	0.387729	0.488289	0	1.094824	0	6.696381	0	0
15095	LOC10321:	3.319224	2.531574	1.393364	1.816879	0.069236	0.121935	1.084173	1.094824	0.695731	4.464254	8.556443	4.019918
92	SUSD6	2471.681	2.531474	0.102198	24.77018	1.88E-135	5.43E-133	758.9209	701.2345	728.4307	4138.363	3961.633	4541.502
12454	LOC10324:	7.156273	2.531233	1.095781	2.309981	0.020889	0.044591	1.084173	4.379295	0.695731	22.32127	6.417332	8.039836
3358	TSHZ2	58.40203	2.529429	0.422928	5.98076	2.22E-09	1.76E-08	13.55216	13.13788	25.04633	89.28508	139.0422	70.34856
8998	IL2RB	16.49081	2.528787	0.811378	3.116657	0.001829	0.005404	1.084173	3.831883	9.740239	42.41041	27.80844	14.06971
19918	TRNAT-UG	1.208088	2.528251	2.420964	1.044316	0.296339	0.395525	0.542086	0.547412	0	0	2.139111	4.019918
14223	LCK	9.235761	2.527093	1.28085	1.972981	0.048498	0.09065	0	0	8.348776	15.62489	21.39111	10.04979
9026	ZNF83	19.98585	2.526804	0.813022	3.107915	0.001884	0.005549	2.168345	4.379295	11.1317	29.01765	64.17332	9.044815
12502	ICAM1	6.093538	2.526406	1.098123	2.300658	0.021411	0.04553	2.710432	0.547412	2.087194	15.62489	8.556443	7.034856
16638	LOC10322:	3.822905	2.526232	1.628521	1.551243	0.120843	0.19309	0	0	3.478657	11.16063	4.278221	4.019918
16397	DRGX	4.568361	2.525737	1.590006	1.588507	0.112172	0.18186	0	0	4.174388	2.232127	14.97377	6.029877
803	GPR15	198.494	2.52547	0.209203	12.07189	1.49E-33	4.92E-32	66.13454	60.2153	49.39693	328.1227	344.3968	342.698
6920	LOC10323:	20.25644	2.525445	0.670393	3.767112	0.000165	0.000634	3.252518	4.379295	10.43597	29.01765	51.33866	23.11453
17731	NKAIN3	2.981238	2.525268	1.8308	1.379325	0.167795	0.251583	0	0.547412	2.087194	6.696381	8.556443	0
14686	LOC11962(	3.935362	2.525206	1.334817	1.8918	0.058518	0.10593	0.542086	2.189647	0.695731	4.464254	10.69555	5.024897
1639	LOC10322:	117.2959	2.524881	0.284373	8.878763	6.76E-19	1.10E-17	35.7777	26.82318	42.43961	160.7131	213.9111	224.1104
14513	NPY4R2	4.002507	2.524816	1.314147	1.921258	0.054699	0.100198	1.084173	1.642235	0.695731	11.16063	6.417332	3.014938
1539	LOC11962:	109.8086	2.524601	0.276501	9.130516	6.82E-20	1.18E-18	28.73058	32.2973	36.17803	191.9629	213.9111	155.7718
9444	LOC10322!	20.6601	2.524413	0.841919	2.998402	0.002714	0.00764	5.96295	0.547412	11.82743	37.94616	55.61688	12.05975
11672	CUNH19or	11.94382	2.524216	1.019106	2.476892	0.013253	0.030186	0.542086	0.547412	9.740239	20.08914	25.66933	15.07469
13093	SDS	8.905541	2.523968	1.153301	2.188473	0.028635	0.058143	1.084173	0	6.957314	26.78552	8.556443	10.04979
13407	LOC11962!	5.20688	2.523785	1.187154	2.125912	0.033511	0.066449	2.168345	1.094824	1.391463	13.39276	2.139111	11.05477
16941	SPP2	2.92782	2.523549	1.681565	1.500715	0.133429	0.209387	0.542086	0	2.087194	2.232127	10.69555	2.009959
14730	LOC11962:	5.909946	2.522792	1.339413	1.883505	0.059632	0.107625	0	1.094824	4.174388	8.928508	19.252	2.009959
17098	CCL5	2.469594	2.5222	1.702133	1.481788	0.138397	0.215188	0.542086	1.642235	0	4.464254	2.139111	6.029877
14038	TLX1	6.751613	2.522018	1.256751	2.006777	0.044773	0.084789	0	0.547412	5.565851	13.39276	14.97377	6.029877
20347	LOC11962:	1.261506	2.521797	2.600591	0.969702	0.332195	0.43404	0	1.094824	0	4.464254	0	2.009959
13807	BHMT	5.037016	2.521718	1.232372	2.046232	0.040734	0.078431	0.542086	0.547412	3.478657	8.928508	10.69555	6.029877
9290	CUNH14or	11.6431	2.520954	0.830755	3.034533	0.002409	0.006896	2.710432	6.02153	1.391463	17.85702	27.80844	14.06971
13363	HEPHL1	7.844368	2.520428	1.181039	2.134076	0.032837	0.065327	0.542086	2.189647	4.174388	15.62489	23.53022	1.004979
9366	SKAP1	12.5895	2.520045	0.835903	3.014758	0.002572	0.0073	1.626259	3.284471	6.261582	20.08914	34.22577	10.04979
9234	DHRS9	10.21378	2.519947	0.827168	3.046475	0.002315	0.006666	2.168345	2.189647	4.87012	17.85702	17.11289	17.08465
20511	LOC11962(	1.297647	2.519869	2.668181	0.944414	0.344958	0.447112	0.542086	0.547412	0	6.696381	0	0
12345	LOC10323!	11.98775	2.51919	1.078779	2.335225	0.019532	0.042062	0	1.094824	9.740239	20.08914	29.94755	11.05477
16006	LOC10322(	3.083069	2.518676	1.514085	1.663497	0.096213	0.159804	1.626259	1.094824	0	4.464254	4.278221	7.034856
15561	LOC10322:	4.333094	2.518533	1.452134	1.734367	0.082853	0.141549	0	0.547412	3.478657	2.232127	10.69555	9.044815
8741	NUP210L	13.63655	2.518393	0.789325	3.190564	0.00142	0.004319	3.794605	3.284471	4.87012	22.32127	38.50399	9.044815
11194	DPEP2	9.716686	2.518314	0.974868	2.583236	0.009788	0.023245	3.252518	1.094824	4.174388	20.08914	25.66933	4.019918
18097	SLC4A1	29.40512	2.517731	1.903685	1.322557	0.185983	0.273214	0	0.547412	25.74206	46.87467	79.14709	24.11951
20452	LOC10323!	1.208975	2.517205	2.638876	0.953893	0.340138	0.442136	0	1.094824	0	0	2.139111	4.019918
16581	LOC10323:	3.754872	2.51709	1.61317	1.560337	0.11868	0.190285	0	0	3.478657	4.464254	8.556443	6.029877
8028	LOC10324(	22.74875	2.516138	0.739188	3.403922	0.000664	0.0022	3.252518	3.831883	13.2189	37.94616	64.17332	14.06971
18956	TRNAC-GC	1.9284	2.515505	2.107268	1.193728	0.232584	0.32618	0.542086	1.094824	0	8.928508	0	1.004979
4654	KCNC3	48.97951	2.515167	0.511691	4.915404	8.86E-07	5.06E-06	7.047123	9.853413	27.13352	82.5887	106.9555	60.29877
19278	LOC11962!	2.297954	2.515034	2.207777	1.139171	0.254632	0.351103	0	0	2.087194	0	10.69555	1.004979

7588	TPRG1	19.43498	2.513651	0.71212	3.529813	0.000416	0.001457	3.794605	4.379295	9.044508	42.41041	44.92132	12.05975
14665	HAAO	4.226354	2.513492	1.326586	1.894706	0.058131	0.105381	0.542086	1.094824	2.087194	8.928508	10.69555	2.009959
15092	LOC11962(	3.939609	2.513426	1.382611	1.817884	0.069082	0.12169	1.626259	1.094824	0.695731	2.232127	14.97377	3.014938
15387	LOC10322{	4.211739	2.511968	1.425007	1.762776	0.077938	0.134658	0	1.642235	2.087194	6.696381	12.83466	2.009959
5899	SLC51A	25.32803	2.511358	0.596191	4.212338	2.53E-05	0.000114	7.047123	8.211177	6.957314	64.73168	44.92132	20.09959
12250	LOC10322	6.66583	2.51118	1.06586	2.356014	0.018472	0.040088	0.542086	3.284471	2.087194	8.928508	17.11289	8.039836
17076	LOC11962	2.776089	2.510844	1.691392	1.484484	0.137681	0.214347	0.542086	0.547412	1.391463	11.16063	0	3.014938
2976	MAP2K6	54.34334	2.510343	0.388846	6.455882	1.08E-10	9.61E-10	13.55216	13.13788	22.2634	82.5887	109.0946	85.42325
12915	LOC11962	5.415224	2.510209	1.131341	2.21879	0.026501	0.054578	1.626259	0.547412	2.782925	8.928508	8.556443	10.04979
12540	LOC10321	6.431616	2.510084	1.09367	2.295103	0.021727	0.046062	1.626259	3.284471	0.695731	6.696381	19.252	7.034856
7873	PPP1R14A	13.35435	2.509975	0.728048	3.447542	0.000566	0.00191	2.710432	3.831883	5.565851	26.78552	17.11289	24.11951
19861	LOC11962	1.244228	2.509476	2.397402	1.046748	0.295216	0.395012	1.084173	0	0	2.232127	2.139111	2.009959
19457	LOC10322{	2.182364	2.50935	2.263155	1.108784	0.267523	0.365529	0.542086	0	1.391463	11.16063	0	0
14337	EDAR	4.773571	2.509266	1.283681	1.954743	0.050613	0.093852	0.542086	1.642235	2.087194	2.232127	17.11289	5.024897
20152	CLEC2A	1.441415	2.507991	2.499234	1.003504	0.315618	0.416371	0	0	1.391463	2.232127	0	5.024897
11410	NOX3	8.903197	2.507478	0.989575	2.533893	0.01128	0.026283	3.252518	1.094824	3.478657	22.32127	19.252	4.019918
13896	LOC11961	8.257603	2.50634	1.234361	2.030475	0.042308	0.080942	0	0.547412	6.957314	15.62489	21.39111	5.024897
14746	POU3F1	5.043926	2.505932	1.331831	1.881569	0.059895	0.107982	0	1.094824	3.478657	6.696381	14.97377	4.019918
19476	LOC11961{	1.46294	2.505868	2.269039	1.104374	0.269431	0.367739	0	0	1.391463	2.232127	2.139111	3.014938
19477	LOC11962(	1.46294	2.505868	2.269039	1.104374	0.269431	0.367739	0	0	1.391463	2.232127	2.139111	3.014938
15985	CUNH10or	4.281677	2.505863	1.502168	1.668164	0.095283	0.158461	2.168345	1.642235	0	0	12.83466	9.044815
11941	RAB25	6.70197	2.505816	1.036996	2.416419	0.015674	0.034896	1.084173	2.737059	2.087194	11.16063	17.11289	6.029877
4681	LOC10323	26.74097	2.505593	0.51175	4.896123	9.77E-07	5.55E-06	7.589209	8.211177	8.348776	35.71403	51.33866	49.24399
17826	LRRC3C	2.07819	2.504993	1.824871	1.372696	0.169847	0.253303	0	0.547412	1.391463	2.232127	4.278221	4.019918
20064	CDH7	1.478443	2.504979	2.458807	1.018778	0.308308	0.408512	0	0	1.391463	4.464254	0	3.014938
3328	LOC11962	44.48795	2.504697	0.416146	6.018791	1.76E-09	1.40E-08	9.215468	14.78012	16.00182	82.5887	77.00798	67.33362
14428	LOC10323	5.907118	2.50468	1.291872	1.938799	0.052526	0.096784	0	0.547412	4.87012	11.16063	12.83466	6.029877
16753	CEMP1	3.796563	2.504314	1.634099	1.532535	0.12539	0.198988	0	3.284471	0	8.928508	8.556443	2.009959
15737	SPIC	4.412285	2.504097	1.462805	1.711846	0.086925	0.146845	0.542086	0	3.478657	4.464254	14.97377	3.014938
8841	DUOX2	21.60906	2.503601	0.792539	3.158962	0.001583	0.004761	2.710432	2.189647	14.61036	46.87467	49.19955	14.06971
18449	LOC11962	2.041162	2.503304	1.991642	1.256905	0.208788	0.300847	0	0.547412	1.391463	0	4.278221	6.029877
5780	DLX3	25.34012	2.501296	0.583803	4.284488	1.83E-05	8.42E-05	4.878777	4.926706	13.2189	44.64254	49.19955	35.17428
14663	LOC10321{	5.606645	2.50091	1.319437	1.895437	0.058035	0.105221	0	1.642235	3.478657	2.232127	19.252	7.034856
17938	SFRP2	2.529035	2.50017	1.84739	1.353353	0.175943	0.260756	0	2.189647	0	6.696381	4.278221	2.009959
17177	LOC10321	2.473841	2.499915	1.699283	1.471159	0.141248	0.218611	1.626259	0.547412	0	2.232127	6.417332	4.019918
5141	LOC11962	27.64185	2.499064	0.542413	4.607313	4.08E-06	2.11E-05	5.420864	7.663765	11.82743	46.87467	59.8951	34.1693
1511	HAVCR2	120.3803	2.498936	0.270862	9.225848	2.81E-20	4.95E-19	41.19856	35.58177	31.30791	174.1059	248.1368	191.9511
14490	LOC11962(	5.605096	2.498468	1.296713	1.92677	0.054008	0.09909	3.794605	1.094824	0	4.464254	19.252	5.024897
13766	SP9	6.443806	2.498414	1.216624	2.053563	0.040018	0.077283	0.542086	1.094824	4.174388	24.5534	4.278221	4.019918
10833	ACOT4	10.99343	2.49834	0.937888	2.663795	0.007726	0.018961	3.252518	1.642235	4.87012	20.08914	32.08666	4.019918
414	S100A1	16712.71	2.497829	0.160863	15.52769	2.25E-54	1.45E-52	4010.355	6648.864	4422.069	29731.93	29532.56	25930.48
16196	CD53	3.41109	2.49746	1.534474	1.627567	0.103617	0.170082	1.626259	0	1.391463	11.16063	4.278221	2.009959
20540	LOC10322	1.283031	2.496624	2.649045	0.942462	0.345956	0.447515	0	1.094824	0	4.464254	2.139111	0
20562	ARHGDIB	1.283031	2.496624	2.649045	0.942462	0.345956	0.447515	0	1.094824	0	4.464254	2.139111	0
20567	LOC10323	1.283031	2.496624	2.649045	0.942462	0.345956	0.447515	0	1.094824	0	4.464254	2.139111	0
10889	BARHL2	16.13334	2.495819	0.940948	2.652452	0.007991	0.01951	0.542086	1.642235	12.52316	37.94616	32.08666	12.05975
18095	LOC11962	1.860366	2.495583	1.88677	1.322675	0.185944	0.273186	0.542086	1.094824	0	2.232127	4.278221	3.014938
11230	DAND5	9.770471	2.495216	0.969157	2.574625	0.010035	0.023756	2.710432	0.547412	5.565851	26.78552	14.97377	8.039836
6392	LOC11962	25.83836	2.493377	0.62615	3.982074	6.83E-05	0.000284	3.794605	7.663765	11.82743	40.17829	68.45154	23.11453
10372	ITGB7	11.38733	2.492893	0.899719	2.770746	0.005593	0.014335	0.542086	5.474118	4.174388	24.5534	23.53022	10.04979
10721	LOC10323(	12.06161	2.492672	0.927799	2.686651	0.007217	0.017897	1.084173	2.189647	7.653045	22.32127	32.08666	7.034856
9352	ANO1	12.28578	2.492433	0.825736	3.018437	0.002541	0.007223	1.626259	3.831883	5.565851	31.24978	21.39111	10.04979
5643	LOC11962(	25.54188	2.490842	0.572629	4.349834	1.36E-05	6.42E-05	5.420864	6.568942	11.1317	37.94616	62.03421	30.14938
20469	LOC11962	1.230501	2.490553	2.618749	0.951047	0.341581	0.443642	0	1.094824	0	0	4.278221	2.009959
8383	ACVRL1	19.78924	2.49043	0.757398	3.28814	0.001009	0.003198	1.626259	3.284471	13.2189	35.71403	42.78221	22.10955
8443	IPCEF1	14.47528	2.490165	0.76138	3.270595	0.001073	0.003379	1.626259	5.474118	6.261582	13.39276	29.94755	30.14938
7841	LOC11962	20.1444	2.489676	0.720471	3.455623	0.000549	0.001861	3.252518	3.284471	11.82743	26.78552	55.61688	20.09959
11813	RNF212B	6.499128	2.488854	1.017528	2.44598	0.014446	0.03251	2.168345	1.642235	2.087194	15.62489	6.417332	11.05477
6409	ENDOU	24.76341	2.488803	0.626131	3.974893	7.04E-05	0.000292	3.794605	4.926706	13.91463	40.17829	55.61688	30.14938
7355	VAX1	24.35058	2.488667	0.688401	3.615142	0.0003	0.001085	2.710432	5.474118	13.91463	53.57105	51.33866	19.09461
17871	LOC11962	3.015438	2.488206	1.821907	1.365715	0.172028	0.25591	0	0	2.782925	11.16063	2.139111	2.009959
12621	LOC10324	6.776558	2.488176	1.092736	2.277015	0.022785	0.047998	2.710432	0.547412	2.782925	15.62489	14.97377	4.019918
18931	FSHR	1.898282	2.488141	2.076392	1.1983	0.2308	0.324098	0	1.642235	0	4.464254	4.278221	1.004979
15976	LOC11962	4.031475	2.48812	1.490609	1.669197	0.095078	0.158219	2.168345	0	1.391463	8.928508	10.69555	1.004979
13182	RAMP2	5.460049	2.487967	1.144592	2.173672	0.02973	0.059958	0.542086	1.642235	2.782925	8.928508	12.83466	6.029877
19598	LOC11962	1.499968	2.486769	2.289008	1.086396	0.277304	0.376052	0	0	1.391463	4.464254	2.139111	1.004979
19601	LOC11962	1.499968	2.486769	2.289008	1.086396	0.277304	0.376052	0	0	1.391463	4.464254	2.139111	1.004979
19602	LOC11961	1.499968	2.486769	2.289008	1.086396	0.277304	0.376052	0	0	1.391463	4.464254	2.139111	1.004979
10932	FAM162B	8.893552	2.485603	0.939983	2.644305	0.008186	0.019907	1.626259	2.189647	4.174388	20.08914	19.252	6.029877
15587	CXCL10	3.328441	2.484127	1.434628	1.731548	0.083354	0.142168	1.084173	0.547412	1.391463	2.232127	10.69555	4.019918
14790	LOC10323	8.2763	2.483431	1.324571	1.874895	0.060807	0.109301	0	0	7.653045	17.85702	17.11289	7.034856
16571	LOC10323	3.164035	2.483002	1.589355	1.56227	0.118225	0.189675	1.084173	1.642235	0	6.696381	8.556443	1.004979
10969	HHATL	11.03632	2.482956	0.942068	2.635645	0.008398	0.020353	1.626259	2.737059	5.565851	22.32127	29.94755	4.019918
299	MAP4K1	564.7488	2.482751	0.141819	17.50648	1.28E-68	1.14E-66	172.3835	170.7925	171.8456	852.6725	1011.799	1008.999

15230	LOC10324	4.047865	2.482593	1.386672	1.790324	0.073402	0.128128	1.626259	0.547412	1.391463	11.16063	8.556443	1.004979
16170	LOC11961	3.747393	2.482294	1.520532	1.632516	0.102571	0.168636	1.626259	1.642235	0	2.232127	14.97377	2.009959
16436	LOC10322	2.714078	2.481985	1.56775	1.583151	0.113387	0.183402	0.542086	0.547412	1.391463	2.232127	8.556443	3.014938
15443	UPP2	3.997997	2.481715	1.415777	1.7529	0.079619	0.137064	0	2.189647	1.391463	6.696381	10.69555	3.014938
11365	LOC10323	9.08037	2.481582	0.975005	2.545199	0.010922	0.025544	1.084173	1.642235	5.565851	15.62489	23.53022	7.034856
13260	RXFP3	6.366292	2.481181	1.15107	2.155542	0.031119	0.062391	0.542086	1.094824	4.174388	13.39276	14.97377	4.019918
13328	XPNPEP2	7.867147	2.480796	1.157684	2.142895	0.032122	0.064072	0	1.642235	5.565851	17.85702	17.11289	5.024897
221	ITGB2	2000.845	2.480291	0.127862	19.39816	8.00E-84	9.62E-82	670.5608	520.0412	635.8985	3196.406	3238.614	3743.549
19615	TNFRSF13	22.05287	2.479221	2.288094	1.083531	0.278573	0.377561	0	0	20.17621	37.94616	47.06043	27.13445
18242	LOC11962	1.896507	2.47911	1.915855	1.293997	0.195666	0.285155	1.084173	0.547412	0	4.464254	4.278221	1.004979
89	ZC3H12A	4897.332	2.477505	0.099179	24.98002	1.01E-137	3.01E-135	1458.212	1465.421	1552.177	8263.334	7613.095	9031.75
11412	ADRA1B	8.910474	2.477338	0.97785	2.533454	0.011294	0.026307	2.710432	0.547412	4.87012	22.32127	14.97377	8.039836
20161	LOC10324	1.447437	2.47699	2.472909	1.00165	0.316513	0.417323	0	0	1.391463	0	4.278221	3.014938
20163	LOC11962	1.447437	2.47699	2.472909	1.00165	0.316513	0.417323	0	0	1.391463	0	4.278221	3.014938
8928	PRSS56	12.86773	2.476326	0.789194	3.137791	0.001702	0.005069	3.252518	1.642235	6.957314	29.01765	19.252	17.08465
13401	MEIOB	5.494249	2.475785	1.163821	2.12729	0.033396	0.066251	0.542086	1.094824	3.478657	13.39276	6.417332	8.039836
6741	ZNF154	20.00211	2.475308	0.644448	3.840975	0.000123	0.000483	5.96295	3.831883	8.348776	35.71403	47.06043	19.09461
12988	KLK15	7.736054	2.474149	1.121488	2.206131	0.027375	0.056039	0.542086	1.642235	4.87012	26.78552	8.556443	4.019918
11192	FCRLB	18.18451	2.473246	0.957338	2.583463	0.009781	0.023235	1.626259	0.547412	14.61036	26.78552	53.47777	12.05975
19647	LOC11961	1.484465	2.473195	2.294542	1.07786	0.281096	0.380341	0	0	1.391463	2.232127	4.278221	1.004979
19648	LOC11962	1.484465	2.473195	2.294542	1.07786	0.281096	0.380341	0	0	1.391463	2.232127	4.278221	1.004979
14182	PTAFR	4.863496	2.469817	1.247453	1.979888	0.047716	0.089447	0.542086	0.547412	3.478657	11.16063	6.417332	7.034856
16917	XCL1	4.505926	2.468526	1.641705	1.503635	0.132675	0.208499	0	0	4.174388	11.16063	10.69555	1.004979
17699	LOC11962	2.96893	2.467811	1.782892	1.384162	0.166309	0.249792	0	0	2.782925	4.464254	8.556443	2.009959
17700	LOC10322	2.96893	2.467811	1.782892	1.384162	0.166309	0.249792	0	0	2.782925	4.464254	8.556443	2.009959
20126	LOC11961	1.266641	2.466949	2.446265	1.008455	0.313236	0.413762	0.542086	0.547412	0	2.232127	4.278221	0
12460	LOC11962	12.85473	2.466154	1.068361	2.308352	0.02098	0.044763	2.168345	0	9.740239	13.39276	42.78221	9.044815
6914	NFE2	20.72812	2.46567	0.654114	3.76948	0.000164	0.000629	2.710432	6.568942	9.740239	46.87467	36.36488	22.10955
3854	AEBP1	52.41707	2.465162	0.448608	5.49514	3.90E-08	2.69E-07	13.55216	14.78012	19.48048	84.82083	132.6249	49.24399
9145	FSD2	14.5938	2.464414	0.803352	3.067663	0.002157	0.006272	3.794605	3.831883	5.565851	24.5534	42.78221	7.034856
6396	MUC19	32.03333	2.464304	0.618964	3.981335	6.85E-05	0.000285	3.794605	10.94824	14.61036	46.87467	89.84265	26.12947
839	ADHFE1	197.2438	2.463868	0.208294	11.82877	2.77E-32	8.78E-31	69.38706	55.28859	57.04997	328.1227	320.8666	352.7478
8970	NRROS	11.54862	2.462735	0.788042	3.125131	0.001777	0.005267	3.794605	2.737059	4.174388	26.78552	10.69555	21.10457
14502	NMRK2	11.97476	2.462289	1.279182	1.924893	0.054243	0.099438	0	0	11.1317	29.01765	25.66933	6.029877
4674	CLCN1	38.45354	2.461983	0.502272	4.901692	9.50E-07	5.40E-06	8.673382	9.853413	16.69755	75.89232	83.42532	36.17926
18980	SERPINB12	2.121241	2.459808	2.070053	1.188283	0.234722	0.328772	0	0.547412	1.391463	2.232127	8.556443	0
10464	LOC11962	10.08096	2.459465	0.893273	2.753319	0.005899	0.014988	4.336691	3.284471	1.391463	15.62489	27.80844	8.039836
10492	LOC10323	9.960545	2.459448	0.895451	2.746602	0.006022	0.015258	3.252518	1.094824	4.87012	26.78552	10.69555	13.06473
3511	LOC11962	74.63717	2.458919	0.420564	5.846713	5.01E-09	3.80E-08	11.38381	20.80165	36.87376	142.8561	145.4595	90.44815
13582	TSBP1	8.280701	2.458836	1.175189	2.09229	0.036413	0.071273	0	2.737059	4.87012	13.39276	25.66933	3.014938
2364	LOC10322	83.5609	2.457498	0.33559	7.322923	2.43E-13	2.73E-12	21.14137	21.34906	34.78657	138.3919	171.1289	114.5677
14040	LOC11962	4.66631	2.457293	1.224487	2.006793	0.044772	0.084789	1.626259	0.547412	2.087194	11.16063	8.556443	4.019918
10841	FOXN1	9.537604	2.456742	0.922754	2.662401	0.007759	0.019026	1.084173	2.189647	5.565851	20.08914	19.252	9.044815
18838	LOC11962	2.990455	2.456726	2.025413	1.212951	0.225149	0.317739	0	0	2.782925	4.464254	10.69555	0
12928	LOC11962	14.7024	2.454887	1.107671	2.216261	0.026674	0.054851	0	0.547412	13.2189	35.71403	25.66933	13.06473
13764	LOC11962	5.320364	2.454852	1.194938	2.054376	0.039939	0.077142	0.542086	2.189647	2.087194	13.39276	10.69555	3.014938
10666	SIRPB2	11.78622	2.454658	0.909147	2.699958	0.006935	0.017285	1.084173	1.642235	8.348776	15.62489	29.94755	14.06971
20331	CUNH9orf	1.468962	2.4534	2.52447	0.971848	0.331126	0.432945	0	0	1.391463	0	6.417332	1.004979
20332	LOC11962	1.468962	2.4534	2.52447	0.971848	0.331126	0.432945	0	0	1.391463	0	6.417332	1.004979
8504	MTRNR2L4	13.08587	2.453061	0.753492	3.255592	0.001132	0.003537	3.252518	3.831883	4.87012	24.5534	29.94755	12.05975
1079	ZNF467	157.4989	2.451651	0.230913	10.61723	2.48E-26	6.11E-25	54.20864	42.15071	50.09266	274.5516	239.5804	284.4092
11678	CNTNAP4	19.56678	2.451599	0.990185	2.475899	0.01329	0.030253	0	1.642235	16.69755	46.87467	32.08666	20.09959
504	DRC1	310.4107	2.450057	0.168547	14.53636	7.13E-48	3.76E-46	95.4072	102.9134	89.74935	529.0141	502.691	542.6889
7589	GNAT1	19.5334	2.449964	0.694132	3.529534	0.000416	0.001458	3.794605	2.737059	11.82743	26.78552	44.92132	27.13445
13133	CLCA1	5.887331	2.449727	1.122437	2.182507	0.029072	0.05885	1.626259	1.642235	2.087194	6.696381	19.252	4.019918
15962	LOC10324	3.113812	2.449132	1.464281	1.672583	0.094409	0.157241	1.626259	0.547412	0.695731	2.232127	8.556443	5.024897
6849	SCNN1A	23.71498	2.447506	0.644364	3.79833	0.000146	0.000565	5.96295	4.926706	11.1317	24.5534	70.59065	25.12449
16768	IQCA1L	27.42099	2.447147	1.598899	1.530519	0.125888	0.199609	0	3.284471	22.2634	53.57105	66.31243	19.09461
6421	SSUH2	19.88438	2.445622	0.615635	3.97252	7.11E-05	0.000294	7.047123	4.926706	6.261582	46.87467	32.08666	22.10955
4375	LOC11962	39.39777	2.444486	0.47967	5.096179	3.47E-07	2.11E-06	9.757555	8.211177	18.78475	66.96381	83.42532	49.24399
8413	PLEKHD1	19.6305	2.444016	0.745484	3.278428	0.001044	0.003299	4.336691	2.737059	11.1317	46.87467	40.6431	12.05975
15316	LOC10322	5.16004	2.443842	1.375437	1.776775	0.075605	0.131225	0	3.284471	1.391463	17.85702	6.417332	2.009959
15317	CCR7	5.16004	2.443842	1.375437	1.776775	0.075605	0.131225	0	3.284471	1.391463	17.85702	6.417332	2.009959
6832	LOC11962	20.02527	2.443567	0.642202	3.804983	0.000142	0.000552	7.047123	7.663765	3.478657	37.94616	44.92132	19.09461
5823	APLNR	32.33298	2.442569	0.573194	4.261329	2.03E-05	9.28E-05	4.878777	7.663765	18.08902	33.4819	70.59065	59.29379
15408	LOC10321	4.073473	2.442552	1.388682	1.7589	0.078595	0.135607	1.084173	0.547412	2.087194	11.16063	8.556443	1.004979
230	CNTNAP2	10846.8	2.442467	0.128454	19.01436	1.30E-80	1.50E-78	2948.408	4117.632	3045.912	18189.6	17910.77	18868.49
4869	COL1A2	33.10871	2.442022	0.513061	4.759709	1.94E-06	1.06E-05	7.047123	10.40082	13.2189	55.80317	77.00798	35.17428
385	CEBPD	1328.73	2.441656	0.153261	15.93133	3.84E-57	2.65E-55	364.8241	517.8516	355.5187	2395.072	2124.137	2214.975
5974	KCNN4	60.5526	2.439283	0.584226	4.175239	2.98E-05	0.000132	9.215468	6.568942	41.04815	107.1421	139.0422	60.29877
15802	LOC11962	4.785983	2.43923	1.434083	1.700899	0.088962	0.149668	0.542086	0.547412	3.478657	0	17.11289	7.034856
13253	DPPA2	8.323812	2.439116	1.130664	2.157242	0.030987	0.062158	1.084173	4.379295	2.087194	17.85702	23.53022	1.004979
3685	RYR1	89.97711	2.439063	0.430963	5.65956	1.52E-08	1.09E-07	16.26259	18.612	49.39693	154.0168	201.0764	100.4979
12864	LOC10323	6.286176	2.438405	1.095573	2.225689	0.026035	0.053804	3.252518	1.094824	1.391463	6.696381	19.252	6.029877

11415	IRF7	7.753198	2.436908	0.962119	2.532854	0.011314	0.026349	2.710432	1.094824	3.478657	8.928508	19.252	11.05477
9240	NCAN	14.71111	2.436223	0.800094	3.044922	0.002327	0.006697	2.168345	3.831883	7.653045	33.4819	32.08666	9.044815
235	LRRC8E	1657.708	2.43559	0.129003	18.88004	1.66E-79	1.88E-77	583.2849	448.8777	521.1028	2685.249	2637.523	3070.212
11370	LOC11962(	8.607798	2.435572	0.957371	2.544021	0.010958	0.025623	2.168345	1.094824	4.87012	8.928508	23.53022	11.05477
2604	PTPN7	92.1675	2.434654	0.348954	6.976996	3.02E-12	3.08E-11	25.47806	17.51718	43.83108	165.1774	158.2942	142.7071
19409	LOC11962ε	14.79558	2.43439	2.179282	1.117061	0.263968	0.361654	0	0	13.91463	24.5534	34.22577	16.07967
14280	CRYBB3	5.577523	2.432914	1.238043	1.965129	0.049399	0.091938	0.542086	0.547412	4.174388	15.62489	8.556443	4.019918
6249	GNG2	29.40353	2.431456	0.600041	4.05215	5.07E-05	0.000216	5.420864	4.379295	18.08902	60.26743	47.06043	41.20416
17438	LOC11961¦	2.483946	2.431415	1.712089	1.420145	0.155565	0.237166	1.084173	0.547412	0.695731	0	8.556443	4.019918
15024	LOC11962(	4.4027	2.431363	1.32944	1.828863	0.06742	0.1193	0	2.737059	1.391463	6.696381	8.556443	7.034856
13054	LOC10322;	5.943056	2.429806	1.106698	2.195546	0.028124	0.057277	1.626259	1.094824	2.782925	11.16063	14.97377	4.019918
12671	TGM3	8.73952	2.429469	1.071978	2.266342	0.02343	0.049159	2.710432	0.547412	4.87012	15.62489	25.66933	3.014938
13330	ACTA2	6.78304	2.429289	1.133897	2.142426	0.032159	0.064138	0.542086	1.642235	4.174388	8.928508	21.39111	4.019918
18696	LOC11962⁄	2.118413	2.427531	1.974868	1.229211	0.218993	0.311399	0	0	2.087194	4.464254	2.139111	4.019918
6779	UNC5CL	18.56814	2.425152	0.634025	3.825012	0.000131	0.000513	6.505036	3.284471	7.653045	33.4819	36.36488	24.11951
20111	LOC10322!	1.24831	2.424794	2.398479	1.010972	0.31203	0.412466	0.542086	0	0.695731	2.232127	0	4.019918
11372	NKX2-1	13.06945	2.424484	0.95328	2.543307	0.010981	0.025671	0.542086	2.737059	9.044508	31.24978	27.80844	7.034856
8017	LOC10323:	15.00384	2.424425	0.711521	3.407382	0.000656	0.002175	2.710432	3.831883	7.653045	26.78552	29.94755	19.09461
19381	LOC11962(	2.133915	2.424106	2.16357	1.120419	0.262535	0.36012	0	0	2.087194	6.696381	0	4.019918
26587	TRNAN-GU	0.167497	2.421735	4.072558	0.594647	0.552079	NA	0	0	0	0	0	1.004979
26605	LOC11962(	0.167497	2.421735	4.072558	0.594647	0.552079	NA	0	0	0	0	0	1.004979
26614	LOC10322⁄	0.167497	2.421735	4.072558	0.594647	0.552079	NA	0	0	0	0	0	1.004979
26631	LOC11962(	0.167497	2.421735	4.072558	0.594647	0.552079	NA	0	0	0	0	0	1.004979
26634	LOC11962(	0.167497	2.421735	4.072558	0.594647	0.552079	NA	0	0	0	0	0	1.004979
26668	LOC11962ε	0.167497	2.421735	4.072558	0.594647	0.552079	NA	0	0	0	0	0	1.004979
26728	ACTL7A	0.167497	2.421735	4.072558	0.594647	0.552079	NA	0	0	0	0	0	1.004979
26747	LOC11961¦	0.167497	2.421735	4.072558	0.594647	0.552079	NA	0	0	0	0	0	1.004979
26752	LOC11962⁄	0.167497	2.421735	4.072558	0.594647	0.552079	NA	0	0	0	0	0	1.004979
26761	LOC11962:	0.167497	2.421735	4.072558	0.594647	0.552079	NA	0	0	0	0	0	1.004979
26777	LOC11962⁄	0.167497	2.421735	4.072558	0.594647	0.552079	NA	0	0	0	0	0	1.004979
26872	LOC11962(	0.167497	2.421735	4.072558	0.594647	0.552079	NA	0	0	0	0	0	1.004979
26875	LOC11962:	0.167497	2.421735	4.072558	0.594647	0.552079	NA	0	0	0	0	0	1.004979
26888	LOC10323ε	0.167497	2.421735	4.072558	0.594647	0.552079	NA	0	0	0	0	0	1.004979
26900	LOC10323ε	0.167497	2.421735	4.072558	0.594647	0.552079	NA	0	0	0	0	0	1.004979
26924	LOC10322:	0.167497	2.421735	4.072558	0.594647	0.552079	NA	0	0	0	0	0	1.004979
26937	SPRR2G	0.167497	2.421735	4.072558	0.594647	0.552079	NA	0	0	0	0	0	1.004979
26972	LOC11962⁄	0.167497	2.421735	4.072558	0.594647	0.552079	NA	0	0	0	0	0	1.004979
26998	LOC11962⁄	0.167497	2.421735	4.072558	0.594647	0.552079	NA	0	0	0	0	0	1.004979
27002	LOC11962⁄	0.167497	2.421735	4.072558	0.594647	0.552079	NA	0	0	0	0	0	1.004979
27011	LOC10321¦	0.167497	2.421735	4.072558	0.594647	0.552079	NA	0	0	0	0	0	1.004979
27034	LOC11962⁄	0.167497	2.421735	4.072558	0.594647	0.552079	NA	0	0	0	0	0	1.004979
27035	LOC10323⁄	0.167497	2.421735	4.072558	0.594647	0.552079	NA	0	0	0	0	0	1.004979
27041	LOC10323⁄	0.167497	2.421735	4.072558	0.594647	0.552079	NA	0	0	0	0	0	1.004979
27042	LOC11962⁄	0.167497	2.421735	4.072558	0.594647	0.552079	NA	0	0	0	0	0	1.004979
27072	LOC10323!	0.167497	2.421735	4.072558	0.594647	0.552079	NA	0	0	0	0	0	1.004979
27081	LOC10323!	0.167497	2.421735	4.072558	0.594647	0.552079	NA	0	0	0	0	0	1.004979
27125	LOC11962⁄	0.167497	2.421735	4.072558	0.594647	0.552079	NA	0	0	0	0	0	1.004979
27195	TRNAE-CU	0.167497	2.421735	4.072558	0.594647	0.552079	NA	0	0	0	0	0	1.004979
27215	LOC11962!	0.167497	2.421735	4.072558	0.594647	0.552079	NA	0	0	0	0	0	1.004979
27224	LOC10324(	0.167497	2.421735	4.072558	0.594647	0.552079	NA	0	0	0	0	0	1.004979
27255	LOC11962!	0.167497	2.421735	4.072558	0.594647	0.552079	NA	0	0	0	0	0	1.004979
27258	LOC10321;	0.167497	2.421735	4.072558	0.594647	0.552079	NA	0	0	0	0	0	1.004979
27265	LOC11962!	0.167497	2.421735	4.072558	0.594647	0.552079	NA	0	0	0	0	0	1.004979
27288	LOC11962!	0.167497	2.421735	4.072558	0.594647	0.552079	NA	0	0	0	0	0	1.004979
27304	LOC11962!	0.167497	2.421735	4.072558	0.594647	0.552079	NA	0	0	0	0	0	1.004979
27364	LOC11962!	0.167497	2.421735	4.072558	0.594647	0.552079	NA	0	0	0	0	0	1.004979
27386	LOC11962(	0.167497	2.421735	4.072558	0.594647	0.552079	NA	0	0	0	0	0	1.004979
27396	LOC11962(	0.167497	2.421735	4.072558	0.594647	0.552079	NA	0	0	0	0	0	1.004979
27435	LOC11962!	0.167497	2.421735	4.072558	0.594647	0.552079	NA	0	0	0	0	0	1.004979
27454	RTP3	0.167497	2.421735	4.072558	0.594647	0.552079	NA	0	0	0	0	0	1.004979
27461	LOC11962!	0.167497	2.421735	4.072558	0.594647	0.552079	NA	0	0	0	0	0	1.004979
27463	LOC11962(	0.167497	2.421735	4.072558	0.594647	0.552079	NA	0	0	0	0	0	1.004979
27477	LOC10322(	0.167497	2.421735	4.072558	0.594647	0.552079	NA	0	0	0	0	0	1.004979
27508	LOC11962(	0.167497	2.421735	4.072558	0.594647	0.552079	NA	0	0	0	0	0	1.004979
27567	LOC11962(	0.167497	2.421735	4.072558	0.594647	0.552079	NA	0	0	0	0	0	1.004979
27568	LOC11962(	0.167497	2.421735	4.072558	0.594647	0.552079	NA	0	0	0	0	0	1.004979
27571	LOC11962(	0.167497	2.421735	4.072558	0.594647	0.552079	NA	0	0	0	0	0	1.004979
27606	LOC10324;	0.167497	2.421735	4.072558	0.594647	0.552079	NA	0	0	0	0	0	1.004979
27607	LOC10324;	0.167497	2.421735	4.072558	0.594647	0.552079	NA	0	0	0	0	0	1.004979
27629	LOC11962(	0.167497	2.421735	4.072558	0.594647	0.552079	NA	0	0	0	0	0	1.004979
27634	LOC11962(	0.167497	2.421735	4.072558	0.594647	0.552079	NA	0	0	0	0	0	1.004979
27659	TRNAT-CG	0.167497	2.421735	4.072558	0.594647	0.552079	NA	0	0	0	0	0	1.004979
27682	LOC10322:	0.167497	2.421735	4.072558	0.594647	0.552079	NA	0	0	0	0	0	1.004979
27708	LOC11962;	0.167497	2.421735	4.072558	0.594647	0.552079	NA	0	0	0	0	0	1.004979

27714	LOC119627	0.167497	2.421735	4.072558	0.594647	0.552079	NA	0	0	0	0	0	1.004979
27724	LOC119627	0.167497	2.421735	4.072558	0.594647	0.552079	NA	0	0	0	0	0	1.004979
27725	LOC119627	0.167497	2.421735	4.072558	0.594647	0.552079	NA	0	0	0	0	0	1.004979
27734	LOC119627	0.167497	2.421735	4.072558	0.594647	0.552079	NA	0	0	0	0	0	1.004979
27735	LOC103220	0.167497	2.421735	4.072558	0.594647	0.552079	NA	0	0	0	0	0	1.004979
27758	LOC103221	0.167497	2.421735	4.072558	0.594647	0.552079	NA	0	0	0	0	0	1.004979
27761	LOC119627	0.167497	2.421735	4.072558	0.594647	0.552079	NA	0	0	0	0	0	1.004979
27775	LOC103219	0.167497	2.421735	4.072558	0.594647	0.552079	NA	0	0	0	0	0	1.004979
27790	LOC119627	0.167497	2.421735	4.072558	0.594647	0.552079	NA	0	0	0	0	0	1.004979
27805	LOC119627	0.167497	2.421735	4.072558	0.594647	0.552079	NA	0	0	0	0	0	1.004979
27807	LOC119627	0.167497	2.421735	4.072558	0.594647	0.552079	NA	0	0	0	0	0	1.004979
27882	LOC119627	0.167497	2.421735	4.072558	0.594647	0.552079	NA	0	0	0	0	0	1.004979
27902	LOC119627	0.167497	2.421735	4.072558	0.594647	0.552079	NA	0	0	0	0	0	1.004979
27931	LOC119628	0.167497	2.421735	4.072558	0.594647	0.552079	NA	0	0	0	0	0	1.004979
27939	LOC119628	0.167497	2.421735	4.072558	0.594647	0.552079	NA	0	0	0	0	0	1.004979
27942	LOC119628	0.167497	2.421735	4.072558	0.594647	0.552079	NA	0	0	0	0	0	1.004979
27990	ADAMTS7	0.167497	2.421735	4.072558	0.594647	0.552079	NA	0	0	0	0	0	1.004979
27994	LOC103222	0.167497	2.421735	4.072558	0.594647	0.552079	NA	0	0	0	0	0	1.004979
28013	LOC119618	0.167497	2.421735	4.072558	0.594647	0.552079	NA	0	0	0	0	0	1.004979
28020	LOC119618	0.167497	2.421735	4.072558	0.594647	0.552079	NA	0	0	0	0	0	1.004979
28023	LOC119618	0.167497	2.421735	4.072558	0.594647	0.552079	NA	0	0	0	0	0	1.004979
28027	LOC103222	0.167497	2.421735	4.072558	0.594647	0.552079	NA	0	0	0	0	0	1.004979
28034	LOC119618	0.167497	2.421735	4.072558	0.594647	0.552079	NA	0	0	0	0	0	1.004979
28054	LOC119618	0.167497	2.421735	4.072558	0.594647	0.552079	NA	0	0	0	0	0	1.004979
28063	LOC119618	0.167497	2.421735	4.072558	0.594647	0.552079	NA	0	0	0	0	0	1.004979
28082	LOC119618	0.167497	2.421735	4.072558	0.594647	0.552079	NA	0	0	0	0	0	1.004979
28090	LOC119618	0.167497	2.421735	4.072558	0.594647	0.552079	NA	0	0	0	0	0	1.004979
28092	LOC103246	0.167497	2.421735	4.072558	0.594647	0.552079	NA	0	0	0	0	0	1.004979
28117	LOC119618	0.167497	2.421735	4.072558	0.594647	0.552079	NA	0	0	0	0	0	1.004979
28181	DEFB108B	0.167497	2.421735	4.072558	0.594647	0.552079	NA	0	0	0	0	0	1.004979
28197	LOC119619	0.167497	2.421735	4.072558	0.594647	0.552079	NA	0	0	0	0	0	1.004979
28209	LOC119619	0.167497	2.421735	4.072558	0.594647	0.552079	NA	0	0	0	0	0	1.004979
28210	LOC119619	0.167497	2.421735	4.072558	0.594647	0.552079	NA	0	0	0	0	0	1.004979
28277	LOC119619	0.167497	2.421735	4.072558	0.594647	0.552079	NA	0	0	0	0	0	1.004979
28289	LOC103229	0.167497	2.421735	4.072558	0.594647	0.552079	NA	0	0	0	0	0	1.004979
28318	LOC119619	0.167497	2.421735	4.072558	0.594647	0.552079	NA	0	0	0	0	0	1.004979
28352	LOC119619	0.167497	2.421735	4.072558	0.594647	0.552079	NA	0	0	0	0	0	1.004979
28362	LOC119619	0.167497	2.421735	4.072558	0.594647	0.552079	NA	0	0	0	0	0	1.004979
28377	LOC119619	0.167497	2.421735	4.072558	0.594647	0.552079	NA	0	0	0	0	0	1.004979
28382	LOC119619	0.167497	2.421735	4.072558	0.594647	0.552079	NA	0	0	0	0	0	1.004979
28434	LOC103230	0.167497	2.421735	4.072558	0.594647	0.552079	NA	0	0	0	0	0	1.004979
28439	LOC119619	0.167497	2.421735	4.072558	0.594647	0.552079	NA	0	0	0	0	0	1.004979
28448	LOC119620	0.167497	2.421735	4.072558	0.594647	0.552079	NA	0	0	0	0	0	1.004979
28499	LOC119620	0.167497	2.421735	4.072558	0.594647	0.552079	NA	0	0	0	0	0	1.004979
28516	LOC119620	0.167497	2.421735	4.072558	0.594647	0.552079	NA	0	0	0	0	0	1.004979
28519	LOC119620	0.167497	2.421735	4.072558	0.594647	0.552079	NA	0	0	0	0	0	1.004979
28524	LOC119620	0.167497	2.421735	4.072558	0.594647	0.552079	NA	0	0	0	0	0	1.004979
28543	LOC119620	0.167497	2.421735	4.072558	0.594647	0.552079	NA	0	0	0	0	0	1.004979
28564	LOC119620	0.167497	2.421735	4.072558	0.594647	0.552079	NA	0	0	0	0	0	1.004979
28580	LOC103231	0.167497	2.421735	4.072558	0.594647	0.552079	NA	0	0	0	0	0	1.004979
28607	LOC119620	0.167497	2.421735	4.072558	0.594647	0.552079	NA	0	0	0	0	0	1.004979
28609	LOC119620	0.167497	2.421735	4.072558	0.594647	0.552079	NA	0	0	0	0	0	1.004979
28624	LOC103216	0.167497	2.421735	4.072558	0.594647	0.552079	NA	0	0	0	0	0	1.004979
28654	LOC119620	0.167497	2.421735	4.072558	0.594647	0.552079	NA	0	0	0	0	0	1.004979
28707	LOC119621	0.167497	2.421735	4.072558	0.594647	0.552079	NA	0	0	0	0	0	1.004979
28709	LOC119621	0.167497	2.421735	4.072558	0.594647	0.552079	NA	0	0	0	0	0	1.004979
28715	LOC119620	0.167497	2.421735	4.072558	0.594647	0.552079	NA	0	0	0	0	0	1.004979
28722	LOC103232	0.167497	2.421735	4.072558	0.594647	0.552079	NA	0	0	0	0	0	1.004979
28765	LOC103235	0.167497	2.421735	4.072558	0.594647	0.552079	NA	0	0	0	0	0	1.004979
28818	LOC103225	0.167497	2.421735	4.072558	0.594647	0.552079	NA	0	0	0	0	0	1.004979
28824	LOC119621	0.167497	2.421735	4.072558	0.594647	0.552079	NA	0	0	0	0	0	1.004979
28844	LOC119621	0.167497	2.421735	4.072558	0.594647	0.552079	NA	0	0	0	0	0	1.004979
28845	LOC119621	0.167497	2.421735	4.072558	0.594647	0.552079	NA	0	0	0	0	0	1.004979
28869	LOC119621	0.167497	2.421735	4.072558	0.594647	0.552079	NA	0	0	0	0	0	1.004979
28908	LOC119621	0.167497	2.421735	4.072558	0.594647	0.552079	NA	0	0	0	0	0	1.004979
28913	LOC103227	0.167497	2.421735	4.072558	0.594647	0.552079	NA	0	0	0	0	0	1.004979
28916	LOC103227	0.167497	2.421735	4.072558	0.594647	0.552079	NA	0	0	0	0	0	1.004979
28929	TRNAC-ACA	0.167497	2.421735	4.072558	0.594647	0.552079	NA	0	0	0	0	0	1.004979
28951	LOC103235	0.167497	2.421735	4.072558	0.594647	0.552079	NA	0	0	0	0	0	1.004979
28959	LOC119621	0.167497	2.421735	4.072558	0.594647	0.552079	NA	0	0	0	0	0	1.004979
28965	LOC119621	0.167497	2.421735	4.072558	0.594647	0.552079	NA	0	0	0	0	0	1.004979
28968	LOC119621	0.167497	2.421735	4.072558	0.594647	0.552079	NA	0	0	0	0	0	1.004979
28985	LOC119621	0.167497	2.421735	4.072558	0.594647	0.552079	NA	0	0	0	0	0	1.004979
29007	LOC119622	0.167497	2.421735	4.072558	0.594647	0.552079	NA	0	0	0	0	0	1.004979

29032	LOC10324:	0.167497	2.421735	4.072558	0.594647	0.552079	NA	0	0	0	0	0	1.004979
29045	LOC11962:	0.167497	2.421735	4.072558	0.594647	0.552079	NA	0	0	0	0	0	1.004979
29051	LOC10324:	0.167497	2.421735	4.072558	0.594647	0.552079	NA	0	0	0	0	0	1.004979
29052	LOC10324:	0.167497	2.421735	4.072558	0.594647	0.552079	NA	0	0	0	0	0	1.004979
29069	CUNH17or	0.167497	2.421735	4.072558	0.594647	0.552079	NA	0	0	0	0	0	1.004979
29091	LOC11962:	0.167497	2.421735	4.072558	0.594647	0.552079	NA	0	0	0	0	0	1.004979
29093	LOC11962:	0.167497	2.421735	4.072558	0.594647	0.552079	NA	0	0	0	0	0	1.004979
29108	LOC10324:	0.167497	2.421735	4.072558	0.594647	0.552079	NA	0	0	0	0	0	1.004979
29111	LOC10324:	0.167497	2.421735	4.072558	0.594647	0.552079	NA	0	0	0	0	0	1.004979
29117	LOC10324:	0.167497	2.421735	4.072558	0.594647	0.552079	NA	0	0	0	0	0	1.004979
29120	LOC10324:	0.167497	2.421735	4.072558	0.594647	0.552079	NA	0	0	0	0	0	1.004979
29131	LOC10324:	0.167497	2.421735	4.072558	0.594647	0.552079	NA	0	0	0	0	0	1.004979
29149	LOC11962:	0.167497	2.421735	4.072558	0.594647	0.552079	NA	0	0	0	0	0	1.004979
29152	LOC11962:	0.167497	2.421735	4.072558	0.594647	0.552079	NA	0	0	0	0	0	1.004979
29155	SMR3B	0.167497	2.421735	4.072558	0.594647	0.552079	NA	0	0	0	0	0	1.004979
29159	LOC10323!	0.167497	2.421735	4.072558	0.594647	0.552079	NA	0	0	0	0	0	1.004979
29177	LOC11961{	0.167497	2.421735	4.072558	0.594647	0.552079	NA	0	0	0	0	0	1.004979
29180	LOC11962:	0.167497	2.421735	4.072558	0.594647	0.552079	NA	0	0	0	0	0	1.004979
29192	LOC11962:	0.167497	2.421735	4.072558	0.594647	0.552079	NA	0	0	0	0	0	1.004979
29211	LOC10324;	0.167497	2.421735	4.072558	0.594647	0.552079	NA	0	0	0	0	0	1.004979
29223	IL31	0.167497	2.421735	4.072558	0.594647	0.552079	NA	0	0	0	0	0	1.004979
29233	LOC11962:	0.167497	2.421735	4.072558	0.594647	0.552079	NA	0	0	0	0	0	1.004979
29238	LOC11962:	0.167497	2.421735	4.072558	0.594647	0.552079	NA	0	0	0	0	0	1.004979
29332	LOC10322:	0.167497	2.421735	4.072558	0.594647	0.552079	NA	0	0	0	0	0	1.004979
29339	LOC11962:	0.167497	2.421735	4.072558	0.594647	0.552079	NA	0	0	0	0	0	1.004979
29437	LOC10324;	0.167497	2.421735	4.072558	0.594647	0.552079	NA	0	0	0	0	0	1.004979
29449	LOC11961{	0.167497	2.421735	4.072558	0.594647	0.552079	NA	0	0	0	0	0	1.004979
29494	LOC10323(	0.167497	2.421735	4.072558	0.594647	0.552079	NA	0	0	0	0	0	1.004979
29504	LOC11962:	0.167497	2.421735	4.072558	0.594647	0.552079	NA	0	0	0	0	0	1.004979
29541	LOC11962:	0.167497	2.421735	4.072558	0.594647	0.552079	NA	0	0	0	0	0	1.004979
14707	LOC10324;	5.349331	2.421566	1.281631	1.889442	0.058833	0.106348	1.626259	0.547412	2.782925	11.16063	14.97377	1.004979
7079	IL9R	21.71102	2.420986	0.653793	3.702988	0.000213	0.0008	8.673382	3.831883	7.653045	49.10679	44.92132	16.07967
4823	LOC10322!	28.78702	2.420941	0.504667	4.797102	1.61E-06	8.87E-06	11.38381	6.02153	9.740239	46.87467	53.47777	45.22408
15977	LOC10324!	5.795347	2.420911	1.450445	1.669082	0.095101	0.158244	0	0	5.565851	15.62489	8.556443	5.024897
4463	C1QL2	30.52056	2.4203	0.480811	5.033788	4.81E-07	2.86E-06	8.131296	10.94824	9.740239	60.26743	42.78221	51.25395
20122	LOC10322:	1.249198	2.420244	2.397982	1.009283	0.312839	0.41332	0	0.547412	0.695731	2.232127	0	4.019918
612	LOC10322!	426.4295	2.419257	0.179468	13.48013	2.05E-41	8.89E-40	148.5317	103.4608	151.6694	752.2268	678.0981	724.5902
13648	KLHL10	8.076177	2.419169	1.163123	2.079891	0.037536	0.073116	0	2.737059	4.87012	11.16063	25.66933	4.019918
12766	CNGB3	6.545111	2.41876	1.076175	2.247553	0.024605	0.051239	2.168345	0.547412	3.478657	8.928508	17.11289	7.034856
10844	LRRIQ4	8.882816	2.418108	0.908436	2.661837	0.007772	0.019053	1.626259	3.831883	2.782925	15.62489	21.39111	8.039836
373	SHOX2	1569.463	2.41809	0.15007	16.11305	2.07E-58	1.47E-56	428.7903	622.9546	431.3534	2611.589	2725.227	2596.867
9466	ADRA2A	13.58678	2.416974	0.807255	2.994066	0.002753	0.007731	2.710432	4.379295	5.565851	22.32127	38.50399	8.039836
17546	LOC10323{	2.775826	2.415196	1.714089	1.409026	0.158828	0.240649	0.542086	0	2.087194	4.464254	8.556443	1.004979
1371	CDH18	130.9588	2.414532	0.250293	9.646822	5.07E-22	9.83E-21	48.24569	41.05589	34.09084	225.4448	228.8848	208.0308
5723	CD34	23.43676	2.414282	0.559925	4.311798	1.62E-05	7.52E-05	4.878777	8.211177	9.044508	42.41041	44.92132	31.15436
15441	PRMT8	4.879624	2.412506	1.375466	1.753956	0.079438	0.13677	0	0.547412	4.174388	6.696381	12.83466	5.024897
15433	GPR101	4.699715	2.411801	1.374262	1.754979	0.079263	0.136539	0	1.642235	2.782925	8.928508	12.83466	2.009959
17583	SOX21	3.45717	2.411589	1.71891	1.402976	0.160624	0.242859	0	0.547412	2.782925	13.39276	0	4.019918
7926	SYNPO2L	16.06241	2.41135	0.702765	3.431234	0.000601	0.002015	5.420864	2.189647	7.653045	26.78552	34.22577	20.09959
5532	LOC11961{	27.10773	2.410907	0.547962	4.399774	1.08E-05	5.21E-05	4.336691	10.40082	11.1317	37.94616	55.61688	43.21412
10801	LOC10323(	10.67326	2.410807	0.902765	2.670471	0.007574	0.018643	2.168345	1.094824	6.957314	26.78552	14.97377	12.05975
16846	FAM20A	4.310882	2.410423	1.59021	1.515789	0.129573	0.204481	0	0	4.174388	13.39276	4.278221	4.019918
6487	DOK2	17.88564	2.410004	0.610829	3.945462	7.96E-05	0.000326	5.420864	6.02153	5.565851	33.4819	25.66933	31.15436
18755	LOC11962:	2.139938	2.409488	1.968648	1.22393	0.220978	0.313234	0	0	2.087194	4.464254	4.278221	2.009959
16831	TBATA	4.705738	2.4088	1.585555	1.519215	0.128708	0.203298	0	1.642235	2.782925	6.696381	17.11289	0
13855	LOC10322¿	5.541443	2.406767	1.180662	2.038489	0.041501	0.079632	1.084173	2.737059	1.391463	17.85702	2.139111	8.039836
17034	LOC10323;	3.477807	2.406551	1.617206	1.488092	0.136727	0.213389	0.542086	0	2.782925	13.39276	2.139111	2.009959
13936	LOC10323!	8.395347	2.40624	1.188079	2.02532	0.042835	0.081713	0	1.094824	6.957314	22.32127	14.97377	5.024897
17255	LOC10321{	3.261758	2.405476	1.6508	1.457158	0.145073	0.22351	0	1.642235	1.391463	13.39276	2.139111	1.004979
860	HAS3	230.5913	2.405278	0.205011	11.73242	8.69E-32	2.69E-30	68.30288	67.33165	84.87923	426.3363	333.7013	402.9968
5440	LOC11962(	32.79987	2.404982	0.541316	4.442845	8.88E-06	4.34E-05	10.29964	4.379295	16.69755	58.0353	64.17332	43.21412
14973	LOC10323{	5.528187	2.404653	1.307649	1.838913	0.065928	0.117051	0.542086	0	4.87012	11.16063	8.556443	8.039836
146	CA12	12717.39	2.404646	0.107647	22.33835	1.57E-110	2.85E-108	3974.035	4472.355	3675.549	21165.03	19711.9	23305.47
14074	SULT2B1	6.170985	2.401842	1.201615	1.998845	0.045625	0.086183	0.542086	0.547412	4.87012	8.928508	17.11289	5.024897
4242	UPK3B	40.09952	2.401366	0.46244	5.192815	2.07E-07	1.30E-06	15.17842	6.568942	16.69755	73.66019	64.17332	64.31869
19608	GAL3ST3	2.176966	2.401	2.213451	1.084732	0.278041	0.376974	0	0	2.087194	6.696381	4.278221	0
7612	BRINP3	23.57915	2.40093	0.681779	3.521564	0.000429	0.001498	3.252518	6.02153	13.2189	35.71403	64.17332	19.09461
14297	LOC11962!	5.466757	2.399594	1.223026	1.962014	0.049761	0.092529	1.084173	3.284471	0.695731	4.464254	19.252	4.019918
15943	MYOC	3.753617	2.399088	1.430208	1.67744	0.093456	0.155831	0	1.642235	2.087194	4.464254	4.278221	10.04979
13811	LOC11962!	10.57593	2.398779	1.172526	2.045821	0.040774	0.078486	0	0.547412	9.740239	11.16063	29.94755	12.05975
14332	LOC11962!	6.889356	2.398464	1.226468	1.955586	0.050514	0.0937	0	1.094824	5.565851	20.08914	8.556443	6.029877
11633	LOC10324:	11.47098	2.397798	0.964227	2.486757	0.012891	0.029461	2.168345	0.547412	8.348776	15.62489	32.08666	10.04979
8008	LOC11962;	14.65183	2.397691	0.703139	3.40998	0.00065	0.002157	3.794605	3.284471	6.957314	31.24978	23.53022	19.09461
18461	URAD	1.923002	2.397268	1.911063	1.254416	0.209691	0.301961	0	1.094824	0.695731	4.464254	4.278221	1.004979

16392	LOC119625	4.068962	2.394787	1.506901	1.589214	0.112012	0.18165	0	1.094824	2.782925	6.696381	12.83466	1.004979
9619	PRTFDC1	18.51054	2.394222	0.810045	2.955664	0.00312	0.008623	0.542086	5.474118	11.82743	33.4819	40.6431	19.09461
14800	LOC10323	5.302658	2.393446	1.277868	1.872999	0.061068	0.109691	0	1.642235	3.478657	6.696381	14.97377	5.024897
3228	ESYT3	68.48711	2.392119	0.389994	6.133728	8.58E-10	7.07E-09	16.26259	20.80165	28.52499	98.21359	164.7115	82.40832
17379	LOC11962	2.556949	2.391383	1.668982	1.432839	0.151904	0.23237	0	1.094824	1.391463	6.696381	2.139111	4.019918
9709	PAPOLB	15.11374	2.38961	0.816463	2.926782	0.003425	0.009378	5.96295	2.737059	5.565851	22.32127	47.06043	7.034856
13938	CYP4F11	6.040779	2.388698	1.179483	2.025207	0.042846	0.081724	0.542086	1.094824	4.174388	17.85702	8.556443	4.019918
18671	TMEM132I	1.907499	2.388663	1.939266	1.231736	0.218048	0.310454	0	1.094824	0.695731	2.232127	6.417332	1.004979
18672	HSD3B2	1.907499	2.388663	1.939266	1.231736	0.218048	0.310454	0	1.094824	0.695731	2.232127	6.417332	1.004979
13504	LOC10322	5.786815	2.388114	1.133767	2.106354	0.035174	0.069244	0.542086	2.189647	2.782925	15.62489	8.556443	5.024897
12585	LOC10323	7.057538	2.387834	1.044215	2.286726	0.022212	0.046921	0.542086	2.737059	3.478657	17.85702	10.69555	7.034856
19654	LOC11961	1.270723	2.386867	2.215238	1.077476	0.281267	0.380437	0	0.547412	0.695731	2.232127	2.139111	2.009959
19655	IL36A	1.270723	2.386867	2.215238	1.077476	0.281267	0.380437	0	0.547412	0.695731	2.232127	2.139111	2.009959
3213	CNTFR	49.16225	2.386829	0.388151	6.149231	7.79E-10	6.44E-09	16.26259	16.42235	14.61036	64.73168	100.5382	82.40832
20833	GPR65	1.323254	2.386721	2.652907	0.899662	0.3683	0.469988	0	0.547412	0.695731	6.696381	0	0
10608	FCMR	11.43845	2.386568	0.877552	2.719573	0.006537	0.016382	0.542086	4.926706	5.565851	17.85702	25.66933	14.06971
7961	LOC10324	24.55699	2.386335	0.697368	3.421915	0.000622	0.002077	3.252518	3.831883	16.69755	49.10679	51.33866	23.11453
3838	ODF3B	45.2875	2.384265	0.432943	5.507108	3.65E-08	2.53E-07	11.38381	10.40082	22.2634	82.5887	72.72976	72.35852
5628	PAH	25.5177	2.38353	0.546676	4.360042	1.30E-05	6.14E-05	8.673382	10.40082	5.565851	29.01765	49.19955	50.24897
151	DEPP1	1949.245	2.38274	0.107927	22.07726	5.23E-108	9.20E-106	601.7159	666.7476	614.3308	3337.03	3005.45	3470.194
648	N4BP2L1	295.0727	2.381753	0.181035	13.15635	1.56E-39	6.42E-38	85.10756	110.5772	89.05362	482.1394	509.1083	494.4499
13357	CFAP99	5.698675	2.381548	1.115182	2.13557	0.032714	0.065115	1.626259	0.547412	3.478657	8.928508	8.556443	11.05477
16907	LOC11962	2.525056	2.381533	1.582549	1.504871	0.132357	0.208122	0.542086	0.547412	1.391463	2.232127	6.417332	4.019918
13574	KCNQ5	5.806565	2.381383	1.137643	2.09326	0.036326	0.071145	1.626259	1.094824	2.782925	15.62489	10.69555	3.014938
17428	LOC11961	2.524169	2.381055	1.672476	1.423671	0.154542	0.235735	1.084173	0	1.391463	2.232127	6.417332	4.019918
3203	IGFN1	139.0852	2.380289	0.386414	6.15995	7.28E-10	6.04E-09	24.93597	46.53	62.61582	238.8376	337.9795	123.6125
8517	MYL10	15.16008	2.379977	0.73171	3.252622	0.001143	0.003569	4.336691	3.831883	6.261582	31.24978	34.22577	11.05477
9698	FGF22	11.48974	2.379237	0.812072	2.929837	0.003391	0.009297	3.252518	1.642235	6.261582	22.32127	21.39111	14.06971
2597	LOC11962	86.37902	2.379101	0.340729	6.982394	2.90E-12	2.97E-11	21.14137	24.08612	38.26523	156.2489	168.9897	109.5428
14796	CLEC3B	4.159373	2.377069	1.268415	1.874046	0.060924	0.109466	1.084173	1.642235	1.391463	2.232127	8.556443	10.04979
9549	PLP1	12.50073	2.376699	0.799587	2.972408	0.002955	0.008225	2.710432	4.379295	4.87012	15.62489	36.36488	11.05477
404	RASSF5	549.6231	2.37603	0.151951	15.63678	4.09E-55	2.69E-53	158.8313	182.8355	192.7176	930.797	823.5576	1008.999
7467	BCAN	27.11399	2.374762	0.664461	3.573968	0.000352	0.001252	3.794605	7.116354	15.30609	44.64254	72.72976	19.09461
17227	SCTR	3.401181	2.373804	1.622249	1.46328	0.143391	0.221283	0	0.547412	2.782925	2.232127	12.83466	2.009959
20220	LOC11961	1.254333	2.373118	2.389983	0.992944	0.320737	0.421681	0.542086	0	0.695731	0	4.278221	2.009959
20221	LOC10323	1.254333	2.373118	2.389983	0.992944	0.320737	0.421681	0.542086	0	0.695731	0	4.278221	2.009959
7595	LOC11961	20.95576	2.372937	0.672719	3.527384	0.00042	0.001469	7.047123	2.737059	10.43597	44.64254	42.78221	18.08963
15585	LOC11962	7.783291	2.372895	1.370311	1.731648	0.083336	0.142156	0	0	7.653045	22.32127	10.69555	6.029877
312	FMO4	540.2308	2.372192	0.138134	17.1731	4.22E-66	3.60E-64	183.2252	178.4563	162.8011	924.1006	864.2007	928.601
1380	TAMALIN	157.9817	2.371937	0.246655	9.616429	6.82E-22	1.31E-20	62.33993	44.88777	46.614	270.0874	222.4675	301.4938
16158	LOC11961	3.781164	2.371158	1.450139	1.635124	0.102023	0.16785	0	1.642235	2.087194	2.232127	10.69555	6.029877
7676	LOC10322	25.49634	2.370763	0.677508	3.49924	0.000467	0.001616	4.878777	3.284471	16.69755	55.80317	49.19955	23.11453
13517	CCER1	12.50532	2.370754	1.126384	2.104747	0.035313	0.069454	0	0.547412	11.82743	15.62489	29.94755	17.08465
17725	ANKRD60	2.323623	2.370675	1.717946	1.379948	0.167603	0.25138	0.542086	1.642235	0	4.464254	4.278221	3.014938
7346	LOC10322	35.43378	2.370191	0.655273	3.617107	0.000298	0.001078	6.505036	4.379295	23.65487	55.80317	94.12087	28.13943
18556	LOC10323	2.309007	2.369887	1.907531	1.242385	0.214095	0.306731	0	2.189647	0	2.232127	6.417332	3.014938
14817	LOC10324	4.518398	2.368903	1.267443	1.869041	0.061617	0.110547	1.084173	0.547412	2.782925	13.39276	4.278221	5.024897
12002	FOXE3	21.60496	2.368683	0.984776	2.405303	0.016159	0.035792	0.542086	0.547412	20.17621	46.87467	36.36488	25.12449
20226	LOC11962	1.255221	2.368595	2.389492	0.991255	0.321561	0.422638	0	0.547412	0.695731	0	4.278221	2.009959
20227	LOC11961	1.255221	2.368595	2.389492	0.991255	0.321561	0.422638	0	0.547412	0.695731	0	4.278221	2.009959
12710	CEBPE	9.139547	2.367779	1.049309	2.256513	0.024039	0.050286	0	2.737059	6.261582	13.39276	21.39111	11.05477
10128	LOC11962	14.56724	2.367391	0.836159	2.831269	0.004636	0.012169	2.168345	4.379295	7.653045	8.928508	49.19955	15.07469
1613	FLVCR2	119.7081	2.364732	0.264339	8.945829	3.69E-19	6.08E-18	35.7777	35.58177	45.22254	232.1412	194.6591	174.8664
17484	AZU1	2.562972	2.362654	1.670486	1.414351	0.157259	0.239117	0	1.094824	1.391463	4.464254	6.417332	2.009959
5359	CLIC5	40.48181	2.362642	0.526808	4.484823	7.30E-06	3.62E-05	10.84173	7.663765	20.87194	87.05295	81.28621	35.17428
7214	PRLHR	24.43234	2.361576	0.644821	3.662377	0.00025	0.000921	3.252518	4.926706	16.00182	40.17829	47.06043	35.17428
5720	LOC11962	41.55104	2.360933	0.54737	4.313233	1.61E-05	7.48E-05	8.131296	8.211177	24.3506	64.73168	102.6773	41.20416
27126	LOC11962	0.618445	2.360592	3.758545	0.62806	0.529965	NA	0	0	0.695731	0	0	3.014938
27251	LOC10321	0.618445	2.360592	3.758545	0.62806	0.529965	NA	0	0	0.695731	0	0	3.014938
29457	LOC11962	0.618445	2.360592	3.758545	0.62806	0.529965	NA	0	0	0.695731	0	0	3.014938
392	MYBPH	627.371	2.360282	0.149201	15.81952	2.28E-56	1.55E-54	217.9187	201.995	194.8048	1066.957	902.7047	1179.846
13290	B3GNT8	7.305217	2.359587	1.097346	2.150267	0.031534	0.06308	0.542086	1.094824	5.565851	15.62489	14.97377	6.029877
9733	LOC11962	10.20535	2.357959	0.807346	2.920629	0.003493	0.009542	3.252518	3.831883	2.782925	22.32127	14.97377	14.06971
14994	LOC11962	5.348015	2.357359	1.284404	1.835372	0.066451	0.11782	0.542086	0.547412	4.174388	6.696381	17.11289	3.014938
19157	LOC10322	1.941699	2.3572	2.033735	1.15905	0.246436	0.341984	0	0.547412	1.391463	6.696381	0	3.014938
1142	TMEM154	170.3573	2.357051	0.227799	10.34706	4.32E-25	1.00E-23	51.49821	65.68942	50.09266	265.6231	271.6671	317.5735
8105	GCM1	14.03957	2.356793	0.697443	3.37919	0.000727	0.002385	4.878777	3.284471	5.565851	29.01765	21.39111	20.09959
14698	AANAT	4.47189	2.356179	1.246169	1.890739	0.058659	0.1061	1.084173	0.547412	2.782925	6.696381	10.69555	5.024897
17733	MRGPRX2	2.547469	2.355824	1.708994	1.378485	0.168053	0.251914	0	1.094824	1.391463	2.232127	8.556443	2.009959
17734	LOC11962	2.547469	2.355824	1.708994	1.378485	0.168053	0.251914	0	1.094824	1.391463	2.232127	8.556443	2.009959
11940	LOC11962	7.519019	2.355515	0.974725	2.416594	0.015666	0.034882	1.626259	2.189647	3.478657	20.08914	10.69555	7.034856
2427	RDH12	80.45601	2.355208	0.325651	7.232305	4.75E-13	5.20E-12	24.39389	22.44388	32.69937	151.7846	102.6773	148.737
1812	LOC11962	110.037	2.354942	0.279617	8.422031	3.70E-17	5.43E-16	29.27266	32.84471	46.614	185.2665	173.268	192.9561
63	MMP1	16238.36	2.35402	0.085171	27.63873	3.81E-168	1.61E-165	5227.881	5655.311	5055.88	27377.04	26465.08	27649

18513	LOC119623	2.584497	2.353559	1.883804	1.249365	0.211532	0.303763	0	1.094824	1.391463	4.464254	8.556443	0
18460	LYPD2	1.926196	2.352838	1.875569	1.254467	0.209672	0.301958	0	0.547412	1.391463	4.464254	2.139111	3.014938
9092	VXN	12.72428	2.352741	0.762548	3.085369	0.002033	0.005944	5.420864	2.737059	4.174388	17.85702	32.08666	14.06971
12211	PSCA	7.182406	2.351542	0.994371	2.364853	0.018037	0.039267	1.084173	3.831883	2.087194	8.928508	17.11289	10.04979
352	H1-1	897.1541	2.350844	0.143533	16.37841	2.73E-60	2.06E-58	258.0331	348.1539	274.8139	1571.417	1514.49	1416.016
18456	LOC103243	2.531966	2.350071	1.871539	1.255689	0.209229	0.301384	0	1.094824	1.391463	0	10.69555	2.009959
19388	LOC119622	1.977839	2.350036	2.099051	1.119571	0.262897	0.360486	0.542086	0	1.391463	8.928508	0	1.004979
14571	TUBB1	4.269569	2.348519	1.228534	1.911643	0.055922	0.10203	1.626259	1.094824	1.391463	8.928508	8.556443	4.019918
83	RNF128	16972.58	2.34829	0.091111	25.77401	1.73E-146	5.56E-144	5311.362	6103.642	5300.082	28019.89	28439.48	28661.01
20374	LOC119623	1.291361	2.348243	2.431971	0.965572	0.334258	0.436157	0.542086	0	0.695731	2.232127	4.278221	0
15989	KCNK18	6.230792	2.347854	1.407597	1.667988	0.095318	0.158486	0	0	6.261582	13.39276	10.69555	7.034856
190	SLC12A1	2634.274	2.347484	0.114465	20.50836	1.81E-93	2.54E-91	813.6716	993.5524	787.5679	4303.541	4447.211	4460.099
12012	MXRA5	8.935188	2.345499	0.975579	2.404212	0.016207	0.03587	1.626259	1.642235	5.565851	8.928508	27.80844	8.039836
20385	CST6	1.292249	2.343674	2.431482	0.963887	0.335103	0.437001	0	0.547412	0.695731	2.232127	4.278221	0
20386	LOC103215	1.292249	2.343674	2.431482	0.963887	0.335103	0.437001	0	0.547412	0.695731	2.232127	4.278221	0
11951	LOC119626	6.944476	2.343348	0.970747	2.413963	0.01578	0.035103	2.710432	2.737059	1.391463	8.928508	12.83466	13.06473
3551	LOC103243	50.46766	2.342944	0.403711	5.803518	6.49E-09	4.86E-08	21.68345	16.42235	11.1317	84.82083	98.39909	70.34856
16775	LOC119619	3.202942	2.341171	1.530627	1.529551	0.126128	0.199887	0	1.094824	2.087194	4.464254	8.556443	3.014938
11033	TRPV5	9.365762	2.34107	0.892622	2.622688	0.008724	0.021022	1.084173	2.737059	5.565851	15.62489	17.11289	14.06971
3806	OTOF	41.92834	2.340312	0.423071	5.531723	3.17E-08	2.21E-07	14.09425	13.6853	13.2189	75.89232	83.42532	51.25395
94	DUSP4	4720.336	2.339879	0.095054	24.61621	8.47E-134	2.40E-131	1605.66	1527.826	1540.349	7698.606	7416.297	8533.281
21049	LACTBL1	1.276746	2.337039	2.678359	0.872563	0.382901	0.483532	0	0.547412	0.695731	0	6.417332	0
21052	LOC103248	1.276746	2.337039	2.678359	0.872563	0.382901	0.483532	0	0.547412	0.695731	0	6.417332	0
9620	SOX7	11.32602	2.336923	0.790671	2.955619	0.00312	0.008623	3.794605	4.379295	2.782925	24.5534	21.39111	11.05477
13515	LOC119622	14.40931	2.336644	1.109892	2.10529	0.035266	0.069371	0	0.547412	13.91463	31.24978	25.66933	15.07469
17855	IL36RN	33.00254	2.336296	1.707168	1.368521	0.171149	0.25483	0	2.189647	30.61218	64.73168	66.31243	34.1693
15625	CALHM1	5.293544	2.336024	1.352446	1.727258	0.084121	0.143097	0	0.547412	4.87012	4.464254	12.83466	9.044815
6204	PROX1	35.58184	2.335672	0.573794	4.070576	4.69E-05	0.000201	5.96295	10.40082	18.78475	62.49956	87.70354	28.13943
13859	LOC103229	5.633046	2.335544	1.146192	2.037655	0.041584	0.079769	1.626259	1.094824	2.782925	17.85702	6.417332	4.019918
11684	ASAH2	8.095038	2.33526	0.943429	2.47529	0.013313	0.030291	1.084173	2.189647	4.87012	13.39276	14.97377	12.05975
15485	DNAJC5B	3.601788	2.334112	1.335869	1.747262	0.080592	0.138362	1.626259	0.547412	1.391463	4.464254	8.556443	5.024897
10694	CUNH19or	12.91736	2.333857	0.866484	2.69348	0.007071	0.017578	1.626259	2.189647	9.044508	26.78552	27.80844	10.04979
6013	BLNK	34.37833	2.333679	0.561143	4.158797	3.20E-05	0.000141	13.01007	17.51718	3.478657	60.26743	40.6431	71.35354
16235	ASB10	4.701288	2.333647	1.440853	1.619629	0.105312	0.17245	0	3.831883	0.695731	6.696381	14.97377	2.009959
12436	TPH2	7.663106	2.332742	1.008417	2.313271	0.020708	0.044268	1.626259	4.379295	1.391463	11.16063	21.39111	6.029877
11866	LOC103244	10.48593	2.332581	0.95864	2.43322	0.014965	0.033526	6.505036	2.189647	1.391463	17.85702	29.94755	5.024897
15348	MAGEA1	4.933727	2.332565	1.31721	1.770838	0.076588	0.132661	0	2.737059	2.087194	8.928508	12.83466	3.014938
18001	IL10RA	2.327705	2.332227	1.737385	1.342378	0.179473	0.265057	0	1.642235	0.695731	4.464254	2.139111	5.024897
14041	CBLC	7.008827	2.332135	1.162311	2.006464	0.044807	0.084836	0	2.189647	4.87012	6.696381	19.252	9.044815
16393	LOC119625	6.950841	2.332094	1.467489	1.589173	0.112021	0.18165	0	0	6.957314	15.62489	17.11289	2.009959
10116	LOC119627	10.2585	2.331541	0.822645	2.834201	0.004594	0.012073	2.710432	3.831883	3.478657	20.08914	21.39111	10.04979
13506	LBX1	17.89287	2.331298	1.10687	2.106207	0.035186	0.06926	0.542086	0	17.39328	35.71403	40.6431	13.06473
12590	NIPAL4	7.566152	2.330719	1.019681	2.285734	0.02227	0.047025	1.084173	2.189647	4.174388	20.08914	12.83466	5.024897
12747	LOC103218	6.588681	2.330663	1.036072	2.249519	0.024479	0.051054	1.084173	3.284471	2.087194	8.928508	17.11289	7.034856
12930	LOC103235	12.71393	2.330473	1.051568	2.216189	0.026679	0.054857	0	1.642235	11.1317	26.78552	25.66933	11.05477
17372	LOC119625	3.171049	2.330026	1.622839	1.435772	0.151067	0.231183	0.542086	0.547412	2.087194	0	12.83466	3.014938
13991	FGF10	6.18612	2.329834	1.158083	2.011802	0.044241	0.084064	0	2.737059	3.478657	11.16063	10.69555	9.044815
10584	HTR1A	24.75422	2.329183	0.85434	2.726295	0.006405	0.016088	1.626259	1.642235	21.56767	49.10679	53.47777	21.10457
17133	KRT36	2.997793	2.32916	1.57824	1.475796	0.139999	0.217234	0.542086	1.094824	1.391463	8.928508	0	6.029877
889	LOC119628	220.6305	2.329084	0.201874	11.53732	8.55E-31	2.56E-29	79.14461	63.49977	76.53045	377.2295	395.7355	331.6432
12884	TAS1R1	5.964746	2.328725	1.048149	2.22175	0.0263	0.054268	2.710432	1.094824	2.087194	11.16063	10.69555	8.039836
6730	LOC119619	18.98663	2.328686	0.605688	3.844697	0.000121	0.000477	7.047123	7.116354	4.87012	29.01765	25.66933	40.19918
7787	ANP32D	20.17661	2.328571	0.671197	3.469279	0.000522	0.001782	3.252518	10.94824	5.565851	49.10679	32.08666	20.09959
9546	LY75	13.08466	2.328483	0.783073	2.973521	0.002944	0.008199	2.710432	3.284471	6.957314	24.5534	29.94755	11.05477
314	TCN2	5836.813	2.327868	0.136121	17.10149	1.45E-65	1.22E-63	2159.13	1621.981	2038.493	9229.845	8663.398	11308.03
18965	MMRN1	1.708372	2.327209	1.954199	1.190876	0.233702	0.327585	0.542086	1.094824	0	4.464254	2.139111	2.009959
18966	LOC103214	1.708372	2.327209	1.954199	1.190876	0.233702	0.327585	0.542086	1.094824	0	4.464254	2.139111	2.009959
13684	LOC103238	5.296433	2.327188	1.123108	2.072096	0.038256	0.074321	1.084173	2.737059	1.391463	6.696381	12.83466	7.034856
11406	RGS22	9.176373	2.325465	0.917521	2.534507	0.011261	0.026246	1.084173	3.831883	4.174388	13.39276	23.53022	9.044815
16479	MRGPRG	2.984231	2.324949	1.475443	1.575763	0.11508	0.185488	1.084173	1.094824	0.695731	4.464254	8.556443	2.009959
16488	DNMT3L	2.984231	2.324949	1.475443	1.575763	0.11508	0.185488	1.084173	1.094824	0.695731	4.464254	8.556443	2.009959
13725	BPIFB1	5.531179	2.324501	1.125444	2.065409	0.038884	0.075318	1.626259	0.547412	3.478657	8.928508	8.556443	10.04979
26793	LOC119619	0.614363	2.324193	3.383486	0.686922	0.492132	NA	0.542086	0	0	0	2.139111	1.004979
27536	ACRV1	0.614363	2.324193	3.383486	0.686922	0.492132	NA	0.542086	0	0	0	2.139111	1.004979
28171	LOC119618	0.614363	2.324193	3.383486	0.686922	0.492132	NA	0.542086	0	0	0	2.139111	1.004979
28346	LOC119619	0.614363	2.324193	3.383486	0.686922	0.492132	NA	0.542086	0	0	0	2.139111	1.004979
28728	LOC103232	0.614363	2.324193	3.383486	0.686922	0.492132	NA	0.542086	0	0	0	2.139111	1.004979
28857	LOC103217	0.614363	2.324193	3.383486	0.686922	0.492132	NA	0.542086	0	0	0	2.139111	1.004979
29381	LOC103233	0.614363	2.324193	3.383486	0.686922	0.492132	NA	0.542086	0	0	0	2.139111	1.004979
26782	LOC103236	0.61525	2.324189	3.381844	0.687255	0.491922	NA	0	0.547412	0	0	2.139111	1.004979
26861	LOC119619	0.61525	2.324189	3.381844	0.687255	0.491922	NA	0	0.547412	0	0	2.139111	1.004979
27033	LOC103234	0.61525	2.324189	3.381844	0.687255	0.491922	NA	0	0.547412	0	0	2.139111	1.004979
27373	LOC119626	0.61525	2.324189	3.381844	0.687255	0.491922	NA	0	0.547412	0	0	2.139111	1.004979
27465	LOC119625	0.61525	2.324189	3.381844	0.687255	0.491922	NA	0	0.547412	0	0	2.139111	1.004979

27878	COX7B2	0.61525	2.324189	3.381844	0.687255	0.491922	NA	0	0.547412	0	0	2.139111	1.004979
27889	LOC11962;	0.61525	2.324189	3.381844	0.687255	0.491922	NA	0	0.547412	0	0	2.139111	1.004979
28506	LOC11962(	0.61525	2.324189	3.381844	0.687255	0.491922	NA	0	0.547412	0	0	2.139111	1.004979
28668	LOC11962(	0.61525	2.324189	3.381844	0.687255	0.491922	NA	0	0.547412	0	0	2.139111	1.004979
29078	LOC11962;	0.61525	2.324189	3.381844	0.687255	0.491922	NA	0	0.547412	0	0	2.139111	1.004979
26663	EFCAB9	0.630753	2.324126	3.354501	0.692838	0.488411	NA	0	0.547412	0	2.232127	0	1.004979
26760	LOC11962⁄	0.630753	2.324126	3.354501	0.692838	0.488411	NA	0	0.547412	0	2.232127	0	1.004979
27026	MRGPRD	0.630753	2.324126	3.354501	0.692838	0.488411	NA	0	0.547412	0	2.232127	0	1.004979
27050	LOC10323!	0.630753	2.324126	3.354501	0.692838	0.488411	NA	0	0.547412	0	2.232127	0	1.004979
27440	TAFA1	0.630753	2.324126	3.354501	0.692838	0.488411	NA	0	0.547412	0	2.232127	0	1.004979
27512	LOC11962(	0.630753	2.324126	3.354501	0.692838	0.488411	NA	0	0.547412	0	2.232127	0	1.004979
27558	UBTFL1	0.630753	2.324126	3.354501	0.692838	0.488411	NA	0	0.547412	0	2.232127	0	1.004979
28485	CBY2	0.630753	2.324126	3.354501	0.692838	0.488411	NA	0	0.547412	0	2.232127	0	1.004979
27048	LOC10323!	0.63997	2.324086	3.337507	0.696354	0.486207	NA	0	0	0.695731	0	2.139111	1.004979
27146	LOC11962⁄	0.63997	2.324086	3.337507	0.696354	0.486207	NA	0	0	0.695731	0	2.139111	1.004979
27157	LOC10323;	0.63997	2.324086	3.337507	0.696354	0.486207	NA	0	0	0.695731	0	2.139111	1.004979
27236	FAM240C	0.63997	2.324086	3.337507	0.696354	0.486207	NA	0	0	0.695731	0	2.139111	1.004979
28137	LOC11961{	0.63997	2.324086	3.337507	0.696354	0.486207	NA	0	0	0.695731	0	2.139111	1.004979
28279	LOC11961{	0.63997	2.324086	3.337507	0.696354	0.486207	NA	0	0	0.695731	0	2.139111	1.004979
28281	LOC10322!	0.63997	2.324086	3.337507	0.696354	0.486207	NA	0	0	0.695731	0	2.139111	1.004979
28611	LOC10324⁄	0.63997	2.324086	3.337507	0.696354	0.486207	NA	0	0	0.695731	0	2.139111	1.004979
28926	LOC10322;	0.63997	2.324086	3.337507	0.696354	0.486207	NA	0	0	0.695731	0	2.139111	1.004979
28934	LOC10323⁄	0.63997	2.324086	3.337507	0.696354	0.486207	NA	0	0	0.695731	0	2.139111	1.004979
28940	LOC10323⁄	0.63997	2.324086	3.337507	0.696354	0.486207	NA	0	0	0.695731	0	2.139111	1.004979
29038	SMCO4	0.63997	2.324086	3.337507	0.696354	0.486207	NA	0	0	0.695731	0	2.139111	1.004979
29245	LOC10323!	0.63997	2.324086	3.337507	0.696354	0.486207	NA	0	0	0.695731	0	2.139111	1.004979
26899	LOC10323{	0.655473	2.324025	3.311781	0.701745	0.482838	NA	0	0	0.695731	2.232127	0	1.004979
27345	LOC11962(	0.655473	2.324025	3.311781	0.701745	0.482838	NA	0	0	0.695731	2.232127	0	1.004979
27618	LOC11962(	0.655473	2.324025	3.311781	0.701745	0.482838	NA	0	0	0.695731	2.232127	0	1.004979
27648	TRNAQ-CU	0.655473	2.324025	3.311781	0.701745	0.482838	NA	0	0	0.695731	2.232127	0	1.004979
28297	LOC11961!	0.655473	2.324025	3.311781	0.701745	0.482838	NA	0	0	0.695731	2.232127	0	1.004979
28530	LOC11962(	0.655473	2.324025	3.311781	0.701745	0.482838	NA	0	0	0.695731	2.232127	0	1.004979
28553	LOC11962(	0.655473	2.324025	3.311781	0.701745	0.482838	NA	0	0	0.695731	2.232127	0	1.004979
28618	LOC11962(	0.655473	2.324025	3.311781	0.701745	0.482838	NA	0	0	0.695731	2.232127	0	1.004979
28754	LOC11962:	0.655473	2.324025	3.311781	0.701745	0.482838	NA	0	0	0.695731	2.232127	0	1.004979
28905	LOC11962:	0.655473	2.324025	3.311781	0.701745	0.482838	NA	0	0	0.695731	2.232127	0	1.004979
29260	LOC11962:	0.655473	2.324025	3.311781	0.701745	0.482838	NA	0	0	0.695731	2.232127	0	1.004979
17281	KRT12	4.11238	2.323826	1.599225	1.453095	0.146197	0.224909	0	0	4.174388	8.928508	8.556443	3.014938
2704	LOC11962:	70.03207	2.323509	0.340277	6.828294	8.59E-12	8.45E-11	18.97302	23.53871	27.13352	136.1597	121.9293	92.45811
12027	LOC10322;	7.9856	2.322277	0.966986	2.401561	0.016325	0.036086	4.878777	1.094824	2.087194	8.928508	12.83466	18.08963
12800	LCP2	6.835796	2.322254	1.036932	2.239544	0.025121	0.052174	2.710432	3.284471	0.695731	17.85702	6.417332	10.04979
18801	LOC10323:	1.947722	2.322034	1.907363	1.217405	0.22345	0.315963	0	0.547412	1.391463	4.464254	4.278221	1.004979
11680	LOC11962:	10.30339	2.321774	0.937793	2.475786	0.013294	0.030259	2.710432	6.568942	0.695731	24.5534	19.252	8.039836
286	AMPD3	622.0587	2.320718	0.130619	17.7671	1.27E-70	1.18E-68	202.1982	218.4173	202.4578	1006.689	1018.217	1084.373
11945	SLC22A16	9.706053	2.319908	0.960598	2.415067	0.015732	0.035014	3.794605	2.189647	3.478657	20.08914	25.66933	3.014938
16143	LOC11962:	4.128108	2.319466	1.415022	1.639174	0.101177	0.166637	2.710432	0.547412	0.695731	13.39276	6.417332	1.004979
13460	TRH	7.497066	2.318982	1.0962	2.115474	0.03439	0.067923	0	2.737059	4.87012	15.62489	10.69555	11.05477
2831	PRRT1B	64.30425	2.317997	0.348818	6.645288	3.03E-11	2.84E-10	18.97302	27.37059	18.08902	113.8385	91.98176	115.5726
20363	LOC11962(	1.715282	2.317563	2.395784	0.967351	0.333369	0.435231	0	1.642235	0	2.232127	6.417332	0
17729	ANGPT4	4.133905	2.31754	1.679865	1.379599	0.16771	0.251485	0	0	4.174388	8.928508	10.69555	1.004979
6644	STYXL2	19.36743	2.316221	0.596905	3.880387	0.000104	0.000417	6.505036	7.116354	5.565851	31.24978	40.6431	25.12449
19666	FBP1	1.72901	2.31591	2.153816	1.075259	0.282259	0.381565	1.084173	0.547412	0	4.464254	4.278221	0
14501	LOC10323:	6.213668	2.314728	1.20233	1.925203	0.054204	0.099373	0	2.737059	3.478657	8.928508	17.11289	5.024897
2405	LOC11962;	91.61558	2.314423	0.318529	7.265975	3.70E-13	4.09E-12	38.48813	33.39212	19.48048	167.4095	141.1813	149.7419
15389	LOC10321(	5.361896	2.3144	1.31363	1.761836	0.078097	0.134915	0.542086	3.284471	1.391463	6.696381	19.252	1.004979
9666	LOC11962:	12.77099	2.31431	0.786794	2.941442	0.003267	0.008985	3.252518	3.284471	6.261582	13.39276	36.36488	14.06971
428	TRABD2B	403.386	2.313851	0.150976	15.3259	5.13E-53	3.19E-51	133.8953	142.8745	128.7103	662.9417	652.4288	699.4657
16910	LOC10323!	3.436329	2.313111	1.537478	1.504484	0.132457	0.208232	0.542086	2.189647	0.695731	11.16063	0	6.029877
8278	LOC10323;	18.17726	2.312978	0.696499	3.320863	0.000897	0.002882	7.047123	2.737059	8.348776	26.78552	47.06043	17.08465
15727	FOXN4	5.976565	2.312833	1.350083	1.713104	0.086693	0.146547	0	0.547412	5.565851	4.464254	19.252	6.029877
15031	LOC11962:	5.840337	2.312816	1.265714	1.827282	0.067657	0.119664	0	1.094824	4.87012	15.62489	6.417332	7.034856
18948	LOC11962!	1.932219	2.312325	1.935262	1.194838	0.23215	0.325718	0	0.547412	1.391463	2.232127	6.417332	1.004979
558	CUNH15or	402.2975	2.311863	0.164246	14.07557	5.37E-45	2.56E-43	126.3061	131.3788	146.1036	705.3521	718.7412	585.903
16535	CEACAM6	3.673016	2.310937	1.472591	1.5693	0.116578	0.187434	1.626259	0	2.087194	11.16063	2.139111	5.024897
15799	LOC10322⁄	5.062524	2.310805	1.357908	1.701738	0.088804	0.149431	1.084173	0	4.174388	2.232127	12.83466	10.04979
242	CORO2B	866.1192	2.310786	0.123703	18.68009	7.19E-78	7.90E-76	266.1644	291.2231	315.862	1428.561	1407.535	1487.37
150	NRBP2	4034.929	2.310391	0.104649	22.07748	5.20E-108	9.20E-106	1294.502	1485.676	1282.929	6482.097	6419.471	7244.897
10038	LOC11962⁄	15.9327	2.308388	0.809005	2.853367	0.004326	0.011456	2.710432	1.642235	11.82743	31.24978	32.08666	16.07967
17904	LOC11962;	2.586804	2.307843	1.695856	1.360872	0.173554	0.257704	0.542086	0	2.087194	4.464254	6.417332	2.009959
6543	PCDH17	24.46057	2.305995	0.587947	3.922117	8.78E-05	0.000357	12.46799	4.379295	7.653045	33.4819	55.61688	33.16432
14241	TEX46	4.681182	2.305944	1.17129	1.968722	0.048985	0.091426	1.084173	2.189647	1.391463	6.696381	10.69555	6.029877
19556	LOC10324(	1.916716	2.303594	2.113377	1.090007	0.27571	0.374809	0	0.547412	1.391463	0	8.556443	1.004979
2951	LOC11962(	61.95176	2.301993	0.354865	6.486957	8.76E-11	7.89E-10	18.43094	16.96977	27.82925	102.6778	96.25998	109.5428
8335	HSPA12B	19.80925	2.300588	0.696682	3.302207	0.000959	0.00306	3.794605	4.379295	11.82743	35.71403	47.06043	16.07967

7480	RASGRP2	18.3564	2.299385	0.644085	3.570001	0.000357	0.001269	9.757555	4.379295	4.174388	33.4819	34.22577	24.11951
10738	LOC11962	11.19994	2.299121	0.856857	2.683202	0.007292	0.018054	2.168345	1.642235	7.653045	24.5534	17.11289	14.06971
2294	POU2F2	155.037	2.298734	0.309334	7.431226	1.08E-13	1.25E-12	29.27266	53.09895	75.13899	283.4801	263.1106	226.1204
18180	LOC11962	2.317337	2.296301	1.75863	1.305733	0.191643	0.280244	0.542086	1.094824	0.695731	0	8.556443	3.014938
283	ZNF385B	1152.645	2.295583	0.128871	17.81308	5.59E-71	5.26E-69	345.8511	443.951	381.2608	1892.844	1828.94	2023.024
16034	LOC11962	3.789555	2.294165	1.381914	1.660136	0.096887	0.160643	1.626259	2.189647	0	4.464254	6.417332	8.039836
236	PLPP3	1000.768	2.293757	0.121511	18.87695	1.76E-79	1.99E-77	319.831	360.197	338.1254	1707.577	1525.186	1753.689
14015	LOC10322	8.118076	2.2932	1.140692	2.010358	0.044393	0.08421	0	2.737059	5.565851	6.696381	25.66933	8.039836
625	MAP1A	351.2598	2.29221	0.172047	13.32314	1.70E-40	7.22E-39	101.3702	135.2107	121.0573	558.0317	598.951	592.9379
9858	EPHA1	16.19195	2.291631	0.792585	2.891339	0.003836	0.010345	2.168345	3.831883	10.43597	29.01765	40.6431	11.05477
1856	LAMB3	124.5963	2.29147	0.276502	8.287338	1.16E-16	1.66E-15	39.03022	37.77142	50.09266	169.6417	263.1106	187.9312
16567	LOC11961	3.883135	2.29012	1.465642	1.562536	0.118162	0.189614	0	0.547412	3.478657	6.696381	8.556443	4.019918
5527	PDE4C	26.75269	2.289746	0.520298	4.400835	1.08E-05	5.19E-05	9.215468	9.306001	8.348776	46.87467	55.61688	31.15436
13285	CPLX3	9.941614	2.289266	1.064126	2.15131	0.031452	0.062941	0	3.831883	6.261582	17.85702	25.66933	6.029877
9498	EVPLL	12.69851	2.288896	0.767042	2.984055	0.002845	0.007962	4.878777	4.926706	2.782925	29.01765	23.53022	11.05477
939	LOC11962	253.9768	2.287854	0.203031	11.26852	1.88E-29	5.31E-28	75.89209	81.56436	102.9682	437.4969	363.6488	462.2906
18890	SMIM2	2.556686	2.287408	1.89797	1.205186	0.228131	0.321046	0	0.547412	2.087194	0	10.69555	2.009959
10709	LOC10324	13.55351	2.286922	0.850309	2.689518	0.007156	0.017765	1.084173	4.379295	8.348776	20.08914	36.36488	11.05477
11323	ZNF804B	8.948915	2.286462	0.895872	2.552218	0.010704	0.025132	2.168345	1.642235	5.565851	13.39276	12.83466	18.08963
7885	SLC4A9	15.65121	2.28587	0.663446	3.445449	0.00057	0.001922	6.505036	4.379295	4.87012	31.24978	27.80844	19.09461
13642	LOC11962	6.905648	2.28423	1.097716	2.080893	0.037444	0.072969	0.542086	1.094824	5.565851	15.62489	8.556443	10.04979
906	EGR3	299.7929	2.284065	0.199701	11.43743	2.72E-30	7.98E-29	84.02339	122.0728	99.48959	569.1924	472.7435	451.2358
737	LOC11962	331.726	2.283502	0.181707	12.56693	3.21E-36	1.16E-34	110.5856	113.8617	116.1871	555.7996	457.7697	636.152
6385	NME9	28.01319	2.283227	0.572917	3.98527	6.74E-05	0.000281	8.131296	7.663765	12.52316	51.33892	66.31243	22.10955
10731	DMBT1	14.39402	2.283201	0.850474	2.684623	0.007261	0.017989	1.626259	2.737059	10.43597	17.85702	40.6431	13.06473
4336	LOC11962	37.70889	2.281883	0.44493	5.128634	2.92E-07	1.79E-06	13.01007	15.32753	9.740239	75.89232	62.03421	50.24897
16115	PXT1	3.406744	2.280956	1.388413	1.642851	0.100414	0.165643	1.626259	0.547412	1.391463	6.696381	2.139111	8.039836
10789	LOC11962	15.6868	2.280649	0.853298	2.672745	0.007523	0.018538	3.252518	1.094824	11.82743	20.08914	42.78221	15.07469
72	NEU1	9532.702	2.28061	0.085756	26.59405	7.95E-156	2.94E-153	3343.589	3066.601	3354.121	15667.3	15433.68	16330.92
3330	ZNF880	57.38827	2.280299	0.379114	6.014818	1.80E-09	1.44E-08	18.43094	25.18094	14.61036	87.05295	117.6511	81.40334
9497	MEST	14.40515	2.280094	0.764026	2.984312	0.002842	0.007956	5.420864	1.642235	7.653045	24.5534	32.08666	15.07469
1156	LOC11962	243.3	2.279043	0.221261	10.30026	7.03E-25	1.62E-23	89.98634	66.23683	92.53227	395.0865	481.2999	334.6582
14312	TPH1	4.705015	2.278595	1.163303	1.95873	0.050144	0.093132	1.626259	1.094824	2.087194	6.696381	10.69555	6.029877
15968	LOC11962	5.214719	2.277799	1.362917	1.671267	0.094669	0.157613	0.542086	0	4.87012	11.16063	10.69555	4.019918
13312	LOC10323	7.58925	2.277138	1.061878	2.144443	0.031997	0.063896	1.084173	4.379295	2.087194	17.85702	17.11289	3.014938
469	LYZ	2214.242	2.274841	0.152763	14.89131	3.75E-50	2.13E-48	854.3281	562.7393	858.5325	3729.884	3743.444	3536.523
20960	SLC1A7	1.310946	2.274554	2.573063	0.883987	0.376703	0.477707	0	0	1.391463	4.464254	0	2.009959
20961	LOC11962	1.310946	2.274554	2.573063	0.883987	0.376703	0.477707	0	0	1.391463	4.464254	0	2.009959
20962	LOC11962	1.310946	2.274554	2.573063	0.883987	0.376703	0.477707	0	0	1.391463	4.464254	0	2.009959
20964	LOC10324	1.310946	2.274554	2.573063	0.883987	0.376703	0.477707	0	0	1.391463	4.464254	0	2.009959
16949	LOC11962	2.994335	2.274105	1.516464	1.49961	0.133715	0.209731	0.542086	1.094824	1.391463	2.232127	10.69555	2.009959
2832	LOC11962	73.21933	2.272893	0.342057	6.64477	3.04E-11	2.85E-10	26.56223	18.06459	30.61218	147.3204	111.2338	105.5228
15276	LOC11961	4.032986	2.271045	1.27301	1.783996	0.074424	0.129521	0.542086	1.642235	2.087194	4.464254	6.417332	9.044815
11333	KCNH6	10.63487	2.269821	0.890052	2.55021	0.010766	0.025254	4.336691	2.189647	4.174388	24.5534	23.53022	5.024897
17468	LOC11962	5.257769	2.269777	1.602607	1.416303	0.156687	0.238476	0.542086	0	4.87012	11.16063	14.97377	0
5585	ADGRG3	27.56487	2.269763	0.518326	4.37903	1.19E-05	5.67E-05	9.215468	9.853413	9.044508	64.73168	36.36488	36.17926
20427	HES5	1.295443	2.269593	2.369829	0.957703	0.338212	0.44015	0	0	1.391463	2.232127	2.139111	2.009959
20428	LOC10323	1.295443	2.269593	2.369829	0.957703	0.338212	0.44015	0	0	1.391463	2.232127	2.139111	2.009959
12661	LOC10323	7.206568	2.268986	1.000418	2.268038	0.023327	0.048981	4.336691	1.642235	1.391463	6.696381	17.11289	12.05975
20073	LOC10321	1.966419	2.268305	2.230786	1.016819	0.30924	0.409562	0	0	2.087194	6.696381	0	3.014938
19422	LOC10321	1.950916	2.267973	2.034463	1.114777	0.264946	0.362642	0	0	2.087194	4.464254	2.139111	3.014938
19423	ARL13A	1.950916	2.267973	2.034463	1.114777	0.264946	0.362642	0	0	2.087194	4.464254	2.139111	3.014938
7930	TSPO2	14.99248	2.267726	0.661037	3.430556	0.000602	0.002019	4.878777	4.379295	6.261582	26.78552	23.53022	24.11951
11097	LRTM2	16.42733	2.267711	0.870181	2.606023	0.00916	0.021945	2.168345	1.642235	13.2189	24.5534	44.92132	12.05975
7332	LOC10321	21.93032	2.267451	0.626011	3.622064	0.000292	0.00106	10.84173	3.831883	7.653045	37.94616	49.19955	22.10955
21384	LOC11962	1.092234	2.266127	2.768983	0.818397	0.413131	0.513612	1.084173	0	0	4.464254	0	1.004979
1094	SAMD9	195.6571	2.265746	0.215059	10.53545	5.93E-26	1.44E-24	55.8349	78.8273	67.48594	339.2833	305.8928	326.6183
10092	DNAAF1	12.04438	2.265225	0.797499	2.840409	0.004506	0.011869	2.168345	6.568942	3.478657	22.32127	25.66933	12.05975
13735	SEPTIN1	8.49174	2.264464	1.097337	2.063599	0.039056	0.075595	4.336691	2.737059	1.391463	20.08914	21.39111	1.004979
20277	RNF224	18.80487	2.264326	2.302572	0.98339	0.325415	0.426649	0	0	19.48048	33.4819	42.78221	17.08465
20886	RPTN	1.040591	2.264058	2.537771	0.892144	0.372316	0.473885	0.542086	0.547412	0	0	2.139111	3.014938
17728	LOC11962	2.720265	2.261492	1.639008	1.379793	0.16765	0.251409	1.626259	0.547412	0.695731	0	6.417332	7.034856
669	SAMD9L	717.6757	2.260953	0.173859	13.00448	1.15E-38	4.58E-37	228.7604	315.3092	198.9792	1113.831	1165.815	1283.359
7711	LOC10323	28.50032	2.260765	0.647999	3.488838	0.000485	0.001673	7.589209	3.831883	18.08902	44.64254	72.72976	24.11951
1692	MHC-DMB	127.346	2.260597	0.258382	8.749036	2.15E-18	3.38E-17	49.87195	35.03436	47.30973	227.677	186.1026	218.0805
19693	LOC10322	1.679674	2.260478	2.110528	1.071048	0.284148	0.383568	1.084173	0	0.695731	0	4.278221	4.019918
14753	UBASH3A	5.738788	2.25989	1.20153	1.880843	0.059993	0.108108	0.542086	3.284471	2.087194	2.232127	19.252	7.034856
8631	SULF1	17.2876	2.259868	0.701873	3.219766	0.001283	0.003952	3.252518	8.758589	5.565851	35.71403	36.36488	14.06971
18290	LOC10321	3.29889	2.259153	1.757936	1.285117	0.198751	0.28889	0	0	3.478657	11.16063	2.139111	3.014938
5140	NRL	33.35113	2.258358	0.490153	4.607455	4.08E-06	2.11E-05	13.01007	7.663765	13.91463	42.41041	74.86887	48.23901
21418	RNF183	1.09401	2.255819	2.767599	0.815082	0.415026	0.515149	0	1.094824	0	4.464254	0	1.004979
17543	LOC11962	2.373062	2.253472	1.599107	1.409206	0.158774	0.240609	0.542086	0.547412	1.391463	4.464254	4.278221	3.014938
6966	LOC11962	25.70124	2.25336	0.600939	3.749733	0.000177	0.000676	7.047123	8.211177	11.1317	42.41041	66.31243	19.09461
4778	LOC11962	38.06006	2.253023	0.466923	4.825256	1.40E-06	7.78E-06	11.9259	19.70683	7.653045	64.73168	62.03421	62.30873

7516	MMRN2	21.1268	2.252657	0.633966	3.553278	0.00038	0.001346	4.336691	5.474118	12.52316	22.32127	44.92132	37.18424
18794	LOC10322	2.627914	2.251775	1.847375	1.218906	0.22288	0.315274	0	0	2.782925	6.696381	4.278221	2.009959
69	KRT8	15828.74	2.250481	0.083457	26.96571	3.73E-160	1.44E-157	5544.459	5562.799	5387.048	26245.35	24751.65	27481.16
11218	TAS2R4	9.529278	2.249265	0.872661	2.577479	0.009952	0.023586	2.168345	2.189647	5.565851	20.08914	17.11289	10.04979
14412	KIF19	11.73587	2.248134	1.158028	1.941347	0.052216	0.09632	0	0.547412	11.82743	22.32127	25.66933	10.04979
11956	LOC11961	10.78706	2.246962	0.931155	2.413091	0.015818	0.035172	2.168345	2.737059	6.261582	11.16063	36.36488	6.029877
7139	LOC10322	18.2413	2.246906	0.609934	3.683851	0.00023	0.000856	4.336691	7.116354	7.653045	31.24978	29.94755	29.1444
21159	LOC10324	1.332471	2.246382	2.621337	0.85696	0.391467	0.491854	0	0	1.391463	4.464254	2.139111	0
19590	CUNH4orf	1.972441	2.245436	2.065339	1.0872	0.276949	0.375819	0	0	2.087194	4.464254	4.278221	1.004979
19591	LOC10322	1.972441	2.245436	2.065339	1.0872	0.276949	0.375819	0	0	2.087194	4.464254	4.278221	1.004979
11360	AR	11.64775	2.244724	0.881585	2.546238	0.010889	0.025483	2.710432	4.379295	4.87012	13.39276	38.50399	6.029877
16230	CCT8L2	3.690031	2.244081	1.383953	1.6215	0.10491	0.171845	0.542086	0.547412	2.782925	6.696381	8.556443	3.014938
157	ST3GAL1	10177.05	2.242858	0.103231	21.72659	1.15E-104	1.95E-102	3550.124	3755.792	3346.468	16408.37	15348.12	18653.42
15894	ARHGAP2	3.450682	2.242822	1.330614	1.685554	0.091882	0.153685	1.084173	1.094824	1.391463	6.696381	6.417332	4.019918
18184	LOC11961	2.358447	2.242581	1.718497	1.304966	0.191904	0.280564	0	1.094824	1.391463	2.232127	6.417332	3.014938
21094	LOC11962	1.279941	2.242178	2.590309	0.865603	0.386708	0.487326	0	0	1.391463	0	4.278221	2.009959
21095	LOC11961	1.279941	2.242178	2.590309	0.865603	0.386708	0.487326	0	0	1.391463	0	4.278221	2.009959
13908	ACADL	7.673943	2.241106	1.104298	2.029439	0.042414	0.081073	2.168345	2.189647	3.478657	20.08914	17.11289	1.004979
18311	CALB2	2.409203	2.240751	1.749902	1.2805	0.200369	0.290892	1.084173	0	1.391463	6.696381	4.278221	1.004979
14298	LOC11961	5.219914	2.239749	1.141597	1.961944	0.049769	0.092538	2.168345	1.094824	2.087194	13.39276	8.556443	4.019918
14731	LOC11962	5.135491	2.239409	1.188993	1.88345	0.059639	0.107631	2.710432	0.547412	2.087194	4.464254	14.97377	6.029877
10543	TRIM54	14.66904	2.23938	0.81878	2.735021	0.006238	0.015729	0.542086	6.568942	8.348776	26.78552	25.66933	20.09959
13105	LIN28A	9.886525	2.239179	1.023936	2.186836	0.028755	0.058332	1.626259	0.547412	8.348776	8.928508	27.80844	12.05975
19987	LOC10324	1.771008	2.23868	2.170322	1.031497	0.302308	0.402104	0	1.094824	0.695731	6.696381	2.139111	0
11297	LOC11962	12.19365	2.237883	0.87429	2.559657	0.010478	0.024657	3.252518	4.379295	4.87012	24.5534	32.08666	4.019918
17482	LOC11962	3.03367	2.237765	1.581411	1.415043	0.157056	0.238836	1.084173	0	2.087194	4.464254	8.556443	2.009959
6371	PHKG1	27.83215	2.237491	0.560216	3.993982	6.50E-05	0.000271	11.9259	10.40082	6.261582	49.10679	64.17332	25.12449
15593	LOC11962	3.887443	2.237208	1.29297	1.730287	0.083579	0.142497	2.168345	1.094824	0.695731	8.928508	6.417332	4.019918
232	CAMK2B	1017.668	2.236444	0.117742	18.99443	1.90E-80	2.17E-78	333.9252	372.7874	363.8675	1624.988	1623.585	1786.853
96	SERPINE2	65575.03	2.236381	0.09151	24.43864	6.65E-132	1.84E-129	22815.33	24262.39	21803.53	106459.1	101671.9	116437.9
17950	LOC10324	3.92938	2.236149	1.654963	1.351177	0.176639	0.261612	0	0	4.174388	6.696381	10.69555	2.009959
19245	H1FOO	1.718477	2.236108	1.952539	1.145231	0.252113	0.348251	0	1.094824	0.695731	2.232127	4.278221	2.009959
19246	LOC10323	1.718477	2.236108	1.952539	1.145231	0.252113	0.348251	0	1.094824	0.695731	2.232127	4.278221	2.009959
10089	ADRA2B	11.61862	2.235969	0.787138	2.840632	0.004502	0.011864	2.168345	3.284471	6.957314	20.08914	17.11289	20.09959
15026	RIT2	7.604796	2.235593	1.22269	1.828422	0.067486	0.119401	0	1.094824	6.957314	11.16063	21.39111	5.024897
17278	LOC11962	3.710669	2.235592	1.537917	1.453649	0.146044	0.224712	1.084173	0	2.782925	6.696381	10.69555	1.004979
21015	TRNAF-GA	1.076732	2.235458	2.5468	0.877752	0.380078	0.480804	1.084173	0	0	2.232127	2.139111	1.004979
20206	LOC10324	1.919911	2.234906	2.244229	0.995846	0.319325	0.420136	0	0	2.087194	0	6.417332	3.014938
16144	LOC10323	4.351526	2.234393	1.363473	1.638752	0.101265	0.166757	0.542086	0.547412	3.478657	6.696381	12.83466	2.009959
13336	F2RL3	6.51944	2.234131	1.043488	2.141022	0.032272	0.064334	1.626259	1.094824	4.174388	15.62489	8.556443	8.039836
15499	LOC11962	4.109349	2.233489	1.280274	1.744539	0.081065	0.139049	1.084173	0.547412	2.782925	8.928508	4.278221	7.034856
11524	KCNK7	10.58882	2.233443	0.890173	2.508999	0.012107	0.027931	2.168345	3.284471	5.565851	13.39276	32.08666	7.034856
7109	MYLK2	24.19873	2.232369	0.604291	3.694196	0.000221	0.000825	10.84173	9.853413	4.174388	46.87467	51.33866	22.10955
12921	RNF39	10.14499	2.229794	1.005382	2.217857	0.026565	0.054653	0	3.831883	6.957314	15.62489	21.39111	13.06473
21217	LOC11962	1.316969	2.229682	2.629588	0.84792	0.396482	0.496677	0	0	1.391463	2.232127	4.278221	0
21218	LOC11962	1.316969	2.229682	2.629588	0.84792	0.396482	0.496677	0	0	1.391463	2.232127	4.278221	0
21220	BCL2L10	1.316969	2.229682	2.629588	0.84792	0.396482	0.496677	0	0	1.391463	2.232127	4.278221	0
21222	LINGO3	1.316969	2.229682	2.629588	0.84792	0.396482	0.496677	0	0	1.391463	2.232127	4.278221	0
4230	MEFV	48.11151	2.228826	0.428741	5.198539	2.01E-07	1.26E-06	23.30971	9.306001	18.08902	75.89232	87.70354	74.36848
21543	LOC11962	1.061229	2.228688	2.792069	0.798221	0.424742	0.524117	1.084173	0	0	0	4.278221	1.004979
21544	LOC10323	1.061229	2.228688	2.792069	0.798221	0.424742	0.524117	1.084173	0	0	0	4.278221	1.004979
590	NLRC5	541.3629	2.227669	0.162483	13.71016	8.83E-43	3.98E-41	182.141	169.1503	222.634	863.8331	806.4447	1003.974
5414	CALHM2	30.44015	2.227489	0.499993	4.455038	8.39E-06	4.12E-05	7.589209	13.6853	11.1317	53.57105	36.36488	60.29877
5459	LOC11962	36.33495	2.226844	0.501929	4.436571	9.14E-06	4.45E-05	8.673382	15.32753	13.91463	62.49956	83.42532	34.1693
5245	LOC11962	45.94942	2.225477	0.488479	4.555927	5.22E-06	2.64E-05	17.88885	12.59047	17.39328	102.6778	91.98176	33.16432
19965	NPPB	1.755505	2.224836	2.148084	1.035731	0.300328	0.399911	0	1.094824	0.695731	4.464254	4.278221	0
17350	RTL4	3.493732	2.224479	1.545559	1.439272	0.150074	0.229954	1.084173	1.094824	1.391463	6.696381	10.69555	0
19326	LOC11961	2.176764	2.223125	1.965273	1.131204	0.257969	0.354865	1.084173	1.094824	0	4.464254	6.417332	0
12732	GRID1	8.90721	2.223105	0.987383	2.251513	0.024353	0.050847	0.542086	3.284471	5.565851	15.62489	21.39111	7.034856
2564	LOC11962	117.5452	2.22251	0.316182	7.029219	2.08E-12	2.15E-11	34.69353	31.20247	59.8329	198.6593	149.7377	231.1453
11463	LOC10324	9.355759	2.221754	0.880233	2.524051	0.011601	0.026905	2.168345	2.189647	5.565851	22.32127	12.83466	11.05477
20060	LOC11962	1.738227	2.221124	2.179324	1.01918	0.308117	0.40832	1.084173	0	0.695731	2.232127	6.417332	0
11330	LOC11962	9.288613	2.220878	0.870621	2.550914	0.010744	0.02521	1.626259	2.737059	5.565851	15.62489	17.11289	13.06473
1598	LOC10323	126.679	2.220323	0.247205	8.981718	2.67E-19	4.43E-18	39.5723	49.26706	45.91827	209.8199	190.3808	225.1154
11711	TLR7	11.24733	2.219476	0.898773	2.469451	0.013532	0.030719	0.542086	3.831883	7.653045	17.85702	23.53022	14.06971
20406	SEMG2	1.941436	2.219331	2.311339	0.960193	0.336958	0.43899	0	0	2.087194	0	8.556443	1.004979
14468	LOC11962	12.17132	2.218227	1.148959	1.930641	0.053527	0.098357	0	0.547412	12.52316	20.08914	27.80844	12.05975
102	BACE2	5710.487	2.216193	0.091337	24.26396	4.71E-130	1.23E-127	2041.497	2032.54	1996.053	9283.416	8817.414	10092
10287	LOC11962	13.39644	2.214892	0.793398	2.791652	0.005244	0.013552	3.252518	3.284471	7.653045	15.62489	38.50399	12.05975
1702	LOC10322	113.9998	2.214519	0.25401	8.71823	2.83E-18	4.41E-17	38.48813	37.77142	45.22254	180.8023	196.7982	184.9162
4430	TMEM178	64.82817	2.214362	0.437763	5.058354	4.23E-07	2.54E-06	10.84173	21.34906	36.87376	124.9991	115.512	79.39338
13486	ADCY2	7.647907	2.214289	1.049464	2.109924	0.034865	0.068729	1.084173	2.737059	4.174388	15.62489	19.252	3.014938
2491	OSR2	84.6823	2.213727	0.309876	7.143904	9.07E-13	9.68E-12	30.89892	24.08612	34.78657	154.0168	149.7377	114.5677
17250	LOC11961	3.746644	2.213069	1.51805	1.457837	0.144885	0.223292	0	0.547412	3.478657	11.16063	4.278221	3.014938

21833	LOC10324(	1.301466	2.212886	2.932207	0.754683	0.450439	0.548378	0	0	1.391463	0	6.417332	0
21834	LOC11962	1.301466	2.212886	2.932207	0.754683	0.450439	0.548378	0	0	1.391463	0	6.417332	0
21835	DYDC2	1.301466	2.212886	2.932207	0.754683	0.450439	0.548378	0	0	1.391463	0	6.417332	0
21836	CFAP161	1.301466	2.212886	2.932207	0.754683	0.450439	0.548378	0	0	1.391463	0	6.417332	0
21837	LOC10324	1.301466	2.212886	2.932207	0.754683	0.450439	0.548378	0	0	1.391463	0	6.417332	0
12368	LOC10323	11.49181	2.212481	0.950083	2.328725	0.019874	0.04272	0.542086	2.737059	9.044508	15.62489	29.94755	11.05477
5773	KLHL4	27.45529	2.212331	0.516056	4.286999	1.81E-05	8.34E-05	6.505036	13.13788	9.740239	40.17829	44.92132	50.24897
5231	PLXNA4	41.12013	2.212021	0.484751	4.56321	5.04E-06	2.56E-05	16.80468	10.40082	16.00182	87.05295	81.28621	35.17428
16527	LOC11962	3.807989	2.211611	1.408353	1.570352	0.116333	0.18712	1.626259	1.094824	1.391463	0	10.69555	8.039836
8827	APOB	14.4318	2.211263	0.698901	3.163914	0.001557	0.004688	2.710432	7.116354	5.565851	24.5534	23.53022	23.11453
13549	KLHL38	11.95669	2.211101	1.053622	2.09857	0.035855	0.070352	0.542086	0.547412	11.82743	20.08914	25.66933	13.06473
18577	LOC10322	2.611585	2.209881	1.78433	1.238493	0.215533	0.308427	1.626259	1.094824	0	8.928508	0	4.019918
3433	LOC10321	51.25628	2.209506	0.37353	5.915207	3.31E-09	2.57E-08	17.88885	15.87494	20.87194	78.12444	98.39909	76.37844
14507	GDAP1L1	6.745819	2.209305	1.148978	1.922843	0.0545	0.099874	4.336691	0	2.782925	15.62489	10.69555	7.034856
17818	LOC11962	2.802119	2.209039	1.607602	1.37412	0.169404	0.252742	0.542086	1.642235	0.695731	2.232127	10.69555	1.004979
19471	LOC10321	2.083489	2.208888	1.998671	1.105178	0.269082	0.367395	1.626259	0	0.695731	0	2.139111	8.039836
15155	SMIM38	5.453866	2.208374	1.223431	1.805066	0.071064	0.124662	1.084173	0.547412	4.174388	8.928508	14.97377	3.014938
9888	SOAT2	18.89452	2.207288	0.76471	2.886437	0.003896	0.010476	5.96295	1.094824	13.2189	33.4819	38.50399	21.10457
10402	LOC11962	12.8122	2.204659	0.797038	2.766067	0.005674	0.014501	3.252518	2.737059	7.653045	20.08914	32.08666	11.05477
14570	ARX	7.445892	2.203448	1.152605	1.911712	0.055913	0.102021	0.542086	0.547412	6.957314	15.62489	14.97377	6.029877
5485	MYOZ3	41.64991	2.203326	0.49897	4.415751	1.01E-05	4.88E-05	8.673382	10.94824	25.04633	71.42806	85.56443	48.23901
14837	GPR150	6.487865	2.202848	1.181623	1.864255	0.062286	0.111604	2.710432	3.284471	0.695731	6.696381	23.53022	2.009959
9661	TMEM82	15.53576	2.20277	0.748322	2.943612	0.003244	0.008927	4.336691	4.379295	7.653045	35.71403	32.08666	9.044815
273	TM4SF4	3259.859	2.202526	0.122778	17.93903	5.85E-72	5.69E-70	1261.435	968.9189	1262.057	5497.729	5037.606	5531.407
1596	RP1	124.966	2.202005	0.245004	8.987634	2.53E-19	4.21E-18	44.45108	44.88777	44.52681	183.0344	228.8848	204.0108
10686	ALDH3B2	10.47277	2.20159	0.816764	2.695503	0.007028	0.017485	2.710432	3.831883	4.87012	8.928508	21.39111	21.10457
16570	SNX32	5.461663	2.200564	1.408583	1.562254	0.118228	0.189675	0	1.642235	4.174388	6.696381	19.252	1.004979
12555	DTHD1	9.359211	2.200071	0.95977	2.29229	0.021889	0.046348	1.626259	2.737059	5.565851	13.39276	27.80844	5.024897
545	TFEB	403.8754	2.199942	0.155475	14.14977	1.87E-45	9.14E-44	138.7741	135.7581	160.0182	651.7811	637.455	699.4657
18402	FCRL4	2.414173	2.199774	1.740002	1.264237	0.206145	0.297814	0	0.547412	2.087194	6.696381	2.139111	3.014938
1110	APOL6	202.2195	2.19865	0.209727	10.48339	1.03E-25	2.47E-24	74.80792	78.27989	62.61582	341.5154	363.6488	292.449
327	IL1R1	2126.75	2.198629	0.130773	16.81259	1.97E-63	1.60E-61	672.7292	901.5873	708.9503	3352.655	3366.96	3757.618
223	LOC10323	1261.524	2.19855	0.113528	19.36575	1.50E-83	1.79E-81	464.568	435.1924	456.3998	2006.682	1955.147	2251.154
17649	DEFB124	3.752666	2.197509	1.57364	1.39645	0.162579	0.244864	0	0.547412	3.478657	8.928508	8.556443	1.004979
3313	CADM2	62.19357	2.196907	0.364303	6.030445	1.64E-09	1.31E-08	20.59928	29.56024	16.00182	111.6063	106.9555	88.43819
10648	ZNF157	11.68228	2.195991	0.811669	2.705525	0.00682	0.017027	5.420864	2.737059	4.174388	15.62489	32.08666	10.04979
12823	LOC10324	7.835674	2.195819	0.982878	2.23407	0.025478	0.052823	1.084173	4.379295	2.782925	15.62489	17.11289	6.029877
14366	JSRP1	6.571707	2.194783	1.125949	1.949274	0.051263	0.094864	1.626259	0.547412	4.87012	13.39276	14.97377	4.019918
9325	NAALADL1	26.86358	2.194398	0.725495	3.024692	0.002489	0.007096	5.420864	3.284471	20.17621	71.42806	42.78221	18.08963
5132	SMOC1	36.33568	2.192894	0.475628	4.610522	4.02E-06	2.08E-05	9.215468	14.23271	15.30609	75.89232	64.17332	39.1942
12190	LOC10323	8.662995	2.19202	0.925589	2.368244	0.017873	0.038978	2.168345	1.642235	5.565851	11.16063	21.39111	10.04979
14404	LOC10322	6.503674	2.191618	1.128165	1.94264	0.05206	0.096085	1.626259	0.547412	4.87012	6.696381	19.252	6.029877
9574	NLRP12	16.01593	2.189618	0.737993	2.96699	0.003007	0.008351	5.420864	3.284471	8.348776	40.17829	27.80844	11.05477
18858	CXCL17	2.450313	2.189426	1.807821	1.211085	0.225863	0.318436	0.542086	0	2.087194	8.928508	2.139111	1.004979
19650	AOC1	2.547799	2.189388	2.031564	1.077686	0.281174	0.380407	2.710432	0	0	0	8.556443	4.019918
10161	LAMA2	17.80118	2.188846	0.775234	2.823465	0.004751	0.01243	1.626259	10.40082	6.957314	24.5534	49.19955	14.06971
16809	LOC11962	3.681499	2.188255	1.436152	1.523693	0.127585	0.201788	1.084173	2.737059	0	6.696381	8.556443	3.014938
9283	LOC10323	21.86917	2.187855	0.72059	3.036201	0.002396	0.006861	8.131296	1.642235	13.91463	26.78552	55.61688	25.12449
1688	SLC43A3	125.8984	2.187719	0.249958	8.752365	2.09E-18	3.29E-17	39.5723	49.26706	46.614	220.9806	216.0502	182.9063
18866	LOC11961	2.451201	2.187107	1.807813	1.209808	0.226352	0.31896	0	0.547412	2.087194	8.928508	2.139111	1.004979
17953	COX4I2	3.060165	2.186389	1.619193	1.350296	0.176921	0.261972	0	0.547412	2.782925	4.464254	8.556443	2.009959
17954	CLDN5	3.060165	2.186389	1.619193	1.350296	0.176921	0.261972	0	0.547412	2.782925	4.464254	8.556443	2.009959
8236	MYO1F	23.32494	2.186286	0.655435	3.335628	0.000851	0.002747	7.589209	10.94824	6.261582	22.32127	72.72976	20.09959
8361	LOC10323	21.25384	2.186146	0.663574	3.294504	0.000986	0.003135	11.9259	4.379295	6.261582	31.24978	55.61688	18.08963
16725	LOC10323	3.259353	2.182709	1.41922	1.537964	0.124058	0.197194	0.542086	2.189647	0.695731	6.696381	6.417332	3.014938
16767	LOC10324	4.839032	2.182331	1.425921	1.530472	0.1259	0.199609	0	4.379295	0.695731	13.39276	8.556443	2.009959
16677	LOC10321	3.482312	2.18178	1.411786	1.545405	0.122248	0.194877	0.542086	1.094824	2.087194	4.464254	10.69555	2.009959
16548	SOX10	4.104839	2.180441	1.392404	1.565954	0.117359	0.188542	0	1.094824	3.478657	4.464254	8.556443	7.034856
8897	LOC10323	39.00117	2.180272	0.693199	3.145232	0.00166	0.004959	4.878777	8.211177	29.22072	44.64254	121.9293	25.12449
729	GRIN3B	295.2007	2.17769	0.172719	12.60832	1.90E-36	6.93E-35	101.3702	103.4608	116.8829	486.6037	451.3524	511.5345
17865	LOC10322	3.503837	2.177669	1.593281	1.366783	0.171693	0.255455	0.542086	1.094824	2.087194	4.464254	12.83466	0
8729	LOC11961	19.87226	2.17741	0.68179	3.193666	0.001405	0.004278	6.505036	7.663765	6.957314	35.71403	51.33866	11.05477
16136	LOC11962	10.17666	2.177164	1.327835	1.639635	0.101081	0.166522	0	0	11.1317	26.78552	17.11289	6.029877
15078	TMPRSS15	6.036133	2.176592	1.195755	1.820265	0.068719	0.121154	3.252518	1.094824	2.087194	2.232127	23.53022	4.019918
15495	LOC11962	8.080464	2.175108	1.246295	1.74526	0.08094	0.138869	0	0.547412	8.348776	11.16063	21.39111	7.034856
6924	ACTRT3	22.35474	2.174495	0.577544	3.765075	0.000166	0.000639	7.047123	5.474118	11.82743	35.71403	44.92132	29.1444
12422	TBX21	11.78757	2.174114	0.938484	2.316623	0.020524	0.043925	0.542086	3.284471	9.044508	17.85702	29.94755	10.04979
4429	SLC5A1	52.57182	2.172053	0.429361	5.058807	4.22E-07	2.53E-06	17.88885	10.94824	28.52499	87.05295	102.6773	68.3386
14197	GUCA1B	6.357078	2.171976	1.098361	1.97747	0.047989	0.089862	2.168345	0.547412	4.174388	13.39276	12.83466	5.024897
2159	SERTAD4	143.3518	2.171395	0.283074	7.670765	1.71E-14	2.11E-13	44.45108	73.35318	37.56949	252.2303	237.4413	215.0656
2468	TMEM71	95.1951	2.169926	0.302817	7.16581	7.73E-13	8.33E-12	41.74065	33.39212	29.22072	124.9991	151.8769	189.9411
8733	LOC11962	15.02422	2.169784	0.67955	3.19297	0.001408	0.004287	4.878777	3.831883	7.653045	29.01765	25.66933	19.09461
11662	LMO2	13.30153	2.169596	0.875233	2.478878	0.01318	0.030045	2.168345	3.831883	8.348776	40.17829	19.252	6.029877
16162	PLCZ1	6.836664	2.169088	1.327297	1.634215	0.102214	0.168132	0.542086	0	6.957314	13.39276	17.11289	3.014938

285	ARRDC4	1464.635	2.168871	0.122038	17.77208	1.16E-70	1.08E-68	481.3727	605.9849	512.0583	2345.965	2320.935	2521.493
13633	NOBOX	7.860264	2.168864	1.041793	2.081857	0.037356	0.07284	5.96295	1.642235	0.695731	17.85702	14.97377	6.029877
12996	KLK7	23.02771	2.168585	0.983918	2.204029	0.027522	0.0563	1.084173	0.547412	23.65487	40.17829	55.61688	17.08465
210	MYRF	2338.441	2.167057	0.109246	19.83653	1.44E-87	1.82E-85	801.2037	940.4535	814.7014	3685.242	3702.801	4086.247
12429	LOC10321	9.518114	2.167025	0.935819	2.315645	0.020578	0.044015	0.542086	4.379295	5.565851	11.16063	21.39111	14.06971
11864	BMX	10.31992	2.166611	0.890228	2.43377	0.014942	0.033483	1.626259	3.284471	6.261582	22.32127	21.39111	7.034856
18085	FGB	2.602105	2.165812	1.634752	1.324857	0.185219	0.272272	1.626259	1.094824	0	4.464254	6.417332	2.009959
13870	SPINK13	6.301248	2.165508	1.063485	2.036237	0.041727	0.079978	1.626259	3.831883	1.391463	15.62489	4.278221	11.05477
16879	SLC38A11	3.8724	2.164235	1.433425	1.509835	0.131086	0.206464	0	2.189647	2.087194	2.232127	10.69555	6.029877
9675	LOC11962	12.67896	2.163321	0.736512	2.937251	0.003311	0.009099	4.336691	3.831883	5.565851	29.01765	19.252	14.06971
17123	EDNRA	3.501897	2.16306	1.463824	1.477678	0.139494	0.216577	0	1.094824	2.782925	6.696381	6.417332	4.019918
16522	KLKB1	3.262547	2.162875	1.37678	1.570966	0.11619	0.186958	0.542086	1.642235	1.391463	6.696381	4.278221	5.024897
15272	KDR	4.361692	2.162424	1.211734	1.78457	0.074331	0.129398	1.084173	2.189647	1.391463	8.928508	8.556443	4.019918
14185	LOC11962	5.666352	2.162331	1.092373	1.979481	0.047762	0.089513	1.084173	1.642235	3.478657	8.928508	12.83466	6.029877
565	NCF4	396.6259	2.162244	0.154619	13.98431	1.94E-44	9.14E-43	138.7741	140.6848	156.5396	611.6028	641.7332	690.4209
871	KIRREL3	239.7262	2.161979	0.185044	11.68357	1.55E-31	4.72E-30	89.98634	86.49107	86.96642	386.158	363.6488	425.1063
18412	LOC11962	2.588377	2.160927	1.710781	1.263123	0.206545	0.298213	0.542086	2.189647	0	2.232127	8.556443	2.009959
8284	ASB12	20.02869	2.160856	0.651344	3.317534	0.000908	0.002914	3.252518	11.49565	6.957314	35.71403	40.6431	22.10955
17942	SERPINB7	3.057563	2.160545	1.597126	1.35277	0.176129	0.260979	2.710432	0.547412	0	8.928508	2.139111	4.019918
16706	LOC10322	3.485506	2.160388	1.401411	1.541581	0.123175	0.196014	0.542086	0.547412	2.782925	4.464254	8.556443	4.019918
16793	LOC11961	4.199533	2.160387	1.414654	1.527148	0.126724	0.200624	0.542086	0.547412	3.478657	8.928508	10.69555	1.004979
14274	LOC10323	5.409725	2.159195	1.098272	1.965993	0.049299	0.09183	2.710432	1.094824	2.087194	6.696381	12.83466	7.034856
346	B3GNT7	1061.215	2.159046	0.131148	16.46267	6.80E-61	5.23E-59	361.5716	414.3907	391.001	1732.131	1531.603	1936.595
18183	FATE1	2.180846	2.158893	1.654326	1.304998	0.191893	0.280564	0.542086	1.094824	0.695731	4.464254	4.278221	2.009959
306	CDH6	10869.13	2.158327	0.124547	17.32941	2.82E-67	2.45E-65	3597.827	4781.642	3555.883	17591.39	16948.17	18739.85
12515	OTOA	9.189774	2.157968	0.938815	2.298608	0.021527	0.045729	1.084173	2.737059	6.261582	15.62489	21.39111	8.039836
6925	LOC10323	31.6053	2.157825	0.573134	3.764961	0.000167	0.000639	4.878777	10.40082	19.48048	49.10679	70.59065	35.17428
6963	GRAP2	31.18595	2.15779	0.575275	3.750882	0.000176	0.000673	8.673382	9.853413	15.30609	64.73168	68.45154	20.09959
5375	ZNF835	30.02992	2.15707	0.48177	4.477387	7.56E-06	3.74E-05	13.55216	9.853413	9.740239	49.10679	40.6431	57.28383
15282	SLC38A4	6.540855	2.156574	1.209591	1.782895	0.074603	0.129782	0.542086	4.379295	2.087194	6.696381	23.53022	2.009959
18567	LOC11962	3.041835	2.156331	1.738522	1.240324	0.214856	0.307622	0	0	3.478657	4.464254	4.278221	6.029877
18953	LOC11961	2.166231	2.155636	1.805233	1.194104	0.232437	0.326035	0	1.642235	0.695731	2.232127	6.417332	2.009959
1344	LARGE1	195.5581	2.155595	0.221499	9.731863	2.21E-22	4.36E-21	66.13454	60.2153	89.74935	301.3371	327.2839	328.6283
16042	GDPD4	5.733333	2.155376	1.300057	1.657908	0.097336	0.161306	0	2.189647	4.174388	17.85702	2.139111	8.039836
11555	LOC10322	9.355293	2.154891	0.860695	2.503665	0.012291	0.028277	3.252518	2.737059	4.174388	13.39276	23.53022	9.044815
1799	LOC10324	108.9272	2.1547	0.25481	8.456107	2.76E-17	4.09E-16	41.19856	41.05589	37.56949	178.5702	173.268	181.9013
21148	TAAR9	1.081701	2.154378	2.50996	0.858331	0.39071	0.491112	0	0.547412	0.695731	2.232127	0	3.014938
21149	GRXCR1	1.081701	2.154378	2.50996	0.858331	0.39071	0.491112	0	0.547412	0.695731	2.232127	0	3.014938
15660	LOC10322	4.072119	2.154179	1.250089	1.72322	0.084849	0.144042	2.168345	1.642235	0.695731	4.464254	6.417332	9.044815
424	C1QTNF6	533.577	2.154155	0.140077	15.37842	2.28E-53	1.43E-51	191.3565	191.0467	206.6322	848.2083	823.5576	940.6608
6883	LOC11962	39.93927	2.15406	0.569277	3.783851	0.000154	0.000596	7.589209	9.853413	26.43779	75.89232	87.70354	32.15934
10645	LOC10323	11.48503	2.153495	0.79548	2.707164	0.006786	0.016948	4.878777	2.189647	5.565851	13.39276	27.80844	15.07469
6692	LOC11962	23.00073	2.153018	0.557818	3.859715	0.000114	0.000451	6.505036	6.568942	12.52316	35.71403	38.50399	38.18922
17392	MUCL1	3.521647	2.152186	1.504564	1.430439	0.152591	0.233236	1.084173	0	2.782925	6.696381	8.556443	2.009959
17000	LOC11962	4.160564	2.151503	1.442019	1.492007	0.135697	0.212207	0	0.547412	4.174388	8.928508	4.278221	7.034856
16438	LOC11962	5.468053	2.151492	1.359615	1.582427	0.113552	0.183646	0	0.547412	5.565851	6.696381	14.97377	5.024897
18542	SPRN	3.761884	2.149494	1.727636	1.244182	0.213433	0.306011	0	0	4.174388	6.696381	10.69555	1.004979
6702	LOC11962	24.21767	2.149292	0.55768	3.853989	0.000116	0.000461	8.131296	8.211177	10.43597	55.80317	23.53022	39.1942
8811	WNT9B	28.43909	2.149222	0.677596	3.171835	0.001515	0.004571	8.131296	7.663765	15.30609	33.4819	91.98176	14.06971
178	CD24	20266.84	2.148751	0.102775	20.90733	4.59E-97	6.86E-95	6944.668	8494.189	6935.746	33225.21	33310.23	32690.98
6576	ARPP21	45.76823	2.148185	0.549389	3.910131	9.22E-05	0.000373	5.96295	18.612	25.74206	102.6778	83.42532	38.18922
14117	LOC11962	6.093468	2.147702	1.078802	1.990822	0.046501	0.08757	0.542086	2.737059	3.478657	8.928508	12.83466	8.039836
1482	LOC10322	171.9255	2.147672	0.230933	9.299982	1.40E-20	2.52E-19	71.5554	49.81448	68.87741	301.3371	254.5542	285.4142
16339	LOC11961	4.399252	2.147639	1.343936	1.598022	0.110038	0.179042	2.710432	0	2.087194	11.16063	6.417332	4.019918
7797	LOC10321	38.02275	2.147284	0.619496	3.46618	0.000528	0.0018	4.878777	8.758589	28.52499	55.80317	91.98176	38.18922
20300	LOC10324	1.487457	2.14695	2.194603	0.978286	0.327933	0.42947	1.084173	0.547412	0	0	4.278221	3.014938
18583	TPSG1	3.06336	2.146404	1.734572	1.237426	0.215929	0.30891	0	0	3.478657	4.464254	6.417332	4.019918
20115	LOC11962	1.727694	2.144789	2.123194	1.010171	0.312414	0.412901	0	0.547412	1.391463	0	6.417332	2.009959
15045	UPK3A	5.862082	2.14282	1.174009	1.825216	0.067968	0.120103	1.626259	3.284471	1.391463	4.464254	21.39111	3.014938
18797	LOC11961	3.100388	2.142362	1.758343	1.218398	0.223073	0.315497	0	0	3.478657	6.696381	6.417332	2.009959
12238	DCST2	8.261119	2.142115	0.908029	2.359083	0.01832	0.039797	1.626259	2.737059	4.87012	11.16063	17.11289	12.05975
11562	LOC11962	10.80477	2.142002	0.855947	2.502493	0.012332	0.028356	2.710432	5.474118	3.478657	29.01765	17.11289	7.034856
12520	PCP4L1	9.656391	2.141771	0.932191	2.297566	0.021587	0.045837	2.710432	1.642235	6.261582	15.62489	25.66933	6.029877
1256	TRPC7	181.6264	2.141567	0.214378	9.989672	1.69E-23	3.58E-22	57.46116	67.87906	76.53045	281.248	310.171	296.4689
1548	LOC10322	137.7768	2.141526	0.235568	9.090909	9.82E-20	1.69E-18	51.49821	49.81448	50.78839	252.2303	220.3284	202.0009
20959	CA6	1.489233	2.141497	2.422069	0.88416	0.37661	0.477703	0	1.642235	0	0	4.278221	3.014938
358	C1R	834.1107	2.141397	0.131575	16.27505	1.48E-59	1.10E-57	284.5953	320.7833	321.4279	1359.365	1232.128	1486.365
10345	LOC11962	13.17651	2.139645	0.770292	2.777705	0.005474	0.014068	2.168345	6.02153	6.261582	29.01765	23.53022	12.05975
15341	GRXCR2	5.216977	2.138627	1.206588	1.772458	0.076318	0.132255	1.084173	3.831883	0.695731	6.696381	14.97377	4.019918
833	JAG2	326.0075	2.138114	0.18025	11.86191	1.87E-32	5.96E-31	119.259	109.4824	134.9719	544.639	457.7697	589.9229
5796	LOC10323	36.41271	2.137339	0.49994	4.27519	1.91E-05	8.76E-05	8.131296	12.04306	20.17621	75.89232	62.03421	40.19918
21214	LOC11962	1.066199	2.137099	2.519761	0.848136	0.396362	0.496671	0	0.547412	0.695731	0	2.139111	3.014938
21215	LOC11961	1.066199	2.137099	2.519761	0.848136	0.396362	0.496671	0	0.547412	0.695731	0	2.139111	3.014938
16048	LOC11962	4.553706	2.137037	1.289531	1.657221	0.097475	0.161476	2.168345	2.737059	0	6.696381	10.69555	5.024897

1784	ATG9B	171.2851	2.136628	0.251454	8.497081	1.94E-17	2.90E-16	60.17159	48.17224	82.79203	247.7661	314.4493	274.3594
8972	AQP3	17.14236	2.136571	0.683692	3.125051	0.001778	0.005268	5.420864	4.379295	9.044508	35.71403	34.22577	14.06971
1434	KCNH3	198.4357	2.136244	0.226552	9.429385	4.13E-21	7.65E-20	87.81799	55.28859	77.92191	325.8905	308.0319	335.6631
9878	TMC8	17.71125	2.136208	0.739897	2.887171	0.003887	0.010461	2.710432	3.284471	13.91463	29.01765	34.22577	23.11453
15187	LOC10322!	7.293164	2.135256	1.187641	1.797898	0.072193	0.126367	0	3.831883	4.174388	15.62489	17.11289	3.014938
3505	TLR1	53.59939	2.133634	0.364704	5.85032	4.91E-09	3.72E-08	21.68345	18.06459	19.48048	93.74933	96.25998	72.35852
17219	HRNR	4.203615	2.133538	1.456591	1.464748	0.14299	0.220761	0	0.547412	4.174388	8.928508	8.556443	3.014938
12413	KRT74	8.318784	2.13095	0.919355	2.317877	0.020456	0.043807	2.168345	2.189647	4.87012	13.39276	19.252	8.039836
1837	ANKRD22	135.3748	2.129531	0.255002	8.351029	6.77E-17	9.79E-16	60.17159	38.31883	52.87558	220.9806	211.772	228.1303
238	SEMA4B	2017.546	2.129201	0.113395	18.77683	1.17E-78	1.31E-76	809.3349	708.3509	736.7795	3156.228	3069.624	3624.961
15139	LOC11961!	4.7765	2.12857	1.177312	1.80799	0.070608	0.123992	0.542086	2.737059	2.087194	6.696381	8.556443	8.039836
19973	CUNH17or	1.539988	2.127021	2.057285	1.033897	0.301184	0.40089	1.084173	0.547412	0	4.464254	2.139111	1.004979
4761	SHD	77.1624	2.126749	0.439664	4.837214	1.32E-06	7.35E-06	27.6464	10.94824	48.00546	140.624	126.2075	109.5428
13287	VSIG10L2	7.706104	2.126125	0.988511	2.150836	0.031489	0.063	2.710432	1.094824	4.87012	20.08914	6.417332	11.05477
11004	ACKR3	13.20814	2.126099	0.808884	2.628434	0.008578	0.020724	1.626259	6.02153	6.957314	26.78552	27.80844	10.04979
19307	TM4SF5	2.42339	2.126035	1.875468	1.133602	0.256961	0.353842	0	0	2.782925	4.464254	4.278221	3.014938
969	DMTN	320.0982	2.124918	0.190773	11.13844	8.15E-29	2.24E-27	108.4173	147.2538	101.5768	506.6928	556.1688	500.4798
14294	KLF14	8.180151	2.12338	1.082008	1.962444	0.049711	0.092456	3.252518	2.189647	3.478657	15.62489	23.53022	1.004979
11313	FCGR3A	11.95157	2.122664	0.830762	2.555079	0.010616	0.024948	2.710432	4.379295	6.261582	8.928508	36.36488	13.06473
21249	LOC11961{	1.118729	2.121303	2.519564	0.841933	0.399826	0.500229	0	0.547412	0.695731	4.464254	0	1.004979
15253	CDCP2	7.024168	2.120854	1.18609	1.788105	0.073759	0.128557	0.542086	1.094824	6.261582	6.696381	23.53022	4.019918
14829	TRNAD-GU	5.509389	2.12085	1.136106	1.866772	0.061934	0.111033	2.168345	1.094824	2.782925	11.16063	12.83466	3.014938
466	LOC10323(	562.1104	2.119462	0.142044	14.92113	2.40E-50	1.37E-48	203.8245	227.1759	199.6749	837.0476	990.4082	914.5313
8178	LOC10324;	35.7218	2.118721	0.631808	3.353426	0.000798	0.002595	3.252518	9.306001	27.82925	60.26743	68.45154	45.22408
7721	LOC11962:	29.39356	2.118177	0.607621	3.486017	0.00049	0.001688	6.505036	14.23271	11.82743	42.41041	81.28621	20.09959
7739	LOC11962!	20.75675	2.117444	0.608261	3.481145	0.000499	0.001715	11.9259	5.474118	5.565851	37.94616	38.50399	25.12449
686	TSNARE1	397.2532	2.116619	0.164249	12.88663	5.35E-38	2.07E-36	137.1479	137.4004	172.5414	689.7272	624.6203	622.0823
15059	GPR34	5.732182	2.116543	1.160903	1.823187	0.068275	0.120532	0.542086	1.094824	4.87012	11.16063	10.69555	6.029877
3890	DLGAP3	84.24791	2.116065	0.387916	5.454962	4.90E-08	3.35E-07	29.81475	24.63353	39.65669	133.9276	201.0764	76.37844
3975	CFAP52	44.80132	2.115189	0.392209	5.393011	6.93E-08	4.63E-07	18.97302	15.32753	16.00182	64.73168	83.42532	70.34856
160	DEPTOR	2202.059	2.114379	0.097743	21.63212	8.96E-104	1.49E-101	823.4292	863.8158	792.438	3448.636	3544.506	3739.529
1002	RHOU	240.1347	2.112847	0.192432	10.97973	4.78E-28	1.27E-26	76.43418	95.24966	99.48959	397.3186	374.3444	397.9719
148	SLC1A1	2489.724	2.112841	0.09505	22.22872	1.81E-109	3.26E-107	944.8565	941.5483	920.4526	3861.58	3993.72	4276.188
776	SLC22A18	1205.62	2.112392	0.172003	12.28111	1.14E-34	3.92E-33	531.7867	340.4901	488.4034	1899.54	1711.289	2262.209
2264	LZTS1	89.57984	2.111599	0.282429	7.47657	7.63E-14	8.96E-13	33.60935	31.74989	36.17803	131.6955	145.4595	158.7868
529	SGK2	901.4452	2.111514	0.148037	14.26347	3.70E-46	1.86E-44	401.1439	289.0334	326.9937	1455.347	1358.335	1577.818
16332	CCDC83	4.478278	2.111016	1.319858	1.599426	0.109726	0.17861	1.626259	0.547412	2.782925	15.62489	4.278221	2.009959
447	PDE1B	646.5758	2.110089	0.139419	15.13487	9.54E-52	5.67E-50	252.0702	226.0811	253.2462	1058.028	943.3478	1146.682
9004	ADPRHL1	16.33176	2.109978	0.677403	3.114804	0.001841	0.005435	4.878777	7.116354	6.261582	17.85702	42.78221	19.09461
16906	LOC10323(	7.328723	2.109165	1.401361	1.505083	0.132303	0.208048	0	0	8.348776	15.62489	14.97377	5.024897
19558	TTPA	2.444915	2.109025	1.935817	1.089475	0.275944	0.375025	0	0	2.782925	4.464254	6.417332	1.004979
4107	LRRC4B	80.88177	2.108845	0.398566	5.291074	1.22E-07	7.87E-07	19.51511	20.80165	51.48412	122.767	156.1551	114.5677
16354	CLRN3	4.684375	2.107398	1.320762	1.595593	0.11058	0.179758	1.626259	2.737059	0.695731	15.62489	6.417332	1.004979
2609	CYTH4	78.51867	2.106884	0.302555	6.963645	3.32E-12	3.38E-11	27.10432	29.56024	32.69937	113.8385	126.2075	141.7021
17721	LOC11962(	2.627712	2.105663	1.524565	1.381156	0.167231	0.250879	1.084173	1.094824	0.695731	4.464254	6.417332	2.009959
5662	CNGB1	31.64473	2.105631	0.485044	4.341117	1.42E-05	6.66E-05	13.01007	9.853413	12.52316	66.96381	51.33866	36.17926
13560	LOC11962(	8.387442	2.105559	1.004013	2.097142	0.035981	0.070542	1.626259	2.189647	5.565851	13.39276	23.53022	4.019918
1393	NR3C2	290.807	2.105208	0.220396	9.551921	1.27E-21	2.43E-20	104.0806	143.4219	81.40057	439.729	455.6306	520.5794
1615	GCNT4	182.2061	2.105179	0.235509	8.938839	3.93E-19	6.47E-18	55.29281	62.40495	89.05362	301.3371	303.7537	281.3943
10667	KIAA2012	13.16292	2.105065	0.779689	2.699878	0.006936	0.017288	7.589209	2.189647	4.87012	22.32127	29.94755	12.05975
12504	VWDE	10.52586	2.104108	0.914662	2.300422	0.021424	0.045545	1.626259	3.284471	6.957314	11.16063	32.08666	8.039836
10012	LOC11962{	18.7986	2.103995	0.736139	2.858148	0.004261	0.011315	3.252518	6.02153	11.82743	35.71403	44.92132	11.05477
9440	NECTIN4	22.78847	2.103665	0.701456	2.998997	0.002709	0.007628	3.252518	7.116354	15.30609	44.64254	51.33866	15.07469
11086	LRRC71	13.6687	2.103287	0.806365	2.608357	0.009098	0.021815	5.96295	4.926706	4.174388	24.5534	36.36488	6.029877
18358	LOC10323!	2.205566	2.102883	1.653115	1.272073	0.203347	0.294476	0.542086	0.547412	1.391463	4.464254	4.278221	2.009959
18913	LOC11962:	2.206453	2.102785	1.750148	1.20149	0.229561	0.322682	0	1.094824	1.391463	4.464254	4.278221	2.009959
20869	EFCAB1	1.103227	2.100822	2.345997	0.895492	0.370524	0.471942	0	0.547412	0.695731	2.232127	2.139111	1.004979
20870	LOC10321;	1.103227	2.100822	2.345997	0.895492	0.370524	0.471942	0	0.547412	0.695731	2.232127	2.139111	1.004979
20871	LOC11962;	1.103227	2.100822	2.345997	0.895492	0.370524	0.471942	0	0.547412	0.695731	2.232127	2.139111	1.004979
20872	LOC10322!	1.103227	2.100822	2.345997	0.895492	0.370524	0.471942	0	0.547412	0.695731	2.232127	2.139111	1.004979
11203	LOC10324{	17.24517	2.100609	0.81358	2.581935	0.009825	0.023313	2.710432	13.13788	3.478657	51.33892	10.69555	22.10955
11478	LOC11961!	9.653195	2.100505	0.833133	2.521214	0.011695	0.027088	2.168345	3.284471	5.565851	17.85702	14.97377	14.06971
329	LOC11962:	1070.693	2.100317	0.125292	16.76343	4.52E-63	3.65E-61	382.713	447.2355	386.1309	1707.577	1617.168	1883.332
7599	ABCC11	24.52485	2.100011	0.595496	3.52649	0.000421	0.001473	9.757555	6.02153	11.82743	31.24978	64.17332	24.11951
6119	DGKI	62.75581	2.099916	0.510834	4.110763	3.94E-05	0.000171	11.38381	18.612	41.04815	116.0706	141.1813	48.23901
16156	DEUP1	4.788545	2.099605	1.283547	1.635784	0.101885	0.167653	0.542086	2.737059	2.087194	2.232127	17.11289	4.019918
19735	LOC11962{	2.429412	2.099522	1.97519	1.062947	0.287806	0.387703	0	0	2.782925	2.232127	8.556443	1.004979
16985	LOC10323!	7.223661	2.097583	1.403083	1.494981	0.134919	0.211195	0	0	8.348776	6.696381	19.252	9.044815
17054	LOC11962(	3.698881	2.096822	1.41048	1.486602	0.13712	0.213753	0	2.189647	2.087194	4.464254	6.417332	7.034856
16189	LOC10321;	5.78702	2.096639	1.287532	1.628417	0.103437	0.16986	1.084173	0	5.565851	15.62489	6.417332	6.029877
13806	LOC11961{	7.175665	2.096411	1.024448	2.046382	0.040719	0.078409	4.878777	1.094824	2.087194	6.696381	19.252	9.044815
4335	LOC10323;	41.31306	2.096076	0.408662	5.129114	2.91E-07	1.79E-06	13.01007	15.87494	18.08902	64.73168	74.86887	61.30375
7524	LOC10323(	21.80251	2.096047	0.590342	3.550564	0.000384	0.001358	10.84173	6.568942	6.957314	49.10679	34.22577	23.11453
8910	KIRREL2	19.29955	2.09556	0.667016	3.141693	0.00168	0.005012	8.131296	3.284471	10.43597	26.78552	47.06043	20.09959

16816	LOC10323!	3.752464	2.095104	1.375811	1.522814	0.127805	0.202052	1.084173	1.642235	1.391463	6.696381	10.69555	1.004979
4493	LOC11962:	76.88477	2.095017	0.417789	5.014538	5.32E-07	3.15E-06	29.27266	12.59047	45.91827	120.5349	145.4595	107.5328
18519	LOC11962!	2.190063	2.094969	1.678306	1.248264	0.211934	0.304243	0.542086	0.547412	1.391463	2.232127	6.417332	2.009959
10954	SH2D3A	12.07193	2.094279	0.793686	2.638676	0.008323	0.0202	3.794605	5.474118	4.174388	17.85702	32.08666	9.044815
14556	RASL11B	6.829705	2.094245	1.093663	1.91489	0.055507	0.101377	1.084173	4.379295	2.087194	11.16063	19.252	3.014938
13257	UCN3	7.417677	2.094174	0.970989	2.156744	0.031026	0.062217	2.710432	2.189647	3.478657	6.696381	21.39111	8.039836
17124	GDF5	6.636222	2.094143	1.417378	1.477477	0.139548	0.216648	0	0	7.653045	11.16063	14.97377	6.029877
13362	RFX6	7.931157	2.093425	0.980694	2.134636	0.032791	0.06524	2.168345	3.831883	2.782925	13.39276	21.39111	4.019918
15854	LOC10324:	4.139889	2.092712	1.237932	1.69049	0.090934	0.152484	2.168345	1.094824	1.391463	4.464254	10.69555	5.024897
10014	KCNQ4	14.56767	2.092487	0.732243	2.857642	0.004268	0.011331	5.96295	6.02153	4.174388	31.24978	29.94755	10.04979
18355	LOC10324(	2.578376	2.092194	1.644006	1.27262	0.203153	0.294243	1.084173	0.547412	1.391463	0	6.417332	6.029877
222	HSPB8	1517.945	2.09215	0.107922	19.38569	1.02E-83	1.22E-81	560.5173	597.2263	574.6741	2339.269	2380.83	2655.156
11150	DSC3	13.66172	2.09168	0.806862	2.592363	0.009532	0.022727	2.710432	7.663765	4.87012	22.32127	36.36488	8.039836
16345	LOC11962:	4.357774	2.091587	1.310073	1.596542	0.110368	0.179512	2.710432	2.189647	0	8.928508	4.278221	8.039836
17037	TNFRSF11E	3.053941	2.090878	1.405545	1.487593	0.136858	0.213557	1.084173	1.642235	0.695731	4.464254	6.417332	4.019918
491	CRACDL	612.2539	2.090811	0.142095	14.71419	5.23E-49	2.83E-47	239.0601	253.4517	205.9365	997.7608	924.0958	1053.218
14953	UNC13C	6.628743	2.089382	1.132628	1.844721	0.065078	0.115703	1.626259	1.642235	4.174388	8.928508	21.39111	2.009959
12580	LOC10321{	11.83178	2.089344	0.913189	2.287964	0.02214	0.046787	1.084173	2.189647	10.43597	13.39276	27.80844	16.07967
17737	LOC10321{	5.925653	2.089341	1.516022	1.378173	0.16815	0.252037	0	0	6.957314	13.39276	2.139111	13.06473
16365	LOC10324:	3.717743	2.087926	1.309316	1.594669	0.110786	0.179973	1.626259	0.547412	2.087194	4.464254	8.556443	5.024897
20420	DEFB123	1.543183	2.086419	2.17693	0.958423	0.33785	0.439828	1.084173	0	0.695731	4.464254	0	3.014938
6467	LOC11962(	24.11905	2.086197	0.527831	3.952398	7.74E-05	0.000318	9.215468	10.40082	7.653045	44.64254	40.6431	32.15934
18116	HHLA2	3.315703	2.085997	1.582547	1.318127	0.187461	0.275082	0	1.642235	2.087194	4.464254	10.69555	1.004979
21365	CUNH3orf!	1.087724	2.085445	2.538073	0.821665	0.411268	0.511715	0	0.547412	0.695731	0	4.278221	1.004979
21367	LOC11961!	1.087724	2.085445	2.538073	0.821665	0.411268	0.511715	0	0.547412	0.695731	0	4.278221	1.004979
21368	LOC10323:	1.087724	2.085445	2.538073	0.821665	0.411268	0.511715	0	0.547412	0.695731	0	4.278221	1.004979
10875	LOC10321!	12.30921	2.084164	0.785019	2.654921	0.007933	0.01939	7.589209	2.737059	3.478657	22.32127	25.66933	12.05975
20749	LOC11962(	1.954961	2.083939	2.288418	0.910646	0.362482	0.464436	2.168345	0	0	0	8.556443	1.004979
12383	LOC10323!	13.09136	2.083047	0.895551	2.325994	0.020019	0.042978	3.794605	5.474118	5.565851	4.464254	49.19955	10.04979
293	LTBP3	1597.578	2.082741	0.11827	17.61004	2.06E-69	1.87E-67	594.6687	594.4892	644.943	2482.125	2365.856	2903.386
248	CASTOR2	1285.914	2.081826	0.112296	18.53875	1.01E-76	1.08E-74	497.6353	478.9853	499.5351	2109.36	1905.948	2224.02
10305	LOC11962:	14.83227	2.080883	0.74654	2.787368	0.005314	0.013709	7.047123	2.737059	6.957314	31.24978	29.94755	11.05477
16549	GPR88	4.622199	2.080716	1.328778	1.565888	0.117375	0.188555	0	3.831883	1.391463	8.928508	8.556443	5.024897
5178	SP7	44.08987	2.080524	0.453196	4.590779	4.42E-06	2.27E-05	13.01007	16.42235	20.87194	58.0353	106.9555	49.24399
14095	LOC10323¿	9.200301	2.08052	1.042921	1.994896	0.046054	0.086864	1.084173	3.831883	5.565851	4.464254	34.22577	6.029877
11388	ASIC4	18.68217	2.080195	0.819079	2.539675	0.011096	0.025902	3.252518	2.189647	16.00182	37.94616	40.6431	12.05975
8272	SELENBP1	17.13393	2.078435	0.625682	3.321871	0.000894	0.002874	5.420864	6.02153	8.348776	33.4819	21.39111	28.13943
7997	RIPPLY3	20.63726	2.078203	0.608863	3.413253	0.000642	0.002134	8.131296	10.94824	4.174388	37.94616	38.50399	24.11951
14863	TMEM215	5.914716	2.077921	1.116982	1.8603	0.062843	0.112406	1.626259	1.642235	3.478657	4.464254	19.252	5.024897
18541	LOC11962:	2.685213	2.077817	1.670004	1.244199	0.213426	0.306011	0	1.642235	1.391463	8.928508	2.139111	2.009959
19548	LOC11962!	1.528568	2.077646	1.904382	1.090982	0.275281	0.374377	0.542086	0.547412	0.695731	2.232127	2.139111	3.014938
1078	LYNX1	293.1078	2.077413	0.195642	10.61843	2.45E-26	6.03E-25	98.11763	140.1374	98.09812	466.5145	483.439	472.3404
16713	OPRM1	4.882186	2.077198	1.349149	1.539636	0.123649	0.196683	0	2.737059	2.782925	8.928508	12.83466	2.009959
8555	LOC11962:	15.72244	2.07678	0.641065	3.239577	0.001197	0.00372	6.505036	5.474118	6.261582	24.5534	21.39111	30.14938
5659	LOC11962(	66.09883	2.07641	0.478165	4.342453	1.41E-05	6.62E-05	24.93597	9.306001	41.74388	129.4634	119.7902	71.35354
20282	SERPINB11	1.767917	2.075938	2.114807	0.981621	0.326287	0.427686	0	0	2.087194	2.232127	4.278221	2.009959
14469	PI15	6.661002	2.075418	1.075111	1.930422	0.053555	0.0984	1.084173	1.094824	5.565851	15.62489	8.556443	8.039836
13226	CFAP77	6.995591	2.07527	0.959073	2.163829	0.030478	0.061261	3.252518	3.284471	1.391463	11.16063	12.83466	10.04979
16008	NRSN2	4.878636	2.075211	1.247892	1.662974	0.096318	0.159958	2.168345	0.547412	2.782925	8.928508	12.83466	2.009959
744	DPF3	368.5374	2.075077	0.165792	12.51613	6.09E-36	2.18E-34	139.8583	157.1072	127.3188	611.6028	541.195	634.142
18058	PI16	2.684325	2.073987	1.556184	1.332739	0.182618	0.26885	0.542086	1.094824	1.391463	8.928508	2.139111	2.009959
152	LOC10322:	5804.261	2.073897	0.094517	21.94203	1.03E-106	1.81E-104	2271.884	2242.199	2172.073	9426.272	8629.172	10083.96
16043	LOC10323(	4.638399	2.071296	1.24944	1.657779	0.097362	0.16134	3.252518	0.547412	1.391463	8.928508	10.69555	3.014938
1081	FA2H	207.1006	2.070244	0.195462	10.59153	3.26E-26	8.02E-25	85.64965	72.25836	81.40057	325.8905	333.7013	343.703
14924	LOC10324:	5.504939	2.069741	1.118875	1.849841	0.064336	0.114606	2.168345	3.284471	0.695731	11.16063	10.69555	5.024897
13942	LOC10321{	6.38976	2.069728	1.022138	2.024902	0.042877	0.08176	2.168345	1.094824	4.174388	11.16063	10.69555	9.044815
2782	TNFRSF18	178.1066	2.069518	0.307624	6.727425	1.73E-11	1.65E-10	80.77087	38.86624	86.96642	314.7299	209.6328	337.6731
12844	LOC10322:	11.15718	2.069353	0.92804	2.229811	0.02576	0.053319	1.084173	2.737059	9.044508	26.78552	19.252	8.039836
1211	LOC10322:	454.9983	2.069266	0.204489	10.1192	4.54E-24	9.97E-23	166.9626	127.547	231.6785	725.4413	782.9145	695.4458
21660	FGFBP1	1.687838	2.068643	2.643392	0.782571	0.433879	0.532533	0	0	2.087194	0	0	8.039836
14150	LOC10324:	8.236336	2.068333	1.041819	1.985309	0.04711	0.08851	1.084173	2.737059	5.565851	15.62489	21.39111	3.014938
13832	PALMD	7.512737	2.068309	1.012069	2.043645	0.040989	0.07878	3.252518	0.547412	4.87012	13.39276	14.97377	8.039836
7378	MUSK	23.98241	2.067989	0.57316	3.608047	0.000309	0.001112	9.757555	7.663765	10.43597	53.57105	19.252	43.21412
17284	HAO1	5.887571	2.067057	1.422827	1.452781	0.146284	0.225004	0	0.547412	6.261582	20.08914	6.417332	2.009959
7226	LOC10324:	25.98457	2.066551	0.564749	3.659241	0.000253	0.000931	5.420864	8.211177	16.69755	46.87467	38.50399	40.19918
15291	FPR2	5.32303	2.066055	1.160216	1.78075	0.074953	0.130314	0.542086	2.737059	2.782925	11.16063	10.69555	4.019918
14380	DMC1	6.220385	2.065763	1.060391	1.948114	0.051401	0.095028	3.252518	1.642235	2.087194	15.62489	10.69555	4.019918
14013	GPX2	6.376033	2.065059	1.027097	2.010579	0.04437	0.084177	1.084173	2.189647	4.174388	8.928508	12.83466	9.044815
3492	LOC11962:	156.3018	2.064996	0.352141	5.864119	4.52E-09	3.44E-08	35.23561	82.11177	62.61582	249.9982	355.0924	152.7569
20995	LOC11962¿	1.804945	2.063548	2.347175	0.879162	0.379313	0.480307	0	0	2.087194	4.464254	4.278221	0
17820	HOXD10	3.3344	2.063396	1.501857	1.373897	0.169474	0.252832	0	1.094824	2.782925	6.696381	6.417332	3.014938
6186	LOC11962:	30.28672	2.062756	0.505239	4.082735	4.45E-05	0.000191	8.131296	9.306001	18.08902	46.87467	47.06043	52.25893
8280	MYO1G	21.04116	2.062546	0.621179	3.320374	0.000899	0.002886	5.96295	7.663765	10.43597	40.17829	44.92132	17.08465
4305	NOXO1	45.28261	2.060838	0.399969	5.152499	2.57E-07	1.59E-06	13.55216	19.15941	20.17621	60.26743	79.14709	79.39338

19459	SLC36A3	2.415745	2.060372	1.858532	1.108602	0.267602	0.3656	0	2.737059	0	4.464254	4.278221	3.014938
14771	RGMA	8.174325	2.060261	1.096923	1.878219	0.060351	0.108621	1.084173	2.737059	5.565851	6.696381	29.94755	3.014938
12814	NLRP6	12.30488	2.059892	0.921248	2.23598	0.025353	0.0526	2.168345	1.642235	10.43597	29.01765	23.53022	7.034856
19502	LOC10324:	1.972239	2.059658	1.87071	1.101003	0.270895	0.369299	1.084173	1.094824	0	2.232127	6.417332	1.004979
11266	ALDH8A1	12.50163	2.059296	0.802471	2.566195	0.010282	0.024263	5.420864	2.737059	6.261582	11.16063	36.36488	13.06473
854	NECTIN1	309.2995	2.059008	0.17511	11.7584	6.39E-32	1.99E-30	109.5014	125.3573	125.2316	524.5498	438.5177	532.6391
11530	LOC10323:	11.29578	2.05819	0.820738	2.507731	0.012151	0.028017	2.710432	2.737059	7.653045	20.08914	23.53022	11.05477
2171	INSC	130.8756	2.056017	0.268441	7.6591	1.87E-14	2.29E-13	57.46116	41.6033	52.17985	249.9982	218.1893	165.8216
20978	TAS2R10	1.752414	2.05564	2.331005	0.881869	0.377848	0.478839	0	0	2.087194	0	6.417332	2.009959
2968	LOC11962(	71.28406	2.055327	0.317789	6.467591	9.96E-11	8.92E-10	28.18849	27.37059	27.82925	93.74933	121.9293	128.6374
2077	CELF5	127.7524	2.055053	0.262005	7.843574	4.38E-15	5.61E-14	40.65648	55.28859	52.17985	198.6593	243.8586	175.8714
20587	LOC11962:	1.513952	2.054455	2.187566	0.939151	0.347653	0.448942	0	1.094824	0.695731	0	4.278221	3.014938
14904	LOC11962:	6.328637	2.054252	1.108065	1.85391	0.063752	0.113718	1.626259	1.642235	4.174388	2.232127	19.252	9.044815
16715	LOC11962(	3.518617	2.053713	1.334014	1.539499	0.123683	0.196704	2.168345	0.547412	1.391463	6.696381	4.278221	6.029877
455	LOC10322!	767.8001	2.05306	0.136402	15.05154	3.37E-51	1.97E-49	303.0263	306.5506	286.6413	1241.063	1086.668	1382.852
19270	CD74	2.246676	2.052546	1.79871	1.141121	0.25382	0.350135	0	0.547412	2.087194	6.696381	2.139111	2.009959
12055	HAPLN2	15.62608	2.051224	0.856143	2.39589	0.01658	0.036564	2.710432	1.642235	13.91463	33.4819	29.94755	12.05975
21117	PCDH8	1.789442	2.050918	2.379348	0.861966	0.388706	0.489334	0	0	2.087194	2.232127	6.417332	0
21118	TMEM204	1.789442	2.050918	2.379348	0.861966	0.388706	0.489334	0	0	2.087194	2.232127	6.417332	0
12566	NXPE2	9.052878	2.049628	0.894978	2.290144	0.022013	0.046571	3.252518	4.926706	2.087194	15.62489	21.39111	7.034856
6522	PRAM1	31.46867	2.049561	0.521373	3.931085	8.46E-05	0.000345	7.589209	13.6853	15.30609	40.17829	74.86887	37.18424
10899	SAG	12.91291	2.049282	0.773058	2.650879	0.008028	0.019581	3.252518	5.474118	6.261582	35.71403	10.69555	16.07967
15902	SMIM24	5.585324	2.04879	1.216512	1.68415	0.092153	0.154064	1.084173	0.547412	4.87012	11.16063	12.83466	3.014938
1864	CSF1R	129.0187	2.048698	0.247672	8.271805	1.32E-16	1.88E-15	42.82482	52.00412	56.35424	185.2665	224.6066	213.0556
10046	LOC11961!	12.57044	2.047944	0.718161	2.851649	0.004349	0.01151	4.336691	6.02153	4.174388	24.5534	19.252	17.08465
5146	LOC11962(	35.3434	2.047654	0.444566	4.605961	4.11E-06	2.12E-05	11.9259	16.96977	12.52316	60.26743	47.06043	63.31371
5182	SRGAP3	44.83123	2.047221	0.446199	4.588131	4.47E-06	2.29E-05	14.09425	13.6853	24.3506	93.74933	74.86887	48.23901
8711	LOC11961{	16.79183	2.047221	0.639932	3.199122	0.001378	0.004207	7.589209	5.474118	6.261582	31.24978	32.08666	18.08963
12127	CCDC144A	12.1108	2.046847	0.860208	2.37948	0.017337	0.038007	5.420864	4.926706	3.478657	11.16063	40.6431	7.034856
11105	SORBS2	16.23898	2.045813	0.785662	2.603936	0.009216	0.022063	2.168345	8.211177	8.348776	29.01765	40.6431	9.044815
15480	TMEM100	6.676658	2.045395	1.16994	1.74829	0.080414	0.138105	0	4.926706	2.782925	15.62489	10.69555	6.029877
7571	ERICH6	26.68578	2.044175	0.578326	3.534644	0.000408	0.001434	7.047123	12.04306	11.82743	33.4819	70.59065	25.12449
268	SLC9A3R1	4074.927	2.044098	0.113457	18.01657	1.44E-72	1.43E-70	1483.148	1826.166	1462.427	6497.722	6271.872	6908.229
3773	LOC10323(	67.47627	2.043921	0.366841	5.571686	2.52E-08	1.78E-07	16.80468	29.01283	33.39511	91.51721	132.6249	101.5029
19216	LOC10324(	2.231173	2.043722	1.775345	1.151169	0.249663	0.34538	0	0.547412	2.087194	4.464254	4.278221	2.009959
8404	LOC11962!	24.42501	2.043412	0.62262	3.281955	0.001031	0.003261	8.131296	3.831883	16.69755	49.10679	40.6431	28.13943
13561	LOC10324:	6.769541	2.04317	0.974315	2.097031	0.035991	0.070556	3.252518	3.284471	1.391463	8.928508	10.69555	13.06473
2048	ADGRF5	104.3027	2.043129	0.259055	7.886856	3.10E-15	4.02E-14	39.03022	39.41365	43.83108	171.8738	166.8506	164.8166
14023	LOC11962{	7.280665	2.04284	1.017071	2.008553	0.044585	0.084524	3.252518	0.547412	4.87012	13.39276	8.556443	13.06473
17330	SSTR4	5.19507	2.042763	1.414981	1.443667	0.148833	0.228316	0	0.547412	5.565851	15.62489	6.417332	3.014938
17171	LOC10323:	4.838872	2.04257	1.387867	1.471733	0.141093	0.21844	0	2.189647	3.478657	2.232127	17.11289	4.019918
4188	LOC10323!	55.53846	2.042221	0.390179	5.234063	1.66E-07	1.05E-06	14.63633	21.89647	28.52499	109.3742	83.42532	75.37346
2381	TTYH1	118.7669	2.041987	0.279644	7.302087	2.83E-13	3.16E-12	42.82482	35.03436	61.92009	198.6593	188.2417	185.9212
6704	ARHGAP45	48.60017	2.041978	0.529989	3.85287	0.000117	0.000463	22.22554	5.474118	29.22072	95.98146	83.42532	55.27387
14042	NGFR	7.272593	2.041737	1.017645	2.006336	0.04482	0.084856	2.168345	2.737059	3.478657	6.696381	23.53022	5.024897
2849	LPAR6	70.87373	2.041371	0.308561	6.615784	3.70E-11	3.45E-10	27.10432	26.82318	29.22072	118.3027	111.2338	112.5577
16211	LOC11962:	4.320318	2.041191	1.255614	1.625653	0.104024	0.170596	1.084173	2.737059	1.391463	2.232127	6.417332	12.05975
413	CD82	2174.277	2.040907	0.131372	15.5353	2.00E-54	1.29E-52	923.7152	733.5318	896.102	3408.458	3125.241	3958.614
12232	LOC11961!	9.05341	2.040478	0.864538	2.360194	0.018265	0.039698	4.878777	2.737059	2.782925	15.62489	19.252	9.044815
12607	DRD1	8.171558	2.039257	0.893879	2.281357	0.022527	0.047505	2.168345	3.831883	3.478657	13.39276	17.11289	9.044815
1169	SOX13	243.8826	2.038231	0.198484	10.26901	9.72E-25	2.21E-23	84.56547	107.2927	95.3152	415.1756	335.8404	425.1063
8455	ZBTB32	20.17681	2.037755	0.623741	3.266992	0.001087	0.003418	4.336691	11.49565	7.653045	44.64254	27.80844	25.12449
17483	LOC11961{	3.912064	2.035542	1.43894	1.414612	0.157182	0.239014	3.252518	0	1.391463	2.232127	8.556443	8.039836
17335	LOC10322:	3.082743	2.034236	1.410506	1.442203	0.149245	0.228883	0.542086	1.094824	2.087194	4.464254	4.278221	6.029877
17449	CA9	3.116576	2.034034	1.433949	1.418484	0.15605	0.237754	0.542086	1.642235	1.391463	6.696381	6.417332	2.009959
8015	ATP1A2	42.25978	2.033901	0.596819	3.407901	0.000655	0.002171	5.96295	9.306001	34.78657	60.26743	91.98176	51.25395
15879	QPRT	4.649391	2.033255	1.204459	1.688107	0.091391	0.153009	2.168345	1.094824	2.087194	6.696381	12.83466	3.014938
16171	FGL1	4.581192	2.032573	1.245241	1.632273	0.102622	0.16871	0.542086	2.189647	2.782925	2.232127	10.69555	9.044815
6387	ZNF415	32.02945	2.032512	0.510253	3.983341	6.80E-05	0.000283	9.215468	14.78012	13.2189	69.19594	55.61688	30.14938
13009	METTL21C	9.783564	2.032243	0.922708	2.202476	0.027632	0.056468	4.336691	2.737059	4.174388	15.62489	27.80844	4.019918
18925	LOC10321!	3.582305	2.031693	1.693727	1.19954	0.230318	0.323564	2.710432	0	1.391463	6.696381	10.69555	0
1836	SMPDL3A	309.0043	2.031658	0.243092	8.357576	6.40E-17	9.27E-16	90.52842	174.077	98.79385	544.639	487.7172	458.2706
1651	TRANK1	141.599	2.031036	0.229534	8.848535	8.87E-19	1.43E-17	52.04029	58.57306	57.04997	216.5163	218.1893	247.2249
13713	LOC10323:	14.64357	2.030356	0.982462	2.066601	0.038772	0.075166	0	5.474118	11.82743	17.85702	40.6431	12.05975
10411	ST8SIA2	14.72273	2.030019	0.734524	2.763722	0.005715	0.014593	3.794605	4.379295	9.044508	31.24978	27.80844	12.05975
16764	ENPP7	9.290533	2.028633	1.325147	1.530874	0.125801	0.199511	0	0	11.1317	11.16063	21.39111	12.05975
322	SERPINA6	922.0833	2.028508	0.12008	16.89291	5.07E-64	4.19E-62	357.2349	374.9771	358.3017	1495.525	1362.613	1583.848
13364	IL3RA	9.765895	2.02719	0.950037	2.1338	0.032859	0.065367	1.084173	2.189647	8.348776	13.39276	23.53022	10.04979
17848	LOC11962(	5.828755	2.026148	1.479846	1.369161	0.170949	0.254632	0	0	6.957314	11.16063	12.83466	4.019918
18590	SELENOP	2.641899	2.026117	1.638741	1.236386	0.216315	0.309331	0	1.094824	2.087194	2.232127	6.417332	4.019918
9099	LOC10322:	22.80833	2.025387	0.656776	3.083835	0.002044	0.005971	4.336691	6.568942	16.00182	51.33892	38.50399	20.09959
15135	LOC10323!	6.686928	2.025205	1.11984	1.808477	0.070532	0.123892	1.084173	3.831883	2.782925	11.16063	19.252	2.009959
9473	FAM155A	21.40664	2.025162	0.676823	2.992159	0.00277	0.007774	7.047123	4.379295	13.91463	20.08914	59.8951	23.11453
18062	CUNH7orf:	3.734758	2.025065	1.520878	1.33151	0.183021	0.269356	0.542086	1.094824	2.782925	0	14.97377	3.014938

17707	LOC11961	3.084683	2.025042	1.463933	1.383289	0.166576	0.250095	1.084173	1.094824	1.391463	2.232127	10.69555	2.009959
10819	DUOX1	19.71877	2.024318	0.759272	2.666129	0.007673	0.018855	2.168345	7.116354	13.91463	42.41041	40.6431	12.05975
174	TM4SF1	44188.77	2.024282	0.095779	21.1349	3.80E-99	5.81E-97	16839.91	19376.19	16097.83	71349.94	72269.85	69198.87
239	NLGN1	3732.627	2.024226	0.108179	18.71178	3.97E-78	4.42E-76	1395.33	1673.985	1349.023	6008.886	5929.615	6038.922
240	RPS6KA3	18607.63	2.024181	0.108286	18.69294	5.65E-78	6.26E-76	6996.167	8418.646	6616.405	29234.17	29066.24	31314.16
7010	ATOH8	23.99729	2.023733	0.542647	3.729372	0.000192	0.000728	10.84173	6.02153	11.82743	35.71403	36.36488	43.21412
9365	LOC11962	15.93202	2.02357	0.671223	3.014752	0.002572	0.0073	3.794605	9.306001	5.565851	29.01765	27.80844	20.09959
15245	LOC10323	6.429188	2.023508	1.130997	1.789137	0.073593	0.128335	0.542086	2.189647	4.87012	6.696381	19.252	5.024897
18990	IL13RA2	1.976321	2.023228	1.704928	1.186694	0.235348	0.329458	0.542086	1.094824	0.695731	2.232127	4.278221	3.014938
18509	CD164L2	3.378399	2.023219	1.619314	1.249429	0.211508	0.303746	0.542086	3.284471	0	11.16063	4.278221	1.004979
318	ALDH1L1	1967.141	2.023205	0.11883	17.02597	5.27E-65	4.41E-63	837.5234	689.7389	805.6569	3046.853	2966.946	3456.124
212	SLC17A3	1706.968	2.023193	0.102233	19.79004	3.63E-87	4.55E-85	703.6281	664.5579	654.6832	2584.803	2800.096	2834.042
2205	LOC10322	124.7094	2.023108	0.266331	7.596205	3.05E-14	3.68E-13	44.45108	62.40495	41.04815	196.4272	181.8244	222.1005
14673	LOC11962	8.434861	2.022703	1.068285	1.893411	0.058303	0.105636	4.336691	5.474118	0	22.32127	6.417332	12.05975
12382	LOC10322	12.25668	2.022553	0.869541	2.326001	0.020018	0.042978	6.505036	4.379295	3.478657	4.464254	40.6431	14.06971
20820	MSTN	1.535478	2.021797	2.242	0.901783	0.367172	0.468841	0	1.094824	0.695731	0	6.417332	1.004979
17542	GNA14	4.391485	2.02175	1.434442	1.409433	0.158707	0.240521	0	0.547412	4.87012	4.464254	6.417332	10.04979
14712	LOC10322	6.203401	2.021712	1.070622	1.888353	0.058979	0.106576	0.542086	2.737059	4.174388	11.16063	8.556443	10.04979
10456	NOS2	14.04662	2.021294	0.733753	2.754734	0.005874	0.014935	3.252518	4.926706	8.348776	29.01765	25.66933	13.06473
7181	LOC10321	21.53225	2.020577	0.5504	3.671106	0.000242	0.000894	9.757555	8.211177	7.653045	29.01765	36.36488	38.18922
11005	LOC11962	14.9598	2.020432	0.768726	2.628287	0.008582	0.020731	6.505036	6.568942	4.174388	31.24978	34.22577	7.034856
11666	IZUMO1R	12.00404	2.019565	0.814945	2.47816	0.013206	0.030095	1.626259	4.926706	7.653045	20.08914	25.66933	12.05975
20353	LOC11962	1.53459	2.019276	2.085138	0.968413	0.332838	0.434709	0.542086	0.547412	0.695731	0	6.417332	1.004979
20354	SPATA4	1.53459	2.019276	2.085138	0.968413	0.332838	0.434709	0.542086	0.547412	0.695731	0	6.417332	1.004979
20355	LOC11962	1.53459	2.019276	2.085138	0.968413	0.332838	0.434709	0.542086	0.547412	0.695731	0	6.417332	1.004979
13962	LOC10324	7.399546	2.018766	0.999747	2.019276	0.043458	0.082749	2.168345	1.642235	4.87012	17.85702	12.83466	5.024897
9894	LOC10324	13.28977	2.017088	0.698914	2.886031	0.003901	0.010483	5.96295	5.474118	4.174388	26.78552	19.252	18.08963
16749	HOXD11	4.055929	2.016134	1.31483	1.53338	0.125182	0.198697	2.168345	1.094824	1.391463	13.39276	4.278221	2.009959
8879	LRFN2	20.93854	2.015222	0.640067	3.148456	0.001641	0.004915	4.336691	6.02153	14.61036	40.17829	36.36488	24.11951
2080	LOC10323	245.0508	2.014632	0.257115	7.835523	4.67E-15	5.97E-14	113.2961	74.44801	105.0554	444.1933	260.9715	472.3404
2478	MCHR1	130.5035	2.014609	0.281556	7.155277	8.35E-13	8.96E-12	46.61943	68.42648	39.65669	247.7661	177.5462	203.0059
10268	CAPN11	17.75604	2.014226	0.720314	2.796315	0.005169	0.013383	5.96295	3.284471	11.82743	22.32127	47.06043	16.07967
12092	MUCL3	10.74516	2.013694	0.843068	2.388531	0.016916	0.037191	3.794605	3.831883	4.87012	24.5534	21.39111	6.029877
4976	TMEM229	37.56234	2.013463	0.428397	4.699992	2.60E-06	1.39E-05	17.88885	11.49565	15.30609	62.49956	59.8951	58.28881
18395	TGM5	3.173189	2.013067	1.590824	1.265424	0.205719	0.297312	0	1.642235	2.087194	11.16063	2.139111	2.009959
1500	LOC10324	162.4793	2.01244	0.217644	9.246487	2.32E-20	4.11E-19	69.92914	64.59459	58.44144	254.4625	280.2235	247.2249
6526	MYO15A	31.97196	2.012215	0.512251	3.928183	8.56E-05	0.000349	14.09425	7.116354	16.69755	53.57105	64.17332	36.17926
9627	CPLX4	20.09017	2.01191	0.681171	2.953607	0.003141	0.008673	3.794605	4.926706	15.30609	40.17829	34.22577	22.10955
12697	LOC11961	8.729875	2.01139	0.890107	2.259719	0.023839	0.049914	2.710432	2.737059	4.87012	22.32127	10.69555	9.044815
3697	LOC11962	59.50262	2.010208	0.356195	5.643566	1.67E-08	1.20E-07	22.22554	22.44388	26.43779	73.66019	119.7902	92.45811
9514	LOC11962	19.90136	2.009675	0.674309	2.980348	0.002879	0.008045	4.336691	9.306001	9.740239	40.17829	42.78221	13.06473
5910	ESPN	38.21071	2.009101	0.477567	4.206948	2.59E-05	0.000116	13.55216	10.94824	20.87194	60.26743	83.42532	40.19918
15085	ZAR1L	7.982346	2.008541	1.10417	1.81905	0.068904	0.121432	5.96295	0	3.478657	11.16063	19.252	8.039836
16957	LOC11961	4.228193	2.008324	1.340346	1.498362	0.134039	0.210145	2.168345	2.737059	0	11.16063	4.278221	5.024897
9382	ZNF280A	19.89387	2.007593	0.666551	3.011911	0.002596	0.007356	4.336691	5.474118	13.91463	40.17829	36.36488	19.09461
13498	CRYBG2	10.27451	2.006041	0.95191	2.107384	0.035084	0.0691	1.626259	1.642235	9.044508	15.62489	25.66933	8.039836
15559	CD300A	7.788839	2.004542	1.155653	1.734554	0.08282	0.141511	0	4.379295	4.87012	8.928508	23.53022	5.024897
10772	CCR10	16.92991	2.004123	0.74942	2.674234	0.00749	0.018485	3.252518	3.831883	13.2189	24.5534	40.6431	16.07967
15123	TEX38	5.731778	2.00186	1.106235	1.809615	0.070355	0.123656	2.710432	3.284471	0.695731	6.696381	14.97377	6.029877
294	PDE2A	1356.869	2.001749	0.113942	17.56812	4.32E-69	3.91E-67	522.0292	526.0628	581.6314	2087.039	2072.798	2351.652
20796	LOC11962	1.51626	2.001681	2.212251	0.904816	0.365563	0.467325	0.542086	0	1.391463	0	2.139111	5.024897
15274	LOC10323	7.152327	2.000813	1.121369	1.784258	0.074382	0.129464	0	4.379295	4.174388	15.62489	10.69555	8.039836
19780	LOC11961	1.996071	2.000579	1.893831	1.056366	0.290801	0.390847	1.626259	0	0.695731	2.232127	6.417332	1.004979
15132	ACMSD	5.785502	1.999865	1.105482	1.809044	0.070444	0.123762	1.084173	1.094824	4.87012	8.928508	10.69555	8.039836
3561	GAL3ST1	58.57533	1.999652	0.344989	5.796283	6.78E-09	5.06E-08	23.8518	19.70683	27.13352	89.28508	91.98176	99.49297
3136	SNCB	80.12108	1.999405	0.320458	6.239209	4.40E-10	3.73E-09	29.27266	37.224	29.91645	93.74933	151.8769	138.6872
10573	LOC10322	15.77063	1.999296	0.732771	2.728406	0.006364	0.016003	8.131296	2.737059	8.348776	11.16063	32.08666	32.15934
15486	LOC11962	5.153758	1.999225	1.144401	1.746963	0.080644	0.138442	1.626259	1.642235	2.782925	11.16063	10.69555	3.014938
1768	S1PR1	182.9438	1.999134	0.233999	8.54336	1.30E-17	1.96E-16	56.91907	91.41777	71.66033	290.1765	275.9453	311.5436
14339	TNNI1	11.63384	1.997478	1.02201	1.95446	0.050647	0.093894	0.542086	1.094824	12.52316	22.32127	19.252	14.06971
21993	PDE6G	1.090919	1.997185	2.739736	0.72897	0.46602	0.563333	0	0	1.391463	0	2.139111	3.014938
9887	LOC10324	21.05345	1.997153	0.69186	2.886642	0.003894	0.01047	3.794605	5.474118	16.00182	40.17829	42.78221	18.08963
16088	AWAT2	6.25625	1.99715	1.211628	1.648319	0.099287	0.164069	1.084173	5.474118	0.695731	11.16063	17.11289	2.009959
8398	BMP3	19.59434	1.996767	0.60827	3.282698	0.001028	0.003254	4.878777	8.758589	9.740239	35.71403	36.36488	22.10955
7962	LOC10322	20.013	1.996376	0.583501	3.421376	0.000623	0.00208	8.673382	8.758589	6.261582	40.17829	32.08666	24.11951
12227	ATP4A	10.31071	1.996147	0.8454	2.361188	0.018216	0.039608	3.252518	2.737059	6.261582	22.32127	19.252	8.039836
8442	LOC11962	21.83454	1.994384	0.609683	3.271184	0.001071	0.003373	8.673382	4.926706	12.52316	35.71403	47.06043	22.10955
26599	LOC11962	0.450948	1.994089	4.056411	0.491589	0.62301	NA	0	0	0.695731	0	0	2.009959
26886	LOC10323	0.450948	1.994089	4.056411	0.491589	0.62301	NA	0	0	0.695731	0	0	2.009959
27086	LOC11962	0.450948	1.994089	4.056411	0.491589	0.62301	NA	0	0	0.695731	0	0	2.009959
27779	LOC11962	0.450948	1.994089	4.056411	0.491589	0.62301	NA	0	0	0.695731	0	0	2.009959
27985	LOC11962	0.450948	1.994089	4.056411	0.491589	0.62301	NA	0	0	0.695731	0	0	2.009959
28211	PROX2	0.450948	1.994089	4.056411	0.491589	0.62301	NA	0	0	0.695731	0	0	2.009959
28537	LOC10324	0.426228	1.994089	4.056411	0.491589	0.62301	NA	0	0.547412	0	0	0	2.009959

28630	LOC10321!	0.425341	1.994089	4.056411	0.491589	0.62301	NA	0.542086	0	0	0	0	2.009959
28634	LOC11962(	0.450948	1.994089	4.056411	0.491589	0.62301	NA	0	0	0.695731	0	0	2.009959
28922	LOC10322;	0.450948	1.994089	4.056411	0.491589	0.62301	NA	0	0	0.695731	0	0	2.009959
28945	LOC10323	0.450948	1.994089	4.056411	0.491589	0.62301	NA	0	0	0.695731	0	0	2.009959
28963	LOC10323	0.426228	1.994089	4.056411	0.491589	0.62301	NA	0	0.547412	0	0	0	2.009959
29263	LOC11962:	0.425341	1.994089	4.056411	0.491589	0.62301	NA	0.542086	0	0	0	0	2.009959
29489	LOC11962:	0.450948	1.994089	4.056411	0.491589	0.62301	NA	0	0	0.695731	0	0	2.009959
29532	LOC11962:	0.426228	1.994089	4.056411	0.491589	0.62301	NA	0	0.547412	0	0	0	2.009959
12472	LOC10324;	9.387808	1.993887	0.864553	2.306262	0.021096	0.044968	2.168345	5.474118	3.478657	22.32127	12.83466	10.04979
19161	MRGPRE	2.424963	1.993346	1.72029	1.158726	0.246568	0.342083	0	2.189647	0.695731	2.232127	6.417332	3.014938
19229	LOC10321	2.013349	1.993261	1.734785	1.148996	0.250558	0.346408	0.542086	1.094824	0.695731	4.464254	4.278221	1.004979
18999	LOC11962(	2.645094	1.993126	1.681944	1.185013	0.236012	0.330248	0	0.547412	2.782925	2.232127	4.278221	6.029877
963	LOC11962;	2175.065	1.992957	0.17871	11.15189	7.01E-29	1.94E-27	607.1367	1117.815	894.7105	3709.795	3506.002	3214.929
11801	LGALS2	13.40975	1.992894	0.813822	2.448808	0.014333	0.032289	4.878777	2.737059	8.348776	20.08914	36.36488	8.039836
8306	ADD2	20.23034	1.992715	0.601723	3.311684	0.000927	0.002968	5.420864	7.116354	11.82743	31.24978	40.6431	25.12449
20264	A1BG	1.554175	1.992007	2.021636	0.985344	0.324455	0.425621	0	0.547412	1.391463	2.232127	2.139111	3.014938
13030	LOC10321!	9.205176	1.991983	0.905866	2.19898	0.027879	0.056882	2.710432	3.284471	4.87012	20.08914	19.252	5.024897
18135	LOC10324	3.777869	1.990965	1.513567	1.315412	0.188371	0.276143	1.626259	2.737059	0	4.464254	12.83466	1.004979
17217	LOC10324;	9.129059	1.990325	1.35836	1.465241	0.142855	0.220584	0	0	11.1317	8.928508	25.66933	9.044815
10265	NRG2	23.32981	1.990209	0.711558	2.796974	0.005158	0.01336	2.710432	6.02153	19.48048	46.87467	42.78221	22.10955
13371	LOC10323;	8.008309	1.989831	0.93333	2.131971	0.033009	0.065631	3.252518	2.737059	3.478657	11.16063	21.39111	6.029877
2222	RSPH4A	108.9545	1.989703	0.263139	7.56142	3.99E-14	4.77E-13	47.7036	36.12918	48.00546	171.8738	171.1289	178.8863
20052	LOC11961!	2.218865	1.987957	1.94844	1.020282	0.307595	0.40781	0	0	2.782925	2.232127	4.278221	4.019918
12167	ACAP1	12.35303	1.987479	0.837716	2.372498	0.017668	0.038605	4.878777	2.189647	7.653045	24.5534	27.80844	7.034856
2550	LOC10321;	85.72804	1.987404	0.281693	7.055219	1.72E-12	1.80E-11	34.69353	32.84471	36.17803	133.9276	139.0422	137.6822
5239	LOC11961!	48.54629	1.986897	0.435741	4.559809	5.12E-06	2.60E-05	24.39389	14.78012	18.78475	78.12444	106.9555	48.23901
13443	LOC10323	7.816887	1.986724	0.937814	2.118462	0.034136	0.067508	4.878777	1.642235	2.782925	17.85702	10.69555	9.044815
19343	RPP38-DT	1.997847	1.985888	1.760532	1.128004	0.259318	0.356406	0.542086	1.094824	0.695731	2.232127	6.417332	1.004979
4496	LOC10324!	46.9743	1.985489	0.396099	5.012614	5.37E-07	3.18E-06	20.0572	20.25424	16.00182	66.96381	96.25998	62.30873
1469	PTGS2	196.4067	1.98544	0.212815	9.329431	1.06E-20	1.93E-19	78.60252	80.46954	80.00911	272.3195	295.1973	371.8424
21605	LOC11962	1.127947	1.984837	2.513838	0.789564	0.429782	0.52875	0	0	1.391463	2.232127	2.139111	1.004979
21606	LOC11962(	1.127947	1.984837	2.513838	0.789564	0.429782	0.52875	0	0	1.391463	2.232127	2.139111	1.004979
21607	LOC10324;	1.127947	1.984837	2.513838	0.789564	0.429782	0.52875	0	0	1.391463	2.232127	2.139111	1.004979
21608	LOC11962:	1.127947	1.984837	2.513838	0.789564	0.429782	0.52875	0	0	1.391463	2.232127	2.139111	1.004979
21609	NTF4	1.127947	1.984837	2.513838	0.789564	0.429782	0.52875	0	0	1.391463	2.232127	2.139111	1.004979
597	ANOS1	597.4806	1.984794	0.145551	13.63641	2.43E-42	1.08E-40	237.4338	270.9688	214.981	946.4218	892.0091	1023.069
9545	LOC11962;	17.42775	1.98374	0.667073	2.973798	0.002941	0.008192	4.878777	8.211177	7.653045	31.24978	38.50399	14.06971
11584	LOC11962;	13.92961	1.983339	0.793908	2.498198	0.012483	0.028647	4.336691	3.284471	9.044508	26.78552	32.08666	8.039836
14877	LOC11962!	5.343832	1.98287	1.067216	1.857984	0.063171	0.112845	2.168345	2.189647	2.087194	11.16063	6.417332	8.039836
15104	TNFRSF8	5.703802	1.982784	1.093496	1.813252	0.069793	0.122847	1.084173	3.831883	2.087194	4.464254	10.69555	12.05975
18753	LOC10321	2.474831	1.98277	1.619766	1.224109	0.220911	0.313172	1.626259	0.547412	0.695731	6.696381	4.278221	1.004979
15867	LOC11962;	4.625723	1.981334	1.17354	1.68834	0.091346	0.152972	2.710432	2.189647	0.695731	6.696381	6.417332	9.044815
11398	PLPP4	13.29221	1.981139	0.780801	2.537315	0.011171	0.026055	4.878777	2.737059	8.348776	33.4819	19.252	11.05477
19329	LOC10321(	2.461991	1.980967	1.753029	1.130025	0.258466	0.355492	0	2.189647	0.695731	4.464254	6.417332	1.004979
7630	SP6	29.58271	1.979112	0.563048	3.515	0.00044	0.001532	13.55216	6.02153	16.00182	58.0353	57.75599	26.12947
722	CFB	348.984	1.978583	0.15644	12.64755	1.15E-36	4.25E-35	143.6529	146.7064	133.5804	537.9426	556.1688	575.8532
7882	LOC10322(	20.87707	1.977506	0.573789	3.446399	0.000568	0.001916	7.047123	10.40082	7.653045	31.24978	42.78221	26.12947
397	CA2	1014.432	1.977327	0.125741	15.72541	1.01E-55	6.78E-54	419.0328	366.7659	448.7467	1647.31	1512.351	1692.385
12097	ESRP2	13.84037	1.97681	0.828061	2.387275	0.016974	0.037302	4.878777	6.02153	5.565851	15.62489	44.92132	6.029877
15111	LOC11962!	5.605594	1.976064	1.090911	1.811388	0.070081	0.123294	1.084173	2.189647	3.478657	11.16063	10.69555	5.024897
19046	TCF21	2.666619	1.97519	1.677402	1.177529	0.238984	0.333526	0	0.547412	2.782925	2.232127	6.417332	4.019918
10380	TFR2	16.37824	1.973632	0.712614	2.769569	0.005613	0.014375	3.252518	5.474118	11.1317	31.24978	32.08666	15.07469
4363	DNAH2	97.54065	1.973191	0.386505	5.10522	3.30E-07	2.01E-06	34.15144	28.46541	55.65851	151.7846	226.7457	88.43819
10388	SLC12A3	15.81548	1.972342	0.712457	2.768366	0.005634	0.014418	2.710432	8.758589	7.653045	22.32127	36.36488	17.08465
19042	FGD2	2.71915	1.971253	1.673416	1.177982	0.238804	0.3334	0	0.547412	2.782925	6.696381	4.278221	2.009959
541	DENND2A	692.6203	1.971176	0.139024	14.17873	1.24E-45	6.10E-44	305.1946	245.7879	294.9901	1046.868	1093.086	1169.796
1351	GPC3	189.1586	1.970646	0.202872	9.713758	2.63E-22	5.18E-21	79.6867	77.73248	72.35606	305.8014	323.0057	276.3694
20808	CD96	1.538672	1.970539	2.181711	0.903208	0.366416	0.468134	0	0.547412	1.391463	0	4.278221	3.014938
1550	GPR137C	155.109	1.96974	0.216864	9.082818	1.06E-19	1.81E-18	61.25576	63.49977	64.70302	258.9267	231.024	251.2449
14495	LOC11962(	7.851119	1.969705	1.022779	1.925836	0.054125	0.099269	1.626259	1.642235	6.261582	11.16063	21.39111	5.024897
20180	LOC11962;	2.240391	1.969311	1.969172	1.000071	0.317276	0.417957	0	0	2.782925	2.232127	6.417332	2.009959
8213	LOC11962	19.89317	1.967744	0.588717	3.342426	0.00083	0.002688	9.757555	6.568942	7.653045	40.17829	32.08666	23.11453
9348	MAP3K7CL	19.11349	1.966765	0.65144	3.019105	0.002535	0.00721	4.336691	6.02153	13.2189	22.32127	40.6431	28.13943
12842	LOC10321!	9.143428	1.966471	0.881646	2.230456	0.025717	0.053239	2.168345	4.926706	4.174388	20.08914	6.417332	17.08465
18987	TBX1	2.4456	1.966247	1.656465	1.187014	0.235222	0.329351	0.542086	1.642235	0.695731	2.232127	8.556443	1.004979
3349	LOC11962!	65.89984	1.965767	0.327949	5.994122	2.05E-09	1.62E-08	28.73058	29.01283	22.95914	107.1421	91.98176	115.5726
8767	TNS4	26.1992	1.965072	0.617484	3.182385	0.001461	0.004429	8.131296	8.211177	15.30609	60.26743	49.19955	16.07967
6464	SEMA6D	29.68446	1.964904	0.497029	3.953298	7.71E-05	0.000317	8.673382	14.78012	12.52316	51.33892	55.61688	35.17428
14705	PKD1L3	7.117037	1.964068	1.039375	1.889664	0.058803	0.106309	1.626259	5.474118	1.391463	8.928508	19.252	6.029877
18408	GP6	2.887863	1.963153	1.553375	1.263799	0.206302	0.297927	2.168345	0	1.391463	4.464254	4.278221	5.024897
18693	ELSPBP1	4.480781	1.962164	1.595298	1.229967	0.218709	0.311046	0	0	5.565851	4.464254	12.83466	4.019918
7522	PRPH	31.43829	1.960839	0.552059	3.551866	0.000383	0.001352	9.215468	13.6853	15.30609	35.71403	85.56443	29.1444
20417	GJA4	1.591203	1.960614	2.045454	0.958523	0.337799	0.439828	0	0.547412	1.391463	4.464254	2.139111	1.004979
19609	CUNH8orf4	2.001929	1.960595	1.807956	1.084426	0.278176	0.377138	0	1.094824	1.391463	2.232127	4.278221	3.014938

22087	LOC10323(	1.112444	1.960324	2.755394	0.71145	0.476806	0.573803	0	0	1.391463	0	4.278221	1.004979
22088	RAG2	1.112444	1.960324	2.755394	0.71145	0.476806	0.573803	0	0	1.391463	0	4.278221	1.004979
22089	LOC10322:	1.112444	1.960324	2.755394	0.71145	0.476806	0.573803	0	0	1.391463	0	4.278221	1.004979
22091	LOC11962:	1.112444	1.960324	2.755394	0.71145	0.476806	0.573803	0	0	1.391463	0	4.278221	1.004979
9194	SCX	18.73337	1.960094	0.641382	3.056049	0.002243	0.006485	7.047123	7.116354	9.044508	40.17829	12.83466	36.17926
478	VAMP8	1023.912	1.960065	0.1322	14.82649	9.88E-50	5.49E-48	458.6051	354.7229	443.1809	1685.256	1578.664	1623.042
371	RBM47	3433.224	1.959403	0.121501	16.12664	1.66E-58	1.19E-56	1273.903	1657.016	1282.233	5488.8	5266.49	5630.9
1207	LOC11962‹	563.6688	1.958718	0.19337	10.1294	4.09E-24	9.01E-23	298.6896	171.3399	221.2426	881.6902	966.878	842.1728
16269	SH2D1A	5.2187	1.957928	1.213006	1.614112	0.106503	0.174036	1.626259	0.547412	4.174388	13.39276	8.556443	3.014938
14834	GNG3	6.056542	1.957678	1.04973	1.864934	0.062191	0.111456	1.084173	2.189647	4.174388	11.16063	10.69555	7.034856
4405	DNAH17	68.59765	1.957344	0.385497	5.077452	3.83E-07	2.31E-06	19.51511	32.84471	31.30791	102.6778	151.8769	73.3635
15999	LOC11962:	4.684276	1.957028	1.174787	1.665858	0.095742	0.159091	2.710432	2.189647	0.695731	8.928508	8.556443	5.024897
18433	CYP7A1	2.891414	1.956591	1.553666	1.259338	0.207908	0.299856	0	2.189647	1.391463	4.464254	4.278221	5.024897
21989	OSTN	0.888597	1.95636	2.681744	0.72951	0.46569	0.563025	0.542086	0.547412	0	2.232127	0	2.009959
218	TGFBR2	10560.23	1.956088	0.100378	19.48727	1.41E-84	1.72E-82	4320.97	4610.302	4054.722	16028.9	15867.92	18478.56
13618	SLC22A31	7.132019	1.955967	0.937712	2.085893	0.036988	0.072208	2.168345	3.831883	2.782925	13.39276	8.556443	12.05975
21717	LOC11962(	1.350079	1.955855	2.521713	0.775606	0.437982	0.536158	1.626259	0	0	4.464254	0	2.009959
201	C1S	3624.686	1.95545	0.097	20.15927	2.23E-90	2.95E-88	1528.684	1433.672	1498.605	5678.531	5394.837	6213.788
22788	LOC10322:	0.924738	1.955149	3.286998	0.594813	0.551969	0.643939	1.084173	0	0	4.464254	0	0
16207	LOC10324‹	5.063997	1.953973	1.201552	1.626207	0.103906	0.17044	2.710432	2.737059	0.695731	2.232127	14.97377	7.034856
350	LOC10321(	53005.29	1.953896	0.118929	16.42913	1.18E-60	8.99E-59	23885.41	19099.2	22266.88	81463.71	75581.2	95735.35
20849	SERPINB1(	2.224888	1.953719	2.17451	0.898464	0.368938	0.470441	0	0	2.782925	0	8.556443	2.009959
21021	NCBP2L	2.261916	1.953283	2.226809	0.877167	0.380396	0.481059	0	0	2.782925	2.232127	8.556443	0
10896	IGFALS	14.11311	1.952256	0.736381	2.65115	0.008022	0.019572	8.131296	2.189647	6.957314	26.78552	23.53022	17.08465
12931	TRNAI-AAU	9.540296	1.952248	0.880949	2.216075	0.026686	0.054865	5.96295	4.379295	1.391463	17.85702	8.556443	19.09461
8784	TMEM20(	20.23033	1.952141	0.614125	3.178736	0.001479	0.004477	5.96295	8.211177	10.43597	40.17829	38.50399	18.08963
18385	LOC11962:	2.46341	1.952057	1.53991	1.267643	0.204925	0.296443	1.084173	0.547412	1.391463	4.464254	4.278221	3.014938
12946	NR4A3	8.968594	1.951297	0.881318	2.214067	0.026824	0.055084	1.626259	3.831883	5.565851	15.62489	17.11289	10.04979
22371	TRNAA-AG	0.889485	1.951033	2.942401	0.663075	0.507282	0.602812	0	1.094824	0	2.232127	0	2.009959
22372	LOC11962:	0.889485	1.951033	2.942401	0.663075	0.507282	0.602812	0	1.094824	0	2.232127	0	2.009959
3938	RNF212	55.41731	1.951013	0.360326	5.414584	6.14E-08	4.15E-07	24.39389	19.15941	24.3506	95.98146	96.25998	72.35852
287	ATP6V1H	2374.524	1.95072	0.110268	17.69064	4.95E-70	4.59E-68	1071.163	936.0742	921.8441	3816.937	3478.194	4022.933
15613	MYOZ2	5.872551	1.950135	1.128964	1.727367	0.084102	0.143097	1.626259	3.284471	2.087194	13.39276	12.83466	2.009959
11416	ADCY4	19.80822	1.948772	0.769492	2.532543	0.011324	0.02637	2.168345	8.211177	13.91463	29.01765	53.47777	12.05975
11275	LOC11961!	18.04751	1.948686	0.760218	2.563326	0.010367	0.024445	8.131296	6.02153	7.653045	31.24978	49.19955	6.029877
200	CTSA	5768.754	1.948239	0.096609	20.1663	1.94E-90	2.57E-88	2368.917	2425.034	2332.092	9158.417	8376.757	9951.307
14597	IL5RA	5.987682	1.948198	1.021709	1.906804	0.056546	0.102985	2.710432	3.284471	1.391463	8.928508	8.556443	11.05477
20477	LOC10323(	1.5757	1.948025	2.049765	0.950365	0.341927	0.44392	0	0.547412	1.391463	2.232127	4.278221	1.004979
16244	CHST13	4.226877	1.947907	1.20393	1.617957	0.105672	0.172945	1.084173	2.737059	1.391463	6.696381	6.417332	7.034856
3939	LTC4S	76.47824	1.947808	0.359809	5.413453	6.18E-08	4.17E-07	36.86187	18.06459	39.65669	140.624	109.0946	114.5677
19173	LOC11961!	2.44702	1.947629	1.682829	1.157354	0.247128	0.342664	1.626259	0	1.391463	2.232127	6.417332	3.014938
9791	POU2F3	15.25046	1.946882	0.669987	2.90585	0.003663	0.009945	4.336691	5.474118	9.044508	29.01765	23.53022	20.09959
16915	LOC11962:	4.005277	1.94687	1.294625	1.50381	0.13263	0.208453	1.084173	0.547412	3.478657	4.464254	6.417332	8.039836
10918	CLDN11	16.78762	1.946347	0.735243	2.647217	0.008116	0.019762	6.505036	5.474118	8.348776	42.41041	29.94755	8.039836
3310	PIWIL2	68.5448	1.946236	0.322656	6.031928	1.62E-09	1.30E-08	25.47806	32.2973	26.43779	116.0706	115.512	95.47305
8095	APOL4	26.25383	1.945645	0.575265	3.382172	0.000719	0.002361	8.131296	8.758589	15.30609	40.17829	62.03421	23.11453
13964	SSTR1	10.44043	1.94532	0.963521	2.01897	0.04349	0.082792	1.626259	1.642235	9.740239	29.01765	8.556443	12.05975
16923	ARHGAP1!	3.782318	1.944757	1.294053	1.502841	0.13288	0.208746	1.084173	1.642235	2.087194	6.696381	2.139111	9.044815
10567	VSX1	13.52183	1.944741	0.712577	2.729165	0.006349	0.015974	4.336691	8.211177	4.174388	15.62489	25.66933	23.11453
13639	LOC10322:	10.14206	1.944173	0.934136	2.081253	0.037411	0.072921	3.794605	1.642235	6.957314	15.62489	27.80844	5.024897
22803	PATE3	0.926513	1.943811	3.285015	0.591721	0.554038	0.645837	0	1.094824	0	4.464254	0	0
22804	LOC11962:	0.926513	1.943811	3.285015	0.591721	0.554038	0.645837	0	1.094824	0	4.464254	0	0
22805	LGSN	0.926513	1.943811	3.285015	0.591721	0.554038	0.645837	0	1.094824	0	4.464254	0	0
15697	LOC10321!	5.444383	1.943798	1.131978	1.717169	0.085948	0.145565	1.084173	2.737059	2.782925	15.62489	6.417332	4.019918
17115	LOC11962:	4.075251	1.942622	1.313556	1.478904	0.139166	0.216169	1.626259	0.547412	2.782925	8.928508	8.556443	2.009959
21812	TRNAQ-CU	1.387994	1.942556	2.563125	0.757886	0.448519	0.546635	1.084173	0.547412	0	6.696381	0	0
21815	METTL11B	1.387994	1.942556	2.563125	0.757886	0.448519	0.546635	1.084173	0.547412	0	6.696381	0	0
135	LOC10323:	10125.85	1.94192	0.084759	22.91111	3.60E-116	7.09E-114	3927.416	4351.377	4268.312	16002.12	16317.14	15888.73
14752	LOC11962:	7.380114	1.941718	1.032296	1.88097	0.059976	0.108084	1.084173	6.568942	1.391463	15.62489	8.556443	11.05477
21805	LOC10323:	1.300211	1.940783	2.556217	0.75924	0.447709	0.545854	0	1.642235	0	0	2.139111	4.019918
9189	LOC10321!	16.33213	1.940692	0.634584	3.058213	0.002227	0.006442	7.047123	6.02153	6.957314	26.78552	32.08666	19.09461
17465	RS1	5.446629	1.940584	1.370091	1.416391	0.156661	0.238468	0.542086	0	6.261582	11.16063	10.69555	4.019918
6485	LOC11961ٍ	29.5668	1.939296	0.491479	3.945839	7.95E-05	0.000326	15.17842	9.306001	11.82743	53.57105	51.33866	36.17926
3071	LCTL	79.82071	1.938182	0.307191	6.309379	2.80E-10	2.43E-09	34.15144	30.10765	35.4823	133.9276	100.5382	144.717
10778	LOC11962(	25.40044	1.938161	0.724958	2.673482	0.007507	0.018511	9.757555	1.642235	20.17621	37.94616	57.75599	25.12449
14679	NCF1	8.326788	1.93777	1.02381	1.892705	0.058397	0.105763	1.626259	1.094824	7.653045	11.16063	21.39111	7.034856
1199	SPEF2	224.0109	1.937641	0.190864	10.15195	3.25E-24	7.20E-23	86.73382	102.366	88.35788	368.301	363.6488	334.6582
9555	RGS4	18.14308	1.936806	0.651836	2.971308	0.002965	0.008251	4.336691	10.94824	6.957314	29.01765	38.50399	19.09461
21093	LOC11961ٍ	1.560198	1.93665	2.235962	0.866137	0.386415	0.487026	0	0.547412	1.391463	0	6.417332	1.004979
4232	GDPD3	58.1276	1.936517	0.372544	5.198095	2.01E-07	1.26E-06	28.73058	22.9913	20.87194	100.4457	64.17332	111.5527
18073	LOC10321ٍ	3.99349	1.936276	1.457025	1.328924	0.183873	0.270473	0.542086	1.642235	2.782925	0	14.97377	4.019918
13953	LOC11961ٍ	11.14938	1.934571	0.957101	2.021282	0.043251	0.082406	1.626259	4.379295	7.653045	24.5534	25.66933	3.014938
14782	LOC11961!	9.106173	1.933878	1.030549	1.876551	0.06058	0.108957	0.542086	4.379295	6.261582	22.32127	17.11289	4.019918
19832	LOC10324ٍ	2.43418	1.933708	1.844646	1.048282	0.294509	0.394792	0	1.642235	1.391463	0	8.556443	3.014938

5826	HSD11B2	35.95064	1.933583	0.453825	4.260633	2.04E-05	9.30E-05	16.26259	10.40082	18.08902	49.10679	70.59065	51.25395
14336	LOC11961ᵹ	9.340919	1.931765	0.988242	1.954748	0.050613	0.093852	1.084173	2.189647	8.348776	24.5534	12.83466	7.034856
511	TNIK	5697.431	1.930643	0.133859	14.42296	3.71E-47	1.93E-45	2095.164	2908.946	2099.717	8716.456	8793.884	9570.419
382	LOC10324ᵹ	1590.017	1.930332	0.120272	16.04967	5.75E-58	4.00E-56	615.8101	753.7861	613.6351	2415.161	2500.62	2641.086
16687	DKKL1	4.507502	1.930003	1.249483	1.544641	0.122433	0.19506	1.626259	1.094824	2.782925	6.696381	12.83466	2.009959
1916	TMEM184ᵹ	130.9302	1.929523	0.236329	8.164582	3.23E-16	4.48E-15	50.95612	56.38342	56.35424	187.4987	220.3284	214.0606
19978	LOC10322ᵹ	2.023454	1.929224	1.866537	1.033584	0.30133	0.400985	0	1.094824	1.391463	2.232127	6.417332	1.004979
11688	LOC10324ᵹ	10.7556	1.929001	0.779865	2.473508	0.013379	0.030432	5.420864	3.831883	4.174388	22.32127	10.69555	18.08963
15166	LOC11962ᵹ	11.60026	1.92865	1.070116	1.802282	0.071501	0.125337	0.542086	2.737059	11.1317	20.08914	32.08666	3.014938
176	SERPINI1	3106.931	1.928553	0.092106	20.93846	2.39E-97	3.61E-95	1266.314	1360.866	1250.229	5078.089	4778.773	4907.315
22437	LOC11962ᵹ	0.925625	1.928436	2.965116	0.650375	0.51545	0.610607	0.542086	0.547412	0	4.464254	0	0
22439	LOC11962ᵹ	0.925625	1.928436	2.965116	0.650375	0.51545	0.610607	0.542086	0.547412	0	4.464254	0	0
6947	SLC15A3	53.3172	1.928183	0.513097	3.75793	0.000171	0.000656	13.55216	12.59047	40.35242	98.21359	106.9555	48.23901
15216	NTN3	6.514401	1.92818	1.075374	1.793032	0.072968	0.127487	0.542086	2.737059	4.87012	8.928508	14.97377	7.034856
17126	SHOX	7.078942	1.927201	1.304618	1.477214	0.139618	0.216732	0	0.547412	8.348776	17.85702	10.69555	5.024897
19529	LOC11962ᵹ	2.022567	1.926747	1.758697	1.095554	0.273274	0.371991	0.542086	0.547412	1.391463	2.232127	6.417332	1.004979
19530	LOC11962ᵹ	2.022567	1.926747	1.758697	1.095554	0.273274	0.371991	0.542086	0.547412	1.391463	2.232127	6.417332	1.004979
11649	LMOD1	14.14927	1.925921	0.77619	2.48125	0.013092	0.029879	2.710432	7.116354	7.653045	17.85702	38.50399	11.05477
19952	GPR17	2.005123	1.925786	1.856225	1.037475	0.299515	0.399088	0	0.547412	2.087194	2.232127	2.139111	5.024897
519	BTBD16	1046.146	1.925147	0.133863	14.38143	6.77E-47	3.47E-45	471.0731	369.503	469.6187	1604.899	1600.055	1761.729
16009	LOC11962ᵹ	5.417625	1.924939	1.157573	1.662909	0.096331	0.159969	2.168345	1.642235	2.782925	8.928508	14.97377	2.009959
17033	LOC11962ᵹ	4.080221	1.924805	1.293329	1.488256	0.136683	0.213335	0.542086	1.094824	3.478657	8.928508	6.417332	4.019918
20782	LOC10322ᵹ	1.335464	1.924655	2.122547	0.906767	0.36453	0.466319	1.084173	0.547412	0	2.232127	2.139111	2.009959
15885	SLC18A2	7.258282	1.924499	1.140718	1.687095	0.091585	0.153276	1.084173	1.642235	6.261582	11.16063	21.39111	2.009959
354	DDX58	1050.316	1.924471	0.11778	16.33954	5.16E-60	3.88E-58	440.7162	470.2267	402.1327	1591.507	1739.097	1658.216
17412	LOC10322ᵹ	3.358918	1.92413	1.347856	1.427548	0.153422	0.234243	1.084173	1.642235	1.391463	4.464254	8.556443	3.014938
229	MYLK	7642.008	1.923625	0.101142	19.01915	1.18E-80	1.37E-78	3074.172	3470.591	3022.257	11718.67	11429.27	13137.09
12121	LOC11962ᵹ	10.2841	1.92362	0.807946	2.380875	0.017272	0.037882	2.710432	6.568942	3.478657	15.62489	19.252	14.06971
4503	FRMPD2	53.66973	1.923527	0.383963	5.009673	5.45E-07	3.22E-06	28.18849	19.70683	18.78475	109.3742	70.59065	75.37346
16599	LOC11962ᵹ	5.918631	1.923378	1.23493	1.557479	0.119357	0.191162	0	3.831883	3.478657	15.62489	8.556443	4.019918
316	RB1CC1	4650.706	1.92097	0.112712	17.04318	3.93E-65	3.30E-63	1824.663	2241.651	1762.983	7234.324	7208.803	7631.814
4609	TMEM169	58.24619	1.920212	0.388792	4.938924	7.86E-07	4.53E-06	16.26259	26.27577	30.61218	118.3027	70.59065	87.43321
19478	KRT38	2.261976	1.919245	1.737798	1.104412	0.269414	0.367739	1.084173	1.642235	0	6.696381	2.139111	2.009959
282	IFI30	1463.794	1.918351	0.10768	17.81523	5.38E-71	5.08E-69	628.2781	633.3555	576.7613	2287.93	2203.284	2453.155
9843	ACAN	22.52178	1.917689	0.662277	2.895599	0.003784	0.010221	4.878777	6.02153	17.39328	31.24978	53.47777	22.10955
19938	LOC10323ᵹ	2.903459	1.917232	1.841956	1.040868	0.297937	0.397264	0	2.189647	1.391463	0	12.83466	1.004979
9380	ETV3L	24.27649	1.916761	0.636248	3.012598	0.00259	0.007341	4.336691	9.306001	16.69755	53.57105	40.6431	21.10457
1545	BCO1	290.3253	1.91567	0.210404	9.104731	8.65E-20	1.49E-18	108.9594	157.6546	97.40239	491.0679	440.6568	446.2109
9613	LOC10321ᵹ	19.81628	1.915573	0.647921	2.956491	0.003112	0.008605	9.757555	7.663765	6.957314	31.24978	49.19955	14.06971
19925	MS4A13	2.520911	1.915072	1.835627	1.043279	0.296819	0.396032	0	1.094824	2.087194	8.928508	0	3.014938
19525	RBP7	3.862336	1.915011	1.745685	1.096997	0.272643	0.371227	0	0	4.87012	4.464254	12.83466	1.004979
13115	KCNE1	8.059688	1.914525	0.876146	2.185166	0.028877	0.058535	3.252518	2.737059	4.174388	11.16063	14.97377	12.05975
6517	GRIP2	62.99931	1.914388	0.4868	3.932592	8.40E-05	0.000343	17.88885	18.612	42.43961	91.51721	158.2942	49.24399
3922	ANKRD30B	63.34015	1.913085	0.352546	5.426478	5.75E-08	3.90E-07	19.51511	32.2973	27.82925	89.28508	117.6511	93.46309
20398	TRNAH-GU	2.057654	1.912856	1.988821	0.961804	0.336148	0.438106	0	0.547412	2.087194	6.696381	0	3.014938
14720	ZNF497	6.08557	1.912627	1.014481	1.885326	0.059386	0.107254	3.252518	2.189647	2.087194	13.39276	8.556443	7.034856
8616	TCTE1	29.3199	1.912344	0.59299	3.224917	0.00126	0.003888	5.420864	9.306001	22.2634	46.87467	59.8951	32.15934
302	LYN	1638.365	1.912199	0.109611	17.44529	3.74E-68	3.29E-66	687.3655	722.0362	656.0747	2491.054	2455.699	2817.962
18677	LOC11961ᵹ	5.509265	1.912156	1.55268	1.23152	0.218128	0.310486	0	0	6.957314	13.39276	10.69555	2.009959
5813	LOC10324ᵹ	35.05903	1.912095	0.448089	4.267219	1.98E-05	9.05E-05	12.46799	15.32753	16.00182	58.0353	66.31243	42.20914
1686	ANK1	177.4078	1.911666	0.218338	8.755552	2.03E-18	3.20E-17	68.84497	88.68071	65.39875	287.9444	280.2235	273.3544
11233	LOC10321ᵹ	23.11104	1.911485	0.742617	2.573983	0.010054	0.023794	4.336691	3.284471	21.56767	40.17829	49.19955	20.09959
10283	GUCY2C	14.92281	1.911348	0.684452	2.792523	0.00523	0.013521	5.420864	5.474118	7.653045	31.24978	25.66933	14.06971
11739	TNNC2	11.47297	1.911256	0.77679	2.460453	0.013876	0.031425	4.336691	7.116354	2.782925	24.5534	14.97377	15.07469
15232	LOC10323ᵹ	8.10359	1.91095	1.067783	1.789643	0.073511	0.128286	4.336691	3.284471	2.782925	0	17.11289	21.10457
20299	LOC10323ᵹ	2.232746	1.910946	1.953299	0.978317	0.327918	0.42947	0	2.737059	0	2.232127	6.417332	2.009959
577	RHPN1	1465.463	1.909965	0.13804	13.83635	1.54E-43	7.09E-42	651.5878	501.4292	697.1228	2294.627	2196.867	2451.145
255	TNIP1	10788.19	1.909773	0.104521	18.27167	1.39E-74	1.45E-72	4344.28	5060.822	4201.522	17060.15	15906.43	18155.96
15442	MAP3K8	5.146217	1.909456	1.089206	1.753072	0.07959	0.137022	1.626259	2.737059	2.087194	6.696381	10.69555	7.034856
19926	LOC10323ᵹ	2.042151	1.908738	1.830186	1.04292	0.296985	0.396234	0	0.547412	2.087194	4.464254	2.139111	3.014938
2473	USP2	103.2191	1.908282	0.26649	7.160809	8.02E-13	8.62E-12	39.5723	45.43518	45.91827	165.1774	143.3204	179.8913
12874	LOC11962ᵹ	9.65714	1.908264	0.858255	2.223424	0.026187	0.054073	5.96295	3.831883	2.087194	15.62489	21.39111	9.044815
11409	LOC11962ᵹ	15.03442	1.90721	0.752596	2.534175	0.011271	0.026264	7.047123	3.284471	8.348776	37.94616	23.53022	10.04979
12071	FAM9A	13.90572	1.907196	0.796755	2.393704	0.016679	0.036735	2.710432	4.926706	9.740239	33.4819	23.53022	9.044815
376	MAP3K5	987.5286	1.906835	0.118476	16.09471	2.78E-58	1.96E-56	408.7331	448.8777	390.3053	1484.364	1565.829	1627.062
22937	LOC11961ᵹ	0.893732	1.905637	3.323925	0.573309	0.566435	0.656495	1.084173	0	0	0	4.278221	0
22938	LOC10322ᵹ	0.893732	1.905637	3.323925	0.573309	0.566435	0.656495	1.084173	0	0	0	4.278221	0
14451	LOC10324ᵹ	8.720259	1.905555	0.985143	1.934293	0.053077	0.097644	6.505036	2.189647	2.087194	6.696381	27.80844	7.034856
21402	LOC10322ᵹ	1.372492	1.905533	2.334622	0.816206	0.414382	0.514734	1.084173	0.547412	0	4.464254	2.139111	0
7898	KIF26B	22.49165	1.905192	0.55367	3.441028	0.00058	0.001951	11.38381	7.663765	9.044508	37.94616	42.78221	26.12947
260	CLU	23482.07	1.904138	0.104915	18.14938	1.30E-73	1.33E-71	9532.589	10967.4	9208.005	36528.76	34268.55	40387.11
15491	LOC10322ᵹ	5.19786	1.903021	1.089741	1.746306	0.080758	0.138599	2.168345	2.189647	2.087194	11.16063	8.556443	5.024897
22384	LOC10324ᵹ	1.343261	1.902856	2.879657	0.660793	0.508745	0.604215	0	1.642235	0	0	6.417332	0
20671	PLA2G2A	2.094682	1.902378	2.054471	0.92597	0.354462	0.455874	0	0.547412	2.087194	8.928508	0	1.004979

3879	PLIN4	61.02467	1.902192	0.347815	5.468984	4.53E-08	3.10E-07	31.9831	21.89647	23.65487	84.82083	96.25998	107.5328
20771	LOC11962	1.763999	1.901919	2.095736	0.907519	0.364133	0.466034	1.626259	0	0.695731	2.232127	0	6.029877
21555	LOC11962	1.594398	1.901295	2.388396	0.796055	0.426	0.52541	0	0	2.087194	4.464254	0	3.014938
16631	LOC11961	4.979976	1.901054	1.224447	1.552581	0.120523	0.192659	1.626259	1.094824	3.478657	6.696381	14.97377	2.009959
720	GGT1	1341.908	1.900444	0.150091	12.66191	9.61E-37	3.55E-35	657.5508	475.1535	570.4997	2073.646	1854.609	2419.991
1684	DENND1C	178.0982	1.900431	0.216635	8.772499	1.75E-18	2.76E-17	62.88202	84.30142	78.61764	270.0874	297.3364	275.3644
17522	LOC10322	5.981964	1.900382	1.345998	1.411876	0.157986	0.239678	2.710432	0.547412	4.174388	15.62489	12.83466	0
19234	S100A12	2.489905	1.900293	1.655619	1.147784	0.251058	0.347009	0	1.094824	2.087194	4.464254	4.278221	3.014938
5724	LOC11961	34.56514	1.900264	0.440761	4.311327	1.62E-05	7.54E-05	11.9259	14.78012	17.39328	55.80317	49.19955	58.28881
18361	TRNAE-UU	2.660394	1.900123	1.493987	1.271847	0.203428	0.294535	1.084173	1.642235	0.695731	2.232127	4.278221	6.029877
22513	LOC10322	0.91101	1.899786	2.979128	0.637699	0.52367	0.61825	0	1.094824	0	2.232127	2.139111	0
22516	LOC11962	0.91101	1.899786	2.979128	0.637699	0.52367	0.61825	0	1.094824	0	2.232127	2.139111	0
13429	CA14	9.031914	1.899724	0.895723	2.120883	0.033932	0.067163	1.626259	5.474118	4.174388	15.62489	19.252	8.039836
15247	ADCYAP1	11.55837	1.899705	1.061875	1.78901	0.073613	0.128354	1.626259	0.547412	12.52316	13.39276	34.22577	7.034856
12416	LOC10322	11.77305	1.899169	0.819588	2.317224	0.020492	0.043876	7.589209	3.831883	3.478657	6.696381	29.94755	19.09461
20918	LOC11962	1.803855	1.898325	2.135445	0.88896	0.374025	0.475353	1.626259	0.547412	0	2.232127	6.417332	0
9507	GPR37L1	18.64427	1.898032	0.636291	2.98296	0.002855	0.007983	7.589209	4.926706	11.1317	22.32127	42.78221	23.11453
10113	SLC39A5	18.18651	1.897793	0.669443	2.834883	0.004584	0.012051	7.589209	6.02153	9.044508	40.17829	34.22577	12.05975
12622	MKRN2OS	9.525821	1.897415	0.833366	2.27681	0.022798	0.048017	3.794605	3.284471	4.87012	22.32127	12.83466	10.04979
11414	GRIN2D	16.94793	1.896282	0.748473	2.533535	0.011292	0.026307	3.252518	10.94824	6.957314	24.5534	44.92132	11.05477
2430	CRB3	127.5339	1.89619	0.262445	7.225095	5.01E-13	5.48E-12	59.6295	40.50847	62.61582	183.0344	205.3546	214.0606
12718	LOC11961	13.20986	1.896057	0.8411	2.254258	0.02418	0.050544	1.084173	6.568942	9.044508	31.24978	19.252	12.05975
1632	LOC10323	178.5965	1.896028	0.213131	8.896073	5.79E-19	9.42E-18	79.14461	71.16354	75.83472	272.3195	320.8666	252.2498
16927	LOC11961	4.500164	1.895024	1.261179	1.502581	0.132947	0.208802	0.542086	1.094824	4.174388	4.464254	10.69555	6.029877
22955	MS4A18	0.895507	1.894247	3.32184	0.57024	0.568515	0.658417	0	1.094824	0	0	4.278221	0
21700	CUNH18or	1.631426	1.893086	2.434571	0.777585	0.436814	0.535147	0	0	2.087194	6.696381	0	1.004979
2350	CDH3	136.161	1.891875	0.257708	7.341159	2.12E-13	2.40E-12	48.24569	56.93083	68.87741	245.534	173.268	224.1104
7774	VWF	27.86436	1.891685	0.544962	3.471223	0.000518	0.001771	7.589209	10.94824	16.69755	51.33892	53.47777	27.13445
16544	LOC10324	6.235985	1.890165	1.206517	1.566629	0.117202	0.188334	2.168345	1.642235	4.174388	0	21.39111	8.039836
20143	APOBEC2	1.802802	1.890128	1.88148	1.004596	0.315091	0.415862	0.542086	1.094824	0.695731	4.464254	0	4.019918
15958	PLN	5.203883	1.888603	1.128295	1.673857	0.094159	0.156864	2.168345	2.189647	2.087194	8.928508	12.83466	3.014938
3183	BAIAP2L2	112.5258	1.888495	0.305521	6.181225	6.36E-10	5.31E-09	55.8349	30.10765	58.44144	156.2489	177.5462	196.976
14831	LOC10324	8.458142	1.888363	1.011786	1.866366	0.06199	0.111119	1.626259	2.737059	6.261582	17.85702	19.252	3.014938
5573	COL11A2	102.6725	1.888317	0.430738	4.383913	1.17E-05	5.56E-05	32.52518	28.46541	69.57314	169.6417	237.4413	78.3884
2566	FOXH1	110.1125	1.886827	0.268465	7.028211	2.09E-12	2.17E-11	44.99317	52.00412	44.52681	149.5525	162.5724	207.0258
15881	OPN1SW	7.635137	1.88673	1.117841	1.687834	0.091443	0.153077	3.794605	1.642235	4.174388	2.232127	29.94755	4.019918
10907	TRIM71	17.44947	1.886344	0.711944	2.649567	0.00806	0.019643	2.710432	10.94824	8.348776	31.24978	36.36488	15.07469
281	WDR91	11233.08	1.884021	0.105655	17.83178	4.00E-71	3.79E-69	5078.265	4411.044	4881.947	17147.2	16216.6	19663.43
315	CTSF	1120.988	1.883742	0.110245	17.08692	1.86E-65	1.57E-63	486.7936	464.2052	484.9248	1725.434	1696.315	1868.257
22204	LOC11962	0.910123	1.883547	2.723007	0.691716	0.489116	0.585699	0.542086	0.547412	0	2.232127	2.139111	0
17231	KLRG1	4.330788	1.883481	1.287475	1.462926	0.143488	0.221381	1.626259	1.642235	2.087194	8.928508	10.69555	1.004979
14323	LOC10323	7.713599	1.882735	0.961829	1.957452	0.050294	0.093352	4.336691	3.831883	1.391463	17.85702	12.83466	6.029877
5688	LOC11962	36.13027	1.882622	0.434589	4.331955	1.48E-05	6.91E-05	13.01007	16.96977	16.69755	53.57105	49.19955	67.33362
13074	LOC11961	9.986507	1.882481	0.858744	2.192133	0.02837	0.057688	3.252518	4.379295	4.87012	17.85702	23.53022	6.029877
14363	CDHR4	8.18354	1.881961	0.965445	1.949319	0.051257	0.094864	1.084173	4.379295	4.87012	15.62489	17.11289	6.029877
4619	MUC22	50.48501	1.881869	0.381207	4.936613	7.95E-07	4.58E-06	16.80468	27.37059	20.17621	87.05295	79.14709	72.35852
6266	CBFA2T3	34.36568	1.881674	0.465359	4.043488	5.27E-05	0.000223	10.29964	16.96977	16.69755	69.19594	42.78221	50.24897
14888	LOC10322	6.754967	1.881138	1.012907	1.857168	0.063287	0.11301	2.168345	1.642235	4.87012	6.696381	17.11289	8.039836
16835	LOC11962	4.969443	1.88108	1.23848	1.518861	0.128797	0.20339	0.542086	1.642235	4.174388	4.464254	14.97377	4.019918
20118	LOC10321	2.062789	1.880271	1.862391	1.0096	0.312687	0.413182	0.542086	0	2.087194	4.464254	4.278221	1.004979
16226	PRPS1L1	5.397978	1.878564	1.158228	1.62193	0.104818	0.171737	1.084173	1.094824	4.87012	4.464254	12.83466	8.039836
20127	IL19	2.063677	1.877926	1.862353	1.008362	0.313281	0.413801	0	0.547412	2.087194	4.464254	4.278221	1.004979
16566	SMPDL3B	4.933528	1.877782	1.201588	1.562751	0.118111	0.189544	2.710432	2.737059	0.695731	4.464254	14.97377	4.019918
14569	LOC11962	7.254364	1.877673	0.982069	1.911955	0.055882	0.101971	2.710432	1.642235	4.87012	11.16063	17.11289	6.029877
10480	ASPHD1	23.21204	1.877628	0.68298	2.74917	0.005975	0.015156	7.589209	6.568942	15.30609	35.71403	62.03421	12.05975
7103	LOC10321	27.64845	1.876844	0.507935	3.695045	0.00022	0.000823	11.9259	8.758589	15.30609	42.41041	34.22577	53.26391
13154	ZFPM2	12.94494	1.876379	0.860786	2.179843	0.029269	0.059154	1.084173	3.831883	11.82743	22.32127	23.53022	15.07469
483	ATL1	660.8986	1.875699	0.126733	14.80037	1.46E-49	8.02E-48	285.6795	268.7792	294.2944	1029.011	1086.668	1000.96
5288	EDNRB	48.98731	1.875481	0.414418	4.525582	6.02E-06	3.03E-05	20.0572	15.32753	27.82925	55.80317	100.5382	74.36848
117	FAM107B	10089.93	1.873369	0.079102	23.68292	5.41E-124	1.23E-121	4250.499	4409.402	4319.1	16053.46	16079.69	15427.44
493	VCAN	47235.67	1.873007	0.127399	14.70189	6.27E-49	3.38E-47	18055.27	24611.09	18114.06	71718.24	71551.11	79364.23
500	NPC1L1	982.6968	1.872042	0.127717	14.65776	1.20E-48	6.39E-47	446.6792	400.158	420.9175	1473.204	1424.648	1730.575
7128	HHIPL2	40.74501	1.871308	0.507427	3.687839	0.000226	0.000844	13.55216	14.23271	24.3506	51.33892	104.8164	36.17926
4809	LOC11962	43.24155	1.87104	0.389188	4.807546	1.53E-06	8.45E-06	16.80468	20.80165	18.08902	66.96381	68.45154	68.3386
16724	HORMAD1	4.799014	1.870903	1.21646	1.537991	0.124051	0.197194	0.542086	2.737059	2.782925	11.16063	8.556443	3.014938
18147	LOC11962	3.195931	1.869536	1.423595	1.31325	0.189099	0.277026	2.168345	1.094824	0.695731	8.928508	4.278221	2.009959
14936	SLC35F4	6.12217	1.869423	1.011862	1.847508	0.064674	0.115114	1.626259	2.737059	3.478657	11.16063	10.69555	7.034856
13805	LOC11962	8.841283	1.869137	0.91336	2.04644	0.040713	0.078403	4.336691	3.284471	3.478657	13.39276	23.53022	5.024897
20603	LOC10323	2.3255	1.869003	1.99579	0.936473	0.34903	0.450369	0	2.189647	0.695731	8.928508	2.139111	0
186	ASPH	7600.059	1.868544	0.090581	20.6284	1.53E-94	2.18E-92	3147.896	3520.953	3134.966	11629.38	11825	12342.15
20246	CCKAR	2.048174	1.868127	1.890317	0.988261	0.323025	0.424164	0	0.547412	2.087194	2.232127	6.417332	1.004979
1060	LOC10322	568.6924	1.86788	0.174672	10.69367	1.09E-26	2.73E-25	224.4238	304.361	204.545	955.3503	797.8883	925.5861
10466	LOC11962	17.66834	1.867875	0.678638	2.752387	0.005916	0.015028	2.710432	10.94824	9.044508	31.24978	29.94755	22.10955
10811	CFAP157	14.08246	1.867577	0.699835	2.668596	0.007617	0.018731	5.96295	7.116354	4.87012	33.4819	14.97377	18.08963

21275	TNFSF11	1.60042	1.867357	2.22514	0.839209	0.401352	0.501502	0	0	2.087194	2.232127	4.278221	1.004979
21276	LOC11962:	1.60042	1.867357	2.22514	0.839209	0.401352	0.501502	0	0	2.087194	2.232127	4.278221	1.004979
11336	LOC10323!	21.08241	1.866293	0.732128	2.549134	0.010799	0.025326	5.420864	4.379295	17.39328	22.32127	59.8951	17.08465
17697	LOC11962(	5.634347	1.866207	1.34694	1.385516	0.165895	0.249216	0.542086	6.568942	0	6.696381	14.97377	5.024897
2772	LOC11962:	178.6861	1.864798	0.276628	6.741171	1.57E-11	1.51E-10	92.15468	56.38342	81.40057	258.9267	372.2053	211.0457
16799	LOC10321‹	4.33487	1.864752	1.222024	1.525953	0.127022	0.201016	1.084173	1.642235	2.782925	8.928508	8.556443	3.014938
2558	LBH	106.6823	1.864178	0.264971	7.035395	1.99E-12	2.07E-11	48.24569	41.05589	48.00546	174.1059	183.9635	144.717
12617	LOC10323:	17.98792	1.864173	0.818084	2.278706	0.022685	0.047798	1.084173	14.23271	7.653045	31.24978	40.6431	13.06473
15918	LOC10324:	6.352039	1.863703	1.108269	1.681634	0.09264	0.15472	1.084173	2.189647	4.87012	6.696381	19.252	4.019918
17407	LOC11962(	4.577414	1.863186	1.304366	1.428422	0.15317	0.233904	0.542086	0.547412	4.87012	8.928508	8.556443	4.019918
6332	PRR36	41.92149	1.862903	0.464343	4.011913	6.02E-05	0.000253	9.215468	21.89647	22.95914	69.19594	77.00798	51.25395
16636	LOC11962(	4.731513	1.861605	1.199624	1.551824	0.120704	0.19289	2.168345	0.547412	3.478657	4.464254	10.69555	7.034856
15855	LOC10324‹	8.360014	1.860843	1.101058	1.69005	0.091018	0.152616	4.878777	4.926706	0.695731	6.696381	29.94755	3.014938
7645	VILL	25.17046	1.8608	0.530008	3.510892	0.000447	0.001553	8.673382	8.758589	15.30609	46.87467	34.22577	37.18424
21471	LOC11961!	1.324931	1.859939	2.302557	0.807771	0.419223	0.51918	0	1.094824	0.695731	0	2.139111	4.019918
15522	SHOC1	6.385352	1.859196	1.069276	1.738742	0.08208	0.140581	1.626259	1.094824	5.565851	11.16063	12.83466	6.029877
499	TMEM63C	622.8145	1.858467	0.126752	14.66221	1.13E-48	6.00E-47	277.5482	264.3999	266.4651	937.4933	983.9909	1006.989
7197	LOC11962:	26.12036	1.857246	0.506237	3.668728	0.000244	0.000901	13.55216	9.853413	10.43597	33.4819	49.19955	40.19918
10846	LOC10324!	19.04884	1.856871	0.69771	2.661378	0.007782	0.019075	4.878777	7.663765	11.82743	44.64254	34.22577	11.05477
12021	LOC10323!	13.65626	1.856809	0.772801	2.402699	0.016275	0.035992	2.710432	7.116354	7.653045	22.32127	32.08666	10.04979
19241	LOC11962(	2.702392	1.855541	1.619597	1.14568	0.251927	0.348048	0	2.189647	1.391463	4.464254	2.139111	6.029877
16632	KCNA5	12.60795	1.855491	1.195241	1.5524	0.120567	0.192717	0	1.094824	15.30609	17.85702	36.36488	5.024897
10401	LOC11962!	17.09444	1.8549	0.670584	2.766098	0.005673	0.014501	5.96295	6.568942	9.740239	13.39276	42.78221	24.11951
17254	LOC11962(	5.16754	1.854308	1.272509	1.457206	0.145059	0.223508	2.710432	3.831883	0	4.464254	14.97377	5.024897
11842	LOC11962‹	12.62066	1.854291	0.760327	2.438807	0.014736	0.033082	4.336691	7.663765	4.174388	13.39276	32.08666	14.06971
10856	LOC10322:	13.04779	1.853207	0.696771	2.659707	0.007821	0.019152	5.96295	4.926706	6.261582	15.62489	21.39111	24.11951
19313	LOC10322:	3.013134	1.853075	1.636696	1.132205	0.257548	0.354432	1.084173	0	2.782925	8.928508	4.278221	1.004979
2985	CD274	84.00336	1.852091	0.287432	6.443571	1.17E-10	1.04E-09	36.31979	39.96106	32.69937	127.2312	141.1813	126.6274
11647	LEXM	11.82384	1.850636	0.74562	2.482009	0.013064	0.02982	6.505036	3.831883	4.87012	24.5534	17.11289	14.06971
21840	LOC11962(	1.584918	1.850245	2.452987	0.754282	0.45068	0.54857	0	0	2.087194	0	6.417332	1.004979
21841	LOC11961!	1.584918	1.850245	2.452987	0.754282	0.45068	0.54857	0	0	2.087194	0	6.417332	1.004979
9358	GREM2	25.73693	1.850027	0.613314	3.016443	0.002558	0.007266	8.131296	7.116354	18.08902	37.94616	62.03421	21.10457
14905	PDCD1	6.577366	1.849692	0.998035	1.853333	0.063835	0.113857	2.710432	1.642235	4.174388	8.928508	14.97377	7.034856
17453	LOC11962‹	3.874809	1.848823	1.303782	1.418046	0.156177	0.237888	1.084173	0.547412	3.478657	6.696381	6.417332	5.024897
19886	TNFSF18	1.824327	1.848524	1.764309	1.047733	0.294762	0.395012	0.542086	1.094824	0.695731	4.464254	2.139111	2.009959
16904	BEST2	6.394037	1.848421	1.227659	1.505647	0.132158	0.207845	0	2.737059	5.565851	8.928508	17.11289	4.019918
15444	LOC10321!	6.370075	1.847968	1.054328	1.752745	0.079646	0.137101	4.336691	1.642235	2.087194	11.16063	14.97377	4.019918
18957	LOC10324:	3.447161	1.847943	1.548061	1.193715	0.23259	0.32618	0	1.642235	2.782925	6.696381	8.556443	1.004979
1011	ROR1	464.5478	1.847917	0.168792	10.9479	6.80E-28	1.79E-26	169.673	240.8612	196.1962	705.3521	697.3501	777.8541
15189	MHC-DRA	7.130016	1.847753	1.027772	1.797824	0.072205	0.126379	1.084173	3.284471	4.87012	20.08914	6.417332	7.034856
20237	LOC10323!	3.016555	1.846957	1.866219	0.989678	0.322331	0.423441	3.794605	0	0	11.16063	2.139111	1.004979
5195	LOC11962:	46.86695	1.846621	0.40304	4.581729	4.61E-06	2.36E-05	18.97302	16.42235	26.43779	55.80317	79.14709	84.41828
15016	LOC10323!	7.094101	1.846186	1.008528	1.830576	0.067164	0.11891	3.252518	4.379295	1.391463	20.08914	6.417332	7.034856
14548	MYLPF	7.509901	1.846018	0.963075	1.916796	0.055264	0.100989	2.710432	2.737059	4.174388	11.16063	19.252	5.024897
6399	LOC10324:	38.56486	1.845233	0.463603	3.980196	6.89E-05	0.000286	11.38381	25.72836	13.2189	53.57105	64.17332	63.31371
19223	UPK2	2.946876	1.845211	1.603978	1.150397	0.249981	0.345718	0	1.094824	2.782925	2.232127	8.556443	3.014938
16104	EMILIN3	5.666356	1.844776	1.120582	1.646266	0.099709	0.164603	2.710432	0.547412	4.174388	6.696381	12.83466	7.034856
17508	TEX13D	3.822338	1.843228	1.304174	1.41333	0.157559	0.23947	2.168345	2.189647	0.695731	6.696381	2.139111	9.044815
512	GRK5	1013.423	1.842792	0.127795	14.41995	3.88E-47	2.01E-45	453.1842	477.8905	395.1754	1584.81	1461.013	1708.465
11835	DLL4	12.55719	1.842058	0.75471	2.44075	0.014657	0.032924	5.96295	3.284471	6.957314	24.5534	23.53022	11.05477
19933	GPR25	1.807937	1.840853	1.767261	1.041642	0.297578	0.396865	1.084173	0.547412	0.695731	2.232127	4.278221	2.009959
19934	LOC10323:	1.807937	1.840853	1.767261	1.041642	0.297578	0.396865	1.084173	0.547412	0.695731	2.232127	4.278221	2.009959
1170	FUCA2	294.7352	1.840275	0.17923	10.26768	9.85E-25	2.24E-23	115.4644	143.9693	127.3188	412.9435	481.2999	487.415
7409	OTOG	46.59127	1.840203	0.511577	3.597118	0.000322	0.001154	15.7205	16.42235	28.52499	51.33892	128.3466	39.1942
14326	CUNH2orf(	8.028883	1.840129	0.940336	1.956885	0.050361	0.093456	2.710432	2.737059	4.87012	17.85702	14.97377	5.024897
17360	LOC10321!	4.291557	1.839969	1.279888	1.437602	0.150547	0.230547	1.084173	1.094824	3.478657	2.232127	12.83466	5.024897
14179	SLFN11	9.56818	1.83972	0.929125	1.980056	0.047697	0.089425	2.168345	3.284471	6.957314	11.16063	27.80844	6.029877
16642	LOC11962!	5.235218	1.839653	1.186808	1.550084	0.121121	0.193475	4.878777	1.094824	0.695731	11.16063	8.556443	5.024897
602	MAP2	3523.869	1.839419	0.135466	13.57849	5.37E-42	2.37E-40	1371.479	1898.972	1346.24	5526.746	5497.514	5502.263
12091	LOC11962!	11.16181	1.839018	0.769889	2.38868	0.016909	0.037179	4.878777	6.02153	3.478657	24.5534	14.97377	13.06473
18089	LOC11962‹	3.170783	1.83876	1.388417	1.324357	0.185384	0.272455	0.542086	2.189647	1.391463	4.464254	6.417332	4.019918
20659	CHRNG	1.361071	1.838747	1.983095	0.927211	0.353817	0.455265	0.542086	0.547412	0.695731	2.232127	2.139111	2.009959
20660	LOC11962(	1.361071	1.838747	1.983095	0.927211	0.353817	0.455265	0.542086	0.547412	0.695731	2.232127	2.139111	2.009959
15683	LOC10323:	6.56417	1.838292	1.068882	1.719826	0.085464	0.144874	3.252518	0.547412	4.87012	6.696381	14.97377	9.044815
16272	LOC10324!	7.253806	1.838272	1.139523	1.613194	0.106702	0.174318	5.96295	0	3.478657	8.928508	17.11289	8.039836
7341	CPLX2	67.09548	1.836836	0.507593	3.618717	0.000296	0.001072	18.97302	15.32753	53.57132	113.8385	147.5986	53.26391
4800	PRSS36	52.76399	1.836629	0.381625	4.81266	1.49E-06	8.25E-06	26.02015	17.51718	26.43779	66.96381	79.14709	100.4979
14267	KRT16	11.40832	1.83604	0.933493	1.966849	0.049201	0.09168	1.626259	1.642235	11.82743	15.62489	25.66933	12.05975
2651	CABP1	109.9857	1.835988	0.266333	6.893566	5.44E-12	5.46E-11	41.74065	49.81448	52.17985	187.4987	183.9635	144.717
12166	LOC11962!	12.85745	1.835916	0.773731	2.37281	0.017653	0.038576	5.420864	3.831883	7.653045	8.928508	34.22577	17.08465
6512	LOC11962‹	29.49781	1.83558	0.4666	3.933947	8.36E-05	0.000341	13.55216	13.13788	11.82743	51.33892	44.92132	42.20914
10996	DIRAS2	14.71443	1.835149	0.697741	2.630129	0.008535	0.020634	9.757555	3.831883	5.565851	20.08914	29.94755	19.09461
13002	ASTL	11.37931	1.833951	0.832369	2.203292	0.027574	0.056381	3.252518	3.284471	8.348776	13.39276	29.94755	10.04979
17326	LOC11962:	4.344975	1.833818	1.269882	1.444085	0.148715	0.228188	0.542086	1.642235	3.478657	6.696381	10.69555	3.014938

20759	LOC11962	2.262601	1.831942	2.013014	0.910049	0.362797	0.464615	0.542086	1.642235	0.695731	0	10.69555	0
537	LOC10322	760.4261	1.83167	0.12875	14.22661	6.26E-46	3.10E-44	309.5313	367.3133	324.2108	1129.456	1215.015	1217.03
15493	LOC10324	7.103484	1.831288	1.048811	1.746061	0.0808	0.138648	4.878777	2.737059	1.391463	15.62489	14.97377	3.014938
10056	RBFOX3	21.58193	1.829652	0.641934	2.850217	0.004369	0.01155	8.673382	8.758589	10.43597	44.64254	44.92132	12.05975
2029	LOC11962	137.2669	1.829508	0.231159	7.914505	2.48E-15	3.25E-14	56.37698	64.04718	60.52863	229.9091	194.6591	218.0805
22226	LOC11962	0.913317	1.829488	2.660748	0.687584	0.491715	0.588124	0.542086	0	0.695731	2.232127	0	2.009959
22227	LOC11962	0.913317	1.829488	2.660748	0.687584	0.491715	0.588124	0.542086	0	0.695731	2.232127	0	2.009959
7592	LOC10323	30.38789	1.829093	0.518392	3.528395	0.000418	0.001464	10.29964	12.59047	16.69755	53.57105	62.03421	27.13445
423	KCNMA1	3630.655	1.826678	0.11874	15.38388	2.10E-53	1.32E-51	1517.842	1837.114	1436.685	5459.783	5399.115	6133.39
11124	GPA33	13.77685	1.826658	0.703271	2.597375	0.009394	0.02245	7.047123	5.474118	5.565851	13.39276	32.08666	19.09461
9797	EMILIN1	24.92309	1.826445	0.6287	2.905115	0.003671	0.009962	10.29964	4.379295	18.08902	40.17829	53.47777	23.11453
11280	LOC10322	16.25201	1.826109	0.712818	2.561816	0.010413	0.024541	3.252518	8.211177	9.740239	29.01765	34.22577	13.06473
21130	LOC10321	1.345568	1.824952	2.120411	0.86066	0.389426	0.489961	0.542086	0.547412	0.695731	0	4.278221	2.009959
12801	LOC10324	11.12557	1.824034	0.814532	2.239365	0.025132	0.052194	5.96295	4.926706	3.478657	13.39276	29.94755	9.044815
11744	GRIK3	15.64559	1.823609	0.741425	2.459599	0.013909	0.031486	7.589209	3.284471	9.740239	13.39276	42.78221	17.08465
378	APOE	17562.67	1.822813	0.113343	16.08226	3.40E-58	2.39E-56	8161.11	6704.7	8358.517	28928.37	24678.92	28544.43
19962	LOC10322	3.657812	1.822778	1.759524	1.035949	0.300226	0.399815	0	0	4.87012	2.232127	12.83466	2.009959
19963	SERPIND1	3.657812	1.822778	1.759524	1.035949	0.300226	0.399815	0	0	4.87012	2.232127	12.83466	2.009959
10344	PRKAG3	17.09309	1.822415	0.655967	2.778212	0.005466	0.014048	9.215468	6.02153	6.957314	26.78552	38.50399	15.07469
15857	HAND2	5.883445	1.822227	1.078415	1.689726	0.09108	0.1527	1.626259	3.284471	2.782925	4.464254	17.11289	6.029877
13701	LOC11962	7.967299	1.821243	0.880679	2.067999	0.03864	0.074976	3.794605	3.284471	3.478657	15.62489	8.556443	13.06473
5168	WDR64	43.50196	1.820969	0.396296	4.594976	4.33E-06	2.23E-05	18.97302	19.70683	18.78475	55.80317	83.42532	64.31869
856	ZBTB46	413.308	1.818466	0.154837	11.74439	7.55E-32	2.34E-30	203.2824	163.6761	180.8902	654.0132	626.7594	651.2267
9162	PIK3R5	23.3533	1.818026	0.593511	3.063173	0.00219	0.006355	7.589209	10.40082	12.52316	40.17829	51.33866	18.08963
2314	ANXA9	120.8052	1.81766	0.245908	7.391641	1.45E-13	1.67E-12	58.54533	49.81448	52.17985	169.6417	194.6591	199.9909
14915	LOC10323	8.401386	1.817597	0.981864	1.85117	0.064145	0.114334	3.794605	6.568942	0.695731	17.85702	6.417332	15.07469
20878	LOC10321	2.088397	1.816858	2.033108	0.893636	0.371517	0.473071	0	0	2.782925	4.464254	4.278221	1.004979
14853	LOC11962	6.884381	1.816326	0.975565	1.86182	0.062628	0.112097	1.626259	3.284471	4.174388	15.62489	8.556443	8.039836
10816	SIGLEC10	18.19828	1.814638	0.680511	2.666583	0.007663	0.018834	5.96295	8.211177	9.740239	17.85702	51.33866	16.07967
9248	CAPN12	29.6153	1.814391	0.596244	3.043037	0.002342	0.006733	8.131296	7.663765	23.65487	33.4819	70.59065	34.1693
219	SPARC	9419.01	1.813581	0.093218	19.45525	2.63E-84	3.19E-82	4112.267	4310.321	4097.162	14466.41	13609.02	15918.87
9308	CPNE9	19.9662	1.813559	0.59833	3.031035	0.002437	0.006959	7.047123	8.758589	10.43597	44.64254	27.80844	21.10457
15934	LOC11961	5.455276	1.812784	1.080038	1.678444	0.09326	0.155595	1.084173	2.737059	3.478657	6.696381	10.69555	8.039836
18411	LOC11962	3.175918	1.812737	1.434528	1.263648	0.206357	0.297973	1.084173	1.642235	1.391463	2.232127	10.69555	2.009959
3637	LOC10324	99.44433	1.812337	0.31764	5.705632	1.16E-08	8.47E-08	42.28274	35.58177	53.57132	165.1774	192.52	107.5328
12764	LOC10323	10.4073	1.812163	0.806189	2.247813	0.024588	0.051214	4.878777	6.02153	2.782925	11.16063	23.53022	14.06971
12584	SLC44A4	13.10595	1.812157	0.792418	2.286872	0.022203	0.046907	2.710432	5.474118	9.044508	24.5534	27.80844	9.044815
16384	LOC10323	5.540753	1.812069	1.138655	1.591412	0.111517	0.18095	1.626259	2.737059	2.782925	13.39276	10.69555	2.009959
10112	LHFPL5	17.67188	1.811305	0.638916	2.834963	0.004583	0.012049	8.673382	5.474118	9.044508	37.94616	27.80844	17.08465
17549	LOC11962	3.641482	1.811136	1.286256	1.408068	0.159111	0.241038	1.626259	1.094824	2.087194	4.464254	8.556443	4.019918
15464	MBD3L1	6.78623	1.81019	1.034001	1.750666	0.080003	0.137538	1.084173	3.831883	4.174388	4.464254	17.11289	10.04979
12385	SLC4A10	10.69971	1.809986	0.778215	2.325819	0.020028	0.042988	5.96295	3.284471	4.87012	15.62489	21.39111	13.06473
2309	CDKN2D	138.3102	1.809242	0.24432	7.405209	1.31E-13	1.51E-12	56.37698	73.90059	53.57132	198.6593	235.3022	212.0507
11843	LOC11962	15.99673	1.806519	0.740757	2.438746	0.014738	0.033082	2.168345	9.306001	9.740239	22.32127	36.36488	16.07967
728	FRK	1163.524	1.806504	0.143197	12.61553	1.73E-36	6.33E-35	473.7835	621.8598	456.3998	1854.898	1672.785	1901.421
4095	FAM240B	65.83154	1.805079	0.340574	5.300103	1.16E-07	7.51E-07	29.27266	22.9913	35.4823	120.5349	96.25998	90.44815
9718	LOC11962	19.03093	1.804023	0.61675	2.925049	0.003444	0.009422	4.878777	9.306001	11.1317	37.94616	27.80844	23.11453
4247	ZNF233	63.28752	1.80401	0.34761	5.189747	2.11E-07	1.32E-06	22.76763	35.58177	26.43779	82.5887	104.8164	107.5328
19658	LOC11962	4.145788	1.803793	1.674903	1.076953	0.281501	0.380695	0	0	5.565851	4.464254	12.83466	2.009959
22300	LOC11962	0.898702	1.803644	2.672944	0.674778	0.499817	0.595858	0	0.547412	0.695731	0	2.139111	2.009959
18832	LOC10322	3.612252	1.801031	1.483753	1.213835	0.224811	0.317364	0.542086	2.189647	2.087194	0	12.83466	4.019918
10184	THSD7A	28.22127	1.800205	0.638671	2.818672	0.004822	0.012588	7.047123	11.49565	18.78475	58.0353	59.8951	14.06971
16794	LOC10321	4.576324	1.799976	1.178698	1.527089	0.126739	0.200629	2.168345	1.094824	2.782925	6.696381	10.69555	4.019918
20447	PIK3CG	2.502317	1.798714	1.884236	0.954612	0.339774	0.441749	0	0	3.478657	2.232127	4.278221	5.024897
14371	LOC10323	8.949214	1.798139	0.922691	1.948798	0.05132	0.09493	2.710432	1.642235	7.653045	13.39276	19.252	9.044815
20612	LOC11962	2.334717	1.798116	1.923062	0.935027	0.349774	0.451133	0	1.642235	1.391463	6.696381	4.278221	0
15365	LOC10321	6.199218	1.797915	1.017735	1.766585	0.077298	0.133734	2.710432	3.284471	2.087194	13.39276	10.69555	5.024897
9280	BIK	18.25592	1.797452	0.591773	3.037403	0.002386	0.006836	9.215468	8.211177	6.957314	33.4819	23.53022	28.13943
15563	TRNAL-UA	5.873505	1.796573	1.035968	1.734197	0.082883	0.141582	3.252518	3.284471	1.391463	6.696381	8.556443	12.05975
18138	CD1D	3.66212	1.796045	1.365944	1.314874	0.188552	0.276362	2.168345	0.547412	2.087194	4.464254	10.69555	2.009959
3086	TSPAN1	120.4764	1.795796	0.285264	6.295211	3.07E-10	2.64E-09	62.88202	36.12918	63.31155	185.2665	173.268	202.0009
19931	LOC10322	3.270447	1.795788	1.723453	1.041971	0.297425	0.396721	0	2.189647	2.087194	8.928508	6.417332	0
15079	LOC10322	7.726207	1.795579	0.986423	1.820294	0.068714	0.121154	2.168345	3.284471	4.87012	4.464254	23.53022	8.039836
265	KIFC2	2479.265	1.795485	0.09939	18.06498	6.01E-73	6.03E-71	1095.014	1083.328	1150.74	3966.49	3553.063	4026.953
2711	RASGRF1	115.6399	1.795264	0.263174	6.82159	9.00E-12	8.83E-11	48.24569	59.66789	46.614	212.0521	160.4333	166.8266
18740	KRT32	3.905551	1.795056	1.464203	1.225961	0.220213	0.3124	1.084173	0	4.174388	4.464254	10.69555	3.014938
3518	KALRN	151.8925	1.794712	0.307282	5.840597	5.20E-09	3.93E-08	48.24569	61.31012	93.92374	232.1412	305.8928	169.8415
698	HIF1A	45414.03	1.794095	0.139749	12.83798	1.00E-37	3.83E-36	17601.54	25791.31	17592.26	70740.57	69561.74	71196.77
9774	ABCA12	26.83062	1.793157	0.616163	2.9102	0.003612	0.009824	5.420864	19.70683	10.43597	42.41041	59.8951	23.11453
20431	LEUTX	1.84816	1.79295	1.872893	0.957316	0.338408	0.440339	1.084173	0	1.391463	4.464254	2.139111	2.009959
12715	TEX29	10.71431	1.792602	0.795078	2.254624	0.024157	0.050508	3.794605	7.663765	2.782925	17.85702	17.11289	15.07469
14761	HENMT1	9.76971	1.791601	0.953179	1.879606	0.060162	0.108353	1.084173	4.926706	6.957314	26.78552	12.83466	6.029877
472	SASH1	1133.955	1.790615	0.120402	14.87193	5.01E-50	2.82E-48	492.7565	539.2006	496.0565	1611.596	1730.541	1933.58
17973	SLC30A2	5.955867	1.790344	1.328339	1.347807	0.17772	0.262876	0	0.547412	7.653045	8.928508	8.556443	10.04979

684	CAPS	873.6729	1.789897	0.138833	12.89241	4.97E-38	1.93E-36	437.4637	338.8479	401.437	1265.616	1328.388	1470.285
10486	LOC11962	20.45809	1.789863	0.651348	2.747936	0.005997	0.015205	4.336691	10.40082	12.52316	33.4819	44.92132	17.08465
18394	LOC10322	2.954379	1.789255	1.413805	1.26556	0.205671	0.297257	2.168345	1.094824	0.695731	4.464254	4.278221	5.024897
10251	COL17A1	25.86233	1.788995	0.638544	2.80168	0.005084	0.013184	9.757555	8.758589	16.00182	24.5534	77.00798	19.09461
9224	FREM1	21.59266	1.788774	0.586649	3.049141	0.002295	0.006614	7.047123	9.853413	11.82743	37.94616	42.78221	20.09959
681	LOC10322	1200.54	1.788715	0.13864	12.90184	4.39E-38	1.71E-36	583.827	454.8992	580.9357	1770.077	1741.236	2072.268
2694	BAIAP3	145.6843	1.788526	0.261288	6.845036	7.65E-12	7.54E-11	71.5554	45.98259	79.31338	236.6055	207.4937	233.1552
1386	VAV1	222.5286	1.788146	0.186379	9.594117	8.46E-22	1.62E-20	94.32303	106.7453	98.79385	330.3548	357.2315	347.7229
4266	IL22RA1	66.17166	1.78751	0.3452	5.178186	2.24E-07	1.40E-06	27.10432	27.37059	35.4823	111.6063	74.86887	120.5975
15153	LOC11962	9.40441	1.787423	0.990058	1.805373	0.071016	0.124594	3.794605	0.547412	8.348776	11.16063	23.53022	9.044815
435	GCNT1	982.2508	1.786732	0.117015	15.26927	1.23E-52	7.49E-51	433.6691	476.7957	413.9602	1522.311	1475.986	1570.783
17402	ZNF432	4.340525	1.786446	1.249991	1.429167	0.152956	0.233662	0.542086	3.831883	1.391463	6.696381	8.556443	5.024897
18367	LOC11962	3.232531	1.786378	1.406292	1.270275	0.203987	0.295257	0.542086	1.642235	2.087194	6.696381	6.417332	2.009959
22709	IQCF5	0.951233	1.78635	2.941794	0.607232	0.543697	0.636468	0	0.547412	0.695731	4.464254	0	0
15420	FAM174B	7.073825	1.786281	1.016544	1.757209	0.078882	0.135998	2.168345	4.379295	2.782925	6.696381	21.39111	5.024897
651	CRIP1	2779.443	1.784489	0.136057	13.11578	2.67E-39	1.09E-37	1077.126	1533.3	1140.999	4337.023	4205.492	4382.715
21354	TSSK3	1.382596	1.784022	2.16642	0.823488	0.41023	0.510723	0.542086	0.547412	0.695731	2.232127	4.278221	0
15543	MCEMP1	8.041754	1.783782	1.026822	1.737187	0.082354	0.14086	0.542086	2.737059	7.653045	11.16063	17.11289	9.044815
5348	POU3F3	45.11149	1.783318	0.397207	4.48965	7.13E-06	3.55E-05	19.51511	24.08612	16.69755	80.35657	72.72976	57.28383
15728	LOC10324	6.814144	1.782878	1.040786	1.713012	0.08671	0.146566	1.084173	2.737059	5.565851	4.464254	14.97377	12.05975
20482	LOC11962	1.832657	1.782251	1.876213	0.949919	0.342153	0.444104	1.084173	0	1.391463	2.232127	4.278221	2.009959
14897	TMEM266	7.952953	1.782093	0.960567	1.855252	0.06356	0.113435	4.878777	1.094824	4.87012	6.696381	17.11289	13.06473
1811	LOC11962	251.6816	1.781658	0.211391	8.428253	3.51E-17	5.15E-16	118.1748	125.9047	94.61947	352.6761	477.0217	341.693
14455	ABCA8	13.97135	1.781605	0.921668	1.933022	0.053234	0.097905	3.794605	3.831883	11.1317	6.696381	51.33866	7.034856
2589	JAKMIP3	147.3273	1.781464	0.254605	6.996971	2.62E-12	2.69E-11	62.88202	81.01695	54.26705	274.5516	203.2155	208.0308
695	RGS17	656.7583	1.781457	0.138653	12.84829	8.79E-38	3.36E-36	312.7838	266.5896	309.6005	921.8684	1084.529	1045.179
7775	MYH11	25.71343	1.781186	0.513125	3.471249	0.000518	0.001771	9.757555	10.40082	14.61036	49.10679	34.22577	36.17926
19244	EDN3	3.459573	1.781133	1.554926	1.145477	0.252011	0.348146	0	0.547412	4.174388	4.464254	8.556443	3.014938
21200	LOC11961	1.885188	1.780205	2.094287	0.850029	0.395309	0.495721	1.084173	0	1.391463	6.696381	2.139111	0
15254	SCN1A	7.073469	1.779371	0.995308	1.78776	0.073815	0.128646	4.336691	1.642235	3.478657	6.696381	19.252	7.034856
13320	GDPD2	10.81392	1.778605	0.829709	2.14365	0.032061	0.063992	2.168345	6.02153	6.261582	17.85702	23.53022	9.044815
18107	LOC11962	4.100289	1.778397	1.347571	1.319705	0.186933	0.274444	1.626259	3.831883	0	6.696381	6.417332	6.029877
17291	LOC10323	3.890109	1.778254	1.226281	1.450119	0.147025	0.226052	2.168345	1.642235	1.391463	6.696381	6.417332	5.024897
8868	ACTN3	26.78994	1.7781	0.564239	3.151324	0.001625	0.004872	9.757555	6.568942	20.17621	35.71403	51.33866	37.18424
21458	LOC11962	2.023784	1.777561	2.194334	0.810069	0.417901	0.517751	2.710432	0	0	0	6.417332	3.014938
20496	ODAPH	1.834432	1.777461	1.876084	0.947432	0.343419	0.445442	0	1.094824	1.391463	2.232127	4.278221	2.009959
351	LOC11962	4815.184	1.776292	0.108157	16.42329	1.30E-60	9.87E-59	2319.588	1912.109	2298.696	7611.553	6975.64	7773.516
13235	RAG1	10.29636	1.774174	0.820947	2.161132	0.030685	0.061637	3.252518	4.379295	6.261582	11.16063	25.66933	11.05477
17075	GALR2	5.120026	1.774129	1.195051	1.484563	0.13766	0.21433	2.168345	0.547412	4.174388	13.39276	6.417332	4.019918
9767	LOC10324	18.90725	1.774083	0.609159	2.912349	0.003587	0.009764	9.215468	7.663765	8.348776	40.17829	29.94755	18.08963
18143	GPR84	3.718733	1.773402	1.34989	1.313738	0.188934	0.276846	1.626259	0.547412	2.782925	8.928508	6.417332	2.009959
5734	RIIAD1	39.34776	1.772759	0.411558	4.307431	1.65E-05	7.66E-05	15.7205	17.51718	20.17621	55.80317	70.59065	56.27885
20898	LOC10323	2.043724	1.772457	1.989983	0.890689	0.373096	0.474627	0	2.737059	0	2.232127	4.278221	3.014938
982	LOC10324	387.3198	1.772247	0.159894	11.08389	1.50E-28	4.07E-27	173.4676	179.5511	174.6286	616.067	521.943	658.2616
9550	LOC11962	20.87412	1.771355	0.595933	2.972406	0.002955	0.008225	5.96295	10.40082	11.82743	29.01765	44.92132	23.11453
13928	LOC10322	12.73521	1.771305	0.874266	2.026048	0.04276	0.081618	6.505036	5.474118	4.87012	22.32127	34.22577	3.014938
21103	LOC11962	1.817154	1.771292	2.048732	0.86458	0.387269	0.487859	1.084173	0	1.391463	0	6.417332	2.009959
20170	LOC11961	3.027321	1.770359	1.768913	1.000818	0.316915	0.417707	0	0	4.174388	6.696381	4.278221	3.014938
13988	LOC10323	9.10311	1.769748	0.879465	2.012301	0.044188	0.083982	5.420864	2.737059	4.174388	6.696381	23.53022	12.05975
17196	MAL2	4.359388	1.769732	1.205291	1.468303	0.142022	0.219575	2.168345	2.189647	1.391463	6.696381	10.69555	3.014938
3215	CYP1A1	91.42175	1.769246	0.287754	6.148459	7.82E-10	6.47E-09	50.41403	35.58177	38.26523	131.6955	151.8769	140.6971
20045	LUM	2.511228	1.769083	1.732649	1.021028	0.307241	0.407463	1.084173	2.189647	0	2.232127	8.556443	1.004979
17256	NTN5	4.620629	1.768954	1.21399	1.457141	0.145077	0.22351	1.626259	0.547412	4.174388	8.928508	6.417332	6.029877
10992	MYBPHL	16.56654	1.768779	0.672339	2.630784	0.008519	0.020603	5.96295	6.568942	9.740239	24.5534	38.50399	14.06971
14331	LOC10324	8.098593	1.768556	0.904325	1.955664	0.050505	0.09369	2.710432	3.284471	4.87012	17.85702	12.83466	7.034856
704	SH3BP5	1914.602	1.76803	0.137986	12.81314	1.38E-37	5.23E-36	738.8637	1048.294	820.963	2837.033	2904.912	3137.546
10703	TRNAW-C	16.99681	1.767995	0.657063	2.690753	0.007129	0.017708	5.96295	11.49565	5.565851	35.71403	17.11289	26.12947
16273	HSPB7	6.440612	1.767327	1.095527	1.613221	0.106696	0.174318	2.710432	1.094824	4.87012	6.696381	19.252	4.019918
15448	LOC11962	8.018607	1.767295	1.00904	1.751462	0.079866	0.137445	0.542086	5.474118	4.87012	8.928508	19.252	9.044815
16554	LOC10323	6.042916	1.766971	1.129257	1.56472	0.117649	0.188939	0.542086	2.737059	4.87012	13.39276	10.69555	4.019918
15898	EXTL1	6.178849	1.76634	1.048508	1.684623	0.092061	0.153947	3.794605	1.094824	3.478657	6.696381	14.97377	7.034856
15463	DENND2D	6.422697	1.766042	1.008664	1.750872	0.079968	0.137486	1.626259	4.926706	2.087194	11.16063	10.69555	8.039836
5209	TREH	42.99957	1.765832	0.386069	4.573879	4.79E-06	2.44E-05	18.97302	20.25424	19.48048	62.49956	68.45154	68.3386
8556	MAEL	23.5588	1.765243	0.544903	3.239551	0.001197	0.00372	12.46799	13.13788	6.261582	37.94616	36.36488	35.17428
8329	LOC11962	24.66302	1.764985	0.533915	3.305742	0.000947	0.003024	14.09425	8.758589	11.1317	37.94616	27.80844	48.23901
1581	LOC11962	245.0789	1.764505	0.19531	9.034375	1.65E-19	2.77E-18	125.764	94.15483	115.4914	359.3724	363.6488	412.0416
21691	CHRNA1	1.402181	1.764298	2.266545	0.778408	0.436328	0.53475	0	0.547412	1.391463	4.464254	0	2.009959
21692	MAGEB18	1.402181	1.764298	2.266545	0.778408	0.436328	0.53475	0	0.547412	1.391463	4.464254	0	2.009959
1823	LOC11962	219.8759	1.763792	0.210298	8.387118	4.98E-17	7.27E-16	94.32303	90.87036	116.8829	325.8905	297.3364	393.952
2344	TG	180.727	1.763406	0.240004	7.347402	2.02E-13	2.29E-12	98.11763	77.18507	69.57314	312.4978	305.8928	221.0955
16257	PERM1	5.263592	1.763033	1.091226	1.615645	0.106171	0.173621	2.710432	1.094824	3.478657	6.696381	8.556443	9.044815
18575	CPZ	2.975904	1.762943	1.42317	1.238744	0.21544	0.308344	2.168345	1.094824	0.695731	4.464254	6.417332	3.014938
15237	CD247	8.004218	1.762768	0.985088	1.789452	0.073542	0.128314	2.710432	6.568942	1.391463	8.928508	21.39111	7.034856
421	KLHL24	888.8831	1.762364	0.11433	15.41472	1.30E-53	8.23E-52	421.7432	390.3046	402.8285	1339.276	1371.17	1407.976

8313	ELAVL3	35.5995	1.762099	0.532352	3.310024	0.000933	0.002983	10.29964	9.853413	28.52499	66.96381	57.75599	40.19918
19206	BSPRY	2.753772	1.762	1.529606	1.151931	0.249349	0.34515	0.542086	1.094824	2.087194	2.232127	8.556443	2.009959
21196	LOC10322(	1.87146	1.761566	2.071044	0.850569	0.395009	0.495438	0	1.094824	1.391463	4.464254	4.278221	0
19671	SLC35G6	2.337024	1.761209	1.638539	1.074866	0.282435	0.381721	0.542086	0.547412	2.087194	6.696381	2.139111	2.009959
4041	PDZD7	69.78552	1.760301	0.329596	5.340781	9.25E-08	6.09E-07	35.7777	26.82318	32.69937	89.28508	132.6249	101.5029
14315	C7	9.072771	1.76017	0.89861	1.958769	0.05014	0.093132	3.794605	2.189647	6.261582	22.32127	12.83466	7.034856
11092	CUNH16or	16.9749	1.759798	0.674937	2.607354	0.009124	0.021869	3.794605	7.663765	11.82743	20.08914	36.36488	22.10955
19596	LOC11962(	2.78886	1.756539	1.616465	1.086654	0.27719	0.376031	0	1.094824	2.782925	6.696381	2.139111	4.019918
11094	SLC5A11	16.34873	1.75632	0.67378	2.606668	0.009143	0.021909	8.673382	3.284471	10.43597	26.78552	27.80844	21.10457
2761	S100A14	107.9651	1.756121	0.259872	6.75764	1.40E-11	1.35E-10	47.7036	45.43518	54.96278	151.7846	186.1026	161.8017
779	LRRN4CL	409.9959	1.755913	0.143616	12.2264	2.25E-34	7.67E-33	185.9356	186.12	189.9347	624.9956	643.8723	629.1171
22448	LOC11962	0.934843	1.755035	2.702492	0.649414	0.516071	0.611179	0.542086	0	0.695731	2.232127	2.139111	0
21306	TPD52L3	1.386679	1.75446	2.107902	0.832325	0.405226	0.505628	0	0.547412	1.391463	2.232127	2.139111	2.009959
10853	PIWIL4	15.48967	1.75409	0.659437	2.65998	0.007815	0.019142	5.96295	9.306001	6.261582	17.85702	21.39111	32.15934
20349	LOC10323	2.996316	1.753992	1.809555	0.969295	0.332398	0.434262	0	0	4.174388	2.232127	8.556443	3.014938
13865	C1QL3	9.145536	1.753036	0.860673	2.03682	0.041668	0.079895	5.420864	3.831883	3.478657	15.62489	6.417332	20.09959
20708	LOC11961	1.836739	1.7525	1.907039	0.918964	0.358114	0.459748	0.542086	0	2.087194	2.232127	2.139111	4.019918
8986	VIPR2	26.32565	1.75156	0.561179	3.121216	0.001801	0.005328	13.55216	8.211177	13.91463	42.41041	57.75599	22.10955
14754	LOC11962	9.565882	1.751338	0.93128	1.88057	0.06003	0.108168	4.336691	1.642235	6.957314	22.32127	17.11289	5.024897
402	HABP2	4971.215	1.75077	0.111696	15.67447	2.26E-55	1.50E-53	2110.884	2595.279	2127.547	7633.874	7217.359	8142.344
21169	TEX43	1.85313	1.750585	2.047156	0.85513	0.392479	0.492893	0	0.547412	2.087194	4.464254	0	4.019918
277	AOPEP	2166.258	1.750333	0.09785	17.88783	1.47E-71	1.41E-69	1057.611	954.6862	966.3709	3258.905	3337.013	3422.96
22458	CCDC192	0.93573	1.750188	2.701784	0.64779	0.517121	0.612095	0	0.547412	0.695731	2.232127	2.139111	0
22459	LOC10323	0.93573	1.750188	2.701784	0.64779	0.517121	0.612095	0	0.547412	0.695731	2.232127	2.139111	0
22460	LOC10324	0.93573	1.750188	2.701784	0.64779	0.517121	0.612095	0	0.547412	0.695731	2.232127	2.139111	0
16198	SKOR2	9.094389	1.74933	1.074963	1.627339	0.103665	0.170141	1.626259	4.379295	6.261582	15.62489	25.66933	1.004979
14443	LOC10323	10.3093	1.748365	0.90321	1.935723	0.052902	0.097375	4.878777	1.094824	8.348776	8.928508	23.53022	15.07469
15215	PRG4	8.097859	1.747887	0.974784	1.793102	0.072957	0.127476	2.168345	6.568942	2.087194	15.62489	17.11289	5.024897
19309	LOC11962	4.237868	1.747747	1.542602	1.132986	0.25722	0.354145	0.542086	4.379295	0.695731	13.39276	6.417332	0
15892	LOC11962	6.290656	1.74685	1.036194	1.685834	0.091828	0.153615	1.626259	2.737059	4.174388	15.62489	8.556443	5.024897
18992	LOC11961	3.01382	1.746578	1.471942	1.186581	0.235393	0.329503	1.626259	1.642235	0.695731	6.696381	6.417332	1.004979
15804	IRX4	5.977416	1.74595	1.026729	1.700497	0.089038	0.149776	3.794605	2.189647	2.087194	8.928508	12.83466	6.029877
12299	LOC11962(	14.67262	1.745904	0.744584	2.344805	0.019037	0.04115	6.505036	9.853413	3.478657	15.62489	38.50399	14.06971
981	CA11	918.2784	1.745037	0.157404	11.08635	1.46E-28	3.96E-27	354.5245	524.9679	386.1309	1462.043	1351.918	1430.086
7910	LOC11962	35.73065	1.744849	0.507941	3.435143	0.000592	0.00199	9.757555	13.13788	26.43779	49.10679	72.72976	43.21412
587	PRRT3	993.9351	1.744418	0.126923	13.74387	5.54E-43	2.51E-41	411.4436	506.9033	453.6169	1540.168	1437.482	1613.997
799	IL6R	421.2934	1.744217	0.144291	12.08817	1.22E-33	4.06E-32	184.3094	197.0683	200.3706	649.549	633.1768	663.2864
5757	LOC10324	47.43677	1.744152	0.405924	4.296741	1.73E-05	8.00E-05	25.47806	23.53871	16.69755	78.12444	51.33866	89.44317
22841	LOC11962	0.91934	1.743859	2.971817	0.586799	0.557339	0.648695	0.542086	0	0.695731	0	4.278221	0
18159	TRNAP-CG	3.483465	1.743545	1.330194	1.310745	0.189944	0.27808	1.626259	1.094824	2.087194	8.928508	2.139111	5.024897
11311	CITED1	17.25532	1.74345	0.682178	2.555712	0.010597	0.024907	5.420864	7.116354	11.1317	17.85702	44.92132	17.08465
635	OAS2	581.3311	1.743124	0.131575	13.2481	4.63E-40	1.94E-38	260.2015	281.3697	261.595	888.3865	857.7834	938.6508
11816	LOC10322	15.71094	1.742297	0.712792	2.444328	0.014512	0.032651	4.878777	9.306001	7.653045	8.928508	36.36488	27.13445
15654	LOC11962	7.923464	1.741944	1.010303	1.72418	0.084675	0.143803	5.420864	1.094824	4.174388	17.85702	14.97377	4.019918
325	RHOBTB1	3290.325	1.741859	0.103505	16.8288	1.50E-63	1.23E-61	1478.812	1645.52	1421.379	4874.965	4879.311	5441.964
12772	DAO	11.35564	1.74102	0.775097	2.246197	0.024691	0.051395	4.336691	5.474118	5.565851	22.32127	21.39111	9.044815
3923	MPZL3	75.56746	1.739923	0.320697	5.425434	5.78E-08	3.92E-07	36.31979	32.2973	36.17803	84.82083	141.1813	122.6075
12892	FAM133A	11.56718	1.739524	0.783495	2.22021	0.026405	0.05445	3.794605	4.379295	7.653045	17.85702	25.66933	10.04979
733	ARHGAP24	720.7617	1.739208	0.138129	12.5912	2.36E-36	8.56E-35	295.4371	380.4512	320.7322	1116.063	1097.364	1114.522
22850	LOC11962(	0.920227	1.738758	2.971009	0.585241	0.558385	0.649629	0	0.547412	0.695731	0	4.278221	0
11694	LOC10323(	17.65954	1.73846	0.703165	2.472336	0.013423	0.030516	4.336691	6.02153	13.91463	31.24978	36.36488	14.06971
10019	COL28A1	22.5716	1.73831	0.608461	2.856897	0.004278	0.011352	8.673382	11.49565	10.43597	49.10679	40.6431	15.07469
721	LRFN5	660.9113	1.738261	0.137418	12.64943	1.13E-36	4.15E-35	336.0935	289.5808	290.12	993.2965	951.9042	1104.472
16172	LOC10323	5.548715	1.73813	1.064903	1.632195	0.102638	0.168726	1.626259	3.831883	2.087194	11.16063	8.556443	6.029877
1264	LOC11962	648.1724	1.737632	0.174549	9.95499	2.40E-23	5.04E-22	319.2889	236.4819	342.2998	1127.224	840.6705	1023.069
8432	PDGFRB	30.13115	1.737231	0.530773	3.27302	0.001064	0.003354	11.38381	9.853413	20.17621	51.33892	59.8951	28.13943
6900	LOC10322	43.83607	1.737084	0.459984	3.776404	0.000159	0.000613	16.26259	21.34906	22.2634	78.12444	89.84265	35.17428
9387	WNT5B	26.26638	1.736933	0.577061	3.009964	0.002613	0.0074	7.047123	10.94824	18.08902	51.33892	47.06043	23.11453
693	RASGRP3	509.3896	1.736733	0.13505	12.85997	7.56E-38	2.90E-36	226.5921	246.8827	231.6785	799.1015	772.219	779.8641
17141	LOC10322	4.613872	1.736228	1.177923	1.473975	0.140488	0.217892	1.084173	3.831883	1.391463	8.928508	6.417332	6.029877
21253	LOC10321	1.890158	1.735405	2.062023	0.841603	0.40001	0.500366	0	0.547412	2.087194	6.696381	0	2.009959
21791	LOC11961	1.371176	1.734627	2.279652	0.760918	0.446706	0.544981	0	0.547412	1.391463	0	4.278221	2.009959
20827	MSMB	2.343934	1.734326	1.925341	0.900789	0.367701	0.469358	0	1.094824	2.087194	4.464254	6.417332	0
11344	DDX4	16.74561	1.7338	0.680515	2.547775	0.010841	0.025407	6.505036	8.211177	8.348776	13.39276	44.92132	19.09461
10178	LOC10321	20.90943	1.733768	0.614831	2.819908	0.004804	0.012547	8.673382	12.04306	7.653045	44.64254	36.36488	16.07967
1017	MROH1	2066.743	1.733461	0.158856	10.91218	1.01E-27	2.64E-26	1104.772	729.6999	1035.248	2928.551	2954.112	3648.075
13689	LOC10323	9.036428	1.730709	0.835523	2.071408	0.038321	0.074421	4.336691	3.831883	4.174388	17.85702	14.97377	9.044815
8349	LOC10323(	33.81421	1.729878	0.524715	3.296793	0.000978	0.003114	12.46799	8.211177	26.43779	55.80317	57.75599	42.20914
6748	LOC11961	34.64901	1.729817	0.450688	3.838171	0.000124	0.000488	18.43094	12.04306	18.08902	42.41041	55.61688	61.30375
5806	LOC10323(	57.74996	1.729462	0.404944	4.270867	1.95E-05	8.92E-05	20.59928	22.9913	36.17803	98.21359	111.2338	57.28383
5249	PCDH18	51.02078	1.729366	0.379822	4.553098	5.29E-06	2.68E-05	17.34676	29.01283	24.3506	87.05295	77.00798	71.35354
1281	IFIT2	350.6155	1.729196	0.17463	9.902074	4.08E-23	8.46E-22	155.5788	191.5941	139.842	553.5675	513.3866	549.7238
20784	LOC11961	2.341106	1.728183	1.906109	0.906655	0.36459	0.466355	0	0.547412	2.782925	6.696381	0	4.019918
15120	LOC11961	7.881228	1.727274	0.954354	1.809888	0.070313	0.123629	1.084173	4.926706	4.87012	13.39276	14.97377	8.039836

13211	BIRC7	13.08578	1.726277	0.797074	2.165766	0.030329	0.061032	2.710432	4.379295	11.1317	31.24978	14.97377	14.06971
15557	LOC103225	6.993426	1.725968	0.994981	1.734675	0.082798	0.141483	2.168345	2.737059	4.87012	17.85702	4.278221	10.04979
6141	LOC119624	55.07773	1.72578	0.420826	4.100932	4.11E-05	0.000178	27.10432	13.6853	36.17803	71.42806	102.6773	79.39338
13528	LOC119621	10.79765	1.72488	0.820489	2.10226	0.035531	0.069824	6.505036	5.474118	2.782925	11.16063	27.80844	11.05477
1959	LOC103228	161.6527	1.724334	0.213619	8.072026	6.91E-16	9.38E-15	72.63957	74.99542	77.22618	272.3195	239.5804	233.1552
15319	KREMEN2	7.480148	1.72413	0.970594	1.776367	0.075673	0.131324	2.710432	3.284471	4.174388	17.85702	12.83466	4.019918
20021	LOC103246	3.919738	1.723273	1.682259	1.024381	0.305655	0.405866	0	0	5.565851	2.232127	10.69555	5.024897
15669	PPY	6.180789	1.723221	1.000411	1.722514	0.084977	0.144177	3.794605	2.189647	2.782925	6.696381	8.556443	13.06473
12611	STH	12.99957	1.722654	0.75542	2.280393	0.022584	0.04761	9.757555	4.379295	4.174388	11.16063	21.39111	27.13445
9494	LMAN1L	22.16921	1.722366	0.57694	2.985348	0.002833	0.007932	9.757555	6.568942	14.61036	29.01765	44.92132	28.13943
10642	CUNH2orf7	19.64383	1.72235	0.636006	2.708071	0.006768	0.016905	5.96295	14.78012	6.261582	31.24978	38.50399	21.10457
13667	CST5	11.11225	1.721951	0.829868	2.074968	0.037989	0.073897	4.336691	2.737059	8.348776	13.39276	27.80844	10.04979
19170	LOC103241	3.43962	1.721488	1.487173	1.157557	0.247045	0.342596	0	2.737059	2.087194	2.232127	8.556443	5.024897
16301	ADGRG7	5.798701	1.721056	1.071359	1.606424	0.108181	0.17643	2.168345	1.094824	4.87012	8.928508	10.69555	7.034856
17925	SPATS1	5.605799	1.720744	1.269135	1.35584	0.17515	0.259769	1.626259	0	6.261582	11.16063	8.556443	6.029877
16977	F10	6.56483	1.719671	1.149318	1.496253	0.134588	0.210764	0.542086	1.642235	6.957314	13.39276	12.83466	4.019918
17337	LOC103235	4.149728	1.719492	1.192697	1.441683	0.149392	0.229081	1.626259	2.737059	1.391463	6.696381	6.417332	6.029877
17485	RGN	4.150616	1.719047	1.215978	1.413715	0.157446	0.239388	1.084173	3.284471	1.391463	6.696381	6.417332	6.029877
11690	ZMYND15	19.21907	1.718801	0.69493	2.473346	0.013385	0.030441	6.505036	8.211177	11.82743	20.08914	55.61688	13.06473
917	GFPT2	943.4322	1.718589	0.150662	11.40691	3.86E-30	1.12E-28	430.9587	512.9249	376.3907	1412.936	1296.301	1631.082
18636	FCN1	5.852946	1.718448	1.392275	1.234274	0.217101	0.309867	0	0.547412	7.653045	8.928508	14.97377	3.014938
19053	CUNH3orf7	2.987784	1.717947	1.460105	1.176591	0.239359	0.333982	0.542086	2.189647	1.391463	2.232127	8.556443	3.014938
13915	CMTM2	9.044758	1.71769	0.847086	2.027764	0.042584	0.081359	4.878777	2.737059	4.87012	15.62489	17.11289	9.044815
20268	ACOT12	3.319887	1.717678	1.743849	0.984993	0.324628	0.425806	0	1.094824	3.478657	8.928508	6.417332	0
5129	LXN	47.11922	1.716247	0.371967	4.613978	3.95E-06	2.05E-05	19.51511	23.53871	22.95914	75.89232	68.45154	72.35852
10183	LOC119618	19.95824	1.715501	0.608588	2.81882	0.00482	0.012584	7.589209	8.211177	11.82743	31.24978	42.78221	18.08963
2383	LOC119625	187.701	1.715402	0.235028	7.298699	2.91E-13	3.24E-12	107.8752	70.06871	84.1835	270.0874	333.7013	260.2897
585	LOC103224	977.1131	1.715255	0.124578	13.76849	3.94E-43	1.79E-41	462.9418	425.8864	482.8376	1457.579	1388.283	1645.151
774	METTL7B	718.0329	1.71495	0.13952	12.29176	1.00E-34	3.44E-33	304.6525	386.4727	315.1663	1078.117	1078.112	1145.677
579	TRIB1	8994.48	1.714538	0.124066	13.81954	1.94E-43	8.92E-42	3801.652	5041.663	3760.428	13394.99	13442.17	14525.97
13474	LOC119622	12.83788	1.713256	0.811145	2.112145	0.034674	0.068414	9.215468	2.189647	6.261582	26.78552	23.53022	9.044815
17947	TEX50	4.362155	1.713096	1.267458	1.351599	0.176504	0.261464	0.542086	2.189647	3.478657	2.232127	10.69555	7.034856
5081	WNT11	49.75152	1.712947	0.368947	4.642801	3.44E-06	1.80E-05	25.47806	20.25424	24.3506	64.73168	81.28621	82.40832
2218	LOC119625	357.8576	1.712649	0.226321	7.567337	3.81E-14	4.57E-13	189.1881	127.547	187.8475	477.6752	451.3524	713.5354
13817	OVGP1	10.64928	1.710832	0.836563	2.045072	0.040848	0.078594	8.131296	4.379295	2.087194	17.85702	21.39111	10.04979
15364	TMEM26	8.324281	1.710665	0.968228	1.766801	0.077262	0.133706	3.794605	2.189647	5.565851	6.696381	25.66933	6.029877
21112	LOC119621	1.581887	1.71066	1.981389	0.863364	0.387937	0.488505	1.084173	0.547412	0.695731	0	2.139111	5.024897
21468	ALLC	1.911683	1.710394	2.117903	0.807588	0.419328	0.51918	0	0.547412	2.087194	6.696381	2.139111	0
20160	MEIKIN	3.214825	1.709483	1.706304	1.001863	0.31641	0.41725	0	1.094824	3.478657	0	10.69555	4.019918
384	LOC119623	2154.31	1.709233	0.107138	15.9535	2.69E-57	1.86E-55	942.6882	1116.173	969.1538	3232.12	3240.753	3424.97
1893	CCDC102B	249.668	1.70921	0.208259	8.207137	2.27E-16	3.18E-15	106.2489	135.7581	107.1426	457.586	363.6488	327.6233
530	YPEL3	929.6652	1.708162	0.119811	14.25717	4.05E-46	2.03E-44	414.6961	469.6793	423.7004	1433.026	1345.501	1491.39
18344	LOC103226	4.205393	1.707672	1.339061	1.275276	0.202212	0.293055	0.542086	0.547412	4.87012	6.696381	8.556443	4.019918
15186	LOC119619	7.923036	1.707374	0.949393	1.798386	0.072116	0.126248	3.794605	1.642235	5.565851	13.39276	17.11289	6.029877
9526	LOC119627	26.98795	1.706955	0.57323	2.977784	0.002903	0.008103	6.505036	19.15941	11.82743	49.10679	49.19955	26.12947
1320	SCN1B	339.086	1.706327	0.174217	9.794267	1.19E-22	2.40E-21	178.3464	139.59	160.7139	493.3001	487.7172	574.8483
19437	CUNH5orf4	3.513676	1.706072	1.534405	1.111878	0.26619	0.364083	0	2.737059	2.087194	6.696381	8.556443	1.004979
1608	ZNF616	240.8006	1.705397	0.190266	8.963204	3.15E-19	5.21E-18	109.5014	124.2625	104.3597	345.9797	414.9875	345.7129
14982	LOC103248	7.597678	1.705274	0.928174	1.837234	0.066175	0.117426	2.168345	4.379295	4.174388	6.696381	17.11289	11.05477
668	ITPKB	951.2723	1.704878	0.131092	13.00517	1.14E-38	4.55E-37	448.3054	491.0284	400.7413	1513.382	1315.553	1538.624
8716	LOC103220	28.02002	1.704709	0.533001	3.198321	0.001382	0.004216	9.215468	19.15941	11.1317	53.57105	27.80844	47.23404
12035	LOC103228	14.96448	1.704627	0.710297	2.399877	0.016401	0.036228	4.878777	4.926706	11.1317	31.24978	23.53022	14.06971
17555	LOC119627	4.884331	1.703786	1.211578	1.406253	0.159649	0.24177	0.542086	1.642235	4.87012	8.928508	4.278221	9.044815
15263	LOC103240	9.566304	1.702928	0.953676	1.785646	0.074157	0.129166	4.878777	2.737059	5.565851	13.39276	27.80844	3.014938
16721	LOC119625	5.111066	1.702608	1.106653	1.53852	0.123922	0.197025	1.084173	3.284471	2.782925	8.928508	8.556443	6.029877
20334	DBX1	2.347129	1.701685	1.751414	0.971607	0.331246	0.433077	0	0.547412	2.782925	4.464254	4.278221	2.009959
14798	LOC103227	7.371169	1.701293	0.908199	1.87326	0.061032	0.109646	4.336691	3.284471	2.782925	8.928508	12.83466	12.05975
4023	LOC119624	81.08545	1.700809	0.317605	5.355115	8.55E-08	5.65E-07	46.61943	29.01283	38.26523	122.767	143.3204	106.5278
16138	LOC103223	6.034488	1.700257	1.036961	1.639654	0.101077	0.166522	1.084173	3.284471	4.174388	8.928508	10.69555	8.039836
976	CBR3	357.9177	1.69955	0.152981	11.10954	1.13E-28	3.07E-27	172.9256	171.8873	160.0182	566.9603	539.0559	536.659
522	MTSS1	1859.892	1.699338	0.118435	14.34832	1.09E-46	5.56E-45	815.2979	996.8369	816.0929	2761.141	2753.035	3016.948
7708	LOC103215	27.76472	1.698911	0.486736	3.490418	0.000482	0.001663	16.26259	10.40082	12.52316	42.41041	42.78221	42.20914
10564	LOC103242	20.79931	1.69889	0.622316	2.729947	0.006334	0.015941	4.878777	10.40082	13.91463	42.41041	32.08666	21.10457
16931	LOC119627	5.616486	1.698814	1.131031	1.502005	0.133096	0.208992	1.626259	3.284471	2.782925	11.16063	12.83466	2.009959
11549	MORN5	16.63445	1.698447	0.678219	2.504274	0.01227	0.028245	11.38381	3.284471	9.044508	24.5534	21.39111	30.14938
14173	LOC103233	9.303845	1.697439	0.856543	1.981733	0.047509	0.089104	2.710432	6.02153	4.174388	15.62489	19.252	8.039836
13065	LOC119619	15.39029	1.697175	0.773618	2.193817	0.028249	0.057472	4.878777	11.49565	4.87012	24.5534	38.50399	8.039836
7586	LOC119627	30.47979	1.696529	0.48052	3.530611	0.000415	0.001453	17.34676	12.04306	13.2189	58.0353	47.06043	35.17428
1536	LRMDA	227.9284	1.696341	0.185574	9.14107	6.18E-20	1.07E-18	111.6698	102.9134	107.1426	343.7476	376.4835	325.6133
8150	LOC103214	25.04261	1.696142	0.504115	3.364591	0.000767	0.002501	10.29964	13.13788	11.82743	40.17829	40.6431	34.1693
14026	LOC119623	10.79737	1.695794	0.844326	2.008458	0.044595	0.084534	3.794605	3.831883	7.653045	29.01765	6.417332	14.06971
12792	LOC119628	11.7746	1.695635	0.756626	2.241047	0.025023	0.052003	5.96295	4.379295	6.261582	11.16063	27.80844	15.07469
8594	DNAH1	33.58185	1.694997	0.524559	3.231282	0.001232	0.003812	8.131296	17.51718	21.56767	55.80317	66.31243	32.15934
18785	TDGF1	3.983322	1.694836	1.389313	1.219909	0.222499	0.314887	0	2.189647	3.478657	8.928508	4.278221	5.024897

14856	YJEFN3	8.627214	1.694232	0.91024	1.861302	0.062702	0.112205	3.252518	3.831883	4.87012	13.39276	21.39111	5.024897
5840	NEFM	70.75474	1.694187	0.398323	4.253295	2.11E-05	9.59E-05	24.39389	38.31883	36.87376	82.5887	168.9897	73.3635
17042	OXCT2	8.715304	1.693669	1.138841	1.487187	0.136965	0.213647	3.252518	0	9.044508	17.85702	17.11289	5.024897
17039	CDX1	8.989266	1.693572	1.138682	1.487309	0.136933	0.213638	0	3.831883	9.044508	4.464254	23.53022	13.06473
463	RALGDS	1759.427	1.693416	0.113344	14.94053	1.80E-50	1.03E-48	874.9274	856.6995	763.2173	2629.446	2502.759	2929.515
19739	LOC10324(	3.323307	1.692766	1.593593	1.062233	0.28813	0.388063	2.710432	1.094824	0.695731	11.16063	4.278221	0
14833	TTLL9	8.694727	1.692612	0.907582	1.864969	0.062186	0.111455	3.794605	2.189647	6.261582	22.32127	8.556443	9.044815
22158	NBPF4	1.130939	1.691217	2.421006	0.69856	0.484827	0.581692	1.084173	0.547412	0	0	2.139111	3.014938
11973	TMEM116	13.37807	1.690903	0.70125	2.41127	0.015897	0.035298	8.131296	6.568942	4.174388	15.62489	25.66933	20.09959
713	RBPMS	847.8029	1.690098	0.132987	12.70873	5.29E-37	1.97E-35	372.4133	460.3733	370.1291	1323.651	1257.797	1302.453
18213	LOC10323:	5.643868	1.68986	1.300753	1.29914	0.193896	0.283024	0	4.379295	3.478657	11.16063	12.83466	2.009959
17857	LOC10321‹	4.453133	1.689576	1.235029	1.368045	0.171298	0.255024	1.626259	0.547412	4.174388	8.928508	6.417332	5.024897
303	CLIP2	6960.616	1.688944	0.097092	17.39535	8.95E-68	7.85E-66	3367.441	3334.833	3187.841	10419.57	9799.266	11654.75
22574	LOC11962(	1.130051	1.688876	2.683872	0.629268	0.529173	0.623186	1.626259	0	0	0	2.139111	3.014938
11001	LOC11962:	17.36669	1.687278	0.641839	2.628819	0.008568	0.020706	10.29964	7.116354	6.957314	20.08914	40.6431	19.09461
18554	LOC10324:	5.151228	1.687044	1.357674	1.242599	0.214016	0.306651	0	1.094824	6.261582	6.696381	12.83466	4.019918
22881	LOC10322!	0.938925	1.686357	2.897031	0.582098	0.560501	0.65115	0	0	1.391463	2.232127	0	2.009959
22882	LOC10323:	0.938925	1.686357	2.897031	0.582098	0.560501	0.65115	0	0	1.391463	2.232127	0	2.009959
22883	LOC11962:	0.938925	1.686357	2.897031	0.582098	0.560501	0.65115	0	0	1.391463	2.232127	0	2.009959
22884	LOC11962‹	0.938925	1.686357	2.897031	0.582098	0.560501	0.65115	0	0	1.391463	2.232127	0	2.009959
574	EFNB1	3069.729	1.68477	0.121636	13.85092	1.26E-43	5.82E-42	1594.276	1275.47	1502.78	4734.341	4184.1	5127.405
19719	LOC10322(	2.784575	1.684765	1.58044	1.06601	0.286419	0.386148	1.626259	2.189647	0	4.464254	6.417332	2.009959
21190	LOC10321ɛ	1.618028	1.684036	1.976487	0.852035	0.394195	0.494557	1.626259	0	0.695731	2.232127	2.139111	3.014938
20706	LOC11962:	1.619803	1.681884	1.829255	0.919437	0.357867	0.459475	0.542086	1.094824	0.695731	2.232127	2.139111	3.014938
410	STON2	1654.027	1.681701	0.108182	15.54511	1.72E-54	1.11E-52	742.6583	862.721	753.4771	2470.965	2498.481	2595.862
3118	SYNGR4	120.0108	1.681537	0.268746	6.256964	3.93E-10	3.35E-09	42.82482	65.14201	64.00729	167.4095	179.6853	200.9959
13222	LOC10321(	12.81347	1.681186	0.776752	2.164378	0.030435	0.061195	9.215468	6.568942	2.087194	24.5534	21.39111	13.06473
11174	MYT1	25.95249	1.68101	0.649941	2.586402	0.009698	0.023074	7.047123	8.211177	21.56767	33.4819	66.31243	19.09461
10643	LOC11962(	18.9971	1.680863	0.62069	2.708053	0.006768	0.016905	5.420864	9.306001	12.52316	20.08914	38.50399	28.13943
16674	LOC10323!	5.587787	1.680325	1.087175	1.545588	0.122204	0.19483	2.168345	2.189647	3.478657	6.696381	14.97377	4.019918
7321	LOC10324:	36.30271	1.680191	0.463305	3.626535	0.000287	0.001043	11.9259	18.06459	22.2634	37.94616	66.31243	61.30375
667	SCARA3	2263.381	1.679596	0.129141	13.00596	1.13E-38	4.51E-37	1004.486	1262.332	964.9794	3424.083	3180.858	3743.549
7931	RAB37	32.61471	1.678833	0.489385	3.430496	0.000602	0.00202	20.0572	15.32753	10.43597	55.80317	59.8951	34.1693
561	PDLIM2	1738.796	1.678823	0.119424	14.05767	6.91E-45	3.28E-43	894.9846	732.437	857.141	2749.98	2421.473	2776.758
19568	FAM110D	3.795555	1.678568	1.541382	1.089002	0.276153	0.37518	0	0.547412	4.87012	8.928508	6.417332	2.009959
19251	LOC10324:	3.501368	1.678315	1.466286	1.144603	0.252374	0.348504	0	2.189647	2.782925	4.464254	8.556443	3.014938
6612	LOC11962:	38.01524	1.67818	0.430459	3.898583	9.68E-05	0.000389	17.34676	23.53871	13.2189	55.80317	59.8951	58.28881
10420	GAL3ST4	35.17502	1.677425	0.607417	2.761571	0.005752	0.014676	6.505036	10.40082	33.39511	60.26743	66.31243	34.1693
7205	LOC11962:	33.86743	1.677264	0.457558	3.665684	0.000247	0.00091	14.09425	18.612	15.30609	69.19594	42.78221	43.21412
307	LGMN	14213.73	1.675916	0.096778	17.31714	3.49E-67	3.02E-65	6636.221	7211.056	6483.521	21816.81	19825.28	23309.49
451	TRPS1	5602.307	1.674641	0.110995	15.08748	1.96E-51	1.15E-49	2433.426	3085.76	2498.371	8410.654	8513.66	8671.968
10361	ZNF665	18.58346	1.674548	0.603676	2.773921	0.005539	0.014211	9.215468	11.49565	5.565851	29.01765	32.08666	24.11951
15515	LOC10323!	7.942193	1.674468	0.962007	1.740598	0.081754	0.140086	1.626259	2.189647	7.653045	11.16063	14.97377	10.04979
349	BCAM	3047.179	1.672417	0.101732	16.43939	9.99E-61	7.61E-59	1453.876	1435.314	1480.516	4629.431	4226.883	5057.057
22329	LOC10322:	1.426901	1.672179	2.494639	0.670309	0.502661	0.59847	0	0	2.087194	4.464254	0	2.009959
22217	LOC10321!	1.18347	1.671662	2.424073	0.689609	0.49044	0.586864	1.084173	0.547412	0	4.464254	0	1.004979
14317	LOC11962ɛ	14.80154	1.671659	0.853688	1.958161	0.050211	0.093234	4.878777	1.094824	15.30609	26.78552	25.66933	15.07469
19168	THEMIS	3.014811	1.671646	1.443711	1.157881	0.246913	0.342455	1.084173	0.547412	2.782925	2.232127	6.417332	5.024897
650	CPT1A	1214.571	1.671645	0.12724	13.13768	2.00E-39	8.19E-38	602.258	623.5021	516.2327	1863.826	1670.645	2010.964
12644	LOC10323!	12.14105	1.671109	0.73554	2.271947	0.02309	0.048548	4.336691	6.568942	6.261582	20.08914	23.53022	12.05975
16822	DAZL	7.287669	1.671059	1.098367	1.521403	0.128159	0.202538	2.710432	3.284471	4.174388	11.16063	21.39111	1.004979
15372	LOC10323:	6.966466	1.670621	0.94644	1.765163	0.077536	0.134095	4.336691	2.737059	2.782925	8.928508	14.97377	8.039836
4073	LOC11962‹	71.95348	1.670011	0.314096	5.31689	1.06E-07	6.89E-07	39.5723	35.58177	27.82925	102.6778	115.512	110.5477
6372	LOC10324(	48.36739	1.668792	0.417898	3.993304	6.52E-05	0.000272	15.7205	30.10765	22.95914	82.5887	85.56443	53.26391
8775	LOC11961ɛ	24.83426	1.667941	0.524388	3.180741	0.001469	0.004451	9.757555	15.32753	10.43597	31.24978	47.06043	35.17428
8532	STAG3	47.6399	1.667464	0.51371	3.245922	0.001171	0.003648	9.757555	20.25424	38.26523	80.35657	91.98176	45.22408
14826	LOC10322!	7.684207	1.667321	0.892868	1.867377	0.061849	0.110903	3.252518	4.926706	2.782925	13.39276	10.69555	11.05477
17078	LOC11962!	6.259908	1.666971	1.123123	1.484229	0.137748	0.21443	0.542086	4.926706	3.478657	4.464254	17.11289	7.034856
18497	FNDC8	4.946966	1.666578	1.329094	1.25392	0.209871	0.301974	0	1.642235	5.565851	11.16063	4.278221	7.034856
14940	LOC11961ɛ	8.221163	1.665854	0.901935	1.846977	0.06475	0.115205	5.420864	2.737059	3.478657	20.08914	8.556443	9.044815
12434	MMP17	26.81049	1.665676	0.719591	2.314754	0.020626	0.044101	6.505036	4.379295	27.82925	24.5534	68.45154	29.1444
13965	LBHD2	10.19482	1.665496	0.824921	2.018976	0.04349	0.082792	6.505036	3.831883	4.174388	8.928508	25.66933	12.05975
677	QPCT	651.5782	1.665208	0.128733	12.93538	2.84E-38	1.12E-36	332.2989	295.6024	310.2962	984.368	949.7651	1037.139
3048	LOC10323‹	135.4412	1.663931	0.262317	6.343199	2.25E-10	1.96E-09	55.29281	76.63765	63.31155	158.481	243.8586	215.0656
22946	LOC11962(	0.923422	1.66337	2.911977	0.571217	0.567853	0.657822	0	0	1.391463	0	2.139111	2.009959
22947	MRM3	0.923422	1.66337	2.911977	0.571217	0.567853	0.657822	0	0	1.391463	0	2.139111	2.009959
22948	LOC10322:	0.923422	1.66337	2.911977	0.571217	0.567853	0.657822	0	0	1.391463	0	2.139111	2.009959
22949	LOC10321!	0.923422	1.66337	2.911977	0.571217	0.567853	0.657822	0	0	1.391463	0	2.139111	2.009959
14311	LOC10323!	8.855308	1.663354	0.849156	1.958832	0.050132	0.093129	3.252518	3.284471	6.261582	11.16063	17.11289	12.05975
520	PPP1R3B	2176.384	1.663286	0.115721	14.37323	7.62E-47	3.90E-45	1039.722	1159.966	934.3672	3316.941	3088.876	3518.433
1092	SLC12A5	332.7254	1.662839	0.157741	10.5416	5.55E-26	1.35E-24	164.7943	156.5598	158.6268	479.9073	504.8301	531.6341
11506	LOC11962‹	16.14104	1.662681	0.661492	2.513533	0.011953	0.027617	5.420864	6.568942	11.1317	24.5534	32.08666	17.08465
15382	ANKRD62	12.07803	1.662243	0.942824	1.763047	0.077893	0.134623	1.626259	7.116354	8.348776	24.5534	27.80844	3.014938
23300	LOC11962:	0.975953	1.662236	3.234894	0.513846	0.60736	0.69299	0	0	1.391463	4.464254	0	0

940	OAS1	575.9695	1.662152	0.14758	11.26273	2.00E-29	5.67E-28	301.4	285.2016	244.2017	828.1191	832.114	964.7803
300	EMC3	5792.321	1.661926	0.095116	17.47263	2.32E-68	2.05E-66	2939.734	2517.547	2888.677	8839.223	8800.301	8768.446
429	VASH1	2537.235	1.660746	0.108409	15.31925	5.69E-53	3.52E-51	1246.257	1183.504	1232.14	3727.652	3529.533	4304.327
1571	RRAGD	227.9516	1.660524	0.183546	9.046929	1.47E-19	2.49E-18	108.4173	117.6935	102.2725	348.2118	344.3968	346.7179
16276	KLK8	7.498154	1.660113	1.029543	1.612476	0.106858	0.174541	1.084173	2.737059	6.957314	8.928508	19.252	6.029877
22302	LOC11961ᵹ	1.167079	1.659397	2.460096	0.674525	0.499977	0.595969	1.626259	0	0	2.232127	2.139111	1.004979
22303	ZNF516-DT	1.167079	1.659397	2.460096	0.674525	0.499977	0.595969	1.626259	0	0	2.232127	2.139111	1.004979
6410	LOC10324ᵹ	41.14756	1.659279	0.417457	3.97473	7.05E-05	0.000292	16.26259	24.08612	18.78475	78.12444	51.33866	58.28881
20358	LOC10322ᵹ	2.353212	1.65923	1.713932	0.968084	0.333002	0.434859	1.084173	2.189647	0	6.696381	2.139111	2.009959
16394	SNTG2	6.362306	1.658628	1.043713	1.589161	0.112024	0.18165	2.168345	3.284471	3.478657	13.39276	12.83466	3.014938
4519	ACKR4	74.44388	1.658575	0.331623	5.001391	5.69E-07	3.35E-06	27.10432	39.41365	41.04815	91.51721	139.0422	108.5378
15920	LOC10322ᵹ	6.328473	1.657796	0.986137	1.681101	0.092743	0.154873	2.168345	2.737059	4.174388	11.16063	10.69555	7.034856
6282	LOC10324ᵹ	42.15234	1.65686	0.410406	4.037129	5.41E-05	0.000229	18.43094	24.63353	17.39328	58.0353	79.14709	55.27387
3691	INKA2	102.1275	1.656801	0.293255	5.649697	1.61E-08	1.16E-07	56.37698	39.41365	50.78839	158.481	186.1026	121.6025
12893	LOC10323ᵹ	13.8805	1.656341	0.746068	2.220094	0.026412	0.054462	8.673382	3.831883	7.653045	8.928508	32.08666	22.10955
21147	LOC11962ᵹ	2.334821	1.656221	1.929091	0.85855	0.390589	0.49103	0	0	3.478657	2.232127	4.278221	4.019918
7969	MCF2L2	29.97826	1.655544	0.484179	3.419283	0.000628	0.002095	10.29964	14.23271	18.78475	53.57105	42.78221	40.19918
20770	LOC10323ᵹ	2.143032	1.655534	1.823587	0.907845	0.36396	0.465859	1.626259	0	1.391463	6.696381	2.139111	1.004979
8488	LOC10323ᵹ	27.30811	1.65489	0.507918	3.258185	0.001121	0.003511	16.26259	10.94824	11.82743	40.17829	53.47777	31.15436
20392	CNDP1	2.633671	1.653867	1.717495	0.962953	0.335571	0.437483	0	1.642235	2.087194	8.928508	2.139111	1.004979
14215	LOC10323ᵹ	9.121744	1.653865	0.83769	1.974317	0.048346	0.090417	4.336691	2.737059	6.261582	15.62489	10.69555	15.07469
18796	LOC10322ᵹ	4.151773	1.653548	1.356736	1.218769	0.222932	0.315314	2.168345	1.094824	2.782925	0	12.83466	6.029877
17204	LCN15	5.455806	1.652365	1.126195	1.467211	0.142319	0.219922	3.252518	1.642235	2.782925	15.62489	6.417332	3.014938
9837	CHRND	20.39059	1.651763	0.56998	2.897931	0.003756	0.010152	7.589209	9.306001	12.52316	35.71403	32.08666	25.12449
22671	LOC11962ᵹ	1.151577	1.651529	2.70054	0.611555	0.540832	0.634203	1.626259	0	0	0	4.278221	1.004979
4112	TMEM19ᵹ	68.14196	1.65054	0.312306	5.285009	1.26E-07	8.13E-07	33.60935	30.65506	34.78657	107.1421	94.12087	108.5378
17456	CYP2E1	4.709558	1.649625	1.163399	1.417936	0.156209	0.237902	1.084173	2.189647	3.478657	8.928508	8.556443	4.019918
9061	CDH20	33.59635	1.649432	0.533395	3.092326	0.001986	0.005827	16.80468	19.15941	11.82743	55.80317	74.86887	23.11453
21179	LOC10321ᵹ	2.077661	1.649224	1.932165	0.853563	0.393347	0.49375	0	1.642235	1.391463	0	6.417332	3.014938
18136	LOC11961ᵹ	4.988468	1.649221	1.253956	1.315215	0.188438	0.276225	3.794605	1.094824	2.087194	13.39276	8.556443	1.004979
11807	LOC10323ᵹ	25.00206	1.648779	0.673615	2.44766	0.014379	0.032375	11.9259	13.13788	10.43597	66.96381	38.50399	9.044815
22328	F9	1.169742	1.648656	2.458886	0.670489	0.502546	0.598377	0	1.642235	0	2.232127	2.139111	1.004979
502	GABRA2	3040.151	1.648355	0.112718	14.62375	1.98E-48	1.05E-46	1349.795	1684.386	1377.548	4495.504	4601.227	4732.448
665	PPP2R2B	1135.004	1.648233	0.126403	13.03956	7.29E-39	2.91E-37	538.8339	624.0495	483.5333	1691.952	1721.984	1749.669
1225	LOC11962ᵹ	371.786	1.647985	0.163417	10.08454	6.47E-24	1.40E-22	171.8414	172.4347	196.892	589.2815	496.2737	603.9927
22041	LOC10323ᵹ	1.828739	1.647982	2.295141	0.718031	0.472738	0.570155	2.710432	0	0	2.232127	0	6.029877
21576	SST	1.883872	1.64769	2.078553	0.79271	0.427947	0.527297	0	0	2.782925	2.232127	4.278221	2.009959
19785	UTS2	2.583448	1.647545	1.560476	1.055796	0.291061	0.391098	0.542086	0.547412	2.782925	4.464254	2.139111	5.024897
16773	LOC11962ᵹ	5.929168	1.647534	1.076909	1.529874	0.126048	0.199785	2.710432	2.189647	3.478657	15.62489	8.556443	3.014938
20760	LOC11962ᵹ	2.547472	1.646711	1.809837	0.909867	0.362893	0.464716	1.626259	0	2.087194	0	8.556443	3.014938
407	PML	2991.023	1.64637	0.105567	15.59556	7.80E-55	5.10E-53	1338.411	1562.861	1446.426	4363.808	4342.395	4892.24
10140	LOC10321ᵹ	23.22225	1.646267	0.581801	2.829604	0.004661	0.012219	9.757555	6.02153	18.08902	37.94616	36.36488	31.15436
11335	LOC11962ᵹ	16.37149	1.645791	0.6456	2.549242	0.010796	0.02532	4.878777	10.40082	8.348776	24.5534	29.94755	20.09959
3981	FAM78A	80.64138	1.645616	0.305388	5.3886	7.10E-08	4.74E-07	34.69353	36.12918	45.91827	138.3919	126.2075	102.5079
7132	LOC10321ᵹ	36.56843	1.6453	0.446423	3.685514	0.000228	0.000851	16.80468	23.53871	12.52316	49.10679	64.17332	53.26391
550	LGR4	3720.589	1.643778	0.116312	14.13253	2.39E-45	1.16E-43	1725.461	2070.859	1616.184	5299.069	5666.504	5945.459
12317	CD226	13.52941	1.643586	0.701645	2.342475	0.019156	0.041349	4.336691	7.663765	7.653045	15.62489	27.80844	18.08963
21987	CRCT1	1.167967	1.643	2.251572	0.729712	0.465566	0.562915	1.084173	0.547412	0	2.232127	2.139111	1.004979
19369	FBXO39	2.824798	1.642294	1.45984	1.124982	0.260597	0.357683	1.626259	1.642235	0.695731	6.696381	4.278221	2.009959
5443	LOC11962ᵹ	59.2934	1.642134	0.369665	4.442222	8.90E-06	4.35E-05	20.0572	38.31883	27.82925	82.5887	100.5382	86.42823
3438	LOC11962ᵹ	144.917	1.640677	0.277529	5.911733	3.39E-09	2.62E-08	50.95612	85.39624	73.74753	225.4448	263.1106	170.8465
22706	LOC11962ᵹ	1.154239	1.640225	2.698964	0.607724	0.543371	0.63617	0	1.642235	0	0	4.278221	1.004979
13598	LOC10323ᵹ	10.2639	1.639937	0.784674	2.08996	0.036621	0.071597	7.047123	4.379295	3.478657	15.62489	14.97377	16.07967
21996	TRNAD-GU	1.168855	1.639631	2.251216	0.728331	0.466411	0.563692	0.542086	1.094824	0	2.232127	2.139111	1.004979
13425	LOC10324ᵹ	10.94744	1.639521	0.772582	2.122133	0.033827	0.066985	5.96295	7.116354	2.782925	15.62489	17.11289	17.08465
14348	FXYD1	15.1339	1.637688	0.838675	1.95271	0.050854	0.094226	3.252518	2.189647	16.69755	26.78552	27.80844	14.06971
6483	ENKUR	45.12611	1.637483	0.414877	3.946908	7.92E-05	0.000325	15.7205	27.37059	22.2634	84.82083	66.31243	54.26889
19621	GPX5	2.81107	1.637221	1.512656	1.082348	0.279098	0.378137	0.542086	2.737059	0.695731	4.464254	6.417332	2.009959
439	ATP6V1B2	3194.794	1.637131	0.107633	15.21031	3.02E-52	1.83E-50	1654.99	1507.572	1504.171	4660.681	4470.741	5370.61
22327	LOC11962ᵹ	1.152464	1.636869	2.441389	0.670466	0.502561	0.598377	1.084173	0.547412	0	0	4.278221	1.004979
17708	TMEM229ᵹ	4.924872	1.63676	1.183366	1.38314	0.166622	0.250135	1.084173	3.831883	2.087194	6.696381	12.83466	3.014938
1626	HSD17B14	278.4908	1.636717	0.183712	8.909169	5.14E-19	8.41E-18	150.7	119.8832	135.6676	468.7467	382.9008	413.0466
13048	LOC11962ᵹ	11.66529	1.63617	0.74501	2.196173	0.02808	0.057211	5.420864	6.568942	4.87012	13.39276	25.66933	14.06971
11177	SLC25A41	17.86527	1.63615	0.632693	2.586008	0.009709	0.023094	5.420864	9.306001	11.1317	29.01765	34.22577	18.08963
14310	CHRNA3	11.59431	1.634857	0.834514	1.959053	0.050107	0.093088	4.878777	2.189647	9.740239	22.32127	21.39111	9.044815
7061	APOC2	36.74362	1.634775	0.440947	3.707416	0.000209	0.000788	18.97302	13.6853	20.87194	49.10679	70.59065	47.23404
2527	LOC10323ᵹ	147.3656	1.634721	0.230703	7.085832	1.38E-12	1.45E-11	77.51835	62.40495	75.13899	209.8199	250.2759	209.0357
17289	LOC10323ᵹ	5.396458	1.634401	1.126506	1.450859	0.146819	0.225761	1.626259	3.284471	2.782925	6.696381	14.97377	3.014938
14437	TMEM253	11.633	1.634105	0.843588	1.93709	0.052734	0.097108	1.626259	6.568942	9.044508	8.928508	23.53022	20.09959
6589	LOC11962ᵹ	41.07667	1.633513	0.418293	3.905187	9.42E-05	0.00038	21.68345	20.80165	17.39328	80.35657	44.92132	61.30375
15414	NGB	9.289667	1.633384	0.929094	1.758039	0.078741	0.135807	6.505036	3.284471	3.478657	29.01765	6.417332	7.034856
14128	LOC10322ᵹ	9.865423	1.632584	0.820985	1.988568	0.046749	0.087968	5.96295	2.737059	5.565851	15.62489	19.252	10.04979
21162	LOC11962ᵹ	2.80159	1.632455	1.906075	0.856448	0.39175	0.49214	0.542086	2.737059	0.695731	0	12.83466	0
23034	LOC11962ᵹ	0.96045	1.632428	2.934856	0.556221	0.57806	0.667089	0	0	1.391463	2.232127	2.139111	0

23035	LOC11962(	0.96045	1.632428	2.934856	0.556221	0.57806	0.667089	0	0	1.391463	2.232127	2.139111	0
23036	LOC10323:	0.96045	1.632428	2.934856	0.556221	0.57806	0.667089	0	0	1.391463	2.232127	2.139111	0
23037	LOC11962:	0.96045	1.632428	2.934856	0.556221	0.57806	0.667089	0	0	1.391463	2.232127	2.139111	0
23038	LOC10324:	0.96045	1.632428	2.934856	0.556221	0.57806	0.667089	0	0	1.391463	2.232127	2.139111	0
6124	ILDR1	45.73087	1.631875	0.397326	4.107143	4.01E-05	0.000174	22.76763	21.89647	21.56767	69.19594	87.70354	51.25395
452	EPAS1	1753.26	1.631501	0.108303	15.06429	2.78E-51	1.64E-49	853.786	916.9148	796.6124	2707.57	2474.951	2769.723
20317	TCN1	2.129305	1.631359	1.672588	0.97535	0.329387	0.431006	0.542086	1.094824	1.391463	4.464254	4.278221	1.004979
1490	SPTSSB	270.2121	1.631058	0.175993	9.267756	1.90E-20	3.39E-19	134.9795	129.1892	132.8847	401.7829	372.2053	450.2308
2412	AK5	168.058	1.630121	0.22464	7.256607	3.97E-13	4.37E-12	72.63957	98.53413	74.44326	249.9982	269.5279	243.205
17125	LOC10323(	7.811002	1.630052	1.103445	1.477239	0.139612	0.216732	3.794605	0.547412	6.957314	11.16063	21.39111	3.014938
18698	LOC10323(	3.301354	1.62934	1.325693	1.229047	0.219054	0.311453	1.084173	1.642235	2.087194	6.696381	4.278221	4.019918
14360	DNAAF6	11.4494	1.629245	0.835561	1.949882	0.05119	0.09477	6.505036	7.116354	2.782925	11.16063	32.08666	9.044815
1814	LOC11962!	1381.054	1.629148	0.193538	8.417727	3.84E-17	5.63E-16	859.2069	477.3431	685.9911	2120.521	2425.751	1717.51
289	PLA2R1	6283.908	1.628632	0.092381	17.6296	1.46E-69	1.34E-67	3159.279	3174.989	2881.024	9113.774	9294.436	10079.94
20504	AADACL4	2.587163	1.628445	1.722748	0.94526	0.344526	0.446704	0	1.642235	2.087194	2.232127	8.556443	1.004979
7176	APOC4	39.92304	1.627892	0.443217	3.672905	0.00024	0.000888	19.51511	13.13788	25.74206	58.0353	74.86887	48.23901
12367	LOC11962!	12.4229	1.627885	0.699049	2.328712	0.019874	0.04272	6.505036	5.474118	6.261582	20.08914	17.11289	19.09461
12956	LOC11962(	13.34842	1.627256	0.735646	2.212009	0.026966	0.055326	8.673382	4.379295	6.261582	15.62489	32.08666	13.06473
22514	CAVIN2	1.432924	1.627067	2.551197	0.637766	0.523626	0.61825	0	0	2.087194	2.232127	4.278221	0
22515	LOC10322!	1.432924	1.627067	2.551197	0.637766	0.523626	0.61825	0	0	2.087194	2.232127	4.278221	0
22518	FAM243A	1.432924	1.627067	2.551197	0.637766	0.523626	0.61825	0	0	2.087194	2.232127	4.278221	0
2152	RAPGEF3	203.866	1.627031	0.21188	7.679031	1.60E-14	1.98E-13	113.8381	79.92212	105.7512	292.4086	318.7275	312.5486
13987	CFAP221	10.24158	1.626774	0.808233	2.012753	0.044141	0.083898	5.420864	6.02153	3.478657	8.928508	23.53022	14.06971
11888	LOC10324:	15.74619	1.626265	0.669719	2.428279	0.015171	0.033926	6.505036	11.49565	4.87012	31.24978	19.252	21.10457
22181	MHC-DQA:	1.905397	1.624818	2.339514	0.694511	0.487362	0.584101	0	0	2.782925	2.232127	6.417332	0
16678	LRRN2	6.309879	1.624786	1.0514	1.545354	0.122261	0.194885	2.168345	1.642235	5.565851	4.464254	14.97377	9.044815
21711	LOC11962(	1.659138	1.624579	2.092514	0.776377	0.437527	0.535749	1.084173	0	1.391463	4.464254	0	3.014938
3353	LOC11962:	95.27141	1.624131	0.271161	5.989539	2.10E-09	1.67E-08	41.74065	45.98259	52.17985	147.3204	149.7377	134.6672
18539	LOC10323(	3.75655	1.62403	1.303552	1.24585	0.21282	0.305184	2.168345	0.547412	2.782925	4.464254	8.556443	4.019918
20437	SLC15A5	2.113802	1.623727	1.6982	0.956146	0.338998	0.440978	0.542086	1.094824	1.391463	2.232127	6.417332	1.004979
14567	LOC11962:	9.876771	1.622981	0.848813	1.912059	0.055869	0.101961	2.710432	6.02153	5.565851	13.39276	23.53022	8.039836
20389	CES4A	2.552442	1.622951	1.684739	0.963325	0.335385	0.43731	0.542086	0.547412	2.782925	0	6.417332	5.024897
6093	PPFIBP2	62.95646	1.62283	0.393372	4.125429	3.70E-05	0.000161	23.8518	33.93953	34.09084	80.35657	141.1813	64.31869
9265	LOC11962‹	30.08073	1.622354	0.533548	3.040689	0.00236	0.006773	16.26259	7.663765	20.17621	40.17829	62.03421	34.1693
12760	CARNS1	15.64809	1.621368	0.721226	2.248073	0.024572	0.051194	7.047123	7.116354	8.348776	31.24978	32.08666	8.039836
948	GUCY1B1	1224.52	1.620782	0.14456	11.21184	3.57E-29	1.00E-27	536.6655	736.2689	528.7558	1859.362	1861.026	1825.043
449	PLEKHO2	1912.309	1.620527	0.107248	15.11008	1.39E-51	8.23E-50	998.5231	913.6303	905.8422	2850.426	2691.001	3114.431
5893	LOC11962(	60.20588	1.620091	0.384028	4.218673	2.46E-05	0.000111	34.15144	18.06459	36.17803	91.51721	106.9555	74.36848
426	AHNAK2	22310.7	1.620011	0.105574	15.34476	3.84E-53	2.40E-51	10497.5	11381.79	10985.6	32365.84	30383.93	38249.52
11050	CHRDL2	33.03255	1.619842	0.619295	2.615624	0.008906	0.021428	13.01007	8.758589	26.43779	58.0353	74.86887	17.08465
528	PLEKHH2	3972.964	1.619778	0.113425	14.28056	2.89E-46	1.46E-44	1922.78	2226.324	1701.759	5955.315	6008.762	6022.842
1065	LOC10321!	910.391	1.619618	0.151757	10.67243	1.37E-26	3.42E-25	491.6723	369.503	482.8376	1285.705	1274.91	1557.718
13984	MCF2	12.31237	1.618473	0.803899	2.013279	0.044085	0.083811	4.336691	7.116354	6.261582	22.32127	27.80844	6.029877
26638	LOC11962(	0.447754	1.617593	4.067273	0.397709	0.690844	NA	0	0.547412	0	0	2.139111	0
26643	LOC11962!	0.447754	1.617593	4.067273	0.397709	0.690844	NA	0	0.547412	0	0	2.139111	0
26651	LOC11962(	0.487976	1.617593	4.067273	0.397709	0.690844	NA	0	0	0.695731	2.232127	0	0
26695	LOC11961(	0.463256	1.617593	4.067273	0.397709	0.690844	NA	0	0.547412	0	2.232127	0	0
26705	LOC11962:	0.472474	1.617593	4.067273	0.397709	0.690844	NA	0	0	0.695731	0	2.139111	0
26707	LOC11962:	0.472474	1.617593	4.067273	0.397709	0.690844	NA	0	0	0.695731	0	2.139111	0
26730	LOC11961!	0.463256	1.617593	4.067273	0.397709	0.690844	NA	0	0.547412	0	2.232127	0	0
26873	LOC11962‹	0.487976	1.617593	4.067273	0.397709	0.690844	NA	0	0	0.695731	2.232127	0	0
26879	LOC10323(	0.447754	1.617593	4.067273	0.397709	0.690844	NA	0	0.547412	0	0	2.139111	0
26910	LOC10322:	0.472474	1.617593	4.067273	0.397709	0.690844	NA	0	0	0.695731	0	2.139111	0
26950	TRNAN-GL	0.472474	1.617593	4.067273	0.397709	0.690844	NA	0	0	0.695731	0	2.139111	0
26958	TRNAN-GL	0.462369	1.617593	4.067273	0.397709	0.690844	NA	0.542086	0	0	2.232127	0	0
26959	TRNAE-CU	0.447754	1.617593	4.067273	0.397709	0.690844	NA	0	0.547412	0	0	2.139111	0
26986	LOC11962‹	0.472474	1.617593	4.067273	0.397709	0.690844	NA	0	0	0.695731	0	2.139111	0
27113	LOC10324:	0.472474	1.617593	4.067273	0.397709	0.690844	NA	0	0	0.695731	0	2.139111	0
27118	LOC10324:	0.487976	1.617593	4.067273	0.397709	0.690844	NA	0	0	0.695731	2.232127	0	0
27156	LOC11962!	0.472474	1.617593	4.067273	0.397709	0.690844	NA	0	0	0.695731	0	2.139111	0
27194	LOC11962!	0.446866	1.617593	4.067273	0.397709	0.690844	NA	0.542086	0	0	0	2.139111	0
27205	LOC11962!	0.463256	1.617593	4.067273	0.397709	0.690844	NA	0	0.547412	0	2.232127	0	0
27254	LOC11962!	0.472474	1.617593	4.067273	0.397709	0.690844	NA	0	0	0.695731	0	2.139111	0
27300	LOC11962!	0.462369	1.617593	4.067273	0.397709	0.690844	NA	0.542086	0	0	2.232127	0	0
27313	TRNAG-GC	0.447754	1.617593	4.067273	0.397709	0.690844	NA	0	0.547412	0	0	2.139111	0
27331	LOC10323!	0.463256	1.617593	4.067273	0.397709	0.690844	NA	0	0.547412	0	2.232127	0	0
27341	LOC11962!	0.487976	1.617593	4.067273	0.397709	0.690844	NA	0	0	0.695731	2.232127	0	0
27391	LOC10322(	0.447754	1.617593	4.067273	0.397709	0.690844	NA	0	0.547412	0	0	2.139111	0
27392	LOC10322(	0.472474	1.617593	4.067273	0.397709	0.690844	NA	0	0	0.695731	0	2.139111	0
27398	LOC11962!	0.487976	1.617593	4.067273	0.397709	0.690844	NA	0	0	0.695731	2.232127	0	0
27402	CCDC54	0.447754	1.617593	4.067273	0.397709	0.690844	NA	0	0.547412	0	0	2.139111	0
27457	LOC11962(	0.472474	1.617593	4.067273	0.397709	0.690844	NA	0	0	0.695731	0	2.139111	0
27513	LOC11962(	0.472474	1.617593	4.067273	0.397709	0.690844	NA	0	0	0.695731	0	2.139111	0
27523	LOC11962(	0.487976	1.617593	4.067273	0.397709	0.690844	NA	0	0	0.695731	2.232127	0	0

27528	LOC11962(	0.472474	1.617593	4.067273	0.397709	0.690844	NA	0	0	0.695731	0	2.139111	0
27551	LOC11962(	0.463256	1.617593	4.067273	0.397709	0.690844	NA	0	0.547412	0	2.232127	0	0
27585	LOC10324;	0.447754	1.617593	4.067273	0.397709	0.690844	NA	0	0.547412	0	0	2.139111	0
27597	OR52Z1	0.472474	1.617593	4.067273	0.397709	0.690844	NA	0	0	0.695731	0	2.139111	0
27600	LOC10324;	0.487976	1.617593	4.067273	0.397709	0.690844	NA	0	0	0.695731	2.232127	0	0
27615	LOC11962(	0.472474	1.617593	4.067273	0.397709	0.690844	NA	0	0	0.695731	0	2.139111	0
27645	TRNAI-AAU	0.487976	1.617593	4.067273	0.397709	0.690844	NA	0	0	0.695731	2.232127	0	0
27661	TRNAA-AG	0.446866	1.617593	4.067273	0.397709	0.690844	NA	0.542086	0	0	0	2.139111	0
27713	LOC11962;	0.462369	1.617593	4.067273	0.397709	0.690844	NA	0.542086	0	0	2.232127	0	0
27815	LOC11962;	0.447754	1.617593	4.067273	0.397709	0.690844	NA	0	0.547412	0	0	2.139111	0
27834	LOC11962;	0.472474	1.617593	4.067273	0.397709	0.690844	NA	0	0	0.695731	0	2.139111	0
27840	LOC10323:	0.462369	1.617593	4.067273	0.397709	0.690844	NA	0.542086	0	0	2.232127	0	0
27869	LOC11962;	0.463256	1.617593	4.067273	0.397709	0.690844	NA	0	0.547412	0	2.232127	0	0
27892	LOC11962;	0.447754	1.617593	4.067273	0.397709	0.690844	NA	0	0.547412	0	0	2.139111	0
27896	LOC11962;	0.487976	1.617593	4.067273	0.397709	0.690844	NA	0	0	0.695731	2.232127	0	0
27933	LOC11962{	0.463256	1.617593	4.067273	0.397709	0.690844	NA	0	0.547412	0	2.232127	0	0
27944	LOC11962;	0.472474	1.617593	4.067273	0.397709	0.690844	NA	0	0	0.695731	0	2.139111	0
27960	LOC11962{	0.472474	1.617593	4.067273	0.397709	0.690844	NA	0	0	0.695731	0	2.139111	0
27965	LOC10324!	0.447754	1.617593	4.067273	0.397709	0.690844	NA	0	0.547412	0	0	2.139111	0
28014	LOC11961{	0.447754	1.617593	4.067273	0.397709	0.690844	NA	0	0.547412	0	0	2.139111	0
28037	LOC11961{	0.447754	1.617593	4.067273	0.397709	0.690844	NA	0	0.547412	0	0	2.139111	0
28083	SPINT3	0.487976	1.617593	4.067273	0.397709	0.690844	NA	0	0	0.695731	2.232127	0	0
28106	DEFB118	0.447754	1.617593	4.067273	0.397709	0.690844	NA	0	0.547412	0	0	2.139111	0
28113	LOC11961{	0.472474	1.617593	4.067273	0.397709	0.690844	NA	0	0	0.695731	0	2.139111	0
28123	LOC11961{	0.463256	1.617593	4.067273	0.397709	0.690844	NA	0	0.547412	0	2.232127	0	0
28157	LOC10321!	0.463256	1.617593	4.067273	0.397709	0.690844	NA	0	0.547412	0	2.232127	0	0
28175	CUNH8orf;	0.463256	1.617593	4.067273	0.397709	0.690844	NA	0	0.547412	0	2.232127	0	0
28192	FSCB	0.487976	1.617593	4.067273	0.397709	0.690844	NA	0	0	0.695731	2.232127	0	0
28205	LOC11961!	0.463256	1.617593	4.067273	0.397709	0.690844	NA	0	0.547412	0	2.232127	0	0
28226	LOC11961!	0.447754	1.617593	4.067273	0.397709	0.690844	NA	0	0.547412	0	0	2.139111	0
28258	LOC11961!	0.472474	1.617593	4.067273	0.397709	0.690844	NA	0	0	0.695731	0	2.139111	0
28267	LOC10321!	0.487976	1.617593	4.067273	0.397709	0.690844	NA	0	0	0.695731	2.232127	0	0
28290	LOC10322!	0.472474	1.617593	4.067273	0.397709	0.690844	NA	0	0	0.695731	0	2.139111	0
28292	LOC11961!	0.472474	1.617593	4.067273	0.397709	0.690844	NA	0	0	0.695731	0	2.139111	0
28322	LOC11961!	0.472474	1.617593	4.067273	0.397709	0.690844	NA	0	0	0.695731	0	2.139111	0
28324	LOC11961!	0.447754	1.617593	4.067273	0.397709	0.690844	NA	0	0.547412	0	0	2.139111	0
28359	HIST3H3	0.462369	1.617593	4.067273	0.397709	0.690844	NA	0.542086	0	0	2.232127	0	0
28392	RGS13	0.447754	1.617593	4.067273	0.397709	0.690844	NA	0	0.547412	0	0	2.139111	0
28425	LOC10323(	0.487976	1.617593	4.067273	0.397709	0.690844	NA	0	0	0.695731	2.232127	0	0
28427	LOC11961!	0.472474	1.617593	4.067273	0.397709	0.690844	NA	0	0	0.695731	0	2.139111	0
28429	LOC10323(	0.463256	1.617593	4.067273	0.397709	0.690844	NA	0	0.547412	0	2.232127	0	0
28455	LOC10323;	0.462369	1.617593	4.067273	0.397709	0.690844	NA	0.542086	0	0	2.232127	0	0
28546	LOC10324;	0.487976	1.617593	4.067273	0.397709	0.690844	NA	0	0	0.695731	2.232127	0	0
28550	LOC11962(	0.462369	1.617593	4.067273	0.397709	0.690844	NA	0.542086	0	0	2.232127	0	0
28569	LOC10323:	0.472474	1.617593	4.067273	0.397709	0.690844	NA	0	0	0.695731	0	2.139111	0
28579	LOC10323:	0.472474	1.617593	4.067273	0.397709	0.690844	NA	0	0	0.695731	0	2.139111	0
28718	LOC11962(	0.487976	1.617593	4.067273	0.397709	0.690844	NA	0	0	0.695731	2.232127	0	0
28740	LOC11962:	0.487976	1.617593	4.067273	0.397709	0.690844	NA	0	0	0.695731	2.232127	0	0
28741	MAGEA10	0.463256	1.617593	4.067273	0.397709	0.690844	NA	0	0.547412	0	2.232127	0	0
28799	LOC11962:	0.472474	1.617593	4.067273	0.397709	0.690844	NA	0	0	0.695731	0	2.139111	0
28807	TNFRSF17	0.462369	1.617593	4.067273	0.397709	0.690844	NA	0.542086	0	0	2.232127	0	0
28839	TAS2R42	0.472474	1.617593	4.067273	0.397709	0.690844	NA	0	0	0.695731	0	2.139111	0
28847	TRNAW-C(	0.472474	1.617593	4.067273	0.397709	0.690844	NA	0	0	0.695731	0	2.139111	0
28867	LOC11962:	0.472474	1.617593	4.067273	0.397709	0.690844	NA	0	0	0.695731	0	2.139111	0
28876	LOC10324;	0.472474	1.617593	4.067273	0.397709	0.690844	NA	0	0	0.695731	0	2.139111	0
28886	OXT	0.472474	1.617593	4.067273	0.397709	0.690844	NA	0	0	0.695731	0	2.139111	0
28895	LOC11962:	0.487976	1.617593	4.067273	0.397709	0.690844	NA	0	0	0.695731	2.232127	0	0
28939	LOC10323‹	0.487976	1.617593	4.067273	0.397709	0.690844	NA	0	0	0.695731	2.232127	0	0
28962	LOC10323‹	0.487976	1.617593	4.067273	0.397709	0.690844	NA	0	0	0.695731	2.232127	0	0
28986	LOC11962:	0.463256	1.617593	4.067273	0.397709	0.690844	NA	0	0.547412	0	2.232127	0	0
29018	LOC11962;	0.463256	1.617593	4.067273	0.397709	0.690844	NA	0	0.547412	0	2.232127	0	0
29060	LOC10324:	0.472474	1.617593	4.067273	0.397709	0.690844	NA	0	0	0.695731	0	2.139111	0
29073	LOC11962;	0.487976	1.617593	4.067273	0.397709	0.690844	NA	0	0	0.695731	2.232127	0	0
29086	LOC10323!	0.472474	1.617593	4.067273	0.397709	0.690844	NA	0	0	0.695731	0	2.139111	0
29090	LOC10323!	0.487976	1.617593	4.067273	0.397709	0.690844	NA	0	0	0.695731	2.232127	0	0
29110	LOC11962;	0.463256	1.617593	4.067273	0.397709	0.690844	NA	0	0.547412	0	2.232127	0	0
29124	LOC11962;	0.487976	1.617593	4.067273	0.397709	0.690844	NA	0	0	0.695731	2.232127	0	0
29135	LOC10324:	0.472474	1.617593	4.067273	0.397709	0.690844	NA	0	0	0.695731	0	2.139111	0
29156	CABS1	0.447754	1.617593	4.067273	0.397709	0.690844	NA	0	0.547412	0	0	2.139111	0
29163	LOC11962;	0.447754	1.617593	4.067273	0.397709	0.690844	NA	0	0.547412	0	0	2.139111	0
29164	LOC10322;	0.472474	1.617593	4.067273	0.397709	0.690844	NA	0	0	0.695731	0	2.139111	0
29166	LOC11962;	0.487976	1.617593	4.067273	0.397709	0.690844	NA	0	0	0.695731	2.232127	0	0
29182	LOC11962;	0.447754	1.617593	4.067273	0.397709	0.690844	NA	0	0.547412	0	0	2.139111	0
29222	LOC11962;	0.447754	1.617593	4.067273	0.397709	0.690844	NA	0	0.547412	0	0	2.139111	0

29280	LOC11962:	0.472474	1.617593	4.067273	0.397709	0.690844	NA	0	0	0.695731	0	2.139111	0
29287	LOC11962:	0.463256	1.617593	4.067273	0.397709	0.690844	NA	0	0.547412	0	2.232127	0	0
29430	LOC11962:	0.487976	1.617593	4.067273	0.397709	0.690844	NA	0	0	0.695731	2.232127	0	0
29458	LOC11962:	0.463256	1.617593	4.067273	0.397709	0.690844	NA	0	0.547412	0	2.232127	0	0
29485	LOC11962:	0.472474	1.617593	4.067273	0.397709	0.690844	NA	0	0	0.695731	0	2.139111	0
29558	LOC11962:	0.447754	1.617593	4.067273	0.397709	0.690844	NA	0	0.547412	0	0	2.139111	0
29565	LOC11962:	0.472474	1.617593	4.067273	0.397709	0.690844	NA	0	0	0.695731	0	2.139111	0
13385	ABCG4	12.3238	1.615611	0.758441	2.130174	0.033157	0.065856	7.047123	3.284471	7.653045	26.78552	17.11289	12.05975
13058	LOC11962!	16.85004	1.615605	0.735991	2.195141	0.028153	0.057318	7.589209	2.737059	14.61036	37.94616	17.11289	21.10457
8674	LOC10322:	28.1331	1.614993	0.503025	3.210564	0.001325	0.00406	13.55216	10.94824	16.69755	55.80317	40.6431	31.15436
15652	LOC11962:	7.533797	1.614583	0.936084	1.724827	0.084559	0.143623	5.96295	2.189647	2.782925	13.39276	12.83466	8.039836
15815	LOC11962(	6.802164	1.613872	0.950199	1.698457	0.089422	0.150317	4.336691	2.189647	3.478657	8.928508	12.83466	9.044815
355	ARRB1	2272.707	1.61327	0.098825	16.32447	6.61E-60	4.95E-58	1122.119	1150.112	1089.515	3312.476	3287.813	3674.205
16063	TRNAE-UU	7.622303	1.612905	0.974759	1.654672	0.097991	0.16218	4.878777	2.737059	3.478657	22.32127	4.278221	8.039836
15318	LOC11962:	7.55285	1.612882	0.907847	1.776601	0.075634	0.131266	3.794605	4.379295	2.782925	15.62489	10.69555	8.039836
15122	LOC11962:	8.304896	1.61255	0.891022	1.809776	0.070331	0.123643	3.252518	3.284471	5.565851	17.85702	12.83466	7.034856
261	CTSL	224656.7	1.612448	0.088935	18.13063	1.83E-73	1.86E-71	117731.4	106698.2	107766	333569.1	321208.9	360966.5
12876	TMEM262	13.8568	1.612394	0.725246	2.223237	0.0262	0.054095	3.794605	8.211177	8.348776	15.62489	32.08666	15.07469
23443	LOC10324(	0.944947	1.611538	3.26897	0.49298	0.622027	0.705245	0	0	1.391463	0	4.278221	0
23444	LOC11962(	0.944947	1.611538	3.26897	0.49298	0.622027	0.705245	0	0	1.391463	0	4.278221	0
23445	LOC10323(	0.944947	1.611538	3.26897	0.49298	0.622027	0.705245	0	0	1.391463	0	4.278221	0
23446	LOC11962:	0.944947	1.611538	3.26897	0.49298	0.622027	0.705245	0	0	1.391463	0	4.278221	0
23447	LOC11962:	0.944947	1.611538	3.26897	0.49298	0.622027	0.705245	0	0	1.391463	0	4.278221	0
23448	LOC11962:	0.944947	1.611538	3.26897	0.49298	0.622027	0.705245	0	0	1.391463	0	4.278221	0
7785	ST3GAL6	36.64742	1.610774	0.464231	3.469768	0.000521	0.001779	21.68345	11.49565	20.87194	46.87467	72.72976	46.22906
1906	LAT2	265.5019	1.609089	0.196541	8.187029	2.68E-16	3.73E-15	147.9896	124.8099	121.753	330.3548	412.8484	455.2557
2775	LOC11962:	125.4702	1.609078	0.238866	6.736312	1.62E-11	1.56E-10	64.50828	59.66789	61.92009	167.4095	205.3546	193.961
1179	MANSC1	547.4511	1.608778	0.157234	10.23176	1.43E-24	3.22E-23	252.6122	318.5937	240.0273	756.691	853.5052	863.2774
11402	ROBO1	16.74252	1.608432	0.63438	2.535439	0.011231	0.026185	7.047123	10.40082	6.957314	29.01765	29.94755	17.08465
4412	SIGLEC15	73.18432	1.607995	0.317082	5.071234	3.95E-07	2.38E-06	28.73058	40.50847	39.65669	95.98146	117.6511	116.5776
22022	LOC11962!	1.156546	1.607705	2.225972	0.722248	0.470142	0.567546	0.542086	0.547412	0.695731	0	2.139111	3.014938
21689	PANO1	1.87179	1.607472	2.064177	0.778747	0.436129	0.534579	2.710432	0	0	2.232127	4.278221	2.009959
4853	LOC10323(	60.2618	1.60734	0.336286	4.779681	1.76E-06	9.62E-06	28.73058	32.84471	27.13352	98.21359	96.25998	78.3884
16944	LOC11962:	6.159557	1.607204	1.071365	1.500146	0.133577	0.209581	1.084173	4.379295	3.478657	11.16063	12.83466	4.019918
7004	LOC11962(	47.53573	1.607127	0.4307	3.73143	0.00019	0.000723	16.80468	20.25424	34.09084	49.10679	85.56443	79.39338
17551	ISM2	7.072446	1.606201	1.141186	1.407483	0.159284	0.241272	0	4.926706	5.565851	8.928508	14.97377	8.039836
16383	GPR158	7.087226	1.605966	1.009109	1.591469	0.111504	0.18094	2.168345	3.284471	4.87012	8.928508	19.252	4.019918
592	WNT2B	836.4265	1.6055	0.117301	13.68697	1.21E-42	5.45E-41	421.7432	425.8864	394.4797	1227.67	1234.267	1314.513
18068	LHX3	4.758997	1.604542	1.206133	1.330318	0.183413	0.269872	1.084173	1.094824	4.87012	8.928508	8.556443	4.019918
17275	LOC10323:	5.426209	1.604189	1.10331	1.453978	0.145952	0.22461	1.626259	4.926706	1.391463	11.16063	6.417332	7.034856
19417	PKIB	2.849518	1.604117	1.438368	1.115235	0.26475	0.362467	1.626259	1.094824	1.391463	6.696381	4.278221	2.009959
778	TSPAN15	1243.804	1.603851	0.131167	12.22752	2.22E-34	7.57E-33	646.709	557.8126	646.3344	1845.969	1653.533	2112.467
15182	SDCBP2	9.571474	1.603672	0.891118	1.799618	0.071921	0.12594	2.710432	4.379295	6.957314	26.78552	8.556443	8.039836
9949	ATP6V1G2	21.35734	1.603367	0.557624	2.875352	0.004036	0.010784	9.757555	14.23271	7.653045	31.24978	32.08666	33.16432
14408	LOC10322(	9.974139	1.602534	0.825323	1.941706	0.052173	0.096263	3.794605	3.284471	7.653045	20.08914	14.97377	10.04979
15018	LOC11962:	9.486744	1.602218	0.875414	1.83024	0.067214	0.118983	4.336691	6.568942	2.782925	20.08914	17.11289	6.029877
15921	ERAS	8.490399	1.600892	0.952467	1.680786	0.092805	0.154966	1.626259	2.737059	8.348776	8.928508	19.252	10.04979
17194	LOC11962:	5.622244	1.600337	1.089631	1.468696	0.141915	0.219435	1.084173	3.831883	3.478657	4.464254	12.83466	8.039836
482	BIRC3	1949.687	1.600318	0.108127	14.80041	1.46E-49	8.02E-48	912.8734	1074.569	913.4953	2874.98	2960.529	2961.674
3954	ZFHX2	131.5076	1.600064	0.295927	5.406962	6.41E-08	4.31E-07	42.28274	75.54283	77.22618	225.4448	211.772	156.7768
18430	LOC11961!	4.548878	1.599455	1.269581	1.259829	0.207731	0.299649	2.710432	0.547412	3.478657	13.39276	2.139111	5.024897
19139	LOC10323:	4.974207	1.598826	1.374715	1.163024	0.24482	0.340067	0.542086	5.474118	1.391463	13.39276	0	9.044815
1476	LOC11962:	318.9329	1.598765	0.171643	9.314497	1.23E-20	2.21E-19	171.2993	146.159	155.8438	522.3177	489.8563	428.1213
21129	LOC11962:	1.840619	1.597688	1.856078	0.860787	0.389355	0.489896	1.084173	1.094824	0.695731	0	2.139111	6.029877
12458	LOC10321(	18.40986	1.597091	0.691661	2.309065	0.02094	0.044685	5.420864	10.40082	11.1317	44.64254	27.80844	11.05477
6934	LOC10323!	44.92031	1.596573	0.424272	3.763087	0.000168	0.000643	13.55216	26.27577	27.13352	80.35657	59.8951	62.30873
17264	ZBED2	7.424169	1.596566	1.096058	1.456644	0.145215	0.223617	5.420864	0.547412	4.87012	17.85702	12.83466	3.014938
326	SARAF	4780.253	1.59635	0.094882	16.82454	1.61E-63	1.32E-61	2475.708	2161.182	2492.11	7194.145	7144.63	7213.743
17286	LOC10322(	6.148362	1.594756	1.098281	1.452047	0.146489	0.225292	3.252518	4.926706	0.695731	11.16063	12.83466	4.019918
20846	MEIOC	2.907906	1.59474	1.774122	0.89889	0.368711	0.470219	0	2.189647	2.087194	11.16063	0	2.009959
13860	DOK7	21.79777	1.594573	0.782619	2.037483	0.041602	0.079797	5.420864	2.737059	24.3506	49.10679	32.08666	17.08465
356	TTC39A	2841.128	1.594498	0.097933	16.28155	1.33E-59	9.97E-58	1467.97	1417.797	1357.372	4120.506	4096.397	4586.726
19101	LOC10323:	3.309683	1.593645	1.36248	1.169665	0.242136	0.336989	1.626259	0.547412	2.782925	4.464254	6.417332	4.019918
9278	CALCRL	23.82169	1.593253	0.524411	3.038176	0.00238	0.00682	11.9259	9.306001	14.61036	37.94616	29.94755	39.1942
886	CD40	1092.273	1.593093	0.137693	11.56993	5.85E-31	1.76E-29	618.5205	472.9638	541.9747	1622.756	1499.517	1797.908
19360	SOD3	2.838097	1.591655	1.414022	1.125623	0.260325	0.357476	1.084173	1.094824	2.087194	4.464254	4.278221	4.019918
4665	LOC11962!	64.85028	1.590766	0.324096	4.908322	9.19E-07	5.23E-06	34.69353	27.37059	34.78657	95.98146	104.8164	91.45313
1091	PLCD4	603.5635	1.590758	0.150855	10.54494	5.36E-26	1.31E-24	306.2788	319.6885	278.9883	866.0653	802.1665	1048.194
20865	LOC11962!	2.400345	1.589325	1.773645	0.896078	0.370211	0.471702	0.542086	2.189647	0.695731	6.696381	4.278221	0
21600	FNDC11	1.858062	1.589136	2.010387	0.790463	0.429258	0.528325	1.626259	1.094824	0	0	6.417332	2.009959
1709	ITGA7	254.4547	1.589001	0.182623	8.700999	3.29E-18	5.12E-17	120.3432	117.1461	143.3207	388.3901	395.7355	361.7926
15438	BCL11A	8.092929	1.588898	0.905797	1.754143	0.079406	0.136741	2.168345	4.926706	4.87012	17.85702	10.69555	8.039836
18329	GABRB3	4.026702	1.587769	1.24172	1.278685	0.201008	0.291549	2.710432	1.094824	2.087194	6.696381	8.556443	3.014938
2279	PLSCR4	176.1573	1.586707	0.212965	7.450568	9.29E-14	1.08E-12	95.94929	92.5126	75.13899	279.0159	245.9977	268.3295

5036	RNASE10	62.97712	1.58639	0.339707	4.669877	3.01E-06	1.59E-05	27.10432	37.224	30.61218	78.12444	96.25998	108.5378
20986	CCL1	2.154912	1.586277	1.800175	0.881179	0.378221	0.479147	0	1.094824	2.087194	4.464254	4.278221	1.004979
14479	TCEA3	9.217583	1.585869	0.822005	1.92927	0.053697	0.098594	3.252518	4.926706	5.565851	13.39276	17.11289	11.05477
4938	COL9A1	58.26982	1.585684	0.335922	4.720388	2.35E-06	1.27E-05	30.35684	25.72836	31.30791	84.82083	91.98176	85.42325
3435	LOC10323(	141.454	1.583685	0.267754	5.914704	3.32E-09	2.57E-08	74.26583	54.19377	85.57496	176.338	201.0764	257.2747
14552	LOC11962(	10.38093	1.582581	0.825909	1.916168	0.055344	0.101107	2.710432	8.758589	4.174388	17.85702	10.69555	18.08963
711	ICOSLG	838.7154	1.582186	0.124292	12.72957	4.05E-37	1.51E-35	421.7432	394.6839	445.9638	1249.991	1172.233	1347.677
441	PLA2G4A	1911.015	1.58107	0.104039	15.19691	3.71E-52	2.23E-50	1013.702	914.1777	946.8904	2727.659	2806.513	3057.148
13013	KIAA1549L	17.58365	1.579631	0.717478	2.201643	0.027691	0.056574	4.878777	14.78012	6.261582	29.01765	38.50399	12.05975
18040	LOC10324!	5.418087	1.579624	1.18176	1.336671	0.18133	0.267228	1.626259	1.642235	4.87012	2.232127	17.11289	5.024897
6843	CEACAM1	43.73423	1.579366	0.415558	3.800587	0.000144	0.000561	14.09425	27.37059	24.3506	66.96381	66.31243	63.31371
15072	LOC10321!	10.27361	1.579332	0.867157	1.821275	0.068565	0.12094	3.252518	4.926706	6.957314	20.08914	21.39111	5.024897
11644	IGSF10	25.41589	1.579199	0.636073	2.482734	0.013038	0.029767	4.336691	14.23271	19.48048	35.71403	55.61688	23.11453
1747	SLC1A3	263.9031	1.578526	0.18364	8.595746	8.27E-18	1.26E-16	116.5486	142.8745	139.1463	377.2295	376.4835	431.1362
12158	LOC10324.	15.65349	1.578199	0.664829	2.373841	0.017604	0.038494	8.673382	4.926706	9.740239	24.5534	29.94755	16.07967
11393	TDRD6	26.60893	1.577849	0.621553	2.538561	0.011131	0.025972	12.46799	13.6853	13.2189	33.4819	72.72976	14.06971
11484	LOC11962!	17.99275	1.577447	0.625848	2.520496	0.011719	0.027129	5.420864	8.758589	13.2189	29.01765	21.39111	30.14938
13282	LOC10322.	16.31275	1.576084	0.732592	2.151379	0.031446	0.06294	10.84173	4.379295	9.044508	15.62489	44.92132	13.06473
3915	LOC10321.	88.69669	1.576048	0.290033	5.434023	5.51E-08	3.74E-07	46.07734	39.41365	48.7012	147.3204	106.9555	143.7121
5794	LOC11961{	56.28966	1.575549	0.368365	4.277136	1.89E-05	8.69E-05	35.7777	20.25424	28.52499	84.82083	91.98176	76.37844
9140	LOC11962.	25.2871	1.575529	0.513398	3.068826	0.002149	0.006251	13.55216	9.853413	14.61036	33.4819	47.06043	33.16432
9284	LOC10322.	24.40794	1.575179	0.518833	3.036005	0.002397	0.006865	14.09425	12.59047	9.740239	44.64254	34.22577	31.15436
17236	LOC10324(	5.487069	1.574018	1.076714	1.461872	0.143776	0.221762	2.168345	3.831883	2.087194	13.39276	6.417332	5.024897
368	LAD1	2090.354	1.573473	0.097434	16.14905	1.15E-58	8.33E-57	1052.19	1069.095	1034.553	3185.245	2934.86	3266.183
12188	LOC11962.	19.5882	1.573473	0.664155	2.369134	0.01783	0.038891	8.131296	4.926706	16.69755	17.85702	42.78221	27.13445
7099	LOC10324!	79.52704	1.573222	0.425652	3.696029	0.000219	0.00082	18.43094	47.62483	53.57132	107.1421	168.9897	81.40334
11959	LOC11962.	17.43312	1.572493	0.651708	2.41288	0.015827	0.035184	5.96295	8.211177	11.82743	35.71403	27.80844	15.07469
17690	TFDP3	6.278675	1.570569	1.132532	1.386776	0.16551	0.248733	4.878777	2.189647	2.087194	20.08914	6.417332	2.009959
19508	LOC11962!	3.051637	1.570536	1.427775	1.099989	0.271337	0.369781	2.168345	1.642235	0.695731	2.232127	8.556443	3.014938
17057	LOC11962.	9.79941	1.570206	1.056455	1.486297	0.1372	0.213842	1.084173	10.94824	2.782925	2.232127	25.66933	16.07967
16480	LOC10323.	6.401543	1.569569	0.995877	1.576067	0.11501	0.185488	3.794605	1.642235	4.174388	8.928508	12.83466	7.034856
932	SERPINA1	32535.89	1.569387	0.138663	11.31802	1.07E-29	3.05E-28	14146.29	20654.94	14398.86	48330.01	46810.16	50875.08
19096	LOC11961{	3.610841	1.568803	1.340113	1.170649	0.24174	0.336545	2.168345	1.094824	2.087194	11.16063	2.139111	3.014938
531	SYK	1319.815	1.568567	0.110045	14.25387	4.24E-46	2.12E-44	684.6551	652.5149	662.3363	1901.772	1876	2141.611
11937	PLEKHG6	21.0385	1.568553	0.648991	2.416912	0.015653	0.034861	6.505036	7.663765	17.39328	37.94616	40.6431	16.07967
1125	HSD3B7	454.226	1.568021	0.15049	10.41945	2.02E-25	4.78E-24	239.0601	210.2061	240.0273	669.6381	626.7594	739.6649
11295	TCEAL2	20.62328	1.567877	0.612491	2.559837	0.010472	0.024648	5.96295	13.13788	11.82743	26.78552	44.92132	21.10457
13206	LOC11962.	19.44081	1.567641	0.723438	2.166933	0.03024	0.060876	6.505036	9.853413	12.52316	37.94616	42.78221	7.034856
555	MGLL	8473.208	1.567479	0.111097	14.10912	3.34E-45	1.60E-43	4498.775	3974.21	4357.366	12254.38	11367.23	14387.29
1027	COL18A1	22282.62	1.567144	0.144067	10.87792	1.47E-27	3.80E-26	12492.38	8780.485	12464.72	33595.74	28972.11	37390.26
21573	MOGAT2	1.666936	1.566515	1.975826	0.79284	0.427871	0.527253	0	1.094824	1.391463	2.232127	4.278221	1.004979
20183	SLAMF9	3.370012	1.565736	1.565829	0.999941	0.317339	0.417998	0.542086	1.642235	2.782925	6.696381	8.556443	0
7857	LOC10322.	34.79811	1.565584	0.453573	3.451665	0.000557	0.001885	21.14137	12.59047	18.78475	46.87467	64.17332	45.22408
15508	LOC11962.	7.84055	1.564768	0.898087	1.742334	0.08145	0.139628	4.878777	3.284471	3.478657	13.39276	14.97377	7.034856
781	SYNM	5085.145	1.56427	0.128013	12.21963	2.44E-34	8.31E-33	2283.81	3061.674	2366.182	7314.68	7187.412	8297.11
4932	ATP2A1	77.04617	1.563858	0.330975	4.724998	2.30E-06	1.24E-05	30.89892	38.31883	47.30973	102.6778	147.5986	95.47305
10504	ENTPD2	20.5074	1.563505	0.569839	2.743765	0.006074	0.015373	10.29964	8.758589	12.52316	22.32127	29.94755	39.1942
4678	TUBB4A	101.5177	1.563376	0.319192	4.897911	9.69E-07	5.50E-06	32.52518	68.97389	52.87558	154.0168	136.9031	163.8117
3471	LOC10321!	240.8711	1.563287	0.265533	5.887356	3.92E-09	3.01E-08	166.4205	80.46954	118.2743	299.105	434.2395	346.7179
10854	LOC11962.	20.25535	1.562956	0.587604	2.65988	0.007817	0.019146	6.505036	11.49565	12.52316	37.94616	29.94755	23.11453
22169	CLEC5A	1.404286	1.562631	2.24403	0.69635	0.486209	0.583061	1.626259	0.547412	0	2.232127	0	4.019918
17260	MYLK4	5.9474	1.562165	1.0724	1.456699	0.145199	0.223614	3.794605	2.189647	2.782925	8.928508	14.97377	3.014938
21234	LOC11962.	1.863032	1.561545	1.846572	0.845646	0.39775	0.497984	0.542086	1.642235	0.695731	0	4.278221	4.019918
14680	LOC11962.	14.22633	1.560922	0.824739	1.892625	0.058408	0.105775	10.29964	3.831883	6.957314	35.71403	23.53022	5.024897
674	LOC11962(	1028.624	1.560664	0.120283	12.97494	1.70E-38	6.70E-37	507.3928	548.5066	509.2754	1475.436	1441.761	1689.37
21036	LOC11962.	2.826842	1.560512	1.78284	0.875296	0.381413	0.482013	2.168345	0	2.087194	0	10.69555	2.009959
15642	TXK	8.865245	1.560497	0.903971	1.72627	0.084299	0.143264	1.626259	5.474118	6.261582	20.08914	10.69555	9.044815
26619	CYP4A22	0.68496	1.560453	3.618843	0.431202	0.666321	NA	0	1.094824	0	0	0	3.014938
21269	LOC10323.	1.92929	1.559731	1.857289	0.839789	0.401027	0.501254	1.626259	0.547412	0.695731	6.696381	0	2.009959
15280	LOC10321!	7.845519	1.559647	0.874601	1.783267	0.074543	0.129687	3.794605	3.831883	4.174388	13.39276	12.83466	9.044815
7410	HRH1	39.547	1.559506	0.433543	3.597122	0.000322	0.001154	21.14137	25.18094	13.2189	58.0353	68.45154	51.25395
2593	INSYN1	211.8765	1.559059	0.222983	6.991817	2.71E-12	2.78E-11	102.9964	84.84883	134.9719	294.6408	342.2577	311.5436
955	SLC7A8	1082.126	1.559023	0.139394	11.18432	4.87E-29	1.35E-27	488.9619	644.8511	512.0583	1654.006	1482.404	1710.475
2779	TCAF2	160.0514	1.558008	0.231391	6.733232	1.66E-11	1.59E-10	97.57555	74.99542	70.26887	223.2127	263.1106	231.1453
21710	LOC11962!	1.650545	1.557341	2.005776	0.776428	0.437496	0.535736	0.542086	0.547412	1.391463	0	6.417332	1.004979
5422	SPAG8	54.15688	1.557205	0.349926	4.450103	8.58E-06	4.21E-05	23.8518	28.46541	29.91645	87.05295	81.28621	74.36848
15891	LOC11962.	10.81942	1.557123	0.923628	1.685878	0.091819	0.15361	3.252518	4.926706	8.348776	2.232127	32.08666	14.06971
12466	HUNK	15.04577	1.556668	0.674838	2.306727	0.02107	0.044934	5.96295	8.211177	8.348776	29.01765	25.66933	13.06473
21199	LOC10324.	1.930178	1.556434	1.830835	0.850123	0.395257	0.495679	1.084173	1.094824	0.695731	6.696381	0	2.009959
567	PNRC1	5205.563	1.556033	0.111382	13.97025	2.37E-44	1.11E-42	2375.422	3040.325	2509.503	7964.229	7687.964	7655.934
6492	SPATA31E:	49.44599	1.553827	0.393935	3.944371	8.00E-05	0.000328	18.43094	23.53871	33.39511	71.42806	85.56443	64.31869
21273	VMO1	1.880842	1.553741	1.850904	0.83945	0.401217	0.501403	1.084173	0.547412	1.391463	2.232127	0	6.029877
20204	ARC	2.386261	1.553718	1.559655	0.996193	0.319156	0.419955	1.626259	0.547412	1.391463	4.464254	4.278221	2.009959
10159	CUNH6orf:	22.81519	1.553067	0.549988	2.823822	0.004745	0.012418	10.84173	10.40082	13.2189	31.24978	47.06043	24.11951

372	KIF13B	4118.689	1.551022	0.096233	16.1173	1.93E-58	1.38E-56	2057.76	2208.807	2023.883	6008.886	5814.103	6598.695
2695	GCNT3	151.6283	1.551015	0.226676	6.842417	7.79E-12	7.68E-11	75.89209	68.97389	87.66215	227.677	205.3546	244.21
672	RGS14	1886.062	1.550764	0.119414	12.98646	1.46E-38	5.78E-37	1019.664	855.0573	1007.419	2890.604	2532.707	3010.918
953	ARHGEF3	650.5325	1.550741	0.138606	11.18809	4.66E-29	1.30E-27	317.1205	367.3133	310.2962	915.1721	939.0696	1054.223
20217	LOC11962⁄	2.388036	1.55049	1.559719	0.994083	0.320183	0.421027	0.542086	1.642235	1.391463	4.464254	4.278221	2.009959
3962	CLEC2B	81.99237	1.550411	0.286974	5.402624	6.57E-08	4.41E-07	44.45108	38.86624	41.74388	133.9276	113.3729	119.5926
22201	CUNH12or	1.194462	1.550181	2.241399	0.691613	0.48918	0.585699	0	1.094824	0.695731	2.232127	2.139111	1.004979
22203	LOC11961⁄	1.194462	1.550181	2.241399	0.691613	0.48918	0.585699	0	1.094824	0.695731	2.232127	2.139111	1.004979
15180	IQCF1	10.98214	1.549789	0.860877	1.800244	0.071822	0.125783	3.794605	4.379295	8.348776	13.39276	29.94755	6.029877
17131	LOC11962⁄	7.445859	1.549733	1.049788	1.476234	0.139881	0.217077	6.505036	0.547412	4.174388	17.85702	8.556443	7.034856
13219	LOC11962(	13.12251	1.548866	0.715537	2.164621	0.030417	0.061172	6.505036	3.831883	9.740239	22.32127	19.252	17.08465
3339	ELL3	104.3438	1.548409	0.257931	6.003182	1.93E-09	1.54E-08	52.04029	57.47824	49.39693	167.4095	154.016	145.722
586	ZBTB20	2708.038	1.547288	0.112541	13.74866	5.19E-43	2.35E-41	1291.792	1578.188	1271.797	3966.49	4038.641	4101.321
16125	LHX4	8.450437	1.547218	0.942757	1.641163	0.100763	0.166127	2.168345	4.926706	5.565851	22.32127	10.69555	5.024897
12549	LOC10321⁄	15.0386	1.547066	0.674646	2.293151	0.021839	0.046266	6.505036	7.116354	9.044508	24.5534	29.94755	13.06473
2345	PAK6	184.7042	1.546688	0.210523	7.346876	2.03E-13	2.30E-12	85.10756	94.70224	104.3597	256.6946	258.8324	308.5287
14388	LOC10323⁄	10.70419	1.546384	0.79446	1.946458	0.0516	0.095342	3.794605	3.831883	9.044508	15.62489	12.83466	19.09461
642	LOC10322(	1020.65	1.54594	0.11713	13.19844	8.96E-40	3.71E-38	512.2716	536.4636	516.2327	1466.507	1433.204	1659.221
19292	LOC10323⁄	3.10825	1.545173	1.35931	1.136733	0.25565	0.352294	1.626259	1.642235	1.391463	6.696381	4.278221	3.014938
22532	LOC11962⁄	1.177184	1.545059	2.430368	0.63573	0.524952	0.619338	1.084173	0	0.695731	0	4.278221	1.004979
22534	LOC11962⁄	1.177184	1.545059	2.430368	0.63573	0.524952	0.619338	1.084173	0	0.695731	0	4.278221	1.004979
1016	ARHGEF10	1979.42	1.544866	0.141465	10.92046	9.20E-28	2.41E-26	1167.112	804.6954	1060.99	2997.747	2746.618	3099.357
14021	LOC10321⁄	10.98523	1.5446	0.768755	2.009222	0.044514	0.084402	3.252518	6.568942	6.957314	20.08914	14.97377	14.06971
10106	LOC10323(	27.12864	1.54256	0.543702	2.837141	0.004552	0.011974	11.38381	13.13788	16.69755	31.24978	64.17332	26.12947
16517	LOC11962⁄	9.741012	1.542498	0.981181	1.572082	0.115932	0.186598	3.794605	3.831883	6.957314	29.01765	12.83466	2.009959
15836	LOC11962⁄	10.33086	1.54236	0.91114	1.69278	0.090497	0.151924	4.336691	1.094824	10.43597	20.08914	14.97377	11.05477
9902	USH2A	28.97728	1.541897	0.534519	2.884646	0.003919	0.010521	15.7205	15.32753	12.52316	53.57105	55.61688	21.10457
6287	LOC11961⁄	46.24973	1.541302	0.382051	4.034287	5.48E-05	0.000232	23.30971	19.70683	27.82925	75.89232	68.45154	62.30873
627	LOC10323(	6498.752	1.541167	0.115699	13.32053	1.76E-40	7.46E-39	3563.676	3027.735	3384.733	9345.916	8671.955	10998.5
6404	LOC11962⁄	47.05266	1.540094	0.38716	3.977926	6.95E-05	0.000289	26.02015	26.82318	18.78475	66.96381	83.42532	60.29877
20873	LOC10323⁄	2.357031	1.539975	1.720748	0.894945	0.370817	0.47227	0.542086	1.642235	1.391463	0	8.556443	2.009959
12691	TMEM88B	14.60546	1.53938	0.680502	2.262123	0.02369	0.049625	7.047123	6.02153	9.044508	26.78552	25.66933	13.06473
18754	LOC11961⁄	3.832545	1.539093	1.257411	1.224017	0.220946	0.313205	2.168345	1.642235	2.087194	8.928508	2.139111	6.029877
754	TMPPE	871.3352	1.538614	0.123518	12.45661	1.29E-35	4.54E-34	470.531	405.6322	464.0528	1310.259	1227.85	1349.687
15929	LOC10323⁄	10.34171	1.537641	0.915448	1.679659	0.093024	0.155253	5.96295	3.284471	6.261582	17.85702	25.66933	3.014938
14925	C2CD6	9.710124	1.537534	0.831224	1.849722	0.064354	0.114629	4.336691	7.663765	2.782925	11.16063	19.252	13.06473
5165	FAM222A	64.78352	1.536638	0.334341	4.59602	4.31E-06	2.22E-05	28.73058	35.03436	36.17803	75.89232	113.3729	99.49297
10775	IGSF9	28.76319	1.5364	0.574651	2.673621	0.007504	0.018511	10.84173	12.04306	20.87194	42.41041	66.31243	20.09959
12267	LOC11962⁄	20.10307	1.53621	0.653304	2.351447	0.018701	0.040528	11.9259	6.02153	12.52316	29.01765	47.06043	14.06971
1253	CYP7B1	629.4731	1.536191	0.153703	9.994543	1.61E-23	3.42E-22	274.2957	379.3564	314.4706	892.8508	988.2691	927.5961
22059	DLK2	2.379444	1.535855	2.147764	0.715095	0.47455	0.571917	0	2.189647	1.391463	0	10.69555	0
16064	LOC11962⁄	8.007354	1.535706	0.928219	1.654466	0.098033	0.162239	2.710432	4.379295	5.565851	4.464254	12.83466	18.08963
9390	WAS	31.30714	1.535351	0.510148	3.00962	0.002616	0.007406	8.673382	15.87494	23.65487	49.10679	51.33866	39.1942
17969	LOC10321⁄	4.978192	1.534932	1.138218	1.34854	0.177485	0.262588	1.626259	3.831883	2.087194	4.464254	12.83466	5.024897
15569	LOC10321(	12.95516	1.53387	0.884908	1.733367	0.08303	0.141779	4.336691	1.094824	14.61036	20.08914	23.53022	14.06971
14420	FCGR2A	10.43531	1.533278	0.79045	1.939753	0.05241	0.096619	4.336691	8.211177	3.478657	13.39276	17.11289	16.07967
9249	DACT3	25.73583	1.533154	0.503861	3.042808	0.002344	0.006737	10.84173	15.32753	13.2189	37.94616	44.92132	32.15934
20107	CUNH12or	3.163975	1.533119	1.515848	1.011394	0.311828	0.412292	1.626259	1.094824	2.087194	11.16063	0	3.014938
21393	LOC11962⁄	1.900948	1.532602	1.874879	0.817441	0.413677	0.514074	0	2.189647	0.695731	2.232127	4.278221	2.009959
22109	LOC11962⁄	1.722661	1.532132	2.167497	0.706867	0.479649	0.576755	0	0.547412	2.087194	6.696381	0	1.004979
12839	PODN	16.92875	1.531133	0.686292	2.231024	0.02568	0.053173	5.420864	9.306001	11.1317	17.85702	42.78221	15.07469
14419	LOC11962⁄	10.02406	1.531109	0.789286	1.939865	0.052396	0.096605	5.420864	4.926706	4.87012	15.62489	19.252	10.04979
11430	LOC10323⁄	21.2316	1.530539	0.604976	2.529917	0.011409	0.026536	13.55216	7.663765	11.82743	40.17829	14.97377	39.1942
13449	LOC10321⁄	14.04086	1.530426	0.72268	2.117708	0.0342	0.067604	6.505036	6.568942	8.348776	13.39276	36.36488	13.06473
1042	DUSP10	696.8171	1.529305	0.141447	10.81185	3.02E-27	7.72E-26	342.0565	414.3907	317.9492	1058.028	1037.469	1011.009
19564	WEE2	4.327371	1.528819	1.40337	1.089391	0.275981	0.375025	0.542086	0.547412	5.565851	4.464254	12.83466	2.009959
10851	LOC11962⁄	22.27311	1.528516	0.574555	2.660348	0.007806	0.019125	11.38381	9.306001	13.2189	35.71403	44.92132	19.09461
399	NFKBIA	2417.062	1.527655	0.097286	15.70274	1.45E-55	9.65E-54	1185.543	1329.116	1221.009	3578.1	3495.307	3693.3
2268	TET1	240.8554	1.527638	0.204399	7.473794	7.79E-14	9.13E-13	118.1748	149.4434	104.3597	321.4263	382.9008	368.8275
12807	LOC10322⁄	13.76662	1.527478	0.68264	2.237606	0.025247	0.052408	5.96295	7.116354	8.348776	13.39276	25.66933	22.10955
15006	LOC10324(	11.03542	1.527337	0.83357	1.832284	0.066909	0.118538	5.420864	7.663765	3.478657	20.08914	23.53022	6.029877
12218	ZDHHC19	17.56029	1.526273	0.645923	2.362932	0.018131	0.039451	7.589209	9.306001	9.740239	35.71403	29.94755	13.06473
28319	LOC11961⁄	0.70471	1.525676	3.224742	0.473116	0.636131	NA	1.084173	0	0	0	2.139111	1.004979
727	MARVELD⁄	970.3123	1.525627	0.120918	12.61703	1.70E-36	6.22E-35	485.1673	551.2437	464.7486	1392.847	1437.482	1490.385
27046	LOC10323⁄	0.720213	1.525254	3.202247	0.476307	0.633855	NA	1.084173	0	0	2.232127	0	1.004979
27841	LOC10323⁄	0.720213	1.525254	3.202247	0.476307	0.633855	NA	1.084173	0	0	2.232127	0	1.004979
18259	TRNAE-CU⁄	4.571976	1.524953	1.180623	1.291651	0.196478	0.286071	2.710432	2.737059	1.391463	11.16063	6.417332	3.014938
12346	NRIP2	17.16063	1.524838	0.653081	2.334837	0.019552	0.042102	8.673382	6.568942	11.1317	17.85702	40.6431	18.08963
571	FBXO30	1792.949	1.524208	0.10982	13.87919	8.47E-44	3.94E-42	922.0889	973.8456	882.1874	2484.357	2579.767	2915.445
560	NT5C2	6757.494	1.523103	0.108342	14.05835	6.85E-45	3.25E-43	3306.185	3995.011	3163.491	9792.341	10227.09	10060.85
4780	TNFSF15	83.2378	1.522093	0.31552	4.824072	1.41E-06	7.82E-06	34.69353	49.26706	45.22254	93.74933	151.8769	124.6175
1656	LOC11962⁄	265.8197	1.521319	0.172325	8.828194	1.06E-18	1.71E-17	136.0637	138.4952	136.3633	408.4792	410.7092	364.8075
7082	CAMK2A	39.73562	1.520497	0.410714	3.702085	0.000214	0.000803	17.88885	19.15941	24.3506	64.73168	62.03421	50.24897
20222	LOC11962⁄	3.064049	1.520477	1.531482	0.992814	0.320801	0.421743	2.168345	0.547412	2.087194	0	8.556443	5.024897

554	PPP3CC	1083.751	1.520033	0.10773	14.10963	3.31E-45	1.59E-43	571.359	553.9808	556.5851	1533.471	1644.976	1642.136
9272	NPBWR1	26.26683	1.519636	0.499991	3.039325	0.002371	0.006798	15.7205	11.49565	13.2189	37.94616	47.06043	32.15934
400	CYP3A5	5481.837	1.519447	0.096893	15.68177	2.02E-55	1.34E-53	2620.446	3111.489	2773.185	8261.102	8087.977	8036.821
4163	P2RX6	82.17246	1.518382	0.289261	5.249184	1.53E-07	9.76E-07	39.5723	47.07742	40.35242	124.9991	130.4858	110.5477
17902	CCL21	5.263847	1.51834	1.115511	1.361117	0.173477	0.257618	2.168345	4.379295	1.391463	8.928508	10.69555	4.019918
606	IRF1	1473.156	1.516952	0.111921	13.55375	7.53E-42	3.30E-40	787.1094	762.5447	742.3454	2049.093	2089.911	2407.931
21960	BCL2A1	1.425811	1.51671	2.065606	0.734269	0.462785	0.560246	1.626259	0.547412	0	2.232127	2.139111	2.009959
18548	BPIFB4	5.861438	1.516495	1.219876	1.243155	0.213811	0.306446	2.168345	0.547412	6.261582	15.62489	8.556443	2.009959
3849	SFRP4	105.2477	1.515637	0.275691	5.497582	3.85E-08	2.66E-07	50.41403	62.40495	50.78839	124.9991	186.1026	156.7768
27342	LOC11962!	0.706486	1.514529	3.222226	0.470026	0.638337	NA	0	1.094824	0	0	2.139111	1.004979
9506	SDR9C7	37.67564	1.514224	0.507604	2.983084	0.002854	0.007981	23.30971	13.6853	20.87194	51.33892	87.70354	29.1444
29487	LOC10322	0.721988	1.514149	3.199813	0.473199	0.636071	NA	0	1.094824	0	2.232127	0	1.004979
1602	RGS20	294.4182	1.514123	0.168668	8.976919	2.78E-19	4.62E-18	143.6529	155.465	157.931	446.4254	462.0479	400.9868
12496	TAS2R3	15.14659	1.514084	0.657605	2.30242	0.021311	0.045336	5.96295	7.116354	10.43597	29.01765	19.252	19.09461
28407	LOC10323(	0.705598	1.514047	2.925685	0.517502	0.604806	NA	0.542086	0.547412	0	0	2.139111	1.004979
29213	LOC11962	0.705598	1.514047	2.925685	0.517502	0.604806	NA	0.542086	0.547412	0	0	2.139111	1.004979
16357	LOC10322	8.010126	1.513158	0.948524	1.595277	0.11065	0.17984	3.252518	2.737059	6.261582	20.08914	10.69555	5.024897
4700	LOC11962	73.02017	1.513061	0.309793	4.884112	1.04E-06	5.88E-06	35.23561	33.39212	45.22254	116.0706	102.6773	105.5228
658	PPM1L	1375.797	1.512331	0.115692	13.07199	4.76E-39	1.92E-37	693.3285	790.4627	659.5533	1959.807	2015.042	2136.586
14467	LOC11962(	12.58154	1.510976	0.782629	1.93064	0.053528	0.098357	7.589209	4.379295	7.653045	6.696381	32.08666	17.08465
3935	LOC11961!	106.617	1.510916	0.278952	5.416402	6.08E-08	4.11E-07	55.8349	64.59459	46.614	138.3919	143.3204	190.9461
14768	MAPK10	11.96519	1.510897	0.804361	1.87838	0.060329	0.108596	8.131296	6.568942	3.478657	15.62489	29.94755	8.039836
7923	LOC11962	33.01498	1.51089	0.440237	3.431995	0.000599	0.00201	17.88885	15.87494	17.39328	49.10679	55.61688	42.20914
18103	TMCO2	4.543278	1.510669	1.144569	1.319858	0.186882	0.274414	3.252518	1.642235	2.087194	6.696381	8.556443	5.024897
655	SNCA	1623.978	1.509875	0.115374	13.08679	3.92E-39	1.59E-37	755.6684	940.4535	836.9648	2361.59	2382.969	2466.22
437	UNC5D	5378.048	1.509862	0.099003	15.25073	1.63E-52	9.90E-51	2722.358	3053.463	2611.776	7790.123	7670.851	8419.718
4490	ADORA2A	92.56384	1.509239	0.300872	5.016208	5.27E-07	3.12E-06	51.49821	37.224	54.96278	142.8561	158.2942	110.5477
20364	LOC11962	2.412756	1.508866	1.560376	0.966989	0.333549	0.435445	0.542086	1.094824	2.087194	4.464254	4.278221	2.009959
21923	LOC10324	2.876637	1.508736	2.036694	0.740777	0.458829	0.55635	0	1.642235	2.782925	0	12.83466	0
11076	CMYA5	27.66061	1.508657	0.577823	2.610934	0.00903	0.021673	13.55216	16.96977	11.82743	71.42806	32.08666	20.09959
1770	THEM6	1294.529	1.508405	0.176715	8.535801	1.39E-17	2.09E-16	729.6483	507.9982	785.4807	1928.558	1574.385	2241.104
572	ENO2	1296.469	1.508002	0.108693	13.87395	9.11E-44	4.23E-42	685.1972	662.9157	678.3381	1861.594	1822.522	2068.248
16762	LOC1032!	8.976647	1.507836	0.984968	1.530848	0.125807	0.199511	1.626259	9.306001	2.782925	24.5534	8.556443	7.034856
18048	LOC11962(	6.885835	1.507505	1.129448	1.334728	0.181966	0.268038	0.542086	2.189647	8.348776	4.464254	10.69555	15.07469
298	CAMK2D	5261.994	1.506523	0.086024	17.51289	1.14E-68	1.02E-66	2848.122	2769.904	2600.644	7651.731	7852.675	7848.89
14661	LOC11962	10.57336	1.505196	0.793976	1.89577	0.057991	0.105155	3.794605	4.926706	7.653045	15.62489	21.39111	10.04979
8484	LOC11962	36.91355	1.504388	0.461567	3.259303	0.001117	0.0035	17.88885	16.42235	22.95914	46.87467	79.14709	38.18922
564	FAM102A	1730.497	1.504371	0.107367	14.01147	1.33E-44	6.25E-43	875.4695	953.044	879.4045	2600.428	2355.161	2719.474
588	ADORA1	1367.315	1.504053	0.10946	13.74061	5.80E-43	2.62E-41	705.2544	764.7343	669.2936	2006.682	1944.452	2113.472
9214	ECT2L	35.13866	1.503819	0.492822	3.051448	0.002277	0.006571	16.26259	16.42235	21.56767	53.57105	74.86887	28.13943
18738	LDHAL6A	5.288506	1.503772	1.226406	1.226162	0.220138	0.312326	1.084173	2.189647	4.87012	4.464254	17.11289	2.009959
8427	LRRC23	41.64428	1.50346	0.45909	3.274871	0.001057	0.003335	21.68345	16.96977	25.74206	64.73168	85.56443	35.17428
4994	LOC11962	83.83899	1.502517	0.320283	4.691216	2.72E-06	1.45E-05	54.20864	34.48694	41.74388	116.0706	154.016	102.5079
872	EMX1	803.6536	1.502395	0.128624	11.68053	1.60E-31	4.89E-30	410.3594	466.3949	381.2608	1178.563	1157.259	1228.085
13036	ZNF404	15.53496	1.501922	0.68335	2.197883	0.027957	0.057015	7.047123	9.306001	7.653045	15.62489	38.50399	15.07469
19444	LOC11962(	6.535805	1.500435	1.351115	1.110516	0.266777	0.364753	0	0.547412	9.740239	8.928508	14.97377	5.024897
1208	LOC10321	1019.641	1.500175	0.148135	10.12711	4.19E-24	9.22E-23	472.6993	649.2304	475.8803	1419.633	1548.716	1551.688
13838	VSIG2	12.20129	1.499718	0.734404	2.042089	0.041143	0.079042	5.96295	6.02153	6.957314	13.39276	27.80844	13.06473
19153	LOC11962	4.302449	1.499222	1.292641	1.159813	0.246125	0.341629	1.626259	2.189647	2.782925	2.232127	14.97377	2.009959
7343	LOC11962	45.81728	1.498922	0.41424	3.618489	0.000296	0.001073	16.26259	22.9913	32.69937	62.49956	79.14709	61.30375
18463	LOC10322(	4.07258	1.498875	1.194939	1.254352	0.209714	0.301969	2.168345	2.737059	1.391463	6.696381	6.417332	5.024897
1080	SPNS2	6253.889	1.498448	0.141365	10.59983	2.99E-26	7.35E-25	3703.534	2605.68	3503.007	9069.132	8216.324	10425.66
13430	ST6GAL2	14.52893	1.497844	0.706224	2.120921	0.033928	0.067163	5.420864	9.306001	7.653045	33.4819	19.252	12.05975
20271	LOC11962	3.087349	1.497597	1.521449	0.984323	0.324957	0.426174	1.084173	1.642235	2.087194	0	10.69555	3.014938
15392	LOC11962	110.7642	1.497047	0.850023	1.761184	0.078207	0.135079	59.08741	10.40082	104.3597	200.8914	173.268	116.5776
7782	TENT5C	40.64427	1.4968	0.431347	3.470063	0.00052	0.001778	17.34676	28.46541	17.39328	73.66019	57.75599	49.24399
11875	LOC10321	17.02449	1.496723	0.615365	2.432253	0.015005	0.033593	5.96295	10.40082	10.43597	24.5534	25.66933	25.12449
19660	LOC10324	3.106046	1.496422	1.389711	1.076786	0.281576	0.380758	1.084173	1.094824	2.782925	2.232127	6.417332	5.024897
10687	ZNF540	22.15906	1.496392	0.555218	2.695144	0.007036	0.017502	14.63633	9.306001	11.1317	22.32127	36.36488	39.1942
8718	P2RY8	30.82705	1.496375	0.467904	3.198038	0.001384	0.004219	13.01007	19.15941	16.69755	40.17829	40.6431	55.27387
790	IKBKE	1110.159	1.49563	0.12283	12.17642	4.15E-34	1.40E-32	568.1065	612.5538	566.3253	1502.221	1578.664	1833.083
8746	LOC11962!	32.06017	1.495616	0.468866	3.189856	0.001423	0.004327	14.63633	22.9913	12.52316	44.64254	51.33866	46.22906
11793	LOC11962	19.24044	1.495594	0.610307	2.450558	0.014263	0.032154	10.29964	5.474118	14.61036	31.24978	25.66933	28.13943
770	ZNF697	1073.389	1.495486	0.121316	12.32723	6.46E-35	2.23E-33	530.7026	628.9762	526.6686	1529.007	1597.916	1627.062
21078	MYMK	2.381751	1.495257	1.721076	0.868792	0.384961	0.485539	0.542086	1.094824	2.087194	0	8.556443	2.009959
16878	MDGA2	6.541949	1.4948	0.989884	1.510076	0.131024	0.20638	2.168345	3.831883	4.174388	15.62489	6.417332	7.034856
22245	LOC10323	1.675265	1.494539	2.181755	0.685017	0.493333	0.589556	0.542086	0	2.087194	0	6.417332	1.004979
9229	LOC10324	30.78309	1.494227	0.490336	3.047354	0.002309	0.00665	11.38381	15.87494	20.87194	44.64254	57.75599	34.1693
19247	LOC11961	3.350591	1.493743	1.304531	1.145042	0.252192	0.348308	2.168345	1.642235	1.391463	4.464254	6.417332	4.019918
9989	LOC10324	26.26864	1.493559	0.521562	2.86363	0.004188	0.011147	11.9259	14.78012	14.61036	53.57105	23.53022	39.1942
16692	LOC11962(	7.395716	1.493388	0.96746	1.543618	0.122681	0.195391	2.168345	6.568942	2.782925	6.696381	17.11289	9.044815
18857	NRG4	5.13003	1.493207	1.233033	1.211004	0.225894	0.318436	3.252518	1.094824	3.478657	13.39276	8.556443	1.004979
8130	ZNF296	45.51961	1.493109	0.442979	3.370609	0.00075	0.002453	24.93597	18.612	27.13352	91.51721	72.72976	38.18922
16147	LOC11962	7.230269	1.492397	0.911212	1.637815	0.10146	0.167048	3.794605	3.284471	4.174388	13.39276	10.69555	8.039836

2392	ARMH4	262.7195	1.492201	0.204907	7.282336	3.28E-13	3.65E-12	118.7169	173.5295	121.0573	368.301	395.7355	398.9768
15800	LOC10323	9.171665	1.492114	0.876914	1.701551	0.08884	0.149481	6.505036	5.474118	2.087194	20.08914	12.83466	8.039836
5032	BCL11B	72.23523	1.491978	0.319342	4.672033	2.98E-06	1.58E-05	35.23561	42.15071	35.4823	131.6955	98.39909	90.44815
19578	LOC10322	3.371951	1.491813	1.371282	1.087896	0.276641	0.375651	0.542086	2.737059	2.087194	6.696381	2.139111	6.029877
17230	LOC10323	6.639006	1.491536	1.019468	1.463053	0.143453	0.22134	2.710432	4.379295	3.478657	2.232127	14.97377	12.05975
5489	LOC11962	82.44251	1.491526	0.337872	4.414471	1.01E-05	4.90E-05	31.9831	41.05589	57.7457	89.28508	136.9031	137.6822
19645	ZNF287	3.418625	1.491501	1.383092	1.078382	0.280863	0.380084	2.168345	1.642235	1.391463	11.16063	2.139111	2.009959
13660	LOC11962	12.74357	1.491087	0.718218	2.076093	0.037885	0.073732	4.336691	8.211177	7.653045	22.32127	12.83466	21.10457
1086	STAB2	653.8596	1.490652	0.141266	10.5521	4.97E-26	1.22E-24	362.1137	339.3953	330.4724	839.2797	977.5736	1074.323
18900	LOC11961	4.12511	1.490555	1.237364	1.204621	0.22835	0.3212	2.168345	2.737059	1.391463	11.16063	4.278221	3.014938
1008	COQ10A	498.0686	1.490414	0.13603	10.95653	6.18E-28	1.63E-26	262.3698	267.6844	254.6377	758.9232	693.0719	751.7246
10878	LOC11962	22.39314	1.489915	0.561336	2.654231	0.007949	0.019427	8.131296	14.23271	12.52316	35.71403	40.6431	23.11453
8764	SVOPL	61.80905	1.489625	0.467939	3.183376	0.001456	0.004416	28.18849	19.15941	49.39693	102.6778	126.2075	45.22408
544	JAG1	3007.129	1.489288	0.105173	14.1603	1.61E-45	7.88E-44	1555.788	1678.912	1506.954	4368.273	4087.84	4845.006
1220	CSMD2	434.0118	1.488811	0.147367	10.10277	5.37E-24	1.17E-22	226.5921	212.9432	245.5932	600.4422	665.2634	653.2367
21445	LOC10323	1.94117	1.488721	1.832575	0.812365	0.416582	0.516439	0	1.642235	1.391463	4.464254	2.139111	2.009959
16589	LOC11961	7.514505	1.488672	0.954821	1.559112	0.11897	0.190657	3.252518	5.474118	2.782925	17.85702	10.69555	5.024897
14555	POU2AF1	13.00632	1.488639	0.777033	1.915798	0.055391	0.101172	3.252518	6.02153	11.1317	15.62489	29.94755	12.05975
2006	GPR68	257.8746	1.488464	0.186905	7.963739	1.67E-15	2.21E-14	139.8583	121.5254	146.7993	334.819	387.179	417.0665
14444	LOC10324	11.76926	1.487709	0.768674	1.935422	0.052939	0.097436	8.673382	6.02153	3.478657	17.85702	23.53022	11.05477
4829	LOC10324	109.8376	1.487554	0.310262	4.794517	1.63E-06	8.97E-06	54.75072	50.36189	66.79021	174.1059	207.4937	105.5228
15342	LOC11962	9.206692	1.487365	0.839366	1.772011	0.076393	0.132374	4.878777	3.831883	5.565851	20.08914	12.83466	8.039836
21025	LOC11962	2.401336	1.486774	1.695977	0.876648	0.380678	0.481347	0	1.094824	2.782925	2.232127	4.278221	4.019918
819	KAT2B	2189.154	1.486586	0.124505	11.93997	7.32E-33	2.38E-31	1069.536	1348.275	1037.335	3131.674	3140.214	3407.885
6131	LOC11962	68.43534	1.485988	0.36207	4.104151	4.06E-05	0.000176	30.89892	38.31883	37.56949	124.9991	115.512	63.31371
21932	LCN1	2.204352	1.485976	2.011067	0.738899	0.459968	0.557553	0	0	3.478657	4.464254	4.278221	1.004979
619	ITGB8	2037.661	1.48587	0.11083	13.40674	5.52E-41	2.37E-39	1012.075	1175.841	1031.074	2841.498	2928.443	3237.039
18414	VSTM2L	4.552495	1.485745	1.176922	1.262399	0.206805	0.298572	3.252518	1.094824	2.782925	4.464254	10.69555	5.024897
10124	LOC10322	23.26755	1.485637	0.524587	2.832012	0.004626	0.012147	10.84173	12.59047	13.2189	29.01765	42.78221	31.15436
6945	LOC10321	46.27866	1.485197	0.395121	3.75884	0.000171	0.000653	26.56223	29.56024	16.69755	69.19594	66.31243	69.34358
13383	LOC10324	14.26296	1.4846	0.696838	2.130483	0.033132	0.065815	4.336691	7.116354	11.1317	26.78552	17.11289	19.09461
18891	LOC11962	4.110495	1.483978	1.23132	1.205192	0.228129	0.321046	1.626259	3.284471	1.391463	8.928508	6.417332	3.014938
11634	ADGRD1	26.46551	1.482744	0.596341	2.486403	0.012904	0.029487	11.9259	8.211177	21.56767	26.78552	64.17332	26.12947
3170	LOC10321	131.4306	1.482725	0.239355	6.194674	5.84E-10	4.90E-09	74.80792	67.33165	65.39875	169.6417	222.4675	188.9361
640	PLXDC2	5643.865	1.48261	0.112268	13.206	8.10E-40	3.37E-38	2747.836	3416.945	2760.662	7988.782	8139.316	8809.65
15837	LOC11962	7.977865	1.482539	0.875892	1.692605	0.090531	0.151971	3.252518	4.379295	4.87012	15.62489	10.69555	9.044815
11810	ZNF418	21.78346	1.482465	0.605884	2.44678	0.014414	0.032446	13.01007	7.663765	13.2189	37.94616	42.78221	16.07967
14093	PPP1R16B	16.65817	1.482066	0.742437	1.996218	0.04591	0.086605	3.794605	10.40082	11.82743	20.08914	42.78221	11.05477
20751	LOC10322	3.212883	1.481982	1.62768	0.910487	0.362566	0.464437	0	2.189647	2.782925	11.16063	2.139111	1.004979
8271	WNT10B	32.38679	1.481512	0.44594	3.32222	0.000893	0.00287	17.34676	16.42235	17.39328	40.17829	57.75599	45.22408
20141	LOC11962	3.357336	1.481406	1.473955	1.005055	0.31487	0.415611	0	3.284471	2.087194	4.464254	4.278221	6.029877
9511	LOC11961	26.41849	1.4813	0.496845	2.981416	0.002869	0.00802	11.9259	16.42235	13.2189	35.71403	47.06043	34.1693
653	ENDOD1	2321.263	1.480444	0.113028	13.09799	3.38E-39	1.38E-37	1143.802	1371.814	1160.48	3205.334	3416.16	3629.986
7263	LOC11962	43.68925	1.480167	0.405812	3.647424	0.000265	0.00097	28.18849	19.70683	20.87194	55.80317	81.28621	56.27885
785	ATP6V0D1	4065.714	1.480052	0.121383	12.19319	3.38E-34	1.14E-32	2329.887	1911.562	2199.903	5774.512	5377.724	6800.696
6140	GPR146	64.60919	1.479479	0.360754	4.10107	4.11E-05	0.000178	27.6464	32.84471	41.74388	73.66019	128.3466	83.4133
11285	SNTG1	28.60145	1.479377	0.577639	2.561076	0.010435	0.02458	10.84173	13.6853	20.17621	44.64254	64.17332	18.08963
16560	CC2D2B	8.18034	1.479019	0.945859	1.563679	0.117893	0.189251	1.626259	7.663765	3.478657	11.16063	17.11289	8.039836
460	P4HA2	3031.918	1.478883	0.098815	14.96618	1.22E-50	7.06E-49	1589.939	1617.054	1600.182	4363.808	4158.431	4862.091
19255	KLRG2	5.37184	1.478451	1.292115	1.144211	0.252536	0.348672	2.168345	3.284471	2.782925	11.16063	12.83466	0
11204	LOC10322	20.78091	1.477901	0.572404	2.58192	0.009825	0.023313	13.55216	9.853413	9.044508	40.17829	29.94755	22.10955
6751	LOC11961	56.0135	1.477789	0.385245	3.835969	0.000125	0.000493	32.52518	18.612	38.26523	80.35657	74.86887	91.45313
5194	PPM1N	70.89319	1.477681	0.322492	4.582065	4.60E-06	2.36E-05	39.5723	35.58177	36.17803	113.8385	119.7902	80.39836
19080	LOC10322	4.364092	1.476709	1.259411	1.172539	0.240981	0.335775	1.084173	4.379295	1.391463	11.16063	2.139111	6.029877
14052	WDR88	12.58803	1.475774	0.736141	2.004744	0.04499	0.085118	7.047123	4.926706	7.653045	22.32127	23.53022	10.04979
11243	LOC11961	69.72176	1.475052	0.573438	2.572294	0.010103	0.023889	16.26259	13.13788	81.40057	120.5349	117.6511	69.34358
9200	ANKRD30A	38.23844	1.474886	0.48286	3.054482	0.002254	0.006515	22.22554	10.40082	27.82925	64.73168	62.03421	42.20914
894	DNM1	1170.065	1.474428	0.128218	11.49938	1.33E-30	3.95E-29	674.3554	530.9895	653.9875	1741.059	1632.141	1787.858
2280	FGFR3	477.6149	1.47441	0.197931	7.449105	9.40E-14	1.10E-12	262.3698	187.7623	310.2962	725.4413	598.951	780.869
21390	LOC11962	2.879832	1.474354	1.802557	0.817924	0.413401	0.513804	0	1.094824	3.478657	0	10.69555	2.009959
514	APPL2	1745.477	1.473342	0.102325	14.39862	5.28E-47	2.73E-45	952.4458	941.0009	881.4916	2457.572	2519.872	2720.479
412	CTSB	11637.04	1.473125	0.09481	15.53767	1.93E-54	1.24E-52	6212.852	6318.775	5962.418	16642.74	15906.43	18779.05
7820	LOC11962	39.17019	1.472951	0.425617	3.460741	0.000539	0.001831	19.51511	24.08612	18.08902	75.89232	49.19955	48.23901
15505	LOC11962	11.47852	1.472821	0.844892	1.743206	0.081298	0.139394	8.131296	2.189647	7.653045	29.01765	12.83466	9.044815
6967	TEX26	48.19472	1.472814	0.392803	3.7495	0.000177	0.000676	22.76763	33.39212	20.17621	82.5887	59.8951	70.34856
14849	LOC10323	10.52116	1.471973	0.790345	1.862444	0.062541	0.11197	5.96295	4.926706	6.261582	11.16063	10.69555	24.11951
11921	LOC10321	22.98392	1.471682	0.608138	2.419979	0.015521	0.034614	7.589209	18.612	9.740239	37.94616	44.92132	19.09461
19357	LOC10322	3.390814	1.470973	1.306287	1.126072	0.260135	0.357271	2.168345	1.094824	2.087194	6.696381	4.278221	4.019918
5650	DISP2	75.52875	1.469734	0.338143	4.346488	1.38E-05	6.51E-05	32.52518	33.39212	54.96278	91.51721	126.2075	114.5677
19841	LOC10323	3.355726	1.469446	1.403459	1.047017	0.295092	0.395012	2.710432	1.094824	1.391463	2.232127	10.69555	2.009959
21517	TRNAQ-CU	2.224163	1.469389	1.833272	0.801512	0.422835	0.522405	2.168345	0.547412	0.695731	8.928508	0	1.004979
9528	LOC10324	28.81143	1.468948	0.493377	2.977337	0.002908	0.008113	18.97302	14.78012	11.82743	33.4819	55.61688	38.18922
535	EPHX1	2301.911	1.468664	0.103206	14.23044	5.93E-46	2.95E-44	1147.597	1329.116	1190.396	3366.047	3257.866	3520.443
10288	LOC11962	30.03712	1.468165	0.525983	2.791276	0.00525	0.013567	14.09425	10.40082	23.65487	58.0353	27.80844	46.22906

19518	LOC11962	4.697779	1.468106	1.336739	1.098275	0.272085	0.370585	3.252518	0.547412	3.478657	15.62489	4.278221	1.004979
19149	LOC11961	4.927483	1.466652	1.263264	1.161002	0.245641	0.341029	1.084173	1.094824	5.565851	13.39276	6.417332	2.009959
17424	LOC10324	7.596535	1.466542	1.028839	1.425435	0.154032	0.235017	5.96295	1.642235	4.174388	20.08914	10.69555	3.014938
6585	S100A5	49.05286	1.466538	0.375335	3.907282	9.33E-05	0.000377	21.14137	26.82318	29.91645	78.12444	77.00798	61.30375
18444	LOC10324	6.274006	1.465875	1.165505	1.257717	0.208494	0.300505	3.794605	0.547412	5.565851	4.464254	19.252	4.019918
662	PPARGC1B	1098.077	1.465675	0.112203	13.06273	5.38E-39	2.16E-37	559.9752	612.5538	580.9357	1546.864	1578.664	1709.47
15810	LOC10322	8.216124	1.46554	0.862493	1.69919	0.089283	0.150133	4.336691	4.379295	4.174388	13.39276	14.97377	8.039836
21453	LOC11962	2.490007	1.465015	1.807089	0.810704	0.417536	0.517419	0.542086	0.547412	2.782925	8.928508	2.139111	0
16036	KCNK4	13.44501	1.464545	0.882897	1.658795	0.097157	0.16107	3.794605	1.642235	16.00182	26.78552	21.39111	11.05477
9969	LOC10324	51.29098	1.46403	0.510016	2.870558	0.004097	0.010927	21.68345	15.87494	43.83108	73.66019	115.512	37.18424
13566	MYCN	15.54799	1.463635	0.698316	2.095948	0.036087	0.070719	5.96295	9.306001	9.740239	8.928508	34.22577	25.12449
901	S100A3	911.8413	1.46333	0.127711	11.45818	2.14E-30	6.31E-29	490.5882	429.7183	537.1046	1368.294	1285.606	1359.737
731	SEMA3F	5074.392	1.463114	0.116177	12.59389	2.28E-36	8.30E-35	2817.223	2472.659	2818.408	7412.894	6528.566	8396.603
2035	SUOX	343.8651	1.463054	0.185215	7.899239	2.81E-15	3.67E-14	178.8885	212.9432	158.6268	491.0679	442.7959	578.8682
16322	LOC11962	8.518997	1.462113	0.913062	1.601329	0.109304	0.178033	2.710432	4.379295	6.261582	15.62489	17.11289	5.024897
2062	GDPGP1	364.5037	1.461865	0.185843	7.866109	3.66E-15	4.72E-14	223.3396	152.1805	208.0237	491.0679	547.6123	564.7985
13792	LOC11962	12.76701	1.46138	0.713656	2.047736	0.040586	0.078232	9.757555	4.379295	6.261582	17.85702	19.252	19.09461
20319	LOC11962	2.686991	1.461046	1.498426	0.975054	0.329534	0.431156	0.542086	1.642235	2.087194	6.696381	2.139111	3.014938
517	ADAMTS1	40887.62	1.460896	0.101524	14.38959	6.01E-47	3.09E-45	21346.82	23447.29	20581.13	57957.18	55509.92	66483.41
15564	LOC10323	9.131485	1.460598	0.842289	1.734083	0.082903	0.141608	4.878777	3.831883	6.261582	11.16063	8.556443	20.09959
16279	LOC11961	8.979694	1.460402	0.906079	1.611783	0.107009	0.174755	4.336691	1.642235	8.348776	13.39276	17.11289	9.044815
1636	DRP2	323.759	1.460058	0.164348	8.883966	6.45E-19	1.05E-17	179.4306	183.9304	154.4524	477.6752	455.6306	491.435
26589	TRNAN-GU	0.283452	1.459961	4.072558	0.358488	0.719979	NA	0	0	0.695731	0	0	1.004979
26646	LOC11962	0.257844	1.459961	4.072558	0.358488	0.719979	NA	0.542086	0	0	0	0	1.004979
26684	SPINK14	0.258732	1.459961	4.072558	0.358488	0.719979	NA	0	0.547412	0	0	0	1.004979
26711	LOC11961	0.283452	1.459961	4.072558	0.358488	0.719979	NA	0	0	0.695731	0	0	1.004979
26712	LOC11961	0.283452	1.459961	4.072558	0.358488	0.719979	NA	0	0	0.695731	0	0	1.004979
26973	UBL4B	0.283452	1.459961	4.072558	0.358488	0.719979	NA	0	0	0.695731	0	0	1.004979
27000	CUNH9orf	0.257844	1.459961	4.072558	0.358488	0.719979	NA	0.542086	0	0	0	0	1.004979
27186	LOC11962	0.258732	1.459961	4.072558	0.358488	0.719979	NA	0	0.547412	0	0	0	1.004979
27193	SMLR1	0.258732	1.459961	4.072558	0.358488	0.719979	NA	0	0.547412	0	0	0	1.004979
27261	LOC11962	0.258732	1.459961	4.072558	0.358488	0.719979	NA	0	0.547412	0	0	0	1.004979
27286	LOC11962	0.258732	1.459961	4.072558	0.358488	0.719979	NA	0	0.547412	0	0	0	1.004979
27292	LOC10321	0.258732	1.459961	4.072558	0.358488	0.719979	NA	0	0.547412	0	0	0	1.004979
27346	LOC11962	0.258732	1.459961	4.072558	0.358488	0.719979	NA	0	0.547412	0	0	0	1.004979
27347	STRIT1	0.258732	1.459961	4.072558	0.358488	0.719979	NA	0	0.547412	0	0	0	1.004979
27441	LOC11962	0.283452	1.459961	4.072558	0.358488	0.719979	NA	0	0	0.695731	0	0	1.004979
27590	LOC10324	0.283452	1.459961	4.072558	0.358488	0.719979	NA	0	0	0.695731	0	0	1.004979
27643	TRNAA-AG	0.283452	1.459961	4.072558	0.358488	0.719979	NA	0	0	0.695731	0	0	1.004979
27765	LOC11962	0.283452	1.459961	4.072558	0.358488	0.719979	NA	0	0	0.695731	0	0	1.004979
27776	LOC10321	0.258732	1.459961	4.072558	0.358488	0.719979	NA	0	0.547412	0	0	0	1.004979
27793	LOC11962	0.257844	1.459961	4.072558	0.358488	0.719979	NA	0.542086	0	0	0	0	1.004979
27829	LOC11962	0.258732	1.459961	4.072558	0.358488	0.719979	NA	0	0.547412	0	0	0	1.004979
27881	LOC11962	0.283452	1.459961	4.072558	0.358488	0.719979	NA	0	0	0.695731	0	0	1.004979
27900	FGFBP2	0.283452	1.459961	4.072558	0.358488	0.719979	NA	0	0	0.695731	0	0	1.004979
28032	LOC10322	0.283452	1.459961	4.072558	0.358488	0.719979	NA	0	0	0.695731	0	0	1.004979
28064	CUNH20or	0.258732	1.459961	4.072558	0.358488	0.719979	NA	0	0.547412	0	0	0	1.004979
28102	LOC11961	0.258732	1.459961	4.072558	0.358488	0.719979	NA	0	0.547412	0	0	0	1.004979
28261	LOC11961	0.258732	1.459961	4.072558	0.358488	0.719979	NA	0	0.547412	0	0	0	1.004979
28311	LOC11961	0.257844	1.459961	4.072558	0.358488	0.719979	NA	0.542086	0	0	0	0	1.004979
28344	LOC11961	0.258732	1.459961	4.072558	0.358488	0.719979	NA	0	0.547412	0	0	0	1.004979
28358	LOC11961	0.283452	1.459961	4.072558	0.358488	0.719979	NA	0	0	0.695731	0	0	1.004979
28401	LOC11961	0.283452	1.459961	4.072558	0.358488	0.719979	NA	0	0	0.695731	0	0	1.004979
28411	LOC11961	0.257844	1.459961	4.072558	0.358488	0.719979	NA	0.542086	0	0	0	0	1.004979
28504	LOC11962	0.257844	1.459961	4.072558	0.358488	0.719979	NA	0.542086	0	0	0	0	1.004979
28529	LOC11962	0.258732	1.459961	4.072558	0.358488	0.719979	NA	0	0.547412	0	0	0	1.004979
28541	LOC11962	0.258732	1.459961	4.072558	0.358488	0.719979	NA	0	0.547412	0	0	0	1.004979
28627	TEX51	0.283452	1.459961	4.072558	0.358488	0.719979	NA	0	0	0.695731	0	0	1.004979
28676	LOC11962	0.283452	1.459961	4.072558	0.358488	0.719979	NA	0	0	0.695731	0	0	1.004979
28758	LOC11962	0.258732	1.459961	4.072558	0.358488	0.719979	NA	0	0.547412	0	0	0	1.004979
28821	LOC11962	0.257844	1.459961	4.072558	0.358488	0.719979	NA	0.542086	0	0	0	0	1.004979
28859	KRTAP8-1	0.258732	1.459961	4.072558	0.358488	0.719979	NA	0	0.547412	0	0	0	1.004979
28864	LOC11962	0.283452	1.459961	4.072558	0.358488	0.719979	NA	0	0	0.695731	0	0	1.004979
28870	LOC11962	0.258732	1.459961	4.072558	0.358488	0.719979	NA	0	0.547412	0	0	0	1.004979
28914	LOC10322	0.258732	1.459961	4.072558	0.358488	0.719979	NA	0	0.547412	0	0	0	1.004979
28953	LOC11962	0.283452	1.459961	4.072558	0.358488	0.719979	NA	0	0	0.695731	0	0	1.004979
28984	PFN3	0.283452	1.459961	4.072558	0.358488	0.719979	NA	0	0	0.695731	0	0	1.004979
29070	PPP1R27	0.257844	1.459961	4.072558	0.358488	0.719979	NA	0.542086	0	0	0	0	1.004979
29151	PF4V1	0.283452	1.459961	4.072558	0.358488	0.719979	NA	0	0	0.695731	0	0	1.004979
29184	LOC11962	0.283452	1.459961	4.072558	0.358488	0.719979	NA	0	0	0.695731	0	0	1.004979
29214	LOC11962	0.258732	1.459961	4.072558	0.358488	0.719979	NA	0	0.547412	0	0	0	1.004979
29236	LOC11962	0.258732	1.459961	4.072558	0.358488	0.719979	NA	0	0.547412	0	0	0	1.004979
20828	SLC6A18	2.421974	1.459698	1.620551	0.900742	0.367726	0.469367	0.542086	0.547412	2.782925	2.232127	6.417332	2.009959
3377	TNFRSF6B	340.7651	1.459482	0.244733	5.963565	2.47E-09	1.94E-08	228.2184	114.4091	203.1536	562.496	410.7092	525.6043

10339	SLC25A48	22.42899	1.459123	0.524946	2.779568	0.005443	0.013996	13.01007	10.40082	12.52316	31.24978	34.22577	33.16432
1012	SOX6	2195.634	1.458248	0.13323	10.94535	7.00E-28	1.84E-26	1045.685	1408.491	1061.686	3160.692	3080.319	3416.93
629	ARRDC3	7508.707	1.458168	0.10967	13.29593	2.44E-40	1.03E-38	3686.187	4619.061	3714.51	11176.26	11151.18	10705.04
12736	SHBG	16.64781	1.457761	0.647642	2.250874	0.024394	0.050919	5.96295	7.116354	13.91463	20.08914	25.66933	27.13445
3206	DOCK8	147.1979	1.457709	0.236842	6.154774	7.52E-10	6.23E-09	63.96619	90.87036	81.40057	218.7484	201.0764	227.1254
10635	KLHL35	26.98863	1.456617	0.537573	2.709619	0.006736	0.016839	13.01007	10.94824	18.78475	46.87467	49.19955	23.11453
8964	LOC11962	30.87492	1.456055	0.46553	3.127733	0.001762	0.005224	15.17842	13.6853	20.87194	37.94616	51.33866	46.22906
21278	LOC11962	2.141514	1.455825	1.735128	0.83903	0.401452	0.501579	1.626259	1.094824	0.695731	0	6.417332	3.014938
7681	LMO1	55.0092	1.455174	0.416009	3.49794	0.000469	0.001623	24.93597	23.53871	38.96096	95.98146	98.39909	48.23901
470	ACSL1	2323.785	1.45488	0.097748	14.88405	4.18E-50	2.37E-48	1253.304	1251.383	1225.183	3296.852	3257.866	3658.125
16803	LOC10322	6.790313	1.454826	0.954284	1.524521	0.127379	0.201521	2.710432	3.831883	4.174388	11.16063	12.83466	6.029877
18488	CCDC70	5.076184	1.45456	1.160534	1.253354	0.210077	0.301974	2.168345	1.094824	4.87012	4.464254	12.83466	5.024897
487	CHST15	3706.613	1.454544	0.098466	14.77208	2.22E-49	1.21E-47	1933.08	2002.98	2013.447	5363.801	5014.075	5912.294
18937	FETUB	4.597914	1.45395	1.213762	1.197887	0.230961	0.324238	4.878777	1.642235	0.695731	8.928508	6.417332	5.024897
4706	LOC10323	78.72863	1.453486	0.297778	4.881101	1.05E-06	5.96E-06	49.32986	39.96106	36.87376	120.5349	109.0946	116.5776
1829	MYOM3	265.1816	1.453394	0.173681	8.368171	5.85E-17	8.51E-16	152.8684	138.4952	134.2762	399.5507	367.927	397.9719
9964	PCDHB5	27.67364	1.453166	0.506013	2.871798	0.004081	0.01089	11.38381	18.612	13.91463	51.33892	40.6431	30.14938
13973	MYL7	15.80111	1.452723	0.720296	2.016842	0.043712	0.083166	4.878777	7.663765	12.52316	22.32127	36.36488	11.05477
19736	LOC11962	3.805951	1.452555	1.366675	1.062838	0.287855	0.38775	4.336691	0.547412	1.391463	2.232127	4.278221	10.04979
19950	LOC10321	3.64435	1.451903	1.398961	1.037844	0.299343	0.398899	0.542086	0.547412	4.87012	4.464254	6.417332	5.024897
764	DPYSL2	4818.991	1.451699	0.117029	12.40461	2.47E-35	8.58E-34	2342.355	2946.718	2453.845	7004.414	6498.618	7667.993
11289	PARVG	24.95536	1.45153	0.566912	2.560418	0.010455	0.02462	7.047123	14.78012	18.08902	33.4819	49.19955	27.13445
897	SMPD1	1180.553	1.450519	0.126266	11.48785	1.52E-30	4.50E-29	638.0357	619.1228	642.8558	1816.951	1475.986	1890.366
13138	SV2C	14.49526	1.450081	0.66453	2.182115	0.029101	0.058881	8.131296	5.474118	9.740239	17.85702	25.66933	20.09959
2375	RGS11	320.0558	1.449924	0.198459	7.305906	2.75E-13	3.08E-12	165.8784	148.3486	202.4578	495.5322	376.4835	531.6341
9059	MGAM	52.89318	1.449493	0.46873	3.09238	0.001986	0.005827	30.89892	22.44388	30.61218	82.5887	117.6511	33.16432
1003	LGALS3	20864.02	1.449246	0.132068	10.97345	5.13E-28	1.36E-26	12323.25	8701.111	12532.21	30316.75	30777.52	30533.29
10568	LOC10321	26.1834	1.449198	0.531022	2.729075	0.006351	0.015977	16.80468	9.853413	16.00182	24.5534	40.6431	49.24399
3018	LOC11962	160.4559	1.447757	0.226595	6.389178	1.67E-10	1.47E-09	78.06044	88.1333	91.14081	225.4448	275.9453	204.0108
14113	LDLRAD2	13.083	1.447139	0.726721	1.991327	0.046445	0.087489	3.794605	8.758589	8.348776	17.85702	25.66933	14.06971
20181	LOC11962	3.661793	1.446757	1.446655	1.000071	0.317276	0.417957	1.084173	0.547412	4.174388	4.464254	10.69555	1.004979
14130	LOC11962	14.84941	1.446744	0.727645	1.988256	0.046783	0.088021	7.047173	6.02153	10.43597	22.32127	34.22577	9.044815
13379	LOC10322	20.14217	1.446252	0.678703	2.130905	0.033097	0.065766	7.589209	19.15941	5.565851	42.41041	14.97377	31.15436
11845	LOC11962	19.68237	1.446015	0.592945	2.438701	0.01474	0.033083	8.673382	7.663765	15.30609	31.24978	32.08666	23.11453
14839	LOC10323	12.15906	1.445955	0.775729	1.863995	0.062322	0.111655	4.878777	6.02153	8.348776	17.85702	27.80844	8.039836
1538	DENND3	393.8958	1.445683	0.158222	9.137053	6.42E-20	1.11E-18	200.572	198.7105	237.2444	575.8888	536.9168	614.0425
17129	LOC11962	6.317839	1.444114	0.977962	1.476657	0.139768	0.216926	2.710432	3.831883	3.478657	11.16063	10.69555	6.029877
11894	POU5F2	21.83911	1.443806	0.595009	2.426527	0.015244	0.034073	15.7205	9.853413	9.740239	15.62489	44.92132	35.17428
1665	MYO16	303.3101	1.442888	0.163857	8.805796	1.30E-18	2.07E-17	170.2151	162.0339	157.2353	464.2824	412.8484	453.2457
2266	MFSD4A	267.4124	1.441751	0.192883	7.474761	7.73E-14	9.07E-13	135.5216	134.1159	163.4969	424.1041	325.1448	422.0914
12360	SLC22A20F	21.58506	1.441564	0.618734	2.32986	0.019814	0.042617	11.9259	10.40082	11.82743	51.33892	29.94755	14.06971
15698	LOC11961	9.752591	1.44156	0.839534	1.717096	0.085962	0.145579	4.336691	5.474118	5.565851	17.85702	19.252	6.029877
21675	LOC11961	2.176767	1.441479	1.84681	0.780524	0.435083	0.53367	2.710432	0	0.695731	2.232127	6.417332	1.004979
19180	LOC11961	5.15024	1.441325	1.245566	1.157164	0.247205	0.342721	2.168345	1.094824	4.87012	8.928508	12.83466	1.004979
19022	SYTL5	7.847715	1.441092	1.220491	1.180748	0.237703	0.332212	3.794605	7.116354	1.391463	13.39276	21.39111	0
15716	SLC52A1	10.74609	1.439849	0.839375	1.715382	0.086275	0.145942	4.878777	3.831883	8.348776	17.85702	23.53022	6.029877
19026	MEIOSIN	4.640573	1.439847	1.220283	1.179929	0.238028	0.332597	0.542086	2.189647	4.87012	8.928508	4.278221	7.034856
11874	CATSPERG	27.68278	1.43974	0.591899	2.432411	0.014999	0.033581	8.131296	11.49565	25.04633	31.24978	62.03421	28.13943
12130	MACC1	19.83798	1.439515	0.60507	2.379088	0.017356	0.038038	9.215468	8.211177	14.61036	20.08914	42.78221	24.11951
2184	RAB30	345.1198	1.439424	0.188353	7.64218	2.14E-14	2.60E-13	168.0468	229.913	159.3225	468.7467	541.195	503.4947
21087	LOC11962	2.210435	1.439247	1.659735	0.867155	0.385857	0.486462	1.084173	1.642235	0.695731	6.696381	2.139111	1.004979
15114	GABRB1	12.4313	1.439134	0.794758	1.810783	0.070174	0.123434	3.252518	4.926706	11.82743	17.85702	25.66933	11.05477
9499	LOC10321	29.40756	1.438926	0.482216	2.983985	0.002845	0.007963	11.38381	18.06459	18.08902	51.33892	36.36488	41.20416
3573	LOC10322	117.9072	1.438793	0.248932	5.77987	7.48E-09	5.56E-08	65.05036	70.61612	54.26705	171.8738	181.8244	163.8117
9839	LOC11962	33.19194	1.438708	0.496528	2.897537	0.003761	0.010162	23.8518	20.25424	9.740239	37.94616	47.06043	60.29877
895	CPEB4	3778.066	1.438588	0.125135	11.4963	1.38E-30	4.09E-29	1877.787	2425.582	1806.119	5354.873	5418.367	5785.667
19430	FXYD7	4.725162	1.438239	1.291966	1.113218	0.265615	0.363426	1.626259	1.642235	4.174388	15.62489	4.278221	1.004979
19715	CUNH16or	3.398612	1.438036	1.347445	1.067232	0.285867	0.385482	1.084173	2.189647	2.087194	4.464254	8.556443	2.009959
16838	HDC	7.212361	1.437514	0.946952	1.518043	0.129004	0.203691	4.336691	3.831883	3.478657	4.464254	17.11289	10.04979
3726	EPS8L1	130.366	1.437466	0.255977	5.615605	1.96E-08	1.40E-07	61.25576	61.31012	88.35788	194.195	203.2155	173.8614
12624	LOC11962	15.78086	1.436557	0.631038	2.276499	0.022816	0.048049	8.131296	8.758589	8.348776	24.5534	27.80844	17.08465
21156	LOC10323	2.193157	1.434513	1.672582	0.857664	0.391078	0.491436	2.168345	0.547412	0.695731	4.464254	4.278221	1.004979
13848	ZNF426	13.54303	1.433929	0.702634	2.040792	0.041272	0.079232	5.96295	10.94824	4.87012	17.85702	23.53022	18.08963
19439	LOC10321	5.840384	1.433751	1.289776	1.111628	0.266298	0.364174	1.084173	8.211177	0	11.16063	8.556443	6.029877
7299	IRAG1	46.06109	1.43346	0.394309	3.635368	0.000278	0.001011	20.0572	24.63353	29.91645	58.0353	83.42532	60.29877
1164	CASZ1	1002.107	1.432861	0.139288	10.28707	8.06E-25	1.84E-23	497.6353	632.2606	496.0565	1493.293	1332.666	1560.733
6041	CASR	55.42537	1.432811	0.345529	4.146721	3.37E-05	0.000148	31.44101	27.918	30.61218	69.19594	91.98176	81.40334
9076	LOC11962	52.27562	1.432757	0.463688	3.089917	0.002002	0.005865	29.27266	14.23271	41.04815	64.73168	109.0946	55.27387
5064	GGN	96.01756	1.432608	0.307868	4.653322	3.27E-06	1.71E-05	40.65648	54.19377	59.8329	158.481	160.4333	102.5079
710	CD276	3943.876	1.432476	0.112527	12.73012	4.02E-37	1.51E-35	1972.652	2421.75	2004.402	5645.049	5456.871	6162.534
17056	LOC11962	7.314228	1.432426	0.963757	1.486293	0.137202	0.213842	4.336691	4.379295	2.782925	13.39276	14.97377	4.019918
13655	LOC11961	13.04861	1.431861	0.689503	2.076658	0.037833	0.073656	7.047123	6.568942	7.653045	13.39276	23.53022	20.09959
13414	RPP25	18.53343	1.431241	0.673857	2.123954	0.033674	0.066738	10.84173	4.379295	14.61036	26.78552	38.50399	16.07967
5700	SRRM3	61.27466	1.430716	0.330622	4.327343	1.51E-05	7.04E-05	33.60935	32.2973	33.39511	104.91	77.00798	86.42823

959	FAM214A	1127.405	1.430292	0.128139	11.16204	6.25E-29	1.73E-27	658.0929	644.8511	528.0601	1533.471	1666.367	1733.59
13886	SSTR2	22.61272	1.430194	0.703486	2.033012	0.042051	0.080508	5.420864	12.04306	18.78475	40.17829	49.19955	10.04979
609	IQSEC1	3092.499	1.43013	0.105958	13.4972	1.62E-41	7.09E-40	1550.367	1837.114	1637.056	4314.701	4395.872	4819.882
14068	DNAI1	15.35344	1.429423	0.714652	2.000166	0.045482	0.08595	9.757555	10.40082	4.174388	26.78552	29.94755	11.05477
14573	LOC10323	14.07558	1.428717	0.747431	1.911505	0.05594	0.102049	7.589209	6.568942	8.348776	13.39276	38.50399	10.04979
1032	LOC10322	762.0586	1.428454	0.131506	10.86228	1.74E-27	4.49E-26	455.3526	389.2098	392.3925	1078.117	1200.041	1057.238
19375	LOC10322	3.863973	1.427715	1.27086	1.123424	0.261257	0.35846	2.710432	2.737059	0.695731	4.464254	8.556443	4.019918
8005	CDC42BPG	70.86082	1.427591	0.418593	3.410447	0.000649	0.002154	36.86187	31.20247	45.91827	122.767	141.1813	47.23404
5442	LOC11962	82.50799	1.426749	0.321183	4.442162	8.91E-06	4.35E-05	42.28274	33.93953	57.7457	120.5349	139.0422	101.5029
18263	LOC10323	5.502374	1.425444	1.104009	1.291152	0.196651	0.28626	4.336691	2.737059	2.087194	2.232127	8.556443	13.06473
14926	LOC11962	12.33076	1.42518	0.770604	1.849432	0.064395	0.114696	5.420864	10.94824	3.478657	13.39276	25.66933	15.07469
11396	LOC11962	22.00509	1.422239	0.560357	2.538094	0.011146	0.026001	9.215468	13.6853	12.52316	42.41041	32.08666	22.10955
14509	MMP23B	17.35678	1.421932	0.739719	1.922259	0.054573	0.099995	11.38381	2.189647	14.61036	26.78552	32.08666	17.08465
656	FNIP2	1271.821	1.421842	0.108659	13.08532	3.99E-39	1.62E-37	730.1903	702.8768	640.7686	1854.898	1811.827	1890.366
5994	DYNC1I1	62.75202	1.420902	0.341044	4.166332	3.10E-05	0.000137	39.03022	27.37059	35.4823	89.28508	106.9555	78.3884
18795	LOC10324	5.589034	1.419578	1.164689	1.218847	0.222902	0.315289	1.084173	4.379295	3.478657	4.464254	17.11289	3.014938
14175	LOC10321	12.18483	1.41917	0.716125	1.981735	0.047509	0.089104	5.420864	6.02153	8.348776	17.85702	21.39111	14.06971
3116	LOC11962	175.8271	1.419025	0.22662	6.26168	3.81E-10	3.25E-09	101.9122	77.73248	107.8384	225.4448	288.7799	253.2548
3436	CXXC4	139.6568	1.418444	0.239871	5.913349	3.35E-09	2.59E-08	62.88202	82.65918	82.0963	225.4448	198.9373	185.9212
9932	LOC11962	33.42006	1.4182	0.492724	2.878285	0.003998	0.010701	11.9259	26.82318	15.30609	55.80317	53.47777	37.18424
21612	LOC11962	2.24977	1.418042	1.796678	0.789258	0.429961	0.528897	1.626259	0.547412	1.391463	8.928508	0	1.004979
12886	P2RX5	20.6806	1.418016	0.638365	2.221325	0.026329	0.054319	13.55216	7.116354	12.52316	46.87467	29.94755	14.06971
14505	LOC11962	13.06066	1.417514	0.737053	1.92322	0.054452	0.099801	7.047123	6.568942	7.653045	8.928508	32.08666	16.07967
17112	LOC11962	8.996438	1.417406	0.957946	1.47963	0.138972	0.215905	10.29964	2.737059	1.391463	13.39276	17.11289	9.044815
18941	LOC10323	5.622511	1.417202	1.184373	1.196584	0.231469	0.324883	3.252518	2.189647	3.478657	6.696381	17.11289	1.004979
1278	LOC11962	432.3447	1.416142	0.142909	9.909364	3.79E-23	7.89E-22	241.7705	224.9863	240.0273	660.7096	607.5074	619.0674
18523	TRNAL-CA	6.417809	1.415888	1.135025	1.247451	0.212232	0.304605	1.084173	1.094824	8.348776	13.39276	8.556443	6.029877
787	ADGRE5	1308.992	1.415289	0.116102	12.19006	3.51E-34	1.19E-32	722.059	719.8465	703.3844	1841.505	1747.653	2119.502
14450	LOC10321	17.77041	1.415145	0.731594	1.934331	0.053072	0.097642	8.131296	9.853413	10.43597	37.94616	34.22577	6.029877
10557	LOC11962	33.11952	1.41501	0.517873	2.732349	0.006288	0.015836	17.88885	27.37059	8.348776	53.57105	51.33866	40.19918
20680	LOC11962	3.669958	1.414913	1.530283	0.924609	0.355169	0.456585	0	0.547412	5.565851	4.464254	6.417332	5.024897
836	SIRPA	7254.163	1.414636	0.119403	11.8476	2.21E-32	7.04E-31	3533.861	4599.354	3741.643	10401.71	9822.796	11425.61
1401	GJB4	769.7568	1.413946	0.148455	9.524425	1.66E-21	3.15E-20	472.6993	378.809	411.1772	1118.296	973.2954	1264.264
20359	LOC11961	2.927026	1.413559	1.460253	0.968023	0.333033	0.434878	0.542086	2.737059	1.391463	4.464254	6.417332	2.009959
11942	LOC11962	26.55499	1.412663	0.58467	2.416171	0.015685	0.034917	8.673382	14.78012	19.48048	49.10679	49.19955	18.08963
17233	ASIC2	7.115763	1.411425	0.965268	1.46221	0.143684	0.221658	1.626259	4.379295	5.565851	13.39276	10.69555	7.034856
20626	SH2D7	2.679818	1.410925	1.513514	0.932218	0.351224	0.452695	1.084173	0.547412	2.782925	2.232127	6.417332	3.014938
9567	LOC11962	28.24569	1.41074	0.475276	2.968254	0.002995	0.008323	13.01007	15.87494	17.39328	37.94616	47.06043	38.18922
2357	KCNV1	247.8148	1.409797	0.192383	7.328079	2.33E-13	2.63E-12	118.1748	143.4219	146.7993	348.2118	327.2839	402.9968
6507	LOC11962	64.10875	1.408364	0.357649	3.937841	8.22E-05	0.000336	30.89892	43.79295	29.91645	122.767	74.86887	82.40832
1523	DAB2	3679.811	1.4079	0.153551	9.168938	4.78E-20	8.34E-19	1716.245	2599.111	1728.197	5220.945	5123.17	5691.199
5117	LOC11962	76.25705	1.407552	0.304644	4.62032	3.83E-06	1.99E-05	39.03022	45.98259	41.04815	93.74933	109.0946	128.6374
16259	MUC1	8.571429	1.406244	0.870442	1.615551	0.106191	0.173633	3.794605	3.831883	6.261582	13.39276	17.11289	7.034856
13738	C1QTNF9	16.0434	1.406158	0.681863	2.06223	0.039186	0.075827	4.878777	13.6853	7.653045	17.85702	32.08666	20.09959
8170	LOC10323	50.72796	1.406023	0.418977	3.35585	0.000791	0.002575	22.22554	29.56024	30.61218	80.35657	98.39909	43.21412
1085	INTS6L	680.0435	1.406003	0.133159	10.55885	4.62E-26	1.13E-24	350.1878	403.9899	365.259	906.2436	1005.382	1049.199
864	THSD1	1087.135	1.405576	0.120051	11.70817	1.16E-31	3.56E-30	595.2108	644.3037	548.9321	1591.507	1469.569	1673.291
17710	LOC11961	7.249848	1.404607	1.015553	1.383097	0.166635	0.250141	2.168345	4.926706	4.87012	2.232127	19.252	10.04979
15553	H19	22.01965	1.4044	0.809373	1.73517	0.082711	0.141379	2.168345	11.49565	22.2634	37.94616	49.19955	9.044815
475	PROS1	5170.719	1.404227	0.094514	14.85727	6.24E-50	3.49E-48	2706.637	3090.14	2711.265	7368.251	7439.827	7708.193
14718	FAM83C	12.99434	1.403609	0.743983	1.886614	0.059212	0.106955	6.505036	5.474118	9.044508	20.08914	27.80844	9.044815
3164	IFI44	153.3023	1.403144	0.226239	6.202039	5.57E-10	4.68E-09	88.90216	85.94365	77.22618	191.9629	256.6933	219.0855
14190	GIMAP2	12.47012	1.403097	0.709047	1.978851	0.047833	0.089615	5.420864	8.758589	6.261582	22.32127	14.97377	17.08465
15578	LOC11962	9.550374	1.40274	0.809626	1.732579	0.083171	0.141929	4.878777	4.379295	6.261582	15.62489	17.11289	9.044815
8472	LOC11962	60.67674	1.40234	0.429919	3.26187	0.001107	0.003473	36.86187	31.74989	29.91645	95.98146	128.3466	41.20416
6065	WFDC3	75.72173	1.401408	0.33854	4.139566	3.48E-05	0.000153	43.36691	29.01283	52.17985	102.6778	132.6249	94.46807
14461	LOC11961	13.72644	1.401033	0.725138	1.932091	0.053348	0.098072	5.96295	6.568942	9.740239	20.08914	29.94755	10.04979
15878	LOC11961	11.54759	1.400996	0.829913	1.688123	0.091388	0.153009	5.420864	8.211177	4.87012	20.08914	25.66933	5.024897
1277	TPP1	1323.342	1.400197	0.14121	9.915672	3.56E-23	7.41E-22	628.2781	871.4796	683.2082	1832.576	1865.305	2059.203
22318	LOC11962	1.489334	1.40008	2.082577	0.672282	0.501404	0.597273	0.542086	1.094824	0.695731	4.464254	2.139111	0
15210	LOC11962	10.22497	1.399038	0.779525	1.794731	0.072697	0.127064	4.878777	6.02153	6.261582	13.39276	10.69555	20.09959
14907	LOC11962	11.73902	1.398768	0.754923	1.852863	0.063902	0.113962	5.96295	5.474118	7.653045	15.62489	25.66933	10.04979
8037	LOC11962	46.93358	1.398717	0.411108	3.402314	0.000668	0.00221	27.10432	20.80165	29.22072	95.98146	49.19955	59.29379
15040	LOC10323	13.89866	1.398554	0.766076	1.825607	0.067909	0.120032	8.673382	8.211177	5.565851	13.39276	38.50399	9.044815
20925	LOC10324	3.429354	1.398421	1.576001	0.887323	0.374905	0.476313	1.084173	1.642235	2.782925	2.232127	12.83466	0
5089	NNMT	81.83409	1.398364	0.301435	4.63903	3.50E-06	1.83E-05	50.95612	43.79295	40.35242	136.1597	94.12087	125.6224
23205	LRRTM1	1.206874	1.398319	2.644689	0.528727	0.596995	0.683952	0	0	2.087194	0	2.139111	3.014938
9686	LOC11961	27.6175	1.398139	0.476354	2.935086	0.003335	0.009152	13.55216	14.78012	17.39328	42.41041	36.36488	41.20416
16446	RBP5	8.564978	1.397795	0.884475	1.580367	0.114023	0.184318	2.168345	4.379295	7.653045	11.16063	14.97377	11.05477
7542	LOC10324	52.16833	1.397178	0.394024	3.545923	0.000391	0.001379	34.15144	21.89647	29.22072	80.35657	94.12087	53.26391
6471	NYAP1	119.1545	1.396795	0.353539	3.950891	7.79E-05	0.00032	45.53526	49.26706	101.5768	183.0344	213.9111	121.6025
15285	LOC11962	12.59332	1.396487	0.783547	1.782264	0.074706	0.129936	9.215468	5.474118	5.565851	29.01765	19.252	7.034856
582	ERLIN2	3019.131	1.396463	0.101215	13.79702	2.66E-43	1.21E-41	1670.71	1787.847	1529.218	4316.934	4218.326	4591.751
3224	SAMD14	146.065	1.396379	0.227527	6.13719	8.40E-10	6.93E-09	72.09749	88.68071	81.40057	203.1236	198.9373	232.1503

2494	MMP15	300.7183	1.395052	0.195333	7.141929	9.20E-13	9.81E-12	159.3734	149.9908	190.6304	406.2471	376.4835	521.5843
15056	LOC10322:	11.03082	1.394359	0.764668	1.823484	0.06823	0.120469	8.131296	4.926706	4.87012	20.08914	17.11289	11.05477
6314	LOC11962!	58.07877	1.39351	0.346715	4.019179	5.84E-05	0.000246	31.44101	27.918	36.17803	93.74933	89.84265	69.34358
1165	EFNA5	695.9205	1.392937	0.135526	10.27799	8.86E-25	2.02E-23	348.5615	429.1709	374.9992	977.6716	981.8518	1063.268
17706	LOC10322(	7.110365	1.391766	1.006118	1.383304	0.166572	0.250095	1.084173	4.379295	6.261582	8.928508	14.97377	7.034856
1168	ECHDC3	1080.269	1.391599	0.135509	10.26941	9.68E-25	2.20E-23	662.4295	519.4938	608.7649	1600.435	1401.117	1689.37
22228	PDHA2	1.492529	1.391599	2.024194	0.687483	0.491778	0.588164	0.542086	0.547412	1.391463	4.464254	0	2.009959
5729	TRPM1	81.1948	1.390852	0.322728	4.30968	1.63E-05	7.59E-05	33.06727	50.9093	50.09266	107.1421	145.4595	100.4979
3398	ARID5A	126.4656	1.389891	0.23373	5.946559	2.74E-09	2.14E-08	67.21871	66.78424	75.83472	187.4987	177.5462	183.9112
20900	LOC10322{	2.469167	1.388417	1.559208	0.890463	0.373218	0.474736	1.084173	2.189647	0.695731	6.696381	2.139111	2.009959
12790	FGD5	20.44611	1.38812	0.619385	2.241127	0.025018	0.052002	11.9259	11.49565	9.740239	37.94616	38.50399	13.06473
22707	LOC11962:	1.458329	1.387046	2.282328	0.607733	0.543364	0.63617	0.542086	1.094824	0.695731	0	6.417332	0
19718	LOC10324:	3.928383	1.386696	1.300495	1.066283	0.286296	0.386001	1.084173	3.831883	1.391463	6.696381	8.556443	2.009959
747	PLS1	3194.742	1.386412	0.110979	12.49258	8.20E-36	2.92E-34	1767.202	1924.7	1614.097	4267.827	4545.61	5049.017
788	USH1C	1212.525	1.385704	0.11376	12.18093	3.93E-34	1.33E-32	659.7191	694.1182	663.032	1660.702	1677.063	1920.516
1742	LOC10323!	364.9897	1.385299	0.160886	8.610451	7.28E-18	1.11E-16	199.4878	194.3312	213.5895	564.7281	462.0479	555.7536
12116	LOC11962(	25.48885	1.384974	0.581316	2.382482	0.017196	0.037732	9.757555	15.87494	16.00182	53.57105	40.6431	17.08465
7880	LOC10321(	43.21325	1.38374	0.401483	3.446575	0.000568	0.001915	27.6464	22.9913	20.87194	51.33892	79.14709	57.28383
15037	LOC10323{	12.73321	1.383134	0.757353	1.826275	0.067809	0.119884	7.047123	5.474118	8.348776	13.39276	32.08666	10.04979
23115	LOC10323:	0.978945	1.382207	2.554401	0.541108	0.588433	0.676768	1.084173	0.547412	0	2.232127	0	2.009959
4859	LOC11962	115.2736	1.381881	0.289525	4.772928	1.82E-06	9.93E-06	72.09749	47.62483	70.9646	180.8023	192.52	127.6324
22969	NKAPL	1.243902	1.381318	2.428771	0.568731	0.569539	0.659172	0	0	2.087194	2.232127	2.139111	1.004979
22970	LOC10323!	1.243902	1.381318	2.428771	0.568731	0.569539	0.659172	0	0	2.087194	2.232127	2.139111	1.004979
16375	LOC11962(	8.571227	1.380911	0.866912	1.592907	0.111181	0.180504	4.878777	4.926706	4.174388	11.16063	19.252	7.034856
862	SEC14L2	1180.906	1.380403	0.117843	11.71395	1.08E-31	3.33E-30	710.1331	587.9203	669.2936	1647.31	1724.123	1746.654
23549	LOC11962:	0.730318	1.380043	2.897011	0.476368	0.633812	0.715403	0.542086	0	0.695731	0	2.139111	1.004979
23550	LOC11961!	0.730318	1.380043	2.897011	0.476368	0.633812	0.715403	0.542086	0	0.695731	0	2.139111	1.004979
23551	LOC11961!	0.730318	1.380043	2.897011	0.476368	0.633812	0.715403	0.542086	0	0.695731	0	2.139111	1.004979
23553	LOC10322:	0.730318	1.380043	2.897011	0.476368	0.633812	0.715403	0.542086	0	0.695731	0	2.139111	1.004979
13845	LOC10323:	14.42791	1.379761	0.675927	2.041286	0.041222	0.079155	9.215468	8.211177	6.261582	17.85702	29.94755	15.07469
18434	LOC10321{	5.157315	1.379005	1.095167	1.259172	0.207968	0.299926	2.710432	1.642235	4.174388	6.696381	10.69555	5.024897
23127	LOC10323!	0.979833	1.378573	2.553866	0.539799	0.589336	0.677409	0.542086	1.094824	0	2.232127	0	2.009959
16545	LOC11962(	7.844991	1.378222	0.8799	1.56634	0.117269	0.188431	3.794605	4.926706	4.174388	11.16063	14.97377	8.039836
9091	MYBPC2	57.90814	1.37809	0.446616	3.085629	0.002031	0.00594	28.18849	19.70683	48.00546	104.91	98.39909	48.23901
777	KBTBD11	3187.46	1.377894	0.112389	12.26004	1.48E-34	5.08E-33	1647.943	1961.924	1707.325	4448.629	4303.891	5055.047
4390	TMOD1	104.9025	1.377059	0.27079	5.085343	3.67E-07	2.22E-06	46.07734	64.04718	65.39875	151.7846	143.3204	158.7868
21207	LOC11962:	2.721816	1.377025	1.621974	0.848981	0.395892	0.496271	0	1.094824	3.478657	4.464254	4.278221	3.014938
1177	LIN7A	871.1878	1.376955	0.134493	10.2381	1.34E-24	3.02E-23	448.3054	558.3601	446.6595	1272.312	1200.041	1301.448
21170	LOC11961!	2.918077	1.376624	1.610291	0.854892	0.392611	0.493036	2.168345	0.547412	2.087194	0	10.69555	2.009959
19421	LOC10322(	4.995418	1.376358	1.234538	1.114877	0.264903	0.36262	2.168345	0.547412	5.565851	13.39276	4.278221	4.019918
23537	CXCL11	0.746708	1.375244	2.878559	0.477754	0.632825	0.714635	0	0.547412	0.695731	2.232127	0	1.004979
23538	LOC10324{	0.746708	1.375244	2.878559	0.477754	0.632825	0.714635	0	0.547412	0.695731	2.232127	0	1.004979
23540	TRNAG-CC	0.746708	1.375244	2.878559	0.477754	0.632825	0.714635	0	0.547412	0.695731	2.232127	0	1.004979
23541	NAT2	0.746708	1.375244	2.878559	0.477754	0.632825	0.714635	0	0.547412	0.695731	2.232127	0	1.004979
23542	LOC11961!	0.746708	1.375244	2.878559	0.477754	0.632825	0.714635	0	0.547412	0.695731	2.232127	0	1.004979
3969	LOC10322:	127.9454	1.375162	0.254692	5.399313	6.69E-08	4.48E-07	71.01331	65.14201	77.22618	227.677	149.7377	176.8764
8686	SAMD5	35.84202	1.375097	0.428695	3.207633	0.001338	0.004096	21.68345	17.51718	20.17621	53.57105	59.8951	42.20914
23563	LOC10323:	0.731206	1.375051	2.896028	0.474806	0.634925	0.71634	0	0.547412	0.695731	0	2.139111	1.004979
15205	MFSD2B	12.13339	1.374093	0.765266	1.795575	0.072562	0.12687	4.336691	6.568942	9.044508	24.5534	19.252	9.044815
21962	IL22	1.975107	1.373529	1.870851	0.734173	0.462843	0.560272	0	0.547412	2.782925	2.232127	4.278221	2.009959
20951	LOC10322:	2.437274	1.372723	1.551264	0.884906	0.376207	0.477374	1.626259	1.642235	0.695731	2.232127	6.417332	2.009959
14270	LOC10321:	14.37694	1.372492	0.698007	1.9663	0.049264	0.091779	7.047123	8.211177	8.348776	33.4819	17.11289	12.05975
20934	TAGLN3	3.469577	1.372205	1.548747	0.88601	0.375612	0.47673	1.084173	1.094824	3.478657	4.464254	10.69555	0
20998	LOC11962:	2.438162	1.372077	1.562438	0.878164	0.379855	0.480632	1.084173	2.189647	0.695731	2.232127	6.417332	2.009959
1076	ADCK5	1288.199	1.371974	0.129155	10.6227	2.34E-26	5.77E-25	806.6245	619.6702	729.1265	1886.147	1736.958	1950.665
17562	LOC10324(	6.902186	1.371557	0.975873	1.405466	0.159883	0.242027	3.252518	3.831883	4.174388	11.16063	14.97377	4.019918
611	STAT6	2940.58	1.370471	0.101662	13.48068	2.03E-41	8.84E-40	1612.165	1764.856	1545.915	4120.506	4068.588	4531.452
1050	SCAMP5	611.482	1.370183	0.127363	10.75811	5.43E-27	1.37E-25	343.6828	356.3651	323.5151	857.1368	887.7309	900.4616
7016	ACTC1	58.27163	1.370132	0.367551	3.727736	0.000193	0.000732	34.69353	28.46541	34.09084	62.49956	115.512	74.36848
559	PACSIN1	4163.154	1.370022	0.097346	14.07369	5.51E-45	2.62E-43	2261.042	2454.047	2256.257	5903.976	5636.557	6467.043
4720	GRM8	84.82014	1.369772	0.281278	4.869816	1.12E-06	6.29E-06	44.45108	46.53	50.78839	133.9276	117.6511	115.5726
20117	LOC10323	3.172562	1.369154	1.356046	1.009666	0.312655	0.41318	1.084173	2.189647	2.087194	2.232127	6.417332	5.024897
1776	LOC10323!	433.1494	1.368362	0.160597	8.520449	1.59E-17	2.38E-16	232.013	268.7792	226.8084	591.5136	573.2817	706.5006
14503	LOC10321!	12.2757	1.36799	0.711062	1.923868	0.054371	0.099666	4.878777	8.758589	6.957314	17.85702	17.11289	18.08963
999	SEMA3B	3431.315	1.367935	0.124502	10.98727	4.40E-28	1.17E-26	2102.753	1680.007	1970.311	4845.948	4417.264	5571.606
21254	LOC10322:	2.460054	1.367935	1.625557	0.841518	0.400058	0.500402	1.084173	1.094824	2.087194	4.464254	0	6.029877
15577	LOC10321!	12.07427	1.367552	0.789297	1.732621	0.083163	0.141929	5.420864	8.758589	5.565851	17.85702	27.80844	7.034856
21063	LOC11962{	3.50631	1.366594	1.569282	0.87084	0.383841	0.484472	4.336691	0	1.391463	11.16063	2.139111	2.009959
16209	LOC11962	10.26049	1.366528	0.840434	1.625979	0.103954	0.170499	7.589209	2.189647	7.653045	8.928508	17.11289	18.08963
11265	LOC11962	22.97232	1.366323	0.532384	2.566423	0.010275	0.024249	13.01007	12.59047	13.2189	22.32127	38.50399	38.18922
539	GINM1	1927.826	1.366068	0.09621	14.19881	9.32E-46	4.60E-44	1047.311	1126.574	1058.207	2747.748	2853.574	2733.544
18912	TMEM210	5.47911	1.365331	1.136294	1.201565	0.229532	0.322658	3.794605	1.642235	3.478657	13.39276	8.556443	2.009959
1315	PLAT	1075.81	1.365188	0.139212	9.806509	1.06E-22	2.13E-21	610.3893	644.8511	553.1064	1466.507	1369.031	1810.973
12965	LOC11961{	16.9109	1.364882	0.617483	2.210397	0.027078	0.055523	8.673382	12.59047	6.957314	22.32127	27.80844	23.11453

19354	CUNH2orf1	4.199068	1.364088	1.211009	1.126406	0.259994	0.357151	3.252518	2.189647	1.391463	8.928508	6.417332	3.014938
3889	LOC11962	112.1179	1.36396	0.249953	5.456869	4.85E-08	3.31E-07	58.54533	68.42648	61.22436	174.1059	147.5986	162.8067
8921	LOC11962	48.80341	1.363622	0.434407	3.139041	0.001695	0.005051	28.18849	15.32753	38.26523	69.19594	85.56443	56.27885
12003	LOC11962	28.58378	1.363085	0.566704	2.405286	0.01616	0.035792	17.34676	12.59047	17.39328	33.4819	70.59065	20.09959
23533	LOC11962	0.962555	1.36294	2.850069	0.478213	0.632499	0.714566	1.626259	0	0	0	2.139111	2.009959
17434	LOC10324	6.394991	1.362703	0.959331	1.420471	0.155471	0.237063	3.794605	2.737059	4.174388	8.928508	10.69555	8.039836
16568	LOC11962	8.111887	1.362217	0.871839	1.562465	0.118179	0.189629	2.710432	5.474118	5.565851	11.16063	10.69555	13.06473
23191	LOC11962	0.963442	1.361985	2.566286	0.530722	0.595611	0.682779	1.084173	0.547412	0	0	2.139111	2.009959
3998	TMC5	108.3278	1.36171	0.253284	5.376212	7.61E-08	5.06E-07	56.91907	67.87906	57.04997	151.7846	162.5724	153.7619
14690	LOC10321	19.31782	1.360453	0.719205	1.891607	0.058543	0.10595	7.047123	7.663765	17.39328	24.5534	49.19955	10.04979
1751	LOC10324	376.8667	1.360199	0.15834	8.590376	8.67E-18	1.32E-16	230.3867	191.0467	213.5895	537.9426	513.3866	574.8483
19695	LOC10324	4.21948	1.360117	1.26996	1.070993	0.284173	0.383568	1.084173	1.094824	4.87012	6.696381	8.556443	3.014938
23321	LOC11962	1.228399	1.359504	2.662276	0.510655	0.609593	0.694899	0	0	2.087194	0	4.278221	1.004979
14096	LOC11962	14.83684	1.359367	0.681482	1.994722	0.046073	0.086889	7.047123	7.116354	10.43597	24.5534	27.80844	12.05975
4591	DNM3	121.2036	1.359106	0.27468	4.94796	7.50E-07	4.34E-06	53.12446	84.84883	65.39875	160.7131	205.3546	157.7818
1028	SNN	710.4215	1.358835	0.124935	10.87637	1.49E-27	3.86E-26	396.8072	406.727	394.4797	959.8146	1003.243	1101.457
1913	EGFLAM	426.2489	1.358794	0.16629	8.171211	3.05E-16	4.24E-15	234.1813	206.3743	276.2054	658.4775	622.4812	559.7736
10657	TRIM7	41.49003	1.358577	0.502854	2.701731	0.006898	0.017208	16.80468	16.96977	35.4823	73.66019	74.86887	31.15436
683	A4GALT	2087.958	1.358358	0.105343	12.89468	4.82E-38	1.88E-36	1178.496	1183.504	1156.306	3031.228	2723.088	3255.129
15203	RNF125	12.93096	1.358139	0.756296	1.795778	0.07253	0.126831	5.420864	4.379295	11.82743	26.78552	17.11289	12.05975
2696	NATD1	227.8163	1.358081	0.198584	6.838837	7.98E-12	7.87E-11	134.4374	115.5039	135.6676	312.4978	290.9191	377.8723
14029	ADAMTS8	14.80851	1.358077	0.676378	2.007865	0.044658	0.084627	7.589209	4.926706	12.52316	22.32127	21.39111	20.09959
19365	LSMEM2	4.184453	1.357801	1.206618	1.125295	0.260464	0.357557	2.710432	2.737059	1.391463	6.696381	8.556443	3.014938
10903	LOC10322	24.65621	1.357672	0.512271	2.650299	0.008042	0.019607	11.9259	13.13788	16.69755	40.17829	27.80844	38.18922
18532	IL34	6.412966	1.357175	1.088839	1.246442	0.212602	0.304922	5.96295	0.547412	4.174388	8.928508	12.83466	6.029877
12858	LOC11962	25.55129	1.356511	0.609174	2.226803	0.02596	0.053674	11.9259	13.6853	16.69755	40.17829	57.75599	13.06473
11284	LOC10322	30.41048	1.3556	0.52927	2.561262	0.010429	0.024571	12.46799	15.32753	22.95914	42.41041	64.17332	25.12449
5109	LOC11962	106.4605	1.354662	0.292991	4.623568	3.77E-06	1.96E-05	72.63957	46.53	59.13717	171.8738	168.9897	119.5926
19387	LOC10322	4.955349	1.354147	1.209496	1.119596	0.262886	0.360486	1.084173	4.926706	2.087194	8.928508	10.69555	2.009959
2373	PLBD2	319.918	1.354	0.185291	7.307409	2.72E-13	3.05E-12	207.077	153.8227	180.1944	468.7467	402.1528	507.5146
18081	LOC11961	6.715012	1.353066	1.020446	1.325955	0.184855	0.271797	5.96295	1.642235	3.478657	15.62489	8.556443	5.024897
14667	LOC11962	15.16569	1.35302	0.714193	1.894473	0.058162	0.105423	4.878777	6.02153	14.61036	29.01765	21.39111	15.07469
9246	LOC11962	35.7308	1.35271	0.444478	3.043366	0.002339	0.006727	17.34676	20.25424	22.2634	46.87467	68.45154	39.1942
7894	LOC10324	53.74562	1.352429	0.392916	3.44203	0.000577	0.001944	37.40396	20.25424	33.39511	58.0353	91.98176	81.40334
11245	LOC10321	30.82118	1.352393	0.525905	2.571551	0.010124	0.023936	18.97302	11.49565	20.87194	60.26743	49.19955	24.11951
630	LOC10324	3061.489	1.351922	0.101819	13.27773	3.12E-40	1.32E-38	1666.373	1872.148	1634.273	4261.13	4256.83	4678.179
15568	CA5A	10.6086	1.351855	0.779812	1.733564	0.082995	0.141729	3.794605	7.663765	6.261582	15.62489	19.252	11.05477
16925	APOA5	11.33597	1.351845	0.899626	1.502675	0.132923	0.208789	2.168345	3.831883	13.2189	8.928508	27.80844	12.05975
13346	CUNH11or	16.16927	1.351527	0.632183	2.137875	0.032527	0.064793	7.589209	10.94824	9.044508	15.62489	25.66933	28.13943
1604	CDHR2	377.8401	1.351495	0.150746	8.965364	3.09E-19	5.13E-18	214.6662	217.3225	206.6322	566.9603	502.691	558.7686
2993	COMMD5	874.9343	1.351233	0.210123	6.430684	1.27E-10	1.13E-09	613.0997	309.2877	557.2808	1200.884	1187.206	1381.847
15841	BMERB1	10.44227	1.350866	0.798172	1.692448	0.090561	0.151982	5.96295	3.831883	7.653045	22.32127	12.83466	10.04979
936	CSTB	1059.932	1.349744	0.119567	11.28859	1.49E-29	4.24E-28	658.6349	546.8644	587.1973	1475.436	1533.742	1557.718
10454	LOC11962	29.66933	1.349733	0.489966	2.75475	0.005874	0.014935	14.09425	14.78012	20.87194	44.64254	53.47777	30.14938
20953	LOC11961	2.492999	1.349537	1.525266	0.884788	0.376271	0.47741	1.626259	1.094824	1.391463	6.696381	2.139111	2.009959
4682	SLC29A3	96.36997	1.348842	0.275575	4.894645	9.85E-07	5.59E-06	55.8349	61.85754	45.22254	124.9991	147.5986	142.7071
6440	LOC11961	73.76773	1.348001	0.340002	3.964683	7.35E-05	0.000303	34.69353	48.71965	41.04815	80.35657	143.3204	94.46807
15428	LOC11962	14.89148	1.347718	0.767507	1.755967	0.079094	0.136292	9.215468	3.831883	12.52316	6.696381	29.94755	27.13445
1096	TNFSF9	866.4352	1.347517	0.12791	10.53492	5.96E-26	1.45E-24	508.477	476.2483	484.9248	1223.206	1120.894	1384.862
11281	ZPLD1	25.89226	1.346781	0.525735	2.56171	0.010416	0.024545	10.84173	20.80165	11.82743	37.94616	42.78221	31.15436
19171	CUNH2orf	5.723001	1.346001	1.162815	1.157536	0.247053	0.342596	0.542086	2.189647	6.957314	8.928508	10.69555	5.024897
20195	LOC11962	3.424549	1.345958	1.349087	0.997681	0.318434	0.419191	3.252518	1.094824	1.391463	2.232127	8.556443	4.019918
664	HBP1	1395.026	1.345957	0.103093	13.05573	5.89E-39	2.36E-37	803.372	751.049	810.527	1944.183	1995.79	2065.233
4660	PLEKHF1	88.68638	1.344961	0.273731	4.913446	8.95E-07	5.11E-06	49.87195	50.9093	50.09266	122.767	119.7902	138.6872
18515	LOC11962	5.423905	1.344813	1.076824	1.248869	0.211713	0.303991	2.710432	4.926706	1.391463	8.928508	8.556443	6.029877
8935	LOC10322	65.51654	1.344713	0.428898	3.135273	0.001717	0.005109	28.18849	39.96106	41.74388	91.51721	145.4595	46.22906
633	HTRA1	3265.124	1.344666	0.101479	13.25071	4.47E-40	1.88E-38	1915.733	1748.433	1874.3	4636.128	4335.977	5080.171
16367	LOC10323	9.382981	1.344658	0.843409	1.594312	0.110866	0.180069	4.878777	2.737059	8.348776	11.16063	17.11289	12.05975
16733	LOC10321	10.70596	1.344578	0.874938	1.53677	0.12435	0.197568	2.710432	8.758589	6.261582	20.08914	21.39111	5.024897
17730	LOC11961	6.701284	1.344195	0.974465	1.379419	0.167766	0.251554	4.878777	2.737059	3.478657	13.39276	10.69555	5.024897
23568	LOC11962	1.016861	1.34412	2.834499	0.4742	0.635357	0.716691	0.542086	1.094824	0	4.464254	0	0
328	CPE	9119.24	1.343751	0.080151	16.76519	4.39E-63	3.55E-61	5157.952	5192.201	5117.104	12841.43	12815.41	13591.34
14809	LOC10324	13.02693	1.343402	0.718061	1.870873	0.061363	0.110147	7.589209	5.474118	9.044508	11.16063	27.80844	17.08465
22756	LOC10321	1.514942	1.343368	2.237938	0.60027	0.548326	0.640561	0	1.094824	1.391463	4.464254	2.139111	0
22757	RAB36	1.514942	1.343368	2.237938	0.60027	0.548326	0.640561	0	1.094824	1.391463	4.464254	2.139111	0
844	GRN	19262.5	1.34335	0.113854	11.79885	3.96E-32	1.25E-30	11344.24	9849.581	11483.74	27055.61	24520.63	31321.19
832	LRP5	1542.613	1.343111	0.113213	11.86359	1.83E-32	5.85E-31	870.5907	849.5831	900.9721	2145.074	2034.294	2455.165
2283	NOXA1	321.7928	1.342644	0.180365	7.44402	9.77E-14	1.14E-12	191.8986	156.0124	200.3706	430.8005	432.1004	519.5744
887	YPEL2	1516.026	1.342624	0.116096	11.56473	6.22E-31	1.86E-29	887.9375	861.6262	825.8331	2178.556	1942.312	2399.891
13376	CDRT1	24.53758	1.342278	0.629728	2.131521	0.033046	0.06568	11.38381	16.96977	12.52316	31.24978	62.03421	13.06473
18501	LOC11962	5.654306	1.342176	1.072009	1.252019	0.210563	0.302568	3.794605	2.189647	3.478657	4.464254	14.97377	5.024897
20789	LOC10322	4.015739	1.341399	1.480592	0.905989	0.364942	0.466673	0.542086	3.284471	2.782925	8.928508	8.556443	0
16021	PRSS8	10.38469	1.341369	0.807082	1.662	0.096513	0.160162	3.252518	6.568942	7.653045	13.39276	21.39111	10.04979
1499	SHC3	434.06	1.341094	0.145023	9.247465	2.30E-20	4.08E-19	227.1342	252.9043	257.4206	642.8526	598.951	625.0972

22917	TRNAP-UG	1.67543	1.340802	2.324728	0.576757	0.564104	0.654363	1.626259	0	1.391463	0	0	7.034856
18673	LOC11962	5.709204	1.340708	1.088546	1.231651	0.218079	0.310471	5.420864	2.737059	1.391463	13.39276	4.278221	7.034856
1942	SSBP3	445.6121	1.340614	0.165317	8.109337	5.09E-16	6.97E-15	225.5079	302.1713	228.8956	649.549	620.3421	647.2068
18978	TLDC2	5.20853	1.34057	1.127357	1.189127	0.23439	0.328341	1.626259	1.642235	5.565851	6.696381	10.69555	5.024897
22880	LOC11962	1.983663	1.340044	2.301905	0.582146	0.560468	0.65115	3.252518	0	0	2.232127	6.417332	0
11998	JAML	20.27882	1.339876	0.556699	2.406821	0.016092	0.035657	11.38381	9.306001	13.91463	31.24978	25.66933	30.14938
16708	KCNJ5	10.16888	1.339441	0.869156	1.541082	0.123297	0.196184	8.131296	3.831883	4.87012	15.62489	23.53022	5.024897
20831	LOC11962	2.716478	1.339266	1.48811	0.899978	0.368132	0.469797	0.542086	2.737059	1.391463	4.464254	2.139111	5.024897
16465	MYL4	8.608523	1.339116	0.848319	1.578553	0.114439	0.184796	5.96295	3.284471	5.565851	8.928508	12.83466	15.07469
12270	MSH4	24.40084	1.339095	0.569614	2.350883	0.018729	0.040579	13.01007	11.49565	16.69755	22.32127	57.75599	25.12449
19238	LOC11962	5.20698	1.338945	1.167637	1.146713	0.2515	0.347548	5.420864	1.094824	2.087194	8.928508	10.69555	3.014938
20225	LOC11962	5.008718	1.338884	1.350043	0.991734	0.321327	0.422372	1.626259	0	6.957314	11.16063	4.278221	6.029877
1947	EPHX2	520.4557	1.338161	0.165162	8.102124	5.40E-16	7.37E-15	319.2889	250.7146	317.9492	714.2806	665.2634	855.2375
22901	PGAM2	1.98113	1.338022	2.310571	0.579087	0.56253	0.653008	0	0.547412	2.782925	0	8.556443	0
12288	LOC10323	20.2952	1.337517	0.569916	2.346867	0.018932	0.040959	8.673382	13.6853	11.82743	31.24978	34.22577	22.10955
10754	LOC10324	30.00547	1.337308	0.499269	2.678533	0.007395	0.01828	13.01007	18.612	18.78475	46.87467	55.61688	27.13445
9108	XKR4	36.67283	1.337052	0.433999	3.08077	0.002065	0.006026	18.97302	26.82318	16.69755	44.64254	55.61688	57.28383
5930	LOC11961	69.7458	1.336851	0.318766	4.19383	2.74E-05	0.000123	44.99317	33.39212	39.65669	104.91	109.0946	86.42823
15506	LOC10321	13.473	1.336406	0.766668	1.743136	0.08131	0.139406	8.131296	6.568942	7.653045	26.78552	25.66933	6.029877
17833	TOX	6.672942	1.336218	0.974143	1.371686	0.170161	0.253672	3.252518	4.379295	3.478657	8.928508	14.97377	5.024897
5702	MRAP2	73.40533	1.335922	0.30883	4.325753	1.52E-05	7.09E-05	43.36691	33.93953	48.00546	104.91	102.6773	107.5328
910	RPS6KA5	1160.439	1.335758	0.116949	11.42175	3.26E-30	9.51E-29	600.6317	717.1095	657.4661	1620.524	1734.819	1632.087
21867	LOC11961	3.004961	1.33468	1.779109	0.750196	0.453137	0.550899	1.084173	3.831883	0	6.696381	6.417332	0
1825	SYT11	422.1243	1.333867	0.159111	8.383229	5.15E-17	7.50E-16	243.3968	268.7792	207.3279	602.6743	575.4208	635.147
22480	LOC11962	1.514054	1.333617	2.078008	0.641776	0.521018	0.616132	0.542086	0.547412	1.391463	4.464254	2.139111	0
22481	LOC10324	1.514054	1.333617	2.078008	0.641776	0.521018	0.616132	0.542086	0.547412	1.391463	4.464254	2.139111	0
19070	LOC11962	4.447267	1.333604	1.135584	1.174377	0.240244	0.334918	2.168345	3.284471	2.087194	6.696381	6.417332	6.029877
2603	TP53INP2	295.2646	1.332778	0.191015	6.97736	3.01E-12	3.07E-11	159.9155	201.4475	141.9292	383.9258	449.2132	435.1561
21785	SERPINB5	2.232064	1.32835	1.744326	0.761526	0.446343	0.544688	1.084173	0	2.782925	2.232127	4.278221	3.014938
1151	BNC2	675.9258	1.328321	0.128837	10.3101	6.34E-25	1.47E-23	380.0025	417.1278	358.3017	966.511	932.6523	1000.96
4058	TNNT2	128.453	1.328319	0.249349	5.327143	9.98E-08	6.54E-07	67.21871	81.56436	70.26887	158.481	220.3284	172.8565
14482	LOC11962	15.51623	1.328216	0.68864	1.928752	0.053762	0.098692	13.01007	8.211177	5.565851	22.32127	12.83466	31.15436
18179	CLCA2	6.912395	1.328193	1.017116	1.305843	0.191606	0.280205	2.710432	2.189647	6.957314	4.464254	17.11289	8.039836
21142	LOC11961	2.965271	1.327381	1.544643	0.859345	0.39015	0.490594	2.710432	2.189647	0	4.464254	6.417332	2.009959
12700	RNF165	22.18051	1.32721	0.587532	2.258957	0.023886	0.050001	12.46799	15.32753	9.740239	20.08914	51.33866	24.11951
1978	ATP6V0B	2488.033	1.327189	0.165173	8.035128	9.35E-16	1.26E-14	1634.39	1011.617	1609.922	3587.028	3281.396	3803.847
21920	LOC11961	1.747179	1.326781	1.789801	0.741301	0.458511	0.556091	1.084173	1.094824	0.695731	4.464254	2.139111	1.004979
576	LOC10321	2601.604	1.324491	0.095684	13.84235	1.42E-43	6.53E-42	1454.96	1539.869	1462.427	3609.349	3589.428	3953.589
15279	LOC10323	11.43023	1.324269	0.742496	1.783536	0.074499	0.129626	8.131296	4.926706	6.261582	20.08914	17.11289	12.05975
15693	LOC11962	16.10638	1.323765	0.770264	1.718587	0.08569	0.145164	11.9259	5.474118	9.740239	13.39276	47.06043	9.044815
1474	SLC31A2	879.1809	1.32346	0.142026	9.318456	1.18E-20	2.13E-19	548.0493	451.6147	509.9711	1165.17	1155.12	1445.16
957	ADGRG1	10543.06	1.322147	0.118349	11.17164	5.61E-29	1.56E-27	6429.144	5474.666	6173.224	14569.09	13275.32	17336.9
21843	LOC11961	2.720194	1.322076	1.753335	0.754035	0.450828	0.548676	0	3.831883	0.695731	2.232127	8.556443	1.004979
6775	COL26A1	69.29274	1.32205	0.345565	3.825764	0.00013	0.000512	41.74065	27.37059	50.09266	84.82083	111.2338	100.4979
15320	GRIN2C	12.93338	1.321876	0.744226	1.776175	0.075704	0.13137	7.589209	7.116354	6.957314	20.08914	27.80844	8.039836
878	CCDC69	3038.145	1.321839	0.113621	11.63377	2.78E-31	8.40E-30	1657.158	1965.208	1586.963	4397.29	4053.615	4568.637
977	SMIM29	1022.629	1.321553	0.118997	11.10573	1.18E-28	3.20E-27	621.7731	530.442	601.1119	1529.007	1403.257	1450.185
8531	LOC11962	48.37839	1.321377	0.40707	3.246072	0.00117	0.003646	29.27266	18.06459	35.4823	64.73168	83.42532	59.29379
18544	LOC10322	6.400586	1.321163	1.061927	1.244118	0.213456	0.306014	2.168345	3.284471	5.565851	2.232127	17.11289	8.039836
22931	LOC10324	2.052358	1.321019	2.300229	0.574299	0.565765	0.655919	0	0	3.478657	6.696381	2.139111	0
18726	LOC10323	5.199998	1.320428	1.075926	1.227248	0.21973	0.312237	2.168345	3.831883	2.782925	6.696381	10.69555	5.024897
17347	NEK5	7.739077	1.320307	0.917157	1.439564	0.149991	0.229867	2.168345	5.474118	5.565851	15.62489	8.556443	9.044815
628	GRINA	3620.485	1.319854	0.099174	13.30844	2.07E-40	8.75E-39	2079.985	2080.712	2055.886	5214.249	4695.348	5596.731
1513	LOC11962	440.7206	1.319827	0.143249	9.213497	3.16E-20	5.55E-19	252.0702	250.7146	251.8548	638.3883	667.4025	583.8931
20478	LOC11962	4.234259	1.319409	1.388426	0.950291	0.341964	0.443946	2.710432	0.547412	4.174388	8.928508	0	9.044815
20290	CD38	3.735585	1.319289	1.346789	0.979581	0.327293	0.428836	0.542086	1.094824	4.87012	4.464254	6.417332	5.024897
1447	CTSC	554.1754	1.319244	0.140768	9.371781	7.13E-21	1.31E-19	320.9151	328.9945	303.3389	790.173	712.3239	869.3072
14963	LOC10324	12.95889	1.31924	0.716435	1.841395	0.065564	0.116488	6.505036	9.853413	5.565851	26.78552	14.97377	14.06971
17930	SASH3	7.960475	1.31868	0.973173	1.355031	0.175408	0.260079	3.252518	8.758589	1.391463	15.62489	10.69555	8.039836
16899	LOC11962	8.484175	1.31743	0.874329	1.50679	0.131865	0.207445	4.878777	3.831883	5.565851	15.62489	14.97377	6.029877
20791	LOC11961	3.301456	1.31725	1.454207	0.90582	0.365031	0.466757	1.626259	1.094824	2.782925	11.16063	2.139111	1.004979
6407	LOC11962	70.86467	1.317103	0.331265	3.975982	7.01E-05	0.000291	40.11439	35.58177	47.30973	75.89232	102.6773	123.6125
22909	LOC10323	1.483049	1.316692	2.276362	0.578419	0.562981	0.653318	0.542086	0.547412	1.391463	0	6.417332	0
17838	HOATZ	9.262869	1.316681	0.960476	1.370864	0.170417	0.253983	2.168345	7.116354	6.261582	15.62489	21.39111	3.014938
20848	LOC10324	3.000298	1.316386	1.464903	0.898617	0.368857	0.47036	1.084173	0.547412	3.478657	4.464254	6.417332	2.009959
21787	LOC10323	2.768061	1.316225	1.728729	0.761383	0.446428	0.544717	0	0.547412	4.174388	4.464254	6.417332	1.004979
12273	LOC10322	23.63755	1.315924	0.559913	2.350229	0.018762	0.040641	17.88885	8.211177	14.61036	42.41041	23.53022	35.17428
6977	CUNH1orf	57.7238	1.315656	0.351385	3.744195	0.000181	0.00069	28.18849	39.41365	31.30791	73.66019	98.39909	75.37346
4877	DNAJB13	99.18565	1.315329	0.276626	4.754893	1.99E-06	1.08E-05	62.33993	61.31012	45.91827	140.624	158.2942	126.6274
5204	LOC11962	87.73068	1.3149	0.287346	4.576012	4.74E-06	2.42E-05	42.82482	49.26706	59.13717	136.1597	113.3729	125.6224
634	GDF15	5176.875	1.31471	0.099231	13.24894	4.58E-40	1.92E-38	3127.296	2851.468	2931.812	7194.145	6881.519	8075.01
763	DOK4	2083.458	1.314273	0.105915	12.40879	2.34E-35	8.16E-34	1258.725	1142.996	1186.918	2910.694	2774.427	3226.989
9397	TCHH	40.686	1.314087	0.43692	3.007618	0.002633	0.007449	19.51511	31.74989	18.08902	55.80317	72.72976	46.22906
22492	ARMS2	1.501746	1.312977	2.051326	0.640062	0.522132	0.617147	0.542086	0	2.087194	2.232127	2.139111	2.009959

18752	LOC10322	6.257387	1.312908	1.072356	1.224321	0.220831	0.313076	1.626259	1.642235	7.653045	11.16063	6.417332	9.044815
18121	LOC10323	8.793602	1.312453	0.996394	1.317203	0.187771	0.27546	4.878777	1.642235	8.348776	15.62489	19.252	3.014938
3880	LOC11961	147.7526	1.31189	0.239889	5.468748	4.53E-08	3.11E-07	77.51835	87.03848	88.35788	243.3018	222.4675	167.8316
18572	THY1	6.697129	1.311382	1.058095	1.239381	0.215205	0.308056	1.084173	7.116354	3.478657	11.16063	4.278221	13.06473
18053	ESPNL	7.040461	1.311314	0.983162	1.333772	0.182279	0.268425	4.878777	2.189647	4.87012	17.85702	6.417332	6.029877
19524	GNLY	6.222465	1.309265	1.193456	1.097037	0.272625	0.371222	3.794605	0.547412	6.261582	4.464254	19.252	3.014938
1449	MSRA	455.9218	1.309094	0.139748	9.367507	7.43E-21	1.36E-19	250.4439	274.2533	261.595	671.8702	633.1768	644.1918
12110	TEKT5	24.37511	1.309049	0.549025	2.384317	0.017111	0.037566	10.84173	20.25424	10.43597	37.94616	40.6431	26.12947
20448	TRNAT-AG	3.475598	1.308756	1.370981	0.954613	0.339773	0.441749	0.542086	2.189647	3.478657	4.464254	2.139111	8.039836
2197	KLHL21	2466.607	1.308213	0.171808	7.614384	2.65E-14	3.21E-13	1664.747	1048.294	1547.307	3455.333	2960.529	4123.431
3730	CUNH10or	138.3963	1.306638	0.232797	5.612779	1.99E-08	1.42E-07	76.43418	90.87036	71.66033	207.5878	181.8244	202.0009
2794	ALS2CL	315.7921	1.306022	0.19474	6.706485	1.99E-11	1.90E-10	190.8144	147.8012	208.7194	473.2109	380.7617	493.4449
5487	LOC11962	95.38462	1.305569	0.295719	4.4149	1.01E-05	4.90E-05	58.54533	43.79295	63.31155	104.91	154.016	147.732
20980	TRNAY-GU	3.020108	1.305273	1.480899	0.881406	0.378098	0.479111	3.252518	1.094824	0.695731	8.928508	2.139111	2.009959
509	OPTN	3789.452	1.304946	0.090085	14.48565	1.49E-47	7.80E-46	2152.083	2313.91	2085.107	5377.194	5324.246	5484.173
3221	LOC11962	262.0237	1.303665	0.212344	6.139399	8.28E-10	6.84E-09	181.5989	129.7366	140.5377	339.2833	451.3524	329.6333
11012	EGF	31.20545	1.303628	0.496283	2.626783	0.00862	0.020809	17.88885	12.04306	23.65487	46.87467	55.61688	31.15436
3463	LOC10321	174.6794	1.303133	0.221077	5.894465	3.76E-09	2.89E-08	87.81799	114.4091	99.48959	285.7123	222.4675	238.1801
8444	LOC10321	64.93208	1.302619	0.39828	3.270609	0.001073	0.003379	46.61943	19.15941	46.614	84.82083	106.9555	85.42325
2165	ASB2	285.8897	1.301944	0.169867	7.664481	1.80E-14	2.20E-13	166.4205	167.508	161.4097	435.2648	363.6488	421.0864
14933	LOC10324	16.068	1.301734	0.704501	1.847739	0.06464	0.115078	5.96295	8.211177	13.2189	29.01765	29.94755	10.04979
1061	FBXO32	3713.866	1.301651	0.1218	10.68677	1.17E-26	2.94E-25	1981.326	2495.65	1955.701	5049.071	5058.997	5742.453
20746	LOC11962	2.960239	1.301582	1.428821	0.910948	0.362323	0.464299	2.168345	1.094824	2.087194	2.232127	2.139111	8.039836
3314	LOC10323	157.7722	1.300977	0.215737	6.030379	1.64E-09	1.31E-08	86.73382	100.1764	86.96642	216.5163	216.0502	240.1901
2949	APLN	199.906	1.300386	0.200418	6.488365	8.68E-11	7.82E-10	106.791	111.672	129.406	276.7837	265.2497	309.5337
883	PPL	4079.116	1.30023	0.112259	11.58241	5.06E-31	1.52E-29	2310.914	2557.508	2202.686	5834.78	5168.091	6400.714
23392	TRNAW-C(	1.001358	1.299998	2.599072	0.500178	0.61695	0.701163	0.542086	1.094824	0	2.232127	2.139111	0
1024	SORBS3	1281.121	1.299796	0.119376	10.88825	1.31E-27	3.40E-26	670.5608	826.0444	724.9521	1790.166	1777.601	1897.401
971	ITM2B	9177.085	1.299525	0.116746	11.13122	8.84E-29	2.42E-27	4850.047	6256.37	4800.546	13102.59	12714.87	13338.09
930	ID2	2283.343	1.299319	0.114599	11.33795	8.51E-30	2.43E-28	1229.452	1507.572	1219.617	3330.333	3234.335	3178.75
7270	PNCK	80.63066	1.298959	0.356378	3.644887	0.000268	0.000978	30.89892	43.24553	65.39875	111.6063	141.1813	91.45313
19827	LOC11962	4.472152	1.29846	1.236325	1.050257	0.2936	0.393673	3.794605	1.642235	2.087194	4.464254	12.83466	2.009959
8660	LOC10321	67.91606	1.298253	0.403945	3.213934	0.001309	0.004019	52.58238	22.9913	41.74388	75.89232	136.9031	77.38342
23700	LOC11962	0.985855	1.297699	2.861242	0.453544	0.650157	0.729301	0.542086	1.094824	0	0	4.278221	0
4092	SHANK1	199.4686	1.29741	0.244642	5.303308	1.14E-07	7.39E-07	88.90216	117.6935	138.4505	281.248	344.3968	226.1204
5225	FAM228A	82.17376	1.297282	0.28405	4.567093	4.95E-06	2.52E-05	52.04029	44.34036	45.91827	120.5349	117.6511	112.5577
19994	CRIP3	4.026436	1.297154	1.258501	1.030714	0.302675	0.402452	2.710432	2.737059	1.391463	11.16063	2.139111	4.019918
1209	MTUS2	1566.237	1.29593	0.127971	10.12675	4.20E-24	9.24E-23	822.8871	1046.651	851.5752	2238.823	2055.685	2381.801
20036	LOC11962	4.457537	1.295862	1.268169	1.021837	0.306858	0.407158	3.252518	2.189647	2.087194	2.232127	14.97377	2.009959
3515	LOC11962(	328.9509	1.294765	0.221521	5.844897	5.07E-09	3.83E-08	181.5989	157.6546	230.2871	524.5498	536.9168	342.698
19030	LOC11961	5.277248	1.294702	1.097802	1.179358	0.238256	0.332841	2.168345	3.284471	3.478657	11.16063	8.556443	3.014938
4285	VWA7	124.2246	1.29429	0.250604	5.164679	2.41E-07	1.49E-06	73.72375	59.12048	82.79203	180.8023	186.1026	162.8067
17656	LOC11962	7.481727	1.293247	0.926905	1.395232	0.162946	0.245343	5.96295	3.284471	3.478657	11.16063	14.97377	6.029877
21333	LOC10324(	2.791954	1.293126	1.56347	0.827087	0.408188	0.50868	1.626259	1.094824	2.087194	8.928508	0	3.014938
1023	IGF1R	2621.11	1.292771	0.118698	10.8913	1.27E-27	3.30E-26	1348.169	1738.58	1473.559	3582.564	3632.21	3951.579
8084	LOC11962	52.27038	1.292471	0.381601	3.386971	0.000707	0.002324	39.5723	28.46541	22.2634	71.42806	85.56443	66.32864
1089	VPS18	1963.518	1.292239	0.122484	10.5503	5.06E-26	1.24E-24	1145.971	1106.319	1168.133	2763.373	2406.5	3190.81
1075	SLC38A3	3514.928	1.291588	0.121506	10.62982	2.17E-26	5.35E-25	1833.878	2398.759	1884.736	4877.197	4860.059	5234.938
23065	LOC10323	1.004553	1.291436	2.341579	0.551523	0.581275	0.669985	0.542086	0.547412	0.695731	2.232127	0	2.009959
1143	MGAT3	1266.84	1.290599	0.124772	10.34369	4.47E-25	1.04E-23	745.3688	696.8552	768.0874	1649.542	1692.037	2049.153
11650	LOC11962	31.31159	1.290542	0.520169	2.481007	0.013101	0.029897	15.17842	13.13788	25.74206	44.64254	62.03421	27.13445
2258	LOC10324	2420.148	1.290065	0.172073	7.497203	6.52E-14	7.68E-13	1730.882	979.3197	1505.563	3408.458	3270.7	3625.966
1529	CAMKMT	1153.951	1.288897	0.140763	9.156517	5.36E-20	9.32E-19	599.5475	808.5272	601.8076	1620.524	1664.228	1629.072
915	LYST	2001.424	1.288486	0.11292	11.41066	3.70E-30	1.07E-28	1103.146	1298.461	1088.124	2776.766	2723.088	3018.958
23343	LOC10324	1.003665	1.288426	2.539884	0.507277	0.61196	0.696952	1.084173	0	0.695731	2.232127	0	2.009959
4838	LOC10322	106.4792	1.288282	0.268956	4.789927	1.67E-06	9.17E-06	60.71367	50.9093	74.44326	140.624	160.4333	151.7519
20155	LOC10324	4.026376	1.287945	1.284725	1.002506	0.316099	0.416944	1.626259	1.094824	4.174388	6.696381	8.556443	2.009959
18439	BPI	7.172925	1.287874	1.023446	1.25837	0.208258	0.300263	5.420864	5.474118	1.391463	4.464254	19.252	7.034856
5233	SGCA	88.66602	1.287621	0.282184	4.563055	5.04E-06	2.56E-05	58.54533	44.88777	50.78839	138.3919	119.7902	119.5926
15722	LOC11962	11.03458	1.286773	0.750476	1.714609	0.086417	0.146126	6.505036	4.926706	7.653045	20.08914	14.97377	12.05975
15109	LOC11961	14.19037	1.286755	0.710099	1.812078	0.069974	0.123129	4.878777	13.6853	6.261582	20.08914	17.11289	23.11453
18178	LOC11962	6.442086	1.28533	0.983994	1.306238	0.191472	0.280024	5.420864	3.831883	2.087194	6.696381	8.556443	12.05975
6455	LOC11961	63.64065	1.284576	0.324512	3.95848	7.54E-05	0.000311	41.19856	33.39212	36.87376	93.74933	79.14709	97.48301
4105	LOC11962	141.17	1.284189	0.24264	5.292574	1.21E-07	7.81E-07	72.09749	97.4393	75.83472	207.5878	218.1893	175.8714
21498	ENTPD8	3.026793	1.283426	1.597538	0.803378	0.421756	0.521555	0	1.094824	4.174388	4.464254	6.417332	2.009959
22395	LOC11962	2.00733	1.283017	1.94868	0.658403	0.510279	0.60575	2.168345	0	1.391463	4.464254	0	4.019918
16810	TERB2	10.30012	1.282902	0.842044	1.523556	0.12762	0.20183	5.420864	3.284471	9.044508	15.62489	21.39111	7.034856
3406	SCARF1	380.7716	1.281643	0.215837	5.938022	2.88E-09	2.25E-08	271.0432	154.3701	242.1145	508.925	494.1346	614.0425
23360	LOC10323	1.00544	1.281436	2.538897	0.504722	0.613754	0.69843	0	1.094824	0.695731	2.232127	0	2.009959
23363	LOC11962	1.00544	1.281436	2.538897	0.504722	0.613754	0.69843	0	1.094824	0.695731	2.232127	0	2.009959
726	LAMA3	3781.073	1.281313	0.101534	12.61954	1.65E-36	6.03E-35	2158.588	2326.5	2131.721	5229.874	4977.711	5862.045
2540	ZXDB	286.7397	1.280974	0.181321	7.064681	1.61E-12	1.68E-11	150.7	186.12	165.5841	430.8005	355.0924	432.1412
20383	LOC11962	3.543797	1.280944	1.32868	0.964073	0.33501	0.436944	2.168345	1.094824	2.782925	8.928508	4.278221	2.009959
15763	ZC2HC1B	14.29073	1.280786	0.750122	1.707438	0.087741	0.147932	8.131296	9.306001	6.957314	20.08914	34.22577	7.034856

22362	LOC10321!	2.244892	1.280428	1.926515	0.664634	0.506285	0.601883	0	3.284471	0.695731	4.464254	0	5.024897
1153	MINDY1	696.6741	1.28036	0.124196	10.30915	6.41E-25	1.48E-23	405.4806	412.7485	402.1327	1011.154	913.4003	1035.129
19378	LOC10324;	5.1866	1.27981	1.14019	1.122453	0.26167	0.358989	3.794605	3.831883	1.391463	2.232127	12.83466	7.034856
7469	LOC10324:	80.52459	1.279444	0.358053	3.573337	0.000352	0.001255	42.28274	68.97389	29.22072	104.91	126.2075	111.5527
20185	TRNAP-CG	3.96265	1.279124	1.279418	0.99977	0.317422	0.41808	3.794605	1.642235	1.391463	2.232127	10.69555	4.019918
19831	GNMT	3.979928	1.279016	1.219487	1.048815	0.294263	0.394483	2.710432	2.737059	1.391463	4.464254	8.556443	4.019918
20382	EPSTI1	4.701653	1.278903	1.326169	0.964359	0.334866	0.436778	2.710432	3.284471	2.087194	0	17.11289	3.014938
745	PLCB4	3175.082	1.278552	0.102162	12.51493	6.19E-36	2.21E-34	1788.885	2032.54	1740.72	4390.594	4372.342	4725.413
506	LOC10323(	6867.499	1.278197	0.088047	14.51725	9.42E-48	4.95E-46	4067.816	4022.382	3942.71	9662.878	9163.95	10345.26
1425	HRCT1	513.6443	1.278166	0.135246	9.450698	3.37E-21	6.28E-20	318.7468	287.9386	293.5986	707.5843	727.2976	746.6997
10961	ADAMTS5	35.26759	1.278071	0.484695	2.636856	0.008368	0.020295	20.0572	15.87494	25.04633	64.73168	57.75599	28.13943
5405	CCDC125	91.52142	1.277904	0.286493	4.460512	8.18E-06	4.02E-05	56.37698	49.81448	52.87558	151.7846	134.764	103.5129
1697	STEAP1	425.3292	1.277583	0.146235	8.736488	2.40E-18	3.77E-17	241.2284	269.3266	234.4615	613.8349	586.1163	607.0076
7122	TRAF1	63.81482	1.276843	0.346119	3.689028	0.000225	0.00084	38.48813	36.67659	35.4823	116.0706	89.84265	66.32864
18853	ASPA	5.823942	1.276259	1.053258	1.211726	0.225617	0.318148	3.252518	3.284471	3.478657	15.62489	4.278221	5.024897
1160	ARRDC2	1061.598	1.275886	0.123971	10.29184	7.67E-25	1.76E-23	660.8033	609.8168	594.1546	1448.65	1381.865	1674.296
3973	FHDC1	158.207	1.27586	0.236397	5.397103	6.77E-08	4.53E-07	99.20181	104.5557	72.35606	220.9806	248.1368	204.0108
21808	LOC11961ᐤ	2.545102	1.275678	1.681547	0.758634	0.448072	0.546214	0	1.642235	2.782925	6.696381	2.139111	2.009959
14842	LOC10324(	12.98775	1.275274	0.684418	1.863298	0.06242	0.111807	8.673382	7.663765	6.261582	17.85702	21.39111	16.07967
20192	PEBP4	4.281913	1.274751	1.276995	0.998242	0.318162	0.418895	1.626259	2.189647	3.478657	6.696381	10.69555	1.004979
17936	B3GAT2	6.730344	1.274319	0.940982	1.354244	0.175659	0.260364	3.252518	4.379295	4.174388	6.696381	12.83466	9.044815
8141	LOC11961!	47.78861	1.274095	0.378413	3.366938	0.00076	0.002482	22.22554	32.84471	28.52499	66.96381	74.86887	61.30375
760	GLB1	6104.708	1.273589	0.102499	12.42542	1.90E-35	6.65E-34	3551.75	3716.379	3453.611	8546.814	7801.337	9558.36
7565	TTC16	51.13799	1.273407	0.360027	3.536977	0.000405	0.001422	31.9831	30.65506	27.13352	60.26743	83.42532	73.3635
24312	LOC10324;	0.7344	1.272309	3.538155	0.359597	0.719149	0.786384	0	0	1.391463	0	0	3.014938
1182	PCMTD1	1828.433	1.272269	0.12458	10.21245	1.74E-24	3.92E-23	992.0181	1253.573	966.3709	2546.857	2562.655	2649.126
620	ABHD5	3181.381	1.270974	0.094878	13.39594	6.39E-41	2.74E-39	1862.067	1831.64	1902.825	4209.791	4477.159	4804.807
15058	LYPD3	14.2088	1.270764	0.696998	1.823195	0.068274	0.120532	4.878777	12.59047	7.653045	15.62489	21.39111	23.11453
3055	NEXMIF	249.7612	1.270511	0.200591	6.333824	2.39E-10	2.08E-09	126.3061	167.508	146.7993	359.3724	301.6146	396.9669
16284	TAFA3	13.02805	1.270465	0.788787	1.610657	0.107255	0.175102	2.710432	7.663765	12.52316	13.39276	27.80844	14.06971
714	BDH2	3038.947	1.269709	0.099912	12.70832	5.32E-37	1.98E-35	1908.686	1632.929	1804.727	4372.737	4134.901	4379.701
17821	SLC9B1	7.016886	1.26959	0.924368	1.373468	0.169607	0.253016	3.252518	5.474118	3.478657	11.16063	10.69555	8.039836
6719	LOC11962(	62.409	1.2681	0.32952	3.84832	0.000119	0.00047	33.06727	33.39212	43.83108	89.28508	83.42532	91.45313
9044	LOC10323!	42.73693	1.268062	0.409065	3.099905	0.001936	0.00569	18.97302	27.918	27.82925	71.42806	62.03421	48.23901
23414	LOC11962:	0.988162	1.268029	2.551365	0.497	0.619189	0.703056	1.084173	0	0.695731	0	2.139111	2.009959
20245	ZNF534	4.323971	1.268019	1.282948	0.988363	0.322975	0.42412	1.626259	4.379295	1.391463	13.39276	2.139111	3.014938
12279	LOC10322(	24.22395	1.267844	0.539613	2.349542	0.018797	0.040696	10.29964	16.96977	15.30609	24.5534	47.06043	31.15436
1552	ZNRF2	1219.041	1.267085	0.139579	9.077917	1.11E-19	1.90E-18	654.2982	859.984	631.0284	1741.059	1762.627	1665.251
19561	LOC11962ᐤ	4.258245	1.266982	1.163005	1.089404	0.275976	0.375025	2.168345	3.284471	2.087194	6.696381	4.278221	7.034856
975	DOCK4	1663.363	1.266956	0.113955	11.11801	1.03E-28	2.80E-27	910.163	1093.181	927.4099	2314.716	2284.57	2450.14
17565	LOC11962(	7.526552	1.266622	0.901456	1.405084	0.159996	0.242168	4.336691	5.474118	3.478657	13.39276	6.417332	12.05975
1510	KLHDC7A	783.0749	1.266535	0.13724	9.228585	2.74E-20	4.83E-19	507.3928	453.8044	420.2217	1093.742	1003.243	1220.045
12491	LOC11962!	42.45194	1.266274	0.549838	2.302995	0.021279	0.045289	27.6464	9.306001	37.56949	46.87467	94.12087	39.1942
10572	LOC10323(	30.28295	1.266048	0.464028	2.728388	0.006364	0.016003	14.09425	21.89647	17.39328	40.17829	44.92132	43.21412
16523	AVPR2	15.73622	1.265472	0.805583	1.570877	0.116211	0.18698	8.673382	2.737059	16.00182	29.01765	29.94755	8.039836
19202	PNPLA1	10.19565	1.2653	1.097753	1.152627	0.249064	0.344826	0	6.02153	11.82743	22.32127	14.97377	6.029877
22093	LOC10324:	1.978987	1.264104	1.778574	0.71074	0.477245	0.57428	0.542086	1.642235	1.391463	0	4.278221	4.019918
2105	GPAA1	4992.773	1.263762	0.162157	7.793468	6.52E-15	8.23E-14	3349.552	2152.423	3308.898	6872.719	6214.116	8058.93
9522	DHRS3	36.58158	1.262856	0.423955	2.978754	0.002894	0.008081	19.51511	22.44388	22.2634	46.87467	64.17332	44.2191
13303	LOC11962!	20.90915	1.262311	0.588225	2.145967	0.031876	0.063701	12.46799	9.306001	14.61036	33.4819	38.50399	17.08465
4409	GPT	185.6804	1.261399	0.248665	5.072689	3.92E-07	2.36E-06	129.0166	74.44801	125.2316	254.4625	254.5542	276.3694
1672	HLX	434.424	1.261298	0.143468	8.79147	1.48E-18	2.35E-17	250.986	259.4732	258.1163	622.7634	569.0034	646.2018
14965	LOC10323(	15.01315	1.259944	0.684356	1.841064	0.065612	0.116559	10.84173	7.663765	7.653045	15.62489	34.22577	14.06971
14187	GP5	16.08196	1.259088	0.636162	1.979193	0.047794	0.089562	10.29964	6.568942	11.82743	24.5534	17.11289	26.12947
20864	LOC11962;	3.030213	1.259026	1.404865	0.89619	0.370151	0.471648	2.710432	1.094824	1.391463	6.696381	4.278221	2.009959
1782	CCDC9B	610.3947	1.258991	0.148076	8.502345	1.86E-17	2.77E-16	376.2079	386.4727	318.645	805.7978	806.4447	968.8002
20133	LOC11962:	4.224932	1.258449	1.250071	1.006702	0.314078	0.41473	1.626259	4.379295	1.391463	2.232127	10.69555	5.024897
12393	LOC11962;	23.21762	1.25843	0.541865	2.322403	0.020211	0.043356	12.46799	14.78012	13.2189	44.64254	32.08666	22.10955
1193	GSN	14232.01	1.257489	0.123707	10.16509	2.84E-24	6.33E-23	9308.165	7248.28	8631.243	19794.5	17754.62	22655.25
14954	LOC10322:	17.56158	1.257213	0.681622	1.844442	0.065119	0.115763	7.589209	10.40082	12.52316	42.41041	21.39111	11.05477
12812	BMP7	25.27514	1.25704	0.56214	2.236168	0.025341	0.052582	16.80468	15.87494	11.1317	49.10679	40.6431	18.08963
16615	NLGN3	12.80506	1.256883	0.80788	1.555778	0.119761	0.191625	8.131296	3.831883	10.43597	11.16063	34.22577	9.044815
15838	LOC10322!	12.87204	1.256719	0.742527	1.692489	0.090553	0.151982	7.047123	4.379295	11.1317	20.08914	23.53022	11.05477
3698	LOC10324:	139.3512	1.256653	0.222702	5.642746	1.67E-08	1.20E-07	80.77087	79.37471	86.96642	200.8914	186.1026	202.0009
19210	LOC11962ᐤ	7.089638	1.256612	1.091068	1.151726	0.249434	0.345195	4.878777	0.547412	6.957314	11.16063	14.97377	4.019918
1822	CAMK2N1	1563.206	1.256226	0.149715	8.390779	4.83E-17	7.05E-16	780.0623	1148.47	837.6606	2200.877	2318.796	2093.372
23373	LOC10322(	1.507769	1.25616	2.500519	0.50236	0.615415	0.699874	0.542086	0	2.087194	0	6.417332	0
945	GSTK1	2998.957	1.255825	0.111816	11.23118	2.87E-29	8.06E-28	1878.871	1543.701	1890.998	4207.559	4038.641	4433.969
3847	GCHFR	219.3102	1.255823	0.228354	5.499455	3.81E-08	2.63E-07	139.8583	94.70224	155.1481	310.2657	280.2235	335.6631
15172	LOC10324!	14.22161	1.255811	0.696969	1.801819	0.071574	0.125415	9.215468	10.94824	4.87012	13.39276	27.80844	19.09461
880	TRIM35	1984.741	1.255696	0.108103	11.61578	3.43E-31	1.04E-29	1193.674	1144.638	1181.352	2685.249	2601.159	3102.372
1293	ZNF503	962.9743	1.255263	0.127049	9.880127	5.08E-23	1.04E-21	549.1335	631.1658	523.8857	1466.507	1285.606	1321.548
3619	SEMA6C	158.5351	1.254978	0.2194	5.720043	1.06E-08	7.82E-08	103.5385	84.30142	93.92374	218.7484	207.4937	243.205
1244	HAVCR1	4315.097	1.254673	0.125227	10.01917	1.26E-23	2.68E-22	2314.167	3054.011	2277.129	5892.815	6246.203	6106.255

20860	LOC11962:	3.971867	1.254347	1.398844	0.896703	0.369878	0.47139	3.794605	1.094824	2.087194	0	12.83466	4.019918
18270	LOC11962(	7.273121	1.254028	0.972345	1.289694	0.197157	0.2869	6.505036	3.284471	2.782925	8.928508	17.11289	5.024897
9015	CCDC178	52.22251	1.253615	0.402899	3.111484	0.001861	0.00549	39.5723	33.39212	19.48048	55.80317	87.70354	77.38342
21327	LOC11962(	3.267585	1.253589	1.513886	0.82806	0.407636	0.508134	4.336691	0.547412	0.695731	4.464254	8.556443	1.004979
23384	LRRC72	1.508656	1.253543	2.500272	0.501362	0.616116	0.700455	0	0.547412	2.087194	0	6.417332	0
1878	GLI4	1314.364	1.253469	0.152226	8.23427	1.81E-16	2.56E-15	887.9375	622.4072	823.0502	1756.684	1702.732	2093.372
20336	RASSF9	4.306633	1.253269	1.29031	0.971293	0.331402	0.433238	1.626259	1.642235	4.174388	6.696381	10.69555	1.004979
8250	FAM187A	49.8252	1.253235	0.376301	3.330409	0.000867	0.002794	22.22554	34.48694	31.30791	75.89232	72.72976	62.30873
15572	LOC11962‹	12.27268	1.253097	0.723063	1.73304	0.083088	0.141851	8.131296	7.116354	6.261582	13.39276	25.66933	13.06473
13628	RTN4RL1	18.03188	1.252621	0.60127	2.083292	0.037225	0.072616	11.38381	8.211177	12.52316	17.85702	32.08666	26.12947
17290	LOC11961‹	9.089098	1.252258	0.86339	1.450396	0.146948	0.225946	4.336691	2.737059	9.044508	13.39276	14.97377	10.04979
14579	LOC11962;	14.29514	1.252176	0.655618	1.909916	0.056144	0.102379	9.757555	9.306001	6.261582	20.08914	19.252	21.10457
13382	GIPR	20.10741	1.252091	0.587675	2.130584	0.033123	0.065804	15.17842	7.116354	13.2189	26.78552	34.22577	24.11951
4624	RAB3A	109.2025	1.252077	0.253824	4.932853	8.10E-07	4.66E-06	62.88202	71.16354	59.13717	147.3204	168.9897	145.722
11755	PDGFRL	27.18344	1.251424	0.509154	2.45785	0.013977	0.031611	20.59928	13.13788	14.61036	46.87467	25.66933	42.20914
3878	ZNF707	137.9562	1.251283	0.228755	5.469976	4.50E-08	3.09E-07	86.19173	72.25836	86.27069	189.7308	205.3546	187.9312
16409	AKAIN1	11.36799	1.251035	0.788301	1.587001	0.112512	0.182287	5.96295	5.474118	8.348776	17.85702	23.53022	7.034856
18072	LOC11962:	6.765229	1.250516	0.940964	1.328973	0.183857	0.270465	3.794605	5.474118	2.782925	8.928508	8.556443	11.05477
17858	LOC10322‹	7.513877	1.250493	0.914149	1.367931	0.171334	0.255048	4.336691	6.02153	2.782925	8.928508	14.97377	8.039836
3814	PLXNB3	196.4731	1.250122	0.226119	5.528591	3.23E-08	2.25E-07	128.4745	87.03848	134.2762	270.0874	269.5279	289.4341
19000	LOC11962!	5.798959	1.249981	1.055064	1.184744	0.236119	0.33038	3.252518	3.284471	3.478657	8.928508	12.83466	3.014938
15720	LOC11962;	17.74159	1.249875	0.7288	1.714976	0.08635	0.146031	6.505036	18.06459	6.261582	33.4819	32.08666	10.04979
14670	LOC10321!	13.84351	1.249674	0.659894	1.893749	0.058258	0.105576	9.215468	7.663765	7.653045	22.32127	17.11289	19.09461
15674	LSMEM1	14.27882	1.248985	0.725363	1.721876	0.085092	0.144317	4.336691	9.306001	11.82743	11.16063	29.94755	19.09461
15511	LOC10322:	18.93921	1.248303	0.716679	1.741788	0.081546	0.139765	7.047123	20.25424	6.261582	11.16063	42.78221	26.12947
2670	LOC10322:	253.8413	1.24803	0.181506	6.87596	6.16E-12	6.13E-11	138.7741	164.2235	149.5822	339.2833	342.2577	388.9271
23767	LOC11961!	1.040693	1.247835	2.818556	0.442721	0.657967	0.735981	1.084173	0	0.695731	4.464254	0	0
20590	TRNAD-GU	3.524732	1.247413	1.328628	0.938873	0.347796	0.449061	1.626259	3.831883	0.695731	6.696381	4.278221	4.019918
1971	CORO2A	416.0934	1.246878	0.154978	8.045508	8.59E-16	1.16E-14	258.5752	237.5767	246.2889	573.6566	526.2212	654.2416
809	DNAH5	2742.242	1.246755	0.103664	12.0269	2.57E-33	8.43E-32	1568.256	1771.425	1540.349	3778.991	3692.105	4102.326
2731	PLA2G15	871.2331	1.246701	0.183232	6.803959	1.02E-11	9.91E-11	608.2209	370.5978	573.2826	1167.402	1123.033	1384.862
20136	LPO	4.309827	1.24667	1.239214	1.006016	0.314408	0.415104	1.626259	1.094824	4.87012	6.696381	8.556443	3.014938
4066	ARSI	154.3275	1.246345	0.234219	5.321283	1.03E-07	6.74E-07	105.1648	74.99542	95.3152	216.5163	196.7982	237.1752
1453	LOC10323:	632.5845	1.246048	0.133146	9.358513	8.09E-21	1.48E-19	343.1407	401.8003	380.5651	850.4404	941.2087	878.3521
7914	LOC10323!	52.88637	1.245764	0.362742	3.434297	0.000594	0.001996	34.15144	29.01283	31.30791	84.82083	55.61688	82.40832
16840	LOC11962:	11.91749	1.245458	0.820516	1.517896	0.129041	0.203714	2.710432	5.474118	13.2189	22.32127	10.69555	17.08465
3356	VNN2	198.6095	1.244764	0.20795	5.985879	2.15E-09	1.70E-08	100.286	128.0944	125.2316	310.2657	252.4151	275.3644
22794	LOC11962‹	1.813896	1.244532	2.096286	0.593684	0.552724	0.64465	0	1.094824	2.087194	6.696381	0	1.004979
4024	ZNF703	146.9732	1.244416	0.232399	5.354645	8.57E-08	5.66E-07	86.73382	94.15483	81.40057	225.4448	168.9897	225.1154
599	HEXB	3044.271	1.244081	0.091436	13.60596	3.69E-42	1.64E-40	1867.488	1798.248	1758.809	4203.095	4147.736	4490.248
17872	LOC11962:	9.433673	1.24408	0.91101	1.365604	0.172063	0.255948	4.878777	2.189647	9.740239	22.32127	6.417332	11.05477
3312	CEBPA	501.0202	1.243816	0.20625	6.030636	1.63E-09	1.31E-08	369.1608	208.5639	316.5578	660.7096	648.1505	802.9786
19535	LOC10321!	5.016643	1.24331	1.135391	1.09505	0.273495	0.372196	4.336691	1.094824	3.478657	4.464254	10.69555	6.029877
9447	LOC10324;	42.5627	1.242881	0.414534	2.998261	0.002715	0.007641	23.8518	23.53871	27.82925	82.5887	51.33866	46.22906
15612	GABRG2	11.8321	1.242815	0.719418	1.727528	0.084073	0.143097	7.589209	7.663765	5.565851	17.85702	19.252	13.06473
14102	LIPC	16.88909	1.241805	0.622892	1.993612	0.046194	0.087085	7.047123	10.40082	12.52316	22.32127	29.94755	19.09461
22523	LOC11962:	1.813009	1.24134	1.949258	0.636827	0.524237	0.618783	0.542086	0.547412	2.087194	6.696381	0	1.004979
828	SESTD1	4662.548	1.241333	0.1044	11.89021	1.33E-32	4.27E-31	2663.27	3095.066	2558.204	6256.652	6586.322	6815.771
15677	ACER1	14.70495	1.241323	0.721066	1.72151	0.085158	0.144411	7.589209	7.663765	10.43597	24.5534	29.94755	8.039836
10747	NUPR2	34.84605	1.240075	0.462624	2.680523	0.007351	0.018184	22.22554	22.9913	16.00182	55.80317	59.8951	32.15934
3123	MFSD12	715.0905	1.239908	0.198357	6.250897	4.08E-10	3.47E-09	488.4198	292.8653	497.4479	935.2612	919.8176	1156.731
20066	LGI1	4.043983	1.239871	1.217787	1.018134	0.308614	0.408876	3.252518	1.094824	2.782925	6.696381	6.417332	4.019918
5497	UNC13A	130.9788	1.239495	0.280992	4.411146	1.03E-05	4.97E-05	65.05036	77.18507	90.44508	169.6417	243.8586	139.6921
1325	LOC10321(	1985.765	1.239304	0.126735	9.77867	1.39E-22	2.79E-21	1090.678	1381.667	1074.209	2687.481	2686.723	2993.834
24065	HBE1	0.771428	1.239039	3.138343	0.394807	0.692986	0.765584	0	0	1.391463	2.232127	0	1.004979
24066	PRL	0.771428	1.239039	3.138343	0.394807	0.692986	0.765584	0	0	1.391463	2.232127	0	1.004979
17978	GSC	8.140852	1.238683	0.919735	1.346782	0.17805	0.263292	4.336691	4.926706	4.87012	17.85702	12.83466	4.019918
22339	CARD11	3.073038	1.238673	1.853149	0.668415	0.503869	0.599613	0	0.547412	4.87012	4.464254	8.556443	0
24088	FTHL17	0.755925	1.238468	3.158644	0.392088	0.694993	0.767005	0	0	1.391463	0	2.139111	1.004979
24089	LOC10323:	0.755925	1.238468	3.158644	0.392088	0.694993	0.767005	0	0	1.391463	0	2.139111	1.004979
14903	LOC11962(	16.69345	1.238315	0.667846	1.854193	0.063712	0.113653	11.9259	10.94824	6.261582	29.01765	29.94755	12.05975
12370	LOC10324:	24.20242	1.238091	0.531831	2.327979	0.019913	0.042796	10.84173	18.06459	13.91463	33.4819	42.78221	26.12947
22564	LOC11961‹	2.298782	1.238053	1.963118	0.630656	0.528265	0.622404	0	0	4.174388	4.464254	2.139111	3.014938
20855	PRIMA1	3.522896	1.237985	1.379023	0.897726	0.369332	0.470807	1.626259	1.094824	3.478657	2.232127	10.69555	2.009959
23291	LOC11962‹	1.260977	1.237958	2.402656	0.515246	0.606381	0.692141	0	2.189647	0	2.232127	2.139111	1.004979
16894	LOC11962!	9.757264	1.237261	0.820847	1.507298	0.131734	0.207289	7.047123	5.474118	4.87012	6.696381	21.39111	13.06473
7365	LOC10322:	55.88592	1.236741	0.342471	3.611228	0.000305	0.0011	34.15144	33.39212	32.00364	71.42806	91.98176	72.35852
1703	MLLT11	3682.367	1.236703	0.141877	8.71671	2.86E-18	4.47E-17	1909.77	2750.744	1921.61	5205.32	4988.406	5318.351
1547	BCL6	1813.436	1.236502	0.135977	9.093447	9.59E-20	1.65E-18	984.4288	1302.84	955.2392	2464.268	2483.507	2690.33
19443	LOC11962:	4.757543	1.236411	1.113262	1.11062	0.266732	0.36471	3.794605	2.737059	2.087194	4.464254	6.417332	9.044815
21871	LOC11962;	3.111014	1.236023	1.64957	0.7493	0.453676	0.55146	0.542086	2.737059	2.087194	11.16063	2.139111	0
10230	SYT8	32.32528	1.235962	0.440159	2.807988	0.004985	0.012954	20.0572	17.51718	20.17621	51.33892	40.6431	44.2191
20098	NPIPB4	5.475186	1.235608	1.219847	1.01292	0.311098	0.411511	4.336691	3.284471	2.087194	0	17.11289	6.029877
17005	LOC11962‹	10.88209	1.235331	0.828301	1.491405	0.135855	0.212391	3.794605	3.831883	11.82743	13.39276	21.39111	11.05477

8875	KCNMB1	45.51686	1.235223	0.392219	3.149324	0.001636	0.004902	33.06727	27.918	20.17621	58.0353	70.59065	63.31371
16976	LOC11962	9.320245	1.235179	0.825525	1.496234	0.134593	0.210764	7.047123	3.284471	6.261582	11.16063	17.11289	11.05477
18432	LOC10324	7.557816	1.23516	0.980558	1.25965	0.207796	0.29971	3.794605	6.568942	2.782925	8.928508	19.252	4.019918
21714	LOC11962	2.740996	1.234777	1.591487	0.775864	0.437829	0.536031	1.626259	3.284471	0	2.232127	4.278221	5.024897
10761	OXER1	31.6251	1.2346	0.461128	2.677346	0.007421	0.018333	22.22554	19.15941	15.30609	33.4819	51.33866	48.23901
20595	LOC11962	4.232374	1.234565	1.31536	0.938576	0.347949	0.449149	2.710432	2.737059	2.087194	0	12.83466	5.024897
11602	LOC11962	33.43404	1.234154	0.49475	2.494499	0.012614	0.028903	18.43094	16.96977	23.65487	49.10679	66.31243	26.12947
3554	ITGB6	208.5345	1.23273	0.21248	5.80163	6.57E-09	4.91E-08	131.727	129.7366	112.7085	229.9091	331.5622	315.5636
1801	GUCY1A2	483.1909	1.232726	0.145957	8.445802	3.02E-17	4.46E-16	288.3899	281.3697	295.6858	607.1385	757.2452	669.3163
10952	LOC11962	38.68463	1.232636	0.467073	2.639064	0.008314	0.02018	18.97302	22.44388	27.13352	49.10679	81.28621	33.16432
2763	VDR	284.5438	1.232451	0.182493	6.753422	1.44E-11	1.39E-10	182.6831	176.2666	152.3652	372.7652	367.927	455.2557
13084	LOC10324	29.1854	1.23219	0.562516	2.190496	0.028488	0.057885	8.673382	20.80165	22.2634	44.64254	55.61688	23.11453
13428	LOC11961	20.269	1.232166	0.580799	2.121502	0.03388	0.067075	8.131296	15.32753	12.52316	35.71403	27.80844	22.10955
15066	RAB44	13.92625	1.231947	0.676111	1.822108	0.068439	0.120765	7.589209	9.306001	7.653045	24.5534	21.39111	13.06473
18319	LAPTM5	8.416672	1.23158	0.962577	1.279461	0.200735	0.291311	7.047123	2.737059	4.87012	17.85702	14.97377	3.014938
13887	LOC10323	19.91317	1.231566	0.605848	2.032798	0.042073	0.080544	8.673382	14.78012	11.82743	22.32127	42.78221	19.09461
15015	GALR3	18.80122	1.231528	0.672626	1.830926	0.067112	0.118831	13.01007	4.379295	16.00182	31.24978	32.08666	16.07967
2085	ABTB1	499.2303	1.2314	0.157444	7.821204	5.23E-15	6.67E-14	314.9522	258.3784	323.5151	714.2806	622.4812	761.7744
12426	LOC10323	33.00344	1.230721	0.53138	2.316085	0.020554	0.043974	11.9259	29.01283	17.39328	55.80317	57.75599	26.12947
1588	MMP14	656.2931	1.230632	0.136709	9.001813	2.22E-19	3.72E-18	363.74	441.2139	372.2163	877.2259	932.6523	950.7106
15483	LOC11961	12.3682	1.230601	0.70405	1.747889	0.080483	0.138193	6.505036	6.568942	9.044508	15.62489	21.39111	15.07469
1488	MPP6	813.7783	1.230495	0.132566	9.282145	1.66E-20	2.97E-19	462.3997	550.1489	447.3553	1084.814	1138.007	1199.945
16431	LOC11962	10.59613	1.230325	0.776719	1.584003	0.113193	0.183144	3.794605	7.116354	8.348776	13.39276	12.83466	18.08963
12665	TAT	22.7942	1.230085	0.542518	2.267361	0.023368	0.049052	16.80468	12.04306	11.82743	42.41041	23.53022	30.14938
19826	LOC11962	6.031821	1.229574	1.170471	1.050495	0.293491	0.393546	3.794605	3.284471	3.478657	2.232127	21.39111	2.009959
16830	LOC10322	12.68473	1.229432	0.809176	1.519363	0.128671	0.203251	4.878777	3.831883	13.91463	15.62489	27.80844	10.04979
22224	LOC11961	3.054707	1.229373	1.787253	0.687856	0.491543	0.587999	0	0	5.565851	4.464254	4.278221	4.019918
1000	JADE2	1500.758	1.228656	0.111846	10.9852	4.50E-28	1.20E-26	919.3785	896.1131	880.7959	2120.521	1897.391	2290.348
21875	LOC10322	2.821809	1.228643	1.641093	0.748673	0.454054	0.551819	2.168345	0	2.782925	6.696381	4.278221	1.004979
18061	HEPACAM	7.558348	1.228267	0.9222	1.331888	0.182897	0.269216	5.420864	4.379295	3.478657	8.928508	17.11289	6.029877
12497	LOC11962	40.77794	1.227963	0.533334	2.302427	0.021311	0.045336	18.97302	30.10765	22.95914	66.96381	85.56443	20.09959
19436	LOC10323	5.61511	1.227639	1.104041	1.11195	0.26616	0.364059	1.084173	2.737059	6.261582	11.16063	6.417332	6.029877
22260	LOC11962	3.148574	1.22739	1.800348	0.681752	0.495396	0.591649	2.168345	0.547412	2.782925	13.39276	0	0
23279	LOC11962	1.242812	1.22717	2.37418	0.516882	0.605239	0.691193	1.626259	0.547412	0	0	4.278221	1.004979
1149	PLXNB1	1187.199	1.227136	0.118909	10.31993	5.73E-25	1.32E-23	705.2544	694.1182	736.7795	1631.685	1510.212	1845.142
1419	LOC10322	674.0642	1.226315	0.12948	9.47111	2.77E-21	5.19E-20	416.8644	387.0202	409.7858	874.9938	932.6523	1023.069
14484	REPS2	24.13293	1.226256	0.636007	1.928053	0.053849	0.098838	14.63633	9.306001	18.78475	31.24978	57.75599	13.06473
1013	SLC17A5	1188.711	1.226171	0.112163	10.93207	8.10E-28	2.13E-26	738.8637	734.0792	664.4235	1593.739	1636.42	1764.744
18443	LOC10321	6.837449	1.225476	0.974219	1.257906	0.208426	0.300439	3.794605	2.737059	5.565851	8.928508	14.97377	5.024897
1912	CACNA1H	466.12	1.224857	0.149885	8.172003	3.03E-16	4.22E-15	300.8579	251.8094	285.2499	705.3521	603.2292	650.2217
17041	LOC10323	11.09036	1.224799	0.823528	1.487258	0.136947	0.213645	5.96295	8.758589	5.565851	17.85702	4.278221	24.11951
1052	TNFRSF10	1374.483	1.223643	0.113756	10.75676	5.51E-27	1.39E-25	887.3954	807.4324	779.9149	1901.772	1801.131	2069.253
2916	EFHB	378.1965	1.223159	0.187339	6.529104	6.62E-11	6.03E-10	189.7302	279.7274	210.1109	584.8173	496.2737	508.5196
5258	SPAAR	97.58341	1.22306	0.268889	4.548574	5.40E-06	2.73E-05	62.33993	49.81448	63.31155	133.9276	145.4595	130.6473
18428	LOC11962	6.875425	1.222522	0.969965	1.260377	0.207533	0.299396	4.336691	4.926706	2.782925	15.62489	8.556443	5.024897
4810	SNCG	127.536	1.221642	0.254128	4.807199	1.53E-06	8.46E-06	86.19173	60.76271	82.0963	178.5702	196.7982	160.7967
13399	LOC10323	26.97411	1.221374	0.574069	2.127575	0.033372	0.066214	24.39389	11.49565	11.82743	49.10679	44.92132	20.09959
10770	LOC11962	34.97826	1.221165	0.456524	2.674919	0.007475	0.018451	16.80468	22.9913	23.65487	31.24978	59.8951	55.27387
17869	LOC10323	8.152377	1.220891	0.893291	1.366734	0.171709	0.255463	4.878777	3.284471	6.261582	15.62489	12.83466	6.029877
17501	LOC10322	9.381105	1.220711	0.864077	1.412734	0.157734	0.23947	7.589209	2.189647	6.957314	13.39276	17.11289	9.044815
6155	LOC10322	87.00329	1.220513	0.298105	4.094244	4.24E-05	0.000183	48.24569	48.17224	59.8329	109.3742	151.8769	104.5179
8855	SLC6A16	49.74095	1.220167	0.386638	3.155842	0.0016	0.004805	37.40396	24.63353	27.13352	60.26743	87.70354	61.30375
10958	LOC11962	43.20574	1.220111	0.462632	2.637327	0.008356	0.020273	32.52518	15.32753	29.22072	66.96381	77.00798	38.18922
2141	KIAA0319	409.0621	1.218867	0.158238	7.702725	1.33E-14	1.65E-13	241.7705	278.6326	217.0682	553.5675	575.4208	587.913
9046	LOC11962	42.68909	1.218689	0.393335	3.098351	0.001946	0.005719	25.47806	29.56024	21.56767	55.80317	68.45154	55.27387
6329	CCDC146	114.1453	1.218671	0.303719	4.012492	6.01E-05	0.000252	68.84497	88.68071	47.30973	138.3919	198.9373	142.7071
9981	LOC10322	61.86856	1.218168	0.425042	2.865996	0.004157	0.011072	28.73058	44.88777	36.87376	71.42806	139.0422	50.24897
19356	LOC11962	6.121214	1.217772	1.081367	1.126142	0.260106	0.357249	2.168345	4.379295	4.174388	11.16063	12.83466	2.009959
15539	CYSRT1	14.73564	1.217237	0.700457	1.737775	0.08225	0.14071	5.96295	6.568942	13.91463	20.08914	27.80844	14.06971
22699	LOC10324	2.053931	1.217193	2.000549	0.608429	0.542903	0.635821	0	2.189647	1.391463	4.464254	4.278221	0
2163	PAPLN	759.8038	1.216827	0.158728	7.66611	1.77E-14	2.18E-13	518.7767	370.0504	484.9248	1044.635	962.5998	1177.836
8044	LOC11962	51.90109	1.216498	0.35766	3.401272	0.000671	0.002217	26.02015	33.93953	34.09084	73.66019	66.31243	77.38342
20769	LOC10323	4.372463	1.216033	1.338334	0.908617	0.363553	0.465359	1.084173	1.094824	5.565851	8.928508	8.556443	1.004979
10206	FPR1	38.64666	1.2153	0.431629	2.815615	0.004868	0.012682	28.18849	16.42235	25.74206	46.87467	51.33866	63.31371
18621	LOC11962	6.357533	1.215262	0.984298	1.234649	0.216961	0.309867	2.710432	4.379295	4.174388	11.16063	10.69555	5.024897
1365	FAM228B	2032.595	1.215247	0.125703	9.667571	4.14E-22	8.06E-21	1102.062	1441.883	1127.781	2794.623	2793.679	2935.545
8861	IFI27L2	2594.766	1.215076	0.385199	3.154413	0.001608	0.004825	1839.299	825.497	2022.491	3821.401	3610.819	3449.09
22617	LOC10322	2.069966	1.214873	1.952571	0.622191	0.533816	0.62747	1.626259	0	2.087194	6.696381	0	2.009959
20467	LOC11962	4.320158	1.214599	1.276792	0.95129	0.341457	0.443526	3.794605	1.642235	2.087194	6.696381	10.69555	1.004979
19024	LOC10322	6.661825	1.213688	1.028296	1.180291	0.237885	0.332431	2.168345	2.737059	6.957314	13.39276	10.69555	4.019918
6393	LOC11962	143.1297	1.213556	0.304799	3.981498	6.85E-05	0.000285	101.9122	54.19377	101.5768	205.3557	245.9977	149.7419
22025	LOC11962	2.268002	1.213391	1.680823	0.721903	0.470354	0.567735	2.710432	0.547412	0.695731	2.232127	6.417332	1.004979
18598	PTPRQ	6.830692	1.213306	0.981803	1.235794	0.216535	0.309497	3.252518	6.02153	2.782925	8.928508	14.97377	5.024897
5179	LOC11962	97.35468	1.2129	0.264262	4.58976	4.44E-06	2.28E-05	59.6295	55.83601	61.22436	120.5349	141.1813	145.722

3388	TLR5	331.5874	1.212707	0.203676	5.954101	2.62E-09	2.05E-08	170.7572	245.2405	184.3688	372.7652	521.943	494.4499
890	MANEA	4476.961	1.212706	0.105241	11.52311	1.01E-30	3.01E-29	2544.553	3037.588	2514.373	6022.279	6372.411	6370.565
2010	LRP3	1588.06	1.212303	0.152354	7.957169	1.76E-15	2.33E-14	1062.489	746.6697	1065.86	2256.68	1978.677	2417.981
10163	LOC119622	39.24466	1.211647	0.429216	2.822932	0.004759	0.012448	26.56223	26.27577	17.39328	53.57105	68.45154	43.21412
20151	SHISA3	4.593279	1.211381	1.206924	1.003693	0.315527	0.416271	1.626259	1.094824	5.565851	6.696381	8.556443	4.019918
19716	TRNAG-UC	4.558252	1.210947	1.134863	1.067042	0.285953	0.385578	3.252518	2.737059	2.087194	6.696381	8.556443	4.019918
1338	CARD6	935.8816	1.210299	0.124152	9.748526	1.87E-22	3.72E-21	542.6285	627.8814	523.19	1361.597	1253.519	1306.473
20422	LOC119627	4.322821	1.210295	1.26287	0.958368	0.337877	0.439842	2.168345	3.284471	2.087194	6.696381	10.69555	1.004979
10559	TMEM207	37.57437	1.210284	0.443122	2.731266	0.006309	0.015885	26.56223	15.32753	25.74206	53.57105	62.03421	42.20914
20351	FNDC7	3.804634	1.209803	1.248578	0.968944	0.332573	0.434448	3.794605	1.642235	1.391463	6.696381	4.278221	5.024897
1432	FMNL1	845.1964	1.209373	0.128226	9.43157	4.04E-21	7.50E-20	519.3187	511.2826	502.318	1241.063	1028.912	1268.284
13773	CUNH15or	23.50425	1.209301	0.589267	2.052213	0.040149	0.077497	10.84173	10.40082	20.87194	40.17829	40.6431	18.08963
1603	PLIN2	844.2099	1.208932	0.134749	8.971727	2.92E-19	4.84E-18	546.9651	459.8259	525.9729	1140.617	1082.39	1309.488
1349	ZBTB38	18778.98	1.20871	0.124338	9.721186	2.45E-22	4.83E-21	10098.53	13696.24	10232.82	25415	25825.48	27405.79
8719	LOC103215	47.6637	1.208604	0.377949	3.197794	0.001385	0.004222	24.93597	34.48694	26.43779	66.96381	74.86887	58.28881
23610	LOC119623	1.02519	1.208505	2.585437	0.467428	0.640194	0.720943	1.084173	0	0.695731	2.232127	2.139111	0
826	ATP6V0A1	3410.248	1.208234	0.101529	11.90039	1.18E-32	3.79E-31	2014.393	2217.018	1952.222	4624.967	4528.497	5124.39
22257	LOC103215	2.254275	1.208078	1.77074	0.682245	0.495084	0.591356	1.626259	1.642235	0.695731	0	8.556443	1.004979
8962	LOC119626	43.62376	1.208031	0.386144	3.128447	0.001757	0.005213	24.93597	22.9913	31.30791	58.0353	64.17332	60.29877
791	GFUS	2874.873	1.207848	0.099437	12.14685	5.96E-34	2.00E-32	1739.013	1699.166	1776.202	4138.363	3651.462	4245.033
13426	ODAD1	26.17899	1.207833	0.569245	2.121817	0.033853	0.067033	13.01007	14.23271	19.48048	58.0353	34.22577	18.08963
847	PLEKHA6	2602.026	1.207733	0.102433	11.7905	4.37E-32	1.37E-30	1506.458	1690.408	1522.956	3426.315	3595.845	3870.176
14590	LOC103224	21.86068	1.207541	0.632638	1.90874	0.056296	0.102578	19.51511	7.663765	11.82743	29.01765	47.06043	16.07967
20247	LOC119619	5.069327	1.207219	1.221723	0.988128	0.32309	0.424228	3.252518	4.926706	0.695731	6.696381	12.83466	2.009959
23398	LOC119625	1.569577	1.207024	2.418235	0.499134	0.617685	0.701799	1.626259	1.094824	0	6.696381	0	0
19562	LOC103230	5.34943	1.206877	1.107848	1.089389	0.275982	0.375025	4.336691	1.642235	3.478657	8.928508	10.69555	3.014938
22387	LOC119627	2.051268	1.206676	1.827223	0.660388	0.509005	0.604442	1.626259	0.547412	1.391463	4.464254	4.278221	0
1677	MCC	567.2293	1.206671	0.137359	8.784799	1.57E-18	2.48E-17	340.4302	363.4814	326.9937	745.5304	763.6625	863.2774
22242	CRNN	3.031725	1.206506	1.760936	0.68515	0.493249	0.589556	1.626259	1.642235	2.087194	0	12.83466	0
2793	LRRC14	1080.47	1.205905	0.179751	6.708753	1.96E-11	1.87E-10	770.8468	481.175	710.3417	1591.507	1255.658	1673.291
659	PCDH9	13404.2	1.205473	0.092237	13.06936	4.93E-39	1.99E-37	7792.492	8885.041	7648.871	18379.33	18635.93	19083.56
2542	SLC2A11	906.6065	1.205381	0.170643	7.063771	1.62E-12	1.69E-11	685.1972	437.382	524.5815	1145.081	1236.406	1410.991
27971	LOC119628	0.515689	1.20519	4.024073	0.299495	0.764562	NA	1.084173	0	0	0	0	2.009959
29398	LOC103247	0.515689	1.20519	4.024073	0.299495	0.764562	NA	1.084173	0	0	0	0	2.009959
14097	LOC103221	17.54032	1.204258	0.603725	1.994714	0.046074	0.086889	7.589209	10.40082	13.91463	24.5534	25.66933	23.11453
28024	LOC119618	0.516576	1.20399	3.574844	0.336795	0.736271	NA	0.542086	0.547412	0	0	0	2.009959
3070	NKIRAS1	198.5172	1.20366	0.190718	6.311195	2.77E-10	2.40E-09	118.7169	124.8099	117.5786	265.6231	275.9453	288.4291
3568	TAOK3	430.9867	1.203515	0.208015	5.785711	7.22E-09	5.38E-08	222.2554	354.7229	205.2408	604.9064	596.8119	601.9827
996	GREM1	24678.46	1.202502	0.109309	11.00094	3.78E-28	1.01E-26	13832.96	17324.49	13694.08	33821.19	33842.87	35555.17
7260	VAMP1	67.32855	1.202381	0.329433	3.649847	0.000262	0.000961	31.9831	48.17224	41.74388	95.98146	102.6773	83.4133
10501	LOC103215	38.07884	1.202048	0.437992	2.74445	0.006061	0.015345	24.93597	24.63353	18.78475	51.33892	70.59065	38.18922
15278	F2RL2	15.06581	1.201909	0.673856	1.78363	0.074484	0.129608	5.96295	12.04306	9.044508	29.01765	19.252	15.07469
3335	KIF26A	217.4096	1.20187	0.200045	6.007989	1.88E-09	1.50E-08	144.195	111.1246	140.5377	316.962	278.0844	313.5536
23514	DNAJB7	1.548879	1.201543	2.497033	0.481188	0.630383	0.712713	0	0	2.782925	2.232127	4.278221	0
5397	LOC103243	104.3169	1.201539	0.269144	4.4643	8.03E-06	3.96E-05	61.25576	73.90059	53.57132	145.0883	160.4333	131.6523
15936	LOC119626	13.22234	1.201163	0.715726	1.678243	0.0933	0.155646	9.757555	4.379295	9.740239	17.85702	23.53022	14.06971
6922	LOC119626	77.26518	1.201126	0.318998	3.765306	0.000166	0.000639	54.75072	50.36189	34.78657	95.98146	126.2075	101.5029
870	ABHD2	8616.113	1.200441	0.102714	11.6872	1.48E-31	4.53E-30	5205.113	5367.373	5107.364	11774.47	10806.79	13435.57
14357	SPATA32	17.5364	1.20018	0.61531	1.950531	0.051113	0.09464	9.757555	9.306001	12.52316	22.32127	34.22577	17.08465
14899	LOC103220	18.28219	1.198254	0.645928	1.855087	0.063584	0.113456	13.01007	9.306001	10.43597	20.08914	42.78221	14.06971
4926	LOC103241	153.1053	1.197423	0.253193	4.729281	2.25E-06	1.22E-05	79.14461	121.5254	78.61764	196.4272	211.772	231.1453
505	LOC103241	5491.199	1.196888	0.082386	14.52782	8.08E-48	4.25E-46	3262.818	3447.052	3297.767	7629.41	7561.756	7748.392
15128	EPO	20.48631	1.195926	0.660944	1.809422	0.070386	0.123689	10.84173	9.853413	16.00182	31.24978	44.92132	10.04979
19317	OSCAR	5.900207	1.19555	1.055924	1.13223	0.257538	0.354432	4.878777	1.642235	4.174388	13.39276	4.278221	7.034856
3410	LOC103233	206.6697	1.195108	0.2013	5.936942	2.90E-09	2.26E-08	132.8112	108.935	136.3633	292.4086	260.9715	308.5287
23575	LOC103218	1.029273	1.194634	2.525945	0.472945	0.636252	0.717487	0.542086	0	1.391463	2.232127	0	2.009959
15151	GPR4	24.83323	1.194145	0.661304	1.805744	0.070958	0.124508	15.17842	9.306001	20.17621	31.24978	62.03421	11.05477
7875	LOC119620	65.60079	1.193876	0.346339	3.447129	0.000567	0.001913	49.87195	28.46541	41.74388	93.74933	81.28621	98.48799
23920	LOC119622	1.011463	1.193715	2.843255	0.419841	0.674602	0.749761	0	1.094824	0.695731	0	4.278221	0
5199	LOC119624	133.4663	1.193703	0.260613	4.58037	4.64E-06	2.37E-05	83.4813	60.2153	100.1853	194.195	181.8244	180.8963
1680	BACE1	1560.961	1.193447	0.135981	8.776537	1.69E-18	2.67E-17	887.3954	1121.099	842.5307	2102.664	2034.294	2377.781
724	RHOB	4485.553	1.193368	0.09444	12.63621	1.33E-36	4.90E-35	2638.876	2833.951	2719.614	6073.618	5918.919	6728.338
1068	FHOD3	3147.572	1.193097	0.111952	10.65726	1.61E-26	4.01E-25	1797.016	2166.109	1785.247	4241.041	4226.883	4669.135
4298	LOC119627	130.9654	1.192636	0.231258	5.157174	2.51E-07	1.55E-06	85.10756	75.54283	77.92191	178.5702	196.7982	171.8515
21575	LOC119622	2.543124	1.192479	1.504137	0.792799	0.427895	0.527258	2.168345	1.642235	0.695731	4.464254	4.278221	2.009959
863	PREPL	23978.69	1.192455	0.10181	11.71258	1.10E-31	3.39E-30	13604.74	16550.45	13636.33	32685.04	33218.25	34177.34
1387	OLFML3	2240.933	1.192283	0.12431	9.591221	8.70E-22	1.67E-20	1220.236	1603.369	1269.71	3098.192	3031.12	3222.969
20042	TOMM20L	5.123809	1.191749	1.166762	1.021415	0.307058	0.407281	5.96295	1.094824	2.087194	11.16063	6.417332	4.019918
19179	LOC103241	5.600824	1.191382	1.029536	1.157203	0.247189	0.342721	3.252518	2.189647	4.87012	6.696381	8.556443	8.039836
23580	SERPINA7	1.03016	1.191276	2.525486	0.471702	0.63714	0.718336	0	0.547412	1.391463	2.232127	0	2.009959
21016	LOC119627	3.354775	1.19075	1.356686	0.87769	0.380112	0.480804	2.168345	1.094824	2.782925	8.928508	2.139111	3.014938
21233	MYADML2	3.070895	1.190695	1.40726	0.846109	0.397492	0.497684	0.542086	1.642235	3.478657	4.464254	4.278221	4.019918
18193	FOXB1	8.602266	1.190611	0.913971	1.302679	0.192685	0.28155	2.710432	5.474118	7.653045	4.464254	19.252	12.05975
18194	GJB6	8.602266	1.190611	0.913971	1.302679	0.192685	0.28155	2.710432	5.474118	7.653045	4.464254	19.252	12.05975

7215	LOC11962(	75.24819	1.190383	0.32505	3.662153	0.00025	0.000922	39.5723	60.76271	36.87376	102.6778	109.0946	102.5079
877	KIAA0930	4321.76	1.190076	0.102233	11.64083	2.56E-31	7.75E-30	2734.826	2463.353	2707.091	6131.653	5454.732	6438.903
23354	LOC10323;	1.298537	1.188268	2.350479	0.505543	0.613178	0.69801	1.626259	0	0.695731	4.464254	0	1.004979
1206	SPIDR	1577.336	1.188151	0.117283	10.13066	4.04E-24	8.90E-23	896.0688	1092.634	897.4935	2145.074	2226.814	2205.93
22069	SRGN	2.039848	1.188082	1.664737	0.713676	0.475428	0.572661	1.084173	0.547412	2.087194	2.232127	4.278221	2.009959
22070	LOC11962(	2.039848	1.188082	1.664737	0.713676	0.475428	0.572661	1.084173	0.547412	2.087194	2.232127	4.278221	2.009959
22971	ELOA2	1.769695	1.188059	2.089293	0.568641	0.5696	0.659214	0.542086	0	2.782925	0	4.278221	3.014938
23413	LOC10322;	1.026078	1.188045	2.390243	0.497039	0.619161	0.703056	0.542086	0.547412	0.695731	2.232127	2.139111	0
20341	LOC11962;	4.080583	1.187935	1.223685	0.970785	0.331655	0.433462	1.626259	1.642235	4.174388	4.464254	8.556443	4.019918
702	RGS3	5611.244	1.187784	0.092631	12.82282	1.22E-37	4.63E-36	3424.902	3409.281	3441.087	7723.159	7247.307	8421.728
5476	LOC1032<	91.66646	1.187072	0.268319	4.424109	9.68E-06	4.70E-05	53.66655	57.47824	56.35424	124.9991	136.9031	120.5975
16832	LOC10323:	10.24307	1.186121	0.780775	1.519158	0.128723	0.203309	4.336691	6.02153	8.348776	17.85702	12.83466	12.05975
16467	LOC11962<	12.97009	1.185804	0.751261	1.578419	0.114469	0.184803	7.589209	4.926706	11.1317	11.16063	29.94755	13.06473
1304	LOC10323:	7023.885	1.185559	0.120544	9.835091	7.95E-23	1.62E-21	4734.582	3788.09	4351.8	9555.736	8712.598	11000.51
11038	ZNF423	33.3312	1.185329	0.452273	2.620828	0.008772	0.021127	21.14137	24.08612	15.30609	44.64254	55.61688	39.1942
1625	FGF13	1239.79	1.185217	0.133028	8.909532	5.12E-19	8.38E-18	679.2342	888.4494	704.7759	1754.452	1630.002	1781.829
21431	LOC11962!	4.014195	1.185125	1.456136	0.813883	0.415712	0.515687	5.420864	0.547412	1.391463	0	10.69555	6.029877
22145	LOC11962:	2.330473	1.185034	1.691428	0.700612	0.483545	0.580494	0.542086	1.642235	2.087194	6.696381	0	3.014938
9608	RASIP1	45.58748	1.185021	0.40045	2.959226	0.003084	0.008534	22.76763	25.72836	34.78657	80.35657	55.61688	54.26889
20311	LOC1032<	3.831129	1.184501	1.212424	0.976969	0.328584	0.430083	2.710432	2.189647	2.087194	6.696381	4.278221	5.024897
5282	ACYP2	132.8058	1.184474	0.26148	4.529892	5.90E-06	2.97E-05	81.31296	62.95236	100.1853	158.481	198.9373	194.966
14795	LOC1032<	17.16833	1.18401	0.631714	1.874283	0.060891	0.109415	12.46799	10.40082	8.348776	35.71403	14.97377	21.10457
12245	LRRC46	28.25289	1.183416	0.501956	2.357608	0.018393	0.039935	13.55216	19.15941	18.78475	31.24978	55.61688	31.15436
2107	PPFIA4	498.5565	1.182971	0.15193	7.786296	6.90E-15	8.71E-14	294.895	280.2748	342.2998	680.7987	635.3159	757.7545
1590	PARP10	1050.588	1.18295	0.131445	8.999585	2.27E-19	3.79E-18	709.049	566.5712	654.6832	1404.008	1373.309	1595.907
4647	MZF1	143.3436	1.182775	0.240489	4.918215	8.73E-07	5.00E-06	91.07051	71.71095	100.881	176.338	216.0502	204.0108
825	KYNU	3193.812	1.182564	0.099338	11.9044	1.12E-32	3.62E-31	1898.929	2140.38	1820.033	4424.076	4468.602	4410.855
9392	PDZD3	57.39646	1.182114	0.39286	3.008998	0.002621	0.007419	37.94605	24.08612	42.43961	91.51721	94.12087	54.26889
17469	SLCO2B1	11.28729	1.181757	0.834421	1.41626	0.156699	0.238476	8.673382	3.284471	8.348776	17.85702	23.53022	6.029877
20600	LOC11962;	3.829709	1.181639	1.26115	0.936953	0.348783	0.450116	1.626259	3.831883	1.391463	6.696381	6.417332	3.014938
2712	EGFL7	1022.669	1.180508	0.173066	6.821137	9.03E-12	8.85E-11	741.0321	476.7957	661.6405	1562.489	1193.624	1500.434
1981	CNGA3	383.1121	1.179425	0.146851	8.03146	9.63E-16	1.29E-14	227.1342	235.9345	240.7231	524.5498	549.7514	520.5794
20895	TREM2	3.32874	1.178831	1.323089	0.890969	0.372946	0.474482	1.084173	1.642235	3.478657	4.464254	4.278221	5.024897
22408	LOC10322:	2.024345	1.178816	1.799119	0.655218	0.512327	0.607828	1.084173	0.547412	2.087194	0	6.417332	2.009959
10024	INCA1	38.12151	1.178624	0.412737	2.855626	0.004295	0.011391	22.76763	23.53871	23.65487	64.73168	42.78221	51.25395
1808	PTGR1	4020.479	1.177275	0.139633	8.431237	3.42E-17	5.03E-16	2140.699	3072.623	2181.118	5649.513	5726.399	5352.521
17836	LOC11961{	8.422431	1.177245	0.858573	1.371165	0.170324	0.253857	6.505036	3.284471	5.565851	11.16063	14.97377	9.044815
2886	VWA1	1041.191	1.176984	0.179258	6.565851	5.17E-11	4.77E-10	753.5001	472.9638	692.2527	1350.437	1285.606	1692.385
12583	DLEC1	27.23699	1.176971	0.514598	2.287167	0.022186	0.046874	17.88885	14.78012	16.69755	53.57105	36.36488	24.11951
21160	TRNAR-UC	3.280517	1.175987	1.372784	0.856644	0.391642	0.492028	3.252518	2.189647	0.695731	2.232127	4.278221	7.034856
16743	PARP15	13.96783	1.175595	0.766156	1.534408	0.124929	0.198366	8.131296	6.568942	10.43597	31.24978	21.39111	6.029877
1087	NOCT	1113.263	1.175573	0.111409	10.55187	4.98E-26	1.22E-24	702.5439	692.4759	656.7704	1526.775	1458.873	1642.136
1679	RAB6B	657.7113	1.175301	0.133902	8.777308	1.67E-18	2.65E-17	405.4806	385.9253	422.3089	812.4942	941.2087	978.85
8903	ODAD3	47.04477	1.17479	0.373639	3.144181	0.001666	0.004973	24.39389	31.74989	30.61218	71.42806	57.75599	66.32864
2954	MST1	261.6695	1.174529	0.181117	6.48492	8.88E-11	7.99E-10	164.2522	154.3701	164.8883	377.2295	312.3102	396.9669
20214	ZYG11A	4.604497	1.174448	1.181116	0.994354	0.32005	0.420923	3.252518	2.189647	2.782925	6.696381	10.69555	2.009959
22034	LOC11962:	2.518508	1.174021	1.6324	0.7192	0.472018	0.569511	2.168345	0.547412	2.087194	0	4.278221	6.029877
16084	LOC11962<	16.82672	1.174005	0.711722	1.649527	0.09904	0.163701	18.43094	6.568942	5.565851	20.08914	34.22577	16.07967
22399	LOC10321;	2.29716	1.173319	1.785506	0.657135	0.511094	0.606609	0	2.737059	1.391463	2.232127	6.417332	1.004979
1397	CLTRN	881.2134	1.173298	0.123088	9.532175	1.54E-21	2.93E-20	504.6824	568.2135	549.6278	1191.956	1324.11	1148.692
17252	LOC10321!	10.49913	1.173049	0.804862	1.457454	0.144991	0.223429	3.252518	9.853413	6.261582	17.85702	10.69555	15.07469
12811	LOC1032<	24.28929	1.172441	0.524288	2.236256	0.025335	0.052574	18.97302	11.49565	13.91463	35.71403	38.50399	27.13445
4411	TLR6	185.794	1.172439	0.231168	5.071809	3.94E-07	2.37E-06	101.3702	146.159	94.61947	252.2303	263.1106	257.2747
17268	LOC1032<	11.73939	1.172266	0.805041	1.456158	0.145349	0.223783	9.215468	2.189647	10.43597	13.39276	17.11289	18.08963
21494	LOC11962:	2.786354	1.171234	1.455959	0.804442	0.421142	0.520892	2.168345	2.189647	0.695731	2.232127	6.417332	3.014938
995	DPYSL5	2019.324	1.171079	0.106444	11.0018	3.75E-28	1.00E-26	1277.698	1229.487	1223.096	2685.249	2616.132	3084.282
2645	SLC11A1	264.4971	1.1709	0.169562	6.905443	5.00E-12	5.03E-11	161.5417	165.3184	161.4097	345.9797	382.9008	369.8324
23652	LOC11961{	1.014657	1.170746	2.536558	0.461549	0.644405	0.724254	0	0.547412	1.391463	0	2.139111	2.009959
23654	LOC11961!	1.014657	1.170746	2.536558	0.461549	0.644405	0.724254	0	0.547412	1.391463	0	2.139111	2.009959
16451	LOC10322<	18.19381	1.170695	0.74115	1.579566	0.114206	0.184559	9.757555	12.04306	11.1317	17.85702	51.33866	7.034856
11742	C1QL4	28.69002	1.170155	0.475672	2.460004	0.013894	0.031456	14.63633	20.25424	18.08902	46.87467	32.08666	40.19918
1128	ABCA1	1367.104	1.169106	0.112371	10.40394	2.38E-25	5.61E-24	806.0824	896.6606	824.4417	1912.933	1745.514	2016.994
2552	GDPD5	717.8313	1.169023	0.165971	7.043524	1.87E-12	1.95E-11	466.1943	385.3779	477.9675	993.2965	804.3056	1179.846
15932	LOC11962!	12.54695	1.168742	0.696331	1.678428	0.093264	0.155595	7.047123	6.568942	9.740239	17.85702	14.97377	19.09461
19104	LOC11962<	6.95827	1.168726	0.999489	1.169324	0.242273	0.337146	2.710432	4.926706	4.87012	13.39276	12.83466	3.014938
20580	LOC11962:	4.548404	1.168601	1.241803	0.941052	0.346678	0.447847	2.710432	4.926706	0.695731	2.232127	10.69555	6.029877
20063	GAD2	5.679318	1.168488	1.146907	1.018816	0.30829	0.408508	0.542086	4.926706	4.87012	11.16063	8.556443	4.019918
17237	LOC10322:	15.56807	1.168381	0.799355	1.461655	0.143836	0.221841	4.336691	10.94824	13.2189	8.928508	44.92132	11.05477
802	CAT	4878.926	1.167579	0.096718	12.07195	1.49E-33	4.92E-32	2872.516	3249.984	2897.025	6756.648	6400.219	7097.165
21424	GLYATL3	3.107923	1.167169	1.433006	0.814491	0.415364	0.515424	0.542086	1.642235	3.478657	6.696381	4.278221	2.009959
11972	LOC1032<	32.41795	1.166901	0.48388	2.411548	0.015885	0.035274	24.39389	12.04306	23.65487	35.71403	53.47777	45.22408
1197	HIP1	5320.233	1.166664	0.11488	10.15551	3.13E-24	6.96E-23	3004.243	3761.814	3073.741	7044.593	7082.595	7954.412
15952	MILR1	14.41976	1.166626	0.696126	1.675884	0.093761	0.156259	4.878777	10.40082	11.1317	26.78552	19.252	14.06971
8052	LOC11962;	58.46383	1.166559	0.343268	3.398388	0.000678	0.002238	31.9831	32.2973	43.83108	89.28508	77.00798	76.37844

1479	SQSTM1	33539.41	1.166333	0.125376	9.302667	1.37E-20	2.46E-19	22928.09	17499.66	21602.46	45113.52	41588.59	52504.15
8535	ADIRF	60.31917	1.165883	0.359291	3.244952	0.001175	0.003659	34.69353	37.224	38.96096	69.19594	115.512	66.32864
4185	ZNF696	170.7525	1.16582	0.222644	5.236244	1.64E-07	1.04E-06	119.259	85.39624	111.317	247.7661	224.6066	236.1702
5056	LOC10324:	110.1877	1.165534	0.250254	4.657407	3.20E-06	1.68E-05	67.7608	65.68942	69.57314	167.4095	154.016	136.6772
2509	LOC10322!	427.1768	1.165524	0.163748	7.11778	1.10E-12	1.16E-11	275.922	221.1544	294.9901	575.8888	569.0034	626.1022
1163	SRC	1410.878	1.165375	0.113268	10.28866	7.93E-25	1.81E-23	861.9173	890.0916	862.7069	1830.344	1852.47	2167.741
5019	LOC11961!	112.8327	1.164589	0.248945	4.678096	2.90E-06	1.53E-05	68.30288	77.18507	63.31155	162.9453	145.4595	159.7917
15179	ANGPTL6	18.86551	1.164313	0.646708	1.80037	0.071802	0.125757	8.673382	11.49565	14.61036	13.39276	44.92132	20.09959
18787	GAS2	11.56564	1.164099	0.954549	1.219527	0.222644	0.315047	4.878777	4.379295	11.82743	8.928508	36.36488	3.014938
11802	GBA	38.43525	1.164086	0.475388	2.448705	0.014337	0.032295	33.60935	17.51718	20.87194	46.87467	36.36488	75.37346
12978	LOC10323:	33.71401	1.163823	0.527204	2.207539	0.027276	0.055875	12.46799	32.2973	17.39328	66.96381	29.94755	43.21412
20296	LOC10321:	5.170184	1.163318	1.189138	0.978286	0.327933	0.42947	1.084173	2.737059	5.565851	8.928508	10.69555	2.009959
1833	MYORG	525.3557	1.162767	0.139033	8.363245	6.10E-17	8.85E-16	316.0364	342.1324	317.2535	683.0309	703.7674	789.9139
9944	PCED1B	52.14886	1.162157	0.404054	2.876245	0.004024	0.010757	27.10432	28.46541	40.35242	102.6778	62.03421	52.25893
2725	ARVCF	764.1825	1.162022	0.170606	6.811149	9.68E-12	9.45E-11	541.5443	361.8392	514.8412	1069.189	936.9305	1160.751
21019	FSCN3	3.834489	1.161862	1.324144	0.877444	0.380246	0.480938	4.336691	0.547412	2.087194	4.464254	8.556443	3.014938
11988	SEPTIN14	42.77053	1.161414	0.482168	2.408732	0.016008	0.0355	28.73058	26.27577	22.95914	66.96381	85.56443	26.12947
11545	LOC10324:	31.00377	1.161262	0.46363	2.504719	0.012255	0.02822	14.63633	19.15941	23.65487	40.17829	49.19955	39.1942
1781	DAAM1	752.0711	1.16125	0.136541	8.504781	1.82E-17	2.72E-16	430.9587	527.705	436.9193	1020.082	988.2691	1108.492
904	TRPM3	1494.6	1.160199	0.101312	11.45172	2.31E-30	6.78E-29	905.2842	967.8241	898.8849	2002.218	2124.137	2069.253
21460	LOC11962!	3.092421	1.160169	1.433924	0.809087	0.418465	0.518401	0.542086	1.642235	3.478657	4.464254	6.417332	2.009959
1237	VCPIP1	2546.775	1.160042	0.11561	10.03407	1.08E-23	2.32E-22	1468.512	1811.386	1444.338	3435.243	3510.281	3610.891
11309	AIM2	53.04021	1.159055	0.453381	2.556469	0.010574	0.024857	24.93597	30.10765	42.43961	69.19594	113.3729	38.18922
1188	SBNO2	2826.153	1.158484	0.113655	10.19298	2.13E-24	4.77E-23	1796.474	1629.645	1825.599	3716.491	3580.871	4407.84
707	B4GALT5	7666.889	1.157469	0.090665	12.76639	2.53E-37	9.50E-36	4878.777	4764.672	4599.48	10428.5	9957.56	11372.35
821	TUSC3	3038.27	1.15728	0.097064	11.9228	9.00E-33	2.92E-31	1993.252	1741.317	1909.783	4049.078	4276.082	4260.108
2374	CGNL1	1432.736	1.156558	0.158297	7.306253	2.75E-13	3.08E-12	766.5101	1122.742	773.6533	1799.094	2012.903	2121.512
16471	LOC10322:	13.2647	1.156395	0.732915	1.577804	0.114611	0.184987	7.589209	5.474118	11.82743	8.928508	25.66933	20.09959
17299	CUNH22or	9.967319	1.156185	0.798241	1.448416	0.147501	0.226678	4.336691	6.568942	7.653045	8.928508	19.252	13.06473
13583	CDK5R2	22.95375	1.156137	0.552592	2.092207	0.03642	0.071282	10.84173	18.06459	13.91463	20.08914	40.6431	34.1693
15571	CYP11A1	14.68007	1.156082	0.666984	1.733298	0.083043	0.141791	7.589209	8.211177	11.1317	24.5534	23.53022	13.06473
23197	LOC11961!	1.285697	1.155901	2.181586	0.529844	0.59622	0.6833	0	1.642235	0.695731	2.232127	2.139111	1.004979
16651	CUNH1orf:	11.84261	1.15576	0.746421	1.548403	0.121525	0.194027	6.505036	4.379295	11.1317	17.85702	17.11289	14.06971
2298	CUNH3orf:	459.8804	1.15529	0.155723	7.418856	1.18E-13	1.37E-12	321.4572	262.7577	268.5523	649.549	678.0981	578.8682
1438	GSTA4	885.2378	1.155223	0.12279	9.408102	5.05E-21	9.34E-20	499.8036	581.3513	565.6296	1265.616	1150.842	1248.185
1937	NEURL1B	813.5233	1.15477	0.142129	8.124807	4.48E-16	6.15E-15	507.3928	451.6147	557.9766	1049.1	1041.747	1273.309
7443	IRF4	64.67987	1.154662	0.32218	3.583899	0.000339	0.001209	39.03022	35.03436	46.614	91.51721	83.42532	92.45811
1138	GOLGA7	1846.879	1.154402	0.111442	10.35875	3.82E-25	8.92E-24	1073.331	1277.659	1082.558	2508.911	2652.497	2486.319
3244	CCDC159	215.594	1.153701	0.188997	6.104345	1.03E-09	8.46E-09	130.6428	147.2538	123.1445	299.105	290.9191	302.4988
23480	TRNAV-CA	1.267532	1.153479	2.365527	0.48762	0.625819	0.708577	1.626259	0	0.695731	0	4.278221	1.004979
1716	DLG4	821.6387	1.153216	0.132731	8.688348	3.68E-18	5.70E-17	451.0159	575.3298	503.0138	1109.367	1144.424	1146.682
17356	LOC11962:	11.06124	1.153173	0.801688	1.438431	0.150312	0.23024	8.673382	3.284471	8.348776	15.62489	21.39111	9.044815
14721	IGSF11	24.3675	1.152825	0.611577	1.885004	0.059429	0.107317	22.76763	9.306001	12.52316	53.57105	29.94755	18.08963
21706	DNAJC5G	2.844742	1.152753	1.484011	0.776782	0.437288	0.53558	0.542086	3.284471	1.391463	6.696381	2.139111	3.014938
2751	ANK3	432.7322	1.152574	0.170174	6.772909	1.26E-11	1.22E-10	237.9759	320.2359	246.9846	549.1032	648.1505	593.9429
2537	FBXL13	419.2321	1.151888	0.162968	7.068195	1.57E-12	1.64E-11	272.6694	221.1544	285.9456	587.0494	613.9248	534.6491
9605	TMEM47	51.91196	1.150671	0.388704	2.960276	0.003074	0.008507	23.8518	43.79295	28.52499	78.12444	74.86887	62.30873
23150	LOC11962:	1.792108	1.1505	2.144008	0.536612	0.591536	0.679308	0	0.547412	2.782925	0	6.417332	1.004979
8420	LOC10324(	57.0642	1.150304	0.351099	3.2763	0.001052	0.003321	39.5723	31.74989	34.09084	84.82083	89.84265	62.30873
22827	HSH2D	1.558157	1.150253	1.954	0.588666	0.556085	0.647634	1.084173	1.094824	0.695731	4.464254	0	2.009959
19479	LOC11962:	7.298561	1.149775	1.041362	1.104107	0.269547	0.367878	4.878777	5.474118	2.782925	20.08914	8.556443	2.009959
20601	MUC13	4.114049	1.149511	1.22701	0.93684	0.348841	0.450169	1.084173	4.379295	2.087194	6.696381	6.417332	4.019918
14276	LOC11962(	21.3094	1.149435	0.584762	1.965646	0.049339	0.091887	13.55216	13.6853	11.82743	26.78552	44.92132	17.08465
1909	BCORL1	938.1561	1.149083	0.140492	8.179017	2.86E-16	3.99E-15	512.8137	689.1915	547.5406	1243.295	1315.553	1320.543
1398	RAB7B	821.932	1.148855	0.12055	9.530145	1.57E-21	2.99E-20	532.8709	529.8946	470.3144	1120.528	1110.198	1167.786
5862	LOC11962(	107.3804	1.148812	0.271166	4.236566	2.27E-05	0.000103	69.38706	74.44801	54.96278	154.0168	166.8506	124.6175
15221	LOC10323!	14.66154	1.148375	0.640752	1.792232	0.073096	0.127669	8.673382	8.758589	9.740239	22.32127	21.39111	17.08465
19284	LOC10321(	6.182698	1.148114	1.008314	1.138647	0.25485	0.351338	1.626259	4.379295	5.565851	8.928508	8.556443	8.039836
5020	NUDT18	241.9192	1.147935	0.245393	4.677953	2.90E-06	1.53E-05	176.7202	103.4608	173.2371	287.9444	310.171	399.9818
21619	LOC11962:	2.832434	1.147133	1.456616	0.787533	0.43097	0.529966	0.542086	2.737059	2.087194	4.464254	2.139111	5.024897
2234	TNFAIP2	657.8182	1.146776	0.152133	7.537975	4.77E-14	5.68E-13	428.7903	370.5978	432.0492	906.2436	770.0798	1039.149
17384	LOC10322!	10.34342	1.146615	0.80065	1.432105	0.152114	0.232624	4.336691	7.663765	6.957314	20.08914	14.97377	8.039836
17993	LOC11962(	9.310372	1.146559	0.852753	1.344539	0.178774	0.264142	3.252518	4.926706	9.044508	15.62489	14.97377	8.039836
1557	CCDC198	728.0566	1.146083	0.12641	9.066391	1.23E-19	2.10E-18	428.7903	495.4077	434.1364	1026.778	1005.382	977.845
2716	TNFRSF14	682.2637	1.145695	0.16804	6.817987	9.23E-12	9.04E-11	466.7364	341.585	468.2272	982.1359	795.7492	1039.149
19427	LOC10324:	6.248957	1.145529	1.028453	1.113838	0.265349	0.363118	2.710432	3.284471	5.565851	15.62489	4.278221	6.029877
23502	LOC11962(	1.270195	1.14528	2.364845	0.484294	0.628177	0.710551	0	1.642235	0.695731	0	4.278221	1.004979
23503	LOC11962(	1.270195	1.14528	2.364845	0.484294	0.628177	0.710551	0	1.642235	0.695731	0	4.278221	1.004979
3107	LOC11961!	302.4417	1.145152	0.182586	6.271863	3.57E-10	3.05E-09	196.7774	159.8442	208.7194	377.2295	462.0479	410.0316
1120	NDRG2	1032.745	1.144102	0.109666	10.43264	1.76E-25	4.18E-24	634.7831	667.295	629.6369	1417.401	1369.031	1478.325
18266	LOC11962:	9.202636	1.143947	0.886563	1.290316	0.196941	0.28665	2.168345	8.211177	6.957314	6.696381	17.11289	14.06971
1139	SQLE	13570.86	1.143441	0.110477	10.35007	4.18E-25	9.76E-24	7899.825	9796.482	7677.396	18649.42	18105.43	19296.61
22135	LOC11962:	2.351998	1.143266	1.626432	0.702929	0.4821	0.579021	0.542086	1.642235	2.087194	6.696381	2.139111	1.004979
21332	LOC10321!	3.370076	1.143058	1.381596	0.827346	0.408041	0.508521	3.252518	2.189647	0.695731	8.928508	2.139111	3.014938

1899	IGFBP6	2126.502	1.142991	0.139458	8.195962	2.49E-16	3.48E-15	1499.411	1074.022	1406.073	2912.926	2671.749	3194.83
1340	CCPG1	4966.078	1.142836	0.117332	9.740218	2.03E-22	4.03E-21	2791.745	3615.655	2879.632	6560.221	7142.491	6806.726
14075	GBGT1	20.05256	1.142738	0.571732	1.998731	0.045637	0.0862	14.09425	13.13788	9.740239	29.01765	34.22577	20.09959
22996	LOC119618	1.540879	1.142366	2.023526	0.564542	0.572385	0.661718	2.168345	0	0.695731	2.232127	2.139111	2.009959
15979	LOC103224	16.75756	1.141578	0.68408	1.668779	0.095161	0.158324	5.420864	13.6853	11.82743	22.32127	34.22577	13.06473
21080	MGAT4D	3.899622	1.141257	1.314426	0.868255	0.385255	0.485846	0.542086	3.284471	3.478657	8.928508	2.139111	5.024897
1172	SLC35A1	1180.27	1.141002	0.111204	10.26045	1.06E-24	2.41E-23	773.5572	696.8552	739.5624	1526.775	1702.732	1642.136
21245	LOC11962	3.08939	1.140905	1.354404	0.842367	0.399582	0.500019	1.626259	2.189647	2.087194	4.464254	2.139111	6.029877
13637	ISM1	23.25153	1.140726	0.547986	2.081669	0.037373	0.072859	9.215468	14.78012	19.48048	37.94616	29.94755	28.13943
19166	LOC11961	7.206504	1.140658	0.984868	1.158184	0.246789	0.342319	7.589209	3.831883	2.087194	13.39276	4.278221	12.05975
12675	LY6G5C	34.59315	1.140653	0.503438	2.265726	0.023468	0.049223	10.29964	25.72836	28.52499	51.33892	53.47777	38.18922
2316	LOC10323	1418.827	1.139593	0.154247	7.388102	1.49E-13	1.71E-12	1005.028	724.7732	931.5843	1886.147	1694.176	2271.254
23625	LOC11962	1.59696	1.13954	2.44913	0.465284	0.641728	0.722101	0	2.189647	0.695731	6.696381	0	0
16543	LOC11962	15.835	1.138957	0.72697	1.566717	0.117181	0.188312	8.673382	12.59047	7.653045	31.24978	27.80844	7.034856
23437	GPX7	1.271614	1.138879	2.30681	0.493703	0.621516	0.704906	1.084173	0	1.391463	0	2.139111	3.014938
23440	TRNAQ-CU	1.271614	1.138879	2.30681	0.493703	0.621516	0.704906	1.084173	0	1.391463	0	2.139111	3.014938
3305	LOC11962	245.3052	1.138683	0.188689	6.0347	1.59E-09	1.28E-08	160.9997	165.3184	132.8847	308.0335	367.927	336.6681
22120	LOC11962	2.842906	1.138237	1.612912	0.705703	0.480373	0.577337	0.542086	0.547412	4.174388	2.232127	8.556443	1.004979
1046	RAP2B	5000.921	1.138085	0.105427	10.79497	3.64E-27	9.24E-26	2980.391	3520.405	2874.066	6682.988	6817.346	7130.329
10840	PALM3	96.66533	1.137191	0.4271	2.662587	0.007754	0.019017	28.18849	49.81448	102.9682	142.8561	164.7115	91.45313
22340	LOC11962	2.592032	1.136679	1.70055	0.668419	0.503866	0.599613	0.542086	2.737059	1.391463	4.464254	6.417332	0
15301	LOC10324	18.29914	1.1364	0.638731	1.779151	0.075215	0.130684	10.84173	10.40082	12.52316	22.32127	40.6431	13.06473
17831	LOC10322	12.69596	1.136346	0.828296	1.371908	0.170092	0.253583	7.589209	13.6853	2.087194	26.78552	14.97377	11.05477
10377	LOC10324	68.50568	1.135798	0.410024	2.770075	0.005604	0.014358	47.7036	27.918	52.87558	55.80317	143.3204	83.4133
1219	DHRS1	1408.038	1.135576	0.112396	10.10337	5.34E-24	1.16E-22	922.631	896.6606	825.8331	1890.612	1816.105	2096.387
9711	HOXC13	53.08258	1.135243	0.387981	2.926029	0.003433	0.009399	23.8518	29.56024	46.614	66.96381	79.14709	72.35852
22846	LOC11962	2.302827	1.135083	1.937667	0.585799	0.558011	0.649335	2.168345	0	2.087194	0	8.556443	1.004979
12241	TRNAY-GU	38.47441	1.134613	0.48116	2.358079	0.01837	0.039892	22.76763	23.53871	27.13352	51.33892	25.66933	80.39836
1269	LOC10322	4835.474	1.134194	0.114135	9.937338	2.86E-23	6.00E-22	3285.043	2677.391	3122.442	6549.061	6085.77	7293.136
21562	LOC10323	3.385518	1.134044	1.426762	0.794837	0.426708	0.526127	2.168345	0.547412	3.478657	6.696381	6.417332	1.004979
19545	GNG8	6.181089	1.134035	1.039025	1.091441	0.275079	0.37416	4.336691	2.189647	4.87012	6.696381	14.97377	4.019918
20929	LOC11962	4.36917	1.133868	1.279128	0.886438	0.375382	0.47673	2.168345	1.094824	4.87012	2.232127	12.83466	3.014938
6771	LOC11961	102.8903	1.133628	0.296195	3.827307	0.00013	0.000509	60.71367	58.02565	73.05179	151.7846	173.268	100.4979
16968	LOC11961	11.68051	1.133395	0.7574	1.496429	0.134542	0.21071	7.047123	4.379295	10.43597	22.32127	12.83466	13.06473
18306	LOC10322	8.021112	1.133024	0.884334	1.281217	0.200118	0.290626	2.710432	6.02153	6.261582	13.39276	10.69555	9.044815
1583	FAM13A	957.8584	1.132911	0.125469	9.029384	1.73E-19	2.90E-18	631.5306	629.5236	540.5833	1236.598	1289.884	1419.031
14011	LOC10321	24.36391	1.132286	0.563019	2.011097	0.044315	0.084085	15.7205	12.59047	17.39328	17.85702	53.47777	29.1444
15477	AXDND1	22.4108	1.132259	0.647264	1.7493	0.080239	0.137828	16.80468	9.853413	14.61036	44.64254	38.50399	10.04979
18208	LOC10323	11.11609	1.132055	0.870568	1.300363	0.193477	0.28249	10.29964	8.758589	1.391463	20.08914	17.11289	9.044815
15323	TRPV1	18.02045	1.131906	0.637569	1.775345	0.075841	0.131582	10.84173	12.04306	10.43597	20.08914	40.6431	14.06971
20627	LOC11962	5.002063	1.131455	1.214252	0.931813	0.351433	0.452943	1.626259	2.737059	4.87012	15.62489	2.139111	3.014938
1067	RSRC1	4586.663	1.131212	0.106075	10.66432	1.49E-26	3.72E-25	2637.25	3241.773	2744.66	6410.669	6404.497	6081.131
3848	ADAP1	442.7115	1.130894	0.205687	5.498131	3.84E-08	2.65E-07	313.3259	200.9001	320.7322	611.6028	511.2474	698.4607
1468	ZHX1	3989.91	1.130673	0.121189	9.329807	1.06E-20	1.92E-19	2281.099	2952.739	2272.259	5301.302	5424.785	5707.278
9920	ASGR1	41.96663	1.130568	0.392591	2.879758	0.00398	0.010666	28.73058	22.44388	27.82925	51.33892	64.17332	57.28383
1366	IGSF8	1102.766	1.130445	0.116998	9.662115	4.37E-22	8.50E-21	719.3486	650.3252	708.2545	1486.597	1413.952	1638.117
18570	LOC10323	10.0369	1.130432	0.911532	1.240145	0.214922	0.307684	9.757555	3.831883	4.87012	8.928508	27.80844	5.024897
905	GCLC	9852.328	1.130127	0.098759	11.44325	2.54E-30	7.47E-29	5954.277	6839.363	5746.045	13272.23	13112.75	14189.31
17315	LOC11962	12.52014	1.130022	0.781458	1.446043	0.148165	0.227489	8.131296	10.40082	4.87012	6.696381	29.94755	15.07469
16940	LOC10321	19.08783	1.129688	0.752604	1.50104	0.133345	0.209267	9.215468	6.02153	20.17621	35.71403	36.36488	7.034856
18861	TEX49	9.051603	1.129514	0.932887	1.210772	0.225983	0.318528	5.96295	4.379295	6.261582	11.16063	23.53022	3.014938
16095	CD177	15.03915	1.129359	0.6857	1.647018	0.099554	0.16444	7.589209	10.40082	9.740239	26.78552	25.66933	10.04979
18328	TRNAC-GC	8.577391	1.128995	0.882856	1.278799	0.200968	0.29152	4.336691	3.284471	8.348776	15.62489	12.83466	7.034856
2741	NOL4L	435.598	1.127216	0.165973	6.791544	1.11E-11	1.08E-10	242.8547	307.6454	269.9438	529.0141	680.2372	583.8931
14846	LOC10322	21.75247	1.126948	0.604995	1.862737	0.062499	0.111918	11.38381	16.96977	11.82743	33.4819	42.78221	14.06971
19146	OIT3	7.221353	1.126932	0.970135	1.161624	0.245388	0.340731	3.794605	2.737059	6.957314	6.696381	17.11289	6.029877
16159	LOC10324	13.15818	1.126694	0.68905	1.635139	0.10202	0.16785	10.29964	7.663765	6.957314	20.08914	12.83466	21.10457
1303	ARAP1	4487.016	1.126606	0.114522	9.837421	7.77E-23	1.58E-21	3032.973	2566.814	2861.543	6129.421	5480.402	6850.945
20012	SBK2	5.695977	1.126358	1.097015	1.026748	0.304539	0.404546	1.626259	2.737059	6.261582	6.696381	12.83466	4.019918
23598	LOC11962	1.507933	1.126173	2.403573	0.468541	0.639398	0.720332	1.626259	0	1.391463	0	0	6.029877
11648	AS3MT	45.45966	1.126144	0.453858	2.48127	0.013092	0.029879	37.40396	26.27577	20.87194	58.0353	91.98176	38.18922
21341	LOC11962	3.341733	1.125879	1.363752	0.825575	0.409045	0.509558	1.626259	3.831883	0.695731	4.464254	6.417332	3.014938
6199	LOC10322	128.106	1.125135	0.276128	4.074687	4.61E-05	0.000198	81.85504	71.71095	86.27069	178.5702	224.6066	125.6224
1929	KNDC1	2597.514	1.124983	0.138197	8.14044	3.94E-16	5.43E-15	1796.474	1306.125	1799.857	3499.975	3313.482	3869.171
1678	BST2	1480.097	1.12489	0.1281	8.781349	1.62E-18	2.56E-17	997.4389	784.9885	1011.593	2035.7	1927.339	2123.522
12235	MIR9-1HG	55.11811	1.124148	0.476339	2.359974	0.018276	0.039712	26.02015	19.15941	58.44144	82.5887	96.25998	48.23901
20238	LOC10324	4.854801	1.12378	1.135757	0.989454	0.322441	0.423564	3.794605	3.284471	2.087194	2.232127	10.69555	7.034856
7827	PSD	75.29669	1.123218	0.324722	3.45901	0.000542	0.001842	47.16151	35.58177	59.8329	113.8385	89.84265	105.5228
974	SNX10	1346.011	1.123115	0.100957	11.12463	9.52E-29	2.60E-27	863.0015	837.5401	839.7478	1908.469	1803.27	1824.038
18538	LOC10324	8.037668	1.122977	0.901249	1.246024	0.212756	0.305109	3.794605	5.474118	5.565851	13.39276	14.97377	5.024897
1525	SLC38A7	871.4049	1.121628	0.122358	9.166768	4.87E-20	8.50E-19	552.9281	566.0238	529.4516	1214.277	1082.39	1283.359
22836	PLET1	1.798191	1.120901	1.907271	0.587699	0.556734	0.648134	1.084173	2.189647	0	2.232127	4.278221	1.004979
17341	SLC38A8	11.48908	1.119796	0.777446	1.440353	0.149768	0.229605	6.505036	7.116354	7.653045	26.78552	12.83466	8.039836
11720	LOC11962	37.85655	1.11929	0.454006	2.465364	0.013687	0.031048	26.56223	14.23271	30.61218	58.0353	53.47777	44.2191

21533	LOC11962:	3.137839	1.119227	1.399329	0.799832	0.423808	0.523241	2.168345	2.189647	1.391463	8.928508	2.139111	2.009959
7487	LOC10322(	72.35049	1.119175	0.313744	3.567154	0.000361	0.001281	42.82482	49.26706	43.83108	95.98146	119.7902	82.40832
835	IGDCC4	24302.35	1.119027	0.094424	11.85104	2.13E-32	6.77E-31	15003.87	15629.15	15342.27	31756.47	31226.74	36855.61
16515	MMP27	17.91969	1.118825	0.711478	1.572537	0.115826	0.186451	11.38381	18.06459	4.174388	37.94616	12.83466	23.11453
477	SAT1	20516.36	1.118072	0.075405	14.82752	9.73E-50	5.42E-48	12781.85	12939.72	13105.49	28002.03	27718.6	28550.46
15258	LOC10321(	19.59133	1.117844	0.625806	1.786245	0.07406	0.12903	12.46799	8.758589	15.30609	42.41041	23.53022	15.07469
1229	LRATD2	2824.466	1.117303	0.11094	10.07124	7.40E-24	1.60E-22	1904.892	1672.343	1774.115	3792.384	3518.837	4284.227
741	LOC10321!	7561.289	1.117261	0.089192	12.52645	5.35E-36	1.92E-34	5029.477	4472.355	4815.157	10294.57	10026.01	10730.17
9784	MMP9	51.88369	1.116633	0.384036	2.907625	0.003642	0.009896	26.56223	42.69812	28.52499	62.49956	87.70354	63.31371
20110	LOC10323:	5.433683	1.116242	1.103939	1.011145	0.311947	0.412388	1.084173	4.926706	4.174388	6.696381	10.69555	5.024897
1036	LRIG1	3310.971	1.116049	0.103077	10.82732	2.56E-27	6.56E-26	2030.113	2181.436	2064.931	4406.219	4188.379	4994.748
23391	LOC11962:	1.577907	1.116024	2.231216	0.500187	0.616944	0.701163	2.168345	0	0.695731	4.464254	2.139111	0
19691	LOC10323:	6.252305	1.115422	1.041256	1.071227	0.284067	0.383502	1.626259	6.568942	3.478657	13.39276	6.417332	6.029877
2369	STAC2	388.1626	1.115278	0.152512	7.312726	2.62E-13	2.94E-12	224.9658	252.3568	259.5078	535.7105	502.691	553.7437
2134	CLCN4	416.7448	1.115204	0.144351	7.725618	1.11E-14	1.39E-13	258.0331	276.9904	255.3334	544.639	577.5599	587.913
1082	RNF139	3202.312	1.114872	0.10548	10.56953	4.13E-26	1.01E-24	2182.44	1797.153	2088.586	4488.807	4410.846	4246.038
15655	RSPH6A	18.45463	1.114203	0.646294	1.723986	0.08471	0.143853	10.29964	8.211177	16.00182	26.78552	36.36488	13.06473
11098	LOC11962!	38.28856	1.11412	0.427554	2.605802	0.009166	0.021957	29.81475	16.96977	25.74206	51.33892	55.61688	50.24897
17059	WDR49	13.0258	1.113887	0.74951	1.486154	0.137238	0.213874	5.420864	10.40082	8.348776	24.5534	21.39111	8.039836
1140	ROBO2	13517.45	1.113735	0.107631	10.34771	4.29E-25	1.00E-23	7961.08	9746.12	7927.859	17801.21	18056.23	19612.17
15901	LOC10323:	15.26011	1.113429	0.661126	1.684141	0.092155	0.154064	14.09425	6.568942	8.348776	22.32127	17.11289	23.11453
21559	LOC11962:	2.853071	1.113342	1.39951	0.795523	0.426309	0.525694	1.084173	2.189647	2.087194	4.464254	4.278221	3.014938
3357	LOC11962⁄	227.5357	1.113233	0.185992	5.985389	2.16E-09	1.71E-08	150.7	137.4004	144.7121	279.0159	318.7275	334.6582
15899	LOC10324⁄	16.60832	1.112722	0.66067	1.684231	0.092137	0.154064	10.84173	14.78012	5.565851	31.24978	17.11289	20.09959
1926	SLC45A4	784.6336	1.112676	0.136653	8.142346	3.88E-16	5.35E-15	538.2918	430.8131	521.1028	1098.206	996.8256	1122.562
10160	LOC11961:	48.67967	1.112103	0.393864	2.823571	0.004749	0.012427	37.94605	32.2973	22.2634	51.33892	74.86887	73.3635
12973	LOC10324!	30.22997	1.111877	0.50351	2.208252	0.027227	0.055794	11.9259	22.44388	22.95914	31.24978	55.61688	37.18424
17670	LOC10324:	12.58589	1.111715	0.799281	1.390894	0.164258	0.24713	4.336691	6.02153	13.2189	26.78552	17.11289	8.039836
10205	LOC10322!	42.89183	1.111699	0.394822	2.815696	0.004867	0.012682	28.73058	23.53871	29.91645	51.33892	53.47777	70.34856
10999	ABCG1	41.89213	1.111631	0.422736	2.629609	0.008548	0.020662	26.02015	20.80165	32.00364	71.42806	59.8951	41.20416
20094	LOC10322:	4.900881	1.110886	1.095926	1.013651	0.310749	0.411131	2.168345	3.831883	3.478657	4.464254	6.417332	9.044815
18142	LOC10323:	9.580296	1.110425	0.844902	1.314264	0.188757	0.276602	9.757555	2.737059	5.565851	13.39276	14.97377	11.05477
17948	TMC4	11.22464	1.110219	0.821458	1.351523	0.176528	0.261478	6.505036	10.40082	4.174388	26.78552	6.417332	13.06473
23285	SELENOW	1.832331	1.109956	2.15303	0.515532	0.606181	0.692031	0	0	3.478657	2.232127	4.278221	1.004979
23286	LOC11962!	1.832331	1.109956	2.15303	0.515532	0.606181	0.692031	0	0	3.478657	2.232127	4.278221	1.004979
4527	SP110	299.2883	1.10934	0.221948	4.998205	5.79E-07	3.40E-06	157.205	254.5465	157.2353	419.6399	367.927	439.176
21293	LOC10321!	3.110028	1.109261	1.328883	0.834732	0.403869	0.504243	2.168345	1.642235	2.087194	4.464254	4.278221	4.019918
1901	BAG3	1648.293	1.109101	0.135346	8.194581	2.51E-16	3.52E-15	909.0788	1218.539	1007.419	2209.806	2070.659	2474.259
17736	SCUBE2	10.61541	1.107722	0.803769	1.378159	0.168154	0.252037	6.505036	7.663765	5.565851	13.39276	23.53022	7.034856
1409	UBXN8	829.8668	1.107479	0.116586	9.499258	2.11E-21	3.99E-20	527.45	498.1448	553.8022	1120.528	1131.59	1147.687
924	SPR	2760.787	1.107146	0.097332	11.37492	5.58E-30	1.60E-28	1799.727	1742.959	1712.195	3776.759	3508.141	4024.943
5567	LOC10322:	131.9397	1.107131	0.252332	4.387591	1.15E-05	5.47E-05	97.57555	75.54283	79.31338	154.0168	171.1289	214.0606
20058	LOC11962!	5.656972	1.107041	1.086207	1.01918	0.308118	0.40832	3.794605	2.737059	4.174388	2.232127	14.97377	6.029877
22763	LOC11962:	2.106565	1.106946	1.846599	0.599451	0.548872	0.641039	0	0.547412	3.478657	4.464254	2.139111	2.009959
16180	INHBB	20.50289	1.106832	0.678983	1.630132	0.103074	0.169358	7.047123	14.78012	16.69755	20.08914	51.33866	13.06473
21831	CUNH4orf:	2.838456	1.106617	1.465556	0.755084	0.450199	0.548236	0.542086	2.737059	2.087194	2.232127	6.417332	3.014938
11373	WDR93	40.14347	1.106171	0.434958	2.543167	0.010985	0.025679	22.22554	29.56024	23.65487	75.89232	51.33866	38.18922
7658	MTARC1	77.73896	1.105506	0.31516	3.507756	0.000452	0.001569	61.25576	45.43518	40.35242	107.1421	119.7902	92.45811
7208	LOC11962(	83.30389	1.10538	0.301691	3.663944	0.000248	0.000916	43.36691	60.76271	53.57132	118.3027	128.3466	95.47305
7899	SPRY3	73.78845	1.105346	0.32127	3.440554	0.000581	0.001954	35.7777	54.74118	49.39693	113.8385	100.5382	88.43819
15694	SLC5A2	16.9677	1.105106	0.643216	1.718093	0.08578	0.145307	8.673382	7.663765	16.00182	31.24978	17.11289	21.10457
3916	LOC11962(	278.1296	1.104858	0.20335	5.433281	5.53E-08	3.76E-07	152.8684	218.9647	158.6268	334.819	391.4573	412.0416
1360	PIK3C2B	1422.27	1.104687	0.114062	9.684975	3.49E-22	6.83E-21	841.8601	941.5483	928.8014	1928.558	1790.436	2102.417
2238	PTHLH	426.0818	1.103788	0.146543	7.532164	4.99E-14	5.93E-13	269.4169	258.3784	282.4669	618.2992	588.2554	539.674
2861	SCD5	404.7179	1.103063	0.167179	6.598077	4.17E-11	3.87E-10	228.7604	245.2405	299.8602	537.9426	515.5257	600.9777
16321	LOC10323:	12.73907	1.102843	0.688601	1.601572	0.10925	0.177956	8.673382	6.568942	9.044508	20.08914	14.97377	17.08465
2435	HSD11B1L	423.51	1.101984	0.152651	7.218982	5.24E-13	5.72E-12	281.8849	249.6198	278.2925	522.3177	581.8381	627.1072
20325	LOC10322:	5.664604	1.101819	1.130867	0.974313	0.329901	0.431509	1.084173	4.926706	4.87012	2.232127	12.83466	8.039836
24107	LOC10323!	1.035295	1.1015	2.827583	0.389555	0.696865	0.768497	0.542086	0	1.391463	0	4.278221	0
1429	LOC10321!	3010.732	1.100732	0.116615	9.439042	3.76E-21	7.00E-20	1820.326	2194.027	1731.675	3977.65	3968.05	4372.666
23452	LOC11962(	1.562404	1.100692	2.23664	0.492118	0.622636	0.705815	2.168345	0	0.695731	2.232127	4.278221	0
13679	LOC10323⁄	26.79853	1.099853	0.53063	2.07273	0.038197	0.074236	22.22554	10.94824	17.39328	40.17829	44.92132	25.12449
17358	LOC11962:	10.36359	1.099765	0.764822	1.437936	0.150452	0.230428	5.420864	8.758589	5.565851	13.39276	14.97377	14.06971
1190	GSR	10054.58	1.099426	0.108036	10.17651	2.52E-24	5.64E-23	6135.334	7269.629	5792.659	13156.16	13467.84	14505.87
1417	ACAP3	2192.131	1.099152	0.11599	9.476227	2.64E-21	4.95E-20	1479.896	1306.672	1403.986	2883.908	2716.671	3361.656
10062	GPR52	50.32556	1.098126	0.385503	2.848556	0.004392	0.011604	42.28274	26.82318	26.43779	66.96381	79.14709	60.29877
1328	CD81	7877.995	1.097797	0.112314	9.774394	1.45E-22	2.90E-21	5469.109	4521.622	5066.316	10321.36	9889.109	12000.46
1305	CNPY4	861.5643	1.097661	0.111614	9.834474	8.00E-23	1.63E-21	544.7968	547.9592	554.4979	1156.242	1167.954	1197.936
13249	LOC11962⁄	24.58293	1.097413	0.508554	2.15791	0.030935	0.062073	14.63633	18.612	13.91463	26.78552	36.36488	37.18424
10959	LOC11962:	45.60753	1.097309	0.416074	2.637291	0.008357	0.020273	23.30971	26.27577	36.87376	66.96381	77.00798	43.21412
1483	MARCHF8	1981.262	1.096861	0.117971	9.297681	1.44E-20	2.57E-19	1213.731	1387.142	1189.701	2645.07	2462.116	2989.814
22272	LOC11962:	2.360327	1.096643	1.616101	0.678574	0.497408	0.593732	1.084173	0.547412	2.782925	4.464254	4.278221	1.004979
5427	LOC11961!	197.8793	1.096108	0.246459	4.447416	8.69E-06	4.26E-05	145.2791	85.94365	148.1908	247.7661	271.6671	288.4291
9792	FANK1	45.98949	1.095958	0.377159	2.905824	0.003663	0.009945	30.35684	32.84471	24.3506	69.19594	59.8951	59.29379

22080	LOC11962(	3.126358	1.095883	1.538129	0.712478	0.476169	0.573322	0.542086	0.547412	4.87012	2.232127	8.556443	2.009959
671	ACSL5	6905.067	1.095388	0.084247	13.00205	1.19E-38	4.72E-37	4356.748	4554.466	4299.62	9091.453	9343.635	9784.48
23173	HSPB3	1.787303	1.094665	2.050769	0.533783	0.593492	0.680878	2.168345	0	1.391463	0	2.139111	5.024897
23424	LOC11962(	2.062058	1.094653	2.208591	0.495634	0.620153	0.703824	1.626259	2.189647	0	0	8.556443	0
19211	LOC10324:	6.514928	1.094312	0.950213	1.151649	0.249465	0.345221	4.878777	3.284471	4.174388	11.16063	8.556443	7.034856
19361	CLVS2	6.241581	1.092954	0.971043	1.125546	0.260358	0.357484	4.336691	3.284471	4.174388	8.928508	10.69555	6.029877
1798	XXYLT1	651.5562	1.092402	0.129149	8.458461	2.71E-17	4.01E-16	418.4907	429.1709	401.437	808.03	932.6523	919.5562
23338	LOC10321:	1.308642	1.092287	2.150019	0.508036	0.611428	0.696496	1.084173	0	1.391463	2.232127	2.139111	1.004979
20265	ARSL	5.241313	1.092224	1.108455	0.985357	0.324449	0.425621	2.168345	1.642235	6.261582	8.928508	6.417332	6.029877
16503	LOC10323:	14.10768	1.092005	0.693355	1.574957	0.115266	0.185685	10.84173	4.926706	11.1317	24.5534	17.11289	16.07967
757	LOC10322:	5327.064	1.091797	0.087763	12.44025	1.58E-35	5.55E-34	3530.609	3317.863	3359.687	7470.929	6866.545	7416.748
7198	LOC11962:	79.77676	1.090828	0.297361	3.668365	0.000244	0.000902	53.66655	50.36189	48.00546	102.6778	130.4858	93.46309
1514	PIK3CB	2914.944	1.090393	0.118481	9.203133	3.48E-20	6.10E-19	1763.949	2110.273	1717.761	3783.455	3745.583	4368.646
18939	TMEM95	9.372187	1.089565	0.910046	1.197263	0.231204	0.324545	5.96295	2.737059	9.044508	8.928508	23.53022	6.029877
19052	LOC10322:	12.64208	1.08947	0.92565	1.176978	0.239204	0.333783	11.38381	3.284471	9.044508	22.32127	27.80844	2.009959
739	CALM1	19056.38	1.08942	0.086845	12.54438	4.27E-36	1.54E-34	11903.13	12829.69	11825.35	25229.73	25051.13	27499.25
22842	LOC11962(	2.871708	1.08926	1.856771	0.586642	0.557444	0.64879	0	0	5.565851	2.232127	6.417332	3.014938
16035	LOC10321(	15.94934	1.089085	0.656202	1.659679	0.096979	0.160785	10.84173	11.49565	7.653045	31.24978	21.39111	13.06473
24096	LOC10324(	1.05488	1.088834	2.784408	0.391047	0.695763	0.767568	0	0	2.087194	2.232127	0	2.009959
24097	LOC11962:	1.05488	1.088834	2.784408	0.391047	0.695763	0.767568	0	0	2.087194	2.232127	0	2.009959
24098	CLDN9	1.05488	1.088834	2.784408	0.391047	0.695763	0.767568	0	0	2.087194	2.232127	0	2.009959
2310	COBL	1080.065	1.088766	0.147109	7.4011	1.35E-13	1.55E-12	610.9313	809.0747	654.6832	1493.293	1281.327	1631.082
2125	SLC35F6	1024.11	1.088739	0.140574	7.744961	9.56E-15	1.20E-13	648.8774	644.3037	676.2509	1406.24	1152.981	1616.007
4135	SPATA6L	201.7792	1.088655	0.206693	5.267012	1.39E-07	8.91E-07	135.5216	114.9565	134.9719	296.8729	295.1973	233.1552
19462	LOC11962:	6.975718	1.087576	0.981706	1.107843	0.26793	0.365991	3.794605	3.831883	6.261582	4.464254	6.417332	17.08465
2340	DGAT1	1247.906	1.087405	0.147891	7.352731	1.94E-13	2.21E-12	888.4796	642.6615	867.577	1698.649	1535.881	1854.187
16286	LOC10323:	21.49441	1.085943	0.674586	1.609792	0.107443	0.175389	9.215468	25.18094	6.261582	42.41041	27.80844	18.08963
1413	EPB41L1	5822.21	1.085773	0.114521	9.481014	2.52E-21	4.74E-20	3430.323	4299.92	3459.176	7801.284	7499.722	8442.833
1444	ANKRD33B	3073.831	1.085752	0.115636	9.389366	6.04E-21	1.11E-19	1910.312	2148.591	1852.037	3819.169	4006.554	4706.319
23092	ST8SIA6	1.566486	1.084211	1.982283	0.546951	0.584413	0.672814	1.626259	0	1.391463	2.232127	2.139111	2.009959
2102	GPC1	13016.37	1.083802	0.139059	7.793841	6.50E-15	8.22E-14	9224.684	6668.023	9146.085	17298.98	15701.07	20059.39
10105	COL5A3	82.00844	1.083675	0.381931	2.837354	0.004549	0.011968	46.07734	50.9093	59.13717	116.0706	162.5724	57.28383
916	TM2D2	1906.629	1.083617	0.094994	11.40719	3.85E-30	1.12E-28	1241.378	1230.034	1195.962	2667.392	2492.064	2612.947
12542	OSBP2	41.57953	1.082505	0.471736	2.294725	0.021749	0.046101	22.22554	26.82318	29.91645	55.80317	85.56443	29.1444
2781	SPTLC3	560.9226	1.081446	0.16073	6.728347	1.72E-11	1.64E-10	342.5986	431.3605	305.4261	790.173	725.1585	770.8193
3326	TIGD5	962.9335	1.08142	0.179657	6.019347	1.75E-09	1.40E-08	725.8536	446.1406	684.5997	1245.527	1202.18	1473.3
8643	LOC11961(	65.74627	1.081415	0.336186	3.216717	0.001297	0.003988	33.06727	47.62483	45.22254	91.51721	102.6773	74.36848
13177	LOC10323!	27.93304	1.080918	0.497086	2.17451	0.029667	0.059854	17.88885	16.42235	18.78475	40.17829	49.19955	25.12449
18887	LOC11962(	7.577606	1.080869	0.896112	1.206177	0.227749	0.320576	3.794605	4.379295	6.261582	11.16063	12.83466	7.034856
7558	ARHGAP22	69.35139	1.080781	0.305349	3.539493	0.000401	0.00141	41.74065	47.62483	43.83108	98.21359	96.25998	88.43819
5016	MMP11	157.2995	1.08078	0.230963	4.679442	2.88E-06	1.52E-05	114.3802	82.11177	106.4469	198.6593	233.1631	209.0357
16600	LOC10323:	14.52982	1.080707	0.693913	1.557409	0.119373	0.191177	10.29964	7.116354	10.43597	11.16063	32.08666	16.07967
10452	LOC11962:	50.5174	1.080661	0.392264	2.754931	0.00587	0.014932	41.74065	21.89647	34.09084	66.96381	62.03421	76.37844
4432	CFAP74	185.3232	1.080573	0.213645	5.057786	4.24E-07	2.54E-06	118.7169	126.9995	111.317	207.5878	293.0582	254.2598
14873	LOC11962:	25.57268	1.08006	0.58094	1.859158	0.063005	0.112619	20.0572	6.568942	22.95914	35.71403	29.94755	38.18922
639	UGCG	25088.97	1.079502	0.081689	13.21477	7.21E-40	3.00E-38	16292.41	16700.44	15360.36	33952.88	33085.62	35142.12
10293	C3AR1	44.01674	1.079309	0.386843	2.790042	0.00527	0.013612	30.35684	26.82318	27.13352	55.80317	72.72976	51.25395
4576	EPM2A	149.4566	1.078766	0.217666	4.956053	7.19E-07	4.18E-06	97.57555	100.1764	91.14081	191.9629	196.7982	219.0855
1986	SMIM8	622.327	1.078606	0.134447	8.022565	1.04E-15	1.39E-14	385.4234	404.5373	407.0029	915.1721	862.0616	759.7645
15520	LOC11962!	16.46029	1.078436	0.620007	1.739392	0.081966	0.140403	8.131296	11.49565	11.82743	24.5534	25.66933	17.08465
1579	BSN	738.4066	1.078396	0.119347	9.035803	1.63E-19	2.74E-18	462.3997	481.175	482.1418	984.368	971.1562	1049.199
1055	ABCA2	3461.794	1.078034	0.100537	10.72278	7.96E-27	2.01E-25	2219.302	2146.949	2315.394	4562.468	4385.177	5141.475
1841	SLC52A2	2126.147	1.077907	0.129363	8.332405	7.92E-17	1.14E-15	1478.27	1228.94	1397.724	2883.908	2487.786	3280.253
15942	TRNAS-GCI	16.92073	1.077285	0.642222	1.677434	0.093458	0.155831	9.757555	8.211177	14.61036	15.62489	34.22577	19.09461
3952	NHSL2	234.1464	1.077004	0.199128	5.408614	6.35E-08	4.27E-07	143.1108	164.2235	142.6249	379.4616	293.0582	282.3992
13315	CD52	30.89654	1.076897	0.502353	2.143708	0.032056	0.063992	20.59928	27.918	11.1317	35.71403	42.78221	47.23404
20223	PRKCQ	4.956772	1.076543	1.085396	0.991844	0.321274	0.422344	3.794605	2.189647	3.478657	6.696381	8.556443	5.024897
3153	ZNF596	249.9241	1.076315	0.173191	6.214597	5.15E-10	4.34E-09	161.5417	163.6761	157.931	323.6584	337.9795	354.7578
19614	LOC10323(	6.761517	1.07614	0.993085	1.083633	0.278527	0.377519	6.505036	2.189647	4.174388	6.696381	14.97377	6.029877
6347	LOC11962!	156.8533	1.076036	0.268502	4.00755	6.14E-05	0.000257	75.35001	127.547	98.79385	196.4272	263.1106	179.8913
15826	MYB	16.2377	1.075918	0.634865	1.694721	0.090128	0.151391	8.673382	10.40082	11.82743	26.78552	25.66933	14.06971
15246	CHRM4	17.98524	1.0756	0.6012	1.789089	0.073601	0.12834	8.673382	12.04306	13.91463	20.08914	32.08666	21.10457
14076	COL24A1	27.45932	1.075587	0.53828	1.998191	0.045696	0.086305	18.43094	16.96977	16.69755	37.94616	55.61688	19.09461
13935	FLT4	32.54111	1.075576	0.531049	2.02538	0.042828	0.081707	14.63633	16.96977	30.61218	46.87467	62.03421	24.11951
1607	EHD1	4310.841	1.075563	0.119998	8.963194	3.15E-19	5.21E-18	2794.455	2483.607	3050.782	5694.156	5249.378	6592.665
2535	LOC10322!	431.4383	1.074806	0.151935	7.074107	1.50E-12	1.58E-11	248.8176	305.4558	278.2925	613.8349	575.4208	566.8084
14353	LOC10322(	21.92587	1.074219	0.550567	1.951115	0.051043	0.094544	17.34676	14.78012	9.740239	33.4819	32.08666	24.11951
1498	ZFYVE1	1638.857	1.073577	0.116045	9.251403	2.22E-20	3.93E-19	1086.883	1007.785	1077.688	2100.431	2059.964	2500.389
13392	CCM2L	34.12292	1.073451	0.504159	2.129191	0.033238	0.065983	11.9259	22.9913	30.61218	53.57105	53.47777	32.15934
4714	RAB27A	203.2031	1.073302	0.220164	4.875009	1.09E-06	6.14E-06	124.6799	148.896	121.0573	218.7484	280.2235	325.6133
6196	CACFD1	181.3306	1.072858	0.263233	4.075692	4.59E-05	0.000197	150.7	78.8273	121.753	254.4625	213.9111	268.3295
6827	LOC10323(	115.742	1.072669	0.281687	3.808023	0.00014	0.000545	88.90216	60.2153	74.44326	194.195	121.9293	154.7668
17566	CPXM1	13.58261	1.072416	0.763276	1.405018	0.160016	0.242174	7.589209	7.116354	11.1317	13.39276	34.22577	8.039836
14910	LOC11962:	26.9682	1.072344	0.579013	1.852021	0.064023	0.114147	10.84173	12.59047	28.52499	31.24978	53.47777	25.12449

1257	SHARPIN	2325.71	1.072026	0.107387	9.982801	1.81E-23	3.83E-22	1479.896	1478.012	1543.828	3243.281	2804.374	3404.87
1785	CDKN2C	850.9707	1.07177	0.126138	8.496779	1.95E-17	2.90E-16	516.0662	614.1961	515.5369	1156.242	1138.007	1165.776
18991	LOC11962(	8.963725	1.071767	0.903145	1.186705	0.235344	0.329458	7.589209	4.379295	4.87012	20.08914	12.83466	4.019918
6078	LOC11962‹	234.9434	1.071691	0.259295	4.133089	3.58E-05	0.000157	170.7572	100.1764	185.7603	294.6408	267.3888	390.937
23075	CUNH6orf2	1.824686	1.071569	1.94759	0.550202	0.582181	0.670738	0	2.737059	0.695731	2.232127	4.278221	1.004979
2214	PGR	461.6442	1.071532	0.141385	7.578849	3.49E-14	4.19E-13	282.9691	321.3307	288.7285	633.9241	611.7857	631.1271
17061	LOC11962‹	12.59559	1.071175	0.720886	1.485916	0.137301	0.213948	5.420864	9.306001	9.740239	22.32127	10.69555	18.08963
3336	TAB2	2480.41	1.070425	0.178211	6.006493	1.90E-09	1.51E-08	1331.906	2197.858	1270.405	3267.834	3390.49	3423.965
1441	YTHDF3	2968.549	1.069898	0.113825	9.399462	5.48E-21	1.01E-19	1790.511	2196.216	1760.896	3924.079	3965.911	4173.68
4645	CUNH9orf1	163.6384	1.069583	0.217391	4.920088	8.65E-07	4.95E-06	95.94929	105.1031	114.7957	245.534	222.4675	197.981
22422	LOC10323	2.097146	1.069318	1.638229	0.652728	0.513931	0.609351	1.084173	2.189647	0.695731	4.464254	2.139111	2.009959
20211	USP50	6.033533	1.06912	1.074549	0.994948	0.319762	0.420606	1.626259	4.926706	4.87012	8.928508	12.83466	3.014938
17169	LOC11962	11.80323	1.069112	0.726371	1.471854	0.14106	0.218422	8.673382	7.663765	6.261582	22.32127	12.83466	13.06473
5139	LOC11962	197.2053	1.068824	0.23197	4.607589	4.07E-06	2.11E-05	105.1648	159.8442	117.5786	225.4448	288.7799	286.4191
1048	GNS	13435.17	1.068204	0.099076	10.78166	4.20E-27	1.07E-25	8911.9	8719.723	8404.435	17379.34	16866.89	20328.72
22677	LOC11962‹	2.336293	1.068186	1.750084	0.610362	0.541622	0.634821	1.626259	2.737059	0	2.232127	6.417332	1.004979
15107	RTBDN	18.25945	1.068168	0.589319	1.812545	0.069902	0.123012	13.55216	8.758589	13.2189	20.08914	27.80844	26.12947
16914	ANXA13	12.5582	1.067165	0.709538	1.504029	0.132574	0.208376	5.420864	10.40082	8.348776	17.85702	19.252	14.06971
23101	LOC10324(	1.552759	1.067007	1.959914	0.544415	0.586156	0.674557	0.542086	1.094824	1.391463	0	4.278221	2.009959
11003	METTL27	45.14455	1.066667	0.405796	2.628577	0.008574	0.020717	27.10432	27.37059	32.69937	46.87467	85.56443	51.25395
24159	LOC11962	1.039377	1.066416	2.797121	0.381255	0.703014	0.773634	0	0	2.087194	0	2.139111	2.009959
17862	SERPINF1	13.11327	1.066401	0.779933	1.367297	0.171532	0.255301	5.96295	14.78012	4.174388	22.32127	21.39111	10.04979
18923	CD200	7.844668	1.066294	0.888951	1.199498	0.230335	0.323564	4.336691	3.831883	6.957314	8.928508	14.97377	8.039836
2786	HCN2	843.89	1.065773	0.158532	6.722752	1.78E-11	1.70E-10	607.1367	450.5199	582.3272	1145.081	981.8518	1296.424
15975	BAALC	17.87196	1.065674	0.638438	1.669189	0.09508	0.158219	10.29964	9.853413	13.91463	29.01765	32.08666	12.05975
22894	LOC11962	2.083418	1.065354	1.836496	0.580101	0.561846	0.652428	0	3.284471	0.695731	2.232127	4.278221	2.009959
19275	KCNB1	8.407159	1.065237	0.934416	1.140003	0.254285	0.350722	2.168345	4.926706	9.044508	11.16063	17.11289	6.029877
4156	FERMT3	410.896	1.06486	0.202713	5.253043	1.50E-07	9.57E-07	314.9522	187.7623	295.6858	560.2639	519.8039	586.908
1503	MXRA8	1320.909	1.064454	0.115128	9.2458	2.33E-20	4.13E-19	885.7691	832.6134	848.7923	1796.862	1610.75	1950.665
14082	LOC10323‹	24.8099	1.06338	0.532258	1.997864	0.045731	0.086335	10.84173	21.89647	15.30609	29.01765	40.6431	31.15436
22800	TRNAI-GAU	2.593024	1.062735	1.793475	0.592556	0.553478	0.64536	0	1.642235	3.478657	0	6.417332	4.019918
19520	LOC10323(	8.959344	1.06258	0.9679	1.09782	0.272283	0.370832	1.626259	7.116354	8.348776	13.39276	19.252	4.019918
2760	CNIH3	362.0623	1.062484	0.157205	6.758602	1.39E-11	1.34E-10	235.8076	227.1759	242.8102	450.8897	477.0217	538.669
2061	SOWAHC	681.6066	1.062292	0.134973	7.870418	3.53E-15	4.56E-14	446.1371	459.8259	420.9175	816.9585	934.7914	1011.009
12878	GJC2	33.91403	1.061981	0.477791	2.222688	0.026237	0.054163	25.47806	15.32753	25.04633	31.24978	64.17332	42.20914
18808	LOC11962	7.596101	1.061362	0.872539	1.216406	0.22383	0.316383	4.878777	4.926706	4.87012	11.16063	10.69555	9.044815
11133	LOC11962‹	40.88247	1.06127	0.408858	2.595693	0.00944	0.022542	30.89892	20.25424	28.52499	60.26743	47.06043	58.28881
1192	GULP1	3478.286	1.061017	0.10434	10.1688	2.73E-24	6.09E-23	2140.699	2435.435	2181.814	4685.235	5073.971	4352.566
18332	FOXS1	9.693185	1.060981	0.830235	1.277928	0.201275	0.291888	4.336691	6.568942	8.348776	13.39276	6.417332	19.09461
2492	LMTK3	470.671	1.060882	0.148533	7.142392	9.17E-13	9.78E-12	300.8579	279.7274	333.9511	691.9594	607.5074	610.0225
17679	LOC11962!	12.63248	1.060512	0.763358	1.389273	0.16475	0.247744	8.131296	10.94824	4.87012	24.5534	19.252	8.039836
22565	LOC10324	2.082531	1.060329	1.681953	0.630415	0.528423	0.622563	0.542086	2.737059	0.695731	2.232127	4.278221	2.009959
4052	PFN4	191.2914	1.060163	0.198866	5.331043	9.77E-08	6.41E-07	122.5115	126.4521	124.5359	232.1412	256.6933	285.4142
2682	APC2	857.1152	1.059999	0.1546	6.856392	7.06E-12	7.00E-11	574.6116	462.0156	633.1155	1207.581	1001.104	1264.264
1993	MYADM	1205.485	1.059989	0.13235	8.008988	1.16E-15	1.54E-14	699.2914	910.3458	736.0838	1540.168	1638.559	1708.465
1275	PCYOX1	6372.431	1.05896	0.106757	9.919373	3.43E-23	7.15E-22	4026.075	4607.565	3768.777	8348.155	8229.159	9254.856
5529	LOC11962	119.1126	1.058779	0.240613	4.400345	1.08E-05	5.20E-05	85.10756	74.99542	71.66033	162.9453	156.1551	163.8117
14659	LOC10321(	22.92751	1.057714	0.557899	1.895889	0.057975	0.10514	13.01007	19.70683	11.82743	20.08914	42.78221	30.14938
19954	CDH22	7.599285	1.057279	1.019439	1.037118	0.299681	0.399269	2.710432	8.211177	3.478657	8.928508	19.252	3.014938
1319	ACSL4	10116.28	1.056773	0.107814	9.801833	1.11E-22	2.23E-21	6157.559	7559.757	5986.768	13633.83	13752.34	13607.42
1931	LOC10323!	1635.758	1.056718	0.129944	8.132085	4.22E-16	5.81E-15	1190.422	920.7467	1078.384	2162.931	2030.016	2432.05
22598	LOC11962(	2.32221	1.056499	1.691238	0.62469	0.532175	0.626067	2.710432	1.094824	0.695731	0	6.417332	3.014938
14000	LOC11962	23.49413	1.056199	0.525123	2.011335	0.04429	0.084069	18.97302	13.6853	13.2189	24.5534	36.36488	34.1693
1286	SHPRH	1894.719	1.056102	0.106736	9.894497	4.40E-23	9.09E-22	1214.273	1345.538	1131.955	2571.41	2494.203	2610.937
14827	KRT17	22.02362	1.056074	0.565577	1.86725	0.061867	0.110928	13.01007	15.32753	13.91463	29.01765	42.78221	18.08963
6626	CYP3A166	93.60413	1.056042	0.271392	3.891203	9.97E-05	0.0004	65.59245	55.28859	61.92009	133.9276	111.2338	133.6623
20493	LOC10322	8.575307	1.055455	1.113201	0.948126	0.343065	0.445049	6.505036	7.116354	2.782925	29.01765	0	6.029877
27039	TRNAR-UC	0.541296	1.055073	3.520958	0.299655	0.76444	NA	0.542086	0	0.695731	0	0	2.009959
20963	LOC11962(	4.209011	1.055065	1.193508	0.884004	0.376694	0.477707	2.710432	2.189647	3.478657	6.696381	2.139111	8.039836
1135	SYNJ2	5254.836	1.054105	0.101583	10.37676	3.16E-25	7.41E-24	3277.996	3772.762	3199.669	7053.521	6751.033	7474.032
14186	LOC10322	35.53242	1.054027	0.532483	1.979455	0.047765	0.089513	37.40396	13.6853	18.08902	66.96381	27.80844	49.24399
2627	MMP19	409.3427	1.053469	0.151919	6.934425	4.08E-12	4.13E-11	262.3698	283.5593	254.6377	517.8535	532.6386	604.9976
16264	LOC10323	15.71503	1.053303	0.652327	1.614685	0.106379	0.173886	13.01007	10.94824	6.261582	22.32127	25.66933	16.07967
20785	LOC10322!	4.950486	1.052992	1.16156	0.906532	0.364654	0.466388	3.794605	1.642235	4.174388	2.232127	12.83466	5.024897
3949	TCF7	264.8203	1.052466	0.194565	5.40934	6.33E-08	4.26E-07	146.9054	205.8268	164.1926	332.5869	378.6226	360.7876
22317	LOC11961	2.904286	1.05246	1.565519	0.672275	0.501408	0.597273	0	2.189647	3.478657	4.464254	4.278221	3.014938
2167	IL27RA	1352.084	1.051873	0.137256	7.6636	1.81E-14	2.22E-13	995.2706	756.5231	891.2319	1772.309	1662.089	2035.083
23517	FCGR1A	1.603514	1.051751	2.188882	0.480497	0.630874	0.713147	1.626259	0	1.391463	4.464254	2.139111	0
18141	LOC11962‹	10.8262	1.051625	0.80015	1.314284	0.18875	0.276602	4.336691	5.474118	11.1317	17.85702	17.11289	9.044815
4848	TMEM145	155.7585	1.051408	0.219841	4.782585	1.73E-06	9.49E-06	97.03346	111.672	96.01093	185.2665	222.4675	222.1005
1171	LDLRAP1	2347.144	1.050924	0.10241	10.26188	1.05E-24	2.38E-23	1534.104	1516.878	1538.262	2908.461	3138.075	3447.08
3338	IL18	460.3236	1.050923	0.175041	6.003871	1.93E-09	1.53E-08	266.7065	354.7229	274.8139	649.549	684.5154	531.6341
19113	CCDC89	8.198816	1.050673	0.900244	1.167098	0.243171	0.338236	2.710432	5.474118	7.653045	15.62489	10.69555	7.034856
21039	LOC11962	4.759788	1.050631	1.200866	0.874895	0.381631	0.482231	3.794605	1.094824	4.174388	8.928508	8.556443	2.009959

17068	LOC10323	12.34996	1.050505	0.707461	1.484895	0.137572	0.214288	5.96295	9.306001	9.044508	17.85702	12.83466	19.09461
1181	LOC11962	1719.398	1.050133	0.102742	10.22111	1.60E-24	3.59E-23	1175.243	1116.173	1067.948	2194.181	2434.308	2328.537
20159	LOC11962	6.303433	1.049976	1.047963	1.001921	0.316382	0.417234	4.336691	1.094824	6.957314	6.696381	10.69555	8.039836
8257	LOC11961	77.1901	1.049888	0.315572	3.326942	0.000878	0.002827	60.71367	37.224	52.87558	107.1421	102.6773	102.5079
26849	LOC11962	0.542184	1.049707	3.519114	0.298287	0.765484	NA	0	0.547412	0.695731	0	0	2.009959
21508	LOC11962	3.68782	1.04904	1.307232	0.80249	0.42227	0.521947	1.084173	3.831883	2.087194	6.696381	6.417332	2.009959
1205	RHOBTB2	2407.245	1.048899	0.103531	10.13126	4.01E-24	8.86E-23	1493.99	1700.261	1514.607	3189.709	3103.85	3441.05
2420	ROGDI	490.1162	1.048798	0.144811	7.242553	4.40E-13	4.84E-12	314.4101	343.2272	302.6431	613.8349	646.0114	720.5703
16229	HHIPL1	25.18577	1.048196	0.646406	1.621575	0.104894	0.171835	19.51511	8.758589	20.17621	42.41041	49.19955	11.05477
19069	CA4	9.000099	1.048164	0.892501	1.174412	0.24023	0.334916	3.252518	4.379295	9.740239	15.62489	14.97377	6.029877
21936	TRNAC-GC	3.482151	1.047968	1.42027	0.737865	0.460596	0.558213	2.710432	1.094824	2.782925	11.16063	2.139111	1.004979
6458	FLT3LG	116.527	1.047679	0.26475	3.957235	7.58E-05	0.000312	61.79785	73.90059	91.83654	162.9453	168.9897	139.6921
15177	STAP2	21.9801	1.047498	0.581659	1.80088	0.071722	0.125639	14.09425	9.853413	18.78475	44.64254	21.39111	23.11453
16850	DEFB110	14.18752	1.04679	0.690801	1.515327	0.12969	0.204617	14.09425	7.116354	6.261582	22.32127	19.252	16.07967
1464	BCL9L	2783.372	1.046709	0.112108	9.336591	9.95E-21	1.81E-19	1669.626	2045.131	1734.458	3718.724	3561.619	3970.674
15209	GFI1	24.07323	1.046562	0.583066	1.794928	0.072665	0.127017	9.215468	13.6853	24.3506	44.64254	21.39111	31.15436
17308	SLC26A9	14.24409	1.04619	0.722714	1.447586	0.147733	0.226917	4.878777	14.23271	8.348776	24.5534	21.39111	12.05975
1346	CHMP7	1970.158	1.045907	0.107523	9.72728	2.31E-22	4.56E-21	1312.391	1323.094	1224.487	2694.177	2408.639	2858.162
1489	CYHR1	2834.36	1.045736	0.112743	9.275362	1.77E-20	3.16E-19	1875.619	1724.347	1955.005	3738.813	3448.246	4264.128
21828	LOC10323	3.405892	1.044803	1.382092	0.755958	0.449675	0.547742	2.168345	0.547412	4.174388	2.232127	4.278221	7.034856
2557	RNASEL	506.5882	1.044735	0.148487	7.035874	1.98E-12	2.06E-11	312.7838	373.3349	307.5133	660.7096	654.5679	730.6201
3996	SH3RF2	236.852	1.0447	0.194278	5.377345	7.56E-08	5.03E-07	143.1108	179.5511	139.842	332.5869	331.5622	294.459
17929	LOC10321	19.22241	1.04385	0.770283	1.355153	0.175369	0.260036	14.63633	7.663765	14.61036	40.17829	34.22577	4.019918
2878	TSTD1	887.0594	1.04379	0.158655	6.578974	4.74E-11	4.38E-10	657.5508	447.7829	633.1155	1151.778	1266.354	1165.776
14144	LOC11962	23.68994	1.04364	0.525325	1.986658	0.04696	0.088266	15.17842	15.32753	15.30609	35.71403	38.50399	22.10955
15294	LOC10324	17.57181	1.042844	0.585872	1.779988	0.075078	0.130505	12.46799	10.94824	11.1317	20.08914	25.66933	25.12449
12114	LOC10321	38.6251	1.042716	0.437513	2.383278	0.017159	0.037657	19.51511	21.34906	35.4823	44.64254	53.47777	57.28383
13950	LOC10323	28.94446	1.042475	0.515563	2.022015	0.043175	0.08228	20.59928	10.94824	25.04633	35.71403	49.19955	32.15934
9480	LOC11962	83.98539	1.042371	0.348668	2.989584	0.002794	0.007834	75.35001	43.24553	45.22254	93.74933	151.8769	94.46807
20278	DLX2	5.47989	1.041953	1.059757	0.9832	0.325509	0.426751	3.252518	5.474118	2.087194	4.464254	8.556443	9.044815
1620	DGKG	1497.392	1.04187	0.116744	8.924369	4.48E-19	7.36E-18	932.3886	1098.656	906.538	2042.396	1923.06	2081.312
1882	ZFP41	690.8471	1.041862	0.12662	8.228277	1.90E-16	2.68E-15	440.1741	449.4251	466.14	993.2965	851.366	944.6807
20519	LOC10321	5.563427	1.041798	1.105204	0.94263	0.34587	0.447515	3.252518	5.474118	2.087194	13.39276	2.139111	7.034856
6118	DNAJC22	128.3203	1.041686	0.253392	4.110961	3.94E-05	0.000171	92.69677	67.33165	91.14081	165.1774	196.7982	156.7768
16358	LOC11962	18.32529	1.04121	0.652722	1.595182	0.110671	0.179863	13.55216	15.87494	6.957314	24.5534	12.83466	36.17926
1295	HMGA2	1661.155	1.041166	0.105472	9.87145	5.54E-23	1.14E-21	1100.977	1138.617	1018.551	2129.449	2374.413	2204.925
19731	LOC11962	9.438629	1.040796	0.978369	1.063807	0.287416	0.387257	2.168345	7.116354	9.044508	4.464254	27.80844	6.029877
22966	LOC10322	2.614549	1.040408	1.828429	0.569017	0.569344	0.659062	0	1.642235	3.478657	0	8.556443	2.009959
2082	SLC27A1	785.7456	1.040153	0.132943	7.824029	5.12E-15	6.53E-14	487.8777	476.2483	580.9357	1055.796	1001.104	1112.512
14346	LOC11961	25.01293	1.040051	0.532482	1.953212	0.050795	0.094129	16.80468	14.23271	17.39328	33.4819	47.06043	21.10457
20314	C2CD4D	6.082085	1.039288	1.065043	0.975818	0.329155	0.430766	2.168345	3.284471	6.261582	8.928508	12.83466	3.014938
19049	CUNH19or	9.537845	1.039233	0.882573	1.177504	0.238995	0.333526	5.96295	2.189647	10.43597	15.62489	14.97377	8.039836
1645	PCBP4	1574.237	1.038766	0.117166	8.865742	7.60E-19	1.23E-17	1075.499	932.7897	1088.124	2051.325	1987.234	2310.448
14447	NANOS3	25.30611	1.0385	0.536745	1.93481	0.053014	0.097554	14.09425	10.94824	25.04633	33.4819	32.08666	36.17926
17440	LOC11961	13.19577	1.03825	0.731185	1.419955	0.155621	0.237224	9.215468	7.116354	9.044508	20.08914	25.66933	8.039836
4085	LOC10322	243.2469	1.037463	0.19548	5.307259	1.11E-07	7.24E-07	136.0637	184.4778	157.2353	308.0335	355.0924	318.5785
1850	HOOK3	926.5159	1.037067	0.125008	8.296008	1.08E-16	1.55E-15	569.1907	668.9372	583.0229	1167.402	1328.388	1242.155
1540	SPRYD3	2099.675	1.037064	0.11371	9.120251	7.50E-20	1.29E-18	1408.882	1470.348	1251.621	2691.945	2673.888	3101.367
2667	DPP4	749.1107	1.037058	0.150813	6.876452	6.14E-12	6.12E-11	461.3155	587.3729	424.3961	966.511	996.8256	1058.243
15718	LMNTD1	18.16672	1.036882	0.604558	1.715108	0.086325	0.146009	9.215468	14.23271	11.82743	24.5534	32.08666	17.08465
843	CYBRD1	6380.188	1.036686	0.087757	11.81319	3.34E-32	1.05E-30	4157.26	4353.566	4037.329	8366.012	8321.14	9045.82
5466	DBNDD1	147.54	1.036349	0.233852	4.431648	9.35E-06	4.55E-05	98.65972	90.32295	102.9682	196.4272	164.7115	232.1503
1175	SNTB1	2354.586	1.036205	0.101047	10.2547	1.13E-24	2.55E-23	1529.768	1655.373	1447.121	3035.693	3155.188	3304.372
1314	LOC10322	2551.723	1.035967	0.105602	9.810089	1.02E-22	2.06E-21	1769.37	1588.589	1665.581	3278.995	3247.17	3760.633
21847	LOC11962	3.645926	1.035692	1.375179	0.753133	0.45137	0.54926	2.168345	1.642235	3.478657	0	8.556443	6.029877
875	NPTN	6886.419	1.035281	0.088864	11.65018	2.29E-31	6.96E-30	4659.774	4528.738	4364.323	9120.471	8802.44	9842.769
18998	NHLH1	8.480718	1.034817	0.87277	1.18567	0.235753	0.329902	6.505036	4.926706	4.87012	17.85702	10.69555	6.029877
6572	EGR2	123.4589	1.034557	0.264487	3.911567	9.17E-05	0.000371	71.5554	100.7238	69.57314	189.7308	160.4333	148.737
1957	KCTD9	2414.686	1.034331	0.128116	8.073399	6.84E-16	9.29E-15	1419.182	1888.571	1445.73	3129.442	3253.587	3351.607
23874	LOC11962	1.572509	1.034327	2.425139	0.426502	0.669742	0.745794	1.626259	0	1.391463	0	6.417332	0
12447	KCNH2	72.66747	1.034206	0.447438	2.311397	0.020811	0.044449	34.69353	46.53	60.52863	93.74933	158.2942	42.20914
2460	SMIM10L2	496.9297	1.033344	0.14388	7.182008	6.87E-13	7.42E-12	342.0565	309.8351	328.3852	691.9594	596.8119	712.5304
2072	FICD	546.8462	1.033333	0.131667	7.848111	4.22E-15	5.42E-14	368.0766	338.3005	371.5206	718.7449	733.715	750.7197
17457	LOC11962	11.589	1.032711	0.728372	1.417833	0.156239	0.237935	5.96295	7.116354	9.740239	13.39276	19.252	14.06971
14557	SH2D3C	22.34394	1.032403	0.53922	1.914623	0.055541	0.101432	12.46799	14.23271	17.39328	22.32127	38.50399	29.1444
11037	LOC10322	49.74653	1.031689	0.393617	2.621045	0.008766	0.021117	40.11439	24.08612	33.39511	58.0353	85.56443	57.28383
1147	KRT18	5388.869	1.030984	0.099873	10.32293	5.55E-25	1.29E-23	3656.915	3482.634	3489.789	7055.753	6650.495	7997.627
24281	LOC11962	1.076405	1.030927	2.825296	0.364892	0.715192	0.783056	0	0	2.087194	2.232127	2.139111	0
8791	CORO1A	62.55422	1.03004	0.324326	3.175941	0.001494	0.004517	41.74065	36.12918	45.22254	89.28508	85.56443	77.38342
5949	RNF207	150.3814	1.030039	0.246089	4.185637	2.84E-05	0.000127	113.8381	83.75401	98.09812	178.5702	248.1368	179.8913
1597	SLC37A4	2851.917	1.029356	0.114551	8.986024	2.56E-19	4.27E-18	1968.858	1742.959	1920.219	3718.724	3450.386	4310.357
13267	LOC11962	75.99138	1.02933	0.477767	2.154462	0.031204	0.062531	27.6464	39.96106	81.40057	120.5349	141.1813	45.22408
15436	REC8	20.1682	1.028943	0.586486	1.754421	0.079358	0.136673	15.7205	10.40082	13.2189	40.17829	21.39111	20.09959
20678	LOC11962	5.886937	1.028631	1.112208	0.924855	0.355041	0.456442	2.168345	4.926706	4.174388	15.62489	6.417332	2.009959

13782	MAMDC4	25.34427	1.028204	0.501375	2.050766	0.04029	0.077718	20.0572	13.6853	16.00182	37.94616	34.22577	30.14938
13368	LOC11962	31.14153	1.027809	0.481903	2.132811	0.03294	0.065508	20.0572	15.32753	25.74206	37.94616	55.61688	32.15934
18012	TLCD3B	17.30399	1.02688	0.765683	1.341128	0.179879	0.265488	9.757555	2.737059	21.56767	29.01765	25.66933	15.07469
13833	LOC10324	28.2838	1.026812	0.502535	2.043265	0.041026	0.078841	18.97302	18.612	17.39328	42.41041	49.19955	23.11453
13843	LOC10322	25.77157	1.026494	0.502825	2.041452	0.041206	0.07914	14.63633	20.25424	16.00182	26.78552	42.78221	34.1693
19154	LOC11962	9.827911	1.026381	0.885059	1.159675	0.246181	0.34169	8.673382	1.642235	9.044508	17.85702	10.69555	11.05477
9562	ROM1	63.69038	1.026329	0.34571	2.968756	0.00299	0.008313	47.7036	48.71965	29.22072	80.35657	87.70354	88.43819
3372	ATP1A3	313.2519	1.026294	0.172031	5.965747	2.44E-09	1.92E-08	185.9356	229.3656	205.2408	399.5507	402.1528	457.2657
2450	SLCO2A1	767.4566	1.026254	0.142586	7.197443	6.14E-13	6.66E-12	460.2313	587.3729	470.3144	991.0644	988.2691	1107.487
2008	RAB13	4210.481	1.026168	0.1289	7.960994	1.71E-15	2.26E-14	2540.217	3351.803	2427.407	5462.015	5668.643	5812.801
11958	SLC7A7	48.56411	1.025981	0.425194	2.41297	0.015823	0.035178	21.68345	31.74989	41.74388	78.12444	74.86887	43.21412
17863	LOC10321	10.79019	1.025899	0.750363	1.367204	0.171561	0.25533	7.589209	6.02153	7.653045	11.16063	19.252	13.06473
17724	LOC11961	11.34652	1.025806	0.743331	1.380012	0.167583	0.251365	7.589209	9.853413	4.87012	17.85702	12.83466	15.07469
1226	WASHC5	6880.945	1.025695	0.101788	10.07681	7.00E-24	1.52E-22	4419.088	5046.042	4134.732	9078.06	9014.212	9593.534
2957	LOC11962	1529.848	1.024964	0.158118	6.482292	9.03E-11	8.12E-10	770.8468	1215.802	1036.64	2209.806	2021.46	1924.536
14035	LOC11962	25.60406	1.024631	0.510544	2.006939	0.044756	0.084771	21.14137	13.6853	15.30609	35.71403	40.6431	27.13445
22795	LOC11961	1.863134	1.024382	1.725676	0.593612	0.552772	0.644678	1.084173	1.094824	1.391463	4.464254	2.139111	1.004979
16082	LOC10324	16.84002	1.024275	0.620479	1.65078	0.098783	0.163298	9.757555	10.94824	12.52316	15.62489	32.08666	20.09959
1137	TNS1	6733.688	1.024083	0.098773	10.36809	3.46E-25	8.10E-24	4197.375	4788.759	4335.798	8812.437	8526.495	9741.266
19103	LOC11962	8.443488	1.02365	0.87516	1.169672	0.242133	0.336989	7.589209	6.02153	2.782925	13.39276	12.83466	8.039836
15665	PTX4	22.2315	1.02308	0.593892	1.722671	0.084948	0.144165	14.63633	7.116354	22.2634	29.01765	34.22577	26.12947
2201	LOC11962	1202.652	1.022512	0.134491	7.602822	2.90E-14	3.50E-13	870.0486	694.6656	818.8758	1618.292	1433.204	1780.824
15736	CASP10	18.57865	1.022277	0.596985	1.7124	0.086823	0.146682	10.29964	9.853413	16.69755	31.24978	19.252	24.11951
12412	CFAP91	34.81505	1.022238	0.440992	2.31804	0.020447	0.043795	20.59928	23.53871	24.3506	40.17829	62.03421	38.18922
16070	LOC10322	19.3346	1.021552	0.618133	1.652642	0.098404	0.162762	14.63633	10.40082	12.52316	29.01765	36.36488	13.06473
20281	LOC11962	6.125728	1.021465	1.039684	0.982476	0.325865	0.427155	4.878777	2.737059	4.174388	13.39276	8.556443	3.014938
1934	PHF20L1	5516.718	1.021373	0.125641	8.129281	4.32E-16	5.94E-15	3274.744	4353.019	3298.462	7095.932	7198.107	7880.044
2538	CIB1	3935.924	1.021293	0.144492	7.068158	1.57E-12	1.64E-11	3002.074	2059.911	2736.311	4984.34	4864.338	5968.573
15248	LOC10323	18.7623	1.020529	0.570454	1.788976	0.073619	0.128355	13.55216	11.49565	11.82743	26.78552	27.80844	21.10457
17685	LOC10324	15.08674	1.020321	0.73526	1.387701	0.165228	0.248379	3.794605	15.32753	10.43597	20.08914	27.80844	13.06473
986	MRPL13	3149.015	1.020087	0.09219	11.06509	1.85E-28	5.00E-27	2133.652	1961.377	2144.94	4343.719	4098.536	4211.869
18413	LOC10322	11.11056	1.020074	0.807581	1.263124	0.206545	0.298213	4.878777	12.59047	4.174388	17.85702	17.11289	10.04979
922	MLF1	3137.664	1.019993	0.0896	11.38386	5.03E-30	1.45E-28	2058.844	2029.256	2126.851	4292.38	4301.752	4016.903
18273	SLC6A11	13.55929	1.019367	0.790636	1.2893	0.197294	0.287039	6.505036	12.04306	7.653045	22.32127	27.80844	5.024897
14321	LOC11962	26.7787	1.018688	0.520308	1.957855	0.050247	0.093283	19.51511	15.32753	17.39328	42.41041	44.92132	21.10457
21054	LOC11962	4.793253	1.018094	1.167221	0.872237	0.383079	0.483716	3.252518	3.831883	2.087194	11.16063	6.417332	2.009959
15287	LAG3	22.30844	1.017513	0.570925	1.782217	0.074714	0.129938	15.7205	9.306001	18.78475	35.71403	34.22577	20.09959
1958	MAPRE2	975.9606	1.017468	0.126038	8.07272	6.87E-16	9.33E-15	602.8	720.9414	614.3308	1274.545	1249.241	1393.907
18826	LOC11962	9.996174	1.01738	0.837709	1.21448	0.224565	0.317117	4.336691	10.40082	4.87012	8.928508	21.39111	10.04979
16040	LOC11962	20.41704	1.017021	0.613422	1.657947	0.097328	0.161306	15.7205	14.78012	10.43597	11.16063	34.22577	36.17926
14480	LOC11962	24.62514	1.016685	0.52701	1.929156	0.053712	0.098613	15.17842	16.96977	16.00182	35.71403	42.78221	21.10457
17540	MYRFL	14.2751	1.016002	0.720533	1.410069	0.158519	0.240264	6.505036	10.94824	10.43597	15.62489	32.08666	10.04979
20635	LOC11962	5.278469	1.015757	1.092128	0.930071	0.352334	0.453935	5.96295	1.642235	2.782925	6.696381	8.556443	6.029877
2100	IRAK2	727.971	1.014999	0.130211	7.79503	6.44E-15	8.15E-14	510.6454	452.7096	485.6205	926.3327	926.2349	1066.283
16174	LOC11962	17.80586	1.014289	0.621632	1.631654	0.102752	0.168893	10.84173	12.04306	11.82743	31.24978	27.80844	13.06473
2360	IQSEC2	497.0576	1.014029	0.138415	7.326016	2.37E-13	2.67E-12	313.3259	322.973	351.3443	642.8526	701.6283	650.2217
4350	XKR6	209.9427	1.01384	0.198061	5.118828	3.07E-07	1.88E-06	134.9795	126.9995	154.4524	276.7837	310.171	256.2698
20755	LOC10321	5.04234	1.013569	1.113069	0.910607	0.362503	0.464437	1.626259	5.474118	2.782925	8.928508	6.417332	5.024897
9656	LOC10322	72.77118	1.013071	0.344	2.94497	0.00323	0.008892	54.20864	31.20247	59.8329	93.74933	94.12087	103.5129
24556	LOC11962	1.060903	1.012889	3.140946	0.322479	0.74709	0.80882	0	0	2.087194	0	4.278221	0
5002	ARHGAP28	198.0148	1.012735	0.216082	4.686806	2.78E-06	1.47E-05	127.9324	156.5598	109.9256	245.534	256.6933	291.444
1322	LAMB2	9092.437	1.012445	0.103402	9.791332	1.23E-22	2.47E-21	6065.404	5866.065	6155.831	11566.88	11153.32	13747.11
1053	LOC11962	3492.193	1.012333	0.094365	10.72784	7.53E-27	1.90E-25	2337.476	2251.505	2359.225	4738.806	4331.699	4934.449
1480	GPAT4	2457.83	1.011844	0.108779	9.301851	1.38E-20	2.48E-19	1604.576	1703.546	1585.572	3171.852	3018.285	3663.15
17636	LOC11961	14.12023	1.011073	0.721941	1.400492	0.161366	0.243248	7.589209	13.6853	6.261582	29.01765	17.11289	11.05477
14678	LOC11962	22.25682	1.010987	0.534084	1.892936	0.058366	0.105714	18.43094	12.59047	13.2189	33.4819	25.66933	30.14938
12570	LOC11962	38.33925	1.010596	0.441439	2.289319	0.022061	0.046648	31.44101	16.96977	28.52499	46.87467	44.92132	61.30375
1416	VCL	10948.82	1.010401	0.106616	9.476991	2.62E-21	4.91E-20	7109.463	8038.742	6647.713	14151.69	13748.06	15997.26
19035	LOC10323	12.52963	1.009748	0.856662	1.1787	0.238517	0.333128	1.626259	12.59047	10.43597	20.08914	21.39111	9.044815
1955	EXTL3	1483.213	1.00967	0.124935	8.081552	6.39E-16	8.70E-15	1045.143	921.2941	991.4172	1930.79	1760.488	2250.149
22252	LOC10321	3.47906	1.009655	1.47689	0.683636	0.494205	0.590439	2.710432	0	4.174388	6.696381	4.278221	3.014938
18429	LOC10323	9.26304	1.00953	0.801109	1.260166	0.20761	0.29949	6.505036	4.926706	6.957314	11.16063	14.97377	11.05477
1542	TMCC3	1030.697	1.009463	0.110723	9.117039	7.72E-20	1.33E-18	679.2342	719.2991	654.6832	1374.99	1336.944	1419.031
20071	LOC10323	6.11394	1.009315	0.992212	1.017237	0.309041	0.409339	3.794605	4.926706	3.478657	11.16063	4.278221	9.044815
2132	ILDR2	1021.394	1.009235	0.130553	7.730454	1.07E-14	1.34E-13	623.9414	766.924	645.6387	1305.794	1302.718	1483.35
19051	CNTD1	10.28566	1.00899	0.857243	1.177017	0.239189	0.33378	7.047123	5.474118	7.653045	6.696381	27.80844	7.034856
1728	LOC10324	1280.707	1.00861	0.116648	8.64664	5.30E-18	8.16E-17	846.1968	897.7554	809.1356	1531.239	1798.992	1800.923
4478	CCDC160	220.2143	1.008389	0.200694	5.024512	5.05E-07	3.00E-06	135.5216	164.771	136.3633	319.1942	310.171	255.2648
2688	CBX7	540.8849	1.007902	0.147153	6.849357	7.42E-12	7.34E-11	400.0597	316.9514	361.7803	738.834	675.959	751.7246
12474	LOC11962	40.33248	1.007898	0.437162	2.305546	0.021136	0.045046	31.44101	20.80165	28.52499	33.4819	68.45154	59.29379
20112	LOC10322	6.892088	1.006731	0.99582	1.010956	0.312037	0.412466	7.047123	1.642235	4.87012	8.928508	12.83466	6.029877
2248	SESN1	508.996	1.006114	0.13397	7.50998	5.91E-14	6.99E-13	329.5885	336.1109	349.2571	656.2453	714.463	668.3113
22811	LOC11961	2.639495	1.005353	1.703674	0.590109	0.555118	0.646904	2.710432	1.642235	0.695731	2.232127	8.556443	0
1951	PLEKHM1	1914.711	1.00499	0.124129	8.09635	5.66E-16	7.72E-15	1363.889	1174.198	1287.799	2395.072	2333.77	2933.535

8957	LOC10324!	69.08487	1.004319	0.320956	3.129148	0.001753	0.005203	41.74065	43.79295	51.48412	100.4457	102.6773	74.36848
17856	LOC11962!	13.73221	1.004162	0.733962	1.368139	0.171269	0.254994	11.38381	12.59047	3.478657	17.85702	14.97377	22.10955
20005	THSD8	7.227018	1.003671	0.975239	1.029154	0.303407	0.403204	5.96295	2.189647	6.261582	15.62489	4.278221	9.044815
3139	KCNQ1	357.4757	1.003653	0.161029	6.232745	4.58E-10	3.88E-09	236.3497	221.1544	258.1163	486.6037	432.1004	510.5296
6509	LOC10324(	123.4407	1.003596	0.254947	3.936489	8.27E-05	0.000338	81.31296	74.99542	89.74935	140.624	203.2155	150.7469
1347	RTL8C	4811.961	1.003313	0.103165	9.725305	2.35E-22	4.64E-21	2938.108	3507.815	3166.273	6361.562	6077.213	6820.796
1348	LOC10323ź	4811.961	1.003313	0.103165	9.725305	2.35E-22	4.64E-21	2938.108	3507.815	3166.273	6361.562	6077.213	6820.796
18643	MFAP5	9.810255	1.002065	0.811646	1.234608	0.216976	0.309867	7.047123	6.568942	5.565851	13.39276	19.252	7.034856
5453	CCDC103	142.4188	1.001884	0.225735	4.438325	9.07E-06	4.42E-05	92.15468	105.1031	87.66215	194.195	175.4071	199.9909
3461	FRY	398.7336	1.001783	0.169945	5.894739	3.75E-09	2.88E-08	245.023	312.5721	237.2444	542.4069	564.7252	490.43
20113	LOC11962¿	5.824203	1.000958	0.990362	1.010699	0.31216	0.412607	3.794605	4.379295	3.478657	6.696381	8.556443	8.039836
13874	LOC10324!	31.97805	1.000441	0.491557	2.035248	0.041826	0.080146	19.51511	19.15941	24.3506	49.10679	55.61688	24.11951
1560	SLC4A4	9423.793	1.000423	0.110404	9.061462	1.29E-19	2.19E-18	5987.344	7163.431	5695.257	12127.15	12115.92	13453.66
22405	LOC11962:	2.965147	1.000202	1.52464	0.656025	0.511808	0.607294	0.542086	1.094824	4.174388	6.696381	4.278221	1.004979
6560	IKZF4	139.9887	0.998517	0.254975	3.916141	9.00E-05	0.000365	80.22878	95.79707	102.9682	171.8738	235.3022	153.7619
2036	LOC10324{	792.0275	0.998377	0.126403	7.898397	2.83E-15	3.69E-14	527.9921	481.7224	576.7613	1082.582	1011.799	1071.308
9802	LOC11961{	93.52814	0.998266	0.343706	2.90442	0.003679	0.009979	81.85504	36.67659	68.87741	102.6778	145.4595	125.6224
2166	SEC11C	3378.8	0.998041	0.13022	7.664289	1.80E-14	2.21E-13	1981.868	2594.185	2183.205	4881.662	4712.461	3919.42
3445	PELI3	305.4675	0.99778	0.168974	5.904916	3.53E-09	2.72E-08	212.4979	194.3312	206.6322	408.4792	363.6488	447.2159
7121	DEFB112	141.9469	0.997343	0.270326	3.689412	0.000225	0.000839	94.86511	117.1461	70.26887	220.9806	194.6591	153.7619
10338	LOC11962!	62.19161	0.997126	0.358681	2.779983	0.005436	0.01398	49.32986	31.74989	43.83108	60.26743	100.5382	87.43321
6978	LOC10322:	99.55996	0.996857	0.266338	3.742823	0.000182	0.000693	74.80792	59.12048	66.09448	131.6955	121.9293	143.7121
22783	STMND1	2.710723	0.996549	1.674166	0.595251	0.551676	0.64371	2.710432	1.094824	1.391463	8.928508	2.139111	0
1582	CLDN1	19659.25	0.996152	0.11031	9.030488	1.71E-19	2.87E-18	12058.71	15250.35	12079.98	25622.59	25908.91	27034.95
1292	MPC2	3815.373	0.995077	0.100709	9.880734	5.05E-23	1.04E-21	2565.153	2305.699	2778.055	5046.839	5159.535	5036.957
3616	ARHGDIG	513.3353	0.994642	0.17385	5.721271	1.06E-08	7.77E-08	344.2248	290.6757	395.1754	783.4766	569.0034	697.4558
1730	KLF6	4274.789	0.994539	0.115119	8.639264	5.66E-18	8.69E-17	2705.553	3262.574	2605.514	5482.104	5435.48	6157.509
1251	ACAD11	2274.209	0.994178	0.099357	10.0061	1.43E-23	3.05E-22	1572.05	1568.882	1420.683	3064.71	2881.382	3137.546
18984	LOC11961!	8.516167	0.993983	0.837302	1.187126	0.235178	0.329339	4.878777	5.474118	6.957314	11.16063	8.556443	14.06971
1129	TNKS1BP1	8519.48	0.993906	0.095593	10.39724	2.55E-25	6.01E-24	5664.803	5692.535	5735.609	11111.53	10434.58	12477.83
21829	LOC11962!	5.31565	0.993788	1.314847	0.75582	0.449757	0.547748	4.878777	5.474118	0	6.696381	12.83466	2.009959
1412	ITGA5	8626.428	0.993625	0.104723	9.488165	2.35E-21	4.43E-20	5973.25	5768.626	5567.938	10897.24	10550.09	13001.42
16186	LOC11961!	16.4492	0.993334	0.609792	1.628973	0.103319	0.169698	11.9259	9.306001	11.82743	17.85702	25.66933	22.10955
16588	ETV2	16.82384	0.992146	0.636268	1.559321	0.11892	0.19059	11.9259	6.568942	15.30609	26.78552	19.252	21.10457
8314	SPATA2L	260.108	0.991758	0.299637	3.309867	0.000933	0.002985	230.3867	88.68071	204.545	316.962	308.0319	412.0416
2092	SLC22A11	661.1968	0.991006	0.126975	7.804749	5.96E-15	7.58E-14	459.6892	410.0114	459.1827	890.6187	849.2269	898.4516
22232	LRRC63	3.715001	0.990677	1.441762	0.68713	0.492001	0.588334	0.542086	6.02153	0.695731	4.464254	8.556443	2.009959
1854	P2RX4	1097.641	0.990611	0.119504	8.289359	1.14E-16	1.63E-15	736.1533	803.0531	666.5107	1412.936	1439.621	1527.569
1066	UGP2	3551.586	0.990513	0.092817	10.67168	1.38E-26	3.44E-25	2339.103	2539.443	2254.17	4839.251	4665.4	4672.15
1321	ELP3	2081.895	0.990316	0.101131	9.792432	1.21E-22	2.44E-21	1356.842	1507.025	1319.107	2756.677	2716.671	2835.047
20027	LOC11962;	6.106767	0.990303	0.967978	1.023064	0.306277	0.406554	4.336691	3.831883	4.174388	6.696381	8.556443	9.044815
4543	LOC10322:	203.8347	0.989763	0.198677	4.981775	6.30E-07	3.69E-06	120.3432	147.2538	142.6249	247.7661	286.6408	278.3793
21784	LOC11961!	4.343927	0.989627	1.298913	0.761889	0.446126	0.544449	3.252518	1.094824	4.174388	13.39276	2.139111	2.009959
13413	LOC11962:	33.54424	0.988434	0.46534	2.12411	0.033661	0.066717	23.30971	19.70683	23.65487	42.41041	62.03421	30.14938
23711	LOC10322(	1.630897	0.98821	2.184265	0.452423	0.650965	0.72981	0	1.094824	2.087194	4.464254	2.139111	0
23713	CHST8	1.630897	0.98821	2.184265	0.452423	0.650965	0.72981	0	1.094824	2.087194	4.464254	2.139111	0
21404	LOC10323!	4.514153	0.988113	1.211187	0.815822	0.414602	0.514935	4.336691	1.094824	3.478657	4.464254	10.69555	3.014938
1418	PAX8	5206.475	0.98794	0.104297	9.472355	2.74E-21	5.13E-20	3282.333	3865.822	3324.9	7024.504	6445.14	7296.151
1796	LOC10322:	4059.638	0.987637	0.116707	8.462518	2.62E-17	3.87E-16	2997.738	2360.987	2810.059	5435.229	4969.154	5784.662
2546	SEMA6B	1332.354	0.987215	0.1398	7.061629	1.65E-12	1.72E-11	941.0619	837.5401	907.2337	1687.488	1512.351	2108.447
2042	TP53I3	2005.83	0.987178	0.125111	7.890406	3.01E-15	3.92E-14	1480.98	1184.052	1374.069	2656.231	2400.082	2939.565
21838	TRNAH-GU	4.304224	0.986887	1.307886	0.754567	0.450509	0.548413	2.168345	4.379295	2.087194	11.16063	0	6.029877
8451	UNC80	83.76486	0.986256	0.301732	3.268653	0.001081	0.003399	45.53526	58.57306	64.70302	93.74933	130.4858	109.5428
17982	TRNAI-AAU	11.71588	0.986155	0.732391	1.346486	0.178146	0.263375	10.84173	7.116354	5.565851	17.85702	12.83466	16.07967
1944	RIN1	1109.297	0.985819	0.121596	8.107358	5.17E-16	7.07E-15	777.3519	688.6441	770.1746	1502.221	1330.527	1586.863
1907	TRMT12	887.9809	0.985078	0.120359	8.184472	2.73E-16	3.81E-15	630.9885	558.9075	600.4162	1102.671	1195.763	1239.14
4267	AMDHD2	877.9465	0.984501	0.190129	5.178076	2.24E-07	1.40E-06	691.1601	404.5373	674.8594	1127.224	1084.529	1285.369
22052	LOC11962:	3.694172	0.98316	1.372944	0.716096	0.473932	0.571353	3.794605	2.737059	0.695731	2.232127	10.69555	2.009959
2988	LOC10324(	355.7958	0.982895	0.152603	6.440857	1.19E-10	1.06E-09	239.6022	251.262	225.417	486.6037	470.6043	461.2856
1551	HACL1	1438.337	0.982587	0.108239	9.07798	1.11E-19	1.90E-18	1047.853	898.8502	953.152	1890.612	1923.06	1916.496
18994	LOC10324(	8.537692	0.982495	0.828241	1.186243	0.235526	0.329655	4.878777	5.474118	6.957314	11.16063	10.69555	12.05975
2130	THRA	752.4002	0.982445	0.127053	7.732571	1.05E-14	1.32E-13	482.4569	528.7998	508.5796	995.5286	917.6785	1081.358
4067	NUDT17	236.0444	0.981168	0.184441	5.319698	1.04E-07	6.79E-07	169.673	143.4219	162.1054	319.1942	329.423	292.449
18364	LOC11962(	13.30945	0.981097	0.771981	1.270882	0.203771	0.294995	4.878777	9.853413	11.82743	11.16063	32.08666	10.04979
14560	DOC2A	25.40263	0.980352	0.512292	1.913659	0.055664	0.101636	14.09425	15.87494	20.87194	37.94616	38.50399	25.12449
16190	LOC10322;	20.51708	0.980021	0.601882	1.62826	0.10347	0.169904	18.97302	8.758589	13.2189	24.5534	38.50399	19.09461
6418	UNC13D	148.7455	0.979858	0.246622	3.973114	7.09E-05	0.000294	119.259	78.8273	102.9682	171.8738	207.4937	212.0507
16634	LOC10323:	17.09142	0.979765	0.631292	1.551999	0.120662	0.192847	11.9259	7.663765	14.61036	22.32127	29.94755	16.07967
15819	LOC10322;	18.93618	0.97956	0.577394	1.69652	0.089788	0.150895	15.17842	9.306001	13.91463	24.5534	23.53022	27.13445
19138	GSDMA	10.36976	0.979179	0.841788	1.163213	0.244743	0.339978	4.878777	4.926706	11.1317	6.696381	23.53022	11.05477
10680	LOC11962:	68.27165	0.978878	0.362951	2.696997	0.006997	0.017417	62.33993	36.12918	38.26523	113.8385	87.70354	71.35354
14788	DNAI3	26.25323	0.978869	0.521859	1.875736	0.060692	0.109108	11.38381	21.34906	20.17621	44.64254	27.80844	32.15934
2626	KLF10	1447.87	0.978373	0.141044	6.936669	4.01E-12	4.06E-11	860.2911	1186.789	878.013	1899.54	1871.722	1990.864
1232	PBXIP1	4651.076	0.978357	0.097273	10.05784	8.48E-24	1.83E-22	3192.889	3240.131	2966.599	5948.618	5859.024	6699.193

2824	UPK1B	414.7732	0.977716	0.146858	6.657557	2.78E-11	2.62E-10	263.9961	293.9601	281.7712	531.2462	534.7777	582.8881
16028	LOC10324!	19.48304	0.977679	0.588715	1.660699	0.096774	0.160505	17.88885	8.211177	13.2189	26.78552	25.66933	25.12449
10588	LOC10322:	73.57816	0.97747	0.358629	2.725576	0.006419	0.016117	55.8349	41.05589	50.09266	127.2312	106.9555	60.29877
5607	LOC11962‹	188.205	0.97731	0.223642	4.369984	1.24E-05	5.89E-05	113.8381	153.8227	112.7085	216.5163	278.0844	254.2598
18172	CYP1D1	13.42931	0.977094	0.747171	1.307724	0.190967	0.279378	9.215468	4.379295	13.2189	22.32127	21.39111	10.04979
4509	BORCS6	225.7658	0.977081	0.195121	5.007564	5.51E-07	3.25E-06	166.4205	137.9478	153.0609	314.7299	258.8324	323.6034
16739	CNGA4	16.77287	0.977029	0.636408	1.535223	0.124729	0.198096	9.757555	12.59047	11.1317	17.85702	34.22577	15.07469
7601	DACH1	120.5218	0.976959	0.277156	3.524946	0.000424	0.001481	68.84497	108.3875	66.09448	160.7131	158.2942	160.7967
16607	LOC10324:	16.03081	0.976924	0.627759	1.556208	0.119659	0.191542	8.131296	9.853413	14.61036	20.08914	21.39111	22.10955
17928	LOC11962!	13.09189	0.976866	0.720726	1.355392	0.175293	0.259937	5.420864	10.94824	9.740239	17.85702	23.53022	11.05477
20124	LOC10324:	6.132374	0.976645	0.968133	1.008792	0.313074	0.41359	3.794605	3.831883	4.87012	6.696381	8.556443	9.044815
14694	LOC10323ł	25.05956	0.97562	0.515915	1.891049	0.058618	0.10605	19.51511	18.06459	12.52316	33.4819	40.6431	26.12947
2719	TMEM50B	1017.846	0.974858	0.143052	6.8147	9.45E-12	9.24E-11	715.0119	561.6445	784.785	1301.33	1343.361	1400.941
19352	LOC11962!	9.679989	0.974468	0.865137	1.126374	0.260007	0.357151	5.96295	5.474118	7.653045	17.85702	17.11289	4.019918
15416	LOC11961!	23.75443	0.974458	0.554338	1.757876	0.078769	0.135837	9.757555	23.53871	14.61036	31.24978	34.22577	29.1444
13496	LOC11961ł	28.55794	0.974327	0.462298	2.107576	0.035068	0.069078	21.14137	18.612	18.08902	37.94616	36.36488	39.1942
1804	LOC10322:	890.9709	0.973879	0.115432	8.436787	3.26E-17	4.81E-16	616.8943	609.2693	576.7613	1243.295	1118.755	1180.851
3294	SLC66A1	984.5413	0.973857	0.161076	6.045936	1.49E-09	1.20E-08	764.8839	534.2739	696.4271	1334.812	1116.616	1460.235
11006	WNT4	48.27789	0.973822	0.370568	2.627919	0.008591	0.020751	33.06727	29.01283	35.4823	55.80317	77.00798	59.29379
3995	TBC1D10A	533.2397	0.973817	0.181076	5.377936	7.53E-08	5.01E-07	403.8543	291.2231	387.5224	694.1915	592.5337	830.113
3723	ZFYVE19	360.9464	0.973593	0.173307	5.617723	1.93E-08	1.38E-07	250.4439	250.1672	232.3743	495.5322	391.4573	545.7039
7628	LOC10321:	94.59283	0.973194	0.276745	3.516577	0.000437	0.001524	55.8349	70.06871	64.70302	127.2312	141.1813	108.5378
14306	LOC11962:	32.14115	0.972561	0.49619	1.96006	0.049989	0.092895	29.81475	13.13788	21.56767	49.10679	47.06043	32.15934
3011	AFMID	593.588	0.972154	0.151822	6.403272	1.52E-10	1.34E-09	427.1641	337.2057	440.398	772.3159	742.2714	842.1728
7999	SRD5A2	87.61064	0.972036	0.284799	3.413064	0.000642	0.002135	55.8349	60.2153	62.61582	116.0706	96.25998	134.6672
19467	LOC11961ł	9.820498	0.971962	0.878561	1.10631	0.268592	0.366801	12.46799	2.737059	4.87012	8.928508	12.83466	17.08465
10824	LOC10322(	55.79717	0.971903	0.364624	2.665496	0.007687	0.018882	37.94605	47.62483	27.13352	78.12444	70.59065	73.3635
20240	LOC10322‹	6.915848	0.971881	0.982673	0.989018	0.322654	0.423795	3.794605	3.831883	6.261582	4.464254	17.11289	6.029877
17181	LOC10322‹	16.72125	0.971677	0.66071	1.470656	0.141384	0.218771	12.46799	15.87494	5.565851	15.62489	25.66933	25.12449
19553	TRNAS-AG.	8.058676	0.971144	0.890651	1.090375	0.275548	0.374657	5.96295	2.737059	7.653045	13.39276	8.556443	10.04979
21640	AKAP4	4.560589	0.971028	1.239086	0.783665	0.433237	0.532237	0.542086	4.379295	4.174388	6.696381	8.556443	3.014938
4781	LOC10323‹	256.3998	0.970789	0.201249	4.823832	1.41E-06	7.83E-06	168.5889	186.6674	164.1926	272.3195	414.9875	331.6432
1830	LOC10322:	792.9102	0.970704	0.116015	8.367089	5.91E-17	8.58E-16	538.8339	546.8644	522.4943	1060.26	1009.66	1079.348
9732	LOC11962:	70.7761	0.970164	0.332176	2.920636	0.003493	0.009542	60.71367	38.31883	43.83108	89.28508	109.0946	83.4133
10462	LOC11962‹	249.915	0.969454	0.352035	2.753858	0.00589	0.014966	106.791	302.7187	96.70666	377.2295	299.4755	316.5685
2177	HERC3	662.1381	0.969337	0.12663	7.654891	1.93E-14	2.36E-13	456.4367	434.645	452.9211	808.03	928.374	892.4218
11885	ABCA13	46.20966	0.968641	0.398842	2.428634	0.015156	0.033901	31.9831	32.2973	28.52499	55.80317	83.42532	45.22408
21538	LOC10324:	3.984901	0.968388	1.21215	0.798901	0.424348	0.523785	3.252518	2.189647	2.782925	2.232127	6.417332	7.034856
6593	LOC10323(	115.1374	0.968009	0.247945	3.904123	9.46E-05	0.000381	80.77087	69.5213	84.1835	138.3919	156.1551	161.8017
16562	TMDD1	23.60813	0.967497	0.618752	1.563627	0.117905	0.189252	17.88885	9.853413	19.48048	29.01765	51.33866	14.06971
15608	LOC10324:	21.18071	0.967243	0.559755	1.727976	0.083993	0.143064	15.7205	14.78012	11.82743	35.71403	29.94755	19.09461
17684	LOC10324:	13.73789	0.966693	0.696448	1.388032	0.165127	0.248242	8.131296	8.211177	11.1317	26.78552	17.11289	11.05477
22355	LOC10321!	2.673267	0.966211	1.44984	0.666426	0.505139	0.600721	1.084173	1.642235	2.782925	2.232127	4.278221	4.019918
1706	SEMA4D	1176.254	0.966119	0.110963	8.706702	3.13E-18	4.88E-17	760.0051	837.5401	794.5252	1540.168	1478.125	1647.161
1287	PLSCR1	2794.231	0.965531	0.097585	9.894267	4.41E-23	9.10E-22	1802.979	2045.678	1827.686	3665.153	3796.921	3626.971
2017	HGH1	977.5859	0.965215	0.121516	7.943134	1.97E-15	2.60E-14	633.699	672.2217	684.5997	1283.473	1176.511	1415.011
21157	SRRM5	5.315057	0.965142	1.125857	0.857251	0.391306	0.491699	1.626259	7.116354	2.087194	4.464254	8.556443	8.039836
11210	LOC10322:	54.01368	0.963919	0.373442	2.581174	0.009846	0.023351	36.31979	27.918	46.614	62.49956	66.31243	84.41828
9959	ACHE	57.2762	0.963776	0.335496	2.872689	0.00407	0.010865	35.7777	37.224	43.83108	71.42806	77.00798	78.3884
3709	PPIL4	936.5123	0.963439	0.171023	5.633372	1.77E-08	1.27E-07	553.4702	822.76	527.3644	1303.562	1197.902	1214.015
15195	LOC10321(	24.14701	0.962854	0.535874	1.796792	0.072369	0.126615	17.34676	13.6853	17.39328	35.71403	40.6431	20.09959
7659	LOC10321:	102.62	0.962741	0.274488	3.507403	0.000453	0.001571	75.89209	56.93083	75.13899	136.1597	154.016	117.5826
1773	VPS37A	2119.035	0.961727	0.112812	8.525016	1.53E-17	2.29E-16	1324.317	1621.434	1368.504	2694.177	2821.487	2884.291
1623	MGAT4B	11891.35	0.961443	0.107826	8.916634	4.81E-19	7.87E-18	8521.056	7380.753	8311.903	15573.55	14122.41	17438.4
11487	LOC10324:	48.98096	0.961108	0.381437	2.519705	0.011745	0.027183	31.9831	31.74989	35.4823	87.05295	51.33866	56.27885
18908	LOC10323:	12.09437	0.960479	0.799093	1.201962	0.229378	0.32251	6.505036	14.23271	3.478657	22.32127	14.97377	11.05477
22375	TRNAM-CA	2.685575	0.960191	1.449615	0.662376	0.50773	0.603263	1.084173	2.189647	2.087194	4.464254	4.278221	2.009959
18435	LOC11962!	10.94208	0.959455	0.762085	1.258987	0.208035	0.300006	9.215468	6.568942	6.261582	11.16063	21.39111	11.05477
1652	SH3PXD2B	5775.49	0.959344	0.108471	8.844231	9.22E-19	1.48E-17	3656.373	4326.196	3790.345	7546.821	6994.892	8338.315
2817	SUMF1	1141.148	0.959195	0.143907	6.665358	2.64E-11	2.49E-10	859.2069	649.2304	820.2673	1506.686	1341.222	1670.276
1860	PDK2	2139.724	0.958475	0.115776	8.278679	1.25E-16	1.78E-15	1366.058	1634.572	1363.633	2796.855	2648.219	3029.008
21375	KRT82	4.849542	0.958339	1.167826	0.820618	0.411864	0.512253	1.084173	2.737059	6.261582	6.696381	4.278221	8.039836
4866	RABEP2	354.2575	0.957083	0.201004	4.761517	1.92E-06	1.05E-05	285.6795	177.3614	260.2035	430.8005	494.1346	477.3652
20089	LOC10323ł	8.838396	0.956989	0.943557	1.014236	0.31047	0.410864	9.215468	1.642235	6.957314	8.928508	19.252	7.034856
20714	LOC11962‹	5.608154	0.95681	1.043352	0.917054	0.359114	0.460898	3.252518	6.02153	2.087194	6.696381	8.556443	7.034856
23114	LOC11961ł	2.170418	0.95643	1.766881	0.54131	0.588294	0.676638	1.626259	0	2.782925	4.464254	2.139111	2.009959
19640	LOC10323:	9.736398	0.956404	0.88607	1.079377	0.280419	0.37958	5.96295	7.663765	6.261582	22.32127	2.139111	14.06971
22730	LOC11962:	2.425068	0.955647	1.582059	0.604053	0.545809	0.638396	2.168345	0.547412	2.087194	4.464254	4.278221	1.004979
14111	LOC10322:	31.93069	0.955457	0.479745	1.991593	0.046416	0.087441	17.34676	29.01283	18.08902	44.64254	51.33866	31.15436
17069	LOC10322ł	18.6065	0.95498	0.643177	1.484786	0.137601	0.214288	8.131296	19.70683	9.740239	35.71403	19.252	19.09461
6711	LOC11961!	156.5798	0.954544	0.247878	3.850861	0.000118	0.000466	126.8482	87.58589	103.664	209.8199	241.7195	169.8415
1881	LYRM2	928.4345	0.954422	0.115951	8.23128	1.85E-16	2.62E-15	662.4295	633.3555	597.6332	1252.223	1264.214	1160.751
20695	LOC11962(	6.126089	0.953921	1.033311	0.923169	0.355919	0.457219	3.794605	3.284471	5.565851	2.232127	12.83466	9.044815
20647	HGFAC	6.216963	0.953512	1.026606	0.928801	0.352992	0.454512	4.878777	3.284471	4.174388	13.39276	8.556443	3.014938

23180	LOC11961	2.167223	0.953472	1.789302	0.532874	0.594121	0.681347	1.626259	0.547412	2.087194	4.464254	4.278221	0
18579	LOC11962	11.59555	0.953294	0.769797	1.23837	0.215579	0.308476	8.131296	6.02153	9.044508	20.08914	19.252	7.034856
16741	LOC11962	18.53993	0.952879	0.620831	1.534845	0.124822	0.198219	18.43094	9.853413	9.044508	29.01765	27.80844	17.08465
1920	NTAQ1	1003.593	0.952869	0.116909	8.150507	3.62E-16	5.02E-15	660.2612	719.2991	669.9893	1435.258	1251.38	1285.369
2365	WDR47	823.2128	0.952586	0.130093	7.322334	2.44E-13	2.74E-12	561.0594	603.2478	521.1028	1053.564	1005.382	1194.921
21026	LOC11962	4.838347	0.952406	1.086604	0.876498	0.380759	0.481427	3.252518	3.284471	3.478657	6.696381	4.278221	8.039836
3648	PPP1R3G	297.6582	0.952183	0.167067	5.699397	1.20E-08	8.76E-08	214.1241	194.3312	201.7621	381.6937	367.927	426.1113
2005	CYB5R1	964.1806	0.952144	0.119555	7.964032	1.67E-15	2.21E-14	701.4598	616.9331	655.379	1256.687	1197.902	1356.722
15290	LOC11962	21.23485	0.952062	0.534435	1.781438	0.074841	0.130127	14.09425	14.78012	14.61036	31.24978	23.53022	29.1444
10080	LOC11962	62.85475	0.951773	0.334907	2.841906	0.004484	0.011827	37.40396	39.96106	50.78839	80.35657	96.25998	72.35852
2737	TFCP2L1	919.5585	0.951718	0.140032	6.796441	1.07E-11	1.04E-10	556.7227	705.6138	621.2881	1174.099	1105.92	1353.707
1041	CUNH12or	10284.55	0.951446	0.087996	10.81242	3.01E-27	7.68E-26	7340.934	6517.485	7175.773	13819.1	13149.11	13704.91
13014	BMP8A	33.74385	0.95142	0.432264	2.201016	0.027735	0.056653	24.93597	23.53871	20.87194	37.94616	44.92132	50.24897
2689	CRY2	1060.086	0.950856	0.13883	6.849068	7.43E-12	7.35E-11	763.2576	664.0105	745.824	1381.687	1195.763	1609.977
19766	LOC10323	11.25553	0.950742	0.898246	1.058443	0.289854	0.389849	7.589209	2.737059	12.52316	6.696381	29.94755	8.039836
20687	DMRT1	7.004507	0.95041	1.029193	0.923452	0.355772	0.45716	1.626259	8.211177	4.174388	11.16063	12.83466	4.019918
2007	GRB14	724.9556	0.950062	0.119322	7.962166	1.69E-15	2.24E-14	493.8407	509.093	479.3589	946.4218	990.4082	930.611
1123	DNAJC3	5843.835	0.949947	0.091119	10.42538	1.90E-25	4.50E-24	4017.944	4089.714	3855.743	7653.963	7225.916	8219.727
9753	SLC25A35	83.45353	0.949851	0.325731	2.916058	0.003545	0.009663	42.82482	71.71095	57.04997	82.5887	121.9293	124.6175
18161	ACTN2	19.41495	0.949794	0.724959	1.310136	0.19015	0.278351	7.589209	20.25424	11.1317	24.5534	44.92132	8.039836
3594	MPND	697.9457	0.949659	0.165077	5.752807	8.78E-09	6.49E-08	558.891	387.0202	482.8376	1017.85	806.4447	934.6309
17890	LOC10321	16.40506	0.948915	0.6966	1.36221	0.173132	0.25727	14.63633	9.853413	8.348776	22.32127	34.22577	9.044815
4643	SNX21	385.2995	0.948822	0.192735	4.922931	8.53E-07	4.88E-06	301.9421	202.5424	286.6413	491.0679	457.7697	571.8333
1720	ESCO2	1396.54	0.948612	0.109266	8.681644	3.90E-18	6.03E-17	912.8734	1027.492	921.1483	1747.755	1852.47	1917.501
1307	CD47	2890.385	0.9484	0.096504	9.82756	8.57E-23	1.74E-21	2082.696	1926.342	1913.261	3602.653	3805.478	4011.878
24155	LOC11962	1.055565	0.948326	2.484771	0.381655	0.702717	0.773411	0.542086	1.642235	0	0	2.139111	2.009959
1711	PPM1B	6199.119	0.94765	0.10898	8.695608	3.45E-18	5.36E-17	4116.604	4801.349	3782.691	7845.926	8167.125	8481.022
8408	LOC10322	92.35614	0.947355	0.288888	3.279317	0.001041	0.00329	66.13454	50.36189	73.05179	136.1597	104.8164	123.6125
2774	PLPP2	774.6347	0.947346	0.140616	6.737106	1.62E-11	1.55E-10	578.4062	465.8475	546.1491	986.6001	941.2087	1129.597
1835	TMEM63B	3588.144	0.94716	0.113312	8.358861	6.33E-17	9.17E-16	2303.325	2808.77	2241.646	4665.145	4558.445	4951.534
1705	ACSS2	4704.254	0.947103	0.108671	8.715346	2.90E-18	4.52E-17	3490.494	2966.425	3187.841	5935.226	5790.573	6854.965
1983	SLC48A1	2045.435	0.946913	0.117957	8.027592	9.94E-16	1.33E-14	1359.553	1563.408	1270.405	2745.516	2468.534	2865.196
3850	GPRC5C	1313.24	0.946606	0.172192	5.497386	3.85E-08	2.66E-07	1024.001	701.7819	969.8495	1694.184	1420.369	2069.253
10894	HDAC9	67.91939	0.946486	0.356947	2.651615	0.008011	0.019549	48.24569	45.43518	43.83108	98.21359	115.512	56.27885
5277	TSNAXIP1	183.9855	0.946464	0.208748	4.53401	5.79E-06	2.92E-05	118.7169	127.547	132.8847	209.8199	239.5804	275.3644
14886	ELMO1	36.22108	0.946367	0.509509	1.857409	0.063253	0.112964	17.88885	27.918	27.82925	31.24978	81.28621	31.15436
12551	SOX18	38.68999	0.946304	0.412733	2.292776	0.021861	0.046305	20.59928	29.01283	29.91645	46.87467	53.47777	52.25893
17009	LOC10323	16.9779	0.946265	0.634736	1.4908	0.136014	0.212589	16.26259	8.758589	9.740239	29.01765	14.97377	23.11453
1879	LOC10322	5465.554	0.945971	0.114886	8.233994	1.81E-16	2.56E-15	3390.208	4328.385	3488.397	7194.145	6855.85	7536.341
2925	ZC3H3	1026.39	0.945092	0.145013	6.517286	7.16E-11	6.51E-10	760.5472	569.3083	777.8277	1305.794	1302.718	1442.146
16512	LOC11962	22.30269	0.944977	0.60069	1.573152	0.115684	0.186255	17.88885	15.87494	11.1317	26.78552	47.06043	15.07469
7151	LOC11962	107.354	0.944924	0.25674	3.680463	0.000233	0.000866	68.84497	72.25836	80.00911	147.3204	121.9293	153.7619
23459	GPR78	1.612364	0.944458	1.922063	0.491377	0.62316	0.706198	1.084173	1.642235	0.695731	2.232127	0	4.019918
1084	STAT1	22593.91	0.943937	0.089387	10.56009	4.56E-26	1.12E-24	15408.26	16215.98	14746.03	28738.63	28563.54	31891.01
17044	LOC10322	15.58232	0.943744	0.63459	1.487171	0.13697	0.213647	7.589209	12.59047	11.82743	17.85702	23.53022	20.09959
15142	LOC10321	25.78824	0.943149	0.521878	1.80722	0.070728	0.124178	20.59928	13.13788	18.78475	29.01765	47.06043	26.12947
22167	PDZD9	3.779943	0.943145	1.353275	0.696935	0.485843	0.582674	0.542086	4.926706	2.087194	6.696381	6.417332	2.009959
6969	KCNK5	107.8253	0.942733	0.251485	3.74866	0.000178	0.000678	78.06044	68.97389	75.13899	138.3919	132.6249	153.7619
3316	PGPEP1	558.3373	0.942108	0.156271	6.028687	1.65E-09	1.33E-08	430.9587	329.5419	388.9138	680.7987	703.7674	816.0433
4595	NIM1K	218.0494	0.942044	0.19049	4.945373	7.60E-07	4.40E-06	148.5317	146.7064	153.7566	247.7661	308.0319	303.5038
6384	HSPA6	141.8734	0.940721	0.236026	3.985672	6.73E-05	0.00028	107.8752	94.70224	90.44508	178.5702	162.5724	217.0756
3661	ZFP36	528.2836	0.940541	0.165396	5.686597	1.30E-08	9.41E-08	316.5784	434.645	335.3425	627.2277	727.2976	728.6101
4072	ARSA	473.5955	0.940408	0.176838	5.3179	1.05E-07	6.85E-07	362.6558	256.1887	356.2145	647.3168	551.8906	667.3064
14703	LOC10321	27.47153	0.940244	0.497412	1.890272	0.058722	0.106176	22.76763	16.96977	16.00182	40.17829	42.78221	26.12947
7340	LURAP1	150.8425	0.94003	0.259748	3.619006	0.000296	0.001071	117.0907	71.71095	121.753	203.1236	190.3808	200.9959
3208	TEX9	1040.678	0.939548	0.152725	6.151911	7.66E-10	6.34E-09	634.2411	850.1305	651.9003	1410.704	1510.212	1186.881
17072	LOC11962	18.7991	0.93913	0.632565	1.484637	0.13764	0.21433	21.14137	7.663765	9.740239	22.32127	27.80844	24.11951
18996	CYP2S1	11.27391	0.938908	0.791713	1.185921	0.235654	0.329781	5.96295	9.853413	6.957314	11.16063	25.66933	8.039836
23960	TRNAG-CC	1.365053	0.938364	2.271743	0.413059	0.679563	0.754015	1.626259	1.094824	0	4.464254	0	1.004979
21250	TRNAR-AC	5.886268	0.938181	1.1145	0.841795	0.399903	0.500278	3.794605	7.663765	0.695731	6.696381	6.417332	10.04979
2865	ZMIZ1	1038.671	0.938056	0.142318	6.591264	4.36E-11	4.05E-10	636.9515	850.1305	651.2046	1285.705	1366.892	1441.141
21386	METTL24	6.245931	0.938039	1.146476	0.818193	0.413247	0.513709	5.96295	1.642235	4.87012	11.16063	12.83466	1.004979
13266	LOC10322	46.1392	0.938028	0.435393	2.154441	0.031206	0.062531	48.24569	25.18094	21.56767	51.33892	64.17332	66.32864
17257	LOC10323	17.48293	0.937816	0.643624	1.457086	0.145093	0.22352	14.09425	6.02153	16.00182	17.85702	27.80844	23.11453
20135	LOC10324	7.576392	0.937773	0.931987	1.006208	0.314316	0.415003	3.252518	3.831883	8.348776	11.16063	12.83466	6.029877
2056	TOM1	1859.648	0.937634	0.119043	7.87644	3.37E-15	4.36E-14	1358.468	1129.858	1342.762	2408.465	2263.179	2655.156
13007	LOC10324	35.49128	0.937379	0.425541	2.202793	0.027609	0.056431	25.47806	27.918	19.48048	44.64254	49.19955	46.22906
24451	LOC11962	0.795946	0.937223	2.776215	0.33759	0.735672	0.799895	1.084173	0.547412	0	0	2.139111	1.004979
21814	LOC11961	4.848158	0.937212	1.236711	0.757826	0.448555	0.546635	6.505036	1.094824	2.087194	6.696381	10.69555	2.009959
3831	NPTXR	427.368	0.936995	0.169964	5.512919	3.53E-08	2.45E-07	275.922	297.792	307.5133	633.9241	447.0741	601.9827
18574	LOC10323	11.36779	0.936975	0.756158	1.239127	0.215298	0.308157	10.29964	6.568942	6.261582	22.32127	10.69555	12.05975
3964	TMEM37	288.8253	0.936898	0.173457	5.401337	6.61E-08	4.44E-07	189.7302	189.4045	214.2853	421.872	367.927	349.7329
21034	LOC10324	6.481706	0.936279	1.069535	0.875407	0.381352	0.482012	2.168345	8.758589	2.087194	11.16063	10.69555	4.019918
24008	LOC11962	1.366828	0.936007	2.309812	0.405231	0.685308	0.758867	0.542086	2.189647	0	4.464254	0	1.004979

1635	RCAN3	1637.218	0.935879	0.10533	8.8852	6.38E-19	1.04E-17	1116.156	1208.138	1047.076	2205.341	2089.911	2156.686
12663	LOC11962!	41.9917	0.935788	0.412649	2.267756	0.023344	0.049009	23.30971	30.10765	32.69937	46.87467	72.72976	46.22906
6480	TMEM91	232.9419	0.935671	0.237018	3.947674	7.89E-05	0.000324	202.7403	112.2194	165.5841	310.2657	280.2235	326.6183
16946	LOC10322‹	17.58823	0.935309	0.623636	1.499768	0.133675	0.20971	9.215468	15.32753	11.1317	31.24978	23.53022	15.07469
21228	LOC11962‹	6.421126	0.935272	1.104322	0.846919	0.39704	0.497236	5.96295	2.189647	4.87012	2.232127	19.252	4.019918
24675	POU1F1	0.797721	0.935003	3.083533	0.303225	0.761719	0.820681	0	1.642235	0	0	2.139111	1.004979
5340	SLC25A45	241.9988	0.934898	0.208004	4.494622	6.97E-06	3.47E-05	150.7	189.9519	159.3225	345.9797	250.2759	355.7627
24670	LOC11961¦	0.813224	0.934724	3.064651	0.305002	0.760365	0.819388	0	1.642235	0	2.232127	0	1.004979
20956	LOC11962‚	5.65151	0.934627	1.056719	0.884461	0.376447	0.477565	2.168345	3.284471	6.261582	4.464254	10.69555	7.034856
4182	LOC10324‚	280.0104	0.934402	0.178361	5.23881	1.62E-07	1.03E-06	186.4777	215.1328	174.6286	401.7829	342.2577	359.7826
2462	CHPF2	3104.901	0.934402	0.13012	7.181094	6.92E-13	7.47E-12	2288.689	1865.032	2251.387	3857.115	3640.766	4726.418
5693	LOC10324‹	224.2907	0.934275	0.215725	4.330861	1.49E-05	6.94E-05	158.8313	124.8099	180.8902	285.7123	258.8324	336.6681
12779	ANKMY1	37.89242	0.93414	0.416461	2.243045	0.024894	0.051785	28.18849	29.01283	20.87194	42.41041	55.61688	51.25395
17668	LOC10323ᛈ	14.92669	0.934081	0.671221	1.391615	0.164039	0.246829	7.589209	10.94824	11.82743	29.01765	17.11289	13.06473
19044	CATSPER4	11.08818	0.933867	0.793004	1.177632	0.238943	0.333526	4.878777	9.853413	7.653045	17.85702	19.252	7.034856
22195	LOC11961¦	3.516773	0.933641	1.347573	0.692831	0.488415	0.58502	3.252518	1.642235	2.087194	6.696381	6.417332	1.004979
24462	TRNAF-GA,	0.796833	0.933452	2.775452	0.336324	0.736626	0.800556	0.542086	1.094824	0	0	2.139111	1.004979
24443	LOC10321ᛈ	0.812336	0.933124	2.759855	0.338106	0.735283	0.799685	0.542086	1.094824	0	2.232127	0	1.004979
22981	LOC11962!	2.692129	0.933063	1.644532	0.567373	0.570461	0.659924	2.710432	0	2.782925	2.232127	6.417332	2.009959
3704	S100A2	6101.088	0.933017	0.165571	5.635144	1.75E-08	1.26E-07	4936.238	2887.597	4759.498	8064.675	7963.909	7994.612
19117	LOC10323(	10.58409	0.932003	0.799659	1.165501	0.243816	0.339062	8.131296	4.926706	8.348776	20.08914	14.97377	7.034856
16877	LOC10324‚	17.18737	0.931839	0.617046	1.510161	0.131002	0.206358	11.38381	15.87494	8.348776	17.85702	23.53022	26.12947
8836	LOC10323‚	84.57684	0.931563	0.294662	3.161463	0.00157	0.004723	60.71367	58.02565	54.26705	127.2312	119.7902	87.43321
22396	LOC11962:	2.961914	0.931531	1.415243	0.658213	0.510401	0.605868	2.710432	2.737059	0.695731	4.464254	2.139111	5.024897
7200	ENHO	110.4947	0.931476	0.254033	3.666753	0.000246	0.000907	82.39713	69.5213	77.22618	138.3919	132.6249	162.8067
1673	CROT	1137.177	0.931466	0.105964	8.790416	1.49E-18	2.37E-17	816.9242	761.9972	768.0874	1464.275	1512.351	1499.429
2303	CERT1	2353.587	0.931356	0.125702	7.409265	1.27E-13	1.47E-12	1587.771	1880.36	1390.071	2959.8	3067.485	3236.034
14946	VSIG1	32.67718	0.930418	0.50393	1.846324	0.064845	0.115343	16.80468	26.82318	22.95914	40.17829	64.17332	25.12449
1725	ENOSF1	1148.567	0.929992	0.107463	8.65405	4.97E-18	7.66E-17	827.7659	768.5662	777.1319	1450.883	1512.351	1554.703
2480	TMEM65	2736.566	0.929913	0.129985	7.154018	8.43E-13	9.03E-12	1747.686	2264.095	1639.839	3558.01	3529.533	3680.235
17029	EFCAB5	17.01226	0.929564	0.623895	1.489937	0.136241	0.212795	8.131296	10.94824	16.00182	20.08914	27.80844	19.09461
2813	EML4	6517.876	0.928771	0.139141	6.675038	2.47E-11	2.34E-10	4022.281	5591.812	3855.048	8089.228	8487.991	9060.895
21091	LOC10323ᛈ	5.954609	0.928705	1.071863	0.866441	0.386249	0.486863	3.252518	3.831883	4.87012	8.928508	12.83466	2.009959
23471	CCR2	1.898846	0.92848	1.899599	0.488777	0.625	0.707921	0	1.094824	2.782925	2.232127	4.278221	1.004979
20985	LOC10322‚	5.719544	0.928396	1.053541	0.881215	0.378202	0.479147	2.168345	3.284471	6.261582	11.16063	6.417332	5.024897
23231	LOC11961¦	2.163661	0.928074	1.770913	0.524066	0.600233	0.686836	1.084173	3.284471	0	4.464254	2.139111	2.009959
9680	LOC11962‹	82.3517	0.927782	0.315957	2.936418	0.00332	0.009119	62.33993	42.15071	66.79021	109.3742	89.84265	123.6125
2966	ESRRG	555.0646	0.927721	0.143408	6.469109	9.86E-11	8.84E-10	398.4335	365.1237	381.2608	712.0485	819.2794	654.2416
1535	BIN3	1658.949	0.927278	0.101421	9.142845	6.08E-20	1.05E-18	1135.671	1143.543	1154.914	2131.681	2066.381	2321.503
18566	LOC11962!	14.06566	0.926838	0.747139	1.240515	0.214785	0.307554	9.215468	4.379295	15.30609	15.62489	27.80844	12.05975
23794	LOC11961ᛈ	1.906704	0.926495	2.120056	0.437014	0.662101	0.739748	0	3.831883	0	4.464254	2.139111	1.004979
2143	DOCK5	3927.004	0.926132	0.120315	7.697544	1.39E-14	1.72E-13	2486.008	3176.083	2463.585	4935.233	5078.249	5422.869
8842	NTF3	81.13143	0.926132	0.293225	3.158435	0.001586	0.004769	52.58238	58.02565	57.7457	118.3027	85.56443	114.5677
2274	HAGH	1174.649	0.926092	0.124176	7.457885	8.79E-14	1.03E-12	822.8871	753.7861	858.5325	1448.65	1437.482	1726.555
2749	IRF9	682.78	0.925921	0.136642	6.776237	1.23E-11	1.19E-10	511.7295	464.7526	434.8321	986.6001	821.4185	877.3471
10998	LOC11962‚	62.33117	0.925389	0.35186	2.629988	0.008539	0.02064	42.28274	35.58177	50.09266	93.74933	91.98176	60.29877
21961	LOC10324(	12.00947	0.925272	1.260122	0.734272	0.462783	0.560246	0	0.547412	24.3506	17.85702	19.252	10.04979
23778	LOC11962:	1.592779	0.925152	2.102354	0.440055	0.659897	0.737767	1.626259	1.642235	0	0	4.278221	2.009959
2139	NFKBIZ	6376.741	0.925076	0.119945	7.712506	1.23E-14	1.53E-13	3850.439	5171.947	4177.867	8207.531	8092.256	8760.406
1753	MFHAS1	2424.169	0.925009	0.107708	8.588108	8.84E-18	1.34E-16	1740.639	1637.856	1644.709	2957.568	3024.702	3539.538
3251	POMK	461.2539	0.924769	0.151645	6.098264	1.07E-09	8.77E-09	332.841	325.71	298.4688	529.0141	618.203	663.2864
14630	GPR162	32.04073	0.924724	0.486324	1.901457	0.057242	0.104018	19.51511	16.96977	29.22072	51.33892	47.06043	28.13943
2615	UBE2J1	2499.19	0.924696	0.132903	6.957652	3.46E-12	3.52E-11	1529.768	2093.85	1549.394	3281.227	3270.7	3270.203
6859	LRRC24	480.4913	0.924459	0.24376	3.792496	0.000149	0.000578	403.3123	195.426	397.2626	676.3345	526.2212	684.391
5728	LOC11962ᛈ	162.2156	0.924335	0.214448	4.310293	1.63E-05	7.57E-05	117.6327	120.4306	97.40239	207.5878	218.1893	212.0507
4749	SUGCT	247.232	0.924126	0.190712	4.845664	1.26E-06	7.06E-06	162.0838	159.2968	190.6304	368.301	284.5017	318.5785
21008	LOC10322‚	5.691733	0.923993	1.052198	0.878155	0.379859	0.480632	2.168345	2.737059	6.957314	6.696381	8.556443	7.034856
20699	LOC11962‹	7.00287	0.922445	1.000377	0.922098	0.356478	0.457824	8.673382	3.284471	2.782925	8.928508	4.278221	14.06971
21401	LOC10323‚	5.149714	0.921959	1.129295	0.816402	0.41427	0.51462	3.252518	2.189647	5.565851	6.696381	2.139111	11.05477
22857	LOC11961ᛈ	2.945298	0.921842	1.576024	0.584916	0.558604	0.649734	0.542086	1.642235	4.174388	0	4.278221	7.034856
2408	LOC10324‚	755.3746	0.921311	0.126838	7.263694	3.77E-13	4.16E-12	527.45	545.7696	490.4906	959.8146	1078.112	930.611
10137	MAGIX	75.21082	0.921076	0.325388	2.830704	0.004645	0.012181	54.75072	37.77142	63.31155	93.74933	111.2338	90.44815
10800	CACNA1A	101.1624	0.921006	0.344873	2.670562	0.007572	0.01864	61.79785	62.95236	83.48776	120.5349	196.7982	81.40334
4804	SLC9B2	328.1698	0.920919	0.191397	4.811573	1.50E-06	8.29E-06	206.5349	273.1585	200.3706	374.9973	489.8563	424.1013
12512	MTMR7	50.24998	0.920838	0.400538	2.299002	0.021505	0.045693	29.81475	41.05589	32.00364	84.82083	70.59065	43.21412
2327	CNNM4	1083.811	0.920615	0.124869	7.372661	1.67E-13	1.91E-12	728.022	828.7815	693.6442	1350.437	1358.335	1543.648
13489	LOC11962!	36.1697	0.919995	0.43621	2.109064	0.034939	0.06886	28.73058	24.08612	21.56767	42.41041	62.03421	38.18922
17313	EDA	22.13546	0.919761	0.63594	1.446301	0.148093	0.227415	19.51511	13.13788	12.52316	20.08914	53.47777	14.06971
18165	LOC10321!	12.20708	0.919628	0.702253	1.309539	0.190352	0.278585	7.589209	9.306001	8.348776	20.08914	12.83466	15.07469
2814	B3GNT5	877.3382	0.91953	0.137762	6.67477	2.48E-11	2.34E-10	576.2378	688.0967	557.9766	1024.546	1185.067	1232.105
22225	CCNI2	3.804663	0.919429	1.337083	0.687638	0.491681	0.588124	0.542086	4.379295	2.782925	6.696381	6.417332	2.009959
23179	LOC11962‹	2.39547	0.918627	1.72396	0.532859	0.594131	0.681347	0.542086	3.831883	0.695731	0	4.278221	5.024897
20193	LOC10322:	7.8135	0.918529	0.920261	0.998118	0.318222	0.418954	4.336691	3.831883	8.348776	4.464254	12.83466	13.06473
2449	SIRT2	1099.26	0.91841	0.127598	7.19768	6.12E-13	6.65E-12	712.3015	847.3935	724.9521	1444.186	1306.997	1559.728

18023	LOC10322	19.15114	0.918357	0.686038	1.338638	0.180688	0.266526	6.505036	24.63353	8.348776	20.08914	34.22577	21.10457
1241	HMGCR	10265.82	0.917956	0.091532	10.02883	1.14E-23	2.44E-22	7411.947	7046.832	6863.39	12979.82	12779.05	14513.91
8212	ADAMTS15	97.7935	0.917829	0.274584	3.342618	0.00083	0.002687	69.92914	58.57306	75.13899	107.1421	143.3204	132.6573
21088	LOC119624	5.393892	0.917776	1.058442	0.867101	0.385887	0.486475	4.336691	3.831883	2.782925	6.696381	10.69555	4.019918
3393	GAMT	921.4739	0.91759	0.154228	5.94957	2.69E-09	2.11E-08	687.9076	515.1145	713.1247	1249.991	1065.277	1297.428
2879	FAM160B2	1118.465	0.917585	0.139509	6.577266	4.79E-11	4.42E-10	821.2608	696.8552	809.8313	1388.383	1292.023	1702.435
5736	LOC11962	189.6741	0.917031	0.212939	4.306551	1.66E-05	7.69E-05	112.754	138.4952	141.9292	285.7123	231.024	228.1303
11355	LOC10323	66.86507	0.916737	0.360017	2.546371	0.010885	0.02548	53.66655	36.12918	50.78839	71.42806	70.59065	118.5876
15645	LOC11962	44.58581	0.916503	0.531107	1.725646	0.084411	0.143437	24.39389	13.13788	54.96278	55.80317	77.00798	42.20914
1448	TMEM51	2426.747	0.916303	0.097814	9.367763	7.41E-21	1.36E-19	1695.104	1755.002	1595.312	3020.068	3159.466	3335.527
14592	TMPRSS13	36.21845	0.915693	0.479847	1.908302	0.056352	0.102667	18.43094	25.18094	31.30791	33.4819	72.72976	36.17926
1871	HIBADH	2893.018	0.915304	0.110946	8.249989	1.58E-16	2.25E-15	1871.824	2283.802	1858.994	3734.348	3769.113	3840.027
15089	LOC10323	29.03259	0.914593	0.502991	1.81831	0.069017	0.121599	28.73058	15.32753	16.00182	46.87467	32.08666	35.17428
2806	CLN8	726.3055	0.91449	0.136761	6.686791	2.28E-11	2.16E-10	534.4972	474.0586	505.7967	917.4042	857.7834	1068.293
2128	ECM1	1513.859	0.914152	0.118125	7.738825	1.00E-14	1.25E-13	996.8968	1114.53	1042.206	1915.165	1805.409	2208.945
18320	LOC119624	12.16251	0.913896	0.714347	1.279345	0.200776	0.291339	7.589209	10.40082	7.653045	13.39276	12.83466	21.10457
22494	LOC11961	3.005167	0.913858	1.428401	0.639777	0.522318	0.617287	1.626259	1.642235	2.782925	6.696381	4.278221	1.004979
20948	CPSF4L	5.982523	0.913661	1.032199	0.88516	0.376071	0.477269	3.252518	2.737059	6.261582	8.928508	10.69555	4.019918
10403	LOC119624	67.00434	0.913627	0.33033	2.765802	0.005678	0.014511	46.61943	42.69812	50.09266	66.96381	111.2338	84.41828
2776	LOC10322	588.5816	0.913452	0.135644	6.734164	1.65E-11	1.58E-10	382.1709	430.8131	411.873	825.887	705.9065	774.8392
21292	LBHD1	5.121964	0.913326	1.0939	0.834927	0.403759	0.50413	4.878777	2.189647	3.478657	4.464254	10.69555	5.024897
21047	LOC11961	6.225091	0.913305	1.046091	0.873065	0.382628	0.483302	6.505036	3.284471	2.782925	8.928508	12.83466	3.014938
6774	DCT	136.0838	0.913251	0.238696	3.826002	0.00013	0.000511	102.9964	98.53413	81.40057	198.6593	154.016	180.8963
18537	LOC10323	13.05758	0.913118	0.73279	1.246084	0.212734	0.305094	5.420864	6.568942	15.30609	17.85702	17.11289	16.07967
22610	LOC11962	2.687679	0.912772	1.465131	0.622997	0.533286	0.626997	2.710432	2.189647	0.695731	2.232127	4.278221	4.019918
10348	LOC10321	67.33589	0.912535	0.328606	2.776988	0.005487	0.014095	49.32986	39.96106	50.78839	71.42806	109.0946	83.4133
1896	SLC20A2	1244.659	0.912332	0.11118	8.205932	2.29E-16	3.21E-15	854.3281	892.8287	846.7051	1649.542	1490.96	1733.59
21297	LOC11962	5.123904	0.912321	1.093867	0.834033	0.404263	0.504593	4.878777	3.284471	2.782925	4.464254	4.278221	11.05477
3004	AUTS2	1223.122	0.91191	0.142254	6.41043	1.45E-10	1.28E-09	769.7626	1018.186	758.3472	1484.364	1709.149	1598.922
4030	HRK	307.5279	0.91163	0.170411	5.349582	8.82E-08	5.82E-07	201.114	218.9647	221.9383	348.2118	427.8221	427.1163
8390	LOC10323	89.64716	0.911595	0.277543	3.284513	0.001022	0.003237	56.37698	64.59459	66.79021	109.3742	109.0946	131.6523
20463	LOC11962	7.92093	0.91152	0.957558	0.951921	0.341137	0.443196	6.505036	3.284471	6.261582	15.62489	12.83466	3.014938
24698	GUCA1ANE	0.79914	0.910107	3.039842	0.299393	0.76464	0.823061	1.084173	0	0.695731	0	0	3.014938
2678	SELENOM	832.3735	0.909883	0.132625	6.86058	6.86E-12	6.81E-11	575.6957	549.6015	611.5479	1169.635	939.0696	1148.692
11878	LOC11962	71.38119	0.909652	0.374153	2.43123	0.015048	0.033679	33.60935	68.97389	45.91827	71.42806	121.9293	86.42823
4453	RLBP1	270.4543	0.909548	0.180498	5.03909	4.68E-07	2.79E-06	191.3565	169.6977	204.545	314.7299	361.5097	380.8872
19018	LOC10323	14.21087	0.909496	0.769863	1.181374	0.237454	0.331934	5.420864	7.116354	16.69755	22.32127	25.66933	8.039836
2693	LOC10324	540.4693	0.909486	0.132849	6.845998	7.59E-12	7.50E-11	358.8612	395.2313	372.912	725.4413	673.8199	716.5504
13949	LOC10323	54.01173	0.909253	0.44964	2.022181	0.043158	0.082253	41.19856	17.51718	53.57132	87.05295	68.45154	56.27885
5314	B4GALNT3	211.749	0.908722	0.201453	4.510829	6.46E-06	3.23E-05	141.4845	131.3788	170.4542	267.8552	258.8324	300.4889
14320	LOC10324	40.66667	0.908293	0.463929	1.957828	0.05025	0.093283	21.68345	42.15071	20.17621	51.33892	68.45154	40.19918
3670	LOC10324	427.1304	0.908285	0.159913	5.679868	1.35E-08	9.76E-08	281.8849	321.3307	290.8157	502.2286	528.3603	638.162
20483	LOC11962	7.067648	0.908206	0.956139	0.949869	0.342179	0.444116	7.589209	2.189647	4.87012	11.16063	8.556443	8.039836
8262	ZBED6CL	93.02638	0.907927	0.27304	3.325251	0.000883	0.002843	67.21871	55.83601	70.9646	116.0706	130.4858	117.5826
3547	TBC1D7	1586.499	0.907649	0.156289	5.807512	6.34E-09	4.75E-08	1249.509	821.1177	1239.793	2104.896	2036.433	2067.243
3747	PNPLA7	442.5518	0.907102	0.162089	5.596311	2.19E-08	1.55E-07	341.5144	273.7059	310.9919	555.7996	524.0821	649.2167
19560	CORIN	10.57885	0.90682	0.832395	1.089411	0.275973	0.375025	6.505036	8.758589	6.261582	13.39276	23.53022	5.024897
4988	SLC3A1	197.9955	0.906793	0.193116	4.695584	2.66E-06	1.42E-05	140.9425	141.7797	129.406	256.6946	275.9453	243.205
7648	CAPN8	110.1907	0.906576	0.258264	3.510273	0.000448	0.001556	75.89209	66.23683	87.66215	138.3919	158.2942	134.6672
23028	LOC11962	2.730932	0.905429	1.624307	0.557425	0.577237	0.6664	1.626259	1.094824	2.782925	4.464254	6.417332	0
17176	LOC11962	16.57615	0.905376	0.615391	1.471221	0.141231	0.218598	11.38381	10.94824	11.82743	24.5534	25.66933	15.07469
22792	TRNAR-AC	3.270453	0.905232	1.524492	0.593792	0.552651	0.644622	3.252518	0	3.478657	4.464254	6.417332	2.009959
2379	RARS2	743.2187	0.904896	0.123907	7.303046	2.81E-13	3.14E-12	519.8608	523.3257	507.1882	1060.26	924.0958	924.5811
2290	CALCOCO1	1761.316	0.904741	0.121675	7.435727	1.04E-13	1.21E-12	1143.802	1417.797	1118.04	2229.895	2301.683	2356.677
5161	EFNA1	205.2556	0.904687	0.196807	4.596832	4.29E-06	2.21E-05	148.5317	134.1159	147.4951	276.7837	233.1631	291.444
10887	LOC10322	60.94521	0.903635	0.340647	2.652697	0.007985	0.0195	38.48813	48.17224	39.65669	93.74933	81.28621	64.31869
11255	LOC11962	17853.73	0.903271	0.351587	2.569123	0.010196	0.024083	9987.399	11622.65	15711.01	16042.3	32069.55	21689.47
14763	LOC10322	28.46755	0.902803	0.480403	1.879263	0.060209	0.108423	15.17842	23.53871	20.87194	31.24978	42.78221	37.18424
24709	FAM106B	0.800916	0.902716	3.038027	0.297139	0.76636	0.824545	0	1.094824	0.695731	0	0	3.014938
5774	ZNF688	200.6224	0.902203	0.210466	4.286692	1.81E-05	8.35E-05	153.4104	119.3358	146.1036	241.0697	301.6146	242.2001
24064	TRNAS-AG	1.334935	0.90216	2.285604	0.394714	0.693054	0.765584	1.084173	1.642235	0	0	4.278221	1.004979
2534	TFDP2	1573.774	0.901854	0.127456	7.075779	1.49E-12	1.56E-11	1066.284	1269.995	955.2392	1962.04	2102.746	2086.337
23560	LOC11961	2.758185	0.9011	1.895995	0.475265	0.634598	0.716078	4.878777	0	0.695731	6.696381	4.278221	0
1969	DPP7	4651.969	0.901061	0.111951	8.04874	8.37E-16	1.13E-14	3412.976	2943.433	3381.95	6194.152	5375.585	6603.72
20475	LOC10322	6.5144	0.901005	0.94783	0.950598	0.341808	0.443821	4.878777	4.379295	4.174388	8.928508	10.69555	6.029877
14799	LOC11962	1075.657	0.901002	0.481012	1.873138	0.061049	0.109669	952.4458	321.8781	976.8068	1334.812	1394.7	1473.3
16575	LOC11962	20.37185	0.900924	0.576812	1.561901	0.118311	0.189751	10.84173	18.06459	13.2189	26.78552	34.22577	19.09461
2170	IP6K3	1208.647	0.900757	0.117595	7.65982	1.86E-14	2.28E-13	853.2439	876.4063	802.874	1580.346	1422.509	1716.505
1884	RAI1	1911.417	0.90067	0.109483	8.226579	1.93E-16	2.72E-15	1287.997	1370.719	1346.936	2377.215	2320.935	2764.699
2023	TENM4	5203.529	0.900271	0.113577	7.926495	2.25E-15	2.96E-14	3408.639	4007.054	3481.44	6446.383	6256.899	7620.759
1369	ACADSB	4733.958	0.899662	0.093151	9.658091	4.54E-22	8.82E-21	3188.01	3578.978	3144.706	6042.368	6241.925	6207.758
3565	NEK11	383.9956	0.899171	0.155328	5.788844	7.09E-09	5.29E-08	273.7536	261.1154	268.5523	457.586	562.5861	480.3802
14167	GALNT6	40.24516	0.898473	0.453298	1.982081	0.04747	0.089068	24.93597	25.18094	33.39511	44.64254	79.14709	34.1693
14458	LOC11961	40.2754	0.898462	0.464851	1.932794	0.053262	0.097936	17.88885	31.74989	34.09084	46.87467	74.86887	36.17926

24753	LOC11962	1.077978	0.898388	3.11019	0.288853	0.772694	0.82975	0	2.189647	0	0	4.278221	0
24754	LOC11962	1.077978	0.898388	3.11019	0.288853	0.772694	0.82975	0	2.189647	0	0	4.278221	0
4127	PTPRH	395.23	0.89806	0.170352	5.271797	1.35E-07	8.70E-07	258.0331	324.0678	246.9846	520.0856	468.4652	553.7437
15266	LOC10324	26.07904	0.897732	0.502885	1.785165	0.074235	0.129259	22.76763	13.13788	18.78475	31.24978	36.36488	34.1693
6833	OCEL1	145.2818	0.897623	0.235925	3.804695	0.000142	0.000552	101.9122	87.03848	116.8829	165.1774	194.6591	206.0208
3201	BCL2L11	611.0279	0.897373	0.145662	6.160666	7.24E-10	6.02E-09	443.4267	456.5415	381.2608	850.4404	697.3501	837.1479
2529	LOC11962	675.7765	0.896956	0.126641	7.082685	1.41E-12	1.49E-11	476.4939	500.8818	437.615	879.458	906.9829	853.2276
19257	CLPSL2	12.51812	0.896569	0.78387	1.143773	0.252718	0.348886	14.09425	6.02153	5.565851	17.85702	23.53022	8.039836
17118	F7	21.53578	0.8965	0.606382	1.478442	0.13929	0.216323	11.9259	16.42235	16.00182	29.01765	42.78221	13.06473
12405	ZNF575	47.39461	0.89637	0.386434	2.319596	0.020363	0.043639	29.27266	37.77142	31.30791	69.19594	70.59065	46.22906
2635	TJP2	1347.769	0.895925	0.129459	6.920552	4.50E-12	4.54E-11	883.6008	1086.065	857.8368	1810.255	1610.75	1838.107
12677	LOC11961	45.20844	0.895876	0.395492	2.265216	0.023499	0.049281	39.5723	29.56024	25.04633	69.19594	55.61688	52.25893
15329	LOC10324	25.00288	0.895413	0.504822	1.773721	0.076109	0.131996	20.59928	17.51718	13.91463	33.4819	36.36488	28.13943
23431	ZNF396	1.877119	0.895266	1.808962	0.494906	0.620667	0.704213	1.084173	2.189647	0.695731	0	4.278221	3.014938
18185	LOC11961	265.4865	0.895221	0.686117	1.304764	0.191973	0.280634	92.15468	372.24	92.53227	383.9258	280.2235	371.8424
19637	LOC11962	12.79574	0.89522	0.829276	1.079519	0.280356	0.379545	3.252518	8.758589	14.61036	11.16063	29.94755	9.044815
3741	ASAP1	4457.293	0.895188	0.159746	5.603829	2.10E-08	1.49E-07	2599.846	4112.705	2638.909	5544.603	5859.024	5988.673
16278	LOC10324	20.89726	0.894892	0.555149	1.611984	0.106965	0.174695	18.43094	11.49565	13.91463	22.32127	32.08666	27.13445
2543	GNPTG	1232.573	0.894823	0.126689	7.063127	1.63E-12	1.70E-11	870.5907	794.842	926.0185	1540.168	1458.873	1804.943
1707	PTGFR	2424.586	0.894791	0.102817	8.702766	3.24E-18	5.04E-17	1718.414	1771.425	1600.182	3212.031	2904.912	3340.552
3607	LOC10322	344.9126	0.894721	0.156088	5.732163	9.92E-09	7.31E-08	240.6863	256.7361	226.8084	453.1218	427.8221	464.3005
1324	NUCB1	8332.592	0.894695	0.091426	9.785997	1.29E-22	2.60E-21	5870.253	5929.017	5691.083	10816.89	10034.57	11653.74
11957	LOC10323	80.29755	0.89455	0.370714	2.413048	0.01582	0.035173	42.82482	73.90059	50.09266	111.6063	139.0422	64.31869
22078	NEUROG2	3.816187	0.894491	1.255149	0.712657	0.476058	0.57324	1.084173	2.737059	4.174388	4.464254	6.417332	4.019918
1791	TRIM16	4161.284	0.894482	0.10552	8.476939	2.31E-17	3.43E-16	3118.623	2677.939	2941.552	5178.535	5131.726	5919.329
28982	LOC11962	0.566904	0.894269	3.900188	0.229289	0.818645	NA	0	0	1.391463	0	0	2.009959
20836	LOC10323	9.087151	0.894216	0.994235	0.899401	0.368439	0.470098	5.420864	5.474118	7.653045	17.85702	17.11289	1.004979
1113	LRRC17	6531.446	0.894212	0.085459	10.46369	1.27E-25	3.03E-24	4684.71	4301.015	4724.016	8486.547	8492.269	8500.116
22498	LOC10321	3.274891	0.893979	1.39818	0.639388	0.522571	0.617501	0.542086	2.737059	3.478657	4.464254	6.417332	2.009959
12129	MST1R	48.62296	0.893683	0.375606	2.37931	0.017345	0.038018	40.11439	27.918	34.09084	53.57105	72.72976	63.31371
21682	LRRC74B	4.615554	0.893347	1.145598	0.779809	0.435504	0.533985	4.878777	3.284471	1.391463	6.696381	6.417332	5.024897
16074	SIX2	36.89676	0.893335	0.540571	1.652578	0.098417	0.162773	19.51511	29.01283	27.82925	44.64254	81.28621	19.09461
15152	EVPL	32.83098	0.893313	0.49476	1.80555	0.070989	0.124553	13.55216	22.44388	32.69937	51.33892	42.78221	34.1693
2123	TRIM2	1388.167	0.893307	0.115295	7.748004	9.33E-15	1.17E-13	926.9677	1075.117	914.191	1805.791	1715.567	1891.371
2176	HCFC1R1	3553.72	0.893177	0.116677	7.655138	1.93E-14	2.36E-13	2214.965	2881.576	2366.182	4689.699	4468.602	4701.294
5391	LOC11961	289.8339	0.893146	0.199881	4.468396	7.88E-06	3.89E-05	227.1342	156.0124	226.8084	350.4439	378.6226	399.9818
20078	HERC5	8.962565	0.893092	0.878935	1.016107	0.309579	0.409909	7.047123	7.116354	4.174388	11.16063	19.252	5.024897
21397	LOC11962	7.782755	0.892608	1.092227	0.817237	0.413793	0.514123	13.01007	2.189647	1.391463	4.464254	8.556443	17.08465
20988	MKRN3	6.492079	0.892463	1.012995	0.881013	0.378311	0.479197	2.710432	7.116354	4.174388	4.464254	6.417332	14.06971
13612	RUNDC3A	41.0034	0.892057	0.427378	2.087279	0.036863	0.071995	25.47806	24.08612	37.56949	37.94616	55.61688	65.32367
19195	CUNH1orf	10.06741	0.891412	0.771945	1.154761	0.248188	0.34374	5.96295	8.758589	6.261582	13.39276	14.97377	11.05477
9639	HOXA2	98.56026	0.890975	0.301906	2.951167	0.003166	0.008731	50.95612	88.1333	67.48594	149.5525	117.6511	117.5826
16864	LOC11962	18.26649	0.890464	0.588582	1.512897	0.130306	0.205419	11.38381	11.49565	16.00182	22.32127	19.252	29.1444
1945	AHR	2607.521	0.890295	0.10985	8.104622	5.29E-16	7.23E-15	1729.256	2064.29	1689.236	3390.601	3315.622	3456.124
8097	F8	145.894	0.889924	0.26314	3.381942	0.00072	0.002363	98.65972	121.5254	84.1835	216.5163	211.772	142.7071
7077	COL21A1	150.5431	0.889678	0.240253	3.703087	0.000213	0.0008	108.4173	119.3358	88.35788	165.1774	231.024	190.9461
22502	TRNAC-GC	3.256726	0.889573	1.392051	0.639038	0.522798	0.61766	2.168345	1.094824	3.478657	2.232127	8.556443	2.009959
11770	PKNOX2	72.75843	0.889181	0.362245	2.454644	0.014102	0.031853	33.60935	68.42648	51.48412	71.42806	109.0946	102.5079
8169	SPSB4	95.53485	0.889046	0.264794	3.357505	0.000786	0.00256	72.63957	67.33165	61.22436	118.3027	124.0684	129.6424
21877	LOC10324	4.364822	0.888991	1.187746	0.748469	0.454177	0.551918	2.710432	2.737059	3.478657	6.696381	8.556443	2.009959
4991	DUSP8	215.8745	0.888869	0.189347	4.694397	2.67E-06	1.42E-05	143.6529	146.7064	163.4969	305.8014	265.2497	270.3395
2418	TRIM6	1969.104	0.88849	0.122579	7.248325	4.22E-13	4.64E-12	1302.091	1602.274	1239.793	2450.875	2575.489	2644.101
19678	LOC10322	11.18183	0.888405	0.827133	1.074078	0.282788	0.382047	4.878777	8.758589	10.43597	15.62489	4.278221	23.11453
23148	LOC11962	2.41522	0.888224	1.653389	0.537214	0.59112	0.67891	1.626259	2.737059	0.695731	0	6.417332	3.014938
7720	PPARGC1A	145.1052	0.88801	0.254721	3.486205	0.00049	0.001687	77.51835	128.0944	99.48959	198.6593	183.9635	182.9063
4656	LOC11961	376.8278	0.887894	0.180663	4.914646	8.89E-07	5.08E-06	317.6626	226.6285	247.6804	466.5145	541.195	461.2856
24249	PDX1	1.627806	0.887794	2.409425	0.368467	0.712525	0.781165	0	0	3.478657	0	4.278221	2.009959
16885	CACNA1C	34.13705	0.887621	0.588174	1.509113	0.13127	0.206681	15.17842	22.9913	32.69937	53.57105	66.31243	14.06971
19709	LOC10324	11.95734	0.887503	0.831156	1.067794	0.285614	0.385257	14.09425	4.379295	6.261582	11.16063	27.80844	8.039836
6643	KSR2	163.8983	0.887429	0.228697	3.880379	0.000104	0.000417	94.32303	136.853	113.4042	218.7484	216.0502	204.0108
1853	DNAJB2	2359.767	0.886652	0.106918	8.292819	1.11E-16	1.59E-15	1645.232	1739.675	1589.746	2924.086	2877.104	3382.761
22499	LOC11962	3.277424	0.886329	1.386768	0.639133	0.522737	0.617615	3.794605	2.189647	0.695731	6.696381	4.278221	2.009959
19137	LOC11962	12.18484	0.886231	0.761778	1.163371	0.244679	0.339906	10.84173	5.474118	9.740239	6.696381	19.252	21.10457
23488	LOC10321	2.758315	0.886137	1.821648	0.486448	0.626649	0.709219	0	2.189647	3.478657	4.464254	6.417332	0
18678	LOC11962	13.11869	0.886013	0.719492	1.231443	0.218157	0.310493	12.46799	7.663765	6.957314	22.32127	19.252	10.04979
1817	CD46	9579.667	0.885711	0.105324	8.409389	4.12E-17	6.03E-16	7273.173	5844.716	7068.631	12140.54	12306.3	12844.64
24347	LOC11962	1.091706	0.885342	2.49569	0.354748	0.722778	0.789217	1.084173	1.094824	0	2.232127	2.139111	0
9801	LOC11962	103.665	0.885006	0.304687	2.904642	0.003677	0.009973	74.26583	66.78424	80.00911	104.91	109.0946	186.9262
14325	ANO7	46.86378	0.884746	0.452054	1.95717	0.050327	0.0934	30.35684	23.53871	44.52681	42.41041	94.12087	46.22906
1795	MRAS	1360.829	0.884709	0.104446	8.470466	2.44E-17	3.62E-16	956.7824	998.4791	914.8868	1743.291	1713.428	1838.107
22484	TRNAE-CU	3.278312	0.884581	1.379273	0.641339	0.521302	0.616386	3.252518	2.737059	0.695731	6.696381	4.278221	2.009959
3533	SPNS1	1341.302	0.884407	0.151887	5.82278	5.79E-09	4.36E-08	1070.078	758.7128	1002.549	1683.024	1563.69	1969.76
13276	LOC10323	44.14499	0.884379	0.410896	2.152318	0.031372	0.062818	26.02015	26.27577	41.74388	46.87467	55.61688	68.3386
21225	PSMA8	5.966818	0.883688	1.042522	0.847645	0.396636	0.496806	4.336691	3.831883	4.174388	4.464254	14.97377	4.019918

1173	NCOA4	16950.5	0.883546	0.086116	10.25996	1.07E-24	2.42E-23	12316.74	11670.27	11766.9	21656.1	20864.89	23428.08
18683	GRM7	16.40375	0.883374	0.717426	1.231309	0.218207	0.310522	6.505036	10.94824	16.69755	17.85702	36.36488	10.04979
21741	LOC11962!	4.347176	0.883144	1.145929	0.770679	0.440897	0.539106	3.252518	3.831883	2.087194	4.464254	6.417332	6.029877
3809	ATG16L2	674.1185	0.883128	0.159671	5.530925	3.19E-08	2.22E-07	523.1133	386.4727	515.5369	819.1906	823.5576	976.84
2561	TCEAL9	2521.468	0.882734	0.125529	7.032095	2.03E-12	2.11E-11	1572.593	2086.186	1658.624	3412.922	3292.091	3106.392
2187	LOC11962!	1343.089	0.882636	0.115625	7.633626	2.28E-14	2.77E-13	910.163	1040.63	884.9703	1696.417	1679.202	1847.152
24590	LOC11962(	1.077091	0.88249	2.77292	0.318253	0.750293	0.811165	0.542086	1.642235	0	0	4.278221	0
4001	ATG4A	430.2898	0.882427	0.164214	5.373648	7.72E-08	5.13E-07	260.7435	353.628	294.2944	540.1747	554.0297	578.8682
3628	LOC11962:	777.585	0.882282	0.154429	5.71318	1.11E-08	8.13E-08	593.5846	443.4036	606.6778	986.6001	934.7914	1100.453
2001	GPCPD1	1691.215	0.881939	0.110509	7.980669	1.46E-15	1.93E-14	1297.755	1147.923	1125.693	2049.093	2220.397	2306.428
21715	LOC10323(	5.262573	0.881788	1.136545	0.77585	0.437838	0.536031	2.168345	3.284471	5.565851	13.39276	2.139111	5.024897
14067	LOC10323:	35.18116	0.881714	0.440804	2.00024	0.045474	0.085941	27.6464	18.612	27.82925	40.17829	55.61688	41.20416
1335	ACO1	5411.176	0.881526	0.090342	9.757601	1.71E-22	3.41E-21	3803.278	3978.589	3644.241	6841.469	6776.703	7422.778
20642	GSTM3	7.636622	0.88066	0.948012	0.928955	0.352912	0.454468	3.252518	4.379295	8.348776	6.696381	17.11289	6.029877
3633	ANKRD12	3532.373	0.880567	0.154228	5.709516	1.13E-08	8.29E-08	2076.733	3215.497	2166.507	4450.861	4804.443	4480.198
1855	PPARD	1580.568	0.880521	0.106233	8.288584	1.15E-16	1.64E-15	1087.425	1175.293	1078.384	2011.146	1942.312	2188.845
22777	OASL	2.72382	0.880513	1.476772	0.596242	0.551014	0.643136	3.252518	1.642235	0.695731	4.464254	4.278221	2.009959
1771	ACADVL	4897.209	0.8802	0.103175	8.531128	1.45E-17	2.18E-16	3651.494	3316.768	3381.254	6078.082	5916.78	7038.876
18314	LOC10322:	14.91326	0.879966	0.687488	1.279973	0.200555	0.291128	13.01007	8.758589	9.740239	8.928508	29.94755	19.09461
7755	DRC3	159.6111	0.879546	0.252977	3.476783	0.000507	0.00174	123.5957	80.46954	134.2762	189.7308	207.4937	222.1005
2114	PCYT1A	2234.742	0.879278	0.113072	7.776235	7.47E-15	9.40E-14	1479.896	1710.662	1536.175	2758.909	2720.949	3201.865
13106	LOC10323	40.70162	0.87909	0.40205	2.186519	0.028778	0.058374	26.56223	32.2973	26.43779	55.80317	59.8951	43.21412
1457	DNAJC6	4816.888	0.879085	0.093996	9.352384	8.57E-21	1.56E-19	3371.235	3597.043	3214.279	6006.654	6094.326	6617.79
2573	HPCAL1	1329.495	0.878624	0.125122	7.022158	2.18E-12	2.26E-11	994.1864	822.76	996.9831	1665.167	1623.585	1874.287
23119	LOC11962	2.46078	0.878451	1.62506	0.540565	0.588807	0.677081	1.084173	0.547412	3.478657	2.232127	6.417332	1.004979
14713	LOC11961{	34.57328	0.8784	0.465212	1.888173	0.059003	0.106612	31.9831	18.612	22.2634	33.4819	59.8951	41.20416
1443	INSIG1	4004.578	0.878381	0.093549	9.389565	6.02E-21	1.11E-19	2976.596	2650.568	2841.367	5087.017	5044.023	5427.894
17368	LOC10322	18.66643	0.878206	0.611412	1.436357	0.150901	0.23098	12.46799	10.40082	16.00182	31.24978	27.80844	14.06971
2398	VPS28	7145.61	0.878195	0.120731	7.273986	3.49E-13	3.87E-12	5468.025	4312.51	5330.694	9124.935	8391.731	10245.77
19790	LOC11962	14.19026	0.878127	0.832535	1.054763	0.291534	0.391633	10.29964	6.02153	13.2189	8.928508	40.6431	6.029877
2101	SIDT2	1778.882	0.878023	0.112653	7.794074	6.49E-15	8.21E-14	1312.391	1203.759	1249.534	2270.073	2089.911	2547.623
17924	LOC10322!	17.88082	0.877889	0.647444	1.355929	0.175122	0.259742	15.7205	6.568942	15.30609	33.4819	17.11289	19.09461
2071	CRELD1	1316.983	0.877319	0.111762	7.849868	4.16E-15	5.35E-14	954.6141	886.8071	948.2819	1624.988	1630.002	1857.202
15074	NOD2	29.94051	0.877187	0.481745	1.820852	0.068629	0.121037	22.22554	17.51718	22.95914	53.57105	34.22577	29.1444
6004	CCDC183	165.3346	0.876881	0.210688	4.161997	3.15E-05	0.00014	120.8853	122.0728	107.1426	194.195	224.6066	223.1054
2255	DCTN6	2363.256	0.876593	0.116874	7.500311	6.37E-14	7.51E-13	1486.401	1886.381	1625.228	3002.211	3240.753	2938.56
20315	LOC10324(	13.29766	0.876371	0.898275	0.975615	0.329255	0.430877	4.878777	14.23271	8.348776	26.78552	23.53022	2.009959
4131	BCL3	439.1021	0.875943	0.166175	5.271194	1.36E-07	8.72E-07	259.1173	356.9125	313.7748	589.2815	532.6386	582.8881
23227	ADAM30	2.757427	0.875667	1.667878	0.525019	0.59957	0.686248	0.542086	1.642235	3.478657	4.464254	6.417332	0
16268	LOC10324:	24.41769	0.875338	0.542277	1.61419	0.106486	0.174019	11.38381	16.96977	22.95914	35.71403	36.36488	23.11453
1405	TSC22D1	4892.867	0.874988	0.091916	9.51944	1.74E-21	3.29E-20	3626.016	3260.932	3475.178	6314.687	6051.544	6628.845
7495	CFAP45	127.4141	0.874147	0.245379	3.562439	0.000367	0.001303	91.6126	102.9134	75.13899	174.1059	151.8769	168.8366
3701	PCMT1	2382.292	0.873995	0.154996	5.638827	1.71E-08	1.23E-07	1917.902	1241.53	1888.215	2977.657	3052.511	3215.934
23870	BANF2	1.378352	0.873508	2.044464	0.427255	0.669194	0.74532	1.084173	0.547412	1.391463	2.232127	0	3.014938
8360	LOC11962	111.677	0.873467	0.265128	3.294513	0.000986	0.003135	75.89209	81.01695	77.92191	176.338	143.3204	115.5726
21513	LOC11962(	6.059346	0.873368	1.088966	0.802015	0.422544	0.522165	1.626259	2.737059	8.348776	8.928508	10.69555	4.019918
1870	MTARC2	1235.091	0.87322	0.105816	8.252215	1.55E-16	2.21E-15	912.8734	840.2771	865.4898	1582.578	1555.133	1654.196
16967	LOC10322:	18.89148	0.873061	0.583246	1.496902	0.134419	0.210616	11.38381	14.23271	13.91463	26.78552	29.94755	17.08465
2079	R3HCC1	1016.665	0.872794	0.111303	7.841628	4.45E-15	5.69E-14	742.6583	688.6441	724.2564	1352.669	1247.102	1344.663
20748	LOC11962	8.315263	0.872747	0.958322	0.910704	0.362451	0.464419	7.047123	5.474118	4.87012	22.32127	2.139111	8.039836
3662	BATF2	360.9318	0.872511	0.153445	5.686141	1.30E-08	9.43E-08	257.491	245.7879	263.6822	455.3539	442.7959	500.4798
22844	FEM1A	2.470058	0.871881	1.487044	0.586318	0.557662	0.648986	2.168345	1.642235	1.391463	4.464254	2.139111	3.014938
23875	TRNAT-UG	1.37924	0.871405	2.044301	0.42626	0.669918	0.745876	0.542086	1.094824	1.391463	2.232127	0	3.014938
23876	MT3	1.37924	0.871405	2.044301	0.42626	0.669918	0.745876	0.542086	1.094824	1.391463	2.232127	0	3.014938
2497	PTK2	6817.676	0.871399	0.122046	7.139925	9.34E-13	9.94E-12	4269.472	5708.41	4481.206	8602.617	8487.991	9356.359
3457	POLB	566.0428	0.870641	0.147653	5.896539	3.71E-09	2.86E-08	417.9486	347.0591	434.1364	763.3874	759.3843	674.3412
17336	LRRC66	16.94849	0.869734	0.603166	1.441948	0.149317	0.22898	13.01007	13.6853	9.044508	22.32127	23.53022	20.09959
6661	SLC22A4	209.0669	0.869552	0.224531	3.872757	0.000108	0.000429	177.2622	119.3358	148.8865	258.9267	237.4413	312.5486
17043	LOC10323:	26.7003	0.869474	0.584653	1.487162	0.136972	0.213647	15.17842	9.306001	32.00364	37.94616	40.6431	25.12449
20489	LOC11962!	8.831472	0.869335	0.916563	0.948472	0.342889	0.444903	7.589209	7.116354	3.478657	20.08914	10.69555	4.019918
13699	SCN4B	43.5491	0.869196	0.420252	2.068274	0.038614	0.074937	21.14137	36.67659	34.09084	53.57105	70.59065	45.22408
1760	HSD17B11	1499.869	0.869172	0.101387	8.572777	1.01E-17	1.53E-16	1092.304	1006.143	1085.341	1986.593	1895.252	1933.58
3982	MXD1	513.2834	0.868991	0.161268	5.388487	7.11E-08	4.74E-07	311.6997	426.9812	351.3443	613.8349	699.4892	676.3512
11456	LOC11962	60.73161	0.868942	0.344053	2.525601	0.01155	0.026803	37.94605	47.07742	43.13534	100.4457	68.45154	67.33362
23233	LOC10321(	1.917341	0.868884	1.657917	0.524082	0.600222	0.686836	1.084173	1.642235	1.391463	2.232127	2.139111	3.014938
1831	RAB5A	6208.919	0.868532	0.103829	8.364984	6.01E-17	8.73E-16	4237.489	4949.15	3995.585	7915.122	8141.455	8014.711
22820	LOC11962	2.989994	0.867747	1.471906	0.58954	0.555499	0.647123	4.336691	0.547412	1.391463	2.232127	6.417332	3.014938
17678	IL4I1	24.13448	0.867645	0.624378	1.389615	0.164646	0.24761	18.43094	9.306001	22.95914	24.5534	53.47777	16.07967
9177	TLE6	85.80839	0.867616	0.283381	3.061665	0.002201	0.006376	66.13454	59.66789	57.04997	93.74933	117.6511	120.5975
4717	PRADC1	454.2115	0.86689	0.177968	4.87104	1.11E-06	6.26E-06	335.0094	251.8094	379.8693	591.5136	554.0297	613.0375
1735	RAB29	2335.032	0.866832	0.100504	8.624856	6.42E-18	9.83E-17	1735.761	1614.317	1615.488	2950.872	2855.713	3238.044
14608	RDH16	29.5695	0.866816	0.455052	1.904872	0.056797	0.103356	22.22554	21.89647	18.78475	37.94616	36.36488	40.19918
3553	NAGPA	812.6773	0.866626	0.149356	5.802438	6.54E-09	4.89E-08	640.7461	481.175	608.0692	1029.011	958.3216	1158.741
17134	LOC11962:	20.29183	0.866413	0.587227	1.475432	0.140097	0.217373	13.01007	10.94824	18.78475	24.5534	36.36488	18.08963

3965	CDK18	477.5507	0.866364	0.160401	5.401238	6.62E-08	4.44E-07	298.1475	344.322	375.6949	542.4069	620.3421	684.391
23315	LOC10322:	1.918229	0.866316	1.69378	0.511469	0.609023	0.69444	0.542086	2.189647	1.391463	2.232127	2.139111	3.014938
5960	SFRP1	166.8374	0.866058	0.207092	4.181995	2.89E-05	0.000129	125.222	119.3358	109.2298	218.7484	222.4675	206.0208
16420	LOC11961!	21.378	0.865703	0.545814	1.586077	0.112722	0.182504	17.88885	10.94824	16.69755	26.78552	27.80844	28.13943
1566	ATP6V1A	9556.282	0.865666	0.095604	9.054679	1.37E-19	2.33E-18	7233.058	6230.641	6857.128	12022.24	11897.73	13096.89
1894	DSCC1	1157.966	0.865398	0.10545	8.206722	2.27E-16	3.19E-15	813.6716	847.9409	800.7868	1444.186	1533.742	1507.469
2646	CCNG2	940.0876	0.865211	0.125327	6.903651	5.07E-12	5.09E-11	635.8673	745.0275	617.8095	1160.706	1262.075	1219.04
2276	TMEM127	1635.041	0.865064	0.116039	7.454928	8.99E-14	1.05E-12	1100.435	1287.513	1091.603	2078.11	1989.373	2263.214
21044	LOC10324	6.3503	0.86466	0.988984	0.874292	0.38196	0.482532	3.794605	2.737059	6.957314	11.16063	6.417332	7.034856
23938	LOC11961!	1.39066	0.86462	2.071227	0.417443	0.676354	0.751112	1.084173	1.094824	0.695731	4.464254	0	1.004979
18940	MN1	18.96002	0.864188	0.722074	1.196813	0.231379	0.324774	8.673382	10.94824	20.17621	17.85702	47.06043	9.044815
11446	KIF5C	83.11152	0.864184	0.341996	2.526887	0.011508	0.026729	48.24569	74.99542	52.87558	80.35657	149.7377	92.45811
21855	LOC11962:	4.984256	0.864117	1.149307	0.751859	0.452136	0.54999	2.710432	2.737059	4.87012	11.16063	6.417332	2.009959
17962	LOC10322(	21.16865	0.86376	0.640243	1.349113	0.177301	0.262413	8.131296	9.853413	27.13352	24.5534	34.22577	23.11453
13201	LOC10323	41.12961	0.863328	0.398021	2.169049	0.030079	0.060575	34.15144	25.18094	28.52499	53.57105	47.06043	58.28881
17391	KIF17	31.08963	0.862585	0.603024	1.430434	0.152593	0.233236	24.39389	24.63353	16.00182	35.71403	72.72976	13.06473
21152	LOC11962:	6.568381	0.862004	1.004468	0.85817	0.390799	0.491177	2.168345	4.379295	7.653045	4.464254	10.69555	10.04979
6408	LOC1032	170.7828	0.861981	0.216814	3.975677	7.02E-05	0.000291	133.8953	123.1677	107.8384	198.6593	213.9111	247.2249
10353	LOC11962!	89.54834	0.861624	0.310421	2.775664	0.005509	0.014146	82.39713	51.45671	55.65851	127.2312	124.0684	96.47803
4648	NR1H3	470.3022	0.861397	0.175151	4.918036	8.74E-07	5.00E-06	397.8914	273.1585	331.8639	542.4069	635.3159	641.1769
2025	ITPR3	13889.46	0.861234	0.108664	7.925677	2.27E-15	2.98E-14	9386.225	10632.38	9575.351	17120.41	16327.83	20294.56
3299	LOC10322!	563.5428	0.861105	0.142485	6.043483	1.51E-09	1.22E-08	391.9284	370.5978	436.9193	801.3336	693.0719	687.406
19729	LOC11961!	12.33449	0.861062	0.80915	1.064157	0.287258	0.387082	4.878777	9.853413	11.1317	11.16063	29.94755	7.034856
18377	LOC11962!	13.15805	0.86094	0.679126	1.267717	0.204899	0.296443	9.215468	8.211177	10.43597	15.62489	21.39111	14.06971
21344	LOC11962:	5.51844	0.860687	1.042994	0.825209	0.409253	0.509745	4.878777	3.284471	3.478657	11.16063	4.278221	6.029877
1141	SCARB2	9173.351	0.860668	0.08318	10.34709	4.31E-25	1.00E-23	6468.717	6739.187	6341.591	11528.94	11617.51	12344.16
22183	LOC10321(	3.827937	0.86059	1.239314	0.694408	0.487426	0.584151	4.336691	1.642235	2.087194	4.464254	6.417332	4.019918
22373	LOC11962	4.115307	0.86031	1.298599	0.662491	0.507657	0.603229	2.710432	1.642235	4.174388	4.464254	10.69555	1.004979
22879	LOC11962(	2.764043	0.859812	1.47658	0.5823	0.560365	0.651134	3.252518	1.094824	1.391463	6.696381	2.139111	2.009959
19690	LOC10324(	10.75669	0.859466	0.802248	1.071322	0.284025	0.383484	3.794605	6.568942	12.52316	15.62489	14.97377	11.05477
20610	LOC11962!	6.883064	0.859362	0.918803	0.935306	0.34963	0.450979	4.878777	4.926706	4.87012	11.16063	6.417332	9.044815
7096	ECHDC2	131.0816	0.859276	0.232338	3.698391	0.000217	0.000813	102.9964	89.77554	86.27069	162.9453	179.6853	164.8166
5237	PPP1R16A	1139.104	0.859271	0.188419	4.560425	5.11E-06	2.59E-05	992.0181	551.2437	887.0575	1473.204	1341.222	1589.878
15851	LOC11962:	25.18136	0.859112	0.507982	1.691225	0.090794	0.152278	18.43094	21.89647	13.2189	29.01765	36.36488	32.15934
12775	LOC10322	47.76567	0.859053	0.382765	2.244336	0.024811	0.051632	29.27266	38.31883	33.39511	62.49956	74.86887	48.23901
20637	LOC11962(	9.56654	0.858609	0.923264	0.929972	0.352386	0.45395	5.96295	3.831883	10.43597	4.464254	25.66933	7.034856
3440	ZNF572	421.5277	0.85856	0.145294	5.909143	3.44E-09	2.66E-08	318.7468	297.2446	283.8584	522.3177	541.195	565.8034
20740	PKP1	7.711198	0.856771	0.939779	0.911672	0.361941	0.463944	2.168345	7.116354	6.957314	11.16063	12.83466	6.029877
23936	LOC10322!	1.363737	0.856743	2.050331	0.417856	0.676053	0.750871	0.542086	1.094824	1.391463	0	2.139111	3.014938
2585	PITPNM2	830.9699	0.855863	0.122181	7.004857	2.47E-12	2.54E-11	622.3152	562.1919	592.7631	1051.332	1007.521	1149.697
6175	ZNF425	199.6895	0.855789	0.209314	4.088546	4.34E-05	0.000187	158.8313	137.9478	131.4932	220.9806	252.4151	296.4689
5363	SLC35E4	396.1992	0.85575	0.190905	4.482593	7.37E-06	3.66E-05	320.9151	215.1328	311.6877	520.0856	455.6306	553.7437
2868	ADAMTS1(	1241.831	0.855362	0.129832	6.588205	4.45E-11	4.13E-10	918.2943	754.3335	980.9812	1667.399	1505.934	1624.047
20337	LOC1032	10.09611	0.855133	0.880522	0.971166	0.331466	0.4333	7.047123	6.568942	7.653045	4.464254	27.80844	7.034856
13180	DDN	47.45617	0.855116	0.393341	2.173981	0.029707	0.05992	24.93597	36.12918	41.04815	49.10679	64.17332	69.34358
22784	CXCL6	3.795512	0.854988	1.436316	0.595265	0.551666	0.64371	3.252518	3.284471	1.391463	0	12.83466	2.009959
2195	CDK2AP2	1236.725	0.854929	0.112202	7.619523	2.55E-14	3.08E-13	848.9073	949.7595	844.6179	1616.06	1503.795	1657.211
14368	IL37	36.49354	0.854755	0.438572	1.948952	0.051301	0.094922	22.22554	27.37059	27.82925	40.17829	64.17332	37.18424
15462	C1QTNF2	42.02567	0.854274	0.487912	1.750877	0.079967	0.137486	40.11439	26.27577	22.2634	44.64254	87.70354	31.15436
6097	MAPK8IP2	195.6185	0.85414	0.207192	4.122449	3.75E-05	0.000163	152.8684	116.5987	148.1908	267.8552	245.9977	242.2001
8121	AJM1	121.3057	0.853937	0.253218	3.372338	0.000745	0.00244	92.15468	71.16354	96.70666	162.9453	139.0422	165.8216
2458	AIFM2	1375.7	0.853282	0.118749	7.185601	6.69E-13	7.24E-12	1028.88	931.6949	983.7642	1823.648	1563.69	1922.526
17681	TSHR	17.26582	0.852487	0.613907	1.388626	0.164947	0.248012	11.9259	10.94824	13.91463	15.62489	32.08666	19.09461
23204	SDR42E2	2.453136	0.852227	1.610705	0.529102	0.596735	0.683684	1.084173	3.284471	0.695731	2.232127	6.417332	1.004979
18646	LOC10322(	21.62095	0.851655	0.690352	1.233653	0.217332	0.309867	23.30971	6.02153	16.69755	13.39276	49.19955	21.10457
3555	RBKS	860.8105	0.851483	0.146772	5.801412	6.58E-09	4.92E-08	678.15	518.9464	646.3344	1176.331	973.2954	1171.806
17853	LOC11962	17.93834	0.851291	0.622035	1.368557	0.171138	0.254829	8.673382	12.59047	16.69755	26.78552	27.80844	15.07469
10498	EXD3	94.50852	0.850018	0.309666	2.744951	0.006052	0.015326	88.36008	47.62483	66.79021	113.8385	119.7902	130.6473
16569	LOC10324:	22.6536	0.849286	0.543576	1.562405	0.118193	0.18964	18.97302	13.6853	15.30609	40.17829	25.66933	22.10955
20645	SHISAL2B	8.05344	0.849166	0.914105	0.928959	0.352911	0.454468	7.047123	3.831883	6.261582	17.85702	4.278221	9.044815
2616	TACC1	3631.248	0.849045	0.12203	6.957666	3.46E-12	3.52E-11	2408.49	3026.093	2345.31	4504.432	4462.185	5040.977
21697	LOC11962(	5.758414	0.849045	1.091527	0.777851	0.436657	0.535029	3.794605	2.737059	5.565851	4.464254	14.97377	3.014938
9963	NEK10	96.43447	0.849002	0.295612	2.872018	0.004079	0.010883	75.89209	57.47824	71.66033	127.2312	151.8769	94.46807
2692	LPCAT3	2404.234	0.848659	0.123952	6.846683	7.56E-12	7.46E-11	1605.118	1999.695	1547.307	3044.621	2926.303	3302.363
1842	UBR3	4130.468	0.848471	0.101853	8.330352	8.06E-17	1.16E-15	2891.489	3199.075	2762.054	5093.714	5123.17	5713.308
20236	LOC11962	8.807683	0.848127	0.856814	0.989862	0.322242	0.423344	5.96295	4.926706	7.653045	11.16063	17.11289	6.029877
22453	KCTD8	3.590034	0.847974	1.306525	0.64903	0.516319	0.611337	1.084173	4.379295	2.087194	6.696381	4.278221	3.014938
16371	TRPC6	23.45126	0.847365	0.531604	1.593977	0.110941	0.180158	17.88885	13.6853	18.08902	35.71403	34.22577	21.10457
19398	LAMP5	13.99018	0.847286	0.757792	1.118099	0.263525	0.361161	9.757555	4.926706	15.30609	8.928508	29.94755	15.07469
10644	ZNF860	79.83749	0.84717	0.312868	2.707758	0.006774	0.016919	43.909	66.23683	61.92009	102.6778	87.70354	116.5776
3699	LOC10323:	5326.795	0.84698	0.150107	5.642521	1.68E-08	1.20E-07	4299.829	2839.425	4282.227	7118.253	6267.594	7153.444
1972	FNIP1	2856.645	0.846894	0.105327	8.040642	8.94E-16	1.20E-14	2122.81	2167.203	1835.339	3475.422	3724.192	3814.902
13794	SMIM41	46.86947	0.846485	0.413396	2.047638	0.040596	0.078239	26.56223	39.41365	33.39511	62.49956	79.14709	40.19918
11932	LOC10322!	58.68037	0.846418	0.349958	2.418629	0.015579	0.034709	40.65648	38.31883	48.00546	75.89232	57.75599	91.45313

4191	KHK	701.0111	0.846375	0.161801	5.230959	1.69E-07	1.07E-06	563.2277	412.7485	530.843	812.4942	855.6443	1031.109
2720	CUNH8orf8	939.664	0.846321	0.124218	6.813162	9.55E-12	9.33E-11	673.8134	645.3985	700.6015	1120.528	1152.981	1344.663
22020	IL17RB	6.394594	0.846192	1.171417	0.722366	0.470069	0.56752	1.084173	6.02153	6.261582	11.16063	12.83466	1.004979
23133	TRNAS-CGA	2.212213	0.84559	1.567029	0.539613	0.589464	0.677448	1.626259	1.642235	1.391463	4.464254	2.139111	2.009959
2642	LOC10323:	1573.033	0.845429	0.122269	6.914511	4.69E-12	4.72E-11	1201.263	1005.596	1172.307	1935.254	1886.696	2237.084
21065	LOC11962{	6.352405	0.845139	0.970829	0.870534	0.384009	0.484579	5.420864	2.737059	5.565851	8.928508	6.417332	9.044815
10222	PCLO	130.862	0.844067	0.300362	2.810162	0.004952	0.012878	72.63957	136.3055	71.66033	178.5702	156.1551	169.8415
4059	TCP11L2	470.5048	0.843261	0.158325	5.326139	1.00E-07	6.57E-07	326.336	343.7746	340.9084	687.4951	498.4128	626.1022
6784	LOC1032‹	164.8217	0.8431	0.220509	3.823425	0.000132	0.000516	105.7068	131.9262	116.1871	187.4987	239.5804	208.0308
7224	LOC10324{	204.2414	0.843054	0.230365	3.659645	0.000253	0.000929	131.727	182.2881	122.4487	281.248	286.6408	221.0955
5766	DLL1	262.9365	0.842562	0.196358	4.290954	1.78E-05	8.20E-05	187.5619	178.4563	201.0664	359.3724	265.2497	385.9121
20911	LOC10323(	8.016175	0.842474	0.946929	0.889691	0.373632	0.474955	2.168345	7.116354	7.653045	11.16063	14.97377	5.024897
16815	FGF11	25.34533	0.842439	0.553203	1.522839	0.127799	0.202052	16.80468	14.78012	22.2634	26.78552	51.33866	20.09959
21038	LOC11961{	6.576937	0.842356	0.962529	0.87515	0.381493	0.482079	5.420864	3.831883	4.87012	4.464254	12.83466	8.039836
14815	SPTBN2	53.82436	0.842346	0.450504	1.869786	0.061514	0.110384	28.18849	42.15071	43.83108	80.35657	96.25998	32.15934
2096	LOC1032‹	5824.383	0.84224	0.10795	7.802149	6.09E-15	7.72E-14	4467.876	3686.819	4361.54	7566.91	6932.858	7930.293
11616	RPA4	60.8348	0.842065	0.338115	2.490466	0.012758	0.029195	49.32986	41.6033	38.96096	73.66019	94.12087	67.33362
21821	LOC10323;	4.681843	0.842047	1.112367	0.756987	0.449058	0.547097	2.168345	3.831883	4.174388	4.464254	6.417332	7.034856
20028	LOC10324;	10.30215	0.84199	0.823013	1.023058	0.30628	0.406554	7.047123	7.663765	6.957314	24.5534	8.556443	7.034856
8499	LOC10323:	114.8592	0.84181	0.258506	3.256439	0.001128	0.003529	68.84497	93.60742	84.1835	165.1774	132.6249	144.717
20010	DPYS	9.083135	0.841374	0.818751	1.027631	0.304123	0.404054	8.131296	4.926706	6.261582	11.16063	14.97377	9.044815
3273	RHOC	10788.63	0.841372	0.138641	6.068705	1.29E-09	1.05E-08	8490.699	5945.44	8752.301	14314.63	12980.12	14248.6
3229	LOC10322{	626.8933	0.841066	0.137139	6.132935	8.63E-10	7.10E-09	449.9317	477.3431	416.7431	874.9938	810.7229	731.6251
8670	PCDH10	126.4931	0.840646	0.261802	3.211002	0.001323	0.004056	72.09749	109.4824	89.74935	151.7846	186.1026	149.7419
15213	LOC11962:	33.49003	0.840354	0.468445	1.793921	0.072826	0.127264	27.6464	18.612	26.43779	26.78552	49.19955	52.25893
15997	FILIP1	27.08841	0.840335	0.504382	1.666067	0.0957	0.159041	15.7205	22.9913	18.78475	42.41041	38.50399	24.11951
3826	FIGN	486.3977	0.840259	0.15224	5.519308	3.40E-08	2.36E-07	330.1306	398.5158	317.2535	587.0494	650.2896	635.147
5268	MFSD3	779.5892	0.83997	0.185012	4.540091	5.62E-06	2.84E-05	630.9885	412.2011	636.5942	966.511	868.4789	1162.761
8580	LOC11962{	101.4197	0.839775	0.259664	3.234086	0.00122	0.003781	81.31296	66.78424	70.26887	124.9991	130.4858	134.6672
24671	LOC11961{	0.820666	0.839515	2.755528	0.304666	0.760621	0.81963	1.084173	0	0.695731	0	2.139111	1.004979
24660	FAM237A	0.836168	0.839496	2.740596	0.306319	0.759362	0.818639	1.084173	0	0.695731	2.232127	0	1.004979
10864	LOC11962;	70.5111	0.839482	0.315994	2.656641	0.007892	0.019313	42.82482	52.55153	57.04997	75.89232	96.25998	98.48799
2938	AKIRIN2	644.3778	0.839395	0.129131	6.500332	8.01E-11	7.25E-10	449.9317	471.869	466.8357	754.4589	842.8096	880.362
22128	PROM1	4.742906	0.838914	1.191527	0.704066	0.481392	0.578327	1.626259	1.642235	6.957314	8.928508	4.278221	5.024897
2475	ZNF441	948.6993	0.8388	0.117164	7.159217	8.11E-13	8.72E-12	653.2141	700.1397	688.7741	1120.528	1298.44	1231.1
23368	LOC1032‹	3.302438	0.838772	1.664886	0.503802	0.614401	0.698987	0	3.831883	3.478657	4.464254	0	8.039836
1739	RELN	26465.27	0.83867	0.097291	8.620182	6.68E-18	1.02E-16	18513.33	20314.45	18124.5	33100.21	31618.19	37120.93
20285	LOC10323{	9.654182	0.838101	0.854705	0.980574	0.326803	0.428299	9.215468	5.474118	5.565851	13.39276	19.252	5.024897
21323	LOC11962‹	6.063084	0.838039	1.011022	0.828902	0.40716	0.507637	4.336691	6.568942	2.087194	8.928508	6.417332	8.039836
23604	EPYC	1.657722	0.837615	1.791228	0.467621	0.640056	0.720841	1.626259	0.547412	1.391463	2.232127	2.139111	2.009959
18189	LOC11962‹	15.96499	0.837107	0.642491	1.302908	0.192606	0.281497	11.9259	8.211177	14.61036	13.39276	23.53022	24.11951
21985	LOC1032‹	4.706459	0.837069	1.146533	0.730087	0.465337	0.562701	2.168345	4.926706	2.782925	8.928508	6.417332	3.014938
18202	LOC11962:	23.41549	0.836991	0.643178	1.301335	0.193144	0.282097	10.84173	21.89647	16.69755	44.64254	36.36488	10.04979
5936	SLC17A9	220.6535	0.836924	0.199633	4.192322	2.76E-05	0.000124	174.5518	130.8314	169.7585	290.1765	280.2235	278.3793
20305	BANK1	10.25688	0.83683	0.855782	0.977854	0.328147	0.429617	4.336691	10.94824	6.261582	17.85702	17.11289	5.024897
19559	ACTG2	11.25581	0.836812	0.768031	1.089555	0.275909	0.375025	10.29964	8.758589	4.87012	11.16063	21.39111	11.05477
24056	LOC11962!	1.358767	0.836743	2.109323	0.396688	0.691598	0.764305	1.626259	0.547412	0.695731	0	4.278221	1.004979
7145	SORL1	143.331	0.836565	0.227177	3.682433	0.000231	0.00086	102.9964	109.4824	94.61947	196.4272	194.6591	161.8017
13990	LOC10324:	41.89268	0.83624	0.415635	2.011959	0.044224	0.084039	38.48813	24.08612	27.13352	49.10679	66.31243	46.22906
24511	LOC11962‹	0.821553	0.835733	2.534849	0.329697	0.741629	0.804346	0.542086	0.547412	0.695731	0	2.139111	1.004979
24513	LOC11962:	0.821553	0.835733	2.534849	0.329697	0.741629	0.804346	0.542086	0.547412	0.695731	0	2.139111	1.004979
24497	LOC10324‹	0.837056	0.835722	2.522551	0.3313	0.740418	0.803494	0.542086	0.547412	0.695731	2.232127	0	1.004979
3646	ALDH5A1	467.3946	0.835643	0.146594	5.700394	1.20E-08	8.72E-08	338.804	363.4814	303.3389	636.1562	579.699	582.8881
14937	LYL1	36.49123	0.835534	0.452283	1.847369	0.064694	0.115142	31.9831	16.96977	29.22072	49.10679	53.47777	38.18922
1857	ATP1B1	13947.58	0.834804	0.100754	8.285527	1.18E-16	1.68E-15	9453.986	11007.9	9605.963	17283.36	16982.4	19351.88
5645	SLC14A1	213.3281	0.834448	0.191854	4.3494	1.37E-05	6.43E-05	162.6259	162.5813	134.2762	272.3195	273.8062	274.3594
22684	LOC1032‹	3.018264	0.833983	1.367358	0.609923	0.541913	0.635106	2.168345	2.189647	2.087194	2.232127	6.417332	3.014938
6857	ZFP28	197.2418	0.833677	0.219781	3.793208	0.000149	0.000577	126.8482	176.2666	122.4487	243.3018	250.2759	264.3096
1496	OSTM1	2819.269	0.833577	0.09005	9.256794	2.11E-20	3.74E-19	2000.841	2108.63	1969.616	3558.01	3702.801	3575.717
4814	ADCK1	352.4164	0.833525	0.17346	4.805291	1.55E-06	8.53E-06	263.454	231.0078	268.5523	450.8897	385.0399	515.5545
1638	MANBA	2361.923	0.833484	0.093874	8.878774	6.76E-19	1.10E-17	1664.747	1788.942	1641.23	2999.979	2988.338	3088.302
20414	GABRA1	8.872626	0.833467	0.868983	0.959129	0.337494	0.439494	5.96295	3.831883	9.044508	13.39276	14.97377	6.029877
11757	VSIG10L	78.3454	0.833425	0.339135	2.457503	0.013991	0.031636	42.28274	60.76271	64.70302	131.6955	96.25998	74.36848
19697	TEX45	10.79463	0.833292	0.778366	1.070566	0.284365	0.383806	7.047123	8.758589	6.957314	17.85702	17.11289	7.034856
22182	LOC11962(	4.460567	0.832857	1.199189	0.694517	0.487358	0.584101	3.794605	2.737059	2.782925	11.16063	4.278221	2.009959
16604	LOC11962:	22.87187	0.832583	0.534876	1.556591	0.119568	0.191442	15.17842	15.87494	18.08902	22.32127	40.6431	25.12449
24063	LOC1032‹	1.360542	0.832379	2.108936	0.394691	0.693071	0.765584	0.542086	1.642235	0.695731	0	4.278221	1.004979
24681	LOC11962(	0.822441	0.832312	2.754111	0.302207	0.762494	0.821316	0	1.094824	0.695731	0	2.139111	1.004979
18982	LOC11962!	15.3847	0.832017	0.700583	1.187607	0.234988	0.329098	5.96295	16.96977	10.43597	11.16063	25.66933	22.10955
3133	PAQR7	1018.865	0.831749	0.133299	6.239717	4.38E-10	3.72E-09	777.3519	677.1484	747.9112	1354.901	1118.755	1437.121
20445	LOC10323;	8.000175	0.831686	0.870676	0.955218	0.339467	0.441415	6.505036	7.116354	3.478657	11.16063	10.69555	9.044815
7039	LOC1032!	309.3857	0.831631	0.22373	3.717114	0.000202	0.000761	272.6694	157.6546	236.5487	372.7652	459.9088	356.7677
20430	LOC1032‹	8.596145	0.830941	0.867916	0.957399	0.338366	0.440307	6.505036	6.02153	5.565851	15.62489	12.83466	5.024897
18802	LOC1032‹	15.91947	0.830894	0.682554	1.217332	0.223478	0.315986	11.9259	8.758589	13.2189	35.71403	12.83466	13.06473

20289	LOC11962:	9.393839	0.830834	0.847915	0.979855	0.327158	0.42868	11.38381	3.831883	4.87012	13.39276	12.83466	10.04979
10037	LOC11962:	116.4581	0.8308	0.291078	2.854215	0.004314	0.011428	105.1648	59.12048	88.35788	140.624	132.6249	172.8565
19221	LOC10324	12.20432	0.830517	0.721789	1.150636	0.249882	0.345618	10.84173	8.211177	6.957314	22.32127	12.83466	12.05975
16173	LOC11962!	38.31373	0.82996	0.508575	1.631933	0.102694	0.168807	36.86187	11.49565	34.09084	58.0353	49.19955	40.19918
14378	ITGAM	38.13005	0.829776	0.425873	1.948413	0.051366	0.094977	24.39389	32.84471	24.3506	49.10679	59.8951	38.18922
2021	PTEN	6776.637	0.829578	0.104532	7.936121	2.09E-15	2.74E-14	4621.286	5529.407	4490.25	8591.457	8573.556	8853.869
16797	NR2E1	23.65103	0.8293	0.543398	1.526136	0.126976	0.200968	21.14137	14.23271	16.00182	17.85702	38.50399	34.1693
22622	LOC10322(	4.973686	0.828997	1.334694	0.621114	0.534525	0.628137	4.336691	3.284471	2.782925	4.464254	14.97377	0
2220	PROCR	1076.39	0.828973	0.109603	7.563412	3.93E-14	4.70E-13	765.426	822.2125	738.171	1383.919	1364.753	1383.857
4250	TMEM59	5255.564	0.828913	0.159786	5.18764	2.13E-07	1.33E-06	4266.22	2679.033	4413.72	6557.989	6823.763	6792.656
3501	ZMYND10	511.5838	0.828447	0.141554	5.852516	4.84E-09	3.68E-08	396.2651	332.8264	377.7821	656.2453	631.0376	675.3462
11954	LOC11962	196.5193	0.828438	0.343264	2.413414	0.015804	0.035147	81.31296	238.6716	104.3597	294.6408	213.9111	246.22
15915	CUNH7orf(	33.78555	0.828304	0.492423	1.682097	0.09255	0.154599	31.44101	20.25424	21.56767	22.32127	59.8951	47.23404
6539	DGLUCY	160.2002	0.828005	0.211001	3.924169	8.70E-05	0.000354	123.0536	106.7453	116.8829	209.8199	194.6591	210.0407
21733	LOC11962:	6.924102	0.827965	1.072431	0.772045	0.440088	0.538357	0.542086	8.211177	6.261582	8.928508	8.556443	9.044815
21191	PPP1R42	6.62716	0.827482	0.971311	0.851923	0.394257	0.494612	4.878777	4.926706	4.174388	8.928508	12.83466	4.019918
29144	LOC11962:	0.537214	0.827482	3.980645	0.207876	0.835325	NA	1.084173	0	0	0	2.139111	0
29349	LOC10323:	0.537214	0.827482	3.980645	0.207876	0.835325	NA	1.084173	0	0	0	2.139111	0
2224	FAM219A	2141.522	0.827481	0.109458	7.559811	4.04E-14	4.83E-13	1488.027	1663.037	1483.299	2752.213	2504.899	2957.655
3593	LOC10324!	502.6355	0.827395	0.143807	5.753508	8.74E-09	6.47E-08	370.7871	343.7746	375.6949	589.2815	626.7594	709.5155
3655	PEDS1	1480.898	0.827376	0.145332	5.693003	1.25E-08	9.08E-08	934.5569	1307.219	962.1965	1787.934	1867.444	2026.039
3166	ACVR1B	1326.105	0.826821	0.13334	6.200868	5.62E-10	4.72E-09	882.5166	1101.393	887.0575	1700.881	1531.603	1853.182
1718	ATP13A3	17210.34	0.826768	0.095196	8.684915	3.79E-18	5.87E-17	12131.35	13129.67	11973.54	20888.24	21048.85	24090.36
26729	LOC11961!	0.552717	0.826563	3.94086	0.209742	0.833869	NA	1.084173	0	0	2.232127	0	0
27647	TRNAV-UA	0.552717	0.826563	3.94086	0.209742	0.833869	NA	1.084173	0	0	2.232127	0	0
29447	LOC11962:	0.552717	0.826563	3.94086	0.209742	0.833869	NA	1.084173	0	0	2.232127	0	0
1930	FGFR1	4757.98	0.825863	0.101526	8.134514	4.14E-16	5.70E-15	3493.205	3465.664	3343.685	5848.173	5621.583	6775.572
16141	TRNAS-AG.	26.79619	0.825394	0.503555	1.639134	0.101185	0.166637	22.22554	13.6853	22.2634	26.78552	40.6431	35.17428
23717	TRNAS-UG	2.461997	0.825389	1.826918	0.451793	0.651418	0.730241	3.252518	0	2.087194	0	6.417332	3.014938
22238	LOC11962:	4.194497	0.825032	1.203	0.685812	0.492832	0.589168	2.710432	2.189647	4.174388	8.928508	2.139111	5.024897
3897	ABHD14A	546.9578	0.824744	0.151395	5.447634	5.10E-08	3.48E-07	368.6187	406.1796	412.5687	720.977	592.5337	780.869
19498	ACTL6B	16.27595	0.824702	0.748964	1.101124	0.270843	0.369287	5.96295	10.40082	18.78475	8.928508	38.50399	15.07469
4462	LOC11962(	15720.95	0.824633	0.163792	5.034623	4.79E-07	2.85E-06	12658.8	9483.362	11902.57	20073.52	15151.32	25056.15
2539	FUT10	1028.434	0.82456	0.116679	7.066907	1.58E-12	1.66E-11	713.3857	793.1997	717.299	1397.311	1311.275	1238.135
2367	KCTD17	1024.914	0.824109	0.112633	7.316746	2.54E-13	2.85E-12	745.3688	720.3939	756.9557	1308.026	1227.85	1390.892
5758	LOC10324(	355.4902	0.823994	0.191781	4.296535	1.73E-05	8.01E-05	295.9792	203.6372	272.7267	412.9435	432.1004	515.5545
2084	ACAP2	6233.666	0.823698	0.105294	7.822807	5.17E-15	6.59E-14	4369.758	5049.874	4084.639	7685.213	7876.205	8336.305
10369	LOC11962:	80.3304	0.823259	0.297088	2.771091	0.005587	0.014324	58.54533	60.76271	55.65851	107.1421	81.28621	118.5876
20979	LOC11962:	7.706	0.822917	0.933426	0.881609	0.377988	0.478994	9.215468	3.831883	3.478657	6.696381	14.97377	8.039836
21012	NPFFR2	7.517267	0.822841	0.936685	0.87846	0.379694	0.480632	4.336691	8.211177	3.478657	15.62489	6.417332	7.034856
3190	GINS4	578.562	0.822533	0.133181	6.176039	6.57E-10	5.48E-09	396.2651	450.5199	408.3943	729.9055	714.463	771.8242
16826	LOC10324(	22.95821	0.822487	0.540962	1.520415	0.128407	0.202882	20.0572	12.59047	16.69755	26.78552	38.50399	23.11453
16531	FAM166B	24.89543	0.822476	0.523934	1.569807	0.11646	0.18729	17.34676	15.32753	20.87194	26.78552	44.92132	24.11951
11271	LOC10323:	67.7311	0.822411	0.320661	2.564739	0.010325	0.024354	44.45108	49.81448	51.48412	100.4457	89.84265	70.34856
23131	LOC11962!	2.491583	0.822333	1.523732	0.539684	0.589415	0.677429	2.168345	1.642235	1.391463	4.464254	4.278221	1.004979
11883	LOC10323(	58.9122	0.821726	0.338318	2.428856	0.015147	0.033886	46.61943	37.224	43.83108	64.73168	87.70354	73.3635
7250	S1PR3	160.0701	0.821513	0.224872	3.653241	0.000259	0.00095	109.5014	132.4737	104.3597	232.1412	183.9635	197.981
1610	MAF1	5927.169	0.821433	0.091742	8.953732	3.44E-19	5.67E-18	4427.761	3973.115	4454.072	7546.821	7309.341	7851.905
23858	LOC11962:	1.643994	0.821348	1.914499	0.429015	0.667913	0.74423	0.542086	1.642235	1.391463	0	4.278221	2.009959
19066	LOC11962!	17.45445	0.821279	0.699063	1.174829	0.240063	0.334736	17.34676	9.306001	10.43597	20.08914	38.50399	9.044815
22347	LOC11962!	4.120074	0.821052	1.230604	0.667194	0.504648	0.600352	2.710432	3.284471	2.782925	2.232127	10.69555	3.014938
1941	PLEKHA1	2152.754	0.821007	0.101179	8.114427	4.88E-16	6.69E-15	1625.717	1613.223	1430.424	2694.177	2720.949	2832.032
3079	RASL11A	755.7912	0.820965	0.13024	6.303471	2.91E-10	2.51E-09	588.1637	495.4077	557.9766	946.4218	917.6785	1029.099
20453	LOC11962:	8.838448	0.820868	0.860951	0.953443	0.340366	0.44241	9.215468	7.116354	2.782925	11.16063	10.69555	12.05975
1285	LAMP2	5644.075	0.819887	0.082834	9.89797	4.25E-23	8.79E-22	3937.173	4192.627	4118.034	7196.377	7103.987	7316.251
3284	UNC5A	842.4481	0.81984	0.135391	6.055331	1.40E-09	1.13E-08	592.5004	649.7778	589.2845	1080.349	934.7914	1207.985
2361	RIPK1	2149.621	0.819817	0.11191	7.325682	2.38E-13	2.68E-12	1489.653	1730.369	1446.426	2720.963	2596.88	2913.435
17663	LOC11962:	19.59365	0.819318	0.587802	1.393867	0.163358	0.245873	16.80468	16.42235	9.044508	20.08914	32.08666	23.11453
16450	LOC10322:	27.71358	0.818941	0.518441	1.579622	0.114194	0.184549	12.46799	19.70683	28.52499	29.01765	36.36488	40.19918
4262	PHF11	327.8072	0.818827	0.158039	5.181157	2.21E-07	1.38E-06	228.2184	249.6198	232.3743	428.5684	432.1004	395.9619
3375	PHETA1	529.3276	0.81881	0.137303	5.963522	2.47E-09	1.94E-08	381.0867	373.3349	397.2626	676.3345	620.3421	727.6051
3429	DENND4A	2017.855	0.818219	0.13827	5.917563	3.27E-09	2.53E-08	1351.421	1777.446	1253.012	2520.071	2519.872	2685.305
4831	NR2F2	1369.951	0.817727	0.170554	4.794525	1.63E-06	8.97E-06	791.4461	1304.482	878.7087	1801.326	1709.149	1734.595
16825	LOC11962:	27.17763	0.816619	0.537084	1.520466	0.128394	0.202874	20.59928	23.53871	13.91463	35.71403	49.19955	20.09959
20517	SCGB3A2	9.446439	0.816592	0.866197	0.942733	0.345817	0.447515	5.420864	4.379295	10.43597	11.16063	19.252	6.029877
16584	LOC11962:	23.56569	0.816069	0.523091	1.56009	0.118739	0.190344	17.88885	14.78012	18.08902	29.01765	38.50399	23.11453
10365	LRRC29	76.82173	0.815742	0.294184	2.772902	0.005556	0.01425	61.79785	55.28859	49.39693	100.4457	100.5382	93.46309
27173	LOC11962	0.538989	0.815298	3.976081	0.205051	0.837533	NA	0	1.094824	0	0	2.139111	0
28283	LOC10322!	0.538989	0.815298	3.976081	0.205051	0.837533	NA	0	1.094824	0	0	2.139111	0
28456	MAGEB5	0.538989	0.815298	3.976081	0.205051	0.837533	NA	0	1.094824	0	0	2.139111	0
28473	LOC11962(	0.538989	0.815298	3.976081	0.205051	0.837533	NA	0	1.094824	0	0	2.139111	0
21669	LOC10321!	5.566564	0.815282	1.044006	0.780917	0.434851	0.533529	3.794605	2.189647	6.261582	6.696381	6.417332	8.039836
24539	TRNAR-UC	1.115538	0.81501	2.511541	0.324506	0.745555	0.807718	1.626259	0	0.695731	2.232127	2.139111	0
15415	LOC11962	34.15377	0.814768	0.46348	1.757935	0.078759	0.135829	27.10432	20.25424	27.82925	44.64254	27.80844	57.28383

21260	LOC10324!	6.663821	0.814422	0.968566	0.840853	0.40043	0.500703	4.336691	7.116354	2.782925	11.16063	8.556443	6.029877
27109	LOC10324:	0.554492	0.814421	3.936499	0.20689	0.836096	NA	0	1.094824	0	2.232127	0	0
28445	LOC10323:	0.554492	0.814421	3.936499	0.20689	0.836096	NA	0	1.094824	0	2.232127	0	0
28484	SIAH3	0.554492	0.814421	3.936499	0.20689	0.836096	NA	0	1.094824	0	2.232127	0	0
29600	LOC11962:	0.554492	0.814421	3.936499	0.20689	0.836096	NA	0	1.094824	0	2.232127	0	0
21890	LOC11962!	4.996986	0.814394	1.092215	0.745636	0.455888	0.553667	2.710432	5.474118	2.782925	6.696381	4.278221	8.039836
10762	LOC11962(	76.46068	0.814358	0.304176	2.677257	0.007423	0.018336	57.46116	53.64636	55.65851	111.6063	74.86887	105.5228
2508	SLC12A7	3230.686	0.81406	0.114341	7.119589	1.08E-12	1.15E-11	2441.557	2166.109	2426.015	4053.543	3674.992	4621.901
1962	ZNF7	2470.35	0.814024	0.100913	8.06659	7.23E-16	9.80E-15	1833.878	1877.075	1665.581	3073.639	3035.398	3336.532
12547	LOC10322(	51.02895	0.813677	0.354775	2.293499	0.021819	0.046231	33.06727	41.05589	36.87376	60.26743	70.59065	64.31869
16334	LOC10322!	26.92425	0.813656	0.508767	1.59927	0.109761	0.178645	14.63633	17.51718	26.43779	29.01765	42.78221	31.15436
22261	LOC10323(	4.486175	0.813571	1.193628	0.681596	0.495495	0.59174	3.252518	2.737059	3.478657	11.16063	4.278221	2.009959
13233	ADM	50.14247	0.81349	0.376336	2.161606	0.030649	0.061573	36.31979	37.224	35.4823	82.5887	44.92132	64.31869
13760	LOC11962:	71.85514	0.813477	0.395854	2.054993	0.03988	0.077049	49.87195	35.58177	69.57314	111.6063	111.2338	53.26391
13712	RTN4R	53.35122	0.813044	0.393373	2.066853	0.038748	0.075128	49.32986	28.46541	38.96096	75.89232	47.06043	80.39836
2057	FHOD1	2213.435	0.812985	0.103222	7.876077	3.38E-15	4.37E-14	1637.643	1617.602	1566.787	2758.909	2620.411	3079.257
22942	LOC11961!	3.628421	0.812853	1.418852	0.572895	0.566716	0.656705	1.084173	1.094824	5.565851	4.464254	8.556443	1.004979
18288	ECM2	21.44241	0.812752	0.632342	1.285305	0.198686	0.288826	7.047123	16.96977	22.2634	26.78552	38.50399	17.08465
20811	CUNH11or	8.05978	0.812576	0.900032	0.90283	0.366616	0.468315	7.047123	7.663765	2.782925	13.39276	6.417332	11.05477
19969	LOC10324(	10.58602	0.81257	0.785596	1.034336	0.300979	0.400697	7.047123	9.853413	6.261582	17.85702	6.417332	16.07967
16805	LOC10323:	26.09867	0.812212	0.532784	1.524469	0.127392	0.20153	13.55216	22.44388	20.17621	31.24978	47.06043	22.10955
3223	FBXO25	588.6862	0.812109	0.132313	6.137778	8.37E-10	6.90E-09	417.9486	460.9207	401.437	776.7802	744.4105	730.6201
2757	PRKCH	673.0029	0.811986	0.120064	6.762959	1.35E-11	1.30E-10	485.1673	498.6922	480.7504	868.2974	859.9225	845.1877
2726	ZNF250	837.2388	0.811936	0.119218	6.81054	9.72E-12	9.48E-11	635.8673	596.1315	593.4589	1002.225	1063.138	1132.612
11798	NRG3	85.59125	0.811904	0.331468	2.449419	0.014309	0.032242	57.46116	79.92212	50.09266	75.89232	115.512	134.6672
7440	LOC11962(	141.8926	0.811289	0.226292	3.585136	0.000337	0.001204	116.0065	95.24966	98.09812	180.8023	173.268	187.9312
17810	LOC11962:	19.36512	0.811173	0.589556	1.375904	0.168851	0.252436	14.63633	18.612	9.044508	20.08914	25.66933	28.13943
10141	PTP4A3	115.0599	0.811162	0.286697	2.82934	0.004664	0.012228	95.4072	66.78424	86.96642	138.3919	188.2417	114.5677
20067	PKLR	9.992919	0.811121	0.796796	1.017978	0.308688	0.408953	4.878777	10.40082	6.261582	13.39276	14.97377	10.04979
18580	LOC11962:	19.45776	0.810758	0.654816	1.238146	0.215662	0.308578	22.76763	9.853413	9.044508	26.78552	34.22577	14.06971
4195	TBC1D8	449.5808	0.810598	0.155237	5.22168	1.77E-07	1.12E-06	333.9252	345.9643	301.2517	595.9779	496.2737	624.0923
3789	LOC11962:	2108.301	0.810586	0.145912	5.55529	2.77E-08	1.94E-07	1670.168	1238.793	1688.54	2667.392	2350.883	3034.033
22121	LOC10322!	5.625319	0.810554	1.148829	0.705548	0.48047	0.577428	2.710432	1.094824	8.348776	11.16063	6.417332	4.019918
21512	LOC11961!	5.864099	0.810215	1.010216	0.802022	0.42254	0.522165	2.710432	3.831883	6.261582	8.928508	6.417332	7.034856
3204	HOXD4	682.8394	0.809608	0.131441	6.159496	7.30E-10	6.06E-09	513.8979	507.4508	468.2272	779.0123	921.9567	906.4915
3325	SPRYD4	528.4442	0.809516	0.134478	6.019707	1.75E-09	1.40E-08	401.686	389.7572	358.9974	669.6381	697.3501	653.2367
2334	PNPLA2	1224.996	0.809453	0.109978	7.360142	1.84E-13	2.09E-12	880.8904	927.3156	864.0984	1591.507	1446.039	1640.126
3499	LMO7	2123.35	0.809408	0.138279	5.853421	4.82E-09	3.66E-08	1389.367	1891.855	1346.936	2700.874	2667.471	2743.594
20416	LOC11961(	8.607241	0.809288	0.84418	0.958668	0.337726	0.439775	5.420864	4.926706	8.348776	8.928508	14.97377	9.044815
20509	MAOB	13.54399	0.809124	0.856584	0.944594	0.344866	0.447036	7.589209	10.40082	11.1317	4.464254	40.6431	7.034856
27826	LOC10321(	0.553604	0.80877	3.506228	0.230667	0.817574	NA	0.542086	0.547412	0	2.232127	0	0
29601	LOC11962:	0.553604	0.80877	3.506228	0.230667	0.817574	NA	0.542086	0.547412	0	2.232127	0	0
2390	CCDC134	1120.287	0.80852	0.11096	7.286562	3.18E-13	3.54E-12	838.6076	841.372	764.6088	1404.008	1379.726	1493.399
16495	PTPRN2	25.79371	0.807671	0.512558	1.575764	0.11508	0.185488	16.80468	16.42235	23.65487	22.32127	36.36488	39.1942
21067	LOC11961!	9.506412	0.807313	0.927369	0.870542	0.384004	0.484579	6.505036	2.737059	11.1317	13.39276	19.252	4.019918
16648	POPDC2	22.79663	0.807146	0.521073	1.549007	0.12138	0.19383	19.51511	15.32753	14.61036	31.24978	29.94755	26.12947
19082	PTK6	15.11178	0.806834	0.688395	1.172051	0.241177	0.336	8.673382	7.116354	17.39328	24.5534	12.83466	20.09959
9728	LOC11961!	150.6775	0.806543	0.275964	2.922644	0.003471	0.009485	82.93921	148.3486	97.40239	220.9806	160.4333	193.961
23116	TRNAA-AG	3.1151	0.80654	1.490922	0.540967	0.58853	0.67685	1.626259	1.642235	3.478657	8.928508	0	3.014938
23744	LOC11962(	2.216461	0.806092	1.809014	0.445598	0.655888	0.734366	2.710432	0.547412	1.391463	2.232127	6.417332	0
1766	HS3ST1	2973.344	0.805952	0.094249	8.551275	1.22E-17	1.83E-16	2097.874	2302.414	2092.76	3658.456	3786.226	3902.335
3413	PYCR3	1895.561	0.805832	0.135791	5.934335	2.95E-09	2.30E-08	1545.488	1171.461	1425.554	2381.679	2149.806	2699.375
12613	ADCY10	61.14968	0.805332	0.353187	2.280187	0.022597	0.047631	52.04029	42.69812	37.56949	75.89232	98.39909	60.29877
2504	TSC22D2	3147.232	0.804957	0.112959	7.126071	1.03E-12	1.10E-11	2111.968	2624.292	2137.983	4055.775	3861.095	4092.276
12635	ANO2	67.22872	0.804167	0.353716	2.273479	0.022997	0.048388	54.75072	40.50847	50.78839	69.19594	119.7902	68.3386
19158	LOC11962!	12.50578	0.804161	0.693823	1.159029	0.246444	0.341984	9.215468	7.663765	10.43597	13.39276	19.252	15.07469
12668	LOC10323:	53.81899	0.804034	0.354691	2.266856	0.023399	0.049106	41.74065	32.2973	43.13534	78.12444	66.31243	61.30375
2323	VDAC3	4653.017	0.803935	0.108974	7.377337	1.61E-13	1.85E-12	3543.619	2934.675	3688.768	6073.618	5901.806	5775.617
3780	HOMEZ	2039.756	0.803814	0.144452	5.564593	2.63E-08	1.85E-07	1304.802	1848.61	1302.409	2566.946	2695.279	2520.489
18702	LOC11962!	14.47854	0.803656	0.654703	1.227511	0.219631	0.312206	11.38381	11.49565	8.348776	22.32127	19.252	14.06971
8649	SKIDA1	131.6628	0.803512	0.249864	3.215793	0.001301	0.003998	91.07051	90.32295	105.0554	158.481	205.3546	139.6921
4303	ETV1	880.5206	0.803413	0.155914	5.152929	2.56E-07	1.58E-06	574.0695	793.7471	555.1936	1122.76	1152.981	1084.373
3108	MSRB2	705.0573	0.802653	0.127976	6.271888	3.57E-10	3.05E-09	516.0662	497.5973	530.1473	805.7978	939.0696	941.6658
3811	GSDMD	2651.023	0.802565	0.145123	5.530229	3.20E-08	2.23E-07	2146.662	1522.9	2130.329	3323.637	3046.094	3736.514
23627	TRNAC-GC	1.939754	0.802506	1.726071	0.464932	0.64198	0.722353	0.542086	2.189647	1.391463	2.232127	4.278221	1.004979
17389	LOC10323(	20.35719	0.802394	0.560828	1.43073	0.152508	0.23316	13.55216	13.13788	17.39328	29.01765	29.94755	19.09461
4767	OPLAH	765.9093	0.802177	0.165981	4.832944	1.35E-06	7.50E-06	597.9213	455.4466	624.071	1042.403	804.3056	1071.308
18747	ECSCR	17.91584	0.80215	0.654947	1.224755	0.220667	0.312922	13.55216	7.663765	17.39328	29.01765	27.80844	12.05975
3288	PPP2R3A	3705.149	0.801965	0.132512	6.052003	1.43E-09	1.16E-08	2432.342	3289.398	2382.184	4604.878	4592.671	4929.424
6052	PTGIS	209.187	0.801783	0.193509	4.143379	3.42E-05	0.00015	164.7943	142.3271	148.8865	274.5516	282.3626	242.2001
20499	LOC10322:	13.74652	0.801732	0.846618	0.946982	0.343648	0.445674	5.420864	20.80165	3.478657	29.01765	10.69555	13.06473
2773	LOC10321(	1245.56	0.80161	0.11892	6.740771	1.58E-11	1.51E-10	887.3954	937.169	905.1465	1462.043	1503.795	1777.809
1646	ADSS2	3207.203	0.801486	0.090448	8.861315	7.91E-19	1.28E-17	2277.847	2472.659	2264.606	4040.15	4117.788	4070.167
2160	ZBTB4	3328.048	0.801032	0.104481	7.666807	1.76E-14	2.17E-13	2306.578	2704.214	2271.563	4234.345	4089.98	4361.611

19501	LOC10324:	11.71025	0.800935	0.727478	1.100975	0.270908	0.369299	7.047123	11.49565	6.957314	17.85702	12.83466	14.06971
1952	YPEL5	2010.962	0.800176	0.09888	8.092427	5.85E-16	7.97E-15	1451.165	1415.06	1532.696	2584.803	2661.054	2420.996
17329	DEF6	26.4266	0.800053	0.554094	1.443896	0.148768	0.22823	26.02015	13.6853	17.39328	29.01765	51.33866	21.10457
5313	FZD5	610.4299	0.799815	0.177251	4.512336	6.41E-06	3.21E-05	385.4234	572.5928	377.7821	781.2444	763.6625	781.874
10919	PAMR1	82.54398	0.799639	0.3021	2.646936	0.008122	0.019775	72.09749	52.55153	57.04997	89.28508	102.6773	121.6025
12096	INHA	93.17891	0.799277	0.334761	2.387602	0.016959	0.037272	81.85504	43.24553	78.61764	149.5525	96.25998	109.5428
2982	LOC11962:	733.5492	0.799103	0.12391	6.449069	1.13E-10	1.00E-09	536.6655	560.5497	511.3626	895.0829	902.7047	994.9297
22043	LOC10321⁄	5.012428	0.798587	1.112206	0.71802	0.472745	0.570155	1.626259	3.831883	5.565851	4.464254	8.556443	6.029877
16989	LOC10324:	30.12134	0.798441	0.534305	1.494355	0.135083	0.211392	19.51511	19.15941	26.43779	33.4819	62.03421	20.09959
3362	LRP10	3254.738	0.798323	0.133579	5.976424	2.28E-09	1.80E-08	2596.594	2000.79	2537.332	3957.561	3717.774	4718.379
9415	LOC11962:	119.468	0.797914	0.265613	3.00405	0.002664	0.007523	109.5014	78.8273	73.74753	154.0168	136.9031	163.8117
21124	TRNAP-CG	7.562625	0.797751	0.926151	0.861361	0.389039	0.489591	4.878777	7.116354	4.174388	15.62489	8.556443	5.024897
10409	RNF122	84.01474	0.797457	0.288523	2.763925	0.005711	0.014586	58.54533	55.28859	70.9646	111.6063	94.12087	113.5627
18028	LOC11961!	17.56592	0.79725	0.595718	1.3383	0.180799	0.2666	10.29964	15.87494	12.52316	22.32127	19.252	25.12449
17269	TIE1	29.53229	0.797197	0.547565	1.455893	0.145422	0.223872	14.09425	18.612	31.30791	53.57105	38.50399	21.10457
3217	ZFAT	733.9162	0.79691	0.129674	6.145475	7.97E-10	6.59E-09	509.5612	590.1099	510.6668	917.4042	889.87	985.8849
10320	LOC10323{	81.03332	0.796833	0.286283	2.783374	0.00538	0.013858	59.6295	65.14201	52.87558	98.21359	104.8164	105.5228
2482	LOC10322:	1723.714	0.796828	0.111397	7.153033	8.49E-13	9.09E-12	1241.92	1371.814	1166.742	2176.324	2059.964	2325.522
2424	SLC44A2	4984.559	0.796725	0.11013	7.234405	4.68E-13	5.13E-12	3472.063	4014.718	3443.87	6252.188	5767.042	6957.473
2058	PLAAT3	2889.025	0.79659	0.101158	7.874744	3.41E-15	4.41E-14	2096.248	2277.781	1961.267	3620.51	3525.254	3853.091
20001	LOC11962!	10.31871	0.796338	0.773472	1.029564	0.303215	0.403008	9.215468	4.926706	8.348776	13.39276	14.97377	11.05477
18066	LOC11962(	17.86066	0.796107	0.5983	1.330614	0.183316	0.269759	15.7205	13.6853	9.740239	26.78552	17.11289	24.11951
3391	ZNF365	809.3336	0.796058	0.133747	5.951951	2.65E-09	2.08E-08	563.7698	670.0321	539.8875	1084.814	975.4345	1022.064
3510	LOC10322{	967.5416	0.795927	0.136107	5.847808	4.98E-09	3.77E-08	633.1569	824.4022	665.1192	1180.795	1221.432	1280.344
3348	CTTNBP2	1650.736	0.795814	0.132725	5.995941	2.02E-09	1.61E-08	1103.688	1441.335	1074.905	2093.735	2047.129	2143.621
16635	LOC11962:	25.05019	0.795511	0.512614	1.551871	0.120693	0.192884	24.39389	15.32753	15.30609	29.01765	32.08666	34.1693
3614	MGST3	5752.38	0.795443	0.13896	5.724277	1.04E-08	7.64E-08	4744.34	3223.708	4649.573	7263.341	7270.837	7362.48
2906	AFF1	4891.658	0.795388	0.121641	6.538817	6.20E-11	5.67E-10	3181.505	4243.536	3304.724	6111.564	6102.883	6405.739
22239	LOC11962⁄	4.744704	0.795261	1.159809	0.685682	0.492913	0.589238	4.336691	4.379295	1.391463	8.928508	6.417332	3.014938
3240	LATS1	2067.158	0.794955	0.129896	6.119941	9.36E-10	7.68E-09	1405.088	1799.89	1330.238	2573.642	2577.628	2716.46
22192	SPI1	4.772393	0.794746	1.146537	0.693171	0.488203	0.584822	1.626259	2.737059	6.261582	6.696381	4.278221	7.034856
1783	LOC10321:	7358.269	0.794443	0.093447	8.501527	1.87E-17	2.79E-16	5173.13	5874.276	5096.928	9598.146	9170.367	9236.766
16856	LOC11962:	26.95213	0.794425	0.52468	1.514113	0.129997	0.205029	15.17842	29.01283	15.30609	26.78552	34.22577	41.20416
20229	LOC10323(	9.786099	0.794414	0.801675	0.990942	0.321714	0.422797	6.505036	7.663765	7.653045	13.39276	6.417332	17.08465
2413	CYP1B1	2133.365	0.793458	0.109392	7.25332	4.07E-13	4.48E-12	1491.822	1718.873	1473.559	2790.159	2519.872	2805.903
3400	PTPRM	7801.542	0.793434	0.133473	5.944524	2.77E-09	2.17E-08	5160.12	7051.759	4913.951	9908.412	9688.032	10086.98
22039	LOC11962:	5.00657	0.793337	1.104013	0.718594	0.472391	0.569832	3.252518	2.737059	4.87012	4.464254	10.69555	4.019918
22896	LOC11962(	3.609356	0.793244	1.36806	0.579831	0.562029	0.65257	0.542086	3.831883	3.478657	2.232127	8.556443	3.014938
2533	BNIP3L	2970.225	0.792469	0.111983	7.076721	1.48E-12	1.55E-11	1955.848	2475.944	2090.673	3774.527	3779.809	3744.554
2421	KIAA1549	4338.145	0.791511	0.109294	7.242034	4.42E-13	4.85E-12	3011.29	3570.22	2952.684	5209.784	5392.698	5892.195
1918	EFR3A	13225.71	0.791343	0.097064	8.152804	3.56E-16	4.93E-15	9167.223	10770.87	9123.125	16676.22	16366.34	17250.47
19391	LOC10323:	13.11881	0.790816	0.706641	1.11912	0.263089	0.360694	6.505036	13.6853	8.348776	17.85702	19.252	13.06473
1917	PARP3	3379.231	0.790752	0.096868	8.163178	3.26E-16	4.53E-15	2482.756	2554.224	2394.707	4153.988	4055.754	4633.96
22316	LOC10322:	4.18465	0.790583	1.175858	0.672346	0.501364	0.597273	2.168345	4.379295	2.782925	4.464254	4.278221	7.034856
23508	LOC10321{	2.790641	0.790197	1.638745	0.482196	0.629666	0.712078	2.168345	0	4.174388	2.232127	2.139111	6.029877
2607	KAZN	1142.405	0.790146	0.113357	6.970395	3.16E-12	3.22E-11	801.2037	902.6821	809.1356	1455.347	1377.587	1508.474
1838	ARMCX3	2391.988	0.790132	0.094727	8.341128	7.36E-17	1.06E-15	1791.053	1811.386	1657.232	3011.139	2962.668	3118.451
1877	PRUNE2	9924.643	0.790117	0.095933	8.23611	1.78E-16	2.52E-15	7400.021	7693.326	6729.114	12020	12201.49	13503.91
4916	LOC10324!	272.7764	0.789988	0.166795	4.736268	2.18E-06	1.18E-05	197.8615	206.3743	196.1962	343.7476	333.7013	358.7777
6658	LOC10323:	243.988	0.78992	0.203927	3.873537	0.000107	0.000428	158.8313	217.3225	160.0182	281.248	337.9795	308.5287
6038	SLC22A12	237.3395	0.789758	0.190395	4.147994	3.35E-05	0.000148	155.0367	195.426	172.5414	274.5516	305.8928	320.5885
17466	DISP3	20.90849	0.789565	0.557462	1.416356	0.156671	0.238469	14.63633	12.59047	18.78475	20.08914	34.22577	25.12449
21020	LOC10321:	9.50621	0.789457	0.899887	0.877284	0.380332	0.481024	7.589209	3.831883	9.044508	11.16063	21.39111	4.019918
15877	ZNF837	48.59651	0.789428	0.467628	1.688155	0.091382	0.153009	36.86187	20.25424	50.09266	84.82083	34.22577	65.32367
2215	PI4K2A	2276.559	0.789412	0.104195	7.57629	3.56E-14	4.27E-13	1678.841	1700.261	1631.49	2903.997	2618.271	3126.491
15812	ANKRD2	30.47138	0.789307	0.464584	1.698953	0.089328	0.150189	22.76763	18.06459	25.74206	40.17829	44.92132	31.15436
10518	LOC11962:	90.09026	0.789201	0.288039	2.739913	0.006146	0.015533	58.54533	62.95236	77.22618	93.74933	130.4858	117.5826
14775	LOC10322:	42.65063	0.788949	0.420226	1.877443	0.060457	0.108775	28.73058	33.93953	29.91645	73.66019	53.47777	36.17926
16744	ANKRD55	33.83503	0.788924	0.514243	1.534146	0.124994	0.198457	22.22554	24.08612	27.82925	71.42806	19.252	38.18922
10020	LOC11962:	204.8877	0.788907	0.276183	2.856463	0.004284	0.011366	171.8414	123.1677	153.0609	247.7661	363.6488	169.8415
8166	LOC11962:	194.5847	0.788892	0.23484	3.359281	0.000781	0.002544	158.8313	111.1246	159.3225	270.0874	196.7982	271.3445
1895	MINK1	2844.246	0.788888	0.096128	8.206622	2.27E-16	3.19E-15	1984.036	2218.113	2056.582	3578.1	3476.055	3752.593
6077	RPS6KL1	341.1691	0.788108	0.190667	4.133433	3.57E-05	0.000156	280.2587	191.5941	278.9883	448.6575	421.4048	426.1113
2523	TPRG1L	1458.478	0.787993	0.111114	7.091738	1.32E-12	1.40E-11	1121.577	995.7421	1095.777	1814.719	1741.236	1981.82
3043	KCNIP2	715.3606	0.787958	0.124105	6.349122	2.17E-10	1.89E-09	549.1335	511.2826	514.1455	843.744	958.3216	915.5363
2568	PCDH1	999.1977	0.787918	0.112173	7.024111	2.15E-12	2.23E-11	745.9108	758.7128	695.7314	1243.295	1229.989	1321.548
3955	IGDCC3	1334.758	0.787761	0.145711	5.406311	6.43E-08	4.32E-07	1089.052	799.7687	1051.946	1683.024	1514.49	1870.267
17507	NOX1	20.34625	0.787373	0.557113	1.413311	0.157564	0.23947	14.63633	15.87494	13.91463	22.32127	34.22577	21.10457
17907	LOC11962:	20.64401	0.78723	0.578711	1.360315	0.17373	0.257923	16.26259	18.06459	10.43597	26.78552	34.22577	18.08963
23528	TRPC4	2.782833	0.78718	1.644419	0.478698	0.632153	0.714289	1.084173	2.737059	2.087194	2.232127	8.556443	0
22047	LOC10323:	5.582587	0.787171	1.098344	0.716689	0.473566	0.571041	3.252518	3.831883	4.87012	6.696381	12.83466	2.009959
22723	CUNH1orf{	4.409181	0.787139	1.301582	0.604755	0.545342	0.638005	2.168345	5.474118	2.087194	0	10.69555	6.029877
16441	LOC10322!	27.48554	0.78705	0.497637	1.581575	0.113747	0.183928	21.68345	14.23271	24.3506	42.41041	32.08666	30.14938
16777	LOC11962(	42.28723	0.78688	0.514572	1.529192	0.126217	0.200005	44.99317	15.87494	31.30791	49.10679	81.28621	31.15436

20425	MAP3K19	9.453312	0.786523	0.821075	0.957919	0.338104	0.440073	7.047123	6.02153	7.653045	6.696381	19.252	10.04979
2994	PIP5K1C	971.4164	0.786505	0.122333	6.429208	1.28E-10	1.14E-09	658.0929	745.5749	738.8667	1209.813	1146.563	1329.588
18553	KCNE5	21.74281	0.78571	0.632212	1.242794	0.213944	0.306564	13.55216	13.6853	20.17621	15.62489	51.33866	16.07967
24610	LOC10323	1.10181	0.78557	2.494197	0.314959	0.752793	0.813206	0.542086	1.094824	0.695731	0	4.278221	0
5623	LOC10322	1048.178	0.785251	0.180008	4.362325	1.29E-05	6.08E-05	659.7191	1026.945	622.6796	1343.74	1247.102	1388.882
2157	BTG1	3201.129	0.785248	0.102348	7.672329	1.69E-14	2.08E-13	2495.766	2184.721	2376.618	3839.258	3948.798	4361.611
7147	FBXL6	889.9441	0.784485	0.213094	3.681399	0.000232	0.000863	809.3349	417.6752	736.7795	1078.117	988.2691	1309.488
24290	TRNAF-GA	1.68191	0.783962	2.156585	0.36352	0.716216	0.783887	0	2.189647	1.391463	2.232127	4.278221	0
22344	LOC10324	4.177905	0.783484	1.17272	0.668092	0.504075	0.599751	4.336691	2.737059	2.087194	4.464254	6.417332	5.024897
2618	LOC10323	1812.662	0.783406	0.112606	6.95704	3.47E-12	3.53E-11	1327.57	1442.43	1230.053	2189.717	2196.867	2489.334
13816	LOC10323	59.69068	0.782565	0.382651	2.045117	0.040843	0.078591	52.58238	47.62483	29.91645	87.05295	87.70354	53.26391
17444	LOC11962	28.74984	0.782347	0.551245	1.419237	0.15583	0.237488	17.88885	18.06459	26.43779	49.10679	44.92132	16.07967
2600	MGST1	10526.72	0.782334	0.11206	6.981388	2.92E-12	2.99E-11	6909.433	8997.261	7313.528	13301.24	13529.87	13108.95
3542	FKBP5	3304.928	0.781899	0.134544	5.811459	6.19E-09	4.65E-08	2139.615	2969.162	2183.901	4160.685	4006.554	4369.651
4227	ASTE1	379.9677	0.781723	0.150352	5.199296	2.00E-07	1.26E-06	293.8108	279.18	264.3779	508.925	464.187	469.3254
15822	LOC10323	31.80258	0.781243	0.460722	1.695692	0.089944	0.151129	20.59928	25.18094	24.3506	29.01765	53.47777	38.18922
3264	PCM1	10792.86	0.781071	0.128453	6.080588	1.20E-09	9.75E-09	7120.304	9679.883	7022.017	13095.89	13718.12	14120.97
4893	ALDH4A1	1466.501	0.780663	0.16451	4.745392	2.08E-06	1.13E-05	1221.863	856.6995	1163.263	1796.862	1559.412	2200.905
14330	SEPTIN3	48.64279	0.780502	0.398996	1.956165	0.050446	0.093587	32.52518	39.41365	34.09084	82.5887	62.03421	41.20416
16686	LOC11961	23.81468	0.780316	0.505183	1.544619	0.122438	0.19506	17.34676	18.06459	17.39328	31.24978	25.66933	33.16432
7216	ACER2	196.3493	0.779838	0.212983	3.661494	0.000251	0.000924	140.9425	165.3184	125.9274	232.1412	286.6408	227.1254
21749	LOC11962	5.909089	0.779574	1.014079	0.768751	0.442041	0.540332	2.710432	4.926706	5.565851	8.928508	4.278221	9.044815
18102	LOC11962	19.33589	0.779543	0.590447	1.320258	0.186749	0.274264	15.7205	9.306001	17.39328	24.5534	29.94755	19.09461
14723	TRNAY-GU	52.51072	0.779508	0.413576	1.884802	0.059457	0.107359	33.60935	40.50847	40.35242	71.42806	91.98176	37.18424
16675	LOC11962	24.68647	0.779222	0.504158	1.545592	0.122203	0.19483	20.0572	14.78012	19.48048	35.71403	29.94755	28.13943
12887	TRNAH-GU	74.28238	0.779049	0.35072	2.221287	0.026332	0.05432	64.50828	35.03436	64.00729	91.51721	111.2338	79.39338
3220	MOSPD1	624.4098	0.778909	0.126866	6.139615	8.27E-10	6.83E-09	474.3256	458.1837	444.5723	823.6549	800.0274	745.6948
18326	LOC11962	16.77113	0.778614	0.608784	1.278966	0.200909	0.291453	10.29964	13.13788	13.91463	15.62489	23.53022	24.11951
3920	SPTBN4	492.6648	0.778292	0.143329	5.430106	5.63E-08	3.82E-07	381.6288	362.3866	345.0828	667.406	558.3079	641.1769
15224	LOC11962	46.01967	0.778273	0.434377	1.791699	0.073181	0.127793	23.30971	44.34036	34.09084	37.94616	79.14709	57.28383
5494	ERICH1	394.7924	0.778134	0.176375	4.411812	1.03E-05	4.96E-05	255.3227	347.6065	269.9438	446.4254	536.9168	512.5395
2053	SUSD4	1599.521	0.777984	0.098751	7.878261	3.32E-15	4.30E-14	1161.149	1190.073	1186.918	1944.183	2004.347	2110.457
6307	SIGIRR	545.556	0.777764	0.193373	4.022098	5.77E-05	0.000243	444.5108	312.0247	452.9211	676.3345	560.447	827.0981
17032	LOC11962	44.97928	0.777737	0.522437	1.488673	0.136573	0.213176	44.99317	10.94824	43.13534	60.26743	66.31243	44.2191
18173	LOC10323	17.59557	0.777449	0.594558	1.307607	0.191007	0.279421	11.9259	15.87494	11.1317	17.85702	25.66933	23.11453
4937	FOXO4	294.6578	0.777389	0.164598	4.722962	2.32E-06	1.25E-05	215.7504	204.732	230.2871	383.9258	376.4835	356.7677
21742	TMEM236	6.451334	0.777131	1.008334	0.770709	0.44088	0.539106	4.336691	6.02153	3.478657	11.16063	10.69555	3.014938
19551	LOC10323	12.10631	0.776711	0.71214	1.090673	0.275417	0.374506	8.131296	8.211177	10.43597	20.08914	10.69555	15.07469
12959	LOC11962	64.14017	0.776623	0.351123	2.211826	0.026979	0.055346	46.61943	37.224	57.04997	98.21359	83.42532	62.30873
23177	LOC11962	4.470941	0.776264	1.454827	0.533578	0.593633	0.680936	4.878777	0	4.87012	2.232127	12.83466	2.009959
2322	TSC22D4	1787.422	0.776239	0.105169	7.38086	1.57E-13	1.80E-12	1309.139	1245.362	1402.594	2263.377	2126.276	2377.781
3083	PTPRJ	4101.725	0.775989	0.123251	6.295988	3.05E-10	2.63E-09	2886.61	3510.005	2679.262	5109.339	4785.191	5639.945
2384	UTP4	2227.238	0.775689	0.106343	7.294233	3.00E-13	3.35E-12	1595.36	1780.183	1554.96	2774.534	2652.497	3005.894
21745	TRNAC-AC	6.39802	0.775666	1.006716	0.770492	0.441008	0.539168	4.878777	3.831883	5.565851	2.232127	12.83466	9.044815
15367	CCNA1	33.14329	0.775345	0.438973	1.76627	0.077351	0.133817	24.93597	22.9913	25.74206	46.87467	32.08666	46.22906
22848	LOC11962	3.364351	0.775325	1.324278	0.58547	0.558232	0.649536	1.626259	2.189647	3.478657	4.464254	6.417332	2.009959
3957	APOBEC3H	533.1729	0.774893	0.143347	5.405707	6.46E-08	4.34E-07	373.4975	440.6665	366.6504	671.8702	643.8723	702.4806
2304	LOC10323	3699.915	0.774738	0.104574	7.408523	1.28E-13	1.47E-12	2615.567	3021.166	2551.943	4886.126	4402.29	4722.399
24767	LOC11962	1.156648	0.77473	2.696389	0.287321	0.773866	0.830671	1.084173	0	1.391463	4.464254	0	0
21507	PYHIN1	6.725141	0.77466	0.965191	0.802598	0.422207	0.521894	2.710432	7.116354	4.87012	8.928508	10.69555	6.029877
24010	LOC10322	1.668714	0.774613	1.911963	0.40514	0.685375	0.758879	0.542086	1.094824	2.087194	0	4.278221	2.009959
5795	ARL11	354.9157	0.774371	0.181054	4.277009	1.89E-05	8.69E-05	284.0533	214.038	289.4243	399.5507	462.0479	480.3802
21718	TRNAN-GU	9.091843	0.774301	0.998429	0.775519	0.438033	0.536181	1.626259	12.59047	5.565851	22.32127	6.417332	6.029877
5923	LOC10322	375.1972	0.773638	0.184194	4.200122	2.67E-05	0.00012	324.7097	225.5337	281.7712	495.5322	417.1266	506.5097
21061	LOC10321	8.727113	0.773346	0.887793	0.871089	0.383706	0.484346	3.252518	6.02153	10.43597	11.16063	6.417332	15.07469
4360	MRPL15	2984.071	0.773298	0.15144	5.106318	3.28E-07	2.00E-06	2540.759	1637.309	2432.277	3698.634	3692.105	3903.34
4451	UBTD1	399.0813	0.773076	0.153331	5.041868	4.61E-07	2.75E-06	311.6997	274.8007	299.1645	513.3892	457.7697	537.664
16004	TAF1L	34.84444	0.772914	0.464491	1.664003	0.096112	0.159646	20.0572	29.01283	27.13352	49.10679	55.61688	28.13943
21885	LOC10322	6.191359	0.772853	1.035135	0.746621	0.455293	0.553045	7.047123	2.189647	4.174388	11.16063	8.556443	4.019918
22216	LOC11962	5.075595	0.77285	1.120693	0.689618	0.490434	0.586864	2.710432	1.642235	6.957314	6.696381	6.417332	6.029877
12443	ZNF563	59.91234	0.772218	0.334013	2.311937	0.020781	0.044402	47.16151	40.50847	45.22254	62.49956	87.70354	76.37844
19358	LOC11962	16.1809	0.771781	0.685576	1.125741	0.260275	0.357445	16.80468	7.116354	11.82743	11.16063	32.08666	18.08963
2116	LIMK1	4477.457	0.771558	0.099306	7.769469	7.88E-15	9.90E-14	3157.111	3593.759	3176.014	5600.407	5347.777	5989.678
15235	LOC11962	3566.317	0.771333	0.431012	1.789587	0.07352	0.128293	1612.165	4180.584	2112.24	4624.967	4344.534	4523.413
2962	IGSF9B	898.7619	0.770987	0.119064	6.475424	9.45E-11	8.49E-10	642.3723	703.4242	649.1174	1131.688	1060.999	1204.97
12024	CELF6	74.79178	0.770971	0.320935	2.402268	0.016294	0.036025	65.59245	45.43518	53.57132	109.3742	98.39909	76.37844
16998	RNF152	51.52278	0.770725	0.516412	1.492461	0.135578	0.212045	27.6464	34.48694	50.78839	71.42806	102.6773	22.10955
20800	LOC11962	14.33692	0.77066	0.852126	0.904397	0.365785	0.467519	13.01007	7.663765	10.43597	13.39276	38.50399	3.014938
4544	CADM4	630.4245	0.77026	0.154652	4.980594	6.34E-07	3.71E-06	501.4299	405.6322	494.665	687.4951	797.8883	895.4367
23377	LOC11961	2.529602	0.770217	1.53331	0.502323	0.61544	0.699874	1.626259	0.547412	3.478657	2.232127	4.278221	3.014938
13762	LOC11962	76.16848	0.769345	0.374444	2.054634	0.039914	0.077105	71.5554	44.34036	51.48412	89.28508	139.0422	61.30375
10220	CPEB3	90.40955	0.769145	0.273633	2.810859	0.004941	0.012853	61.79785	74.99542	63.31155	118.3027	115.512	108.5378
22090	LOC10324	5.29479	0.769076	1.080809	0.711575	0.476728	0.573803	3.252518	4.379295	4.174388	2.232127	10.69555	7.034856
3558	STBD1	1803.705	0.768721	0.132554	5.799301	6.66E-09	4.98E-08	1196.385	1595.158	1211.268	2310.251	2177.615	2331.552

15194	SPTA1	40.17624	0.768683	0.427761	1.796991	0.072337	0.126569	30.89892	24.63353	32.69937	51.33892	66.31243	35.17428
3093	LZTS2	2470.519	0.768642	0.122249	6.287512	3.23E-10	2.77E-09	1932.538	1630.74	1925.089	2964.265	2845.017	3525.468
9598	LOC11962	109.4661	0.768617	0.259538	2.961486	0.003062	0.00848	81.31296	71.16354	91.83654	127.2312	130.4858	154.7668
4766	BTG2	540.5237	0.768607	0.159005	4.833846	1.34E-06	7.47E-06	355.0666	477.8905	365.9547	660.7096	710.1847	673.3362
6608	ZNF586	206.6337	0.768309	0.197026	3.899532	9.64E-05	0.000388	167.5047	146.159	143.3207	287.9444	256.6933	238.1801
22107	FBLN2	5.864594	0.767733	1.085137	0.707498	0.479257	0.576335	7.047123	1.642235	4.174388	4.464254	12.83466	5.024897
15583	HAL	39.1311	0.767419	0.443074	1.732033	0.083268	0.142048	28.73058	29.01283	27.82925	58.0353	62.03421	29.1444
16288	LOC11962	29.22095	0.767359	0.476963	1.608843	0.107651	0.175706	16.80468	22.9913	25.74206	33.4819	32.08666	44.2191
18993	LOC11962	17.28588	0.767263	0.646798	1.186247	0.235525	0.329655	11.9259	17.51718	9.044508	11.16063	29.94755	24.11951
18839	PTTG1IP2	17.25507	0.766969	0.632557	1.212489	0.225325	0.317972	11.38381	9.306001	17.39328	31.24978	17.11289	17.08465
9195	LOC10322	111.1048	0.766842	0.25095	3.055757	0.002245	0.006491	75.89209	88.68071	81.40057	156.2489	134.764	129.6424
2377	TXNDC11	1331.54	0.766579	0.104945	7.30461	2.78E-13	3.11E-12	986.5972	1008.333	965.6751	1651.774	1600.055	1776.804
6700	CRPPA	219.1757	0.766378	0.198787	3.855263	0.000116	0.000459	166.4205	149.4434	170.4542	243.3018	325.1448	260.2897
2650	UNC93B1	5020.85	0.76626	0.111146	6.894155	5.42E-12	5.44E-11	3806.53	3577.336	3775.038	6535.668	5441.898	6988.627
16468	CATSPERE	34.61439	0.765474	0.484971	1.578391	0.114476	0.184803	35.7777	18.612	21.56767	49.10679	53.47777	29.1444
18337	LOC10321	19.89884	0.765466	0.59935	1.27716	0.201546	0.29219	20.0572	9.306001	14.61036	20.08914	34.22577	21.10457
22920	LOC11961	3.343511	0.765403	1.327901	0.576401	0.564344	0.654585	2.168345	3.831883	1.391463	2.232127	6.417332	4.019918
8856	LOC10323	133.4131	0.764602	0.2423	3.155597	0.001602	0.004808	114.3802	83.75401	98.09812	158.481	183.9635	161.8017
4692	LAMA5	2966.443	0.764562	0.15639	4.888817	1.01E-06	5.75E-06	2328.261	1712.852	2557.509	3866.044	3140.214	4193.779
19720	IGFBPL1	12.32047	0.764551	0.717269	1.065919	0.28646	0.386184	8.673382	8.758589	9.740239	11.16063	23.53022	12.05975
18933	LOC11962	15.40986	0.764267	0.637823	1.198244	0.230822	0.324111	11.9259	9.853413	12.52316	13.39276	25.66933	19.09461
18847	BMP4	29.03423	0.763824	0.630028	1.212365	0.225373	0.317985	10.29964	14.78012	38.96096	53.57105	38.50399	18.08963
16231	LOC10322	33.67139	0.763187	0.47072	1.621317	0.10495	0.171899	25.47806	31.20247	17.39328	40.17829	55.61688	32.15934
3520	RTKN	735.8543	0.762677	0.130612	5.839248	5.24E-09	3.96E-08	569.7328	503.0715	567.7168	910.7078	859.9225	1003.974
18044	LOC11962	21.0712	0.762394	0.570869	1.335497	0.181714	0.267727	21.14137	14.78012	10.43597	29.01765	29.94755	21.10457
4171	NFE2L3	1240.652	0.762243	0.14533	5.2449	1.56E-07	9.97E-07	810.9612	1128.216	821.6587	1537.935	1557.273	1587.868
4533	LOC11961	516.7496	0.762125	0.152632	4.993234	5.94E-07	3.48E-06	358.3191	373.8823	414.6559	698.6557	695.211	559.7736
2245	BAHD1	1475.466	0.761964	0.101328	7.519805	5.49E-14	6.50E-13	1090.678	1106.319	1090.211	1808.023	1796.853	1960.715
4789	MKNK1	334.3731	0.761891	0.158078	4.819722	1.44E-06	7.98E-06	262.9119	241.4086	240.0273	392.8543	444.935	424.1013
21366	SNAI3	7.808228	0.761795	0.927167	0.821638	0.411283	0.511715	8.131296	4.926706	4.174388	4.464254	17.11289	8.039836
10931	LOC11961	189.754	0.761401	0.287936	2.644339	0.008185	0.019907	154.4946	94.15483	171.8456	261.1589	293.0582	163.8117
22873	LOC10324	3.387816	0.76105	1.306098	0.582689	0.560102	0.650971	1.626259	3.284471	2.782925	4.464254	2.139111	6.029877
1713	LEPROT	6759.514	0.761047	0.087555	8.692222	3.55E-18	5.52E-17	5202.945	4811.75	5039.878	8120.478	8470.878	8911.153
1734	MCL1	15938.19	0.760898	0.088184	8.628568	6.21E-18	9.52E-17	11896.63	12236.3	11361.99	19336.92	19294.78	21502.54
2727	RAB11FIP1	1996.411	0.760251	0.111672	6.807875	9.91E-12	9.66E-11	1448.455	1573.262	1429.728	2274.537	2487.786	2764.699
12548	LOC11962	74.31535	0.760217	0.331475	2.29344	0.021823	0.046235	56.91907	61.31012	46.614	71.42806	126.2075	83.4133
18986	KRT39	16.30436	0.759873	0.640152	1.187019	0.23522	0.329351	10.29964	9.306001	16.69755	15.62489	27.80844	18.08963
2034	RAB11A	4800.902	0.759837	0.096175	7.900555	2.78E-15	3.63E-14	3783.763	3237.393	3675.549	6028.975	5940.31	6139.42
13356	TMEM269	58.75007	0.75965	0.355715	2.135556	0.032716	0.065115	53.66655	36.12918	40.35242	69.19594	89.84265	63.31371
22152	LOC10321	5.365486	0.759468	1.086407	0.699063	0.484512	0.581472	1.626259	6.02153	4.174388	8.928508	6.417332	5.024897
7614	LOC10322	157.4938	0.759069	0.215568	3.521257	0.00043	0.0015	108.9594	116.5987	125.9274	178.5702	213.9111	200.9959
9589	CD99L2	546.1984	0.758828	0.256022	2.96392	0.003037	0.008421	297.0633	625.1443	293.5986	665.1738	787.1927	609.0176
3242	RNF170	1010.156	0.758569	0.124055	6.114797	9.67E-10	7.93E-09	821.2608	666.7476	763.913	1225.438	1311.275	1272.304
4174	PRRG1	986.307	0.758211	0.144583	5.244123	1.57E-07	1.00E-06	680.8605	879.1434	639.3771	1194.188	1227.85	1296.424
17673	SH2D6	21.91837	0.758194	0.545476	1.389968	0.164539	0.247511	13.55216	15.32753	20.17621	20.08914	34.22577	28.13943
20276	NKX6-1	12.97209	0.7581	0.770832	0.983483	0.32537	0.42661	4.878777	10.40082	13.2189	15.62489	25.66933	8.039836
6245	IQCG	290.3155	0.757143	0.186795	4.053328	5.05E-05	0.000215	208.7033	247.9776	190.6304	319.1942	408.5701	366.8175
2052	SAR1B	3266.794	0.756979	0.096075	7.879023	3.30E-15	4.27E-14	2539.133	2251.505	2498.371	3939.704	4154.153	4217.899
3210	ST3GAL2	1932.31	0.756966	0.123049	6.151756	7.66E-10	6.35E-09	1556.872	1274.922	1481.908	2475.429	2162.641	2642.091
21666	LOC10323	6.707305	0.756961	0.968985	0.78119	0.434691	0.533382	7.047123	4.379295	3.478657	4.464254	12.83466	8.039836
1647	NDFIP1	5485.014	0.756821	0.085447	8.857177	8.21E-19	1.32E-17	3948.015	4290.614	3996.281	6799.059	6997.031	6879.084
20921	LOC11962	8.138647	0.756814	0.852438	0.887822	0.374636	0.476063	5.96295	6.02153	6.261582	6.696381	12.83466	11.05477
3860	GNPDA1	3052.879	0.756413	0.137883	5.485894	4.11E-08	2.83E-07	2549.432	1786.205	2477.499	3863.812	3636.488	4003.838
2469	DBT	1086	0.756129	0.105528	7.165219	7.77E-13	8.36E-12	791.9882	832.6134	798.0039	1370.526	1371.17	1351.697
6662	PTGER4	212.3894	0.756006	0.195212	3.872753	0.000108	0.000429	167.5047	152.1805	155.8438	272.3195	231.024	295.464
19355	LOC10322	15.88493	0.755968	0.671248	1.126214	0.260075	0.357225	8.131296	14.23271	12.52316	31.24978	17.11289	12.05975
5424	FKBP7	452.2841	0.75583	0.169892	4.448885	8.63E-06	4.23E-05	318.7468	399.6106	288.7285	566.9603	616.0639	523.5943
15217	LOC11961	38.67826	0.755484	0.421458	1.792547	0.073045	0.127615	29.27266	21.89647	34.78657	53.57105	51.33866	41.20416
3420	ZNF251	657.8408	0.755224	0.127448	5.925747	3.11E-09	2.42E-08	480.2885	523.8731	464.7486	843.744	787.1927	847.1977
17213	LOC11962	25.61042	0.754988	0.515141	1.465594	0.142759	0.220474	21.68345	22.9913	12.52316	33.4819	27.80844	35.17428
3735	ARID5B	6464.463	0.754728	0.134586	5.60776	2.05E-08	1.46E-07	4222.853	5924.638	4287.097	7785.659	7963.909	8602.624
2905	DDR1	3414.655	0.754507	0.115381	6.539266	6.18E-11	5.66E-10	2639.961	2411.896	2579.772	4042.382	3882.486	4931.434
10779	LOC10321	268.5081	0.754492	0.282212	2.673494	0.007507	0.018511	130.6428	308.7403	160.0182	348.2118	318.7275	344.708
12101	CALR3	87.14598	0.754272	0.316113	2.386081	0.017029	0.037411	44.99317	78.8273	70.26887	109.3742	121.9293	97.48301
22855	LOC11962	4.271014	0.754144	1.289112	0.585011	0.558541	0.649696	2.168345	4.926706	2.087194	11.16063	4.278221	1.004979
16884	LOC10322	30.35567	0.753936	0.49955	1.50923	0.13124	0.206646	21.14137	22.9913	22.95914	29.01765	59.8951	26.12947
15050	LOC11962	36.62072	0.75365	0.413158	1.824118	0.068134	0.120351	27.10432	28.46541	25.74206	46.87467	51.33866	40.19918
15541	RNF227	33.23762	0.753632	0.433692	1.737713	0.082261	0.140719	26.56223	21.34906	26.43779	37.94616	44.92132	42.20914
1614	METTL9	7666.653	0.75259	0.084139	8.944566	3.73E-19	6.15E-18	5785.688	5874.824	5475.406	9424.04	9448.452	9991.506
13305	LOC10322	56.17806	0.752563	0.350707	2.145844	0.031885	0.063711	34.69353	49.81448	41.04815	78.12444	62.03421	71.35354
3005	SNX2	3193.889	0.752297	0.117364	6.409967	1.46E-10	1.29E-09	2219.844	2767.167	2151.897	3946.401	3961.633	4116.396
18852	ELK1	15.76026	0.752129	0.620681	1.211779	0.225597	0.318136	13.55216	10.40082	11.1317	17.85702	23.53022	18.08963
3617	MOSPD2	1838.618	0.75209	0.131459	5.721084	1.06E-08	7.78E-08	1297.755	1628.003	1182.743	2350.43	2282.431	2290.348
20729	LOC10321	10.16098	0.751831	0.822799	0.913749	0.360849	0.46279	3.252518	10.94824	8.348776	13.39276	14.97377	10.04979

10399	LOC119622	119.3034	0.751814	0.271742	2.76665	0.005664	0.014479	80.22878	73.90059	114.0999	142.8561	136.9031	167.8316
19657	TSPAN11	13.54153	0.751747	0.697943	1.077089	0.28144	0.380633	8.131296	9.853413	11.82743	17.85702	23.53022	10.04979
5338	BDKRB2	477.6168	0.751688	0.167186	4.496129	6.92E-06	3.45E-05	356.1507	379.9038	336.0383	524.5498	545.4732	723.5852
13927	LOC119628	70.57177	0.751607	0.370853	2.026697	0.042693	0.081497	72.63957	31.74989	52.87558	91.51721	96.25998	78.3884
12932	IL2RG	81.89655	0.751309	0.339033	2.216032	0.026689	0.054867	60.17159	76.63765	44.52681	111.6063	124.0684	74.36848
5634	GHR	335.5565	0.751304	0.172444	4.356799	1.32E-05	6.23E-05	230.9288	291.2231	228.8956	406.2471	412.8484	443.1959
3073	ATP2B4	3669.609	0.750712	0.119033	6.306755	2.85E-10	2.47E-09	2612.314	3137.217	2460.802	4470.95	4329.56	5006.808
3120	MAPT	724.2331	0.749593	0.119823	6.255853	3.95E-10	3.37E-09	529.0763	564.929	526.6686	881.6902	930.5131	912.5214
1874	EPDR1	4911.45	0.749442	0.090949	8.240229	1.72E-16	2.44E-15	3808.157	3643.026	3544.056	6026.743	5893.25	6553.471
2518	IKBKB	1760.352	0.749402	0.105557	7.09952	1.25E-12	1.32E-11	1366.058	1231.677	1344.849	2229.895	2062.103	2327.532
24654	LOC119618	1.146115	0.74925	2.441511	0.30688	0.758935	0.818378	0	0.547412	2.087194	2.232127	0	2.009959
2388	GLIS3	6647.938	0.749143	0.102776	7.289075	3.12E-13	3.47E-12	4545.936	5546.377	4788.719	8156.192	8171.403	8679.003
16808	SLC35D3	27.41587	0.748694	0.491237	1.524099	0.127484	0.20164	19.51511	22.9913	18.08902	42.41041	36.36488	25.12449
5624	ALKAL1	435.4152	0.748661	0.171625	4.362191	1.29E-05	6.09E-05	280.2587	372.24	319.3407	600.4422	566.8643	473.3453
13416	SH2D4B	107.3694	0.748588	0.352503	2.123635	0.033701	0.066781	85.64965	60.76271	91.83654	151.7846	181.8244	72.35852
8541	EFNA3	128.7806	0.748298	0.230732	3.24315	0.001182	0.00368	92.15468	102.366	93.228	171.8738	158.2942	154.7668
3344	RHBDD2	1186.951	0.748166	0.124689	6.000242	1.97E-09	1.57E-08	932.9306	821.1177	908.6252	1421.865	1366.892	1670.276
2386	SCML1	1783.244	0.747364	0.10247	7.293497	3.02E-13	3.37E-12	1381.236	1377.836	1235.619	2154.003	2267.457	2283.313
23311	C1QTNF5	3.076554	0.747303	1.460282	0.511752	0.608825	0.694333	1.626259	2.737059	2.782925	0	4.278221	7.034856
19920	LOC103239	12.73979	0.746971	0.715325	1.04424	0.296375	0.395529	9.757555	9.853413	8.348776	22.32127	17.11289	9.044815
18174	LOC119626	22.36603	0.746969	0.571587	1.306834	0.191269	0.279789	16.80468	13.6853	18.78475	33.4819	36.36488	15.07469
1818	MGAT2	2494.468	0.746965	0.088858	8.406267	4.23E-17	6.19E-16	1863.693	1864.485	1860.386	3189.709	3043.954	3144.581
19737	LOC103226	13.05168	0.746395	0.70254	1.062424	0.288043	0.387984	11.38381	6.568942	11.1317	22.32127	12.83466	14.06971
2055	STXBP5	2708.035	0.746054	0.094719	7.876527	3.37E-15	4.35E-14	1997.046	2046.773	2029.448	3381.672	3189.414	3603.856
18240	LOC103239	20.0612	0.745513	0.576114	1.294039	0.195652	0.285149	9.757555	15.87494	19.48048	22.32127	27.80844	25.12449
14935	LOC119619	20848.91	0.745205	0.40334	1.847585	0.064662	0.115102	8199.056	23100.23	15443.15	26894.9	27600.94	23855.2
18685	LOC119624	3200.654	0.745115	0.605274	1.231038	0.218309	0.310609	1138.381	4500.82	1536.871	4118.274	3585.149	4324.427
1738	HSPB1	7299.958	0.745012	0.086405	8.62237	6.56E-18	1.00E-16	5152.531	5745.087	5469.84	9325.827	8984.265	9122.199
14376	MYO15B	52.58431	0.744983	0.382324	1.948565	0.051347	0.094955	45.53526	28.46541	43.13534	66.96381	79.14709	52.25893
4748	TRIM24	5778.152	0.744943	0.153718	4.846177	1.26E-06	7.05E-06	3624.932	5636.699	3693.638	7176.288	7279.394	7257.962
3027	LRIG3	1723.487	0.74491	0.116963	6.36878	1.91E-10	1.67E-09	1246.257	1409.038	1212.66	2147.306	1967.982	2357.682
3642	PINK1	1586.811	0.744572	0.130572	5.702375	1.18E-08	8.62E-08	1304.802	1049.936	1208.485	1924.093	1790.436	2243.114
13403	SRCIN1	145.5015	0.744555	0.350046	2.127021	0.033418	0.066286	79.14461	84.84883	160.7139	232.1412	209.6328	106.5278
19625	TRIL	12.98654	0.744543	0.68835	1.081634	0.279415	0.37852	12.46799	8.758589	7.653045	17.85702	17.11289	14.06971
16996	LOC103220	24.89579	0.744539	0.49884	1.49254	0.135558	0.212035	16.80468	19.15941	19.48048	35.71403	32.08666	26.12947
13791	CEP295NL	59.35214	0.744476	0.363527	2.047921	0.040568	0.078203	54.75072	44.88777	34.09084	55.80317	79.14709	87.43321
23192	TRNAC-GC	3.736107	0.74442	1.402839	0.530653	0.595659	0.682805	1.626259	3.831883	2.782925	11.16063	0	3.014938
20039	LOC119625	11.87776	0.744249	0.728634	1.02143	0.307051	0.407281	11.38381	5.474118	9.740239	15.62489	14.97377	14.06971
19789	RCSD1	20.11136	0.743969	0.705302	1.054825	0.291506	0.391615	8.673382	14.23271	21.56767	20.08914	47.06043	9.044815
21378	LOC103228	7.34171	0.743886	0.906759	0.820378	0.412001	0.512363	5.420864	6.568942	4.174388	11.16063	10.69555	6.029877
12180	UBE2L5	100.6528	0.743612	0.313712	2.370366	0.01777	0.038787	68.84497	101.2712	54.26705	131.6955	143.3204	104.5179
17672	LOC119625	22.5765	0.743352	0.534637	1.390386	0.164412	0.247334	21.14137	15.87494	13.2189	29.01765	32.08666	24.11951
2440	DNAJB1	2844.661	0.742851	0.103024	7.210464	5.58E-13	6.08E-12	2115.763	2240.557	2032.231	3504.439	3302.787	3872.186
4065	NPDC1	5564.557	0.742353	0.139489	5.321936	1.03E-07	6.71E-07	4573.041	3328.264	4593.914	6937.451	6184.169	7770.501
9300	BACH2	122.9014	0.742157	0.244753	3.032272	0.002427	0.006938	87.27591	89.77554	97.40239	171.8738	160.4333	130.6473
17420	LOC103223	26.28083	0.741129	0.519784	1.425841	0.153914	0.234892	26.02015	12.59047	20.17621	31.24978	38.50399	29.1444
23418	KLB	2.798665	0.741034	1.492149	0.496622	0.619456	0.70324	3.794605	1.642235	0.695731	2.232127	6.417332	2.009959
20344	LOC119618	11.89436	0.740728	0.763371	0.970339	0.331878	0.433689	10.84173	11.49565	4.174388	20.08914	10.69555	14.06971
18686	LOC103219	20.46757	0.740321	0.601484	1.230823	0.218389	0.310692	9.215468	20.25424	16.00182	35.71403	23.53022	18.08963
21051	LOC119625	8.450595	0.740042	0.848047	0.872642	0.382858	0.483532	6.505036	8.211177	4.174388	8.928508	12.83466	10.04979
12961	LOC119625	60.56154	0.739687	0.334508	2.211265	0.027018	0.055417	52.04029	37.224	46.614	78.12444	77.00798	72.35852
6712	INPP5J	214.5441	0.739678	0.19209	3.850675	0.000118	0.000467	155.5788	180.0985	146.7993	261.1589	265.2497	278.3793
24862	LOC119626	0.862664	0.739313	2.720662	0.27174	0.785822	0.840214	0	0.547412	1.391463	2.232127	0	1.004979
24864	LOC119623	0.862664	0.739313	2.720662	0.27174	0.785822	0.840214	0	0.547412	1.391463	2.232127	0	1.004979
2829	CBLN4	2091.16	0.739121	0.111149	6.649795	2.94E-11	2.76E-10	1661.495	1408.491	1633.577	2658.463	2434.308	2750.629
24871	TRNAA-UG	0.847161	0.739071	2.734904	0.270237	0.786978	0.841146	0	0.547412	1.391463	0	2.139111	1.004979
24872	LOC119625	0.847161	0.739071	2.734904	0.270237	0.786978	0.841146	0	0.547412	1.391463	0	2.139111	1.004979
21084	LOC119627	14.1791	0.738988	0.85194	0.867418	0.385713	0.486349	20.59928	4.379295	6.261582	17.85702	29.94755	6.029877
23511	UTF1	3.08336	0.738828	1.533967	0.481645	0.630058	0.712457	1.084173	4.926706	0.695731	2.232127	8.556443	1.004979
22978	LOC103244	4.202954	0.738597	1.300617	0.567882	0.570115	0.659592	7.047123	1.094824	1.391463	2.232127	6.417332	7.034856
17320	MYOM1	29.38235	0.738183	0.510726	1.445361	0.148357	0.227717	23.30971	22.9913	18.78475	33.4819	55.61688	22.10955
5229	ARHGEF37	400.4971	0.737885	0.161684	4.563747	5.02E-06	2.55E-05	307.9051	308.7403	287.3371	433.0326	494.1346	571.8333
2755	OTUB2	1478.184	0.737515	0.108971	6.767986	1.31E-11	1.26E-10	1177.954	1098.656	1051.25	1828.112	1743.375	1969.76
11557	LOC119625	78.93888	0.737499	0.294579	2.503573	0.012295	0.028282	59.6295	53.64636	65.39875	89.28508	94.12087	111.5527
22447	TRNAT-AG	4.777326	0.73716	1.134824	0.649582	0.515963	0.611078	3.252518	3.284471	4.174388	2.232127	10.69555	5.024897
8428	SH2B2	149.7465	0.736776	0.224988	3.274737	0.001058	0.003336	107.3331	102.9134	125.9274	205.3557	186.1026	170.8465
3342	ARHGAP39	730.8491	0.73635	0.1227	6.001197	1.96E-09	1.56E-08	554.0123	518.399	574.6741	906.2436	877.0354	954.7305
3405	PITPNA	4727.251	0.736107	0.1239	5.941127	2.83E-09	2.21E-08	3217.283	4244.631	3178.101	5792.37	5942.449	5988.673
22379	LOC103231	4.805137	0.735633	1.11193	0.661582	0.508239	0.60376	3.252518	3.831883	3.478657	6.696381	8.556443	3.014938
5266	LYSMD4	340.9917	0.735345	0.161833	4.543863	5.52E-06	2.79E-05	242.8547	249.0724	278.2925	397.3186	417.1266	461.2856
9980	NOVA2	120.8702	0.735154	0.256505	2.866042	0.004156	0.011072	82.93921	82.65918	106.4469	133.9276	177.5462	141.7021
19269	LOC119622	14.68474	0.734876	0.643928	1.141239	0.25377	0.350121	13.01007	10.94824	9.044508	15.62489	21.39111	18.08963
2200	CD109	6151.185	0.734339	0.096576	7.603748	2.88E-14	3.48E-13	4512.327	5028.525	4317.013	7660.66	7362.819	8025.766
1759	WWTR1	15191.63	0.734149	0.085599	8.576577	9.77E-18	1.48E-16	11204.38	12178.27	10840.89	19011.03	18582.45	19332.79

10910	ASTN2	92.35566	0.733972	0.277065	2.649096	0.008071	0.019665	79.14461	61.31012	66.79021	118.3027	124.0684	104.5179
14316	LOC11962!	48.6209	0.733917	0.374733	1.958509	0.05017	0.093167	34.15144	38.86624	37.56949	46.87467	59.8951	74.36848
3425	CDC37L1	1668.763	0.733672	0.123947	5.919241	3.23E-09	2.51E-08	1161.149	1425.46	1170.22	2060.253	2258.901	1936.595
2672	FOXO3	3720.646	0.73366	0.106733	6.873789	6.25E-12	6.22E-11	2717.479	2946.17	2725.876	4453.093	4282.5	5198.759
2479	CDC42SE1	8537.913	0.733256	0.102481	7.155031	8.37E-13	8.97E-12	6057.815	7190.802	5994.421	10586.98	10244.2	11153.26
13203	SPDEF	60.96836	0.732681	0.337906	2.168296	0.030136	0.060681	47.16151	46.53	42.43961	84.82083	85.56443	59.29379
2765	KANSL1L	1346.874	0.73257	0.108572	6.747352	1.51E-11	1.45E-10	1081.462	934.9794	1019.246	1703.113	1679.202	1663.241
24203	LOC11961!	1.425485	0.732552	1.960638	0.373629	0.70868	0.778427	0.542086	0.547412	2.087194	2.232127	2.139111	1.004979
2444	ECE1	10328.77	0.732511	0.101721	7.201201	5.97E-13	6.49E-12	7481.876	8244.569	7563.991	12665.09	11754.41	14262.67
4639	FAIM	1256.379	0.73231	0.148712	4.924359	8.46E-07	4.85E-06	851.0756	1166.535	813.31	1580.346	1606.472	1520.534
4089	JRK	1925.796	0.732235	0.138036	5.304667	1.13E-07	7.34E-07	1658.242	1191.716	1495.127	2357.126	2222.536	2630.031
10710	LOC11962:	121.0095	0.73221	0.272254	2.689437	0.007157	0.017766	82.39713	97.98671	93.228	180.8023	104.8164	166.8266
4465	SHTN1	2668.825	0.731861	0.145414	5.032957	4.83E-07	2.88E-06	1745.518	2519.189	1752.547	3214.263	3476.055	3305.377
4365	ARL8A	1774.252	0.73181	0.143374	5.104203	3.32E-07	2.02E-06	1176.327	1650.447	1173.699	2234.359	2177.615	2233.064
17740	LOC10321	24.74114	0.731527	0.530882	1.377947	0.16822	0.252092	21.14137	12.59047	21.56767	37.94616	32.08666	23.11453
13693	BCAS1	70.55917	0.731526	0.353473	2.069535	0.038496	0.07474	37.94605	65.68942	54.26705	107.1421	91.98176	66.32864
20209	LOC10324!	12.49979	0.731292	0.734791	0.995238	0.319621	0.420462	8.673382	5.474118	13.91463	15.62489	19.252	12.05975
13692	LOC10323:	55.88272	0.730911	0.352994	2.070603	0.038396	0.074551	35.7777	51.45671	38.26523	75.89232	70.59065	63.31371
6023	INPP5D	252.1425	0.730809	0.175926	4.154067	3.27E-05	0.000144	191.3565	202.5424	175.3243	303.5693	314.4493	325.6133
12981	LOC10322!	69.45985	0.73051	0.33094	2.207377	0.027288	0.055885	40.65648	66.78424	49.39693	87.05295	83.42532	89.44317
20717	B4GALNT2	9.686801	0.730163	0.796552	0.916655	0.359324	0.46112	8.131296	6.568942	6.957314	17.85702	8.556443	10.04979
3488	PPP1R14C	738.2591	0.730154	0.124435	5.867779	4.42E-09	3.37E-08	563.2277	546.317	560.0638	863.8331	894.1483	1001.965
19921	LOC11962!	13.5738	0.729845	0.698936	1.044222	0.296383	0.395529	9.757555	13.6853	7.653045	13.39276	12.83466	24.11951
4986	CUNH4orf:	2889.02	0.729844	0.155413	4.696147	2.65E-06	1.41E-05	1846.888	2799.464	1871.517	3729.884	3732.748	3353.616
19733	LOC10324	14.46382	0.729716	0.686015	1.063702	0.287464	0.387293	12.46799	12.59047	6.957314	22.32127	21.39111	11.05477
4930	RAPGEF5	495.9205	0.729611	0.154411	4.72513	2.30E-06	1.24E-05	381.6288	408.9166	329.7767	647.3168	547.6123	660.2715
9832	SLC35C1	136.7672	0.729193	0.251586	2.898382	0.003751	0.010141	121.9694	78.8273	107.8384	171.8738	173.268	166.8266
19012	CUNH10or	17.30781	0.728929	0.616349	1.182655	0.236946	0.331328	10.29964	16.42235	11.82743	24.5534	25.66933	15.07469
3187	METTL7A	950.4321	0.728628	0.117918	6.17908	6.45E-10	5.38E-09	676.5238	775.1351	695.0356	1212.045	1135.868	1207.985
21976	IHH	6.242146	0.72856	0.9956	0.73178	0.464303	0.561699	4.336691	2.737059	6.957314	6.696381	10.69555	6.029877
2622	GTF2B	2374.239	0.728544	0.104881	6.946411	3.75E-12	3.80E-11	1736.845	1673.985	1950.831	2854.89	3129.519	2899.366
9039	LOC11961!	181.5829	0.728174	0.234716	3.102363	0.00192	0.005646	152.3263	106.1979	150.278	263.391	220.3284	196.976
2950	IGSF3	1509.72	0.728102	0.112222	6.488022	8.70E-11	7.84E-10	1146.513	1159.418	1108.996	1816.951	1739.097	2087.342
17741	LOC10324(	25.58538	0.727605	0.528054	1.3779	0.168234	0.2521	11.9259	25.72836	20.17621	35.71403	27.80844	32.15934
4498	LOC11962:	913.63	0.727132	0.145091	5.011564	5.40E-07	3.19E-06	760.5472	612.5538	695.7314	1098.206	1003.243	1311.498
5196	LOC10321!	461.2818	0.726951	0.158663	4.581731	4.61E-06	2.36E-05	382.713	309.2877	352.0401	604.9064	502.691	616.0524
16509	LOC11962:	40.18901	0.726694	0.461806	1.573593	0.115581	0.186125	29.27266	26.82318	34.09084	33.4819	81.28621	36.17926
4200	SRPX2	480.8195	0.726681	0.139285	5.217207	1.82E-07	1.15E-06	378.3763	367.3133	342.2998	591.5136	573.2817	632.1321
21547	REEP1	8.216725	0.726485	0.911215	0.797271	0.425294	0.524734	4.336691	4.379295	9.740239	6.696381	17.11289	7.034856
24577	LOC11962(	1.413177	0.72643	2.273526	0.319517	0.749335	0.810557	0.542086	0	2.782925	0	2.139111	3.014938
20728	TRNAL-AA(	12.31074	0.726249	0.794729	0.913833	0.360805	0.462755	12.46799	3.831883	11.1317	24.5534	12.83466	9.044815
22810	IL5	3.95363	0.725447	1.229055	0.590248	0.555025	0.64688	3.252518	3.831883	2.087194	2.232127	4.278221	8.039836
21952	LOC10321!	7.316524	0.725377	0.985819	0.735812	0.461845	0.559318	5.96295	8.758589	2.087194	4.464254	8.556443	14.06971
9757	ETFRF1	139.2265	0.725157	0.248817	2.91442	0.003564	0.00971	94.32303	122.0728	97.40239	154.0168	211.772	155.7718
23839	LOC10322(	2.843632	0.724971	1.67951	0.431656	0.665991	0.742704	0	1.094824	5.565851	2.232127	2.139111	6.029877
10028	ZNF419	121.1844	0.724836	0.253876	2.855078	0.004303	0.011405	89.44425	100.7238	82.0963	176.338	151.8769	126.6274
3008	SEL1L	8222.802	0.724348	0.113074	6.405957	1.49E-10	1.32E-09	5784.604	7121.28	5699.431	10024.48	9694.45	11012.57
3418	TOM1L2	1299.946	0.724103	0.122193	5.925915	3.11E-09	2.42E-08	1058.695	940.4535	945.4989	1640.613	1439.621	1774.794
17760	LOC11962!	27.82028	0.723788	0.526248	1.375376	0.169015	0.252436	15.17842	24.08612	22.95914	31.24978	51.33866	22.10955
20132	PXDNL	11.64231	0.723733	0.718741	1.006945	0.313961	0.414596	7.589209	10.94824	7.653045	15.62489	14.97377	13.06473
6365	LOC11962	261.5518	0.72344	0.181004	3.996811	6.42E-05	0.000268	201.114	212.9432	179.4987	305.8014	310.171	359.7826
2679	LOC11962(	2150.28	0.722926	0.105375	6.86048	6.86E-12	6.81E-11	1600.239	1722.158	1548.002	2723.195	2459.977	2848.112
24255	LMOD3	1.704854	0.722704	1.96486	0.367815	0.713011	0.781505	1.084173	0.547412	2.087194	2.232127	4.278221	0
2869	EMP3	3273.21	0.722493	0.10968	6.587303	4.48E-11	4.15E-10	2549.974	2288.729	2577.685	4066.935	3670.714	4485.223
6084	LOC10323:	307.7279	0.722354	0.174967	4.128507	3.65E-05	0.00016	253.1543	203.0898	242.1145	386.158	350.8141	411.0366
19689	LOC11962(	16.16314	0.722053	0.673967	1.071348	0.284013	0.383484	7.047123	16.96977	13.2189	15.62489	14.97377	29.1444
18555	KRTCAP3	18.19746	0.72198	0.581069	1.242502	0.214051	0.306686	13.01007	16.42235	11.82743	24.5534	19.252	24.11951
15446	ACOT6	45.0451	0.721171	0.411474	1.75265	0.079662	0.137111	23.30971	33.93953	44.52681	62.49956	57.75599	48.23901
22258	LOC11962:	5.641206	0.720849	1.056879	0.682054	0.495205	0.591473	5.420864	3.284471	4.174388	2.232127	10.69555	8.039836
19359	LOC11962!	19.25147	0.72081	0.64034	1.125667	0.260306	0.357469	7.589209	21.34906	14.61036	15.62489	34.22577	22.10955
9763	FUT4	123.5962	0.720755	0.24741	2.913195	0.003578	0.009742	101.3702	84.30142	93.228	147.3204	179.6853	135.6722
22276	LOC10324	6.242727	0.72058	1.063287	0.677691	0.497968	0.594293	4.878777	6.02153	2.782925	8.928508	12.83466	2.009959
24469	LOC11962	1.690239	0.720415	2.146709	0.33559	0.73718	0.800929	0.542086	1.094824	2.087194	0	6.417332	0
12066	LOC10321	76.05672	0.720211	0.300829	2.394089	0.016662	0.036708	64.50828	55.28859	52.17985	84.82083	109.0946	90.44815
4358	CHRAC1	1192.646	0.71979	0.14092	5.107795	3.26E-07	1.99E-06	807.1666	1087.707	807.7441	1468.74	1510.212	1474.305
21625	LOC10323	9.138216	0.719677	0.915393	0.786195	0.431753	0.530776	5.420864	12.04306	2.782925	17.85702	10.69555	6.029877
24128	LOC11962	1.972804	0.719529	1.863447	0.386128	0.699402	0.770624	1.084173	0.547412	2.782925	0	6.417332	1.004979
22149	LOC10323(	5.987496	0.719408	1.027861	0.699908	0.483985	0.580917	3.794605	2.189647	7.653045	6.696381	8.556443	7.034856
23041	TRNAD-GU	3.980021	0.719133	1.294304	0.555613	0.578475	0.667452	2.168345	6.02153	0.695731	6.696381	4.278221	4.019918
12924	LOC11962(	63.96073	0.718872	0.324253	2.217011	0.026622	0.054763	44.99317	50.36189	50.78839	71.42806	72.72976	93.46309
23817	LOC10324:	2.280504	0.718499	1.653383	0.434563	0.66388	0.741035	0.542086	3.831883	0.695731	4.464254	2.139111	2.009959
16405	HEY1	30.39402	0.718352	0.452457	1.587666	0.112362	0.182087	23.30971	22.9913	22.2634	35.71403	44.92132	33.16432
15848	LOC10322!	40.72892	0.718346	0.424656	1.691596	0.090723	0.152188	36.31979	25.72836	29.22072	58.0353	59.8951	35.17428
17459	PCP2	28.77059	0.718328	0.50676	1.41749	0.15634	0.23806	20.59928	13.6853	31.30791	33.4819	36.36488	37.18424

23068	CUNH17or	3.419874	0.718276	1.303391	0.551082	0.581577	0.670264	2.710432	2.737059	2.087194	6.696381	4.278221	2.009959
4529	DOLK	570.9792	0.718235	0.143775	4.995549	5.87E-07	3.44E-06	418.4907	458.1837	421.6132	712.0485	624.6203	790.9188
16176	LOC119622	44.58887	0.717608	0.439948	1.631119	0.102865	0.169057	34.15144	22.9913	43.83108	40.17829	79.14709	47.23404
5312	KLHDC9	339.6702	0.717526	0.159	4.512748	6.40E-06	3.20E-05	245.5651	255.0939	271.3352	383.9258	444.935	437.1661
4403	LOC11961	553.6342	0.716871	0.141141	5.0791	3.79E-07	2.29E-06	458.6051	384.2831	415.3516	640.6204	697.3501	725.5952
3354	ENG	1468.47	0.716655	0.119657	5.98922	2.11E-09	1.67E-08	1108.567	1153.944	1075.601	1799.094	1621.446	2052.168
22116	LOC103238	6.266866	0.716598	1.01488	0.706092	0.480131	0.577137	4.336691	2.189647	7.653045	6.696381	10.69555	6.029877
5816	SERINC2	316.7591	0.715806	0.167799	4.26584	1.99E-05	9.10E-05	238.518	251.8094	229.5914	426.3363	342.2577	412.0416
17354	LOC103216	59.65939	0.71563	0.497417	1.438693	0.150238	0.230152	48.24569	14.78012	72.35606	71.42806	89.84265	61.30375
16192	LOC119626	35.52229	0.714936	0.439138	1.628044	0.103516	0.169958	22.76763	32.2973	26.43779	35.71403	40.6431	55.27387
3188	KDM4C	914.3383	0.714806	0.115684	6.178954	6.45E-10	5.38E-09	668.9346	741.743	667.2064	1116.063	1127.311	1164.771
17140	LOC103228	27.09269	0.714644	0.484757	1.474232	0.140419	0.217797	16.26259	23.53871	21.56767	37.94616	32.08666	31.15436
21295	LOC119622	10.19341	0.713683	0.855362	0.834364	0.404076	0.504455	5.96295	6.02153	11.1317	4.464254	23.53022	10.04979
18851	LOC103244	22.82377	0.713495	0.588671	1.212044	0.225495	0.31801	12.46799	24.63353	13.91463	33.4819	36.36488	16.07967
2971	LOC103246	3994.792	0.712837	0.110269	6.464509	1.02E-10	9.09E-10	2761.93	3181.01	3144.706	4964.25	4472.88	5443.974
3214	PLEKHG3	1813.307	0.7128	0.115922	6.148958	7.80E-10	6.45E-09	1452.249	1309.409	1365.721	2258.913	2002.208	2491.344
4788	GAB1	1403.921	0.712742	0.147869	4.82009	1.43E-06	7.97E-06	950.2774	1320.905	919.7569	1770.077	1736.958	1725.55
13671	TRNAR-CCI	60.69959	0.712618	0.343506	2.074541	0.038029	0.073952	42.82482	37.77142	57.7457	69.19594	81.28621	75.37346
14440	LOC119622	49.68844	0.712496	0.367957	1.936358	0.052824	0.097246	34.69353	32.84471	45.22254	69.19594	59.8951	56.27885
2182	STOM	7511.446	0.712387	0.093188	7.644583	2.10E-14	2.55E-13	5682.691	5972.263	5430.879	9073.596	8864.475	10044.77
22489	LOC103214	6.041672	0.712151	1.112087	0.640373	0.52193	0.616964	7.589209	1.094824	4.87012	13.39276	4.278221	5.024897
22859	LOC119620	4.229805	0.712016	1.217888	0.584632	0.558795	0.649879	3.794605	4.379295	1.391463	2.232127	8.556443	5.024897
3178	OAF	2810.405	0.711825	0.115043	6.187439	6.11E-10	5.12E-09	2263.753	1971.23	2163.029	3428.547	3129.519	3906.355
9794	LOC103246	148.8924	0.711615	0.244925	2.90544	0.003667	0.009954	90.52842	125.9047	121.0573	216.5163	177.5462	161.8017
20548	LOC11961	12.50726	0.71126	0.754609	0.942554	0.345909	0.447515	13.55216	5.474118	9.044508	13.39276	23.53022	10.04979
2490	PTPRS	4757.784	0.7109	0.099475	7.146547	8.90E-13	9.50E-12	3430.865	3802.87	3597.627	5837.012	5467.567	6410.764
4520	SKI	740.5797	0.710787	0.142119	5.001348	5.69E-07	3.35E-06	499.8036	631.1658	556.5851	933.0291	829.9749	992.9197
23053	LOC119622	3.745324	0.710574	1.283657	0.553554	0.579884	0.668729	1.626259	3.284471	3.478657	8.928508	2.139111	3.014938
22365	LOC119624	5.408143	0.710458	1.069118	0.664527	0.506353	0.601903	5.96295	4.379295	2.087194	6.696381	4.278221	9.044815
19563	LOC103242	14.52656	0.710445	0.652134	1.089416	0.275971	0.375025	11.9259	10.94824	9.740239	20.08914	21.39111	13.06473
19949	LOC103248	15.0769	0.710364	0.684413	1.037917	0.299309	0.398873	8.673382	15.32753	9.740239	17.85702	27.80844	11.05477
7881	CRISPLD2	208.2066	0.709704	0.205921	3.446491	0.000568	0.001916	153.9525	181.7407	138.4505	279.0159	226.7457	269.3345
20593	LOC103231	12.31069	0.70947	0.75576	0.938751	0.347859	0.449076	11.9259	9.853413	6.261582	22.32127	6.417332	17.08465
21299	LOC103231	10.00069	0.709444	0.850611	0.834041	0.404258	0.504593	9.215468	8.758589	4.174388	17.85702	14.97377	5.024897
19143	LOC103217	18.00783	0.708992	0.61009	1.162109	0.245191	0.340493	16.80468	10.40082	13.2189	29.01765	23.53022	15.07469
3141	ABCC1	3400.888	0.708797	0.113812	6.227774	4.73E-10	4.00E-09	2515.823	2726.658	2509.503	4046.846	3792.643	4813.852
2649	BPNT2	5433.701	0.708305	0.102694	6.897261	5.30E-12	5.32E-11	3946.931	4620.156	3811.216	6792.362	6541.4	6890.139
21329	CYP27C1	10.50914	0.708048	0.855186	0.827946	0.407701	0.508169	9.757555	6.02153	7.653045	8.928508	25.66933	5.024897
3447	DIXDC1	1164.874	0.707941	0.119939	5.902516	3.58E-09	2.76E-08	874.9274	945.3802	837.6606	1339.276	1401.117	1590.883
15880	BFSP1	37.576	0.707536	0.419189	1.687869	0.091436	0.153075	32.52518	26.82318	25.74206	42.41041	57.75599	40.19918
4559	MON1A	630.6324	0.707341	0.142419	4.966611	6.81E-07	3.97E-06	472.6993	457.0889	510.6668	810.2621	673.8199	859.2574
16355	LOC103242	32.4743	0.707258	0.443273	1.595536	0.110592	0.179767	26.02015	27.37059	20.17621	40.17829	44.92132	36.17926
3943	MICALL2	1448.919	0.707024	0.130652	5.411519	6.25E-08	4.21E-07	1143.802	948.6647	1212.66	1877.219	1610.75	1900.416
3089	PLCH1	1120.791	0.706972	0.112387	6.290514	3.16E-10	2.72E-09	840.2339	895.0183	822.3545	1312.491	1373.309	1481.34
3762	VSIG10	737.0709	0.706922	0.126619	5.583077	2.36E-08	1.67E-07	610.3893	539.748	530.843	870.5295	926.2349	944.6807
2912	PGAP6	4321.502	0.706529	0.108173	6.531492	6.51E-11	5.94E-10	3436.285	3055.653	3365.948	5272.284	4849.364	5949.478
3437	LOC103223	1416.381	0.706488	0.119479	5.913098	3.36E-09	2.60E-08	1118.324	985.8887	1129.868	1683.024	1638.559	1942.625
17817	LOC103239	24.26792	0.706435	0.514077	1.374182	0.169385	0.252742	17.88885	20.80165	16.00182	35.71403	32.08666	23.11453
2515	STXBP3	2328.252	0.706365	0.099461	7.101958	1.23E-12	1.30E-11	1830.626	1625.266	1852.733	2919.622	2859.991	2881.276
2955	S100A11	10046.32	0.705812	0.10884	6.48483	8.88E-11	7.99E-10	7076.938	8801.287	7031.757	12636.07	12090.25	12641.64
21921	LOC119627	8.203908	0.705511	0.952177	0.740945	0.458727	0.556327	6.505036	7.663765	4.174388	4.464254	21.39111	5.024897
18522	LOC103232	21.67577	0.705192	0.565279	1.247513	0.21221	0.304589	13.55216	15.87494	19.48048	24.5534	38.50399	18.08963
4564	MAP7	1919.38	0.704762	0.141981	4.963778	6.91E-07	4.03E-06	1301.007	1801.532	1276.667	2343.733	2340.187	2453.155
4972	EXOSC4	915.6732	0.704698	0.149889	4.701469	2.58E-06	1.38E-05	750.2475	577.5195	765.3045	1087.046	1039.608	1274.314
5454	PRR14	476.3762	0.704659	0.158784	4.437849	9.09E-06	4.43E-05	312.2417	420.9597	353.4315	580.353	605.3683	585.903
3180	FSCN1	4243.894	0.704631	0.113934	6.184567	6.23E-10	5.21E-09	3398.882	2875.554	3412.562	5401.747	4697.487	5677.129
20342	HPX	13.81949	0.704464	0.725754	0.970665	0.331715	0.433519	10.29964	8.758589	11.82743	29.01765	14.97377	8.039836
2838	MPP1	1564.136	0.704431	0.106195	6.633351	3.28E-11	3.07E-10	1202.89	1205.948	1164.655	1810.255	1901.669	2099.402
2190	CFLAR	2918.016	0.704075	0.092347	7.624216	2.46E-14	2.98E-13	2311.998	2212.639	2137.287	3555.778	3516.698	3773.698
22933	LOC11961	4.27359	0.703893	1.226399	0.573951	0.566001	0.656092	4.336691	1.094824	4.174388	4.464254	8.556443	3.014938
19778	MCOLN3	15.09807	0.703776	0.666159	1.056469	0.290754	0.390823	10.84173	12.59047	10.43597	17.85702	27.80844	11.05477
4943	PRKCD	946.0706	0.703704	0.149149	4.718138	2.38E-06	1.28E-05	671.1029	863.2684	626.854	1113.831	1123.033	1278.334
22323	TMEM89	5.710483	0.703683	1.04844	0.671172	0.502111	0.597976	2.710432	6.568942	3.478657	8.928508	8.556443	4.019918
23976	LOC119622	2.247723	0.703672	1.713829	0.410585	0.681377	0.755522	1.626259	2.737059	0.695731	0	6.417332	2.009959
5711	LOC103235	349.6444	0.703438	0.16284	4.319812	1.56E-05	7.27E-05	235.8076	293.4127	269.248	441.9611	419.2657	438.171
7573	CYB561D1	282.6953	0.703191	0.198966	3.534227	0.000409	0.001436	258.0331	179.0037	209.4151	305.8014	370.0661	373.8524
2918	CREBRF	2154.479	0.703175	0.107718	6.527923	6.67E-11	6.08E-10	1593.192	1819.597	1504.867	2645.07	2705.975	2658.171
19237	LOC119627	17.07863	0.703038	0.612998	1.146884	0.251429	0.347469	12.46799	16.42235	10.43597	15.62489	21.39111	26.12947
17889	LOC119621	23.70798	0.702764	0.515876	1.362275	0.173111	0.257262	18.43094	14.23271	21.56767	26.78552	32.08666	29.1444
18551	LOC103240	27.65704	0.701787	0.56459	1.243003	0.213867	0.306487	27.6464	15.87494	18.78475	26.78552	57.75599	19.09461
11182	LOC103240	400.9388	0.701543	0.271377	2.585124	0.009734	0.023143	374.5817	233.1974	304.7303	522.3177	682.3763	288.4291
20253	LOC119621	15.14821	0.701486	0.710818	0.986871	0.323706	0.42489	16.26259	4.379295	13.91463	17.85702	21.39111	17.08465
18881	WDR86	22.16307	0.701274	0.581189	1.20662	0.227578	0.320437	15.7205	11.49565	22.95914	40.17829	23.53022	19.09461
4392	RALGPS1	588.7753	0.701176	0.137935	5.083368	3.71E-07	2.24E-06	464.0259	452.1622	432.7449	683.0309	686.6545	814.0334

3300	KCTD7	991.2951	0.701068	0.116021	6.042589	1.52E-09	1.22E-08	760.0051	789.3678	717.299	1136.153	1270.632	1274.314
2709	HPS3	2245.83	0.700942	0.102707	6.824685	8.81E-12	8.65E-11	1661.495	1862.842	1609.227	2714.266	2750.896	2876.251
16077	LOC10324€	37.34106	0.700624	0.42415	1.651831	0.098569	0.162984	35.23561	26.27577	23.65487	40.17829	53.47777	45.22408
8547	GSTO1	950.4133	0.70017	0.216002	3.241495	0.001189	0.003698	911.2472	434.645	827.2246	1187.492	1152.981	1188.891
7990	LOC10324	205.5609	0.699993	0.204968	3.415137	0.000637	0.002121	153.9525	136.853	180.8902	249.9982	235.3022	276.3694
3776	INF2	2635.615	0.699936	0.125689	5.56877	2.57E-08	1.81E-07	2090.827	1744.601	2195.728	3122.746	2992.616	3667.17
23383	LOC10323	4.336416	0.699761	1.39529	0.501517	0.616008	0.700389	0	4.926706	4.87012	8.928508	4.278221	3.014938
20091	RINL	14.24568	0.699292	0.689606	1.014046	0.310561	0.410943	14.09425	7.116354	11.1317	13.39276	25.66933	14.06971
2588	TBC1D2B	1970.696	0.69898	0.099843	7.000757	2.55E-12	2.62E-11	1581.808	1515.236	1411.639	2370.519	2421.473	2523.503
9469	LOC10322	124.0572	0.698773	0.23341	2.993759	0.002756	0.007737	99.74389	88.68071	95.3152	149.5525	158.2942	152.7569
21042	LOC11962	10.25301	0.698597	0.798646	0.874727	0.381722	0.482275	6.505036	6.568942	10.43597	6.696381	19.252	12.05975
20403	LOC11962	15.74074	0.698373	0.72704	0.96057	0.336768	0.438807	8.673382	12.04306	14.61036	17.85702	34.22577	7.034856
6058	ZNF471	325.0642	0.698239	0.168587	4.141701	3.45E-05	0.000151	235.2655	261.6628	249.0718	348.2118	414.9875	441.186
5927	HCN3	332.6178	0.698188	0.16633	4.197616	2.70E-05	0.000121	273.7536	261.6628	226.1127	377.2295	427.8221	429.1262
3127	MFGE8	1146.782	0.69816	0.11178	6.245847	4.22E-10	3.58E-09	912.8734	854.5098	858.5325	1453.115	1311.275	1490.385
18308	LOC11962(	21.39494	0.697982	0.544894	1.28095	0.200211	0.290726	14.09425	18.612	16.00182	22.32127	34.22577	23.11453
12685	LOC10324	115.4529	0.697801	0.308415	2.262536	0.023664	0.049595	105.1648	54.19377	105.7512	133.9276	136.9031	156.7768
6624	NXF3	250.2245	0.697742	0.179291	3.891682	9.96E-05	0.0004	207.6191	182.2881	183.6731	312.4978	286.6408	328.6283
4202	ZMYND8	4367.258	0.697521	0.133716	5.216423	1.82E-07	1.15E-06	2936.482	4090.808	2967.99	5287.909	5330.664	5589.696
16954	LOC11962(	39.9342	0.697113	0.465073	1.498932	0.133891	0.20995	27.6464	39.41365	22.95914	66.96381	53.47777	29.1444
2404	GAS1	4512.161	0.696877	0.095897	7.26691	3.68E-13	4.07E-12	3413.518	3672.586	3244.891	5696.388	5240.821	5804.761
3473	VASN	1380.404	0.696789	0.118372	5.886423	3.95E-09	3.02E-08	1022.917	1023.113	1115.257	1868.29	1510.212	1742.634
18207	CUNH9orf	21.99177	0.696674	0.535705	1.300481	0.193436	0.282447	18.43094	14.23271	17.39328	24.5534	34.22577	23.11453
24073	LOC11962	2.313982	0.696656	1.767383	0.394173	0.693453	0.765814	2.710432	1.642235	0.695731	6.696381	2.139111	0
19155	LOC11962	16.60285	0.696655	0.600807	1.159531	0.24624	0.341753	11.38381	12.04306	14.61036	20.08914	21.39111	20.09959
5685	CLCN7	2115.447	0.696528	0.160738	4.333297	1.47E-05	6.87E-05	1850.683	1233.319	1763.679	2466.5	2344.465	3034.033
22367	LOC10321€	5.443525	0.696419	1.048171	0.664413	0.506426	0.601928	2.168345	5.474118	4.87012	6.696381	6.417332	7.034856
22738	HSPB2	5.795543	0.696375	1.155207	0.602814	0.546632	0.639116	3.794605	3.284471	6.261582	13.39276	0	8.039836
2736	PLPP5	1826.167	0.69626	0.102386	6.800352	1.04E-11	1.01E-10	1451.707	1435.861	1295.452	2189.717	2239.649	2344.617
13946	GAB2	61.24485	0.696206	0.344153	2.022955	0.043078	0.082118	39.5723	55.83601	43.83108	80.35657	85.56443	62.30873
2240	MALT1	12111.25	0.696074	0.092415	7.532081	4.99E-14	5.93E-13	9070.731	9890.089	8778.043	14490.97	14462.53	15975.15
20230	LOC11962	15.21389	0.695985	0.702513	0.990707	0.321829	0.422927	7.047123	12.59047	14.61036	22.32127	25.66933	9.044815
3387	MBD5	1309.842	0.695977	0.116887	5.954291	2.61E-09	2.05E-08	963.2875	1109.056	926.7142	1687.488	1544.438	1628.067
3799	CERCAM	1341.344	0.695751	0.125477	5.544832	2.94E-08	2.06E-07	1084.173	950.8543	1042.206	1618.292	1480.265	1872.277
7206	LOC11962	322.8589	0.695708	0.189863	3.664259	0.000248	0.000915	291.6425	210.7535	239.3316	357.1403	385.0399	453.2457
6468	COQ8B	860.0063	0.695344	0.175948	3.951986	7.75E-05	0.000319	748.6213	505.2611	720.082	917.4042	1022.495	1246.175
11541	LOC11961	83.3371	0.695258	0.277516	2.505286	0.012235	0.028181	62.33993	62.40495	66.09448	107.1421	100.5382	101.5029
3021	HGSNAT	2301.904	0.695245	0.108845	6.387483	1.69E-10	1.48E-09	1910.854	1614.317	1751.852	2714.266	2768.009	3052.123
9431	TRNAL-UA	162.5113	0.694895	0.231584	3.000613	0.002694	0.007595	137.6899	101.2712	134.9719	194.195	181.8244	225.1154
4233	VWA5A	753.9112	0.694786	0.133676	5.197535	2.02E-07	1.27E-06	629.3623	532.6317	568.4125	852.6725	928.374	1012.014
3024	LOC10324	9205.355	0.693694	0.108776	6.377285	1.80E-10	1.58E-09	7534.458	6606.166	6967.054	11256.62	10145.8	12722.04
2441	SLC4A7	7382.041	0.693647	0.096228	7.208398	5.66E-13	6.17E-12	5714.675	6042.332	5167.893	8852.616	8905.118	9609.614
12442	COL15A1	81.01468	0.693321	0.29989	2.311917	0.020782	0.044402	58.00324	73.90059	52.87558	104.91	106.9555	89.44317
22256	LOC11962	6.326991	0.693313	1.016129	0.682308	0.495044	0.591335	3.794605	5.474118	5.565851	8.928508	2.139111	12.05975
7453	LOC10322	367.3152	0.693299	0.193655	3.580077	0.000343	0.001225	233.6392	347.6065	259.5078	515.6213	421.4048	426.1113
23366	LOC11962	3.132444	0.693142	1.375631	0.503872	0.614351	0.698987	3.252518	1.094824	2.782925	2.232127	6.417332	3.014938
21788	LOC11961	8.617386	0.693109	0.910323	0.761388	0.446425	0.544717	3.794605	3.831883	12.52316	8.928508	8.556443	14.06971
5174	CPEB2	823.8381	0.693056	0.150939	4.591636	4.40E-06	2.26E-05	555.0964	758.1654	576.7613	1026.778	951.9042	1074.323
6997	LFNG	2848.043	0.692858	0.185487	3.735349	0.000187	0.000712	2597.678	1446.262	2490.023	3339.262	3217.222	3997.808
5114	TSHZ1	607.8259	0.692684	0.149884	4.621484	3.81E-06	1.98E-05	413.6119	532.6317	450.1382	705.3521	725.1585	820.0632
16702	ERVMER34	39.01159	0.692608	0.449091	1.542247	0.123014	0.195804	31.9831	36.67659	20.87194	31.24978	62.03421	51.25395
6476	KIAA0825	236.6431	0.692567	0.175399	3.948525	7.86E-05	0.000323	178.8885	183.9304	179.4987	301.3371	288.7799	287.4241
13578	FMO5	61.35874	0.692565	0.330912	2.0929	0.036358	0.071187	46.07734	41.05589	54.26705	75.89232	68.45154	82.40832
24383	S100A7	1.734544	0.692467	1.98291	0.349217	0.726926	0.792541	0	0.547412	3.478657	2.232127	2.139111	2.009959
22520	LOC10324(	6.949291	0.69219	1.085665	0.637572	0.523752	0.618293	1.626259	4.926706	9.044508	13.39276	10.69555	2.009959
15719	LOC10323	56.61998	0.692121	0.403555	1.715061	0.086334	0.146014	25.47806	58.02565	45.91827	84.82083	64.17332	61.30375
6376	ZBTB42	585.7891	0.691552	0.173273	3.991117	6.58E-05	0.000274	482.4569	368.9556	496.7522	689.7272	624.6203	852.2226
3540	B3GALNT1	1069.302	0.691397	0.118907	5.814622	6.08E-09	4.56E-08	839.6918	733.5318	880.1002	1345.973	1324.11	1292.404
19746	LOC10322	19.94075	0.691102	0.651382	1.060977	0.2887	0.388691	11.9259	20.80165	12.52316	13.39276	44.92132	16.07967
2699	DENND6A	2885.712	0.690278	0.100991	6.835014	8.20E-12	8.08E-11	2136.362	2400.948	2089.281	3566.939	3403.325	3717.419
16419	LOC11962	37.99404	0.689773	0.434813	1.586366	0.112656	0.182409	29.81475	36.12918	20.87194	40.17829	57.75599	43.21412
4380	KCTD21	640.9576	0.68956	0.135399	5.092813	3.53E-07	2.14E-06	495.4669	509.6404	468.9229	696.4236	832.114	843.1778
6959	ANG	334.4316	0.68947	0.183705	3.75314	0.000175	0.000667	264.5381	210.7535	291.5114	455.3539	408.5701	375.8623
2037	TRIM38	3009.683	0.689438	0.087297	7.897599	2.84E-15	3.71E-14	2375.422	2289.824	2246.517	3718.724	3692.105	3735.509
3233	TSC22D3	2956.653	0.689364	0.11244	6.130934	8.74E-10	7.18E-09	2449.688	2048.415	2296.609	3609.349	3354.126	3981.729
4792	CASP8AP2	2089.735	0.689065	0.143017	4.818051	1.45E-06	8.04E-06	1433.276	1975.062	1391.463	2421.858	2686.723	2630.031
10609	LOC11962(	107.8435	0.688896	0.253336	2.719301	0.006542	0.016394	74.26583	81.01695	92.53227	131.6955	136.9031	130.6473
2647	LIMD1	2600.622	0.688617	0.099763	6.902535	5.11E-12	5.13E-11	2004.635	2046.773	1927.872	3120.514	3024.702	3479.239
6120	LOC11962	526.7032	0.688434	0.167473	4.110719	3.94E-05	0.000171	448.3054	326.8049	436.2236	691.9594	577.5599	679.3661
3490	ADGRL1	1151.413	0.688289	0.117339	5.865795	4.47E-09	3.40E-08	826.1396	913.0829	910.7124	1319.187	1394.7	1544.653
14683	FYB1	63.06582	0.688252	0.363709	1.892313	0.058449	0.105828	31.44101	61.31012	52.17985	73.66019	83.42532	76.37844
1875	FDFT1	6848.794	0.688211	0.083524	8.239644	1.73E-16	2.45E-15	5158.494	5416.64	5164.414	8415.119	8175.681	8762.416
20813	LOC11962	12.07357	0.687886	0.762051	0.902676	0.366698	0.468348	8.673382	4.926706	13.91463	15.62489	19.252	10.04979
24552	LOC11962€	1.73477	0.687837	2.129745	0.322967	0.74672	0.808552	2.710432	1.094824	0	4.464254	2.139111	0

3293	TFPI	4973.124	0.687829	0.113719	6.04848	1.46E-09	1.18E-08	3538.198	4427.467	3462.655	6151.742	6057.961	6200.723
9905	PWWP2B	216.3537	0.687604	0.238427	2.883917	0.003928	0.010542	179.9727	126.9995	192.0219	292.4086	203.2155	303.5038
15198	TRNAD-GU	65.1276	0.687578	0.382764	1.79635	0.072439	0.126713	44.99317	33.93953	70.9646	100.4457	62.03421	78.3884
18452	LOC10323:	24.48699	0.687464	0.547014	1.256758	0.208841	0.300891	17.34676	12.04306	27.13352	35.71403	23.53022	31.15436
3056	RHPN2	1881.579	0.687463	0.108575	6.331674	2.43E-10	2.11E-09	1516.758	1415.06	1397.724	2245.52	2164.78	2549.633
14728	AMHR2	68.34102	0.687447	0.364878	1.884045	0.059559	0.107508	66.67662	34.48694	54.96278	87.05295	100.5382	66.32864
20297	LOC10324:	12.35828	0.687034	0.702244	0.978342	0.327905	0.42947	9.215468	10.94824	8.348776	17.85702	10.69555	17.08465
12875	LOC10324:	77.70265	0.686843	0.30891	2.223441	0.026186	0.054073	59.6295	51.45671	66.79021	89.28508	117.6511	81.40334
2661	PRKACB	3112.109	0.686455	0.099788	6.879115	6.02E-12	6.02E-11	2307.662	2607.322	2242.342	3749.973	3809.756	3955.599
7021	LOC10322:	457.1988	0.686168	0.18413	3.726551	0.000194	0.000735	325.2518	419.3174	303.3389	658.4775	560.447	476.3603
5809	VKORC1	1044.259	0.685922	0.160671	4.269121	1.96E-05	8.98E-05	907.9947	610.3642	885.666	1270.08	1208.598	1382.852
4351	RSU1	2357.943	0.685888	0.134046	5.116801	3.11E-07	1.90E-06	1566.088	2189.1	1666.972	2977.657	2885.66	2862.182
4447	FZD1	736.4016	0.685287	0.13585	5.044459	4.55E-07	2.72E-06	578.9482	532.0843	587.893	857.1368	834.2532	1028.094
22866	MHC-DQB:	5.474566	0.685277	1.174496	0.583465	0.55958	0.650564	8.673382	2.189647	1.391463	11.16063	6.417332	3.014938
3564	RMDN3	2317.324	0.685227	0.118365	5.789082	7.08E-09	5.28E-08	1959.1	1612.675	1764.375	2723.195	2673.888	3170.71
23469	TRNAV-AA	2.867158	0.685074	1.399196	0.48962	0.624403	0.707305	1.626259	2.737059	2.087194	4.464254	4.278221	2.009959
23316	LOC11962:	3.687621	0.685001	1.339682	0.511316	0.60913	0.694533	3.794605	3.831883	0.695731	2.232127	8.556443	3.014938
16903	INKA1	40.14476	0.684934	0.454862	1.505807	0.132117	0.207793	41.74065	18.612	32.69937	46.87467	40.6431	60.29877
7578	LOC11962:	1873.351	0.684718	0.193779	3.533493	0.00041	0.001439	1135.129	2042.393	1132.651	2310.251	2310.24	2309.443
18792	LOC10323:	19.55686	0.68433	0.561252	1.219291	0.222734	0.315124	16.80468	14.23271	13.91463	29.01765	19.252	24.11951
3226	IL4R	3200.113	0.684122	0.111508	6.135191	8.51E-10	7.01E-09	2412.826	2568.456	2390.533	3868.276	3514.559	4446.029
2862	ANKRD52	4738.926	0.683921	0.103721	6.593869	4.29E-11	3.98E-10	3431.949	3895.93	3586.495	5620.496	5471.845	6426.844
3820	FASTKD5	912.488	0.683505	0.123693	5.525801	3.28E-08	2.28E-07	699.2914	741.743	663.032	1026.778	1123.033	1221.05
22429	LOC11962:	5.702422	0.683353	1.048288	0.651876	0.514481	0.609813	3.794605	4.926706	4.174388	4.464254	12.83466	4.019918
5888	TACC2	1430.318	0.683135	0.161854	4.220684	2.44E-05	0.00011	968.1663	1403.564	923.2355	1705.345	1662.089	1919.511
2742	UBE2V2	1949.827	0.683104	0.100639	6.787632	1.14E-11	1.11E-10	1404.004	1584.21	1502.084	2323.644	2477.09	2407.931
5255	CD68	2121.992	0.683066	0.150095	4.550903	5.34E-06	2.70E-05	1408.882	2047.868	1431.119	2562.482	2470.673	2810.928
3841	DZIP1L	697.8527	0.682815	0.124083	5.502873	3.74E-08	2.59E-07	553.4702	527.705	528.7558	816.9585	840.6705	919.5562
2378	ESYT2	9279.035	0.682753	0.093477	7.303974	2.79E-13	3.12E-12	7095.368	7526.365	6753.464	10932.96	11057.06	12308.99
7236	LOC11962:	425.5362	0.682479	0.186661	3.656245	0.000256	0.00094	300.3158	369.503	313.7748	526.782	410.7092	632.1321
22623	COX6A2	4.860232	0.682224	1.098385	0.621115	0.534524	0.628137	2.710432	4.926706	3.478657	4.464254	8.556443	5.024897
3792	PLEKHA5	10883.27	0.682202	0.122873	5.552078	2.82E-08	1.98E-07	7757.798	10020.37	7291.265	13225.35	13782.29	13222.51
5486	PSMB10	1585.964	0.682127	0.15448	4.415636	1.01E-05	4.88E-05	1374.189	915.82	1363.633	2029.003	1916.643	1916.496
20172	LOC10324:	21.4226	0.682029	0.681541	1.000715	0.316965	0.417733	16.26259	15.32753	16.69755	35.71403	38.50399	6.029877
18077	LOC10323:	33.13811	0.682001	0.513553	1.328005	0.184177	0.27086	20.59928	27.37059	27.13352	46.87467	57.75599	19.09461
17918	LOC10322:	26.17737	0.68192	0.502213	1.357829	0.174518	0.258941	14.09425	21.89647	24.3506	33.4819	32.08666	31.15436
20966	LOC10323:	10.92353	0.681818	0.771908	0.883289	0.37708	0.47814	10.29964	4.379295	10.43597	13.39276	14.97377	12.05975
6116	TRIM3	478.4385	0.681207	0.165688	4.111375	3.93E-05	0.000171	354.5245	337.2057	415.3516	544.639	530.4994	688.4109
16184	LOC10321:	37.05162	0.681003	0.418046	1.629016	0.10331	0.169694	23.30971	31.20247	30.61218	44.64254	51.33866	41.20416
23364	LOC11962:	3.460556	0.680947	1.34938	0.504637	0.613814	0.698435	0.542086	3.284471	4.174388	4.464254	4.278221	4.019918
23415	LOC10323:	3.163909	0.680907	1.370465	0.496844	0.619299	0.703142	1.084173	2.189647	4.174388	2.232127	4.278221	5.024897
2422	NDRG1	18620.73	0.680852	0.094044	7.239714	4.50E-13	4.94E-12	14401.61	15067.51	13455.44	22609.21	21451	24739.58
14913	LOC11962:	61.25171	0.680805	0.367726	1.851389	0.064114	0.114294	39.03022	63.49977	37.56949	84.82083	81.28621	61.30375
3737	LOC10324:	4864.039	0.6808	0.121446	5.605787	2.07E-08	1.47E-07	3396.713	4360.135	3457.089	6040.136	5510.349	6419.809
2914	LDB1	2092.535	0.680605	0.104232	6.529736	6.59E-11	6.01E-10	1504.832	1728.727	1592.529	2598.196	2438.586	2692.34
5333	LMLN	519.6862	0.680394	0.151142	4.501696	6.74E-06	3.36E-05	436.9216	395.7787	368.0419	631.6919	571.1425	714.5404
5047	PCYOX1L	535.4268	0.680193	0.145905	4.661892	3.13E-06	1.65E-05	388.6759	460.9207	386.1309	671.8702	607.5074	697.4558
6423	FAH	442.0103	0.680038	0.171257	3.970876	7.16E-05	0.000296	348.5615	284.1067	389.6096	520.0856	502.691	607.0076
18011	LOC11961:	42.63805	0.679808	0.506887	1.341144	0.179874	0.265488	41.19856	22.44388	33.39511	49.10679	85.56443	24.11951
13281	LOC11961:	87.23436	0.679242	0.315712	2.15146	0.03144	0.062934	76.97626	47.62483	77.22618	118.3027	87.70354	115.5726
14949	PROM2	61.48413	0.679163	0.368012	1.845491	0.064966	0.11553	47.7036	42.69812	50.09266	69.19594	106.9555	52.25893
11316	SNAP25	107.2555	0.679143	0.265868	2.554439	0.010636	0.024987	84.02339	82.11177	80.70484	109.3742	164.7115	122.6075
6033	TLR3	1053.187	0.679107	0.163665	4.149371	3.33E-05	0.000147	685.1972	1026.945	718.6905	1227.67	1255.658	1404.961
5922	HPS1	940.3143	0.678921	0.161623	4.200644	2.66E-05	0.000119	834.2709	595.0366	743.7368	1127.224	998.9647	1342.653
3122	CLN5	1358.024	0.678669	0.108554	6.251923	4.05E-10	3.45E-09	1053.274	1042.272	1034.553	1828.112	1600.055	1589.878
24437	LOC11962:	1.444867	0.678552	1.998228	0.339577	0.734175	0.798709	1.084173	1.642235	0.695731	2.232127	0	3.014938
4408	ADAMTSL5	596.3966	0.67841	0.133689	5.074542	3.88E-07	2.34E-06	456.9788	441.7613	480.7504	714.2806	686.6545	797.9537
3323	PM20D2	1933.146	0.678364	0.112654	6.021668	1.73E-09	1.38E-08	1459.839	1660.847	1339.283	2412.929	2323.074	2402.906
2426	TKT	62391.4	0.678347	0.09378	7.233426	4.71E-13	5.16E-12	47855.38	49917.94	46195.87	75644.55	70791.73	83942.92
5614	LOC10322:	983.5374	0.678207	0.155367	4.365208	1.27E-05	6.01E-05	855.4123	598.3211	818.1801	1223.206	1101.642	1304.463
17226	ADAM22	36.81083	0.678063	0.463347	1.463402	0.143357	0.221244	19.51511	30.65506	34.09084	42.41041	62.03421	32.15934
19017	LOC10321:	23.2843	0.677812	0.573724	1.181426	0.237433	0.331922	14.63633	20.80165	17.39328	29.01765	42.78221	15.07469
3167	STMN3	2977.522	0.677765	0.109322	6.1997	5.66E-10	4.75E-09	2382.47	2173.772	2321.656	3624.974	3292.091	4070.167
4550	GAA	3258.646	0.677716	0.136289	4.972645	6.60E-07	3.86E-06	2768.435	2046.773	2709.874	4006.668	3585.149	4434.974
17664	LOC10324:	28.12276	0.677169	0.485879	1.393701	0.163408	0.245935	19.51511	21.34906	23.65487	29.01765	47.06043	28.13943
19130	LOC10323:	21.78159	0.677141	0.581596	1.16428	0.24431	0.339519	17.34676	23.53871	9.044508	24.5534	32.08666	24.11951
3682	TRPM6	788.6767	0.676882	0.119469	5.665758	1.46E-08	1.06E-07	604.4263	641.5666	576.0656	953.1182	951.9042	1004.979
6912	AP4S1	316.9622	0.676736	0.179457	3.771027	0.000163	0.000625	250.986	256.7361	220.5468	433.0326	412.8484	327.6233
4103	RAB2A	4637.669	0.676692	0.127833	5.29358	1.20E-07	7.77E-07	3137.596	4304.847	3264.372	5694.156	5899.667	5525.377
21446	LOC10322:	10.85287	0.676609	0.83311	0.812148	0.416706	0.51656	8.131296	13.13788	3.478657	8.928508	21.39111	10.04979
9570	LOC10323:	149.6185	0.676523	0.227989	2.96735	0.003004	0.008344	117.0907	102.366	125.2316	212.0521	171.1289	169.8415
6112	LOC11962:	318.1677	0.676479	0.164431	4.114063	3.89E-05	0.000169	247.7335	257.831	230.9828	381.6937	363.6488	427.1163
4689	ANO8	789.8727	0.676431	0.138285	4.891581	1.00E-06	5.67E-06	627.1939	582.4462	618.5052	988.8323	827.8358	1094.423
7618	LOC10322:	1355.491	0.675543	0.191881	3.52064	0.000431	0.001502	1279.324	685.3596	1168.133	1674.095	1557.273	1768.764

3846	NAA60	2013.028	0.675231	0.122759	5.500478	3.79E-08	2.62E-07	1486.401	1692.05	1477.733	2412.929	2201.145	2807.913
19248	KLHDC8A	24.4747	0.675147	0.589608	1.145078	0.252177	0.348308	19.51511	15.87494	20.17621	29.01765	49.19955	13.06473
3899	ABHD4	2043.851	0.674841	0.12394	5.444884	5.18E-08	3.53E-07	1523.805	1701.356	1503.475	2430.786	2220.397	2883.286
20750	FCER1G	11.74968	0.674425	0.740738	0.910478	0.362571	0.464437	7.047123	11.49565	8.348776	11.16063	21.39111	11.05477
3600	SHISA2	1583.056	0.674408	0.117442	5.742483	9.33E-09	6.89E-08	1209.395	1293.534	1161.176	1825.88	1822.522	2185.83
18601	LOC10323ε	21.83923	0.674388	0.545737	1.235738	0.216556	0.309507	14.09425	21.34906	14.61036	26.78552	32.08666	22.10955
9024	LETM2	273.9915	0.673932	0.216781	3.108814	0.001878	0.005534	190.8144	270.4214	170.4542	328.1227	378.6226	305.5138
20721	LOC11961ᶜ	11.54763	0.673826	0.736134	0.915357	0.360004	0.461884	10.29964	7.663765	8.348776	20.08914	12.83466	10.04979
8667	RCBTB2	187.0973	0.673528	0.209663	3.212431	0.001316	0.004037	156.663	131.9262	146.1036	203.1236	222.4675	262.2996
14127	STPG1	58.92198	0.673386	0.33861	1.988676	0.046737	0.087952	49.32986	42.69812	45.22254	60.26743	70.59065	85.42325
20109	CD27	17.47729	0.672946	0.66548	1.011219	0.311912	0.412361	11.38381	12.04306	16.69755	11.16063	38.50399	15.07469
3758	MFAP3L	1368.546	0.672728	0.120457	5.584796	2.34E-08	1.66E-07	980.6342	1192.263	994.2001	1622.756	1655.672	1765.749
22986	MB	4.623617	0.67266	1.187588	0.566409	0.571116	0.660538	5.420864	3.831883	1.391463	8.928508	2.139111	6.029877
4062	LOC10324₇	1824.056	0.672172	0.126274	5.323139	1.02E-07	6.68E-07	1609.997	1224.56	1387.984	2113.824	2216.119	2391.851
16425	LOC10322₁	42.94465	0.672047	0.423906	1.585368	0.112883	0.182708	31.9831	22.9913	45.22254	49.10679	47.06043	61.30375
22177	ADIG	6.932759	0.671993	0.966599	0.695214	0.486921	0.583726	4.336691	7.116354	4.174388	13.39276	8.556443	4.019918
16228	LOC11961ε	38.89856	0.671824	0.414307	1.621559	0.104898	0.171835	33.06727	29.56024	26.43779	55.80317	51.33866	37.18424
10764	LOC11962ϵ	117.3886	0.671559	0.25093	2.67628	0.007444	0.018385	78.60252	95.24966	98.09812	127.2312	160.4333	144.717
3104	LY6E	4064.142	0.671231	0.106992	6.273665	3.53E-10	3.02E-09	3239.508	2900.735	3269.937	5176.302	4457.907	5340.461
2771	CYP2U1	1899.07	0.671202	0.099567	6.741189	1.57E-11	1.51E-10	1543.32	1450.094	1403.986	2312.484	2252.484	2432.05
27080	LOC10323!	0.348192	0.671062	4.040349	0.16609	0.868086	NA	1.084173	0	0	0	0	1.004979
27500	LOC11962ϵ	0.348192	0.671062	4.040349	0.16609	0.868086	NA	1.084173	0	0	0	0	1.004979
27885	LOC11962₇	0.348192	0.671062	4.040349	0.16609	0.868086	NA	1.084173	0	0	0	0	1.004979
27959	LOC11962ε	0.348192	0.671062	4.040349	0.16609	0.868086	NA	1.084173	0	0	0	0	1.004979
3318	GNPTAB	3432.754	0.670838	0.111336	6.025347	1.69E-09	1.35E-08	2507.692	2981.752	2459.41	4176.31	3968.05	4503.313
4050	ASB8	887.4383	0.670769	0.125775	5.333089	9.66E-08	6.34E-07	739.9479	624.5969	692.2527	1015.618	1099.503	1152.711
19688	MMP21	18.30844	0.670748	0.625889	1.071674	0.283867	0.383309	13.01007	17.51718	11.82743	11.16063	34.22577	22.10955
16448	LOC10322₄	51.56708	0.670413	0.424303	1.580034	0.114099	0.184408	35.23561	44.34036	39.65669	33.4819	98.39909	58.28881
4046	CUNH8orf!	831.3105	0.670385	0.125585	5.338079	9.39E-08	6.17E-07	668.9346	608.7219	651.2046	964.2789	971.1562	1123.567
24084	DEFB113	2.341896	0.670263	1.706748	0.392714	0.694531	0.766678	2.710432	0.547412	2.087194	6.696381	0	2.009959
18396	MEGF11	33.49799	0.670055	0.52959	1.265234	0.205788	0.297394	33.60935	12.04306	31.30791	42.41041	53.47777	28.13943
20880	LOC10323₄	11.21922	0.669959	0.750365	0.892845	0.37194	0.473565	6.505036	8.211177	11.1317	11.16063	19.252	11.05477
21889	LOC10324ϵ	13.2664	0.669824	0.897628	0.746216	0.455537	0.553267	8.131296	11.49565	10.43597	11.16063	36.36488	2.009959
2800	ILRUN	6454.373	0.669728	0.100022	6.695828	2.14E-11	2.04E-10	5202.403	4856.09	4895.862	7600.392	7379.932	8791.56
8285	LOC11961ᶜ	207.8524	0.669673	0.201862	3.317473	0.000908	0.002915	169.673	171.3399	141.2335	220.9806	269.5279	274.3594
3456	ITFG1	4991.589	0.669637	0.113565	5.896518	3.71E-09	2.86E-08	3562.05	4477.829	3520.401	6051.296	6139.248	6198.713
23832	LOC11962₂	2.315861	0.669366	1.545873	0.433002	0.665013	0.741833	1.626259	1.094824	2.782925	2.232127	2.139111	4.019918
13969	TRNAH-GL	94.98097	0.669133	0.331576	2.018041	0.043587	0.082952	84.02339	43.79295	92.53227	124.9991	106.9555	117.5826
17828	LOC11962₄	27.34268	0.669108	0.487557	1.372368	0.169949	0.253427	27.10432	17.51718	18.78475	31.24978	34.22577	35.17428
18884	LOC11962(	21.07558	0.668984	0.554512	1.206437	0.227649	0.320486	18.97302	16.96977	12.52316	33.4819	21.39111	23.11453
7908	SLC26A11	196.6435	0.668901	0.194713	3.435324	0.000592	0.00199	162.0838	138.4952	155.1481	247.7661	233.1631	243.205
24039	HOXD1	2.272443	0.668754	1.667995	0.400933	0.68847	0.761386	1.626259	2.189647	1.391463	0	6.417332	2.009959
12433	TEKT2	84.36428	0.668729	0.288842	2.315206	0.020602	0.044052	74.80792	63.49977	56.35424	102.6778	113.3729	95.47305
2498	DHRS7	2357.767	0.668728	0.093718	7.135503	9.64E-13	1.03E-11	1908.144	1782.92	1772.028	2805.784	2956.251	2921.475
5263	BAK1	1851.986	0.668657	0.147095	4.54576	5.47E-06	2.76E-05	1595.902	1119.457	1578.614	2249.984	2102.746	2465.215
23801	LOC11962₃	2.568203	0.668388	1.531748	0.436356	0.662578	0.74008	1.626259	2.737059	1.391463	2.232127	6.417332	1.004979
2810	RIT1	1393.656	0.668019	0.09993	6.684898	2.31E-11	2.19E-10	1102.604	1066.358	1062.382	1703.113	1672.785	1754.694
23164	REG4	4.074133	0.667806	1.24782	0.535179	0.592526	0.680034	2.168345	2.189647	4.87012	8.928508	4.278221	2.009959
5120	NFAT5	3065.549	0.667749	0.144576	4.618682	3.86E-06	2.01E-05	2096.79	2972.994	2036.406	3620.51	3722.053	3944.544
12212	LOC11962₁	86.07774	0.667378	0.282208	2.364844	0.018038	0.039267	69.92914	61.31012	68.87741	89.28508	115.512	111.5527
11251	LRFN3	122.718	0.667305	0.259626	2.570252	0.010162	0.024014	98.65972	110.5772	75.83472	151.7846	132.6249	166.8266
24033	LOC11962!	2.342784	0.667232	1.66142	0.401604	0.687976	0.76103	2.168345	1.094824	2.087194	6.696381	0	2.009959
22733	LOC10322ϵ	4.9101	0.666966	1.104645	0.603783	0.545988	0.638503	4.336691	3.284471	3.478657	8.928508	6.417332	3.014938
22664	LOC10322ϵ	5.161658	0.666893	1.088814	0.612495	0.54021	0.633669	4.878777	2.737059	4.174388	4.464254	10.69555	4.019918
7411	TBX15	320.4393	0.66662	0.185385	3.595871	0.000323	0.00116	215.7504	297.792	228.1999	421.872	387.179	371.8424
11341	LOC10321₇	448.0391	0.666342	0.261527	2.547887	0.010838	0.025405	495.4669	207.4691	334.6468	537.9426	652.4288	460.2806
24490	LOC11962₇	1.428477	0.666113	2.004029	0.332387	0.739597	0.802833	1.626259	1.094824	0.695731	0	2.139111	3.014938
16819	DOC2B	44.70932	0.66606	0.437567	1.522191	0.127961	0.202262	24.93597	33.93953	43.83108	75.89232	53.47777	36.17926
4321	ATP6V0C	3606.878	0.665855	0.129502	5.141676	2.72E-07	1.68E-06	2448.604	3363.298	2554.726	4560.235	4248.274	4466.129
2979	SPEG	1804.71	0.665731	0.103186	6.451734	1.11E-10	9.87E-10	1402.92	1361.961	1426.945	2124.985	2115.58	2395.871
13071	LOC10322!	98.84484	0.665591	0.303561	2.192612	0.028335	0.057627	74.80792	73.90059	80.70484	80.35657	164.7115	118.5876
3296	LIPG	2020.447	0.665323	0.110057	6.045247	1.49E-09	1.20E-08	1602.949	1672.891	1414.422	2339.269	2462.116	2631.036
6022	LOC11962₄	371.5912	0.665104	0.160106	4.154153	3.26E-05	0.000144	302.4842	301.6239	258.1163	415.1756	489.8563	462.2906
27078	LOC10323!	0.34908	0.664971	4.039763	0.164606	0.869254	NA	0.542086	0.547412	0	0	0	1.004979
27289	LOC11962!	0.34908	0.664971	4.039763	0.164606	0.869254	NA	0.542086	0.547412	0	0	0	1.004979
27970	LOC11962ε	0.34908	0.664971	4.039763	0.164606	0.869254	NA	0.542086	0.547412	0	0	0	1.004979
28640	LOC11962(	0.34908	0.664971	4.039763	0.164606	0.869254	NA	0.542086	0.547412	0	0	0	1.004979
28711	LOC11962(	0.34908	0.664971	4.039763	0.164606	0.869254	NA	0.542086	0.547412	0	0	0	1.004979
29046	LOC11962₂	0.34908	0.664971	4.039763	0.164606	0.869254	NA	0.542086	0.547412	0	0	0	1.004979
21917	LOC11961ε	10.4285	0.664762	0.896303	0.741671	0.458287	0.555895	2.168345	12.59047	9.044508	15.62489	17.11289	6.029877
3498	LMBRD1	1118.963	0.664515	0.113525	5.853486	4.81E-09	3.66E-08	862.4594	927.3156	808.4399	1310.259	1388.283	1417.021
11318	LOC11962₂	122.8238	0.664124	0.260074	2.553591	0.010662	0.025044	107.8752	77.18507	99.48959	136.1597	177.5462	138.6872
21929	LOC11962!	7.50888	0.664066	0.897343	0.740035	0.459278	0.556793	4.336691	6.568942	6.261582	11.16063	10.69555	6.029877
8392	RTL5	218.1699	0.66398	0.202164	3.28436	0.001022	0.003238	140.4004	191.5941	175.3243	265.6231	256.6933	279.3843

6188	THNSL1	378.7471	0.663947	0.162719	4.080326	4.50E-05	0.000193	291.6425	331.7316	255.3334	464.2824	464.187	465.3055
21232	LOC10323	10.07719	0.663753	0.784265	0.846338	0.397364	0.497547	5.96295	7.663765	9.740239	8.928508	17.11289	11.05477
19723	LOC11962	15.30562	0.663733	0.623122	1.065173	0.286798	0.38658	10.84173	12.04306	12.52316	20.08914	19.252	17.08465
24755	LOC10321	1.43559	0.66371	2.297215	0.288919	0.772643	0.82975	0	0.547412	2.782925	0	4.278221	1.004979
21183	LOC11961	10.11083	0.663666	0.77824	0.852779	0.393782	0.494203	9.215468	5.474118	9.044508	11.16063	10.69555	15.07469
6183	MIEF2	773.4831	0.663665	0.162524	4.083486	4.44E-05	0.000191	662.4295	486.1017	650.5088	968.7431	812.8621	1060.253
24192	LOC11962	2.855902	0.66366	1.767524	0.375474	0.707308	0.777272	2.710432	1.642235	2.087194	0	10.69555	0
21873	LOC11962	8.108374	0.663558	0.886062	0.748885	0.453927	0.551689	7.047123	3.831883	7.653045	13.39276	10.69555	6.029877
19572	LOC10324	18.88763	0.663383	0.609579	1.088264	0.276479	0.375546	10.84173	15.87494	16.69755	35.71403	17.11289	17.08465
18079	ZNF883	27.48515	0.663203	0.499948	1.326544	0.18466	0.27154	20.0572	24.08612	18.78475	44.64254	34.22577	23.11453
16461	NEBL	43.36404	0.663181	0.420099	1.578632	0.114421	0.184796	26.02015	43.79295	29.91645	62.49956	57.75599	40.19918
3668	CHFR	1239.723	0.662842	0.116656	5.682005	1.33E-08	9.65E-08	997.4389	957.4233	929.4971	1415.169	1452.456	1686.356
5660	LOC10323	469.2488	0.662792	0.152651	4.341887	1.41E-05	6.64E-05	365.9083	377.1667	348.5614	616.067	487.7172	620.0723
18345	CACNA2D3	32.69703	0.66211	0.519463	1.274606	0.202449	0.293383	24.93597	18.06459	32.00364	40.17829	59.8951	21.10457
19320	LOC10322	34.96121	0.662099	0.585105	1.131592	0.257806	0.35475	27.6464	30.10765	22.2634	31.24978	83.42532	15.07469
23243	LOC11962	4.060406	0.662078	1.266299	0.522845	0.601082	0.687503	1.084173	3.284471	4.87012	6.696381	6.417332	2.009959
3861	KANK1	1343.182	0.662005	0.120683	5.4855	4.12E-08	2.84E-07	1010.449	1124.931	988.6343	1680.792	1467.43	1786.853
6445	VAMP2	418.0262	0.661809	0.167044	3.96188	7.44E-05	0.000307	280.2587	380.4512	310.9919	484.3716	530.4994	521.5843
19298	SOWAHA	20.73504	0.661776	0.582718	1.135673	0.256094	0.352796	19.51511	10.94824	17.39328	20.08914	36.36488	20.09959
14866	CUNH2orf	51.31465	0.661628	0.355752	1.859801	0.062914	0.112509	40.11439	37.77142	41.74388	51.33892	70.59065	66.32864
13291	LOC10323	86.27713	0.661142	0.307484	2.150164	0.031542	0.063092	72.63957	49.26706	79.31338	91.51721	113.3729	111.5527
2617	SGPP1	2247.65	0.660844	0.094986	6.957269	3.47E-12	3.52E-11	1808.4	1727.084	1692.019	2738.82	2650.358	2869.216
3059	KDM5B	2612.378	0.660822	0.104478	6.324989	2.53E-10	2.20E-09	2042.581	2165.561	1868.039	3071.407	3101.71	3424.97
3195	HFE	1125.877	0.660607	0.107143	6.16568	7.02E-10	5.84E-09	868.9645	886.8071	862.0112	1439.722	1304.858	1392.902
8508	TNFAIP8L1	199.0417	0.660576	0.202972	3.254512	0.001136	0.003549	146.3633	177.9088	137.7548	254.4625	239.5804	238.1801
15141	CUNH7orf	54.72511	0.660523	0.36546	1.807376	0.070704	0.124144	36.86187	35.03436	55.65851	71.42806	62.03421	67.33362
27014	SUSD3	0.562821	0.660167	3.488086	0.189263	0.849886	NA	0.542086	0	0.695731	0	2.139111	0
28229	LOC11961	0.562821	0.660167	3.488086	0.189263	0.849886	NA	0.542086	0	0.695731	0	2.139111	0
28636	LOC11962	0.562821	0.660167	3.488086	0.189263	0.849886	NA	0.542086	0	0.695731	0	2.139111	0
28778	CYLC1	0.562821	0.660167	3.488086	0.189263	0.849886	NA	0.542086	0	0.695731	0	2.139111	0
17545	LOC10323	34.33512	0.659905	0.468323	1.409079	0.158812	0.240643	18.43094	25.72836	36.17803	31.24978	49.19955	45.22408
27612	LOC11962	0.578324	0.659814	3.458037	0.190806	0.848678	NA	0.542086	0	0.695731	2.232127	0	0
28815	TRNAK-CU	0.578324	0.659814	3.458037	0.190806	0.848678	NA	0.542086	0	0.695731	2.232127	0	0
4060	COL27A1	982.9911	0.659528	0.123843	5.325527	1.01E-07	6.59E-07	690.618	841.9194	754.8685	1147.313	1232.128	1231.1
23016	LOC11962	4.052406	0.659242	1.178463	0.559408	0.575883	0.665183	3.252518	3.284471	2.782925	6.696381	4.278221	4.019918
8283	LOC10323	192.0975	0.659074	0.198557	3.319327	0.000902	0.002896	159.9155	133.5685	153.7566	225.4448	241.7195	238.1801
27269	LOC11962	0.349967	0.658899	4.039181	0.163127	0.870419	NA	0	1.094824	0	0	0	1.004979
28501	LOC11962	0.349967	0.658899	4.039181	0.163127	0.870419	NA	0	1.094824	0	0	0	1.004979
3241	LOC10324	1979.723	0.658511	0.107654	6.11691	9.54E-10	7.83E-09	1582.35	1398.09	1629.403	2441.947	2290.988	2535.563
20053	LOC11961	15.9137	0.658103	0.64533	1.019792	0.307827	0.408077	11.9259	12.59047	11.82743	24.5534	23.53022	11.05477
7975	LOC10322	239.2919	0.657944	0.192485	3.418162	0.00063	0.002102	170.7572	206.9217	178.803	261.1589	333.7013	284.4092
2517	SERINC1	7361.623	0.657936	0.092661	7.100493	1.24E-12	1.31E-11	5759.668	5958.578	5420.443	8924.044	8477.296	9629.713
15157	SPON2	55.06997	0.657857	0.3645	1.804819	0.071103	0.124713	50.41403	33.39212	43.83108	82.5887	59.8951	60.29877
23491	TEX52	3.182404	0.657817	1.353819	0.485897	0.62704	0.709628	2.168345	2.737059	2.782925	2.232127	2.139111	7.034856
4386	TBC1D20	779.7922	0.657552	0.129209	5.089075	3.60E-07	2.18E-06	604.4263	641.0192	573.2826	872.7616	939.0696	1048.194
21754	LOC11962	8.989636	0.657432	0.856206	0.767843	0.442581	0.540866	8.131296	3.284471	9.740239	11.16063	8.556443	13.06473
7829	LOC10323	216.6548	0.657044	0.189965	3.458773	0.000543	0.001843	179.9727	152.7279	172.5414	272.3195	245.9977	276.3694
4801	CNNM3	1023.297	0.65688	0.136504	4.812167	1.49E-06	8.27E-06	860.2911	708.8983	818.1801	1216.509	1120.894	1415.011
3705	ATAD2	9629.05	0.656655	0.116533	5.634919	1.75E-08	1.26E-07	6840.046	8811.141	6773.641	11582.51	11621.79	12145.18
18680	LOC11962	22.59115	0.656494	0.533139	1.231374	0.218183	0.310514	19.51511	18.06459	15.30609	31.24978	19.252	32.15934
19227	LOC10323	19.62526	0.65592	0.570458	1.149813	0.250221	0.345978	16.26259	13.6853	15.30609	22.32127	32.08666	18.08963
17669	LOC11962	33.90456	0.655866	0.471412	1.39128	0.164141	0.246968	22.22554	19.15941	38.26523	40.17829	36.36488	47.23404
6333	LOC10321	413.9613	0.655762	0.163464	4.011662	6.03E-05	0.000253	368.0766	295.6024	301.9474	453.1218	528.3603	536.659
10349	LOC11962	130.903	0.655692	0.236141	2.776693	0.005492	0.014107	107.8752	92.5126	105.0554	174.1059	139.0422	166.8266
7611	CFAP54	260.9583	0.655256	0.186013	3.522641	0.000427	0.001492	204.9086	215.1328	185.0645	350.4439	335.8404	274.3594
19145	LOC10321	22.15574	0.65524	0.563905	1.161968	0.245248	0.340555	15.7205	17.51718	18.78475	13.39276	36.36488	31.15436
22133	LOC10324	6.913278	0.655174	0.931483	0.703367	0.481827	0.578745	4.878777	5.474118	5.565851	6.696381	12.83466	6.029877
2860	TATDN1	1801.219	0.655142	0.099275	6.599258	4.13E-11	3.84E-10	1424.061	1386.047	1383.114	2261.145	2286.709	2066.238
26613	LOC11962	0.563709	0.654767	3.486361	0.187808	0.851027	NA	0	0.547412	0.695731	0	2.139111	0
27370	LOC10323	0.563709	0.654767	3.486361	0.187808	0.851027	NA	0	0.547412	0.695731	0	2.139111	0
27415	LOC11962	0.563709	0.654767	3.486361	0.187808	0.851027	NA	0	0.547412	0.695731	0	2.139111	0
27884	LOC11962	0.563709	0.654767	3.486361	0.187808	0.851027	NA	0	0.547412	0.695731	0	2.139111	0
28195	LOC10322	0.563709	0.654767	3.486361	0.187808	0.851027	NA	0	0.547412	0.695731	0	2.139111	0
29088	LOC10323	0.563709	0.654767	3.486361	0.187808	0.851027	NA	0	0.547412	0.695731	0	2.139111	0
3099	LAT	2862.855	0.654638	0.104219	6.281385	3.36E-10	2.88E-09	2101.669	2386.716	2188.771	3446.404	3272.839	3780.733
22222	LOC11962	10.32767	0.654561	0.950994	0.688291	0.49127	0.587724	10.84173	2.737059	10.43597	2.232127	25.66933	10.04979
22697	ACTL8	5.525555	0.65456	1.075633	0.608535	0.542833	0.63582	4.878777	1.642235	6.261582	8.928508	6.417332	5.024897
29081	DEFB126	0.579212	0.65443	3.456386	0.189339	0.849827	NA	0	0.547412	0.695731	2.232127	0	0
19063	LOC11962	22.76826	0.65385	0.556473	1.17499	0.239999	0.334699	16.80468	15.87494	20.17621	17.85702	42.78221	23.11453
8378	LOC10323	210.1893	0.653789	0.198707	3.290212	0.001001	0.003177	149.0738	187.7623	153.0609	243.3018	271.6671	256.2698
3784	LOC10323	797.6446	0.653235	0.117523	5.558345	2.72E-08	1.91E-07	596.295	642.6615	622.6796	986.6001	932.6523	1004.979
17349	LOC11962	36.83699	0.652696	0.453488	1.439279	0.150071	0.229954	34.69353	18.612	32.00364	51.33892	49.19955	35.17428
22816	LOC11962	4.93037	0.652337	1.106024	0.589803	0.555322	0.647049	4.336691	4.926706	2.087194	8.928508	4.278221	5.024897
9188	GPBAR1	167.8056	0.652287	0.213281	3.058351	0.002226	0.00644	122.5115	121.5254	146.7993	214.2842	211.772	189.9411

20169	LOC10321!	18.41056	0.651861	0.651282	1.00089	0.31688	0.417684	11.9259	14.23271	16.69755	37.94616	8.556443	21.10457
24189	LOC11962(	2.310003	0.651709	1.733368	0.375978	0.706933	0.776957	3.252518	0	2.087194	2.232127	4.278221	2.009959
2911	ATL3	8490.096	0.651202	0.09964	6.535551	6.34E-11	5.79E-10	6413.424	7275.103	6131.481	10332.52	9854.883	10933.17
9723	LOC10322	152.0033	0.651072	0.222661	2.924054	0.003455	0.009447	109.5014	120.978	123.1445	180.8023	211.772	165.8216
14714	LOC11962(	59.85004	0.650947	0.344873	1.887498	0.059093	0.106769	48.78777	42.15071	48.00546	64.73168	94.12087	61.30375
3230	ZFP90	2980.786	0.650871	0.10614	6.132201	8.67E-10	7.13E-09	2276.763	2581.594	2099.717	3631.671	3625.793	3669.18
19151	SERPINA1(	25.03228	0.650716	0.560777	1.160383	0.245893	0.341343	18.97302	18.612	20.87194	13.39276	49.19955	29.1444
18080	ANKDD1A	29.17134	0.65051	0.490557	1.326065	0.184818	0.271758	26.02015	24.63353	16.69755	33.4819	47.06043	27.13445
24614	LOC11962(	1.439898	0.650496	2.069215	0.314368	0.753241	0.813558	2.168345	1.094824	0	2.232127	2.139111	1.004979
17376	LOC11962(	42.60162	0.650331	0.453333	1.434556	0.151414	0.23166	47.16151	18.06459	34.09084	53.57105	53.47777	49.24399
3238	LOC10324:	1654.364	0.650244	0.106197	6.123013	9.18E-10	7.54E-09	1222.405	1326.379	1314.932	2151.77	1869.583	2041.113
15053	LOC11961	71.09963	0.650164	0.356512	1.823679	0.068201	0.120449	73.18166	37.224	55.65851	71.42806	102.6773	86.42823
23203	LOC10321	4.063042	0.650138	1.227774	0.529526	0.596441	0.683376	4.336691	1.094824	4.174388	4.464254	4.278221	6.029877
24883	LOC11962	1.162303	0.649726	2.414543	0.269089	0.787861	0.841751	0.542086	2.189647	0	2.232127	0	2.009959
14377	AMIGO3	57.94752	0.649292	0.333244	1.948398	0.051367	0.094977	46.61943	39.96106	48.7012	69.19594	74.86887	68.3386
4433	IP6K1	2659.946	0.649203	0.128374	5.057127	4.26E-07	2.55E-06	2388.433	1797.153	2032.231	3142.835	3018.285	3580.742
4215	OSBPL5	1118.225	0.64916	0.124646	5.208037	1.91E-07	1.20E-06	909.6209	785.536	921.1483	1243.295	1375.448	1474.305
2198	SAT2	3327.172	0.648966	0.085275	7.610292	2.73E-14	3.31E-13	2612.856	2538.349	2621.516	4066.935	4113.51	4009.868
21315	LOC11962!	13.14448	0.648464	0.78058	0.830747	0.406117	0.506526	9.215468	15.32753	5.565851	29.01765	10.69555	9.044815
16511	CCDC116	46.25826	0.648451	0.412135	1.573395	0.115627	0.186176	36.31979	29.56024	41.74388	42.41041	81.28621	46.22906
19395	LOC10321!	26.92333	0.647892	0.579353	1.118302	0.263438	0.361082	10.29964	33.39212	18.78475	29.01765	44.92132	25.12449
3603	ELOVL5	11576.31	0.647515	0.11291	5.734768	9.76E-09	7.20E-08	8122.08	10536.58	8405.131	13843.65	14197.28	14353.12
22922	LOC11962:	4.64261	0.647359	1.12406	0.575912	0.564675	0.654912	2.168345	4.379295	4.174388	6.696381	6.417332	4.019918
22392	LOC10323!	6.082508	0.64721	0.981771	0.659227	0.50975	0.605203	4.336691	5.474118	4.174388	8.928508	8.556443	5.024897
7379	MSRB1	1747.761	0.647034	0.17933	3.608065	0.000308	0.001112	1631.138	945.9276	1512.52	2040.164	1967.982	2388.836
20144	COLGALT2	16.16226	0.646797	0.643966	1.004396	0.315188	0.415968	9.757555	14.23271	13.91463	11.16063	27.80844	20.09959
9764	ACADS	766.7275	0.646638	0.221972	2.913152	0.003578	0.009742	742.1162	371.1452	681.8167	957.5825	791.4709	1056.233
22635	LOC11962:	6.6685	0.646267	1.045209	0.618314	0.536369	0.629969	8.673382	4.379295	2.087194	11.16063	10.69555	3.014938
21532	LOC11962!	8.964182	0.646255	0.807965	0.799855	0.423795	0.523241	7.589209	7.116354	6.261582	8.928508	12.83466	11.05477
3289	LRRK2	2018.447	0.645712	0.106702	6.051557	1.43E-09	1.16E-08	1596.444	1646.067	1482.604	2243.288	2543.403	2598.877
7532	CSDC2	598.6889	0.645479	0.1819	3.548544	0.000387	0.001367	529.6184	373.3349	500.9266	756.691	590.3945	841.1678
4324	CHCHD10	1876.072	0.6454	0.125669	5.135697	2.81E-07	1.73E-06	1542.236	1322.547	1531.305	2147.306	2092.05	2620.986
4217	ZNF34	704.3765	0.64533	0.123935	5.20702	1.92E-07	1.21E-06	546.9651	543.0325	561.4552	841.5119	810.7229	922.5712
8524	LOC10323!	214.5461	0.645292	0.198644	3.248477	0.00116	0.003619	146.9054	166.9606	187.1517	267.8552	280.2235	238.1801
3334	BCAR1	3318.229	0.645075	0.10736	6.008516	1.87E-09	1.49E-08	2723.442	2370.293	2675.087	4194.167	3662.157	4284.227
4146	TTI2	784.334	0.64503	0.12262	5.2604	1.44E-07	9.22E-07	622.8572	626.2391	589.2845	948.654	889.87	1029.099
4340	RAB27B	1883.136	0.644314	0.125688	5.126294	2.96E-07	1.81E-06	1310.765	1724.347	1372.678	2263.377	2342.326	2285.323
22988	TRNAE-CU(	5.276763	0.643686	1.137453	0.565902	0.571461	0.660879	2.710432	2.189647	7.653045	8.928508	2.139111	8.039836
12107	IRX5	105.6741	0.643438	0.269797	2.384898	0.017084	0.037513	76.97626	99.62895	70.26887	140.624	121.9293	124.6175
2827	UBE2E1	2846.088	0.643367	0.096677	6.654821	2.84E-11	2.67E-10	2217.675	2337.996	2108.762	3308.012	3660.018	3444.065
5395	LOC10323!	1383.075	0.643004	0.144018	4.464761	8.02E-06	3.95E-05	1209.395	899.3976	1134.738	1665.167	1497.377	1892.376
2584	ATP6V0E1	5715.118	0.642866	0.091758	7.006068	2.45E-12	2.52E-11	4610.445	4102.304	4676.011	6993.254	6870.823	7037.871
16538	LGI4	40.71991	0.642716	0.409749	1.568558	0.116751	0.187678	28.73058	29.01283	36.87376	58.0353	53.47777	38.18922
23409	LOC10321	4.05554	0.642703	1.291864	0.497501	0.618836	0.702796	2.168345	1.094824	6.261582	2.232127	8.556443	4.019918
16789	PCYT1B	57.49288	0.6427	0.420737	1.527559	0.126622	0.200507	33.60935	44.34036	55.65851	58.0353	109.0946	44.2191
17693	FER1L6	45.96664	0.642578	0.463594	1.386079	0.165723	0.24901	45.53526	30.65506	29.91645	60.26743	81.28621	28.13943
3513	EDEM3	5107.663	0.642521	0.109925	5.84509	5.06E-09	3.83E-08	3731.723	4516.695	3720.076	6241.027	5839.772	6596.685
19160	GPHA2	24.12819	0.642242	0.554231	1.158798	0.246539	0.342079	21.14137	13.6853	20.87194	33.4819	38.50399	17.08465
3381	MTMR10	2759.228	0.642237	0.107788	5.95832	2.55E-09	2.00E-08	2054.507	2399.853	2012.751	3209.799	3351.986	3526.473
21780	LOC11962	8.999967	0.642138	0.841806	0.76281	0.445577	0.543877	10.29964	3.831883	6.957314	11.16063	10.69555	11.05477
20316	LOC11962:	16.57894	0.642007	0.658196	0.975404	0.32936	0.430992	7.589209	17.51718	13.2189	24.5534	23.53022	13.06473
4385	SCARB1	1334.665	0.64197	0.126108	5.090655	3.57E-07	2.16E-06	1135.671	948.1173	1048.467	1562.489	1495.238	1818.008
9429	SWSAP1	209.4574	0.641886	0.213877	3.001192	0.002689	0.007582	153.9525	162.5813	175.3243	296.8729	194.6591	273.3544
22074	LOC11962(	9.236263	0.641498	0.899027	0.713547	0.475507	0.572681	5.96295	12.59047	2.782925	8.928508	17.11289	8.039836
2767	TRIM8	4800.455	0.641461	0.095094	6.745578	1.52E-11	1.46E-10	3624.932	3928.775	3702.682	5792.37	5471.845	6282.127
3875	ENPP5	1657.416	0.641336	0.117188	5.472715	4.43E-08	3.04E-07	1207.226	1438.051	1236.315	2002.218	2173.336	1887.351
24729	LOC11962:	1.759264	0.640982	2.177236	0.294402	0.768451	0.826126	0	0	4.174388	2.232127	2.139111	2.009959
17405	LOC10324(	34.0083	0.640955	0.448617	1.428735	0.15308	0.23382	27.6464	29.56024	21.56767	51.33892	42.78221	31.15436
9994	LOC10324(	163.4456	0.640756	0.223806	2.862992	0.004197	0.011163	108.9594	140.6848	132.189	218.7484	203.2155	176.8764
21667	GPR1	8.960987	0.640729	0.820443	0.780956	0.434829	0.533526	7.589209	7.663765	5.565851	8.928508	14.97377	9.044815
12738	LOC10321	179.615	0.640472	0.284557	2.250769	0.0244	0.050927	174.5518	104.0082	141.9292	158.481	303.7537	194.966
5332	PPIC	996.3676	0.640317	0.142231	4.501946	6.73E-06	3.36E-05	892.2742	639.9244	804.2655	1218.741	1215.015	1207.985
2570	SDC4	5252.933	0.640184	0.091162	7.022485	2.18E-12	2.25E-11	3968.614	4155.403	4200.13	6312.455	6045.127	6835.87
22705	LOC10322!	6.391923	0.640141	1.052712	0.608088	0.543129	0.635943	1.626259	8.211177	4.87012	8.928508	10.69555	4.019918
23669	LOC11961	2.909485	0.639687	1.393003	0.459214	0.64608	0.725677	3.252518	2.189647	1.391463	4.464254	2.139111	4.019918
20158	LOC11961!	15.11759	0.639683	0.638451	1.00193	0.316377	0.417234	10.29964	10.94824	14.61036	15.62489	17.11289	22.10955
3219	RNF103	1630.503	0.639627	0.104168	6.140343	8.23E-10	6.80E-09	1245.714	1370.719	1209.181	1937.486	1980.816	2039.103
3424	LRRC8D	3978.474	0.639509	0.108005	5.921081	3.20E-09	2.48E-08	2913.714	3531.901	2887.285	4723.181	4879.311	4935.454
2766	PHKB	3746.67	0.639478	0.094779	6.74703	1.51E-11	1.45E-10	2871.431	3118.058	2802.406	4522.289	4368.064	4797.772
12307	LOC11962:	125.0401	0.638819	0.272655	2.342961	0.019131	0.041327	116.5486	73.35318	104.3597	162.9453	126.2075	166.8266
5752	LOC10322	959.4871	0.63874	0.148571	4.299224	1.71E-05	7.92E-05	815.84	616.9331	821.6587	1183.027	1048.164	1271.299
2710	MAPRE3	9864.804	0.638604	0.093573	6.824626	8.82E-12	8.65E-11	7352.317	8292.194	7509.029	11834.74	11429.27	12771.28
23214	LOC11961!	4.326897	0.638427	1.210243	0.527519	0.597833	0.68466	2.168345	6.02153	2.087194	2.232127	6.417332	7.034856
5745	RETSAT	915.0671	0.638419	0.148404	4.301909	1.69E-05	7.84E-05	790.904	601.0582	758.3472	1151.778	973.2954	1215.02

7591	LOC11962(	259.5605	0.638301	0.180876	3.528936	0.000417	0.001461	210.3295	211.301	188.5432	274.5516	337.9795	334.6582
3989	GRAMD1B	809.2581	0.637299	0.118419	5.381718	7.38E-08	4.92E-07	659.7191	621.8598	619.8967	924.1006	1013.938	1016.034
6273	HDHD3	474.1947	0.636915	0.157604	4.041223	5.32E-05	0.000225	351.272	356.9125	408.3943	600.4422	491.9955	636.152
5017	GRAMD4	1952.699	0.63688	0.136127	4.678572	2.89E-06	1.53E-05	1665.831	1343.896	1580.702	2408.465	2012.903	2704.4
3522	IPO13	2132.188	0.63684	0.109094	5.837553	5.30E-09	4.00E-08	1703.235	1640.593	1669.06	2566.946	2338.048	2875.246
22443	LOC11962(	6.662404	0.636751	0.979494	0.650082	0.515639	0.610804	4.878777	7.663765	2.782925	8.928508	10.69555	5.024897
17296	LOC10322	37.54763	0.636744	0.439429	1.449025	0.147331	0.226456	29.81475	27.918	29.22072	53.57105	55.61688	29.1444
16537	NDUFS7	714.3977	0.636656	0.405865	1.568638	0.116732	0.18766	690.618	286.2964	701.2972	917.4042	772.219	918.5512
7370	LOC10321	269.8931	0.636596	0.176369	3.609459	0.000307	0.001107	196.2353	235.3871	203.1536	314.7299	325.1448	344.708
10204	LOC11962	201.3325	0.636283	0.225979	2.815671	0.004868	0.012682	147.9896	191.0467	132.189	229.9091	288.7799	218.0805
14452	LOC11962	123.5661	0.636146	0.328921	1.934037	0.053109	0.097695	119.8011	70.06871	98.09812	165.1774	196.7982	91.45313
9759	SERTAD1	888.2102	0.636029	0.218265	2.914019	0.003568	0.00972	860.2911	414.9382	811.9185	1102.671	1046.025	1093.418
22959	LOC11962	6.153013	0.635948	1.116145	0.569772	0.568832	0.658645	6.505036	3.284471	4.174388	13.39276	8.556443	1.004979
12173	LOC11962	109.4703	0.635872	0.26815	2.371326	0.017724	0.038711	101.9122	80.46954	73.74753	154.0168	124.0684	122.6075
14172	LOC10324	65.85642	0.635642	0.320699	1.98205	0.047474	0.089068	49.32986	50.9093	53.57132	95.98146	77.00798	68.3386
19217	LOC10323	25.44066	0.635616	0.552159	1.151148	0.249672	0.34538	23.8518	11.49565	24.3506	42.41041	21.39111	29.1444
2380	ARMC8	3066.118	0.635579	0.087035	7.302561	2.82E-13	3.15E-12	2452.941	2398.211	2354.355	3741.045	3649.323	3800.832
3711	FUT11	1657.289	0.635386	0.112795	5.633124	1.77E-08	1.27E-07	1288.539	1354.844	1255.099	2037.932	1805.409	2201.91
18783	GRAMD2A	36.76758	0.635365	0.520625	1.220389	0.222317	0.314663	33.60935	14.78012	38.26523	24.5534	64.17332	45.22408
3200	DENND4C	6547.947	0.635348	0.103126	6.160916	7.23E-10	6.01E-09	4865.225	5728.665	4795.676	7761.106	7724.329	8412.683
11246	TMEM185/	171.8587	0.635035	0.246979	2.571206	0.010134	0.023957	173.4676	105.1031	125.9274	187.4987	216.0502	223.1054
3260	KPNA4	8331.508	0.634978	0.104317	6.086976	1.15E-09	9.38E-09	6129.371	7409.219	6043.123	10037.88	9996.064	10373.4
4149	ZNF438	971.2143	0.634728	0.120715	5.258063	1.46E-07	9.33E-07	718.8065	822.2125	738.8667	1185.259	1255.658	1106.482
18436	LOC11962	28.26325	0.634057	0.503642	1.258943	0.208051	0.300013	26.02015	14.23271	25.74206	37.94616	38.50399	27.13445
22691	LOC10324	6.14797	0.633687	1.039541	0.609584	0.542138	0.63522	3.252518	7.116354	4.174388	11.16063	2.139111	9.044815
23727	TRNAV-CA	2.893095	0.633264	1.40899	0.449446	0.65311	0.731741	3.794605	1.642235	1.391463	2.232127	4.278221	4.019918
23857	LOC11962	2.620838	0.632705	1.474719	0.429034	0.667898	0.74423	1.626259	1.094824	3.478657	2.232127	4.278221	3.014938
21256	LOC11962	12.77735	0.63256	0.751755	0.841443	0.4001	0.500407	14.09425	4.379295	11.82743	11.16063	17.11289	18.08963
4653	FBXO33	654.5991	0.632355	0.128642	4.915636	8.85E-07	5.06E-06	526.908	524.9679	489.0992	729.9055	840.6705	816.0433
19631	LOC10322(	20.37698	0.63233	0.585482	1.080016	0.280135	0.379369	14.63633	12.59047	20.17621	24.5534	34.22577	16.07967
2326	LOC10324	5601.729	0.632249	0.085739	7.3741	1.65E-13	1.89E-12	4359.459	4575.268	4248.136	6792.362	6601.296	7033.851
9532	NCAM2	360.7126	0.632192	0.212401	2.976412	0.002916	0.008134	250.986	376.0719	220.5468	475.443	417.1266	424.1013
15396	LOC10322	58.44195	0.632111	0.359087	1.760329	0.078352	0.135294	41.19856	51.45671	43.83108	95.98146	59.8951	58.28881
21832	LOC11962	8.709888	0.631958	0.837104	0.754934	0.450289	0.54832	4.878777	9.306001	6.261582	8.928508	12.83466	10.04979
2572	STK35	5004.225	0.631818	0.089972	7.022414	2.18E-12	2.25E-11	3906.816	4051.942	3822.348	6035.671	5760.625	6447.948
12763	LOC10322	97.54648	0.631715	0.281037	2.247803	0.024589	0.051214	80.77087	73.35318	74.44326	151.7846	98.39909	106.5278
18219	LOC10323	26.81879	0.631217	0.486093	1.298552	0.194098	0.283225	21.14137	21.89647	19.48048	40.17829	32.08666	26.12947
2406	ITGAV	13550.45	0.6312	0.086889	7.26441	3.75E-13	4.14E-12	10558.22	11205.52	10137.5	16171.76	15929.96	17299.72
3536	RRAGC	1206.081	0.631032	0.108432	5.819635	5.90E-09	4.43E-08	942.1461	967.2767	933.6715	1412.936	1409.674	1570.783
19438	LOC10321	20.44782	0.630654	0.567232	1.11181	0.26622	0.364104	11.38381	19.15941	17.39328	31.24978	21.39111	22.10955
2463	CRIP2	4864.927	0.630358	0.087797	7.17971	6.99E-13	7.54E-12	3751.238	3800.133	3907.227	6087.01	5563.827	6080.126
8520	LOC11961	190.7775	0.630271	0.19383	3.251676	0.001147	0.00358	156.663	141.2322	150.9737	234.3733	235.3022	226.1204
3065	TNFRSF1A	5495.314	0.630256	0.099806	6.314809	2.70E-10	2.35E-09	4175.149	4633.294	4137.514	6533.436	6252.62	7239.872
17877	LOC10323	32.52459	0.630239	0.461867	1.364546	0.172396	0.256371	28.18849	30.65506	18.08902	33.4819	38.50399	46.22906
12721	LOC11962	89.96982	0.630178	0.279678	2.253228	0.024245	0.050668	73.72375	78.27989	59.13717	116.0706	109.0946	103.5129
27792	LOC11962	0.606924	0.630078	3.346189	0.188297	0.850644	NA	1.084173	0.547412	0	0	0	2.009959
18918	LOC10322	24.2703	0.629757	0.524653	1.200332	0.23001	0.323228	14.09425	21.34906	21.56767	37.94616	23.53022	27.13445
23770	LOC10321	4.094875	0.629111	1.423668	0.441895	0.658565	0.736557	2.710432	0	6.957314	4.464254	6.417332	4.019918
16107	LOC10324	46.7312	0.628946	0.382516	1.644233	0.100128	0.165264	36.31979	31.20247	43.13534	62.49956	44.92132	62.30873
18362	LOC10324	32.55704	0.628833	0.49443	1.271834	0.203432	0.294535	28.18849	19.70683	27.82925	60.26743	34.22577	25.12449
17842	LOC10324	32.26916	0.628787	0.459067	1.369707	0.170778	0.254464	30.35684	24.08612	20.87194	37.94616	49.19955	31.15436
21387	LOC10322	9.891586	0.628582	0.76839	0.81805	0.413329	0.513786	8.673382	7.663765	6.957314	11.16063	12.83466	12.05975
24607	TRNAA-UG	1.470475	0.628338	1.993989	0.315116	0.752674	0.813176	0.542086	1.642235	1.391463	2.232127	0	3.014938
9115	ZC3H12B	177.044	0.628188	0.20401	3.079203	0.002076	0.006054	130.6428	150.5382	135.6676	205.3557	231.024	209.0357
6073	KIAA1217	1146.483	0.627953	0.151865	4.134931	3.55E-05	0.000155	797.409	1098.656	808.4399	1252.223	1409.674	1512.494
3479	FHL2	2344.052	0.627916	0.106816	5.878478	4.14E-09	3.16E-08	1970.484	1710.662	1848.558	2810.248	2663.193	3061.167
6432	LOC10324	346.105	0.627907	0.158264	3.967457	7.26E-05	0.0003	275.3799	291.7705	249.0718	424.1041	402.1528	434.1511
7949	ZC4H2	270.5166	0.627625	0.183268	3.424628	0.000616	0.002059	188.104	244.1457	205.9365	310.2657	337.9795	336.6681
17167	PRLR	48.40136	0.627559	0.426204	1.472438	0.140903	0.218203	33.06727	42.69812	36.87376	58.0353	85.56443	34.1693
2808	CNPPD1	2603.971	0.6275	0.093858	6.685596	2.30E-11	2.18E-10	2015.477	2058.816	2069.801	3058.014	3061.067	3360.651
18603	LOC10323	46.85733	0.62745	0.507946	1.235268	0.216731	0.309724	49.32986	13.13788	48.7012	53.57105	47.06043	69.34358
10960	TPTE2	140.5829	0.627283	0.237884	2.636927	0.008366	0.020293	103.5385	96.89189	132.189	167.4095	162.5724	180.8963
2261	DERL1	7221.593	0.627263	0.083693	7.494841	6.64E-14	7.80E-13	5824.718	5571.01	5636.12	8633.867	8494.408	9169.433
10154	RIPOR3	348.6134	0.627144	0.222045	2.824408	0.004737	0.012402	312.7838	214.038	298.4688	426.3363	314.4493	525.6043
20476	LOC10324	14.3458	0.626941	0.659686	0.950363	0.341928	0.44392	11.38381	10.40082	11.82743	24.5534	12.83466	15.07469
3931	HADH	3314.83	0.626747	0.115672	5.418322	6.02E-08	4.07E-07	2813.97	2346.754	2662.564	3906.222	3679.27	4480.198
21556	CCN3	10.5703	0.626316	0.786879	0.795949	0.426061	0.525461	6.505036	9.306001	9.044508	20.08914	6.417332	12.05975
4015	UBE2L6	1538.077	0.626246	0.11686	5.358958	8.37E-08	5.54E-07	1119.95	1358.129	1150.74	1910.701	1775.462	1913.481
22164	LOC11962	8.22794	0.626168	0.897543	0.697647	0.485398	0.58222	5.96295	7.663765	5.565851	17.85702	4.278221	8.039836
13086	GALNT9	91.35763	0.626096	0.285883	2.190041	0.028521	0.057938	82.39713	63.49977	70.26887	120.5349	89.84265	121.6025
26659	LOC11962	0.607811	0.626045	3.344705	0.187175	0.851523	NA	0.542086	1.094824	0	0	0	2.009959
19322	LOC10324	26.52565	0.625888	0.553199	1.131397	0.257888	0.354808	13.55216	16.42235	32.69937	24.5534	42.78221	29.1444
11683	LOC11962	112.2133	0.625583	0.252715	2.475449	0.013307	0.030283	101.9122	81.01695	82.0963	133.9276	132.6249	141.7021
16050	LOC11962	89.58465	0.624864	0.377101	1.657021	0.097515	0.161523	95.94929	34.48694	81.40057	113.8385	96.25998	115.5726

16155	LOC10321!	48.89874	0.624703	0.381858	1.635954	0.101849	0.167605	35.23561	48.71965	32.00364	51.33892	57.75599	68.3386
20466	SPDYC	16.53434	0.624572	0.65643	0.951467	0.341367	0.443431	13.01007	13.6853	11.82743	13.39276	34.22577	13.06473
4956	KAT6A	3908.579	0.62456	0.132583	4.710702	2.47E-06	1.32E-05	2758.135	3727.327	2742.573	4732.109	4449.35	5041.982
21104	LOC11962:	14.53553	0.624484	0.722311	0.864565	0.387278	0.487859	15.7205	4.926706	13.91463	11.16063	21.39111	20.09959
17816	LOC11962:	44.00532	0.623829	0.45394	1.374255	0.169363	0.252723	45.53526	30.10765	29.22072	26.78552	62.03421	70.34856
5304	HPS6	921.4392	0.623417	0.138076	4.515028	6.33E-06	3.17E-05	816.9242	638.8296	723.5606	1020.082	1110.198	1219.04
24190	EVA1C	2.346664	0.623127	1.658021	0.375825	0.707047	0.777018	2.710432	2.189647	0.695731	4.464254	0	4.019918
5777	TECPR1	1183.825	0.623091	0.14539	4.285646	1.82E-05	8.39E-05	1031.59	790.4627	978.1983	1439.722	1244.962	1618.017
15492	CCDC189	54.66939	0.622806	0.356664	1.746197	0.080777	0.138617	47.7036	42.15071	38.26523	82.5887	62.03421	55.27387
4151	TCAF1	6807.72	0.622518	0.118427	5.256575	1.47E-07	9.40E-07	4970.932	6241.042	4874.99	7973.158	7856.953	8929.243
3355	AACS	3664.395	0.621975	0.103897	5.986478	2.14E-09	1.70E-08	3059.535	2767.167	2838.584	4305.773	4122.066	4893.245
16640	LOC10321!	45.84524	0.621758	0.400942	1.550744	0.120963	0.193257	38.48813	27.37059	41.74388	55.80317	68.45154	43.21412
3886	AP5B1	1308.797	0.621657	0.113902	5.4578	4.82E-08	3.30E-07	1104.23	986.4361	1006.028	1573.65	1475.986	1706.455
4497	PPP1R37	1901.68	0.62142	0.123995	5.011661	5.40E-07	3.19E-06	1602.407	1361.961	1536.175	2252.216	2051.407	2605.912
21918	LOC10324(	9.327486	0.621325	0.837892	0.741534	0.45837	0.555971	4.878777	6.568942	10.43597	8.928508	17.11289	8.039836
3566	ZNF623	2573.628	0.620991	0.107285	5.788218	7.11E-09	5.30E-08	1959.1	2231.798	1896.564	2999.979	3026.842	3327.487
3135	LAMP1	5898.462	0.620968	0.099525	6.239323	4.39E-10	3.73E-09	4468.418	5019.219	4461.725	7035.664	6697.555	7708.193
14017	LOC10323(	81.78688	0.620957	0.30891	2.010158	0.044414	0.084238	62.88202	62.95236	66.09448	131.6955	87.70354	79.39338
11679	LOC11962;	248.9138	0.620759	0.250722	2.475892	0.01329	0.030253	234.7234	176.2666	173.9328	323.6584	382.9008	202.0009
5703	ZFP3	1250.246	0.620744	0.143503	4.325654	1.52E-05	7.09E-05	831.5605	1189.526	935.063	1482.132	1531.603	1531.589
20399	LOC10322{	15.22587	0.620509	0.645274	0.961621	0.33624	0.438205	14.09425	12.04306	9.740239	24.5534	12.83466	18.08963
21520	LOC11962!	13.58568	0.620336	0.774124	0.801339	0.422935	0.522478	10.84173	5.474118	15.30609	29.01765	12.83466	8.039836
3681	PRR13	3321.554	0.619807	0.109359	5.667612	1.45E-08	1.05E-07	2450.23	2922.084	2486.544	4078.096	3741.305	4251.063
16742	SERPINC1	51.61814	0.619514	0.403663	1.53473	0.12485	0.198252	56.37698	35.58177	29.22072	60.26743	77.00798	51.25395
8426	OTUD1	377.7916	0.618786	0.188951	3.274846	0.001057	0.003335	353.4403	260.568	283.1627	415.1756	410.7092	543.6939
16413	DNAH6	47.58393	0.618744	0.389953	1.586711	0.112578	0.182349	37.94605	39.96106	33.39511	60.26743	72.72976	41.20416
4899	MGAT4A	727.3286	0.618397	0.130381	4.743019	2.11E-06	1.14E-05	618.5205	513.4723	589.2845	910.7078	855.6443	876.3421
20658	LOC11962:	14.67031	0.618386	0.666663	0.927584	0.353623	0.455081	9.757555	8.758589	16.00182	22.32127	17.11289	14.06971
4585	PPP2R5B	1034.313	0.618112	0.124846	4.951	7.38E-07	4.28E-06	852.7019	747.7645	852.2709	1205.349	1167.954	1379.837
12198	NRXN2	146.3867	0.617958	0.261101	2.366738	0.017946	0.039112	101.3702	136.853	105.7512	194.195	207.4937	132.6573
3927	DEDD2	1189.741	0.617711	0.113888	5.423865	5.83E-08	3.95E-07	945.9407	993.005	879.4045	1466.507	1339.083	1514.504
8888	ZNF517	348.4663	0.617383	0.196174	3.147128	0.001649	0.004931	314.9522	211.8484	300.556	401.7829	389.3181	472.3404
19532	LOC11962(	41.70088	0.617294	0.563594	1.095281	0.273394	0.372115	41.19856	8.758589	48.7012	60.26743	47.06043	44.2191
17199	LOC10322;	41.20538	0.617241	0.420513	1.467828	0.142151	0.219732	29.27266	41.6033	25.74206	55.80317	55.61688	39.1942
3871	DRAM1	1747.476	0.617175	0.112702	5.476171	4.35E-08	2.98E-07	1365.516	1472.538	1303.105	1904.004	2175.476	2264.219
15775	KLLN	49.10332	0.617165	0.361812	1.705764	0.088052	0.148391	39.03022	38.86624	37.56949	64.73168	64.17332	50.24897
22060	ZNF843	8.824343	0.617094	0.863022	0.715038	0.474585	0.571934	8.673382	3.831883	8.348776	15.62489	6.417332	10.04979
16694	JAK3	48.84196	0.616966	0.399743	1.543408	0.122732	0.195449	38.48813	26.82318	50.09266	55.80317	70.59065	51.25395
10254	LOC10324(	342.1763	0.61684	0.220259	2.800521	0.005102	0.013228	237.4338	365.6711	206.6322	390.6222	440.6568	412.0416
4295	SLC2A13	952.9214	0.616789	0.11957	5.158392	2.49E-07	1.54E-06	716.0961	814.0014	729.1265	1095.974	1142.285	1220.045
10630	EFNA2	142.7288	0.616646	0.227419	2.7115	0.006698	0.016751	99.20181	122.6202	116.8829	176.338	160.4333	180.8963
16649	LOC11962!	48.91675	0.616425	0.398051	1.548609	0.121476	0.193971	47.7036	30.10765	36.87376	71.42806	64.17332	43.21412
14471	PRXL2B	106.4236	0.616187	0.319252	1.930096	0.053595	0.09846	101.9122	53.09895	98.09812	102.6778	139.0422	143.7121
19784	CARD14	44.50807	0.616186	0.583554	1.055919	0.291005	0.391042	33.60935	15.87494	54.96278	55.80317	87.70354	19.09461
4393	LOC10324;	959.264	0.615977	0.121178	5.083252	3.71E-07	2.25E-06	717.7224	793.1997	765.3045	1055.796	1174.372	1249.189
6207	MTERF2	402.2884	0.615849	0.151319	4.069885	4.70E-05	0.000201	305.7367	350.3436	296.3816	493.3001	485.5781	482.3901
3347	WDR7	3004.661	0.615658	0.102661	5.997025	2.01E-09	1.60E-08	2323.382	2601.848	2195.728	3542.386	3580.871	3783.748
14971	LOC11962:	81.96949	0.615414	0.334526	1.83966	0.065818	0.116878	69.92914	49.81448	74.44326	131.6955	68.45154	97.48301
6713	WDR53	374.464	0.615315	0.159801	3.85051	0.000118	0.000467	272.6694	335.016	279.684	448.6575	453.4915	457.2657
6197	XYLB	497.1165	0.615187	0.150946	4.075535	4.59E-05	0.000197	386.5076	442.8562	347.17	636.1562	586.1163	583.8931
15431	CCDC154	54.22056	0.615171	0.350461	1.755321	0.079204	0.136459	43.909	38.31883	46.614	73.66019	53.47777	69.34358
27923	LOC11962{	0.608699	0.615043	3.784085	0.162534	0.870885	NA	0	1.642235	0	0	0	2.009959
3019	TNFRSF10E	4912.297	0.614529	0.096195	6.388387	1.68E-10	1.48E-09	4038.001	3819.292	3793.127	5828.084	5540.297	6454.983
11359	LOC10323:	147.9337	0.6143	0.241252	2.546306	0.010887	0.02548	124.6799	126.4521	98.79385	151.7846	216.0502	169.8415
2931	EAF1	2300.839	0.61399	0.094323	6.509429	7.54E-11	6.84E-10	1815.989	1838.209	1805.423	2683.017	2718.81	2943.585
5191	ZNF710	581.3334	0.613827	0.13392	4.583537	4.57E-06	2.34E-05	454.2684	455.4466	470.3144	743.2983	631.0376	733.635
24486	LOC11961{	1.734342	0.613792	1.842801	0.333076	0.739077	0.802433	1.084173	1.642235	1.391463	0	4.278221	2.009959
16862	LOC11962:	39.7566	0.613233	0.405314	1.512982	0.130284	0.205417	32.52518	26.27577	35.4823	42.41041	55.61688	46.22906
20616	CAMKV	18.55814	0.61319	0.656517	0.934005	0.350301	0.451725	11.9259	13.13788	18.08902	33.4819	25.66933	9.044815
8197	APOBEC3C	795.306	0.613028	0.183117	3.34774	0.000815	0.002642	522.0292	836.9927	526.6686	964.2789	971.1562	950.7106
5536	SLC27A4	1160.744	0.612594	0.13932	4.397038	1.10E-05	5.27E-05	1025.085	758.7128	972.6325	1370.526	1339.083	1498.424
4562	TONSL	2169.036	0.612547	0.123373	4.965016	6.87E-07	4.00E-06	1831.168	1484.581	1834.644	2600.428	2402.221	2861.177
24533	TRNAQ-CU	1.735229	0.612155	1.881628	0.325333	0.744929	0.807237	0.542086	2.189647	1.391463	0	4.278221	2.009959
7845	AP5Z1	1662.159	0.61202	0.177181	3.454212	0.000552	0.00187	1545.488	939.3587	1463.123	1888.379	1816.105	2320.498
18736	LOC11961{	27.17061	0.612008	0.499047	1.226355	0.220065	0.312256	25.47806	17.51718	20.87194	31.24978	42.78221	25.12449
5514	SYNGAP1	514.1145	0.611957	0.138918	4.405153	1.06E-05	5.10E-05	384.8813	416.5804	417.4388	593.7458	678.0981	593.9429
11219	LOC11962:	274.9226	0.611875	0.237433	2.577038	0.009965	0.023614	255.8648	171.3399	221.9383	372.7652	391.4573	236.1702
5966	RUFY2	877	0.61184	0.146362	4.180311	2.91E-05	0.00013	633.699	834.803	612.2436	1060.26	1056.721	1064.273
14760	LOC10322(	67.63798	0.61176	0.325423	1.879887	0.060123	0.108291	56.91907	44.88777	57.7457	84.82083	94.12087	67.33362
5235	ERO1A	5643.586	0.611644	0.134091	4.561418	5.08E-06	2.58E-05	3947.473	5512.984	3933.665	6919.594	6652.634	6895.164
19418	LOC11961{	23.73808	0.611367	0.548187	1.115253	0.264742	0.362467	16.26259	22.44388	16.69755	35.71403	34.22577	17.08465
18196	LOC10321;	29.28856	0.611166	0.46922	1.302514	0.192741	0.281601	21.14137	20.25424	27.82925	35.71403	40.6431	30.14938
22475	LOC11962:	6.704836	0.611111	0.950821	0.642719	0.520406	0.615573	6.505036	5.474118	4.174388	4.464254	8.556443	11.05477
20783	LOC11962(	14.10771	0.61084	0.673735	0.906647	0.364594	0.466355	11.9259	9.306001	11.82743	24.5534	14.97377	12.05975

5450	AGPAT4	510.7332	0.610763	0.13756	4.439981	9.00E-06	4.39E-05	407.6489	398.5158	408.3943	636.1562	560.447	653.2367
6146	CYSTM1	1472.66	0.610473	0.14892	4.099339	4.14E-05	0.000179	1279.866	911.4407	1307.279	1852.665	1638.559	1846.147
19566	LOC11962:	20.2575	0.610297	0.560262	1.089305	0.276019	0.375037	13.01007	14.23271	20.87194	26.78552	23.53022	23.11453
5712	TRAPPC9	860.6276	0.609995	0.141227	4.319257	1.57E-05	7.29E-05	755.1263	632.2606	660.9448	1004.457	924.0958	1186.881
3754	RUBCN	2181.234	0.609906	0.109087	5.59098	2.26E-08	1.60E-07	1688.599	1827.808	1669.755	2531.232	2453.56	2916.45
12594	LOC10322:	143.9112	0.60985	0.266849	2.285379	0.022291	0.047054	92.69677	145.0641	102.2725	207.5878	171.1289	144.717
7519	ORAI1	553.8105	0.609843	0.171655	3.552721	0.000381	0.001348	499.2615	351.9858	465.4443	720.977	579.699	705.4956
18036	RAC2	30.1449	0.609485	0.455913	1.336845	0.181273	0.267188	23.30971	21.34906	27.13352	31.24978	40.6431	37.18424
23094	LOC11962;	6.133	0.609415	1.116379	0.545886	0.585145	0.673598	5.420864	3.831883	4.87012	6.696381	14.97377	1.004979
5060	KCTD11	644.0016	0.609393	0.13089	4.655755	3.23E-06	1.70E-05	527.45	511.83	493.9693	725.4413	755.1061	850.2126
21134	KCNE3	13.17526	0.609352	0.708316	0.860282	0.389633	0.49013	8.131296	11.49565	11.1317	17.85702	21.39111	9.044815
4675	EPB41L4B	3638.095	0.609325	0.124312	4.901595	9.51E-07	5.41E-06	2653.513	3426.251	2563.77	4390.594	4258.969	4535.472
16432	LOC10323:	47.48553	0.609102	0.384555	1.583915	0.113213	0.183165	41.19856	35.58177	34.78657	66.96381	64.17332	42.20914
10202	LOC11962‹	167.1909	0.608975	0.216284	2.815631	0.004868	0.012682	146.9054	119.8832	130.1018	225.4448	181.8244	198.9859
8127	TMEM102	326.5165	0.608951	0.180638	3.371117	0.000749	0.002449	281.3428	225.5337	272.031	374.9973	352.9533	452.2408
22493	LOC11961‹	7.000075	0.608906	0.951587	0.639884	0.522248	0.617257	7.589209	3.284471	5.565851	6.696381	12.83466	6.029877
4090	LOC10322(	922.1372	0.608885	0.114791	5.304272	1.13E-07	7.35E-07	725.8536	760.355	706.8631	1120.528	1052.442	1166.781
3828	PECR	1844.829	0.608753	0.110354	5.51638	3.46E-08	2.40E-07	1564.461	1306.672	1513.216	2296.859	2147.667	2240.099
6318	PLEKHG4	4450.588	0.608722	0.151518	4.017493	5.88E-05	0.000248	4014.692	2767.714	3798.693	5109.339	4735.991	6277.102
17917	LOC11962(	36.09186	0.608664	0.448268	1.357813	0.174523	0.258941	27.6464	21.34906	36.17803	46.87467	51.33866	33.16432
3114	DNASE1L1	4404.44	0.608625	0.097162	6.26403	3.75E-10	3.20E-09	3673.719	3345.781	3452.219	5267.82	4962.737	5724.363
19557	LOC10324!	19.61189	0.60841	0.558364	1.089631	0.275876	0.375015	14.09425	17.51718	15.30609	20.08914	23.53022	27.13445
14898	VMAC	120.324	0.608177	0.327813	1.855255	0.06356	0.113435	129.5586	58.02565	99.48959	151.7846	111.2338	171.8515
5009	TMEM120/	914.9303	0.607947	0.129802	4.683642	2.82E-06	1.50E-05	782.2306	682.6225	714.5161	1033.475	1039.608	1237.13
3577	ANXA6	4124.247	0.607825	0.105221	5.776673	7.62E-09	5.66E-08	3214.03	3468.949	3127.313	4741.038	4654.705	5539.447
6739	CUNH4orf:	409.389	0.607633	0.158169	3.841677	0.000122	0.000482	287.8479	362.934	321.4279	488.8358	521.943	473.3453
3694	TPM2	3761.449	0.607617	0.1076	5.647005	1.63E-08	1.18E-07	2736.452	3301.441	2907.461	4589.253	4233.3	4800.787
21175	LOC11962(	13.23441	0.607183	0.710476	0.854615	0.392765	0.493112	10.84173	10.94824	9.044508	24.5534	14.97377	9.044815
11130	ATP8B3	128.5062	0.60713	0.23381	2.596682	0.009413	0.022484	100.286	95.24966	109.2298	167.4095	156.1551	142.7071
5287	CLSTN2	663.3063	0.607054	0.134124	4.52607	6.01E-06	3.02E-05	559.9752	515.1145	505.7967	747.7625	765.8016	885.3869
3421	SERPINE1	41584.28	0.606726	0.102435	5.923036	3.16E-09	2.46E-08	32106.69	35355.14	31444.97	48463.94	46138.48	55996.45
5425	FEM1C	1613.632	0.606568	0.136354	4.448484	8.65E-06	4.24E-05	1153.56	1517.973	1168.829	1917.397	1807.549	2116.487
4319	SREBF1	7184.147	0.606466	0.117946	5.14191	2.72E-07	1.67E-06	5925.546	5168.115	6001.379	8435.208	7649.46	9925.177
4836	PMM1	1186.035	0.606459	0.126561	4.791827	1.65E-06	9.09E-06	974.6713	847.3935	1003.94	1377.222	1324.11	1588.873
22775	LOC11961‹	6.145209	0.606291	1.016335	0.596547	0.55081	0.642926	5.96295	4.926706	4.174388	4.464254	4.278221	13.06473
13377	LOC10324:	134.2304	0.606222	0.284417	2.131458	0.033051	0.065685	140.4004	71.71095	107.1426	158.481	166.8506	160.7967
8909	ZNF547	253.2719	0.605987	0.192869	3.141954	0.001678	0.005008	227.6763	167.508	206.6322	325.8905	299.4755	292.449
19348	MOV10L1	27.65395	0.605906	0.537422	1.127431	0.25956	0.356647	21.14137	19.15941	24.3506	42.41041	42.78221	16.07967
7995	ZC2HC1C	292.3754	0.605797	0.17746	3.413701	0.000641	0.002131	238.518	250.1672	206.6322	314.7299	391.4573	352.7478
19964	LOC10321‹	20.44486	0.605712	0.58478	1.035795	0.300298	0.399891	15.7205	16.96977	15.30609	20.08914	38.50399	16.07967
11417	LOC11962‹	152.1784	0.605661	0.239176	2.532285	0.011332	0.026386	126.3061	97.4393	137.7548	174.1059	209.6328	167.8316
3361	ESYT1	7327.824	0.605491	0.101309	5.976674	2.28E-09	1.80E-08	5917.957	5717.716	5808.661	8522.261	8092.256	9908.093
25158	LOC11961‹	1.192361	0.605472	2.744711	0.220596	0.825407	0.872272	0	0	2.782925	2.232127	2.139111	0
6542	LOC11961ᵋ	1474.921	0.605111	0.154227	3.923507	8.73E-05	0.000355	1213.189	996.2895	1306.584	1709.809	1493.099	2130.556
2789	NDUFA5	2625.737	0.605074	0.090077	6.717301	1.85E-11	1.76E-10	2017.103	2133.811	2096.239	3218.727	3202.249	3086.292
18895	LOC11961‹	28.56351	0.60506	0.502105	1.205046	0.228185	0.321094	25.47806	19.15941	23.65487	44.64254	19.252	39.1942
5666	PLEKHA4	608.6908	0.604911	0.139379	4.340042	1.42E-05	6.68E-05	439.632	541.9377	468.2272	714.2806	727.2976	760.7695
2984	GALNT11	2917.092	0.604881	0.093841	6.445798	1.15E-10	1.02E-09	2325.008	2358.798	2263.91	3415.154	3384.073	3755.608
4777	TBCC	604.8222	0.604613	0.125304	4.825189	1.40E-06	7.78E-06	480.2885	473.5112	485.6205	761.1553	703.7674	724.5902
13673	BICRA	78.98783	0.604512	0.291476	2.073966	0.038082	0.074045	55.8349	67.87906	64.00729	104.91	89.84265	91.45313
6696	LOC11962:	392.0828	0.603745	0.156472	3.858496	0.000114	0.000453	336.6356	288.486	310.9919	444.1933	455.6306	516.5594
19629	CUNH19or	22.69992	0.603395	0.558413	1.080554	0.279895	0.379083	19.51511	11.49565	22.95914	35.71403	21.39111	25.12449
12633	ZNF385A	166.5177	0.603371	0.265295	2.274336	0.022946	0.048283	92.15468	175.1718	128.7103	212.0521	201.0764	189.9411
3534	UBL3	2139.61	0.603179	0.103591	5.822692	5.79E-09	4.36E-08	1744.434	1746.244	1609.227	2566.946	2400.082	2770.728
23529	LOC10323;	6.498696	0.603139	1.260089	0.478648	0.632189	0.714299	8.131296	5.474118	1.391463	11.16063	12.83466	0
21373	LOC10324‹	15.3638	0.603008	0.734515	0.820961	0.411668	0.512057	8.131296	20.25424	7.653045	31.24978	12.83466	12.05975
23975	LOC11962;	3.238956	0.602717	1.467072	0.41083	0.681197	0.755355	0.542086	1.094824	6.261582	2.232127	4.278221	5.024897
3404	LPCAT1	1862.818	0.60265	0.101413	5.942535	2.81E-09	2.19E-08	1517.3	1516.331	1407.465	2205.341	2147.667	2382.806
10675	S1PR2	144.4358	0.602633	0.223345	2.698217	0.006971	0.017361	106.2489	122.6202	116.1871	176.338	158.2942	186.9262
3324	ARHGAP1	7762.712	0.602112	0.100012	6.020387	1.74E-09	1.39E-08	6186.29	6226.262	6092.52	9069.132	8554.304	10447.77
22033	PPP1R36	9.728637	0.602001	0.836156	0.719963	0.471548	0.568969	7.589209	6.02153	9.044508	17.85702	12.83466	5.024897
20410	RASL10A	17.2808	0.60176	0.62697	0.959791	0.337161	0.439168	15.7205	10.94824	13.91463	20.08914	29.94755	13.06473
4927	SLC25A44	1503.294	0.601488	0.12719	4.729034	2.26E-06	1.22E-05	1151.391	1378.93	1054.033	1758.916	1769.045	1907.451
5445	UTP23	1711.239	0.601392	0.135389	4.441953	8.91E-06	4.35E-05	1196.385	1628.55	1251.621	2122.753	2113.441	1954.685
23806	PP2D1	4.121951	0.601382	1.380649	0.43558	0.663142	0.740554	2.168345	3.831883	3.478657	6.696381	8.556443	0
21537	SOX9	10.84851	0.601364	0.752313	0.799353	0.424086	0.523486	7.589209	8.211177	9.740239	13.39276	17.11289	9.044815
25266	LOC11962:	0.866543	0.601326	2.946718	0.204066	0.838302	0.882065	0.542086	1.642235	0	0	0	3.014938
11460	LOC10324;	225.7537	0.601309	0.238169	2.524711	0.011579	0.026862	214.6662	122.0728	201.7621	267.8552	273.8062	274.3594
14436	FRAT1	74.9636	0.601296	0.310375	1.937323	0.052706	0.097062	60.71367	60.2153	57.04997	75.89232	115.512	80.39836
23359	LOC11961‹	4.11195	0.600615	1.189389	0.504978	0.613574	0.698312	2.710432	2.189647	4.87012	4.464254	6.417332	4.019918
21518	LOC11962;	11.7954	0.600513	0.749227	0.80151	0.422837	0.522405	8.131296	10.40082	9.044508	22.32127	12.83466	8.039836
19005	LOC11962:	30.33575	0.60027	0.506942	1.184101	0.236373	0.330636	20.59928	31.20247	20.87194	46.87467	19.252	43.21412
4406	RIPK2	1271.495	0.600156	0.118217	5.076731	3.84E-07	2.32E-06	1012.075	1100.845	921.8441	1457.579	1499.517	1637.112
3290	RNGTT	1839.677	0.599594	0.099097	6.050549	1.44E-09	1.17E-08	1455.502	1488.96	1447.817	2093.735	2205.423	2346.627

2730	STMP1	2925.999	0.599573	0.0881	6.805581	1.01E-11	9.80E-11	2258.332	2390	2330.004	3564.707	3548.785	3464.164
23642	LOC11962:	3.816974	0.59941	1.295533	0.462675	0.643598	0.723714	2.168345	3.831883	2.782925	6.696381	6.417332	1.004979
4549	CACNB3	1517.474	0.599143	0.120429	4.975078	6.52E-07	3.81E-06	1273.903	1058.694	1291.277	1805.791	1730.541	1944.635
6430	LOC11962(	448.0734	0.59903	0.150958	3.968187	7.24E-05	0.000299	332.2989	378.2616	357.6059	602.6743	491.9955	525.6043
11332	CUNH20or	144.8511	0.598994	0.234827	2.550782	0.010748	0.025215	100.8281	126.9995	118.2743	147.3204	196.7982	178.8863
7951	LOC10323:	347.1884	0.598851	0.17488	3.424342	0.000616	0.002061	242.8547	326.8049	258.1163	419.6399	425.683	410.0316
18525	CUNH9orf:	26.16839	0.598622	0.480091	1.246893	0.212437	0.304865	20.0572	20.80165	21.56767	33.4819	29.94755	31.15436
19667	LOC11962;	20.62066	0.598524	0.556666	1.075194	0.282288	0.381565	12.46799	16.42235	20.17621	29.01765	23.53022	22.10955
5701	SYNPO	1904.846	0.598455	0.138313	4.3268	1.51E-05	7.06E-05	1328.112	1849.705	1367.808	2330.341	2239.649	2313.463
18504	LOC10321!	27.95427	0.598267	0.478117	1.2513	0.210825	0.302896	21.68345	23.53871	20.87194	42.41041	32.08666	27.13445
21698	UBAP1L	12.64981	0.598095	0.769071	0.777685	0.436755	0.535099	13.01007	5.474118	11.1317	17.85702	21.39111	7.034856
17446	FOSB	39.13313	0.598057	0.421442	1.419071	0.155878	0.237521	23.30971	36.12918	33.39511	53.57105	49.19955	39.1942
24849	LOC11961!	1.185248	0.598009	2.184986	0.27369	0.784323	0.839098	1.626259	0.547412	0.695731	2.232127	0	2.009959
11529	TMEM198	168.0589	0.598004	0.238431	2.508083	0.012139	0.027991	146.9054	107.2927	148.8865	203.1236	168.9897	233.1552
23793	LOC11962‹	3.772773	0.597838	1.368393	0.436891	0.662191	0.739748	2.710432	2.737059	3.478657	0	10.69555	3.014938
17463	CUNH1orf!	41.71651	0.597732	0.421912	1.416721	0.156564	0.238348	41.74065	22.9913	35.4823	46.87467	44.92132	58.28881
18359	LOC10322;	31.54186	0.59765	0.469858	1.271982	0.20338	0.294491	27.6464	19.15941	27.82925	49.10679	36.36488	29.1444
18955	BHLHA15	27.64694	0.597393	0.500438	1.193741	0.232579	0.32618	22.76763	18.06459	24.3506	37.94616	40.6431	22.10955
20288	PTPN22	19.52768	0.597047	0.60925	0.979971	0.327101	0.428626	17.88885	10.40082	18.08902	35.71403	14.97377	20.09959
17371	ARHGAP3C	42.58448	0.596921	0.415727	1.435849	0.151045	0.231163	25.47806	36.67659	38.96096	46.87467	66.31243	41.20416
20244	LOC11962:	17.35199	0.596855	0.603883	0.988362	0.322975	0.42412	17.88885	11.49565	11.82743	24.5534	19.252	19.09461
22648	LOC11962‹	6.162322	0.596818	0.969574	0.615547	0.538194	0.63175	3.794605	6.02153	4.87012	6.696381	8.556443	7.034856
7854	LOC11962(	409.5	0.596811	0.172868	3.452416	0.000556	0.001881	294.3529	372.7874	313.0791	508.925	417.1266	550.7287
4511	EVI5L	887.6915	0.596793	0.119206	5.006412	5.55E-07	3.27E-06	706.3385	728.6051	685.9911	1129.456	973.2954	1102.462
6685	LOC10322!	636.6098	0.59652	0.154418	3.863019	0.000112	0.000445	527.9921	430.2657	564.9339	792.4051	675.959	828.1031
3171	CPQ	3464.087	0.596066	0.096224	6.194531	5.85E-10	4.90E-09	2816.139	2774.283	2690.393	4073.632	3916.712	4513.363
18870	CUNH6orf:	28.68947	0.596023	0.493096	1.208736	0.226764	0.319477	23.8518	20.25424	24.3506	22.32127	49.19955	32.15934
5750	CTSO	941.3831	0.595888	0.138574	4.300148	1.71E-05	7.89E-05	685.1972	884.0701	679.0338	1118.296	1155.12	1126.582
3160	FURIN	6417.625	0.59574	0.095973	6.207388	5.39E-10	4.53E-09	5123.258	4943.676	5272.252	7758.873	7063.343	8344.345
19109	LOC10322:	24.98119	0.595059	0.509664	1.16755	0.242988	0.338052	15.7205	24.08612	19.48048	31.24978	34.22577	25.12449
3738	PARP9	2848.793	0.594882	0.10613	5.605238	2.08E-08	1.48E-07	2192.739	2488.534	2130.329	3437.476	3208.666	3635.011
15249	DNAAF4	92.34308	0.594734	0.332474	1.788815	0.073645	0.128392	67.21871	91.96518	60.52863	82.5887	158.2942	93.46309
5590	MCOLN1	511.5715	0.594672	0.135869	4.376814	1.20E-05	5.73E-05	429.8745	393.5891	400.0455	580.353	637.455	628.1122
3941	NSMCE2	1242.454	0.594576	0.109854	5.412439	6.22E-08	4.19E-07	1031.048	919.1044	1017.855	1542.4	1510.212	1434.106
15377	LOC1032‹!	66.48709	0.593871	0.33666	1.764006	0.077731	0.134388	66.67662	40.50847	51.48412	82.5887	81.28621	76.37844
2898	NSMAF	3147.585	0.593669	0.090605	6.552255	5.67E-11	5.20E-10	2492.513	2570.098	2467.759	3604.885	3792.643	3957.609
19077	LOC10322!	26.43308	0.593607	0.505872	1.173434	0.240622	0.335322	27.10432	18.612	18.08902	26.78552	27.80844	40.19918
8749	LOC11962:	307.8856	0.593586	0.186111	3.189425	0.001426	0.004332	278.6324	213.4906	241.4188	397.3186	397.8746	318.5785
15028	LOC11962‹	78.17648	0.59357	0.324745	1.827802	0.067579	0.119542	62.33993	65.14201	57.7457	89.28508	126.2075	68.3386
3647	CREG1	7117.004	0.593504	0.10413	5.699634	1.20E-08	8.75E-08	5393.217	6423.33	5203.375	8582.528	8436.652	8662.923
14283	IQCN	84.40399	0.593388	0.301937	1.965272	0.049383	0.091938	63.42411	75.54283	61.22436	104.91	121.9293	79.39338
18118	LOC10321!	35.85126	0.593349	0.450224	1.317897	0.187538	0.275179	29.81475	29.01283	25.74206	44.64254	57.75599	28.13943
19669	LOC11961{	22.65755	0.593292	0.551841	1.075113	0.282324	0.381595	18.43094	15.87494	20.87194	22.32127	19.252	39.1942
2451	SLC35G1	12042.49	0.592536	0.082335	7.196622	6.17E-13	6.69E-12	9811.763	9662.366	9340.889	14258.83	14006.9	15174.19
4891	LCAT	736.1642	0.592419	0.12477	4.74809	2.05E-06	1.12E-05	583.2849	602.153	579.5442	832.5834	855.6443	963.7753
4428	TCP11L1	2966.855	0.592351	0.117092	5.058866	4.22E-07	2.53E-06	2186.234	2718.447	2196.424	3428.547	3469.638	3801.837
6799	ZNF341	387.078	0.592281	0.155138	3.817771	0.000135	0.000527	313.868	335.016	276.2054	459.8182	481.2999	456.2607
15944	LOC11962:	56.15759	0.592204	0.353029	1.677492	0.093446	0.155831	53.66655	45.43518	35.4823	66.96381	62.03421	73.3635
16444	CBLN3	48.25105	0.592146	0.3745	1.581162	0.113841	0.184047	43.909	39.41365	31.30791	64.73168	59.8951	50.24897
22924	TRNAG-UC	7.458275	0.592128	1.028602	0.575663	0.564843	0.655025	3.794605	2.189647	11.82743	15.62489	4.278221	7.034856
21884	NPW	13.56629	0.592038	0.792678	0.746883	0.455134	0.552904	9.215468	6.02153	16.69755	15.62489	27.80844	6.029877
3801	ISCU	3072.586	0.591996	0.106852	5.540356	3.02E-08	2.11E-07	2345.608	2711.878	2298.001	3653.992	3499.585	3926.455
15987	LOC11961{	66.40733	0.59182	0.354783	1.66812	0.095292	0.158462	49.32986	48.17224	60.52863	64.73168	113.3729	62.30873
13442	TBXAS1	97.88139	0.591672	0.279268	2.118655	0.03412	0.06748	80.77087	68.42648	83.48776	133.9276	126.2075	94.46807
6107	LGALS8	647.591	0.591604	0.143696	4.117067	3.84E-05	0.000167	464.0259	590.6573	496.7522	732.1376	782.9145	819.0583
21610	LOC1032‹:	17.03077	0.59158	0.749306	0.789504	0.429817	0.528769	17.88885	7.663765	14.61036	42.41041	8.556443	11.05477
3045	SYTL2	2563.516	0.591525	0.093211	6.346097	2.21E-10	1.93E-09	2089.743	2068.122	1980.747	3060.246	2956.251	3225.984
19473	LOC11961{	25.26736	0.591506	0.535516	1.104554	0.269353	0.367727	15.7205	17.51718	27.13352	22.32127	42.78221	26.12947
22231	LOC10322;	9.046825	0.591378	0.86054	0.687217	0.491946	0.588295	7.047123	8.211177	6.261582	4.464254	19.252	9.044815
21495	LOC10322!	11.23091	0.59136	0.735432	0.804099	0.42134	0.521113	8.673382	7.663765	10.43597	17.85702	10.69555	12.05975
3783	MIDEAS	3450.79	0.591278	0.106371	5.558661	2.72E-08	1.91E-07	2574.91	3049.631	2638.909	4075.864	3944.52	4420.905
4119	CPT2	1292.617	0.591089	0.112022	5.276561	1.32E-07	8.50E-07	1010.449	1116.72	969.1538	1493.293	1538.021	1628.067
22983	TRNAS-AG,	5.901486	0.590915	1.042037	0.567077	0.570662	0.660098	2.168345	5.474118	6.261582	8.928508	8.556443	4.019918
2825	OAT	5405.293	0.590649	0.088751	6.655121	2.83E-11	2.66E-10	4397.405	4494.251	4051.94	6361.562	6434.445	6692.158
24009	LOC11962:	3.496231	0.590294	1.456818	0.405194	0.685335	0.758867	1.626259	4.379295	2.782925	0	2.139111	10.04979
5751	FAM185A	500.0349	0.590209	0.137255	4.300079	1.71E-05	7.89E-05	424.4536	397.421	376.3907	593.7458	586.1163	622.0823
10412	SLC37A1	166.7499	0.590008	0.213495	2.76357	0.005717	0.014598	137.1479	126.9995	137.0591	174.1059	201.0764	224.1104
14951	LOC10322(	77.54327	0.589637	0.319517	1.845399	0.06498	0.115543	73.72375	47.62483	63.31155	102.6778	100.5382	77.38342
5663	LOC11962(	737.0947	0.589616	0.135824	4.341026	1.42E-05	6.66E-05	602.258	519.4938	645.6387	912.9399	825.6967	916.5413
5299	HHAT	616.4345	0.589498	0.130478	4.517994	6.24E-06	3.13E-05	496.009	511.2826	471.0101	680.7987	763.6625	775.8442
3761	UROD	1363.843	0.589399	0.105556	5.583778	2.35E-08	1.66E-07	1109.109	1051.578	1110.387	1595.971	1574.385	1741.629
21915	LOC10322;	10.31637	0.589398	0.794298	0.742036	0.458066	0.555677	5.420864	7.116354	12.52316	8.928508	12.83466	15.07469
4437	KDM3A	1677.283	0.589288	0.116657	5.051452	4.38E-07	2.63E-06	1327.027	1446.262	1249.534	1904.004	1903.808	2233.064
5112	LOC10322:	1094.863	0.588966	0.127419	4.622292	3.80E-06	1.97E-05	941.0619	806.885	880.1002	1263.384	1206.458	1471.29

2666	LOC10324(	7046.469	0.588314	0.085549	6.876912	6.12E-12	6.10E-11	5522.776	5954.746	5411.399	8316.905	8462.322	8610.664
19580	LOC10323{	27.36875	0.588228	0.540914	1.08747	0.276829	0.375811	16.80468	31.20247	16.69755	33.4819	44.92132	21.10457
5147	GPHN	1466.501	0.588182	0.127716	4.605375	4.12E-06	2.13E-05	1093.388	1352.655	1070.731	1756.684	1649.254	1876.297
5227	PRDX1	34139.59	0.588078	0.128789	4.566215	4.97E-06	2.53E-05	30387.74	21530.25	29912.97	40749.71	39787.46	42469.43
17186	LOC10321§	42.04754	0.588073	0.400207	1.469423	0.141718	0.219224	36.86187	27.918	36.17803	40.17829	59.8951	51.25395
3105	ATP5MF	6273.158	0.588027	0.093739	6.273057	3.54E-10	3.03E-09	5030.019	4687.487	5315.388	7783.427	7726.468	7096.16
3756	CELSR2	7515.593	0.587852	0.105234	5.586139	2.32E-08	1.64E-07	6193.337	5754.393	6075.822	8571.368	8289.054	10209.59
21092	LOC11961§	165.8854	0.587668	0.678366	0.8663	0.386326	0.486937	54.20864	261.1154	82.0963	223.2127	196.7982	177.8814
5551	SYNJ1	969.5412	0.587537	0.133774	4.391997	1.12E-05	5.38E-05	697.1231	893.3761	735.3881	1149.545	1118.755	1223.06
9158	PUSL1	531.982	0.587498	0.191771	3.063534	0.002187	0.00635	482.4569	306.5506	488.4034	633.9241	586.1163	694.4408
4155	MYO5B	3138.796	0.587488	0.111827	5.253539	1.49E-07	9.55E-07	2476.793	2710.236	2343.223	3674.081	3439.69	4188.754
5748	SEC62	6327.262	0.587149	0.136528	4.300584	1.70E-05	7.88E-05	4374.095	6274.434	4522.95	7258.877	7844.119	7689.098
17580	LOC11962{	50.05863	0.586805	0.41805	1.403671	0.160417	0.242587	48.78777	37.77142	32.00364	58.0353	85.56443	38.18922
4615	CASP6	1072.94	0.586721	0.118837	4.93721	7.92E-07	4.57E-06	895.5267	886.8071	791.7423	1180.795	1356.196	1326.573
24196	LOC11962(	2.641475	0.586717	1.56815	0.374146	0.708296	0.77823	2.168345	0.547412	3.478657	2.232127	6.417332	1.004979
24434	LOC10323{	1.774565	0.58614	1.724806	0.33983	0.733985	0.798631	1.084173	1.094824	2.087194	2.232127	2.139111	2.009959
4827	MAP3K12	1088.806	0.586118	0.122221	4.795542	1.62E-06	8.94E-06	902.5738	869.29	842.5307	1377.222	1142.285	1398.931
22457	LOC11961{	7.946888	0.586083	0.904413	0.648026	0.516968	0.611996	4.336691	8.758589	5.565851	11.16063	12.83466	5.024897
13213	LOC10324	105.4936	0.585879	0.270534	2.165635	0.030339	0.061043	87.27591	71.16354	93.92374	118.3027	149.7377	112.5577
22512	TRNAK-UU	7.388784	0.585807	0.918305	0.637922	0.523524	0.618243	4.878777	4.926706	8.348776	6.696381	6.417332	13.06473
3182	KDELR3	3012.053	0.585659	0.094719	6.183141	6.28E-10	5.25E-09	2416.079	2422.297	2394.012	3462.029	3484.611	3893.29
21775	LOC10322{	10.60308	0.585431	0.76646	0.763812	0.444979	0.543273	7.047123	7.116354	11.1317	11.16063	17.11289	10.04979
5556	LOC10323§	1175.743	0.585253	0.133311	4.390133	1.13E-05	5.42E-05	960.035	858.8891	1007.419	1424.097	1217.154	1586.863
4384	SAYSD1	920.2664	0.585237	0.114956	5.090982	3.56E-07	2.16E-06	764.8839	694.1182	751.3899	1095.974	1069.555	1145.677
3958	PLCD1	1707.336	0.585233	0.10828	5.404807	6.49E-08	4.36E-07	1376.899	1292.439	1432.511	2035.7	1899.53	2206.935
20705	MESP2	17.19097	0.584923	0.6359	0.919835	0.357659	0.45923	14.09425	15.87494	11.1317	31.24978	10.69555	20.09959
7936	HIVEP3	545.791	0.584888	0.170582	3.428774	0.000606	0.002031	368.6187	535.3688	407.0029	662.9417	605.3683	695.4458
19707	EFCAB12	21.19013	0.584806	0.547192	1.068741	0.285186	0.38472	20.59928	15.32753	15.30609	20.08914	25.66933	30.14938
19555	LOC11962	24.71119	0.584492	0.536122	1.090222	0.275615	0.374699	24.39389	13.6853	20.87194	24.5534	40.6431	24.11951
23061	LOC10322	6.147681	0.584102	1.057861	0.552153	0.580843	0.669603	8.131296	2.737059	4.174388	2.232127	8.556443	11.05477
16886	IGSF1	68.59268	0.58409	0.387147	1.508703	0.131375	0.206822	53.12446	52.55153	57.04997	84.82083	119.7902	44.2191
6880	DLGAP4	1261.431	0.583586	0.154162	3.78555	0.000153	0.000593	838.0655	1256.31	935.7587	1506.686	1443.9	1587.868
3209	TMX4	5774.148	0.583482	0.094847	6.151807	7.66E-10	6.35E-09	4471.67	4929.991	4469.378	6772.273	6594.878	7406.699
10077	MCF2L	198.387	0.583219	0.20517	2.842617	0.004474	0.011805	163.7101	155.465	157.931	194.195	273.8062	245.215
11705	RAB26	157.3058	0.583216	0.236018	2.471062	0.013471	0.030597	142.0266	101.8186	134.9719	162.9453	201.0764	200.9959
3651	SLC6A8	10235.73	0.58302	0.102331	5.697422	1.22E-08	8.86E-08	8151.353	8417.551	8023.87	11986.52	11069.9	13765.2
6233	WDR44	1087.62	0.582892	0.143576	4.059824	4.91E-05	0.000209	772.4731	1053.768	786.1764	1290.169	1300.579	1322.553
18591	MAMSTR	33.75557	0.582663	0.471265	1.236383	0.216316	0.309331	26.02015	27.37059	26.43779	55.80317	42.78221	24.11951
12237	FRAT2	124.3055	0.582634	0.246914	2.359662	0.018292	0.039739	111.6698	100.1764	85.57496	167.4095	143.3204	137.6822
4721	DBR1	1464.193	0.58245	0.119608	4.869664	1.12E-06	6.30E-06	1171.991	1299.008	1044.293	1863.826	1700.593	1705.45
4219	MRPL14	4858.461	0.582447	0.111862	5.206835	1.92E-07	1.21E-06	4124.193	3323.885	4225.872	5899.512	5675.061	5902.244
5944	INSIG2	541.6017	0.582171	0.138983	4.18879	2.80E-05	0.000125	443.4267	460.3733	395.8711	694.1915	624.6203	631.1271
3827	SELENOT	10089.81	0.582125	0.105524	5.516521	3.46E-08	2.40E-07	7651.549	7978.527	8608.284	12323.57	13251.79	10725.14
23790	LOC10324(	3.225455	0.581517	1.328305	0.437789	0.661539	0.739261	2.168345	2.737059	2.782925	2.232127	6.417332	3.014938
5828	UBXN7	2204.558	0.581303	0.136452	4.26012	2.04E-05	9.32E-05	1594.276	2153.518	1551.481	2593.732	2611.854	2722.489
11383	SCN3A	136.7455	0.581055	0.22862	2.54158	0.011035	0.025773	112.2119	109.4824	107.1426	142.8561	183.9635	164.8166
15957	LOC10322	60.91713	0.580954	0.347076	1.673851	0.09416	0.156864	43.36691	56.38342	46.614	55.80317	91.98176	71.35354
24780	PMCH	1.450002	0.580658	2.032525	0.285683	0.775121	0.831581	1.626259	1.094824	0.695731	0	4.278221	1.004979
5411	LOC10321{	4970.703	0.580469	0.130255	4.456421	8.33E-06	4.09E-05	4381.684	3460.737	4115.947	5667.37	5277.186	6921.294
5435	ZNF436	869.8076	0.580389	0.130574	4.444908	8.79E-06	4.30E-05	667.8504	791.0101	633.1155	1026.778	1026.773	1073.318
3350	STT3B	11688.51	0.580369	0.096831	5.993607	2.05E-09	1.63E-08	8959.061	10367.98	8780.826	13604.81	13891.38	14526.98
14862	LOC10324	66.74056	0.580343	0.311949	1.860379	0.062832	0.112393	52.04029	56.93083	52.17985	78.12444	72.72976	88.43819
3752	ATP6V1G1	7176.556	0.580193	0.103755	5.591935	2.25E-08	1.59E-07	5967.829	5113.921	6173.224	9002.168	8721.154	8081.04
5351	SLC35E3	564.26	0.579744	0.129147	4.489034	7.15E-06	3.55E-05	430.4166	466.9423	460.5742	667.406	673.8199	686.401
18679	DEGS2	31.30893	0.579734	0.470769	1.231462	0.21815	0.310493	33.06727	18.06459	24.3506	37.94616	34.22577	40.19918
4696	LSM1	936.5051	0.579347	0.118571	4.886096	1.03E-06	5.82E-06	752.4159	698.4975	800.0911	1151.778	1152.981	1063.268
10373	FAM161B	203.2189	0.579216	0.209059	2.770589	0.005595	0.014341	168.0468	174.077	144.0164	279.0159	248.1368	206.0208
3367	FBXL3	2165.895	0.578867	0.096944	5.971142	2.36E-09	1.86E-08	1761.239	1788.942	1664.189	2540.161	2511.316	2729.524
9133	LOC11962	304.6865	0.578393	0.188275	3.072061	0.002126	0.006188	205.9928	294.5076	233.07	354.9082	365.7879	373.8524
3111	IDI1	8609.929	0.578363	0.092267	6.268335	3.65E-10	3.12E-09	6730.002	7498.447	6489.086	10357.07	10569.35	10015.63
8907	AMIGO2	288.9042	0.577677	0.183789	3.143152	0.001671	0.004989	230.9288	204.1846	260.8993	383.9258	303.7537	349.7329
24262	IL36G	2.622411	0.577579	1.573077	0.367165	0.713496	0.781811	1.626259	3.284471	1.391463	0	6.417332	3.014938
15953	LOC10322	66.99105	0.577261	0.344581	1.675254	0.093884	0.156454	52.04029	41.05589	67.48594	93.74933	81.28621	66.32864
6635	CARD19	569.0443	0.577224	0.148537	3.886061	0.000102	0.000408	498.7195	413.8433	460.5742	683.0309	605.3683	752.7296
6203	ARHGEF6	1105.573	0.577216	0.141799	4.07066	4.69E-05	0.000201	793.0724	1066.358	802.874	1272.312	1339.083	1359.737
16847	KCNK6	51.50461	0.576555	0.380392	1.515689	0.129598	0.204509	34.69353	38.31883	50.09266	66.96381	72.72976	46.22906
3149	RAD21	11225.3	0.576446	0.092679	6.219806	4.98E-10	4.20E-09	8647.904	9705.611	8686.902	12950.8	13099.91	14260.66
23685	LOC11962(	4.379697	0.576443	1.264529	0.455856	0.648494	0.727895	3.794605	3.284471	3.478657	0	10.69555	5.024897
22818	LOC11961{	6.182072	0.576111	0.976896	0.589736	0.555368	0.647053	4.878777	4.926706	4.87012	6.696381	10.69555	5.024897
4484	TTC7A	2720.212	0.576051	0.114739	5.02054	5.15E-07	3.05E-06	2360.244	2067.574	2131.025	3060.246	3028.981	3673.2
24910	LOC11962	1.17152	0.575926	2.192289	0.262705	0.792778	0.846086	0.542086	1.642235	0.695731	0	2.139111	2.009959
3369	PRICKLE2	4292.957	0.575761	0.096438	5.970298	2.37E-09	1.87E-08	3328.41	3648.5	3370.818	4908.447	4969.154	5532.412
22273	LOC11962!	9.693451	0.575698	0.848719	0.678314	0.497572	0.593901	9.215468	5.474118	8.348776	6.696381	21.39111	7.034856
22856	ARTN	5.901815	0.575643	0.984191	0.584889	0.558622	0.649734	4.878777	4.379295	4.87012	6.696381	8.556443	6.029877

18964	LOC10322	28.00279	0.575259	0.482489	1.192273	0.233154	0.326851	26.02015	19.70683	21.56767	26.78552	42.78221	31.15436
17429	LOC10321	43.03867	0.575189	0.404009	1.423704	0.154532	0.235735	34.69353	38.31883	29.91645	44.64254	68.45154	42.20914
20046	LOC11962	25.94675	0.575028	0.56317	1.021056	0.307228	0.407463	20.59928	31.20247	11.1317	29.01765	23.53022	40.19918
4818	SIL1	1173.368	0.574814	0.11973	4.800933	1.58E-06	8.71E-06	982.8026	884.6175	965.6751	1332.58	1319.831	1554.703
4235	LOC11961	3060.495	0.574734	0.110605	5.196255	2.03E-07	1.28E-06	2626.408	2258.621	2495.588	3738.813	3289.952	3953.589
4388	CRAT	1365.601	0.57447	0.112901	5.088257	3.61E-07	2.19E-06	1070.078	1092.087	1134.738	1611.596	1495.238	1789.868
20992	LOC10323	16.19561	0.574364	0.652808	0.879836	0.378948	0.47989	18.43094	8.758589	11.82743	13.39276	25.66933	19.09461
22037	IFI27	9.706279	0.574005	0.798415	0.71893	0.472184	0.569633	8.131296	8.758589	6.261582	8.928508	17.11289	9.044815
20766	HNRNPA1L	19.46885	0.574	0.631542	0.908887	0.36341	0.465243	24.39389	11.49565	10.43597	22.32127	32.08666	16.07967
3924	KIF1B	5839.987	0.573823	0.105768	5.425284	5.79E-08	3.92E-07	4447.819	5209.718	4427.634	6732.095	6661.191	7561.466
6710	CYS1	416.8724	0.573674	0.148967	3.851015	0.000118	0.000466	365.3662	311.4773	328.3852	482.1394	513.3866	500.4798
21345	EPGN	15.04032	0.573608	0.69526	0.825025	0.409357	0.509851	16.26259	7.663765	11.82743	15.62489	27.80844	11.05477
24221	HCST	2.672053	0.573383	1.546349	0.370798	0.710788	0.780129	0.542086	1.094824	4.87012	2.232127	4.278221	3.014938
7057	ABHD18	438.2527	0.573147	0.154559	3.708264	0.000209	0.000786	382.713	316.9514	354.823	544.639	554.0297	476.3603
16556	LOC11962	48.47592	0.57302	0.366237	1.564615	0.117673	0.188955	40.11439	39.41365	38.26523	49.10679	55.61688	68.3386
18279	ZNF556	33.74568	0.572965	0.444988	1.287597	0.197886	0.287808	27.6464	30.65506	22.2634	49.10679	40.6431	32.15934
4020	TMEM68	1115.763	0.572814	0.106928	5.357002	8.46E-08	5.60E-07	930.7623	865.4581	894.0148	1321.419	1375.448	1307.478
13548	LOC10324	139.3981	0.572609	0.272849	2.098629	0.03585	0.070347	138.232	86.49107	109.9256	160.7131	205.3546	135.6722
23308	FIBCD1	5.06894	0.57225	1.117316	0.512165	0.608535	0.694093	2.710432	4.379295	4.87012	11.16063	4.278221	3.014938
13781	SETD9	84.33949	0.572154	0.278991	2.050798	0.040287	0.077717	69.92914	62.40495	71.66033	95.98146	100.5382	105.5228
18772	LOC10321	46.53549	0.572027	0.467878	1.2226	0.221481	0.313795	42.28274	29.56024	38.96096	53.57105	87.70354	27.13445
4010	LRRC75B	1028.261	0.571894	0.106602	5.364752	8.11E-08	5.37E-07	844.0285	806.885	830.0075	1236.598	1229.989	1222.055
8866	PGGHG	426.8112	0.571822	0.181408	3.152126	0.001621	0.00486	382.713	268.2318	381.2608	479.9073	491.9955	556.7586
23312	LOC11962	4.736482	0.571765	1.117483	0.511654	0.608893	0.694352	5.420864	2.737059	3.478657	4.464254	4.278221	8.039836
19652	FOXP3	22.77406	0.571688	0.530506	1.077626	0.281201	0.38041	15.7205	16.42235	22.95914	22.32127	32.08666	27.13445
16353	TMTC2	59.08895	0.571585	0.358221	1.595623	0.110573	0.179758	46.07734	54.19377	41.04815	66.96381	91.98176	54.26889
10149	PPP1R3D	184.1661	0.571512	0.202245	2.825846	0.004716	0.012352	148.5317	137.4004	160.0182	216.5163	205.3546	237.1752
5932	RAB7A	6682.759	0.571066	0.136183	4.19336	2.75E-05	0.000123	4587.677	6680.614	4863.162	7883.872	7948.935	8132.294
6904	DHRS4	420.9909	0.571022	0.15124	3.775612	0.00016	0.000615	341.5144	362.934	309.6005	542.4069	494.1346	475.3553
10035	TXNRD2	345.6249	0.57087	0.199987	2.854527	0.00431	0.011418	318.7468	220.607	297.773	410.7114	346.5359	479.3752
22994	LOC11962	5.912348	0.570861	1.010788	0.564768	0.572231	0.661602	5.96295	3.831883	4.174388	8.928508	8.556443	4.019918
19455	SNX22	25.48367	0.570464	0.514168	1.109489	0.267219	0.365152	25.47806	15.32753	20.17621	35.71403	32.08666	24.11951
8128	CCDC74B	555.8443	0.570168	0.169139	3.370999	0.000749	0.00245	471.6151	380.9986	493.9693	640.6204	569.0034	778.8591
6242	ZBTB5	574.5255	0.569844	0.140563	4.054009	5.03E-05	0.000214	459.1472	499.2396	432.0492	649.549	652.4288	754.7396
20322	LOC10323	19.83965	0.569754	0.584649	0.974523	0.329797	0.431436	12.46799	18.06459	16.69755	26.78552	29.94755	15.07469
5595	SLC13A3	517.954	0.569639	0.130195	4.375264	1.21E-05	5.76E-05	422.8274	412.7485	415.3516	620.5313	618.203	618.0624
5230	ST3GAL5	1046.765	0.56962	0.124813	4.56378	5.02E-06	2.55E-05	816.3821	918.0096	797.3082	1238.83	1142.285	1367.777
5533	SRSF12	848.4233	0.569553	0.129482	4.398712	1.09E-05	5.23E-05	657.5508	753.7861	634.507	1084.814	1018.217	941.6658
2846	BNIP3	3590.493	0.569501	0.086035	6.619434	3.61E-11	3.37E-10	2974.428	2805.486	2894.243	4232.113	4276.082	4360.606
22701	LOC10321	7.76699	0.569439	0.936072	0.608328	0.54297	0.635868	6.505036	6.02153	6.261582	15.62489	2.139111	10.04979
3524	CTDSPL	3957.067	0.569432	0.097618	5.833252	5.44E-09	4.10E-08	3116.455	3401.617	3043.129	4796.841	4408.707	4975.653
8971	ZNF613	371.8058	0.569138	0.182115	3.125161	0.001777	0.005267	304.6525	353.628	238.6359	439.729	462.0479	432.1412
21343	LOC11962	14.53999	0.569062	0.689583	0.825227	0.409243	0.509745	8.673382	12.59047	13.2189	22.32127	21.39111	9.044815
18781	SLC12A8	29.70409	0.569061	0.466039	1.22106	0.222063	0.314337	20.59928	22.44388	28.52499	42.41041	32.08666	32.15934
21419	TRNAM-CA	12.74575	0.568879	0.698199	0.814781	0.415198	0.515338	11.38381	7.116354	12.52316	13.39276	14.97377	17.08465
16997	LOC11962	51.73039	0.568823	0.381116	1.492518	0.135563	0.212035	49.87195	45.98259	28.52499	60.26743	68.45154	57.28383
4455	LOC10323	13468.73	0.568629	0.112865	5.038118	4.70E-07	2.81E-06	10024.26	12677.51	9841.12	16544.53	15925.68	15799.28
4693	COL4A5	1639.994	0.568232	0.116251	4.887962	1.02E-06	5.77E-06	1415.93	1278.207	1274.58	1841.505	1843.913	2185.83
19850	LOC10321	31.32845	0.568099	0.543236	1.045767	0.295668	0.395012	40.65648	13.13788	21.56767	31.24978	49.19955	32.15934
18631	CYP46A1	35.8369	0.568029	0.460274	1.234111	0.217161	0.309867	38.48813	20.25424	27.13352	44.64254	51.33866	33.16432
5291	MAPK8IP3	2897.912	0.568021	0.125599	4.522503	6.11E-06	3.07E-05	2516.907	1995.863	2495.588	3484.35	3105.989	3788.773
8050	CERS1	278.4286	0.567966	0.167082	3.399329	0.000676	0.002231	211.9558	228.8181	233.07	319.1942	335.8404	341.693
20700	LOC10321	20.72637	0.567891	0.615837	0.922145	0.356453	0.457824	24.39389	15.87494	9.044508	29.01765	29.94755	16.07967
21197	TNFRSF13C	15.30746	0.567755	0.667641	0.850389	0.395109	0.49554	11.38381	15.32753	10.43597	8.928508	25.66933	20.09959
4546	LOC10322	4610.545	0.567732	0.113996	4.98026	6.35E-07	3.71E-06	3914.948	3349.613	3886.355	5390.587	4971.293	6150.474
5084	DYNLT2B	805.079	0.567261	0.122211	4.641643	3.46E-06	1.81E-05	676.5238	607.6271	663.032	899.5472	1013.938	969.8052
4816	TGFB1I1	959.1376	0.567256	0.118119	4.80241	1.57E-06	8.65E-06	726.9378	820.5703	774.349	1124.992	1090.946	1217.03
17991	LOC10323	45.21761	0.56717	0.421734	1.344851	0.178673	0.264022	35.23561	38.86624	34.09084	44.64254	81.28621	37.18424
5574	PRKAG2	3193.45	0.567139	0.129383	4.38342	1.17E-05	5.57E-05	2316.877	3096.161	2305.654	3901.758	3893.181	3647.07
24055	LOC11962	2.677248	0.567061	1.427929	0.397121	0.691278	0.763984	2.168345	2.189647	2.087194	4.464254	2.139111	3.014938
12133	RPUSD1	231.5586	0.566925	0.238311	2.378928	0.017363	0.038043	226.5921	128.6418	205.9365	267.8552	258.8324	301.4938
21301	CUNHXorf	18.1005	0.566836	0.679857	0.833759	0.404417	0.504738	14.09425	14.78012	13.91463	24.5534	34.22577	7.034856
14685	LOC11962	71.80465	0.566785	0.299563	1.892038	0.058486	0.10588	59.08741	55.28859	59.13717	93.74933	79.14709	84.41828
6473	CFAP69	549.9362	0.56678	0.143463	3.95071	7.79E-05	0.00032	464.0259	440.6665	422.3089	627.2277	744.4105	600.9777
20719	AMY2A	17.52274	0.566771	0.618837	0.915863	0.359739	0.461588	9.757555	17.51718	14.61036	26.78552	21.39111	15.07469
13499	HMGN5	122.3364	0.566698	0.268919	2.10732	0.03509	0.069106	99.20181	122.0728	74.44326	151.7846	134.764	151.7519
7754	SPSB3	502.5145	0.56632	0.162884	3.476826	0.000507	0.00174	430.4166	336.6583	450.8339	598.21	549.7514	649.2167
3919	MLEC	17113.69	0.566268	0.104275	5.430532	5.62E-08	3.81E-07	13397.12	15301.26	12698.49	19794.5	19311.89	22178.89
17919	LOC11962	39.14321	0.56619	0.417004	1.357756	0.174541	0.258953	36.86187	31.20247	25.74206	55.80317	47.06043	38.18922
4526	HLTF	17039.03	0.565761	0.113185	4.998558	5.78E-07	3.39E-06	12611.1	16110.88	12498.81	20323.52	19962.18	20727.7
4736	LOC10321	811.9642	0.565507	0.116402	4.858232	1.18E-06	6.65E-06	666.2241	664.5579	634.507	919.6363	998.9647	987.8948
4865	CCDC88C	1535.987	0.565452	0.118704	4.763561	1.90E-06	1.04E-05	1170.907	1395.9	1150.044	1890.612	1724.123	1884.337
23862	LOC11962	3.820958	0.565446	1.320188	0.428307	0.668428	0.744705	2.710432	3.284471	3.478657	0	6.417332	7.034856
23011	LOC10323	6.530203	0.565193	1.008592	0.560378	0.575222	0.664563	4.336691	4.379295	7.653045	4.464254	4.278221	14.06971

4281	BTD	1695.987	0.565088	0.109366	5.166931	2.38E-07	1.48E-06	1415.93	1270.543	1422.075	1991.057	1899.53	2176.786
13341	LOC11962	115.9679	0.564971	0.264081	2.139383	0.032405	0.064574	107.8752	81.56436	91.14081	113.8385	164.7115	136.6772
3690	ATP2C1	4057.505	0.564952	0.099996	5.649752	1.61E-08	1.16E-07	3192.347	3575.147	3054.261	4685.235	4748.826	5089.216
17385	GCKR	52.46983	0.564818	0.394601	1.431365	0.152326	0.232935	37.94605	38.31883	49.39693	87.05295	59.8951	42.20914
3525	SLC30A7	4463.603	0.56451	0.096777	5.83308	5.44E-09	4.10E-08	3595.659	3857.611	3352.729	5044.607	5390.559	5540.452
23753	TRAPPC3L	4.814822	0.564427	1.271111	0.444042	0.657012	0.73526	3.794605	1.642235	6.261582	11.16063	0	6.029877
23745	LOC11962	4.440986	0.564385	1.26748	0.445281	0.656116	0.734591	5.96295	1.642235	2.782925	6.696381	8.556443	1.004979
7188	NUCB2	4141.332	0.564307	0.153779	3.669592	0.000243	0.000898	2844.869	4324.553	2853.194	4962.018	5168.091	4695.264
18448	LOC10322	35.57335	0.563725	0.448452	1.257045	0.208737	0.300807	27.6464	37.77142	20.17621	46.87467	42.78221	38.18922
6477	AMER1	569.8872	0.56367	0.142756	3.948479	7.86E-05	0.000323	419.0328	501.9766	461.2699	680.7987	624.6203	731.6251
6587	PDE3A	576.8958	0.563619	0.14431	3.905609	9.40E-05	0.000379	448.8475	503.0715	448.7467	649.549	635.3159	775.8442
13008	LOC10321	99.6795	0.563521	0.255852	2.202528	0.027628	0.056465	78.06044	79.37471	84.1835	116.0706	119.7902	120.5975
16236	CLDN23	54.96901	0.563137	0.34771	1.619558	0.105327	0.172464	49.32986	38.86624	44.52681	75.89232	59.8951	61.30375
22579	TCP11	8.003414	0.563118	0.897372	0.627519	0.530319	0.624409	7.589209	3.284471	8.348776	8.928508	12.83466	7.034856
8691	CDKL3	254.608	0.56299	0.175629	3.205559	0.001348	0.004122	210.3295	208.5639	196.892	285.7123	333.7013	292.449
6018	ANKRD54	678.4376	0.562935	0.135398	4.157643	3.22E-05	0.000142	534.4972	565.4764	546.8449	819.1906	710.1847	894.4317
5596	MBNL2	2414.11	0.562831	0.128647	4.375011	1.21E-05	5.77E-05	1755.818	2327.595	1763.679	2935.247	2780.844	2921.475
8640	MXD3	416.2995	0.562749	0.174894	3.217655	0.001292	0.003977	344.7669	279.18	386.8266	506.6928	440.6568	539.674
17895	LOC10324	39.84622	0.562686	0.413084	1.362159	0.173148	0.25727	35.7777	27.918	32.00364	60.26743	44.92132	38.18922
15002	LOC11962	90.18538	0.562562	0.306952	1.832736	0.066842	0.11845	77.51835	65.14201	77.92191	75.89232	106.9555	137.6822
23022	LOC10322	5.930086	0.562454	1.006582	0.558776	0.576315	0.665508	2.710432	6.02153	5.565851	6.696381	8.556443	6.029877
21202	PHYHD1	17.45345	0.562446	0.661917	0.849722	0.39548	0.495888	10.29964	18.06459	13.2189	17.85702	34.22577	11.05477
15439	LOC10323	93.2159	0.562355	0.320596	1.754093	0.079415	0.136747	60.71367	95.79707	68.87741	145.0883	81.28621	107.5328
4489	PLEC	73739.94	0.562322	0.112101	5.016198	5.27E-07	3.12E-06	60149.36	54287.92	64214.61	87307.41	77615.49	98864.86
6891	KLHDC8B	534.813	0.562254	0.148794	3.778735	0.000158	0.000608	454.2684	386.4727	458.487	584.8173	620.3421	704.4906
5495	CUTA	3741.667	0.562158	0.127428	4.411584	1.03E-05	4.96E-05	3250.35	2530.685	3288.026	4555.771	4002.276	4822.896
19218	LOC10322	564.6315	0.562156	0.48831	1.15123	0.249638	0.34538	604.9684	205.8268	557.9766	560.2639	624.6203	834.133
25223	LOC11962	1.166551	0.561936	2.667267	0.210679	0.833138	0.878057	1.626259	1.094824	0	0	4.278221	0
21252	LOC10323	13.99504	0.56192	0.667601	0.841701	0.399955	0.500321	10.84173	10.94824	12.52316	17.85702	10.69555	21.10457
20922	LOC10323	23.50118	0.561815	0.632863	0.887735	0.374683	0.476099	17.34676	16.96977	21.56767	29.01765	47.06043	9.044815
24747	LOC10321	1.769595	0.5617	1.935705	0.290178	0.77168	0.828994	2.168345	0.547412	1.391463	2.232127	4.278221	0
20304	LOC11962	32.26647	0.561426	0.574081	0.977956	0.328096	0.429592	16.80468	48.71965	12.52316	35.71403	40.6431	39.1942
20087	LOC11962	24.93866	0.561424	0.55316	1.014938	0.310135	0.410462	18.43094	13.13788	28.52499	26.78552	40.6431	22.10955
4705	LOC10324	6042.946	0.561248	0.114982	4.881182	1.05E-06	5.96E-06	4453.782	5681.04	4513.209	7020.039	7046.231	7543.376
4053	RETREG2	3959.553	0.560799	0.10521	5.330287	9.81E-08	6.43E-07	3337.626	3119.7	3148.184	4493.272	4372.342	5286.192
4492	PPP2CB	5939.426	0.560732	0.11181	5.015026	5.30E-07	3.14E-06	5325.456	4241.347	4836.029	6908.433	6663.33	7661.963
19014	HES4	29.99817	0.560723	0.474349	1.182089	0.23717	0.331607	19.51511	24.08612	28.52499	37.94616	42.78221	27.13445
14402	LOC10323	83.5914	0.560716	0.288589	1.942955	0.052022	0.096028	65.59245	72.80577	63.31155	93.74933	117.6511	88.43819
23585	LOC11961	4.440722	0.560588	1.190751	0.470786	0.637794	0.718921	5.420864	2.189647	3.478657	2.232127	4.278221	9.044815
9360	KIAA1586	325.5094	0.560437	0.185824	3.015959	0.002562	0.007276	250.986	303.2661	231.6785	412.9435	423.5439	330.6382
22669	LOC10324	10.11448	0.560386	0.91577	0.611929	0.540585	0.633969	8.131296	6.02153	9.740239	13.39276	21.39111	2.009959
4856	CYFIP2	4764.409	0.56025	0.117323	4.775281	1.79E-06	9.82E-06	3727.928	4157.045	3674.157	5392.819	5091.083	6543.421
5921	TUT7	4905.137	0.560239	0.133333	4.201809	2.65E-05	0.000119	3477.484	4873.06	3543.36	5787.905	5739.234	6009.777
7186	MAGEE1	453.5098	0.559787	0.152501	3.670702	0.000242	0.000895	411.9856	335.016	353.4315	558.0317	504.8301	557.7636
24321	LOC11962	2.353574	0.559477	1.560862	0.358441	0.720013	0.787038	2.168345	2.737059	0.695731	2.232127	4.278221	2.009959
15552	PDIA2	90.24066	0.559391	0.322377	1.73521	0.082704	0.141376	85.10756	49.81448	84.1835	127.2312	85.56443	109.5428
12541	LOC10323	172.4941	0.559299	0.243725	2.294791	0.021745	0.046096	156.663	126.4521	132.189	243.3018	224.6066	151.7519
6270	CD58	1013.245	0.559046	0.1383	4.042271	5.29E-05	0.000224	736.6954	907.6088	809.1356	1261.152	1317.692	1047.189
7367	NCOA1	1515.128	0.558582	0.154723	3.610215	0.000306	0.001104	1052.732	1556.839	1065.86	1819.183	1803.27	1792.883
24392	LOC11962	3.234898	0.55841	1.604333	0.348064	0.727792	0.793233	4.878777	2.737059	0	2.232127	8.556443	1.004979
5380	ATF6B	2631.834	0.558113	0.124733	4.474457	7.66E-06	3.79E-05	1902.723	2454.595	2032.927	3127.21	2870.687	3402.86
7159	NLRX1	1603.348	0.558046	0.151741	3.677629	0.000235	0.000874	1444.118	1063.074	1388.68	1886.147	1632.141	2205.93
5969	PLEK2	583.416	0.558013	0.133569	4.177702	2.94E-05	0.000131	499.8036	434.645	482.1418	714.2806	663.1243	706.5006
7283	LOC10322	587.6056	0.557966	0.153335	3.638868	0.000274	0.001	499.8036	416.033	511.3626	607.1385	780.7754	710.5205
23325	LOC11962	6.257904	0.55793	1.093743	0.51011	0.609974	0.695227	3.794605	6.02153	4.87012	11.16063	10.69555	1.004979
23238	LOC10323	5.045009	0.55781	1.066002	0.523273	0.600784	0.687316	3.252518	4.926706	4.174388	4.464254	6.417332	7.034856
19966	LOC10324	23.9379	0.557804	0.538712	1.035438	0.300464	0.400052	18.97302	20.80165	18.08902	17.85702	42.78221	25.12449
19967	LOC11961	23.9379	0.557804	0.538712	1.035438	0.300464	0.400052	18.97302	20.80165	18.08902	17.85702	42.78221	25.12449
21079	ADRA2C	16.88002	0.557775	0.642279	0.868431	0.385158	0.485765	14.63633	9.306001	17.39328	11.16063	25.66933	23.11453
24328	TRNAQ-CU	2.965112	0.557256	1.558584	0.35754	0.720688	0.787516	4.336691	1.094824	2.087194	2.232127	0	8.039836
23336	LOC10323	5.375666	0.557101	1.095257	0.508649	0.610998	0.696066	6.505036	1.642235	4.87012	8.928508	4.278221	6.029877
5787	TMEM192	736.6261	0.557064	0.130115	4.281305	1.86E-05	8.54E-05	544.2547	660.7261	583.0229	868.2974	894.1483	869.3072
24766	TRNAA-AG	1.772257	0.556939	1.935472	0.287754	0.773535	0.83035	0.542086	2.189647	1.391463	2.232127	4.278221	0
8568	EOMES	624.2502	0.556845	0.172002	3.237428	0.001206	0.003742	433.6691	637.7348	443.8766	712.0485	776.4972	741.6748
14347	LOC10323	98.16397	0.556566	0.284983	1.952982	0.050822	0.094173	76.97626	78.8273	81.40057	154.0168	96.25998	101.5029
9170	ABCD1	1012.258	0.556353	0.181679	3.062283	0.002197	0.006369	952.4458	599.4159	910.0166	1171.867	1043.886	1395.916
21725	TRNAL-CA	17.22376	0.556226	0.718656	0.773981	0.438942	0.537136	25.47806	7.663765	9.044508	22.32127	10.69555	28.13943
21490	SOX30	15.85895	0.5558	0.68979	0.805753	0.420385	0.520053	11.9259	6.568942	20.17621	24.5534	12.83466	19.09461
8302	LRGUK	358.7176	0.555641	0.167746	3.312392	0.000925	0.002962	277.0061	313.667	277.5968	433.0326	479.1608	371.8424
20439	NKD2	19.31419	0.55523	0.580769	0.956025	0.339059	0.441014	14.63633	16.96977	14.61036	26.78552	27.80844	15.07469
4450	ATP6AP1	9257.839	0.555179	0.110093	5.042834	4.59E-07	2.74E-06	7965.959	6893.557	7641.913	10727.6	9927.613	12390.39
3571	OGDH	15861.85	0.55514	0.096032	5.780752	7.44E-09	5.54E-08	12685.36	13584.57	12277.57	18408.35	17592.05	20623.18
3177	HMGCS1	18732.63	0.555057	0.089698	6.188054	6.09E-10	5.10E-09	15224.5	15957.6	14341.11	21709.67	21391.11	23771.78
23547	LOC10322	5.058217	0.55472	1.163502	0.476768	0.633528	0.715264	5.420864	1.094824	5.565851	6.696381	8.556443	3.014938

12971	LOC11962(	119.9171	0.554682	0.251101	2.209003	0.027174	0.055696	100.8281	94.15483	98.09812	145.0883	115.512	165.8216
6816	DIO1	406.5921	0.554667	0.145567	3.810381	0.000139	0.000541	323.0835	324.0678	339.5169	484.3716	511.2474	457.2657
3702	DHCR24	13034.45	0.554406	0.098334	5.638	1.72E-08	1.24E-07	10795.65	10429.84	10463.1	15055.7	14190.86	17271.58
3390	FKBP9	7035.376	0.554406	0.093145	5.952042	2.65E-09	2.08E-08	5638.782	6080.65	5384.265	8182.978	8051.613	8873.969
5110	NAV2	1206.447	0.554049	0.119839	4.623273	3.78E-06	1.97E-05	932.3886	1084.97	916.9739	1341.508	1467.43	1495.409
10078	WDR81	894.6799	0.554023	0.194914	2.8424	0.004478	0.011811	836.9814	529.8946	811.2228	1113.831	829.9749	1246.175
3317	VPS26C	2413.188	0.553946	0.091933	6.025524	1.69E-09	1.35E-08	1989.457	1937.838	1942.482	2834.801	2778.705	2995.844
17448	P2RY2	42.57228	0.553686	0.390296	1.418628	0.156007	0.237704	37.40396	30.65506	34.78657	58.0353	51.33866	43.21412
3189	ACBD5	3299.139	0.553335	0.089588	6.176415	6.56E-10	5.47E-09	2670.317	2765.524	2588.816	3778.991	3938.103	4053.082
4514	COPB2	15554.96	0.553071	0.110532	5.003711	5.62E-07	3.31E-06	11801.76	14575.39	11452.43	18372.64	17803.82	19323.75
6935	ZNF774	402.3313	0.552805	0.146911	3.762858	0.000168	0.000644	306.2788	347.0591	324.9066	484.3716	477.0217	474.3503
21615	PLAC8	13.62599	0.552598	0.700856	0.788462	0.430427	0.529396	15.7205	7.663765	9.740239	11.16063	21.39111	16.07967
3825	ARMC10	4373.212	0.552367	0.100078	5.519367	3.40E-08	2.36E-07	3446.043	3915.637	3276.895	5078.089	5234.404	5288.202
5788	COQ8A	1214.304	0.552324	0.129017	4.281018	1.86E-05	8.55E-05	1028.88	866.0055	1063.773	1370.526	1364.753	1591.887
4076	STX1A	2530.738	0.552129	0.103871	5.315513	1.06E-07	6.94E-07	2099.501	2004.075	2059.365	2941.943	2770.148	3309.397
11872	LOC11962:	328.0117	0.551975	0.226913	2.432537	0.014993	0.033577	193.5248	361.8392	242.8102	379.4616	391.4573	398.9768
13698	LOC10324:	117.6553	0.551821	0.266791	2.068368	0.038605	0.074925	94.32303	114.4091	77.22618	158.481	119.7902	141.7021
21309	LOC11962!	14.26569	0.551588	0.663266	0.831625	0.405621	0.506051	15.7205	9.306001	9.740239	15.62489	17.11289	18.08963
4617	FAM214B	2015.754	0.551497	0.111709	4.936908	7.94E-07	4.57E-06	1697.272	1565.598	1648.883	2256.68	2241.788	2684.3
20696	LOC10321!	20.592	0.551162	0.597438	0.922543	0.356245	0.457614	20.0572	13.13788	16.00182	33.4819	27.80844	13.06473
11292	TRAPPC2B	156.9606	0.551106	0.215273	2.560032	0.010466	0.024641	134.9795	117.1461	130.7975	189.7308	171.1289	197.981
26588	LOC11962(	0.091235	0.551076	4.080473	0.135052	0.892571	NA	0	0.547412	0	0	0	0
26629	LOC11962(	0.091235	0.551076	4.080473	0.135052	0.892571	NA	0	0.547412	0	0	0	0
26637	INSL5	0.090348	0.551076	4.080473	0.135052	0.892571	NA	0.542086	0	0	0	0	0
26647	ASB17	0.091235	0.551076	4.080473	0.135052	0.892571	NA	0	0.547412	0	0	0	0
26650	LOC11962(	0.115955	0.551076	4.080473	0.135052	0.892571	NA	0	0	0.695731	0	0	0
26655	LOC10322<	0.115955	0.551076	4.080473	0.135052	0.892571	NA	0	0	0.695731	0	0	0
26664	LOC10324<	0.091235	0.551076	4.080473	0.135052	0.892571	NA	0	0.547412	0	0	0	0
26674	NMUR2	0.091235	0.551076	4.080473	0.135052	0.892571	NA	0	0.547412	0	0	0	0
26688	LOC11961{	0.091235	0.551076	4.080473	0.135052	0.892571	NA	0	0.547412	0	0	0	0
26716	LOC11961{	0.115955	0.551076	4.080473	0.135052	0.892571	NA	0	0	0.695731	0	0	0
26734	LOC11961!	0.090348	0.551076	4.080473	0.135052	0.892571	NA	0.542086	0	0	0	0	0
26754	LOC11962(	0.115955	0.551076	4.080473	0.135052	0.892571	NA	0	0	0.695731	0	0	0
26773	LOC10323(	0.091235	0.551076	4.080473	0.135052	0.892571	NA	0	0.547412	0	0	0	0
26774	LOC11962:	0.115955	0.551076	4.080473	0.135052	0.892571	NA	0	0	0.695731	0	0	0
26779	LOC11962:	0.091235	0.551076	4.080473	0.135052	0.892571	NA	0	0.547412	0	0	0	0
26800	LOC11962:	0.115955	0.551076	4.080473	0.135052	0.892571	NA	0	0	0.695731	0	0	0
26812	LOC11961{	0.091235	0.551076	4.080473	0.135052	0.892571	NA	0	0.547412	0	0	0	0
26852	TRNAG-GC	0.115955	0.551076	4.080473	0.135052	0.892571	NA	0	0	0.695731	0	0	0
26856	LOC11962:	0.091235	0.551076	4.080473	0.135052	0.892571	NA	0	0.547412	0	0	0	0
26874	LOC11962<	0.091235	0.551076	4.080473	0.135052	0.892571	NA	0	0.547412	0	0	0	0
26939	IVL	0.115955	0.551076	4.080473	0.135052	0.892571	NA	0	0	0.695731	0	0	0
26946	LOC11962<	0.115955	0.551076	4.080473	0.135052	0.892571	NA	0	0	0.695731	0	0	0
26951	LOC11962<	0.090348	0.551076	4.080473	0.135052	0.892571	NA	0.542086	0	0	0	0	0
26955	LOC11962<	0.115955	0.551076	4.080473	0.135052	0.892571	NA	0	0	0.695731	0	0	0
26978	LOC11962<	0.091235	0.551076	4.080473	0.135052	0.892571	NA	0	0.547412	0	0	0	0
26983	LOC10321!	0.091235	0.551076	4.080473	0.135052	0.892571	NA	0	0.547412	0	0	0	0
26985	LOC11962<	0.091235	0.551076	4.080473	0.135052	0.892571	NA	0	0.547412	0	0	0	0
27021	TEKT4	0.091235	0.551076	4.080473	0.135052	0.892571	NA	0	0.547412	0	0	0	0
27030	SCGB1A1	0.090348	0.551076	4.080473	0.135052	0.892571	NA	0.542086	0	0	0	0	0
27082	LOC10323!	0.091235	0.551076	4.080473	0.135052	0.892571	NA	0	0.547412	0	0	0	0
27097	LOC11962<	0.091235	0.551076	4.080473	0.135052	0.892571	NA	0	0.547412	0	0	0	0
27100	LOC11962<	0.091235	0.551076	4.080473	0.135052	0.892571	NA	0	0.547412	0	0	0	0
27139	LOC11962<	0.090348	0.551076	4.080473	0.135052	0.892571	NA	0.542086	0	0	0	0	0
27144	LOC11962<	0.091235	0.551076	4.080473	0.135052	0.892571	NA	0	0.547412	0	0	0	0
27223	LOC11962!	0.115955	0.551076	4.080473	0.135052	0.892571	NA	0	0	0.695731	0	0	0
27230	LOC11962!	0.090348	0.551076	4.080473	0.135052	0.892571	NA	0.542086	0	0	0	0	0
27239	LOC10321{	0.115955	0.551076	4.080473	0.135052	0.892571	NA	0	0	0.695731	0	0	0
27242	LOC11962!	0.090348	0.551076	4.080473	0.135052	0.892571	NA	0.542086	0	0	0	0	0
27244	LOC11962!	0.115955	0.551076	4.080473	0.135052	0.892571	NA	0	0	0.695731	0	0	0
27246	LOC11962!	0.091235	0.551076	4.080473	0.135052	0.892571	NA	0	0.547412	0	0	0	0
27263	LOC11962!	0.115955	0.551076	4.080473	0.135052	0.892571	NA	0	0	0.695731	0	0	0
27264	LOC11962!	0.091235	0.551076	4.080473	0.135052	0.892571	NA	0	0.547412	0	0	0	0
27276	LOC11962!	0.115955	0.551076	4.080473	0.135052	0.892571	NA	0	0	0.695731	0	0	0
27279	LOC11962!	0.115955	0.551076	4.080473	0.135052	0.892571	NA	0	0	0.695731	0	0	0
27323	LOC11962!	0.091235	0.551076	4.080473	0.135052	0.892571	NA	0	0.547412	0	0	0	0
27324	LOC11962!	0.091235	0.551076	4.080473	0.135052	0.892571	NA	0	0.547412	0	0	0	0
27327	LOC11962!	0.090348	0.551076	4.080473	0.135052	0.892571	NA	0.542086	0	0	0	0	0
27404	LOC11962!	0.115955	0.551076	4.080473	0.135052	0.892571	NA	0	0	0.695731	0	0	0
27433	LOC11962(	0.090348	0.551076	4.080473	0.135052	0.892571	NA	0.542086	0	0	0	0	0
27503	LOC11962(	0.091235	0.551076	4.080473	0.135052	0.892571	NA	0	0.547412	0	0	0	0
27532	LINC02873	0.090348	0.551076	4.080473	0.135052	0.892571	NA	0.542086	0	0	0	0	0
27574	LOC11962(	0.115955	0.551076	4.080473	0.135052	0.892571	NA	0	0	0.695731	0	0	0
27581	LOC10324?	0.115955	0.551076	4.080473	0.135052	0.892571	NA	0	0	0.695731	0	0	0

27613	LOC11962(	0.115955	0.551076	4.080473	0.135052	0.892571	NA	0	0	0.695731	0	0	0
27614	LOC11962(	0.115955	0.551076	4.080473	0.135052	0.892571	NA	0	0	0.695731	0	0	0
27632	TRNAQ-UL	0.091235	0.551076	4.080473	0.135052	0.892571	NA	0	0.547412	0	0	0	0
27638	TRNAY-GU	0.091235	0.551076	4.080473	0.135052	0.892571	NA	0	0.547412	0	0	0	0
27663	TRNAA-AG	0.091235	0.551076	4.080473	0.135052	0.892571	NA	0	0.547412	0	0	0	0
27674	MHC-DPA1	0.090348	0.551076	4.080473	0.135052	0.892571	NA	0.542086	0	0	0	0	0
27675	SMIM40	0.091235	0.551076	4.080473	0.135052	0.892571	NA	0	0.547412	0	0	0	0
27679	LOC11962(	0.115955	0.551076	4.080473	0.135052	0.892571	NA	0	0	0.695731	0	0	0
27691	LOC11962(	0.115955	0.551076	4.080473	0.135052	0.892571	NA	0	0	0.695731	0	0	0
27694	LOC11962(	0.115955	0.551076	4.080473	0.135052	0.892571	NA	0	0	0.695731	0	0	0
27695	DEFB114	0.091235	0.551076	4.080473	0.135052	0.892571	NA	0	0.547412	0	0	0	0
27716	LOC11962;	0.115955	0.551076	4.080473	0.135052	0.892571	NA	0	0	0.695731	0	0	0
27726	LOC11962;	0.115955	0.551076	4.080473	0.135052	0.892571	NA	0	0	0.695731	0	0	0
27749	LOC11962;	0.115955	0.551076	4.080473	0.135052	0.892571	NA	0	0	0.695731	0	0	0
27759	LOC11962;	0.091235	0.551076	4.080473	0.135052	0.892571	NA	0	0.547412	0	0	0	0
27770	LOC11962;	0.091235	0.551076	4.080473	0.135052	0.892571	NA	0	0.547412	0	0	0	0
27868	LOC11962;	0.091235	0.551076	4.080473	0.135052	0.892571	NA	0	0.547412	0	0	0	0
27893	LOC11962;	0.115955	0.551076	4.080473	0.135052	0.892571	NA	0	0	0.695731	0	0	0
27901	LOC11962;	0.115955	0.551076	4.080473	0.135052	0.892571	NA	0	0	0.695731	0	0	0
27906	LOC10321!	0.115955	0.551076	4.080473	0.135052	0.892571	NA	0	0	0.695731	0	0	0
27908	LOC11962;	0.091235	0.551076	4.080473	0.135052	0.892571	NA	0	0.547412	0	0	0	0
27911	CUNH10or	0.091235	0.551076	4.080473	0.135052	0.892571	NA	0	0.547412	0	0	0	0
27932	MBL2	0.115955	0.551076	4.080473	0.135052	0.892571	NA	0	0	0.695731	0	0	0
27935	LOC11962{	0.091235	0.551076	4.080473	0.135052	0.892571	NA	0	0.547412	0	0	0	0
27951	LOC11962;	0.115955	0.551076	4.080473	0.135052	0.892571	NA	0	0	0.695731	0	0	0
27954	LOC11962{	0.115955	0.551076	4.080473	0.135052	0.892571	NA	0	0	0.695731	0	0	0
28007	LOC11961{	0.091235	0.551076	4.080473	0.135052	0.892571	NA	0	0.547412	0	0	0	0
28017	LOC11961{	0.091235	0.551076	4.080473	0.135052	0.892571	NA	0	0.547412	0	0	0	0
28029	LOC11961{	0.091235	0.551076	4.080473	0.135052	0.892571	NA	0	0.547412	0	0	0	0
28051	LOC11961{	0.115955	0.551076	4.080473	0.135052	0.892571	NA	0	0	0.695731	0	0	0
28067	LOC11961{	0.091235	0.551076	4.080473	0.135052	0.892571	NA	0	0.547412	0	0	0	0
28188	LOC11961!	0.115955	0.551076	4.080473	0.135052	0.892571	NA	0	0	0.695731	0	0	0
28189	LOC11961!	0.115955	0.551076	4.080473	0.135052	0.892571	NA	0	0	0.695731	0	0	0
28235	LOC11961!	0.090348	0.551076	4.080473	0.135052	0.892571	NA	0.542086	0	0	0	0	0
28238	LOC11961!	0.091235	0.551076	4.080473	0.135052	0.892571	NA	0	0.547412	0	0	0	0
28249	LOC11961!	0.090348	0.551076	4.080473	0.135052	0.892571	NA	0.542086	0	0	0	0	0
28257	LOC10321{	0.091235	0.551076	4.080473	0.135052	0.892571	NA	0	0.547412	0	0	0	0
28264	LOC11961!	0.091235	0.551076	4.080473	0.135052	0.892571	NA	0	0.547412	0	0	0	0
28266	LOC11961!	0.090348	0.551076	4.080473	0.135052	0.892571	NA	0.542086	0	0	0	0	0
28270	LOC10321!	0.091235	0.551076	4.080473	0.135052	0.892571	NA	0	0.547412	0	0	0	0
28288	LOC10322!	0.091235	0.551076	4.080473	0.135052	0.892571	NA	0	0.547412	0	0	0	0
28305	TMEM52	0.115955	0.551076	4.080473	0.135052	0.892571	NA	0	0	0.695731	0	0	0
28316	LOC11961!	0.091235	0.551076	4.080473	0.135052	0.892571	NA	0	0.547412	0	0	0	0
28320	LOC11961!	0.115955	0.551076	4.080473	0.135052	0.892571	NA	0	0	0.695731	0	0	0
28325	LOC11961!	0.115955	0.551076	4.080473	0.135052	0.892571	NA	0	0	0.695731	0	0	0
28326	LOC11961!	0.115955	0.551076	4.080473	0.135052	0.892571	NA	0	0	0.695731	0	0	0
28327	LOC11961!	0.115955	0.551076	4.080473	0.135052	0.892571	NA	0	0	0.695731	0	0	0
28363	LOC11961!	0.090348	0.551076	4.080473	0.135052	0.892571	NA	0.542086	0	0	0	0	0
28381	LOC11961!	0.091235	0.551076	4.080473	0.135052	0.892571	NA	0	0.547412	0	0	0	0
28423	LOC10323(	0.091235	0.551076	4.080473	0.135052	0.892571	NA	0	0.547412	0	0	0	0
28430	LOC10323(	0.115955	0.551076	4.080473	0.135052	0.892571	NA	0	0	0.695731	0	0	0
28433	LOC11961!	0.115955	0.551076	4.080473	0.135052	0.892571	NA	0	0	0.695731	0	0	0
28440	LOC11962(	0.091235	0.551076	4.080473	0.135052	0.892571	NA	0	0.547412	0	0	0	0
28454	LOC11962(	0.091235	0.551076	4.080473	0.135052	0.892571	NA	0	0.547412	0	0	0	0
28463	LOC10323:	0.091235	0.551076	4.080473	0.135052	0.892571	NA	0	0.547412	0	0	0	0
28474	LOC11962(	0.091235	0.551076	4.080473	0.135052	0.892571	NA	0	0.547412	0	0	0	0
28476	LOC10321/	0.115955	0.551076	4.080473	0.135052	0.892571	NA	0	0	0.695731	0	0	0
28488	LOC11962(	0.091235	0.551076	4.080473	0.135052	0.892571	NA	0	0.547412	0	0	0	0
28495	LOC10321/	0.091235	0.551076	4.080473	0.135052	0.892571	NA	0	0.547412	0	0	0	0
28533	LOC11962(	0.091235	0.551076	4.080473	0.135052	0.892571	NA	0	0.547412	0	0	0	0
28544	LOC10324;	0.115955	0.551076	4.080473	0.135052	0.892571	NA	0	0	0.695731	0	0	0
28561	LOC10323:	0.091235	0.551076	4.080473	0.135052	0.892571	NA	0	0.547412	0	0	0	0
28563	LOC11962(	0.115955	0.551076	4.080473	0.135052	0.892571	NA	0	0	0.695731	0	0	0
28577	LOC10323:	0.091235	0.551076	4.080473	0.135052	0.892571	NA	0	0.547412	0	0	0	0
28581	LOC10323:	0.115955	0.551076	4.080473	0.135052	0.892571	NA	0	0	0.695731	0	0	0
28589	LOC11962(	0.115955	0.551076	4.080473	0.135052	0.892571	NA	0	0	0.695731	0	0	0
28658	LOC11962(	0.090348	0.551076	4.080473	0.135052	0.892571	NA	0.542086	0	0	0	0	0
28667	LOC10322:	0.090348	0.551076	4.080473	0.135052	0.892571	NA	0.542086	0	0	0	0	0
28674	LOC11962(	0.091235	0.551076	4.080473	0.135052	0.892571	NA	0	0.547412	0	0	0	0
28678	LOC11962(	0.115955	0.551076	4.080473	0.135052	0.892571	NA	0	0	0.695731	0	0	0
28701	LOC10323;	0.091235	0.551076	4.080473	0.135052	0.892571	NA	0	0.547412	0	0	0	0
28705	LOC11962(	0.115955	0.551076	4.080473	0.135052	0.892571	NA	0	0	0.695731	0	0	0
28712	LOC11962(	0.091235	0.551076	4.080473	0.135052	0.892571	NA	0	0.547412	0	0	0	0
28725	LOC11962:	0.115955	0.551076	4.080473	0.135052	0.892571	NA	0	0	0.695731	0	0	0

28730	BRS3	0.115955	0.551076	4.080473	0.135052	0.892571	NA	0	0	0.695731	0	0	0
28734	LOC11962:	0.090348	0.551076	4.080473	0.135052	0.892571	NA	0.542086	0	0	0	0	0
28738	LOC11962(	0.115955	0.551076	4.080473	0.135052	0.892571	NA	0	0	0.695731	0	0	0
28739	LOC11962(	0.091235	0.551076	4.080473	0.135052	0.892571	NA	0	0.547412	0	0	0	0
28744	ARHGAP4	0.091235	0.551076	4.080473	0.135052	0.892571	NA	0	0.547412	0	0	0	0
28764	LOC11962:	0.091235	0.551076	4.080473	0.135052	0.892571	NA	0	0.547412	0	0	0	0
28794	TRNAI-UAU	0.115955	0.551076	4.080473	0.135052	0.892571	NA	0	0	0.695731	0	0	0
28796	LOC11962:	0.090348	0.551076	4.080473	0.135052	0.892571	NA	0.542086	0	0	0	0	0
28810	LOC10322(	0.091235	0.551076	4.080473	0.135052	0.892571	NA	0	0.547412	0	0	0	0
28814	LOC11962:	0.091235	0.551076	4.080473	0.135052	0.892571	NA	0	0.547412	0	0	0	0
28820	LOC11962:	0.091235	0.551076	4.080473	0.135052	0.892571	NA	0	0.547412	0	0	0	0
28842	TAS2R20	0.090348	0.551076	4.080473	0.135052	0.892571	NA	0.542086	0	0	0	0	0
28851	LOC11962:	0.115955	0.551076	4.080473	0.135052	0.892571	NA	0	0	0.695731	0	0	0
28866	LOC11962:	0.091235	0.551076	4.080473	0.135052	0.892571	NA	0	0.547412	0	0	0	0
28925	LOC11962:	0.091235	0.551076	4.080473	0.135052	0.892571	NA	0	0.547412	0	0	0	0
28927	LOC11962:	0.090348	0.551076	4.080473	0.135052	0.892571	NA	0.542086	0	0	0	0	0
28941	OVOL3	0.115955	0.551076	4.080473	0.135052	0.892571	NA	0	0	0.695731	0	0	0
28943	LOC11962:	0.091235	0.551076	4.080473	0.135052	0.892571	NA	0	0.547412	0	0	0	0
28961	LOC10323(	0.091235	0.551076	4.080473	0.135052	0.892571	NA	0	0.547412	0	0	0	0
28973	CCL4L2	0.091235	0.551076	4.080473	0.135052	0.892571	NA	0	0.547412	0	0	0	0
28977	LOC11962:	0.115955	0.551076	4.080473	0.135052	0.892571	NA	0	0	0.695731	0	0	0
29012	LOC11962;	0.091235	0.551076	4.080473	0.135052	0.892571	NA	0	0.547412	0	0	0	0
29028	LOC10324:	0.115955	0.551076	4.080473	0.135052	0.892571	NA	0	0	0.695731	0	0	0
29039	LOC11962;	0.115955	0.551076	4.080473	0.135052	0.892571	NA	0	0	0.695731	0	0	0
29048	KRTAP16-1	0.091235	0.551076	4.080473	0.135052	0.892571	NA	0	0.547412	0	0	0	0
29066	LOC11962;	0.115955	0.551076	4.080473	0.135052	0.892571	NA	0	0	0.695731	0	0	0
29072	LOC11962;	0.115955	0.551076	4.080473	0.135052	0.892571	NA	0	0	0.695731	0	0	0
29074	LOC11962;	0.091235	0.551076	4.080473	0.135052	0.892571	NA	0	0.547412	0	0	0	0
29103	LOC11962;	0.115955	0.551076	4.080473	0.135052	0.892571	NA	0	0	0.695731	0	0	0
29104	LOC10324:	0.115955	0.551076	4.080473	0.135052	0.892571	NA	0	0	0.695731	0	0	0
29136	LOC11962;	0.091235	0.551076	4.080473	0.135052	0.892571	NA	0	0.547412	0	0	0	0
29137	LOC10323:	0.090348	0.551076	4.080473	0.135052	0.892571	NA	0.542086	0	0	0	0	0
29140	LOC11962;	0.115955	0.551076	4.080473	0.135052	0.892571	NA	0	0	0.695731	0	0	0
29154	LOC11962;	0.090348	0.551076	4.080473	0.135052	0.892571	NA	0.542086	0	0	0	0	0
29193	LOC11962;	0.115955	0.551076	4.080473	0.135052	0.892571	NA	0	0	0.695731	0	0	0
29194	LOC11962;	0.115955	0.551076	4.080473	0.135052	0.892571	NA	0	0	0.695731	0	0	0
29252	LOC11962;	0.115955	0.551076	4.080473	0.135052	0.892571	NA	0	0	0.695731	0	0	0
29289	LOC11962:	0.115955	0.551076	4.080473	0.135052	0.892571	NA	0	0	0.695731	0	0	0
29290	LOC11962:	0.090348	0.551076	4.080473	0.135052	0.892571	NA	0.542086	0	0	0	0	0
29301	LOC10323:	0.115955	0.551076	4.080473	0.135052	0.892571	NA	0	0	0.695731	0	0	0
29340	LOC11962:	0.090348	0.551076	4.080473	0.135052	0.892571	NA	0.542086	0	0	0	0	0
29351	LOC11962:	0.115955	0.551076	4.080473	0.135052	0.892571	NA	0	0	0.695731	0	0	0
29383	LOC11962:	0.090348	0.551076	4.080473	0.135052	0.892571	NA	0.542086	0	0	0	0	0
29388	DGKQ	0.115955	0.551076	4.080473	0.135052	0.892571	NA	0	0	0.695731	0	0	0
29397	LOC11962:	0.090348	0.551076	4.080473	0.135052	0.892571	NA	0.542086	0	0	0	0	0
29401	LOC10323:	0.090348	0.551076	4.080473	0.135052	0.892571	NA	0.542086	0	0	0	0	0
29414	LOC10323:	0.090348	0.551076	4.080473	0.135052	0.892571	NA	0.542086	0	0	0	0	0
29423	LOC11962:	0.091235	0.551076	4.080473	0.135052	0.892571	NA	0	0.547412	0	0	0	0
29486	LOC11962:	0.115955	0.551076	4.080473	0.135052	0.892571	NA	0	0	0.695731	0	0	0
29495	LOC11962:	0.091235	0.551076	4.080473	0.135052	0.892571	NA	0	0.547412	0	0	0	0
29517	TRNAK-UU	0.090348	0.551076	4.080473	0.135052	0.892571	NA	0.542086	0	0	0	0	0
29521	LOC11962:	0.091235	0.551076	4.080473	0.135052	0.892571	NA	0	0.547412	0	0	0	0
29549	LOC11962:	0.091235	0.551076	4.080473	0.135052	0.892571	NA	0	0.547412	0	0	0	0
29554	LOC11962:	0.115955	0.551076	4.080473	0.135052	0.892571	NA	0	0	0.695731	0	0	0
29564	LOC11962:	0.091235	0.551076	4.080473	0.135052	0.892571	NA	0	0.547412	0	0	0	0
29572	LOC11962:	0.090348	0.551076	4.080473	0.135052	0.892571	NA	0.542086	0	0	0	0	0
29583	LOC11962:	0.091235	0.551076	4.080473	0.135052	0.892571	NA	0	0.547412	0	0	0	0
29584	LOC11962:	0.115955	0.551076	4.080473	0.135052	0.892571	NA	0	0	0.695731	0	0	0
29588	LOC11962:	0.091235	0.551076	4.080473	0.135052	0.892571	NA	0	0.547412	0	0	0	0
29602	LOC11962:	0.115955	0.551076	4.080473	0.135052	0.892571	NA	0	0	0.695731	0	0	0
4206	GALNT12	1786.383	0.550722	0.10564	5.213213	1.86E-07	1.17E-06	1495.074	1421.628	1436.685	2091.503	1967.982	2305.423
23617	LOC11962ŧ	5.11483	0.550631	1.181133	0.466189	0.64108	0.721616	4.878777	1.094824	6.261582	11.16063	4.278221	3.014938
22743	LOC11962!	8.388721	0.550429	0.914891	0.601633	0.547418	0.639894	4.878777	8.758589	6.261582	17.85702	8.556443	4.019918
5890	GALT	1018.257	0.550186	0.130382	4.219788	2.45E-05	0.00011	868.4224	770.7558	845.3136	1171.867	1082.39	1370.792
22220	LOC10323;	13.54742	0.550003	0.798422	0.688863	0.490909	0.587346	14.63633	8.211177	10.43597	2.232127	25.66933	20.09959
9785	LOC10324;	219.9319	0.54997	0.189153	2.907534	0.003643	0.009897	174.5518	187.7623	171.1499	285.7123	265.2497	235.1652
17166	LOC11961ŧ	47.22494	0.549942	0.373458	1.472566	0.140868	0.218163	37.40396	42.69812	34.78657	46.87467	66.31243	55.27387
16947	LOC11962(	50.10414	0.549413	0.366347	1.499705	0.133691	0.20972	40.11439	45.98259	35.4823	71.42806	51.33866	56.27885
10147	ZNF784	231.2824	0.549358	0.19436	2.826496	0.004706	0.01233	172.3835	217.8699	173.9328	270.0874	260.9715	292.449
4157	ATP6V1C1	2845.457	0.548798	0.104502	5.251545	1.51E-07	9.65E-07	2313.625	2487.439	2134.504	3218.727	3298.509	3619.936
8838	SAC3D1	370.4055	0.548698	0.173632	3.160111	0.001577	0.004744	323.0835	255.6413	326.298	435.2648	395.7355	486.4101
17111	HCN1	51.53428	0.548691	0.370795	1.479768	0.138935	0.215861	39.03022	52.55153	34.09084	64.73168	53.47777	65.32367
21289	LPAR3	17.49181	0.548681	0.656488	0.835783	0.403277	0.503599	15.17842	10.94824	16.00182	13.39276	36.36488	13.06473
3692	ALDH1A2	3260.684	0.548569	0.09711	5.648924	1.61E-08	1.16E-07	2554.311	2839.972	2553.334	3712.027	3833.286	4071.172

21665	LOC10321٤	14.22973	0.548074	0.701439	0.781356	0.434593	0.533287	9.215468	17.51718	7.653045	13.39276	23.53022	14.06971
23726	LOC10323	4.162504	0.548028	1.218976	0.44958	0.653013	0.731702	4.878777	2.189647	2.782925	6.696381	6.417332	2.009959
18339	LOC11962!	35.43327	0.547813	0.429041	1.276832	0.201662	0.292337	23.8518	29.01283	33.39511	37.94616	49.19955	39.1942
11948	PDGFD	150.3713	0.547689	0.22684	2.414427	0.01576	0.035067	108.4173	129.7366	130.1018	160.7131	173.268	199.9909
17949	LOC10324	38.8199	0.547581	0.40524	1.35125	0.176615	0.261592	29.81475	31.74989	32.69937	55.80317	40.6431	42.20914
17430	LOC11961	399162.5	0.547288	0.384532	1.423258	0.154662	0.235897	213675.3	507061.5	252302.1	471141.7	490789	460005.2
10397	ANKRD23	239.9871	0.547013	0.197706	2.766806	0.005661	0.014474	227.1342	166.4132	191.3261	265.6231	308.0319	281.3943
10440	LOC10322	192.3064	0.546933	0.198373	2.757094	0.005832	0.01485	157.205	168.0554	142.6249	245.534	220.3284	220.0905
5119	SNX33	1177.07	0.546711	0.118337	4.61993	3.84E-06	1.99E-05	993.6443	882.4278	998.3745	1354.901	1315.553	1517.519
6885	LOC10323	518.6186	0.546493	0.14447	3.782734	0.000155	0.000599	410.3594	449.9725	407.6986	589.2815	569.0034	685.396
23260	LOC11962	5.633664	0.546212	1.048365	0.521013	0.602357	0.688464	5.96295	5.474118	2.087194	6.696381	8.556443	5.024897
12637	LOC11962٤	146.7263	0.546047	0.240244	2.272892	0.023033	0.048455	121.9694	129.1892	105.0554	158.481	213.9111	151.7519
3719	MAP7D1	4959.08	0.545768	0.097083	5.621663	1.89E-08	1.35E-07	3991.382	4001.033	4108.989	6017.814	5319.968	6315.291
22137	WFIKKN1	12.73407	0.545767	0.776794	0.702589	0.482312	0.579239	10.84173	6.568942	13.91463	4.464254	23.53022	17.08465
9956	RASSF6	243.5267	0.545755	0.189921	2.873593	0.004058	0.010837	200.572	206.9217	185.0645	261.1589	340.1186	267.3245
5092	TDP2	927.7832	0.545391	0.117705	4.633548	3.59E-06	1.88E-05	704.7123	771.8507	784.785	1162.938	1095.225	1047.189
20072	LOC11962	21.18648	0.545287	0.536163	1.017017	0.309145	0.409457	15.7205	15.87494	20.17621	24.5534	25.66933	25.12449
6244	IL11RA	880.2537	0.545244	0.134517	4.05334	5.05E-05	0.000215	776.8098	622.4072	750.6941	1060.26	966.878	1104.472
7560	APOBR	1180.623	0.5452	0.154106	3.537817	0.000403	0.001419	851.0756	1198.284	829.3118	1392.847	1469.569	1342.653
20779	LOC11962!	24.54806	0.545178	0.601131	0.90692	0.364449	0.466282	17.88885	30.65506	10.43597	42.41041	27.80844	18.08963
4887	ZFYVE26	2521.8	0.544818	0.114733	4.748579	2.05E-06	1.11E-05	2093.538	2013.928	2052.408	2984.354	2611.854	3374.721
23044	LOC11962	6.583457	0.544599	0.980894	0.555206	0.578754	0.667686	2.710432	4.926706	8.348776	8.928508	8.556443	6.029877
7203	IER5L	498.2221	0.544352	0.148481	3.666129	0.000246	0.000909	406.5648	379.3564	432.7449	613.8349	517.6648	639.1669
10607	CYB5RL	206.5858	0.544309	0.20013	2.71978	0.006533	0.016373	175.0939	171.3399	160.0182	209.8199	243.8586	279.3843
10061	LOC11962	383.7717	0.544197	0.191035	2.848674	0.00439	0.011601	366.9925	237.0293	332.5596	435.2648	485.5781	445.2059
15726	LOC11962٤	141.6826	0.544131	0.317588	1.713325	0.086653	0.146488	81.31296	177.9088	86.27069	176.338	160.4333	167.8316
6026	EZR	16388.83	0.544104	0.130995	4.153621	3.27E-05	0.000144	11904.76	16250.47	11850.39	19171.74	18186.72	20968.9
4166	FNTA	1326.508	0.544103	0.103684	5.247718	1.54E-07	9.83E-07	1048.937	1099.203	1086.732	1611.596	1617.168	1495.409
7217	HNF4G	675.5546	0.543917	0.14855	3.661514	0.000251	0.000924	507.9349	637.1874	500.9266	814.7263	859.9225	732.63
18225	LOC11962	35.76014	0.543906	0.419452	1.296706	0.194732	0.284058	32.52518	25.72836	29.22072	37.94616	44.92132	44.2191
13819	LOC10322	147.0826	0.543834	0.265939	2.04496	0.040859	0.078604	147.4475	85.94365	125.2316	200.8914	156.1551	166.8266
19552	FER1L5	28.36772	0.543676	0.498622	1.090356	0.275556	0.374657	23.8518	17.51718	27.13352	37.94616	40.6431	23.11453
7030	TET2	1750.633	0.543471	0.146015	3.722029	0.000198	0.000747	1235.415	1770.33	1269.014	2004.45	2074.937	2149.651
5942	CLCF1	768.3284	0.543094	0.12964	4.189249	2.80E-05	0.000125	584.9112	653.0623	640.7686	946.4218	815.0012	969.8052
21479	LOC11962	14.92212	0.542839	0.672632	0.807037	0.419645	0.519324	9.757555	11.49565	14.61036	20.08914	23.53022	10.04979
20617	LOC10323	21.93854	0.542649	0.581106	0.933821	0.350396	0.451825	17.88885	18.06459	17.39328	13.39276	42.78221	22.10955
7320	CLYBL	379.2354	0.542555	0.149559	3.627697	0.000286	0.001039	294.895	321.8781	308.9047	475.443	432.1004	442.191
6714	PBK	1339.525	0.542356	0.14086	3.850316	0.000118	0.000467	1160.607	883.5227	1228.662	1504.454	1677.063	1582.843
23449	LOC11961	4.788584	0.542328	1.100554	0.492777	0.62217	0.705377	3.794605	3.284471	4.87012	4.464254	4.278221	8.039836
20367	GRIK4	24.72809	0.542147	0.560895	0.966574	0.333757	0.435652	13.55216	21.89647	24.3506	24.5534	44.92132	19.09461
20761	LOC11961٤	21.95396	0.54196	0.595816	0.90961	0.363028	0.464867	21.68345	15.32753	16.00182	44.64254	14.97377	19.09461
8175	SLC18B1	369.5771	0.541858	0.161544	3.354232	0.000796	0.002588	322.5414	276.9904	306.1218	397.3186	432.1004	482.3901
4045	COX20	1652.403	0.541735	0.10148	5.338315	9.38E-08	6.17E-07	1335.159	1391.521	1306.584	1993.289	2040.712	1847.152
22406	LOC10321	12.1302	0.541669	0.825981	0.655789	0.51196	0.607447	12.46799	7.116354	9.740239	4.464254	29.94755	9.044815
8974	LOC10323	286.5206	0.541252	0.173241	3.124267	0.001782	0.005281	253.1543	216.2277	232.3743	308.0335	346.5359	362.7976
4446	KIAA1671	1398.968	0.541165	0.107274	5.044685	4.54E-07	2.72E-06	1089.594	1196.095	1136.825	1616.06	1591.498	1763.739
2740	PON2	6249.111	0.540481	0.079581	6.791613	1.11E-11	1.08E-10	5080.976	5175.779	5018.31	7448.608	7420.575	7350.42
23545	TRNAS-CG	6.040978	0.540226	1.131854	0.477293	0.633154	0.714903	5.96295	3.284471	5.565851	13.39276	0	8.039836
4910	CITED2	3922.943	0.53998	0.113938	4.739233	2.15E-06	1.16E-05	2874.684	3670.944	3046.608	4761.127	4440.794	4743.503
7572	TTC21A	403.2273	0.539879	0.15274	3.534628	0.000408	0.001434	351.8141	324.0678	312.3834	444.1933	466.3261	520.5794
11642	PKD1L1	155.1559	0.539841	0.217244	2.484956	0.012957	0.029587	126.3061	125.9047	125.9274	176.338	209.6328	166.8266
17727	LOC10322	62.28171	0.539556	0.391021	1.379865	0.167628	0.25139	50.95612	31.74989	68.87741	78.12444	87.70354	56.27885
4236	ZNFX1	2963.496	0.539489	0.103824	5.196197	2.03E-07	1.28E-06	2428.547	2586.521	2236.081	3359.351	3394.769	3775.708
3231	SCPEP1	5425.306	0.539323	0.087952	6.132025	8.68E-10	7.14E-09	4651.101	4301.015	4319.1	6437.454	6184.169	6658.994
22562	LOC10322	8.062487	0.539258	0.854846	0.630825	0.528155	0.62233	6.505036	6.02153	6.957314	11.16063	10.69555	7.034856
4944	TRAK1	3508.396	0.539197	0.114287	4.717899	2.38E-06	1.28E-05	2716.937	3218.781	2649.345	3950.865	3965.911	4548.537
23396	LOC11962	6.617596	0.538978	1.078795	0.499611	0.617349	0.701497	1.626259	2.737059	11.82743	8.928508	8.556443	6.029877
21536	LOC10322٤	13.16799	0.53868	0.67386	0.799394	0.424062	0.523481	9.757555	12.04306	10.43597	17.85702	12.83466	16.07967
24353	LOC11962٤	2.392909	0.538464	1.524128	0.353293	0.723869	0.790213	2.710432	1.642235	1.391463	4.464254	2.139111	2.009959
5760	MAP3K1	6000.729	0.538338	0.125328	4.295444	1.74E-05	8.05E-05	4367.048	5850.737	4465.9	7042.361	6751.033	7527.296
24131	LOC10321	3.30093	0.538063	1.396999	0.385156	0.700122	0.771321	3.252518	1.094824	3.478657	6.696381	4.278221	1.004979
24453	GLIPR1L2	2.396459	0.538007	1.594621	0.337388	0.735824	0.799946	0.542086	3.831883	1.391463	4.464254	2.139111	2.009959
4811	MAGT1	7056.811	0.537273	0.111769	4.806999	1.53E-06	8.47E-06	6286.576	4888.935	6099.477	8325.834	8207.768	8532.276
18018	CBLN2	51.62237	0.53707	0.400641	1.340528	0.180074	0.265693	40.65648	36.67659	48.00546	51.33892	89.84265	43.21412
5197	ACOX2	922.261	0.536795	0.117164	4.581582	4.61E-06	2.36E-05	757.2947	735.7215	768.7832	1011.154	1088.807	1171.806
6627	BCOR	2415.202	0.536571	0.137927	3.890244	0.0001	0.000402	1733.05	2396.569	1785.942	2790.159	2710.253	3075.237
23255	LOC10324	5.673887	0.536314	1.028542	0.521431	0.602067	0.68828	5.96295	4.926706	2.782925	8.928508	6.417332	5.024897
5131	CCNE2	747.9063	0.53623	0.116238	4.613196	3.97E-06	2.05E-05	606.0526	606.5323	618.5052	906.2436	872.7572	877.3471
13650	MAN1C1	119.071	0.535754	0.257738	2.078682	0.037647	0.073321	111.6698	84.30142	96.70666	147.3204	117.6511	156.7768
23925	LOC11961٤	3.872957	0.535745	1.277863	0.419251	0.675033	0.750083	1.084173	5.474118	2.782925	4.464254	6.417332	3.014938
19399	LOC10321!	29.69667	0.53568	0.479188	1.117892	0.263613	0.361264	23.8518	18.06459	30.61218	42.41041	32.08666	31.15436
7492	GOLIM4	7471.471	0.535322	0.150212	3.56377	0.000366	0.001297	5238.723	7900.247	5163.718	8888.33	8629.172	9008.636
25410	TRNAG-CC	0.885406	0.535183	2.982533	0.179439	0.857593	0.897309	2.168345	0	0	0	2.139111	1.004979
4834	PFKFB3	1884.799	0.535008	0.111621	4.793094	1.64E-06	9.03E-06	1646.858	1410.133	1565.396	2147.306	2124.137	2414.966

4634	HOMER3	1621.241	0.534773	0.108523	4.92775	8.32E-07	4.77E-06	1223.489	1421.628	1330.238	1843.737	1897.391	2010.964
9628	ZNF174	281.2492	0.534594	0.180994	2.953661	0.00314	0.008673	228.7604	223.8914	240.0273	290.1765	318.7275	385.9121
8180	ZSCAN2	459.2549	0.534426	0.159407	3.352587	0.000801	0.002602	417.4065	390.852	317.9492	537.9426	515.5257	575.8532
23134	LOC11962	6.587233	0.534367	0.990638	0.539417	0.589599	0.677526	3.252518	7.663765	4.87012	11.16063	8.556443	4.019918
24449	LOC11962(	2.669708	0.534053	1.58124	0.337743	0.735557	0.799819	2.168345	3.284471	1.391463	0	2.139111	7.034856
8609	WHRN	324.8717	0.533937	0.165506	3.2261	0.001255	0.003875	288.932	269.3266	236.5487	399.5507	385.0399	369.8324
4683	XRN1	5962.297	0.53388	0.10909	4.893958	9.88E-07	5.61E-06	4525.337	5573.747	4516.688	6794.595	7078.317	7285.096
22676	TRNAG-UC	8.685282	0.533859	0.874572	0.610423	0.541582	0.634821	8.131296	4.379295	8.348776	13.39276	12.83466	5.024897
3812	DIP2C	1549.711	0.533716	0.096527	5.529178	3.22E-08	2.24E-07	1281.492	1267.806	1250.229	1839.273	1803.27	1856.197
6645	AUH	558.4689	0.533391	0.137466	3.880173	0.000104	0.000418	417.4065	480.6276	472.4016	680.7987	618.203	681.3761
4928	ATXN1	2549.146	0.532912	0.112702	4.728495	2.26E-06	1.22E-05	1907.602	2309.53	2037.101	3017.836	2795.818	3226.989
27493	LOC11962(	0.632531	0.532848	2.980222	0.178795	0.858099	NA	0.542086	0.547412	0.695731	0	0	2.009959
4441	DIP2B	4606.18	0.532846	0.105537	5.0489	4.44E-07	2.66E-06	3635.231	4112.705	3551.709	5392.819	5033.327	5911.289
4824	STARD8	1154.089	0.532807	0.111088	4.796273	1.62E-06	8.91E-06	948.6511	932.7897	953.152	1325.883	1287.745	1476.315
16806	TRNAI-AAU	87.34645	0.532712	0.34945	1.524427	0.127402	0.201534	78.60252	47.07742	90.44508	75.89232	98.39909	133.6623
15113	LOC10323	76.3536	0.532656	0.294137	1.81091	0.070155	0.123408	60.17159	68.42648	58.44144	98.21359	83.42532	89.44317
4241	ENTPD4	7715.224	0.532089	0.102465	5.192898	2.07E-07	1.30E-06	6091.425	7068.729	5766.222	9024.489	8898.7	9441.782
19803	LOC11962(	28.99208	0.53206	0.505329	1.052898	0.292388	0.392523	23.8518	29.56024	16.69755	37.94616	42.78221	23.11453
3716	NGLY1	2916.973	0.531916	0.094526	5.627203	1.83E-08	1.31E-07	2279.473	2542.728	2332.787	3374.976	3550.924	3420.95
8256	ZIK1	326.7999	0.531899	0.159848	3.327537	0.000876	0.002821	285.1374	247.4301	269.9438	381.6937	378.6226	397.9719
9448	TTLL11	238.485	0.531755	0.177372	2.997969	0.002718	0.007648	190.8144	206.3743	188.5432	276.7837	275.9453	292.449
4316	PJA2	8477.568	0.531733	0.103381	5.143427	2.70E-07	1.66E-06	6538.646	7815.398	6445.951	9836.984	9790.71	10437.72
21030	RRH	18.48081	0.53171	0.607045	0.875898	0.381086	0.481748	15.7205	18.612	10.43597	20.08914	29.94755	16.07967
5555	FAM126B	1259.742	0.53168	0.121106	4.390213	1.13E-05	5.42E-05	1013.159	1083.875	998.3745	1475.436	1328.388	1659.221
10691	FBXO16	217.7717	0.531543	0.197279	2.694376	0.007052	0.017535	164.2522	202.5424	166.2798	249.9982	282.3626	241.1951
4473	CANX	57762.23	0.531472	0.105717	5.02731	4.97E-07	2.96E-06	44266.77	54134.65	43324.58	66970.51	66825.82	71051.04
8209	SCRIB	3422.881	0.531395	0.158927	3.343644	0.000827	0.002677	3144.643	2097.135	3160.708	4031.221	3535.95	4567.632
22028	LOC10321	14.60722	0.531327	0.737057	0.720875	0.470986	0.568421	5.420864	16.96977	13.2189	11.16063	27.80844	13.06473
17561	LOC10322(	54.12404	0.531225	0.377951	1.405538	0.159861	0.242008	37.94605	56.93083	36.87376	64.73168	77.00798	51.25395
13170	CNNM1	130.7024	0.531171	0.244037	2.176597	0.029511	0.05957	98.11763	123.1677	100.881	142.8561	143.3204	175.8714
6933	LOC10322	420.0879	0.53073	0.141028	3.763283	0.000168	0.000643	353.4403	341.585	335.3425	504.4607	496.2737	489.425
6310	NSFL1C	3333.679	0.530689	0.131958	4.021656	5.78E-05	0.000244	2434.51	3286.113	2462.889	3984.347	3668.575	4165.64
20515	LOC11962	22.9924	0.530684	0.562338	0.943709	0.345318	0.447511	23.8518	16.42235	15.30609	26.78552	38.50399	17.08465
23986	TRNAV-AA	3.5927	0.53063	1.296793	0.409186	0.682403	0.756345	1.084173	4.926706	2.782925	4.464254	4.278221	4.019918
21350	LOC10324	16.41171	0.530562	0.643731	0.824198	0.409827	0.510317	10.84173	13.6853	15.30609	15.62489	29.94755	13.06473
23684	LOC11962(	4.762812	0.530265	1.162928	0.455974	0.648409	0.727831	3.252518	3.284471	4.87012	4.464254	10.69555	2.009959
21216	NOL4	24.63432	0.530214	0.625156	0.848131	0.396365	0.496671	10.84173	22.9913	25.74206	31.24978	44.92132	12.05975
23781	LOC11962	5.066902	0.529558	1.204167	0.439771	0.660103	0.737935	3.794605	2.737059	5.565851	4.464254	12.83466	1.004979
19985	LOC10323	26.23947	0.529215	0.512995	1.031616	0.302252	0.40207	23.30971	22.44388	18.08902	24.5534	44.92132	24.11951
5856	SMIM19	746.3305	0.529125	0.124728	4.242215	2.21E-05	0.0001	583.2849	590.6573	658.1619	868.2974	928.374	849.2077
16563	CUNH16or	69.70601	0.529055	0.338354	1.563615	0.117908	0.189252	66.13454	41.05589	64.70302	69.19594	87.70354	89.44317
3912	ATXN3	2561.418	0.528946	0.097307	5.435859	5.45E-08	3.71E-07	2115.221	2221.945	1952.918	3098.192	2956.251	3023.983
19917	KANK3	26.64949	0.528632	0.505931	1.044869	0.296083	0.395209	19.51511	20.80165	24.3506	33.4819	40.6431	21.10457
7037	FAHD1	644.0632	0.528227	0.142098	3.717332	0.000201	0.000761	501.4299	598.3211	481.4461	814.7263	718.7412	749.7147
23439	LOC10322	5.649106	0.527996	1.069413	0.493725	0.621501	0.704906	4.878777	3.831883	4.87012	4.464254	12.83466	3.014938
5642	SMIM3	792.5954	0.527948	0.121354	4.350494	1.36E-05	6.40E-05	666.7662	605.9849	676.9466	892.8508	941.2087	971.8151
9558	LOC11962	692.5548	0.527722	0.177657	2.97046	0.002974	0.008271	481.3727	730.7948	487.7077	863.8331	827.8358	763.7844
16440	LOC11962(	60.92083	0.527457	0.333425	1.581939	0.113664	0.183804	54.20864	41.6033	53.57132	80.35657	68.45154	67.33362
5073	MMUT	1485.566	0.527345	0.113494	4.646464	3.38E-06	1.77E-05	1169.822	1342.801	1138.912	1808.023	1659.95	1793.888
10267	RESF1	1181.105	0.527151	0.18848	2.796847	0.00516	0.013363	760.0051	1329.116	814.0057	1410.704	1332.666	1440.136
15147	LOC10324	94.42395	0.527099	0.291815	1.80628	0.070875	0.124394	66.67662	98.53413	66.79021	118.3027	102.6773	113.5627
25320	LOC11962	0.886294	0.526927	2.677731	0.196781	0.843999	0.88613	1.626259	0.547412	0	0	2.139111	1.004979
25321	LOC11962	0.886294	0.526927	2.677731	0.196781	0.843999	0.88613	1.626259	0.547412	0	0	2.139111	1.004979
25310	LOC11961	0.901796	0.526656	2.664348	0.197668	0.843305	0.885738	1.626259	0.547412	0	2.232127	0	1.004979
3632	OXR1	5152.736	0.526486	0.092196	5.71048	1.13E-08	8.25E-08	4154.008	4530.38	3983.062	5921.833	6276.151	6050.981
7390	LOC11962(	6911.604	0.526027	0.146004	3.602815	0.000315	0.001132	5579.695	5818.988	5604.116	8227.62	6237.647	10001.56
19618	LOC10324	27.58883	0.525967	0.485648	1.083023	0.278798	0.377809	20.0572	24.08612	22.95914	37.94616	36.36488	24.11951
7893	TNFSF12	408.0187	0.525922	0.152712	3.443885	0.000573	0.001931	319.831	367.3133	317.9492	464.2824	464.187	514.5495
4971	ARHGEF17	3193.703	0.525764	0.111799	4.702776	2.57E-06	1.37E-05	2717.479	2508.788	2634.735	3705.331	3349.847	4246.038
5049	SP100	4740.031	0.525681	0.112772	4.661434	3.14E-06	1.65E-05	3573.433	4500.273	3583.712	5524.514	5617.305	5640.95
4485	LSS	8272.527	0.525507	0.104695	5.019407	5.18E-07	3.07E-06	7190.776	6371.326	6791.73	9464.218	8937.204	10879.91
23622	LOC11961	4.768112	0.525482	1.128477	0.465656	0.641462	0.721923	4.878777	2.737059	4.174388	2.232127	8.556443	6.029877
4176	LNX2	2258.617	0.525445	0.100208	5.243518	1.58E-07	1.00E-06	1782.38	1970.135	1806.119	2529	2663.193	2800.878
3674	PLOD1	6570.679	0.525193	0.092541	5.675213	1.39E-08	1.00E-07	5390.507	5508.058	5270.165	7718.695	7185.273	8351.379
29010	LOC11962	0.631644	0.524736	3.306383	0.158704	0.873902	NA	1.084173	0	0.695731	0	0	2.009959
19828	LOC10323	27.25142	0.524702	0.49977	1.049888	0.29377	0.39388	26.56223	16.96977	22.95914	31.24978	40.6431	25.12449
5098	TAF2	5872.915	0.524689	0.113329	4.629778	3.66E-06	1.91E-05	4522.084	5562.251	4367.106	6660.667	6864.406	7260.977
16750	SIGLEC1	94.30081	0.524087	0.341908	1.532829	0.125318	0.1989	73.18166	56.38342	100.881	127.2312	134.764	73.3635
4093	CCAR2	4843.326	0.524083	0.098875	5.300452	1.16E-07	7.50E-07	4024.449	3984.063	3917.663	5591.478	5245.099	6297.201
5616	POLR2A	1092.583	0.523898	0.120034	4.36457	1.27E-05	6.03E-05	917.2101	962.8974	810.527	1285.705	1221.432	1357.727
4732	SYT13	1692.83	0.523602	0.107715	4.861001	1.17E-06	6.56E-06	1430.024	1338.422	1403.986	1915.165	1884.556	2184.825
5158	LOC11962	907.4675	0.523513	0.113843	4.598551	4.25E-06	2.19E-05	754.0421	739.5534	742.3454	999.9929	1097.364	1111.507
4371	CALM2	36051.02	0.523442	0.102638	5.09986	3.40E-07	2.07E-06	29410.35	27002.73	32327.85	43624.69	45118.12	38822.36
13893	PEX11A	139.8763	0.523334	0.257566	2.031846	0.042169	0.080693	147.9896	98.53413	98.09812	151.7846	168.9897	173.8614

25303	LOC11962(	0.902684	0.523171	2.636943	0.1984	0.842732	0.885395	1.084173	1.094824	0	2.232127	0	1.004979
25304	LOC11962:	0.902684	0.523171	2.636943	0.1984	0.842732	0.885395	1.084173	1.094824	0	2.232127	0	1.004979
6869	LZTS3	856.956	0.523064	0.138056	3.788792	0.000151	0.000586	685.1972	798.6738	627.5497	1029.011	921.9567	1079.348
10295	CCDC25	769.4773	0.52296	0.187444	2.789946	0.005272	0.013613	534.4972	847.9409	511.3626	901.7793	919.8176	901.4666
6864	PRDX3	7823.134	0.52292	0.13793	3.791205	0.00015	0.000581	7177.766	4948.603	7137.508	9325.827	8791.745	9557.355
6125	THBS3	1648.792	0.522832	0.127318	4.106509	4.02E-05	0.000174	1488.569	1189.526	1386.593	1828.112	1833.218	2166.736
4805	PNPLA3	1481.448	0.522704	0.108663	4.810324	1.51E-06	8.34E-06	1222.405	1208.685	1222.4	1665.167	1653.533	1916.496
21599	LOC10324	24.87164	0.522678	0.661124	0.790589	0.429184	0.528258	10.29964	39.96106	10.43597	26.78552	40.6431	21.10457
20674	LOC10322!	22.13535	0.522396	0.564556	0.925323	0.354798	0.45624	14.09425	26.27577	13.91463	22.32127	32.08666	24.11951
21060	LOC11961!	19.97637	0.522322	0.599401	0.871406	0.383532	0.48415	17.88885	15.32753	16.00182	11.16063	36.36488	23.11453
4310	CUNH8orf:	1889.866	0.522166	0.101395	5.149815	2.61E-07	1.61E-06	1611.623	1466.516	1579.31	2252.216	2107.024	2322.508
9303	LOC11962(	428.1075	0.5221	0.172227	3.031458	0.002434	0.006955	339.8882	408.9166	308.209	455.3539	483.439	572.8383
5534	LSG1	1568.889	0.522047	0.118685	4.398578	1.09E-05	5.23E-05	1262.519	1442.978	1160.48	1816.951	1779.74	1950.665
4929	PACS1	1059.867	0.521958	0.110397	4.728	2.27E-06	1.22E-05	877.6378	840.8245	895.4063	1229.902	1197.902	1317.528
14423	SEC31B	96.0482	0.521516	0.2689	1.939443	0.052447	0.096673	80.22878	82.65918	72.35606	122.767	119.7902	98.48799
22690	LOC11962!	10.70349	0.521476	0.855596	0.609488	0.542201	0.63522	7.047123	12.04306	6.957314	4.464254	25.66933	8.039836
22951	C1QTNF12	6.924537	0.521294	0.912984	0.570978	0.568014	0.657952	5.420864	5.474118	6.261582	8.928508	6.417332	9.044815
23864	LOC10323:	3.911404	0.521019	1.216949	0.428135	0.668553	0.744782	2.168345	3.831883	3.478657	6.696381	4.278221	3.014938
11068	HOXD3	196.9063	0.52075	0.199386	2.611771	0.009007	0.021634	154.4946	180.6459	150.9737	229.9091	218.1893	247.2249
4276	TET3	1970.718	0.520741	0.100682	5.172135	2.31E-07	1.44E-06	1607.828	1731.464	1517.39	2334.805	2278.153	2354.667
5402	LOC10324:	10013.18	0.520332	0.1166	4.462553	8.10E-06	3.99E-05	7620.65	9619.12	7442.934	11495.45	11343.7	12557.22
13361	LOC10324(	153.3913	0.519873	0.243529	2.134752	0.032781	0.065226	158.8313	112.7668	105.7512	183.0344	186.1026	173.8614
25422	LOC11962:	0.892151	0.519687	2.924601	0.177695	0.858963	0.898258	0	1.642235	0.695731	0	0	3.014938
12348	ISG20	170.8615	0.519625	0.222596	2.334391	0.019575	0.042145	142.5687	118.7884	160.7139	216.5163	177.5462	209.0357
7957	PPP1R13L	420.7357	0.519576	0.151781	3.423194	0.000619	0.002068	325.2518	368.9556	342.2998	546.8711	455.6306	485.4051
2932	CD63	16380.47	0.519422	0.079806	6.508563	7.59E-11	6.88E-10	13245.34	13944.77	13202.2	19133.79	18854.12	19902.61
19680	LOC11962(	28.1845	0.519246	0.483569	1.07378	0.282922	0.382188	22.22554	23.53871	22.95914	33.4819	42.78221	24.11951
4902	KSR1	974.522	0.518983	0.109465	4.741071	2.13E-06	1.15E-05	771.931	822.76	807.7441	1167.402	1148.702	1128.592
5599	POLK	2444.898	0.518687	0.118578	4.374216	1.22E-05	5.79E-05	1884.834	2293.108	1852.037	2676.32	3071.763	2891.326
6762	TRAF5	715.5534	0.518546	0.13534	3.831433	0.000127	0.000501	583.2849	643.2089	536.4089	801.3336	924.0958	804.9886
7869	SDF2L1	462.9864	0.518449	0.150287	3.449721	0.000561	0.001896	401.1439	336.1109	406.3071	513.3892	556.1688	564.7985
14651	LOC11962:	108.8993	0.518401	0.273304	1.896794	0.057855	0.104981	74.26583	108.3875	84.87923	145.0883	126.2075	114.5677
21861	FAM24B	15.83586	0.518203	0.690475	0.750501	0.452953	0.550812	12.46799	11.49565	14.61036	11.16063	34.22577	11.05477
17659	TMEM8B	57.96254	0.518137	0.37147	1.394829	0.163067	0.245492	61.79785	33.39212	47.30973	66.96381	77.00798	61.30375
6693	TAP2	1082.353	0.517961	0.134228	3.858818	0.000114	0.000453	943.2303	784.9885	946.8904	1238.83	1155.12	1425.061
9409	SFT2D2	418.7279	0.517848	0.172292	3.005645	0.00265	0.007488	374.5817	298.3394	363.8675	426.3363	483.439	565.8034
4790	CAPZA2	13906.83	0.517534	0.107389	4.819254	1.44E-06	8.00E-06	11696.06	10030.77	12587.17	16533.36	17262.62	15330.96
6678	RRBP1	5667.765	0.517388	0.133833	3.865918	0.000111	0.000441	4089.5	5694.725	4204.305	6618.256	6265.455	7134.349
8900	PPCDC	617.4078	0.517357	0.164507	3.14489	0.001661	0.004963	552.9281	448.3303	527.3644	683.0309	622.4812	870.3122
23846	LOC10324:	5.146612	0.517201	1.200267	0.430905	0.666537	0.743097	2.710432	6.02153	4.174388	8.928508	0	9.044815
17591	LOC10323(	54.22106	0.516892	0.368867	1.401295	0.161126	0.24309	33.60935	53.64636	46.614	73.66019	53.47777	64.31869
21550	LOC11961{	15.62369	0.516884	0.648848	0.796618	0.425673	0.525128	10.84173	17.51718	9.740239	22.32127	19.252	14.06971
20621	NOXRED1	24.37132	0.516487	0.553582	0.93299	0.350825	0.452283	14.63633	19.70683	25.04633	31.24978	38.50399	17.08465
4569	TANC1	2980.538	0.516457	0.104121	4.960138	7.04E-07	4.10E-06	2433.426	2641.809	2286.869	3334.798	3407.603	3778.723
24951	LOC11962:	1.504412	0.516417	2.029396	0.254468	0.799134	0.851468	0.542086	0.547412	2.782925	0	2.139111	3.014938
4110	HEXA	4858.207	0.516378	0.097686	5.286131	1.25E-07	8.08E-07	4180.028	3906.331	3913.489	5571.389	5337.081	6240.923
4196	CPSF1	8421.625	0.516277	0.09888	5.221244	1.78E-07	1.13E-06	7136.567	6502.705	7159.076	9807.966	9116.89	10806.54
14869	LOC10322(	117.0533	0.516201	0.277606	1.859476	0.06296	0.112569	123.5957	85.94365	78.61764	127.2312	158.2942	128.6374
4088	RAB1B	3708.264	0.516081	0.09727	5.305673	1.12E-07	7.30E-07	3110.492	3051.273	2999.994	4303.541	4040.78	4743.503
8862	TOR4A	479.7439	0.515953	0.163581	3.154111	0.00161	0.004829	410.9015	332.8264	443.1809	504.4607	586.1163	600.9777
16757	LOC10323:	63.53019	0.515847	0.336716	1.531995	0.125524	0.199147	55.29281	41.05589	61.22436	75.89232	66.31243	81.40334
7415	RNASE4	686.9897	0.515798	0.143488	3.594702	0.000325	0.001164	604.9684	479.5327	612.2436	845.9761	772.219	806.9985
6829	CKAP2	4509.347	0.515591	0.135411	3.807608	0.00014	0.000546	3207.525	4586.216	3341.598	5339.248	5294.299	5287.197
5744	DST	102006.2	0.515369	0.119799	4.301954	1.69E-05	7.84E-05	76116.52	99567.64	76252.16	115358.6	115661.7	129080.6
5913	LOC10323:	43476.12	0.515345	0.122543	4.205417	2.61E-05	0.000117	38684.91	28873.78	39818.79	52247.4	51404.97	49826.88
8474	PUS3	826.8495	0.515317	0.158023	3.261015	0.00111	0.003482	612.0155	841.9194	586.5015	997.7608	988.2691	934.6309
17651	NFKBID	57.37554	0.515077	0.368853	1.396429	0.162585	0.244864	63.96619	38.31883	38.96096	66.96381	72.72976	63.31371
26696	LOC11961{	0.603932	0.51493	3.824945	0.134624	0.892909	NA	0	0	1.391463	2.232127	0	0
27359	LOC10324:	0.603932	0.51493	3.824945	0.134624	0.892909	NA	0	0	1.391463	2.232127	0	0
27540	LOC10324{	0.603932	0.51493	3.824945	0.134624	0.892909	NA	0	0	1.391463	2.232127	0	0
27794	LOC10322:	0.603932	0.51493	3.824945	0.134624	0.892909	NA	0	0	1.391463	2.232127	0	0
28841	TAS2R19	0.603932	0.51493	3.824945	0.134624	0.892909	NA	0	0	1.391463	2.232127	0	0
28967	LOC11962:	0.603932	0.51493	3.824945	0.134624	0.892909	NA	0	0	1.391463	2.232127	0	0
26698	IL4	0.588429	0.514863	3.859445	0.133403	0.893874	NA	0	0	1.391463	0	2.139111	0
26949	LOC11962	0.588429	0.514863	3.859445	0.133403	0.893874	NA	0	0	1.391463	0	2.139111	0
27049	LOC10323!	0.588429	0.514863	3.859445	0.133403	0.893874	NA	0	0	1.391463	0	2.139111	0
27427	LOC11962(	0.588429	0.514863	3.859445	0.133403	0.893874	NA	0	0	1.391463	0	2.139111	0
27455	LOC11962(	0.588429	0.514863	3.859445	0.133403	0.893874	NA	0	0	1.391463	0	2.139111	0
27584	LOC10324:	0.588429	0.514863	3.859445	0.133403	0.893874	NA	0	0	1.391463	0	2.139111	0
27848	LOC11962:	0.588429	0.514863	3.859445	0.133403	0.893874	NA	0	0	1.391463	0	2.139111	0
28049	LOC11961{	0.588429	0.514863	3.859445	0.133403	0.893874	NA	0	0	1.391463	0	2.139111	0
28942	LGALS7B	0.588429	0.514863	3.859445	0.133403	0.893874	NA	0	0	1.391463	0	2.139111	0
28952	LOC11962:	0.588429	0.514863	3.859445	0.133403	0.893874	NA	0	0	1.391463	0	2.139111	0
29031	LOC10324:	0.588429	0.514863	3.859445	0.133403	0.893874	NA	0	0	1.391463	0	2.139111	0

6427	RNF168	1548.034	0.514811	0.129714	3.968821	7.22E-05	0.000299	1181.206	1502.645	1140.304	1828.112	1779.74	1856.197
5832	WWP1	2019.107	0.514644	0.12088	4.257488	2.07E-05	9.42E-05	1585.061	1898.424	1506.954	2218.734	2382.969	2522.498
15816	LOC11962(	88.04642	0.514258	0.302957	1.69746	0.08961	0.150624	65.05036	87.58589	64.00729	129.4634	87.70354	94.46807
6048	SOS2	2748.681	0.514234	0.124074	4.14458	3.40E-05	0.00015	2073.48	2676.296	2042.667	3191.942	3170.162	3337.537
4017	KANK2	6905.249	0.514231	0.095978	5.357791	8.42E-08	5.58E-07	5454.473	6062.038	5549.849	8229.852	7512.557	8622.724
5706	PLD1	1075.475	0.514215	0.118923	4.323925	1.53E-05	7.14E-05	915.0418	927.3156	817.4844	1270.08	1172.233	1350.692
4894	IGF2R	21986.65	0.513976	0.108312	4.745337	2.08E-06	1.13E-05	17631.9	19568.33	17140.04	24488.67	23767.66	29323.29
14106	LOC10324(	110.6302	0.513807	0.257813	1.992942	0.046268	0.087199	88.36008	90.32295	93.228	156.2489	124.0684	111.5527
21029	OVOL1	17.78464	0.513738	0.586423	0.876054	0.381001	0.481664	16.26259	12.59047	15.30609	22.32127	17.11289	23.11453
5622	FAT1	91961.8	0.513725	0.117765	4.362305	1.29E-05	6.08E-05	69075.36	88608.46	69598.18	104729.2	101798.1	117961.5
24686	LOC11962:	2.105149	0.513525	1.704292	0.301313	0.763176	0.82187	0.542086	1.094824	3.478657	2.232127	4.278221	1.004979
6303	KLC4	845.152	0.513204	0.12751	4.024831	5.70E-05	0.00024	700.3756	642.1141	750.6941	968.7431	932.6523	1076.333
24873	TRNAW-C(	1.79609	0.512644	1.896526	0.270307	0.786924	0.841146	1.084173	1.094824	2.087194	2.232127	4.278221	0
6003	SCAMP4	2363.822	0.512633	0.123157	4.162429	3.15E-05	0.000139	2053.965	1722.158	2074.671	2631.678	2566.933	3133.526
7254	SUFU	422.0294	0.512583	0.1404	3.650874	0.000261	0.000958	349.1036	344.8694	349.9529	504.4607	481.2999	502.4897
4434	SLC29A1	3321.21	0.512498	0.101382	5.055116	4.30E-07	2.58E-06	2725.068	2761.145	2732.833	3566.939	3843.982	4297.292
9673	GLT8D2	269.4615	0.512454	0.174413	2.938166	0.003302	0.009074	213.582	242.5034	211.5023	301.3371	310.171	337.6731
6498	RUSC1	864.7489	0.512109	0.129944	3.940987	8.11E-05	0.000332	705.2544	695.213	743.0411	1008.921	898.4265	1137.637
22062	PCSK1	12.69683	0.512021	0.716403	0.714711	0.474788	0.572126	10.84173	12.04306	8.348776	22.32127	8.556443	14.06971
21263	LOC10321;	17.17797	0.511918	0.609159	0.840369	0.400702	0.500995	13.01007	16.42235	12.52316	26.78552	19.252	15.07469
5709	PLEKHG5	1285.514	0.511633	0.118391	4.321568	1.55E-05	7.21E-05	1076.041	1018.733	1090.907	1450.883	1386.144	1690.375
5786	LOC11961(	913.6782	0.5112	0.119396	4.281558	1.86E-05	8.53E-05	775.1835	698.4975	789.6551	1062.492	1024.634	1131.607
7067	ABHD14B	593.4814	0.511057	0.13792	3.705457	0.000211	0.000794	472.1572	500.3344	500.2309	647.3168	660.9852	779.8641
5654	PUF60	7167.833	0.511041	0.117616	4.345011	1.39E-05	6.55E-05	6378.188	5056.443	6304.718	8218.692	7831.284	9217.672
4301	LRP12	2397.377	0.511009	0.099151	5.153848	2.55E-07	1.58E-06	1928.743	2106.441	1899.347	2689.713	2804.374	2955.645
4600	PLEKHA2	1781.05	0.511	0.103386	4.942638	7.71E-07	4.45E-06	1458.754	1538.775	1412.335	2093.735	1963.704	2218.995
23664	LOC11961(	4.816193	0.510951	1.110328	0.460181	0.645387	0.725051	4.878777	4.926706	2.087194	6.696381	4.278221	6.029877
4680	MYCBP2	8054.733	0.510806	0.104316	4.89673	9.74E-07	5.54E-06	6309.343	7346.267	6279.671	9075.828	9031.325	10285.96
24429	CTAGE1	3.626533	0.510698	1.501288	0.340173	0.733726	0.798482	1.084173	5.474118	2.087194	6.696381	6.417332	0
18836	ERBB3	40.91823	0.510602	0.420923	1.213053	0.22511	0.317706	28.18849	37.224	34.78657	49.10679	62.03421	34.1693
8223	PPP1R13B	714.1367	0.510573	0.152862	3.340093	0.000838	0.002708	530.1605	708.8983	528.7558	841.5119	802.1665	873.3272
4955	SFXN5	1682.654	0.510535	0.108378	4.710687	2.47E-06	1.32E-05	1440.866	1352.655	1373.374	2055.789	1781.879	2091.362
4125	PLPPR5	2175.205	0.510455	0.096807	5.272931	1.34E-07	8.65E-07	1749.855	1907.73	1724.718	2515.607	2609.715	2543.603
6657	ZBTB47	1094.342	0.510152	0.1317	3.873603	0.000107	0.000428	957.3245	834.803	921.1483	1107.135	1315.553	1430.086
5587	ERAP2	1483.216	0.510089	0.116497	4.378557	1.19E-05	5.68E-05	1135.129	1371.814	1164.654	1669.631	1788.297	1769.769
6319	SYNGR1	1485.516	0.510012	0.12697	4.016782	5.90E-05	0.000248	1308.054	1052.126	1320.498	1636.149	1721.984	1874.287
4499	B2M	33860.27	0.509978	0.101766	5.011273	5.41E-07	3.20E-06	26742.75	26130.7	30934.3	40714	42016.41	36623.46
24473	LOC10321(	2.417629	0.509828	1.524753	0.334368	0.738102	0.8018	2.710432	1.094824	2.087194	4.464254	2.139111	2.009959
23323	SMIM33	6.594247	0.509554	0.998365	0.510388	0.60978	0.695035	2.710432	6.568942	6.957314	4.464254	12.83466	6.029877
4763	ITGB1	79497.45	0.509465	0.105344	4.836202	1.32E-06	7.39E-06	61937.16	75044.14	59834.99	91615.42	91295.1	97257.89
6812	SLC46A3	634.2162	0.509035	0.13351	3.812715	0.000137	0.000536	504.6824	575.8772	491.1863	720.977	733.715	778.8591
6163	KBTBD4	1068.763	0.508969	0.124363	4.092589	4.27E-05	0.000184	960.577	816.191	873.8386	1178.563	1208.598	1374.812
14462	LOC11962:	99.31219	0.508968	0.263431	1.932074	0.05335	0.098072	91.07051	74.99542	80.00911	107.1421	124.0684	118.5876
5606	FJX1	1484.971	0.508571	0.116364	4.370504	1.24E-05	5.88E-05	1235.957	1207.043	1239.793	1765.612	1535.881	1925.541
15244	VPS37D	116.025	0.508483	0.284197	1.789193	0.073584	0.128327	109.5014	68.97389	108.5341	160.7131	119.7902	128.6374
7053	MAP3K6	724.4446	0.508023	0.136896	3.711008	0.000206	0.000778	591.9583	592.2996	615.7223	754.4589	821.4185	970.8102
16605	LOC11961(	76.47076	0.507763	0.326237	1.556425	0.119607	0.191482	60.17159	82.11177	46.614	91.51721	91.98176	86.42823
27122	LOC11962‹	0.3738	0.50776	4.021111	0.126273	0.899515	NA	0.542086	0	0.695731	0	0	1.004979
27151	LOC11962‹	0.3738	0.50776	4.021111	0.126273	0.899515	NA	0.542086	0	0.695731	0	0	1.004979
6553	CTR9	3537.313	0.507667	0.129561	3.918366	8.92E-05	0.000362	2705.553	3489.75	2570.727	4031.221	3959.494	4467.134
10016	RSPH3	329.8922	0.507564	0.177634	2.857357	0.004272	0.011339	267.7907	310.9299	240.0273	341.5154	400.0137	419.0764
4772	PIK3R4	4230.997	0.507497	0.105076	4.829787	1.37E-06	7.61E-06	3467.184	3693.935	3326.987	5006.661	4464.324	5426.889
4370	TMF1	5545.538	0.507492	0.099507	5.100083	3.40E-07	2.07E-06	4464.081	5042.758	4233.525	6243.259	6703.973	6585.63
11583	STK32C	181.7576	0.507485	0.203133	2.498296	0.012479	0.028642	165.8784	139.0426	144.7121	220.9806	213.9111	206.0208
5609	HK2	12190.55	0.507439	0.116158	4.368522	1.25E-05	5.93E-05	9136.324	11613.34	9459.859	13886.06	13412.22	15635.47
25203	LOC11962(	1.197128	0.507354	2.365757	0.214457	0.83019	0.875679	0	1.642235	1.391463	0	2.139111	2.009959
5635	LMF2	1892.12	0.507158	0.116411	4.35663	1.32E-05	6.23E-05	1604.034	1455.568	1632.186	2363.822	1944.452	2352.657
24430	LOC11962(	2.683271	0.507036	1.490798	0.34011	0.733774	0.798501	2.168345	2.189647	2.087194	2.232127	6.417332	1.004979
4097	SDF4	3434.909	0.506892	0.09565	5.299428	1.16E-07	7.54E-07	2880.105	2975.183	2660.477	3979.882	3871.79	4242.018
5248	SLC39A14	13660.49	0.506678	0.111271	4.553544	5.27E-06	2.67E-05	12338.43	10308.31	11217.97	15421.77	14657.19	18019.28
9609	MAML3	520.7098	0.506609	0.17121	2.958991	0.003086	0.008539	371.3292	527.1576	393.784	582.5851	586.1163	663.2864
4087	CLTB	3561.324	0.506591	0.095475	5.305998	1.12E-07	7.29E-07	2829.149	3167.872	2830.931	4270.059	4010.833	4259.103
4886	PREX1	2424.87	0.506513	0.106664	4.748671	2.05E-06	1.11E-05	2060.47	1996.411	1959.18	2832.569	2569.072	3131.516
3580	MORF4L1	8968.019	0.506487	0.087713	5.774343	7.73E-09	5.74E-08	6950.089	7846.601	7430.411	10613.76	10725.5	10241.75
4329	DOP1A	1954.297	0.506416	0.098684	5.131677	2.87E-07	1.76E-06	1610.539	1655.921	1582.093	2225.431	2209.701	2442.1
7747	EME2	972.9736	0.505982	0.145451	3.47872	0.000504	0.001729	869.5065	682.6225	863.4026	1178.563	992.5473	1251.199
11556	SETD1B	228.4401	0.505949	0.202082	2.503681	0.012291	0.028277	162.6259	204.732	200.3706	225.4448	293.0582	284.4092
7369	TSPAN7	628.0982	0.505445	0.140031	3.609529	0.000307	0.001107	492.7565	572.5928	495.3607	691.9594	705.9065	810.0135
6720	ZNF16	956.3415	0.505229	0.131284	3.848366	0.000119	0.00047	865.7119	722.0362	786.8722	995.5286	1167.954	1199.945
7421	DGKH	546.2234	0.505081	0.140626	3.591649	0.000329	0.001177	487.8777	441.2139	427.8748	584.8173	648.1505	687.406
23507	LOC11962:	5.762661	0.505036	1.046637	0.482532	0.629428	0.711845	6.505036	2.737059	4.87012	11.16063	4.278221	5.024897
3483	COMMD2	3611.381	0.505014	0.085991	5.872865	4.28E-09	3.27E-08	2973.344	3078.644	2903.287	4236.577	4301.752	4174.685
7219	FBXO44	1458.37	0.504977	0.137928	3.661172	0.000251	0.000924	1321.064	1017.639	1282.929	1667.399	1548.716	1912.476
20153	TSPO	915.4749	0.504846	0.503278	1.003116	0.315805	0.416597	995.8127	332.8264	942.716	1113.831	819.2794	1288.384

22335	ALOX15	12.35993	0.504766	0.754375	0.669118	0.50342	0.599213	5.420864	13.6853	11.1317	15.62489	19.252	9.044815
16709	GLIS1	70.78221	0.50474	0.327567	1.540873	0.123348	0.196253	67.21871	45.98259	61.92009	73.66019	100.5382	75.37346
22907	LOC11962:	8.432193	0.504562	0.871927	0.578674	0.562809	0.65318	4.878777	10.94824	4.87012	11.16063	10.69555	8.039836
23264	LOC11962:	6.581277	0.504496	0.969941	0.52013	0.602973	0.689072	5.96295	6.02153	4.174388	4.464254	12.83466	6.029877
4821	ERMP1	2880.795	0.504496	0.105109	4.79972	1.59E-06	8.76E-06	2470.83	2440.909	2240.255	3156.228	3262.144	3714.404
7662	USP20	1949.353	0.504343	0.143846	3.506139	0.000455	0.001578	1745.518	1400.279	1696.193	2254.448	1912.365	2687.315
9935	AAMDC	286.335	0.504284	0.175243	2.877627	0.004007	0.010722	236.3497	218.4173	253.942	332.5869	372.2053	304.5088
8485	TBC1D25	471.696	0.503995	0.15464	3.259143	0.001117	0.003501	355.0666	433.5502	384.0437	560.2639	496.2737	600.9777
21907	LOC10322(	15.33852	0.503894	0.677913	0.743302	0.457299	0.55495	13.55216	8.211177	16.00182	13.39276	27.80844	13.06473
16044	TMEM160	85.4883	0.503433	0.303722	1.657544	0.097409	0.161398	81.31296	52.55153	77.92191	107.1421	100.5382	93.46309
10158	SELPLG	298.7568	0.502902	0.178092	2.823836	0.004745	0.012418	234.7234	283.5593	224.7212	334.819	335.8404	378.8773
20309	LOC11962:	36.07521	0.5028	0.51457	0.977127	0.328507	0.430023	22.76763	21.89647	45.22254	22.32127	62.03421	42.20914
3942	RAB9A	2533.108	0.502548	0.092854	5.412213	6.23E-08	4.20E-07	2055.049	2035.825	2196.424	3026.764	3003.311	2881.276
28347	LOC11961!	0.374687	0.502185	4.017799	0.12499	0.900531	NA	0	0.547412	0.695731	0	0	1.004979
28743	LOC11962(	0.374687	0.502185	4.017799	0.12499	0.900531	NA	0	0.547412	0.695731	0	0	1.004979
28920	LOC11962:	0.374687	0.502185	4.017799	0.12499	0.900531	NA	0	0.547412	0.695731	0	0	1.004979
28975	LOC11962:	0.374687	0.502185	4.017799	0.12499	0.900531	NA	0	0.547412	0.695731	0	0	1.004979
21624	LOC11961ℓ	15.14231	0.501855	0.638128	0.786448	0.431605	0.530624	12.46799	10.40082	14.61036	22.32127	14.97377	16.07967
5401	PDLIM4	1822.477	0.501734	0.11243	4.46264	8.10E-06	3.98E-05	1528.141	1503.193	1500.693	2113.824	1910.226	2378.786
24296	LOC11961!	3.317118	0.501635	1.382981	0.36272	0.716814	0.784347	3.794605	2.737059	1.391463	6.696381	4.278221	1.004979
6667	SWAP70	2234.664	0.501422	0.129552	3.870443	0.000109	0.000433	1720.582	2199.501	1631.49	2511.143	2609.715	2735.554
10071	SV2A	251.1741	0.501306	0.176162	2.845712	0.004431	0.011697	202.1982	210.2061	210.1109	323.6584	284.5017	276.3694
24053	LOC10323ℓ	4.262687	0.501205	1.260296	0.397689	0.69086	0.763585	3.252518	4.926706	2.087194	11.16063	2.139111	2.009959
17652	TPGS1	65.2975	0.500896	0.358692	1.396454	0.162578	0.244864	53.12446	37.77142	70.9646	75.89232	87.70354	66.32864
14629	LOC11962(	124.292	0.500836	0.263389	1.901506	0.057236	0.104014	106.2489	96.89189	104.3597	191.9629	117.6511	128.6374
5520	LRP1	12904.38	0.50055	0.113662	4.403841	1.06E-05	5.12E-05	10301.81	10932.91	10838.1	14066.86	13609.02	17677.59
25060	TRNAL-CA/	1.828974	0.499882	2.115725	0.23627	0.813223	0.862711	0	0.547412	4.174388	2.232127	0	4.019918
5722	SH3BP5L	1598.237	0.499854	0.115908	4.31251	1.61E-05	7.50E-05	1403.462	1214.159	1358.763	1886.147	1704.871	2022.019
4313	TSPAN14	3042.886	0.499768	0.097115	5.146137	2.66E-07	1.64E-06	2581.957	2578.31	2407.231	3513.368	3360.543	3815.907
9112	PIPOX	416.3062	0.499714	0.162246	3.079978	0.00207	0.00604	309.5313	401.8003	322.8194	499.9964	481.2999	482.3901
5062	SLC12A4	4699.867	0.49969	0.107375	4.65371	3.26E-06	1.71E-05	4145.334	3575.147	3967.06	5468.711	5014.075	6028.872
14537	GNRH1	101.3362	0.499397	0.260213	1.919185	0.054961	0.100512	88.90216	78.8273	84.1835	131.6955	104.8164	119.5926
6653	TRIM26	1350.029	0.499288	0.128792	3.876695	0.000106	0.000423	1014.786	1288.607	1055.424	1515.614	1527.325	1698.415
15779	LOC11962	87.79107	0.49915	0.292719	1.705218	0.088154	0.14852	83.4813	59.12048	75.83472	89.28508	115.512	103.5129
22165	CDCP1	12.07776	0.498991	0.715448	0.697452	0.48552	0.582339	11.38381	9.853413	8.348776	17.85702	14.97377	10.04979
9652	LOC11961ℓ	848.3792	0.498915	0.169355	2.945965	0.003219	0.008868	657.0087	595.0366	860.6197	1051.332	827.8358	1098.443
23736	LOC11961!	5.17512	0.498669	1.114385	0.447484	0.654526	0.733088	5.96295	3.284471	3.478657	11.16063	2.139111	5.024897
21623	LOC10322!	15.98374	0.498563	0.633897	0.786505	0.431572	0.530608	17.88885	9.853413	11.82743	17.85702	21.39111	17.08465
20652	CHADL	25.86128	0.498316	0.536862	0.928202	0.353303	0.454801	25.47806	16.42235	22.95914	15.62489	38.50399	36.17926
13139	BBOF1	199.8555	0.49822	0.228327	2.182041	0.029106	0.058881	160.9997	188.3097	144.7121	238.8376	282.3626	183.9112
12396	IL15RA	174.0219	0.497893	0.214434	2.321897	0.020239	0.043404	147.9896	154.3701	132.189	200.8914	177.5462	231.1453
20958	LOC11962:	22.7718	0.49622	0.561192	0.884224	0.376575	0.477682	17.34676	12.59047	26.43779	33.4819	25.66933	21.10457
5023	PLPBP	1277.766	0.495796	0.106023	4.676311	2.92E-06	1.55E-05	1039.722	1097.561	1046.38	1502.221	1413.952	1566.763
18347	TEX101	44.54569	0.495573	0.388954	1.274118	0.202622	0.293601	37.40396	29.56024	43.83108	58.0353	49.19955	49.24399
21522	GDF6	22.43748	0.495353	0.618565	0.80081	0.423242	0.522808	14.09425	19.15941	21.56767	31.24978	38.50399	10.04979
24071	LOC11962	3.638589	0.494927	1.255338	0.394258	0.693391	0.765809	3.252518	1.642235	4.174388	4.464254	4.278221	4.019918
4631	RPN2	18112.22	0.494709	0.100362	4.929226	8.26E-07	4.74E-06	14502.98	16679.64	13931.32	20890.48	20165.4	22503.5
10653	MMAA	257.0338	0.494636	0.183018	2.702663	0.006879	0.017166	195.1511	234.2923	208.7194	323.6584	308.0319	272.3494
9154	ATP8B1	848.085	0.494614	0.161308	3.066277	0.002167	0.006295	618.5205	875.3115	618.5052	1046.868	911.2611	1018.044
23663	LOC11962(	5.400111	0.494285	1.073998	0.460229	0.645352	0.725051	3.794605	5.474118	4.174388	2.232127	10.69555	6.029877
19031	LOC10322(	45.07453	0.493373	0.418349	1.179333	0.238266	0.332841	41.19856	29.56024	40.35242	53.57105	70.59065	35.17428
11443	TSPAN4	1288.85	0.493226	0.195158	2.52732	0.011494	0.026703	1321.064	719.2991	1174.395	1464.275	1330.527	1723.54
6271	NBEAL2	1456.042	0.493167	0.122012	4.041947	5.30E-05	0.000225	1227.826	1139.164	1268.318	1602.667	1563.69	1934.585
8911	PANX1	475.7452	0.492907	0.156916	3.141215	0.001682	0.00502	395.181	437.382	353.4315	604.9064	487.7172	575.8532
4221	MICU1	4941.965	0.492895	0.094732	5.203075	1.96E-07	1.23E-06	4244.536	4024.572	4054.722	5618.264	5437.619	6272.077
4106	MKRN1	3095.76	0.492668	0.093108	5.291373	1.21E-07	7.86E-07	2558.106	2669.18	2493.501	3537.921	3488.889	3826.962
9220	SPATA6	332.3217	0.49262	0.16153	3.049719	0.002291	0.006605	275.3799	266.5896	283.8584	412.9435	406.431	348.7279
23230	LOC11962(	9.173134	0.492321	0.937911	0.524912	0.599644	0.686248	5.96295	3.284471	13.2189	17.85702	10.69555	4.019918
7523	LOC10322:	863.2368	0.492148	0.138594	3.550998	0.000384	0.001356	700.9177	827.1393	624.7668	968.7431	1009.66	1048.194
5307	SGMS2	5948.736	0.492087	0.109017	4.513846	6.37E-06	3.19E-05	4649.475	5541.45	4644.703	6888.344	6462.253	7506.192
23302	LOC10322ℓ	6.659048	0.491804	0.957462	0.513653	0.607494	0.693084	4.878777	8.211177	3.478657	8.928508	6.417332	8.039836
6441	UBA7	1726.92	0.491731	0.124038	3.964365	7.36E-05	0.000304	1530.852	1290.25	1490.952	1821.416	1965.843	2262.209
17803	LOC11962:	64.20414	0.491583	0.35726	1.375979	0.168828	0.252436	44.45108	61.85754	52.17985	80.35657	94.12087	52.25893
5912	R3HCC1L	1109.668	0.491505	0.116869	4.205602	2.60E-05	0.000117	922.631	931.1475	918.3654	1279.009	1180.789	1426.066
8126	NIPSNAP3/	669.2583	0.491372	0.145735	3.371672	0.000747	0.002444	604.4263	474.0586	588.5887	801.3336	823.5576	723.5852
5170	WBP2	3266.878	0.491345	0.106941	4.594559	4.34E-06	2.23E-05	2544.011	2890.334	2719.614	3571.403	3660.018	4215.889
5126	SSR3	8859.024	0.491298	0.106428	4.616241	3.91E-06	2.03E-05	8008.784	6354.356	7732.358	10424.03	10276.29	10358.32
3504	EIF3H	9548.578	0.491268	0.083971	5.850431	4.90E-09	3.72E-08	7791.949	8396.202	7627.303	11189.65	11001.45	11284.91
22207	GPRIN2	28.01019	0.491245	0.7109	0.691019	0.489554	0.586067	15.7205	13.6853	39.65669	26.78552	64.17332	8.039836
6988	CGN	1144.42	0.491185	0.131401	3.738067	0.000185	0.000705	881.4324	1081.686	894.7105	1290.169	1249.241	1469.28
5308	SERINC5	1655.107	0.490659	0.108711	4.513448	6.38E-06	3.19E-05	1381.778	1441.335	1310.062	1906.236	1796.853	2094.377
23282	LOC10324	7.244294	0.490635	0.950008	0.516453	0.605538	0.691445	7.589209	3.831883	6.261582	8.928508	12.83466	4.019918
10007	LOC10323!	311.593	0.490265	0.171447	2.85957	0.004242	0.01127	254.2385	291.2231	232.3743	341.5154	374.3444	375.8623
6939	CAPN5	753.7734	0.489861	0.130242	3.761161	0.000169	0.000648	579.4903	690.8337	608.7649	933.0291	866.3398	844.1828

7783	SLC36A1	589.3358	0.489851	0.141168	3.469978	0.000521	0.001778	490.0461	515.1145	468.9229	687.4951	609.6465	764.7894
4299	SERF2	6232.261	0.489805	0.09499	5.156364	2.52E-07	1.56E-06	4897.208	5530.502	5120.583	7700.838	7296.506	6847.93
22304	LOC11962!	12.34153	0.489765	0.726132	0.674485	0.500003	0.595973	13.01007	9.306001	8.348776	8.928508	21.39111	13.06473
7800	PFKL	3896.985	0.489758	0.141344	3.465015	0.00053	0.001807	3577.77	2592.542	3559.362	4562.468	4023.667	5066.102
8279	RNASEK	1603.977	0.489509	0.147419	3.320517	0.000899	0.002885	1170.907	1659.205	1171.612	1948.647	1852.47	1821.023
18438	LOC11962(	67.38699	0.489474	0.388952	1.258444	0.208231	0.30024	62.88202	50.36189	53.57132	58.0353	126.2075	53.26391
24892	LOC11962{	2.683069	0.489204	1.833456	0.266821	0.789607	0.843312	3.252518	3.284471	0	0	8.556443	1.004979
8200	AFG1L	460.0718	0.489079	0.146173	3.345892	0.00082	0.002659	402.7702	344.322	402.1327	515.6213	551.8906	543.6939
5367	FAM102B	1692.611	0.489014	0.109138	4.480676	7.44E-06	3.69E-05	1382.32	1481.844	1366.416	1870.522	1888.835	2165.731
15143	LOC11962⁄	96.22107	0.488509	0.270341	1.807008	0.070761	0.124228	79.14461	84.84883	75.83472	131.6955	96.25998	109.5428
6620	CUNH11or	733.6217	0.488322	0.125438	3.892928	9.90E-05	0.000398	653.2141	588.4677	592.7631	823.6549	832.114	911.5164
10076	MARCHF2	655.1374	0.488259	0.171735	2.843092	0.004468	0.011788	645.6249	448.8777	544.7577	752.2268	660.9852	878.3521
7825	RUNX2	1085.696	0.488114	0.14111	3.459112	0.000542	0.001841	821.2608	1082.781	807.0484	1225.438	1296.301	1281.349
5572	ZNRF3	1038.028	0.487509	0.111191	4.384434	1.16E-05	5.55E-05	867.3382	836.4453	891.9276	1218.741	1144.424	1269.289
17815	LOC10323⁄	61.81075	0.487129	0.354413	1.374466	0.169297	0.252639	39.03022	67.87906	47.30973	71.42806	74.86887	70.34856
10701	KIF9	231.6917	0.487067	0.180943	2.691833	0.007106	0.017654	195.6932	182.2881	199.6749	279.0159	280.2235	253.2548
23658	LOC10323⁄	5.173804	0.487064	1.056402	0.46106	0.644756	0.724526	4.878777	3.284471	4.87012	6.696381	4.278221	7.034856
22580	LOC11962:	11.73559	0.48655	0.775539	0.62737	0.530417	0.624496	8.673382	12.59047	8.348776	4.464254	19.252	17.08465
18425	LOC11962⁄	54.40438	0.486369	0.385615	1.261281	0.207208	0.298975	38.48813	43.79295	52.17985	75.89232	74.86887	41.20416
9653	FOXP4	358.1252	0.486368	0.165103	2.94584	0.003221	0.00887	258.5752	324.6152	310.9919	439.729	412.8484	401.9918
19512	LOC10323!	46.76216	0.486258	0.442377	1.099192	0.271684	0.370168	44.99317	22.9913	48.00546	55.80317	70.59065	38.18922
18437	SCUBE1	51.94691	0.486248	0.386378	1.258479	0.208219	0.300238	28.73058	49.81448	51.48412	64.73168	55.61688	61.30375
23421	LOC10322:	6.04637	0.486155	0.980397	0.495876	0.619982	0.703677	5.420864	4.926706	4.87012	4.464254	8.556443	8.039836
17212	CELSR3	79.05246	0.485974	0.331433	1.466284	0.142571	0.22021	55.29281	67.33165	73.05179	109.3742	106.9555	62.30873
4187	LOC10323(	7500.638	0.48593	0.092824	5.234955	1.65E-07	1.05E-06	6146.717	6589.196	6017.381	8366.012	8470.878	9413.643
5080	SAMD4B	2243.239	0.48592	0.104646	4.643473	3.43E-06	1.79E-05	1707.572	2021.044	1880.562	2573.642	2571.211	2705.405
4785	AK4	3200.245	0.485654	0.100721	4.821776	1.42E-06	7.91E-06	2633.456	2738.154	2633.343	3772.295	3386.212	4038.008
12440	CDKN1C	177.8435	0.485552	0.209944	2.312765	0.020736	0.044313	163.168	134.1159	146.7993	191.9629	231.024	199.9909
23694	LOC10322!	5.800681	0.485315	1.068433	0.454231	0.649663	0.728931	5.96295	1.642235	6.957314	8.928508	4.278221	7.034856
10911	SLC66A2	296.8387	0.485242	0.183173	2.649095	0.008071	0.019665	268.8748	204.732	269.248	357.1403	327.2839	353.7528
5633	SH2B1	1644.536	0.484997	0.111296	4.357714	1.31E-05	6.20E-05	1456.044	1278.207	1381.723	1921.861	1771.184	2058.198
19189	LOC11962:	54.44634	0.484888	0.419558	1.15571	0.2478	0.343309	34.69353	68.97389	32.00364	53.57105	79.14709	58.28881
20295	LOC10323⁄	26.12176	0.484686	0.495352	0.978468	0.327843	0.42947	20.0572	19.70683	25.04633	35.71403	32.08666	24.11951
12917	ETFBKMT	172.1049	0.48447	0.218399	2.218277	0.026536	0.054615	165.8784	122.6202	141.9292	209.8199	190.3808	202.0009
11670	NAGLU	1164.632	0.484427	0.195511	2.477747	0.013221	0.030119	1154.644	671.1269	1090.211	1366.062	1120.894	1584.853
6029	ITPRIPL2	1782.873	0.484325	0.116632	4.152593	3.29E-05	0.000145	1476.101	1655.373	1329.543	2015.611	2025.738	2194.875
18390	LOC10324⁄	65.08008	0.48427	0.382449	1.266233	0.20543	0.296958	39.03022	72.80577	49.39693	89.28508	87.70354	52.25893
9394	ZNF836	364.1942	0.484158	0.160935	3.008409	0.002626	0.007432	278.0903	341.0376	290.8157	437.4969	425.683	412.0416
15960	LOC11962⁄	98.26328	0.484146	0.289282	1.673615	0.094206	0.156922	85.10756	64.04718	98.09812	100.4457	111.2338	130.6473
10261	CDC42EP4	429.5979	0.483873	0.172897	2.798616	0.005132	0.013297	397.8914	312.0247	368.7376	453.1218	459.9088	585.903
4466	GPI	21687.28	0.483699	0.096121	5.032175	4.85E-07	2.89E-06	18356.67	17786.5	18120.32	24997.59	22963.35	27899.24
5861	SFMBT1	1794.152	0.48365	0.114158	4.236662	2.27E-05	0.000103	1427.855	1671.248	1390.071	1970.968	2171.197	2133.571
25243	LOC11962⁄	1.807082	0.483625	2.318683	0.208577	0.834778	0.879158	0	1.642235	2.782925	0	6.417332	0
7009	TRAF3IP2	735.6083	0.483618	0.129666	3.729724	0.000192	0.000727	589.79	678.7906	571.8912	821.4227	883.4527	868.3023
22016	LOC10323⁄	17.09204	0.483611	0.668704	0.723207	0.469553	0.566974	17.88885	11.49565	12.52316	33.4819	17.11289	10.04979
10364	GDF1	254.3552	0.483578	0.17439	2.772974	0.005555	0.014248	197.8615	216.2277	223.3298	292.4086	288.7799	307.5237
19610	LOC11962!	43.68319	0.483529	0.446038	1.084053	0.278341	0.377343	27.6464	52.00412	28.52499	53.57105	64.17332	36.17926
5797	CD70	842.3498	0.483012	0.112983	4.275084	1.91E-05	8.76E-05	706.3385	705.6138	695.0356	1017.85	960.4607	968.8002
17128	LOC11961!	69.83633	0.482965	0.327032	1.476813	0.139726	0.216874	69.38706	49.81448	54.26705	91.51721	87.70354	66.32864
5274	TEX264	1208.722	0.482867	0.106467	4.535362	5.75E-06	2.90E-05	1018.58	1026.945	982.3727	1386.151	1351.918	1486.365
18152	S1PR5	56.76119	0.482279	0.36747	1.312431	0.189375	0.277354	37.94605	54.19377	48.7012	69.19594	81.28621	49.24399
10682	ILVBL	957.7905	0.482192	0.178868	2.695799	0.007022	0.017476	902.0317	598.8685	900.9721	1037.939	990.4082	1316.523
5441	ERI1	996.6405	0.48184	0.108456	4.442732	8.88E-06	4.34E-05	825.5975	855.6047	814.0057	1185.259	1131.59	1167.786
24806	LOC11962:	1.814585	0.481779	1.723131	0.279595	0.779788	0.835712	2.168345	1.642235	0.695731	2.232127	2.139111	2.009959
8690	MAST3	448.3044	0.481636	0.150226	3.206081	0.001346	0.004116	336.0935	411.1063	374.9992	504.4607	547.6123	515.5545
19659	FZD9	34.60328	0.481584	0.447225	1.076828	0.281557	0.380751	33.60935	20.25424	32.69937	37.94616	44.92132	38.18922
9002	ACOT13	1071.279	0.481096	0.154401	3.115887	0.001834	0.005416	766.5101	1100.845	814.7014	1169.635	1351.918	1224.065
5621	LMNA	19423.22	0.480653	0.110174	4.362665	1.28E-05	6.08E-05	17450.3	14630.13	16577.89	22191.81	20454.18	25235.03
5837	SPTAN1	23160.3	0.480368	0.112908	4.25451	2.10E-05	9.54E-05	18358.84	21592.66	18073.71	25952.94	24638.28	30345.36
20306	LOC10324:	28.92789	0.480321	0.4912	0.977852	0.328148	0.429617	28.73058	23.53871	19.48048	46.87467	27.80844	27.13445
4723	SH3BGRL	1923.214	0.479684	0.098549	4.867447	1.13E-06	6.36E-06	1672.336	1510.857	1637.752	2232.127	2196.867	2289.343
16547	LOC10324:	73.63768	0.479645	0.306274	1.566062	0.117334	0.188513	59.08741	66.23683	59.8329	93.74933	68.45154	94.46807
21178	LOC11962⁄	21.30468	0.479579	0.561498	0.854107	0.393046	0.493401	15.7205	14.23271	22.95914	29.01765	27.80844	18.08963
9126	TRIM21	742.8595	0.47902	0.155861	3.073373	0.002117	0.006166	555.0964	755.4283	551.0192	904.0114	819.2794	872.3222
7251	USP12	3466.842	0.478956	0.131163	3.651602	0.000261	0.000955	2595.51	3514.384	2580.468	3910.686	4034.363	4165.64
25394	LOC11962⁄	1.246996	0.478874	2.623654	0.182522	0.855173	0.895252	1.626259	0	1.391463	4.464254	0	0
5839	CTNNB1	38431.37	0.478556	0.112508	4.253524	2.10E-05	9.58E-05	35096.84	28765.94	32489.26	43292.1	40705.14	50238.92
19499	TMEM202	39.75028	0.477742	0.433903	1.101034	0.270882	0.369299	30.89892	38.31883	29.22072	46.87467	62.03421	31.15436
7921	CCDC97	641.0596	0.477489	0.139096	3.432791	0.000597	0.002005	531.7867	591.2048	487.012	698.6557	733.715	803.9836
5845	DNAJC13	7047.017	0.477035	0.112231	4.250478	2.13E-05	9.70E-05	5584.574	6729.881	5366.176	7897.265	7880.484	8823.72
10114	PABPC5	293.2468	0.477034	0.168286	2.834667	0.004587	0.012057	248.8176	264.3999	222.634	337.0512	335.8404	350.7378
22550	LOC10324⁄	11.82075	0.477011	0.754857	0.631923	0.527437	0.621808	9.215468	8.758589	11.1317	13.39276	21.39111	7.034856
19749	LOC10321!	36.53773	0.476948	0.449759	1.060453	0.288939	0.388957	33.06727	24.08612	33.39511	51.33892	49.19955	28.13943
4255	ILK	5179.083	0.476729	0.091923	5.186186	2.15E-07	1.34E-06	4451.613	4043.184	4502.773	5852.637	5921.058	6303.231

20291	LOC11962(	33.39623	0.476653	0.486792	0.979172	0.327495	0.429079	39.5723	19.15941	24.3506	37.94616	49.19955	30.14938
21774	PADI4	18.2923	0.476649	0.623968	0.7639	0.444927	0.543234	16.80468	10.94824	17.39328	29.01765	23.53022	12.05975
9636	KRBOX4	303.1172	0.476412	0.161394	2.951861	0.003159	0.008714	258.0331	247.9776	253.2462	350.4439	374.3444	334.6582
7287	ELF1	1439.985	0.476339	0.130921	3.638357	0.000274	0.001001	1141.092	1369.077	1106.909	1506.686	1659.95	1856.197
21579	LOC11961{	18.81467	0.476098	0.601107	0.792036	0.428339	0.527708	13.01007	13.6853	20.87194	13.39276	27.80844	24.11951
11638	LOC10323<	307.0779	0.475979	0.191524	2.48522	0.012947	0.029575	226.5921	296.6972	244.8974	421.872	335.8404	316.5685
7686	LOC10324(	564.3866	0.47591	0.136087	3.497116	0.00047	0.001627	465.1101	470.2267	484.9248	631.6919	611.7857	722.5802
23741	LOC11961!	5.433589	0.475869	1.066256	0.446299	0.655381	0.733881	5.96295	3.284471	4.174388	4.464254	10.69555	4.019918
5044	GLUL	7535.804	0.47558	0.101941	4.665247	3.08E-06	1.62E-05	5928.257	7063.802	5924.153	8761.098	8449.487	9088.029
18877	DHRS12	42.0708	0.475438	0.39387	1.207095	0.227396	0.320248	31.9831	37.224	36.87376	53.57105	38.50399	54.26889
8157	ZBTB34	637.1278	0.47521	0.141292	3.36331	0.00077	0.00251	520.945	572.0453	510.6668	669.6381	712.3239	837.1479
19955	LOC10321{	40.40328	0.475179	0.458245	1.036953	0.299758	0.399352	38.48813	29.56024	32.00364	44.64254	70.59065	27.13445
19630	CUNH6orf!	43.52635	0.47501	0.439716	1.080265	0.280024	0.379238	35.7777	28.46541	43.83108	51.33892	70.59065	31.15436
5655	WDR45	1663.917	0.474851	0.109299	4.3445	1.40E-05	6.56E-05	1484.775	1303.388	1392.158	1977.665	1790.436	2035.083
23951	LOC10321(	5.170598	0.474777	1.144573	0.414807	0.678283	0.752877	2.168345	8.758589	2.087194	6.696381	4.278221	7.034856
25308	LOC11961!	1.192158	0.474764	2.399853	0.197831	0.843178	0.885723	1.084173	1.094824	0.695731	0	4.278221	0
5992	AHNAK	102408	0.474561	0.113865	4.16777	3.08E-05	0.000136	79547.92	95382.13	82222.23	112764.8	109096.8	135434
23057	LOC11962<	8.177353	0.474505	0.858663	0.552609	0.580531	0.669363	8.131296	6.568942	5.565851	8.928508	12.83466	7.034856
18055	CUNH16or	57.41257	0.47431	0.355761	1.333225	0.182458	0.26866	46.07734	55.83601	41.04815	66.96381	81.28621	53.26391
23357	LOC10323(	6.701517	0.474218	0.938684	0.505194	0.613422	0.698179	4.336691	4.926706	7.653045	6.696381	8.556443	8.039836
16533	LOC10321{	75.4955	0.473786	0.301902	1.56934	0.116569	0.187434	57.46116	62.95236	68.18167	93.74933	96.25998	74.36848
21448	KLHL41	19.11279	0.473772	0.583633	0.811764	0.416927	0.516785	12.46799	18.612	16.69755	17.85702	29.94755	19.09461
11041	PRICKLE3	231.6609	0.473751	0.180899	2.618863	0.008822	0.021243	190.8144	199.8053	191.3261	247.7661	290.9191	269.3345
6621	RAB11FIP5	5367.55	0.47354	0.121658	3.892374	9.93E-05	0.000399	4850.047	3976.947	4662.792	6107.099	5525.323	7083.095
7609	RECQL4	1470.728	0.473395	0.134383	3.522721	0.000427	0.001492	1342.206	1045.557	1310.062	1763.38	1523.047	1840.117
9032	MYD88	509.7162	0.473232	0.152419	3.104814	0.001904	0.005604	364.8241	457.0889	460.5742	582.5851	571.1425	622.0823
7952	CEP70	693.6013	0.473096	0.138161	3.424236	0.000617	0.002061	553.4702	660.1786	529.4516	767.8517	823.5576	827.0981
6611	LOC10323<	5158.318	0.472964	0.121312	3.898737	9.67E-05	0.000389	4807.222	3715.284	4442.941	5939.69	5461.15	6583.621
5088	EEF1A2	5985.57	0.472926	0.101935	4.639494	3.49E-06	1.82E-05	5172.046	4749.345	5124.062	7035.664	6254.76	7577.545
22002	LOC10322:	15.46937	0.472783	0.650408	0.726902	0.467286	0.564622	16.26259	12.59047	10.43597	11.16063	19.252	23.11453
20923	LOC11962<	26.84171	0.472765	0.532773	0.887366	0.374882	0.476306	21.68345	20.25424	24.3506	40.17829	38.50399	16.07967
4652	STX3	3561.73	0.472366	0.096082	4.916304	8.82E-07	5.04E-06	2887.694	3219.329	2846.237	3982.115	4164.848	4270.158
22851	TPO	10.61153	0.472195	0.806751	0.585304	0.558343	0.649629	9.215468	12.04306	4.87012	13.39276	17.11289	7.034856
5400	MCPH1	1218.251	0.472176	0.105778	4.463854	8.05E-06	3.96E-05	1021.833	1070.19	969.8495	1415.169	1405.396	1427.071
5964	GYS1	1463.177	0.472021	0.112904	4.18073	2.91E-05	0.00013	1202.348	1309.409	1170.22	1660.702	1587.22	1849.162
5535	MANEAL	1459.678	0.471932	0.107327	4.397151	1.10E-05	5.27E-05	1276.071	1179.125	1218.226	1654.006	1608.611	1822.028
5175	CRIM1	17152.26	0.471463	0.10268	4.591585	4.40E-06	2.26E-05	14210.25	15565.65	13352.48	19475.31	18428.44	21881.42
18155	LOC11962(	63.9789	0.470877	0.358961	1.311777	0.189595	0.277631	48.24569	64.04718	48.7012	49.10679	98.39909	75.37346
8429	ABHD16A	1643.243	0.470416	0.14366	3.274509	0.001058	0.003338	1581.808	1138.617	1416.509	1881.683	1711.289	2129.551
5550	DAPK1	2881.746	0.470209	0.107059	4.392039	1.12E-05	5.38E-05	2425.294	2443.099	2387.75	3136.138	3129.519	3768.673
5981	BCL10	1464.206	0.470144	0.112708	4.171365	3.03E-05	0.000135	1160.065	1340.064	1186.222	1645.078	1642.837	1810.973
5128	LAMC1	17552.42	0.470083	0.10187	4.614524	3.94E-06	2.04E-05	14144.12	16103.76	13911.15	19758.79	19102.26	22294.46
24502	LOC11962:	3.606164	0.469875	1.421475	0.330554	0.740981	0.803975	2.168345	3.284471	3.478657	0	10.69555	2.009959
7623	SNX27	1298.943	0.46982	0.133503	3.51916	0.000433	0.00151	1010.449	1278.754	980.2855	1433.026	1495.238	1595.907
5658	PSME4	6231.311	0.469732	0.108154	4.343158	1.40E-05	6.60E-05	4972.558	5857.306	4851.335	7031.2	6853.711	7821.755
5326	GJB3	1945.436	0.469444	0.104185	4.50585	6.61E-06	3.30E-05	1611.623	1648.257	1640.535	2238.823	2079.216	2454.16
24223	LOC10323<	5.51884	0.469393	1.266403	0.37065	0.710898	0.780218	3.794605	2.737059	6.957314	8.928508	10.69555	0
9370	PKDCC	1361.879	0.469116	0.155642	3.014059	0.002578	0.007313	1347.627	930.6001	1152.827	1546.864	1390.422	1802.933
15739	LOC11961{	95.61828	0.469052	0.27402	1.711742	0.086944	0.146859	78.60252	87.03848	76.53045	98.21359	102.6773	130.6473
6040	ORC4	2393.736	0.469047	0.113103	4.14707	3.37E-05	0.000148	2126.063	1746.791	2150.506	2756.677	2874.965	2707.415
21176	LOC10323(	23.48376	0.46868	0.548569	0.854369	0.3929	0.493259	11.9259	24.63353	22.2634	29.01765	29.94755	23.11453
20443	LOC10324;	25.57268	0.468614	0.490444	0.955488	0.339331	0.441281	22.76763	22.9913	18.78475	26.78552	29.94755	32.15934
15110	DDX60	105.0523	0.468449	0.258606	1.811443	0.070072	0.123287	95.94929	90.32295	77.22618	122.767	130.4858	113.5627
7376	FCHSD2	938.2052	0.468388	0.129808	3.608307	0.000308	0.001111	754.5842	894.4709	713.1247	1046.868	1101.642	1118.542
14638	HORMAD2	123.0676	0.46836	0.246597	1.899296	0.057526	0.104476	100.8281	96.89189	110.6213	167.4095	139.0422	123.6125
8078	TMEM144	559.0926	0.468248	0.138135	3.389773	0.0007	0.002302	479.7464	429.7183	496.0565	689.7272	650.2896	609.0176
11868	RAB32	1113.391	0.468231	0.192461	2.432868	0.01498	0.033553	1143.26	619.6702	1040.814	1209.813	1341.222	1325.568
6656	LOC10321!	2399.637	0.467901	0.120774	3.874202	0.000107	0.000427	2160.756	1826.713	2060.061	2732.123	2485.647	3132.521
5918	PARP14	3787.272	0.467706	0.111296	4.202348	2.64E-05	0.000119	2997.738	3637.004	2901.896	4397.29	4280.36	4509.343
11917	DHDH	329.8617	0.467677	0.193169	2.42108	0.015474	0.034521	306.8209	232.65	294.2944	379.4616	318.7275	447.2159
7184	IMPDH1	1154.897	0.467663	0.127401	3.670804	0.000242	0.000895	1047.853	851.7728	1010.898	1374.99	1227.85	1416.016
22696	LOC11962<	13.18773	0.467659	0.76825	0.608733	0.542701	0.635694	8.673382	16.96977	6.957314	26.78552	10.69555	9.044815
16510	LOC10324{	106.7652	0.467115	0.296875	1.573436	0.115618	0.186172	99.74389	84.84883	82.79203	100.4457	173.268	99.49297
13323	ZNF57	154.1467	0.466933	0.217822	2.143642	0.032062	0.063992	121.9694	142.8745	123.8402	165.1774	186.1026	184.9162
22595	LOC10323;	14.25408	0.466494	0.745956	0.625364	0.531732	0.625629	9.757555	10.40082	15.30609	8.928508	32.08666	9.044815
21945	RGCC	16.44079	0.466463	0.632981	0.73693	0.461165	0.558652	13.01007	19.15941	9.044508	20.08914	19.252	18.08963
11216	LOC10323(	295.4631	0.466449	0.180954	2.577718	0.009946	0.023572	224.9658	291.7705	227.5042	345.9797	335.8404	346.7179
20458	LOC11961{	28.66256	0.466438	0.489612	0.952669	0.340758	0.442812	29.81475	16.42235	25.74206	33.4819	36.36488	30.14938
21729	LOC10324:	17.8996	0.466351	0.602856	0.77357	0.439185	0.537334	15.7205	15.32753	13.91463	13.39276	29.94755	19.09461
25236	FAM167B	1.545522	0.466104	2.224209	0.209559	0.834012	0.878559	0	0.547412	3.478657	2.232127	0	3.014938
24445	LOC11962(	3.362108	0.46575	1.377942	0.338004	0.73536	0.799736	3.794605	3.831883	0.695731	6.696381	2.139111	3.014938
5500	PRKX	1880.59	0.465674	0.1056	4.409767	1.03E-05	5.00E-05	1525.973	1719.968	1495.127	2145.074	2124.137	2273.264
7314	IFNLR1	607.4269	0.465299	0.128192	3.629712	0.000284	0.001031	532.8709	496.5025	503.0138	714.2806	665.2634	732.63
5858	PNPLA8	928.2043	0.465089	0.109739	4.238148	2.25E-05	0.000102	789.2778	787.1782	763.2173	1093.742	1052.442	1083.368

7324	LZTR1	2071.556	0.464878	0.128204	3.626082	0.000288	0.001045	1882.124	1504.288	1840.905	2171.86	2350.883	2679.275
20665	DHFR2	28.26626	0.464837	0.501533	0.926833	0.354013	0.455429	23.8518	22.9913	23.65487	26.78552	49.19955	23.11453
7781	LOC103234	629.2384	0.464721	0.133918	3.470198	0.00052	0.001777	550.7598	507.9982	530.843	729.9055	660.9852	794.9388
15967	TSTD3	114.7131	0.464517	0.277874	1.671683	0.094587	0.157487	93.23886	113.3142	80.70484	167.4095	124.0684	109.5428
9468	NFKBIE	888.3928	0.464411	0.155118	2.993921	0.002754	0.007734	836.4393	608.7219	798.6996	964.2789	960.4607	1161.756
8345	MFSD5	741.233	0.464142	0.140718	3.298376	0.000972	0.003098	659.7191	557.2652	656.0747	852.6725	767.9407	953.7255
15721	LOC119625	110.4239	0.464123	0.27064	1.714908	0.086362	0.146043	83.4813	110.5772	82.79203	131.6955	145.4595	108.5378
7758	PLGRKT	1890.165	0.463791	0.133415	3.476303	0.000508	0.001742	1662.037	1294.629	1810.293	2220.966	2205.423	2147.641
20025	AIFM3	40.99829	0.46359	0.453053	1.023259	0.306185	0.406489	28.73058	22.9913	51.48412	49.10679	53.47777	40.19918
14399	CCDC13	126.7217	0.463561	0.238545	1.943288	0.051981	0.095974	104.0806	118.7884	96.70666	156.2489	134.764	149.7419
21664	LOC119624	19.21981	0.463469	0.593112	0.781419	0.434556	0.533266	16.26259	21.34906	10.43597	26.78552	21.39111	19.09461
21072	LOC103217	25.37681	0.463423	0.532861	0.869688	0.384471	0.485059	24.39389	24.08612	16.00182	33.4819	17.11289	37.18424
15889	CMPK2	109.7601	0.463404	0.274816	1.686238	0.09175	0.153513	111.6698	76.09024	90.44508	113.8385	119.7902	146.727
22077	TMEM249	18.09776	0.463092	0.649644	0.71284	0.475945	0.57313	12.46799	10.94824	21.56767	29.01765	23.53022	11.05477
6595	SOGA1	1490.402	0.46309	0.118625	3.903822	9.47E-05	0.000382	1209.937	1356.486	1197.354	1732.131	1555.133	1891.371
9892	BRI3	2845.895	0.462952	0.160404	2.886169	0.0039	0.010481	2843.785	1761.024	2577.685	3178.549	3080.319	3634.006
5257	ANXA11	4217.501	0.462817	0.101744	4.548838	5.39E-06	2.73E-05	3450.38	3711.452	3482.136	5084.785	4374.481	5201.774
24658	TRNAM-CA	2.777016	0.4628	1.510146	0.30646	0.759254	0.818556	1.626259	0.547412	4.87012	4.464254	2.139111	3.014938
15847	LOC103214	142.1033	0.462756	0.273545	1.691698	0.090703	0.152165	93.78094	133.5685	128.7103	189.7308	188.2417	118.5876
8612	LOC119627	1488.529	0.462746	0.143454	3.225743	0.001256	0.003879	1482.606	1048.294	1222.4	1669.631	1895.252	1612.992
7458	PDP2	979.5152	0.462579	0.129241	3.579202	0.000345	0.001229	806.6245	923.4837	742.3454	1053.564	1172.233	1178.841
16336	LOC119625	98.19968	0.462455	0.289287	1.598604	0.109909	0.178864	71.01331	103.4608	72.35606	109.3742	130.4858	102.5079
6253	CALR	28223.21	0.462234	0.114133	4.049974	5.12E-05	0.000218	22276.5	27643.2	21303.29	31990.84	31323	34802.44
8483	ASMTL	717.4961	0.462181	0.141799	3.259404	0.001116	0.003499	591.4162	611.459	613.6351	743.2983	774.3581	970.8102
19209	LOC103235	69.6135	0.462157	0.401244	1.151811	0.249399	0.345164	59.08741	47.07742	67.48594	120.5349	81.28621	42.20914
22587	LOC103231	11.6097	0.462151	0.738112	0.626126	0.531232	0.625245	13.55216	8.758589	6.957314	15.62489	10.69555	14.06971
21138	LOC119628	36.19334	0.461572	0.536834	0.859804	0.389897	0.490369	29.27266	23.53871	37.56949	78.12444	23.53022	25.12449
20488	LOC103229	27.5771	0.461547	0.486346	0.949009	0.342616	0.444575	22.22554	19.15941	28.52499	35.71403	25.66933	34.1693
9719	CLMN	540.0079	0.461455	0.157762	2.925007	0.003444	0.009422	439.09	531.5369	392.3925	654.0132	581.8381	641.1769
5025	ZSWIM8	5152.152	0.461426	0.098698	4.675144	2.94E-06	1.55E-05	4317.718	4330.575	4365.019	5707.549	5570.244	6621.81
18967	LOC119619	657920.7	0.461351	0.38743	1.190799	0.233732	0.32761	354745.1	885162.2	420928.6	764369.6	790795	731523.6
21967	HOXA11	15.5347	0.461304	0.629036	0.73335	0.463345	0.560751	11.9259	13.13788	13.91463	15.62489	23.53022	15.07469
9298	SH3BP2	479.3207	0.46116	0.152067	3.032604	0.002425	0.006932	385.9655	416.5804	410.4815	578.1209	472.7435	612.0325
6610	ATP7A	1347.023	0.46106	0.118255	3.898871	9.66E-05	0.000389	1110.735	1256.31	1034.553	1595.971	1469.569	1615.002
13060	ANKRD9	157.9617	0.460907	0.210009	2.1947	0.028185	0.057374	128.4745	129.7366	141.2335	178.5702	181.8244	187.9312
21974	LOC103215	19.69795	0.460677	0.629482	0.731836	0.464269	0.56169	17.34676	20.25424	11.82743	11.16063	38.50399	19.09461
24822	MTNR1A	2.440929	0.46065	1.66117	0.277305	0.781546	0.836998	1.626259	2.189647	2.087194	4.464254	4.278221	0
8021	PPP1R2	4021.365	0.460408	0.135162	3.406352	0.000658	0.002182	2911.546	4139.528	3101.57	4830.323	4742.408	4402.815
8092	CARMIL1	3228.325	0.460336	0.13608	3.382826	0.000717	0.002357	2458.362	3311.841	2383.576	3816.937	3471.777	3927.46
25172	LOC103232	2.393001	0.460181	2.108097	0.218292	0.827201	0.873601	0	4.926706	1.391463	0	0	8.039836
10150	LOC103216	299.9874	0.460023	0.162804	2.825624	0.004719	0.01236	252.6122	262.2103	244.2017	341.5154	331.5622	367.8225
8252	MTUS1	895.7471	0.459959	0.138163	3.329099	0.000871	0.002807	671.1029	879.6908	712.4289	1002.225	1041.747	1067.288
7510	MOSPD3	595.0171	0.459922	0.129368	3.555136	0.000378	0.001337	470.531	514.5671	516.9284	716.5128	680.2372	671.3263
4996	TXNL1	4505.253	0.459896	0.098057	4.690079	2.73E-06	1.45E-05	3597.827	4123.653	3653.981	5287.909	5422.646	4945.504
22847	LOC103240	11.91774	0.459894	0.785248	0.585667	0.5581	0.64941	7.589209	12.04306	9.740239	17.85702	19.252	5.024897
9635	STARD10	496.932	0.459891	0.155785	2.952087	0.003156	0.008709	447.7633	372.7874	436.2236	620.5313	496.2737	608.0126
10708	CHMP4B	2729.141	0.459823	0.17097	2.689494	0.007156	0.017765	1906.518	3101.635	1885.432	3111.585	3114.545	3255.129
7423	UBXN2B	2191.913	0.459771	0.128035	3.59098	0.000329	0.00118	1677.215	2197.311	1662.102	2526.768	2511.316	2576.767
3979	LOC103247	4363.197	0.459518	0.085264	5.389378	7.07E-08	4.72E-07	3656.915	3819.84	3544.751	5095.946	5024.771	5036.957
9168	DNAJB9	542.7662	0.459493	0.150044	3.062385	0.002196	0.006367	479.7464	392.4943	498.8394	669.6381	596.8119	619.0674
24981	LOC119622	1.829659	0.459091	1.843294	0.24906	0.803314	0.854894	0.542086	2.189647	2.087194	0	2.139111	4.019918
5646	PLXNB2	21743.1	0.459036	0.105542	4.349296	1.37E-05	6.43E-05	18849.43	17010.82	19086	24698.49	22655.32	28158.52
5741	SRPRB	3336.337	0.45852	0.106501	4.305303	1.67E-05	7.72E-05	2997.196	2533.969	2905.374	3705.331	3730.609	4145.54
10525	ZNF266	364.7996	0.45829	0.167335	2.73876	0.006167	0.015579	330.1306	262.7577	329.7767	395.0865	444.935	426.1113
14976	LOC119628	115.6268	0.458156	0.24918	1.838656	0.065966	0.117097	100.286	99.08154	93.228	111.6063	151.8769	137.6822
23747	LOC119618	8.774606	0.45798	1.029411	0.444895	0.656395	0.734841	13.01007	3.284471	5.565851	2.232127	23.53022	5.024897
5591	ERF	1760.884	0.457942	0.104633	4.376668	1.21E-05	5.73E-05	1383.404	1568.882	1499.997	2107.128	1931.617	2074.278
6296	HERC1	6342.365	0.457741	0.11363	4.028335	5.62E-05	0.000237	5175.299	6001.823	4861.075	7136.11	6793.815	8086.065
24496	TRNAA-CG	3.07843	0.457493	1.37962	0.331608	0.740185	0.803308	1.084173	3.284471	3.478657	4.464254	2.139111	4.019918
23917	LOC119623	5.542836	0.457229	1.087334	0.420505	0.674117	0.749316	5.420864	1.642235	6.957314	8.928508	4.278221	6.029877
12614	PXMP4	405.6233	0.457174	0.200534	2.279775	0.022621	0.047676	392.4705	246.8827	388.2181	446.4254	449.2132	510.5296
9083	POMGNT2	700.1942	0.457103	0.147992	3.088703	0.00201	0.005884	611.4734	549.054	615.7223	754.4589	718.7412	951.7156
24145	LOC119619	5.224652	0.457099	1.191324	0.38369	0.701208	0.772073	3.252518	5.474118	4.87012	6.696381	0	11.05477
24792	LOC103218	2.794519	0.456714	1.615653	0.282681	0.777422	0.833646	3.252518	2.189647	1.391463	8.928508	0	1.004979
6682	PLD3	5066.816	0.456697	0.118184	3.864287	0.000111	0.000443	4568.704	3797.943	4453.377	5899.512	5168.091	6513.272
12944	LOC103221	430.6121	0.456387	0.206095	2.21445	0.026798	0.055039	444.5108	272.0637	375.6949	459.8182	440.6568	590.9279
5834	ZCCHC24	2356.844	0.456251	0.107195	4.256277	2.08E-05	9.47E-05	2008.972	2006.812	1951.526	2729.891	2445.003	2998.859
9626	SHPK	429.799	0.456247	0.154471	2.953612	0.003141	0.008673	398.4335	345.4169	345.7785	488.8358	455.6306	544.6989
6993	LRRC8A	1470.639	0.45587	0.122018	3.736082	0.000187	0.000711	1207.226	1361.413	1154.218	1776.773	1510.212	1813.988
6415	BPHL	885.4282	0.455779	0.114704	3.97353	7.08E-05	0.000293	774.6414	739.5534	727.0393	1049.1	969.0171	1053.218
10475	MMP24OS	363.8558	0.455713	0.1657	2.750231	0.005955	0.015114	269.4169	339.3953	313.7748	404.015	404.2919	452.2408
5869	LRCH3	1656.662	0.455591	0.107687	4.23071	2.33E-05	0.000106	1341.122	1516.878	1334.413	1816.951	1982.956	1947.65
6180	NDUFB9	5519.736	0.455215	0.11141	4.085939	4.39E-05	0.000189	4893.414	3980.779	5093.449	6435.222	6500.757	6214.793
15935	SLC8A2	134.0139	0.455118	0.271146	1.6785	0.09325	0.155595	88.36008	135.7581	113.4042	149.5525	190.3808	126.6274

16504	TRNAK-CU	80.46345	0.454964	0.288927	1.574664	0.115334	0.185782	60.17159	70.06871	73.05179	91.51721	100.5382	87.43321
8713	PTPRA	8492.674	0.454944	0.14222	3.198869	0.00138	0.00421	6205.805	9078.825	6209.402	9520.022	9857.022	10084.97
9221	PRR12	534.5661	0.454765	0.149133	3.049385	0.002293	0.006611	412.5277	489.9336	454.3126	587.0494	571.1425	692.4309
18860	LOC10323	48.76907	0.454711	0.375527	1.210863	0.225948	0.318496	46.61943	36.12918	41.04815	64.73168	42.78221	61.30375
16956	MBOAT7	89.50454	0.4547	0.303429	1.498536	0.133994	0.210087	76.43418	57.47824	91.83654	111.6063	111.2338	88.43819
12702	P3H3	318.12	0.454592	0.201301	2.258273	0.023929	0.050082	291.1004	201.995	313.7748	352.6761	357.2315	391.942
21161	LOC11962	22.89303	0.454445	0.530489	0.856653	0.391637	0.492028	22.76763	18.612	16.00182	26.78552	32.08666	21.10457
12240	LOC10323	196.3488	0.454441	0.192649	2.358911	0.018329	0.039809	166.9626	165.8658	163.4969	247.7661	213.9111	220.0905
24695	CRYGD	3.379631	0.454427	1.514269	0.300096	0.764104	0.822583	0.542086	0.547412	7.653045	2.232127	4.278221	5.024897
17263	LOC10321	76.49349	0.45441	0.311943	1.456709	0.145197	0.223614	55.8349	78.8273	59.8329	84.82083	79.14709	100.4979
8174	CCDC106	627.6161	0.454279	0.135427	3.354434	0.000795	0.002587	553.4702	485.5543	553.1064	700.8879	688.7936	783.884
4982	ATP6AP2	7176.412	0.453707	0.096591	4.697215	2.64E-06	1.41E-05	6460.585	5494.372	6219.838	8211.995	8040.917	8630.764
19420	LOC11961	58.74454	0.453705	0.406921	1.11497	0.264863	0.362584	65.05036	31.20247	51.48412	62.49956	91.98176	50.24897
13148	LOC10323	176.8389	0.453607	0.207956	2.181263	0.029164	0.058969	148.5317	137.9478	162.8011	183.0344	209.6328	219.0855
8203	LOC10324	493.9938	0.453308	0.135512	3.345155	0.000822	0.002665	428.2482	423.6967	397.9583	595.9779	558.3079	559.7736
4626	CTBP2	5569.827	0.453186	0.091893	4.931666	8.15E-07	4.69E-06	4809.39	4741.134	4561.911	6191.92	6169.195	6945.413
21172	HTRA3	22.65541	0.453148	0.530129	0.854788	0.392668	0.493061	23.30971	14.78012	19.48048	24.5534	25.66933	28.13943
4481	HACD3	6102.29	0.453141	0.090226	5.022313	5.11E-07	3.03E-06	5321.662	5027.978	5110.843	7006.647	6633.382	7513.227
5319	LEPROTL1	1450.199	0.453125	0.100472	4.509953	6.48E-06	3.24E-05	1180.122	1267.806	1224.487	1714.274	1649.254	1665.251
6417	HMGCL	2873.056	0.453096	0.114037	3.973224	7.09E-05	0.000294	2623.156	2106.441	2550.551	3243.281	3206.527	3508.383
5452	TMED3	2969.62	0.453059	0.10208	4.438293	9.07E-06	4.42E-05	2682.243	2288.729	2552.638	3482.118	3266.422	3545.568
6561	ZBTB1	1476.015	0.452682	0.115607	3.915691	9.01E-05	0.000365	1239.209	1379.478	1118.736	1691.952	1743.375	1683.341
25553	LOC11962	0.915096	0.452571	2.87812	0.157245	0.875052	0.910363	1.084173	0	1.391463	0	0	3.014938
22823	LOC11962	11.96376	0.452554	0.767587	0.589581	0.555472	0.647123	15.7205	4.926706	9.740239	15.62489	10.69555	15.07469
6673	FZD3	777.8337	0.452527	0.116998	3.867815	0.00011	0.000437	644.5407	687.5492	637.9857	908.4757	892.0091	896.4417
7080	UBA5	1654.437	0.452437	0.122189	3.702766	0.000213	0.000801	1334.617	1604.464	1253.012	1852.665	1908.087	1973.78
22958	MLC1	10.45282	0.452314	0.793702	0.569879	0.568759	0.658614	8.673382	10.40082	6.957314	20.08914	8.556443	8.039836
9281	KBTBD3	422.4995	0.452196	0.148891	3.037098	0.002389	0.006842	365.3662	328.4471	374.9992	495.5322	513.3866	457.2657
17201	LOC10323	75.37831	0.452024	0.307967	1.467769	0.142167	0.219732	74.26583	51.45671	65.39875	89.28508	83.42532	88.43819
25247	LOC11962	1.547523	0.451959	2.173933	0.207899	0.835308	0.879576	1.626259	2.189647	0	4.464254	0	1.004979
17586	CTRL	68.24075	0.451879	0.322139	1.402744	0.160693	0.242922	53.12446	65.68942	52.87558	82.5887	89.84265	65.32367
23933	ST6GALNA	6.439487	0.451839	1.080782	0.418067	0.675898	0.750763	4.336691	6.568942	4.87012	11.16063	10.69555	1.004979
5581	FAM160B1	1799.374	0.451821	0.10314	4.380641	1.18E-05	5.64E-05	1602.949	1528.374	1431.119	2026.771	2017.181	2189.85
7381	ZNF160	1671.257	0.451219	0.125101	3.606833	0.00031	0.001116	1375.273	1628.55	1232.14	1935.254	1884.556	1971.77
20993	LOC10323	29.51372	0.4511	0.512696	0.879858	0.378936	0.47989	24.93597	23.53871	25.04633	49.10679	36.36488	18.08963
23105	TRNASTOP	10.31834	0.450914	0.830315	0.543063	0.587086	0.675511	11.38381	7.663765	6.957314	4.464254	21.39111	10.04979
12034	FBXO6	404.9102	0.450665	0.187738	2.400497	0.016373	0.03617	406.5648	263.8525	357.6059	444.1933	457.7697	499.4748
24390	TRNAN-GU	4.580229	0.450558	1.294066	0.348172	0.727711	0.793202	2.168345	7.663765	1.391463	6.696381	8.556443	1.004979
19135	COL20A1	67.33625	0.450427	0.38707	1.163682	0.244553	0.339767	60.17159	32.2973	77.92191	89.28508	77.00798	67.33362
21633	SSPN	27.11699	0.45004	0.573692	0.784462	0.432769	0.531834	22.76763	19.70683	25.74206	15.62489	57.75599	21.10457
8030	VAMP3	5024.732	0.450027	0.132212	3.403826	0.000664	0.0022	3695.945	5135.27	3913.489	5593.71	5533.879	6276.097
7992	UBP1	7495.238	0.449815	0.131718	3.414974	0.000638	0.002122	5594.873	7737.666	5677.864	8368.244	8366.062	9226.717
7551	ADAM9	10982.11	0.449805	0.127019	3.541246	0.000398	0.001402	8478.231	11265.74	8108.053	12667.32	12261.38	13111.97
25474	LOC11962	0.911013	0.449721	2.661222	0.16899	0.865804	0.903529	1.626259	0	0.695731	0	2.139111	1.004979
25475	LOC11962	0.911013	0.449721	2.661222	0.16899	0.865804	0.903529	1.626259	0	0.695731	0	2.139111	1.004979
7393	LOC10324	788.4636	0.449291	0.124748	3.601584	0.000316	0.001137	643.9986	720.3939	637.2899	921.8684	853.5052	953.7255
7889	COMMD9	1253.313	0.449178	0.13039	3.444887	0.000571	0.001925	1181.206	937.7164	1063.773	1453.115	1311.275	1572.793
7193	GLB1L	654.3256	0.449133	0.122426	3.668612	0.000244	0.000901	560.5173	557.2652	542.6705	723.2091	776.4972	765.7944
9172	OPHN1	414.1776	0.448937	0.146606	3.062195	0.002197	0.006369	348.5615	373.3349	329.7767	462.0503	474.8826	496.4599
9820	VPS36	689.2549	0.448913	0.154771	2.900492	0.003726	0.010087	514.44	696.3078	539.8875	756.691	767.9407	860.2624
6804	EIF2AK3	1779.816	0.448758	0.117596	3.816088	0.000136	0.00053	1457.128	1705.188	1353.893	2053.557	1993.651	2115.482
13508	LOC10324	502.9392	0.448681	0.213059	2.105907	0.035212	0.069301	569.7328	305.4558	399.3498	575.8888	639.5941	527.6142
19403	LOC10322	42.59066	0.448489	0.401553	1.116889	0.264042	0.361654	31.44101	39.41365	36.17803	53.57105	57.75599	37.18424
24201	LOC10323	5.200855	0.44817	1.199086	0.37376	0.708583	0.778384	9.215468	3.284471	0.695731	6.696381	4.278221	7.034856
6650	POP1	1093.38	0.448147	0.11555	3.878375	0.000105	0.00042	949.7353	965.6344	862.0112	1247.759	1191.485	1343.658
18960	LOC11962	60.25264	0.448112	0.375439	1.193567	0.232647	0.326221	59.08741	35.03436	59.13717	51.33892	85.56443	71.35354
6569	TBC1D22B	1298.221	0.447894	0.114485	3.912244	9.14E-05	0.00037	1084.173	1184.052	1029.682	1477.668	1411.813	1601.937
9536	RIPOR1	762.366	0.447847	0.150534	2.97505	0.002929	0.008167	559.9752	770.7558	605.2863	870.5295	847.0878	920.5612
14549	LOC10324	152.4206	0.447657	0.233573	1.916556	0.055294	0.101038	134.4374	109.4824	143.3207	198.6593	149.7377	178.8863
7517	PPP1R12B	835.9925	0.447591	0.125965	3.553289	0.00038	0.001346	653.2141	761.9972	704.7759	941.9576	1037.469	916.5413
3862	RAB1A	7124.093	0.447587	0.081609	5.484511	4.15E-08	2.85E-07	5907.657	6238.305	5939.459	8095.925	8158.568	8404.643
21757	LOC10324	19.29709	0.447562	0.583153	0.767486	0.442793	0.541051	12.46799	22.44388	13.91463	22.32127	23.53022	21.10457
6877	STEAP2	2555.064	0.447224	0.118096	3.786949	0.000153	0.00059	2007.346	2502.219	1977.964	2899.533	2894.217	3049.108
5373	HECTD2	1923.622	0.44713	0.099842	4.478396	7.52E-06	3.72E-05	1567.714	1730.369	1587.659	2209.806	2149.806	2296.378
7606	MAP1S	1123.081	0.447098	0.126891	3.523485	0.000426	0.001489	978.4659	867.1003	1009.506	1337.044	1144.424	1401.946
25569	PAGE4	0.916871	0.447097	2.876715	0.155419	0.876491	0.911319	0	1.094824	1.391463	0	0	3.014938
25089	LOC10324	1.54144	0.44692	1.923986	0.232288	0.816314	0.864998	0.542086	0.547412	2.782925	2.232127	2.139111	1.004979
19200	LOC10322	50.7441	0.446888	0.387556	1.153092	0.248873	0.344598	52.58238	39.96106	35.4823	78.12444	47.06043	51.25395
6227	LOC10322	3061.865	0.446832	0.109937	4.06442	4.82E-05	0.000206	2680.617	2289.824	2801.71	3553.546	3704.94	3340.552
11599	CHST12	339.2873	0.446731	0.179057	2.494912	0.012599	0.028877	300.8579	267.6844	293.5986	453.1218	314.4493	406.0117
8857	FNDC3B	5367.541	0.446723	0.141569	3.155511	0.001602	0.004809	4116.062	5647.1	3868.266	5959.779	5884.693	6729.343
8373	CAPN1	4385.577	0.44645	0.135633	3.291615	0.000996	0.003163	4140.998	3104.92	3896.791	4926.304	4455.768	5788.682
7389	LPCAT4	883.1158	0.446401	0.123902	3.602868	0.000315	0.001132	766.5101	760.355	720.082	995.5286	932.6523	1123.567
19594	LOC11962	44.21709	0.446286	0.410627	1.08684	0.277108	0.375978	39.5723	26.27577	45.91827	53.57105	57.75599	42.20914

5937	PPM1D	3320.84	0.446247	0.106473	4.191196	2.77E-05	0.000124	2658.392	3157.471	2618.733	3845.955	3722.053	3922.435
7646	LOC10322'	1353.67	0.446174	0.127098	3.510478	0.000447	0.001555	1054.358	1315.978	1065.165	1537.935	1664.228	1484.355
15144	FAM43B	122.8333	0.446078	0.246865	1.806971	0.070767	0.12423	113.8381	106.1979	91.83654	158.481	121.9293	144.717
20758	LOC11961'	25.45786	0.445902	0.489853	0.910278	0.362676	0.464483	20.59928	20.25424	23.65487	29.01765	32.08666	27.13445
12866	IRX3	241.7788	0.445714	0.200279	2.225464	0.02605	0.053827	178.8885	245.2405	190.6304	256.6946	288.7799	290.4391
7482	DENND6B	1533.294	0.445543	0.124818	3.569551	0.000358	0.001271	1351.421	1167.082	1381.027	1810.255	1559.412	1930.566
7351	RAB5B	5104.488	0.445499	0.123203	3.615964	0.000299	0.001082	3984.335	5120.49	3864.788	5805.762	5606.609	6244.942
7368	CNNM2	804.9877	0.44541	0.12339	3.609785	0.000306	0.001106	675.9817	727.5103	644.2472	892.8508	898.4265	990.9098
25385	TRNAT-AG	0.911901	0.445329	2.426125	0.183556	0.854362	0.89475	1.084173	0.547412	0.695731	0	2.139111	1.004979
10619	KMT5C	370.4011	0.445271	0.163984	2.715333	0.006621	0.016576	333.3831	274.2533	335.3425	395.0865	432.1004	452.2408
5516	CORO1B	3879.659	0.445145	0.101055	4.404998	1.06E-05	5.10E-05	3453.09	3081.929	3326.987	4533.45	4102.814	4779.682
10660	KBTBD7	302.9046	0.445079	0.164769	2.701222	0.006909	0.017229	266.7065	249.0724	253.2462	381.6937	323.0057	343.703
7644	ST6GALNA	2140.937	0.445017	0.126743	3.511186	0.000446	0.001552	2070.77	1557.387	1815.163	2381.679	2359.439	2661.186
8684	ZNF691	521.8496	0.444977	0.138716	3.207836	0.001337	0.004094	407.1069	476.2483	443.1809	613.8349	579.699	611.0275
6794	NPHP3	1397.731	0.444818	0.116458	3.819549	0.000134	0.000523	1185.543	1295.176	1069.339	1700.881	1546.577	1588.873
8562	SRRT	4049.068	0.444561	0.137281	3.23833	0.001202	0.003733	2926.724	4195.364	3170.448	4580.325	4408.707	5012.838
20682	LOC11962:	31.91201	0.444525	0.480975	0.924216	0.355374	0.456782	35.23561	16.96977	29.22072	33.4819	36.36488	40.19918
22198	CUNH4orf!	16.88945	0.44429	0.641898	0.692151	0.488842	0.585452	8.131296	16.42235	18.08902	20.08914	23.53022	15.07469
8820	HIC2	652.187	0.444289	0.140266	3.167484	0.001538	0.004635	578.4062	495.9551	587.1973	714.2806	710.1847	827.0981
10523	MPV17	1141.987	0.444142	0.162137	2.739297	0.006157	0.015555	842.4022	1225.655	832.0947	1330.348	1405.396	1216.025
8376	PPP2R2A	1422.589	0.444121	0.134958	3.290804	0.000999	0.003171	1037.553	1396.995	1182.048	1508.918	1749.793	1660.226
22861	LOC11961'	14.44121	0.443795	0.759538	0.584296	0.559021	0.650085	10.84173	4.379295	21.56767	13.39276	21.39111	15.07469
10073	CUNH8orf:	663.3893	0.443619	0.155914	2.845283	0.004437	0.011711	615.8101	459.2785	609.4607	810.2621	785.0536	700.4707
21628	LOC10324:	21.80369	0.443195	0.563821	0.786056	0.431835	0.530808	13.01007	22.9913	20.17621	20.08914	21.39111	33.16432
20207	LOC11962:	46.23536	0.443072	0.444951	0.995778	0.319358	0.420158	59.6295	26.27577	31.30791	44.64254	66.31243	49.24399
22835	LOC10323(	11.41568	0.442946	0.753655	0.587731	0.556713	0.648134	8.131296	11.49565	9.740239	15.62489	6.417332	17.08465
8898	MIF	6837.187	0.442664	0.140743	3.145198	0.00166	0.004959	5755.331	4662.306	6970.533	8707.527	7514.696	7412.729
25396	GPR32	0.912789	0.442607	2.425672	0.182468	0.855216	0.895256	0.542086	1.094824	0.695731	0	2.139111	1.004979
4113	SCP2	4657.766	0.44258	0.083747	5.284757	1.26E-07	8.14E-07	3953.978	4042.089	3851.569	5225.409	5437.619	5435.934
22678	LOC11962!	12.30319	0.442558	0.724924	0.610488	0.541538	0.634821	11.9259	11.49565	8.348776	13.39276	8.556443	20.09959
25388	LOC11962/	0.928291	0.442531	2.415083	0.183237	0.854612	0.894884	0.542086	1.094824	0.695731	2.232127	0	1.004979
25389	LOC10321(	0.928291	0.442531	2.415083	0.183237	0.854612	0.894884	0.542086	1.094824	0.695731	2.232127	0	1.004979
7660	LOC10323/	761.1852	0.442403	0.126175	3.506274	0.000454	0.001577	654.2982	593.3944	690.8613	832.5834	883.4527	912.5214
18406	TRNAH-GU	91.39963	0.442379	0.350003	1.263931	0.206255	0.297908	99.74389	43.79295	89.05362	118.3027	91.98176	105.5228
21180	LOC11962(	26.40369	0.442295	0.518271	0.853405	0.393435	0.493837	29.81475	18.612	19.48048	26.78552	23.53022	40.19918
4338	LDHA	25279.08	0.442294	0.08627	5.126866	2.95E-07	1.81E-06	20641.02	23145.67	20515.03	29042.2	29271.59	29058.98
4353	H2AFY	11303.06	0.442134	0.08649	5.111962	3.19E-07	1.95E-06	9223.6	10180.76	9349.238	13412.85	12530.91	13121.01
5902	MAGED1	4874.992	0.442098	0.105008	4.210121	2.55E-05	0.000115	4054.264	4325.101	4029.676	5285.677	5242.96	6312.276
21874	FRMD1	22.49877	0.442051	0.590269	0.748896	0.45392	0.551689	15.17842	21.34906	21.56767	29.01765	10.69555	37.18424
10442	LOC10323:	322.5663	0.441992	0.160334	2.7567	0.005839	0.014865	295.4371	260.0206	264.3779	381.6937	370.0661	363.8026
24003	TBX6	6.084451	0.441424	1.086585	0.406249	0.68456	0.758181	6.505036	4.379295	4.174388	4.464254	14.97377	2.009959
7175	MYO9A	6728.397	0.441348	0.120155	3.673154	0.00024	0.000888	5233.844	6749.04	5140.063	7412.894	7741.442	8093.1
21231	SLC27A3	24.37751	0.440638	0.520571	0.846452	0.397301	0.497492	17.34676	20.80165	23.65487	37.94616	21.39111	25.12449
20326	ANKRD34A	36.51552	0.440538	0.452169	0.974277	0.329919	0.431511	22.76763	36.67659	32.69937	40.17829	55.61688	31.15436
23014	LOC11961ℇ	9.505939	0.440504	0.786372	0.560173	0.575362	0.664654	8.131296	8.211177	7.653045	11.16063	12.83466	9.044815
8929	PLPP6	701.2379	0.440463	0.140377	3.137709	0.001703	0.005069	526.3659	681.5277	576.7613	805.7978	815.0012	801.9736
4345	RANBP6	3315.49	0.4404	0.085974	5.122447	3.02E-07	1.85E-06	2866.553	2744.175	2830.931	3792.384	3777.669	3881.231
15614	LOC11962/	258.4532	0.440143	0.254803	1.727389	0.084098	0.143097	254.7806	148.896	257.4206	265.6231	248.1368	375.8623
20829	LOC11962/	30.57583	0.440078	0.488671	0.90056	0.367822	0.469457	24.39389	27.37059	25.74206	22.32127	53.47777	30.14938
23718	LOC11962!	7.358474	0.439846	0.97378	0.451689	0.651493	0.730245	8.673382	2.737059	6.957314	8.928508	12.83466	4.019918
7760	HNF1B	8485.496	0.439838	0.126545	3.475742	0.000509	0.001745	6372.767	8691.805	6542.658	9705.288	9343.635	10256.82
25395	TRNAT-UG	1.231493	0.439798	2.409659	0.182515	0.855179	0.895252	1.626259	0	1.391463	2.232127	2.139111	0
17162	LOC11962:	233.5294	0.439757	0.298602	1.472721	0.140826	0.218149	271.5853	114.4091	210.8066	247.7661	213.9111	342.698
11515	ZBTB7A	1240.638	0.439722	0.175133	2.510794	0.012046	0.027811	865.7119	1401.374	891.9276	1381.687	1443.9	1459.23
6780	SLC4A2	7534.39	0.43932	0.114855	3.825013	0.000131	0.000513	6790.174	5683.777	6719.374	8830.294	7666.573	9516.151
14652	SDSL	228.5739	0.439215	0.23156	1.896762	0.057859	0.104982	210.8716	141.7797	230.9828	276.7837	224.6066	286.4191
21782	LOC10321!	20.36371	0.43917	0.576049	0.762384	0.445831	0.544138	15.17842	15.87494	20.17621	33.4819	21.39111	16.07967
6383	CDK5	1284.035	0.439104	0.110161	3.986035	6.72E-05	0.00028	1119.408	1030.776	1124.302	1448.65	1403.257	1577.818
12832	MYOZ1	191.4366	0.43901	0.196575	2.233299	0.025529	0.052891	162.6259	174.6244	149.5822	220.9806	226.7457	214.0606
6137	ACAA2	3327.773	0.438554	0.106919	4.101758	4.10E-05	0.000178	2655.681	3184.295	2638.213	3785.687	3788.365	3914.395
7456	CAMTA2	1198.396	0.438538	0.122517	3.579403	0.000344	0.001228	931.8465	1151.754	969.8495	1339.276	1394.7	1402.951
12461	RELL2	231.6089	0.438121	0.189805	2.308269	0.020984	0.044769	219.0029	177.3614	194.8048	270.0874	245.9977	282.3992
24688	LOC11962:	3.096926	0.438077	1.454331	0.301222	0.763245	0.821892	2.168345	3.831883	2.087194	4.464254	0	6.029877
9131	SMARCC2	5240.874	0.437976	0.142543	3.072589	0.002122	0.006179	3706.245	5575.389	4073.507	6062.457	5743.512	6284.137
5010	OCIAD2	3163.299	0.437956	0.093546	4.681718	2.84E-06	1.51E-05	2768.435	2631.956	2655.607	3874.972	3593.706	3455.119
25314	LOC11962/	1.52505	0.437783	2.217547	0.197418	0.843501	0.885852	1.084173	0	2.782925	0	4.278221	1.004979
23605	CPED1	6.736567	0.43769	0.935977	0.467629	0.64005	0.720841	6.505036	4.926706	5.565851	6.696381	10.69555	6.029877
19795	LOC11961ℇ	41.78323	0.437608	0.415229	1.053895	0.291931	0.392028	28.73058	37.224	39.65669	46.87467	62.03421	36.17926
5166	USP38	2905.389	0.437193	0.09514	4.595268	4.32E-06	2.22E-05	2451.857	2601.848	2353.659	3176.317	3407.603	3441.05
7366	ERVH48-1	13277.29	0.437039	0.121038	3.61077	0.000305	0.001102	10142.98	13463.59	10239.08	15203.02	14687.13	15927.92
7835	KIAA1841	1057.723	0.436924	0.126392	3.456897	0.000546	0.001854	928.0519	976.5827	790.3508	1156.242	1294.162	1200.95
7375	BMP2	1057.32	0.436793	0.121047	3.608453	0.000308	0.00111	940.5199	795.9368	959.4136	1225.438	1208.598	1214.015
9592	SMIM6	690.5789	0.436739	0.147389	2.963175	0.003045	0.008439	646.709	543.0325	573.9784	821.4227	675.959	882.372
8941	ZDHHC14	542.9771	0.43671	0.139346	3.133996	0.001724	0.005127	480.2885	477.3431	429.2663	591.5136	601.0901	678.3611

7503	MAPK6	3891.311	0.436693	0.122682	3.559569	0.000371	0.001316	2984.728	3913.994	3022.257	4274.523	4577.697	4574.667
7723	LOC11961	5673.95	0.436143	0.125143	3.485158	0.000492	0.001693	5431.163	3961.072	5078.839	6508.882	6175.612	6888.129
5046	FZD6	2812.213	0.43606	0.093532	4.662153	3.13E-06	1.65E-05	2358.618	2289.276	2523.418	3160.692	3343.43	3197.845
13645	SLX1A	336.529	0.435974	0.209543	2.080588	0.037472	0.073007	331.7569	216.2277	313.0791	328.1227	380.7617	449.2258
6413	ANK2	11364.73	0.435961	0.109691	3.974441	7.05E-05	0.000292	9068.021	10994.22	8922.755	12906.16	12282.77	14014.44
19809	ZNF165	37.84638	0.435752	0.414366	1.05161	0.292978	0.393197	36.31979	32.2973	28.52499	40.17829	38.50399	51.25395
9088	MAP3K11	1464.588	0.435744	0.141184	3.086348	0.002026	0.005928	1370.394	1043.914	1325.368	1667.399	1493.099	1887.351
4300	DSTN	11146.53	0.435689	0.084523	5.154646	2.54E-07	1.57E-06	9029.533	9939.356	9456.381	13196.33	12823.97	12433.61
12638	LOC10321	196.6831	0.435475	0.191601	2.272827	0.023037	0.048459	166.9626	158.202	176.7158	223.2127	228.8848	226.1204
7091	UBQLN2	1113.662	0.435283	0.117617	3.700849	0.000215	0.000806	934.5569	1007.238	901.6679	1305.794	1167.954	1364.762
20054	LOC11962	50.99363	0.435233	0.426776	1.019816	0.307816	0.408077	66.67662	28.46541	34.78657	53.57105	64.17332	58.28881
5965	CCNL1	3430.352	0.435227	0.104114	4.18029	2.91E-05	0.00013	2705.553	3173.894	2875.458	3825.866	3773.391	4227.949
25035	TRNAG-GC	1.823802	0.43407	1.810609	0.239737	0.810534	0.860717	2.168345	1.094824	1.391463	0	4.278221	2.009959
23548	FAM221B	7.408992	0.433819	0.910039	0.476704	0.633573	0.715285	4.336691	6.02153	8.348776	11.16063	8.556443	6.029877
8062	RAB24	1674.517	0.433692	0.127722	3.395587	0.000685	0.002258	1499.953	1278.207	1502.084	1854.898	1724.123	2187.84
24628	LOC11962	3.67377	0.433341	1.392375	0.311224	0.75563	0.815674	0.542086	3.831883	4.87012	2.232127	8.556443	2.009959
8860	CHEK2	675.35	0.43308	0.137292	3.154439	0.001608	0.004825	630.4464	507.9982	587.1973	783.4766	737.9932	804.9886
7290	HCFC2	1125.256	0.433046	0.119059	3.637245	0.000276	0.001005	877.6378	1053.22	939.9331	1305.794	1334.805	1240.145
9374	SPRY4	467.5305	0.432839	0.143638	3.013413	0.002583	0.007325	383.2551	435.1924	375.6949	517.8535	545.4732	547.7138
5824	BRK1	3750.178	0.432597	0.101523	4.261083	2.03E-05	9.29E-05	3201.562	3043.062	3325.596	4506.664	4534.915	3889.271
9400	LOC11961	2025.308	0.432515	0.143821	3.007316	0.002636	0.007454	1879.413	1364.15	1932.046	2258.913	2179.754	2537.573
13755	LOC10323	192.2039	0.432511	0.210266	2.056968	0.039689	0.07671	140.9425	191.5941	157.931	223.2127	222.4675	217.0756
11354	SPIRE2	564.5605	0.432122	0.169668	2.546866	0.01087	0.025451	544.7968	411.6537	489.7949	611.6028	564.7252	764.7894
7900	JMJD1C	6413.502	0.432121	0.125667	3.438631	0.000585	0.001967	4957.922	6578.795	4844.378	7205.306	7270.837	7623.774
19510	LHX6	57.5508	0.431989	0.392804	1.099757	0.271438	0.369857	40.11439	62.95236	42.43961	64.73168	89.84265	45.22408
12372	LOC11962	4119.426	0.431978	0.185577	2.327757	0.019925	0.042815	3654.204	2355.513	4514.601	4928.536	4141.318	5122.38
6223	DPYD	5605.32	0.43188	0.106236	4.06531	4.80E-05	0.000205	5149.278	4403.381	4771.326	6087.01	6115.717	7105.205
16647	DNAH3	107.1312	0.431796	0.278748	1.549053	0.121369	0.193825	95.4072	88.1333	87.66215	151.7846	128.3466	91.45313
7441	MED25	1318.379	0.431675	0.120412	3.584985	0.000337	0.001204	1115.072	1086.612	1170.916	1551.328	1324.11	1662.236
24609	CHAD	3.651154	0.431406	1.369432	0.315025	0.752742	0.813206	2.168345	4.379295	2.782925	0	8.556443	4.019918
10419	KRBA1	1083.629	0.431054	0.156084	2.761668	0.005751	0.014673	1005.028	730.7948	1036.64	1225.438	1123.033	1380.842
20102	TRIM46	44.35772	0.431013	0.425878	1.012058	0.311511	0.41194	42.28274	25.72836	44.52681	49.10679	66.31243	38.18922
7685	S100A6	46603.47	0.431007	0.123243	3.497217	0.00047	0.001626	42398.74	32080.52	44599.86	54613.45	54686.36	51241.89
12118	CNBD2	248.8911	0.430813	0.18084	2.382285	0.017206	0.037746	219.0029	191.0467	226.8084	294.6408	267.3888	294.459
6152	VPS11	2268.619	0.430731	0.10518	4.09517	4.22E-05	0.000182	1954.221	2027.613	1820.033	2553.553	2434.308	2821.982
6343	DUSP16	1604.216	0.430534	0.107398	4.008779	6.10E-05	0.000256	1329.738	1470.348	1302.409	1828.112	1754.071	1940.615
6820	BCR	1019.428	0.430387	0.112968	3.809824	0.000139	0.000542	883.0587	869.29	857.141	1120.528	1127.311	1259.239
22401	LOC10322	17.89336	0.430238	0.6553	0.656551	0.51147	0.607001	24.39389	9.853413	11.82743	22.32127	12.83466	26.12947
20270	LOC11962	33.83507	0.430047	0.436868	0.984387	0.324925	0.426174	28.73058	33.93953	23.65487	35.71403	42.78221	38.18922
14857	OXLD1	180.5017	0.429997	0.231053	1.861031	0.06274	0.112266	174.5518	118.241	169.7585	189.7308	209.6328	221.0955
22729	LOC11962	12.88338	0.429923	0.711788	0.604005	0.54584	0.638396	9.215468	14.78012	9.044508	8.928508	19.252	16.07967
15227	LOC11962	122.9184	0.429658	0.239909	1.790922	0.073306	0.127986	117.0907	100.1764	97.40239	133.9276	141.1813	147.732
7306	NAGA	1240.138	0.429591	0.118247	3.633013	0.00028	0.001019	1047.311	1104.677	1024.117	1330.348	1345.501	1588.873
5664	ATP9A	4614.957	0.429591	0.09897	4.340611	1.42E-05	6.67E-05	4051.011	3944.65	3809.825	5080.321	4967.015	5836.921
9687	BMP2K	4247.83	0.429579	0.146377	2.934736	0.003338	0.009162	3161.99	4590.595	3108.528	4787.912	4697.487	5140.47
9001	MCTP1	528.4001	0.429469	0.13783	3.115928	0.001834	0.005416	449.9317	475.1535	426.4833	558.0317	641.7332	619.0674
8805	ZER1	751.0966	0.429201	0.135272	3.172871	0.001509	0.004558	636.9515	648.1356	639.3771	888.3865	737.9932	955.7355
11063	ZNF70	370.1271	0.429159	0.164252	2.612804	0.00898	0.021581	293.2687	355.8177	299.1645	419.6399	393.5964	459.2756
24432	LOC10324	3.972522	0.429086	1.262865	0.339772	0.734028	0.798631	1.626259	4.926706	3.478657	2.232127	8.556443	3.014938
8902	LOC10323	2051.506	0.428423	0.136245	3.1445	0.001664	0.004969	1942.838	1455.021	1854.124	2281.234	2149.806	2626.011
17106	AGMAT	100.2241	0.428362	0.289351	1.480423	0.138761	0.215617	97.03346	74.44801	86.27069	82.5887	128.3466	132.6573
20899	LOC11962	29.57815	0.428333	0.480953	0.890592	0.373148	0.47467	17.88885	29.01283	28.52499	31.24978	40.6431	30.14938
6257	SLC25A12	2162.368	0.428084	0.105785	4.046754	5.19E-05	0.000221	1987.831	1717.231	1829.774	2484.357	2338.048	2616.967
18540	OTUD7A	67.66663	0.42795	0.343808	1.244735	0.213229	0.305755	47.7036	68.42648	56.35424	62.49956	102.6773	68.3386
13731	KISS1R	166.8983	0.427897	0.207266	2.064485	0.038972	0.075454	138.232	146.159	144.0164	185.2665	179.6853	208.0308
10514	WASL	1493.45	0.427521	0.155965	2.741132	0.006123	0.015482	1082.546	1615.96	1123.606	1642.845	1704.871	1790.873
3908	ENSA	9370.984	0.427487	0.078598	5.438932	5.36E-08	3.65E-07	7913.919	8180.522	7883.332	10917.33	10571.48	10759.31
21247	LOC10322	34.4699	0.426783	0.506873	0.841993	0.399792	0.500211	29.81475	24.63353	32.69937	31.24978	66.31243	22.10955
8049	TTC30A	698.3291	0.426782	0.125529	3.399864	0.000674	0.002227	621.231	589.5625	578.1528	825.887	737.9932	837.1479
21504	LOC10322	25.63428	0.426727	0.531457	0.802939	0.42201	0.521724	15.17842	31.20247	19.48048	31.24978	23.53022	33.16432
21928	IL33	19.72145	0.426464	0.575922	0.74049	0.459003	0.556485	15.17842	22.44388	12.52316	24.5534	23.53022	20.09959
14989	LOC10324	159.6521	0.426316	0.232219	1.835839	0.066382	0.117737	127.9324	115.5039	166.2798	178.5702	179.6853	189.9411
18389	CCDC78	56.80745	0.426306	0.336606	1.266484	0.20534	0.29686	53.12446	45.43518	47.30973	64.73168	59.8951	70.34856
15115	KCNJ11	158.8986	0.426038	0.2353	1.810616	0.0702	0.123472	155.0367	109.4824	141.9292	205.3557	164.7115	176.8764
6179	AMBRA1	2402.704	0.425957	0.104245	4.086114	4.39E-05	0.000189	2096.248	2091.113	1969.616	2716.499	2534.846	3007.904
21048	LOC11962	27.43132	0.425921	0.48786	0.873039	0.382642	0.483302	27.6464	19.70683	22.2634	33.4819	36.36488	25.12449
6795	GLG1	8596.515	0.425857	0.111499	3.81938	0.000134	0.000523	6965.81	8091.294	6960.097	9314.666	9153.255	11093.97
20083	LOC11962	4592.376	0.425753	0.419333	1.015309	0.309959	0.410317	5240.349	1938.385	4580	5406.212	5456.871	4932.439
18524	LOC11962	60.39299	0.425646	0.341347	1.246958	0.212413	0.304848	58.00324	43.24553	52.87558	62.49956	83.42532	62.30873
21459	HUS1B	29.91199	0.425598	0.525446	0.809975	0.417954	0.517793	29.27266	15.32753	31.30791	31.24978	49.19955	23.11453
8645	RRAGB	594.0801	0.425187	0.132193	3.216414	0.001298	0.003992	518.7767	533.7265	468.2272	709.8164	654.5679	679.3661
23783	TRNAA-UG	7.141904	0.424984	0.968119	0.438979	0.660676	0.738514	8.673382	2.737059	6.957314	11.16063	4.278221	9.044815
10937	LOC10323	5606.913	0.424786	0.160816	2.641444	0.008255	0.020067	3913.322	6263.486	4182.041	6682.988	6828.041	5771.597
14816	LOC11962	170.6345	0.424786	0.227235	1.869372	0.061571	0.11048	117.0907	163.1287	157.2353	209.8199	177.5462	198.9859

24326	PTCHD1	5.261741	0.424729	1.186568	0.357947	0.720383	0.78728	4.878777	6.02153	2.087194	11.16063	6.417332	1.004979
7582	DICER1	6620.639	0.424633	0.120226	3.531949	0.000413	0.001446	5285.342	6571.679	5106.668	7196.377	7251.585	8312.185
22476	CTSS	17.96641	0.424626	0.660804	0.64259	0.52049	0.615645	14.09425	16.96977	13.91463	31.24978	23.53022	8.039836
24418	LOC11962	4.275724	0.424547	1.237289	0.343127	0.731503	0.796421	2.710432	3.831883	4.174388	2.232127	10.69555	2.009959
16771	GLIPR1L1	99.23104	0.424541	0.277449	1.530159	0.125977	0.199697	76.43418	90.32295	89.05362	116.0706	89.84265	133.6623
19008	LOC10323	96.35412	0.424244	0.358431	1.183615	0.236565	0.330866	118.1748	65.68942	61.22436	104.91	149.7377	78.3884
10540	NDUFC2	2375.248	0.42411	0.154969	2.736732	0.006205	0.015652	2319.045	1494.434	2272.954	2770.07	2667.471	2727.514
8891	PAQR4	1705.806	0.424099	0.134775	3.146713	0.001651	0.004937	1556.872	1261.784	1557.743	1886.147	1741.236	2231.054
10646	CTXN1	420.8287	0.423997	0.156627	2.70705	0.006788	0.016952	311.1576	384.2831	383.348	466.5145	496.2737	483.3951
18902	LOC10322	10303.79	0.423978	0.35203	1.204382	0.228442	0.321296	6199.842	13417.06	6784.077	12238.75	12436.79	10746.25
17908	ITPKA	83.2869	0.423949	0.311679	1.360207	0.173764	0.257952	88.90216	53.64636	70.9646	87.05295	102.6773	96.47803
10390	GPATCH11	1042.869	0.423939	0.153157	2.768001	0.00564	0.014431	789.2778	1103.035	778.5234	1180.795	1242.823	1162.761
24420	PRRX2	4.280032	0.423926	1.237558	0.342551	0.731936	0.796828	4.878777	4.379295	1.391463	4.464254	8.556443	2.009959
9313	NRXN1	3185.226	0.423792	0.139855	3.030223	0.002444	0.006976	2432.884	3367.677	2362.008	3450.868	3741.305	3756.613
21925	GATA3	26.72727	0.42379	0.572058	0.740816	0.458805	0.55635	13.55216	24.08612	29.91645	44.64254	32.08666	16.07967
5924	THSD4	1725.759	0.423789	0.100906	4.199859	2.67E-05	0.00012	1467.97	1491.15	1467.993	1904.004	1905.948	2117.492
24013	LOC11962	5.814819	0.423771	1.047602	0.404515	0.685834	0.759261	4.878777	6.568942	3.478657	2.232127	10.69555	7.034856
6304	NRBP1	4852.733	0.423654	0.105267	4.024557	5.71E-05	0.000241	3949.641	4630.009	3858.526	5578.085	5262.212	5837.926
20883	LOC11962	43.32582	0.423387	0.47449	0.892299	0.372233	0.473869	27.6464	48.71965	34.09084	29.01765	81.28621	39.1942
6453	STIM1	4519.03	0.423351	0.106934	3.959016	7.53E-05	0.00031	3823.877	4089.166	3674.853	5151.749	4646.148	5728.383
23848	LOC11962	7.120345	0.423189	0.983025	0.430497	0.666834	0.743366	2.710432	4.926706	10.43597	8.928508	10.69555	5.024897
10982	HELB	530.5149	0.422954	0.160615	2.633333	0.008455	0.020468	416.8644	535.3688	409.7858	580.353	575.4208	665.2964
7246	FAM120B	2173.376	0.422667	0.115673	3.653962	0.000258	0.000947	1804.606	2068.669	1701.759	2464.268	2316.657	2684.3
9006	CARHSP1	2417.946	0.422455	0.135638	3.114586	0.001842	0.005438	2232.312	1659.205	2309.828	2841.498	2592.602	2872.231
5517	CTNND1	8937.383	0.422374	0.095895	4.404567	1.06E-05	5.11E-05	7606.014	8218.294	7094.373	10116	9730.814	10858.8
20307	LOC10323	47.10051	0.422214	0.431815	0.977768	0.328189	0.42965	37.94605	39.96106	41.04815	69.19594	66.31243	28.13943
13483	NTN1	189.4934	0.422049	0.199991	2.110337	0.034829	0.068674	166.4205	148.3486	172.5414	214.2842	203.2155	232.1503
20754	FAM71D	32.31676	0.422027	0.463541	0.910441	0.36259	0.464437	33.60935	25.72836	22.95914	31.24978	49.19955	31.15436
9724	MAP1LC3B	1235.046	0.42195	0.144304	2.924036	0.003455	0.009447	945.3986	1201.569	1024.117	1185.259	1478.125	1575.808
24839	HS3ST6	2.746236	0.421943	1.534213	0.275023	0.783299	0.838359	4.336691	1.094824	1.391463	2.232127	6.417332	1.004979
6355	NR3C1	3264.817	0.421917	0.105379	4.003807	6.23E-05	0.000261	2700.674	2953.834	2724.484	3542.386	3520.976	4146.545
5690	STK40	3169.829	0.421914	0.097416	4.331042	1.48E-05	6.93E-05	2809.092	2592.542	2732.833	3526.761	3450.386	3907.36
26971	LOC11962	0.657251	0.421733	3.267781	0.129058	0.897312	NA	0.542086	0	1.391463	0	0	2.009959
20969	LOC11961	30.35925	0.421504	0.477297	0.883107	0.377178	0.478211	23.30971	31.74989	22.2634	46.87467	27.80844	30.14938
25366	LOC11962	1.58255	0.421355	2.243773	0.187788	0.851043	0.891905	0	0.547412	3.478657	4.464254	0	1.004979
18222	STAB1	62.1557	0.421328	0.324663	1.29774	0.194377	0.283586	55.8349	49.26706	53.57132	80.35657	70.59065	63.31371
14729	FN3K	163.6507	0.421288	0.223672	1.883512	0.059631	0.107625	118.7169	159.2968	141.9292	200.8914	171.1289	189.9411
11796	METRN	344.0698	0.421216	0.17195	2.449639	0.0143	0.032228	318.7468	252.3568	309.6005	419.6399	404.2919	359.7826
8465	TMEM117	624.0766	0.421146	0.129055	3.263321	0.001101	0.003458	506.3087	574.235	520.4071	698.6557	727.2976	717.5553
9941	DIAPH2	613.1015	0.421097	0.146377	2.876796	0.004017	0.010744	485.7094	608.7219	477.2717	700.8879	716.6021	689.4159
25036	LOC11961	2.128779	0.421062	1.757742	0.239547	0.810681	0.860839	2.168345	1.094824	2.087194	0	6.417332	1.004979
9508	OXSM	1210.069	0.421019	0.141144	2.982903	0.002855	0.007984	1136.213	834.2556	1134.042	1361.597	1422.509	1371.797
20732	LOC10322	34.05599	0.420929	0.460789	0.913496	0.360982	0.462893	22.76763	24.08612	40.35242	40.17829	42.78221	34.1693
8458	LOC10324	1469.012	0.420847	0.12886	3.265936	0.001091	0.003429	1143.26	1475.275	1149.348	1676.327	1700.593	1669.271
16703	LOC11962	104.7325	0.420838	0.272906	1.542061	0.123059	0.195864	99.74389	95.79707	71.66033	129.4634	126.2075	105.5228
19692	LOC10324	44.39329	0.420735	0.392755	1.071241	0.284061	0.383502	41.74065	39.96106	31.30791	49.10679	62.03421	42.20914
4711	LOC10323	10249.33	0.420564	0.086217	4.877989	1.07E-06	6.05E-06	8930.331	8796.36	8575.585	11814.65	11005.72	12373.31
22934	GALNT3	12.3525	0.420278	0.73217	0.574016	0.565957	0.656092	8.673382	9.306001	13.2189	15.62489	19.252	8.039836
16630	LOC11962	92.74401	0.420183	0.270616	1.552695	0.120496	0.192627	74.80792	77.18507	86.96642	95.98146	106.9555	114.5677
10735	ZNF709	370.2427	0.420005	0.156471	2.684239	0.00727	0.018003	321.4572	292.8653	333.9511	433.0326	449.2132	390.937
9411	RCOR3	707.5121	0.41957	0.139627	3.004948	0.002656	0.007504	578.9482	686.4544	549.6278	765.6196	868.4789	795.9437
4904	HADHB	7672.088	0.419438	0.088475	4.740738	2.13E-06	1.15E-05	6590.686	6797.76	6310.284	8609.314	8400.288	9324.2
9833	CHMP1A	2850.871	0.419389	0.144697	2.898398	0.003751	0.010141	2767.351	1952.071	2603.427	3156.228	2934.86	3691.29
10367	CLCN2	551.3663	0.419337	0.151269	2.772133	0.005569	0.014281	498.7195	431.9079	489.0992	611.6028	558.3079	718.5603
19435	LOC11962	45.73457	0.419181	0.376968	1.11198	0.266147	0.364059	44.45108	36.12918	36.17803	55.80317	55.61688	46.22906
11750	LOC11962	399.1888	0.418893	0.170361	2.458858	0.013938	0.031535	383.7971	293.4127	347.17	415.1756	513.3866	442.191
6286	WBP1L	2311.918	0.41886	0.103821	4.034434	5.47E-05	0.000231	2005.177	2056.079	1878.475	2671.856	2427.891	2832.032
20251	LOC11962	38.71781	0.41858	0.424077	0.987038	0.323624	0.424846	41.74065	27.37059	29.91645	53.57105	38.50399	41.20416
6121	MTCL1	2466.984	0.418567	0.101885	4.108247	3.99E-05	0.000173	2136.362	2040.204	2164.42	2754.445	2633.245	3073.227
23872	LOC11961	6.762531	0.418443	0.980318	0.426844	0.669493	0.74557	3.794605	7.663765	5.565851	6.696381	12.83466	4.019918
7240	SMAP1	3184.566	0.418422	0.114466	3.655414	0.000257	0.000943	2567.321	3136.122	2474.716	3671.849	3535.95	3721.439
23615	LOC10323	8.008069	0.418298	0.896892	0.466385	0.64094	0.721549	6.505036	10.40082	3.478657	8.928508	10.69555	8.039836
20472	CATSPER2	35.47807	0.418187	0.439873	0.9507	0.341757	0.443784	39.03022	26.82318	25.04633	35.71403	47.06043	39.1942
20684	LOC11962	34.14339	0.418078	0.452518	0.923893	0.355542	0.456954	28.18849	30.10765	28.52499	55.80317	32.08666	30.14938
19741	LOC10322	44.15245	0.417871	0.393608	1.061643	0.288398	0.388382	47.16151	32.84471	33.39511	44.64254	55.61688	51.25395
4673	ARHGAP18	4789.284	0.417854	0.08523	4.902646	9.46E-07	5.38E-06	4063.479	4285.14	3951.754	5479.872	5512.488	5442.969
10479	DUS2	647.8464	0.417803	0.151965	2.749343	0.005971	0.01515	639.6619	473.5112	553.1064	696.4236	729.4367	794.9388
6688	ADPRH	2188.269	0.417698	0.108199	3.860471	0.000113	0.00045	1976.447	1844.778	1805.423	2430.786	2314.518	2757.664
10389	LYPLA2	2088.378	0.417679	0.150893	2.768047	0.005639	0.014431	2047.46	1407.943	1913.261	2325.876	2143.389	2692.34
5852	NIPAL3	2884.157	0.417266	0.098314	4.244205	2.19E-05	9.97E-05	2437.762	2504.409	2474.021	3071.407	3238.614	3578.732
7956	HYAL2	1237.307	0.417241	0.121882	3.42332	0.000619	0.002067	1155.728	932.2423	1092.994	1366.062	1407.535	1469.28
6731	CLSTN1	16780.61	0.417193	0.108521	3.844337	0.000121	0.000477	14685.66	14712.79	13723.3	17961.93	17523.59	22076.38
13114	LOC11962	456.9487	0.416858	0.190753	2.185322	0.028865	0.058516	446.6792	301.6239	429.2663	484.3716	472.7435	607.0076
16277	LOC11962	116.3548	0.416756	0.25849	1.612268	0.106904	0.174604	112.2119	87.03848	98.09812	140.624	147.5986	112.5577

22767	PRR25	14.96304	0.416575	0.696034	0.598498	0.549508	0.64166	14.63633	8.758589	14.61036	29.01765	10.69555	12.05975
19991	PCDHB16	43.545	0.416468	0.403905	1.031103	0.302492	0.402269	34.15144	48.17224	29.22072	46.87467	55.61688	47.23404
15586	PCDHB12	198.6041	0.416413	0.240479	1.731594	0.083346	0.142163	163.7101	213.4906	134.2762	183.0344	243.8586	253.2548
15130	CFAP94	158.1673	0.416281	0.230078	1.809306	0.070404	0.123706	133.3532	153.8227	118.2743	162.9453	211.772	168.8366
6114	GBF1	4802.338	0.416163	0.101192	4.112601	3.91E-05	0.00017	4024.449	4317.437	4008.108	5341.48	5067.553	6055.001
19029	LOC10322{	52.26447	0.416095	0.352767	1.179517	0.238192	0.332773	51.49821	42.15071	40.35242	60.26743	62.03421	57.28383
6354	ACTR1A	6899.486	0.416055	0.103914	4.003848	6.23E-05	0.000261	6078.957	6035.763	5626.38	7741.016	7123.239	8791.56
6515	PLEKHM3	1636.948	0.415969	0.105749	3.933549	8.37E-05	0.000342	1358.468	1514.141	1337.196	1819.183	1846.053	1946.645
19620	LOC11962⁄	65.39814	0.415794	0.384078	1.082575	0.278997	0.37804	71.01331	36.67659	61.92009	53.57105	72.72976	96.47803
11222	CRABP2	310.0656	0.415779	0.161422	2.575727	0.010003	0.023697	269.959	259.4732	265.7694	379.4616	355.0924	330.6382
7164	ATG9A	2435.174	0.415663	0.11308	3.675824	0.000237	0.00088	2000.841	2238.914	2026.665	2691.945	2541.263	3111.416
6433	CEMIP2	8913.886	0.415642	0.104767	3.967313	7.27E-05	0.0003	7266.668	8455.323	7198.732	9953.054	9544.712	11064.82
23630	LOC10323(	8.941152	0.415518	0.895377	0.464071	0.642597	0.722955	12.46799	3.284471	6.957314	8.928508	14.97377	7.034856
11433	ZFYVE27	1101.12	0.41529	0.16417	2.529636	0.011418	0.026552	1122.661	741.743	970.5453	1147.313	1189.346	1435.111
8287	SLC9A8	705.5096	0.41501	0.125105	3.317289	0.000909	0.002916	621.7731	558.3601	635.2027	787.9408	810.7229	819.0583
20768	LOC10322(	39.05641	0.414997	0.456672	0.908741	0.363487	0.465315	29.81475	47.62483	22.2634	55.80317	40.6431	38.18922
22312	ONECUT1	17.68781	0.414658	0.616058	0.673082	0.500895	0.596822	10.29964	15.32753	19.48048	24.5534	21.39111	15.07469
10665	BDH1	587.711	0.414556	0.153539	2.7	0.006934	0.017284	562.1436	416.5804	533.626	667.406	663.1243	683.386
6315	KLF13	4345.301	0.414201	0.103073	4.018508	5.86E-05	0.000247	3757.201	3791.922	3635.196	4854.876	4509.245	5523.367
23169	LOC11962(	10.2094	0.414164	0.775165	0.534292	0.59314	0.680591	10.84173	8.758589	6.957314	8.928508	10.69555	15.07469
7672	SLC35A3	866.9036	0.413984	0.11819	3.502711	0.000461	0.001596	780.6044	703.4242	747.9112	1004.457	936.9305	1028.094
23593	LOC11962:	9.233951	0.413953	0.881431	0.469637	0.638614	0.719602	7.589209	4.926706	11.1317	4.464254	19.252	8.039836
5985	PNMA1	2735.326	0.413799	0.099212	4.170874	3.03E-05	0.000135	2414.453	2419.56	2206.86	3091.496	2954.112	3325.477
21198	LOC10323(	26.33536	0.413454	0.486234	0.850319	0.395148	0.495566	25.47806	19.70683	22.2634	33.4819	29.94755	27.13445
11023	ZNF350	420.6159	0.412976	0.157357	2.624447	0.008679	0.02093	314.4101	400.7054	367.3462	495.5322	466.3261	479.3752
11160	BRMS1L	821.6103	0.412823	0.159401	2.589842	0.009602	0.022873	627.1939	862.1736	621.9838	1002.225	966.878	849.2077
23751	CABP7	8.912583	0.412786	0.928619	0.444516	0.65667	0.734994	8.131296	4.379295	10.43597	2.232127	19.252	9.044815
7256	DZIP1	8265.957	0.412745	0.113061	3.650644	0.000262	0.000958	6561.413	8254.423	6459.866	9261.095	9296.575	9762.371
14523	REL	406.9809	0.412698	0.214984	1.919672	0.054899	0.100461	298.1475	476.2483	273.4224	433.0326	470.6043	490.43
15418	HOGA1	243.5174	0.412365	0.234617	1.75761	0.078814	0.135898	253.1543	148.896	226.8084	263.391	250.2759	318.5785
7520	ASL	2214.705	0.412294	0.116066	3.55224	0.000382	0.00135	2062.097	1659.753	1982.834	2453.108	2447.143	2683.295
22269	LOC10324!	17.02189	0.412148	0.606894	0.67911	0.497068	0.593406	13.55216	18.06459	12.52316	15.62489	19.252	23.11453
24239	LOC11962;	5.3106	0.412095	1.116126	0.369219	0.711965	0.780776	4.336691	1.642235	7.653045	8.928508	4.278221	5.024897
9123	VIPAS39	1320.293	0.412078	0.133995	3.075314	0.002103	0.006128	1052.732	1338.969	1008.115	1459.811	1497.377	1564.753
22596	MIA	19.86855	0.411977	0.659116	0.625044	0.531942	0.625849	23.30971	6.02153	21.56767	24.5534	25.66933	18.08963
20429	LOC11961!	33.49138	0.411279	0.429504	0.957566	0.338281	0.440218	29.27266	25.72836	31.30791	37.94616	38.50399	38.18922
6913	MAPKBP1	2011.41	0.411247	0.109096	3.769604	0.000164	0.000629	1741.724	1653.184	1791.508	2229.895	2115.58	2536.568
14789	LOC11962(	163.5999	0.411212	0.219321	1.874933	0.060802	0.109299	151.2421	120.978	150.278	171.8738	188.2417	198.9859
24784	LOC11962;	3.131291	0.411036	1.448332	0.2838	0.776564	0.832962	3.794605	2.189647	2.087194	6.696381	0	4.019918
17220	LOC10321(	116.733	0.410913	0.280538	1.464731	0.142994	0.220761	117.0907	75.54283	106.4469	147.3204	145.4595	108.5378
20381	LOC11961!	41.46511	0.410867	0.425907	0.964686	0.334702	0.436586	48.24569	25.72836	32.69937	42.41041	53.47777	46.22906
9790	TAF11	651.7746	0.410825	0.141374	2.905941	0.003662	0.009943	597.3792	484.4595	596.9375	712.0485	787.1927	732.63
24856	LOC11962(	2.802849	0.410786	1.505923	0.27278	0.785022	0.839629	3.794605	1.094824	2.087194	6.696381	2.139111	1.004979
7677	OSTF1	755.5506	0.410763	0.117396	3.498955	0.000467	0.001617	672.1871	633.3555	641.4643	843.744	864.2007	878.3521
11704	CSAD	355.1213	0.410554	0.166115	2.471502	0.013455	0.030562	336.0935	264.3999	315.1663	386.158	412.8484	416.0615
8611	GPRIN1	980.0439	0.410549	0.127267	3.22588	0.001256	0.003877	862.4594	793.1997	873.8386	1116.063	992.5473	1242.155
9756	GRAMD2B	461.4864	0.410531	0.140826	2.915159	0.003555	0.009688	394.6389	399.0632	397.2626	482.1394	539.0559	556.7586
10481	RNASET2	753.8259	0.410152	0.1492	2.749012	0.005978	0.015162	649.9616	558.9075	736.7795	874.9938	763.6625	938.6508
7549	PALM	1104.438	0.410123	0.115763	3.54278	0.000396	0.001394	1002.86	872.027	972.6325	1279.009	1193.624	1306.473
6444	CNTN1	3949.95	0.41007	0.103494	3.962254	7.42E-05	0.000306	3229.751	3759.077	3189.233	4618.271	4265.387	4637.98
5667	MPZL1	7762.975	0.40995	0.094462	4.339855	1.43E-05	6.69E-05	6467.09	7256.491	6281.063	8794.58	8547.886	9230.736
10017	DEF8	2146.78	0.409877	0.143451	2.857258	0.004273	0.011341	2069.686	1526.732	1940.395	2368.287	2162.641	2812.938
4510	SOD1	10338.63	0.40944	0.081783	5.006404	5.55E-07	3.27E-06	8795.893	8849.459	8993.719	12057.95	12120.2	11214.57
7434	NDUFA3	2167.209	0.4093	0.114128	3.586333	0.000335	0.001199	1749.313	2115.199	1721.239	2502.214	2395.804	2519.484
9674	TOPORS	2945.781	0.409068	0.139229	2.938092	0.003302	0.009075	2243.153	3138.312	2211.034	3337.03	3341.291	3403.865
16086	LOC11962⁄	130.2103	0.408939	0.247995	1.648985	0.099151	0.163864	93.78094	116.0513	125.2316	138.3919	171.1289	136.6772
19744	ROPN1L	56.53236	0.408878	0.385251	1.061329	0.28854	0.388515	59.6295	41.6033	43.83108	46.87467	91.98176	55.27387
6826	DTX3	2323.683	0.408866	0.107366	3.808143	0.00014	0.000545	2024.693	1955.902	2016.23	2544.625	2445.003	2955.645
23287	LOC11962:	10.84719	0.408843	0.79305	0.515532	0.606182	0.692031	5.96295	13.13788	8.348776	15.62489	14.97377	7.034856
12217	CLDN4	1566.992	0.408766	0.172913	2.363991	0.018079	0.039342	1592.65	1000.121	1450.6	1736.595	1548.716	2073.273
10658	MRPL43	2304.277	0.408691	0.151273	2.701679	0.006899	0.017209	2192.739	1483.486	2263.91	2689.713	2631.106	2564.708
11273	MELTF	1666.009	0.408458	0.159328	2.563633	0.010358	0.024426	1668	1111.246	1518.782	1977.665	1630.002	2090.357
7419	LOC11962!	7263.601	0.408149	0.113617	3.592325	0.000328	0.001174	5772.136	7236.237	5722.391	8203.067	7944.657	8703.122
24680	SLC25A52	3.725516	0.408034	1.348815	0.302513	0.762261	0.821099	1.084173	1.642235	6.957314	2.232127	6.417332	4.019918
5542	TMBIM1	5521.9	0.407977	0.09282	4.395367	1.11E-05	5.30E-05	4876.067	4720.332	4648.181	6249.956	5863.302	6773.562
17408	LOC10323:	96.88417	0.407952	0.285589	1.428457	0.15316	0.233904	73.18166	100.1764	76.53045	91.51721	128.3466	111.5527
13734	PTS	350.8138	0.407854	0.197639	2.063626	0.039053	0.075595	320.9151	235.3871	346.4742	462.0503	389.3181	350.7378
23155	LOC10322{	12.37005	0.407737	0.760473	0.536163	0.591846	0.679466	9.215468	7.663765	14.61036	11.16063	23.53022	8.039836
19958	ZNF30	41.5908	0.40766	0.393372	1.036321	0.300053	0.399684	37.40396	32.2973	36.87376	53.57105	49.19955	40.19918
4398	ARL6IP5	8864.259	0.407604	0.0802	5.08236	3.73E-07	2.25E-06	7786.529	7338.055	7735.837	10223.14	10060.24	10041.75
24106	TRNAK-UU	7.069081	0.406908	1.044429	0.389599	0.696833	0.768494	3.794605	9.853413	4.174388	4.464254	17.11289	3.014938
23868	LOC10321!	8.146015	0.406649	0.951608	0.427329	0.66914	0.745314	5.420864	9.853413	5.565851	17.85702	2.139111	8.039836
5696	NNT	4539.919	0.406089	0.093831	4.327898	1.51E-05	7.03E-05	4024.449	3910.163	3786.866	4915.144	5016.215	5586.681
18587	WBP2NL	64.88427	0.406064	0.328318	1.236802	0.216161	0.309175	67.21871	46.53	53.57132	75.89232	72.72976	73.3635

11451	PPP1R3E	330.2572	0.405795	0.160624	2.52637	0.011525	0.026754	262.9119	308.7403	281.0755	368.301	378.6226	381.8922
7052	TLR2	980.3471	0.405076	0.10914	3.711526	0.000206	0.000777	852.7019	853.415	826.5289	1104.903	1088.807	1155.726
10136	ZDHHC12	453.4188	0.404975	0.143058	2.830845	0.004643	0.01218	389.7601	376.6193	407.0029	482.1394	511.2474	553.7437
6516	CDIPT	3512.633	0.404889	0.102936	3.933425	8.37E-05	0.000342	3079.051	3063.864	2932.508	3903.99	3666.436	4429.95
22885	LOC10323:	14.00484	0.404851	0.695575	0.582037	0.560541	0.651169	13.55216	13.6853	8.348776	24.5534	12.83466	11.05477
9564	PLPPR2	970.2588	0.404845	0.136371	2.96869	0.002991	0.008314	867.3382	758.7128	883.5788	1122.76	954.0434	1235.12
9579	FGFR4	1985.623	0.404698	0.136462	2.965643	0.003021	0.008383	1807.316	1430.387	1893.781	2258.913	2012.903	2510.439
10115	SNX8	1190.158	0.404644	0.142747	2.834697	0.004587	0.012057	1134.045	852.3202	1090.907	1285.705	1268.493	1509.479
13208	DECR2	610.5527	0.404385	0.186689	2.166091	0.030304	0.060996	608.2209	389.7572	580.9357	716.5128	601.0901	766.7993
4725	MYL6	25870.12	0.404338	0.08308	4.866824	1.13E-06	6.38E-06	21608.65	22186.6	23005.05	30033.27	30525.11	27862.05
12900	LOC11961!	253.6734	0.404101	0.182092	2.219215	0.026472	0.054561	210.8716	229.913	217.0682	272.3195	265.2497	326.6183
23328	LOC11962(	10.53647	0.403581	0.791811	0.509694	0.610266	0.69547	10.29964	5.474118	11.1317	11.16063	17.11289	8.039836
15803	PPIL6	154.1644	0.403572	0.237283	1.700804	0.08898	0.149688	137.1479	147.2538	112.0128	165.1774	209.6328	153.7619
9028	DCAF5	2630.169	0.403521	0.129918	3.105968	0.001897	0.005585	2064.265	2703.12	2028.753	2903.997	2892.078	3188.8
6992	TTYH3	3527.118	0.403066	0.107843	3.737544	0.000186	0.000707	3056.825	2873.912	3188.537	4013.364	3600.123	4429.95
4871	TM7SF3	8609.725	0.402982	0.084683	4.75872	1.95E-06	1.06E-05	7636.913	7318.896	7294.048	9676.27	9412.087	10320.13
16062	LOC11962:	172.2054	0.402938	0.243499	1.654779	0.097969	0.162154	145.8212	128.6418	168.367	187.4987	248.1368	154.7668
14352	ZNF571	177.0288	0.402916	0.206363	1.952459	0.050884	0.094257	160.4576	149.4434	146.7993	227.677	181.8244	195.971
16033	LOC10324	143.1922	0.40258	0.242498	1.660139	0.096886	0.160643	134.9795	119.8832	112.7085	167.4095	192.52	131.6523
16765	FAM110A	106.754	0.402498	0.262971	1.530582	0.125873	0.199602	96.49137	85.94365	91.83654	145.0883	117.6511	103.5129
17221	LOC10322:	101.1618	0.402299	0.274729	1.464348	0.143099	0.22091	74.26583	100.7238	85.57496	133.9276	106.9555	105.5228
11926	ZNF606	438.425	0.402131	0.166208	2.419444	0.015544	0.034651	346.3932	442.8562	342.9956	464.2824	547.6123	486.4101
19243	YY2	67.1912	0.402073	0.350941	1.145702	0.251919	0.348048	71.5554	60.2153	41.74388	60.26743	91.98176	77.38342
8617	PRKD2	740.8985	0.402047	0.124675	3.224752	0.001261	0.00389	604.4263	641.5666	672.0765	839.2797	793.6101	894.4317
11186	TCTA	543.3447	0.401674	0.155402	2.584744	0.009745	0.023161	512.8137	401.8003	493.2735	584.8173	583.9772	683.386
9030	SNX14	903.9196	0.401543	0.129321	3.105009	0.001903	0.005602	707.4227	879.1434	748.607	1062.492	1028.912	996.9396
16921	MATK	130.6404	0.401378	0.267046	1.503026	0.132832	0.208696	125.222	87.03848	127.3188	120.5349	151.8769	171.8515
9721	SPHK2	652.7864	0.401271	0.137209	2.924529	0.00345	0.009434	552.386	511.83	624.7668	776.7802	695.211	755.7446
11202	LOC11962:	478.5461	0.401146	0.155356	2.582103	0.00982	0.023305	395.181	452.7096	392.3925	549.1032	474.8826	607.0076
7135	HSD17B4	5815.735	0.400956	0.108806	3.685045	0.000229	0.000852	4765.481	5694.725	4580	6310.223	6622.687	6921.294
7017	PDCD10	3977.941	0.400827	0.107526	3.727727	0.000193	0.000732	3436.828	3178.273	3667.2	4475.415	4973.432	4136.495
7508	ZNF862	1661.146	0.400804	0.112723	3.555656	0.000377	0.001335	1503.206	1369.624	1427.641	1897.308	1704.871	2064.228
13871	IL7	247.0897	0.400726	0.196807	2.036136	0.041737	0.079992	233.0971	180.6459	224.0255	265.6231	320.8666	258.2797
22009	LOC10323	32.79859	0.400657	0.553258	0.724177	0.468957	0.56646	23.8518	26.82318	32.69937	46.87467	53.47777	13.06473
10664	MBD6	829.4652	0.400565	0.148341	2.700298	0.006928	0.017271	633.699	860.5314	649.8131	953.1182	954.0434	925.5861
6102	SH2B3	4482.564	0.400535	0.097192	4.121074	3.77E-05	0.000164	3852.066	4046.468	3699.899	4982.107	4789.469	5525.377
14716	ZNF790	198.1921	0.400244	0.212119	1.886885	0.059176	0.106903	162.0838	203.6372	147.4951	214.2842	222.4675	239.1851
10538	INTS9	752.8032	0.400007	0.146156	2.736846	0.006203	0.015649	645.6249	723.131	582.3272	828.1191	765.8016	971.8151
22874	LOC11962	18.26981	0.399765	0.686036	0.582717	0.560084	0.650971	21.68345	14.23271	10.43597	17.85702	36.36488	9.044815
18962	LOC11962	77.88623	0.399647	0.334956	1.193131	0.232818	0.326414	88.90216	49.81448	62.61582	102.6778	74.86887	88.43819
24387	STMN2	4.690694	0.399645	1.146993	0.348429	0.727518	0.79309	3.794605	5.474118	2.782925	8.928508	2.139111	5.024897
7886	BCL2	3079.82	0.39961	0.115981	3.445474	0.00057	0.001922	2426.379	2992.7	2553.334	3272.298	3418.299	3815.907
15466	LOC10324	140.2033	0.399573	0.228271	1.750436	0.080043	0.137589	133.8953	107.8401	121.0573	151.7846	166.8506	159.7917
9529	TANGO2	556.42	0.399313	0.134136	2.976922	0.002912	0.008123	515.5241	470.7742	453.6169	607.1385	650.2896	641.1769
14693	LOC11962	174.0835	0.399259	0.211117	1.891178	0.058601	0.10603	138.7741	160.3917	149.5822	223.2127	194.6591	177.8814
24029	LOC10323{	6.530366	0.399204	0.992212	0.402338	0.687436	0.760559	6.505036	4.379295	5.565851	11.16063	8.556443	3.014938
5509	SLC33A1	3647.504	0.399197	0.090573	4.407481	1.05E-05	5.05E-05	3108.323	3293.229	3039.65	4111.578	4010.833	4321.412
19220	PRSS53	60.80062	0.399014	0.346654	1.151045	0.249714	0.345407	58.54533	45.43518	53.57132	51.33892	85.56443	70.34856
23040	LOC11962	13.081	0.399002	0.718014	0.555702	0.578415	0.667411	13.01007	10.40082	9.740239	22.32127	14.97377	8.039836
12169	ESRRA	1475.254	0.398948	0.168197	2.371909	0.017696	0.038661	1475.559	938.2638	1407.465	1555.793	1585.081	1889.361
7859	HM13	5167.008	0.398614	0.115493	3.45142	0.000558	0.001886	4109.015	5122.68	4143.776	5964.243	5435.48	6226.853
8803	PNKD	1608.587	0.398493	0.125595	3.172849	0.00151	0.004558	1546.572	1196.095	1422.771	1872.755	1704.871	1908.456
22995	LOC10321(	12.48281	0.398464	0.70572	0.56462	0.572332	0.661685	8.673382	9.306001	14.61036	13.39276	12.83466	16.07967
5754	BLOC1S2	2846.911	0.398426	0.092723	4.296927	1.73E-05	8.00E-05	2478.419	2472.112	2414.884	3171.852	3441.829	3102.372
10066	ADCY9	668.1214	0.398252	0.139882	2.847047	0.004413	0.011654	567.0223	578.0669	588.5887	774.5481	650.2896	850.2126
7373	SMYD4	1127.496	0.398101	0.110315	3.608763	0.000308	0.001109	1013.159	911.4407	996.2873	1247.759	1272.771	1323.558
20320	EFCC1	48.07291	0.397853	0.408192	0.974671	0.329724	0.431383	40.11439	47.62483	35.4823	46.87467	79.14709	39.1942
5945	SEC24C	9161.935	0.397471	0.094908	4.187957	2.81E-05	0.000126	8150.811	7665.408	7914.64	10053.5	9758.623	11428.63
25479	TRNAT-AG	1.249988	0.397422	2.366977	0.167903	0.86666	0.904244	2.710432	0.547412	0	2.232127	0	2.009959
7511	MAP3K7	2472.19	0.397342	0.111775	3.554821	0.000378	0.001339	1993.252	2406.422	2003.706	2810.248	2691.001	2928.51
9911	QSER1	2397.535	0.39724	0.13786	2.881484	0.003958	0.010617	1875.077	2529.043	1805.423	2709.802	2609.715	2856.152
17513	KCNJ4	126.6793	0.397193	0.281232	1.41233	0.157853	0.239623	113.2961	76.63765	138.4505	131.6955	158.2942	141.7021
22887	LOC11962(	14.34514	0.396871	0.682245	0.581713	0.56076	0.651366	16.80468	12.04306	8.348776	20.08914	10.69555	18.08963
22734	SLC2A4	17.63438	0.396594	0.656978	0.603665	0.546067	0.638567	17.34676	16.42235	11.1317	15.62489	34.22577	11.05477
15273	LOC10322	147.9705	0.396496	0.222193	1.784464	0.074348	0.129415	134.4374	126.4521	123.1445	145.0883	181.8244	176.8764
9812	HMGN3	1182.87	0.39613	0.136484	2.902391	0.003703	0.010034	889.0216	1207.043	967.7623	1337.044	1364.753	1331.598
11234	LOC11962(	427.0683	0.396052	0.153884	2.573706	0.010062	0.023808	355.0666	358.5547	396.5669	468.7467	442.7959	540.679
5393	GMFB	3556.845	0.395989	0.088659	4.466424	7.95E-06	3.92E-05	3200.478	2943.433	3073.045	4037.918	3963.772	4122.426
21703	LOC11962{	26.53713	0.395909	0.509309	0.777346	0.436955	0.535246	20.0572	20.80165	27.13352	40.17829	29.94755	21.10457
8985	NIN	5276.332	0.395831	0.126814	3.12135	0.0018	0.005327	4085.163	5490.54	4096.466	5807.994	5933.893	6243.937
22772	CDK15	15.87503	0.39578	0.663044	0.596914	0.550565	0.642751	13.55216	11.49565	15.30609	22.32127	23.53022	9.044815
8991	PYROXD1	631.1544	0.395502	0.126767	3.119926	0.001809	0.005349	543.1705	549.6015	540.5833	703.12	770.0798	680.3711
21852	LOC10322	38.98463	0.395282	0.525414	0.752324	0.451856	0.549725	28.73058	33.93953	36.87376	42.41041	74.86887	17.08465
11449	CCDC22	1078.942	0.395248	0.156429	2.526694	0.011514	0.026736	1058.695	730.7948	1009.506	1225.438	1116.616	1332.603

7282	TEP1	5533.18	0.395202	0.108601	3.639016	0.000274	0.000999	4859.804	4511.768	4976.566	6051.296	5696.452	7103.195
24638	TRNAQ-UU	3.740357	0.395153	1.27806	0.309182	0.757183	0.817019	4.336691	1.642235	3.478657	6.696381	4.278221	2.009959
20191	LOC10323	45.60372	0.395035	0.395672	0.99839	0.31809	0.418822	33.60935	45.43518	39.65669	60.26743	36.36488	58.28881
12518	BBC3	860.1788	0.394773	0.171777	2.298177	0.021552	0.04577	826.6817	547.4118	857.141	988.8323	921.9567	1019.049
8401	TRADD	816.5158	0.394753	0.120253	3.282699	0.001028	0.003254	700.9177	699.0449	719.3862	948.654	849.2269	981.8649
8795	ACSF2	1352.137	0.394736	0.124297	3.175743	0.001495	0.004518	1175.785	1280.396	1052.642	1444.186	1467.43	1692.385
19092	LOC119624	66.08128	0.394666	0.337001	1.171112	0.241554	0.336349	67.7608	42.15071	61.22436	78.12444	74.86887	72.35852
24629	TRNAW-CC	3.77271	0.394289	1.26872	0.310777	0.75597	0.816008	1.626259	3.284471	4.87012	6.696381	2.139111	4.019918
25294	LOC11961	1.572243	0.394042	1.974178	0.199598	0.841795	0.88476	1.626259	1.642235	0.695731	4.464254	0	1.004979
23215	ASB16	17.54865	0.393933	0.746841	0.527465	0.59787	0.68466	12.46799	12.59047	19.48048	26.78552	29.94755	4.019918
23199	CA13	14.56057	0.39378	0.743333	0.529749	0.596286	0.683306	9.757555	19.70683	7.653045	13.39276	27.80844	9.044815
18076	HBQ1	99.8515	0.39359	0.296281	1.328435	0.184034	0.270666	95.4072	64.59459	100.1853	118.3027	91.98176	128.6374
6577	GALK2	2327.682	0.393319	0.100596	3.909884	9.23E-05	0.000373	2100.585	1967.945	1973.79	2537.928	2532.707	2853.137
17274	LOC11962(	104.1561	0.393161	0.270377	1.454119	0.145913	0.224563	85.10756	106.1979	77.92191	133.9276	111.2338	110.5477
13202	TECPR2	251.4761	0.393111	0.181284	2.168479	0.030122	0.060657	237.4338	195.426	218.4597	285.7123	299.4755	272.3494
9086	ALDH3B1	731.8724	0.393058	0.127299	3.087664	0.002017	0.005903	677.0659	595.0366	628.2454	770.0838	853.5052	867.2973
24378	LOC11962	5.003645	0.392866	1.123578	0.349656	0.726597	0.792313	6.505036	1.642235	4.87012	6.696381	4.278221	6.029877
12950	PARD6A	396.8715	0.392855	0.17753	2.212899	0.026905	0.055232	363.74	276.9904	387.5224	488.8358	447.0741	417.0665
8757	PPP2R1A	7756.508	0.392572	0.123211	3.186191	0.001442	0.004377	7345.27	5848.548	6935.746	8508.868	7871.927	10028.69
7126	H6PD	8973.781	0.392543	0.106437	3.688032	0.000226	0.000843	7847.784	7681.83	7759.492	10062.43	8982.126	11509.02
21800	LOC11962	26.9738	0.39242	0.516522	0.759736	0.447412	0.545617	22.22554	28.46541	18.78475	22.32127	44.92132	25.12449
24334	LOC11962	5.323367	0.3924	1.099268	0.356965	0.721118	0.787825	2.168345	3.284471	8.348776	6.696381	6.417332	5.024897
7974	DYNC1LI1	4037.995	0.392011	0.114683	3.418208	0.00063	0.002102	3335.457	4015.813	3128.008	4609.342	4378.76	4760.588
22517	NMB	19.63481	0.39172	0.614177	0.637798	0.523605	0.61825	25.47806	9.853413	16.00182	20.08914	19.252	27.13445
8663	OGA	10447.09	0.391667	0.121893	3.213219	0.001313	0.004028	8010.952	10778.54	8325.122	11424.03	11722.33	12421.55
9320	RABGAP1L	909.5278	0.391594	0.129404	3.026146	0.002477	0.007065	777.3519	878.596	706.1673	1006.689	998.9647	1089.398
22627	LOC10322	16.58433	0.391584	0.632018	0.619577	0.535536	0.629214	10.84173	11.49565	20.87194	20.08914	17.11289	19.09461
22076	LOC11962	20.28688	0.391379	0.548836	0.713107	0.47578	0.572957	16.80468	17.51718	18.78475	20.08914	21.39111	27.13445
21450	LOC10321	44.36442	0.391253	0.482291	0.811238	0.417229	0.517111	20.59928	61.85754	32.69937	60.26743	38.50399	52.25893
14772	LOC10323	487.323	0.390828	0.208114	1.87795	0.060388	0.10868	541.0022	308.7403	414.6559	491.0679	643.8723	524.5993
5055	ARPC3	6732.574	0.390638	0.083865	4.657936	3.19E-06	1.68E-05	6011.196	5551.303	5915.804	7658.428	7758.554	7500.162
9901	SLC25A16	551.4986	0.390242	0.135281	2.884674	0.003918	0.010521	461.8576	451.0673	519.0156	638.3883	624.6203	614.0425
13463	LOC10324	454.0849	0.390104	0.184457	2.114881	0.03444	0.068008	469.9889	314.7618	397.2626	468.7467	489.8563	583.8931
7715	CEP170B	2165.293	0.389856	0.111751	3.488601	0.000486	0.001673	1931.996	1722.158	1975.181	2395.072	2276.014	2691.335
17390	LOC10323	129.5143	0.389778	0.27247	1.430535	0.152563	0.233232	127.3903	82.11177	125.9274	174.1059	136.9031	130.6473
16205	LOC10322	193.9194	0.389178	0.23926	1.626592	0.103824	0.170327	196.2353	154.9175	155.8438	169.6417	207.4937	279.3843
10438	NAPRT	3209.954	0.389041	0.1411	2.757199	0.00583	0.014847	3120.249	2221.945	3001.385	3575.867	3294.23	4046.047
23970	C4BPA	6.562161	0.389003	0.945201	0.411556	0.680665	0.754922	7.047123	4.379295	5.565851	8.928508	6.417332	7.034856
21443	LOC10323	31.36742	0.388376	0.478088	0.812352	0.41659	0.516439	24.93597	33.39212	22.95914	24.5534	49.19955	33.16432
22356	LOC10324	24.95823	0.388265	0.582792	0.666215	0.505274	0.600827	17.88885	12.04306	35.4823	20.08914	32.08666	32.15934
10169	ORAI2	1750.547	0.388244	0.13758	2.821947	0.004773	0.012479	1685.889	1234.961	1631.49	1973.2	1848.192	2129.551
7155	OSBPL10	2502.955	0.388097	0.105507	3.678409	0.000235	0.000872	2114.679	2378.504	2014.142	2796.855	2733.783	2979.764
13752	B3GALT1	259.6462	0.388072	0.188589	2.05777	0.039612	0.076577	236.8917	249.0724	188.5432	305.8014	278.0844	299.4839
11034	DZANK1	399.6228	0.387799	0.147866	2.622646	0.008725	0.021022	332.2989	369.503	337.4297	433.0326	464.187	461.2856
22839	LOC11962	15.57769	0.387548	0.659991	0.587203	0.557068	0.648437	19.51511	11.49565	9.044508	20.08914	19.252	14.06971
17415	DES	94.54138	0.387521	0.271551	1.427067	0.153561	0.234419	80.77087	90.87036	73.05179	113.8385	111.2338	97.48301
14554	MFSD9	204.0662	0.387483	0.202232	1.916033	0.055361	0.101125	188.104	158.7494	185.0645	243.3018	198.9373	250.2399
8853	LOC10322	3137.685	0.387481	0.122768	3.156209	0.001598	0.0048	2997.738	2352.776	2811.45	3361.583	3351.986	3950.574
5491	ARFIP1	3097.679	0.387434	0.087771	4.414143	1.01E-05	4.91E-05	2787.408	2624.292	2640.996	3488.814	3523.115	3521.448
10928	FLCN	2204.481	0.387219	0.146376	2.645366	0.00816	0.019852	2188.403	1511.404	2035.014	2470.965	2267.457	2753.644
14808	NAT14	695.8118	0.387115	0.206885	1.871161	0.061323	0.110094	689.5339	410.0114	712.4289	792.4051	660.9852	909.5064
7661	HACD2	4201.315	0.386825	0.110325	3.506229	0.000455	0.001577	3445.501	4132.959	3346.468	4495.504	4926.372	4861.086
21650	LOC11962	31.67322	0.386501	0.493574	0.783066	0.433588	0.532418	33.60935	21.34906	26.43779	53.57105	29.94755	25.12449
15510	LRRC56	163.4587	0.386359	0.221804	1.741895	0.081527	0.139742	139.3162	121.5254	164.8883	185.2665	181.8244	187.9312
7546	SIAE	1156.936	0.386284	0.108993	3.544114	0.000394	0.001388	1002.86	1023.113	986.5471	1294.634	1238.545	1395.916
21432	LOC10323	35.63407	0.38623	0.474731	0.813576	0.415888	0.515834	37.40396	20.80165	33.39511	46.87467	49.19955	26.12947
7810	LONP2	3567.627	0.386102	0.111499	3.462826	0.000535	0.00182	2925.64	3540.112	2813.538	4024.525	3991.581	4110.366
10258	PTOV1	2846.5	0.38601	0.137862	2.799965	0.005111	0.013245	2683.328	2056.079	2670.217	3113.817	2864.269	3691.29
10469	LOC10323(	1635.134	0.385856	0.140226	2.751669	0.005929	0.015057	1261.977	1717.231	1275.276	1874.987	1732.68	1948.655
22940	LOC10324	14.3408	0.385729	0.673009	0.573141	0.566549	0.65657	8.673382	13.6853	14.61036	15.62489	21.39111	12.05975
23729	LOC11962	8.131313	0.385561	0.857887	0.449431	0.653121	0.731741	8.673382	4.926706	7.653045	8.928508	8.556443	10.04979
23461	LOC11962(	9.708882	0.385346	0.785076	0.490839	0.62354	0.706561	7.047123	9.853413	8.348776	13.39276	8.556443	11.05477
24566	LOC11962	4.369786	0.385341	1.19975	0.321184	0.748071	0.809552	2.168345	6.02153	3.478657	2.232127	4.278221	8.039836
19250	PAPPA2	81.90811	0.385178	0.336477	1.144737	0.252318	0.348461	56.91907	80.46954	73.74753	98.21359	119.7902	62.30873
12634	NFATC1	301.9712	0.384675	0.169137	2.274342	0.022945	0.048283	240.1443	290.1283	256.0291	328.1227	348.675	348.7279
7083	PLOD3	4744.586	0.384565	0.103885	3.701823	0.000214	0.000803	4187.075	4063.438	4104.815	5263.355	4845.086	6003.747
24317	ANXA2R	5.303744	0.384474	1.071577	0.358793	0.71975	0.78688	4.878777	4.379295	4.87012	2.232127	6.417332	9.044815
6098	SQOR	5032.649	0.384323	0.093239	4.121913	3.76E-05	0.000164	4497.691	4356.303	4250.919	5531.211	5401.254	6158.514
20809	USP43	37.41793	0.384316	0.425509	0.903191	0.366424	0.468134	34.15144	27.37059	36.17803	31.24978	51.33866	44.2191
12060	LOC11962	325.7322	0.383981	0.160327	2.394992	0.016621	0.036639	277.5482	301.6239	269.248	381.6937	344.3968	379.8822
11976	USE1	428.524	0.383905	0.159283	2.41021	0.015943	0.035392	356.6928	408.3692	353.4315	482.1394	427.8221	542.6889
19228	LOC10323	3609.753	0.383658	0.333711	1.14967	0.25028	0.346042	3829.84	1853.536	3715.206	4060.239	3696.383	4503.313
11212	GPRASP1	454.2034	0.38346	0.148662	2.579407	0.009897	0.023467	401.1439	396.8736	381.9565	491.0679	581.8381	472.3404
17471	LOC10322	106.6829	0.383339	0.270745	1.415868	0.156814	0.238618	106.791	79.37471	91.83654	100.4457	139.0422	122.6075

12845	RNPEPL1	1766.355	0.383297	0.1719	2.229776	0.025762	0.05332	1734.676	1128.216	1740.024	2026.771	1694.176	2274.269
19734	LOC11962(	63.88583	0.383293	0.360396	1.063532	0.287541	0.387366	46.61943	53.09895	67.48594	46.87467	89.84265	79.39338
18004	LOC10324;	84.78086	0.383206	0.285528	1.342096	0.179565	0.265148	67.21871	81.01695	71.66033	109.3742	91.98176	87.43321
8648	TBC1D2	2453.626	0.382957	0.119086	3.215818	0.001301	0.003998	2250.201	1982.726	2163.725	2669.624	2487.786	3167.695
7161	TMEM214	3075.76	0.382941	0.104167	3.676211	0.000237	0.000879	2620.446	2829.572	2565.857	3245.513	3371.238	3821.937
8632	P3H1	1982.411	0.382744	0.11888	3.219581	0.001284	0.003954	1712.993	1791.679	1664.189	2241.055	1953.008	2531.543
24975	LOC11962;	2.786918	0.382724	1.523107	0.251278	0.801599	0.853305	2.168345	1.642235	3.478657	0	6.417332	3.014938
10029	NAP1L3	573.5263	0.382587	0.134002	2.85509	0.004302	0.011405	503.5982	524.4205	466.14	676.3345	605.3683	665.2964
12019	SAP30	364.4084	0.382205	0.159037	2.403246	0.01625	0.035944	311.1576	347.0591	289.4243	408.4792	436.3786	393.952
20190	FAM47E	45.3376	0.382198	0.3828	0.998429	0.318072	0.418818	42.28274	37.224	37.56949	60.26743	53.47777	41.20416
22286	PGBD5	20.62457	0.382188	0.565037	0.676395	0.49879	0.595007	22.22554	16.96977	13.91463	29.01765	23.53022	18.08963
24042	LOC11962‹	7.161024	0.38218	0.95417	0.400537	0.688761	0.761614	9.215468	3.284471	6.261582	4.464254	10.69555	9.044815
8048	PEPD	2010.286	0.38217	0.112392	3.400327	0.000673	0.002223	1850.683	1670.701	1721.239	2216.502	2077.076	2525.513
19058	LOC10323;	98.28703	0.382012	0.324852	1.175957	0.239612	0.334248	116.5486	69.5213	68.87741	95.98146	143.3204	95.47305
16079	ZNF527	143.9661	0.381244	0.23082	1.651693	0.098597	0.163021	140.4004	111.1246	124.5359	167.4095	145.4595	174.8664
12505	EFR3B	275.709	0.381088	0.16566	2.300415	0.021425	0.045545	237.4338	238.6716	242.1145	325.8905	301.6146	308.5287
13480	B3GAT3	1276.13	0.381064	0.180515	2.110978	0.034774	0.068581	1352.505	778.967	1196.658	1372.758	1369.031	1586.863
7624	MAPK3	1876.702	0.380882	0.108233	3.519075	0.000433	0.00151	1660.953	1598.442	1637.752	2093.735	1933.756	2335.572
11685	PLK3	354.7145	0.380604	0.153777	2.475039	0.013322	0.03031	306.8209	290.6757	327.6895	388.3901	410.7092	404.0017
23450	LOC10324;	11.18423	0.380596	0.772546	0.492652	0.622258	0.705447	8.131296	10.40082	10.43597	6.696381	21.39111	10.04979
25467	LOC11962!	1.538575	0.380558	2.245445	0.16948	0.865419	0.90334	3.252518	0	0.695731	0	4.278221	1.004979
8123	DVL3	1448.032	0.380227	0.11275	3.372315	0.000745	0.00244	1212.647	1344.443	1220.313	1696.417	1488.821	1725.55
22160	TMEM121	25.09757	0.380146	0.544264	0.698459	0.48489	0.581715	23.30971	13.6853	27.82925	35.71403	29.94755	20.09959
24645	LOC11962!	4.367161	0.380088	1.233441	0.308152	0.757967	0.817632	1.626259	3.284471	6.261582	4.464254	8.556443	2.009959
14559	ADI1	215.9217	0.379619	0.198354	1.913847	0.05564	0.101599	201.114	189.9519	173.9328	245.534	209.6328	275.3644
19285	CPA5	60.25754	0.379414	0.333252	1.13852	0.254904	0.351393	56.91907	50.36189	49.39693	60.26743	81.28621	63.31371
6823	ZZEF1	4169.691	0.379388	0.099595	3.809316	0.000139	0.000543	3660.167	3635.362	3585.799	4475.415	4436.516	5224.888
21926	DAPK2	30.55887	0.379164	0.51191	0.740683	0.458885	0.556393	29.27266	18.612	31.30791	24.5534	53.47777	26.12947
20613	LOC10324;	47.81201	0.379029	0.405447	0.934843	0.349869	0.451234	47.7036	33.93953	41.74388	53.57105	72.72976	37.18424
9543	ZNF304	937.0042	0.37882	0.127377	2.974002	0.002939	0.008189	779.5202	900.4924	763.2173	1142.849	979.7127	1056.233
7929	GNAI1	1856.517	0.378671	0.110379	3.430637	0.000602	0.002019	1534.104	1795.511	1514.607	2051.325	2072.798	2170.756
5187	CLTA	6425.354	0.378142	0.082438	4.586978	4.50E-06	2.30E-05	5592.163	5447.295	5723.782	7502.179	7106.126	7180.576
20092	CLEC11A	62.8757	0.377998	0.372791	1.013968	0.310598	0.410972	52.04029	38.31883	73.74753	87.05295	57.75599	68.3386
16938	LOC11962;	108.7352	0.377941	0.251717	1.501455	0.133238	0.209123	86.19173	97.4393	99.48959	133.9276	119.7902	115.5726
9124	IMP3	738.2634	0.37785	0.122896	3.074559	0.002108	0.006143	686.8234	601.6056	637.9857	837.0476	829.9749	836.1429
10739	ACYP1	452.7724	0.377736	0.140798	2.682828	0.0073	0.018072	415.2382	370.0504	396.5669	513.3892	504.8301	516.5594
11751	COMTD1	444.1218	0.377615	0.153578	2.458793	0.01394	0.031538	388.6759	353.0806	416.7431	558.0317	457.7697	490.43
7813	CUNH12or	1771.275	0.377476	0.109013	3.462663	0.000535	0.00182	1488.569	1678.912	1451.296	2107.128	2004.347	1897.401
10784	LOC10323(	491.0156	0.377455	0.141196	2.673274	0.007511	0.018516	427.1641	460.9207	392.3925	571.4245	545.4732	548.7188
11320	MYBL1	1142.866	0.377369	0.147814	2.552995	0.01068	0.025082	874.9274	1217.991	889.8404	1238.83	1324.11	1311.498
14900	STARD5	203.9773	0.377121	0.203308	1.854919	0.063608	0.113491	195.6932	153.8227	182.9774	209.8199	252.4151	229.1353
10099	SLC41A2	734.928	0.377098	0.132833	2.838896	0.004527	0.011917	601.1738	711.6354	607.3735	828.1191	782.9145	878.3521
7815	SLC23A2	3821.346	0.377027	0.108918	3.461572	0.000537	0.001827	3227.04	3393.406	3361.774	4205.327	3865.373	4875.155
10297	TBC1D8B	1941.537	0.376702	0.135048	2.789389	0.005281	0.013634	1495.616	2005.169	1570.961	2078.11	2098.468	2400.896
8760	NISCH	3119.807	0.376646	0.118238	3.185487	0.001445	0.004386	2921.846	2393.832	2833.714	3549.082	3172.301	3848.066
22943	CLEC4A	16.25205	0.37634	0.657298	0.572556	0.566945	0.656943	17.88885	12.59047	11.1317	22.32127	23.53022	10.04979
13681	MISP3	427.137	0.376305	0.181584	2.072345	0.038233	0.074295	317.1205	454.8992	341.6041	462.0503	536.9168	450.2308
7563	CC2D1B	2475.442	0.376248	0.106369	3.537186	0.000404	0.001421	2268.089	2062.648	2137.983	2790.159	2547.681	3046.093
5537	TAGLN2	18727.71	0.376081	0.085536	4.396775	1.10E-05	5.27E-05	16889.79	15542.12	16474.92	20819.05	20325.83	22314.56
9629	TMUB1	1109.883	0.37607	0.127331	2.953476	0.003142	0.008675	989.3076	856.6995	1052.642	1350.437	1152.981	1257.229
12172	BRF2	379.678	0.375948	0.158541	2.371304	0.017725	0.038711	352.8982	301.6239	339.5169	397.3186	421.4048	465.3055
10224	NHLRC3	689.4817	0.375792	0.133793	2.808748	0.004973	0.012932	571.9011	656.3468	571.8912	839.2797	716.6021	780.869
21758	LOC11962:	28.42624	0.375217	0.488967	0.767366	0.442864	0.541113	30.89892	25.72836	17.39328	29.01765	36.36488	31.15436
9531	ATAD2B	1676.44	0.374931	0.125968	2.976393	0.002917	0.008134	1334.075	1703.546	1342.762	1890.612	1828.94	1958.705
8436	MAPK12	1542.21	0.374684	0.114499	3.272368	0.001067	0.003361	1406.714	1284.228	1343.457	1732.131	1572.246	1914.486
16701	TTLL6	123.3391	0.374216	0.242558	1.542789	0.122882	0.19561	102.9964	121.5254	97.40239	131.6955	149.7377	136.6772
16182	LOC11962‹	152.5669	0.374146	0.229574	1.629741	0.103156	0.169473	138.232	122.0728	139.842	185.2665	139.0422	190.9461
6234	CD164	7750.899	0.374048	0.092142	4.059479	4.92E-05	0.00021	7042.244	6296.331	6921.831	8468.69	8509.382	9266.916
18584	LOC10321!	82.61229	0.373997	0.302253	1.237362	0.215953	0.308927	77.51835	73.35318	63.31155	93.74933	113.3729	74.36848
22417	MIXL1	21.30337	0.373845	0.571686	0.653934	0.513154	0.608565	17.88885	20.80165	16.00182	31.24978	27.80844	14.06971
7311	BPNT1	1948.147	0.373697	0.102906	3.631424	0.000282	0.001025	1667.458	1819.049	1607.835	2160.699	2128.415	2305.423
18341	LOC11961ŧ	92.2987	0.373554	0.292675	1.276346	0.201833	0.292554	76.43418	72.25836	92.53227	82.5887	130.4858	99.49297
19924	TFAP2E	73.91952	0.373379	0.357774	1.043616	0.296663	0.395844	79.6867	41.05589	73.74753	73.66019	74.86887	100.4979
10757	NAA80	493.1191	0.373366	0.139409	2.678208	0.007402	0.018292	406.0227	454.8992	429.962	553.5675	528.3603	585.903
23183	LOC11962:	14.09944	0.373286	0.700932	0.532556	0.594341	0.681558	15.17842	12.59047	8.348776	22.32127	17.11289	9.044815
9461	WDR20	1056.154	0.373278	0.124649	2.994632	0.002748	0.007721	918.2943	1003.406	840.4435	1241.063	1084.529	1249.189
11011	MVD	4096.142	0.37324	0.142086	2.626852	0.008618	0.020807	3940.426	2769.356	4000.455	4738.806	4237.578	4890.23
8610	CEBPZOS	1096.317	0.373215	0.11569	3.225996	0.001255	0.003876	917.7522	1035.156	910.0166	1290.169	1244.962	1179.846
7094	MPP5	4155.597	0.373215	0.100884	3.699443	0.000216	0.00081	3519.225	4003.77	3340.902	4613.806	4661.122	4794.757
8987	TOB2	5841.801	0.373151	0.119562	3.120983	0.001802	0.005332	4548.105	5926.828	4799.851	6473.168	6207.699	7095.155
22497	ABCA10	32.65225	0.373041	0.583351	0.63948	0.52251	0.617457	24.93597	25.72836	33.39511	33.4819	66.31243	12.05975
8529	OS9	9054.595	0.373004	0.114904	3.246211	0.00117	0.003645	7702.505	8851.649	7122.202	10078.05	9275.184	11297.98
18275	LOC10321!	84.20544	0.372926	0.289394	1.288644	0.197522	0.287339	80.77087	72.80577	65.39875	93.74933	109.0946	83.4133
25645	LOC11962‹	0.952124	0.372894	2.606266	0.143076	0.88623	0.918714	1.084173	0	1.391463	2.232127	0	1.004979

6111	TMEM30A	7096.881	0.372757	0.090603	4.114176	3.89E-05	0.000169	6210.141	6322.606	6029.208	7671.82	7698.659	8648.853
11439	S100A13	4660.372	0.37275	0.147429	2.528342	0.01146	0.026634	4634.296	3033.209	4518.775	5307.998	5078.249	5389.705
24734	TRNAQ-UU	3.44726	0.372589	1.270591	0.293241	0.769338	0.826912	2.710432	2.737059	3.478657	4.464254	4.278221	3.014938
6670	LOC10324(	5463.459	0.372567	0.096311	3.868361	0.00011	0.000437	5026.767	4515.053	4749.758	6008.886	5833.355	6646.934
6687	TRIO	10998.43	0.372535	0.096446	3.862616	0.000112	0.000446	9925.601	9707.801	9132.17	12013.31	11581.15	13630.54
16250	LOC10324(	171.0068	0.372296	0.230203	1.617249	0.105825	0.173129	149.6158	137.4004	163.4969	162.9453	175.4071	237.1752
7698	LOC10323(	5986.888	0.372289	0.106553	3.49394	0.000476	0.001644	4935.154	5784.501	4941.084	6627.185	6306.098	7327.305
10381	NAPA	2627.607	0.372191	0.134386	2.76957	0.005613	0.014375	2458.904	1944.407	2474.716	2758.909	2718.81	3409.895
17018	LOC11962:	106.0305	0.372134	0.249708	1.490274	0.136152	0.212694	94.32303	91.96518	90.44508	127.2312	117.6511	114.5677
11668	CCDC28A	356.5963	0.372021	0.150126	2.478061	0.01321	0.030096	313.868	301.6239	317.9492	395.0865	400.0137	411.0366
12286	LOC11962:	684.7978	0.37185	0.15841	2.347386	0.018906	0.040909	613.0997	684.8122	491.1863	720.977	862.0616	736.65
9051	STAT2	1852.263	0.371833	0.1201	3.096035	0.001961	0.005761	1737.929	1621.981	1490.257	1988.825	1920.921	2353.662
7818	SLC12A6	1351.664	0.371765	0.107409	3.461201	0.000538	0.001829	1188.253	1215.254	1134.738	1542.4	1420.369	1608.972
8887	KIAA2013	2780.534	0.371679	0.118089	3.147455	0.001647	0.004927	2529.917	2214.828	2535.245	3028.996	2823.626	3550.592
15165	METTL25	189.0605	0.371424	0.206013	1.802918	0.071401	0.12517	179.4306	149.9908	163.4969	212.0521	237.4413	191.9511
25442	TRNAD-GU	1.583767	0.371226	2.137332	0.173687	0.862112	0.900843	2.168345	0	2.087194	2.232127	0	3.014938
7768	ATXN1L	3719.061	0.371223	0.106904	3.472507	0.000516	0.001765	3061.162	3603.065	3068.871	4151.756	3957.355	4472.159
5668	PRSS23	6671.812	0.370962	0.08548	4.339745	1.43E-05	6.69E-05	5843.691	6043.974	5571.417	7432.983	7337.15	7801.656
24721	LOC11962{	4.408272	0.370939	1.254996	0.29557	0.767559	0.825434	1.084173	3.284471	6.957314	6.696381	6.417332	2.009959
8085	SLC10A3	3036.632	0.370833	0.109504	3.386489	0.000708	0.002328	2616.109	2648.378	2686.915	3502.207	2990.477	3775.708
21484	LOC10324!	34.76889	0.370647	0.459311	0.806964	0.419687	0.519335	28.18849	26.27577	37.56949	24.5534	42.78221	49.24399
8613	ZNF654	1327.638	0.370552	0.114883	3.225488	0.001258	0.003882	1169.28	1256.858	1045.684	1549.096	1486.682	1458.225
12819	MEAK7	330.0824	0.370405	0.16576	2.234587	0.025444	0.052765	316.5784	282.4645	266.4651	348.2118	365.7879	400.9868
22418	LOC10324:	19.76946	0.370345	0.566374	0.653887	0.513184	0.608574	14.63633	15.32753	21.56767	22.32127	25.66933	19.09461
13078	SLC12A9	1232.617	0.370063	0.168893	2.191108	0.028444	0.05782	1240.836	809.6221	1179.265	1372.758	1221.432	1571.788
22320	IZUMO1	25.00016	0.370028	0.550557	0.672097	0.501522	0.597355	22.22554	21.34906	20.87194	24.5534	44.92132	16.07967
23954	LOC11962:	8.708842	0.36974	0.892751	0.414158	0.678759	0.75331	7.589209	6.568942	8.348776	4.464254	19.252	6.029877
7455	MCM4	5423.741	0.369558	0.103239	3.579647	0.000344	0.001227	4689.589	4849.521	4667.662	5767.816	5685.756	6882.099
12455	LPIN3	521.7621	0.36924	0.159858	2.309794	0.0209	0.04461	495.4669	435.7398	439.7022	508.925	558.3079	692.4309
17035	SCAND1	407.2749	0.369156	0.248126	1.487775	0.13681	0.213508	415.7802	205.8268	445.9638	433.0326	455.6306	487.415
11589	PPP1R9B	3566.286	0.369132	0.147861	2.496484	0.012543	0.028774	2682.785	3940.818	2713.352	4247.738	3766.974	4046.047
7737	TP53INP1	1943.869	0.36906	0.106014	3.48123	0.000499	0.001715	1664.205	1800.985	1629.403	2075.878	2121.998	2370.747
25313	LOC11962:	1.862605	0.368969	1.867257	0.197599	0.843359	0.885738	1.084173	2.189647	1.391463	2.232127	4.278221	0
16507	LOC10322:	345.4025	0.368289	0.233914	1.574465	0.11538	0.185828	356.1507	198.7105	351.3443	412.9435	325.1448	428.1213
16802	LOC10322!	180.242	0.367887	0.241271	1.52479	0.127312	0.201439	184.8515	116.0513	171.1499	225.4448	183.9635	199.9909
7225	LENG8	5667.599	0.367597	0.100445	3.659677	0.000253	0.000929	4612.613	5341.097	4900.036	6191.92	6070.796	6889.134
11151	SBF1	5551.538	0.367594	0.141823	2.591921	0.009544	0.022754	5405.685	3802.322	5339.738	6392.812	5565.966	6802.706
16427	TBX19	139.5681	0.367592	0.231943	1.584837	0.113003	0.182881	132.2691	112.7668	121.753	133.9276	166.8506	169.8415
6923	TBC1D9	3308.467	0.367478	0.097601	3.765108	0.000166	0.000639	2775.482	3062.222	2835.105	3669.617	3531.672	3976.704
17095	ZNF565	113.3681	0.367438	0.24786	1.48244	0.138223	0.214956	97.57555	105.6505	92.53227	138.3919	130.4858	115.5726
8975	SPACA6	892.1468	0.367114	0.117524	3.123731	0.001786	0.00529	761.6313	832.066	741.6496	1037.939	1011.799	967.7952
10503	STX4	532.2833	0.367017	0.133762	2.743818	0.006073	0.015372	458.6051	482.8172	453.6169	562.496	633.1768	602.9877
23710	LOC11962:	10.03139	0.367014	0.810912	0.452594	0.650841	0.72977	4.878777	13.13788	8.348776	8.928508	12.83466	12.05975
12524	RABGEF1	855.8423	0.366983	0.159777	2.296848	0.021627	0.045909	642.3723	926.2208	672.0765	1024.546	958.3216	911.5164
23997	VSNL1	8.729009	0.36669	0.901436	0.406784	0.684166	0.757952	7.589209	9.853413	4.87012	8.928508	17.11289	4.019918
9471	CDKL2	863.661	0.366622	0.122524	2.992244	0.002769	0.007774	715.554	829.8763	718.6905	968.7431	956.1825	992.9197
14358	SLC2A8	336.8717	0.366596	0.187944	1.95056	0.051109	0.09464	325.7939	238.1241	321.4279	337.0512	380.7617	418.0715
13980	SYT3	238.1423	0.366555	0.181879	2.015384	0.043864	0.083415	214.1241	203.6372	207.3279	236.6055	288.7799	278.3793
24793	LOC11961{	5.306255	0.366389	1.298339	0.282199	0.777791	0.834009	4.878777	1.094824	7.653045	2.232127	14.97377	1.004979
17644	GLRA2	106.3803	0.366373	0.261927	1.398761	0.161885	0.243919	97.03346	99.62895	80.70484	122.767	132.6249	105.5228
10221	BRPF3	1493.349	0.366293	0.130321	2.810701	0.004943	0.012858	1213.189	1490.602	1214.747	1618.292	1552.994	1870.267
13955	SRD5A3	259.1146	0.366073	0.18116	2.02071	0.04331	0.082507	247.1914	200.9001	232.3743	276.7837	290.9191	306.5187
24908	LOC11961{	3.151929	0.366062	1.390788	0.263205	0.792393	0.845743	4.336691	1.642235	2.087194	6.696381	2.139111	2.009959
11518	DCDC2	1885.218	0.365755	0.14576	2.509288	0.012097	0.027922	1483.148	2045.131	1415.118	2033.468	2113.441	2221.005
13433	IFI44L	483.7628	0.365718	0.172448	2.120745	0.033943	0.067177	362.6558	519.4938	385.4352	533.4783	566.8643	534.6491
11328	HMBOX1	1074.229	0.365633	0.143303	2.551459	0.010727	0.025177	828.308	1123.836	863.4026	1158.474	1283.466	1187.886
17183	HOXA4	146.7769	0.365312	0.248488	1.470136	0.141525	0.218963	118.1748	151.6331	113.4042	203.1236	147.5986	146.727
23804	LOC11962:	9.055779	0.365267	0.837984	0.435887	0.662919	0.740367	8.673382	8.211177	6.957314	4.464254	14.97377	11.05477
21310	LOC11962:	35.54889	0.365035	0.439104	0.831317	0.405794	0.506243	32.52518	34.48694	27.13352	37.94616	29.94755	51.25395
7607	AHSA1	2924.176	0.364951	0.103586	3.523179	0.000426	0.00149	2433.426	2793.99	2442.713	3399.529	3084.598	3390.801
11159	IRAK4	548.5383	0.364896	0.140892	2.589897	0.0096	0.022872	520.945	426.9812	490.4906	618.2992	622.4812	612.0325
7391	CLPX	2645.839	0.36466	0.101228	3.602376	0.000315	0.001134	2137.447	2520.831	2281.303	2959.8	2996.894	2978.759
20726	LOC10322:	51.17319	0.364315	0.398433	0.91437	0.360522	0.462438	47.16151	30.65506	56.35424	46.87467	72.72976	53.26391
21810	LOC10321:	40.61783	0.364239	0.480275	0.758397	0.448213	0.546343	26.56223	51.45671	27.13352	55.80317	55.61688	27.13445
22185	LOC11962{	25.04999	0.364224	0.524638	0.69424	0.487532	0.584215	26.02015	20.80165	18.08902	26.78552	38.50399	20.09959
8758	RAB3D	1827.683	0.364204	0.114325	3.185695	0.001444	0.004383	1674.505	1546.438	1580.006	1993.289	1867.444	2304.418
10814	KMT2C	4296.032	0.36408	0.136514	2.66699	0.007653	0.018815	3315.4	4634.936	3321.422	4691.931	4725.295	5087.206
22319	LOC11962(	40.41192	0.363972	0.541482	0.672177	0.501471	0.597321	18.97302	66.23683	20.87194	37.94616	49.19955	49.24399
20713	LOC10324:	50.77524	0.363896	0.396603	0.917531	0.358865	0.4606	44.99317	32.2973	54.96278	71.42806	57.75599	43.21412
23209	LOC11962(	15.10894	0.363821	0.688832	0.52817	0.597381	0.684277	21.14137	8.758589	9.740239	20.08914	12.83466	18.08963
7989	LOC10321!	2474.338	0.363575	0.106455	3.41528	0.000637	0.00212	2255.079	2146.402	2097.63	2573.642	2691.001	3082.272
11387	PEX6	2235.21	0.363524	0.143136	2.539718	0.011094	0.025901	2267.005	1587.494	2014.838	2437.483	2299.544	2804.898
9104	LOC10322!	1157.026	0.363281	0.117872	3.082001	0.002056	0.006004	1070.078	903.7769	1061.686	1305.794	1330.527	1270.294
9226	AHCYL2	874.4511	0.363154	0.119107	3.048981	0.002296	0.006617	786.5673	757.618	749.9984	915.1721	1069.555	967.7952

7564	NDST1	3769.814	0.363086	0.102648	3.537188	0.000404	0.001421	3446.585	3159.114	3294.288	4203.095	3871.79	4644.01
22834	LOC11962	17.21339	0.363046	0.617168	0.588245	0.556368	0.64781	17.34676	14.78012	13.2189	11.16063	25.66933	21.10457
20370	LOC10322	47.35587	0.363039	0.375798	0.966048	0.33402	0.435932	44.99317	43.24553	36.17803	42.41041	62.03421	55.27387
24289	TIMD4	6.85677	0.363014	0.998302	0.363631	0.716134	0.783829	7.589209	3.831883	6.261582	4.464254	14.97377	4.019918
14308	RNH1	5618.574	0.362989	0.185224	1.959728	0.050028	0.092954	6007.401	3254.911	5487.233	6229.866	5525.323	7206.708
7047	CTDSP1	2654.522	0.362936	0.097737	3.713407	0.000204	0.000771	2374.338	2244.936	2353.659	2899.533	2840.739	3213.924
9840	PLCL2	712.271	0.362886	0.125286	2.896472	0.003774	0.010196	655.9245	621.8598	594.1546	787.9408	761.5234	852.2226
10548	TNK2	2293.747	0.362746	0.132668	2.734249	0.006252	0.015757	2201.413	1718.873	2105.979	2508.911	2303.822	2923.485
21267	SLC9A9	41.11272	0.362707	0.431871	0.839852	0.400992	0.501254	35.23561	48.71965	23.65487	44.64254	49.19955	45.22408
11681	PHYKPL	479.6985	0.362695	0.146507	2.475608	0.013301	0.030272	452.6421	412.7485	396.5669	502.2286	524.0821	589.9229
24909	LOC10321	4.380155	0.362296	1.37864	0.262792	0.792711	0.846048	2.168345	5.474118	3.478657	4.464254	10.69555	0
22930	LOC11962	16.12224	0.362195	0.630563	0.574399	0.565698	0.655869	14.09425	13.6853	14.61036	24.5534	10.69555	19.09461
10430	LOC10323	1909.661	0.362142	0.131183	2.760581	0.00577	0.014707	1804.606	1485.128	1730.284	2069.182	1899.53	2469.235
5976	STMN1	4699.677	0.362099	0.086745	4.174297	2.99E-05	0.000133	4048.301	3995.559	4292.663	5430.765	5311.412	5119.365
5954	TMEM64	4442.21	0.362055	0.086525	4.184388	2.86E-05	0.000128	3925.247	4034.425	3703.378	5026.75	4928.511	5034.947
13052	LOC10322	336.886	0.362023	0.164854	2.196024	0.02809	0.057216	271.0432	303.8136	310.2962	339.2833	414.9875	381.8922
19961	STMN4	57.18195	0.362019	0.349442	1.035992	0.300206	0.399815	40.65648	58.57306	50.78839	58.0353	72.72976	62.30873
22079	LOC11962	28.93011	0.361991	0.507981	0.712608	0.476088	0.573251	16.80468	33.39212	25.04633	35.71403	38.50399	24.11951
23067	LOC10322	19.75683	0.361672	0.656321	0.551059	0.581593	0.670264	13.55216	21.34906	16.00182	20.08914	38.50399	9.044815
8333	FBXO31	2057.847	0.361597	0.109482	3.302792	0.000957	0.003054	1790.511	1802.627	1817.25	2283.466	2083.494	2569.733
19301	LOC10321	69.30938	0.361576	0.318424	1.13552	0.256158	0.352844	65.59245	57.47824	57.7457	95.98146	72.72976	66.32864
19225	MPZ	80.09283	0.361399	0.314256	1.150017	0.250137	0.345898	61.25576	88.1333	61.92009	75.89232	89.84265	103.5129
22759	LOC10321	16.63924	0.361323	0.60247	0.599736	0.548682	0.64092	15.17842	15.32753	13.2189	15.62489	21.39111	19.09461
10109	ABI1	3687.648	0.361293	0.127353	2.83693	0.004555	0.011979	2843.243	3877.318	2963.12	4111.578	4250.413	4080.217
9754	HSPA4L	4846.401	0.361161	0.123867	2.915718	0.003549	0.009672	3792.436	5073.413	3863.396	5334.783	5439.758	5574.621
8168	GAK	2768.21	0.361103	0.107522	3.358407	0.000784	0.002552	2422.584	2448.026	2405.143	3209.799	2735.923	3387.786
6865	ANKZF1	5070.082	0.360939	0.095219	3.790617	0.00015	0.000582	4619.66	4395.169	4308.664	5573.621	5347.777	6175.599
24388	CYP2C9	6.29813	0.360384	1.03469	0.348302	0.727614	0.793161	5.420864	4.379295	6.261582	11.16063	8.556443	2.009959
20491	LOC11962	52.27237	0.360012	0.379622	0.948342	0.342955	0.44495	42.82482	56.38342	36.87376	71.42806	59.8951	46.22906
8510	LOC10323	1299.451	0.35987	0.110588	3.254137	0.001137	0.003553	1227.284	1066.358	1122.91	1412.936	1456.734	1510.484
18392	CUNH15or	113.2519	0.359718	0.284112	1.266115	0.205472	0.297003	72.09749	124.2625	101.5768	118.3027	132.6249	130.6473
9822	RCCD1	843.9574	0.359657	0.124012	2.900177	0.00373	0.010095	800.6616	678.2432	740.9539	933.0291	921.9567	988.8998
11992	FOXP2	681.5556	0.359604	0.149334	2.408051	0.016038	0.035554	544.7968	703.4242	544.0619	772.3159	718.7412	805.9935
13087	CEP162	679.3358	0.359388	0.164099	2.190067	0.028519	0.057938	539.3759	736.2689	510.6668	749.9947	733.715	805.9935
9456	INPP5F	2366	0.359261	0.119916	2.995954	0.002736	0.007692	1949.885	2405.328	1863.864	2660.695	2594.741	2721.484
8080	SH3GL1	2530.637	0.359181	0.10601	3.388173	0.000704	0.002315	2210.628	2138.191	2308.437	2883.908	2558.376	3084.282
11981	TMED1	986.8281	0.359117	0.149054	2.409307	0.015983	0.035465	989.8497	692.4759	912.1038	1142.849	1063.138	1120.552
18904	GPX6	71.16101	0.359089	0.29835	1.203585	0.22875	0.321695	62.33993	63.49977	61.22436	80.35657	79.14709	80.39836
18169	GATA6	133.7685	0.359019	0.27423	1.30919	0.19047	0.278697	102.9964	153.2753	95.3152	124.9991	173.268	152.7569
14406	PLA2G4C	220.0471	0.358599	0.184634	1.942213	0.052111	0.096167	193.5248	187.2148	197.5877	267.8552	228.8848	245.215
17906	LOC10323	116.5551	0.358589	0.263604	1.360336	0.173724	0.257923	100.8281	104.0082	100.1853	169.6417	109.0946	115.5726
23362	LOC10321	11.95881	0.358523	0.710395	0.504681	0.613783	0.69843	10.84173	12.04306	8.348776	15.62489	12.83466	12.05975
5843	FTL	205395.4	0.35851	0.084302	4.252669	2.11E-05	9.61E-05	179990	188873.5	171156.9	228851.1	221733.8	241766.9
10439	NAGK	1141.266	0.358316	0.129955	2.757227	0.005829	0.014847	1027.254	1007.238	972.6325	1227.67	1129.45	1483.35
10638	ING4	630.5009	0.358194	0.132225	2.708971	0.006749	0.016867	571.9011	515.1145	571.1955	756.691	654.5679	713.5354
12089	LZTFL1	682.6896	0.358067	0.149869	2.389202	0.016885	0.037131	567.0223	690.8337	533.626	832.5834	778.6363	693.4358
13075	LOC10323	914.2676	0.35803	0.16334	2.19194	0.028384	0.057712	885.7691	603.7952	914.8868	1044.635	1022.495	1014.024
7394	SELENOS	1486.32	0.357889	0.099374	3.601429	0.000316	0.001138	1337.869	1312.694	1258.578	1685.256	1632.141	1691.38
8833	INAVA	3011.779	0.357453	0.113041	3.162148	0.001566	0.004713	2835.112	2450.215	2643.083	3220.959	3129.519	3791.788
7727	S100A16	13157.95	0.35724	0.102546	3.483717	0.000495	0.001701	12134.06	10331.85	12150.95	14631.59	13833.63	15865.61
10219	CHIC1	1039.18	0.357087	0.127022	2.811223	0.004935	0.012839	874.9274	1018.733	842.5307	1080.349	1174.372	1244.165
26591	TRNAN-GU	0.399407	0.356982	4.013031	0.088956	0.929117	NA	0	0	1.391463	0	0	1.004979
27138	LOC10323	0.399407	0.356982	4.013031	0.088956	0.929117	NA	0	0	1.391463	0	0	1.004979
27658	TRNAT-CG	0.399407	0.356982	4.013031	0.088956	0.929117	NA	0	0	1.391463	0	0	1.004979
28539	GSG1L2	0.399407	0.356982	4.013031	0.088956	0.929117	NA	0	0	1.391463	0	0	1.004979
28633	LOC11962	0.399407	0.356982	4.013031	0.088956	0.929117	NA	0	0	1.391463	0	0	1.004979
28708	LOC11962	0.399407	0.356982	4.013031	0.088956	0.929117	NA	0	0	1.391463	0	0	1.004979
29079	LOC11962	0.399407	0.356982	4.013031	0.088956	0.929117	NA	0	0	1.391463	0	0	1.004979
29514	LOC11962	0.399407	0.356982	4.013031	0.088956	0.929117	NA	0	0	1.391463	0	0	1.004979
16323	LOC11962	220.3981	0.356596	0.222742	1.600937	0.109391	0.178163	219.0029	167.508	195.5005	267.8552	186.1026	286.4191
10725	USP35	1129.646	0.356165	0.132609	2.685829	0.007235	0.017934	1067.368	902.6821	1008.115	1232.134	1129.45	1438.126
9847	ELOVL6	2532.424	0.356003	0.122978	2.894857	0.003793	0.010241	2003.551	2606.775	2053.799	2752.213	2939.138	2839.067
25227	LOC11962	2.489279	0.355941	1.69105	0.210485	0.833289	0.878122	3.252518	1.642235	1.391463	2.232127	6.417332	0
13674	ZNF786	284.2155	0.355663	0.171516	2.073641	0.038113	0.074099	234.1813	275.8956	238.6359	312.4978	314.4493	329.6333
16351	DNAJC28	178.6764	0.355632	0.22285	1.595839	0.110525	0.179701	135.5216	189.9519	144.0164	203.1236	207.4937	191.9511
11741	LOC11961	3622.118	0.355543	0.144516	2.460236	0.013885	0.031439	2758.678	4008.697	2764.836	4153.988	4122.066	3924.445
9167	SYVN1	1011.507	0.355535	0.116095	3.06246	0.002195	0.006366	845.6547	960.1603	858.5325	1109.367	1114.477	1180.851
10995	CAMKK2	671.1124	0.355386	0.135119	2.630164	0.008534	0.020634	597.3792	638.2822	532.2345	698.6557	772.219	787.9039
11361	MFSD13A	642.7404	0.355309	0.139567	2.545794	0.010903	0.025513	616.8943	503.0715	574.6741	680.7987	710.1847	770.8193
18297	CUNH22or	99.30397	0.355278	0.276629	1.284312	0.199033	0.289189	84.56547	104.0082	72.35606	120.5349	104.8164	109.5428
6974	LOC10324	2244.551	0.355189	0.094825	3.745745	0.00018	0.000686	1925.491	2054.984	1931.35	2477.661	2457.838	2619.981
9396	PTPN23	1328.204	0.355093	0.118043	3.00817	0.002628	0.007436	1119.95	1259.047	1122.215	1363.83	1478.125	1626.057
9651	LOC10323	1060.664	0.355006	0.120491	2.946314	0.003216	0.008859	850.5335	948.1173	990.7215	1243.295	1227.85	1103.467
9463	YWHAH	5679.637	0.354896	0.118528	2.994195	0.002752	0.007731	4533.468	5874.824	4545.213	6504.418	6164.917	6454.983

12163	LOC10322!	2503.502	0.354815	0.149514	2.373119	0.017639	0.038547	2516.907	1751.17	2328.613	2640.606	2547.681	3236.034
12075	AGRN	11416.84	0.354584	0.148197	2.392653	0.016727	0.036824	11147.46	7780.364	11139.35	12959.73	11001.45	14472.71
12820	PIM2	317.8821	0.354482	0.158633	2.234608	0.025443	0.052765	275.922	276.9904	285.9456	350.4439	340.1186	377.8723
18324	FBXL15	102.9172	0.354439	0.27711	1.279055	0.200878	0.291425	86.19173	81.01695	105.7512	111.6063	96.25998	136.6772
20774	FAM177B	52.30827	0.354331	0.390491	0.9074	0.364195	0.466069	59.6295	30.65506	47.30973	55.80317	64.17332	56.27885
9893	DCTN1	7131.584	0.354269	0.122751	2.88607	0.003901	0.010483	5774.846	7411.409	5596.463	8111.549	7448.383	8446.852
9485	GSTT2B	961.9222	0.354082	0.118497	2.988103	0.002807	0.007868	884.1429	841.372	811.9185	1031.243	1031.051	1171.806
21128	LOC11962;	43.73632	0.353952	0.411109	0.860969	0.389255	0.489793	37.94605	38.31883	37.56949	49.10679	66.31243	33.16432
22639	LOC10323!	20.73928	0.353704	0.572969	0.617318	0.537025	0.630625	15.17842	17.51718	21.56767	17.85702	34.22577	18.08963
12840	LOC10322;	306.5397	0.353687	0.158549	2.23078	0.025696	0.053202	274.2957	269.3266	264.3779	341.5154	337.9795	351.7428
11180	FOXO1	1509.41	0.35367	0.136799	2.585329	0.009729	0.023134	1201.263	1572.714	1204.311	1683.024	1570.107	1825.043
10711	RERE	1274.339	0.353605	0.131488	2.689266	0.007161	0.017774	1001.776	1301.745	1054.033	1345.973	1480.265	1462.245
22620	CAGE1	20.42397	0.353242	0.568045	0.621855	0.534037	0.627647	21.68345	14.78012	17.39328	15.62489	29.94755	23.11453
16047	LOC11962;	577.8741	0.35304	0.213018	1.657327	0.097453	0.161451	380.0025	711.0879	431.3534	638.3883	648.1505	658.2616
8124	TMED5	3307.367	0.353033	0.104687	3.37227	0.000746	0.00244	2734.826	3231.919	2746.052	3859.348	3595.845	3676.215
19198	VSIG8	70.07904	0.353022	0.306017	1.153602	0.248663	0.344344	59.08741	66.78424	58.44144	87.05295	72.72976	76.37844
24293	LOC10324(	6.336998	0.352803	0.971656	0.363095	0.716534	0.784138	6.505036	4.926706	5.565851	6.696381	4.278221	10.04979
23228	LOC10324(	23.5924	0.352783	0.672067	0.524923	0.599637	0.686248	8.131296	30.65506	22.95914	46.87467	12.83466	20.09959
12805	AGPAT2	1103.468	0.352573	0.157562	2.237675	0.025242	0.052407	1091.762	768.0188	1051.946	1214.277	1105.92	1388.882
12004	PIAS1	2203.095	0.352154	0.146413	2.405203	0.016163	0.035797	1715.703	2421.75	1668.364	2573.642	2365.856	2473.254
23429	LOC10323(	12.32428	0.351708	0.709983	0.495374	0.620336	0.703898	10.84173	13.13788	8.348776	17.85702	10.69555	13.06473
11731	PIGQ	873.732	0.351576	0.142719	2.46341	0.013762	0.031188	803.9141	699.5923	805.6569	941.9576	855.6443	1135.627
21339	TMEM220	48.09746	0.351425	0.425481	0.825946	0.408834	0.509346	39.5723	49.81448	36.17803	42.41041	83.42532	37.18424
14724	LOC10324{	237.5716	0.351227	0.186368	1.88459	0.059485	0.107402	185.9356	217.3225	224.0255	276.7837	245.9977	275.3644
25311	IGFBP1	3.204257	0.351079	1.776054	0.197674	0.8433	0.885738	5.420864	2.737059	0	8.928508	2.139111	0
24840	LOC10321!	3.77607	0.350882	1.276259	0.27493	0.78337	0.838402	3.252518	1.642235	4.87012	4.464254	6.417332	2.009959
12152	HS1BP3	1349.226	0.350867	0.147728	2.375093	0.017545	0.038382	1323.233	977.6775	1262.057	1372.758	1431.065	1728.565
7663	MYO5A	7429.807	0.350864	0.100116	3.504555	0.000457	0.001587	6332.111	7132.776	6132.176	7964.229	8060.169	8957.382
10025	IGFBP2	3568.502	0.350411	0.122713	2.855533	0.004296	0.011394	3434.117	2685.602	3297.071	3915.151	3679.27	4399.8
25098	LOC11962;	2.844644	0.350322	1.520294	0.23043	0.817758	0.866208	3.794605	2.737059	0.695731	6.696381	2.139111	1.004979
10598	PRKACA	1130.768	0.350298	0.12866	2.72266	0.006476	0.016245	885.7691	1122.742	976.8068	1218.741	1227.85	1352.702
12135	ACP2	1311.049	0.350109	0.14718	2.378788	0.01737	0.03805	1270.65	978.2249	1214.747	1426.329	1274.91	1701.43
13615	LOC11962:	872.4282	0.350087	0.167785	2.086523	0.036931	0.072113	961.6612	622.9546	715.9076	915.1721	1063.138	955.7355
9624	SPPL3	1289.834	0.350072	0.118494	2.954343	0.003133	0.008655	1177.954	1200.474	1026.9	1419.633	1358.335	1555.708
21634	LOC10322(	36.51564	0.350016	0.446302	0.784259	0.432888	0.531932	37.94605	26.82318	30.61218	37.94616	55.61688	30.14938
20162	LOC11961{	53.59222	0.349956	0.349363	1.001696	0.31649	0.417323	45.53526	50.36189	45.91827	49.10679	66.31243	64.31869
16252	CUNH9orf:	1186.694	0.349919	0.216462	1.616539	0.105978	0.173359	1265.23	628.4288	1238.402	1392.847	1155.12	1440.136
20020	TMED6	72.61002	0.349864	0.341367	1.024891	0.305415	0.405567	73.72375	61.85754	55.65851	55.80317	111.2338	77.38342
13446	TREX1	500.0721	0.34985	0.165185	2.117922	0.034182	0.06758	471.0731	363.4814	487.7077	540.1747	521.943	616.0524
8027	PKM	34304.46	0.349824	0.102769	3.403979	0.000664	0.0022	32200.47	27610.36	30692.89	37718.48	35355.22	42249.34
17851	LOC10323{	230.6543	0.34961	0.255397	1.368892	0.171033	0.254701	241.2284	130.284	235.8529	270.0874	282.3626	224.1104
6631	UBR2	5837.084	0.349585	0.089934	3.887113	0.000101	0.000407	5181.262	5338.907	4884.034	6403.972	6306.098	6908.229
15171	PRR5	602.8501	0.349368	0.193895	1.801843	0.07157	0.125415	611.4734	394.1365	587.893	700.8879	551.8906	770.8193
20149	TDRD12	63.36552	0.349358	0.347995	1.003917	0.315419	0.41617	54.20864	58.02565	53.57132	98.21359	59.8951	56.27885
9455	MAP4K2	877.3278	0.349324	0.116592	2.996109	0.002734	0.007689	780.6044	776.23	760.4344	1004.457	909.122	1033.119
23728	LOC11962;	12.04829	0.349298	0.777225	0.449417	0.653131	0.731741	5.96295	8.758589	16.69755	17.85702	14.97377	8.039836
14411	LOC10323:	319.396	0.349287	0.179908	1.941472	0.052201	0.096299	272.6694	316.404	251.159	345.9797	408.5701	321.5934
10187	GPSM3	755.4002	0.34907	0.123852	2.818439	0.004826	0.012594	636.9515	712.7302	642.8558	814.7263	892.0091	833.128
7770	ADAM10	9634.701	0.348972	0.100517	3.471792	0.000517	0.001769	8058.114	9298.884	8073.267	10461.98	10276.29	11639.67
12281	ZSCAN30	387.8671	0.348706	0.148432	2.349266	0.01881	0.040721	350.1878	326.2574	346.4742	462.0503	417.1266	425.1063
9023	CENPB	2820.279	0.348619	0.112137	3.108879	0.001878	0.005533	2579.789	2289.276	2581.163	3073.639	2881.382	3516.423
12868	GCC2	3839.88	0.348606	0.156668	2.225132	0.026072	0.053865	2887.694	4413.781	2831.627	4412.915	4297.473	4195.789
10706	REEP4	871.7625	0.348068	0.129393	2.690002	0.007145	0.017743	829.9342	703.9716	771.5661	928.5648	926.2349	1070.303
8134	FGD6	5207.95	0.347886	0.103255	3.369183	0.000754	0.002464	4412.583	5101.878	4236.308	5544.603	5927.476	6024.852
14055	BLOC1S3	301.7496	0.347647	0.173486	2.003896	0.045081	0.085271	243.3968	295.6024	257.4206	312.4978	367.927	333.6532
25513	LOC11962;	1.275596	0.347646	2.138979	0.162529	0.87089	0.907376	2.168345	0.547412	0.695731	2.232127	0	2.009959
25515	LOC11961{	1.275596	0.347646	2.138979	0.162529	0.87089	0.907376	2.168345	0.547412	0.695731	2.232127	0	2.009959
13617	GET4	1940.12	0.347504	0.166593	2.085948	0.036983	0.072204	2003.551	1252.478	1869.43	2220.966	1914.504	2379.791
21447	LOC10323;	37.62829	0.347293	0.427746	0.811916	0.41684	0.516701	28.18849	40.50847	29.91645	51.33892	40.6431	35.17428
8933	ACSS3	1731.651	0.347222	0.110712	3.136248	0.001711	0.005092	1556.33	1614.317	1406.073	1872.755	1846.053	2094.377
11669	PTGDS	689.9486	0.347071	0.140055	2.478105	0.013208	0.030096	636.4094	571.4979	619.2009	743.2983	690.9327	878.3521
23039	LOC10321(	18.0135	0.346946	0.624025	0.555981	0.578224	0.66722	14.63633	21.89647	10.43597	26.78552	19.252	15.07469
12577	SEC24B	2776.769	0.346581	0.151436	2.288636	0.022101	0.046716	2088.659	3112.036	2133.808	2908.461	3260.005	3157.645
21166	SPATA17	45.47574	0.346523	0.405036	0.855537	0.392254	0.492681	28.73058	52.55153	38.96096	46.87467	53.47777	52.25893
21285	LOC10323!	46.32362	0.346153	0.413337	0.837458	0.402335	0.502517	37.94605	38.86624	44.52681	42.41041	77.00798	37.18424
8079	SLC39A7	3101.259	0.346141	0.102128	3.38929	0.000701	0.002306	2680.617	2947.265	2568.64	3455.333	3266.422	3689.28
8672	SEC23B	3081.126	0.346062	0.107779	3.210842	0.001323	0.004057	2675.196	2881.576	2588.816	3428.547	3118.823	3793.797
9968	RAB10	11113.43	0.345918	0.120497	2.87076	0.004095	0.010921	8824.624	11706.4	8830.918	12424.02	12355.5	12539.13
15781	ZSWIM3	249.8735	0.345797	0.20282	1.704942	0.088205	0.148583	236.3497	194.3312	233.07	263.391	237.4413	334.6582
22386	LOC11962;	28.06319	0.345672	0.523238	0.660641	0.508843	0.604288	23.8518	24.08612	25.04633	31.24978	47.06043	17.08465
24372	LOC10323{	6.012994	0.345601	0.984837	0.350922	0.725647	0.791536	3.794605	6.568942	5.565851	6.696381	6.417332	7.034856
6338	TMEM183/	3524.538	0.345472	0.086152	4.010009	6.07E-05	0.000255	3082.845	3200.169	3029.91	3890.597	4017.25	3926.455
9424	TMEM115	1783.034	0.345132	0.114966	3.002047	0.002682	0.007565	1652.821	1459.947	1605.748	1899.54	1863.165	2216.985
25499	LOC11962:	1.277371	0.345011	2.100593	0.164244	0.869539	0.906572	1.084173	1.642235	0.695731	2.232127	0	2.009959

5324	CMTM6	12052.71	0.344518	0.076435	4.507358	6.56E-06	3.28E-05	10726.26	10656.47	10479.11	13319.1	13564.1	13571.24
8332	AGO2	6625.912	0.344392	0.104243	3.303755	0.000954	0.003044	5692.991	6124.991	5704.997	7408.429	6618.408	8205.657
20048	LOC11962	110.7024	0.344326	0.337321	1.020766	0.307365	0.407587	70.47123	99.08154	121.0573	127.2312	168.9897	77.38342
22172	PPP1R1A	28.63725	0.344309	0.494951	0.695642	0.486653	0.583514	30.35684	25.72836	19.48048	22.32127	42.78221	31.15436
15818	ADAMTS13	336.0179	0.344223	0.202847	1.696955	0.089705	0.150766	350.7299	231.5552	308.9047	328.1227	363.6488	433.1462
13055	WHAMM	555.0276	0.344199	0.156778	2.195452	0.028131	0.057282	445.0529	570.9505	453.6169	564.7281	622.4812	673.3362
22455	LOC11962	77.10123	0.344133	0.530306	0.648933	0.516382	0.611371	24.39389	140.6848	38.96096	66.96381	94.12087	97.48301
22980	LOC11962	21.23602	0.343815	0.605673	0.567658	0.570267	0.659728	28.73058	10.94824	16.00182	31.24978	21.39111	19.09461
11713	MAGI1	1327.71	0.343799	0.139243	2.469059	0.013547	0.030747	1041.348	1395.353	1075.601	1504.454	1379.726	1569.778
22698	LOC11962	20.47099	0.343663	0.56484	0.608426	0.542905	0.635821	18.43094	21.34906	13.91463	20.08914	29.94755	19.09461
8476	PDPK1	4703.692	0.343609	0.10537	3.260967	0.00111	0.003483	4109.015	4448.268	3887.051	5127.196	4857.92	5792.702
12945	RELB	650.7279	0.3436	0.155169	2.214365	0.026804	0.055046	623.3993	478.4379	621.9838	729.9055	656.707	793.9338
16711	FOXD2	138.3759	0.343504	0.22299	1.540446	0.123452	0.196395	122.5115	122.0728	121.753	162.9453	141.1813	159.7917
25716	TRNAC-GC	1.287904	0.343393	2.58564	0.132808	0.894346	0.92458	2.168345	1.094824	0	4.464254	0	0
9428	ABHD11	2509.399	0.343342	0.114401	3.001225	0.002689	0.007582	2396.564	1921.963	2320.96	2767.837	2714.531	2934.54
23999	LOC10323	9.192317	0.343333	0.844487	0.406558	0.684333	0.758073	9.215468	4.379295	10.43597	13.39276	10.69555	7.034856
24411	LOC10321	7.663246	0.343274	0.994673	0.345113	0.73001	0.79499	9.757555	6.02153	4.174388	17.85702	2.139111	6.029877
8618	AFG3L2	5654.891	0.342941	0.106352	3.224602	0.001261	0.003891	5302.689	4873.607	4788.719	6060.225	5811.964	7092.14
9386	ACAA1	1756.56	0.342896	0.113902	3.01045	0.002609	0.007389	1636.559	1421.081	1592.529	2008.914	1781.879	2098.397
10822	HIVEP2	1753.151	0.342501	0.128491	2.665566	0.007686	0.018881	1359.553	1809.743	1469.385	1868.29	2015.042	1996.894
20076	LOC10322	57.95642	0.342398	0.336843	1.01649	0.309396	0.409708	43.909	54.19377	54.96278	69.19594	64.17332	61.30375
25415	LOC11962	2.21043	0.342227	1.917316	0.178493	0.858336	0.89785	1.626259	4.379295	0	2.232127	0	5.024897
14115	KCTD14	278.5891	0.342054	0.171817	1.990802	0.046503	0.08757	254.7806	251.262	233.07	285.7123	308.0319	338.6781
11754	ZFP30	486.7649	0.341995	0.139137	2.457978	0.013972	0.031602	437.4637	446.1406	402.1327	553.5675	564.7252	516.5594
25535	LOC10324	1.278258	0.341988	2.138338	0.159931	0.872935	0.908794	0.542086	2.189647	0.695731	2.232127	0	2.009959
21830	ZNF708	35.53552	0.341916	0.452649	0.755366	0.450029	0.548054	31.44101	36.67659	25.04633	55.80317	32.08666	32.15934
11772	SNPH	557.3319	0.341285	0.139054	2.454343	0.014114	0.031874	514.9821	477.8905	485.6205	620.5313	562.5861	682.3811
22814	LOC10324	19.65272	0.34116	0.578225	0.590012	0.555183	0.64695	15.7205	14.23271	21.56767	26.78552	23.53022	16.07967
23015	LOC11962	18.75793	0.34115	0.609409	0.559804	0.575613	0.6649	13.01007	13.6853	23.65487	20.08914	14.97377	27.13445
23869	LOC11962	10.44988	0.341031	0.798218	0.427241	0.669204	0.74532	8.131296	5.474118	13.91463	11.16063	14.97377	9.044815
12254	RASSF4	414.8246	0.340941	0.144743	2.355483	0.018499	0.040134	383.2551	351.4384	364.5632	466.5145	442.7959	480.3802
10176	SMCR8	1506.972	0.340784	0.120845	2.82002	0.004802	0.012544	1272.819	1454.473	1266.927	1651.774	1531.603	1864.237
7638	TOR1AIP1	4301.856	0.34077	0.097008	3.512819	0.000443	0.001543	3659.625	4161.972	3567.015	4767.823	4791.608	4863.096
17073	STPG4	143.8575	0.340433	0.229316	1.484561	0.13766	0.21433	121.9694	135.2107	122.4487	149.5525	188.2417	145.722
10956	PARD3B	1933.617	0.340214	0.128944	2.638455	0.008328	0.020209	1571.508	2004.075	1546.611	2145.074	2030.016	2304.418
22173	TRNAL-UA	40.18763	0.339896	0.488664	0.695562	0.486703	0.583548	50.41403	16.96977	39.65669	40.17829	40.6431	53.26391
11575	KHDRBS3	603.843	0.339857	0.135914	2.500528	0.012401	0.028482	514.9821	586.8255	498.8394	651.7811	663.1243	707.5055
12042	NDUFAF1	563.2209	0.339792	0.141677	2.398349	0.016469	0.036359	500.8878	511.2826	479.3589	700.8879	549.7514	637.157
19725	LOC11962	69.01989	0.339782	0.319148	1.064654	0.287033	0.386858	66.13454	49.26706	67.48594	71.42806	83.42532	76.37844
21911	LOC10323	30.62941	0.339565	0.457024	0.742992	0.457487	0.555076	28.73058	26.82318	25.04633	31.24978	42.78221	29.1444
11288	PLEKHF2	893.6643	0.339533	0.132602	2.560543	0.010451	0.024613	755.6684	890.0916	721.4734	921.8684	1058.86	1014.024
24895	LOC10322	4.390753	0.339434	1.276408	0.265929	0.790294	0.843943	5.420864	4.379295	2.087194	0	6.417332	8.039836
22908	LOC11962	17.99946	0.339354	0.586509	0.5786	0.562859	0.653205	14.63633	16.96977	16.00182	15.62489	25.66933	19.09461
7848	GIPC1	2780.631	0.33895	0.098131	3.454063	0.000552	0.001871	2580.331	2348.944	2439.93	3187.477	2892.078	3235.029
15325	MYH7B	225.9597	0.338734	0.190829	1.775068	0.075887	0.131644	195.6932	193.7838	206.6322	265.6231	275.9453	218.0805
11259	SCAF11	7503.346	0.338615	0.13186	2.567997	0.010229	0.024152	5910.368	8140.561	5828.837	8107.085	8652.703	8380.524
10732	DUSP22	739.5298	0.3383	0.126032	2.68424	0.007269	0.018003	641.2882	666.7476	656.0747	787.9408	778.6363	906.4915
12815	ANTXR1	1836.024	0.338217	0.1513	2.235408	0.025391	0.052673	1411.593	2047.32	1406.769	1953.111	2089.911	2107.442
21906	LOC10322	42.58548	0.338111	0.45481	0.743412	0.457232	0.554894	38.48813	32.2973	40.35242	62.49956	57.75599	24.11951
23341	OLFML2A	17.82815	0.338055	0.666234	0.507411	0.611866	0.696912	16.80468	14.78012	14.61036	33.4819	19.252	8.039836
8614	GUSB	2869.77	0.337951	0.104781	3.225303	0.001258	0.003884	2505.523	2654.4	2451.757	3133.906	2945.555	3527.478
8668	INPPL1	6301.122	0.337813	0.105187	3.211544	0.00132	0.004049	5755.873	5224.498	5727.956	6716.47	6502.896	7879.039
21337	ARHGAP8	43.00141	0.337695	0.40879	0.826084	0.408757	0.509294	42.28274	29.56024	41.74388	40.17829	62.03421	42.20914
21455	LOC11962	42.38561	0.337502	0.416511	0.810307	0.417764	0.517653	43.909	37.224	29.91645	51.33892	57.75599	34.1693
7987	SEC61B	2996.477	0.33725	0.098716	3.416383	0.000635	0.002112	2527.207	2856.942	2555.421	3437.476	3414.021	3187.795
9991	MITF	3074.17	0.336969	0.117687	2.863253	0.004193	0.011158	2560.274	3119.7	2473.325	3287.923	3330.595	3673.2
9681	RAB43	2457.035	0.336929	0.114751	2.936171	0.003323	0.009125	2275.136	2047.868	2197.815	2707.57	2438.586	3075.237
11112	ARHGAP31	1540.278	0.336836	0.129498	2.601091	0.009293	0.022233	1292.334	1527.826	1269.014	1578.114	1625.724	1948.655
10190	EXD2	990.2028	0.336796	0.119531	2.817653	0.004838	0.012621	873.8432	905.4191	850.8795	1071.421	1024.634	1215.02
8000	AGPAT3	4917.614	0.336739	0.098672	3.412721	0.000643	0.002137	4494.98	4313.058	4237.7	5357.105	5073.971	6028.872
11655	NETO2	736.7114	0.336555	0.135715	2.479873	0.013143	0.029979	601.7159	740.6482	611.5479	832.5834	793.6101	840.1628
11700	ARRDC1	1052.682	0.336434	0.136101	2.471938	0.013438	0.030544	957.3245	820.0229	1019.246	1142.849	1063.138	1313.508
23080	LOC11962	19.28243	0.336344	0.61237	0.54925	0.582834	0.671345	15.7205	16.42235	18.08902	22.32127	32.08666	11.05477
21762	LOC11962	5081.588	0.336301	0.438831	0.766357	0.443464	0.541747	5307.026	2235.082	5934.589	5879.422	4564.862	6568.546
18915	LOC10321	91.73544	0.336205	0.279848	1.201384	0.229602	0.322706	81.31296	88.1333	73.05179	91.51721	121.9293	94.46807
24303	EVX2	9.242671	0.336009	0.930583	0.361074	0.718044	0.785467	3.794605	7.663765	12.52316	15.62489	12.83466	3.014938
14101	TRPT1	1183.226	0.335911	0.168475	1.993835	0.04617	0.087046	1177.412	835.3504	1131.259	1189.724	1163.676	1601.937
13930	ARID4A	1326.511	0.335911	0.165819	2.025772	0.042788	0.08166	1019.664	1520.71	977.5026	1406.24	1546.577	1488.375
11146	NSD3	3198.439	0.335756	0.129506	2.592599	0.009525	0.02272	2632.913	3385.195	2466.368	3374.976	3642.905	3688.275
17245	SLC24A1	146.0561	0.335567	0.230052	1.458658	0.144659	0.223008	123.0536	144.5167	118.2743	162.9453	181.8244	145.722
10972	PXK	816.4403	0.335432	0.12729	2.635179	0.008409	0.020376	684.113	797.579	685.9911	863.8331	913.4003	953.7255
14944	TPMT	247.2037	0.335353	0.181611	1.846544	0.064813	0.115301	202.7403	220.607	233.7657	247.7661	290.9191	287.4241
10294	TMEM62	901.4628	0.3352	0.120145	2.789976	0.005271	0.013613	859.749	732.9844	799.3953	1015.618	983.9909	1017.039
10234	ZHX3	3196.789	0.335113	0.119381	2.807081	0.004999	0.012987	2785.782	3223.708	2474.021	3569.171	3341.291	3786.763

23381	LOC11961!	14.82449	0.335107	0.667731	0.501859	0.615767	0.700148	11.9259	13.6853	13.2189	13.39276	25.66933	11.05477
18910	LOC11962:	88.36181	0.334994	0.278736	1.201832	0.229429	0.322547	76.43418	87.03848	70.9646	89.28508	106.9555	99.49297
17563	ABLIM2	143.483	0.334944	0.238353	1.405244	0.159949	0.242113	147.4475	128.0944	105.7512	147.3204	160.4333	171.8515
11769	TMEM150/	695.464	0.334686	0.136343	2.454737	0.014099	0.031848	659.177	602.153	588.5887	720.977	731.5758	870.3122
11110	TCF19	855.4632	0.334594	0.128545	2.602924	0.009243	0.022118	814.7558	702.3294	756.9557	935.2612	881.3136	1042.164
18462	DGCR6L	152.4957	0.334511	0.266671	1.254398	0.209697	0.301961	164.7943	93.60742	147.4951	160.7131	160.4333	187.9312
15478	PDE7A	241.9683	0.334485	0.191323	1.748276	0.080416	0.138105	224.9658	226.6285	192.7176	236.6055	267.3888	303.5038
17464	RTL8B	178.9563	0.334295	0.236006	1.416466	0.156639	0.238447	165.8784	119.8832	189.2389	189.7308	216.0502	192.9561
14870	APBB3	245.3238	0.334004	0.17963	1.859405	0.06297	0.112579	200.572	215.1328	236.5487	265.6231	271.6671	282.3992
14487	CCDC24	386.8036	0.333926	0.17324	1.927538	0.053913	0.098928	388.1338	279.18	359.6931	428.5684	432.1004	433.1462
19862	LOC11962!	70.59425	0.333904	0.318962	1.046846	0.295171	0.395012	71.01331	56.38342	59.13717	71.42806	96.25998	69.34358
11165	CDV3	6411.205	0.333849	0.12901	2.587785	0.00966	0.023	5148.736	6884.251	4986.307	7053.521	6909.327	7485.087
8906	CRYL1	1680.339	0.33371	0.106165	3.143308	0.001671	0.004987	1568.256	1375.098	1520.173	1863.826	1798.992	1955.69
9339	MAPKAPK:	2700.357	0.333677	0.110418	3.021951	0.002512	0.007149	2500.102	2261.358	2414.188	2859.355	2789.4	3377.736
23797	LOC10324(	10.80724	0.333624	0.763594	0.436912	0.662175	0.739748	10.29964	8.211177	9.740239	17.85702	10.69555	8.039836
12015	GM2A	1096.32	0.333437	0.138707	2.403898	0.016221	0.035891	1062.489	967.2767	886.3618	1131.688	1108.059	1422.046
13888	LOC10323:	316.6534	0.333312	0.163977	2.032671	0.042086	0.080562	285.6795	255.0939	299.8602	368.301	342.2577	348.7279
11879	ZNF81	1036.266	0.333203	0.137056	2.431151	0.015051	0.033684	849.4493	1067.453	836.2691	1064.725	1178.65	1221.05
13228	TLK1	1836.699	0.332919	0.153875	2.163563	0.030498	0.061293	1380.694	2068.669	1428.337	1988.825	2032.155	2121.512
7830	SERPINH1	12065.79	0.332723	0.096205	3.458474	0.000543	0.001844	10676.39	10958.09	10414.4	13303.48	12274.22	14768.17
12401	INO80D	1131.845	0.332457	0.143259	2.320675	0.020304	0.043528	944.8565	1192.263	871.0557	1203.116	1215.015	1364.762
9029	JUND	3239.328	0.332311	0.107015	3.105267	0.001901	0.005598	2736.994	3040.873	2830.931	3667.385	3234.335	3925.45
23202	RNF208	15.87389	0.332183	0.62714	0.529679	0.596335	0.683306	15.17842	10.40082	16.69755	15.62489	19.252	18.08963
15207	TRNP1	824.5892	0.332165	0.185042	1.795081	0.072641	0.126991	588.1637	976.0353	625.4625	937.4933	904.8438	915.5363
14300	ARHGAP3:	424.3562	0.33214	0.169354	1.961216	0.049854	0.092683	380.5446	330.6367	418.8303	495.5322	400.0137	520.5794
10418	GOLGA2	4088.092	0.331883	0.120164	2.761907	0.005746	0.014664	3371.235	4257.769	3231.672	4437.468	4522.08	4708.329
14085	SNX16	403.1516	0.331788	0.166143	1.997	0.045825	0.086483	346.9353	380.9986	347.17	433.0326	387.179	523.5943
22748	LOC10324!	19.97364	0.33165	0.551746	0.601091	0.547779	0.640175	19.51511	18.06459	15.30609	22.32127	23.53022	21.10457
16680	LOC10321(	153.6102	0.331477	0.214507	1.545301	0.122273	0.194888	130.6428	147.2538	130.1018	167.4095	175.4071	170.8465
9441	SERP1	4419.118	0.331217	0.110445	2.998918	0.002709	0.007629	4220.142	3440.483	4086.03	4859.34	4637.592	5271.117
13332	TPRA1	1395.996	0.331214	0.15461	2.142249	0.032173	0.064156	1400.209	982.0568	1331.63	1473.204	1416.091	1772.784
7450	SH3GLB1	4350.641	0.331089	0.092425	3.582237	0.000341	0.001216	3701.908	4154.856	3704.77	4830.323	4791.608	4920.379
23263	LOC10323{	17.17478	0.331002	0.636339	0.520167	0.602947	0.689072	9.215468	15.87494	20.17621	22.32127	21.39111	14.06971
10496	SEC24D	3585.163	0.330537	0.120412	2.745045	0.00605	0.015324	2966.297	3702.694	2862.239	3854.883	3871.79	4253.073
7566	NACC1	4949.096	0.330188	0.093357	3.536824	0.000405	0.001423	4404.994	4386.411	4371.28	5354.873	5187.343	5989.678
8707	GORASP1	2422.33	0.330036	0.103116	3.200636	0.001371	0.004187	2257.79	2075.786	2110.849	2680.785	2496.342	2912.431
9527	GABARAPL	2663.704	0.329874	0.110794	2.977372	0.002907	0.008113	2443.725	2119.031	2515.069	2984.354	3174.44	2745.604
9453	TNS2	6320.674	0.329783	0.110043	2.996868	0.002728	0.007671	5283.174	5801.47	5727.956	6729.863	6363.854	8017.726
8706	DYNLT3	1313.061	0.329487	0.102939	3.200816	0.00137	0.004185	1179.038	1171.461	1137.521	1511.15	1480.265	1398.931
22762	LOC10323;	25.36899	0.329473	0.549635	0.599439	0.54888	0.641039	14.63633	34.48694	18.08902	26.78552	32.08666	26.12947
24243	ST6GALNA	8.86212	0.329093	0.891673	0.369074	0.712073	0.780863	3.794605	8.758589	11.1317	4.464254	14.97377	10.04979
20654	LOC10323(	67.442	0.32899	0.354481	0.92809	0.353361	0.454832	53.12446	78.8273	47.30973	58.0353	91.98176	75.37346
23913	CCDC184	10.21859	0.328858	0.781099	0.42102	0.67374	0.749023	7.589209	7.663765	11.82743	15.62489	8.556443	10.04979
18027	ZCWPW1	130.3739	0.328667	0.245569	1.338388	0.18077	0.266587	128.4745	113.3142	102.9682	167.4095	145.4595	124.6175
23988	LOC11962(	14.86994	0.3286	0.805066	0.408165	0.683152	0.757112	19.51511	3.284471	17.39328	8.928508	14.97377	25.12449
12531	CUNH6orf:	511.1259	0.32856	0.143112	2.295828	0.021686	0.046017	415.7802	483.912	461.9656	575.8888	526.2212	602.9877
6801	SAR1A	8061.617	0.328391	0.086019	3.817665	0.000135	0.000527	7375.627	6773.126	7299.614	8752.17	8840.944	9328.219
9868	LOC10323:	1802.018	0.328199	0.113579	2.889594	0.003857	0.010392	1620.838	1530.016	1649.579	1955.343	1818.244	2238.089
10579	SH3PXD2A	1051.708	0.328134	0.120323	2.727111	0.006389	0.016056	890.1058	973.8456	938.5416	1055.796	1178.65	1273.309
11199	CLN3	908.4437	0.328088	0.12704	2.582563	0.009807	0.02328	859.2069	725.8681	834.1819	1033.475	932.6523	1065.278
15310	ZNF713	258.8642	0.328074	0.18454	1.777794	0.075438	0.130994	228.2184	251.262	206.6322	314.7299	293.0582	259.2847
23922	DYNLT2	11.36216	0.328002	0.781958	0.419462	0.674878	0.749975	10.84173	10.40082	8.348776	11.16063	21.39111	6.029877
22364	LOC10324	27.02463	0.32789	0.493452	0.664481	0.506382	0.601903	29.81475	22.9913	19.48048	29.01765	25.66933	35.17428
10973	NCDN	1357.908	0.327664	0.12435	2.635001	0.008414	0.020379	1208.853	1118.362	1291.973	1477.668	1356.196	1694.395
18782	LOC10324:	105.3936	0.327595	0.268425	1.220437	0.222299	0.314654	88.36008	105.6505	84.87923	116.0706	136.9031	100.4979
13188	B3GALT4	595.8932	0.327546	0.150864	2.171133	0.029921	0.060312	567.5644	471.869	550.3235	651.7811	586.1163	747.7047
10110	ETNK2	1820.399	0.327514	0.115478	2.836173	0.004566	0.012006	1727.087	1560.124	1562.613	1962.04	1850.331	2260.199
25412	LOC11962(	1.58146	0.327501	1.828272	0.179132	0.857834	0.897431	1.626259	1.094824	1.391463	2.232127	2.139111	1.004979
9584	USF1	1383.285	0.327427	0.110451	2.964452	0.003032	0.008411	1272.819	1198.284	1212.66	1598.203	1394.7	1623.042
24137	LOC10321!	9.759857	0.327412	0.852065	0.384257	0.700788	0.771798	9.757555	10.94824	4.87012	6.696381	19.252	7.034856
12430	GALNT18	566.2002	0.327374	0.141394	2.315338	0.020594	0.044041	525.2817	486.1017	491.8821	616.067	708.0456	569.8234
14384	PCLAF	989.1658	0.327092	0.167926	1.947836	0.051435	0.095063	974.6713	645.3985	1013.681	1082.582	1093.086	1125.577
21988	LOC10324;	33.56466	0.327038	0.448185	0.729694	0.465577	0.562915	31.9831	31.74989	25.04633	31.24978	49.19955	32.15934
18889	LOC10323;	108.6013	0.326918	0.271212	1.205397	0.22805	0.320965	76.97626	105.1031	107.8384	127.2312	104.8164	129.6424
20079	LOC10324:	65.86779	0.326808	0.321654	1.016022	0.309619	0.409942	58.00324	63.49977	52.87558	87.05295	68.45154	65.32367
23929	LOC10321!	11.41002	0.326787	0.780447	0.418718	0.675422	0.750391	10.84173	7.116354	11.82743	13.39276	19.252	6.029877
14158	FAM200A	396.9239	0.326782	0.164758	1.983412	0.047321	0.088857	321.9993	390.3046	341.6041	502.2286	421.4048	404.0017
12658	BCL7A	449.4293	0.326496	0.143928	2.268458	0.023301	0.048939	383.2551	423.6967	391.001	506.6928	466.3261	525.6043
21740	LOC11962‹	42.78299	0.326468	0.423403	0.771057	0.440673	0.538882	37.94605	34.48694	42.43961	26.78552	57.75599	57.28383
18455	USP27X	119.0758	0.326446	0.259955	1.255778	0.209196	0.301354	97.03346	125.9047	93.92374	149.5525	113.3729	134.6672
20029	KCNA4	73.42881	0.326395	0.319113	1.02282	0.306393	0.406663	62.33993	57.47824	74.44326	82.5887	98.39909	65.32367
8750	LMTK2	3442.322	0.326188	0.102288	3.188935	0.001428	0.004339	2892.031	3305.273	2972.164	3674.081	3689.966	4120.416
12565	LAGE3	1556.519	0.326083	0.142374	2.290334	0.022002	0.046555	1544.946	1098.656	1502.78	1705.345	1666.367	1821.023
12337	SPATS2	1011.305	0.326032	0.139527	2.33669	0.019455	0.041928	820.1767	1064.716	807.0484	1098.206	1155.12	1122.562

25418	LOC11961	1.582348	0.325964	1.828195	0.178298	0.858489	0.897869	1.084173	1.642235	1.391463	2.232127	2.139111	1.004979
13398	LOC10324	910.6478	0.325917	0.153156	2.128012	0.033336	0.066147	886.8533	677.1484	865.4898	973.2074	898.4265	1162.761
12236	LOC11962	1914.875	0.325849	0.138088	2.359726	0.018288	0.039735	1654.448	1556.292	1881.953	2234.359	2406.5	1755.699
16119	LOC11961	192.0171	0.32577	0.198329	1.642572	0.100471	0.165707	182.141	155.465	173.9328	200.8914	224.6066	215.0656
24149	LOC11962	13.09613	0.325757	0.849554	0.383445	0.70139	0.772142	21.68345	2.737059	10.43597	20.08914	8.556443	15.07469
23187	LOC11962	19.49826	0.325733	0.612638	0.531688	0.594942	0.682113	16.26259	11.49565	23.65487	31.24978	19.252	15.07469
24976	LOC11962	4.746628	0.325532	1.297095	0.25097	0.801837	0.853493	4.336691	0.547412	7.653045	2.232127	10.69555	3.014938
21516	LOC11962	39.18423	0.325328	0.405796	0.801703	0.422725	0.522316	30.89892	32.2973	41.04815	46.87467	42.78221	41.20416
10611	PICALM	21028.47	0.32519	0.119624	2.718447	0.006559	0.016432	16893.58	22332.21	16779.65	23350.28	23072.45	23742.64
9632	RREB1	1772.209	0.325186	0.110131	2.952713	0.00315	0.008694	1551.451	1703.546	1468.689	1879.451	1916.643	2113.472
8654	MRPL18	1693.09	0.325161	0.101153	3.214548	0.001307	0.004014	1567.172	1414.512	1529.218	1897.308	1826.801	1923.531
23878	ITM2A	11.0799	0.325149	0.762792	0.426262	0.669917	0.745876	9.215468	8.211177	11.82743	8.928508	19.252	9.044815
9345	TLE5	4447.864	0.325125	0.107659	3.019959	0.002528	0.007192	3612.464	4395.169	3841.829	4995.5	4618.34	5223.883
10553	NFU1	1191.542	0.325015	0.118904	2.733431	0.006268	0.01579	982.2605	1171.461	1017.159	1372.758	1343.361	1262.254
12387	ZNF649	648.4702	0.325014	0.139748	2.325715	0.020034	0.042997	607.1367	605.4375	514.1455	658.4775	785.0536	720.5703
10063	BAG4	1350.349	0.324994	0.114095	2.848447	0.004393	0.011607	1136.213	1325.831	1132.651	1544.632	1499.517	1463.25
10904	NDRG4	1111.419	0.324935	0.122603	2.650303	0.008042	0.019607	978.4659	991.9102	996.2873	1183.027	1120.894	1397.926
23190	NIBAN3	24.55007	0.324747	0.611713	0.530881	0.595501	0.682682	18.43094	21.34906	24.3506	33.4819	40.6431	9.044815
20438	LOC11962	96.10671	0.324672	0.339596	0.956054	0.339045	0.441014	119.259	60.76271	74.44326	107.1421	132.6249	82.40832
11213	LOC10323	958.9396	0.324636	0.125899	2.57854	0.009922	0.023524	825.0555	954.6862	773.6533	1095.974	1046.025	1058.243
10473	STK3	2453.642	0.324625	0.118024	2.750496	0.005951	0.015105	2013.851	2520.284	2001.619	2783.462	2661.054	2741.584
24425	LOC10324	6.994514	0.32444	0.951063	0.341134	0.733003	0.797825	7.589209	4.379295	6.261582	11.16063	8.556443	4.019918
23882	LOC10321	11.108	0.324366	0.762038	0.425657	0.670358	0.746199	14.09425	8.758589	6.957314	8.928508	12.83466	15.07469
22219	LOC11962	34.53671	0.324309	0.470595	0.689148	0.49073	0.587158	28.73058	31.20247	32.69937	49.10679	19.252	46.22906
8636	ADPGK	1925.121	0.324206	0.100722	3.218808	0.001287	0.003963	1755.276	1629.645	1748.373	2127.217	2030.016	2260.199
18093	LOC10322	137.4436	0.324199	0.245085	1.322801	0.185902	0.273155	148.5317	104.5557	113.4042	151.7846	147.5986	158.7868
13914	LOC11962	355.3204	0.324118	0.15981	2.028149	0.042545	0.081289	335.5515	283.0119	329.0809	401.7829	376.4835	406.0117
22602	LOC10321	24.42209	0.32397	0.519387	0.623753	0.53279	0.626652	20.59928	25.72836	18.08902	26.78552	34.22577	21.10457
10649	NME7	1117.921	0.323968	0.119756	2.705226	0.006826	0.01704	963.2875	1072.927	937.8459	1312.491	1281.327	1139.647
23854	LOC10322	11.5139	0.323915	0.754054	0.429564	0.667513	0.743935	13.01007	8.211177	9.740239	15.62489	6.417332	16.07967
12171	KEAP1	2662.644	0.323806	0.136526	2.371759	0.017704	0.03867	2630.203	2004.075	2466.368	2930.783	2579.767	3364.671
9622	NCSTN	3008.154	0.323748	0.109556	2.955096	0.003126	0.008636	2482.756	3004.743	2530.375	3374.976	3180.858	3475.219
14063	ZSWIM1	681.4892	0.323635	0.161717	2.001246	0.045366	0.085761	554.0123	739.5534	521.1028	814.7263	720.8803	738.6599
15223	NPAS2	285.2063	0.323378	0.180474	1.791827	0.073161	0.127766	226.05	273.7059	261.595	339.2833	275.9453	334.6582
14662	RBM43	570.3181	0.323197	0.170509	1.895477	0.058029	0.105218	434.7533	631.1658	454.3126	611.6028	643.8723	646.2018
13530	LOC11962	384.9032	0.322836	0.153595	2.101866	0.035565	0.069881	353.9824	360.197	313.0791	406.2471	425.683	450.2308
24958	LOC11962	3.488168	0.322722	1.273088	0.253495	0.799886	0.85203	3.252518	3.831883	2.087194	4.464254	4.278221	3.014938
11806	PTPRE	683.4906	0.322695	0.131835	2.447709	0.014377	0.032374	601.7159	666.7476	553.1064	738.834	780.7754	759.7645
13503	LOC11962	479.6458	0.322633	0.153164	2.106463	0.035164	0.069234	423.9115	481.175	372.2163	531.2462	549.7514	519.5744
22963	MUSTN1	21.94656	0.322454	0.566456	0.569248	0.569188	0.658967	23.8518	13.13788	20.87194	26.78552	29.94755	17.08465
14109	SPPL2B	1395.424	0.322347	0.161837	1.991797	0.046393	0.087417	1375.273	924.0311	1423.466	1571.417	1433.204	1645.151
7416	ABCC4	15887.14	0.322282	0.089693	3.593181	0.000327	0.001171	14047.08	15006.2	13310.73	17370.41	16963.15	18625.28
13774	GAREM1	849.1843	0.322188	0.157004	2.0521	0.04016	0.077507	677.0659	930.6001	657.4661	937.4933	900.5656	991.9147
16083	TFAP2A	478.0909	0.321767	0.194973	1.650317	0.098878	0.163444	330.1306	550.1489	395.1754	497.7643	547.6123	547.7138
11458	TNRC6B	3310.048	0.321574	0.127341	2.52531	0.01156	0.026821	2661.102	3492.487	2677.174	3508.904	3568.037	3952.584
20150	ANKRD37	65.94629	0.321496	0.320306	1.003715	0.315516	0.416271	66.67662	50.36189	58.44144	78.12444	72.72976	69.34358
8528	RFTN1	2444.25	0.321324	0.098968	3.246747	0.001167	0.003639	2229.601	2195.669	2099.022	2685.249	2545.542	2910.421
13662	ANKRD13D	539.6549	0.321199	0.154731	2.075855	0.037907	0.073764	482.4569	419.3174	541.279	582.5851	554.0297	658.2616
16195	FBXL8	223.7319	0.321196	0.197339	1.627636	0.103602	0.170069	215.7504	170.2451	212.1981	249.9982	228.8848	265.3146
21913	LOC11962	36.37627	0.321135	0.432696	0.742172	0.457983	0.555628	29.27266	35.58177	31.30791	53.57105	36.36488	32.15934
13005	MAP1B	43489.67	0.32108	0.145748	2.202988	0.027596	0.056411	32277.45	49604.27	34129.8	46885.83	47237.98	50802.72
11773	ZNF394	925.1843	0.320987	0.130792	2.454174	0.014121	0.031887	766.5101	938.2638	764.6088	991.0644	1020.356	1070.303
12476	MUL1	1029.535	0.32087	0.139185	2.305343	0.021147	0.045063	1018.58	844.109	889.1447	1091.51	1020.356	1313.508
24826	LOC11962	4.767644	0.320845	1.158307	0.276995	0.781784	0.837176	5.96295	5.474118	1.391463	4.464254	4.278221	7.034856
21955	LOC10322	43.86259	0.320465	0.435909	0.735165	0.462239	0.559719	49.32986	29.01283	37.56949	44.64254	68.45154	34.1693
12340	DYNC2I1	617.9766	0.320111	0.137023	2.336178	0.019482	0.041971	597.9213	521.136	532.9302	631.6919	684.5154	739.6649
17066	FBXW9	293.6476	0.319802	0.215328	1.485187	0.137494	0.214185	304.1105	186.6674	293.5986	334.819	308.0319	334.6582
9697	PTPRK	9677.438	0.319651	0.109037	2.931581	0.003372	0.009246	8078.713	9770.753	7982.822	10749.92	10036.71	11445.71
14273	LIN7B	302.6679	0.319622	0.162577	1.965971	0.049302	0.09183	275.922	253.4517	278.9883	321.4263	346.5359	339.6831
11491	KDM4B	951.5138	0.319449	0.126853	2.518255	0.011794	0.027286	914.4997	806.885	822.3545	1053.564	954.0434	1157.736
20408	LOC10321	69.93489	0.319406	0.332754	0.959886	0.337113	0.439148	76.97626	56.93083	53.57132	60.26743	83.42532	88.43819
17590	LOC10321	154.0642	0.319329	0.227686	1.402496	0.160767	0.242979	151.7842	119.8832	140.5377	147.3204	183.9635	180.8963
23739	LOC11961	16.33086	0.319238	0.714629	0.446719	0.655078	0.733614	7.047123	13.6853	22.2634	24.5534	21.39111	9.044815
23979	NOG	12.86303	0.319206	0.778341	0.410111	0.681725	0.755814	7.589209	8.211177	18.08902	24.5534	10.69555	8.039836
10138	AGFG2	1239.702	0.319087	0.112762	2.829745	0.004659	0.012216	1083.631	1048.841	1180.656	1386.151	1287.745	1451.19
13344	LOC10322	707.2203	0.319025	0.149191	2.138362	0.032487	0.064724	672.7292	531.5369	687.3826	756.691	733.715	861.2674
21195	HSF4	56.18859	0.318954	0.374983	0.850581	0.395002	0.495438	40.65648	48.17224	59.8329	73.66019	70.59065	44.2191
18780	LOC10324	134.2025	0.31878	0.261059	1.221102	0.222047	0.314331	91.6126	148.3486	118.9701	147.3204	141.1813	157.7818
9603	MRPS22	1983.442	0.31864	0.107631	2.960469	0.003072	0.008503	1872.908	1641.688	1777.594	2377.215	2151.945	2079.303
10879	FBXO45	1576.678	0.318608	0.12005	2.653966	0.007955	0.01944	1382.32	1526.184	1305.192	1770.077	1565.829	1910.466
14801	MOB3A	284.1005	0.318492	0.170044	1.872991	0.06107	0.109691	255.3227	235.9345	265.7694	321.4263	333.7013	292.449
8905	LOC11962	1655.514	0.318426	0.101301	3.143369	0.00167	0.004986	1430.024	1571.619	1419.292	1825.88	1831.079	1855.192
18420	ZNF391	180.7661	0.318336	0.252298	1.261744	0.207041	0.298824	124.1378	211.8484	146.7993	176.338	222.4675	203.0059
22223	LOC11962	36.50625	0.317967	0.462224	0.687907	0.491511	0.587987	36.86187	31.74989	27.82925	62.49956	29.94755	30.14938

20099	LOC10323:	87.54147	0.317939	0.313939	1.012743	0.311183	0.411603	83.4813	54.74118	95.3152	107.1421	94.12087	90.44815
22293	TRNAN-GU	39.39329	0.317937	0.470862	0.675224	0.499533	0.59568	26.02015	48.17224	29.91645	64.73168	36.36488	31.15436
24284	TRNAS-AG.	7.999161	0.317879	0.872433	0.364359	0.71559	0.783395	7.047123	7.116354	7.653045	6.696381	6.417332	13.06473
11759	HEXIM1	2727.858	0.317846	0.129402	2.456267	0.014039	0.031739	2115.221	2806.58	2367.574	3051.318	2723.088	3303.368
23158	FITM1	19.73369	0.317843	0.592932	0.536052	0.591922	0.679488	16.26259	12.59047	23.65487	17.85702	29.94755	18.08963
14090	LOC11962	353.2404	0.317797	0.159161	1.996702	0.045858	0.086524	348.5615	299.9817	294.2944	395.0865	393.5964	387.9221
23769	LOC10321	12.72584	0.317657	0.717994	0.442423	0.658183	0.736161	11.38381	9.853413	13.2189	6.696381	17.11289	18.08963
13298	RSAD1	1049.451	0.31765	0.147886	2.147937	0.031719	0.063411	1050.563	789.3678	967.0666	1176.331	1013.938	1299.438
6727	DBI	8719.102	0.317369	0.08251	3.846445	0.00012	0.000474	7546.926	7933.092	7807.497	9997.697	9739.371	9290.03
24126	LOC10322	9.59692	0.316872	0.819866	0.386492	0.699132	0.77039	10.84173	5.474118	9.044508	15.62489	8.556443	8.039836
13389	RFX2	447.6161	0.316431	0.148585	2.129636	0.033202	0.065926	410.9015	379.9038	409.09	468.7467	466.3261	550.7287
15003	TMEM141	1039.59	0.316406	0.172649	1.832654	0.066854	0.118464	1021.833	671.1269	1086.732	1180.795	1078.112	1198.941
12150	MAPK11	1823.782	0.316363	0.133179	2.375479	0.017526	0.038348	1716.245	1419.439	1744.894	1843.737	1888.835	2329.542
15030	LOC10324	389.7952	0.316218	0.173047	1.827352	0.067647	0.119654	330.1306	410.5589	299.1645	435.2648	455.6306	408.0217
16881	SMIM17	161.0629	0.316069	0.209402	1.509393	0.131198	0.206612	150.7	134.6633	144.7121	176.338	186.1026	173.8614
22129	LOC11962	32.1409	0.315934	0.448724	0.704072	0.481388	0.578327	31.44101	26.82318	27.82925	26.78552	42.78221	37.18424
14145	ADAM15	2309.832	0.315863	0.159002	1.986536	0.046974	0.088286	2349.402	1629.645	2200.598	2417.394	2211.84	3050.113
15338	ABHD6	276.2285	0.315797	0.178152	1.77263	0.07629	0.132214	251.5281	220.607	264.3779	325.8905	316.5884	278.3793
12185	ZNF512B	2425.991	0.315675	0.133216	2.369649	0.017805	0.038847	2289.231	1833.282	2368.27	2604.892	2417.195	3043.078
22353	LOC11961	31.18833	0.315458	0.473306	0.666499	0.505092	0.600709	35.23561	20.80165	27.13352	29.01765	42.78221	32.15934
13610	CHST2	554.5162	0.315389	0.151088	2.087445	0.036848	0.071977	462.9418	558.9075	463.3571	609.3707	556.1688	676.3512
25217	LOC11961	2.544576	0.315356	1.491119	0.211489	0.832506	0.877668	1.626259	1.642235	3.478657	2.232127	4.278221	2.009959
24783	LOC11962	5.364758	0.314926	1.106388	0.284643	0.775918	0.832336	4.336691	7.116354	2.782925	2.232127	10.69555	5.024897
23765	LOC11961	13.70448	0.31492	0.711084	0.442874	0.657857	0.735932	6.505036	14.23271	16.00182	11.16063	19.252	15.07469
19339	LOC11962	154.6922	0.314863	0.278995	1.128565	0.259082	0.356156	104.6227	192.689	115.4914	165.1774	190.3808	159.7917
19264	XKR5	98.58347	0.314656	0.275419	1.142463	0.253262	0.34951	102.9964	83.75401	77.92191	102.6778	100.5382	123.6125
10830	DAG1	6807.214	0.314497	0.11801	2.665006	0.007699	0.018898	5804.119	6956.509	5448.272	7270.038	7061.204	8303.14
11334	CORO6	837.7707	0.314377	0.123287	2.549953	0.010774	0.025271	712.3015	755.9757	774.349	977.6716	838.5314	967.7952
12743	ZNF75D	662.5246	0.314312	0.139683	2.250184	0.024437	0.050982	580.5745	662.3683	529.4516	687.4951	761.5234	753.7346
14050	ARID4B	4857.809	0.31431	0.156754	2.005109	0.044951	0.085056	3750.696	5655.859	3585.104	5100.41	5642.974	5411.814
11000	KATNA1	1013.468	0.314192	0.119517	2.628837	0.008568	0.020706	896.0688	964.5396	847.4008	1218.741	1071.694	1082.363
[truncated: 2,124,826 more chars]
