# Supplementary material for: Comparative Transcriptomic Analyses of a Vero Cell Line in Suspension versus Adherent Culture Conditions
Source: Int J Cell Biol. 2023 Aug 30;2023:9364689. doi: 10.1155/2023/9364689 (PMC10482560; doi:10.1155/2023/9364689)
Supplement: Supplementary Materials — Two supplementary materials are made available. Supplementary Table S1: compiling the differentially expressed gene list. Supplementary Table S2: containing the raw results from metabolic analysis. [file 9364689.f1.zip › Supplementary_table_S2_MaREA_-_Results__Adherent_vs_Suspension.pdf]

ids	Log2(fold change)	
DM_Proline	-4.284582819	proline metabolism
Proline_DM_COOP	-4.284582819	proline metabolism
SOD2	-4.268787475	Folate metabolism
Folate_DM_COOP	-2.545283038	Folate metabolism
DM_Folate	-2.545283038	Folate metabolism
CA12_CA2_CA9_CA14_CA1_CA3_CA4_CA7_CA13	-2.369776277	nitrogen metabolism
Aspartate_DM_COOP	-2.05460882	aspartate metabolism
DM_Aspartate	-2.05460882	aspartate metabolism
carnitineAcylTransferaseI	-1.670562162	lipids metabolism
ENO1_ENO3_ENO2	-1.505199688	glycolytic pathway
MTHFD1_3	-1.25347137	folate metabolic pathway
Palmitate_DM_COOP	-1.167201395	palmitate metabolism
DM_Palmitate	-1.167201395	palmitate metabolism
SQLE	-1.143418033	
GCL	-1.129948759	
mGSRnadph	-1.099258939	
mGSRnadh	-1.099258939	
DM_Urea	-1.061293465	
palmitateActivation	-0.9670331	
HMGCR	-0.917450812	
TranspCystineSer	-0.912675278	
ACO1_IREB2	-0.880865788	
ACO2_ACO1_1	-0.880865788	
betaOxidation	-0.878995563	
Urea_DM_COOP	-0.83646439	
Palmitate_UP_COOP	-0.778284167	
ALDH4A1_2	-0.777546472	
ALDH4A1_1	-0.777546472	
FDFT1_2	-0.687774654	
FDFT1_1	-0.687774654	
TKTL1_TKTL2_TKT_2	-0.678250385	
TKTL1_TKTL2_TKT_1	-0.678250385	
Complex1ROS	-0.605831865	
OAT	-0.590359872	
carnitineAcylTransferaseII	-0.589582149	
IDI1_IDI2	-0.578715919	
AKG_DM_COOP	-0.569817648	
HMGCS1_HMGCS2	-0.554812071	
DLSTP1_DLD_OGDH_PDHX_DLST_DHTKD1_OGDHL	-0.554765462	
DHCR24_LEFTY1	-0.553829688	
LSS	-0.524680714	
GCK_HKDC1_HK1_ADPGK_HK2_HK3	-0.507108916	
GPI	-0.483360682	
GS	-0.475363661	
MPC1_MPC2_MPC1L	-0.219084955	
LDH	-0.182734938	
DM_Glucose	0.074627175	
Glucose_DM_COOP	0.074627175	
DM_AKG	0.213720885	
LHPP_PPA2_PRUNE_EIF4A2_NAV2_PPA1_1	0.42297536	
TYMS	0.424278355	
PRPS2_PRPS1_PRPS1L1	0.47260837	
PYCRL_PYCR2_PYCR1_LEFTY1_2	0.479447542	
PYCRL_PYCR2_PYCR1_LEFTY1_1	0.479447542	
MDH2	0.492942548	
LactateL_DM_COOP	0.494256927	
DM_LactateL	0.494256927	
ACLY	0.495021758	
GART_1	0.514908059	
GART_2	0.514908059	
OGDH_GART_OGDHL	0.514908059	
GLS	0.549620339	
GLS2	0.549620339	
ACACB_PRKAG2_PRKAB2_ACACA_PRKAA2	0.558136215	
ME2_ME3_1	0.562417156	
Asparagine_DM_COOP	0.585687666	
DM_Asparagine	0.585687666	
RPE_LOC729020	0.592214156	
TranspCystineGlu	0.600260089	
SLC25A5_SLC25A4_SLC25A6	0.621127462	
ALDH3B1_ALDH3A1_ALDH3B2_ALDH1A3	0.621127462	
Glutamine_DM_COOP	0.629720883	
DM_Glutamine	0.629720883	
TNRC6B_ADSL_1	0.635729181	
TNRC6B_ADSL_2	0.635729181	
SHMT1_2	0.647763394	
TransportFolate	0.669747064	
G6PPer	0.681511534	
ATIC_1	0.716214326	
ATIC_2	0.716214326	
IDH3	0.751429959	
CAD_3	0.758558062	
CAD_1	0.758558062	
CAD_2	0.758558062	
ARID4B_GGPS1_FDPS_FDPSP7_1	0.767508061	
ARID4B_GGPS1_FDPS_FDPSP7_2	0.767508061	
Alanine_DM_COOP	0.775208763	
DM_Alanine	0.775208763	
Serine_DM_COOP	0.900339379	
DM_Serine	0.900339379	
RPIA_LOC101060545	0.911031623	
Glycine_DM_COOP	0.915279126	
DM_Glycine	0.915279126	
ATPsynthase	0.929361346	
AK	1.020522549	
PFAS	1.027643105	
CA5B_CA5A	1.150245473	
IMPDH2	1.161815824	
CMPK1_DTYMK	1.188041537	
DHFRL1_DHFR_4	1.290720546	
DHFRL1_DHFR_5	1.290720546	
DHFRL1_DHFR_6	1.290720546	
DHFRL1_DHFR_2	1.290720546	
Ex_SC_H2O	1.324704252	
MTHFD1_AL445665_1_AQP7P4_LOC286297_2	1.46153521	
AK2_TAF9_AK1_AK7_AK3_AK5_AKD1_AK4_AK8_1	1.579405647	
Arginine_DM_COOP	1.604353361	
DM_Arginines	1.604353361	
OGDH_ODC1_OGDHL	1.633632399	
MTHFD1_2	1.661709798	
MTHFD2_2	1.661709798	mitochondrial one-carbon metabolism (Fig. 1d), a metabolic system recently implicated in rapid cancer cell proliferation
MTHFD2_4	1.661709798	mitochondrial one-carbon metabolism (Fig. 1d), a metabolic system recently implicated in rapid cancer cell proliferation
ALDOC_ALDOB_ALDOA_2	1.705661546	
ALDOC_ALDOB_ALDOA_1	1.705661546	
VPS29_PSPH_PSPHP1	2.031170948	One carbon pool by folate
CTPS2_GATM_CTPS1	2.040658166	Pyrimidine metabolism
ASNS	2.544354243	asparagine metabolism, adaptation to nutrient deprivation and/or hypoxia.
PEPCK	2.853019955	metabolic pathway of gluconeogenesis
AGAT	2.879052713	galactose metabolism
PHGDH_UBAC2	4.191346901	Glycine, serine and threonine metabolism
PSAT1	4.226572589	Serine pathway has a key role in cancer metabolism, using glycolysis-derived glucose for serine production and tumor growth. unrestrained cell cycle progression
